# Genome-wide mapping and characterization of microsatellites in the swamp eel genome

**DOI:** 10.1038/s41598-017-03330-7

**Published:** 2017-06-09

**Authors:** Zhigang Li, Feng Chen, Chunhua Huang, Weixin Zheng, Chunlai Yu, Hanhua Cheng, Rongjia Zhou

**Affiliations:** Hubei Key Laboratory of Cell Homeostasis, Laboratory of Molecular and Developmental Genetics, College of Life Sciences, Wuhan University, Wuhan, 430072 P. R. China

## Abstract

We described genome-wide screening and characterization of microsatellites in the swamp eel genome. A total of 99,293 microsatellite loci were identified in the genome with an overall density of 179 microsatellites per megabase of genomic sequences. The dinucleotide microsatellites were the most abundant type representing 71% of the total microsatellite loci and the AC-rich motifs were the most recurrent in all repeat types. Microsatellite frequency decreased as numbers of repeat units increased, which was more obvious in long than short microsatellite motifs. Most of microsatellites were located in non-coding regions, whereas only approximately 1% of the microsatellites were detected in coding regions. Trinucleotide repeats were most abundant microsatellites in the coding regions, which represented amino acid repeats in proteins. There was a chromosome-biased distribution of microsatellites in non-coding regions, with the highest density of 203.95/Mb on chromosome 8 and the least on chromosome 7 (164.06/Mb). The most abundant dinucleotides (AC)n was mainly located on chromosome 8. Notably, genomic mapping showed that there was a chromosome-biased association of genomic distributions between microsatellites and transposon elements. Thus, the novel dataset of microsatellites in swamp eel provides a valuable resource for further studies on QTL-based selection breeding, genetic resource conservation and evolutionary genetics.

## Introduction

Swamp eel (*Monopterus albus*) taxonomically belongs to teleosts, the family Synbranchidae of the order Synbranchiformes (Neoteleostei, Teleostei, and Vertebrata). The fish is distributed mainly in China, Korea, Japan, Thailand, Lao, Indonesia, Malaysia, Philippines and India. They are also found in southeastern United States and northern Australia. Swamp eel is an economically important species in southeast Asia for food production. In addition, because of its natural sex reversal characteristic from female via intersex into male during its life cycle and relative small genome size, swamp eel is an ideal model for studies of comparative genomics and sexual differentiation^1, 2^. Recently, our group has sequenced the whole genome of swamp eel. With the availability of genome sequence resources, it poses a challenge for mining of useful genetic markers and genes in a genome-wide level and utilization of them in genetic improvement of swamp eel.

Microsatellites, also known as, simple sequence repeats (SSRs), are short tandem repeats of 1–6 nucleotides. Microsatellites are observed in almost all known eukaryotic and prokaryotic genomes, and present in both coding and non-coding regions^3–5^. This makes microsatellite a powerful genetic marker for a variety of applications, such as genetic linkage mapping, population genetics, QTL (quantitative trait loci)-based selection breeding, molecular breeding, and evolutionary studies^6–10^. In comparison with other genetic marker systems, such as restriction fragment length polymorphism, random amplified polymorphic DNA, amplified fragment length polymorphism, sequence-related amplified polymorphism, and target region amplification polymorphism, microsatellites are characterized by their high frequency of distribution, co-dominance, reproducibility, and high polymorphism^11, 12^. Efforts have been made worldwide to compile and develop microsatellite databases in eukaryotes^13–17^. In teleost fishes, valuable microsatellites and related genetic linkage maps have been characterized^18–21^. Although a few of SSR markers in swamp eel have been reported^22–24^, a genome-wide characterization of microsatellites remains to be identified in this species.

Recent development in high-throughput DNA sequencing technologies provides new opportunities to promote mining of molecular markers. In this study, taking advantage of the whole genome sequences of swamp eel we obtained recently, we conducted a genome-wide detection of microsatellite sequences. We analyzed distribution of microsatellite motifs (dinucleotides﻿﻿,﻿ trinucleotides, tetranucleotides, pentanucleotides and hexanucleotides) in the genome, characterized microsatellites in both coding and noncoding regions. We found that trinucleotide repeats were most abundant microsatellites in coding regions though their low enrichment, and microsatellites were abundant and chromosome-biased in non-coding regions. In particular, a chromosome-biased association of genomic distributions between microsatellites and transposon elements (TEs) was described. The novel set of microsatellites in swamp eel provides a valuable dataset for further studies on QTL-based selection breeding, genetic resource conservation and evolutionary genetics.

## Results

### Identification of microsatellites in the swamp eel genome

**Figure 1:**
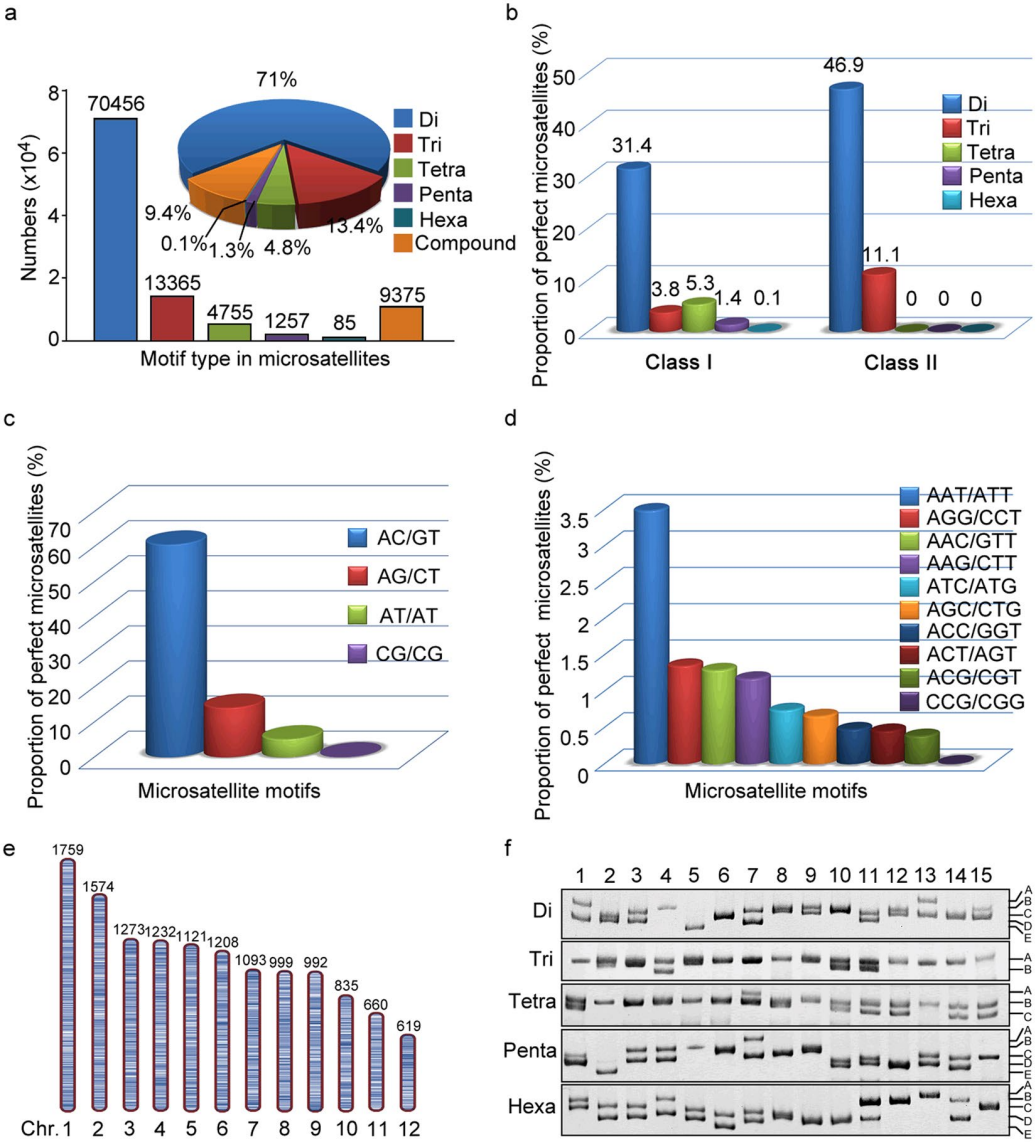
Distribution and classification of microsatellites identified in the swamp eel genome. (**a**) Numbers and proportions of microsatellites with different motif types. Microsatellite proportion was indicated in the pie chart. Di, dinucleotide repeats; Tri, trinucleotides repeats; Tetra, trinucleotide repeats; Penta, pentanucleotide repeats; Hexa, hexanucleotide repeats; Compound, ≥2 microsatellites interrupted by ≤100 bases. (**b**) Percentage of long and hypervariable class I (≥20 bp) and variable class II (12–19 bp) microsatellites in the genome. (**c**,**d)** Proportion distribution of selected motifs of dinucleotide repeats (**c**) and trinucleotides repeats (**d**). (**e**) Schematic diagram of distribution of trinucleotide repeats on the 12 chromosomes of swamp eel. Trinucleotide repeat loci were presented as short bars on the chromosomes. Numbers of the repeats on each chromosome were indicated above each chromosome. (**f**) Representative image of PCR profiles showed variation of microsatellite alleles in each repeat type. The numbers on the top panel indicated individual animal (1–15) and the alleles were indicated with uppercase letters in the right panel.

To screen microsatellites in the genome of swamp eel, we searched the genome for all potential microsatellite motifs from dinucleotides, trinucleotides, tetranucleotides, pentanucleotides and hexanucleotides. A total of 99,293 microsatellites were identified with average frequency of 179 microsatellites per megabase of genomic sequences (Supplementary Table 1). Of the 99,293 microsatellites, the dinucleotides were the most abundant (70,456) with a proportion of 70.95%, followed by trinucleotides (13,365; 13.46%), tetranucleotides (4,755; 4.79%), pentanucleotides (1,257; 1.27%), hexanucleotides (85; 0.09%) and compound microsatellites (9,375; 9.44%) (Fig. 1a). In the perfect matched repeats, two classes of microsatellites were divided, based on length of the repeat motifs. A total of 37,777 (42.01%) microsatellites were classified into long and hypervariable class I type (≥20 bp) and the remaining 52,141 (57.99%) microsatellites as variable class II type (12–19 bp) (Fig. 1b). The proportion of different microsatellite motifs is not uniform, particularly in the cases of dinucleotides and trinucleotides. Among dinucleotides, AC/GT motifs (55.24%) were most recurrent, followed by AG/CT (11.30%), AT/AT (4.36%) and CG/CG (0.05%) motifs (Fig. 1c). Among the trinucleotides, AAT/ATT (3.16%) motifs were most abundant followed by AAC/GTT (1.22%) and AGG/CCT (1.17%), whereas CCG/CGG (0.01%) motifs were least (Fig. 1d). Moreover, AAAT, AAAAT and AAAAAT were the most abundant repeats in each class. Analysis of physical location and density of microsatellites on the chromosomes showed that distribution of microsatellites across the chromosomes was uniform with regard to a certain motif type, whereas there were variable densities in different microsatellite types across the chromosomes, for example, 112.56–149.02/Mb in dinucleotides, 22.38–27.17/Mb in trinucleotides, 7.51–9.49/Mb in tetranucleotides, 1.79–2.60/Mb in pentanucleotides, and 0.07–0.26/Mb in hexanucleotides (Fig.1e). Finally, PCR analysis indicated that alleles of microsatellites ranged from 2 to 5 in these repeat types (Fig. 1f).

**Figure 2:**
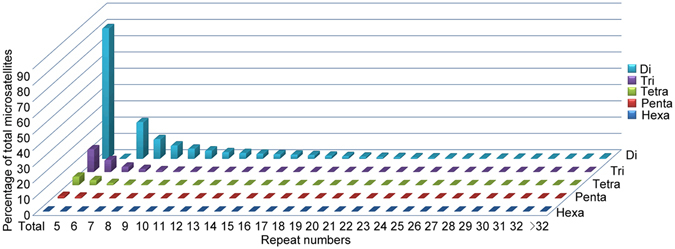
Percentage distribution of microsatellites with different motif types and repeat numbers. The vertical axis showed the abundance of microsatellites with different motif repeat numbers (from 5 to >32). Motif types were indicated in different colors.

We also investigated the microsatellite motif distribution with regard to repeat numbers. For all five microsatellite types, microsatellite frequency decreased as the number of repeat units increased, which was more obvious in long than short microsatellite motifs (Fig. 2). Moreover, the mean repeat number in dinucleotides (10.33) was approximately 1.5 times of those in trinucleotides, tetranucleotides, pentanucleotides and hexanucleotides (6.16, 6.44, 6.37, and 6.99 respectively). The trends are similar to those in the human genome^25^.

### Trinucleotide repeats are most abundant microsatellites in coding regions

**Figure 3:**
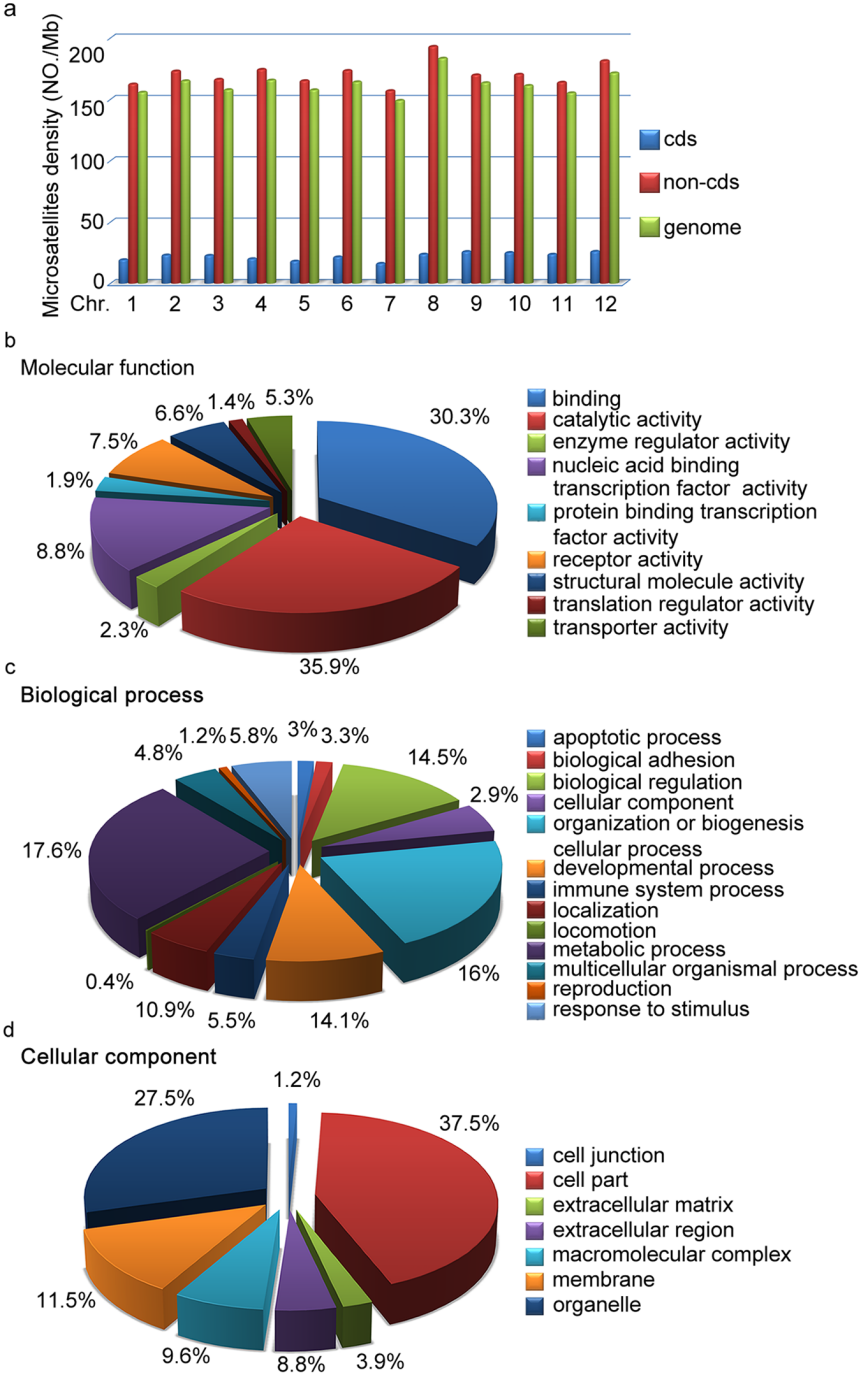
Distribution of microsatellites in coding regions (cds) in the swamp eel genome. (**a**) Microsatellites density in cds and non-cds regions on individual chromosome. (**b**–**d**) Gene ontology classification of microsatellites-containing transcripts. (**b**) Pie chart indicated the percentage of different functional groups in the category of molecular function. (**c**) Pie chart indicated the percentage of different molecular process groups in the category of biological process. (**d**) Pie chart indicated the percentage of different cell part groups in the category of cellular component.

We investigated distribution of microsatellites in both coding and non-coding regions of the genome. Microsatellites were mainly located in non-coding regions (98,602, 99%), whereas there were approximately 1% (691) of the microsatellites located in coding regions (Fig. 3a). In coding regions, trinucleotide repeats were most abundant microsatellites (~75.5%), followed by dinucleotide repeats (20.3%), which represented amino acid repeats in proteins. For these microsatellites in coding regions, we analyzed their GO annotation by Blast2GO. A total of 375 genes were assigned to the molecular function category (Fig. 3b). Catalytic activity (35.9%) was the most dominant group followed by binding (30.3%). Metabolic process (17.6%) was the most enriched group that were annotated to the biological process category (Fig. 3c). With regard to the cellular component, 37.5% sequences were assigned to the cell part followed by organelle (27.5%), membrane (11.5%) and macromolecular complex (9.6%) (Fig. 3d). To investigate whether particular GO terms were overrepresented in microsatellite-containing genes, we performed an overrepresentation analysis (Fisher’s exact test, available through PANTHER version 11.1^26^). No GO term was significantly enriched in microsatellite-containing genes compared to all the other genes in the genome (false discovery rate (FDR) = 0.05). In addition, no chromosome-biased distribution of GO enriched genes was detected (FDR = 0.05).

### Abundant and chromosome-biased microsatellites in non-coding regions

**Figure 4:**
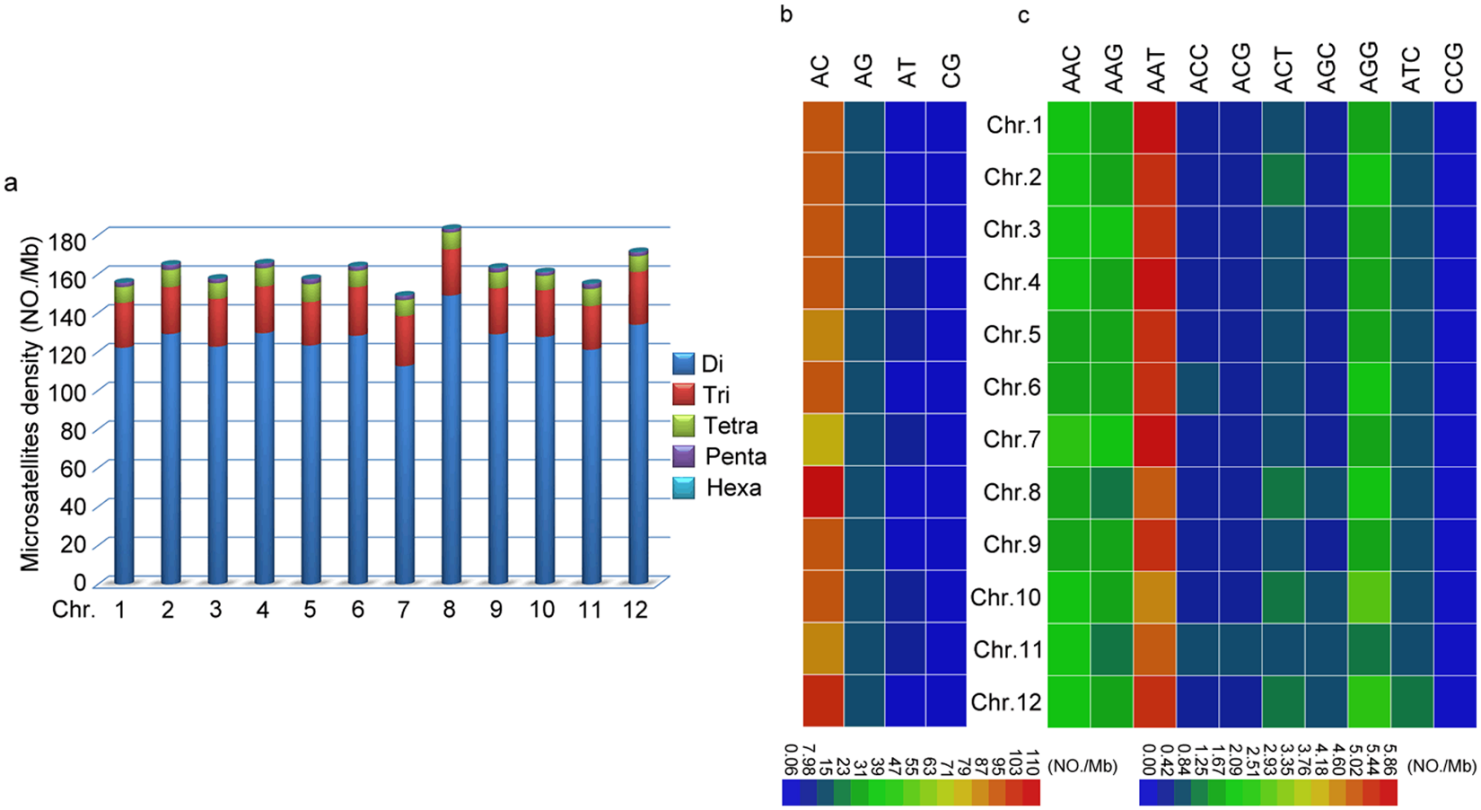
Distribution of microsatellites with different motif types in the swamp eel genome. (**a**) Density of microsatellites with different motif types (Di, dinucleotide repeats; Tri, trinucleotides repeats; Tetra, trinucleotide repeats; Penta, pentanucleotide repeats; Hexa, hexanucleotide repeats) among chromosomes. (**b**,**c**) Heatmap of dinucleotide (**b**) and trinucleotide (**c**) repeats showed their relative numbers among chromosomes. Color key indicated numbers per Mb.

As most of the microsatellites were located in non-coding regions, we analyzed their distribution patterns in the genome. We found that there was a chromosome-biased distribution of these microsatellites in non-coding regions, with the highest density of 203.95/Mb on chromosome 8 and followed by chromosome 12. The least density of the microsatellites was detected on chromosome 7 (164.06/Mb) (Fig. 3a). For the repeat types, dinucleotide repeats were the most abundant class of microsatellites, particularly on the chromosome 8, whereas the chromosome 7 had the least level of distribution of dinucleotide repeats (Fig. 4a). The most abundant repeat type of dinucleotides (AC)n was mainly located on chromosome 8 (Fig. 4b), whereas the most enriched type of trinucleotides was mainly located on chromosome 1, 4 and 7 (Fig. 4c). These results indicated that there were repeat type- and chromosome-biased distributions of the microsatellites in the genome.

### Genomic mapping and their chromosome-biased association of microsatellites with TEs

**Figure 5:**
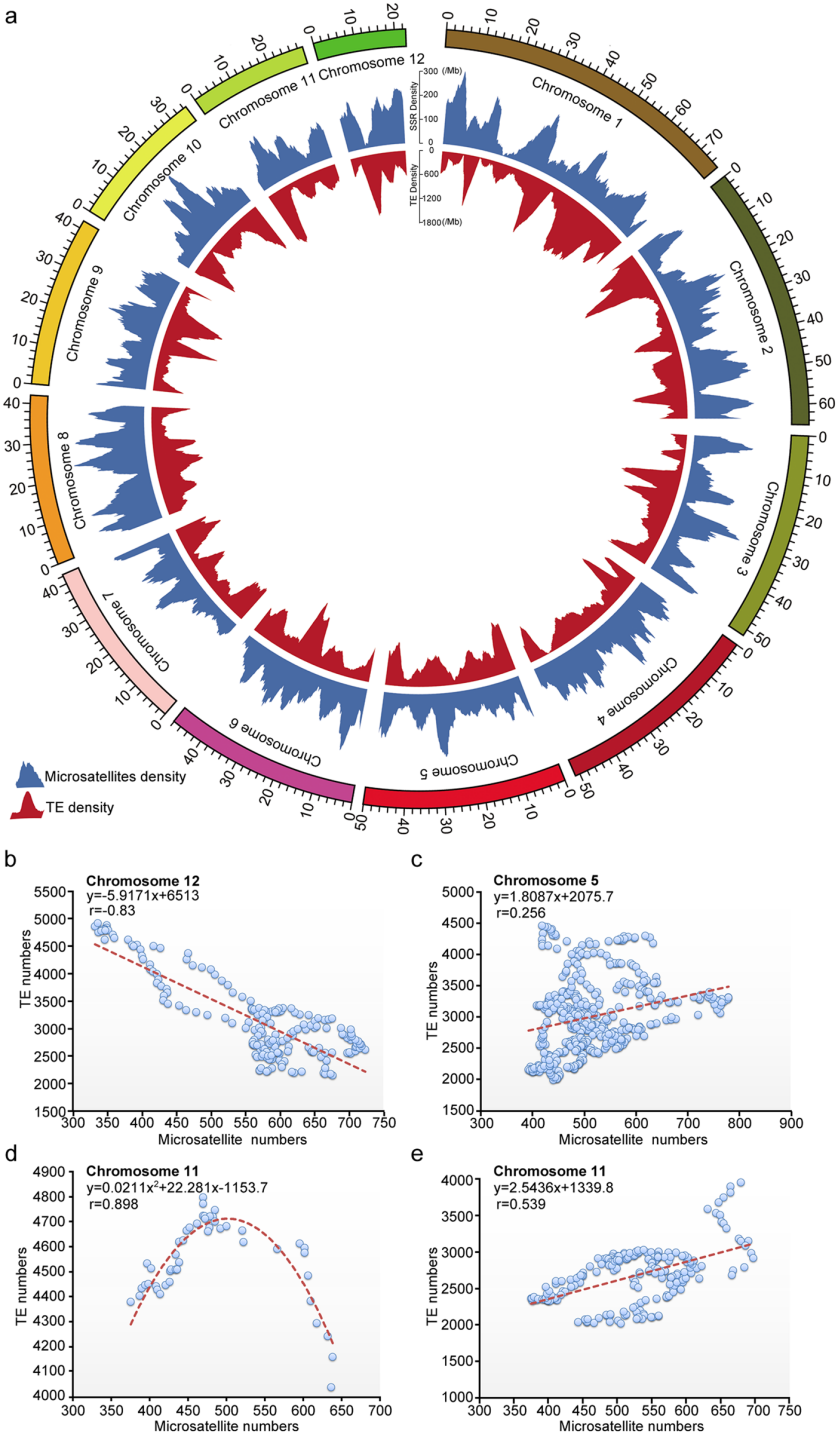
Association analysis between microsatellites and TEs in the genome. Circos was used to plot the assembled chromosomes, microsatellites density and TEs density. The outermost layer showed the chromosomes and the numbers indicated the length in Mb. The middle layer indicated the distribution of microsatellites in 3 Mb windows with 100 kb of step. The inner layer indicated the distribution of TEs in 3 Mb windows with 100 kb of step. (**b**,**c**) Correlation analysis of distribution densities between microsatellites and TEs on chromosome 12 (**b**) and chromosome 5 (**c**). (**d**,**e**) Correlation analysis of distribution densities between microsatellites and TEs on chromosome 11. The data were split into two groups: ≥4000 (**d**) and <4000 (**e**) according to the threshold value of TE numbers in 3 Mb windows on the chromosome 11. The equation and correlation coefficient (r) were indicated in each panel.

As the association between microsatellites and TEs in genomes remains elusive^8, 27–29^, we tested their distributions in the swamp eel genome. Sliding window analysis in a genome-wide, using a window of 3 Mb with a step of 100 kb, showed a distinct distribution pattern among chromosomes (Fig. 5a). Thus, we analyzed correlation between microsatellite and TE densities in individual chromosome using the same sliding window parameters. An obvious negative correlation was observed on chromosome 12 (r = −0.83, p = 1.3813E-51) (Fig. 5b) and also on chromosomes 2, 4 and 7, whereas positive correlation was only detected on chromosome 5 (r = 0.256, p = 1.6817E-8) (Fig. 5c). Notably, on the chromosome 11, there were two types of distribution patterns according to TEs numbers in 3 Mb windows. A quadratic function was observed when TEs ≥4000 (Fig. 5d), whereas a linear correlation detected when <4000 (Fig. 5e), which indicated a threshold value of TE numbers associated with microsatellite density on the chromosome 11. A similar quadratic function was also detected on chromosomes 9 and 10. No obvious association was detected on chromosomes 1, 3, 6 and 8. These results suggested a chromosome-biased association between microsatellites and TEs in the genome.

## Discussion

Swamp eel is an increasingly emerging model species in biology, in addition to its economic importance in fish production^1, 2^. Microsatellites that are widely distributed in a genome are important genetic markers for assessing genetic diversity, genetic map construction, comparative genomics, and marker-assisted selection breeding. Characterization of the genome-wide microsatellites in this study, together with SSR markers from previous reports in swamp eel^22–24, 30, 31^, provides a resourceful dataset for genetic improvement of this species, and genomic and evolutionary biology studies.

The genomic data are excellent sources for SSR mining and has been utilized in various species^9, 25, 32, 33^. In the present study, we identified a total of 99,293 microsatellites based on the whole-genome sequences of swamp eel. The distribution frequency of microsatellites (179/Mb) estimated in the genome is comparable to that documented in buffalo genome (170/Mb)^33^, but lower than those in human and mouse^34^. These differences could be due to the variation in search criteria, sizes of the databases and bioinformatics software tools used in different studies for identification of microsatellites. The most abundant dinucleotide and trinucleotide motifs are AC/GT and AAT/ATT, which are in agreement with those in human^25^ and buffalos^7, 33^, but different from those of cattle and goat^35, 36^. Predominant repeats in various classes are AAT, AGG and AAC in trimers, AAAT, AAAC, and AAAG in tetramers, AAAAT and AAAAC in pentamers and AAAAAT, AAAAAC, AAAAAG and AAAAAG in hexamers. It reflects a prevalence of the A-rich repeats during genome evolution in teleost fishes. The abundance of the repeats is probably influenced by their secondary structures and the effect on DNA replication^25^ or reflects a genetic adaptation to water environment during fish speciation. Thus, the characterization of the microsatellites in the swamp eel provides a useful resource for further studies in genome evolution in the teleost fish species.

The frequency and density of microsatellites are probably correlated with genome sizes. For example, the microsatellite density is higher in large genomes than in small genomes among mammals^37^. However, the microsatellite frequency in plants is lower in large genomes than in small genomes^38^. The distributions of microsatellites also vary in different regions in a genome. It is well known that noncoding regions generally contain more abundant microsatellites than coding regions^39, 40^. There is no apparent difference of microsatellite contents between intergenic regions and introns^41^. In addition, the microsatellite density is higher at the end of chromosome arms than at other regions in human and mouse genomes^42, 43^. Although the trends for different repeat types are similar between chromosomes within a genome, the density of repeats could vary among different chromosomes of the same species. The density is higher on autosomes than on X chromosomes in mammals (such as humans, mice, and rats) but with exception in *Drosophila* ^44^. This can be expected, since different chromosomes in a genome have different organizations of genes, euchromatin, and heterochromatin. This variation is due in part to AT/GC content of genomes, with biased toward either high AT or CG. The bias is favorite for enhancement of expansion through slippage during DNA replication^45^.

Transposition often generates genetic variations, and microsatellites are probably associated with relevant elements^46–48^. Alu elements are widely distributed in the human genome, representing more than 10% of its total size. Since Alu repeats contain a poly(A) tail and a central linker region rich in adenines, there is a certain extent of association with A-rich microsatellites. A significant association was observed between the 3′ end of Alu sequences, not only with (A)n mononucleotide repeats but also with (AAC)n, (AAT)n, and A-rich tetra- to hexanucleotide repeats, moreover, this association was weaker with (AT)n dinucleotide repeats^49^. The (AC)n dinucleotide repeats were preferentially associated with Alu elements, 75% of them were at the 3′ end of the elements, while the remainder were in the central linker region^46^. However, a high density of transposable elements does not always coincide with a high density of microsatellites. For example, analysis in five complete plant genomes showed that microsatellites were preferentially located in unique regions of the genomes and exhibited a lack of association with transposon-rich regions^38^. It was hypothesized that microsatellite can be derived from TEs and the opposite evolutionary direction may occur^50, 51^. The direction of transition from TE to microsatellite might depend on transposition rate with an optimal value and the opposite transition is linked to recombination rate^50, 51^. A chromosome-biased association between microsatellites and TEs in our study is presumably at least partially related to their distant contact and recombination behavior of chromosomes. A chromosome-biased association between microsatellites and TEs in the fish genome observed in this study provides a new layer in understanding of complexity of these repeats in genome structure and evolution.

Microsatellites are closely related to genome stability and regulations of gene expression, expansions of which are risk factors of many genetic disorders in human, such as fragile X syndrome^52^, Huntington’s disease^53^ and myotonic dystrophy^54^. In fishes, a microsatellite marker, (GT)ntt(GT)n, in the 3′ untranslated regions of *rtp3* is significantly associated with nervous necrosis virus disease resistance^55^. Whether there is a particular GO term enrichment in microsatellite-associated genes is an interesting issue. In our study, gene ontology annotation of microsatellite-containing genes revealed that these genes were involved in various aspects of biological activities in swamp eel. In line with this, no GO term was overrepresented in the microsatellite-containing genes compared to total genomic genes. Similar results were reported in functional annotation of microsatellite-containing genes in *Acipenser fulvescens* ^56^ and *Carcharodon carcharias* ^57^. In addition, GO term was not enriched in particular chromosome either. Nevertheless, both microsatellites and TEs are associated with three-dimensional chromosome architecture^58, 59^. Some G-rich TEs and microsatellites can form structures made of four DNA strands known as G-quadruplexes contributing to change in chromatin status, transcription enhancement/inhibition and the evolution of regulatory networks^58, 59^.

## Materials and Methods

### Animals and ethics statement

Swamp eels (*Monopterus albus*) were obtained from markets in Wuhan, China. All animal experiments and methods were performed in accordance with the relevant approved guidelines and regulations, as well as under the approval of the Ethics Committee of Wuhan University.

### Screening and identification of microsatellites

Genomic sequences of swamp eels were sequenced by our lab (DDBJ/EMBL/GenBank under the accession AONE00000000). The Perl script MIcroSAtelitte (MISA, http://pgrc.ipk-gatersleben.de/misa/) was used to identify microsatellites in the genomes. The genomic sequence data were loaded into a local pool. The configuration file was written in an independent text document named as “misa.in” and was placed in the same folder with the Perl script named as “misa.pl”. The sequence of each chromosome was screened for potential motif repeats by calling the genomic sequence data file and the configuration file. To identify the presence of microsatellites, only 2 to 6 nucleotides motifs were considered, and the minimum repeat unit was defined as 6 for dinucleotide repeats, 5 for trinucleotide repeats, 4 for tetranucleotides, and 3 for pentanucleotides and hexanucleotides. Compound microsatellites were defined as ≥2 microsatellites interrupted by ≤100 bases^33^.

### PCR amplification

Total genomic DNA was isolated from gonad samples by previous method^60^. Primers were designed by Primer3 software^61^. PCR amplification was conducted in 25 μl reactions containing 50 ng of template DNA, 2.5 mM MgCl_2_, 2.5 μl 10 × PCR buffer, 0.5 mM each primer, 0.5 U Taq DNA polymerase, and 2.5 mM dNTPs. Primer sequences were listed in Supplementary Table 2. The PCR cycling profile was 95 °C for 5 min, 35 cycles at 94 °C for 30 s, 60 °C for 30 s, 72 °C for 30 s, and a final extension at 72 °C for 5 min.

### Functional assignments of the transcripts containing microsatellites

To assign putative functions to the microsatellite-containing transcripts, Blast2go program was run locally to BLAST against a reference database that stores UniProt entries and their associated Gene Ontology (GO)^62^. The GO categorization results were expressed as three independent hierarchies for biological process, cellular component and molecular function (http://www.geneontology.org/). GO term overrepresentation was analyzed by PANTHER version 11.1^26^ (http://www.pantherdb.org/).

### TE analysis

TE elements were analyzed using previous methods^63^. To analyze density of TEs in swamp eel genome, we combined homology-based and *de novo* approaches. The homology-based approach utilized database Repbase (release 22.01) with RepeatMasker (version 4.0.6)^64^. The *de novo* approach utilized two prediction programs (RepeatModeler version 1.0.8^65^ and LTR-FINDER version 1.0.5) to build the *de novo* repeat libraries based on the genome sequences. The multicopy genes and contaminations were removed from the libraries. Then, the RepeatMasker was used again to find repeats in these repetitive sequence libraries. Finally, we combined all the results generated by these methods and analyzed the density of TEs in the genome.

### Circos program

The Circos program (http://circos.ca) was applied to draw the circos maps. Genomic sequences were assembled into chromosomes. Mapping of transposon elements (TEs) and microsatellites onto chromosomes was performed by calling “circos.conf” files containing locus information. The densities of microsatellites and TEs were described as numbers in a sliding window of 3 Mb with a step of 100 kb.

### Ethics Approval

All animal experiments and methods were performed in accordance with the relevant approved guidelines and regulations, as well as under the approval of the Ethics Committee of Wuhan University.

## Electronic supplementary material

Supplementary information.pdf
