# Supplementary material for: Genome-wide mapping and characterization of microsatellites in the swamp eel genome
Source: Sci Rep. 2017 Jun 9;7:3157. doi: 10.1038/s41598-017-03330-7 (PMC5466649; doi:10.1038/s41598-017-03330-7)
Supplement: Supplementary file 1 — Supplementary information.pdf [file 41598_2017_3330_MOESM1_ESM.pdf]

Supplementary information for

Genome-wide mapping and characterization of microsatellites in the swamp eel genome

Running title: Genome-wide characterization of microsatellites

Zhigang Li, Feng Chen, Chunhua Huang, Weixin Zheng, Chunlai Yu, Hanhua Cheng*, Rongjia Zhou*

Hubei Key Laboratory of Cell Homeostasis, Laboratory of Molecular and Developmental Genetics, College of Life Sciences, Wuhan University, Wuhan 430072, P. R. China

Including:

Table S1

Table S2

Table S1 Microsatellite Information

Microsatellite ID	Microsatellite type	Microsatellites	Size(bp)	Start	End
chr1_1	p2	(CA)30	60	9881	9940
chr1_2	p2	(TG)8	16	12001	12016
chr1_3	p4	(GAGG)5	20	12598	12617
chr1_4	p2	(GT)11	22	36270	36291
chr1_5	p2	(TC)6	12	37054	37065
chr1_6	p2	(AC)7	14	38417	38430
chr1_7	p3	(GAG)5	15	38786	38800
chr1_8	p2	(TC)10	20	42011	42030
chr1_9	p2	(AC)7	14	42892	42905
chr1_10	p2	(AG)6	12	45023	45034
chr1_11	p2	(AG)6	12	45156	45167
chr1_12	p2	(TG)7	14	50547	50560
chr1_13	c	(GC)8(AC)12	40	51817	51856
chr1_14	p2	(CA)12	24	60457	60480
chr1_15	p2	(GT)7	14	66164	66177
chr1_16	p2	(GA)6	12	69838	69849
chr1_17	p2	(AC)16	32	72081	72112
chr1_18	p2	(CA)10	20	78613	78632
chr1_19	p2	(AC)9	18	79674	79691
chr1_20	p2	(AC)19	38	81046	81083
chr1_21	p2	(AC)9	18	84716	84733
chr1_22	p2	(CA)13	26	84849	84874
chr1_23	p2	(TG)21	42	85346	85387
chr1_24	p2	(CA)14	28	85793	85820
chr1_25	p3	(AAT)7	21	87022	87042
chr1_26	p3	(CTC)5	15	97885	97899
chr1_27	p2	(GT)8	16	101415	101430
chr1_28	p2	(CA)9	18	107514	107531
chr1_29	p3	(CAG)5	15	111749	111763
chr1_30	p2	(CA)6	12	117843	117854
chr1_31	p2	(CA)16	32	119772	119803
chr1_32	c	ttttgtcaggaggggtgaacgtgggagatgtccttagctttgggtgggcagt	176	120461	120636
chr1_33	p3	(TCC)5	15	124652	124666
chr1_34	p3	(AGA)5	15	125352	125366
chr1_35	p2	(GT)6	12	127981	127992
chr1_36	c	aggaccatgtgtccctacagagccagggcagccatctgcagcaccgact(	89	135666	135754
chr1_37	p5	(ATAAA)5	25	142504	142528
chr1_38	p2	(AC)12	24	152316	152339
chr1_39	p2	(AG)6	12	159152	159163
chr1_40	p2	(AC)19	38	171117	171154
chr1_41	p2	(AC)11	22	171357	171378
chr1_42	p2	(AC)7	14	175922	175935
chr1_43	c	gagactgtaacagcaaatattagattcatgtatcttcacttcatgggacagaca	100	176080	176179
chr1_44	p2	(CA)13	26	177253	177278
chr1_45	p2	(GT)15	30	186284	186313
chr1_46	p2	(CA)13	26	186490	186515
chr1_47	p2	(GA)8	16	188913	188928
chr1_48	c	(CA)21g(AC)6	55	198309	198363

chr1_49	p2	(CA)16	32	198970	199001
chr1_50	c	GA)6ggcgcctggagacacgagcgtctctcccccctctctctct(CA	72	202320	202391
chr1_51	p2	(TG)8	16	203160	203175
chr1_52	p2	(TG)9	18	203572	203589
chr1_53	p2	(TG)7	14	207164	207177
chr1_54	p2	(CA)12	24	207877	207900
chr1_55	p2	(CA)10	20	208250	208269
chr1_56	p3	(TCA)5	15	209816	209830
chr1_57	p3	(GAT)8	24	212572	212595
chr1_58	p2	(GT)12	24	214107	214130
chr1_59	p4	(AAAG)7	28	218136	218163
chr1_60	c	)8gcgccgtggctgcatgtgtagcgatgacgagggaaggagcgcag(G	75	225103	225177
chr1_61	p2	(TG)17	34	229129	229162
chr1_62	p2	(AC)25	50	242375	242424
chr1_63	c	(GT)18(GA)6	48	248124	248171
chr1_64	c	(TG)6tatg(TA)8	32	272191	272222
chr1_65	p3	(TAT)5	15	281760	281774
chr1_66	p2	(GT)6	12	291886	291897
chr1_67	p2	(GT)10	20	301522	301541
chr1_68	p2	(CA)13	26	306019	306044
chr1_69	p2	(CT)9	18	318494	318511
chr1_70	p2	(GT)11	22	326977	326998
chr1_71	p3	(TAT)7	21	327597	327617
chr1_72	p2	(TG)7	14	338153	338166
chr1_73	p3	(AAG)5	15	339429	339443
chr1_74	c	(AAC)9(AAT)5ac(TAA)5	59	339743	339801
chr1_75	p2	(TC)7	14	346559	346572
chr1_76	p3	(CTC)12	36	347676	347711
chr1_77	p2	(TG)22	44	348463	348506
chr1_78	p2	(CA)6	12	359883	359894
chr1_79	p2	(AC)7	14	364063	364076
chr1_80	p2	(TG)13	26	368405	368430
chr1_81	c	(TG)13c(GT)6	39	371417	371455
chr1_82	p2	(GA)7	14	374608	374621
chr1_83	p2	(GT)14	28	374783	374810
chr1_84	p2	(GT)25	50	377704	377753
chr1_85	p2	(TG)13	26	380411	380436
chr1_86	p2	(AC)13	26	381343	381368
chr1_87	p2	(AC)10	20	382802	382821
chr1_88	p2	(TG)29	58	385666	385723
chr1_89	p2	(AC)19	38	390949	390986
chr1_90	p3	(CAG)5	15	392155	392169
chr1_91	p2	(CA)20	40	393426	393465
chr1_92	p3	(AGA)6	18	402573	402590
chr1_93	p2	(AG)9	18	403741	403758
chr1_94	p2	(GT)11	22	404803	404824
chr1_95	p2	(TG)10	20	413764	413783
chr1_96	p2	(TG)15	30	415199	415228
chr1_97	p2	(TG)17	34	425635	425668
chr1_98	p5	(CAAAT)6	30	435383	435412
chr1_99	c	ttacatgtagcagaggaacgctgttagcatgtgctgatcgacaggctagtc	122	440976	441097
chr1_100	p2	(AC)15	30	442551	442580

chr1_101	c	(AC)13atacat(AC)13	58	442923	442980
chr1_102	p3	(GAG)5	15	445237	445251
chr1_103	p2	(CT)9	18	445583	445600
chr1_104	p2	(AC)18	36	448962	448997
chr1_105	p2	(GT)10	20	453402	453421
chr1_106	p4	(CATT)5	20	457114	457133
chr1_107	p2	(GT)7	14	461519	461532
chr1_108	p2	(AC)6	12	462399	462410
chr1_109	p2	(CA)17	34	463139	463172
chr1_110	p2	(AC)20	40	466358	466397
chr1_111	p2	(AC)7	14	466704	466717
chr1_112	p2	(GA)7	14	466930	466943
chr1_113	p2	(CA)12	24	474580	474603
chr1_114	p3	(TTA)5	15	476617	476631
chr1_115	p3	(ATG)8	24	478026	478049
chr1_116	p2	(AC)8	16	482246	482261
chr1_117	p2	(TG)7	14	486160	486173
chr1_118	p2	(CA)11	22	486413	486434
chr1_119	p2	(CA)11	22	489233	489254
chr1_120	p2	(GA)8	16	490126	490141
chr1_121	p2	(TG)15	30	496893	496922
chr1_122	p4	(CTTT)7	28	499777	499804
chr1_123	p2	(GT)17	34	501115	501148
chr1_124	p2	(CA)8	16	504285	504300
chr1_125	p2	(CA)19	38	506713	506750
chr1_126	p2	(CT)15	30	506879	506908
chr1_127	p2	(AC)8	16	507084	507099
chr1_128	p2	(AC)21	42	510236	510277
chr1_129	p2	(CA)14	28	516771	516798
chr1_130	p2	(AC)10	20	517598	517617
chr1_131	p2	(AC)17	34	517738	517771
chr1_132	p2	(TG)8	16	518952	518967
chr1_133	p2	(CT)6	12	535850	535861
chr1_134	p4	(ATCT)10	40	536359	536398
chr1_135	p2	(TC)6	12	537830	537841
chr1_136	p2	(TC)15	30	539609	539638
chr1_137	p2	(AC)8	16	540369	540384
chr1_138	p2	(GT)18	36	540690	540725
chr1_139	p2	(TG)7	14	542302	542315
chr1_140	p2	(AC)13	26	542466	542491
chr1_141	p2	(AG)7	14	543983	543996
chr1_142	p2	(GT)7	14	544336	544349
chr1_143	p2	(GT)16	32	548075	548106
chr1_144	p2	(AC)8	16	552868	552883
chr1_145	p2	(AT)6	12	553827	553838
chr1_146	p2	(AC)8	16	560086	560101
chr1_147	p2	(TG)6	12	561825	561836
chr1_148	c	(CT)7(CA)21	56	563045	563100
chr1_149	p2	(TG)10	20	564109	564128
chr1_150	p2	(AG)7	14	565054	565067
chr1_151	c	.aggcccagggagcttgtttggaagatgttgggagttgtgatatactggagg	157	568320	568476
chr1_152	p4	(ATGG)5	20	569154	569173

chr1_153	p2	(CA)8	16	570863	570878
chr1_154	p2	(TG)8	16	571959	571974
chr1_155	p2	(GT)13	26	575289	575314
chr1_156	p2	(TG)27	54	575467	575520
chr1_157	p2	(CA)20	40	584896	584935
chr1_158	p2	(GT)23	46	595258	595303
chr1_159	p2	(TG)9	18	595421	595438
chr1_160	p2	(TG)6	12	596894	596905
chr1_161	p2	(AC)8	16	600503	600518
chr1_162	p2	(GT)8	16	602846	602861
chr1_163	p3	(CAG)5	15	603139	603153
chr1_164	p2	(TG)7	14	604090	604103
chr1_165	p2	(CA)14	28	606550	606577
chr1_166	p2	(CA)7	14	612706	612719
chr1_167	p2	(TG)11	22	615498	615519
chr1_168	p2	(TG)11	22	624449	624470
chr1_169	p2	(CA)18	36	627170	627205
chr1_170	p2	(TG)9	18	632170	632187
chr1_171	c	(CA)6g(AC)10	33	633160	633192
chr1_172	p2	(TG)6	12	639473	639484
chr1_173	p2	(TG)6	12	639588	639599
chr1_174	p2	(AC)12	24	640884	640907
chr1_175	p2	(AC)6	12	642630	642641
chr1_176	p2	(TG)6	12	648239	648250
chr1_177	p2	(CT)9	18	648516	648533
chr1_178	p2	(CT)9	18	658236	658253
chr1_179	p2	(AC)7	14	662170	662183
chr1_180	p2	(CA)18	36	663449	663484
chr1_181	p2	(GT)16	32	663642	663673
chr1_182	p4	(GATG)7	28	667418	667445
chr1_183	p2	(CA)8	16	668047	668062
chr1_184	p2	(CA)6	12	668640	668651
chr1_185	p5	(GTATG)6	30	675473	675502
chr1_186	c	gcaactccgcatacttgtcaggcagaaaacattaatcttctttagtagtggag	127	680439	680565
chr1_187	p3	(GAT)11	33	681225	681257
chr1_188	p2	(CT)9	18	691244	691261
chr1_189	p2	(CA)20	40	692657	692696
chr1_190	p2	(GT)10	20	692989	693008
chr1_191	p2	(TG)14	28	693173	693200
chr1_192	p2	(TG)7	14	697466	697479
chr1_193	p2	(TG)6	12	709557	709568
chr1_194	p2	(TC)11	22	717266	717287
chr1_195	p2	(AC)6	12	717563	717574
chr1_196	p2	(GT)6	12	732095	732106
chr1_197	c	gagaagtggtttaatataacaactctcattaacttatacttgatgtgctaccag	142	732883	733024
chr1_198	p2	(AG)7	14	735712	735725
chr1_199	p2	(TG)16	32	740185	740216
chr1_200	p2	(AC)15	30	741898	741927
chr1_201	p3	(ATG)5	15	748063	748077
chr1_202	p3	(CAT)5	15	748940	748954
chr1_203	c	atggatattcattacagaaccattacagcagaggttacacgggacgggag	123	760145	760267
chr1_204	p3	(AAT)5	15	761448	761462

chr1_205	p2	(CA)26	52	769038	769089
chr1_206	p2	(CA)6	12	769309	769320
chr1_207	p2	(TA)7	14	774331	774344
chr1_208	p2	(CA)12	24	776153	776176
chr1_209	p2	(TC)8	16	780653	780668
chr1_210	p2	(CT)6	12	801308	801319
chr1_211	p3	(GAT)7	21	802636	802656
chr1_212	p2	(TG)13	26	804259	804284
chr1_213	p3	(GAA)5	15	811727	811741
chr1_214	p2	(CA)13	26	825742	825767
chr1_215	p2	(TG)10	20	831491	831510
chr1_216	p2	(TG)13	26	838533	838558
chr1_217	p2	(AC)6	12	862733	862744
chr1_218	p2	(AC)18	36	862963	862998
chr1_219	p2	(TG)11	22	869901	869922
chr1_220	p2	(AC)6	12	877845	877856
chr1_221	p2	(GT)8	16	880031	880046
chr1_222	p3	(TTG)5	15	883426	883440
chr1_223	c	(TG)8cagatgacacattcacacattcggttttaaagcttacatac(CA)8	73	889617	889689
chr1_224	p3	(CCA)5	15	891148	891162
chr1_225	p3	(CCA)5	15	891460	891474
chr1_226	p2	(GT)9	18	891824	891841
chr1_227	p2	(GT)20	40	916432	916471
chr1_228	p2	(TG)15	30	927082	927111
chr1_229	p2	(CA)6	12	929742	929753
chr1_230	p2	(AC)7	14	934833	934846
chr1_231	p2	(CA)16	32	935050	935081
chr1_232	p2	(TG)6	12	936279	936290
chr1_233	p2	(AT)15	30	937803	937832
chr1_234	p3	(TTA)7	21	938073	938093
chr1_235	p5	(TTAAA)5	25	938376	938400
chr1_236	p2	(CA)10	20	940676	940695
chr1_237	p2	(AC)7	14	951311	951324
chr1_238	p2	(AC)6	12	953163	953174
chr1_239	p3	(AGC)5	15	962294	962308
chr1_240	p2	(AC)7	14	975581	975594
chr1_241	p2	(TA)7	14	986249	986262
chr1_242	p2	(AC)9	18	987819	987836
chr1_243	p2	(TG)6	12	997150	997161
chr1_244	p2	(AC)6	12	1000674	1000685
chr1_245	c	(TAT)6(TGT)7	39	1011694	1011732
chr1_246	p2	(TG)7	14	1012313	1012326
chr1_247	p4	(AGTA)5	20	1014812	1014831
chr1_248	p2	(GA)16	32	1033654	1033685
chr1_249	p5	(TGTAA)5	25	1042790	1042814
chr1_250	p4	(TTGT)5	20	1049273	1049292
chr1_251	p2	(CT)18	36	1055366	1055401
chr1_252	p2	(GT)8	16	1063519	1063534
chr1_253	p2	(CA)13	26	1067218	1067243
chr1_254	p2	(TG)27	54	1075949	1076002
chr1_255	p2	(GT)6	12	1092780	1092791
chr1_256	p2	(GA)12	24	1093672	1093695

chr1_257	c	gaggagaagtatagcaatgatgat(GAG)6agggaagatgatgaacac[illegible]	148	1096593	1096740
chr1_258	p3	(CCA)5	15	1102532	1102546
chr1_259	p2	(AC)9	18	1107456	1107473
chr1_260	p2	(AG)16	32	1108002	1108033
chr1_261	p2	(TG)10	20	1131356	1131375
chr1_262	p2	(CT)6	12	1137893	1137904
chr1_263	p2	(GT)12	24	1149441	1149464
chr1_264	p3	(TTC)6	18	1151003	1151020
chr1_265	p4	(TGTT)5	20	1155682	1155701
chr1_266	p2	(AG)9	18	1156242	1156259
chr1_267	c	(CA)9(CT)7	32	1157276	1157307
chr1_268	c	(CT)16cact(CA)6	48	1157596	1157643
chr1_269	p3	(AAT)6	18	1161025	1161042
chr1_270	p2	(GT)9	18	1163903	1163920
chr1_271	c	(CT)8ttgtatgaaggtaggtgtt(TG)21	77	1171333	1171409
chr1_272	p2	(TG)11	22	1173401	1173422
chr1_273	p3	(AAC)6	18	1180846	1180863
chr1_274	p4	(AATC)5	20	1181872	1181891
chr1_275	p2	(AC)9	18	1182937	1182954
chr1_276	p2	(CA)50	100	1185447	1185546
chr1_277	p2	(GT)14	28	1191093	1191120
chr1_278	p2	(GT)14	28	1209491	1209518
chr1_279	c	(TG)12(AG)9	42	1212707	1212748
chr1_280	p2	(CA)7	14	1213362	1213375
chr1_281	p3	(AGC)6	18	1213580	1213597
chr1_282	p2	(TC)6	12	1231228	1231239
chr1_283	p2	(TG)7	14	1231876	1231889
chr1_284	p3	(AAT)8	24	1235454	1235477
chr1_285	p2	(TC)7	14	1235901	1235914
chr1_286	p3	(CAA)7	21	1244020	1244040
chr1_287	p2	(TG)6	12	1245970	1245981
chr1_288	p2	(TG)7	14	1250990	1251003
chr1_289	p2	(CA)12	24	1252690	1252713
chr1_290	p2	(CA)6	12	1253639	1253650
chr1_291	p2	(CA)8	16	1253890	1253905
chr1_292	p2	(AC)11	22	1280229	1280250
chr1_293	p3	(AGA)7	21	1297940	1297960
chr1_294	p2	(AC)6	12	1307533	1307544
chr1_295	p2	(TG)6	12	1318401	1318412
chr1_296	p2	(GT)15	30	1321907	1321936
chr1_297	p2	(CA)6	12	1326395	1326406
chr1_298	p2	(TG)6	12	1332566	1332577
chr1_299	p2	(TG)8	16	1333226	1333241
chr1_300	p2	(GT)11	22	1333958	1333979
chr1_301	p2	(TG)11	22	1349121	1349142
chr1_302	p2	(TG)7	14	1349377	1349390
chr1_303	p2	(TG)6	12	1364426	1364437
chr1_304	p2	(CA)13	26	1368894	1368919
chr1_305	p2	(TG)6	12	1389268	1389279
chr1_306	p2	(TG)6	12	1397587	1397598
chr1_307	p2	(AT)8	16	1399489	1399504
chr1_308	p2	(GT)11	22	1400648	1400669

chr1_309	p2	(AC)16	32	1410569	1410600
chr1_310	p2	(GT)18	36	1410758	1410793
chr1_311	c*	(CGCA)7(CA)9*	44	1413672	1413715
chr1_312	p2	(GT)6	12	1421347	1421358
chr1_313	p2	(GA)14	28	1425845	1425872
chr1_314	p2	(TG)9	18	1431162	1431179
chr1_315	p2	(AC)21	42	1433799	1433840
chr1_316	c	aagccccctgtgtgtgtatgtgtgtctctgccctcactgaaaagtgtgtctgg	92	1444963	1445054
chr1_317	p3	(TTG)5	15	1445793	1445807
chr1_318	c	(GTT)10ttgtttagttgtttttgttga(TGT)5	66	1452226	1452291
chr1_319	p4	(AGAC)8	32	1452451	1452482
chr1_320	p2	(AC)14	28	1453499	1453526
chr1_321	p3	(TTA)8	24	1455067	1455090
chr1_322	p2	(AC)18	36	1459128	1459163
chr1_323	p2	(GT)6	12	1468014	1468025
chr1_324	p3	(TGA)7	21	1470000	1470020
chr1_325	p2	(TC)6	12	1470423	1470434
chr1_326	p2	(TG)9	18	1482150	1482167
chr1_327	p2	(AT)7	14	1484575	1484588
chr1_328	p2	(GT)6	12	1503699	1503710
chr1_329	p3	(GAG)6	18	1513198	1513215
chr1_330	p2	(AC)11	22	1514810	1514831
chr1_331	p2	(GT)10	20	1515123	1515142
chr1_332	p2	(AC)6	12	1517018	1517029
chr1_333	p2	(AC)6	12	1519038	1519049
chr1_334	p3	(GAG)5	15	1521050	1521064
chr1_335	p2	(AC)16	32	1526917	1526948
chr1_336	c	tttcattaaactgcatttaatgggttaataaatgtattttacatttaatttggaggt	120	1532214	1532333
chr1_337	c	(TC)15actcact(CA)10	57	1536353	1536409
chr1_338	p2	(TG)16	32	1539319	1539350
chr1_339	p3	(CTC)5	15	1549837	1549851
chr1_340	p2	(GT)13	26	1550640	1550665
chr1_341	p2	(GT)8	16	1552726	1552741
chr1_342	p2	(CA)6	12	1553124	1553135
chr1_343	p3	(TAT)5	15	1553732	1553746
chr1_344	p3	(GCA)5	15	1554210	1554224
chr1_345	c	(TG)8agtgagagt(GA)6	37	1556001	1556037
chr1_346	p3	(ACC)5	15	1559448	1559462
chr1_347	p2	(TG)7	14	1577804	1577817
chr1_348	p2	(AC)6	12	1579328	1579339
chr1_349	p2	(AC)8	16	1579855	1579870
chr1_350	p2	(CA)16	32	1580442	1580473
chr1_351	p2	(AC)12	24	1585745	1585768
chr1_352	p3	(AGA)5	15	1593328	1593342
chr1_353	p2	(TA)6	12	1594944	1594955
chr1_354	p2	(CA)17	34	1595845	1595878
chr1_355	p3	(ATA)5	15	1602980	1602994
chr1_356	p2	(CT)7	14	1609010	1609023
chr1_357	p2	(TG)17	34	1610701	1610734
chr1_358	p2	(GT)6	12	1611478	1611489
chr1_359	p3	(TCC)5	15	1616027	1616041
chr1_360	p4	(TCCA)5	20	1617467	1617486

chr1_361	p4	(ATAG)17	68	1630959	1631026
chr1_362	p4	(TTGA)5	20	1635710	1635729
chr1_363	p2	(GA)7	14	1636902	1636915
chr1_364	p3	(CCT)5	15	1643795	1643809
chr1_365	p3	(TAA)5	15	1647428	1647442
chr1_366	p2	(TG)12	24	1648409	1648432
chr1_367	p3	(GGA)8	24	1658740	1658763
chr1_368	p2	(GT)9	18	1662316	1662333
chr1_369	p2	(AC)8	16	1662516	1662531
chr1_370	p2	(CA)10	20	1662778	1662797
chr1_371	p3	(TAT)6	18	1665288	1665305
chr1_372	p3	(GCT)5	15	1673275	1673289
chr1_373	p2	(CA)8	16	1673774	1673789
chr1_374	p2	(AG)8	16	1683230	1683245
chr1_375	p3	(ATC)5	15	1686791	1686805
chr1_376	p2	(GA)7	14	1688505	1688518
chr1_377	c	(GA)7caggtgagagagggacaggg(GA)7	48	1688663	1688710
chr1_378	p2	(GA)6	12	1689003	1689014
chr1_379	c	caggtgtgagagagacaggtgtgagagagaatggtgagggagagacagg	78	1689118	1689195
chr1_380	c	(GA)7caggtgtgagagagagacaggtgagggagagagacaggtgag	1204	1689443	1690646
chr1_381	p3	(CCT)7	21	1697612	1697632
chr1_382	p2	(AC)34	68	1703525	1703592
chr1_383	p2	(AC)24	48	1703731	1703778
chr1_384	p3	(CTT)7	21	1706341	1706361
chr1_385	p2	(CA)22	44	1713957	1714000
chr1_386	p6	(TCCTGA)5	30	1716771	1716800
chr1_387	p2	(AG)27	54	1721731	1721784
chr1_388	c	(TG)10tatat(AC)7	39	1729354	1729392
chr1_389	p2	(TG)12	24	1731619	1731642
chr1_390	p2	(CA)10	20	1734465	1734484
chr1_391	p3	(ATG)6	18	1742947	1742964
chr1_392	p2	(AC)7	14	1743456	1743469
chr1_393	p2	(TG)6	12	1743996	1744007
chr1_394	p2	(GT)9	18	1752056	1752073
chr1_395	p2	(AC)11	22	1771428	1771449
chr1_396	p2	(CA)7	14	1775870	1775883
chr1_397	p2	(CA)20	40	1781174	1781213
chr1_398	p2	(TG)10	20	1784074	1784093
chr1_399	p2	(AC)12	24	1784238	1784261
chr1_400	c	(CA)9cgggcacgtcatctttgtcactgtcg(TCA)8	68	1792307	1792374
chr1_401	p2	(AC)6	12	1792543	1792554
chr1_402	p2	(CA)13	26	1792732	1792757
chr1_403	p3	(AGA)5	15	1797667	1797681
chr1_404	p2	(CA)6	12	1798333	1798344
chr1_405	p2	(CA)9	18	1800129	1800146
chr1_406	p3	(AAT)6	18	1809329	1809346
chr1_407	p3	(TAA)6	18	1810603	1810620
chr1_408	p2	(AC)8	16	1810847	1810862
chr1_409	p3	(TGA)7	21	1826304	1826324
chr1_410	p2	(AC)6	12	1830869	1830880
chr1_411	p2	(TG)17	34	1832028	1832061
chr1_412	p2	(AC)8	16	1835869	1835884

chr1_413	p2	(AC)6	12	1858870	1858881
chr1_414	p2	(TG)25	50	1859083	1859132
chr1_415	c	(TG)9tacgcgcgcccgtgcgc(TG)10	55	1867174	1867228
chr1_416	p3	(CTC)5	15	1868427	1868441
chr1_417	p2	(GT)8	16	1870339	1870354
chr1_418	p4	(TGTT)5	20	1875935	1875954
chr1_419	p3	(GTT)6	18	1884137	1884154
chr1_420	p2	(CA)11	22	1888360	1888381
chr1_421	p2	(AG)8	16	1892067	1892082
chr1_422	p2	(GA)7	14	1895530	1895543
chr1_423	p2	(TG)6	12	1902723	1902734
chr1_424	p2	(CA)7	14	1911244	1911257
chr1_425	p2	(GA)10	20	1911657	1911676
chr1_426	c	tgtactggaactccctgtccaggacatactgggtccactggcacatggtgg	130	1920321	1920450
chr1_427	p2	(CA)12	24	1938694	1938717
chr1_428	p2	(TG)10	20	1942992	1943011
chr1_429	p2	(GA)8	16	1947925	1947940
chr1_430	p2	(AC)9	18	1951541	1951558
chr1_431	p2	(CA)12	24	1956579	1956602
chr1_432	p2	(TG)6	12	1971689	1971700
chr1_433	c	(AT)10ctatagatagatagatagata(AC)7	55	1974227	1974281
chr1_434	p3	(AGG)5	15	1997253	1997267
chr1_435	p2	(TG)6	12	2021793	2021804
chr1_436	p3	(AGA)9	27	2024519	2024545
chr1_437	p3	(AGA)6	18	2025828	2025845
chr1_438	c*	(CA)8(CAC)7*	35	2042369	2042403
chr1_439	p2	(AT)7	14	2051524	2051537
chr1_440	p2	(GT)7	14	2052242	2052255
chr1_441	p2	(GT)12	24	2061839	2061862
chr1_442	p2	(TC)9	18	2074730	2074747
chr1_443	p4	(GTCT)8	32	2074872	2074903
chr1_444	p2	(CA)6	12	2077350	2077361
chr1_445	c	(AGT)8ac(TAG)6	44	2089629	2089672
chr1_446	p3	(TAT)6	18	2091867	2091884
chr1_447	p2	(CA)14	28	2111796	2111823
chr1_448	p3	(ACT)10	30	2113134	2113163
chr1_449	p2	(AT)8	16	2116824	2116839
chr1_450	p3	(GTG)5	15	2132257	2132271
chr1_451	c	gtctgagaaagaaaagaaggaaggga(GT)6ttgaggaaagatggcaa	169	2132623	2132791
chr1_452	p3	(GGA)5	15	2136010	2136024
chr1_453	p2	(GT)14	28	2139840	2139867
chr1_454	c	atgaacagacccagatggctgacttcagtgcaagcagaatttattttgtgctg	111	2140462	2140572
chr1_455	p3	(GTT)7	21	2142647	2142667
chr1_456	p3	(ATA)6	18	2167939	2167956
chr1_457	p2	(TA)8	16	2170714	2170729
chr1_458	p2	(AT)6	12	2187113	2187124
chr1_459	p3	(GTG)8	24	2188181	2188204
chr1_460	p2	(TA)6	12	2189014	2189025
chr1_461	c	agtagtttgagcctcattattctcgtcactataacaaaactcctgattggccca	100	2190156	2190255
chr1_462	p2	(GT)6	12	2201911	2201922
chr1_463	p2	(TG)18	36	2202037	2202072
chr1_464	p5	(AGCTT)5	25	2203524	2203548

chr1_465	p2	(GT)16	32	2205103	2205134
chr1_466	p2	(AC)19	38	2206165	2206202
chr1_467	p3	(TCC)6	18	2208124	2208141
chr1_468	p3	(TCT)6	18	2220190	2220207
chr1_469	p3	(TCT)13	39	2234821	2234859
chr1_470	c	(GT)12(ATGT)5gta(TG)7tatgtgtgtgta(TG)6	85	2237349	2237433
chr1_471	p2	(AC)9	18	2240996	2241013
chr1_472	p5	(ATTAA)5	25	2244008	2244032
chr1_473	p2	(AG)6	12	2244653	2244664
chr1_474	p2	(AC)7	14	2246673	2246686
chr1_475	p2	(TG)18	36	2251513	2251548
chr1_476	p5	(CCTCT)6	30	2255842	2255871
chr1_477	c	(CT)16cacactctct(CA)9	60	2260894	2260953
chr1_478	p3	(TAA)5	15	2264445	2264459
chr1_479	p2	(TA)9	18	2264573	2264590
chr1_480	p3	(CTC)5	15	2272968	2272982
chr1_481	p2	(TG)14	28	2297873	2297900
chr1_482	c	(TC)8actcactcact(CA)16	59	2298564	2298622
chr1_483	p2	(CT)6	12	2308195	2308206
chr1_484	p2	(TG)6	12	2308845	2308856
chr1_485	p2	(CT)6	12	2308960	2308971
chr1_486	p2	(AC)40	80	2325400	2325479
chr1_487	p5	(TTCAG)6	30	2340464	2340493
chr1_488	p2	(AC)7	14	2350406	2350419
chr1_489	p4	(AGCA)5	20	2354069	2354088
chr1_490	p2	(CT)6	12	2365179	2365190
chr1_491	p2	(GT)14	28	2365672	2365699
chr1_492	p2	(GT)21	42	2368027	2368068
chr1_493	p2	(AC)10	20	2370099	2370118
chr1_494	p2	(TG)10	20	2373136	2373155
chr1_495	p2	(TG)15	30	2374938	2374967
chr1_496	p2	(AC)11	22	2375366	2375387
chr1_497	p3	(TTA)6	18	2382492	2382509
chr1_498	p2	(AC)17	34	2389141	2389174
chr1_499	p2	(TG)7	14	2391987	2392000
chr1_500	p2	(CA)18	36	2392165	2392200
chr1_501	p2	(TG)11	22	2393600	2393621
chr1_502	p2	(AC)9	18	2405499	2405516
chr1_503	p2	(GT)25	50	2432333	2432382
chr1_504	c	(TG)16tatat(TGTA)8	69	2446473	2446541
chr1_505	p2	(TA)7	14	2454530	2454543
chr1_506	p2	(GT)10	20	2469454	2469473
chr1_507	p2	(TG)7	14	2477461	2477474
chr1_508	p2	(TG)11	22	2484401	2484422
chr1_509	p2	(AC)6	12	2491163	2491174
chr1_510	c	(AC)6g(CA)6	25	2492789	2492813
chr1_511	c	(TA)6ttt(TA)6	27	2494457	2494483
chr1_512	p2	(TC)13	26	2494814	2494839
chr1_513	p2	(GA)6	12	2506327	2506338
chr1_514	p3	(GGA)5	15	2533540	2533554
chr1_515	p2	(GT)11	22	2545464	2545485
chr1_516	p2	(CA)17	34	2545680	2545713

chr1_517	p2	(AC)6	12	2547147	2547158
chr1_518	p2	(TG)7	14	2550625	2550638
chr1_519	c	(GT)8gaa(AG)7	33	2552361	2552393
chr1_520	p2	(AC)7	14	2553356	2553369
chr1_521	p2	(TC)11	22	2554038	2554059
chr1_522	p2	(TC)7	14	2556401	2556414
chr1_523	p3	(AAG)5	15	2559582	2559596
chr1_524	p2	(TG)9	18	2566703	2566720
chr1_525	p2	(TG)7	14	2572219	2572232
chr1_526	p2	(TA)6	12	2575018	2575029
chr1_527	p2	(TA)7	14	2577294	2577307
chr1_528	p2	(GT)10	20	2578067	2578086
chr1_529	p3	(AAT)8	24	2581017	2581040
chr1_530	p2	(AC)10	20	2582699	2582718
chr1_531	p2	(TG)12	24	2584765	2584788
chr1_532	p4	(AAAG)7	28	2598604	2598631
chr1_533	p4	(TCCA)5	20	2600491	2600510
chr1_534	p4	(ATGG)5	20	2610736	2610755
chr1_535	p4	(TGGA)5	20	2624613	2624632
chr1_536	p4	(AAAC)5	20	2629081	2629100
chr1_537	p2	(CA)6	12	2646331	2646342
chr1_538	p2	(AC)6	12	2661970	2661981
chr1_539	p2	(TC)7	14	2662470	2662483
chr1_540	p2	(TG)9	18	2666149	2666166
chr1_541	p2	(AC)10	20	2666706	2666725
chr1_542	p2	(TA)10	20	2667934	2667953
chr1_543	p3	(AAT)6	18	2670188	2670205
chr1_544	p2	(GT)10	20	2673704	2673723
chr1_545	p2	(AG)6	12	2675638	2675649
chr1_546	p2	(AC)8	16	2679730	2679745
chr1_547	p3	(GAG)5	15	2684862	2684876
chr1_548	p3	(AAG)12	36	2687224	2687259
chr1_549	p2	(TG)6	12	2689680	2689691
chr1_550	p2	(AC)7	14	2694176	2694189
chr1_551	p2	(CA)16	32	2695926	2695957
chr1_552	p2	(CT)12	24	2697368	2697391
chr1_553	p2	(AC)7	14	2704923	2704936
chr1_554	p2	(AC)19	38	2715805	2715842
chr1_555	p2	(GT)6	12	2716070	2716081
chr1_556	p2	(TG)6	12	2720148	2720159
chr1_557	p3	(TCA)5	15	2720942	2720956
chr1_558	p2	(GT)9	18	2730153	2730170
chr1_559	p4	(CATC)5	20	2734437	2734456
chr1_560	c	(TATG)9(TAGA)5	56	2751479	2751534
chr1_561	p2	(AC)6	12	2758080	2758091
chr1_562	p2	(GT)14	28	2761185	2761212
chr1_563	p2	(GA)6	12	2762339	2762350
chr1_564	p2	(CA)7	14	2763000	2763013
chr1_565	p2	(GT)9	18	2763313	2763330
chr1_566	p2	(AC)7	14	2767913	2767926
chr1_567	p2	(CA)6	12	2768763	2768774
chr1_568	c	(GA)9tgagtgtggtgtgttctt(AG)14	64	2768935	2768998

chr1_569	p2	(GT)19	38	2769482	2769519
chr1_570	p2	(TA)6	12	2769741	2769752
chr1_571	p2	(AC)10	20	2771274	2771293
chr1_572	p2	(TG)7	14	2776770	2776783
chr1_573	p3	(AGG)5	15	2784421	2784435
chr1_574	p2	(AG)10	20	2793807	2793826
chr1_575	p2	(GA)15	30	2801825	2801854
chr1_576	p2	(TG)11	22	2807192	2807213
chr1_577	p2	(CA)20	40	2813958	2813997
chr1_578	p2	(AT)7	14	2815686	2815699
chr1_579	p2	(TG)7	14	2817592	2817605
chr1_580	p2	(AC)6	12	2831981	2831992
chr1_581	p2	(TA)12	24	2839285	2839308
chr1_582	p2	(AG)7	14	2858114	2858127
chr1_583	p2	(GT)18	36	2858284	2858319
chr1_584	p2	(GT)25	50	2858925	2858974
chr1_585	p3	(AGC)6	18	2863967	2863984
chr1_586	p2	(AG)13	26	2864718	2864743
chr1_587	p2	(AC)7	14	2865909	2865922
chr1_588	p4	(AAAT)5	20	2872067	2872086
chr1_589	c*	(CT)13(CTAT)6*	48	2884979	2885026
chr1_590	p2	(TG)6	12	2893630	2893641
chr1_591	p2	(CA)19	38	2897568	2897605
chr1_592	p2	(AC)6	12	2897753	2897764
chr1_593	p2	(TG)10	20	2898281	2898300
chr1_594	p2	(AC)7	14	2899766	2899779
chr1_595	p3	(GAT)5	15	2901522	2901536
chr1_596	p2	(TG)13	26	2903183	2903208
chr1_597	p3	(TAT)8	24	2906982	2907005
chr1_598	p2	(AC)6	12	2912065	2912076
chr1_599	p2	(AC)14	28	2914647	2914674
chr1_600	p2	(GT)7	14	2918769	2918782
chr1_601	p3	(TAA)5	15	2923672	2923686
chr1_602	p2	(AC)7	14	2923988	2924001
chr1_603	p4	(CTTC)6	24	2927656	2927679
chr1_604	p2	(AC)10	20	2932711	2932730
chr1_605	p2	(CA)6	12	2932969	2932980
chr1_606	p2	(AC)7	14	2935231	2935244
chr1_607	p2	(CA)7	14	2936578	2936591
chr1_608	p2	(CA)7	14	2941150	2941163
chr1_609	p2	(CA)11	22	2957710	2957731
chr1_610	p2	(CA)6	12	2961467	2961478
chr1_611	p2	(TG)18	36	2961965	2962000
chr1_612	p2	(GT)8	16	2966753	2966768
chr1_613	p3	(GAT)5	15	2967624	2967638
chr1_614	p2	(AC)11	22	2970501	2970522
chr1_615	p2	(AC)6	12	2970676	2970687
chr1_616	p3	(CCT)5	15	2972876	2972890
chr1_617	p3	(CTG)6	18	2991229	2991246
chr1_618	c	(GAA)7aaagaagaagaaa(AAG)6aaa(AAG)35	160	2999364	2999523
chr1_619	p2	(AC)7	14	3003624	3003637
chr1_620	p2	(AG)6	12	3011588	3011599

chr1_621	p2	(GT)6	12	3031221	3031232
chr1_622	c	ggaactgtcagctgttgtgcagctgttgtttgactgcattagccagtggctag	124	3031370	3031493
chr1_623	p2	(AT)7	14	3033782	3033795
chr1_624	p3	(AAT)6	18	3053501	3053518
chr1_625	p3	(TAT)9	27	3063320	3063346
chr1_626	p2	(TC)13	26	3064807	3064832
chr1_627	p2	(GT)11	22	3066125	3066146
chr1_628	p2	(CA)26	52	3067672	3067723
chr1_629	c	(AC)8(AG)9	34	3067879	3067912
chr1_630	p2	(AC)7	14	3084075	3084088
chr1_631	p2	(TC)7	14	3085428	3085441
chr1_632	p2	(TA)6	12	3087748	3087759
chr1_633	p2	(TG)10	20	3092744	3092763
chr1_634	p2	(TG)26	52	3093823	3093874
chr1_635	p3	(ACT)8	24	3093977	3094000
chr1_636	p3	(AGG)6	18	3104518	3104535
chr1_637	p3	(AAC)5	15	3108625	3108639
chr1_638	p2	(CA)19	38	3111142	3111179
chr1_639	p2	(AC)9	18	3119467	3119484
chr1_640	p2	(TA)6	12	3123540	3123551
chr1_641	c	(TC)8(AC)6	28	3124762	3124789
chr1_642	p2	(GT)7	14	3124944	3124957
chr1_643	p2	(TG)7	14	3128620	3128633
chr1_644	p2	(TG)8	16	3130433	3130448
chr1_645	p2	(TG)8	16	3130670	3130685
chr1_646	p2	(AC)25	50	3130813	3130862
chr1_647	p2	(CA)6	12	3132511	3132522
chr1_648	p2	(CA)7	14	3133845	3133858
chr1_649	p2	(CA)15	30	3134181	3134210
chr1_650	c	(CA)6aaaactctcatgcatgtaaatataaagc(AT)8	56	3138391	3138446
chr1_651	p2	(TG)16	32	3139799	3139830
chr1_652	p2	(TG)6	12	3147937	3147948
chr1_653	c	(TCA)5(TTA)8	39	3148840	3148878
chr1_654	p2	(TG)9	18	3149544	3149561
chr1_655	p2	(AC)8	16	3151160	3151175
chr1_656	p2	(TG)7	14	3152824	3152837
chr1_657	p2	(TG)11	22	3157515	3157536
chr1_658	p3	(CAA)8	24	3158162	3158185
chr1_659	p3	(ATA)5	15	3158401	3158415
chr1_660	p2	(AG)9	18	3159404	3159421
chr1_661	p2	(TG)6	12	3162462	3162473
chr1_662	p2	(TG)7	14	3163162	3163175
chr1_663	c	gagctgagaggtaattatagaagcccggtgcattaatcttctcttccacctc	146	3163495	3163640
chr1_664	p2	(TG)8	16	3164277	3164292
chr1_665	p2	(TG)13	26	3166748	3166773
chr1_666	p2	(TC)8	16	3167950	3167965
chr1_667	c	(AT)8(AC)8	32	3169262	3169293
chr1_668	p2	(GT)8	16	3171540	3171555
chr1_669	p2	(TG)22	44	3173232	3173275
chr1_670	p3	(TTA)7	21	3173601	3173621
chr1_671	p2	(AC)22	44	3173757	3173800
chr1_672	p2	(AC)9	18	3175074	3175091

chr1_673	p2	(TG)15	30	3175775	3175804
chr1_674	p2	(TG)7	14	3179804	3179817
chr1_675	p2	(TG)9	18	3183197	3183214
chr1_676	p3	(CTC)6	18	3184859	3184876
chr1_677	p2	(GT)14	28	3192279	3192306
chr1_678	p3	(GTT)7	21	3192896	3192916
chr1_679	p2	(GT)16	32	3194712	3194743
chr1_680	p2	(CA)16	32	3197116	3197147
chr1_681	c	(AC)6tctg(TC)13agat(AC)8	62	3197386	3197447
chr1_682	p2	(TG)17	34	3201169	3201202
chr1_683	p2	(TG)10	20	3201800	3201819
chr1_684	p2	(GT)9	18	3201925	3201942
chr1_685	c	(CT)28cactctcactctcact(CA)7	86	3202052	3202137
chr1_686	c	ttgtgcacacccagacccgggaattgatgatgtgtgtcgtgcgcatgacga	133	3218923	3219055
chr1_687	c	(TG)24tatg(TA)6	64	3221712	3221775
chr1_688	p2	(TC)18	36	3224787	3224822
chr1_689	p2	(GT)24	48	3225611	3225658
chr1_690	p2	(CA)7	14	3225789	3225802
chr1_691	p2	(CA)9	18	3226497	3226514
chr1_692	p2	(TG)10	20	3229034	3229053
chr1_693	p2	(GT)9	18	3229840	3229857
chr1_694	p2	(CA)7	14	3231971	3231984
chr1_695	c*	(CCA)8(CA)20*	62	3234262	3234323
chr1_696	p2	(TG)14	28	3235258	3235285
chr1_697	p2	(GT)20	40	3236528	3236567
chr1_698	p3	(TTG)5	15	3238978	3238992
chr1_699	p2	(GT)12	24	3244141	3244164
chr1_700	p2	(GT)16	32	3246386	3246417
chr1_701	p3	(ATT)11	33	3268284	3268316
chr1_702	p2	(CA)6	12	3271450	3271461
chr1_703	p2	(TG)24	48	3273279	3273326
chr1_704	p4	(TTTA)5	20	3274388	3274407
chr1_705	p2	(CT)9	18	3277363	3277380
chr1_706	c	tgtttgcctggacagtagaggatttttgtttcttgcccttgctgttatgcacacc	122	3296713	3296834
chr1_707	p2	(GT)15	30	3298595	3298624
chr1_708	c	(TG)15(AG)10	50	3299094	3299143
chr1_709	p2	(GT)16	32	3299948	3299979
chr1_710	p2	(TG)8	16	3300087	3300102
chr1_711	p2	(TG)15	30	3301077	3301106
chr1_712	p2	(GT)16	32	3301712	3301743
chr1_713	p2	(TG)9	18	3306867	3306884
chr1_714	c	(TG)20(AG)25	90	3311074	3311163
chr1_715	p2	(AC)10	20	3312790	3312809
chr1_716	p2	(TG)9	18	3314621	3314638
chr1_717	p2	(CA)7	14	3318098	3318111
chr1_718	p2	(CA)6	12	3318443	3318454
chr1_719	p2	(CA)26	52	3318946	3318997
chr1_720	p2	(GT)13	26	3320362	3320387
chr1_721	p2	(GT)11	22	3323507	3323528
chr1_722	p2	(AC)14	28	3326028	3326055
chr1_723	p2	(GT)7	14	3329274	3329287
chr1_724	p3	(CTC)9	27	3329696	3329722

chr1_725	p2	(AC)6	12	3329866	3329877
chr1_726	p2	(AC)7	14	3331354	3331367
chr1_727	p2	(CA)7	14	3331630	3331643
chr1_728	p2	(CA)15	30	3331770	3331799
chr1_729	p2	(TA)6	12	3333542	3333553
chr1_730	p2	(AG)12	24	3335662	3335685
chr1_731	p4	(CATT)5	20	3339052	3339071
chr1_732	p2	(CA)6	12	3341750	3341761
chr1_733	p2	(GT)6	12	3343767	3343778
chr1_734	p2	(AC)28	56	3349766	3349821
chr1_735	p2	(TG)12	24	3352201	3352224
chr1_736	p2	(CT)6	12	3355695	3355706
chr1_737	p2	(TC)6	12	3359150	3359161
chr1_738	p2	(AC)6	12	3362003	3362014
chr1_739	p2	(TG)6	12	3363736	3363747
chr1_740	p2	(GT)6	12	3364593	3364604
chr1_741	p2	(CA)10	20	3367578	3367597
chr1_742	p2	(CA)14	28	3400884	3400911
chr1_743	p2	(AC)35	70	3403529	3403598
chr1_744	p2	(TA)7	14	3406084	3406097
chr1_745	p3	(ATT)6	18	3411281	3411298
chr1_746	p3	(TGG)5	15	3413511	3413525
chr1_747	p3	(AAT)5	15	3417617	3417631
chr1_748	p3	(TTA)6	18	3424223	3424240
chr1_749	c	(TA)8c(AT)9(AC)11	57	3425625	3425681
chr1_750	p2	(GA)6	12	3433290	3433301
chr1_751	p2	(AC)15	30	3443386	3443415
chr1_752	p2	(AC)6	12	3445494	3445505
chr1_753	p2	(GT)10	20	3468351	3468370
chr1_754	p2	(CA)17	34	3470825	3470858
chr1_755	p2	(AC)6	12	3471600	3471611
chr1_756	p2	(GT)12	24	3471767	3471790
chr1_757	p2	(TG)6	12	3471965	3471976
chr1_758	p2	(AC)19	38	3472976	3473013
chr1_759	p2	(CA)11	22	3479477	3479498
chr1_760	p2	(TG)6	12	3484168	3484179
chr1_761	p2	(GT)26	52	3487009	3487060
chr1_762	p2	(AG)10	20	3489020	3489039
chr1_763	p3	(AAT)7	21	3496206	3496226
chr1_764	p2	(TG)7	14	3502794	3502807
chr1_765	p2	(CA)7	14	3504222	3504235
chr1_766	p2	(TG)18	36	3508226	3508261
chr1_767	c	(GT)7gagtgagt(GA)7	36	3521979	3522014
chr1_768	p2	(CT)8	16	3522116	3522131
chr1_769	p4	(TACC)7	28	3523402	3523429
chr1_770	p2	(GT)6	12	3525013	3525024
chr1_771	c	(GT)16(GA)6	44	3532187	3532230
chr1_772	p2	(TG)6	12	3533362	3533373
chr1_773	p3	(CCT)8	24	3533977	3534000
chr1_774	p2	(GT)6	12	3535558	3535569
chr1_775	p2	(GT)8	16	3537789	3537804
chr1_776	p2	(GT)9	18	3545703	3545720

chr1_777	p2	(GT)15	30	3550987	3551016
chr1_778	p4	(AATA)5	20	3553333	3553352
chr1_779	p4	(AACT)7	28	3554959	3554986
chr1_780	p2	(AT)6	12	3558027	3558038
chr1_781	p2	(AT)12	24	3559426	3559449
chr1_782	p2	(AC)31	62	3562045	3562106
chr1_783	p2	(GA)6	12	3580046	3580057
chr1_784	p3	(TGC)5	15	3589949	3589963
chr1_785	p2	(TG)10	20	3607457	3607476
chr1_786	p3	(TGC)6	18	3610166	3610183
chr1_787	p2	(GT)12	24	3610896	3610919
chr1_788	p2	(GT)6	12	3619612	3619623
chr1_789	p2	(GT)12	24	3630835	3630858
chr1_790	p2	(GT)6	12	3634862	3634873
chr1_791	p2	(GT)9	18	3635140	3635157
chr1_792	p2	(TG)10	20	3635377	3635396
chr1_793	p2	(TG)6	12	3638472	3638483
chr1_794	p2	(TG)7	14	3642087	3642100
chr1_795	p2	(AT)9	18	3647941	3647958
chr1_796	p2	(TG)8	16	3649044	3649059
chr1_797	p2	(GA)8	16	3650462	3650477
chr1_798	p2	(TG)6	12	3651009	3651020
chr1_799	p2	(CA)15	30	3656563	3656592
chr1_800	p4	(ATGG)5	20	3674008	3674027
chr1_801	p2	(GA)8	16	3676628	3676643
chr1_802	p2	(AC)6	12	3680101	3680112
chr1_803	p2	(TG)6	12	3685060	3685071
chr1_804	p2	(CA)14	28	3685222	3685249
chr1_805	p2	(TG)8	16	3685625	3685640
chr1_806	c	(TG)6aa(TG)8agac(AG)6	46	3686527	3686572
chr1_807	p2	(TG)12	24	3687867	3687890
chr1_808	p2	(CA)12	24	3688050	3688073
chr1_809	p2	(AC)9	18	3691528	3691545
chr1_810	p2	(CA)6	12	3695531	3695542
chr1_811	p2	(TG)6	12	3697961	3697972
chr1_812	p2	(TC)11	22	3707504	3707525
chr1_813	p2	(CA)11	22	3708694	3708715
chr1_814	p2	(TC)8	16	3712972	3712987
chr1_815	p2	(TC)9	18	3731070	3731087
chr1_816	p3	(AAT)5	15	3733926	3733940
chr1_817	p2	(TG)9	18	3735115	3735132
chr1_818	p3	(AAT)7	21	3735376	3735396
chr1_819	p3	(TTA)8	24	3736321	3736344
chr1_820	p2	(AC)21	42	3737362	3737403
chr1_821	c	(CT)7gtctg(TC)6	31	3738888	3738918
chr1_822	p2	(GT)15	30	3739026	3739055
chr1_823	p2	(TG)6	12	3739451	3739462
chr1_824	c	(TG)15(AG)17aaattgtgagtacttgcgcgcgtgc(GT)6	101	3743298	3743398
chr1_825	p2	(TG)14	28	3743832	3743859
chr1_826	p2	(AC)6	12	3746229	3746240
chr1_827	p3	(CTG)6	18	3751619	3751636
chr1_828	p2	(GT)7	14	3758215	3758228

chr1_829	p2	(AG)7	14	3758930	3758943
chr1_830	p2	(TG)17	34	3760758	3760791
chr1_831	p2	(GT)8	16	3766139	3766154
chr1_832	p2	(GT)6	12	3771042	3771053
chr1_833	p2	(AG)9	18	3772476	3772493
chr1_834	p2	(AC)7	14	3781783	3781796
chr1_835	p4	(GATA)22	88	3808863	3808950
chr1_836	p3	(GAT)5	15	3828452	3828466
chr1_837	p3	(TGA)6	18	3829804	3829821
chr1_838	p3	(GAA)5	15	3835514	3835528
chr1_839	p3	(GTT)5	15	3865442	3865456
chr1_840	p2	(TG)25	50	3867532	3867581
chr1_841	p2	(TG)17	34	3875629	3875662
chr1_842	p2	(GT)23	46	3879183	3879228
chr1_843	p2	(AC)16	32	3881559	3881590
chr1_844	p2	(GA)6	12	3883022	3883033
chr1_845	c	cagctgagagacgaagcatgccaggacaagcatcaggcctgcctctatc	97	3894415	3894511
chr1_846	c	(TG)9tc(TG)8	36	3894756	3894791
chr1_847	p2	(GT)7	14	3900419	3900432
chr1_848	p2	(TG)10	20	3902565	3902584
chr1_849	p2	(AC)9	18	3925507	3925524
chr1_850	p2	(GT)12	24	3927339	3927362
chr1_851	p2	(TA)6	12	3928691	3928702
chr1_852	p3	(TAA)11	33	3932942	3932974
chr1_853	p2	(AC)9	18	3936655	3936672
chr1_854	p2	(AT)6	12	3937364	3937375
chr1_855	c	gctgatgttctgtgtaagaaggcaaggtgctcgctgttacacgaccagcag	125	3950354	3950478
chr1_856	c	(TA)8tgtatatatgtatatgtac(GT)6t(TG)6	60	3962960	3963019
chr1_857	p2	(CA)15	30	3969145	3969174
chr1_858	c	(CT)9cc(CT)6	32	3976175	3976206
chr1_859	p4	(TTTG)6	24	3989670	3989693
chr1_860	p2	(GT)10	20	4007283	4007302
chr1_861	p3	(TTG)7	21	4014596	4014616
chr1_862	p2	(TG)6	12	4027945	4027956
chr1_863	p2	(CA)15	30	4037762	4037791
chr1_864	p2	(TG)10	20	4040390	4040409
chr1_865	p2	(AC)11	22	4052146	4052167
chr1_866	p2	(TG)15	30	4082773	4082802
chr1_867	p2	(TG)8	16	4087371	4087386
chr1_868	p3	(CCT)10	30	4089520	4089549
chr1_869	p2	(TG)10	20	4090177	4090196
chr1_870	p2	(GT)18	36	4090617	4090652
chr1_871	p2	(AG)8	16	4092736	4092751
chr1_872	p2	(TG)6	12	4094259	4094270
chr1_873	p4	(GTCA)5	20	4097484	4097503
chr1_874	p2	(TG)6	12	4098213	4098224
chr1_875	p2	(TG)6	12	4099565	4099576
chr1_876	p4	(TTTA)6	24	4100481	4100504
chr1_877	c*	(CTCACA)5(CA)7*	40	4100683	4100722
chr1_878	p4	(GTAG)6	24	4103961	4103984
chr1_879	p2	(CT)6	12	4107346	4107357
chr1_880	p3	(TGC)7	21	4121719	4121739

chr1_881	p2	(TG)14	28	4124069	4124096
chr1_882	p2	(TA)6	12	4127135	4127146
chr1_883	p2	(CA)6	12	4134164	4134175
chr1_884	p2	(GT)7	14	4148536	4148549
chr1_885	p2	(TG)11	22	4154567	4154588
chr1_886	p3	(TTG)5	15	4160121	4160135
chr1_887	p2	(CA)15	30	4163142	4163171
chr1_888	p2	(TG)18	36	4165398	4165433
chr1_889	c*	(GT)19(GTGA)5*	56	4165634	4165689
chr1_890	p2	(AC)9	18	4167364	4167381
chr1_891	p2	(CA)8	16	4175008	4175023
chr1_892	p2	(CA)13	26	4177343	4177368
chr1_893	p2	(CA)6	12	4178304	4178315
chr1_894	p2	(AC)13	26	4179596	4179621
chr1_895	p2	(TG)9	18	4184011	4184028
chr1_896	p2	(AC)11	22	4184250	4184271
chr1_897	p2	(AC)8	16	4184604	4184619
chr1_898	p2	(AG)6	12	4185063	4185074
chr1_899	p2	(TG)7	14	4193612	4193625
chr1_900	p2	(TG)10	20	4200999	4201018
chr1_901	p2	(GT)7	14	4206682	4206695
chr1_902	p2	(AG)7	14	4221631	4221644
chr1_903	p5	(GATTT)5	25	4223482	4223506
chr1_904	p3	(CCT)5	15	4228975	4228989
chr1_905	p2	(GT)6	12	4236887	4236898
chr1_906	p2	(CA)6	12	4254483	4254494
chr1_907	p2	(CA)6	12	4261662	4261673
chr1_908	c	(CA)30c(CA)6	73	4280113	4280185
chr1_909	p2	(CT)6	12	4280877	4280888
chr1_910	p4	(TTTA)5	20	4284278	4284297
chr1_911	p2	(GT)7	14	4294010	4294023
chr1_912	p2	(TG)9	18	4294226	4294243
chr1_913	p2	(CT)6	12	4303940	4303951
chr1_914	p2	(TG)6	12	4306874	4306885
chr1_915	p2	(AC)6	12	4340754	4340765
chr1_916	p2	(TA)8	16	4342687	4342702
chr1_917	p2	(TA)8	16	4344253	4344268
chr1_918	p2	(TG)12	24	4346224	4346247
chr1_919	p2	(CA)13	26	4346585	4346610
chr1_920	p2	(TG)10	20	4346731	4346750
chr1_921	p2	(AC)6	12	4348356	4348367
chr1_922	p2	(AC)22	44	4353509	4353552
chr1_923	c	(AT)7(AC)6	26	4363633	4363658
chr1_924	p2	(CA)6	12	4366580	4366591
chr1_925	p2	(TA)7	14	4368614	4368627
chr1_926	p4	(AAAC)6	24	4372718	4372741
chr1_927	p2	(TG)8	16	4384095	4384110
chr1_928	p2	(CA)19	38	4389899	4389936
chr1_929	p2	(AC)19	38	4390160	4390197
chr1_930	p2	(AC)6	12	4393216	4393227
chr1_931	p3	(TCC)5	15	4393801	4393815
chr1_932	p2	(GT)7	14	4398270	4398283

chr1_933	p3	(TTG)5	15	4401524	4401538
chr1_934	p2	(AC)9	18	4410961	4410978
chr1_935	p2	(AC)16	32	4420452	4420483
chr1_936	p3	(GAT)6	18	4426898	4426915
chr1_937	c	(TG)11c(GT)12(GA)6	59	4432429	4432487
chr1_938	p2	(TG)8	16	4435612	4435627
chr1_939	p2	(GT)9	18	4437882	4437899
chr1_940	p2	(TG)23	46	4439156	4439201
chr1_941	p2	(AC)7	14	4445689	4445702
chr1_942	p3	(TAA)6	18	4448120	4448137
chr1_943	p2	(GT)9	18	4452522	4452539
chr1_944	p2	(CA)6	12	4457021	4457032
chr1_945	p2	(GT)7	14	4458675	4458688
chr1_946	p2	(TG)8	16	4462802	4462817
chr1_947	p2	(CA)12	24	4463301	4463324
chr1_948	p2	(GT)7	14	4472580	4472593
chr1_949	c	atatgtgcatctgtgaaagtatttttaattcctactgttctttgaatgcatgtacc	115	4473092	4473206
chr1_950	p2	(TG)12	24	4473727	4473750
chr1_951	p2	(GA)13	26	4476134	4476159
chr1_952	p2	(AC)7	14	4478758	4478771
chr1_953	p2	(TG)11	22	4479445	4479466
chr1_954	p2	(TG)9	18	4481398	4481415
chr1_955	p2	(GT)7	14	4484090	4484103
chr1_956	p2	(CA)6	12	4496333	4496344
chr1_957	p3	(TTG)5	15	4515118	4515132
chr1_958	p3	(TTA)5	15	4521046	4521060
chr1_959	c	(CA)30c(CA)6	73	4526407	4526479
chr1_960	p2	(AC)8	16	4531540	4531555
chr1_961	p3	(CTG)5	15	4536367	4536381
chr1_962	p2	(CA)10	20	4545311	4545330
chr1_963	p2	(CA)12	24	4550450	4550473
chr1_964	p2	(TG)6	12	4551606	4551617
chr1_965	p2	(AC)9	18	4551727	4551744
chr1_966	p2	(AC)8	16	4554561	4554576
chr1_967	p2	(TG)6	12	4558989	4559000
chr1_968	c	(TG)8tatgc(GT)15	51	4564178	4564228
chr1_969	p2	(AC)12	24	4564982	4565005
chr1_970	p4	(TTTA)5	20	4565466	4565485
chr1_971	p5	(AGTTG)5	25	4566650	4566674
chr1_972	p2	(AC)7	14	4571660	4571673
chr1_973	p2	(CA)8	16	4577676	4577691
chr1_974	p2	(TG)11	22	4578755	4578776
chr1_975	p2	(TG)6	12	4580607	4580618
chr1_976	p2	(CA)6	12	4586051	4586062
chr1_977	p2	(TG)7	14	4587181	4587194
chr1_978	p2	(AC)10	20	4587316	4587335
chr1_979	p3	(AAC)5	15	4590570	4590584
chr1_980	p2	(GT)9	18	4595912	4595929
chr1_981	p2	(TG)16	32	4596037	4596068
chr1_982	p2	(AC)10	20	4601860	4601879
chr1_983	p3	(GAG)7	21	4619882	4619902
chr1_984	p2	(TG)11	22	4622181	4622202

chr1_985	p2	(GT)6	12	4623424	4623435
chr1_986	p3	(GAA)5	15	4628093	4628107
chr1_987	p2	(TG)6	12	4630896	4630907
chr1_988	p2	(AC)24	48	4637632	4637679
chr1_989	c	(AC)6aa(AC)18	50	4644507	4644556
chr1_990	p2	(CA)22	44	4654661	4654704
chr1_991	p2	(AC)19	38	4655341	4655378
chr1_992	c	tggttgttgcatcgttctataaagtacaggtagtggatggctctgttcttggcct	158	4656273	4656430
chr1_993	p2	(GT)8	16	4657535	4657550
chr1_994	p2	(CA)20	40	4661939	4661978
chr1_995	p2	(CA)19	38	4662432	4662469
chr1_996	p2	(AC)6	12	4663738	4663749
chr1_997	p3	(TTG)6	18	4671740	4671757
chr1_998	p2	(TG)17	34	4672959	4672992
chr1_999	c	(AC)6t(CA)11	35	4676636	4676670
chr1_1000	c	(TC)6ttt(CA)9	33	4678808	4678840
chr1_1001	p2	(CA)7	14	4679645	4679658
chr1_1002	p2	(TG)12	24	4696987	4697010
chr1_1003	p2	(GT)9	18	4697387	4697404
chr1_1004	p2	(TG)7	14	4699548	4699561
chr1_1005	p2	(CT)6	12	4707730	4707741
chr1_1006	c	acagtttgggtaaatgggtgtgtgtgcatacagaaaaggtttttgatgagaga	116	4716882	4716997
chr1_1007	p2	(GT)11	22	4719644	4719665
chr1_1008	p2	(CA)8	16	4719799	4719814
chr1_1009	p3	(AAG)10	30	4739360	4739389
chr1_1010	c	gcaatctg(GT)7tgttcctttgcttaggttaaaggcaaatgtgaagagccaa	157	4739669	4739825
chr1_1011	p2	(CA)15	30	4743435	4743464
chr1_1012	p2	(AC)17	34	4745700	4745733
chr1_1013	p2	(TG)9	18	4746363	4746380
chr1_1014	p4	(TCCA)5	20	4750764	4750783
chr1_1015	p2	(TG)11	22	4752405	4752426
chr1_1016	p2	(GT)12	24	4757094	4757117
chr1_1017	p2	(AC)11	22	4757359	4757380
chr1_1018	p2	(GA)6	12	4761281	4761292
chr1_1019	p2	(TG)15	30	4766179	4766208
chr1_1020	p2	(TG)8	16	4775386	4775401
chr1_1021	p2	(AC)7	14	4775530	4775543
chr1_1022	p4	(AATC)6	24	4777969	4777992
chr1_1023	p3	(GAT)5	15	4790812	4790826
chr1_1024	p4	(ATGG)5	20	4803283	4803302
chr1_1025	p3	(TAC)5	15	4806583	4806597
chr1_1026	p2	(AC)15	30	4810671	4810700
chr1_1027	p2	(AT)8	16	4811389	4811404
chr1_1028	p2	(TG)14	28	4813987	4814014
chr1_1029	p2	(CT)7	14	4817508	4817521
chr1_1030	p2	(CT)7	14	4820190	4820203
chr1_1031	p2	(TG)15	30	4822581	4822610
chr1_1032	p2	(CA)16	32	4822720	4822751
chr1_1033	p2	(GA)10	20	4823672	4823691
chr1_1034	c	(TG)6cgcaaatgcgtttgtctgcctgcccatgtgcttatc(TG)9	66	4825908	4825973
chr1_1035	p2	(AC)15	30	4826156	4826185
chr1_1036	p2	(CA)19	38	4826348	4826385

chr1_1037	p2	(TC)6	12	4828090	4828101
chr1_1038	c	tgtatgtctgcatgtttg(GT)6ctgatgcaacacaaaaagcgaacatcct	108	4828790	4828897
chr1_1039	p2	(TC)15	30	4829188	4829217
chr1_1040	p3	(AGT)5	15	4830094	4830108
chr1_1041	p2	(TG)8	16	4833444	4833459
chr1_1042	c	(CA)14cg(CA)6	42	4834328	4834369
chr1_1043	p2	(TC)6	12	4835084	4835095
chr1_1044	c	(TG)10c(GT)7	35	4840356	4840390
chr1_1045	c	(AC)8(TC)6	28	4841086	4841113
chr1_1046	c	atggggaatcccttagcaaatgaaataaaaataaaccatgtgtgaaacttgt	162	4843429	4843590
chr1_1047	p2	(AC)9	18	4843702	4843719
chr1_1048	p2	(AC)11	22	4848201	4848222
chr1_1049	p2	(AC)11	22	4850067	4850088
chr1_1050	p4	(AATC)5	20	4852293	4852312
chr1_1051	c	:atttgtgtgtccgtgtgcaaatgaaagaaagagagagagggagagcaaa(	82	4853894	4853975
chr1_1052	p2	(CT)6	12	4856277	4856288
chr1_1053	p2	(GT)11	22	4858838	4858859
chr1_1054	p2	(CA)6	12	4859010	4859021
chr1_1055	p2	(GA)12	24	4861354	4861377
chr1_1056	c	(GT)10gc(GT)10	42	4861481	4861522
chr1_1057	p2	(AC)6	12	4862679	4862690
chr1_1058	p3	(TGT)5	15	4862821	4862835
chr1_1059	p2	(TC)6	12	4867260	4867271
chr1_1060	c	(TG)7ta(TG)8	32	4868811	4868842
chr1_1061	p2	(TG)6	12	4876050	4876061
chr1_1062	p2	(TG)13	26	4877860	4877885
chr1_1063	c	(GT)7gc(GT)6	28	4878211	4878238
chr1_1064	c	(TAT)10catc(ATT)7	55	4879489	4879543
chr1_1065	p2	(GT)20	40	4880018	4880057
chr1_1066	p2	(CA)7	14	4886049	4886062
chr1_1067	p2	(AG)9	18	4892187	4892204
chr1_1068	p2	(TG)9	18	4901268	4901285
chr1_1069	p2	(CA)7	14	4904150	4904163
chr1_1070	p2	(GT)9	18	4904316	4904333
chr1_1071	p2	(AC)8	16	4905865	4905880
chr1_1072	p2	(AC)7	14	4906026	4906039
chr1_1073	p2	(CA)7	14	4906690	4906703
chr1_1074	p2	(TG)10	20	4909334	4909353
chr1_1075	p2	(CA)13	26	4927180	4927205
chr1_1076	p2	(CA)10	20	4930916	4930935
chr1_1077	c	(TG)13(GT)9	44	4931086	4931129
chr1_1078	p2	(GT)9	18	4931258	4931275
chr1_1079	c	ggagaaatcaagtgggtgaaacattgtgtgtatgtgtgaaggtgtactgaca	96	4931403	4931498
chr1_1080	p2	(AC)7	14	4942229	4942242
chr1_1081	p2	(TG)7	14	4945973	4945986
chr1_1082	p4	(TCCC)5	20	4946818	4946837
chr1_1083	c	(TG)15tatgtgtgtgta(TG)6	54	4950303	4950356
chr1_1084	p2	(AC)7	14	4950541	4950554
chr1_1085	p2	(AT)6	12	4952330	4952341
chr1_1086	p3	(AGG)5	15	4956134	4956148
chr1_1087	p2	(CA)7	14	4960068	4960081
chr1_1088	p2	(TG)8	16	4962594	4962609

chr1_1089	p3	(AGA)5	15	4970178	4970192
chr1_1090	p2	(AC)16	32	4971572	4971603
chr1_1091	p2	(CA)11	22	4979985	4980006
chr1_1092	p3	(CCT)5	15	4992404	4992418
chr1_1093	p2	(CA)8	16	4996397	4996412
chr1_1094	p3	(TGC)5	15	5008419	5008433
chr1_1095	p2	(CA)7	14	5010997	5011010
chr1_1096	p2	(GT)20	40	5033480	5033519
chr1_1097	p2	(GT)7	14	5035413	5035426
chr1_1098	p2	(AC)15	30	5036673	5036702
chr1_1099	p2	(AC)33	66	5037990	5038055
chr1_1100	p2	(AC)12	24	5039095	5039118
chr1_1101	p2	(AC)8	16	5039531	5039546
chr1_1102	p3	(AAC)5	15	5059268	5059282
chr1_1103	p2	(AC)9	18	5059562	5059579
chr1_1104	p2	(GT)9	18	5064402	5064419
chr1_1105	p2	(GA)6	12	5066164	5066175
chr1_1106	p2	(GT)7	14	5071817	5071830
chr1_1107	p3	(TGT)5	15	5072551	5072565
chr1_1108	p2	(CA)20	40	5073971	5074010
chr1_1109	p5	(GATTT)6	30	5074305	5074334
chr1_1110	p2	(TG)6	12	5077129	5077140
chr1_1111	p3	(TAC)5	15	5080263	5080277
chr1_1112	p2	(AC)9	18	5085854	5085871
chr1_1113	p2	(CA)7	14	5109339	5109352
chr1_1114	p2	(CA)13	26	5109619	5109644
chr1_1115	p2	(CA)8	16	5110377	5110392
chr1_1116	p2	(AC)17	34	5110670	5110703
chr1_1117	p3	(CAT)6	18	5110897	5110914
chr1_1118	c	;tatgtattctaaaagaaacgcaatcttttgctcacgcctgcgctttctctcgca	106	5115333	5115438
chr1_1119	p2	(CA)12	24	5137404	5137427
chr1_1120	p2	(TG)13	26	5146375	5146400
chr1_1121	p2	(GT)11	22	5149605	5149626
chr1_1122	p5	(CAAAT)6	30	5155066	5155095
chr1_1123	p2	(TC)10	20	5160131	5160150
chr1_1124	p2	(AC)10	20	5176001	5176020
chr1_1125	c	tttcttgtgtaggcatgtacaaggtcactgaatgcatgcctttatcctcataca	147	5192190	5192336
chr1_1126	p2	(GT)7	14	5194972	5194985
chr1_1127	c	(TC)6cctc(TG)16	48	5195503	5195550
chr1_1128	p2	(GT)15	30	5205274	5205303
chr1_1129	p2	(TG)14	28	5205430	5205457
chr1_1130	c	(TC)12(AC)10	44	5209148	5209191
chr1_1131	c	(CTAT)5(CTGT)8	52	5210998	5211049
chr1_1132	p2	(AG)6	12	5217432	5217443
chr1_1133	p2	(TG)7	14	5218236	5218249
chr1_1134	p3	(TAA)12	36	5224223	5224258
chr1_1135	p3	(ATC)5	15	5225524	5225538
chr1_1136	p2	(AC)6	12	5225907	5225918
chr1_1137	p2	(AC)6	12	5236900	5236911
chr1_1138	p2	(CA)10	20	5237048	5237067
chr1_1139	p2	(TG)6	12	5237905	5237916
chr1_1140	p2	(TG)8	16	5240846	5240861

chr1_1141	p2	(GT)7	14	5243231	5243244
chr1_1142	p2	(AC)7	14	5245057	5245070
chr1_1143	p2	(GT)16	32	5245606	5245637
chr1_1144	p2	(TG)6	12	5254187	5254198
chr1_1145	p5	(ACAAA)5	25	5257760	5257784
chr1_1146	c	(GT)13(GA)9	44	5264268	5264311
chr1_1147	p2	(CA)19	38	5274962	5274999
chr1_1148	p4	(ATGG)5	20	5277941	5277960
chr1_1149	p2	(AC)9	18	5278351	5278368
chr1_1150	p2	(TA)6	12	5280422	5280433
chr1_1151	p4	(ATTT)5	20	5281393	5281412
chr1_1152	p2	(TG)15	30	5283395	5283424
chr1_1153	c	;tccacacgtcaaatgagccgagagcca(AAC)5aggagttggagcata	174	5299420	5299593
chr1_1154	p2	(TG)11	22	5307323	5307344
chr1_1155	c	(TCTA)18tct(TCCA)5	95	5308330	5308424
chr1_1156	p2	(TG)7	14	5316333	5316346
chr1_1157	p3	(CCA)5	15	5316640	5316654
chr1_1158	p3	(TCT)14	42	5321965	5322006
chr1_1159	c	cataattagcagggtgtctatgcttgagtggttgtgtgttgagcagtggagtg	108	5332522	5332629
chr1_1160	p2	(GT)10	20	5333793	5333812
chr1_1161	p2	(CT)10	20	5335343	5335362
chr1_1162	p2	(TG)6	12	5336473	5336484
chr1_1163	c	(TG)6ca(TG)9	32	5340053	5340084
chr1_1164	p2	(AC)11	22	5343582	5343603
chr1_1165	p2	(AC)11	22	5346920	5346941
chr1_1166	p2	(AC)6	12	5354943	5354954
chr1_1167	p2	(TG)8	16	5357603	5357618
chr1_1168	p2	(GT)7	14	5362083	5362096
chr1_1169	p2	(GT)19	38	5364921	5364958
chr1_1170	p2	(GA)16	32	5369041	5369072
chr1_1171	p2	(CT)6	12	5370182	5370193
chr1_1172	p2	(CT)6	12	5372448	5372459
chr1_1173	p2	(AC)9	18	5375268	5375285
chr1_1174	p2	(GA)8	16	5375460	5375475
chr1_1175	p2	(TG)6	12	5377308	5377319
chr1_1176	p2	(CA)19	38	5379955	5379992
chr1_1177	p2	(AC)14	28	5383417	5383444
chr1_1178	p2	(TC)6	12	5387447	5387458
chr1_1179	p2	(CA)14	28	5389880	5389907
chr1_1180	p2	(AG)10	20	5390094	5390113
chr1_1181	c	tgtgagggggcagcgagatccctgcatgcagggtttaacagctaataccc	144	5390842	5390985
chr1_1182	p2	(CA)6	12	5391188	5391199
chr1_1183	p2	(TG)28	56	5391302	5391357
chr1_1184	p2	(AC)9	18	5392926	5392943
chr1_1185	p2	(AC)6	12	5397952	5397963
chr1_1186	p2	(AC)18	36	5402398	5402433
chr1_1187	p2	(GT)12	24	5404315	5404338
chr1_1188	p2	(GT)12	24	5405298	5405321
chr1_1189	p2	(AC)6	12	5406507	5406518
chr1_1190	p3	(CTT)5	15	5408168	5408182
chr1_1191	p2	(TG)11	22	5408295	5408316
chr1_1192	p2	(CT)10	20	5411394	5411413

chr1_1193	p2	(AC)35	70	5411759	5411828
chr1_1194	p2	(TC)7	14	5412288	5412301
chr1_1195	c	(GT)12gc(GT)21	68	5414538	5414605
chr1_1196	p2	(AC)6	12	5416082	5416093
chr1_1197	p2	(CA)10	20	5416532	5416551
chr1_1198	p2	(GA)6	12	5418322	5418333
chr1_1199	p2	(AT)6	12	5418562	5418573
chr1_1200	p2	(TG)6	12	5422036	5422047
chr1_1201	p2	(AC)12	24	5432329	5432352
chr1_1202	p2	(AC)6	12	5433947	5433958
chr1_1203	p3	(ACA)5	15	5437360	5437374
chr1_1204	c	(AC)22aaagcgt(GA)6	63	5440064	5440126
chr1_1205	c	(CT)6cacacaa(AC)11	41	5440369	5440409
chr1_1206	p3	(CAA)6	18	5442051	5442068
chr1_1207	p3	(TTA)5	15	5444448	5444462
chr1_1208	p2	(CA)11	22	5447481	5447502
chr1_1209	p2	(TC)7	14	5451143	5451156
chr1_1210	p2	(AG)10	20	5451520	5451539
chr1_1211	p2	(AC)8	16	5455470	5455485
chr1_1212	p2	(AC)12	24	5457752	5457775
chr1_1213	p2	(GT)14	28	5458009	5458036
chr1_1214	c	(GT)6atgtgtgtgc(GT)14	50	5459375	5459424
chr1_1215	p2	(AC)28	56	5465322	5465377
chr1_1216	c	(CT)12(CA)22	68	5468078	5468145
chr1_1217	p2	(GA)12	24	5468258	5468281
chr1_1218	p2	(AC)11	22	5469838	5469859
chr1_1219	p2	(TG)13	26	5471824	5471849
chr1_1220	p3	(TAT)6	18	5472324	5472341
chr1_1221	p3	(ACA)6	18	5477364	5477381
chr1_1222	p3	(CTC)5	15	5478620	5478634
chr1_1223	p2	(CA)7	14	5479611	5479624
chr1_1224	p2	(CA)11	22	5483534	5483555
chr1_1225	p2	(TG)20	40	5489841	5489880
chr1_1226	p2	(GT)18	36	5492549	5492584
chr1_1227	p2	(TG)17	34	5497261	5497294
chr1_1228	p2	(AC)14	28	5506879	5506906
chr1_1229	p2	(GT)6	12	5511048	5511059
chr1_1230	p3	(TAA)6	18	5516953	5516970
chr1_1231	p2	(AC)6	12	5528537	5528548
chr1_1232	p2	(AC)17	34	5537227	5537260
chr1_1233	p2	(AC)11	22	5538799	5538820
chr1_1234	p2	(TG)9	18	5541665	5541682
chr1_1235	p3	(AAC)11	33	5544123	5544155
chr1_1236	p2	(TC)12	24	5547441	5547464
chr1_1237	p3	(GCT)11	33	5551131	5551163
chr1_1238	p2	(CA)25	50	5552311	5552360
chr1_1239	p2	(TG)16	32	5559036	5559067
chr1_1240	c	(CA)8cct(AC)6	31	5559189	5559219
chr1_1241	p4	(AAAT)5	20	5569346	5569365
chr1_1242	p2	(GT)17	34	5569582	5569615
chr1_1243	p2	(GA)6	12	5571160	5571171
chr1_1244	p2	(CA)17	34	5593472	5593505

chr1_1245	p5	(GAGGA)6	30	5596980	5597009
chr1_1246	p2	(GT)12	24	5614742	5614765
chr1_1247	p2	(GT)6	12	5621773	5621784
chr1_1248	p2	(TG)24	48	5622818	5622865
chr1_1249	p3	(CTC)5	15	5624656	5624670
chr1_1250	p2	(CA)21	42	5626664	5626705
chr1_1251	p2	(CA)11	22	5629270	5629291
chr1_1252	p2	(TA)6	12	5631721	5631732
chr1_1253	p2	(TG)7	14	5634765	5634778
chr1_1254	p2	(TG)8	16	5643491	5643506
chr1_1255	c	(CA)12t(AC)20	65	5645128	5645192
chr1_1256	p4	(AAAC)5	20	5651305	5651324
chr1_1257	p2	(CA)6	12	5662003	5662014
chr1_1258	p2	(CA)15	30	5664085	5664114
chr1_1259	c	(TC)9(AC)16	50	5664461	5664510
chr1_1260	p3	(TTA)6	18	5667081	5667098
chr1_1261	p2	(CA)17	34	5673884	5673917
chr1_1262	p5	(TTTGA)6	30	5681963	5681992
chr1_1263	p2	(TG)7	14	5693184	5693197
chr1_1264	p2	(GT)10	20	5704983	5705002
chr1_1265	p2	(AC)10	20	5705119	5705138
chr1_1266	p2	(TG)7	14	5707676	5707689
chr1_1267	p2	(GT)8	16	5711043	5711058
chr1_1268	c	(GT)12(GA)8	40	5712441	5712480
chr1_1269	p2	(TG)9	18	5713527	5713544
chr1_1270	p2	(AC)19	38	5714594	5714631
chr1_1271	p2	(TG)24	48	5717712	5717759
chr1_1272	p2	(TG)11	22	5717892	5717913
chr1_1273	p2	(TG)10	20	5718109	5718128
chr1_1274	p2	(TG)7	14	5718665	5718678
chr1_1275	p2	(TC)10	20	5719891	5719910
chr1_1276	c	(CA)12(CT)11	46	5723065	5723110
chr1_1277	p2	(AC)16	32	5723268	5723299
chr1_1278	p2	(AC)7	14	5724246	5724259
chr1_1279	p2	(AC)8	16	5725361	5725376
chr1_1280	p2	(TG)7	14	5725481	5725494
chr1_1281	c	(TG)7ca(TG)7	30	5730466	5730495
chr1_1282	c	(GT)9agctctgtttctgtgcctgtgcttctatgcact(CA)8	67	5738603	5738669
chr1_1283	p2	(CA)8	16	5739473	5739488
chr1_1284	p2	(TC)7	14	5741168	5741181
chr1_1285	p2	(GT)6	12	5741499	5741510
chr1_1286	p2	(GT)8	16	5742320	5742335
chr1_1287	p2	(AC)9	18	5742534	5742551
chr1_1288	p2	(CA)6	12	5743609	5743620
chr1_1289	p3	(AGG)10	30	5744030	5744059
chr1_1290	p2	(CT)10	20	5746013	5746032
chr1_1291	p2	(AC)13	26	5749214	5749239
chr1_1292	p2	(TG)14	28	5749359	5749386
chr1_1293	p2	(AC)14	28	5749904	5749931
chr1_1294	p2	(CA)14	28	5750111	5750138
chr1_1295	p2	(GT)6	12	5755407	5755418
chr1_1296	p2	(AC)19	38	5764901	5764938

chr1_1297	c	TCA)5ccaggaaatggctcctgctgctttctcttcacaggtacatt(TTC)	71	5766109	5766179
chr1_1298	p3	(TGG)5	15	5766500	5766514
chr1_1299	p2	(CA)6	12	5767370	5767381
chr1_1300	p2	(TG)6	12	5769490	5769501
chr1_1301	c	(AC)6aaacacacacgcgtgtgtgtgtttgtgtggacctgcactg(AC)6	64	5770321	5770384
chr1_1302	p2	(AC)8	16	5773069	5773084
chr1_1303	p2	(CA)18	36	5774987	5775022
chr1_1304	p2	(TA)7	14	5776272	5776285
chr1_1305	p3	(TGA)5	15	5776982	5776996
chr1_1306	p2	(TG)15	30	5779271	5779300
chr1_1307	p2	(AG)6	12	5780152	5780163
chr1_1308	c	ctcaggtgtaccttagtgatgtgtgtttctttctctctgctctgactcacagtgca	85	5782964	5783048
chr1_1309	p2	(AC)9	18	5783153	5783170
chr1_1310	c	(GT)7(GC)6	26	5784796	5784821
chr1_1311	p2	(AC)7	14	5784930	5784943
chr1_1312	p3	(CCT)5	15	5785997	5786011
chr1_1313	p2	(CA)7	14	5787793	5787806
chr1_1314	p2	(AC)8	16	5789780	5789795
chr1_1315	p2	(AC)12	24	5792623	5792646
chr1_1316	p2	(AC)8	16	5793008	5793023
chr1_1317	p2	(CA)6	12	5794934	5794945
chr1_1318	p2	(CA)14	28	5795271	5795298
chr1_1319	c	(GT)8c(TG)8	33	5798120	5798152
chr1_1320	p2	(AC)6	12	5807719	5807730
chr1_1321	p2	(AG)11	22	5807953	5807974
chr1_1322	p2	(GT)12	24	5809460	5809483
chr1_1323	p2	(AC)16	32	5809989	5810020
chr1_1324	p2	(AC)6	12	5811925	5811936
chr1_1325	p2	(CA)9	18	5814715	5814732
chr1_1326	p2	(CA)6	12	5814844	5814855
chr1_1327	p2	(AC)11	22	5815539	5815560
chr1_1328	p2	(AC)9	18	5816285	5816302
chr1_1329	p2	(GA)6	12	5817709	5817720
chr1_1330	p2	(GT)9	18	5820056	5820073
chr1_1331	p2	(GT)25	50	5820180	5820229
chr1_1332	p2	(AC)9	18	5820621	5820638
chr1_1333	p2	(AG)7	14	5820919	5820932
chr1_1334	p2	(AC)7	14	5825119	5825132
chr1_1335	p2	(TG)6	12	5825429	5825440
chr1_1336	p2	(TC)6	12	5830115	5830126
chr1_1337	p2	(AC)9	18	5831141	5831158
chr1_1338	p2	(CA)9	18	5832339	5832356
chr1_1339	p3	(GAG)5	15	5832818	5832832
chr1_1340	p2	(AC)8	16	5833177	5833192
chr1_1341	c	(TG)8ta(TG)6	30	5843331	5843360
chr1_1342	p2	(AC)19	38	5844396	5844433
chr1_1343	p2	(CA)7	14	5844584	5844597
chr1_1344	p2	(GT)7	14	5848532	5848545
chr1_1345	p2	(AC)10	20	5849998	5850017
chr1_1346	p2	(AC)8	16	5853298	5853313
chr1_1347	p2	(CA)14	28	5853755	5853782
chr1_1348	p2	(GT)6	12	5857802	5857813

chr1_1349	p2	(AC)15	30	5858662	5858691
chr1_1350	p3	(CAT)5	15	5864542	5864556
chr1_1351	p2	(AC)22	44	5869538	5869581
chr1_1352	c	agttgggcagtgttagcacatgataaaaataaaaaaaaagacagtaagtgc	96	5872247	5872342
chr1_1353	p2	(AC)7	14	5877843	5877856
chr1_1354	p2	(TG)6	12	5879273	5879284
chr1_1355	p4	(ATGG)5	20	5881534	5881553
chr1_1356	p4	(CCAT)7	28	5888177	5888204
chr1_1357	c	(TG)6ac(TG)7	28	5898738	5898765
chr1_1358	p3	(CTT)6	18	5901063	5901080
chr1_1359	p3	(ATA)5	15	5925316	5925330
chr1_1360	p2	(TA)8	16	5935411	5935426
chr1_1361	p2	(CA)6	12	5937835	5937846
chr1_1362	p2	(TC)7	14	5952231	5952244
chr1_1363	p3	(AAC)7	21	5960835	5960855
chr1_1364	p2	(AC)13	26	5962006	5962031
chr1_1365	c	(TG)7(TA)7	28	5965178	5965205
chr1_1366	p2	(TG)6	12	5967757	5967768
chr1_1367	p2	(CA)12	24	5973456	5973479
chr1_1368	p2	(AC)11	22	5975504	5975525
chr1_1369	p2	(CA)7	14	5996475	5996488
chr1_1370	p2	(GA)7	14	6000639	6000652
chr1_1371	p2	(AG)8	16	6005593	6005608
chr1_1372	p2	(AG)6	12	6008133	6008144
chr1_1373	p2	(AG)6	12	6030049	6030060
chr1_1374	p2	(GT)9	18	6032254	6032271
chr1_1375	p2	(GA)6	12	6033929	6033940
chr1_1376	p2	(AC)8	16	6041209	6041224
chr1_1377	p2	(CA)6	12	6042954	6042965
chr1_1378	p3	(ATC)5	15	6044828	6044842
chr1_1379	p5	(TCAGT)5	25	6052534	6052558
chr1_1380	p2	(AC)9	18	6054129	6054146
chr1_1381	c	(CT)13(CA)20	66	6054801	6054866
chr1_1382	p2	(GA)6	12	6058494	6058505
chr1_1383	p2	(TC)9	18	6063339	6063356
chr1_1384	p2	(TC)9	18	6064643	6064660
chr1_1385	p2	(TC)7	14	6065518	6065531
chr1_1386	p2	(GT)13	26	6068291	6068316
chr1_1387	p3	(CCT)10	30	6071909	6071938
chr1_1388	p2	(GT)12	24	6073274	6073297
chr1_1389	p2	(TG)6	12	6079368	6079379
chr1_1390	c	(TG)7t(TG)15	45	6079886	6079930
chr1_1391	c	(GGA)5ggtggaggtggaggtggt(GGA)5(GGT)5	63	6083092	6083154
chr1_1392	p2	(TC)6	12	6086433	6086444
chr1_1393	p2	(GT)8	16	6095183	6095198
chr1_1394	p2	(GT)11	22	6103514	6103535
chr1_1395	p2	(AC)20	40	6105392	6105431
chr1_1396	p2	(AC)9	18	6106165	6106182
chr1_1397	c	(CT)16t(TC)11	55	6108089	6108143
chr1_1398	p2	(GA)6	12	6110696	6110707
chr1_1399	p2	(TG)10	20	6119052	6119071
chr1_1400	p2	(CA)11	22	6119284	6119305

chr1_1401	p2	(GT)8	16	6119432	6119447
chr1_1402	c	(CA)13g(AC)9	45	6120986	6121030
chr1_1403	p2	(TC)7	14	6121140	6121153
chr1_1404	c	(AC)11tt(TC)8	40	6123480	6123519
chr1_1405	p2	(GT)9	18	6127558	6127575
chr1_1406	p3	(TCC)5	15	6128663	6128677
chr1_1407	p2	(AC)13	26	6133805	6133830
chr1_1408	p2	(TG)10	20	6141482	6141501
chr1_1409	p2	(TC)6	12	6142984	6142995
chr1_1410	p2	(CT)19	38	6144645	6144682
chr1_1411	p3	(TCA)7	21	6144828	6144848
chr1_1412	p2	(AG)25	50	6157885	6157934
chr1_1413	p2	(GT)14	28	6162296	6162323
chr1_1414	p2	(TG)16	32	6162427	6162458
chr1_1415	p2	(TG)6	12	6166442	6166453
chr1_1416	c	(CA)9cgttctctaaataca(AC)6	45	6179280	6179324
chr1_1417	p2	(AC)12	24	6180649	6180672
chr1_1418	p2	(CT)6	12	6180984	6180995
chr1_1419	p2	(TG)6	12	6189271	6189282
chr1_1420	p2	(TC)7	14	6201669	6201682
chr1_1421	c	(TG)6aa(AG)6	26	6204840	6204865
chr1_1422	c	(TC)7(AC)9	32	6206470	6206501
chr1_1423	p2	(TG)15	30	6207023	6207052
chr1_1424	p2	(GT)12	24	6207204	6207227
chr1_1425	p2	(CA)13	26	6207329	6207354
chr1_1426	p2	(CT)9	18	6211258	6211275
chr1_1427	p2	(TG)7	14	6220401	6220414
chr1_1428	p3	(TGT)10	30	6221801	6221830
chr1_1429	p2	(CA)10	20	6222453	6222472
chr1_1430	c	(TATC)5catctatctatccatctatcc(ATCT)8	73	6223513	6223585
chr1_1431	p2	(TG)7	14	6230580	6230593
chr1_1432	p2	(AC)7	14	6232511	6232524
chr1_1433	p2	(TG)17	34	6239251	6239284
chr1_1434	p3	(ATT)8	24	6242043	6242066
chr1_1435	c	CA)12cttagaggccaaattctccctctctgtacacagtctctct(CA)1[illegible]	92	6242428	6242519
chr1_1436	p5	(TTTTA)5	25	6244726	6244750
chr1_1437	p2	(AC)15	30	6246969	6246998
chr1_1438	p4	(TCTA)8	32	6248097	6248128
chr1_1439	p2	(CT)6	12	6252850	6252861
chr1_1440	p2	(AC)9	18	6254237	6254254
chr1_1441	p2	(GA)10	20	6256339	6256358
chr1_1442	p2	(TG)6	12	6263796	6263807
chr1_1443	p2	(TC)10	20	6264148	6264167
chr1_1444	p3	(AAC)5	15	6269504	6269518
chr1_1445	p2	(AC)6	12	6297163	6297174
chr1_1446	p2	(TG)6	12	6307616	6307627
chr1_1447	p2	(AC)6	12	6314039	6314050
chr1_1448	p3	(AAT)5	15	6314807	6314821
chr1_1449	p2	(TG)14	28	6317268	6317295
chr1_1450	p3	(TGC)6	18	6322797	6322814
chr1_1451	p2	(GT)6	12	6327789	6327800
chr1_1452	p2	(GT)11	22	6328079	6328100

chr1_1453	p2	(CA)10	20	6335987	6336006
chr1_1454	p4	(CTGT)5	20	6339384	6339403
chr1_1455	p3	(ATA)5	15	6344393	6344407
chr1_1456	p2	(AC)24	48	6348131	6348178
chr1_1457	p2	(TG)8	16	6349209	6349224
chr1_1458	p2	(AC)6	12	6350075	6350086
chr1_1459	p2	(AC)24	48	6350241	6350288
chr1_1460	p2	(CA)6	12	6355360	6355371
chr1_1461	p2	(CA)10	20	6356127	6356146
chr1_1462	p2	(CT)7	14	6359170	6359183
chr1_1463	p2	(CA)17	34	6371392	6371425
chr1_1464	p2	(GT)7	14	6375966	6375979
chr1_1465	p2	(TC)6	12	6378274	6378285
chr1_1466	p2	(GT)9	18	6379257	6379274
chr1_1467	p3	(CAG)5	15	6392340	6392354
chr1_1468	p2	(AC)9	18	6394629	6394646
chr1_1469	p2	(GT)13	26	6397088	6397113
chr1_1470	p2	(TG)7	14	6399465	6399478
chr1_1471	p2	(CA)13	26	6401174	6401199
chr1_1472	p2	(TG)7	14	6401464	6401477
chr1_1473	p2	(CA)8	16	6403683	6403698
chr1_1474	p2	(TG)8	16	6405016	6405031
chr1_1475	p2	(GT)7	14	6406707	6406720
chr1_1476	p2	(AC)7	14	6407232	6407245
chr1_1477	c	aaattcttgttggacaaaacgctccttgtcttaaaacttgagaacccgacccg	120	6408832	6408951
chr1_1478	p2	(TC)6	12	6409686	6409697
chr1_1479	p2	(AC)13	26	6415441	6415466
chr1_1480	p2	(TG)6	12	6416453	6416464
chr1_1481	p2	(AC)6	12	6416587	6416598
chr1_1482	p2	(AC)11	22	6416828	6416849
chr1_1483	p2	(AG)9	18	6418908	6418925
chr1_1484	p2	(AG)13	26	6423208	6423233
chr1_1485	p2	(AC)14	28	6426435	6426462
chr1_1486	p2	(CA)11	22	6427002	6427023
chr1_1487	p2	(CT)6	12	6429414	6429425
chr1_1488	p2	(CT)7	14	6433850	6433863
chr1_1489	p2	(AC)7	14	6435619	6435632
chr1_1490	p2	(GA)7	14	6439313	6439326
chr1_1491	p2	(CA)9	18	6439661	6439678
chr1_1492	p2	(AC)7	14	6439862	6439875
chr1_1493	p2	(TG)6	12	6440282	6440293
chr1_1494	p2	(AC)22	44	6445225	6445268
chr1_1495	p2	(TG)12	24	6448807	6448830
chr1_1496	p2	(CA)13	26	6450098	6450123
chr1_1497	p2	(TG)7	14	6451421	6451434
chr1_1498	p2	(TA)9	18	6457836	6457853
chr1_1499	p2	(AC)9	18	6459120	6459137
chr1_1500	p2	(AC)20	40	6459540	6459579
chr1_1501	p2	(TG)6	12	6460806	6460817
chr1_1502	c*	(TC)7(TCTA)7*	40	6464942	6464981
chr1_1503	p2	(CT)6	12	6471259	6471270
chr1_1504	p3	(TAG)5	15	6475262	6475276

chr1_1505	p2	(GA)10	20	6490091	6490110
chr1_1506	p2	(TG)6	12	6490917	6490928
chr1_1507	p2	(AC)7	14	6494615	6494628
chr1_1508	p2	(CA)7	14	6504682	6504695
chr1_1509	c	(GT)15atgtgtgtgt(GA)8	56	6509726	6509781
chr1_1510	p2	(TG)8	16	6510697	6510712
chr1_1511	p2	(AT)6	12	6517877	6517888
chr1_1512	p2	(TG)12	24	6519304	6519327
chr1_1513	p2	(GT)17	34	6522770	6522803
chr1_1514	p2	(AC)10	20	6524033	6524052
chr1_1515	c	)8gcacgtacatgggcaatgacgactagaatagaggggtgagaaagca((	76	6524279	6524354
chr1_1516	p2	(TC)8	16	6524616	6524631
chr1_1517	p2	(CA)12	24	6529062	6529085
chr1_1518	p3	(GAG)5	15	6530561	6530575
chr1_1519	p2	(TC)14	28	6532983	6533010
chr1_1520	p2	(AC)12	24	6533505	6533528
chr1_1521	c	(TG)6ccctcaatcaaagtcaccccagtcccacactgatgta(TG)8	65	6541873	6541937
chr1_1522	c	ccatatgcttctatatgccaccattacacattactaactttgtgtctcctctctc	117	6549361	6549477
chr1_1523	p2	(AC)7	14	6554433	6554446
chr1_1524	p2	(AC)6	12	6554783	6554794
chr1_1525	p5	(TCAAA)5	25	6555883	6555907
chr1_1526	p2	(TG)6	12	6559544	6559555
chr1_1527	c	aagaaagatagattcattagctttgagagacacaatcagagcagttaacct(	90	6560591	6560680
chr1_1528	c	nnnnnnnnnnnnnagagagggagttataaagcaaaaggagagaagtga	122	6561897	6562018
chr1_1529	p2	(GT)6	12	6562583	6562594
chr1_1530	p2	(TG)7	14	6565345	6565358
chr1_1531	c	(AC)15atctttatctgtta(TG)9	62	6565497	6565558
chr1_1532	p2	(TG)6	12	6565779	6565790
chr1_1533	p5	(AAATC)15	75	6568761	6568835
chr1_1534	c	(TTC)5(TTA)5	30	6574645	6574674
chr1_1535	p3	(TTG)5	15	6575083	6575097
chr1_1536	p2	(CA)11	22	6576084	6576105
chr1_1537	p2	(CA)7	14	6577395	6577408
chr1_1538	p2	(AC)14	28	6579315	6579342
chr1_1539	p2	(TG)16	32	6579677	6579708
chr1_1540	p3	(TTG)5	15	6582525	6582539
chr1_1541	p2	(CA)20	40	6583231	6583270
chr1_1542	p2	(TG)6	12	6583679	6583690
chr1_1543	p2	(TG)6	12	6592118	6592129
chr1_1544	c	(TC)10(TA)13	46	6592449	6592494
chr1_1545	c	(AC)9atatatagagac(AG)12	54	6592769	6592822
chr1_1546	p2	(TG)6	12	6595752	6595763
chr1_1547	p2	(CA)12	24	6596277	6596300
chr1_1548	p2	(GA)6	12	6596912	6596923
chr1_1549	p2	(TG)7	14	6607155	6607168
chr1_1550	p2	(AC)18	36	6607323	6607358
chr1_1551	p2	(AC)7	14	6610133	6610146
chr1_1552	p2	(CA)6	12	6613388	6613399
chr1_1553	p2	(TG)13	26	6623951	6623976
chr1_1554	p2	(AC)6	12	6627761	6627772
chr1_1555	p2	(CA)15	30	6630968	6630997
chr1_1556	p2	(TG)10	20	6632774	6632793

chr1_1557	c*	(AG)6(AGA)6*	28	6633328	6633355
chr1_1558	p2	(AC)13	26	6637903	6637928
chr1_1559	p2	(TG)15	30	6642237	6642266
chr1_1560	p2	(CA)24	48	6648180	6648227
chr1_1561	c	(GT)13(GA)10	46	6658745	6658790
chr1_1562	p2	(TG)11	22	6658914	6658935
chr1_1563	p2	(TG)12	24	6665479	6665502
chr1_1564	c	tgagagcctctatgttaactgggttgaaattgactcactggtttagtggagca	134	6666857	6666990
chr1_1565	p2	(CA)6	12	6667302	6667313
chr1_1566	p2	(AC)6	12	6687753	6687764
chr1_1567	p2	(CA)23	46	6694831	6694876
chr1_1568	p2	(CT)7	14	6702609	6702622
chr1_1569	p2	(TG)7	14	6705443	6705456
chr1_1570	p2	(AC)8	16	6708809	6708824
chr1_1571	p2	(TG)6	12	6711349	6711360
chr1_1572	p2	(TG)18	36	6718756	6718791
chr1_1573	p2	(GT)6	12	6720833	6720844
chr1_1574	p2	(AC)6	12	6722380	6722391
chr1_1575	p2	(TG)11	22	6722971	6722992
chr1_1576	c	(TC)13(AC)25	76	6725235	6725310
chr1_1577	p2	(AC)6	12	6726891	6726902
chr1_1578	p2	(CA)6	12	6727014	6727025
chr1_1579	p2	(CA)7	14	6727195	6727208
chr1_1580	p3	(TTG)5	15	6731570	6731584
chr1_1581	p2	(GA)9	18	6736076	6736093
chr1_1582	c	(TG)15(AG)12	54	6753590	6753643
chr1_1583	p2	(TG)9	18	6754364	6754381
chr1_1584	p2	(TG)6	12	6757104	6757115
chr1_1585	p2	(GT)14	28	6758145	6758172
chr1_1586	p2	(GT)6	12	6759713	6759724
chr1_1587	p2	(CA)11	22	6761080	6761101
chr1_1588	p2	(CA)7	14	6761247	6761260
chr1_1589	c	agttgtgtttaaatcagagaaaggcagaggacatttgcatacacttgcacat	84	6761392	6761475
chr1_1590	p2	(AC)6	12	6764926	6764937
chr1_1591	p2	(TG)6	12	6769320	6769331
chr1_1592	p2	(TG)7	14	6770040	6770053
chr1_1593	p2	(AC)6	12	6770237	6770248
chr1_1594	p3	(ATA)7	21	6771336	6771356
chr1_1595	p2	(CA)9	18	6778389	6778406
chr1_1596	p2	(AC)7	14	6779632	6779645
chr1_1597	p2	(TG)16	32	6781263	6781294
chr1_1598	p3	(TGT)7	21	6785948	6785968
chr1_1599	p2	(TG)15	30	6787385	6787414
chr1_1600	p5	(ATTTG)5	25	6790709	6790733
chr1_1601	p2	(CA)12	24	6795489	6795512
chr1_1602	p2	(GT)13	26	6798003	6798028
chr1_1603	c	ctgagaagggacaatagtagccccttaaagtgacctcttcagcacttagtgt	83	6798732	6798814
chr1_1604	p3	(CCT)5	15	6815428	6815442
chr1_1605	p2	(AC)12	24	6815781	6815804
chr1_1606	p2	(TG)6	12	6820787	6820798
chr1_1607	p2	(TG)11	22	6821888	6821909
chr1_1608	p2	(TG)8	16	6822020	6822035

chr1_1609	p2	(AC)9	18	6824993	6825010
chr1_1610	p2	(CA)6	12	6825269	6825280
chr1_1611	p2	(AC)7	14	6825417	6825430
chr1_1612	p2	(AC)11	22	6825745	6825766
chr1_1613	p2	(GT)11	22	6828787	6828808
chr1_1614	c	(GT)6t(TG)10(AG)10	53	6833698	6833750
chr1_1615	p2	(GT)19	38	6835264	6835301
chr1_1616	p2	(AC)6	12	6835859	6835870
chr1_1617	p2	(TG)11	22	6836465	6836486
chr1_1618	p2	(TG)16	32	6837865	6837896
chr1_1619	p2	(TG)16	32	6842959	6842990
chr1_1620	p2	(TG)13	26	6849171	6849196
chr1_1621	p2	(CA)7	14	6849332	6849345
chr1_1622	p2	(GA)6	12	6851045	6851056
chr1_1623	p2	(GT)6	12	6853020	6853031
chr1_1624	p2	(TG)6	12	6853282	6853293
chr1_1625	p2	(GT)6	12	6858747	6858758
chr1_1626	p2	(AC)8	16	6861133	6861148
chr1_1627	p2	(TA)7	14	6867306	6867319
chr1_1628	p2	(GT)9	18	6867914	6867931
chr1_1629	p2	(CA)6	12	6868118	6868129
chr1_1630	p2	(GT)14	28	6871759	6871786
chr1_1631	p2	(GT)17	34	6874002	6874035
chr1_1632	p2	(TC)8	16	6879496	6879511
chr1_1633	p2	(TA)6	12	6888918	6888929
chr1_1634	p2	(AC)10	20	6889044	6889063
chr1_1635	p2	(CA)6	12	6890386	6890397
chr1_1636	p5	(ATTTG)5	25	6894184	6894208
chr1_1637	c	gcacattctcccaaccacccactcacacatacactacaactactgtgactct	86	6908320	6908405
chr1_1638	p2	(TG)6	12	6919184	6919195
chr1_1639	p4	(TCAT)5	20	6926325	6926344
chr1_1640	p2	(TG)6	12	6930027	6930038
chr1_1641	p2	(CA)11	22	6933338	6933359
chr1_1642	p2	(TG)9	18	6937493	6937510
chr1_1643	p2	(TG)6	12	6939304	6939315
chr1_1644	p3	(GAT)6	18	6941099	6941116
chr1_1645	p2	(CA)6	12	6948154	6948165
chr1_1646	p2	(TG)9	18	6952156	6952173
chr1_1647	p3	(AGC)5	15	6957900	6957914
chr1_1648	c	GAT)6a(GATG)5ggtggatagatagatggatagatggatg(GAT	101	6963595	6963695
chr1_1649	p2	(TG)14	28	6965907	6965934
chr1_1650	p2	(TG)7	14	6967878	6967891
chr1_1651	c*	(GT)11(GTGC)5*	40	6969665	6969704
chr1_1652	p2	(TG)7	14	6973560	6973573
chr1_1653	p2	(AC)19	38	6975313	6975350
chr1_1654	p2	(TG)6	12	6980650	6980661
chr1_1655	p2	(AC)6	12	6982470	6982481
chr1_1656	p2	(AC)6	12	6997722	6997733
chr1_1657	p2	(CA)12	24	6997935	6997958
chr1_1658	p2	(AC)11	22	6998282	6998303
chr1_1659	p2	(AC)8	16	7024798	7024813
chr1_1660	p2	(GT)10	20	7030243	7030262

chr1_1661	p2	(AC)10	20	7030429	7030448
chr1_1662	c	(AC)6agaaagcacaaagactgacagaaggcaagc(TG)9	60	7030802	7030861
chr1_1663	p2	(AC)6	12	7031465	7031476
chr1_1664	p2	(TG)9	18	7036394	7036411
chr1_1665	p2	(TG)13	26	7038152	7038177
chr1_1666	c	(TATC)6(TACC)7	52	7055756	7055807
chr1_1667	p2	(CA)10	20	7056080	7056099
chr1_1668	p2	(AG)7	14	7060083	7060096
chr1_1669	p3	(ATG)5	15	7065079	7065093
chr1_1670	p2	(CA)29	58	7068162	7068219
chr1_1671	p2	(CA)8	16	7069057	7069072
chr1_1672	p2	(TG)6	12	7069792	7069803
chr1_1673	p2	(TG)10	20	7071025	7071044
chr1_1674	p2	(GT)11	22	7078584	7078605
chr1_1675	c	(TG)12gtctttttcccactcaa(AC)8	57	7078747	7078803
chr1_1676	p2	(TG)6	12	7079279	7079290
chr1_1677	p2	(TG)17	34	7079392	7079425
chr1_1678	p2	(GT)7	14	7080033	7080046
chr1_1679	p2	(GT)6	12	7081030	7081041
chr1_1680	p2	(GT)18	36	7081323	7081358
chr1_1681	p2	(GT)9	18	7082212	7082229
chr1_1682	p4	(TCCT)5	20	7082359	7082378
chr1_1683	p2	(TG)37	74	7086239	7086312
chr1_1684	p2	(CA)21	42	7089969	7090010
chr1_1685	p2	(TG)11	22	7091126	7091147
chr1_1686	p2	(CA)13	26	7093209	7093234
chr1_1687	c	ıgcagcagttggcttcaggaacatgtggcagatctgagcggaaggtaaaa	117	7094502	7094618
chr1_1688	p2	(GT)9	18	7094731	7094748
chr1_1689	p2	(CA)7	14	7098466	7098479
chr1_1690	p3	(TGT)7	21	7099373	7099393
chr1_1691	p2	(TG)19	38	7099506	7099543
chr1_1692	c	:aaagtcggcgacctaatccacagagttcaaccagaaacacttggagtcag	142	7103989	7104130
chr1_1693	p2	(CA)8	16	7106412	7106427
chr1_1694	p2	(GT)8	16	7112911	7112926
chr1_1695	p2	(GT)12	24	7118341	7118364
chr1_1696	p2	(TG)7	14	7120551	7120564
chr1_1697	p4	(AAAC)6	24	7125783	7125806
chr1_1698	p2	(GT)18	36	7125923	7125958
chr1_1699	p2	(GA)7	14	7129749	7129762
chr1_1700	p3	(TCT)5	15	7130856	7130870
chr1_1701	p2	(AC)13	26	7131286	7131311
chr1_1702	p3	(GAG)6	18	7133995	7134012
chr1_1703	p3	(CCT)6	18	7134128	7134145
chr1_1704	c	:gtgcgggttgcatgcatgaatgcacttgtaaatgtgtgttgctgtatgggag	115	7134334	7134448
chr1_1705	p3	(CCT)5	15	7134708	7134722
chr1_1706	c	(GT)8ttctgttgtctctgttac(GT)6	46	7136994	7137039
chr1_1707	c	:caccttctacaaactcggcagcccagcgcctaatagcacccagagtcttc	105	7137149	7137253
chr1_1708	c	(AC)9aatgtcgggcc(GT)6	41	7137579	7137619
chr1_1709	p2	(TG)7	14	7138446	7138459
chr1_1710	p2	(CA)6	12	7146175	7146186
chr1_1711	p2	(CA)6	12	7148161	7148172
chr1_1712	p2	(TG)17	34	7150236	7150269

chr1_1713	p2	(TC)6	12	7152033	7152044
chr1_1714	p3	(GGA)5	15	7157516	7157530
chr1_1715	p2	(AC)21	42	7160023	7160064
chr1_1716	p2	(CA)9	18	7162258	7162275
chr1_1717	p2	(GT)21	42	7162452	7162493
chr1_1718	p2	(AC)14	28	7167854	7167881
chr1_1719	c	agcactatcacatttctatttcaaagtcgaagaaaggacg(GT)16cctcg	162	7169466	7169627
chr1_1720	p2	(TG)21	42	7169740	7169781
chr1_1721	p2	(CT)6	12	7170709	7170720
chr1_1722	p2	(TG)21	42	7170843	7170884
chr1_1723	p3	(AGG)10	30	7172965	7172994
chr1_1724	p2	(AG)6	12	7175148	7175159
chr1_1725	p2	(TG)24	48	7178003	7178050
chr1_1726	p2	(GT)20	40	7180802	7180841
chr1_1727	c	ıcgctgagctcagcaggctggaggatgtgtggggtgatgacattgaactag	133	7181686	7181818
chr1_1728	p3	(TAG)6	18	7182237	7182254
chr1_1729	p2	(GT)6	12	7184136	7184147
chr1_1730	c	(TG)19(AG)6	50	7186760	7186809
chr1_1731	p2	(AC)10	20	7191804	7191823
chr1_1732	p3	(GAG)5	15	7193360	7193374
chr1_1733	p2	(GT)20	40	7193478	7193517
chr1_1734	p2	(TG)12	24	7196549	7196572
chr1_1735	p2	(GT)9	18	7197047	7197064
chr1_1736	p2	(CA)8	16	7197600	7197615
chr1_1737	p2	(AC)11	22	7198728	7198749
chr1_1738	p2	(TG)8	16	7200028	7200043
chr1_1739	p2	(TG)15	30	7200866	7200895
chr1_1740	p2	(TG)12	24	7201079	7201102
chr1_1741	c	acatcactgtctcctcttgctctaatg(AC)27gtctgtgtcgcgtgttcagc	258	7201226	7201483
chr1_1742	c	ctcaatgggtgcagtccagcnnnnnnnnnnnnnnnnnnnnnnnn	148	7202615	7202762
chr1_1743	p2	(AC)26	52	7202883	7202934
chr1_1744	p2	(CA)11	22	7204131	7204152
chr1_1745	p2	(AC)14	28	7204297	7204324
chr1_1746	c	(CA)6ccctctctctgagtctttgtggc(TG)8	51	7205593	7205643
chr1_1747	c	(TC)8(AC)8	32	7206967	7206998
chr1_1748	p3	(ATA)5	15	7209097	7209111
chr1_1749	p2	(TG)16	32	7216706	7216737
chr1_1750	p2	(TG)8	16	7225747	7225762
chr1_1751	p2	(TG)8	16	7227030	7227045
chr1_1752	p2	(TG)6	12	7238266	7238277
chr1_1753	p3	(CCT)6	18	7243600	7243617
chr1_1754	p2	(TG)16	32	7246766	7246797
chr1_1755	p2	(GT)13	26	7247945	7247970
chr1_1756	p2	(TA)6	12	7249080	7249091
chr1_1757	c	gtcactcattcatctca(AC)13ctggcgcactgaccaactctcaacagc	231	7253160	7253390
chr1_1758	p2	(GT)6	12	7256152	7256163
chr1_1759	p2	(CA)15	30	7257497	7257526
chr1_1760	p2	(CA)7	14	7257859	7257872
chr1_1761	p2	(TG)24	48	7266251	7266298
chr1_1762	c	aggcgtgcgtatccagtaggnnnnnnnnnnnnnnnnnnnnnnnn	165	7272778	7272942
chr1_1763	p2	(TG)8	16	7273784	7273799
chr1_1764	p2	(GT)11	22	7275992	7276013

chr1_1765	p2	(GT)16	32	7279251	7279282
chr1_1766	p2	(TG)7	14	7283870	7283883
chr1_1767	p2	(CA)6	12	7284769	7284780
chr1_1768	c	atagttagtagtctggtgtgacatctgttgccatggtgaggagagctgcctgc	184	7285993	7286176
chr1_1769	p2	(TG)14	28	7286744	7286771
chr1_1770	c	acacaggagccaaagagcaacttttctttgaagccccgagaggaaaaaga	144	7288149	7288292
chr1_1771	p2	(TG)11	22	7289941	7289962
chr1_1772	p2	(TG)16	32	7290828	7290859
chr1_1773	p2	(GT)17	34	7291038	7291071
chr1_1774	p2	(GT)6	12	7316758	7316769
chr1_1775	p2	(AC)8	16	7320820	7320835
chr1_1776	p2	(CA)11	22	7323486	7323507
chr1_1777	p2	(TG)6	12	7334728	7334739
chr1_1778	p3	(TTC)5	15	7337267	7337281
chr1_1779	p2	(AC)14	28	7345864	7345891
chr1_1780	p2	(GA)7	14	7351530	7351543
chr1_1781	p2	(TG)9	18	7355706	7355723
chr1_1782	p2	(GT)10	20	7365596	7365615
chr1_1783	p2	(GT)6	12	7369244	7369255
chr1_1784	p2	(AC)13	26	7379436	7379461
chr1_1785	p2	(AC)6	12	7379774	7379785
chr1_1786	p3	(CCT)5	15	7382507	7382521
chr1_1787	p2	(AC)16	32	7384387	7384418
chr1_1788	p2	(CA)7	14	7391765	7391778
chr1_1789	c	ccgatgcatatatgtgtggcgcccattgcagagcctgacaagcagtcgggg	103	7398718	7398820
chr1_1790	p3	(GTG)5	15	7408009	7408023
chr1_1791	p2	(CA)6	12	7411956	7411967
chr1_1792	p3	(GGA)6	18	7428278	7428295
chr1_1793	p3	(AGA)5	15	7429673	7429687
chr1_1794	p2	(TG)6	12	7433881	7433892
chr1_1795	p2	(TG)17	34	7433995	7434028
chr1_1796	p2	(GT)20	40	7450654	7450693
chr1_1797	p3	(ATT)5	15	7451907	7451921
chr1_1798	p2	(AC)6	12	7456694	7456705
chr1_1799	p2	(GT)7	14	7458204	7458217
chr1_1800	p2	(TG)23	46	7498751	7498796
chr1_1801	p2	(TA)12	24	7503365	7503388
chr1_1802	p2	(TA)12	24	7506334	7506357
chr1_1803	p2	(TG)10	20	7529407	7529426
chr1_1804	p3	(TAT)5	15	7530906	7530920
chr1_1805	p2	(GT)11	22	7545120	7545141
chr1_1806	p2	(TG)6	12	7558218	7558229
chr1_1807	p2	(AT)8	16	7596021	7596036
chr1_1808	c	aaagacagaaataaaactaaatttaaatcaatcaaaaaataaatacttcttta	125	7600613	7600737
chr1_1809	p2	(TA)7	14	7601738	7601751
chr1_1810	p2	(AC)6	12	7612240	7612251
chr1_1811	p2	(TG)15	30	7627776	7627805
chr1_1812	c	(AT)8ctatat(ATAG)5	42	7637627	7637668
chr1_1813	p2	(AC)13	26	7639053	7639078
chr1_1814	p2	(TC)15	30	7648255	7648284
chr1_1815	p2	(GT)9	18	7649593	7649610
chr1_1816	p3	(TTA)6	18	7649855	7649872

chr1_1817	p3	(GAG)5	15	7656069	7656083
chr1_1818	p2	(CA)6	12	7664911	7664922
chr1_1819	p2	(TG)19	38	7677457	7677494
chr1_1820	p3	(TGA)5	15	7691707	7691721
chr1_1821	p2	(GT)17	34	7702341	7702374
chr1_1822	p2	(AT)6	12	7714500	7714511
chr1_1823	p3	(AAG)5	15	7715513	7715527
chr1_1824	p3	(AAC)5	15	7722826	7722840
chr1_1825	p2	(AC)25	50	7723350	7723399
chr1_1826	p2	(AC)28	56	7729900	7729955
chr1_1827	c	(CT)12(CA)19	62	7744562	7744623
chr1_1828	p2	(CA)7	14	7758174	7758187
chr1_1829	p2	(CA)13	26	7785839	7785864
chr1_1830	p2	(GA)10	20	7794968	7794987
chr1_1831	p4	(ATTC)5	20	7796464	7796483
chr1_1832	p3	(GAG)5	15	7803025	7803039
chr1_1833	p2	(CA)6	12	7815564	7815575
chr1_1834	p2	(GA)13	26	7826214	7826239
chr1_1835	p3	(AAT)5	15	7856662	7856676
chr1_1836	p3	(AAC)5	15	7859953	7859967
chr1_1837	p2	(CA)7	14	7880531	7880544
chr1_1838	p2	(TG)8	16	7898698	7898713
chr1_1839	p3	(ATT)5	15	7899630	7899644
chr1_1840	p4	(TTTG)6	24	7918280	7918303
chr1_1841	p2	(GT)8	16	7919687	7919702
chr1_1842	p2	(GT)8	16	7921567	7921582
chr1_1843	p2	(CA)6	12	7926345	7926356
chr1_1844	p5	(AAAAC)6	30	7951566	7951595
chr1_1845	p2	(GT)6	12	7957164	7957175
chr1_1846	p2	(GA)15	30	7975359	7975388
chr1_1847	p2	(AC)12	24	7982830	7982853
chr1_1848	p5	(ATTTG)7	35	7998814	7998848
chr1_1849	p2	(AG)6	12	8000168	8000179
chr1_1850	p4	(GAAA)5	20	8005212	8005231
chr1_1851	p2	(GT)9	18	8036378	8036395
chr1_1852	p2	(TG)6	12	8076969	8076980
chr1_1853	p2	(GA)6	12	8083962	8083973
chr1_1854	p2	(AC)15	30	8085160	8085189
chr1_1855	p4	(CATT)5	20	8088768	8088787
chr1_1856	p2	(GT)7	14	8090755	8090768
chr1_1857	p2	(CA)7	14	8109439	8109452
chr1_1858	p4	(AAAT)5	20	8110839	8110858
chr1_1859	c	(AC)16g(CA)6	45	8134412	8134456
chr1_1860	c	(AC)9(TC)11	40	8138942	8138981
chr1_1861	p5	(TGATT)6	30	8148631	8148660
chr1_1862	p2	(GT)9	18	8167832	8167849
chr1_1863	p3	(TAG)11	33	8173383	8173415
chr1_1864	p2	(TG)17	34	8180013	8180046
chr1_1865	p3	(TTG)6	18	8180683	8180700
chr1_1866	p3	(TCT)5	15	8185654	8185668
chr1_1867	p3	(AAT)5	15	8192048	8192062
chr1_1868	p3	(CAT)6	18	8242376	8242393

chr1_1869	p3	(ACA)7	21	8245394	8245414
chr1_1870	p2	(AC)7	14	8252258	8252271
chr1_1871	p2	(TC)6	12	8261096	8261107
chr1_1872	p2	(CA)9	18	8262190	8262207
chr1_1873	p2	(TG)9	18	8265996	8266013
chr1_1874	p3	(CAA)5	15	8267856	8267870
chr1_1875	c	(CAC)5tactacc(ACT)8(AAT)7	67	8271749	8271815
chr1_1876	p2	(GT)9	18	8276030	8276047
chr1_1877	p2	(TG)13	26	8283746	8283771
chr1_1878	p2	(TG)6	12	8299196	8299207
chr1_1879	p2	(CA)7	14	8309976	8309989
chr1_1880	p3	(AAT)6	18	8313689	8313706
chr1_1881	p2	(TC)11	22	8327755	8327776
chr1_1882	p2	(TC)6	12	8329077	8329088
chr1_1883	p3	(CCA)5	15	8330166	8330180
chr1_1884	p4	(TAAC)6	24	8338027	8338050
chr1_1885	p2	(AC)10	20	8339459	8339478
chr1_1886	p2	(CA)16	32	8346612	8346643
chr1_1887	p2	(GT)12	24	8354305	8354328
chr1_1888	p2	(TG)6	12	8361060	8361071
chr1_1889	c	(ATCT)7atccatttctctcttc(CT)6	58	8364186	8364243
chr1_1890	p3	(AAT)5	15	8366118	8366132
chr1_1891	c	'A)5ttgttgtcaggtgaaaggtttatgcatgataggcaaaa(AAT)5(C	80	8381847	8381926
chr1_1892	p2	(AC)23	46	8383690	8383735
chr1_1893	p2	(TG)8	16	8385744	8385759
chr1_1894	c	(TA)6tgtatacacacat(AC)10	45	8392449	8392493
chr1_1895	p3	(ATA)7	21	8396030	8396050
chr1_1896	p2	(TA)7	14	8396355	8396368
chr1_1897	p2	(AG)6	12	8397696	8397707
chr1_1898	p2	(AC)13	26	8403236	8403261
chr1_1899	p2	(AG)8	16	8405240	8405255
chr1_1900	p2	(GT)7	14	8405969	8405982
chr1_1901	p3	(AAT)7	21	8413160	8413180
chr1_1902	p2	(GT)6	12	8429120	8429131
chr1_1903	p2	(AT)6	12	8434513	8434524
chr1_1904	p2	(CT)6	12	8449715	8449726
chr1_1905	p2	(AC)21	42	8456083	8456124
chr1_1906	p2	(AC)6	12	8462569	8462580
chr1_1907	p3	(AAT)5	15	8476047	8476061
chr1_1908	p2	(CA)18	36	8476861	8476896
chr1_1909	p2	(GT)12	24	8491656	8491679
chr1_1910	p2	(CA)6	12	8500170	8500181
chr1_1911	p3	(TTG)5	15	8528510	8528524
chr1_1912	p2	(CA)18	36	8563457	8563492
chr1_1913	c	(TG)21(TA)9	60	8564563	8564622
chr1_1914	c	(CA)12(TA)11	46	8569307	8569352
chr1_1915	p2	(GT)7	14	8580785	8580798
chr1_1916	c	(AT)8aagaagg(GT)8	39	8596242	8596280
chr1_1917	c	agagaggtaagacacttacagccatagccacatagaaaatagttgctcata	119	8597488	8597606
chr1_1918	p2	(AC)17	34	8612491	8612524
chr1_1919	p2	(TC)6	12	8614535	8614546
chr1_1920	p2	(AC)6	12	8616545	8616556

chr1_1921	p2	(GA)7	14	8627851	8627864
chr1_1922	p2	(CA)10	20	8635822	8635841
chr1_1923	p3	(ATA)9	27	8637640	8637666
chr1_1924	p2	(GT)6	12	8661186	8661197
chr1_1925	p2	(AC)15	30	8673518	8673547
chr1_1926	p2	(GT)13	26	8684842	8684867
chr1_1927	c	(GT)7gc(GT)6	28	8686364	8686391
chr1_1928	p2	(GT)7	14	8690367	8690380
chr1_1929	p2	(GT)7	14	8691282	8691295
chr1_1930	p2	(AT)6	12	8692227	8692238
chr1_1931	p2	(AT)6	12	8720464	8720475
chr1_1932	p4	(TAGA)5	20	8730142	8730161
chr1_1933	p3	(GAG)6	18	8731228	8731245
chr1_1934	p2	(TG)6	12	8736058	8736069
chr1_1935	p2	(AC)16	32	8750198	8750229
chr1_1936	p2	(AG)6	12	8750580	8750591
chr1_1937	p2	(AT)8	16	8754512	8754527
chr1_1938	p3	(ATA)6	18	8773797	8773814
chr1_1939	p2	(TG)15	30	8782482	8782511
chr1_1940	p4	(TTTC)6	24	8789715	8789738
chr1_1941	p3	(ATG)6	18	8798925	8798942
chr1_1942	p3	(ATT)5	15	8805527	8805541
chr1_1943	p2	(CA)15	30	8810986	8811015
chr1_1944	p2	(CA)19	38	8817917	8817954
chr1_1945	p2	(CA)20	40	8821183	8821222
chr1_1946	p2	(AC)14	28	8836161	8836188
chr1_1947	p2	(AC)14	28	8838985	8839012
chr1_1948	p2	(AC)7	14	8839750	8839763
chr1_1949	p2	(GT)23	46	8852883	8852928
chr1_1950	p2	(GT)17	34	8855211	8855244
chr1_1951	p2	(GT)6	12	8857227	8857238
chr1_1952	p4	(ATCA)6	24	8882013	8882036
chr1_1953	p3	(AGT)13	39	8900464	8900502
chr1_1954	p4	(CTAT)17	68	8918259	8918326
chr1_1955	p4	(GATG)5	20	8930107	8930126
chr1_1956	p2	(GT)18	36	8942211	8942246
chr1_1957	p3	(ATT)5	15	8952566	8952580
chr1_1958	p2	(GT)7	14	8953827	8953840
chr1_1959	p2	(AC)15	30	8959550	8959579
chr1_1960	c	tttttttttttttgctcattggattcaaaacacccatgcattccccacacaccctc	86	8968901	8968986
chr1_1961	p2	(TA)9	18	8973885	8973902
chr1_1962	c	(ATA)5attatagtaag(GT)6	38	8976580	8976617
chr1_1963	p2	(TA)8	16	8985316	8985331
chr1_1964	p2	(AC)7	14	8986580	8986593
chr1_1965	p2	(CA)6	12	8995012	8995023
chr1_1966	p4	(ATCT)20	80	9003235	9003314
chr1_1967	p2	(GT)6	12	9015429	9015440
chr1_1968	p2	(TA)8	16	9019689	9019704
chr1_1969	p2	(AC)9	18	9029871	9029888
chr1_1970	p2	(CA)12	24	9040716	9040739
chr1_1971	p3	(TTC)6	18	9045625	9045642
chr1_1972	p4	(CATT)5	20	9061402	9061421

chr1_1973	p3	(TCC)5	15	9076284	9076298
chr1_1974	p2	(AC)25	50	9077645	9077694
chr1_1975	p2	(AG)7	14	9078777	9078790
chr1_1976	p2	(AC)12	24	9082049	9082072
chr1_1977	p2	(CA)11	22	9092532	9092553
chr1_1978	p2	(TG)6	12	9100259	9100270
chr1_1979	p2	(CA)7	14	9112407	9112420
chr1_1980	p2	(TG)13	26	9113567	9113592
chr1_1981	p2	(AG)8	16	9130920	9130935
chr1_1982	p2	(GT)7	14	9137283	9137296
chr1_1983	p2	(GT)8	16	9141726	9141741
chr1_1984	p2	(AC)7	14	9150308	9150321
chr1_1985	p2	(CT)6	12	9165595	9165606
chr1_1986	p2	(AT)6	12	9170076	9170087
chr1_1987	c	(GT)9aaagttttagaaggg(GT)7	47	9173376	9173422
chr1_1988	p3	(TTC)5	15	9181053	9181067
chr1_1989	p2	(TA)6	12	9186730	9186741
chr1_1990	p3	(TGA)5	15	9186957	9186971
chr1_1991	p4	(TTAT)6	24	9188334	9188357
chr1_1992	p4	(ATCT)11	44	9189746	9189789
chr1_1993	p4	(AAAT)5	20	9190338	9190357
chr1_1994	p3	(TTG)5	15	9202380	9202394
chr1_1995	p2	(TG)23	46	9230743	9230788
chr1_1996	p2	(AG)6	12	9240060	9240071
chr1_1997	p2	(CA)15	30	9243872	9243901
chr1_1998	p4	(TTCA)5	20	9246383	9246402
chr1_1999	c*	(TAGA)6(AG)12*	45	9246902	9246946
chr1_2000	p2	(CA)15	30	9255838	9255867
chr1_2001	p3	(AAT)6	18	9258200	9258217
chr1_2002	p2	(AG)6	12	9262019	9262030
chr1_2003	p2	(CA)16	32	9263317	9263348
chr1_2004	p2	(AC)12	24	9271108	9271131
chr1_2005	p2	(TG)6	12	9280087	9280098
chr1_2006	p3	(TTC)8	24	9285020	9285043
chr1_2007	p2	(GT)7	14	9285177	9285190
chr1_2008	p2	(GT)6	12	9289550	9289561
chr1_2009	p2	(TG)7	14	9297423	9297436
chr1_2010	p2	(CA)6	12	9303988	9303999
chr1_2011	p2	(CA)6	12	9311861	9311872
chr1_2012	p2	(AC)11	22	9316737	9316758
chr1_2013	p3	(AGC)7	21	9347211	9347231
chr1_2014	p2	(AC)9	18	9376711	9376728
chr1_2015	p2	(TC)10	20	9378920	9378939
chr1_2016	p5	(CTTAT)5	25	9388017	9388041
chr1_2017	p2	(TG)21	42	9393390	9393431
chr1_2018	p2	(TA)8	16	9394278	9394293
chr1_2019	c	(TC)10tt(TC)7	36	9418246	9418281
chr1_2020	p2	(CA)7	14	9418514	9418527
chr1_2021	p4	(AAAT)5	20	9438192	9438211
chr1_2022	p2	(TG)25	50	9447883	9447932
chr1_2023	p2	(CT)8	16	9448342	9448357
chr1_2024	p2	(AC)7	14	9454380	9454393

chr1_2025	p4	(CATC)5	20	9469977	9469996
chr1_2026	p2	(TG)20	40	9477222	9477261
chr1_2027	p2	(TG)8	16	9485996	9486011
chr1_2028	p2	(CA)12	24	9486205	9486228
chr1_2029	p2	(GT)6	12	9487288	9487299
chr1_2030	p3	(AAT)7	21	9488590	9488610
chr1_2031	p2	(AG)6	12	9491240	9491251
chr1_2032	p2	(TG)6	12	9496973	9496984
chr1_2033	p2	(CA)8	16	9501706	9501721
chr1_2034	p2	(CA)10	20	9503601	9503620
chr1_2035	p2	(CA)18	36	9511047	9511082
chr1_2036	p2	(TC)7	14	9513038	9513051
chr1_2037	p2	(AC)12	24	9519993	9520016
chr1_2038	p2	(GT)6	12	9520255	9520266
chr1_2039	p4	(ATGG)5	20	9521269	9521288
chr1_2040	p2	(AT)8	16	9525109	9525124
chr1_2041	p2	(AC)7	14	9526272	9526285
chr1_2042	p2	(GT)7	14	9530823	9530836
chr1_2043	p2	(AC)25	50	9532272	9532321
chr1_2044	p2	(CA)8	16	9535640	9535655
chr1_2045	p3	(ACT)6	18	9539915	9539932
chr1_2046	p3	(CTG)7	21	9552544	9552564
chr1_2047	p2	(GT)6	12	9557285	9557296
chr1_2048	p2	(GT)7	14	9560206	9560219
chr1_2049	p2	(AC)8	16	9560694	9560709
chr1_2050	p2	(GA)6	12	9561094	9561105
chr1_2051	p2	(TG)8	16	9564969	9564984
chr1_2052	p2	(TC)9	18	9573739	9573756
chr1_2053	p3	(GAG)6	18	9577094	9577111
chr1_2054	p3	(AAT)5	15	9578488	9578502
chr1_2055	p2	(GT)15	30	9580468	9580497
chr1_2056	p2	(CA)14	28	9586572	9586599
chr1_2057	c	(AC)6g(CA)9	31	9589398	9589428
chr1_2058	p2	(GA)6	12	9594137	9594148
chr1_2059	p2	(AC)6	12	9594487	9594498
chr1_2060	p2	(GT)6	12	9599394	9599405
chr1_2061	p3	(TGC)5	15	9602920	9602934
chr1_2062	c	(TAT)5(TAC)7	36	9603915	9603950
chr1_2063	p2	(AC)6	12	9612196	9612207
chr1_2064	p2	(GT)8	16	9614500	9614515
chr1_2065	p2	(CA)13	26	9616462	9616487
chr1_2066	c	atatatacatacaaagatatacaggagcagaacaatgcatgttggtaaatgt	107	9617948	9618054
chr1_2067	p2	(TG)9	18	9621880	9621897
chr1_2068	p2	(AC)12	24	9628482	9628505
chr1_2069	p2	(CT)6	12	9631493	9631504
chr1_2070	p2	(CA)16	32	9634274	9634305
chr1_2071	p2	(GA)7	14	9640786	9640799
chr1_2072	p2	(TG)14	28	9641391	9641418
chr1_2073	p3	(TAT)13	39	9644543	9644581
chr1_2074	p3	(TGG)5	15	9647706	9647720
chr1_2075	p2	(CA)6	12	9664250	9664261
chr1_2076	p4	(CTCC)5	20	9676122	9676141

chr1_2077	p2	(TC)23	46	9734959	9735004
chr1_2078	p2	(GA)6	12	9750053	9750064
chr1_2079	p2	(TC)6	12	9756052	9756063
chr1_2080	p2	(TG)6	12	9791265	9791276
chr1_2081	p2	(CA)23	46	9798795	9798840
chr1_2082	p3	(TTA)6	18	9802100	9802117
chr1_2083	p3	(CAT)5	15	9804841	9804855
chr1_2084	p2	(AT)10	20	9815089	9815108
chr1_2085	p2	(GT)7	14	9816765	9816778
chr1_2086	p2	(TC)6	12	9817389	9817400
chr1_2087	p5	(GCTAA)5	25	9818209	9818233
chr1_2088	p4	(ATGT)5	20	9822005	9822024
chr1_2089	p2	(GT)12	24	9825427	9825450
chr1_2090	p2	(AC)19	38	9826342	9826379
chr1_2091	p2	(TC)12	24	9840696	9840719
chr1_2092	p2	(AC)9	18	9841822	9841839
chr1_2093	p2	(TA)7	14	9843447	9843460
chr1_2094	p2	(TG)15	30	9852052	9852081
chr1_2095	c	(CT)7(CA)10	34	9854011	9854044
chr1_2096	p2	(CA)21	42	9854353	9854394
chr1_2097	p2	(TG)8	16	9856847	9856862
chr1_2098	c	tgtgtgaatgagatggagggaggagagctagaggcttactgtgtgtcagc	83	9865811	9865893
chr1_2099	p2	(GT)12	24	9866577	9866600
chr1_2100	p2	(TC)6	12	9867535	9867546
chr1_2101	p2	(TG)8	16	9869092	9869107
chr1_2102	p2	(TG)6	12	9878522	9878533
chr1_2103	p2	(CA)13	26	9881246	9881271
chr1_2104	p3	(GAA)5	15	9894358	9894372
chr1_2105	p2	(GT)14	28	9895859	9895886
chr1_2106	c	(TG)6tctgaga(GT)8	35	9896254	9896288
chr1_2107	p2	(TG)14	28	9896939	9896966
chr1_2108	p2	(GT)17	34	9903758	9903791
chr1_2109	p2	(TG)11	22	9903893	9903914
chr1_2110	c	agaacacacactaatgcccaaaaacaactctcaggtggtctgcttcactc	147	9908100	9908246
chr1_2111	p4	(GGGA)5	20	9909476	9909495
chr1_2112	p2	(AC)6	12	9909686	9909697
chr1_2113	p2	(TG)11	22	9910937	9910958
chr1_2114	p3	(TAT)6	18	9911882	9911899
chr1_2115	p3	(ATT)6	18	9912913	9912930
chr1_2116	c	(TTA)7ttgttgccactacattggtaaatatattttat(TTTA)5	73	9916320	9916392
chr1_2117	p3	(GGA)5	15	9924717	9924731
chr1_2118	p2	(GT)7	14	9926662	9926675
chr1_2119	p2	(CA)6	12	9927089	9927100
chr1_2120	p2	(GT)8	16	9927520	9927535
chr1_2121	p2	(AG)6	12	9928684	9928695
chr1_2122	p2	(AC)6	12	9938741	9938752
chr1_2123	p3	(TGT)5	15	9941394	9941408
chr1_2124	p3	(ACA)5	15	9943966	9943980
chr1_2125	p4	(ATCT)6	24	9945718	9945741
chr1_2126	p2	(CA)6	12	9947282	9947293
chr1_2127	p2	(AC)6	12	9949738	9949749
chr1_2128	p4	(GATG)6	24	9951768	9951791

chr1_2129	p2	(CA)6	12	9954859	9954870
chr1_2130	p3	(AAT)5	15	9962031	9962045
chr1_2131	p2	(CA)14	28	9964449	9964476
chr1_2132	p2	(TG)6	12	9965763	9965774
chr1_2133	p2	(TG)17	34	9971889	9971922
chr1_2134	c	(TG)24(AG)10	68	9973725	9973792
chr1_2135	p3	(AAT)5	15	9973903	9973917
chr1_2136	p2	(TG)7	14	9975755	9975768
chr1_2137	p4	(TTCA)6	24	9986749	9986772
chr1_2138	p3	(AAT)7	21	9998500	9998520
chr1_2139	p3	(TTA)5	15	10040620	10040634
chr1_2140	p3	(CAT)5	15	10057594	10057608
chr1_2141	p2	(GT)12	24	10058382	10058405
chr1_2142	c	tcccagcagaaggggcacactataggctaatcagttggtagtttcaatccat	152	10063687	10063838
chr1_2143	p2	(CG)6	12	10064244	10064255
chr1_2144	p2	(CA)44	88	10068925	10069012
chr1_2145	p5	(TTGAT)11	55	10072077	10072131
chr1_2146	p2	(GT)6	12	10089861	10089872
chr1_2147	p2	(AC)8	16	10092247	10092262
chr1_2148	p2	(CA)6	12	10101687	10101698
chr1_2149	p2	(AC)6	12	10102811	10102822
chr1_2150	p2	(AC)6	12	10115339	10115350
chr1_2151	p2	(CA)9	18	10131032	10131049
chr1_2152	p2	(TG)15	30	10137729	10137758
chr1_2153	p2	(AC)7	14	10139564	10139577
chr1_2154	p2	(CA)37	74	10147293	10147366
chr1_2155	p2	(CA)8	16	10147637	10147652
chr1_2156	p2	(GT)6	12	10149037	10149048
chr1_2157	p2	(AC)8	16	10149463	10149478
chr1_2158	p3	(CAG)6	18	10154971	10154988
chr1_2159	c	(TG)13(AG)12	50	10155137	10155186
chr1_2160	p2	(CA)8	16	10158739	10158754
chr1_2161	p2	(GT)21	42	10162713	10162754
chr1_2162	p2	(GT)14	28	10163219	10163246
chr1_2163	c	tcttgtttctttaacgctgctgagtggagctctgctgtggtaattgatcctgtttg	123	10166006	10166128
chr1_2164	p2	(TC)10	20	10168515	10168534
chr1_2165	p2	(TG)10	20	10169445	10169464
chr1_2166	p2	(GA)6	12	10169670	10169681
chr1_2167	p2	(GT)11	22	10170088	10170109
chr1_2168	p2	(AG)11	22	10176743	10176764
chr1_2169	p3	(GAG)6	18	10177771	10177788
chr1_2170	p3	(TTG)5	15	10178254	10178268
chr1_2171	p2	(GA)6	12	10178689	10178700
chr1_2172	p2	(GT)7	14	10178935	10178948
chr1_2173	p2	(AC)17	34	10183392	10183425
chr1_2174	p2	(TG)6	12	10183772	10183783
chr1_2175	p2	(TG)7	14	10185658	10185671
chr1_2176	p2	(CA)18	36	10192291	10192326
chr1_2177	p2	(GT)10	20	10193774	10193793
chr1_2178	p2	(CA)12	24	10194122	10194145
chr1_2179	p2	(TG)10	20	10196714	10196733
chr1_2180	p2	(TG)15	30	10197301	10197330

chr1_2181	p2	(TG)25	50	10203960	10204009
chr1_2182	p2	(GT)37	74	10205151	10205224
chr1_2183	p2	(AG)8	16	10206444	10206459
chr1_2184	c	(GA)6gc(GA)6	26	10206677	10206702
chr1_2185	p2	(AC)9	18	10208910	10208927
chr1_2186	p2	(GA)6	12	10212191	10212202
chr1_2187	p2	(TG)12	24	10214284	10214307
chr1_2188	p2	(CT)8	16	10216139	10216154
chr1_2189	p2	(AC)11	22	10218831	10218852
chr1_2190	p2	(CA)9	18	10225396	10225413
chr1_2191	p2	(AG)10	20	10226776	10226795
chr1_2192	p2	(GT)10	20	10227887	10227906
chr1_2193	p2	(TG)18	36	10235779	10235814
chr1_2194	p2	(CT)10	20	10236971	10236990
chr1_2195	p2	(CT)9	18	10266520	10266537
chr1_2196	p2	(AC)8	16	10271818	10271833
chr1_2197	p2	(GT)6	12	10272672	10272683
chr1_2198	p2	(TG)10	20	10286191	10286210
chr1_2199	p2	(TG)19	38	10293457	10293494
chr1_2200	c	(GCAC)5gcatgcacgcacg(CA)51	135	10312342	10312476
chr1_2201	p2	(AC)15	30	10327825	10327854
chr1_2202	p2	(TG)10	20	10329830	10329849
chr1_2203	p2	(AC)7	14	10331064	10331077
chr1_2204	p2	(GT)9	18	10344179	10344196
chr1_2205	p2	(AG)6	12	10344773	10344784
chr1_2206	p2	(CA)6	12	10349009	10349020
chr1_2207	p3	(TTA)8	24	10352836	10352859
chr1_2208	c	[illegible]ggaccgggtcgcgggggcagcagtttaagcaaagatgcccagacttccc	123	10358343	10358465
chr1_2209	p2	(TG)20	40	10360423	10360462
chr1_2210	p3	(AGC)5	15	10360678	10360692
chr1_2211	p3	(AGC)5	15	10360980	10360994
chr1_2212	p3	(TAA)5	15	10364864	10364878
chr1_2213	p2	(GT)16	32	10367144	10367175
chr1_2214	c	(GT)10at(GA)13	48	10368301	10368348
chr1_2215	p2	(AG)9	18	10368962	10368979
chr1_2216	p2	(GT)16	32	10370787	10370818
chr1_2217	p4	(ATGG)5	20	10378525	10378544
chr1_2218	p4	(AATG)5	20	10380527	10380546
chr1_2219	p3	(TTA)6	18	10386666	10386683
chr1_2220	c	(GC)6(AC)13	38	10388479	10388516
chr1_2221	c	(AT)6gtatatatac(TG)23	68	10395864	10395931
chr1_2222	p2	(TG)8	16	10399781	10399796
chr1_2223	p2	(AC)9	18	10400757	10400774
chr1_2224	p2	(TG)7	14	10403282	10403295
chr1_2225	p2	(GT)6	12	10419453	10419464
chr1_2226	p2	(GT)20	40	10420449	10420488
chr1_2227	p2	(TG)7	14	10431373	10431386
chr1_2228	p2	(CT)7	14	10439521	10439534
chr1_2229	p2	(AC)7	14	10446362	10446375
chr1_2230	p3	(GAT)5	15	10447837	10447851
chr1_2231	c*	(ATTT)5(TC)8*g(CT)8(AT)9	70	10454510	10454579
chr1_2232	p2	(AC)7	14	10455308	10455321

chr1_2233	p2	(GA)7	14	10459713	10459726
chr1_2234	p2	(CA)8	16	10460293	10460308
chr1_2235	p2	(TG)6	12	10469554	10469565
chr1_2236	p2	(GT)10	20	10469851	10469870
chr1_2237	p2	(AC)6	12	10472536	10472547
chr1_2238	c	(AG)6aa(AG)10	34	10473964	10473997
chr1_2239	p3	(ATT)5	15	10474776	10474790
chr1_2240	p2	(CT)6	12	10483625	10483636
chr1_2241	p2	(CA)7	14	10484293	10484306
chr1_2242	p2	(AG)10	20	10486282	10486301
chr1_2243	p3	(CAG)5	15	10488866	10488880
chr1_2244	p2	(AG)7	14	10494204	10494217
chr1_2245	p2	(AT)7	14	10496391	10496404
chr1_2246	p2	(TG)6	12	10497271	10497282
chr1_2247	p2	(CA)9	18	10504659	10504676
chr1_2248	p5	(CTCTC)5	25	10509013	10509037
chr1_2249	p2	(GT)9	18	10512449	10512466
chr1_2250	p2	(GA)6	12	10512669	10512680
chr1_2251	p2	(CA)7	14	10515526	10515539
chr1_2252	c	(CA)9cg(CA)12	44	10536596	10536639
chr1_2253	c	(AC)15aaactgtgacagatatatatgtgt(AG)6	66	10541888	10541953
chr1_2254	p2	(AC)10	20	10549106	10549125
chr1_2255	p2	(GA)10	20	10552702	10552721
chr1_2256	p2	(CT)6	12	10553647	10553658
chr1_2257	p2	(TG)15	30	10557656	10557685
chr1_2258	p3	(TAC)5	15	10558235	10558249
chr1_2259	p2	(CA)7	14	10561668	10561681
chr1_2260	p2	(AC)13	26	10566663	10566688
chr1_2261	p2	(CA)6	12	10568791	10568802
chr1_2262	p2	(AG)7	14	10571562	10571575
chr1_2263	p2	(CA)19	38	10577989	10578026
chr1_2264	p2	(AC)10	20	10578940	10578959
chr1_2265	c	(ACT)5(AAT)6ac(TAA)6	53	10586848	10586900
chr1_2266	p2	(AG)7	14	10589131	10589144
chr1_2267	p3	(CTG)8	24	10614534	10614557
chr1_2268	p2	(CA)6	12	10616432	10616443
chr1_2269	p2	(GA)6	12	10624019	10624030
chr1_2270	p2	(AG)10	20	10627448	10627467
chr1_2271	p2	(TG)12	24	10628286	10628309
chr1_2272	p2	(AC)6	12	10638145	10638156
chr1_2273	p2	(AC)6	12	10652383	10652394
chr1_2274	p2	(TC)15	30	10664122	10664151
chr1_2275	p2	(CA)6	12	10665138	10665149
chr1_2276	c	ttaataaattaataggcagaatgagaggtgcaagaagtaggggactgggt	109	10667854	10667962
chr1_2277	p2	(CA)12	24	10668393	10668416
chr1_2278	p2	(CT)8	16	10673278	10673293
chr1_2279	c	(CA)6(CT)10	32	10675936	10675967
chr1_2280	p2	(GT)11	22	10679754	10679775
chr1_2281	p3	(ATT)5	15	10684403	10684417
chr1_2282	c	(GT)18gaag(GA)9gg(GT)6	72	10690992	10691063
chr1_2283	p2	(TC)6	12	10703045	10703056
chr1_2284	p2	(TC)6	12	10743436	10743447

chr1_2285	p2	(TC)6	12	10744447	10744458
chr1_2286	p3	(AAC)8	24	10765437	10765460
chr1_2287	p2	(AG)7	14	10766851	10766864
chr1_2288	p3	(ATA)6	18	10773040	10773057
chr1_2289	p2	(TG)6	12	10773824	10773835
chr1_2290	p4	(ATCC)5	20	10774376	10774395
chr1_2291	c	(GT)8gc(GT)11(GA)8	56	10785128	10785183
chr1_2292	p2	(GA)9	18	10786508	10786525
chr1_2293	p2	(TA)7	14	10791719	10791732
chr1_2294	p2	(CA)7	14	10794285	10794298
chr1_2295	p2	(CA)6	12	10800652	10800663
chr1_2296	p2	(AC)7	14	10802149	10802162
chr1_2297	p2	(TC)7	14	10813180	10813193
chr1_2298	p2	(TG)8	16	10815004	10815019
chr1_2299	p3	(TCA)5	15	10817882	10817896
chr1_2300	p2	(TC)7	14	10821362	10821375
chr1_2301	p2	(GT)9	18	10822348	10822365
chr1_2302	p2	(GT)6	12	10824845	10824856
chr1_2303	p2	(AT)6	12	10846973	10846984
chr1_2304	p2	(TG)13	26	10852557	10852582
chr1_2305	p2	(TA)8	16	10853794	10853809
chr1_2306	p2	(TG)20	40	10856071	10856110
chr1_2307	p2	(AC)6	12	10862508	10862519
chr1_2308	p2	(TG)6	12	10869647	10869658
chr1_2309	p2	(AC)6	12	10870306	10870317
chr1_2310	p2	(AC)6	12	10871380	10871391
chr1_2311	p2	(CA)6	12	10874861	10874872
chr1_2312	p2	(TG)6	12	10896600	10896611
chr1_2313	p2	(TG)8	16	10899670	10899685
chr1_2314	c	(CA)11t(AC)15	53	10901741	10901793
chr1_2315	p2	(CA)7	14	10902763	10902776
chr1_2316	p2	(CA)6	12	10907033	10907044
chr1_2317	p2	(AC)6	12	10911452	10911463
chr1_2318	p2	(TA)7	14	10916785	10916798
chr1_2319	p3	(ATA)7	21	10919726	10919746
chr1_2320	p2	(TG)20	40	10923812	10923851
chr1_2321	p2	(AC)7	14	10925043	10925056
chr1_2322	p2	(TG)19	38	10925460	10925497
chr1_2323	p2	(TG)10	20	10926561	10926580
chr1_2324	p2	(TG)6	12	10937784	10937795
chr1_2325	p2	(CA)15	30	10943744	10943773
chr1_2326	p2	(TG)6	12	10945313	10945324
chr1_2327	p3	(ATA)5	15	10946361	10946375
chr1_2328	p2	(TG)7	14	10954583	10954596
chr1_2329	p3	(ATA)5	15	10956496	10956510
chr1_2330	p2	(AC)6	12	10963755	10963766
chr1_2331	p3	(CAC)5	15	10969213	10969227
chr1_2332	p2	(TG)9	18	10969663	10969680
chr1_2333	p2	(GT)10	20	10972969	10972988
chr1_2334	p2	(TG)6	12	10973189	10973200
chr1_2335	p2	(TG)8	16	10977707	10977722
chr1_2336	p2	(GT)16	32	10978058	10978089

chr1_2337	p3	(TCT)5	15	10985232	10985246
chr1_2338	p2	(TG)10	20	11005273	11005292
chr1_2339	p2	(AG)6	12	11006543	11006554
chr1_2340	c	(GT)13ttgc(GT)11	52	11006833	11006884
chr1_2341	p3	(AAT)5	15	11007451	11007465
chr1_2342	p2	(AC)6	12	11018635	11018646
chr1_2343	p2	(TG)18	36	11022303	11022338
chr1_2344	p3	(TTA)6	18	11025709	11025726
chr1_2345	p2	(TG)6	12	11030056	11030067
chr1_2346	p2	(GT)9	18	11032737	11032754
chr1_2347	p2	(GT)6	12	11039825	11039836
chr1_2348	p2	(TG)6	12	11039990	11040001
chr1_2349	p2	(TG)12	24	11042552	11042575
chr1_2350	p2	(AC)6	12	11043049	11043060
chr1_2351	p2	(TG)6	12	11044206	11044217
chr1_2352	p2	(AC)6	12	11058204	11058215
chr1_2353	p2	(GT)19	38	11059501	11059538
chr1_2354	c	(CA)12(GA)8	40	11065307	11065346
chr1_2355	p4	(GTAA)7	28	11066052	11066079
chr1_2356	p2	(CT)11	22	11068802	11068823
chr1_2357	p2	(TG)8	16	11071375	11071390
chr1_2358	p2	(GT)9	18	11075931	11075948
chr1_2359	p2	(AC)8	16	11076090	11076105
chr1_2360	p2	(TG)7	14	11076511	11076524
chr1_2361	p3	(TAA)7	21	11092569	11092589
chr1_2362	c	(TG)8tc(TG)36tttgc(GT)7	109	11106052	11106160
chr1_2363	p3	(ATA)7	21	11106381	11106401
chr1_2364	p2	(TG)6	12	11106683	11106694
chr1_2365	p2	(CA)6	12	11108809	11108820
chr1_2366	p2	(AC)6	12	11116223	11116234
chr1_2367	p2	(AC)7	14	11117232	11117245
chr1_2368	p2	(AC)9	18	11117511	11117528
chr1_2369	p2	(GA)17	34	11122920	11122953
chr1_2370	p2	(AC)17	34	11130180	11130213
chr1_2371	p2	(AC)7	14	11141901	11141914
chr1_2372	p2	(AC)6	12	11142733	11142744
chr1_2373	p2	(AC)6	12	11155954	11155965
chr1_2374	p2	(AC)10	20	11156184	11156203
chr1_2375	p4	(AATC)5	20	11175761	11175780
chr1_2376	p3	(ATT)5	15	11178682	11178696
chr1_2377	p2	(CT)7	14	11181007	11181020
chr1_2378	p3	(TAT)5	15	11194732	11194746
chr1_2379	p2	(TG)6	12	11211900	11211911
chr1_2380	c	(TG)7ta(TG)12	40	11215332	11215371
chr1_2381	p2	(CA)6	12	11216270	11216281
chr1_2382	p2	(GA)7	14	11220011	11220024
chr1_2383	p3	(TTA)5	15	11221427	11221441
chr1_2384	p2	(AC)8	16	11223733	11223748
chr1_2385	p2	(AC)13	26	11228023	11228048
chr1_2386	p2	(AT)6	12	11235289	11235300
chr1_2387	p2	(TG)7	14	11236072	11236085
chr1_2388	p2	(GA)8	16	11253936	11253951

chr1_2389	c	gaccctgtatcaatcacagttcgcattcctcagctctgctcgcctgtacacat	123	11260142	11260264
chr1_2390	p3	(TCA)5	15	11261866	11261880
chr1_2391	c	gtaagaaaaacaagcagggtgtatgtgtgtgacagtccccacttagtaggaa	107	11265809	11265915
chr1_2392	p3	(TTA)9	27	11267021	11267047
chr1_2393	c	(CT)17(CA)16	66	11267312	11267377
chr1_2394	p3	(TAT)5	15	11276587	11276601
chr1_2395	p2	(AC)7	14	11276764	11276777
chr1_2396	p3	(AAT)5	15	11278229	11278243
chr1_2397	p2	(AC)22	44	11309365	11309408
chr1_2398	p2	(AC)11	22	11311728	11311749
chr1_2399	p2	(TC)6	12	11319158	11319169
chr1_2400	p2	(AC)6	12	11338342	11338353
chr1_2401	p2	(AC)12	24	11342256	11342279
chr1_2402	p2	(GT)14	28	11343035	11343062
chr1_2403	p2	(AC)17	34	11343426	11343459
chr1_2404	p2	(GT)11	22	11343566	11343587
chr1_2405	p2	(GT)6	12	11347300	11347311
chr1_2406	p3	(ACT)7	21	11351385	11351405
chr1_2407	p4	(TCCT)12	48	11360104	11360151
chr1_2408	p2	(GT)7	14	11375719	11375732
chr1_2409	p4	(ATAG)5	20	11380774	11380793
chr1_2410	p2	(GT)17	34	11405437	11405470
chr1_2411	p2	(TG)6	12	11408447	11408458
chr1_2412	p2	(AC)6	12	11411443	11411454
chr1_2413	p2	(AC)10	20	11412154	11412173
chr1_2414	c	(TC)7(AC)23	60	11412540	11412599
chr1_2415	p3	(ATT)6	18	11419730	11419747
chr1_2416	p2	(CT)7	14	11420888	11420901
chr1_2417	p3	(TAA)8	24	11426557	11426580
chr1_2418	p2	(AC)13	26	11440310	11440335
chr1_2419	p2	(TG)10	20	11449440	11449459
chr1_2420	p2	(CA)8	16	11454509	11454524
chr1_2421	p2	(TG)6	12	11462683	11462694
chr1_2422	p4	(TCAT)5	20	11469075	11469094
chr1_2423	c	atgcttctatatgccaccactacacatcactaactttgtgtctccgccctcccg	117	11471661	11471777
chr1_2424	p2	(GT)6	12	11473921	11473932
chr1_2425	p3	(AAG)5	15	11480573	11480587
chr1_2426	p2	(TG)6	12	11488263	11488274
chr1_2427	c	(TG)9ttttgtc(TG)8tt(TG)6	55	11490963	11491017
chr1_2428	p2	(GA)7	14	11491124	11491137
chr1_2429	p2	(GT)6	12	11493614	11493625
chr1_2430	p3	(CAA)6	18	11494087	11494104
chr1_2431	p2	(GA)12	24	11508425	11508448
chr1_2432	p2	(GT)7	14	11508588	11508601
chr1_2433	p2	(AC)6	12	11508860	11508871
chr1_2434	p2	(TC)6	12	11510554	11510565
chr1_2435	p2	(CA)6	12	11515745	11515756
chr1_2436	p2	(TG)16	32	11515875	11515906
chr1_2437	p2	(AC)6	12	11522663	11522674
chr1_2438	p4	(ATGG)5	20	11525075	11525094
chr1_2439	p2	(AC)6	12	11526351	11526362
chr1_2440	p2	(TG)6	12	11529492	11529503

chr1_2441	p2	(TG)6	12	11529687	11529698
chr1_2442	p2	(GA)6	12	11532380	11532391
chr1_2443	p2	(CA)7	14	11532520	11532533
chr1_2444	p2	(TG)8	16	11536287	11536302
chr1_2445	p2	(AC)8	16	11544496	11544511
chr1_2446	p2	(TC)7	14	11544636	11544649
chr1_2447	p2	(CA)17	34	11546720	11546753
chr1_2448	p2	(CA)7	14	11546904	11546917
chr1_2449	p2	(TG)6	12	11554746	11554757
chr1_2450	p2	(TA)6	12	11569151	11569162
chr1_2451	p2	(TG)15	30	11570646	11570675
chr1_2452	p2	(GT)16	32	11577126	11577157
chr1_2453	p2	(GT)10	20	11635621	11635640
chr1_2454	p3	(ATA)6	18	11636524	11636541
chr1_2455	p2	(AC)6	12	11647987	11647998
chr1_2456	p2	(GT)8	16	11665836	11665851
chr1_2457	p2	(AT)6	12	11682647	11682658
chr1_2458	p2	(CA)6	12	11694410	11694421
chr1_2459	c	1c(CA)15cccacacccacacaaacatacatatatacacatatagatata	112	11702344	11702455
chr1_2460	p3	(GAT)5	15	11710545	11710559
chr1_2461	p2	(TG)16	32	11713503	11713534
chr1_2462	p2	(CT)7	14	11714573	11714586
chr1_2463	c	ttttacctgctttgtggggcttaaactcagggtgtaggatgactctctct(CA	107	11715029	11715135
chr1_2464	p2	(AC)7	14	11718284	11718297
chr1_2465	p2	(AC)9	18	11722631	11722648
chr1_2466	p3	(AAT)7	21	11723797	11723817
chr1_2467	p2	(TC)9	18	11746324	11746341
chr1_2468	p2	(TG)7	14	11750639	11750652
chr1_2469	p3	(CAT)5	15	11751899	11751913
chr1_2470	p2	(TG)6	12	11754984	11754995
chr1_2471	p2	(TG)28	56	11757689	11757744
chr1_2472	p2	(AG)7	14	11766025	11766038
chr1_2473	c	(TG)6tcagag(CT)6	30	11770990	11771019
chr1_2474	c	(TAA)5(TGA)5	30	11773210	11773239
chr1_2475	p2	(CA)7	14	11773519	11773532
chr1_2476	c	(AT)10(GT)10	40	11774057	11774096
chr1_2477	c*	(ATAC)7(AC)26*	78	11779007	11779084
chr1_2478	p3	(GAA)5	15	11785713	11785727
chr1_2479	c	(AC)13atacacataccgttagccatctgacat(CA)7	67	11786198	11786264
chr1_2480	p2	(CA)6	12	11789766	11789777
chr1_2481	p2	(GT)8	16	11797917	11797932
chr1_2482	p2	(GT)7	14	11799972	11799985
chr1_2483	p3	(AAT)5	15	11835331	11835345
chr1_2484	p2	(AC)8	16	11836702	11836717
chr1_2485	p3	(TTC)8	24	11837470	11837493
chr1_2486	p3	(CAA)6	18	11852216	11852233
chr1_2487	p2	(CA)6	12	11859535	11859546
chr1_2488	c	(CA)6g(TC)9	31	11866727	11866757
chr1_2489	p2	(TC)8	16	11870179	11870194
chr1_2490	p2	(AC)29	58	11874824	11874881
chr1_2491	p2	(GT)9	18	11876304	11876321
chr1_2492	p2	(GT)20	40	11877221	11877260

chr1_2493	p2	(CA)14	28	11877491	11877518
chr1_2494	p2	(TG)6	12	11879347	11879358
chr1_2495	p2	(TA)9	18	11883718	11883735
chr1_2496	p2	(TA)6	12	11887941	11887952
chr1_2497	p2	(AC)10	20	11897096	11897115
chr1_2498	p3	(TTC)5	15	11897393	11897407
chr1_2499	p3	(TTA)7	21	11898851	11898871
chr1_2500	p2	(TC)6	12	11900319	11900330
chr1_2501	p2	(CA)17	34	11906183	11906216
chr1_2502	p3	(CTC)5	15	11934836	11934850
chr1_2503	p2	(CT)6	12	11942072	11942083
chr1_2504	p2	(TG)9	18	11946204	11946221
chr1_2505	p2	(CT)9	18	11953773	11953790
chr1_2506	p2	(CT)10	20	11956712	11956731
chr1_2507	p2	(TG)20	40	11972480	11972519
chr1_2508	p2	(AC)11	22	11975100	11975121
chr1_2509	p2	(TG)10	20	11975632	11975651
chr1_2510	p2	(TG)7	14	11975834	11975847
chr1_2511	p3	(CAT)6	18	11983806	11983823
chr1_2512	p4	(AGAT)10	40	11988606	11988645
chr1_2513	p2	(CA)9	18	11989632	11989649
chr1_2514	c	(CT)7tt(CA)6ct(CA)6	42	11990529	11990570
chr1_2515	p2	(TA)6	12	12010575	12010586
chr1_2516	p2	(CA)6	12	12024201	12024212
chr1_2517	p2	(CA)13	26	12028888	12028913
chr1_2518	p2	(TG)6	12	12030739	12030750
chr1_2519	p2	(GT)11	22	12034311	12034332
chr1_2520	c	(GGA)8gggagaagagacaggacagttgtgtgggtc(TG)18	90	12035295	12035384
chr1_2521	p2	(CA)22	44	12051955	12051998
chr1_2522	p3	(TGA)6	18	12056596	12056613
chr1_2523	p2	(GA)6	12	12059185	12059196
chr1_2524	p2	(AG)7	14	12066689	12066702
chr1_2525	p2	(TG)12	24	12090594	12090617
chr1_2526	p4	(GTAG)7	28	12100730	12100757
chr1_2527	p2	(GT)6	12	12101560	12101571
chr1_2528	p2	(CA)19	38	12106909	12106946
chr1_2529	p4	(CATC)5	20	12111367	12111386
chr1_2530	p4	(CATC)5	20	12119151	12119170
chr1_2531	p2	(AC)14	28	12129366	12129393
chr1_2532	p3	(AAT)7	21	12133328	12133348
chr1_2533	p4	(AACT)6	24	12134148	12134171
chr1_2534	p2	(CT)8	16	12135960	12135975
chr1_2535	p2	(CA)8	16	12167551	12167566
chr1_2536	p2	(TG)6	12	12177842	12177853
chr1_2537	p2	(TC)6	12	12206570	12206581
chr1_2538	p2	(CT)6	12	12207698	12207709
chr1_2539	p2	(TA)6	12	12221747	12221758
chr1_2540	c	(GT)7atatacatatac(AT)7	40	12226352	12226391
chr1_2541	p2	(GT)12	24	12239983	12240006
chr1_2542	p2	(TA)6	12	12250680	12250691
chr1_2543	p2	(GT)7	14	12258463	12258476
chr1_2544	p2	(AC)22	44	12264034	12264077

chr1_2545	p2	(TG)19	38	12265424	12265461
chr1_2546	p2	(GT)10	20	12269873	12269892
chr1_2547	p3	(TGG)5	15	12277638	12277652
chr1_2548	p2	(TG)12	24	12279402	12279425
chr1_2549	p4	(CATC)5	20	12284853	12284872
chr1_2550	p2	(TC)8	16	12297836	12297851
chr1_2551	p2	(GT)7	14	12299284	12299297
chr1_2552	p4	(ATGG)5	20	12300077	12300096
chr1_2553	p2	(TA)7	14	12304732	12304745
chr1_2554	p2	(GT)7	14	12311080	12311093
chr1_2555	p2	(TA)10	20	12315539	12315558
chr1_2556	p4	(GATA)7	28	12325476	12325503
chr1_2557	p2	(AT)6	12	12329496	12329507
chr1_2558	p3	(TAG)5	15	12344816	12344830
chr1_2559	p2	(AC)6	12	12350692	12350703
chr1_2560	p2	(CA)6	12	12353008	12353019
chr1_2561	p2	(CA)6	12	12365350	12365361
chr1_2562	c	(AAT)5a(AAT)6	34	12369046	12369079
chr1_2563	p2	(AG)13	26	12376266	12376291
chr1_2564	p2	(AC)6	12	12378089	12378100
chr1_2565	p2	(AT)8	16	12380164	12380179
chr1_2566	p2	(AC)9	18	12394810	12394827
chr1_2567	p2	(CA)7	14	12399146	12399159
chr1_2568	p3	(ATA)8	24	12403310	12403333
chr1_2569	p2	(CA)16	32	12411070	12411101
chr1_2570	c	(TG)6c(GT)6	25	12413193	12413217
chr1_2571	p2	(GT)6	12	12418404	12418415
chr1_2572	p3	(TTA)5	15	12420501	12420515
chr1_2573	p4	(TTTA)5	20	12425610	12425629
chr1_2574	p3	(AGT)6	18	12440248	12440265
chr1_2575	p4	(AATG)5	20	12449903	12449922
chr1_2576	p3	(AAC)5	15	12452687	12452701
chr1_2577	c	(GT)9gcgc(GT)8	38	12455927	12455964
chr1_2578	p2	(CA)18	36	12469734	12469769
chr1_2579	p2	(AC)6	12	12485543	12485554
chr1_2580	p2	(TG)6	12	12487954	12487965
chr1_2581	p2	(AC)9	18	12493058	12493075
chr1_2582	p4	(CAAA)5	20	12529023	12529042
chr1_2583	p2	(CA)8	16	12543134	12543149
chr1_2584	p2	(GT)6	12	12548631	12548642
chr1_2585	c	aaggattttccctccactacagaagtgaggcagccatgtgcagcacgaac	87	12549245	12549331
chr1_2586	p2	(AT)11	22	12584270	12584291
chr1_2587	p3	(ACT)13	39	12596273	12596311
chr1_2588	p3	(TTG)9	27	12624193	12624219
chr1_2589	p4	(AAAT)5	20	12628671	12628690
chr1_2590	p3	(GAG)7	21	12640499	12640519
chr1_2591	p4	(AAAT)5	20	12655702	12655721
chr1_2592	p2	(GA)8	16	12658219	12658234
chr1_2593	p3	(TAT)5	15	12660302	12660316
chr1_2594	c	(CTA)7(ATA)6	39	12667756	12667794
chr1_2595	c	cactgtaggatgatgggtgttctcctaaaatggcctgtctttgccttcccttt	125	12674991	12675115
chr1_2596	p2	(TC)6	12	12683645	12683656

chr1_2597	p2	(CA)6	12	12687254	12687265
chr1_2598	p3	(AAC)5	15	12690101	12690115
chr1_2599	p2	(GA)8	16	12697158	12697173
chr1_2600	p2	(GT)6	12	12697492	12697503
chr1_2601	p2	(CA)17	34	12706005	12706038
chr1_2602	p2	(TG)9	18	12708822	12708839
chr1_2603	p4	(CATC)5	20	12722279	12722298
chr1_2604	p2	(AC)7	14	12729045	12729058
chr1_2605	p2	(CA)13	26	12735545	12735570
chr1_2606	p4	(AGAT)19	76	12736902	12736977
chr1_2607	p2	(GT)7	14	12747671	12747684
chr1_2608	p3	(CAA)7	21	12750595	12750615
chr1_2609	p2	(GT)13	26	12757754	12757779
chr1_2610	p4	(TCTA)5	20	12766135	12766154
chr1_2611	c	actcaccggtgtctgtcatggctgctggttggcaggctctctgctggcttgct	152	12766259	12766410
chr1_2612	c	(CT)6ttgtccctgctcccactgcgcttgcgca(CT)11	62	12766546	12766607
chr1_2613	p3	(AAT)5	15	12770565	12770579
chr1_2614	p2	(GT)6	12	12774079	12774090
chr1_2615	c	(TG)8a(GT)6	29	12783699	12783727
chr1_2616	p2	(AT)6	12	12784518	12784529
chr1_2617	p2	(GT)8	16	12785270	12785285
chr1_2618	c	(TG)13(AG)12	50	12791634	12791683
chr1_2619	p2	(AG)30	60	12812290	12812349
chr1_2620	p2	(AT)6	12	12825910	12825921
chr1_2621	p3	(TTC)7	21	12832562	12832582
chr1_2622	p4	(TATC)5	20	12837978	12837997
chr1_2623	p2	(TG)11	22	12840525	12840546
chr1_2624	p2	(GT)11	22	12849794	12849815
chr1_2625	p4	(ATGG)5	20	12855369	12855388
chr1_2626	p2	(GT)26	52	12864782	12864833
chr1_2627	p2	(GT)10	20	12864939	12864958
chr1_2628	p2	(AC)8	16	12873816	12873831
chr1_2629	p2	(AC)7	14	12880281	12880294
chr1_2630	p2	(TG)6	12	12880712	12880723
chr1_2631	p2	(CT)7	14	12886905	12886918
chr1_2632	p3	(TTG)5	15	12891702	12891716
chr1_2633	p2	(TG)7	14	12894285	12894298
chr1_2634	p2	(TG)14	28	12908792	12908819
chr1_2635	c	(GT)26(GA)9	70	12919519	12919588
chr1_2636	p2	(TG)6	12	12936402	12936413
chr1_2637	p2	(CA)14	28	12937173	12937200
chr1_2638	p2	(CA)22	44	12937830	12937873
chr1_2639	p2	(AC)9	18	12938819	12938836
chr1_2640	p2	(AC)12	24	12941102	12941125
chr1_2641	p2	(CA)7	14	12949047	12949060
chr1_2642	p2	(TG)15	30	12955904	12955933
chr1_2643	p2	(GT)14	28	12956563	12956590
chr1_2644	p3	(TTG)6	18	12962501	12962518
chr1_2645	p2	(TG)14	28	12966893	12966920
chr1_2646	p2	(CA)8	16	12969365	12969380
chr1_2647	p3	(ATG)5	15	12970478	12970492
chr1_2648	p2	(CA)8	16	12979731	12979746

chr1_2649	p2	(AT)6	12	12998886	12998897
chr1_2650	p4	(TAAA)5	20	12999060	12999079
chr1_2651	p2	(AC)9	18	13021822	13021839
chr1_2652	p2	(AC)6	12	13025520	13025531
chr1_2653	p3	(TAA)5	15	13026438	13026452
chr1_2654	p2	(GT)6	12	13032837	13032848
chr1_2655	p2	(TG)8	16	13048022	13048037
chr1_2656	p4	(ATGG)5	20	13048275	13048294
chr1_2657	c	G)5gtggatgggtatagatatatata(ATGG)5gtatagatatatata(A	98	13048524	13048621
chr1_2658	p2	(CA)14	28	13050052	13050079
chr1_2659	p3	(ATA)5	15	13063955	13063969
chr1_2660	c	(TTA)7tttatttattat(TTA)5	48	13067532	13067579
chr1_2661	p4	(TGAT)5	20	13076218	13076237
chr1_2662	p3	(CAC)5	15	13082085	13082099
chr1_2663	p4	(TAGA)5	20	13089027	13089046
chr1_2664	p2	(TG)10	20	13094121	13094140
chr1_2665	p2	(TG)6	12	13095802	13095813
chr1_2666	c	acattgagtgtaaattagtgatgtctggccattttctttccacaaaaacaggaa	174	13098588	13098761
chr1_2667	p5	(GATAA)5	25	13109860	13109884
chr1_2668	p2	(GT)10	20	13111236	13111255
chr1_2669	p2	(TC)38	76	13112507	13112582
chr1_2670	c	(CT)21cacact(CA)8	64	13113202	13113265
chr1_2671	p2	(GT)6	12	13119091	13119102
chr1_2672	p2	(AC)7	14	13119536	13119549
chr1_2673	p4	(ATGG)5	20	13123048	13123067
chr1_2674	p4	(AATC)5	20	13124238	13124257
chr1_2675	c	(GAT)6gaa(GAG)7	42	13124732	13124773
chr1_2676	c	aatgctgctgattaaagacagtagaaggaatgtgggagtggatgcatttaga	126	13128735	13128860
chr1_2677	p5	(AAATC)5	25	13138992	13139016
chr1_2678	p2	(TG)7	14	13148226	13148239
chr1_2679	p2	(TG)10	20	13151777	13151796
chr1_2680	p2	(TG)7	14	13158235	13158248
chr1_2681	p3	(AGA)5	15	13170966	13170980
chr1_2682	p2	(TG)9	18	13172639	13172656
chr1_2683	p3	(ATT)7	21	13184245	13184265
chr1_2684	p2	(GT)15	30	13186374	13186403
chr1_2685	p4	(TATG)5	20	13187836	13187855
chr1_2686	p4	(TTAT)5	20	13188464	13188483
chr1_2687	p2	(TG)15	30	13190386	13190415
chr1_2688	p2	(GT)12	24	13193492	13193515
chr1_2689	p3	(ATG)7	21	13201686	13201706
chr1_2690	p2	(AC)6	12	13202039	13202050
chr1_2691	p2	(CT)10	20	13215543	13215562
chr1_2692	p2	(CA)6	12	13218367	13218378
chr1_2693	p2	(AC)6	12	13236547	13236558
chr1_2694	p5	(TGATT)5	25	13250644	13250668
chr1_2695	p2	(TC)10	20	13293029	13293048
chr1_2696	p3	(GAA)5	15	13294547	13294561
chr1_2697	p3	(CAG)6	18	13299223	13299240
chr1_2698	p2	(TG)13	26	13313415	13313440
chr1_2699	p2	(CA)6	12	13313598	13313609
chr1_2700	c	(CA)8t(AC)10	37	13314347	13314383

chr1_2701	c	(TC)12(AC)29	82	13323972	13324053
chr1_2702	p2	(CA)7	14	13331593	13331606
chr1_2703	c	(AC)7gcg(CA)7cg(CA)10	53	13343321	13343373
chr1_2704	p2	(GT)8	16	13348342	13348357
chr1_2705	p3	(CAC)5	15	13348790	13348804
chr1_2706	p2	(AC)16	32	13349574	13349605
chr1_2707	p3	(AAC)7	21	13349868	13349888
chr1_2708	p2	(TG)6	12	13352272	13352283
chr1_2709	p2	(GT)6	12	13353058	13353069
chr1_2710	p3	(TAA)7	21	13371284	13371304
chr1_2711	p2	(AC)6	12	13378754	13378765
chr1_2712	p4	(TCTA)5	20	13380107	13380126
chr1_2713	p2	(AC)6	12	13384072	13384083
chr1_2714	p4	(ATAC)7	28	13403132	13403159
chr1_2715	c	(CA)8a(AC)6	29	13415228	13415256
chr1_2716	p2	(CA)18	36	13416725	13416760
chr1_2717	p2	(CA)7	14	13416987	13417000
chr1_2718	p2	(AC)17	34	13417464	13417497
chr1_2719	p3	(AGT)6	18	13418406	13418423
chr1_2720	p2	(TG)11	22	13427072	13427093
chr1_2721	p3	(TTA)5	15	13429072	13429086
chr1_2722	p2	(AC)6	12	13431045	13431056
chr1_2723	p2	(TC)6	12	13439806	13439817
chr1_2724	p2	(CA)15	30	13452741	13452770
chr1_2725	c	(ATT)6(AGT)19	75	13462389	13462463
chr1_2726	p2	(GA)11	22	13465406	13465427
chr1_2727	p4	(ATCT)7	28	13471684	13471711
chr1_2728	p2	(CA)8	16	13473449	13473464
chr1_2729	p2	(AC)6	12	13488427	13488438
chr1_2730	p2	(CA)15	30	13488816	13488845
chr1_2731	p3	(CAT)9	27	13489284	13489310
chr1_2732	p3	(TAT)6	18	13490220	13490237
chr1_2733	c	(TTTG)8ttttagttttctctcttg(CT)7	64	13492275	13492338
chr1_2734	c	(TG)21(AG)12	66	13492822	13492887
chr1_2735	p5	(TATGC)17	85	13493358	13493442
chr1_2736	p2	(AC)6	12	13501758	13501769
chr1_2737	c	(TAA)5c(ATT)5	31	13503139	13503169
chr1_2738	c	(TCTG)6(TCTA)5	44	13503700	13503743
chr1_2739	p2	(AC)20	40	13503856	13503895
chr1_2740	p2	(GA)11	22	13505819	13505840
chr1_2741	c	(AC)10atatatatatg(TA)6	43	13509029	13509071
chr1_2742	p2	(GA)12	24	13509878	13509901
chr1_2743	p2	(GT)12	24	13510972	13510995
chr1_2744	p3	(TGG)5	15	13522228	13522242
chr1_2745	c	(GT)7(GA)6	26	13533046	13533071
chr1_2746	p2	(TG)8	16	13535027	13535042
chr1_2747	p2	(GT)7	14	13535349	13535362
chr1_2748	p2	(AC)7	14	13535641	13535654
chr1_2749	p2	(TG)25	50	13535843	13535892
chr1_2750	p2	(CA)9	18	13539033	13539050
chr1_2751	p2	(AC)11	22	13539963	13539984
chr1_2752	p2	(TG)6	12	13542992	13543003

chr1_2753	p3	(CCA)5	15	13551657	13551671
chr1_2754	p2	(TG)6	12	13551854	13551865
chr1_2755	p3	(ATA)5	15	13552521	13552535
chr1_2756	p5	(ATATA)5	25	13554739	13554763
chr1_2757	p2	(TG)17	34	13558144	13558177
chr1_2758	p4	(TTCT)8	32	13558767	13558798
chr1_2759	c	)10gtcccataatttatggtgtgcattggagtggtgcattttgaccaggca(	106	13560741	13560846
chr1_2760	p4	(ATGG)5	20	13563139	13563158
chr1_2761	p3	(TGA)5	15	13563767	13563781
chr1_2762	p2	(AC)9	18	13583503	13583520
chr1_2763	p5	(AGAAG)7	35	13597080	13597114
chr1_2764	p2	(TC)8	16	13604216	13604231
chr1_2765	p2	(CA)10	20	13606626	13606645
chr1_2766	p3	(GAG)5	15	13609624	13609638
chr1_2767	p2	(AG)12	24	13611282	13611305
chr1_2768	p2	(TG)6	12	13620605	13620616
chr1_2769	p2	(GT)16	32	13622167	13622198
chr1_2770	p2	(TA)8	16	13633954	13633969
chr1_2771	p2	(AC)7	14	13646748	13646761
chr1_2772	p2	(TC)7	14	13664824	13664837
chr1_2773	p2	(TA)7	14	13665148	13665161
chr1_2774	p2	(CT)6	12	13667375	13667386
chr1_2775	p2	(AC)8	16	13670463	13670478
chr1_2776	p4	(TTTC)5	20	13674684	13674703
chr1_2777	p2	(GT)6	12	13684000	13684011
chr1_2778	p3	(ATT)5	15	13687366	13687380
chr1_2779	p4	(ATGG)5	20	13688744	13688763
chr1_2780	p2	(CA)8	16	13698204	13698219
chr1_2781	p2	(AT)7	14	13705417	13705430
chr1_2782	p2	(GT)8	16	13705935	13705950
chr1_2783	p2	(AC)6	12	13709685	13709696
chr1_2784	p2	(AC)12	24	13710177	13710200
chr1_2785	p2	(TG)8	16	13710846	13710861
chr1_2786	p2	(AG)11	22	13714490	13714511
chr1_2787	p4	(AATA)5	20	13725471	13725490
chr1_2788	p2	(AC)6	12	13753951	13753962
chr1_2789	c	G)18tctgagcgaat(AC)7ctctgctttaccccaggaaaagcagag(G	100	13757134	13757233
chr1_2790	p2	(GT)21	42	13763986	13764027
chr1_2791	p2	(TG)10	20	13779489	13779508
chr1_2792	p2	(TG)10	20	13783287	13783306
chr1_2793	p2	(AT)8	16	13793146	13793161
chr1_2794	p2	(AC)9	18	13815289	13815306
chr1_2795	p2	(TC)7	14	13818876	13818889
chr1_2796	p2	(GA)6	12	13833830	13833841
chr1_2797	p2	(CT)7	14	13842078	13842091
chr1_2798	p2	(GT)7	14	13843146	13843159
chr1_2799	c	(GT)25(GA)10	70	13849575	13849644
chr1_2800	p4	(TCCA)6	24	13850353	13850376
chr1_2801	p3	(CAT)8	24	13881889	13881912
chr1_2802	p2	(AC)6	12	13885153	13885164
chr1_2803	p2	(CA)6	12	13887001	13887012
chr1_2804	p2	(CA)8	16	13889012	13889027

chr1_2805	p2	(GT)18	36	13893379	13893414
chr1_2806	p2	(AC)11	22	13910152	13910173
chr1_2807	c	(GT)7gacagatttttgggtttgctgg(GT)6	49	13912276	13912324
chr1_2808	c	(GT)7(GA)17	48	13926171	13926218
chr1_2809	p2	(TG)10	20	13926362	13926381
chr1_2810	p2	(TC)6	12	13927695	13927706
chr1_2811	p2	(AT)11	22	13940409	13940430
chr1_2812	p3	(TGC)5	15	13945042	13945056
chr1_2813	p5	(GAAGA)5	25	13946923	13946947
chr1_2814	p2	(AC)11	22	13958294	13958315
chr1_2815	p2	(TG)7	14	13960955	13960968
chr1_2816	p2	(GT)7	14	13965190	13965203
chr1_2817	p2	(TG)20	40	13971236	13971275
chr1_2818	p2	(TG)9	18	13975657	13975674
chr1_2819	p3	(TGT)5	15	13980375	13980389
chr1_2820	p2	(CA)14	28	13984022	13984049
chr1_2821	p2	(AC)19	38	13985658	13985695
chr1_2822	p2	(AG)7	14	13997897	13997910
chr1_2823	p2	(TG)6	12	13999818	13999829
chr1_2824	p2	(AC)14	28	14004464	14004491
chr1_2825	p3	(TTG)7	21	14007003	14007023
chr1_2826	c	(TA)7(TG)11	36	14032902	14032937
chr1_2827	p4	(CATC)5	20	14049834	14049853
chr1_2828	p2	(AT)7	14	14075906	14075919
chr1_2829	p3	(TAA)6	18	14077105	14077122
chr1_2830	p2	(GT)7	14	14077446	14077459
chr1_2831	p2	(TA)6	12	14090472	14090483
chr1_2832	p2	(GT)16	32	14097945	14097976
chr1_2833	p3	(GGT)6	18	14099405	14099422
chr1_2834	p2	(AC)7	14	14104838	14104851
chr1_2835	p2	(CA)6	12	14107523	14107534
chr1_2836	p2	(AC)8	16	14109077	14109092
chr1_2837	p2	(AC)6	12	14109996	14110007
chr1_2838	p2	(GT)7	14	14111103	14111116
chr1_2839	c	(GT)12at(GA)6	38	14111751	14111788
chr1_2840	p2	(TG)12	24	14112127	14112150
chr1_2841	p2	(TG)6	12	14128157	14128168
chr1_2842	p3	(TAT)9	27	14129422	14129448
chr1_2843	p2	(TG)6	12	14147754	14147765
chr1_2844	p2	(TG)6	12	14164772	14164783
chr1_2845	p2	(CA)13	26	14170548	14170573
chr1_2846	p2	(GA)11	22	14181274	14181295
chr1_2847	p2	(TG)19	38	14182434	14182471
chr1_2848	p2	(AC)7	14	14183024	14183037
chr1_2849	p2	(AC)9	18	14185273	14185290
chr1_2850	p3	(CTC)5	15	14190785	14190799
chr1_2851	p2	(TG)6	12	14191743	14191754
chr1_2852	p4	(TATT)5	20	14201278	14201297
chr1_2853	p4	(ATCC)6	24	14214558	14214581
chr1_2854	p3	(TTC)6	18	14225935	14225952
chr1_2855	p2	(AC)6	12	14239238	14239249
chr1_2856	p2	(AG)7	14	14240857	14240870

chr1_2857	p4	(CCTC)5	20	14245143	14245162
chr1_2858	p3	(AAC)5	15	14252989	14253003
chr1_2859	p2	(TG)19	38	14253732	14253769
chr1_2860	p3	(AAC)5	15	14254600	14254614
chr1_2861	p2	(AC)7	14	14278966	14278979
chr1_2862	p5	(AAATC)5	25	14282212	14282236
chr1_2863	p2	(GT)10	20	14298238	14298257
chr1_2864	p2	(TC)7	14	14299665	14299678
chr1_2865	p2	(GT)8	16	14302243	14302258
chr1_2866	p2	(AC)10	20	14314946	14314965
chr1_2867	p2	(CA)6	12	14318730	14318741
chr1_2868	p2	(GT)10	20	14320660	14320679
chr1_2869	p2	(AG)9	18	14330017	14330034
chr1_2870	p2	(TA)7	14	14333311	14333324
chr1_2871	p2	(AC)17	34	14339838	14339871
chr1_2872	p2	(AC)16	32	14346391	14346422
chr1_2873	p2	(TC)20	40	14361583	14361622
chr1_2874	p2	(TG)15	30	14370389	14370418
chr1_2875	p2	(TG)13	26	14378135	14378160
chr1_2876	p2	(AC)7	14	14399082	14399095
chr1_2877	p4	(CATC)6	24	14416591	14416614
chr1_2878	p4	(TTTG)5	20	14431044	14431063
chr1_2879	p2	(AC)6	12	14436781	14436792
chr1_2880	c	(AC)7ggg(GA)6	29	14439044	14439072
chr1_2881	p4	(CTAT)5	20	14445729	14445748
chr1_2882	p4	(AAAC)5	20	14452851	14452870
chr1_2883	p3	(ATA)5	15	14454787	14454801
chr1_2884	p2	(TG)6	12	14460352	14460363
chr1_2885	p2	(GT)10	20	14462665	14462684
chr1_2886	p2	(CT)6	12	14474329	14474340
chr1_2887	p2	(AC)6	12	14477736	14477747
chr1_2888	p3	(AAC)7	21	14492397	14492417
chr1_2889	c	actggcaactcattatttttcttttagatcagctgtggataaaggaaatt(TG	98	14505886	14505983
chr1_2890	p4	(ATCT)6	24	14512047	14512070
chr1_2891	p2	(TG)10	20	14513828	14513847
chr1_2892	p2	(CA)25	50	14514596	14514645
chr1_2893	p2	(AT)7	14	14515403	14515416
chr1_2894	p4	(TTTA)5	20	14515958	14515977
chr1_2895	c	(TAA)8tagtaataatg(ATA)6	53	14519730	14519782
chr1_2896	p3	(AAT)7	21	14524929	14524949
chr1_2897	p3	(ATT)8	24	14526930	14526953
chr1_2898	p2	(AG)6	12	14527066	14527077
chr1_2899	p2	(AC)6	12	14534389	14534400
chr1_2900	c	GT)7ctctgtgctacatgaacacatgcttgtatgtgtgtgtttgcacac(TG	71	14538349	14538419
chr1_2901	p2	(TG)9	18	14538786	14538803
chr1_2902	c	(AC)6tacaactgtat(AC)6	35	14543820	14543854
chr1_2903	p2	(GT)21	42	14557772	14557813
chr1_2904	p2	(GT)6	12	14560570	14560581
chr1_2905	p3	(ATC)5	15	14561602	14561616
chr1_2906	p2	(GA)7	14	14562150	14562163
chr1_2907	p2	(GA)11	22	14569894	14569915
chr1_2908	p2	(TG)7	14	14570405	14570418

chr1_2909	p2	(CA)6	12	14571240	14571251
chr1_2910	c	(GA)17gg(GA)8	52	14571493	14571544
chr1_2911	p2	(TG)10	20	14572257	14572276
chr1_2912	p2	(TG)6	12	14572645	14572656
chr1_2913	p3	(GGT)5	15	14579019	14579033
chr1_2914	c	(AC)6gggcacacact(CA)6	35	14581268	14581302
chr1_2915	p2	(GT)16	32	14583647	14583678
chr1_2916	p3	(TGT)6	18	14600603	14600620
chr1_2917	p3	(AAC)5	15	14602597	14602611
chr1_2918	p2	(CT)18	36	14610365	14610400
chr1_2919	p2	(AG)6	12	14613144	14613155
chr1_2920	p2	(CA)6	12	14623746	14623757
chr1_2921	p2	(GA)6	12	14625209	14625220
chr1_2922	p2	(CA)10	20	14634604	14634623
chr1_2923	p2	(CT)6	12	14637334	14637345
chr1_2924	p2	(AC)8	16	14640493	14640508
chr1_2925	p2	(AC)7	14	14648536	14648549
chr1_2926	p2	(AC)9	18	14657564	14657581
chr1_2927	p2	(GA)7	14	14662109	14662122
chr1_2928	p2	(CA)7	14	14664544	14664557
chr1_2929	c	(AC)6at(AC)17	48	14673235	14673282
chr1_2930	p2	(TG)14	28	14675280	14675307
chr1_2931	p2	(AC)18	36	14688116	14688151
chr1_2932	p3	(TGC)5	15	14698868	14698882
chr1_2933	p2	(TG)17	34	14705117	14705150
chr1_2934	p3	(TAT)5	15	14708273	14708287
chr1_2935	p2	(TC)7	14	14717183	14717196
chr1_2936	c	(GC)7(AC)26	66	14739388	14739453
chr1_2937	c	(CA)26(GA)8	68	14739642	14739709
chr1_2938	p2	(AC)7	14	14743181	14743194
chr1_2939	p3	(TAG)10	30	14750202	14750231
chr1_2940	p2	(TG)7	14	14750963	14750976
chr1_2941	c	(TG)8catgctttt(TTTG)6	49	14757106	14757154
chr1_2942	p2	(TG)11	22	14758835	14758856
chr1_2943	c	(TG)10tt(TG)13	48	14771993	14772040
chr1_2944	p2	(GT)9	18	14792656	14792673
chr1_2945	p2	(GT)10	20	14807596	14807615
chr1_2946	p2	(TG)16	32	14809963	14809994
chr1_2947	c	(TG)7agaa(AG)14	46	14811964	14812009
chr1_2948	c	(TA)8tt(TA)9	36	14812802	14812837
chr1_2949	p2	(TG)11	22	14816540	14816561
chr1_2950	p2	(TG)12	24	14819023	14819046
chr1_2951	p4	(ATAA)6	24	14819190	14819213
chr1_2952	p2	(CA)6	12	14823257	14823268
chr1_2953	p2	(AC)21	42	14836255	14836296
chr1_2954	p2	(TG)6	12	14838361	14838372
chr1_2955	c	(TG)8(AG)7	30	14838879	14838908
chr1_2956	p2	(CA)11	22	14847479	14847500
chr1_2957	p2	(CA)20	40	14851208	14851247
chr1_2958	p3	(TTG)5	15	14851723	14851737
chr1_2959	c	(CAG)5(TAG)7	36	14858412	14858447
chr1_2960	p4	(CATC)5	20	14863002	14863021

chr1_2961	p2	(TG)34	68	14869333	14869400
chr1_2962	c	(TG)27c(GT)7	69	14881590	14881658
chr1_2963	p3	(TTA)5	15	14882032	14882046
chr1_2964	p3	(TTC)6	18	14887158	14887175
chr1_2965	p2	(TG)11	22	14891049	14891070
chr1_2966	p2	(AC)14	28	14901928	14901955
chr1_2967	p3	(TGT)5	15	14911737	14911751
chr1_2968	p2	(TG)7	14	14912358	14912371
chr1_2969	p2	(TG)6	12	14918091	14918102
chr1_2970	p2	(CA)7	14	14918819	14918832
chr1_2971	p2	(GT)12	24	14919967	14919990
chr1_2972	p3	(GGA)5	15	14920989	14921003
chr1_2973	p2	(TG)10	20	14923003	14923022
chr1_2974	p3	(GAG)10	30	14925062	14925091
chr1_2975	p2	(GT)11	22	14931795	14931816
chr1_2976	p2	(GT)6	12	14932786	14932797
chr1_2977	p2	(TC)6	12	14937196	14937207
chr1_2978	p2	(GT)7	14	14940399	14940412
chr1_2979	p2	(CT)8	16	14942511	14942526
chr1_2980	p2	(TG)6	12	14943709	14943720
chr1_2981	p2	(TG)8	16	14944314	14944329
chr1_2982	p3	(ATC)15	45	14947149	14947193
chr1_2983	p2	(TG)14	28	14953952	14953979
chr1_2984	p3	(AGG)7	21	14954710	14954730
chr1_2985	p2	(CT)6	12	14957642	14957653
chr1_2986	p2	(TG)8	16	14957915	14957930
chr1_2987	p2	(GT)18	36	14959772	14959807
chr1_2988	p2	(AG)16	32	14961756	14961787
chr1_2989	p3	(TCC)5	15	14963231	14963245
chr1_2990	p2	(GT)16	32	14964497	14964528
chr1_2991	p2	(CA)10	20	14965968	14965987
chr1_2992	p3	(GTA)8	24	14966918	14966941
chr1_2993	p3	(GAG)5	15	14967188	14967202
chr1_2994	p2	(AC)11	22	14967678	14967699
chr1_2995	p2	(GT)8	16	14972760	14972775
chr1_2996	p2	(AG)12	24	14994226	14994249
chr1_2997	p2	(GA)6	12	15007874	15007885
chr1_2998	p2	(TA)9	18	15009996	15010013
chr1_2999	p2	(GT)7	14	15015048	15015061
chr1_3000	p4	(CCTT)5	20	15026449	15026468
chr1_3001	p2	(AC)8	16	15027274	15027289
chr1_3002	p2	(TG)17	34	15029676	15029709
chr1_3003	c	tccatgataataaataggcccagagcacagtttgaaaccagatacctttgtg	147	15030677	15030823
chr1_3004	p2	(AG)6	12	15038760	15038771
chr1_3005	p4	(CATC)5	20	15042376	15042395
chr1_3006	p2	(AC)8	16	15053315	15053330
chr1_3007	p2	(AC)10	20	15059183	15059202
chr1_3008	p2	(CA)25	50	15059627	15059676
chr1_3009	p4	(TTTG)5	20	15062715	15062734
chr1_3010	c	(TG)7(TA)6tg(TA)7	42	15069390	15069431
chr1_3011	p2	(AC)10	20	15074522	15074541
chr1_3012	p2	(TG)10	20	15101161	15101180

chr1_3013	c	(CA)11cg(CA)21	66	15111263	15111328
chr1_3014	p2	(GT)6	12	15128370	15128381
chr1_3015	p2	(AC)8	16	15129431	15129446
chr1_3016	p4	(ATGG)5	20	15138398	15138417
chr1_3017	p2	(TG)7	14	15155069	15155082
chr1_3018	p2	(TG)16	32	15158691	15158722
chr1_3019	p2	(TG)6	12	15173282	15173293
chr1_3020	p2	(TG)9	18	15178838	15178855
chr1_3021	p2	(TC)7	14	15183918	15183931
chr1_3022	c	(GT)6a(TG)7	27	15185268	15185294
chr1_3023	p2	(TG)6	12	15186789	15186800
chr1_3024	p4	(TAAA)5	20	15195627	15195646
chr1_3025	c	(TGC)5(TGA)6	33	15201026	15201058
chr1_3026	p2	(GA)6	12	15201966	15201977
chr1_3027	c	(AC)12agggccac(GA)6	44	15204754	15204797
chr1_3028	p3	(ATA)5	15	15205797	15205811
chr1_3029	p2	(GA)13	26	15208212	15208237
chr1_3030	p2	(AC)8	16	15215432	15215447
chr1_3031	p2	(CA)7	14	15218022	15218035
chr1_3032	p2	(GT)9	18	15241845	15241862
chr1_3033	c	(TG)6tacaa(AC)9	35	15246083	15246117
chr1_3034	p4	(ATTT)5	20	15250389	15250408
chr1_3035	p2	(GT)7	14	15255905	15255918
chr1_3036	p2	(AG)7	14	15259391	15259404
chr1_3037	p2	(CA)12	24	15260978	15261001
chr1_3038	c	(TG)9ttt(GA)8	37	15264062	15264098
chr1_3039	p2	(CA)16	32	15273225	15273256
chr1_3040	p2	(TG)6	12	15273386	15273397
chr1_3041	p2	(GT)7	14	15280464	15280477
chr1_3042	p3	(TTG)5	15	15281702	15281716
chr1_3043	p2	(CA)11	22	15282556	15282577
chr1_3044	p2	(CA)6	12	15282808	15282819
chr1_3045	c	gttgctttctcatggccacagtgggtcccagttgtagtgtgtctgtatgtgtgtg	129	15283089	15283217
chr1_3046	c	(CA)8tacatgcactaatggcgcagaactct(CA)7	56	15283716	15283771
chr1_3047	p2	(TG)16	32	15285369	15285400
chr1_3048	p2	(AC)6	12	15288736	15288747
chr1_3049	p2	(AG)7	14	15289271	15289284
chr1_3050	p2	(GT)14	28	15292399	15292426
chr1_3051	c	agagtgctacgttctcacaatacattggcaaacacatgtgcccatatagacg	118	15295169	15295286
chr1_3052	c	ataaaaaaaatttaaataaaaatcttttcatctgtgcaagcaacctcaagtgtg	132	15297213	15297344
chr1_3053	p3	(CTG)8	24	15298313	15298336
chr1_3054	p3	(GCA)6	18	15299455	15299472
chr1_3055	p2	(CA)6	12	15307231	15307242
chr1_3056	p2	(AC)8	16	15312173	15312188
chr1_3057	p3	(CTG)9	27	15313746	15313772
chr1_3058	p2	(TG)15	30	15316039	15316068
chr1_3059	p2	(CA)23	46	15318189	15318234
chr1_3060	p2	(AC)7	14	15326440	15326453
chr1_3061	p2	(TC)8	16	15327519	15327534
chr1_3062	c	(TG)8cgtgtgtatg(TA)14	54	15336889	15336942
chr1_3063	p2	(TA)6	12	15349669	15349680
chr1_3064	c	gagagagagtgaggagaggaaccgtgtgtgtatgttggaaaaagaaaaata	89	15350389	15350477

chr1_3065	c	agacatctaatcacataccaaaacacaatgacatactctctcactcacgag	104	15350670	15350773
chr1_3066	p2	(TC)7	14	15353197	15353210
chr1_3067	p2	(TG)6	12	15353588	15353599
chr1_3068	p2	(CT)6	12	15356120	15356131
chr1_3069	p2	(CA)8	16	15357574	15357589
chr1_3070	p2	(TG)6	12	15357769	15357780
chr1_3071	p2	(TG)14	28	15360047	15360074
chr1_3072	p2	(AC)6	12	15360851	15360862
chr1_3073	p2	(GT)7	14	15360969	15360982
chr1_3074	c	gggaggacgtctgggtgagattttaattaaagtttgagacaaagaggtcata	140	15361155	15361294
chr1_3075	p2	(TG)21	42	15362057	15362098
chr1_3076	p2	(CA)6	12	15362816	15362827
chr1_3077	p2	(TG)7	14	15366211	15366224
chr1_3078	p2	(TG)8	16	15369073	15369088
chr1_3079	p2	(GT)13	26	15376112	15376137
chr1_3080	p2	(GT)13	26	15376267	15376292
chr1_3081	p3	(TTA)5	15	15381543	15381557
chr1_3082	p2	(CA)8	16	15383081	15383096
chr1_3083	p2	(TG)6	12	15383243	15383254
chr1_3084	p3	(TTA)5	15	15390000	15390014
chr1_3085	p3	(TTA)6	18	15396122	15396139
chr1_3086	p2	(CT)8	16	15398120	15398135
chr1_3087	p2	(GT)6	12	15398425	15398436
chr1_3088	p2	(CA)11	22	15411116	15411137
chr1_3089	p3	(TCC)6	18	15411667	15411684
chr1_3090	p3	(GAG)10	30	15411851	15411880
chr1_3091	p2	(TG)17	34	15427247	15427280
chr1_3092	p2	(CT)7	14	15427431	15427444
chr1_3093	p2	(TG)6	12	15432245	15432256
chr1_3094	c	(GT)10(GA)15	50	15443305	15443354
chr1_3095	p3	(TTC)5	15	15447069	15447083
chr1_3096	p3	(GGT)5	15	15448941	15448955
chr1_3097	c	tggcaggtgctcctcataaagcatcaaataaagcttatcctaaatggcgcca	164	15454144	15454307
chr1_3098	p2	(AC)25	50	15455999	15456048
chr1_3099	p2	(AC)24	48	15457159	15457206
chr1_3100	p2	(TC)7	14	15466679	15466692
chr1_3101	p2	(AC)6	12	15467230	15467241
chr1_3102	p2	(AC)16	32	15469261	15469292
chr1_3103	p2	(CA)6	12	15472091	15472102
chr1_3104	p2	(TG)6	12	15484601	15484612
chr1_3105	p2	(AC)8	16	15487595	15487610
chr1_3106	c	(GA)7gcgtgtgt(GA)8	38	15493094	15493131
chr1_3107	p2	(GA)7	14	15493242	15493255
chr1_3108	p2	(AC)6	12	15504185	15504196
chr1_3109	p2	(AC)10	20	15531713	15531732
chr1_3110	p3	(GCT)5	15	15543222	15543236
chr1_3111	p2	(AC)6	12	15553645	15553656
chr1_3112	p2	(GT)12	24	15560269	15560292
chr1_3113	p2	(AG)7	14	15572571	15572584
chr1_3114	p2	(CT)14	28	15577668	15577695
chr1_3115	p2	(TG)7	14	15585047	15585060
chr1_3116	p2	(CT)6	12	15592294	15592305

chr1_3117	p2	(TC)6	12	15600396	15600407
chr1_3118	p2	(GA)7	14	15607232	15607245
chr1_3119	p3	(TAA)6	18	15635142	15635159
chr1_3120	p2	(CA)15	30	15641366	15641395
chr1_3121	p2	(CA)8	16	15652343	15652358
chr1_3122	p2	(AC)11	22	15654344	15654365
chr1_3123	p2	(CT)6	12	15678391	15678402
chr1_3124	p2	(TG)7	14	15683398	15683411
chr1_3125	p2	(CA)11	22	15686027	15686048
chr1_3126	p2	(AC)6	12	15703794	15703805
chr1_3127	p2	(AC)16	32	15713731	15713762
chr1_3128	p2	(AC)12	24	15715270	15715293
chr1_3129	p2	(CA)8	16	15719474	15719489
chr1_3130	p2	(CA)6	12	15725872	15725883
chr1_3131	c	[illegible]acctcctgttaaaggtggtggcaggggcatacctccagaaaacaaagagg[illegible]	108	15726849	15726956
chr1_3132	p2	(TG)6	12	15737505	15737516
chr1_3133	p2	(TA)6	12	15751355	15751366
chr1_3134	p2	(GA)6	12	15753896	15753907
chr1_3135	p2	(AC)9	18	15754946	15754963
chr1_3136	p3	(TGA)15	45	15765105	15765149
chr1_3137	c	(ATAC)6acacac(AT)11	52	15770617	15770668
chr1_3138	p2	(AG)14	28	15777919	15777946
chr1_3139	p2	(TG)12	24	15790476	15790499
chr1_3140	p2	(GA)15	30	15791162	15791191
chr1_3141	p2	(TG)8	16	15825862	15825877
chr1_3142	p2	(CA)19	38	15830147	15830184
chr1_3143	p2	(AC)7	14	15832295	15832308
chr1_3144	p2	(CA)20	40	15844350	15844389
chr1_3145	p3	(TTA)7	21	15848026	15848046
chr1_3146	p2	(CA)18	36	15868437	15868472
chr1_3147	p2	(AC)13	26	15871527	15871552
chr1_3148	p2	(TG)11	22	15875323	15875344
chr1_3149	p2	(CT)6	12	15892612	15892623
chr1_3150	p2	(CA)9	18	15897344	15897361
chr1_3151	p2	(GA)6	12	15901640	15901651
chr1_3152	p2	(AT)6	12	15908433	15908444
chr1_3153	p2	(CA)8	16	15921004	15921019
chr1_3154	p2	(TA)7	14	15923174	15923187
chr1_3155	c	(TG)6ac(AG)10	34	15925297	15925330
chr1_3156	p3	(CTT)5	15	15928582	15928596
chr1_3157	c	(TA)8(GA)6	28	15931901	15931928
chr1_3158	p2	(GT)6	12	15936175	15936186
chr1_3159	p2	(CA)8	16	15949363	15949378
chr1_3160	p2	(AG)9	18	15975813	15975830
chr1_3161	p3	(AGG)8	24	15976180	15976203
chr1_3162	p2	(CA)7	14	15976815	15976828
chr1_3163	p2	(GT)12	24	15977753	15977776
chr1_3164	p2	(CA)7	14	15981685	15981698
chr1_3165	p2	(AC)8	16	15983580	15983595
chr1_3166	p2	(GT)12	24	15985254	15985277
chr1_3167	p2	(AG)8	16	15988537	15988552
chr1_3168	p2	(TG)16	32	15988987	15989018

chr1_3169	p2	(CA)9	18	16005578	16005595
chr1_3170	p2	(CA)15	30	16010749	16010778
chr1_3171	p2	(CT)6	12	16011778	16011789
chr1_3172	p2	(TG)15	30	16012475	16012504
chr1_3173	p2	(GT)9	18	16014531	16014548
chr1_3174	p2	(AG)9	18	16031789	16031806
chr1_3175	p3	(AAC)6	18	16038610	16038627
chr1_3176	p2	(TG)12	24	16049847	16049870
chr1_3177	p2	(AC)7	14	16050180	16050193
chr1_3178	p2	(GT)12	24	16068208	16068231
chr1_3179	p3	(TGT)5	15	16075985	16075999
chr1_3180	p2	(GT)13	26	16077086	16077111
chr1_3181	p2	(CA)7	14	16079438	16079451
chr1_3182	p2	(TG)11	22	16105531	16105552
chr1_3183	p2	(AC)17	34	16112782	16112815
chr1_3184	p2	(AC)6	12	16117428	16117439
chr1_3185	p2	(TG)15	30	16117547	16117576
chr1_3186	p2	(GA)6	12	16119657	16119668
chr1_3187	p3	(CAT)5	15	16132760	16132774
chr1_3188	p2	(CT)18	36	16137603	16137638
chr1_3189	p2	(GT)14	28	16140314	16140341
chr1_3190	p2	(TG)15	30	16143115	16143144
chr1_3191	p2	(AC)7	14	16143431	16143444
chr1_3192	p2	(AT)9	18	16147657	16147674
chr1_3193	p4	(ATCC)5	20	16149410	16149429
chr1_3194	c	(CT)13tgtgtgtttgcttccttcccc(TG)6	59	16162747	16162805
chr1_3195	p2	(TC)6	12	16167702	16167713
chr1_3196	p3	(TTA)5	15	16170137	16170151
chr1_3197	p4	(TAAA)7	28	16171247	16171274
chr1_3198	p2	(GA)6	12	16177863	16177874
chr1_3199	p2	(AC)6	12	16179070	16179081
chr1_3200	p2	(TG)6	12	16183373	16183384
chr1_3201	p3	(CCA)7	21	16197086	16197106
chr1_3202	p2	(AC)10	20	16207408	16207427
chr1_3203	p2	(CA)10	20	16224888	16224907
chr1_3204	p2	(TA)6	12	16226575	16226586
chr1_3205	c	(CA)7(TA)11caa(AC)6	51	16226974	16227024
chr1_3206	p2	(CT)7	14	16229669	16229682
chr1_3207	p2	(AG)6	12	16232129	16232140
chr1_3208	p2	(CA)10	20	16232411	16232430
chr1_3209	p4	(ATGA)5	20	16233358	16233377
chr1_3210	p2	(GT)6	12	16238121	16238132
chr1_3211	p2	(CA)6	12	16239422	16239433
chr1_3212	p2	(TG)14	28	16248814	16248841
chr1_3213	c	(AG)17aa(AG)7	50	16249980	16250029
chr1_3214	p2	(AC)6	12	16250836	16250847
chr1_3215	p2	(AC)17	34	16251150	16251183
chr1_3216	p2	(TG)12	24	16264890	16264913
chr1_3217	p3	(CAG)5	15	16273668	16273682
chr1_3218	p2	(GT)8	16	16285238	16285253
chr1_3219	p2	(GT)8	16	16288698	16288713
chr1_3220	p5	(GACAA)8	40	16305258	16305297

chr1_3221	p2	(CT)6	12	16320462	16320473
chr1_3222	p2	(GA)6	12	16323948	16323959
chr1_3223	p2	(AC)6	12	16332379	16332390
chr1_3224	p3	(AAT)6	18	16332693	16332710
chr1_3225	c	(AC)8tcc(CA)11	41	16338797	16338837
chr1_3226	p3	(AAC)5	15	16341487	16341501
chr1_3227	p2	(AC)6	12	16353253	16353264
chr1_3228	p2	(CA)9	18	16357996	16358013
chr1_3229	p2	(AC)9	18	16360851	16360868
chr1_3230	p2	(AC)11	22	16361036	16361057
chr1_3231	p2	(TC)8	16	16363457	16363472
chr1_3232	p2	(AC)6	12	16367955	16367966
chr1_3233	p2	(TA)7	14	16376003	16376016
chr1_3234	p2	(CA)22	44	16383697	16383740
chr1_3235	p2	(AC)12	24	16387638	16387661
chr1_3236	p2	(TG)6	12	16397232	16397243
chr1_3237	p2	(CA)6	12	16397855	16397866
chr1_3238	p2	(GT)16	32	16398026	16398057
chr1_3239	p2	(AG)7	14	16399310	16399323
chr1_3240	p3	(TAT)5	15	16400864	16400878
chr1_3241	c	(CT)6g(TC)7(AC)11	49	16404791	16404839
chr1_3242	p3	(CAA)6	18	16405491	16405508
chr1_3243	p2	(GA)14	28	16410018	16410045
chr1_3244	p2	(AC)7	14	16410458	16410471
chr1_3245	p3	(TGA)7	21	16410793	16410813
chr1_3246	p2	(AC)8	16	16417540	16417555
chr1_3247	p4	(ATTC)5	20	16434382	16434401
chr1_3248	p2	(GT)6	12	16458026	16458037
chr1_3249	p2	(TG)7	14	16460790	16460803
chr1_3250	p2	(AC)18	36	16461462	16461497
chr1_3251	p4	(CATC)7	28	16479002	16479029
chr1_3252	p2	(AC)18	36	16486100	16486135
chr1_3253	p2	(CA)18	36	16489060	16489095
chr1_3254	p2	(GA)6	12	16505754	16505765
chr1_3255	p2	(CT)7	14	16508198	16508211
chr1_3256	c	(TG)12ta(TG)7	40	16509392	16509431
chr1_3257	p2	(TA)12	24	16511167	16511190
chr1_3258	p2	(AC)8	16	16511631	16511646
chr1_3259	p2	(TC)12	24	16512899	16512922
chr1_3260	p2	(GT)10	20	16516016	16516035
chr1_3261	p2	(TG)18	36	16518301	16518336
chr1_3262	p2	(AC)7	14	16518653	16518666
chr1_3263	p2	(CT)11	22	16525663	16525684
chr1_3264	p2	(AC)6	12	16528618	16528629
chr1_3265	p2	(CA)9	18	16528757	16528774
chr1_3266	p2	(TG)9	18	16532616	16532633
chr1_3267	c	(AGT)6(AAT)5	33	16540455	16540487
chr1_3268	p2	(TG)8	16	16554261	16554276
chr1_3269	p2	(TG)13	26	16557479	16557504
chr1_3270	p2	(CA)15	30	16558786	16558815
chr1_3271	p2	(TG)6	12	16562127	16562138
chr1_3272	p2	(GT)6	12	16569337	16569348

chr1_3273	p2	(TG)10	20	16570015	16570034
chr1_3274	p3	(CTC)5	15	16575449	16575463
chr1_3275	p4	(ATCC)5	20	16587628	16587647
chr1_3276	c	:caatgtgggttcccagtgccaaacattgtgtctgttggacaggaagaacac	161	16592299	16592459
chr1_3277	p2	(GT)9	18	16597866	16597883
chr1_3278	p2	(TC)9	18	16599446	16599463
chr1_3279	c	GT)8ataagagccttactggagatggagcatgtgtttgttgcagg(GT)1	91	16600136	16600226
chr1_3280	p2	(TA)7	14	16602179	16602192
chr1_3281	p2	(TG)15	30	16602849	16602878
chr1_3282	p2	(CA)7	14	16605666	16605679
chr1_3283	p2	(TG)6	12	16606092	16606103
chr1_3284	p2	(AC)12	24	16608778	16608801
chr1_3285	p2	(TG)10	20	16609194	16609213
chr1_3286	p5	(GGAGA)6	30	16610468	16610497
chr1_3287	c	(GA)7ctgaaaagagaaatc(AG)6	41	16610793	16610833
chr1_3288	p2	(TG)19	38	16611945	16611982
chr1_3289	c	)10cgtgtgtgtattgctccacgctaaacaagtcctcaagcnnnnnnn(T(	85	16612642	16612726
chr1_3290	p2	(TG)10	20	16617346	16617365
chr1_3291	p2	(TG)13	26	16617558	16617583
chr1_3292	c	(CA)6(GA)10	32	16620964	16620995
chr1_3293	p2	(TG)6	12	16621151	16621162
chr1_3294	p2	(GT)19	38	16621298	16621335
chr1_3295	p3	(TTG)9	27	16621565	16621591
chr1_3296	p2	(CA)7	14	16627485	16627498
chr1_3297	p2	(AC)9	18	16631551	16631568
chr1_3298	p2	(GT)8	16	16637397	16637412
chr1_3299	p2	(CA)6	12	16646051	16646062
chr1_3300	c*	(AC)6(ACAT)6*	34	16649416	16649449
chr1_3301	c	(ATT)7actactagtagtggcagtaaaag(TAA)5	59	16651029	16651087
chr1_3302	p2	(AC)9	18	16654854	16654871
chr1_3303	c	(AC)8c(CA)13	43	16660019	16660061
chr1_3304	p2	(CA)13	26	16661660	16661685
chr1_3305	p2	(TA)7	14	16668965	16668978
chr1_3306	p2	(AG)7	14	16676292	16676305
chr1_3307	p2	(AC)12	24	16686417	16686440
chr1_3308	p3	(ATG)5	15	16698942	16698956
chr1_3309	p3	(AAG)5	15	16699420	16699434
chr1_3310	c	;gccaaaccagctgccatcatgtctgagggtggacatgatggaacggactc	94	16725560	16725653
chr1_3311	p2	(TC)6	12	16731330	16731341
chr1_3312	p2	(CA)17	34	16733302	16733335
chr1_3313	p2	(TG)7	14	16733492	16733505
chr1_3314	c	(GT)9(GA)9gtgcgtgtgcatgtgtgggctgctaggtt(TG)8	81	16736205	16736285
chr1_3315	p4	(CATC)5	20	16737414	16737433
chr1_3316	p2	(CA)8	16	16740122	16740137
chr1_3317	p2	(TG)11	22	16740627	16740648
chr1_3318	p2	(CA)19	38	16740937	16740974
chr1_3319	p2	(AG)8	16	16741816	16741831
chr1_3320	p2	(GT)8	16	16742455	16742470
chr1_3321	p2	(CA)9	18	16744751	16744768
chr1_3322	p2	(CA)11	22	16745569	16745590
chr1_3323	p2	(AC)9	18	16748854	16748871
chr1_3324	p2	(GT)7	14	16749315	16749328

chr1_3325	p2	(CA)8	16	16749446	16749461
chr1_3326	p2	(CA)7	14	16750594	16750607
chr1_3327	c	5aactggcactgatcggcagccacaaatacacacatacacagtgtgcgcg	82	16750888	16750969
chr1_3328	c	(AAT)6gatgaca(ATG)5	40	16751389	16751428
chr1_3329	p2	(TG)6	12	16754847	16754858
chr1_3330	p2	(GT)15	30	16757754	16757783
chr1_3331	p2	(CA)7	14	16759715	16759728
chr1_3332	p2	(TG)12	24	16761475	16761498
chr1_3333	p2	(GT)6	12	16762497	16762508
chr1_3334	p2	(TG)6	12	16762648	16762659
chr1_3335	p2	(AC)6	12	16765777	16765788
chr1_3336	p3	(AAC)5	15	16769588	16769602
chr1_3337	p2	(TA)12	24	16770174	16770197
chr1_3338	p2	(CT)6	12	16773985	16773996
chr1_3339	p2	(TG)9	18	16774232	16774249
chr1_3340	p2	(TG)6	12	16774429	16774440
chr1_3341	p2	(TG)10	20	16775441	16775460
chr1_3342	p3	(CTC)8	24	16778926	16778949
chr1_3343	p3	(ATT)5	15	16780220	16780234
chr1_3344	p3	(TTA)6	18	16781534	16781551
chr1_3345	c	gggaggacaaggaggtggtgtttgtttctgtt(TG)10tatgcgtgcgtgtc	237	16782414	16782650
chr1_3346	p2	(GT)6	12	16784583	16784594
chr1_3347	p2	(TG)14	28	16790615	16790642
chr1_3348	p2	(CA)20	40	16791806	16791845
chr1_3349	p2	(TG)12	24	16792876	16792899
chr1_3350	c	(GT)7ttgtttatttgtgtgtgtgcc(TG)6	47	16795509	16795555
chr1_3351	p2	(CA)6	12	16795667	16795678
chr1_3352	p2	(GT)18	36	16795796	16795831
chr1_3353	p2	(GA)6	12	16798779	16798790
chr1_3354	c	gtgcctgattaaagttggttggaaagaaatgtgcgtgtgccacaactgggtg	102	16799164	16799265
chr1_3355	p2	(CA)6	12	16800277	16800288
chr1_3356	c	(AC)8(TC)34	84	16803350	16803433
chr1_3357	p2	(AC)10	20	16803700	16803719
chr1_3358	p2	(TG)11	22	16804074	16804095
chr1_3359	c	:atg(AC)8gcacacacggaacccattacaaacctgccacagtcacagc	186	16804980	16805165
chr1_3360	p2	(CA)7	14	16805952	16805965
chr1_3361	p2	(TC)6	12	16807259	16807270
chr1_3362	p2	(GT)7	14	16809835	16809848
chr1_3363	p2	(AC)10	20	16810232	16810251
chr1_3364	p2	(TG)14	28	16810396	16810423
chr1_3365	p2	(TG)14	28	16810543	16810570
chr1_3366	p3	(TAT)5	15	16811122	16811136
chr1_3367	p2	(TC)6	12	16813730	16813741
chr1_3368	c	(AGA)5aaaa(AAG)6(AAC)8	61	16816478	16816538
chr1_3369	p2	(GA)8	16	16818827	16818842
chr1_3370	p2	(TG)24	48	16822327	16822374
chr1_3371	p2	(CA)6	12	16822515	16822526
chr1_3372	c	(CA)10cg(CA)8	38	16828237	16828274
chr1_3373	p2	(GT)12	24	16830399	16830422
chr1_3374	c	(GT)9gc(GT)6	32	16831261	16831292
chr1_3375	p2	(AC)10	20	16834203	16834222
chr1_3376	p2	(AC)15	30	16836335	16836364

chr1_3377	p2	(CA)10	20	16837673	16837692
chr1_3378	p2	(AT)9	18	16840277	16840294
chr1_3379	p2	(AT)9	18	16841640	16841657
chr1_3380	p2	(GT)10	20	16843805	16843824
chr1_3381	p2	(AT)7	14	16844936	16844949
chr1_3382	p2	(TG)6	12	16845876	16845887
chr1_3383	p2	(AC)6	12	16851187	16851198
chr1_3384	p2	(AC)6	12	16851898	16851909
chr1_3385	p2	(GT)6	12	16853691	16853702
chr1_3386	p2	(AC)17	34	16855633	16855666
chr1_3387	p2	(TG)8	16	16856359	16856374
chr1_3388	p2	(AC)17	34	16861431	16861464
chr1_3389	p2	(AC)17	34	16870291	16870324
chr1_3390	p4	(TAGT)5	20	16870428	16870447
chr1_3391	p2	(TG)9	18	16874779	16874796
chr1_3392	p2	(TG)24	48	16876521	16876568
chr1_3393	p3	(AGG)5	15	16878312	16878326
chr1_3394	p4	(TCTA)14	56	16882738	16882793
chr1_3395	p2	(TG)6	12	16889036	16889047
chr1_3396	p2	(TC)6	12	16890535	16890546
chr1_3397	p2	(TA)8	16	16895978	16895993
chr1_3398	p2	(TG)12	24	16924759	16924782
chr1_3399	p2	(AC)13	26	16924917	16924942
chr1_3400	p2	(AC)19	38	16927305	16927342
chr1_3401	p2	(AG)15	30	16932257	16932286
chr1_3402	p2	(GT)7	14	16934083	16934096
chr1_3403	p3	(TGT)6	18	16936705	16936722
chr1_3404	p5	(TCTCT)5	25	16936983	16937007
chr1_3405	p2	(TG)22	44	16937153	16937196
chr1_3406	p2	(TC)7	14	16937433	16937446
chr1_3407	c*	(AGTG)5(GT)13*	43	16938292	16938334
chr1_3408	p2	(AC)24	48	16943716	16943763
chr1_3409	p2	(CA)6	12	16956204	16956215
chr1_3410	p2	(GT)6	12	16972685	16972696
chr1_3411	c	(TG)6(AG)7	26	16977136	16977161
chr1_3412	p2	(CA)11	22	16982827	16982848
chr1_3413	p2	(GT)8	16	16996730	16996745
chr1_3414	p3	(GCA)5	15	17001237	17001251
chr1_3415	p2	(GT)6	12	17006656	17006667
chr1_3416	p2	(TG)27	54	17010102	17010155
chr1_3417	p2	(GT)17	34	17021270	17021303
chr1_3418	p2	(TG)8	16	17025779	17025794
chr1_3419	p2	(CA)7	14	17054355	17054368
chr1_3420	p2	(GT)6	12	17062133	17062144
chr1_3421	p2	(TG)14	28	17065344	17065371
chr1_3422	p2	(AC)9	18	17081279	17081296
chr1_3423	p2	(GT)10	20	17082202	17082221
chr1_3424	p2	(GT)6	12	17085423	17085434
chr1_3425	c	acttgggtgtttctatcagaacacaactacatccagcatttgagcttgtacac	132	17086641	17086772
chr1_3426	p2	(GT)29	58	17086904	17086961
chr1_3427	p2	(TG)10	20	17089090	17089109
chr1_3428	p2	(CA)16	32	17094648	17094679

chr1_3429	p2	(GT)6	12	17098119	17098130
chr1_3430	p2	(GT)17	34	17098367	17098400
chr1_3431	p2	(CA)6	12	17098683	17098694
chr1_3432	p2	(GA)6	12	17099393	17099404
chr1_3433	p4	(ATGG)5	20	17105582	17105601
chr1_3434	c	ccaaccgtcatgcccagcaaatgccttttgcttgaattcacatgcaaatttgc	98	17105777	17105874
chr1_3435	p2	(AC)6	12	17107750	17107761
chr1_3436	p2	(AC)14	28	17109000	17109027
chr1_3437	p2	(AC)9	18	17109215	17109232
chr1_3438	p2	(CA)7	14	17112676	17112689
chr1_3439	p2	(GA)15	30	17113262	17113291
chr1_3440	p2	(CA)7	14	17114762	17114775
chr1_3441	p2	(AC)8	16	17114920	17114935
chr1_3442	p2	(AC)6	12	17116634	17116645
chr1_3443	p2	(AC)16	32	17117162	17117193
chr1_3444	p2	(TG)19	38	17117456	17117493
chr1_3445	p3	(TTC)5	15	17118504	17118518
chr1_3446	p2	(CA)6	12	17120822	17120833
chr1_3447	p2	(TG)18	36	17122061	17122096
chr1_3448	p2	(GT)7	14	17133086	17133099
chr1_3449	p2	(AC)13	26	17133385	17133410
chr1_3450	p2	(TG)14	28	17135777	17135804
chr1_3451	p5	(TTTGA)5	25	17136076	17136100
chr1_3452	p2	(AC)16	32	17139851	17139882
chr1_3453	p2	(TG)7	14	17145619	17145632
chr1_3454	p2	(GT)8	16	17145751	17145766
chr1_3455	p2	(TG)6	12	17147332	17147343
chr1_3456	p2	(TG)11	22	17149074	17149095
chr1_3457	p2	(TG)8	16	17157801	17157816
chr1_3458	p2	(TC)6	12	17158545	17158556
chr1_3459	p2	(GT)6	12	17162518	17162529
chr1_3460	p2	(GT)17	34	17163535	17163568
chr1_3461	p2	(CT)6	12	17165133	17165144
chr1_3462	p2	(GT)10	20	17165311	17165330
chr1_3463	c	(TC)6actcactcactcactctctctcacacact(CA)9	59	17168100	17168158
chr1_3464	p2	(AC)11	22	17175988	17176009
chr1_3465	p2	(GT)15	30	17187154	17187183
chr1_3466	p2	(GT)20	40	17189564	17189603
chr1_3467	p2	(AC)22	44	17190216	17190259
chr1_3468	p2	(GT)8	16	17191850	17191865
chr1_3469	c	(CA)8aacacatat(AC)7	39	17192715	17192753
chr1_3470	p2	(GT)6	12	17195580	17195591
chr1_3471	p3	(ATT)11	33	17196575	17196607
chr1_3472	c	(TG)6(TC)8tgtct(CA)10	53	17196711	17196763
chr1_3473	p2	(CA)6	12	17196866	17196877
chr1_3474	c	aggggattacaggaagactggtgtgcagacagacccctgcaaactcactg	148	17197877	17198024
chr1_3475	p2	(GT)9	18	17199878	17199895
chr1_3476	p2	(TG)18	36	17200325	17200360
chr1_3477	c	(TG)9(TC)13	44	17200825	17200868
chr1_3478	p2	(CA)6	12	17202696	17202707
chr1_3479	c	(AC)6agag(AC)7atatatatatagacacac(AT)9	66	17202846	17202911
chr1_3480	c	gagaaggggactgcctacagatttgcctgcatcaaatccaggcgcctctgg	120	17205029	17205148

chr1_3481	p2	(TG)10	20	17205313	17205332
chr1_3482	p2	(AC)6	12	17205965	17205976
chr1_3483	p2	(TG)14	28	17208210	17208237
chr1_3484	p2	(AC)8	16	17209691	17209706
chr1_3485	c	(TG)8(AG)8	32	17209884	17209915
chr1_3486	p2	(CA)24	48	17210775	17210822
chr1_3487	p2	(GT)23	46	17211970	17212015
chr1_3488	p2	(TG)18	36	17212475	17212510
chr1_3489	p2	(TG)10	20	17215486	17215505
chr1_3490	p3	(ACA)6	18	17215640	17215657
chr1_3491	p2	(AC)6	12	17215794	17215805
chr1_3492	p2	(GT)12	24	17216015	17216038
chr1_3493	p2	(AG)7	14	17216554	17216567
chr1_3494	p2	(TC)15	30	17218014	17218043
chr1_3495	p2	(CA)12	24	17218841	17218864
chr1_3496	p2	(GT)6	12	17220143	17220154
chr1_3497	p2	(TG)11	22	17221895	17221916
chr1_3498	p2	(TG)20	40	17222794	17222833
chr1_3499	p2	(AC)8	16	17224528	17224543
chr1_3500	p2	(GT)6	12	17226165	17226176
chr1_3501	p2	(CA)18	36	17226322	17226357
chr1_3502	p2	(CT)8	16	17226504	17226519
chr1_3503	p2	(AC)6	12	17227582	17227593
chr1_3504	c	gagagagggcggacaggcaaaaagaaagaatggggagagcttatccctc	151	17228141	17228291
chr1_3505	p2	(TG)8	16	17228467	17228482
chr1_3506	p4	(CATC)6	24	17228584	17228607
chr1_3507	p2	(AC)6	12	17229110	17229121
chr1_3508	p2	(TG)9	18	17235601	17235618
chr1_3509	p2	(TG)7	14	17236483	17236496
chr1_3510	p2	(GT)7	14	17237495	17237508
chr1_3511	p2	(GT)15	30	17238821	17238850
chr1_3512	p4	(GTTA)5	20	17239566	17239585
chr1_3513	c	caaggaccacatacatatcacacatgcatgtacgcacacaaaaacacccac	94	17240114	17240207
chr1_3514	p2	(GT)18	36	17242395	17242430
chr1_3515	p2	(GT)9	18	17244066	17244083
chr1_3516	p2	(CA)16	32	17244382	17244413
chr1_3517	p2	(CA)14	28	17245581	17245608
chr1_3518	p2	(GT)15	30	17245822	17245851
chr1_3519	p2	(CA)12	24	17247996	17248019
chr1_3520	p2	(TG)10	20	17248589	17248608
chr1_3521	p2	(AC)6	12	17248894	17248905
chr1_3522	p2	(TG)24	48	17250267	17250314
chr1_3523	p2	(AC)10	20	17250752	17250771
chr1_3524	p2	(GT)11	22	17251497	17251518
chr1_3525	p4	(ATGG)5	20	17252664	17252683
chr1_3526	p2	(AC)10	20	17255693	17255712
chr1_3527	p2	(CA)6	12	17255826	17255837
chr1_3528	p2	(TG)10	20	17257352	17257371
chr1_3529	c	(TG)21(AG)12	66	17258932	17258997
chr1_3530	p2	(CA)6	12	17259718	17259729
chr1_3531	c	tggattgaaatttgtcattgtggatatagaaacaggtgttactttgtttagaatg	141	17260711	17260851
chr1_3532	p2	(GT)13	26	17261665	17261690

chr1_3533	p2	(CT)7	14	17265175	17265188
chr1_3534	p2	(CA)12	24	17266080	17266103
chr1_3535	p2	(TG)6	12	17266226	17266237
chr1_3536	p2	(AC)10	20	17291464	17291483
chr1_3537	p2	(CA)6	12	17296256	17296267
chr1_3538	p2	(TG)6	12	17296527	17296538
chr1_3539	p2	(GT)18	36	17296769	17296804
chr1_3540	p2	(AC)14	28	17300069	17300096
chr1_3541	c	tgaggccctttgtggaaagaaatcatgtcgaaaaatagagctggggtaag[illegible]	125	17305640	17305764
chr1_3542	p2	(TG)8	16	17311839	17311854
chr1_3543	p2	(CT)10	20	17315333	17315352
chr1_3544	p2	(AC)12	24	17316737	17316760
chr1_3545	p2	(TG)6	12	17319764	17319775
chr1_3546	p2	(TG)8	16	17319980	17319995
chr1_3547	p2	(AC)6	12	17320100	17320111
chr1_3548	p3	(CTT)5	15	17336315	17336329
chr1_3549	p2	(AC)16	32	17337709	17337740
chr1_3550	p2	(GT)8	16	17337866	17337881
chr1_3551	p2	(AG)6	12	17352477	17352488
chr1_3552	p3	(ATA)5	15	17353662	17353676
chr1_3553	p3	(AGC)5	15	17356684	17356698
chr1_3554	p2	(TG)14	28	17357726	17357753
chr1_3555	p2	(AC)6	12	17357865	17357876
chr1_3556	p2	(TC)7	14	17358122	17358135
chr1_3557	p2	(TG)7	14	17359638	17359651
chr1_3558	p2	(CA)7	14	17359875	17359888
chr1_3559	p2	(AC)10	20	17361119	17361138
chr1_3560	p2	(CA)8	16	17361275	17361290
chr1_3561	c	(CA)6ggaaatcaaaaagctctcagcacgcacgtat(AC)8	59	17369467	17369525
chr1_3562	p2	(GT)6	12	17373929	17373940
chr1_3563	p3	(AAT)7	21	17374479	17374499
chr1_3564	p2	(AC)8	16	17376290	17376305
chr1_3565	p2	(AG)6	12	17379923	17379934
chr1_3566	p2	(TC)7	14	17381515	17381528
chr1_3567	p3	(TGA)6	18	17387097	17387114
chr1_3568	p2	(CA)7	14	17391307	17391320
chr1_3569	p2	(TG)14	28	17391509	17391536
chr1_3570	p3	(GAA)5	15	17396796	17396810
chr1_3571	p2	(AC)6	12	17400747	17400758
chr1_3572	p3	(CCT)10	30	17401687	17401716
chr1_3573	p2	(AG)6	12	17405381	17405392
chr1_3574	p2	(CA)9	18	17408696	17408713
chr1_3575	p2	(TG)11	22	17409252	17409273
chr1_3576	p2	(CA)6	12	17412276	17412287
chr1_3577	p3	(AAT)5	15	17412887	17412901
chr1_3578	p2	(TG)8	16	17419070	17419085
chr1_3579	p2	(AC)7	14	17421240	17421253
chr1_3580	p2	(TG)6	12	17426467	17426478
chr1_3581	p2	(AC)37	74	17426664	17426737
chr1_3582	p2	(AC)7	14	17427013	17427026
chr1_3583	p2	(GT)13	26	17427171	17427196
chr1_3584	p2	(TG)16	32	17429901	17429932

chr1_3585	p2	(TG)6	12	17430372	17430383
chr1_3586	p2	(TG)10	20	17433487	17433506
chr1_3587	p2	(TG)7	14	17434052	17434065
chr1_3588	p4	(AAAT)6	24	17435194	17435217
chr1_3589	p2	(GT)9	18	17437665	17437682
chr1_3590	p2	(TC)14	28	17443942	17443969
chr1_3591	p2	(GT)10	20	17446560	17446579
chr1_3592	p2	(TG)15	30	17447230	17447259
chr1_3593	p2	(GT)8	16	17449593	17449608
chr1_3594	p2	(TG)10	20	17452013	17452032
chr1_3595	p2	(AG)6	12	17452226	17452237
chr1_3596	c	tcagcctaagtttctagccatgtgtctcttggctcgacattaaaagacatgac	118	17454952	17455069
chr1_3597	p2	(CA)11	22	17456745	17456766
chr1_3598	p2	(CT)7	14	17458066	17458079
chr1_3599	p2	(TC)6	12	17458536	17458547
chr1_3600	p2	(AG)17	34	17460435	17460468
chr1_3601	p2	(AC)7	14	17464195	17464208
chr1_3602	p2	(GT)18	36	17473239	17473274
chr1_3603	p2	(GA)6	12	17476073	17476084
chr1_3604	p2	(AC)25	50	17476472	17476521
chr1_3605	p3	(CAG)5	15	17477682	17477696
chr1_3606	p3	(TTA)6	18	17478227	17478244
chr1_3607	c	(CA)10ccaacaagacaattcctgaaca(TC)13	68	17479005	17479072
chr1_3608	p2	(GT)6	12	17484054	17484065
chr1_3609	p2	(GT)6	12	17486795	17486806
chr1_3610	p2	(TG)7	14	17488744	17488757
chr1_3611	p2	(GT)6	12	17490320	17490331
chr1_3612	p2	(TG)6	12	17496036	17496047
chr1_3613	p2	(TG)6	12	17496154	17496165
chr1_3614	p2	(TG)7	14	17496532	17496545
chr1_3615	p2	(CA)14	28	17498503	17498530
chr1_3616	p3	(AAG)6	18	17506445	17506462
chr1_3617	p2	(CA)16	32	17506821	17506852
chr1_3618	p2	(GA)6	12	17508381	17508392
chr1_3619	p2	(AC)10	20	17508818	17508837
chr1_3620	p2	(CT)8	16	17511683	17511698
chr1_3621	p2	(CA)8	16	17514359	17514374
chr1_3622	p2	(CA)15	30	17518338	17518367
chr1_3623	p2	(AC)16	32	17518503	17518534
chr1_3624	p2	(AC)9	18	17520416	17520433
chr1_3625	p2	(CA)13	26	17532605	17532630
chr1_3626	p2	(CA)14	28	17548933	17548960
chr1_3627	p2	(AT)9	18	17551889	17551906
chr1_3628	c	(TG)6ta(TG)6	26	17552173	17552198
chr1_3629	p2	(TG)6	12	17559741	17559752
chr1_3630	p2	(GA)6	12	17560121	17560132
chr1_3631	c	(CA)7cc(CA)7	30	17562629	17562658
chr1_3632	p2	(AC)17	34	17565229	17565262
chr1_3633	p2	(GT)15	30	17582945	17582974
chr1_3634	p2	(GT)19	38	17601116	17601153
chr1_3635	p3	(TTC)5	15	17609680	17609694
chr1_3636	p4	(ATCC)15	60	17612136	17612195

chr1_3637	p5	(TGTGA)30	150	17612891	17613040
chr1_3638	p2	(TG)19	38	17615106	17615143
chr1_3639	p2	(AT)6	12	17617966	17617977
chr1_3640	p2	(GA)10	20	17634402	17634421
chr1_3641	p2	(AC)6	12	17657944	17657955
chr1_3642	p3	(AGA)5	15	17658710	17658724
chr1_3643	p4	(AAAC)5	20	17663563	17663582
chr1_3644	p2	(CA)28	56	17665149	17665204
chr1_3645	p2	(TG)6	12	17678873	17678884
chr1_3646	p3	(TCA)5	15	17686978	17686992
chr1_3647	p3	(CTT)6	18	17687901	17687918
chr1_3648	p2	(GT)11	22	17730920	17730941
chr1_3649	p2	(TC)8	16	17782020	17782035
chr1_3650	p2	(CA)16	32	17782584	17782615
chr1_3651	p4	(CTAC)5	20	17787274	17787293
chr1_3652	p2	(CA)10	20	17793443	17793462
chr1_3653	p2	(CT)14	28	17794144	17794171
chr1_3654	p3	(AAT)8	24	17796935	17796958
chr1_3655	p3	(ATA)8	24	17799034	17799057
chr1_3656	p4	(ATAG)14	56	17808223	17808278
chr1_3657	p2	(AC)14	28	17809319	17809346
chr1_3658	p5	(AAATT)6	30	17816632	17816661
chr1_3659	p2	(AC)24	48	17820175	17820222
chr1_3660	p3	(TAT)9	27	17822256	17822282
chr1_3661	p2	(TG)16	32	17822533	17822564
chr1_3662	p2	(AC)7	14	17833477	17833490
chr1_3663	p2	(TG)7	14	17876075	17876088
chr1_3664	p2	(TG)6	12	17879038	17879049
chr1_3665	p2	(GT)8	16	17883879	17883894
chr1_3666	p3	(TTA)5	15	17884135	17884149
chr1_3667	p2	(TG)8	16	17886071	17886086
chr1_3668	p2	(TG)6	12	17887229	17887240
chr1_3669	p2	(TG)6	12	17892301	17892312
chr1_3670	p2	(AC)6	12	17893353	17893364
chr1_3671	p5	(AATCA)5	25	17898907	17898931
chr1_3672	p2	(CA)8	16	17911378	17911393
chr1_3673	p2	(TG)8	16	17914959	17914974
chr1_3674	c	)6acgatacaccattagtcattgccgcacattcatgacagatactgtacac([illegible]	75	17921145	17921219
chr1_3675	p2	(TG)7	14	17922422	17922435
chr1_3676	p2	(TG)6	12	17926293	17926304
chr1_3677	p2	(CA)25	50	17938420	17938469
chr1_3678	p2	(AC)6	12	17939965	17939976
chr1_3679	c	(GA)8gc(GA)19	56	17947078	17947133
chr1_3680	p2	(CA)19	38	17949107	17949144
chr1_3681	p3	(TCA)5	15	17957343	17957357
chr1_3682	p2	(CA)10	20	17957855	17957874
chr1_3683	p2	(AC)12	24	17958905	17958928
chr1_3684	p2	(CA)18	36	17962810	17962845
chr1_3685	p2	(TA)7	14	17965193	17965206
chr1_3686	p2	(GT)10	20	17967487	17967506
chr1_3687	p2	(GT)8	16	17969239	17969254
chr1_3688	p3	(CTG)5	15	17969614	17969628

chr1_3689	p2	(TG)13	26	17978555	17978580
chr1_3690	p2	(CA)16	32	17986332	17986363
chr1_3691	p2	(TG)6	12	18023042	18023053
chr1_3692	c	(TATG)6tgtatatgtg(TA)6	46	18024483	18024528
chr1_3693	c	(CA)6cgcacacgcacacg(CA)21	68	18028766	18028833
chr1_3694	c	(TG)27g(AT)10	75	18043518	18043592
chr1_3695	p2	(TG)11	22	18046538	18046559
chr1_3696	p2	(AT)7	14	18064235	18064248
chr1_3697	p2	(CA)6	12	18073402	18073413
chr1_3698	p2	(TG)7	14	18074907	18074920
chr1_3699	p2	(AC)28	56	18076993	18077048
chr1_3700	p2	(CA)8	16	18081740	18081755
chr1_3701	p2	(AG)6	12	18081930	18081941
chr1_3702	p2	(AC)6	12	18085838	18085849
chr1_3703	c	(CT)9cat(AC)8	37	18086241	18086277
chr1_3704	p2	(AG)7	14	18089708	18089721
chr1_3705	p2	(TG)19	38	18089876	18089913
chr1_3706	p2	(CA)14	28	18090302	18090329
chr1_3707	p2	(AC)11	22	18090846	18090867
chr1_3708	p2	(AC)16	32	18091176	18091207
chr1_3709	p3	(ACA)5	15	18097033	18097047
chr1_3710	p2	(CT)10	20	18103205	18103224
chr1_3711	c	(CA)8cttacaaacacacacagac(AG)11	57	18104980	18105036
chr1_3712	c	gaagttattgtacagcccttgttcttcacataggatatttaggatggcaatatg	150	18105218	18105367
chr1_3713	p2	(TG)8	16	18106168	18106183
chr1_3714	p2	(TG)10	20	18108302	18108321
chr1_3715	p2	(CA)6	12	18108475	18108486
chr1_3716	c	aactagtggcaagcgaggacgaaatagatgggcg(GA)9cccgcacc	194	18113286	18113479
chr1_3717	p2	(GT)7	14	18117744	18117757
chr1_3718	p2	(AC)9	18	18121992	18122009
chr1_3719	p2	(AG)6	12	18124239	18124250
chr1_3720	p2	(AC)9	18	18126030	18126047
chr1_3721	p2	(TG)14	28	18141084	18141111
chr1_3722	p2	(TG)11	22	18143588	18143609
chr1_3723	p2	(GT)11	22	18144069	18144090
chr1_3724	p2	(AC)6	12	18154105	18154116
chr1_3725	p2	(CA)8	16	18160091	18160106
chr1_3726	p2	(TG)6	12	18162951	18162962
chr1_3727	p2	(CA)13	26	18166742	18166767
chr1_3728	p2	(TG)6	12	18167874	18167885
chr1_3729	p2	(TG)6	12	18170700	18170711
chr1_3730	p2	(TA)6	12	18173602	18173613
chr1_3731	p2	(GT)9	18	18175043	18175060
chr1_3732	c	(TC)13(AC)14	54	18176591	18176644
chr1_3733	p2	(AC)7	14	18179024	18179037
chr1_3734	p2	(AC)10	20	18189556	18189575
chr1_3735	p2	(CA)9	18	18190845	18190862
chr1_3736	p2	(CT)8	16	18204149	18204164
chr1_3737	c	(AC)7atacat(AC)11	42	18205664	18205705
chr1_3738	p2	(AC)8	16	18209938	18209953
chr1_3739	p2	(AC)6	12	18217771	18217782
chr1_3740	c	gatgtnnnnnnnnnnnnnnnnnnnnnnnnnnnnnnnnnnnnnnn	185	18219774	18219958

chr1_3741	p2	(TC)8	16	18236169	18236184
chr1_3742	p2	(CA)14	28	18248105	18248132
chr1_3743	p2	(AC)18	36	18269208	18269243
chr1_3744	p2	(CT)10	20	18282895	18282914
chr1_3745	p2	(GA)6	12	18285570	18285581
chr1_3746	p2	(GT)37	74	18288066	18288139
chr1_3747	p2	(TA)6	12	18301391	18301402
chr1_3748	p2	(TG)6	12	18305910	18305921
chr1_3749	p3	(TTG)6	18	18309747	18309764
chr1_3750	p2	(TA)6	12	18316361	18316372
chr1_3751	p3	(GAG)5	15	18323208	18323222
chr1_3752	p2	(GA)7	14	18324049	18324062
chr1_3753	p2	(GT)8	16	18328426	18328441
chr1_3754	p2	(TA)7	14	18349022	18349035
chr1_3755	p2	(TG)12	24	18372323	18372346
chr1_3756	p2	(AT)13	26	18391770	18391795
chr1_3757	p3	(TTC)5	15	18397017	18397031
chr1_3758	c	(TA)12(CA)6	36	18415403	18415438
chr1_3759	p2	(AC)7	14	18424145	18424158
chr1_3760	p2	(CA)6	12	18429447	18429458
chr1_3761	p2	(CA)6	12	18430455	18430466
chr1_3762	p2	(CA)14	28	18430670	18430697
chr1_3763	p2	(TG)45	90	18433570	18433659
chr1_3764	p2	(TG)6	12	18439496	18439507
chr1_3765	p2	(GT)17	34	18467521	18467554
chr1_3766	p2	(CT)11	22	18472747	18472768
chr1_3767	p3	(AAT)5	15	18477388	18477402
chr1_3768	p3	(TTG)6	18	18481673	18481690
chr1_3769	c	(TC)6(AC)19	50	18488461	18488510
chr1_3770	p2	(AC)10	20	18503738	18503757
chr1_3771	p3	(CTG)7	21	18507611	18507631
chr1_3772	p3	(TGA)5	15	18507994	18508008
chr1_3773	p2	(AC)13	26	18523735	18523760
chr1_3774	p2	(GT)15	30	18535289	18535318
chr1_3775	p2	(TG)8	16	18538038	18538053
chr1_3776	p2	(CA)15	30	18540587	18540616
chr1_3777	p2	(CA)45	90	18544188	18544277
chr1_3778	p3	(AAC)5	15	18566467	18566481
chr1_3779	p4	(AAAT)6	24	18588086	18588109
chr1_3780	p2	(AC)13	26	18592161	18592186
chr1_3781	p2	(GT)6	12	18593525	18593536
chr1_3782	p2	(TA)6	12	18607243	18607254
chr1_3783	p2	(TC)6	12	18610785	18610796
chr1_3784	p2	(GA)8	16	18615107	18615122
chr1_3785	p2	(GT)10	20	18625613	18625632
chr1_3786	p2	(AC)6	12	18626621	18626632
chr1_3787	c	(AC)7(AT)17	48	18630983	18631030
chr1_3788	p2	(GT)19	38	18663368	18663405
chr1_3789	p2	(GT)19	38	18664275	18664312
chr1_3790	p2	(GT)6	12	18665363	18665374
chr1_3791	p2	(CT)11	22	18667808	18667829
chr1_3792	p2	(AC)16	32	18672461	18672492

chr1_3793	p2	(GA)6	12	18676628	18676639
chr1_3794	p2	(TC)11	22	18684629	18684650
chr1_3795	p2	(AC)11	22	18690527	18690548
chr1_3796	p2	(AC)11	22	18695737	18695758
chr1_3797	p2	(GA)6	12	18702598	18702609
chr1_3798	c	(CA)7(GA)18a(AG)10	71	18725944	18726014
chr1_3799	p2	(CA)18	36	18771055	18771090
chr1_3800	p2	(AC)7	14	18796981	18796994
chr1_3801	p3	(CAG)5	15	18803377	18803391
chr1_3802	p3	(TTA)5	15	18814095	18814109
chr1_3803	p2	(AT)6	12	18815346	18815357
chr1_3804	p3	(AGA)5	15	18815632	18815646
chr1_3805	p2	(TA)6	12	18833260	18833271
chr1_3806	p2	(AT)7	14	18835052	18835065
chr1_3807	c	(CA)17cctatatatacagtatatatatgtat(AC)14	88	18840136	18840223
chr1_3808	p2	(GT)6	12	18844212	18844223
chr1_3809	p2	(TG)6	12	18849240	18849251
chr1_3810	p2	(GT)7	14	18859601	18859614
chr1_3811	p2	(CA)17	34	18871267	18871300
chr1_3812	p2	(TG)12	24	18877867	18877890
chr1_3813	p2	(AC)13	26	18900887	18900912
chr1_3814	p2	(TG)10	20	18902856	18902875
chr1_3815	p4	(GAGT)6	24	18912447	18912470
chr1_3816	p2	(TC)7	14	18924973	18924986
chr1_3817	p2	(AC)28	56	18930542	18930597
chr1_3818	p2	(GA)8	16	18933552	18933567
chr1_3819	p2	(GT)13	26	18943340	18943365
chr1_3820	p3	(TGT)5	15	18943498	18943512
chr1_3821	p3	(TCT)5	15	18945804	18945818
chr1_3822	c	(GT)13(TGC)5	41	18950741	18950781
chr1_3823	p2	(AT)6	12	18951173	18951184
chr1_3824	p2	(GT)9	18	18959268	18959285
chr1_3825	p2	(CT)8	16	18966054	18966069
chr1_3826	p2	(TG)10	20	18972630	18972649
chr1_3827	p2	(TG)11	22	18978162	18978183
chr1_3828	p2	(GA)6	12	18995057	18995068
chr1_3829	p2	(GT)6	12	19011583	19011594
chr1_3830	p2	(TG)8	16	19019532	19019547
chr1_3831	p2	(AC)27	54	19025540	19025593
chr1_3832	c	(CA)6ag(CA)6	26	19031108	19031133
chr1_3833	p2	(AT)7	14	19042314	19042327
chr1_3834	p3	(CCA)5	15	19053118	19053132
chr1_3835	p2	(CA)10	20	19067610	19067629
chr1_3836	p2	(CT)8	16	19070501	19070516
chr1_3837	p2	(AG)23	46	19085048	19085093
chr1_3838	p2	(CA)7	14	19099035	19099048
chr1_3839	p2	(CA)23	46	19099226	19099271
chr1_3840	p2	(TC)6	12	19106468	19106479
chr1_3841	p2	(TG)6	12	19118071	19118082
chr1_3842	p2	(TA)6	12	19122267	19122278
chr1_3843	p4	(TACC)6	24	19123116	19123139
chr1_3844	c	(TATC)9(TGTC)8	68	19137660	19137727

chr1_3845	p2	(CA)6	12	19138916	19138927
chr1_3846	p2	(AT)6	12	19139054	19139065
chr1_3847	p2	(TC)7	14	19139718	19139731
chr1_3848	p2	(TG)12	24	19151040	19151063
chr1_3849	p4	(AATA)5	20	19174562	19174581
chr1_3850	c	(GA)15aa(GT)9	50	19179755	19179804
chr1_3851	p2	(CA)6	12	19182228	19182239
chr1_3852	p2	(TG)7	14	19183960	19183973
chr1_3853	p2	(CA)6	12	19184479	19184490
chr1_3854	p2	(AC)7	14	19218459	19218472
chr1_3855	p3	(ATC)6	18	19230895	19230912
chr1_3856	p5	(CATAT)6	30	19235182	19235211
chr1_3857	p2	(CT)7	14	19263905	19263918
chr1_3858	p2	(TG)6	12	19275185	19275196
chr1_3859	p2	(AT)6	12	19279882	19279893
chr1_3860	p2	(TG)6	12	19288608	19288619
chr1_3861	p2	(CA)14	28	19292429	19292456
chr1_3862	p2	(CA)9	18	19303737	19303754
chr1_3863	p2	(TC)8	16	19308166	19308181
chr1_3864	p4	(AGAT)14	56	19310354	19310409
chr1_3865	p2	(CT)9	18	19310948	19310965
chr1_3866	p2	(GA)13	26	19313290	19313315
chr1_3867	p2	(CA)17	34	19314440	19314473
chr1_3868	p3	(TTG)6	18	19317587	19317604
chr1_3869	p2	(AT)6	12	19318040	19318051
chr1_3870	p2	(CT)6	12	19320613	19320624
chr1_3871	p2	(GA)7	14	19323799	19323812
chr1_3872	p2	(CA)14	28	19326174	19326201
chr1_3873	p2	(TG)16	32	19332277	19332308
chr1_3874	c	(CA)13cc(CA)8	44	19337653	19337696
chr1_3875	p2	(AC)12	24	19341655	19341678
chr1_3876	p2	(CA)8	16	19342052	19342067
chr1_3877	p2	(AG)6	12	19355068	19355079
chr1_3878	p2	(TG)6	12	19358055	19358066
chr1_3879	p2	(GT)6	12	19359669	19359680
chr1_3880	p4	(TTCA)5	20	19369660	19369679
chr1_3881	p2	(AC)6	12	19371136	19371147
chr1_3882	p2	(CA)6	12	19377158	19377169
chr1_3883	c	[illegible]caagtgagtttaaggcctttttggtatatatagcttactgacacaggtgcattc[illegible]	122	19377353	19377474
chr1_3884	p2	(TG)6	12	19408399	19408410
chr1_3885	p3	(ATT)5	15	19410031	19410045
chr1_3886	p2	(TG)15	30	19431824	19431853
chr1_3887	c	(ATA)5tcc(ATCT)5	38	19436157	19436194
chr1_3888	c	(TA)6ttcatcacttggtttgtccct(AC)9	51	19452425	19452475
chr1_3889	p2	(GT)10	20	19471186	19471205
chr1_3890	p3	(TCC)5	15	19478982	19478996
chr1_3891	p4	(AATA)5	20	19485556	19485575
chr1_3892	p2	(AC)12	24	19509874	19509897
chr1_3893	p2	(TG)24	48	19510308	19510355
chr1_3894	p3	(TTA)5	15	19544813	19544827
chr1_3895	p2	(AT)10	20	19546023	19546042
chr1_3896	p2	(AC)10	20	19547138	19547157

chr1_3897	p2	(CA)7	14	19572865	19572878
chr1_3898	p4	(AGAT)12	48	19577511	19577558
chr1_3899	p2	(CA)29	58	19578673	19578730
chr1_3900	p3	(AGG)5	15	19581023	19581037
chr1_3901	p2	(TG)14	28	19598696	19598723
chr1_3902	p3	(AAG)5	15	19606041	19606055
chr1_3903	p2	(GT)6	12	19612605	19612616
chr1_3904	p2	(TG)10	20	19618410	19618429
chr1_3905	p2	(CA)7	14	19620874	19620887
chr1_3906	p2	(GT)6	12	19621544	19621555
chr1_3907	p3	(TTA)14	42	19630808	19630849
chr1_3908	p2	(AC)6	12	19645784	19645795
chr1_3909	p2	(AC)15	30	19652923	19652952
chr1_3910	p2	(AC)17	34	19655740	19655773
chr1_3911	p2	(AC)6	12	19658298	19658309
chr1_3912	p2	(AC)8	16	19658967	19658982
chr1_3913	p2	(AG)10	20	19660709	19660728
chr1_3914	p2	(CA)18	36	19666145	19666180
chr1_3915	p3	(CAT)14	42	19666891	19666932
chr1_3916	p2	(GA)11	22	19669392	19669413
chr1_3917	p3	(TCC)5	15	19681311	19681325
chr1_3918	c	(TG)9c(GT)10	39	19681551	19681589
chr1_3919	p2	(GT)6	12	19687700	19687711
chr1_3920	p2	(CA)7	14	19693006	19693019
chr1_3921	p2	(AC)6	12	19698112	19698123
chr1_3922	p2	(AG)8	16	19713877	19713892
chr1_3923	p2	(TG)8	16	19716127	19716142
chr1_3924	p3	(TAT)5	15	19717414	19717428
chr1_3925	p2	(CA)11	22	19724360	19724381
chr1_3926	p3	(GAA)8	24	19738141	19738164
chr1_3927	p2	(AC)7	14	19756951	19756964
chr1_3928	p2	(AG)8	16	19762514	19762529
chr1_3929	p3	(AAC)5	15	19798302	19798316
chr1_3930	p2	(TG)7	14	19800164	19800177
chr1_3931	p2	(TG)8	16	19802658	19802673
chr1_3932	p3	(TTG)7	21	19804288	19804308
chr1_3933	p2	(TG)27	54	19812976	19813029
chr1_3934	p3	(ATT)5	15	19824600	19824614
chr1_3935	p2	(AC)6	12	19824734	19824745
chr1_3936	p3	(AAT)6	18	19834092	19834109
chr1_3937	p5	(AATTG)7	35	19866372	19866406
chr1_3938	p2	(GT)33	66	19880061	19880126
chr1_3939	p2	(AG)7	14	19904917	19904930
chr1_3940	p2	(GT)12	24	19910545	19910568
chr1_3941	p3	(TTA)10	30	19923442	19923471
chr1_3942	p2	(GT)6	12	19935884	19935895
chr1_3943	p2	(CA)14	28	19942990	19943017
chr1_3944	p2	(AC)13	26	19956865	19956890
chr1_3945	p2	(TG)7	14	19965169	19965182
chr1_3946	p4	(TTTA)5	20	19978935	19978954
chr1_3947	p2	(AG)16	32	19979882	19979913
chr1_3948	p4	(AATC)5	20	19988260	19988279

chr1_3949	p2	(AG)6	12	19988574	19988585
chr1_3950	p2	(TG)8	16	19991378	19991393
chr1_3951	p2	(TG)9	18	19995061	19995078
chr1_3952	p2	(TG)6	12	20026768	20026779
chr1_3953	p3	(GTA)9	27	20027746	20027772
chr1_3954	p2	(AC)21	42	20067752	20067793
chr1_3955	p2	(TG)14	28	20080424	20080451
chr1_3956	p2	(AG)7	14	20089111	20089124
chr1_3957	p2	(CA)23	46	20096989	20097034
chr1_3958	p2	(CA)10	20	20100575	20100594
chr1_3959	p2	(TG)12	24	20106251	20106274
chr1_3960	p2	(AT)7	14	20124236	20124249
chr1_3961	p5	(GAATA)11	55	20152506	20152560
chr1_3962	p2	(CT)11	22	20156101	20156122
chr1_3963	c	(TG)6a(GT)18	49	20156279	20156327
chr1_3964	p2	(TG)15	30	20159857	20159886
chr1_3965	p2	(GT)17	34	20161840	20161873
chr1_3966	p2	(GT)9	18	20164531	20164548
chr1_3967	p2	(GT)6	12	20171415	20171426
chr1_3968	p2	(AG)8	16	20172685	20172700
chr1_3969	p2	(TG)9	18	20173199	20173216
chr1_3970	p2	(TG)6	12	20173550	20173561
chr1_3971	p2	(TG)7	14	20177491	20177504
chr1_3972	p3	(TGA)7	21	20188312	20188332
chr1_3973	c	(CT)6cat(AC)14	43	20197333	20197375
chr1_3974	p2	(AC)8	16	20197657	20197672
chr1_3975	p2	(AC)7	14	20200159	20200172
chr1_3976	p2	(AT)6	12	20219460	20219471
chr1_3977	p2	(AC)6	12	20221290	20221301
chr1_3978	p2	(TC)20	40	20227935	20227974
chr1_3979	p2	(GT)8	16	20231349	20231364
chr1_3980	p3	(AGG)6	18	20233869	20233886
chr1_3981	p2	(TG)11	22	20234613	20234634
chr1_3982	p2	(CT)7	14	20235372	20235385
chr1_3983	p2	(GT)9	18	20240327	20240344
chr1_3984	p2	(CA)12	24	20240476	20240499
chr1_3985	p2	(AC)8	16	20244138	20244153
chr1_3986	p3	(ATA)5	15	20250170	20250184
chr1_3987	p2	(TC)6	12	20250343	20250354
chr1_3988	p2	(CA)9	18	20253091	20253108
chr1_3989	p2	(TG)12	24	20278244	20278267
chr1_3990	p3	(TGC)5	15	20278566	20278580
chr1_3991	p2	(GT)16	32	20287837	20287868
chr1_3992	p2	(TG)6	12	20288481	20288492
chr1_3993	p2	(TG)7	14	20288626	20288639
chr1_3994	p2	(TG)11	22	20289829	20289850
chr1_3995	p2	(TG)9	18	20291184	20291201
chr1_3996	p2	(GT)7	14	20293760	20293773
chr1_3997	p2	(TG)14	28	20293953	20293980
chr1_3998	p2	(GT)14	28	20299185	20299212
chr1_3999	p2	(AC)11	22	20301558	20301579
chr1_4000	p3	(ACA)7	21	20311378	20311398

chr1_4001	p2	(TG)7	14	20313157	20313170
chr1_4002	p2	(CA)10	20	20317935	20317954
chr1_4003	p2	(AC)6	12	20323706	20323717
chr1_4004	p2	(TG)6	12	20329531	20329542
chr1_4005	p2	(CT)7	14	20332654	20332667
chr1_4006	p4	(TTTC)5	20	20343151	20343170
chr1_4007	p2	(CT)7	14	20348035	20348048
chr1_4008	p2	(AC)11	22	20354012	20354033
chr1_4009	p2	(GT)14	28	20358401	20358428
chr1_4010	p2	(GT)6	12	20361472	20361483
chr1_4011	p2	(AC)16	32	20376236	20376267
chr1_4012	p2	(TG)15	30	20378239	20378268
chr1_4013	p2	(AG)6	12	20395053	20395064
chr1_4014	p2	(CA)6	12	20398189	20398200
chr1_4015	p2	(AC)8	16	20406736	20406751
chr1_4016	p2	(CA)20	40	20408906	20408945
chr1_4017	p3	(ACA)5	15	20411161	20411175
chr1_4018	p2	(GT)6	12	20428453	20428464
chr1_4019	p4	(GATA)5	20	20430578	20430597
chr1_4020	p4	(TTGT)5	20	20444143	20444162
chr1_4021	c	(CA)6g(AC)12	37	20450620	20450656
chr1_4022	p5	(CTCCT)6	30	20462004	20462033
chr1_4023	c	gcccagaccacccattgttcagggttcattgatcaagatcaagctgacacc	125	20466388	20466512
chr1_4024	p3	(ATA)5	15	20478243	20478257
chr1_4025	p2	(AC)17	34	20483724	20483757
chr1_4026	p2	(CA)6	12	20497038	20497049
chr1_4027	p3	(TTA)5	15	20511374	20511388
chr1_4028	p2	(AC)6	12	20513763	20513774
chr1_4029	p2	(TC)6	12	20520326	20520337
chr1_4030	p2	(CT)6	12	20523429	20523440
chr1_4031	p5	(AGATT)5	25	20525607	20525631
chr1_4032	p2	(TC)6	12	20530523	20530534
chr1_4033	p4	(ATTC)6	24	20535170	20535193
chr1_4034	c	(GT)6g(GT)30	73	20550529	20550601
chr1_4035	p2	(TA)9	18	20553581	20553598
chr1_4036	c	(TA)9c(AT)9	37	20565490	20565526
chr1_4037	p2	(GT)7	14	20576724	20576737
chr1_4038	p2	(GT)10	20	20576946	20576965
chr1_4039	p2	(TG)6	12	20584834	20584845
chr1_4040	p2	(CA)27	54	20586625	20586678
chr1_4041	p2	(GA)13	26	20588814	20588839
chr1_4042	p2	(CA)16	32	20589930	20589961
chr1_4043	p2	(CA)9	18	20598981	20598998
chr1_4044	p2	(TC)6	12	20608622	20608633
chr1_4045	p4	(TTAC)5	20	20614021	20614040
chr1_4046	p2	(GT)6	12	20623631	20623642
chr1_4047	c	(CT)9t(TC)8	35	20626744	20626778
chr1_4048	p2	(TG)11	22	20628145	20628166
chr1_4049	p2	(GT)6	12	20640434	20640445
chr1_4050	p2	(CA)6	12	20640619	20640630
chr1_4051	c	(GT)6a(TG)10	33	20646803	20646835
chr1_4052	p2	(TC)27	54	20647007	20647060

chr1_4053	p2	(AC)27	54	20647939	20647992
chr1_4054	p2	(CT)7	14	20655459	20655472
chr1_4055	p2	(AG)6	12	20659039	20659050
chr1_4056	p2	(AC)22	44	20663854	20663897
chr1_4057	p2	(TG)7	14	20671700	20671713
chr1_4058	p2	(GT)6	12	20680161	20680172
chr1_4059	p4	(TTTA)5	20	20681080	20681099
chr1_4060	p2	(AC)22	44	20688730	20688773
chr1_4061	p4	(CATC)5	20	20695652	20695671
chr1_4062	c	aatgatcagtgtcaagatttgaggagtggacttcctttctgacttgtccgag	121	20696586	20696706
chr1_4063	p2	(CA)18	36	20698183	20698218
chr1_4064	p3	(AGT)10	30	20708322	20708351
chr1_4065	p2	(AG)6	12	20717862	20717873
chr1_4066	p2	(AC)6	12	20736195	20736206
chr1_4067	p2	(CA)6	12	20741089	20741100
chr1_4068	p2	(TG)6	12	20776183	20776194
chr1_4069	p2	(GT)7	14	20776444	20776457
chr1_4070	p4	(ATGA)5	20	20777396	20777415
chr1_4071	p2	(GC)6	12	20779040	20779051
chr1_4072	p2	(GT)12	24	20801164	20801187
chr1_4073	p3	(AAC)7	21	20802757	20802777
chr1_4074	p2	(CA)7	14	20809763	20809776
chr1_4075	p2	(TG)20	40	20817983	20818022
chr1_4076	p2	(TG)10	20	20818202	20818221
chr1_4077	p3	(CTG)5	15	20825954	20825968
chr1_4078	p4	(TTTG)5	20	20846247	20846266
chr1_4079	p2	(GA)7	14	20848593	20848606
chr1_4080	p2	(GT)10	20	20848819	20848838
chr1_4081	p2	(CA)6	12	20851964	20851975
chr1_4082	c	(TG)10tat(AC)6	35	20866167	20866201
chr1_4083	c	(GT)6g(GT)30	73	20870053	20870125
chr1_4084	p2	(CT)9	18	20876541	20876558
chr1_4085	p3	(GGA)7	21	20877103	20877123
chr1_4086	p3	(TAT)7	21	20885832	20885852
chr1_4087	p4	(TTTG)5	20	20895842	20895861
chr1_4088	p4	(TGTT)9	36	20902194	20902229
chr1_4089	p3	(CAA)6	18	20910322	20910339
chr1_4090	p2	(CA)6	12	20914671	20914682
chr1_4091	p2	(CA)7	14	20929226	20929239
chr1_4092	p2	(AT)6	12	20929918	20929929
chr1_4093	p4	(AAAT)5	20	20931382	20931401
chr1_4094	p2	(GT)6	12	20932305	20932316
chr1_4095	p3	(AGC)5	15	20941952	20941966
chr1_4096	p2	(GT)12	24	20948647	20948670
chr1_4097	p2	(CA)7	14	20961943	20961956
chr1_4098	p3	(AGA)5	15	20965178	20965192
chr1_4099	p2	(GT)10	20	20968518	20968537
chr1_4100	p4	(AAAT)5	20	20978097	20978116
chr1_4101	p5	(TTAGC)5	25	20983447	20983471
chr1_4102	p2	(AT)8	16	20983764	20983779
chr1_4103	p3	(AAT)6	18	20988900	20988917
chr1_4104	c	(CA)14a(AC)6	41	21002182	21002222

chr1_4105	p2	(CA)6	12	21016101	21016112
chr1_4106	p3	(AGT)9	27	21042690	21042716
chr1_4107	p2	(AT)6	12	21051291	21051302
chr1_4108	p2	(TG)6	12	21059714	21059725
chr1_4109	c	(GT)6g(GT)30	73	21060975	21061047
chr1_4110	p3	(CAA)7	21	21067813	21067833
chr1_4111	p2	(GT)6	12	21070469	21070480
chr1_4112	p3	(GGT)5	15	21097263	21097277
chr1_4113	p2	(TG)12	24	21100841	21100864
chr1_4114	p3	(ACA)6	18	21130512	21130529
chr1_4115	p2	(TG)7	14	21137252	21137265
chr1_4116	p3	(AAC)5	15	21158867	21158881
chr1_4117	p2	(CT)6	12	21167449	21167460
chr1_4118	p4	(TTAT)6	24	21200040	21200063
chr1_4119	p2	(TG)8	16	21205311	21205326
chr1_4120	p2	(GT)15	30	21212808	21212837
chr1_4121	p2	(CA)6	12	21213089	21213100
chr1_4122	c	(GT)6a(TG)7	27	21214627	21214653
chr1_4123	p3	(CTT)5	15	21222815	21222829
chr1_4124	p2	(AC)6	12	21224295	21224306
chr1_4125	p2	(AC)6	12	21243985	21243996
chr1_4126	p2	(CA)6	12	21249615	21249626
chr1_4127	p2	(AC)8	16	21257740	21257755
chr1_4128	p2	(GA)6	12	21261599	21261610
chr1_4129	c	(TG)7agtat(GA)6gc(GA)6	45	21266012	21266056
chr1_4130	p2	(TG)10	20	21267422	21267441
chr1_4131	p2	(GT)8	16	21267856	21267871
chr1_4132	p2	(AC)7	14	21277513	21277526
chr1_4133	p2	(CA)6	12	21277927	21277938
chr1_4134	p2	(CA)16	32	21283071	21283102
chr1_4135	p2	(CA)6	12	21286067	21286078
chr1_4136	p2	(GT)13	26	21294654	21294679
chr1_4137	p3	(ACC)6	18	21298907	21298924
chr1_4138	p3	(TTG)5	15	21301819	21301833
chr1_4139	p2	(TG)14	28	21304130	21304157
chr1_4140	p2	(GT)13	26	21308406	21308431
chr1_4141	p2	(CA)10	20	21310775	21310794
chr1_4142	p4	(CAGT)5	20	21321774	21321793
chr1_4143	p2	(AC)6	12	21330786	21330797
chr1_4144	p2	(TG)6	12	21339800	21339811
chr1_4145	p3	(TTA)8	24	21344409	21344432
chr1_4146	p4	(TTTA)7	28	21354477	21354504
chr1_4147	p2	(TA)9	18	21396037	21396054
chr1_4148	p2	(TA)9	18	21400198	21400215
chr1_4149	p3	(TGT)7	21	21431395	21431415
chr1_4150	p2	(GA)25	50	21445942	21445991
chr1_4151	c	(TTA)7(TTG)10	51	21447568	21447618
chr1_4152	p3	(ATC)5	15	21465888	21465902
chr1_4153	p2	(TG)6	12	21468755	21468766
chr1_4154	c	T)7aggaccatcccgaagtggggcaagtggtaaataaatgtgtatg(TA	81	21470002	21470082
chr1_4155	p2	(AG)13	26	21470625	21470650
chr1_4156	p2	(AC)21	42	21482636	21482677

chr1_4157	p2	(CA)11	22	21485853	21485874
chr1_4158	p2	(GT)12	24	21486194	21486217
chr1_4159	p4	(TATC)7	28	21532049	21532076
chr1_4160	c	(TA)9tc(TA)7	34	21535037	21535070
chr1_4161	c	(GT)12(GA)21	66	21536595	21536660
chr1_4162	c	(TA)7agtgtgtc(TG)6	34	21540783	21540816
chr1_4163	p4	(TCTA)7	28	21541973	21542000
chr1_4164	p3	(AAT)5	15	21542496	21542510
chr1_4165	p2	(CA)9	18	21550116	21550133
chr1_4166	p2	(CA)7	14	21552481	21552494
chr1_4167	p2	(TA)8	16	21556109	21556124
chr1_4168	p3	(TAT)6	18	21557692	21557709
chr1_4169	p4	(TTTA)5	20	21568204	21568223
chr1_4170	p2	(TG)20	40	21596074	21596113
chr1_4171	p2	(GT)8	16	21608109	21608124
chr1_4172	p2	(GA)7	14	21609662	21609675
chr1_4173	p2	(TA)6	12	21611177	21611188
chr1_4174	p2	(AC)7	14	21625467	21625480
chr1_4175	p2	(TC)6	12	21637427	21637438
chr1_4176	c	(TG)11ta(TG)8	40	21640446	21640485
chr1_4177	p2	(GT)8	16	21645038	21645053
chr1_4178	c	(TG)6(AG)9	30	21669446	21669475
chr1_4179	p2	(CA)9	18	21682409	21682426
chr1_4180	p3	(CAG)5	15	21694550	21694564
chr1_4181	p2	(TG)6	12	21696509	21696520
chr1_4182	p2	(TG)14	28	21697145	21697172
chr1_4183	c	(CA)11t(AC)7	37	21703313	21703349
chr1_4184	p2	(CA)9	18	21703563	21703580
chr1_4185	p2	(TC)11	22	21703692	21703713
chr1_4186	p3	(TTA)6	18	21705402	21705419
chr1_4187	c	(CTCTCC)7(CT)7(CA)9	74	21709863	21709936
chr1_4188	c	ctcctgccctgggtcatgcaacatgttattctccttctacaggcctcatgagtt	124	21716962	21717085
chr1_4189	p2	(GA)6	12	21718041	21718052
chr1_4190	p2	(AC)16	32	21724828	21724859
chr1_4191	p2	(CA)14	28	21742071	21742098
chr1_4192	p2	(AC)9	18	21764304	21764321
chr1_4193	p2	(TG)20	40	21785998	21786037
chr1_4194	p2	(GT)6	12	21791098	21791109
chr1_4195	p2	(CT)6	12	21799339	21799350
chr1_4196	p2	(TG)7	14	21826816	21826829
chr1_4197	p2	(GT)55	110	21831804	21831913
chr1_4198	p2	(AC)9	18	21854446	21854463
chr1_4199	p2	(GT)6	12	21868057	21868068
chr1_4200	p2	(TG)7	14	21870332	21870345
chr1_4201	c	(CT)7cgatctctctctctt(AC)7	43	21870915	21870957
chr1_4202	p2	(CA)6	12	21904311	21904322
chr1_4203	p3	(TGG)8	24	21905513	21905536
chr1_4204	p2	(TG)12	24	21911150	21911173
chr1_4205	p2	(TC)8	16	21925627	21925642
chr1_4206	c	ttcaatattcctaatatattccttcaagctcaggtcctcttatctctt(AG)6aa	126	21936259	21936384
chr1_4207	p2	(TA)11	22	21937796	21937817
chr1_4208	p2	(AC)7	14	21948717	21948730

chr1_4209	p2	(AC)7	14	21963704	21963717
chr1_4210	p3	(AAT)8	24	21965689	21965712
chr1_4211	p2	(CA)6	12	21972903	21972914
chr1_4212	p3	(CTC)6	18	21999451	21999468
chr1_4213	p2	(CA)6	12	22012898	22012909
chr1_4214	p2	(GA)7	14	22015795	22015808
chr1_4215	p2	(TA)8	16	22030914	22030929
chr1_4216	p2	(AT)6	12	22067928	22067939
chr1_4217	p3	(TTA)5	15	22078733	22078747
chr1_4218	p2	(CT)11	22	22087872	22087893
chr1_4219	p2	(CT)11	22	22088555	22088576
chr1_4220	p2	(AC)6	12	22096283	22096294
chr1_4221	p2	(TG)11	22	22107266	22107287
chr1_4222	p3	(TCC)6	18	22119865	22119882
chr1_4223	p2	(GT)10	20	22125860	22125879
chr1_4224	c*	(TC)13(TCTA)7*	52	22129162	22129213
chr1_4225	p3	(AAT)5	15	22131847	22131861
chr1_4226	p2	(CA)6	12	22141296	22141307
chr1_4227	p2	(GA)6	12	22147072	22147083
chr1_4228	p2	(AG)6	12	22150403	22150414
chr1_4229	p2	(AC)14	28	22151751	22151778
chr1_4230	p2	(AC)11	22	22153849	22153870
chr1_4231	p2	(AC)8	16	22197992	22198007
chr1_4232	p2	(CA)7	14	22200096	22200109
chr1_4233	c	:atcccacagtctctga(AC)11gttgccttctacttacacataacagacta	229	22204523	22204751
chr1_4234	p3	(AGA)13	39	22211955	22211993
chr1_4235	p3	(GGA)5	15	22217956	22217970
chr1_4236	p2	(AC)11	22	22228033	22228054
chr1_4237	p2	(CA)6	12	22229198	22229209
chr1_4238	p2	(TA)6	12	22243244	22243255
chr1_4239	p2	(AC)14	28	22257970	22257997
chr1_4240	p2	(TG)27	54	22261102	22261155
chr1_4241	p2	(TG)34	68	22262351	22262418
chr1_4242	p2	(AC)7	14	22265887	22265900
chr1_4243	p2	(CA)7	14	22268420	22268433
chr1_4244	p2	(CT)6	12	22271681	22271692
chr1_4245	p2	(GT)6	12	22272761	22272772
chr1_4246	p3	(AGA)5	15	22274228	22274242
chr1_4247	p2	(TG)14	28	22280120	22280147
chr1_4248	p2	(AC)16	32	22282437	22282468
chr1_4249	c	(TG)11ttggttgagagagggagagagaa(AG)6	57	22287350	22287406
chr1_4250	p2	(AC)9	18	22288725	22288742
chr1_4251	p3	(AAT)5	15	22293217	22293231
chr1_4252	p2	(TC)10	20	22301577	22301596
chr1_4253	p5	(TATGA)11	55	22310656	22310710
chr1_4254	p2	(TG)10	20	22311303	22311322
chr1_4255	p2	(GT)6	12	22370823	22370834
chr1_4256	p2	(GA)6	12	22377998	22378009
chr1_4257	p2	(AC)6	12	22393154	22393165
chr1_4258	p4	(ATTG)5	20	22394013	22394032
chr1_4259	p2	(AC)13	26	22409371	22409396
chr1_4260	p2	(TG)9	18	22432594	22432611

chr1_4261	p2	(AC)7	14	22446406	22446419
chr1_4262	p2	(AC)8	16	22456205	22456220
chr1_4263	p2	(AC)11	22	22476240	22476261
chr1_4264	p4	(ATTT)5	20	22483139	22483158
chr1_4265	p3	(TCA)6	18	22491456	22491473
chr1_4266	p2	(GT)7	14	22494094	22494107
chr1_4267	p2	(AC)8	16	22495607	22495622
chr1_4268	p3	(AGG)8	24	22496317	22496340
chr1_4269	p2	(TC)6	12	22499870	22499881
chr1_4270	p2	(AC)6	12	22500198	22500209
chr1_4271	p2	(AC)13	26	22509015	22509040
chr1_4272	p2	(AC)9	18	22546629	22546646
chr1_4273	p2	(AC)7	14	22565553	22565566
chr1_4274	p2	(GT)9	18	22566501	22566518
chr1_4275	p3	(AGT)7	21	22593140	22593160
chr1_4276	p2	(GT)8	16	22599888	22599903
chr1_4277	p2	(GT)7	14	22602606	22602619
chr1_4278	p2	(CT)6	12	22603677	22603688
chr1_4279	p2	(GT)25	50	22605198	22605247
chr1_4280	p2	(AT)10	20	22606470	22606489
chr1_4281	p2	(TA)7	14	22608146	22608159
chr1_4282	p2	(GT)20	40	22615742	22615781
chr1_4283	c	tagaaaagtttagcttgtttggttaccagtacagaacaacaccaccaaacaa	119	22619058	22619176
chr1_4284	p3	(GCT)6	18	22631380	22631397
chr1_4285	p2	(AT)11	22	22638057	22638078
chr1_4286	p2	(CA)19	38	22646505	22646542
chr1_4287	p2	(TG)11	22	22648561	22648582
chr1_4288	p2	(GT)16	32	22653905	22653936
chr1_4289	p4	(GTAT)8	32	22665225	22665256
chr1_4290	p2	(CA)14	28	22668225	22668252
chr1_4291	p2	(TG)15	30	22701570	22701599
chr1_4292	p5	(TGATT)5	25	22714482	22714506
chr1_4293	p2	(TG)11	22	22717407	22717428
chr1_4294	p2	(AC)7	14	22718851	22718864
chr1_4295	p3	(TTG)5	15	22729470	22729484
chr1_4296	p2	(GT)8	16	22734977	22734992
chr1_4297	p3	(TAA)5	15	22736643	22736657
chr1_4298	p2	(TA)12	24	22738674	22738697
chr1_4299	p2	(CA)8	16	22761739	22761754
chr1_4300	p3	(AAT)6	18	22767637	22767654
chr1_4301	p2	(GT)14	28	22791962	22791989
chr1_4302	p2	(GA)7	14	22800933	22800946
chr1_4303	p2	(TG)10	20	22819908	22819927
chr1_4304	p2	(TG)8	16	22826851	22826866
chr1_4305	p5	(AAATC)5	25	22832225	22832249
chr1_4306	p2	(AC)8	16	22846270	22846285
chr1_4307	p4	(TTTA)6	24	22859741	22859764
chr1_4308	p2	(AG)6	12	22863779	22863790
chr1_4309	p2	(AT)9	18	22868345	22868362
chr1_4310	p2	(CA)8	16	22869276	22869291
chr1_4311	p2	(TG)15	30	22869660	22869689
chr1_4312	p2	(AC)19	38	22876792	22876829

chr1_4313	p2	(GT)15	30	22879317	22879346
chr1_4314	p2	(CT)6	12	22889728	22889739
chr1_4315	p2	(AC)7	14	22937827	22937840
chr1_4316	p2	(AC)8	16	22981844	22981859
chr1_4317	p3	(CTG)8	24	22983465	22983488
chr1_4318	p2	(CA)15	30	22984641	22984670
chr1_4319	p2	(AC)16	32	22986927	22986958
chr1_4320	p2	(TG)6	12	23008948	23008959
chr1_4321	p2	(AC)7	14	23010521	23010534
chr1_4322	p2	(AG)6	12	23040030	23040041
chr1_4323	p2	(TG)6	12	23042574	23042585
chr1_4324	p2	(CA)16	32	23072764	23072795
chr1_4325	p2	(AC)16	32	23077679	23077710
chr1_4326	p2	(TG)7	14	23085754	23085767
chr1_4327	p2	(TG)6	12	23087809	23087820
chr1_4328	p2	(GT)11	22	23096633	23096654
chr1_4329	p2	(TA)6	12	23122955	23122966
chr1_4330	p3	(TCA)8	24	23126576	23126599
chr1_4331	p2	(CA)6	12	23135702	23135713
chr1_4332	p3	(TTG)6	18	23137559	23137576
chr1_4333	p3	(TCT)5	15	23137712	23137726
chr1_4334	p2	(TC)6	12	23145756	23145767
chr1_4335	p2	(TG)13	26	23178590	23178615
chr1_4336	p2	(AC)24	48	23188519	23188566
chr1_4337	p2	(TG)9	18	23200589	23200606
chr1_4338	p2	(CA)21	42	23202115	23202156
chr1_4339	p2	(AT)6	12	23209759	23209770
chr1_4340	p2	(TC)6	12	23219119	23219130
chr1_4341	p2	(CA)21	42	23222924	23222965
chr1_4342	p3	(TAA)7	21	23231298	23231318
chr1_4343	p2	(TA)11	22	23234269	23234290
chr1_4344	p4	(AAAT)5	20	23251678	23251697
chr1_4345	p2	(CA)7	14	23258362	23258375
chr1_4346	p3	(TAT)6	18	23268313	23268330
chr1_4347	p2	(GT)16	32	23269971	23270002
chr1_4348	p2	(CT)6	12	23288120	23288131
chr1_4349	p2	(TG)16	32	23301952	23301983
chr1_4350	p3	(TTC)7	21	23305308	23305328
chr1_4351	p2	(GT)8	16	23320828	23320843
chr1_4352	p2	(AT)7	14	23327925	23327938
chr1_4353	p2	(CA)8	16	23330152	23330167
chr1_4354	p2	(CT)17	34	23330541	23330574
chr1_4355	p2	(GT)9	18	23331363	23331380
chr1_4356	p2	(AC)12	24	23340993	23341016
chr1_4357	p2	(CA)11	22	23369352	23369373
chr1_4358	p2	(AG)9	18	23371398	23371415
chr1_4359	p3	(AAT)6	18	23375482	23375499
chr1_4360	p2	(TG)14	28	23402587	23402614
chr1_4361	p2	(GT)16	32	23403035	23403066
chr1_4362	p3	(AGT)7	21	23422149	23422169
chr1_4363	p5	(AGAAA)5	25	23441223	23441247
chr1_4364	p2	(TG)8	16	23441777	23441792

chr1_4365	p2	(TG)13	26	23447496	23447521
chr1_4366	p2	(GT)12	24	23462665	23462688
chr1_4367	p2	(CA)22	44	23467719	23467762
chr1_4368	p2	(TG)7	14	23479682	23479695
chr1_4369	p2	(CA)10	20	23483421	23483440
chr1_4370	p5	(AAATC)5	25	23494935	23494959
chr1_4371	p4	(CTTT)7	28	23495093	23495120
chr1_4372	p2	(TC)6	12	23506640	23506651
chr1_4373	p2	(CT)6	12	23514025	23514036
chr1_4374	p3	(TTG)10	30	23533610	23533639
chr1_4375	p2	(TG)7	14	23535577	23535590
chr1_4376	p4	(ATCC)5	20	23543451	23543470
chr1_4377	p2	(TG)9	18	23552336	23552353
chr1_4378	p2	(TA)9	18	23570134	23570151
chr1_4379	p2	(GT)21	42	23574203	23574244
chr1_4380	p4	(AAAT)5	20	23582509	23582528
chr1_4381	p2	(AC)6	12	23583988	23583999
chr1_4382	p4	(CATC)8	32	23590512	23590543
chr1_4383	c	(GGA)5gtcttcttcttt(TTC)5	42	23595483	23595524
chr1_4384	c	gaactaaggataatgtatcgcttcctgatcactatcttatataatcacgtgttg	156	23643756	23643911
chr1_4385	p2	(AC)7	14	23646222	23646235
chr1_4386	p2	(CA)7	14	23646436	23646449
chr1_4387	p2	(TG)17	34	23663766	23663799
chr1_4388	p2	(AC)13	26	23668253	23668278
chr1_4389	p4	(TACT)5	20	23674385	23674404
chr1_4390	p2	(CA)8	16	23687384	23687399
chr1_4391	p2	(AC)8	16	23695404	23695419
chr1_4392	p2	(TG)16	32	23730476	23730507
chr1_4393	p2	(GT)7	14	23739144	23739157
chr1_4394	p2	(GT)6	12	23800273	23800284
chr1_4395	p2	(TC)15	30	23832870	23832899
chr1_4396	p2	(TG)10	20	23845113	23845132
chr1_4397	p2	(TG)13	26	23845417	23845442
chr1_4398	p2	(TG)16	32	23849469	23849500
chr1_4399	p2	(CA)7	14	23850629	23850642
chr1_4400	p2	(CA)6	12	23859135	23859146
chr1_4401	p2	(AC)14	28	23860609	23860636
chr1_4402	p2	(AT)8	16	23863599	23863614
chr1_4403	p2	(CA)12	24	23872314	23872337
chr1_4404	p2	(AC)18	36	23875054	23875089
chr1_4405	p4	(TTTA)6	24	23880461	23880484
chr1_4406	p2	(CA)11	22	23899400	23899421
chr1_4407	p2	(CA)6	12	23905791	23905802
chr1_4408	c	(TTC)7(TTA)8	45	23906810	23906854
chr1_4409	p2	(TG)6	12	23923572	23923583
chr1_4410	c	gaacggcggcagagagagacttttagtttggagtaggtgagagagaggc	112	23931243	23931354
chr1_4411	p2	(TG)14	28	23937395	23937422
chr1_4412	p2	(TG)12	24	23937637	23937660
chr1_4413	p2	(AG)7	14	23943967	23943980
chr1_4414	p4	(GTTT)6	24	23947364	23947387
chr1_4415	p2	(CA)14	28	23955350	23955377
chr1_4416	c	ctaaataacatctgatataaagtatatatagataagatataccaagatggat	136	23985649	23985784

chr1_4417	p2	(AC)9	18	24003279	24003296
chr1_4418	c	(AT)12(AC)6	36	24032948	24032983
chr1_4419	p3	(CAG)10	30	24035063	24035092
chr1_4420	p2	(GA)7	14	24042531	24042544
chr1_4421	p3	(GAT)5	15	24042658	24042672
chr1_4422	p2	(TG)32	64	24063148	24063211
chr1_4423	p4	(ATCC)5	20	24067073	24067092
chr1_4424	p2	(TG)13	26	24068701	24068726
chr1_4425	p2	(GT)6	12	24070156	24070167
chr1_4426	p2	(GT)12	24	24072921	24072944
chr1_4427	c	(AC)6at(AC)6	26	24082898	24082923
chr1_4428	p2	(AC)9	18	24087220	24087237
chr1_4429	p2	(AG)8	16	24092599	24092614
chr1_4430	p2	(AC)14	28	24099760	24099787
chr1_4431	p2	(CT)11	22	24102237	24102258
chr1_4432	p2	(TG)7	14	24120236	24120249
chr1_4433	p2	(AC)6	12	24121974	24121985
chr1_4434	p2	(GT)6	12	24148787	24148798
chr1_4435	p3	(AAT)5	15	24165263	24165277
chr1_4436	p2	(AT)6	12	24166427	24166438
chr1_4437	p2	(TC)6	12	24169921	24169932
chr1_4438	p2	(TG)6	12	24170378	24170389
chr1_4439	p2	(CA)9	18	24175095	24175112
chr1_4440	c	(AC)10ag(AC)22	66	24186808	24186873
chr1_4441	p4	(CAAA)5	20	24202643	24202662
chr1_4442	p4	(ATGG)5	20	24216997	24217016
chr1_4443	p3	(AGG)5	15	24223887	24223901
chr1_4444	p2	(GT)11	22	24224055	24224076
chr1_4445	p2	(CA)6	12	24229358	24229369
chr1_4446	p2	(CT)7	14	24236397	24236410
chr1_4447	p2	(GA)8	16	24236518	24236533
chr1_4448	p2	(AC)6	12	24255323	24255334
chr1_4449	p2	(TG)9	18	24259709	24259726
chr1_4450	p2	(TG)7	14	24260886	24260899
chr1_4451	p2	(CA)7	14	24266961	24266974
chr1_4452	p2	(CT)6	12	24272353	24272364
chr1_4453	p2	(TG)10	20	24286612	24286631
chr1_4454	c	(TTC)13ttg(TTC)7	63	24288925	24288987
chr1_4455	p2	(TG)14	28	24309061	24309088
chr1_4456	p2	(GT)11	22	24311258	24311279
chr1_4457	p2	(GT)6	12	24315364	24315375
chr1_4458	p2	(CA)6	12	24326718	24326729
chr1_4459	p4	(TTAA)5	20	24339875	24339894
chr1_4460	p2	(AT)8	16	24348022	24348037
chr1_4461	p2	(CA)13	26	24349309	24349334
chr1_4462	p2	(CA)10	20	24349711	24349730
chr1_4463	c	(GT)6c(TG)6	25	24353196	24353220
chr1_4464	p2	(TC)8	16	24353863	24353878
chr1_4465	p2	(GT)8	16	24354026	24354041
chr1_4466	p3	(TAA)11	33	24362394	24362426
chr1_4467	p2	(TA)8	16	24382838	24382853
chr1_4468	p2	(AC)6	12	24384433	24384444

chr1_4469	p2	(TG)7	14	24386212	24386225
chr1_4470	c	tacaaagatggatcaaagtaacatagctttatacttaatattacaatgtaaag	119	24410572	24410690
chr1_4471	p2	(AC)12	24	24414669	24414692
chr1_4472	p2	(AC)12	24	24417640	24417663
chr1_4473	p3	(ATA)5	15	24425243	24425257
chr1_4474	p2	(AC)13	26	24426079	24426104
chr1_4475	p2	(CA)13	26	24426596	24426621
chr1_4476	p2	(GT)24	48	24426891	24426938
chr1_4477	p2	(CA)7	14	24432452	24432465
chr1_4478	p2	(GT)8	16	24435351	24435366
chr1_4479	p2	(CA)6	12	24441191	24441202
chr1_4480	p2	(CT)6	12	24442888	24442899
chr1_4481	p2	(GT)10	20	24443286	24443305
chr1_4482	p2	(AC)9	18	24445673	24445690
chr1_4483	p2	(CA)10	20	24458076	24458095
chr1_4484	p3	(ATA)5	15	24468735	24468749
chr1_4485	p2	(CT)6	12	24471316	24471327
chr1_4486	p2	(AC)12	24	24483876	24483899
chr1_4487	p2	(TG)14	28	24484892	24484919
chr1_4488	p3	(TTC)9	27	24505946	24505972
chr1_4489	p3	(TTC)9	27	24506223	24506249
chr1_4490	p2	(CA)6	12	24520921	24520932
chr1_4491	p3	(TTC)5	15	24531188	24531202
chr1_4492	p3	(GCA)9	27	24537993	24538019
chr1_4493	p2	(GA)8	16	24560271	24560286
chr1_4494	p2	(AC)6	12	24564443	24564454
chr1_4495	p2	(GT)18	36	24564811	24564846
chr1_4496	p2	(CA)6	12	24575741	24575752
chr1_4497	p2	(AC)6	12	24581238	24581249
chr1_4498	p2	(GT)12	24	24584143	24584166
chr1_4499	p2	(TC)6	12	24588628	24588639
chr1_4500	p3	(TAT)5	15	24590188	24590202
chr1_4501	p2	(AG)20	40	24592269	24592308
chr1_4502	p3	(GAG)7	21	24594910	24594930
chr1_4503	p2	(CA)23	46	24599392	24599437
chr1_4504	p2	(GT)7	14	24599762	24599775
chr1_4505	c	(GT)26(GC)7	66	24605063	24605128
chr1_4506	p2	(CA)6	12	24610350	24610361
chr1_4507	p2	(TG)13	26	24624586	24624611
chr1_4508	p4	(AAAC)5	20	24632710	24632729
chr1_4509	p2	(TG)13	26	24634772	24634797
chr1_4510	c	(TG)6ggtgtgg(GT)6	31	24642548	24642578
chr1_4511	p2	(TG)7	14	24644505	24644518
chr1_4512	p2	(TA)9	18	24647335	24647352
chr1_4513	p3	(ATC)7	21	24648019	24648039
chr1_4514	p2	(GT)7	14	24654674	24654687
chr1_4515	p2	(GT)28	56	24661551	24661606
chr1_4516	p2	(AC)11	22	24665051	24665072
chr1_4517	p3	(AAT)5	15	24667226	24667240
chr1_4518	p2	(TG)6	12	24679014	24679025
chr1_4519	p3	(CTA)7	21	24689029	24689049
chr1_4520	p3	(AAG)21	63	24710842	24710904

chr1_4521	p3	(GAT)13	39	24724452	24724490
chr1_4522	p2	(GT)7	14	24734703	24734716
chr1_4523	p2	(GT)10	20	24737623	24737642
chr1_4524	p2	(GT)7	14	24737915	24737928
chr1_4525	p2	(GT)11	22	24739305	24739326
chr1_4526	c	(GT)8c(TG)13	43	24743657	24743699
chr1_4527	p2	(GA)6	12	24753376	24753387
chr1_4528	p2	(GT)6	12	24777668	24777679
chr1_4529	p2	(TG)7	14	24778374	24778387
chr1_4530	p2	(CA)12	24	24802330	24802353
chr1_4531	p4	(TTCC)7	28	24816637	24816664
chr1_4532	p2	(CA)6	12	24824894	24824905
chr1_4533	p2	(GT)6	12	24826213	24826224
chr1_4534	p3	(GTG)5	15	24850794	24850808
chr1_4535	p2	(CA)23	46	24865225	24865270
chr1_4536	c	(CA)20cccgattctccctctttttgtgggtatcc(CA)19	108	24869096	24869203
chr1_4537	p3	(GAG)8	24	24888372	24888395
chr1_4538	p2	(AC)18	36	24892282	24892317
chr1_4539	p2	(AC)9	18	24893078	24893095
chr1_4540	p2	(AC)7	14	24942632	24942645
chr1_4541	p4	(AATG)6	24	24948895	24948918
chr1_4542	c	(CT)6(CA)11	34	24950289	24950322
chr1_4543	p2	(TG)6	12	24961094	24961105
chr1_4544	p3	(TTA)9	27	24963757	24963783
chr1_4545	p2	(GT)15	30	24967825	24967854
chr1_4546	c	(CT)10ccccttctctccctatctttctccctatc(TG)9	67	24973482	24973548
chr1_4547	p2	(CA)10	20	24979401	24979420
chr1_4548	p2	(TG)15	30	24980659	24980688
chr1_4549	c	(AG)6aa(AG)6	26	24984420	24984445
chr1_4550	p2	(AC)6	12	25000384	25000395
chr1_4551	p4	(AAAT)5	20	25001576	25001595
chr1_4552	p2	(CA)6	12	25005354	25005365
chr1_4553	p3	(GGA)5	15	25016985	25016999
chr1_4554	p2	(GT)27	54	25027268	25027321
chr1_4555	p3	(TAT)5	15	25035841	25035855
chr1_4556	p2	(AT)9	18	25044607	25044624
chr1_4557	p3	(ATT)7	21	25048893	25048913
chr1_4558	p3	(AAT)5	15	25053270	25053284
chr1_4559	p2	(CA)26	52	25055210	25055261
chr1_4560	p2	(TG)13	26	25057986	25058011
chr1_4561	p3	(CTA)5	15	25064942	25064956
chr1_4562	p4	(TAGC)5	20	25076172	25076191
chr1_4563	p4	(TATT)8	32	25085841	25085872
chr1_4564	p2	(GA)6	12	25096318	25096329
chr1_4565	p2	(GA)14	28	25123459	25123486
chr1_4566	p2	(CA)10	20	25162185	25162204
chr1_4567	p2	(AC)6	12	25171482	25171493
chr1_4568	c	(CG)7cacacacacg(CA)13	50	25172934	25172983
chr1_4569	p2	(CA)22	44	25180993	25181036
chr1_4570	c	(AC)9ag(AC)9ag(AC)9ag(AC)10	80	25188148	25188227
chr1_4571	p2	(TA)6	12	25191650	25191661
chr1_4572	p2	(AT)7	14	25205496	25205509

chr1_4573	p3	(AGA)5	15	25219581	25219595
chr1_4574	p2	(AC)7	14	25220997	25221010
chr1_4575	p3	(AAG)8	24	25248801	25248824
chr1_4576	p2	(AC)14	28	25257505	25257532
chr1_4577	p2	(TG)16	32	25258505	25258536
chr1_4578	p2	(TG)11	22	25282923	25282944
chr1_4579	c	acactcaaacatacacagtcaaagcaatacagtgtcgcacacacacaga	116	25299561	25299676
chr1_4580	p3	(ATC)5	15	25322219	25322233
chr1_4581	p2	(CA)8	16	25341831	25341846
chr1_4582	p2	(TG)13	26	25351267	25351292
chr1_4583	p2	(AC)21	42	25354099	25354140
chr1_4584	p2	(CA)6	12	25363446	25363457
chr1_4585	c	(AT)6(AG)7	26	25387882	25387907
chr1_4586	p3	(ATA)6	18	25389623	25389640
chr1_4587	p3	(TGC)5	15	25417916	25417930
chr1_4588	p2	(AC)14	28	25437561	25437588
chr1_4589	p4	(CATC)6	24	25443197	25443220
chr1_4590	p2	(GT)20	40	25444206	25444245
chr1_4591	p2	(CA)28	56	25445288	25445343
chr1_4592	p2	(GT)6	12	25451152	25451163
chr1_4593	p2	(AC)8	16	25499847	25499862
chr1_4594	p2	(AC)11	22	25512504	25512525
chr1_4595	p2	(AC)14	28	25519230	25519257
chr1_4596	p2	(CT)7	14	25538377	25538390
chr1_4597	p3	(CAG)8	24	25540487	25540510
chr1_4598	p2	(CT)6	12	25543627	25543638
chr1_4599	p2	(AC)11	22	25544359	25544380
chr1_4600	p3	(AGG)5	15	25548558	25548572
chr1_4601	p2	(TG)6	12	25549218	25549229
chr1_4602	p3	(AGC)5	15	25556920	25556934
chr1_4603	p2	(GT)12	24	25562192	25562215
chr1_4604	p2	(TG)38	76	25563627	25563702
chr1_4605	p2	(GT)26	52	25574892	25574943
chr1_4606	p2	(TC)8	16	25577395	25577410
chr1_4607	p2	(CT)6	12	25582573	25582584
chr1_4608	p3	(ATT)7	21	25592020	25592040
chr1_4609	p3	(GCT)5	15	25601860	25601874
chr1_4610	p2	(AC)17	34	25608465	25608498
chr1_4611	p2	(CA)27	54	25620689	25620742
chr1_4612	p2	(TC)6	12	25623355	25623366
chr1_4613	p5	(TTTGA)5	25	25631062	25631086
chr1_4614	p3	(GAG)6	18	25640128	25640145
chr1_4615	p2	(AC)27	54	25651021	25651074
chr1_4616	p3	(AAC)5	15	25651388	25651402
chr1_4617	p3	(CAG)5	15	25661439	25661453
chr1_4618	p3	(AAC)6	18	25661613	25661630
chr1_4619	p2	(CA)11	22	25669401	25669422
chr1_4620	p5	(AAATC)5	25	25678494	25678518
chr1_4621	p2	(CA)10	20	25686086	25686105
chr1_4622	c	(AC)16aa(AC)12	58	25703486	25703543
chr1_4623	p2	(CA)16	32	25706580	25706611
chr1_4624	c	(TG)11taacatacctggccaaataaaggcg(ATT)7	68	25709362	25709429

chr1_4625	p2	(CA)19	38	25710042	25710079
chr1_4626	p2	(AC)11	22	25717753	25717774
chr1_4627	p2	(AT)6	12	25723934	25723945
chr1_4628	p3	(GCT)5	15	25726834	25726848
chr1_4629	p2	(TG)10	20	25729486	25729505
chr1_4630	p2	(AC)10	20	25738965	25738984
chr1_4631	p2	(AC)7	14	25739717	25739730
chr1_4632	p2	(TG)7	14	25744327	25744340
chr1_4633	p2	(GA)9	18	25754274	25754291
chr1_4634	p4	(CATT)5	20	25754538	25754557
chr1_4635	p3	(ATA)6	18	25765978	25765995
chr1_4636	p2	(AT)7	14	25774311	25774324
chr1_4637	p2	(TC)14	28	25779316	25779343
chr1_4638	p2	(CA)8	16	25800113	25800128
chr1_4639	p3	(ATT)7	21	25811926	25811946
chr1_4640	p3	(TTG)5	15	25816222	25816236
chr1_4641	p3	(CAG)6	18	25818626	25818643
chr1_4642	p2	(GT)9	18	25823461	25823478
chr1_4643	p2	(CA)18	36	25823617	25823652
chr1_4644	p2	(TG)13	26	25823870	25823895
chr1_4645	p2	(GT)16	32	25824527	25824558
chr1_4646	p4	(CCAT)5	20	25831382	25831401
chr1_4647	p3	(TCT)5	15	25866040	25866054
chr1_4648	p2	(TG)9	18	25868747	25868764
chr1_4649	p4	(TCAT)6	24	25869826	25869849
chr1_4650	c	(GT)10gacagacagag(GT)6	43	25877073	25877115
chr1_4651	p3	(GGA)5	15	25891114	25891128
chr1_4652	p2	(GT)10	20	25897524	25897543
chr1_4653	p2	(GT)17	34	25897942	25897975
chr1_4654	p2	(GT)17	34	25901663	25901696
chr1_4655	p2	(TC)6	12	25931249	25931260
chr1_4656	p3	(GGT)5	15	25954197	25954211
chr1_4657	p2	(TG)6	12	25958747	25958758
chr1_4658	p2	(AG)7	14	25962148	25962161
chr1_4659	p2	(CA)6	12	25967658	25967669
chr1_4660	p3	(TTA)5	15	25968171	25968185
chr1_4661	p3	(GAG)5	15	25975532	25975546
chr1_4662	p2	(GT)6	12	25980503	25980514
chr1_4663	p2	(AT)8	16	25981316	25981331
chr1_4664	p2	(AC)6	12	26010104	26010115
chr1_4665	p3	(AGA)5	15	26012403	26012417
chr1_4666	c	(TG)8tatatat(AC)11	45	26018159	26018203
chr1_4667	p3	(GGA)5	15	26051937	26051951
chr1_4668	p2	(AC)18	36	26060035	26060070
chr1_4669	p2	(TG)9	18	26076478	26076495
chr1_4670	p2	(GT)10	20	26080801	26080820
chr1_4671	p2	(CT)6	12	26097915	26097926
chr1_4672	p2	(GT)10	20	26100052	26100071
chr1_4673	p4	(AAAC)5	20	26128252	26128271
chr1_4674	p2	(AT)9	18	26131011	26131028
chr1_4675	p2	(TG)6	12	26131187	26131198
chr1_4676	p2	(CT)7	14	26142234	26142247

chr1_4677	p2	(AG)6	12	26144880	26144891
chr1_4678	p2	(AC)8	16	26154861	26154876
chr1_4679	p2	(CT)14	28	26166302	26166329
chr1_4680	p2	(GT)6	12	26171757	26171768
chr1_4681	p2	(TG)6	12	26198121	26198132
chr1_4682	p2	(TG)7	14	26200925	26200938
chr1_4683	c	(CTA)5ctt(CTA)7	39	26223789	26223827
chr1_4684	p2	(TA)10	20	26226563	26226582
chr1_4685	p4	(AAAC)5	20	26230240	26230259
chr1_4686	p2	(TG)6	12	26241153	26241164
chr1_4687	p2	(GA)6	12	26245901	26245912
chr1_4688	p2	(AC)11	22	26252516	26252537
chr1_4689	p4	(ATCC)5	20	26263021	26263040
chr1_4690	p2	(AC)17	34	26267098	26267131
chr1_4691	p2	(TG)11	22	26297197	26297218
chr1_4692	p2	(TG)6	12	26302400	26302411
chr1_4693	p2	(AC)10	20	26306140	26306159
chr1_4694	p3	(AAC)6	18	26312311	26312328
chr1_4695	p3	(TAT)7	21	26334462	26334482
chr1_4696	p2	(TC)6	12	26352529	26352540
chr1_4697	p3	(TAC)5	15	26352993	26353007
chr1_4698	p3	(ATC)6	18	26374342	26374359
chr1_4699	c	(TG)6tctttctt(TC)17tg(TC)7	70	26375637	26375706
chr1_4700	p2	(TA)6	12	26376070	26376081
chr1_4701	p2	(CA)7	14	26379467	26379480
chr1_4702	p2	(TG)6	12	26383242	26383253
chr1_4703	p2	(TG)20	40	26388785	26388824
chr1_4704	p3	(TCA)7	21	26391540	26391560
chr1_4705	p2	(GT)32	64	26392028	26392091
chr1_4706	p2	(GT)6	12	26397739	26397750
chr1_4707	p2	(AG)7	14	26402121	26402134
chr1_4708	p3	(AAG)5	15	26402659	26402673
chr1_4709	c	(AT)9(AG)11	40	26411648	26411687
chr1_4710	p2	(GT)11	22	26419344	26419365
chr1_4711	p2	(AC)6	12	26445758	26445769
chr1_4712	c	(CG)7(CA)7	28	26446225	26446252
chr1_4713	p3	(CAA)6	18	26461873	26461890
chr1_4714	c	(GT)21(GA)11	64	26468070	26468133
chr1_4715	p2	(CA)6	12	26480093	26480104
chr1_4716	p4	(TTTG)6	24	26483242	26483265
chr1_4717	p4	(ATCT)22	88	26489688	26489775
chr1_4718	p3	(TAC)5	15	26497029	26497043
chr1_4719	p2	(TG)6	12	26498475	26498486
chr1_4720	p2	(GT)8	16	26502701	26502716
chr1_4721	p2	(TG)11	22	26513770	26513791
chr1_4722	p2	(TC)9	18	26542131	26542148
chr1_4723	p2	(AG)11	22	26553145	26553166
chr1_4724	p2	(CA)7	14	26554521	26554534
chr1_4725	p2	(GA)6	12	26561062	26561073
chr1_4726	c	(GT)6g(GT)30	73	26561682	26561754
chr1_4727	p2	(GT)9	18	26597372	26597389
chr1_4728	p2	(TA)9	18	26601791	26601808

chr1_4729	p2	(TA)8	16	26615746	26615761
chr1_4730	p2	(GT)6	12	26617239	26617250
chr1_4731	p2	(GT)11	22	26622014	26622035
chr1_4732	c	(TG)19(TA)9	56	26623237	26623292
chr1_4733	p2	(TA)14	28	26623422	26623449
chr1_4734	p2	(AT)7	14	26639161	26639174
chr1_4735	p2	(CA)6	12	26680962	26680973
chr1_4736	p2	(TG)6	12	26700386	26700397
chr1_4737	p2	(TG)14	28	26704229	26704256
chr1_4738	p2	(AG)17	34	26708868	26708901
chr1_4739	p2	(TG)6	12	26710349	26710360
chr1_4740	p2	(CA)6	12	26720779	26720790
chr1_4741	p2	(TG)8	16	26723103	26723118
chr1_4742	p4	(ATTT)5	20	26727512	26727531
chr1_4743	p3	(GAT)6	18	26756035	26756052
chr1_4744	p2	(GT)8	16	26789413	26789428
chr1_4745	p2	(AC)10	20	26791766	26791785
chr1_4746	p2	(GT)13	26	26800538	26800563
chr1_4747	p2	(GT)17	34	26810845	26810878
chr1_4748	p2	(CA)6	12	26815421	26815432
chr1_4749	p2	(TG)6	12	26828163	26828174
chr1_4750	p4	(TTCA)6	24	26832112	26832135
chr1_4751	p2	(GT)18	36	26832459	26832494
chr1_4752	p2	(TG)14	28	26835325	26835352
chr1_4753	p2	(GT)11	22	26835543	26835564
chr1_4754	p3	(CAA)9	27	26846267	26846293
chr1_4755	p3	(ATT)7	21	26852348	26852368
chr1_4756	p2	(TC)7	14	26865612	26865625
chr1_4757	p2	(AC)25	50	26879073	26879122
chr1_4758	p3	(TGT)7	21	26883011	26883031
chr1_4759	p2	(CA)6	12	26886989	26887000
chr1_4760	p2	(CA)7	14	26888940	26888953
chr1_4761	p3	(TCA)5	15	26890981	26890995
chr1_4762	p2	(TG)6	12	26894719	26894730
chr1_4763	p4	(ATGG)7	28	26905762	26905789
chr1_4764	p2	(TG)12	24	26912264	26912287
chr1_4765	p2	(TG)10	20	26918794	26918813
chr1_4766	p5	(TCTCC)5	25	26923900	26923924
chr1_4767	p2	(GT)6	12	26931213	26931224
chr1_4768	p3	(TGT)6	18	26939308	26939325
chr1_4769	p2	(TA)7	14	26939899	26939912
chr1_4770	p2	(AC)7	14	26982728	26982741
chr1_4771	c	(CT)12(CA)24	72	27004572	27004643
chr1_4772	p2	(AC)13	26	27005188	27005213
chr1_4773	p2	(TG)8	16	27006937	27006952
chr1_4774	p2	(GT)7	14	27008554	27008567
chr1_4775	p2	(AT)7	14	27015588	27015601
chr1_4776	p2	(GA)6	12	27022804	27022815
chr1_4777	p3	(TGT)6	18	27024801	27024818
chr1_4778	p2	(CA)6	12	27027514	27027525
chr1_4779	p2	(TG)7	14	27030710	27030723
chr1_4780	p3	(AAG)13	39	27031744	27031782

chr1_4781	p2	(GT)6	12	27033239	27033250
chr1_4782	p2	(AC)6	12	27046426	27046437
chr1_4783	p2	(TG)12	24	27048013	27048036
chr1_4784	c	(AGA)5(GGA)6	33	27048636	27048668
chr1_4785	c	(GT)9gaacaggttttgatcaccacagtgtaatgccaag(CA)17	86	27050138	27050223
chr1_4786	p2	(CA)29	58	27051392	27051449
chr1_4787	p2	(GT)16	32	27058300	27058331
chr1_4788	p2	(TG)22	44	27058469	27058512
chr1_4789	p2	(TG)13	26	27062580	27062605
chr1_4790	c	(GT)10(GA)23	66	27069180	27069245
chr1_4791	p2	(GT)28	56	27071834	27071889
chr1_4792	p3	(AAT)5	15	27075503	27075517
chr1_4793	p3	(TTA)5	15	27081501	27081515
chr1_4794	p2	(TC)8	16	27083609	27083624
chr1_4795	p2	(TG)7	14	27097686	27097699
chr1_4796	p2	(CA)15	30	27098186	27098215
chr1_4797	p2	(AT)6	12	27120785	27120796
chr1_4798	p3	(ATA)5	15	27131078	27131092
chr1_4799	p2	(GT)23	46	27143711	27143756
chr1_4800	p2	(AT)7	14	27155090	27155103
chr1_4801	p3	(TGT)7	21	27157473	27157493
chr1_4802	p3	(GAG)6	18	27165365	27165382
chr1_4803	p2	(CA)33	66	27176624	27176689
chr1_4804	p2	(TG)7	14	27182503	27182516
chr1_4805	p2	(GT)12	24	27186951	27186974
chr1_4806	p3	(AAT)5	15	27262927	27262941
chr1_4807	p2	(CT)6	12	27267432	27267443
chr1_4808	p3	(TTA)6	18	27280851	27280868
chr1_4809	p2	(GT)11	22	27281629	27281650
chr1_4810	p2	(CA)6	12	27293872	27293883
chr1_4811	p3	(ATA)5	15	27311132	27311146
chr1_4812	p4	(AGAC)6	24	27313610	27313633
chr1_4813	p3	(TAT)5	15	27315808	27315822
chr1_4814	p2	(TA)7	14	27317874	27317887
chr1_4815	p2	(AC)11	22	27319973	27319994
chr1_4816	p3	(GTG)5	15	27325005	27325019
chr1_4817	p4	(GATG)6	24	27328787	27328810
chr1_4818	p2	(TG)18	36	27332634	27332669
chr1_4819	p3	(CAG)5	15	27333165	27333179
chr1_4820	p4	(TTTG)5	20	27354932	27354951
chr1_4821	p2	(GT)6	12	27357876	27357887
chr1_4822	p3	(TAT)8	24	27375287	27375310
chr1_4823	p2	(TA)12	24	27375534	27375557
chr1_4824	p2	(AC)23	46	27378973	27379018
chr1_4825	p2	(GT)12	24	27395894	27395917
chr1_4826	p5	(TTTGA)5	25	27396750	27396774
chr1_4827	c	(TG)7c(GT)11	37	27399519	27399555
chr1_4828	p2	(TC)10	20	27404232	27404251
chr1_4829	p2	(AC)8	16	27412790	27412805
chr1_4830	p3	(CAG)5	15	27414688	27414702
chr1_4831	p3	(AAT)5	15	27424483	27424497
chr1_4832	c	[illegible]cacacacgctgagttttactgcaggaacacatgcagtaaaagccagagag[illegible]	108	27426931	27427038

chr1_4833	p2	(GT)6	12	27432285	27432296
chr1_4834	p3	(ATT)7	21	27434890	27434910
chr1_4835	p2	(CT)6	12	27443144	27443155
chr1_4836	p2	(TG)7	14	27460823	27460836
chr1_4837	p5	(GGAGA)5	25	27473227	27473251
chr1_4838	p2	(AC)24	48	27478726	27478773
chr1_4839	c	(TG)15gtg(TA)26	85	27483915	27483999
chr1_4840	p2	(AC)9	18	27485461	27485478
chr1_4841	p3	(TAA)5	15	27512447	27512461
chr1_4842	p3	(GGA)5	15	27520958	27520972
chr1_4843	p3	(GGT)5	15	27527150	27527164
chr1_4844	p2	(AC)8	16	27539868	27539883
chr1_4845	p2	(CA)10	20	27547623	27547642
chr1_4846	p2	(AC)16	32	27554161	27554192
chr1_4847	p2	(TG)7	14	27566197	27566210
chr1_4848	p2	(CA)10	20	27582164	27582183
chr1_4849	p2	(AG)21	42	27601128	27601169
chr1_4850	p2	(CG)7	14	27605294	27605307
chr1_4851	p2	(TG)6	12	27605629	27605640
chr1_4852	p3	(ATG)5	15	27609404	27609418
chr1_4853	p2	(GT)6	12	27609625	27609636
chr1_4854	c	(TGC)6(TGT)5	33	27625207	27625239
chr1_4855	p3	(GTT)5	15	27626212	27626226
chr1_4856	p2	(GT)6	12	27634837	27634848
chr1_4857	p2	(CA)7	14	27634999	27635012
chr1_4858	p2	(AC)12	24	27635239	27635262
chr1_4859	p2	(AC)13	26	27637111	27637136
chr1_4860	p2	(AC)9	18	27639185	27639202
chr1_4861	c	[illegible]ggggtaaatctccgtctgtcggctctgtctgtctgtccatctctcccactcg[illegible]	96	27644562	27644657
chr1_4862	p2	(GA)8	16	27650681	27650696
chr1_4863	p2	(TG)28	56	27656813	27656868
chr1_4864	p3	(ATT)7	21	27672009	27672029
chr1_4865	p2	(AC)15	30	27672361	27672390
chr1_4866	p2	(CA)8	16	27675658	27675673
chr1_4867	p3	(CAG)6	18	27686309	27686326
chr1_4868	p2	(CA)15	30	27695419	27695448
chr1_4869	p3	(TCA)5	15	27703809	27703823
chr1_4870	p2	(AC)14	28	27712817	27712844
chr1_4871	p2	(CA)7	14	27713355	27713368
chr1_4872	p3	(AAC)5	15	27726232	27726246
chr1_4873	p2	(TG)8	16	27751664	27751679
chr1_4874	c	(CAGA)13caaac(AGAT)18	129	27751892	27752020
chr1_4875	c	(AGAC)5agatagacagatagac(AGAT)5	56	27756093	27756148
chr1_4876	p2	(AT)9	18	27762443	27762460
chr1_4877	p2	(TC)12	24	27776468	27776491
chr1_4878	p4	(ATCT)5	20	27776755	27776774
chr1_4879	p2	(AC)18	36	27789693	27789728
chr1_4880	p2	(GT)7	14	27797575	27797588
chr1_4881	p2	(AC)8	16	27802993	27803008
chr1_4882	p2	(AC)10	20	27809738	27809757
chr1_4883	c	(GAG)6c(AGG)5	34	27822085	27822118
chr1_4884	p2	(CT)6	12	27830371	27830382

chr1_4885	p3	(CAG)6	18	27833632	27833649
chr1_4886	p2	(AG)6	12	27851829	27851840
chr1_4887	p3	(TAA)6	18	27855733	27855750
chr1_4888	p3	(AAT)5	15	27859237	27859251
chr1_4889	p4	(ATAG)6	24	27865341	27865364
chr1_4890	p2	(TG)8	16	27871676	27871691
chr1_4891	p2	(CA)7	14	27873393	27873406
chr1_4892	p2	(TG)31	62	27878278	27878339
chr1_4893	p2	(TA)9	18	27883105	27883122
chr1_4894	p2	(AC)7	14	27883980	27883993
chr1_4895	p2	(AC)10	20	27905126	27905145
chr1_4896	p2	(GT)10	20	27906459	27906478
chr1_4897	p2	(CA)7	14	27913635	27913648
chr1_4898	p2	(CA)6	12	27915974	27915985
chr1_4899	p2	(TA)6	12	27916312	27916323
chr1_4900	p2	(TG)13	26	27916621	27916646
chr1_4901	p2	(TG)9	18	27918196	27918213
chr1_4902	p2	(GT)6	12	27919299	27919310
chr1_4903	p3	(TGA)5	15	27942109	27942123
chr1_4904	p2	(GA)6	12	27951883	27951894
chr1_4905	p2	(GA)17	34	27957475	27957508
chr1_4906	p4	(TGGA)5	20	27960491	27960510
chr1_4907	c	[illegible])6agaggagggagagagagagggagggg(GA)8gggagggg(GA[illegible]	84	27961141	27961224
chr1_4908	p2	(TG)10	20	27988533	27988552
chr1_4909	p2	(TG)8	16	27990087	27990102
chr1_4910	p3	(AGG)7	21	28008797	28008817
chr1_4911	p2	(GA)8	16	28031668	28031683
chr1_4912	p2	(AC)6	12	28037429	28037440
chr1_4913	p2	(AG)6	12	28044713	28044724
chr1_4914	p2	(TG)6	12	28082235	28082246
chr1_4915	p2	(AC)6	12	28083642	28083653
chr1_4916	p2	(AT)8	16	28103728	28103743
chr1_4917	p2	(CA)32	64	28106794	28106857
chr1_4918	p2	(AC)10	20	28107089	28107108
chr1_4919	p2	(AC)6	12	28110206	28110217
chr1_4920	p2	(GT)19	38	28120540	28120577
chr1_4921	p2	(GA)23	46	28121020	28121065
chr1_4922	c	(CA)16cg(CA)7	48	28125872	28125919
chr1_4923	p2	(CA)15	30	28132612	28132641
chr1_4924	p2	(GA)6	12	28133131	28133142
chr1_4925	p2	(TG)7	14	28146701	28146714
chr1_4926	p2	(AC)15	30	28155227	28155256
chr1_4927	p3	(TCA)10	30	28155379	28155408
chr1_4928	p2	(CA)9	18	28156012	28156029
chr1_4929	p4	(ATGG)6	24	28159419	28159442
chr1_4930	p2	(TG)14	28	28162890	28162917
chr1_4931	p4	(AAAC)7	28	28174724	28174751
chr1_4932	p3	(ATA)7	21	28192976	28192996
chr1_4933	p2	(AC)21	42	28215342	28215383
chr1_4934	p3	(GTA)5	15	28244852	28244866
chr1_4935	p3	(TAT)5	15	28255247	28255261
chr1_4936	p2	(TG)6	12	28255465	28255476

chr1_4937	p3	(TAT)5	15	28257366	28257380
chr1_4938	p2	(TG)6	12	28257584	28257595
chr1_4939	p2	(TA)6	12	28261028	28261039
chr1_4940	p4	(ATGG)7	28	28262073	28262100
chr1_4941	p2	(GT)12	24	28264995	28265018
chr1_4942	p2	(GT)15	30	28277725	28277754
chr1_4943	c	(TG)11(AG)7	36	28305087	28305122
chr1_4944	p2	(GT)12	24	28307819	28307842
chr1_4945	p3	(TAG)5	15	28312638	28312652
chr1_4946	p2	(AC)9	18	28317693	28317710
chr1_4947	p2	(AC)21	42	28335421	28335462
chr1_4948	p2	(TA)8	16	28338746	28338761
chr1_4949	p2	(AC)12	24	28346727	28346750
chr1_4950	p4	(GCTA)6	24	28349648	28349671
chr1_4951	p3	(CAG)5	15	28360361	28360375
chr1_4952	p2	(TG)12	24	28368788	28368811
chr1_4953	p2	(CT)7	14	28375810	28375823
chr1_4954	p2	(CA)12	24	28377399	28377422
chr1_4955	p2	(AG)7	14	28381932	28381945
chr1_4956	p2	(TG)6	12	28382231	28382242
chr1_4957	p2	(AC)7	14	28384564	28384577
chr1_4958	p2	(GA)6	12	28384965	28384976
chr1_4959	p2	(TG)7	14	28387341	28387354
chr1_4960	p3	(GGA)9	27	28387761	28387787
chr1_4961	p3	(TCC)5	15	28391742	28391756
chr1_4962	p2	(AG)7	14	28392511	28392524
chr1_4963	c	ctttgtcttgtgcattgtttcctcaggagcccttcccttcgtatctggagatttac	117	28395183	28395299
chr1_4964	p2	(CA)8	16	28400266	28400281
chr1_4965	p2	(TG)12	24	28402657	28402680
chr1_4966	p2	(AC)10	20	28403375	28403394
chr1_4967	p2	(GT)6	12	28403778	28403789
chr1_4968	c	5ttccccctctgcctaaactcacccctccatctcaccccccaagtctcgc(A	76	28404074	28404149
chr1_4969	p2	(CA)8	16	28404713	28404728
chr1_4970	p2	(AC)17	34	28412361	28412394
chr1_4971	p2	(GT)6	12	28416319	28416330
chr1_4972	c	(TA)7(CA)18	50	28417845	28417894
chr1_4973	p2	(AC)6	12	28429701	28429712
chr1_4974	p2	(AC)7	14	28432419	28432432
chr1_4975	p2	(TC)6	12	28440065	28440076
chr1_4976	p2	(AC)8	16	28441347	28441362
chr1_4977	p2	(AC)10	20	28443151	28443170
chr1_4978	p2	(GT)19	38	28443688	28443725
chr1_4979	p2	(CA)11	22	28447012	28447033
chr1_4980	p2	(CA)11	22	28447310	28447331
chr1_4981	p2	(GT)6	12	28447590	28447601
chr1_4982	p2	(CA)11	22	28448672	28448693
chr1_4983	p2	(CA)7	14	28448901	28448914
chr1_4984	p2	(AC)16	32	28451191	28451222
chr1_4985	p2	(TG)13	26	28452074	28452099
chr1_4986	p2	(AC)7	14	28453480	28453493
chr1_4987	p2	(TG)7	14	28453806	28453819
chr1_4988	p2	(AC)6	12	28457614	28457625

chr1_4989	p2	(AG)19	38	28463812	28463849
chr1_4990	p2	(AC)6	12	28476463	28476474
chr1_4991	p5	(ATATT)7	35	28488179	28488213
chr1_4992	p2	(AT)6	12	28489296	28489307
chr1_4993	p2	(AC)8	16	28491951	28491966
chr1_4994	p3	(GAG)5	15	28494704	28494718
chr1_4995	c	[illegible])13tttctctaaatgtgcgtgtgttgtccggtggagcagagagatagg(G[illegible]	95	28502908	28503002
chr1_4996	p2	(TA)7	14	28507455	28507468
chr1_4997	p3	(TTA)7	21	28512404	28512424
chr1_4998	p2	(CA)7	14	28522784	28522797
chr1_4999	p3	(ATA)6	18	28530881	28530898
chr1_5000	p2	(AC)12	24	28548070	28548093
chr1_5001	p2	(AG)9	18	28559431	28559448
chr1_5002	p2	(AC)7	14	28587425	28587438
chr1_5003	p3	(AAC)5	15	28588799	28588813
chr1_5004	p2	(AT)7	14	28598693	28598706
chr1_5005	p2	(AC)6	12	28620597	28620608
chr1_5006	p2	(CT)7	14	28625471	28625484
chr1_5007	c	(AT)7gaaacatgcacatgtccaccaca(CT)7	51	28632086	28632136
chr1_5008	p2	(TG)6	12	28635132	28635143
chr1_5009	p2	(AC)22	44	28641501	28641544
chr1_5010	p2	(GT)17	34	28643931	28643964
chr1_5011	c	[illegible]C)7aaacaagatgaaatgcacacacatatgtgggggagagcgtg(CA)[illegible]	97	28644374	28644470
chr1_5012	p3	(ATG)5	15	28647914	28647928
chr1_5013	p2	(CA)6	12	28648584	28648595
chr1_5014	p2	(CA)13	26	28652985	28653010
chr1_5015	c	(AC)15(AT)7acacatactg(TA)7	68	28671604	28671671
chr1_5016	p2	(TG)9	18	28691395	28691412
chr1_5017	p2	(CA)6	12	28707257	28707268
chr1_5018	p2	(CT)6	12	28722930	28722941
chr1_5019	p2	(CA)16	32	28726851	28726882
chr1_5020	p2	(GT)10	20	28733345	28733364
chr1_5021	p2	(GT)12	24	28737732	28737755
chr1_5022	p2	(GT)6	12	28742760	28742771
chr1_5023	p2	(AC)8	16	28743961	28743976
chr1_5024	p2	(GT)7	14	28758499	28758512
chr1_5025	p2	(TG)10	20	28763553	28763572
chr1_5026	c	(AC)15agacacagagca(AG)12	66	28764750	28764815
chr1_5027	c	(AC)9c(CA)9	37	28766975	28767011
chr1_5028	c	[illegible]gtgttagaaatgattgtggactgaagtaaggcttgtgagagtgttactgcag[illegible]	196	28767714	28767909
chr1_5029	p3	(CAT)5	15	28775143	28775157
chr1_5030	c	(GA)12c(AG)7	39	28778093	28778131
chr1_5031	p2	(CA)8	16	28779708	28779723
chr1_5032	p2	(AT)6	12	28780901	28780912
chr1_5033	p2	(AG)16	32	28787643	28787674
chr1_5034	p2	(AC)23	46	28794311	28794356
chr1_5035	p3	(CAA)5	15	28794717	28794731
chr1_5036	p2	(GT)12	24	28795997	28796020
chr1_5037	p2	(TG)6	12	28796697	28796708
chr1_5038	p2	(AC)14	28	28820183	28820210
chr1_5039	p2	(TG)6	12	28822890	28822901
chr1_5040	p2	(CA)12	24	28830220	28830243

chr1_5041	p2	(CA)6	12	28833528	28833539
chr1_5042	p2	(CA)10	20	28840220	28840239
chr1_5043	p2	(GT)15	30	28867021	28867050
chr1_5044	p2	(AC)6	12	28881894	28881905
chr1_5045	p2	(TG)7	14	28883323	28883336
chr1_5046	p2	(TG)23	46	28885530	28885575
chr1_5047	p2	(AT)6	12	28887932	28887943
chr1_5048	p2	(CA)14	28	28890660	28890687
chr1_5049	c	(CT)6(CA)11	34	28891243	28891276
chr1_5050	p3	(GCT)8	24	28893916	28893939
chr1_5051	p2	(CA)10	20	28906233	28906252
chr1_5052	p2	(TC)8	16	28912415	28912430
chr1_5053	p2	(CA)27	54	28931300	28931353
chr1_5054	p5	(CATAA)11	55	28943653	28943707
chr1_5055	p2	(GT)15	30	28949761	28949790
chr1_5056	p4	(GATA)11	44	28952015	28952058
chr1_5057	p3	(ATT)6	18	28975366	28975383
chr1_5058	c	(TAT)5tgc(TGT)6	36	28976331	28976366
chr1_5059	p2	(CT)7	14	28983681	28983694
chr1_5060	p2	(AC)7	14	28989250	28989263
chr1_5061	p5	(TTATC)5	25	28994578	28994602
chr1_5062	p5	(TTTGA)5	25	28996000	28996024
chr1_5063	p2	(GA)10	20	29009303	29009322
chr1_5064	p3	(GAG)5	15	29013446	29013460
chr1_5065	p2	(CA)10	20	29015913	29015932
chr1_5066	p4	(TTTA)5	20	29020564	29020583
chr1_5067	p4	(TTCT)6	24	29028859	29028882
chr1_5068	c	gtcaggagaatacatct(AC)17aatctccctctgcagcaggcagtgacaa	197	29029878	29030074
chr1_5069	p2	(TG)6	12	29056192	29056203
chr1_5070	p3	(CTG)6	18	29067436	29067453
chr1_5071	p2	(AC)8	16	29082845	29082860
chr1_5072	p2	(TG)6	12	29106450	29106461
chr1_5073	p2	(TG)6	12	29107307	29107318
chr1_5074	p3	(ATG)5	15	29108070	29108084
chr1_5075	p2	(TG)6	12	29110573	29110584
chr1_5076	p2	(GT)11	22	29114537	29114558
chr1_5077	p2	(AT)10	20	29115339	29115358
chr1_5078	p2	(TG)6	12	29116086	29116097
chr1_5079	p2	(AT)8	16	29131530	29131545
chr1_5080	p2	(TG)40	80	29133549	29133628
chr1_5081	p2	(AC)10	20	29135783	29135802
chr1_5082	p3	(GTT)5	15	29138286	29138300
chr1_5083	p2	(AC)25	50	29144969	29145018
chr1_5084	p2	(TC)7	14	29147079	29147092
chr1_5085	p2	(GA)7	14	29150969	29150982
chr1_5086	c	(AC)12at(AC)10	46	29162620	29162665
chr1_5087	p2	(GA)8	16	29167718	29167733
chr1_5088	p2	(TG)8	16	29168656	29168671
chr1_5089	c	(GT)9(GA)9	36	29168847	29168882
chr1_5090	p2	(AC)6	12	29180980	29180991
chr1_5091	c	(TC)11tatac(AT)10	47	29189508	29189554
chr1_5092	p2	(GA)6	12	29197182	29197193

chr1_5093	p2	(GT)9	18	29209739	29209756
chr1_5094	p2	(GT)17	34	29210105	29210138
chr1_5095	p4	(TAGG)5	20	29216212	29216231
chr1_5096	p2	(TG)19	38	29220769	29220806
chr1_5097	p2	(TG)7	14	29232905	29232918
chr1_5098	p2	(TG)6	12	29233509	29233520
chr1_5099	p2	(GT)17	34	29234513	29234546
chr1_5100	p2	(AC)6	12	29250859	29250870
chr1_5101	p2	(AC)6	12	29272346	29272357
chr1_5102	p2	(GT)23	46	29275619	29275664
chr1_5103	p2	(GT)6	12	29285041	29285052
chr1_5104	p2	(TG)15	30	29288520	29288549
chr1_5105	p3	(CAT)5	15	29295144	29295158
chr1_5106	p5	(AAATC)5	25	29299359	29299383
chr1_5107	p3	(TGA)6	18	29305071	29305088
chr1_5108	p2	(AT)9	18	29322156	29322173
chr1_5109	p2	(CA)6	12	29327382	29327393
chr1_5110	p4	(AAAT)5	20	29332576	29332595
chr1_5111	p2	(AT)6	12	29341450	29341461
chr1_5112	p2	(AC)6	12	29341712	29341723
chr1_5113	p2	(AC)6	12	29343190	29343201
chr1_5114	p3	(AAT)8	24	29348964	29348987
chr1_5115	p2	(GT)10	20	29361300	29361319
chr1_5116	p2	(CA)10	20	29394160	29394179
chr1_5117	p2	(AC)9	18	29396696	29396713
chr1_5118	p3	(GAA)8	24	29401387	29401410
chr1_5119	p2	(CA)7	14	29409013	29409026
chr1_5120	p2	(CA)23	46	29410883	29410928
chr1_5121	p2	(GT)10	20	29413358	29413377
chr1_5122	p2	(AC)6	12	29418572	29418583
chr1_5123	p2	(AG)24	48	29450596	29450643
chr1_5124	p2	(GT)6	12	29451146	29451157
chr1_5125	c	(CA)10c(GT)7	35	29452546	29452580
chr1_5126	p2	(CA)20	40	29453511	29453550
chr1_5127	p3	(ATT)5	15	29462732	29462746
chr1_5128	p2	(AC)7	14	29468275	29468288
chr1_5129	p2	(GT)8	16	29469078	29469093
chr1_5130	p2	(AC)21	42	29472711	29472752
chr1_5131	p2	(TG)11	22	29481227	29481248
chr1_5132	p2	(AT)8	16	29490008	29490023
chr1_5133	p2	(TG)6	12	29502873	29502884
chr1_5134	p2	(TG)15	30	29508835	29508864
chr1_5135	c	(AC)16agacacacaagatacatct(CA)8	67	29510086	29510152
chr1_5136	p2	(CA)24	48	29512780	29512827
chr1_5137	p4	(TTTG)5	20	29514459	29514478
chr1_5138	p2	(AC)6	12	29521378	29521389
chr1_5139	p2	(CA)45	90	29524060	29524149
chr1_5140	p2	(CA)6	12	29525970	29525981
chr1_5141	p2	(TA)11	22	29537231	29537252
chr1_5142	p2	(TG)8	16	29560392	29560407
chr1_5143	p4	(ATCT)12	48	29565295	29565342
chr1_5144	p2	(GA)6	12	29578087	29578098

chr1_5145	p2	(CA)6	12	29584785	29584796
chr1_5146	p2	(TG)6	12	29589675	29589686
chr1_5147	p2	(TG)7	14	29596062	29596075
chr1_5148	p2	(TG)8	16	29613258	29613273
chr1_5149	p3	(TTG)5	15	29615376	29615390
chr1_5150	p3	(GTG)6	18	29635638	29635655
chr1_5151	p2	(TC)6	12	29638950	29638961
chr1_5152	p2	(GT)37	74	29658274	29658347
chr1_5153	p3	(CAG)6	18	29674257	29674274
chr1_5154	p3	(AAC)5	15	29681336	29681350
chr1_5155	p2	(CA)20	40	29689002	29689041
chr1_5156	p2	(TA)6	12	29693093	29693104
chr1_5157	p2	(GT)7	14	29701205	29701218
chr1_5158	p2	(AC)10	20	29706480	29706499
chr1_5159	p2	(TG)8	16	29707757	29707772
chr1_5160	p2	(TG)7	14	29711278	29711291
chr1_5161	p2	(AC)8	16	29715881	29715896
chr1_5162	p2	(AC)6	12	29719265	29719276
chr1_5163	p3	(TTA)5	15	29739824	29739838
chr1_5164	p2	(CT)9	18	29740903	29740920
chr1_5165	p2	(CA)14	28	29743621	29743648
chr1_5166	p2	(GT)6	12	29743853	29743864
chr1_5167	p3	(ATA)5	15	29766937	29766951
chr1_5168	p2	(CA)9	18	29772115	29772132
chr1_5169	p4	(TCCA)5	20	29784430	29784449
chr1_5170	p2	(TA)7	14	29793346	29793359
chr1_5171	p2	(CA)6	12	29797692	29797703
chr1_5172	p2	(CA)11	22	29800587	29800608
chr1_5173	p2	(AC)9	18	29802407	29802424
chr1_5174	p2	(AG)6	12	29805882	29805893
chr1_5175	p2	(CA)8	16	29810275	29810290
chr1_5176	p4	(CAGA)5	20	29833400	29833419
chr1_5177	p2	(TG)9	18	29835616	29835633
chr1_5178	p2	(CA)12	24	29837217	29837240
chr1_5179	p4	(GGAA)6	24	29844027	29844050
chr1_5180	p2	(TG)13	26	29847626	29847651
chr1_5181	p2	(AC)8	16	29848996	29849011
chr1_5182	p2	(AC)16	32	29851536	29851567
chr1_5183	p3	(ATA)5	15	29868276	29868290
chr1_5184	p2	(CA)13	26	29872243	29872268
chr1_5185	p2	(TG)16	32	29877789	29877820
chr1_5186	c	(TAT)5tgccagtatgttttt(TTG)5	45	29882603	29882647
chr1_5187	p2	(TG)6	12	29884161	29884172
chr1_5188	p2	(TG)6	12	29891255	29891266
chr1_5189	p3	(TTG)5	15	29900938	29900952
chr1_5190	p2	(CA)14	28	29902478	29902505
chr1_5191	p2	(TG)19	38	29908478	29908515
chr1_5192	p2	(TA)6	12	29915100	29915111
chr1_5193	p3	(CAC)5	15	29920808	29920822
chr1_5194	p2	(TG)7	14	29923501	29923514
chr1_5195	p2	(TG)6	12	29926135	29926146
chr1_5196	p2	(GA)10	20	29927400	29927419

chr1_5197	p2	(GA)7	14	29932899	29932912
chr1_5198	p2	(AG)8	16	29951099	29951114
chr1_5199	p3	(AAT)10	30	29955500	29955529
chr1_5200	p3	(CCA)5	15	29956113	29956127
chr1_5201	p2	(GT)6	12	29962168	29962179
chr1_5202	p2	(AC)7	14	29964938	29964951
chr1_5203	p2	(AC)9	18	29965814	29965831
chr1_5204	p2	(AC)10	20	29967275	29967294
chr1_5205	p2	(CA)7	14	29974751	29974764
chr1_5206	p2	(AC)13	26	29977358	29977383
chr1_5207	p4	(ATGT)5	20	29995046	29995065
chr1_5208	p2	(GT)6	12	30001119	30001130
chr1_5209	p2	(TG)6	12	30002811	30002822
chr1_5210	p2	(TG)6	12	30033582	30033593
chr1_5211	p2	(AG)6	12	30034443	30034454
chr1_5212	p2	(CA)12	24	30034998	30035021
chr1_5213	p2	(AC)8	16	30035672	30035687
chr1_5214	p2	(TG)26	52	30039811	30039862
chr1_5215	p4	(CAAA)5	20	30040462	30040481
chr1_5216	p2	(TG)9	18	30044288	30044305
chr1_5217	c	(GA)7(GT)11	36	30050703	30050738
chr1_5218	p3	(TAA)8	24	30054553	30054576
chr1_5219	p4	(CTAT)20	80	30057089	30057168
chr1_5220	p2	(GT)6	12	30059563	30059574
chr1_5221	p2	(CA)16	32	30060627	30060658
chr1_5222	p3	(GTT)7	21	30070141	30070161
chr1_5223	p2	(CA)6	12	30082375	30082386
chr1_5224	p2	(GT)13	26	30086791	30086816
chr1_5225	p2	(TC)8	16	30088198	30088213
chr1_5226	p2	(GA)6	12	30090673	30090684
chr1_5227	p2	(TG)10	20	30091383	30091402
chr1_5228	p2	(AC)6	12	30095421	30095432
chr1_5229	c	(GT)17tc(GA)10	56	30131610	30131665
chr1_5230	p2	(TA)6	12	30146710	30146721
chr1_5231	p2	(CA)6	12	30173649	30173660
chr1_5232	p3	(GGT)5	15	30182346	30182360
chr1_5233	p2	(AC)6	12	30187744	30187755
chr1_5234	p2	(TG)7	14	30197665	30197678
chr1_5235	p2	(TG)6	12	30198662	30198673
chr1_5236	c	(GT)12(GA)8	40	30203652	30203691
chr1_5237	p2	(AC)14	28	30204895	30204922
chr1_5238	p2	(GT)16	32	30207626	30207657
chr1_5239	p2	(GT)9	18	30208489	30208506
chr1_5240	p2	(GA)7	14	30212804	30212817
chr1_5241	p2	(TG)6	12	30224689	30224700
chr1_5242	p2	(CA)8	16	30227282	30227297
chr1_5243	p2	(AC)11	22	30241203	30241224
chr1_5244	p2	(AC)7	14	30242010	30242023
chr1_5245	p2	(AG)24	48	30248197	30248244
chr1_5246	p3	(GAG)5	15	30256978	30256992
chr1_5247	p2	(GT)7	14	30261725	30261738
chr1_5248	p2	(CA)15	30	30263435	30263464

chr1_5249	p2	(TG)6	12	30263664	30263675
chr1_5250	p2	(TG)15	30	30265639	30265668
chr1_5251	p2	(TC)10	20	30267939	30267958
chr1_5252	p2	(CA)7	14	30271321	30271334
chr1_5253	p2	(CA)13	26	30275631	30275656
chr1_5254	p2	(GT)18	36	30276856	30276891
chr1_5255	p2	(TG)18	36	30281898	30281933
chr1_5256	p2	(AC)29	58	30285364	30285421
chr1_5257	p2	(TG)8	16	30287405	30287420
chr1_5258	p2	(CA)8	16	30290556	30290571
chr1_5259	p2	(AC)16	32	30298084	30298115
chr1_5260	p2	(CT)6	12	30300443	30300454
chr1_5261	p3	(TAA)7	21	30314637	30314657
chr1_5262	p3	(CTG)7	21	30321408	30321428
chr1_5263	p2	(CA)8	16	30328499	30328514
chr1_5264	p2	(CA)6	12	30329396	30329407
chr1_5265	p2	(AC)7	14	30331654	30331667
chr1_5266	p2	(AC)16	32	30331805	30331836
chr1_5267	p2	(TC)7	14	30332006	30332019
chr1_5268	p2	(GT)9	18	30334456	30334473
chr1_5269	p2	(AG)7	14	30336236	30336249
chr1_5270	p2	(CA)9	18	30336482	30336499
chr1_5271	p2	(CA)9	18	30337671	30337688
chr1_5272	p2	(GT)6	12	30337800	30337811
chr1_5273	p2	(AC)6	12	30340715	30340726
chr1_5274	p2	(CT)10	20	30348031	30348050
chr1_5275	p2	(TC)7	14	30349320	30349333
chr1_5276	p2	(AC)6	12	30350508	30350519
chr1_5277	p2	(CA)12	24	30353372	30353395
chr1_5278	p2	(TG)8	16	30359925	30359940
chr1_5279	p2	(TG)8	16	30360751	30360766
chr1_5280	c	(TC)12(AC)17	58	30363988	30364045
chr1_5281	p2	(TG)12	24	30370073	30370096
chr1_5282	p2	(TG)7	14	30373230	30373243
chr1_5283	p2	(AC)14	28	30374028	30374055
chr1_5284	p2	(AC)8	16	30374892	30374907
chr1_5285	p3	(TAC)13	39	30378038	30378076
chr1_5286	c	(GT)12(GC)6	36	30385096	30385131
chr1_5287	p2	(GT)7	14	30386719	30386732
chr1_5288	p2	(AC)12	24	30395834	30395857
chr1_5289	p2	(AG)7	14	30401417	30401430
chr1_5290	p2	(GT)8	16	30401991	30402006
chr1_5291	p2	(AC)15	30	30404447	30404476
chr1_5292	p3	(TAA)8	24	30406305	30406328
chr1_5293	p2	(GT)13	26	30417070	30417095
chr1_5294	p2	(GA)10	20	30421977	30421996
chr1_5295	p2	(GT)9	18	30422382	30422399
chr1_5296	p2	(CA)19	38	30437068	30437105
chr1_5297	p2	(TG)9	18	30439712	30439729
chr1_5298	c	(CTG)5gcaaaaacataagggtgtaatacacag(CA)13	68	30446979	30447046
chr1_5299	p3	(TCA)6	18	30448682	30448699
chr1_5300	c	(TG)10g(GT)6	33	30448877	30448909

chr1_5301	p2	(TG)6	12	30455003	30455014
chr1_5302	p2	(AC)7	14	30455606	30455619
chr1_5303	p2	(GT)20	40	30463840	30463879
chr1_5304	p2	(TG)9	18	30464137	30464154
chr1_5305	p4	(ATAG)5	20	30465805	30465824
chr1_5306	p2	(GA)8	16	30466764	30466779
chr1_5307	p2	(AG)6	12	30467235	30467246
chr1_5308	p2	(CA)26	52	30468757	30468808
chr1_5309	p2	(CA)14	28	30470448	30470475
chr1_5310	p3	(GCA)5	15	30482236	30482250
chr1_5311	p2	(GT)8	16	30482399	30482414
chr1_5312	c	(TC)6acacacacacat(AC)13	50	30484420	30484469
chr1_5313	p2	(CA)7	14	30486186	30486199
chr1_5314	p2	(TG)10	20	30490278	30490297
chr1_5315	p2	(TC)7	14	30497632	30497645
chr1_5316	p2	(CT)12	24	30497950	30497973
chr1_5317	p2	(TC)12	24	30518753	30518776
chr1_5318	p2	(TC)7	14	30519455	30519468
chr1_5319	p3	(GAG)7	21	30528420	30528440
chr1_5320	p2	(TG)18	36	30539143	30539178
chr1_5321	p2	(TG)6	12	30553032	30553043
chr1_5322	p2	(CT)6	12	30561966	30561977
chr1_5323	p2	(AG)13	26	30574788	30574813
chr1_5324	p2	(GA)7	14	30577081	30577094
chr1_5325	p2	(CA)6	12	30585250	30585261
chr1_5326	p2	(TG)8	16	30585407	30585422
chr1_5327	p2	(CA)19	38	30585868	30585905
chr1_5328	p2	(TG)8	16	30589177	30589192
chr1_5329	p2	(TC)7	14	30591571	30591584
chr1_5330	p2	(GT)13	26	30616348	30616373
chr1_5331	p4	(ATAG)5	20	30625050	30625069
chr1_5332	p3	(GAA)5	15	30636288	30636302
chr1_5333	p2	(GT)8	16	30640816	30640831
chr1_5334	p2	(AC)6	12	30642905	30642916
chr1_5335	p2	(GT)36	72	30648185	30648256
chr1_5336	p2	(AG)7	14	30656730	30656743
chr1_5337	p2	(TG)6	12	30658074	30658085
chr1_5338	p3	(TGC)5	15	30666417	30666431
chr1_5339	c	(CT)12(CA)7ct(CA)14	68	30685620	30685687
chr1_5340	p2	(AC)11	22	30692549	30692570
chr1_5341	p2	(TG)6	12	30696678	30696689
chr1_5342	p2	(CA)10	20	30697226	30697245
chr1_5343	p3	(CAT)7	21	30700077	30700097
chr1_5344	p2	(AC)6	12	30700901	30700912
chr1_5345	p2	(AC)6	12	30702103	30702114
chr1_5346	p2	(TG)10	20	30710776	30710795
chr1_5347	p2	(TC)6	12	30723302	30723313
chr1_5348	p4	(TATC)5	20	30724112	30724131
chr1_5349	p2	(AC)14	28	30728422	30728449
chr1_5350	p2	(CT)8	16	30729993	30730008
chr1_5351	p3	(CTG)6	18	30737066	30737083
chr1_5352	p2	(CA)6	12	30737948	30737959

chr1_5353	p4	(CTAT)21	84	30740587	30740670
chr1_5354	p2	(AC)10	20	30741500	30741519
chr1_5355	c	(TG)6tt(TG)10	34	30741774	30741807
chr1_5356	p2	(CT)6	12	30742261	30742272
chr1_5357	p2	(AC)6	12	30750796	30750807
chr1_5358	p2	(AG)6	12	30762775	30762786
chr1_5359	p2	(CA)7	14	30767610	30767623
chr1_5360	p2	(AT)7	14	30775633	30775646
chr1_5361	p2	(GT)10	20	30781694	30781713
chr1_5362	p2	(TC)12	24	30791396	30791419
chr1_5363	p2	(CA)7	14	30796118	30796131
chr1_5364	p2	(AC)6	12	30799496	30799507
chr1_5365	p2	(AC)8	16	30825726	30825741
chr1_5366	p2	(AG)6	12	30835151	30835162
chr1_5367	p2	(TG)14	28	30844470	30844497
chr1_5368	p2	(GT)7	14	30845449	30845462
chr1_5369	p2	(AC)6	12	30848295	30848306
chr1_5370	p2	(AC)12	24	30863185	30863208
chr1_5371	p2	(GT)15	30	30866695	30866724
chr1_5372	p2	(CA)6	12	30876494	30876505
chr1_5373	p2	(CT)6	12	30878301	30878312
chr1_5374	p5	(AAATC)5	25	30878669	30878693
chr1_5375	p2	(CA)14	28	30887214	30887241
chr1_5376	p2	(CA)6	12	30888546	30888557
chr1_5377	p2	(AC)6	12	30889433	30889444
chr1_5378	p2	(AC)13	26	30890500	30890525
chr1_5379	p2	(AC)9	18	30911163	30911180
chr1_5380	p2	(CA)6	12	30912133	30912144
chr1_5381	p6	(AGGTAC)16	96	30913240	30913335
chr1_5382	p6	(AGGTAC)16	96	30915239	30915334
chr1_5383	p6	(AGGTAC)16	96	30915675	30915770
chr1_5384	p2	(AC)7	14	30927934	30927947
chr1_5385	p2	(AC)9	18	30958383	30958400
chr1_5386	p2	(TG)7	14	30969063	30969076
chr1_5387	p2	(TA)9	18	30971440	30971457
chr1_5388	p2	(TG)7	14	30975173	30975186
chr1_5389	p2	(TA)10	20	30977547	30977566
chr1_5390	p2	(TG)28	56	30985427	30985482
chr1_5391	p3	(GTT)6	18	30986826	30986843
chr1_5392	p2	(TG)12	24	30987435	30987458
chr1_5393	p2	(AC)14	28	30993991	30994018
chr1_5394	p2	(TC)6	12	30999253	30999264
chr1_5395	p2	(AC)20	40	31009234	31009273
chr1_5396	p2	(AC)20	40	31011808	31011847
chr1_5397	c	(AG)8ac(AG)12	42	31013170	31013211
chr1_5398	p2	(AC)15	30	31016267	31016296
chr1_5399	p2	(GT)6	12	31017241	31017252
chr1_5400	p3	(ATT)8	24	31019988	31020011
chr1_5401	p2	(TG)12	24	31020196	31020219
chr1_5402	p2	(AC)15	30	31020353	31020382
chr1_5403	p3	(TCT)6	18	31046521	31046538
chr1_5404	p2	(GA)7	14	31050768	31050781

chr1_5405	p2	(CT)6	12	31091821	31091832
chr1_5406	p2	(GA)6	12	31092371	31092382
chr1_5407	p2	(AT)6	12	31108634	31108645
chr1_5408	p2	(GT)8	16	31111366	31111381
chr1_5409	p3	(ATA)8	24	31113352	31113375
chr1_5410	p2	(CA)13	26	31120240	31120265
chr1_5411	p2	(GT)8	16	31122093	31122108
chr1_5412	p3	(TAA)5	15	31124434	31124448
chr1_5413	p2	(CA)9	18	31133547	31133564
chr1_5414	p2	(CA)8	16	31143908	31143923
chr1_5415	p2	(TA)8	16	31144794	31144809
chr1_5416	c	(GT)10gggctgtgtgcatagtggggtaa(GT)7	57	31150279	31150335
chr1_5417	p2	(TG)10	20	31150632	31150651
chr1_5418	p2	(CA)20	40	31153618	31153657
chr1_5419	p3	(AGC)5	15	31159088	31159102
chr1_5420	p2	(TG)6	12	31178433	31178444
chr1_5421	p2	(GT)13	26	31181376	31181401
chr1_5422	p2	(TC)13	26	31199840	31199865
chr1_5423	p2	(GT)12	24	31201822	31201845
chr1_5424	p2	(CA)7	14	31203512	31203525
chr1_5425	p2	(AC)6	12	31209639	31209650
chr1_5426	p2	(TG)17	34	31209825	31209858
chr1_5427	p2	(TG)6	12	31215229	31215240
chr1_5428	p3	(ATA)5	15	31215349	31215363
chr1_5429	p2	(GT)10	20	31215947	31215966
chr1_5430	p2	(AC)15	30	31216414	31216443
chr1_5431	p2	(GA)10	20	31230348	31230367
chr1_5432	p2	(CA)7	14	31235871	31235884
chr1_5433	p2	(CA)7	14	31237650	31237663
chr1_5434	p2	(TG)14	28	31241672	31241699
chr1_5435	p3	(CTC)5	15	31244116	31244130
chr1_5436	p2	(TG)9	18	31246723	31246740
chr1_5437	p2	(TG)6	12	31251389	31251400
chr1_5438	p2	(TG)11	22	31254771	31254792
chr1_5439	p2	(AG)7	14	31278495	31278508
chr1_5440	p2	(AC)10	20	31285216	31285235
chr1_5441	p2	(AC)8	16	31285400	31285415
chr1_5442	p2	(CA)6	12	31290449	31290460
chr1_5443	p2	(AC)6	12	31292454	31292465
chr1_5444	p2	(CA)6	12	31292774	31292785
chr1_5445	p2	(AC)16	32	31295648	31295679
chr1_5446	p2	(TG)6	12	31311023	31311034
chr1_5447	c	gacacgtcgaaagacacattcacacagtcacacacattctcactgtcctgtt	79	31312060	31312138
chr1_5448	p2	(CA)8	16	31316065	31316080
chr1_5449	c	(AG)6g(GA)11	35	31319138	31319172
chr1_5450	p2	(TG)19	38	31319719	31319756
chr1_5451	p2	(TG)16	32	31320582	31320613
chr1_5452	p3	(AGC)5	15	31322827	31322841
chr1_5453	p2	(CA)7	14	31327473	31327486
chr1_5454	p3	(TTG)6	18	31332430	31332447
chr1_5455	p2	(AG)7	14	31332798	31332811
chr1_5456	p2	(TC)12	24	31341117	31341140

chr1_5457	p3	(TAT)5	15	31351480	31351494
chr1_5458	p2	(TC)7	14	31354966	31354979
chr1_5459	p2	(TG)7	14	31357964	31357977
chr1_5460	p2	(TG)17	34	31376256	31376289
chr1_5461	p3	(CCA)8	24	31382752	31382775
chr1_5462	p3	(ATT)7	21	31384182	31384202
chr1_5463	p2	(CA)11	22	31415922	31415943
chr1_5464	p2	(GT)9	18	31433164	31433181
chr1_5465	c	gctgatacacaggttttgcatttatttgcctgaattagtttagccaattatggttt	128	31434618	31434745
chr1_5466	p4	(TATT)6	24	31449435	31449458
chr1_5467	p2	(TA)6	12	31468619	31468630
chr1_5468	p2	(AC)6	12	31483637	31483648
chr1_5469	p2	(TC)8	16	31486903	31486918
chr1_5470	p2	(GT)6	12	31488918	31488929
chr1_5471	p2	(GT)15	30	31489046	31489075
chr1_5472	p2	(TG)16	32	31495659	31495690
chr1_5473	p2	(TG)17	34	31497920	31497953
chr1_5474	p2	(GT)14	28	31498390	31498417
chr1_5475	p2	(TC)8	16	31499640	31499655
chr1_5476	p2	(AG)6	12	31500857	31500868
chr1_5477	p2	(CA)18	36	31501755	31501790
chr1_5478	p2	(AC)10	20	31510136	31510155
chr1_5479	p2	(CA)6	12	31512239	31512250
chr1_5480	c	(TC)6(AC)6	24	31516672	31516695
chr1_5481	p2	(GT)24	48	31533055	31533102
chr1_5482	p2	(TG)8	16	31536812	31536827
chr1_5483	p2	(AT)10	20	31538966	31538985
chr1_5484	p2	(TG)17	34	31546412	31546445
chr1_5485	p2	(AC)10	20	31550200	31550219
chr1_5486	p4	(TTAT)6	24	31552162	31552185
chr1_5487	p2	(TC)7	14	31563858	31563871
chr1_5488	p2	(AC)6	12	31566209	31566220
chr1_5489	p2	(AG)6	12	31571801	31571812
chr1_5490	p2	(TG)10	20	31580904	31580923
chr1_5491	p2	(TC)9	18	31587271	31587288
chr1_5492	p3	(TTA)5	15	31589852	31589866
chr1_5493	p2	(CT)6	12	31598208	31598219
chr1_5494	p2	(AC)6	12	31600550	31600561
chr1_5495	p2	(TG)7	14	31614357	31614370
chr1_5496	p2	(GA)7	14	31619522	31619535
chr1_5497	p2	(TC)10	20	31624053	31624072
chr1_5498	c	(AG)6(TG)7(AG)7	40	31624984	31625023
chr1_5499	p2	(AC)6	12	31625174	31625185
chr1_5500	p3	(TCG)7	21	31632887	31632907
chr1_5501	p3	(CTC)9	27	31636228	31636254
chr1_5502	p2	(TC)16	32	31640607	31640638
chr1_5503	p2	(AG)6	12	31642282	31642293
chr1_5504	p2	(AC)17	34	31644089	31644122
chr1_5505	p4	(TCAT)5	20	31646710	31646729
chr1_5506	p2	(CA)7	14	31648114	31648127
chr1_5507	c	agctgtgaagaagctgaggcaaaaagaaagtttttttgactgacattttatag	82	31655891	31655972
chr1_5508	p2	(GT)8	16	31661190	31661205

chr1_5509	p2	(AT)7	14	31665624	31665637
chr1_5510	p2	(TG)6	12	31665905	31665916
chr1_5511	p2	(TG)13	26	31666609	31666634
chr1_5512	p2	(CA)7	14	31673809	31673822
chr1_5513	p2	(CA)6	12	31677523	31677534
chr1_5514	p2	(AC)6	12	31680258	31680269
chr1_5515	p2	(TG)16	32	31683266	31683297
chr1_5516	p2	(TG)9	18	31683856	31683873
chr1_5517	p2	(CA)7	14	31685281	31685294
chr1_5518	c	ggttgataatgctaagctaaactaagcagttgtccaattcagcctaaattgtc	142	31686098	31686239
chr1_5519	p2	(AC)6	12	31693188	31693199
chr1_5520	p3	(GAA)7	21	31693744	31693764
chr1_5521	p2	(AC)11	22	31696998	31697019
chr1_5522	p2	(TG)13	26	31702220	31702245
chr1_5523	p2	(AC)6	12	31706526	31706537
chr1_5524	c	(GA)9caggcggactggtgc(GA)6	45	31706873	31706917
chr1_5525	p2	(AC)16	32	31718676	31718707
chr1_5526	p2	(CT)13	26	31722092	31722117
chr1_5527	p2	(TG)12	24	31725126	31725149
chr1_5528	p2	(TC)6	12	31730173	31730184
chr1_5529	p2	(TG)7	14	31747143	31747156
chr1_5530	p2	(TA)7	14	31749327	31749340
chr1_5531	p3	(AGA)8	24	31760913	31760936
chr1_5532	p2	(TG)15	30	31768878	31768907
chr1_5533	p3	(TAA)6	18	31775268	31775285
chr1_5534	p2	(CA)22	44	31779723	31779766
chr1_5535	p2	(AG)8	16	31779937	31779952
chr1_5536	p2	(GA)8	16	31794189	31794204
chr1_5537	p2	(TG)6	12	31795376	31795387
chr1_5538	p2	(GT)32	64	31797010	31797073
chr1_5539	p2	(TG)19	38	31807693	31807730
chr1_5540	p2	(GT)15	30	31808588	31808617
chr1_5541	p3	(GGA)10	30	31812002	31812031
chr1_5542	c	(TG)8ag(GA)6	30	31824129	31824158
chr1_5543	p2	(TC)6	12	31828348	31828359
chr1_5544	p2	(AT)7	14	31829791	31829804
chr1_5545	p2	(GT)13	26	31842925	31842950
chr1_5546	p2	(TG)6	12	31843632	31843643
chr1_5547	p2	(TG)9	18	31843763	31843780
chr1_5548	c	agccatggcccatttaatcatgtatgaccctttaatgcacacaggtgacattt	102	31844066	31844167
chr1_5549	p2	(GT)12	24	31845161	31845184
chr1_5550	p2	(AG)7	14	31856987	31857000
chr1_5551	p2	(TG)19	38	31858365	31858402
chr1_5552	p2	(TC)9	18	31858812	31858829
chr1_5553	p2	(AC)7	14	31860005	31860018
chr1_5554	p2	(GA)7	14	31861945	31861958
chr1_5555	p2	(TG)7	14	31867939	31867952
chr1_5556	p2	(GT)20	40	31876296	31876335
chr1_5557	p2	(AC)15	30	31879700	31879729
chr1_5558	p3	(GGA)5	15	31879926	31879940
chr1_5559	p2	(TG)11	22	31880155	31880176
chr1_5560	p2	(CA)18	36	31883945	31883980

chr1_5561	p2	(AC)27	54	31884104	31884157
chr1_5562	c	(AG)9c(GA)6gc(GA)6	45	31886390	31886434
chr1_5563	p2	(TG)19	38	31889016	31889053
chr1_5564	p2	(CA)8	16	31890962	31890977
chr1_5565	p2	(AC)12	24	31892617	31892640
chr1_5566	p3	(AAT)6	18	31893453	31893470
chr1_5567	p2	(TG)10	20	31894608	31894627
chr1_5568	p2	(GT)21	42	31905414	31905455
chr1_5569	p2	(CA)26	52	31910427	31910478
chr1_5570	c	(ACAT)7(AC)12	52	31911387	31911438
chr1_5571	p2	(CT)7	14	31911955	31911968
chr1_5572	c	(TC)11(AC)7	36	31912110	31912145
chr1_5573	p2	(AG)13	26	31914211	31914236
chr1_5574	p2	(CA)7	14	31916430	31916443
chr1_5575	p2	(GT)11	22	31925418	31925439
chr1_5576	p2	(TG)12	24	31926283	31926306
chr1_5577	p2	(TA)6	12	31927174	31927185
chr1_5578	p2	(GT)11	22	31928535	31928556
chr1_5579	p2	(TG)18	36	31929400	31929435
chr1_5580	p2	(TA)6	12	31930303	31930314
chr1_5581	p2	(TC)7	14	31937734	31937747
chr1_5582	p2	(TG)6	12	31938465	31938476
chr1_5583	p2	(CA)9	18	31938731	31938748
chr1_5584	p2	(CA)16	32	31944473	31944504
chr1_5585	p2	(AC)26	52	31947199	31947250
chr1_5586	p2	(AC)6	12	31948887	31948898
chr1_5587	p3	(AAT)5	15	31952736	31952750
chr1_5588	p2	(CA)6	12	31957719	31957730
chr1_5589	p3	(TCA)5	15	31958050	31958064
chr1_5590	p4	(AAAT)5	20	31959976	31959995
chr1_5591	p4	(TATC)13	52	31963808	31963859
chr1_5592	p5	(AAATC)6	30	31967844	31967873
chr1_5593	p2	(GT)10	20	31978597	31978616
chr1_5594	p2	(TG)10	20	31987225	31987244
chr1_5595	p2	(AC)8	16	31989163	31989178
chr1_5596	p2	(TG)8	16	31991761	31991776
chr1_5597	p2	(TG)24	48	31992138	31992185
chr1_5598	p4	(CCTA)8	32	31999242	31999273
chr1_5599	p2	(TG)8	16	32001652	32001667
chr1_5600	p4	(TAGA)5	20	32009795	32009814
chr1_5601	p2	(CA)6	12	32020790	32020801
chr1_5602	p2	(TG)8	16	32023461	32023476
chr1_5603	p2	(CA)17	34	32049091	32049124
chr1_5604	p2	(TA)6	12	32049862	32049873
chr1_5605	p2	(TG)6	12	32050236	32050247
chr1_5606	p2	(AC)21	42	32056494	32056535
chr1_5607	p2	(CA)6	12	32073475	32073486
chr1_5608	p2	(CT)6	12	32084013	32084024
chr1_5609	p4	(AATG)5	20	32087593	32087612
chr1_5610	p2	(TC)6	12	32099253	32099264
chr1_5611	p2	(GT)14	28	32101371	32101398
chr1_5612	p2	(TC)6	12	32111979	32111990

chr1_5613	p2	(TC)6	12	32118625	32118636
chr1_5614	p3	(AAG)6	18	32125668	32125685
chr1_5615	p2	(GT)8	16	32134520	32134535
chr1_5616	p2	(CT)9	18	32137637	32137654
chr1_5617	p2	(CA)6	12	32137988	32137999
chr1_5618	p3	(TAA)6	18	32168702	32168719
chr1_5619	c	(GAT)5tgatgaagaaataggtcaatagcactagac(TG)8	61	32169697	32169757
chr1_5620	p2	(CA)29	58	32198312	32198369
chr1_5621	p2	(CA)12	24	32204027	32204050
chr1_5622	p2	(CA)11	22	32204435	32204456
chr1_5623	p2	(AC)47	94	32209349	32209442
chr1_5624	p2	(AT)7	14	32224265	32224278
chr1_5625	p2	(CA)6	12	32225010	32225021
chr1_5626	p2	(GT)9	18	32231676	32231693
chr1_5627	p2	(CA)13	26	32233005	32233030
chr1_5628	p2	(TC)7	14	32234858	32234871
chr1_5629	p2	(CA)14	28	32245806	32245833
chr1_5630	p2	(TC)7	14	32250942	32250955
chr1_5631	p2	(AC)6	12	32256459	32256470
chr1_5632	p2	(CA)7	14	32257070	32257083
chr1_5633	p3	(AAG)5	15	32257517	32257531
chr1_5634	p2	(GT)8	16	32258312	32258327
chr1_5635	c	8caaggtgaaaccagtgaggaggatggaaactttgtagtactatttaag(T	92	32260273	32260364
chr1_5636	p2	(AG)7	14	32264011	32264024
chr1_5637	c*	(TATG)5(TG)10*	38	32264291	32264328
chr1_5638	p2	(CA)7	14	32266018	32266031
chr1_5639	p2	(AG)12	24	32266202	32266225
chr1_5640	p2	(AT)9	18	32271267	32271284
chr1_5641	p2	(CA)10	20	32276134	32276153
chr1_5642	p3	(CAC)22	66	32311265	32311330
chr1_5643	p2	(GA)6	12	32325216	32325227
chr1_5644	p3	(AGC)5	15	32330687	32330701
chr1_5645	p2	(CT)6	12	32331000	32331011
chr1_5646	p4	(CCCT)6	24	32331978	32332001
chr1_5647	p2	(GA)6	12	32334409	32334420
chr1_5648	c	(GT)11atgtgtg(TGGA)5	49	32335842	32335890
chr1_5649	p2	(TA)11	22	32336802	32336823
chr1_5650	p2	(GT)11	22	32337461	32337482
chr1_5651	c	(GT)19atacctgggttagggat(TG)11	77	32339240	32339316
chr1_5652	p2	(GT)20	40	32341751	32341790
chr1_5653	p2	(AC)21	42	32341913	32341954
chr1_5654	p2	(CA)7	14	32345515	32345528
chr1_5655	p2	(GT)17	34	32350890	32350923
chr1_5656	p4	(CCAT)5	20	32351485	32351504
chr1_5657	p2	(GA)7	14	32363556	32363569
chr1_5658	p3	(CCA)5	15	32373309	32373323
chr1_5659	p2	(CA)13	26	32376170	32376195
chr1_5660	p2	(TG)6	12	32380511	32380522
chr1_5661	p2	(TG)6	12	32388599	32388610
chr1_5662	p2	(TG)6	12	32388913	32388924
chr1_5663	p2	(CA)6	12	32389754	32389765
chr1_5664	p2	(TG)6	12	32392223	32392234

chr1_5665	p3	(ATA)5	15	32392344	32392358
chr1_5666	p2	(TG)12	24	32399295	32399318
chr1_5667	p2	(GT)8	16	32402436	32402451
chr1_5668	p4	(TTTA)6	24	32402575	32402598
chr1_5669	p2	(TG)18	36	32403521	32403556
chr1_5670	p2	(TG)18	36	32410870	32410905
chr1_5671	p2	(CA)16	32	32412385	32412416
chr1_5672	p2	(GT)14	28	32413883	32413910
chr1_5673	p2	(GT)15	30	32416067	32416096
chr1_5674	p2	(TG)12	24	32417884	32417907
chr1_5675	p2	(CA)11	22	32425144	32425165
chr1_5676	p2	(CA)13	26	32427739	32427764
chr1_5677	p2	(AC)9	18	32430851	32430868
chr1_5678	p2	(TG)9	18	32444859	32444876
chr1_5679	p2	(AC)8	16	32453683	32453698
chr1_5680	p2	(AG)6	12	32460912	32460923
chr1_5681	p3	(GTG)6	18	32466753	32466770
chr1_5682	p2	(TG)9	18	32468589	32468606
chr1_5683	p2	(AC)11	22	32470721	32470742
chr1_5684	p2	(TG)7	14	32471229	32471242
chr1_5685	p2	(AT)6	12	32471707	32471718
chr1_5686	p2	(CA)6	12	32475317	32475328
chr1_5687	p2	(AC)6	12	32476018	32476029
chr1_5688	p2	(GT)11	22	32478728	32478749
chr1_5689	p2	(TG)15	30	32483331	32483360
chr1_5690	p2	(TG)13	26	32491537	32491562
chr1_5691	p2	(TG)13	26	32494690	32494715
chr1_5692	p3	(GAA)5	15	32499663	32499677
chr1_5693	p2	(AG)6	12	32504326	32504337
chr1_5694	p3	(GAA)5	15	32506487	32506501
chr1_5695	p3	(TAA)5	15	32510940	32510954
chr1_5696	p2	(GT)20	40	32519712	32519751
chr1_5697	p2	(AC)12	24	32520098	32520121
chr1_5698	p2	(GT)8	16	32537022	32537037
chr1_5699	p2	(TG)9	18	32562260	32562277
chr1_5700	p2	(AC)18	36	32564904	32564939
chr1_5701	p3	(AAT)6	18	32565636	32565653
chr1_5702	p2	(TG)11	22	32567080	32567101
chr1_5703	p2	(CA)15	30	32567298	32567327
chr1_5704	c	(CT)7atctcgctcgctcgccccc(CT)6	45	32585692	32585736
chr1_5705	p2	(TA)8	16	32586178	32586193
chr1_5706	p4	(TATC)15	60	32591917	32591976
chr1_5707	p2	(CA)6	12	32593429	32593440
chr1_5708	p3	(TAA)5	15	32593804	32593818
chr1_5709	p2	(GT)13	26	32599006	32599031
chr1_5710	p2	(TG)12	24	32601623	32601646
chr1_5711	p2	(TG)8	16	32607108	32607123
chr1_5712	p2	(TG)6	12	32608860	32608871
chr1_5713	c	(CA)6(TA)12	36	32609123	32609158
chr1_5714	p2	(TG)9	18	32612521	32612538
chr1_5715	p2	(TG)6	12	32615673	32615684
chr1_5716	p2	(GT)6	12	32631450	32631461

chr1_5717	p4	(AGAC)5	20	32660595	32660614
chr1_5718	p2	(CA)9	18	32678850	32678867
chr1_5719	p2	(GT)6	12	32683454	32683465
chr1_5720	p3	(GTA)6	18	32687006	32687023
chr1_5721	p2	(TG)7	14	32688439	32688452
chr1_5722	p2	(GT)6	12	32689932	32689943
chr1_5723	p2	(AC)15	30	32697915	32697944
chr1_5724	p2	(AC)16	32	32707793	32707824
chr1_5725	p3	(ACA)5	15	32713592	32713606
chr1_5726	p2	(AT)6	12	32715382	32715393
chr1_5727	p2	(CA)8	16	32716770	32716785
chr1_5728	p2	(TG)6	12	32726278	32726289
chr1_5729	p2	(AC)6	12	32726694	32726705
chr1_5730	p2	(TG)10	20	32727736	32727755
chr1_5731	p2	(AC)13	26	32728394	32728419
chr1_5732	p2	(CA)6	12	32731470	32731481
chr1_5733	p2	(GT)7	14	32732822	32732835
chr1_5734	p2	(TG)13	26	32732946	32732971
chr1_5735	p2	(TA)6	12	32734401	32734412
chr1_5736	p2	(TG)10	20	32742607	32742626
chr1_5737	p2	(GT)8	16	32742855	32742870
chr1_5738	p2	(GT)16	32	32743505	32743536
chr1_5739	p2	(CA)6	12	32753807	32753818
chr1_5740	p2	(CT)7	14	32764217	32764230
chr1_5741	p2	(GT)7	14	32766443	32766456
chr1_5742	p2	(AG)9	18	32771448	32771465
chr1_5743	p2	(TA)11	22	32785688	32785709
chr1_5744	p2	(CT)8	16	32791401	32791416
chr1_5745	p2	(CA)11	22	32795139	32795160
chr1_5746	p2	(TG)15	30	32797098	32797127
chr1_5747	p2	(TG)19	38	32797802	32797839
chr1_5748	p2	(CA)6	12	32798702	32798713
chr1_5749	c	tgttacctagacattttgttacacaataacgacagggtaaaacgcgaggcca	88	32800965	32801052
chr1_5750	p3	(TCC)5	15	32803248	32803262
chr1_5751	c	(TG)17(AG)10	54	32804067	32804120
chr1_5752	p2	(TC)6	12	32807114	32807125
chr1_5753	p2	(AC)6	12	32810148	32810159
chr1_5754	p2	(TG)23	46	32811925	32811970
chr1_5755	p2	(CA)15	30	32819388	32819417
chr1_5756	p4	(AAGA)5	20	32821050	32821069
chr1_5757	p2	(TA)6	12	32828252	32828263
chr1_5758	p2	(AC)11	22	32829832	32829853
chr1_5759	p2	(AT)10	20	32830077	32830096
chr1_5760	p2	(TG)8	16	32834049	32834064
chr1_5761	p2	(GT)8	16	32834266	32834281
chr1_5762	p2	(AC)7	14	32834737	32834750
chr1_5763	p2	(TG)9	18	32836401	32836418
chr1_5764	p2	(AC)14	28	32838952	32838979
chr1_5765	p2	(TC)7	14	32846363	32846376
chr1_5766	p2	(AC)6	12	32852607	32852618
chr1_5767	p2	(CA)6	12	32852843	32852854
chr1_5768	p2	(TC)13	26	32853300	32853325

chr1_5769	p2	(GT)9	18	32854934	32854951
chr1_5770	p2	(TG)6	12	32855166	32855177
chr1_5771	p2	(GT)11	22	32856158	32856179
chr1_5772	c	gacagctgatgagggtctgcatatggctcacctctgaccctcatgccacttc	138	32857275	32857412
chr1_5773	p2	(TG)6	12	32861366	32861377
chr1_5774	p2	(AC)7	14	32868768	32868781
chr1_5775	p2	(TG)6	12	32869363	32869374
chr1_5776	p2	(CA)17	34	32870102	32870135
chr1_5777	p2	(GT)6	12	32870927	32870938
chr1_5778	p2	(GT)9	18	32876096	32876113
chr1_5779	p2	(GT)6	12	32877996	32878007
chr1_5780	p3	(CCT)5	15	32889817	32889831
chr1_5781	p2	(TG)6	12	32890108	32890119
chr1_5782	p2	(CA)6	12	32890906	32890917
chr1_5783	c	(GT)6(GA)8	28	32904087	32904114
chr1_5784	p2	(GT)21	42	32904344	32904385
chr1_5785	p4	(CCAT)5	20	32911405	32911424
chr1_5786	p2	(AC)12	24	32912740	32912763
chr1_5787	p2	(TG)15	30	32915019	32915048
chr1_5788	p2	(CA)14	28	32916789	32916816
chr1_5789	c	(CT)6atctctt(TC)6ttactt(TC)14	65	32917019	32917083
chr1_5790	p2	(AC)6	12	32919166	32919177
chr1_5791	p2	(TG)6	12	32920249	32920260
chr1_5792	p2	(TG)6	12	32921175	32921186
chr1_5793	p2	(GT)26	52	32943506	32943557
chr1_5794	p2	(TA)6	12	32944076	32944087
chr1_5795	p2	(TG)13	26	32944389	32944414
chr1_5796	p2	(GT)11	22	32944571	32944592
chr1_5797	p2	(TA)8	16	32960883	32960898
chr1_5798	p2	(CA)10	20	32961478	32961497
chr1_5799	c	(TGT)29(TCT)12	123	32983164	32983286
chr1_5800	p2	(TG)6	12	32998441	32998452
chr1_5801	p2	(AC)6	12	33000692	33000703
chr1_5802	p5	(GTAGT)5	25	33008726	33008750
chr1_5803	c*	(AC)17(CAT)7*	54	33019755	33019808
chr1_5804	p2	(TG)6	12	33020678	33020689
chr1_5805	p3	(ATA)6	18	33024036	33024053
chr1_5806	p2	(GT)8	16	33024743	33024758
chr1_5807	p4	(AGAT)12	48	33028402	33028449
chr1_5808	p2	(CA)6	12	33029747	33029758
chr1_5809	p2	(AC)11	22	33035156	33035177
chr1_5810	p2	(TG)10	20	33036376	33036395
chr1_5811	p2	(GT)6	12	33036769	33036780
chr1_5812	p2	(AG)7	14	33037001	33037014
chr1_5813	c	(GT)9gagtgtgtgt(GA)8	44	33037577	33037620
chr1_5814	p4	(TATC)10	40	33049276	33049315
chr1_5815	p2	(CA)17	34	33061085	33061118
chr1_5816	p3	(CCA)6	18	33063779	33063796
chr1_5817	p3	(AAG)6	18	33066161	33066178
chr1_5818	c	(CA)6g(CA)7	27	33073843	33073869
chr1_5819	p2	(TG)10	20	33075314	33075333
chr1_5820	p2	(TG)8	16	33077635	33077650

chr1_5821	c	(AC)6g(CA)6	25	33077760	33077784
chr1_5822	c	gttttcctactatgtggggactgctcactcact(CA)13tacacccctactt	145	33088852	33088996
chr1_5823	p3	(TCC)5	15	33100430	33100444
chr1_5824	c	(TA)8tttatatata(TG)7tatac(AT)8	61	33102426	33102486
chr1_5825	p2	(TG)10	20	33109806	33109825
chr1_5826	p2	(TG)7	14	33116577	33116590
chr1_5827	p3	(TCC)5	15	33117741	33117755
chr1_5828	p2	(TG)10	20	33120402	33120421
chr1_5829	p2	(AC)13	26	33120599	33120624
chr1_5830	p2	(TG)24	48	33124823	33124870
chr1_5831	c	ggtgtgatggggctcagtttggcctcaggcctcccgctgtggattctgtatg	140	33128389	33128528
chr1_5832	c	(GT)6c(TG)32	77	33129547	33129623
chr1_5833	p2	(AC)6	12	33130190	33130201
chr1_5834	p2	(TG)6	12	33130881	33130892
chr1_5835	p2	(AC)17	34	33131255	33131288
chr1_5836	p2	(TC)8	16	33132454	33132469
chr1_5837	p2	(AG)6	12	33132746	33132757
chr1_5838	p2	(CA)6	12	33133600	33133611
chr1_5839	p2	(GT)7	14	33134101	33134114
chr1_5840	p2	(GT)15	30	33136923	33136952
chr1_5841	p2	(AC)6	12	33137959	33137970
chr1_5842	p2	(AC)7	14	33139873	33139886
chr1_5843	p2	(AC)7	14	33142484	33142497
chr1_5844	c	(GT)7gcgcgcgcgcatgtggatgcgtgc(GT)8	54	33143212	33143265
chr1_5845	p2	(AG)8	16	33144879	33144894
chr1_5846	p4	(TCCA)5	20	33160452	33160471
chr1_5847	p2	(AC)14	28	33163738	33163765
chr1_5848	p2	(GT)11	22	33165986	33166007
chr1_5849	p2	(CA)7	14	33166620	33166633
chr1_5850	p2	(CT)7	14	33171786	33171799
chr1_5851	p2	(GT)8	16	33172058	33172073
chr1_5852	p2	(AC)6	12	33172218	33172229
chr1_5853	c	(GT)8(AC)6	28	33174673	33174700
chr1_5854	p2	(CA)11	22	33181644	33181665
chr1_5855	p2	(CA)11	22	33182760	33182781
chr1_5856	p2	(TG)17	34	33183356	33183389
chr1_5857	p2	(TG)7	14	33183843	33183856
chr1_5858	p2	(GA)6	12	33184096	33184107
chr1_5859	c	gcataaaatctaataagaatcagatgcagcagcattataacattttaatcttc	128	33187548	33187675
chr1_5860	p2	(AT)11	22	33188438	33188459
chr1_5861	p2	(AC)6	12	33189017	33189028
chr1_5862	p2	(AC)8	16	33189516	33189531
chr1_5863	p2	(AC)14	28	33189652	33189679
chr1_5864	c	(GT)11agtg(GT)7agtg(GT)12	68	33195900	33195967
chr1_5865	p2	(AG)11	22	33198792	33198813
chr1_5866	c	(TA)6cacat(AC)6	29	33200846	33200874
chr1_5867	p2	(AC)10	20	33201258	33201277
chr1_5868	p2	(AG)7	14	33202411	33202424
chr1_5869	p2	(GT)15	30	33205569	33205598
chr1_5870	p3	(ATT)6	18	33208786	33208803
chr1_5871	c	(TA)7(TG)8	30	33226992	33227021
chr1_5872	p2	(GT)6	12	33238935	33238946

chr1_5873	p2	(TA)7	14	33264330	33264343
chr1_5874	p2	(AC)6	12	33291562	33291573
chr1_5875	p2	(CA)22	44	33332976	33333019
chr1_5876	p2	(TC)6	12	33333745	33333756
chr1_5877	p4	(ATGT)7	28	33347816	33347843
chr1_5878	p2	(GT)7	14	33360693	33360706
chr1_5879	p2	(TG)10	20	33364911	33364930
chr1_5880	p2	(TG)20	40	33369636	33369675
chr1_5881	p2	(CA)8	16	33370800	33370815
chr1_5882	p3	(AGG)5	15	33379153	33379167
chr1_5883	p3	(TGT)7	21	33379272	33379292
chr1_5884	p2	(TG)12	24	33380309	33380332
chr1_5885	p2	(AC)11	22	33383949	33383970
chr1_5886	p2	(TG)9	18	33415838	33415855
chr1_5887	p3	(TAT)13	39	33416527	33416565
chr1_5888	p2	(TG)9	18	33419434	33419451
chr1_5889	p2	(GT)10	20	33419855	33419874
chr1_5890	p2	(AC)18	36	33421754	33421789
chr1_5891	p2	(TC)8	16	33423029	33423044
chr1_5892	p2	(GT)6	12	33423240	33423251
chr1_5893	p2	(CT)15	30	33426934	33426963
chr1_5894	p3	(CTC)8	24	33428270	33428293
chr1_5895	p2	(CA)6	12	33429041	33429052
chr1_5896	p2	(TG)13	26	33430394	33430419
chr1_5897	p2	(AC)9	18	33431095	33431112
chr1_5898	c	(TC)6tttcacacacg(CA)13	49	33432163	33432211
chr1_5899	p2	(TG)23	46	33434459	33434504
chr1_5900	p2	(GT)6	12	33438823	33438834
chr1_5901	c	(TG)22(AG)7	58	33439041	33439098
chr1_5902	p3	(AAG)8	24	33439386	33439409
chr1_5903	p3	(TTA)5	15	33440051	33440065
chr1_5904	p2	(CT)6	12	33440328	33440339
chr1_5905	c	(TC)6(AC)6	24	33442318	33442341
chr1_5906	p2	(TG)14	28	33442601	33442628
chr1_5907	p4	(AAAC)6	24	33443737	33443760
chr1_5908	p2	(CA)16	32	33444244	33444275
chr1_5909	p2	(GT)14	28	33444887	33444914
chr1_5910	p2	(TG)10	20	33445790	33445809
chr1_5911	p2	(AC)11	22	33449753	33449774
chr1_5912	p2	(GT)18	36	33450469	33450504
chr1_5913	p2	(AT)8	16	33452386	33452401
chr1_5914	p2	(AC)16	32	33456987	33457018
chr1_5915	p2	(CA)6	12	33457144	33457155
chr1_5916	p2	(CA)6	12	33458571	33458582
chr1_5917	p2	(GT)8	16	33459518	33459533
chr1_5918	p2	(GT)27	54	33459719	33459772
chr1_5919	p3	(CAA)5	15	33463377	33463391
chr1_5920	p2	(GT)18	36	33465071	33465106
chr1_5921	p2	(AC)6	12	33468655	33468666
chr1_5922	c	GT)15acatgccgtagtggtgggaatctttggcaagctca(CAATT)[illegible]	100	33469199	33469298
chr1_5923	p2	(AC)6	12	33474800	33474811
chr1_5924	p2	(TG)18	36	33475583	33475618

chr1_5925	p2	(TG)22	44	33476599	33476642
chr1_5926	p2	(TG)7	14	33479170	33479183
chr1_5927	p2	(TG)11	22	33480072	33480093
chr1_5928	p2	(TA)9	18	33481929	33481946
chr1_5929	p2	(CA)9	18	33483083	33483100
chr1_5930	p2	(TG)11	22	33484003	33484024
chr1_5931	p2	(CA)14	28	33484515	33484542
chr1_5932	p2	(CT)14	28	33485295	33485322
chr1_5933	p4	(AAAG)5	20	33489177	33489196
chr1_5934	c	(GT)7ga(GT)6	28	33492527	33492554
chr1_5935	p2	(AC)18	36	33495166	33495201
chr1_5936	p3	(GAG)5	15	33529374	33529388
chr1_5937	p2	(AG)12	24	33548056	33548079
chr1_5938	p2	(CA)25	50	33552544	33552593
chr1_5939	p3	(CAC)5	15	33559121	33559135
chr1_5940	p2	(CA)10	20	33559479	33559498
chr1_5941	p5	(AGTTG)6	30	33578435	33578464
chr1_5942	p2	(CA)11	22	33580415	33580436
chr1_5943	p2	(TG)6	12	33594471	33594482
chr1_5944	p2	(AC)16	32	33601936	33601967
chr1_5945	p2	(CA)6	12	33605813	33605824
chr1_5946	p3	(AAC)23	69	33613081	33613149
chr1_5947	c	(CA)30c(CA)6	73	33644425	33644497
chr1_5948	c	.C)7gtcttaatgccactggcgctgaaagataacttgtgaga(GT)9(AT	88	33646386	33646473
chr1_5949	p2	(AC)6	12	33650614	33650625
chr1_5950	p2	(AC)11	22	33653444	33653465
chr1_5951	p3	(GAA)5	15	33664420	33664434
chr1_5952	p2	(GT)38	76	33669208	33669283
chr1_5953	p2	(CA)10	20	33672325	33672344
chr1_5954	p2	(GT)12	24	33674328	33674351
chr1_5955	p2	(TG)35	70	33675531	33675600
chr1_5956	p2	(CA)10	20	33678117	33678136
chr1_5957	p2	(GT)12	24	33680120	33680143
chr1_5958	p2	(GA)6	12	33682947	33682958
chr1_5959	p2	(CA)10	20	33688121	33688140
chr1_5960	p2	(GT)13	26	33690123	33690148
chr1_5961	p2	(GA)6	12	33692954	33692965
chr1_5962	c	(GA)9gc(GA)13	46	33699180	33699225
chr1_5963	p2	(CA)11	22	33702791	33702812
chr1_5964	p2	(AG)9	18	33703509	33703526
chr1_5965	p2	(CA)11	22	33703793	33703814
chr1_5966	p2	(AG)9	18	33704508	33704525
chr1_5967	p2	(AC)18	36	33705473	33705508
chr1_5968	p2	(TC)6	12	33705637	33705648
chr1_5969	p2	(AG)9	18	33706299	33706316
chr1_5970	p2	(TG)6	12	33710104	33710115
chr1_5971	p3	(CAA)10	30	33711811	33711840
chr1_5972	p2	(AC)7	14	33715193	33715206
chr1_5973	p2	(AC)10	20	33721056	33721075
chr1_5974	p2	(CA)7	14	33731196	33731209
chr1_5975	c	(AGAC)10agatagac(AGAT)7	76	33737302	33737377
chr1_5976	p2	(CA)10	20	33739314	33739333

chr1_5977	p2	(AC)9	18	33747823	33747840
chr1_5978	p2	(TG)19	38	33754757	33754794
chr1_5979	p3	(TGA)13	39	33774929	33774967
chr1_5980	p2	(GA)19	38	33777722	33777759
chr1_5981	p4	(AGAT)11	44	33778073	33778116
chr1_5982	p3	(TTC)5	15	33778363	33778377
chr1_5983	p3	(TAT)5	15	33780988	33781002
chr1_5984	c	(GT)22(GA)9	62	33782748	33782809
chr1_5985	p2	(GT)11	22	33783051	33783072
chr1_5986	p2	(AC)7	14	33785358	33785371
chr1_5987	p2	(AC)9	18	33787151	33787168
chr1_5988	p2	(CA)7	14	33794022	33794035
chr1_5989	p2	(GT)13	26	33795156	33795181
chr1_5990	p2	(GT)11	22	33796927	33796948
chr1_5991	p2	(GT)7	14	33797962	33797975
chr1_5992	p2	(GT)11	22	33799355	33799376
chr1_5993	p2	(AC)34	68	33799550	33799617
chr1_5994	p2	(AG)40	80	33801633	33801712
chr1_5995	p2	(AC)19	38	33807410	33807447
chr1_5996	p2	(AC)15	30	33807769	33807798
chr1_5997	p3	(AAG)5	15	33808549	33808563
chr1_5998	p2	(CA)14	28	33809380	33809407
chr1_5999	p2	(TG)7	14	33809872	33809885
chr1_6000	p2	(AC)11	22	33810043	33810064
chr1_6001	p2	(TG)6	12	33810177	33810188
chr1_6002	p2	(TG)8	16	33813939	33813954
chr1_6003	p2	(CA)21	42	33819127	33819168
chr1_6004	p2	(AC)13	26	33823330	33823355
chr1_6005	p2	(GT)19	38	33827641	33827678
chr1_6006	p2	(GT)7	14	33832166	33832179
chr1_6007	p3	(CAT)7	21	33842840	33842860
chr1_6008	p2	(TC)6	12	33844482	33844493
chr1_6009	p2	(GA)6	12	33848228	33848239
chr1_6010	p2	(CA)30	60	33850026	33850085
chr1_6011	p2	(AC)10	20	33860825	33860844
chr1_6012	p2	(CA)19	38	33871014	33871051
chr1_6013	p3	(AAT)6	18	33874384	33874401
chr1_6014	p3	(GCA)5	15	33875431	33875445
chr1_6015	p2	(CA)13	26	33877354	33877379
chr1_6016	p2	(TG)18	36	33881439	33881474
chr1_6017	p2	(TA)6	12	33884206	33884217
chr1_6018	p2	(TG)12	24	33895106	33895129
chr1_6019	p2	(AC)6	12	33902727	33902738
chr1_6020	p2	(TC)10	20	33930170	33930189
chr1_6021	p2	(AC)9	18	33935476	33935493
chr1_6022	p2	(AC)6	12	33948403	33948414
chr1_6023	p2	(CA)9	18	33950673	33950690
chr1_6024	p4	(TCAC)8	32	33970239	33970270
chr1_6025	p2	(CA)6	12	33973025	33973036
chr1_6026	p3	(TAT)5	15	33994218	33994232
chr1_6027	p2	(GA)7	14	33994494	33994507
chr1_6028	p3	(AGT)5	15	33998831	33998845

chr1_6029	p2	(TG)6	12	34004926	34004937
chr1_6030	p2	(TG)13	26	34011694	34011719
chr1_6031	p2	(TG)6	12	34017056	34017067
chr1_6032	p2	(AC)16	32	34019044	34019075
chr1_6033	p2	(GT)7	14	34020907	34020920
chr1_6034	p2	(TG)13	26	34029108	34029133
chr1_6035	p2	(TG)23	46	34030795	34030840
chr1_6036	p2	(AC)6	12	34033995	34034006
chr1_6037	p2	(AC)10	20	34034784	34034803
chr1_6038	p2	(TG)6	12	34035912	34035923
chr1_6039	p4	(AAAT)5	20	34037867	34037886
chr1_6040	p2	(GA)6	12	34045571	34045582
chr1_6041	p2	(AC)6	12	34056119	34056130
chr1_6042	p3	(AAC)7	21	34056550	34056570
chr1_6043	p2	(AC)23	46	34063855	34063900
chr1_6044	p2	(GT)10	20	34065895	34065914
chr1_6045	p2	(AG)7	14	34073457	34073470
chr1_6046	p2	(AC)6	12	34073960	34073971
chr1_6047	c	(GT)6ctgcatt(TG)6	31	34077157	34077187
chr1_6048	p2	(CA)6	12	34079628	34079639
chr1_6049	p3	(TTA)5	15	34097529	34097543
chr1_6050	p4	(CTGT)5	20	34100009	34100028
chr1_6051	p4	(ATGG)5	20	34102323	34102342
chr1_6052	p2	(GT)15	30	34108578	34108607
chr1_6053	p3	(CTT)7	21	34113104	34113124
chr1_6054	p2	(AC)17	34	34117392	34117425
chr1_6055	p2	(GT)27	54	34122104	34122157
chr1_6056	p2	(GT)7	14	34128806	34128819
chr1_6057	p3	(CAA)5	15	34130790	34130804
chr1_6058	p2	(AC)12	24	34132106	34132129
chr1_6059	p2	(TG)19	38	34138316	34138353
chr1_6060	p2	(AC)12	24	34138571	34138594
chr1_6061	p3	(AAG)6	18	34150204	34150221
chr1_6062	p4	(AAAT)5	20	34157752	34157771
chr1_6063	p2	(AG)6	12	34163607	34163618
chr1_6064	p2	(CA)8	16	34184599	34184614
chr1_6065	p4	(ATGG)5	20	34193028	34193047
chr1_6066	p2	(GT)8	16	34196351	34196366
chr1_6067	p4	(CATT)6	24	34197795	34197818
chr1_6068	p2	(CA)11	22	34208244	34208265
chr1_6069	p3	(TTA)8	24	34237297	34237320
chr1_6070	p2	(AC)7	14	34238549	34238562
chr1_6071	p4	(ATAG)17	68	34246059	34246126
chr1_6072	p2	(CT)6	12	34248692	34248703
chr1_6073	p2	(TG)6	12	34251207	34251218
chr1_6074	p2	(GT)6	12	34256192	34256203
chr1_6075	p2	(GT)10	20	34259994	34260013
chr1_6076	p2	(TC)9	18	34265941	34265958
chr1_6077	p2	(TG)14	28	34274967	34274994
chr1_6078	p2	(TG)6	12	34276382	34276393
chr1_6079	p2	(GT)6	12	34286375	34286386
chr1_6080	p2	(AT)6	12	34300951	34300962

chr1_6081	p2	(GT)11	22	34301452	34301473
chr1_6082	c	(TG)18c(GT)6gg(GT)6	63	34314516	34314578
chr1_6083	p2	(AC)6	12	34318206	34318217
chr1_6084	c	agacttctacttttagtatgtgggcagcattggggtcttattgctgcaagtgca	141	34318425	34318565
chr1_6085	p2	(CA)6	12	34318734	34318745
chr1_6086	p2	(CA)6	12	34321858	34321869
chr1_6087	p3	(ATA)10	30	34329353	34329382
chr1_6088	c	(TG)28(TA)8(TG)21	114	34329754	34329867
chr1_6089	p2	(AT)6	12	34336716	34336727
chr1_6090	c	(CA)6attgtgtgtatgc(GA)25gcgc(GA)9	97	34337625	34337721
chr1_6091	p2	(CT)12	24	34341707	34341730
chr1_6092	p2	(CA)6	12	34344268	34344279
chr1_6093	c	attcctgtgacagagattgatgaaggcctattatttgatgtccagctgagctc	149	34344544	34344692
chr1_6094	p2	(CA)23	46	34344909	34344954
chr1_6095	p3	(AAG)5	15	34346241	34346255
chr1_6096	p3	(GTG)5	15	34347676	34347690
chr1_6097	p2	(AT)6	12	34349892	34349903
chr1_6098	p2	(AC)11	22	34353324	34353345
chr1_6099	p2	(GT)10	20	34357082	34357101
chr1_6100	p2	(AC)13	26	34360617	34360642
chr1_6101	p2	(CA)13	26	34360770	34360795
chr1_6102	p2	(CA)8	16	34365949	34365964
chr1_6103	p3	(ATC)13	39	34370234	34370272
chr1_6104	p2	(CA)7	14	34379315	34379328
chr1_6105	p2	(TG)17	34	34387883	34387916
chr1_6106	p3	(TAC)5	15	34390725	34390739
chr1_6107	p2	(GA)35	70	34398260	34398329
chr1_6108	p2	(AC)7	14	34404099	34404112
chr1_6109	p2	(CA)19	38	34404419	34404456
chr1_6110	p2	(TC)6	12	34415854	34415865
chr1_6111	p2	(TG)7	14	34423658	34423671
chr1_6112	p2	(CA)13	26	34423884	34423909
chr1_6113	p2	(CA)6	12	34426807	34426818
chr1_6114	p2	(AC)8	16	34444531	34444546
chr1_6115	p6	(ATACAT)6	36	34452731	34452766
chr1_6116	p3	(ATA)10	30	34454166	34454195
chr1_6117	p2	(TC)10	20	34465880	34465899
chr1_6118	p3	(CAA)5	15	34468044	34468058
chr1_6119	p3	(GGA)6	18	34471411	34471428
chr1_6120	p2	(AC)15	30	34485794	34485823
chr1_6121	p3	(GCT)5	15	34496942	34496956
chr1_6122	p2	(AC)10	20	34501841	34501860
chr1_6123	p2	(GT)6	12	34508250	34508261
chr1_6124	p2	(CA)12	24	34508651	34508674
chr1_6125	p2	(TG)6	12	34508873	34508884
chr1_6126	p2	(TG)6	12	34509549	34509560
chr1_6127	p2	(TG)10	20	34526311	34526330
chr1_6128	p2	(AC)7	14	34528064	34528077
chr1_6129	p3	(GAT)6	18	34540194	34540211
chr1_6130	p2	(TG)11	22	34540412	34540433
chr1_6131	p2	(AC)8	16	34543286	34543301
chr1_6132	p2	(TA)7	14	34545494	34545507

chr1_6133	p2	(TA)9	18	34549326	34549343
chr1_6134	p2	(TG)6	12	34554097	34554108
chr1_6135	p2	(AG)6	12	34554271	34554282
chr1_6136	p2	(CA)13	26	34592719	34592744
chr1_6137	p3	(ATA)8	24	34593234	34593257
chr1_6138	p3	(ATA)5	15	34602078	34602092
chr1_6139	p2	(AT)7	14	34604624	34604637
chr1_6140	p3	(GCT)6	18	34606455	34606472
chr1_6141	p2	(CT)7	14	34608572	34608585
chr1_6142	p2	(CT)7	14	34619122	34619135
chr1_6143	p3	(ATA)6	18	34619406	34619423
chr1_6144	c	(TG)13a(GT)8	43	34623198	34623240
chr1_6145	p2	(GT)26	52	34626628	34626679
chr1_6146	p2	(GT)15	30	34650732	34650761
chr1_6147	p2	(TG)6	12	34651139	34651150
chr1_6148	p2	(CA)6	12	34653630	34653641
chr1_6149	p2	(TG)6	12	34661426	34661437
chr1_6150	p2	(TG)16	32	34676796	34676827
chr1_6151	p2	(TG)28	56	34679407	34679462
chr1_6152	p2	(AC)6	12	34679707	34679718
chr1_6153	c	(TG)15t(CA)6	43	34682540	34682582
chr1_6154	p3	(TTA)5	15	34683050	34683064
chr1_6155	p2	(TG)10	20	34683581	34683600
chr1_6156	p2	(GT)13	26	34685239	34685264
chr1_6157	p2	(TA)6	12	34691709	34691720
chr1_6158	p2	(TG)14	28	34700234	34700261
chr1_6159	p2	(TG)9	18	34725013	34725030
chr1_6160	p2	(AC)8	16	34753856	34753871
chr1_6161	p2	(TG)6	12	34754830	34754841
chr1_6162	p2	(GA)7	14	34759904	34759917
chr1_6163	p2	(AG)6	12	34763284	34763295
chr1_6164	p2	(AC)21	42	34765261	34765302
chr1_6165	c	(TC)21a(CT)7	57	34767470	34767526
chr1_6166	p2	(TG)6	12	34778498	34778509
chr1_6167	p2	(GT)14	28	34782044	34782071
chr1_6168	p2	(AT)7	14	34785882	34785895
chr1_6169	p3	(TTC)6	18	34790034	34790051
chr1_6170	p2	(CT)6	12	34794534	34794545
chr1_6171	p2	(CT)6	12	34796899	34796910
chr1_6172	p2	(AC)7	14	34797455	34797468
chr1_6173	p2	(TC)11	22	34798988	34799009
chr1_6174	p2	(AC)6	12	34802426	34802437
chr1_6175	p2	(AC)8	16	34809978	34809993
chr1_6176	p2	(AC)7	14	34812999	34813012
chr1_6177	p2	(CA)6	12	34815782	34815793
chr1_6178	p2	(TG)14	28	34816052	34816079
chr1_6179	p2	(AG)6	12	34822688	34822699
chr1_6180	p2	(TG)7	14	34824266	34824279
chr1_6181	p2	(GT)17	34	34824432	34824465
chr1_6182	p2	(TG)15	30	34825564	34825593
chr1_6183	p2	(TG)17	34	34828385	34828418
chr1_6184	p2	(CA)18	36	34836463	34836498

chr1_6185	p2	(CA)15	30	34838216	34838245
chr1_6186	p2	(AC)7	14	34839211	34839224
chr1_6187	p2	(CA)21	42	34839453	34839494
chr1_6188	p2	(AC)10	20	34841176	34841195
chr1_6189	p2	(AT)9	18	34841612	34841629
chr1_6190	p2	(AC)11	22	34844621	34844642
chr1_6191	p2	(TG)23	46	34846019	34846064
chr1_6192	p2	(CA)6	12	34847699	34847710
chr1_6193	p2	(GT)10	20	34850288	34850307
chr1_6194	p2	(GT)13	26	34854941	34854966
chr1_6195	p2	(GT)9	18	34869769	34869786
chr1_6196	p3	(TGA)5	15	34873337	34873351
chr1_6197	p2	(TG)12	24	34874772	34874795
chr1_6198	p2	(TC)13	26	34875602	34875627
chr1_6199	p2	(AC)19	38	34877308	34877345
chr1_6200	c	(TG)10(AG)7	34	34878397	34878430
chr1_6201	p2	(TA)6	12	34882343	34882354
chr1_6202	p2	(AG)6	12	34882464	34882475
chr1_6203	p2	(TG)6	12	34883036	34883047
chr1_6204	p3	(ATA)6	18	34890395	34890412
chr1_6205	p2	(GT)6	12	34890729	34890740
chr1_6206	p3	(ATA)6	18	34894159	34894176
chr1_6207	p2	(CT)14	28	34898231	34898258
chr1_6208	p2	(TG)16	32	34901544	34901575
chr1_6209	p2	(GT)10	20	34905266	34905285
chr1_6210	p2	(AC)13	26	34906156	34906181
chr1_6211	p2	(CA)13	26	34906388	34906413
chr1_6212	c	atattgtgatgatgtacacaatctgtgtgggcaaaagatgtatagattaatgct	125	34918726	34918850
chr1_6213	p2	(AC)7	14	34940326	34940339
chr1_6214	p2	(TG)6	12	34950418	34950429
chr1_6215	p2	(CT)18	36	34958768	34958803
chr1_6216	p2	(CA)15	30	34973248	34973277
chr1_6217	p2	(TG)7	14	34975317	34975330
chr1_6218	p2	(GT)16	32	34978342	34978373
chr1_6219	p2	(TG)26	52	34979800	34979851
chr1_6220	p2	(TG)7	14	34980011	34980024
chr1_6221	p2	(GT)6	12	34980198	34980209
chr1_6222	p2	(TG)8	16	34983504	34983519
chr1_6223	c	(CT)6(CA)11	34	34991577	34991610
chr1_6224	p2	(TG)7	14	34991735	34991748
chr1_6225	p2	(GT)12	24	34996551	34996574
chr1_6226	p2	(AT)7	14	35002323	35002336
chr1_6227	p2	(TC)9	18	35032699	35032716
chr1_6228	p2	(TG)15	30	35035536	35035565
chr1_6229	p2	(CT)6	12	35041649	35041660
chr1_6230	p2	(CA)9	18	35057157	35057174
chr1_6231	p3	(GTG)6	18	35065650	35065667
chr1_6232	c	(TCT)5ttttt(TAC)5cacca(CTT)5	55	35067572	35067626
chr1_6233	p3	(CTT)5	15	35068014	35068028
chr1_6234	p2	(TG)6	12	35072600	35072611
chr1_6235	p2	(TG)6	12	35073802	35073813
chr1_6236	p3	(TAT)5	15	35078780	35078794

chr1_6237	p3	(TAT)5	15	35080301	35080315
chr1_6238	c	acactcactttcctgaaatactctgaccaacaggacccctagtgatgaaga	150	35092173	35092322
chr1_6239	p4	(ATTG)5	20	35093797	35093816
chr1_6240	p2	(CA)16	32	35097244	35097275
chr1_6241	p2	(AC)15	30	35098732	35098761
chr1_6242	p3	(CAG)5	15	35100630	35100644
chr1_6243	p2	(AC)11	22	35106339	35106360
chr1_6244	p5	(ATTTG)5	25	35121424	35121448
chr1_6245	p2	(GT)16	32	35122379	35122410
chr1_6246	p2	(CA)8	16	35123552	35123567
chr1_6247	p2	(AC)6	12	35124230	35124241
chr1_6248	p2	(TG)6	12	35125528	35125539
chr1_6249	p3	(TTA)6	18	35126163	35126180
chr1_6250	p2	(TG)15	30	35135228	35135257
chr1_6251	p2	(TG)6	12	35136538	35136549
chr1_6252	p4	(TGTA)9	36	35136999	35137034
chr1_6253	p2	(AG)6	12	35141476	35141487
chr1_6254	c	tgctggcttgggtacagatggcacattgtcaaaagctgacatgttttgttttg	144	35142934	35143077
chr1_6255	p4	(AATC)7	28	35144205	35144232
chr1_6256	p2	(AC)7	14	35145814	35145827
chr1_6257	p2	(AC)9	18	35148505	35148522
chr1_6258	p2	(TG)8	16	35149225	35149240
chr1_6259	p2	(TG)7	14	35149370	35149383
chr1_6260	p2	(CA)9	18	35150039	35150056
chr1_6261	c	(TG)6c(GT)7	27	35150466	35150492
chr1_6262	p2	(AT)6	12	35152220	35152231
chr1_6263	p2	(TG)9	18	35154420	35154437
chr1_6264	p3	(TGT)5	15	35154877	35154891
chr1_6265	p2	(CA)21	42	35158728	35158769
chr1_6266	p6	(AGCCCG)6	36	35161698	35161733
chr1_6267	p2	(CA)10	20	35162631	35162650
chr1_6268	p2	(AC)15	30	35163179	35163208
chr1_6269	c	gaatgccagtctgatcctttccattcattgagaaaagaaaagtgaggaggtg	96	35163750	35163845
chr1_6270	p2	(GA)7	14	35168392	35168405
chr1_6271	p2	(GA)6	12	35170157	35170168
chr1_6272	p2	(AC)9	18	35172780	35172797
chr1_6273	p2	(GT)16	32	35176353	35176384
chr1_6274	p2	(CA)13	26	35181980	35182005
chr1_6275	p2	(CA)8	16	35182163	35182178
chr1_6276	p2	(CT)10	20	35182325	35182344
chr1_6277	p2	(AG)6	12	35183032	35183043
chr1_6278	p2	(AC)6	12	35189726	35189737
chr1_6279	c	(GT)12(TG)6	36	35190110	35190145
chr1_6280	p2	(CA)9	18	35199660	35199677
chr1_6281	p2	(CA)6	12	35199915	35199926
chr1_6282	c	(GA)8gtc(TG)20	59	35204851	35204909
chr1_6283	p2	(CA)17	34	35206560	35206593
chr1_6284	p2	(GT)7	14	35208042	35208055
chr1_6285	p2	(CT)7	14	35214660	35214673
chr1_6286	p2	(GT)6	12	35222903	35222914
chr1_6287	c	(AATG)5(AATC)5	40	35227620	35227659
chr1_6288	p2	(TG)16	32	35231641	35231672

chr1_6289	p2	(CT)8	16	35234354	35234369
chr1_6290	p2	(GT)6	12	35237213	35237224
chr1_6291	p2	(TG)6	12	35237492	35237503
chr1_6292	p2	(TG)6	12	35240930	35240941
chr1_6293	p2	(GT)7	14	35243648	35243661
chr1_6294	p2	(TG)13	26	35243766	35243791
chr1_6295	p3	(TAT)5	15	35250131	35250145
chr1_6296	c	(ATA)6atcacttttatttgaccaagtatatt(AC)6	56	35251607	35251662
chr1_6297	p2	(TG)21	42	35253750	35253791
chr1_6298	p2	(AC)16	32	35255363	35255394
chr1_6299	c	(TC)6(AC)6	24	35255730	35255753
chr1_6300	p4	(TTTG)5	20	35260173	35260192
chr1_6301	p2	(TG)8	16	35260731	35260746
chr1_6302	p2	(GT)11	22	35262940	35262961
chr1_6303	p2	(CA)19	38	35264364	35264401
chr1_6304	p2	(TG)7	14	35264905	35264918
chr1_6305	p2	(CA)8	16	35272323	35272338
chr1_6306	p2	(AG)8	16	35272981	35272996
chr1_6307	p2	(CA)9	18	35273859	35273876
chr1_6308	p2	(TG)11	22	35278300	35278321
chr1_6309	p2	(TG)8	16	35278697	35278712
chr1_6310	p2	(AC)7	14	35286563	35286576
chr1_6311	p2	(CA)6	12	35286877	35286888
chr1_6312	p2	(AC)16	32	35287708	35287739
chr1_6313	p2	(CA)9	18	35288383	35288400
chr1_6314	c	(CA)8(TA)7	30	35288517	35288546
chr1_6315	p2	(AC)14	28	35294813	35294840
chr1_6316	p3	(AGA)5	15	35295185	35295199
chr1_6317	p2	(TG)16	32	35297084	35297115
chr1_6318	p2	(CA)13	26	35297441	35297466
chr1_6319	p2	(TA)8	16	35298478	35298493
chr1_6320	p2	(GT)6	12	35299476	35299487
chr1_6321	p2	(AG)6	12	35299699	35299710
chr1_6322	p2	(GT)7	14	35300466	35300479
chr1_6323	p2	(TG)7	14	35301376	35301389
chr1_6324	p2	(AG)6	12	35302464	35302475
chr1_6325	p2	(AC)8	16	35302689	35302704
chr1_6326	p2	(CA)7	14	35307126	35307139
chr1_6327	p2	(TG)16	32	35308762	35308793
chr1_6328	p2	(CA)6	12	35309103	35309114
chr1_6329	p2	(CA)6	12	35309388	35309399
chr1_6330	p2	(GT)11	22	35312938	35312959
chr1_6331	c	gagaaat(AG)6aaagaactttaaaatgcaaacatacagaaacaatctct	137	35313713	35313849
chr1_6332	p2	(GT)6	12	35314364	35314375
chr1_6333	c	(TG)9tttgtt(TG)6	36	35315603	35315638
chr1_6334	p2	(AC)6	12	35315818	35315829
chr1_6335	c	(GA)29ggaagagagaa(AG)10	89	35317754	35317842
chr1_6336	p2	(TG)12	24	35318535	35318558
chr1_6337	p2	(TG)36	72	35318909	35318980
chr1_6338	p3	(TTA)10	30	35320482	35320511
chr1_6339	p3	(CAC)5	15	35322164	35322178
chr1_6340	p2	(AC)16	32	35322555	35322586

chr1_6341	c	(CT)12ccctctctctttctctcgcc(CT)6	58	35326043	35326100
chr1_6342	p2	(AG)11	22	35328839	35328860
chr1_6343	p3	(CAG)7	21	35329820	35329840
chr1_6344	p2	(TG)8	16	35332288	35332303
chr1_6345	p2	(TC)8	16	35332647	35332662
chr1_6346	p2	(TC)7	14	35333302	35333315
chr1_6347	p2	(GT)12	24	35338295	35338318
chr1_6348	c	(CA)8cgcacacacacaggcaccca(CAC)5	51	35338983	35339033
chr1_6349	p2	(GA)8	16	35341555	35341570
chr1_6350	c	(GT)22(GA)10	64	35341706	35341769
chr1_6351	c	ıtcactctctctttcttaaatgaaagttgtttggttggttagcagagcaaagaa	132	35341931	35342062
chr1_6352	p3	(TCT)5	15	35348935	35348949
chr1_6353	c	(CT)9(CA)26	70	35349924	35349993
chr1_6354	p2	(AC)12	24	35351122	35351145
chr1_6355	c	tatgctgagcttgcccctgggtcattagaagttgaaaatgtttatggccaatg	110	35356255	35356364
chr1_6356	p2	(TA)6	12	35357162	35357173
chr1_6357	p2	(TG)22	44	35358076	35358119
chr1_6358	p2	(AC)6	12	35360125	35360136
chr1_6359	p2	(AC)6	12	35361390	35361401
chr1_6360	p2	(TG)8	16	35361934	35361949
chr1_6361	p2	(TA)6	12	35362373	35362384
chr1_6362	p2	(CA)6	12	35366562	35366573
chr1_6363	p2	(TG)7	14	35371492	35371505
chr1_6364	p2	(TG)6	12	35373618	35373629
chr1_6365	c	(CCA)9ccc(CA)7	44	35373945	35373988
chr1_6366	p2	(AC)7	14	35374748	35374761
chr1_6367	p2	(GT)24	48	35375199	35375246
chr1_6368	c	(CA)6tg(CA)8	30	35375805	35375834
chr1_6369	p2	(AC)9	18	35376085	35376102
chr1_6370	p2	(AC)14	28	35377175	35377202
chr1_6371	p2	(TC)10	20	35379941	35379960
chr1_6372	p2	(TG)8	16	35381395	35381410
chr1_6373	p2	(AC)9	18	35381531	35381548
chr1_6374	p2	(TG)6	12	35382751	35382762
chr1_6375	p2	(CA)6	12	35383746	35383757
chr1_6376	p2	(CA)7	14	35383947	35383960
chr1_6377	p2	(GT)8	16	35384129	35384144
chr1_6378	p2	(TC)12	24	35388922	35388945
chr1_6379	p2	(TG)15	30	35389650	35389679
chr1_6380	c	(CT)7ccttcccagc(CT)6	36	35390644	35390679
chr1_6381	p2	(AC)8	16	35392564	35392579
chr1_6382	c	(AGC)9(AAC)6	45	35398584	35398628
chr1_6383	p3	(CCA)5	15	35399741	35399755
chr1_6384	p3	(CCA)6	18	35414835	35414852
chr1_6385	p2	(GT)7	14	35416757	35416770
chr1_6386	p2	(AC)41	82	35416932	35417013
chr1_6387	p2	(CA)7	14	35419943	35419956
chr1_6388	p3	(TGT)5	15	35421706	35421720
chr1_6389	p2	(GT)15	30	35426223	35426252
chr1_6390	p2	(AG)7	14	35426509	35426522
chr1_6391	p2	(TG)11	22	35430637	35430658
chr1_6392	p2	(AG)8	16	35432398	35432413

chr1_6393	p3	(CCA)5	15	35438270	35438284
chr1_6394	p2	(GT)6	12	35438612	35438623
chr1_6395	p2	(AC)8	16	35451634	35451649
chr1_6396	p2	(AC)10	20	35455530	35455549
chr1_6397	p2	(AC)15	30	35459626	35459655
chr1_6398	c	;cttgtgagtgttaatgaggggtggggggtgtcaggcacctgatggtgag	81	35463579	35463659
chr1_6399	p2	(AG)10	20	35464452	35464471
chr1_6400	c	(GT)6atcagagagaacaag(GA)6	39	35473317	35473355
chr1_6401	p2	(AG)6	12	35473806	35473817
chr1_6402	p2	(CA)6	12	35474591	35474602
chr1_6403	p2	(GT)16	32	35476762	35476793
chr1_6404	p2	(GT)13	26	35477027	35477052
chr1_6405	p2	(GT)6	12	35479165	35479176
chr1_6406	p2	(TG)8	16	35482589	35482604
chr1_6407	p3	(ATT)8	24	35483333	35483356
chr1_6408	p2	(AC)6	12	35485783	35485794
chr1_6409	p2	(TG)6	12	35489966	35489977
chr1_6410	p2	(AC)7	14	35496678	35496691
chr1_6411	p2	(GA)6	12	35499040	35499051
chr1_6412	c	(AC)8tcaaagccactaacagact(CA)7	49	35503831	35503879
chr1_6413	p2	(TG)21	42	35506319	35506360
chr1_6414	p2	(TG)17	34	35506738	35506771
chr1_6415	p2	(AC)12	24	35509675	35509698
chr1_6416	p2	(AC)7	14	35509819	35509832
chr1_6417	p2	(TG)6	12	35512726	35512737
chr1_6418	p2	(TG)6	12	35513500	35513511
chr1_6419	p2	(CA)19	38	35513754	35513791
chr1_6420	c	(AT)6(GT)6	24	35514245	35514268
chr1_6421	p4	(ATGA)6	24	35516218	35516241
chr1_6422	c	(TC)9(AC)9	36	35524279	35524314
chr1_6423	p2	(GT)7	14	35524661	35524674
chr1_6424	c	(GT)10ccccagaatctgtgtgaggcacgc(GT)12	68	35528122	35528189
chr1_6425	p4	(ATCT)11	44	35529853	35529896
chr1_6426	p2	(GT)11	22	35531468	35531489
chr1_6427	p4	(TCCA)5	20	35531896	35531915
chr1_6428	p2	(AC)7	14	35538130	35538143
chr1_6429	p2	(AC)12	24	35539661	35539684
chr1_6430	p2	(GT)15	30	35543043	35543072
chr1_6431	p2	(TC)7	14	35545852	35545865
chr1_6432	p2	(TG)6	12	35548219	35548230
chr1_6433	p2	(AC)17	34	35554790	35554823
chr1_6434	p2	(AT)8	16	35556494	35556509
chr1_6435	p2	(TC)8	16	35561010	35561025
chr1_6436	p2	(AG)16	32	35561735	35561766
chr1_6437	c	)18tttctggacacaagctgaaacatgcattttgtctgtgtgtgcacatgta(T	97	35561958	35562054
chr1_6438	p2	(CA)6	12	35568772	35568783
chr1_6439	p2	(CA)16	32	35570900	35570931
chr1_6440	p2	(TG)8	16	35576886	35576901
chr1_6441	p3	(TTG)5	15	35577763	35577777
chr1_6442	p2	(TG)12	24	35579894	35579917
chr1_6443	p2	(CT)6	12	35580110	35580121
chr1_6444	p2	(GT)15	30	35588708	35588737

chr1_6445	p2	(AC)6	12	35590978	35590989
chr1_6446	p2	(CA)22	44	35598429	35598472
chr1_6447	p2	(CA)10	20	35612451	35612470
chr1_6448	p2	(TG)22	44	35616245	35616288
chr1_6449	p2	(TG)12	24	35622816	35622839
chr1_6450	p2	(GT)12	24	35627226	35627249
chr1_6451	p2	(TG)10	20	35627365	35627384
chr1_6452	p2	(AC)17	34	35632116	35632149
chr1_6453	p2	(GT)6	12	35633796	35633807
chr1_6454	p2	(TG)25	50	35639237	35639286
chr1_6455	p2	(GT)19	38	35640818	35640855
chr1_6456	p2	(GT)31	62	35641224	35641285
chr1_6457	p2	(TA)8	16	35642569	35642584
chr1_6458	p2	(GT)10	20	35644675	35644694
chr1_6459	p2	(TG)8	16	35655980	35655995
chr1_6460	p2	(AT)6	12	35656548	35656559
chr1_6461	p2	(AT)10	20	35664880	35664899
chr1_6462	p2	(CA)19	38	35665144	35665181
chr1_6463	p2	(TG)9	18	35668752	35668769
chr1_6464	p2	(CT)8	16	35681768	35681783
chr1_6465	p2	(AC)11	22	35682798	35682819
chr1_6466	p2	(TA)6	12	35687123	35687134
chr1_6467	p2	(AC)6	12	35688813	35688824
chr1_6468	p2	(AC)11	22	35688965	35688986
chr1_6469	p2	(CA)6	12	35689225	35689236
chr1_6470	p2	(CA)10	20	35692813	35692832
chr1_6471	p2	(AC)9	18	35693696	35693713
chr1_6472	p2	(GA)6	12	35700336	35700347
chr1_6473	p4	(ATGG)7	28	35701207	35701234
chr1_6474	p2	(TG)9	18	35703468	35703485
chr1_6475	c	(CA)6g(AC)8	29	35710443	35710471
chr1_6476	p2	(AC)12	24	35718512	35718535
chr1_6477	p3	(AAT)5	15	35720150	35720164
chr1_6478	p2	(CA)8	16	35728712	35728727
chr1_6479	p2	(CA)8	16	35737904	35737919
chr1_6480	p2	(GT)13	26	35754705	35754730
chr1_6481	p2	(TG)7	14	35763868	35763881
chr1_6482	p3	(TAA)5	15	35765811	35765825
chr1_6483	p2	(GT)6	12	35770991	35771002
chr1_6484	p2	(CA)6	12	35773319	35773330
chr1_6485	c	(CG)8(CA)7	30	35774847	35774876
chr1_6486	c	(AT)6acatatatacatatatatatacatatacatatatacat(AG)16	82	35775475	35775556
chr1_6487	p3	(ATA)5	15	35778440	35778454
chr1_6488	p2	(AC)13	26	35782746	35782771
chr1_6489	p2	(TG)7	14	35788436	35788449
chr1_6490	p2	(AC)19	38	35791985	35792022
chr1_6491	p2	(AC)9	18	35800741	35800758
chr1_6492	p2	(GA)10	20	35813801	35813820
chr1_6493	p2	(GT)11	22	35834768	35834789
chr1_6494	p2	(CA)10	20	35836155	35836174
chr1_6495	p2	(GT)6	12	35839981	35839992
chr1_6496	p2	(GT)10	20	35842740	35842759

chr1_6497	p2	(AG)6	12	35847955	35847966
chr1_6498	p2	(TG)6	12	35848303	35848314
chr1_6499	p2	(TA)7	14	35864667	35864680
chr1_6500	p2	(TG)7	14	35871014	35871027
chr1_6501	p2	(TG)6	12	35879464	35879475
chr1_6502	p2	(AG)6	12	35884627	35884638
chr1_6503	p2	(AC)19	38	35885469	35885506
chr1_6504	p2	(TG)17	34	35889096	35889129
chr1_6505	p2	(GT)13	26	35890637	35890662
chr1_6506	p3	(TAT)7	21	35898414	35898434
chr1_6507	p2	(CA)16	32	35899059	35899090
chr1_6508	p2	(GT)6	12	35903148	35903159
chr1_6509	p2	(CA)7	14	35906453	35906466
chr1_6510	p2	(CA)6	12	35907407	35907418
chr1_6511	p2	(TA)7	14	35917106	35917119
chr1_6512	p2	(TA)11	22	35920499	35920520
chr1_6513	p2	(AC)7	14	35920985	35920998
chr1_6514	p2	(TA)11	22	35921385	35921406
chr1_6515	p2	(AC)8	16	35921871	35921886
chr1_6516	p2	(TA)11	22	35922290	35922311
chr1_6517	p2	(AC)9	18	35922776	35922793
chr1_6518	c	5ttttctattttcactctatttccttttacttctctatattttccctcaatttctccactt	97	35932700	35932796
chr1_6519	p2	(TG)7	14	35934778	35934791
chr1_6520	p2	(TG)8	16	35935132	35935147
chr1_6521	p3	(TAA)6	18	35945763	35945780
chr1_6522	p2	(AC)19	38	35948814	35948851
chr1_6523	p2	(TG)9	18	35950783	35950800
chr1_6524	p2	(TG)9	18	35959121	35959138
chr1_6525	p2	(AC)7	14	35959642	35959655
chr1_6526	p2	(AG)9	18	35963778	35963795
chr1_6527	p2	(AC)6	12	35964637	35964648
chr1_6528	p2	(AC)13	26	35967543	35967568
chr1_6529	c	A)6aaatgttatttgcaggcagtaacatctatttttaacttgttttttttt(TA	73	35973196	35973268
chr1_6530	p2	(TG)9	18	35976576	35976593
chr1_6531	p2	(AC)6	12	35977038	35977049
chr1_6532	p3	(TAA)6	18	35980170	35980187
chr1_6533	p4	(CCAT)5	20	35982796	35982815
chr1_6534	p2	(TG)6	12	35991512	35991523
chr1_6535	p3	(TCA)5	15	35994595	35994609
chr1_6536	p2	(CA)6	12	35997272	35997283
chr1_6537	p2	(AC)6	12	35998019	35998030
chr1_6538	p2	(CA)6	12	36007639	36007650
chr1_6539	p2	(GT)12	24	36010686	36010709
chr1_6540	c	(CA)6cttccccgcctc(CA)9	42	36016329	36016370
chr1_6541	p2	(CA)6	12	36019664	36019675
chr1_6542	p2	(CA)15	30	36019923	36019952
chr1_6543	p2	(AC)12	24	36020642	36020665
chr1_6544	p3	(TTG)6	18	36030828	36030845
chr1_6545	p2	(AC)6	12	36033103	36033114
chr1_6546	p3	(ACA)5	15	36040403	36040417
chr1_6547	p3	(ATA)5	15	36040874	36040888
chr1_6548	p2	(AC)11	22	36046230	36046251

chr1_6549	p2	(GT)16	32	36052791	36052822
chr1_6550	p2	(TG)9	18	36054109	36054126
chr1_6551	p3	(ATT)5	15	36065953	36065967
chr1_6552	p2	(TG)6	12	36074995	36075006
chr1_6553	p2	(CA)14	28	36077251	36077278
chr1_6554	p2	(CA)8	16	36078282	36078297
chr1_6555	p2	(AC)11	22	36082699	36082720
chr1_6556	p2	(TG)6	12	36084833	36084844
chr1_6557	p2	(TG)6	12	36090207	36090218
chr1_6558	p2	(CT)17	34	36092563	36092596
chr1_6559	p3	(TGA)8	24	36095072	36095095
chr1_6560	p2	(AC)6	12	36099358	36099369
chr1_6561	p2	(AC)6	12	36104654	36104665
chr1_6562	p2	(CA)6	12	36106218	36106229
chr1_6563	c	ggtcaaaaggtgacattttatgacttccctttcaaataggaatatgactgttg	118	36113174	36113291
chr1_6564	c	(GT)7gg(GT)15	46	36117259	36117304
chr1_6565	p2	(AC)9	18	36119910	36119927
chr1_6566	p2	(GT)12	24	36120147	36120170
chr1_6567	p2	(AG)6	12	36121845	36121856
chr1_6568	c	aaccgtatccttaccaacacatatttatgtttgtagggatgtgtccgtttccttg	159	36134079	36134237
chr1_6569	p2	(GT)11	22	36136001	36136022
chr1_6570	p3	(TAT)5	15	36145297	36145311
chr1_6571	p2	(AC)6	12	36147484	36147495
chr1_6572	p2	(AC)10	20	36147720	36147739
chr1_6573	p4	(AATG)5	20	36194289	36194308
chr1_6574	p3	(ATT)5	15	36195822	36195836
chr1_6575	p2	(TG)20	40	36200555	36200594
chr1_6576	p2	(AC)6	12	36205250	36205261
chr1_6577	p2	(AC)13	26	36218870	36218895
chr1_6578	p3	(GTG)5	15	36226970	36226984
chr1_6579	p2	(TC)8	16	36227823	36227838
chr1_6580	p3	(AAT)6	18	36232804	36232821
chr1_6581	p2	(CT)7	14	36247709	36247722
chr1_6582	p2	(TG)9	18	36248002	36248019
chr1_6583	c	(AC)12g(CA)9	43	36248412	36248454
chr1_6584	p2	(AC)7	14	36251833	36251846
chr1_6585	p2	(TG)10	20	36253317	36253336
chr1_6586	p4	(ATTG)6	24	36254066	36254089
chr1_6587	p5	(AGTTG)5	25	36261633	36261657
chr1_6588	p2	(TG)11	22	36283282	36283303
chr1_6589	p2	(AT)8	16	36301092	36301107
chr1_6590	p2	(CA)14	28	36304066	36304093
chr1_6591	p2	(AC)6	12	36309397	36309408
chr1_6592	p2	(AG)7	14	36314763	36314776
chr1_6593	p4	(AATC)5	20	36340978	36340997
chr1_6594	p2	(CT)6	12	36367885	36367896
chr1_6595	p2	(GT)16	32	36369200	36369231
chr1_6596	p2	(CA)7	14	36369994	36370007
chr1_6597	p2	(GT)9	18	36370211	36370228
chr1_6598	p2	(CT)6	12	36370361	36370372
chr1_6599	p3	(TCT)5	15	36374744	36374758
chr1_6600	p2	(TG)6	12	36426852	36426863

chr1_6601	c	ctctctgcgtnnnnnnnnnnnnnnnnnnnnnnnnnnnncctgttgaga	133	36449688	36449820
chr1_6602	p2	(AC)7	14	36472164	36472177
chr1_6603	p2	(TG)6	12	36476377	36476388
chr1_6604	p2	(AC)6	12	36484290	36484301
chr1_6605	p2	(GT)18	36	36484885	36484920
chr1_6606	p2	(CA)10	20	36485850	36485869
chr1_6607	p2	(TG)22	44	36485993	36486036
chr1_6608	p2	(AC)13	26	36488401	36488426
chr1_6609	p3	(TTA)5	15	36497271	36497285
chr1_6610	c	6aaatggctttatacgtgcagattgcgcagcacgcatttgtgatccccatg(	74	36505109	36505182
chr1_6611	c	(TA)6gaa(AG)23	61	36536104	36536164
chr1_6612	p2	(TA)6	12	36539844	36539855
chr1_6613	p2	(TG)14	28	36540675	36540702
chr1_6614	p3	(TTC)8	24	36543978	36544001
chr1_6615	c*	(TC)6tttctcactcg(CTCA)8(CA)12*	77	36569818	36569894
chr1_6616	p2	(TG)9	18	36586877	36586894
chr1_6617	p2	(CA)7	14	36587042	36587055
chr1_6618	p2	(TG)6	12	36604674	36604685
chr1_6619	p4	(ATGG)6	24	36606116	36606139
chr1_6620	p2	(GT)6	12	36606467	36606478
chr1_6621	c	(TC)6ttctgaagttcctcaagttcatcaagatccctc(CA)10	65	36623477	36623541
chr1_6622	p3	(CAG)5	15	36625592	36625606
chr1_6623	p2	(CA)6	12	36643207	36643218
chr1_6624	c	(TG)15ggtgtgtgtgt(GA)11	63	36645924	36645986
chr1_6625	p2	(AC)7	14	36651099	36651112
chr1_6626	p2	(CA)7	14	36663817	36663830
chr1_6627	p3	(ATA)5	15	36667455	36667469
chr1_6628	p3	(ATA)5	15	36669857	36669871
chr1_6629	p2	(TG)6	12	36670773	36670784
chr1_6630	p2	(GT)15	30	36675510	36675539
chr1_6631	p2	(TG)22	44	36677900	36677943
chr1_6632	c	(TTA)6actgaaaaac(TG)14	56	36681269	36681324
chr1_6633	p2	(TG)15	30	36694825	36694854
chr1_6634	p2	(TG)6	12	36696794	36696805
chr1_6635	p2	(GT)19	38	36700422	36700459
chr1_6636	p2	(CA)6	12	36710188	36710199
chr1_6637	p2	(TG)6	12	36714890	36714901
chr1_6638	p3	(TTA)6	18	36715672	36715689
chr1_6639	p4	(AAAG)7	28	36718688	36718715
chr1_6640	p2	(TG)17	34	36727430	36727463
chr1_6641	p3	(TTA)7	21	36728412	36728432
chr1_6642	p2	(TC)14	28	36757553	36757580
chr1_6643	p2	(GT)6	12	36769263	36769274
chr1_6644	p3	(ATA)10	30	36777136	36777165
chr1_6645	p2	(CA)9	18	36794181	36794198
chr1_6646	p2	(AG)8	16	36807471	36807486
chr1_6647	p2	(TG)6	12	36811057	36811068
chr1_6648	p4	(ACAT)9	36	36818795	36818830
chr1_6649	p4	(ACAG)5	20	36836068	36836087
chr1_6650	p3	(TAT)5	15	36856403	36856417
chr1_6651	p4	(AATC)5	20	36859166	36859185
chr1_6652	p2	(CA)6	12	36873176	36873187

chr1_6653	p2	(AC)12	24	36876156	36876179
chr1_6654	p2	(GT)22	44	36911174	36911217
chr1_6655	p3	(AAT)6	18	36922162	36922179
chr1_6656	p3	(AAT)5	15	36922324	36922338
chr1_6657	p3	(TTA)7	21	36943691	36943711
chr1_6658	p2	(CA)9	18	36945289	36945306
chr1_6659	c	(TG)7aga(GT)6(GA)20	69	36957098	36957166
chr1_6660	p2	(AC)7	14	36960174	36960187
chr1_6661	p2	(GT)8	16	36964372	36964387
chr1_6662	p2	(TG)11	22	36965224	36965245
chr1_6663	p5	(TCTAA)7	35	36978515	36978549
chr1_6664	p2	(TG)6	12	36995583	36995594
chr1_6665	p2	(AT)6	12	36996301	36996312
chr1_6666	p2	(CA)7	14	37009234	37009247
chr1_6667	p4	(TACA)7	28	37026946	37026973
chr1_6668	p2	(CA)6	12	37028159	37028170
chr1_6669	p2	(TA)15	30	37030611	37030640
chr1_6670	p2	(AC)6	12	37033011	37033022
chr1_6671	c	ιC)5cag(CA)6gtcacttatttacacaatgttagtaaatcctacacctt(C	86	37039833	37039918
chr1_6672	p2	(CA)15	30	37072530	37072559
chr1_6673	c	(TA)11a(AC)23	69	37077563	37077631
chr1_6674	p2	(AC)11	22	37080942	37080963
chr1_6675	p2	(TC)12	24	37099630	37099653
chr1_6676	p2	(AT)10	20	37124218	37124237
chr1_6677	p2	(CA)6	12	37127510	37127521
chr1_6678	p2	(AC)7	14	37146457	37146470
chr1_6679	c	(AT)6aaattatggtagaaaagcattcatagtg(AT)12	64	37155448	37155511
chr1_6680	p2	(CA)6	12	37156380	37156391
chr1_6681	p3	(AAT)5	15	37157118	37157132
chr1_6682	p4	(GAAA)5	20	37171743	37171762
chr1_6683	p3	(AAT)5	15	37185340	37185354
chr1_6684	p2	(AC)15	30	37186789	37186818
chr1_6685	p2	(TA)6	12	37188193	37188204
chr1_6686	p3	(TGA)5	15	37197053	37197067
chr1_6687	c	(GT)13(GA)16	58	37199922	37199979
chr1_6688	p2	(GT)17	34	37204736	37204769
chr1_6689	p2	(TG)10	20	37205640	37205659
chr1_6690	p2	(TC)15	30	37210812	37210841
chr1_6691	p4	(TAGG)6	24	37214699	37214722
chr1_6692	p3	(ATT)5	15	37224620	37224634
chr1_6693	p2	(AC)10	20	37238214	37238233
chr1_6694	p2	(AC)17	34	37239021	37239054
chr1_6695	p2	(AC)20	40	37252427	37252466
chr1_6696	p2	(AC)6	12	37259772	37259783
chr1_6697	p5	(TGATT)5	25	37260157	37260181
chr1_6698	p2	(TG)14	28	37273796	37273823
chr1_6699	p2	(AC)21	42	37287539	37287580
chr1_6700	p2	(AC)9	18	37304026	37304043
chr1_6701	p2	(TG)9	18	37305431	37305448
chr1_6702	p2	(AC)11	22	37305790	37305811
chr1_6703	p2	(GT)11	22	37306630	37306651
chr1_6704	c	:accctgatcttgatcaaggtgcacagaacaatggggtggcctgagttgcaş	94	37307995	37308088

chr1_6705	p2	(TG)6	12	37308770	37308781
chr1_6706	p2	(TG)8	16	37310807	37310822
chr1_6707	p2	(AC)25	50	37320035	37320084
chr1_6708	p3	(AAT)5	15	37320457	37320471
chr1_6709	c	(TA)7(CA)21	56	37338458	37338513
chr1_6710	p4	(ATTG)6	24	37348237	37348260
chr1_6711	p3	(AAT)8	24	37352945	37352968
chr1_6712	p3	(ATA)8	24	37362706	37362729
chr1_6713	p2	(CA)12	24	37370402	37370425
chr1_6714	p2	(TG)14	28	37381881	37381908
chr1_6715	p2	(AG)12	24	37383233	37383256
chr1_6716	c	(TA)6(TG)28	68	37383746	37383813
chr1_6717	p2	(GT)6	12	37393162	37393173
chr1_6718	p3	(ATT)5	15	37420102	37420116
chr1_6719	p3	(AGC)5	15	37420889	37420903
chr1_6720	p2	(AT)10	20	37426577	37426596
chr1_6721	p2	(GT)8	16	37434137	37434152
chr1_6722	p2	(GA)13	26	37445942	37445967
chr1_6723	p2	(GA)12	24	37446353	37446376
chr1_6724	c	(TG)20(AG)12	64	37448064	37448127
chr1_6725	p2	(GT)8	16	37450330	37450345
chr1_6726	p3	(TTG)11	33	37457224	37457256
chr1_6727	p3	(TTG)7	21	37461973	37461993
chr1_6728	p2	(AT)6	12	37465086	37465097
chr1_6729	p3	(CAT)5	15	37499880	37499894
chr1_6730	p2	(CA)8	16	37516854	37516869
chr1_6731	p2	(CT)12	24	37517321	37517344
chr1_6732	p2	(CT)12	24	37518537	37518560
chr1_6733	p2	(AC)21	42	37521291	37521332
chr1_6734	p2	(CA)7	14	37529145	37529158
chr1_6735	p2	(AC)19	38	37534541	37534578
chr1_6736	p3	(TTA)9	27	37534950	37534976
chr1_6737	p2	(AC)15	30	37536861	37536890
chr1_6738	p3	(TTA)7	21	37542108	37542128
chr1_6739	p3	(CTT)6	18	37593846	37593863
chr1_6740	p3	(TAT)5	15	37600686	37600700
chr1_6741	p3	(TTA)5	15	37608789	37608803
chr1_6742	p3	(TTA)5	15	37613796	37613810
chr1_6743	p2	(CA)18	36	37630275	37630310
chr1_6744	p2	(GT)7	14	37643942	37643955
chr1_6745	p2	(AC)7	14	37644881	37644894
chr1_6746	p2	(TG)13	26	37651313	37651338
chr1_6747	p2	(TG)17	34	37652468	37652501
chr1_6748	p3	(TTA)7	21	37656316	37656336
chr1_6749	p3	(ATA)5	15	37669585	37669599
chr1_6750	p3	(ATA)5	15	37672390	37672404
chr1_6751	p2	(AT)7	14	37677206	37677219
chr1_6752	p4	(ATGG)5	20	37700700	37700719
chr1_6753	p2	(CA)7	14	37701123	37701136
chr1_6754	p2	(TG)6	12	37704094	37704105
chr1_6755	p2	(TC)6	12	37718896	37718907
chr1_6756	p2	(TC)7	14	37719630	37719643

chr1_6757	c	gctctctcaaacacacacatacaccctacttgtttttcctactacaagggga	201	37731702	37731902
chr1_6758	p2	(AC)8	16	37737935	37737950
chr1_6759	p3	(ATT)9	27	37750707	37750733
chr1_6760	p5	(TTGAT)5	25	37757312	37757336
chr1_6761	p2	(AC)6	12	37763433	37763444
chr1_6762	p2	(AT)6	12	37768981	37768992
chr1_6763	p2	(GT)9	18	37772162	37772179
chr1_6764	p2	(TG)12	24	37774145	37774168
chr1_6765	p5	(GATTT)5	25	37779251	37779275
chr1_6766	p2	(AC)18	36	37786954	37786989
chr1_6767	p2	(GA)7	14	37803942	37803955
chr1_6768	p3	(TAT)6	18	37806429	37806446
chr1_6769	p2	(CA)8	16	37807709	37807724
chr1_6770	p3	(TAA)5	15	37808371	37808385
chr1_6771	p2	(AC)14	28	37830004	37830031
chr1_6772	p2	(TG)19	38	37871289	37871326
chr1_6773	p2	(TG)20	40	37876494	37876533
chr1_6774	p3	(TAA)5	15	37882019	37882033
chr1_6775	p3	(CTT)6	18	37898257	37898274
chr1_6776	p4	(TTTA)10	40	37898636	37898675
chr1_6777	p3	(TTG)14	42	37898778	37898819
chr1_6778	p3	(TAT)5	15	37899977	37899991
chr1_6779	p3	(AAT)5	15	37901156	37901170
chr1_6780	p2	(TC)6	12	37921604	37921615
chr1_6781	p2	(CA)27	54	37923339	37923392
chr1_6782	p2	(GT)8	16	37931244	37931259
chr1_6783	p3	(TTC)5	15	37931640	37931654
chr1_6784	p2	(GT)6	12	37978164	37978175
chr1_6785	p2	(AG)12	24	37980838	37980861
chr1_6786	p2	(TG)8	16	37990540	37990555
chr1_6787	p2	(AC)8	16	38001058	38001073
chr1_6788	p2	(AT)18	36	38013645	38013680
chr1_6789	p2	(CA)6	12	38020368	38020379
chr1_6790	p2	(CA)20	40	38029779	38029818
chr1_6791	p2	(CT)6	12	38031713	38031724
chr1_6792	p2	(AC)6	12	38034784	38034795
chr1_6793	p3	(TTG)5	15	38035172	38035186
chr1_6794	p2	(AC)16	32	38038093	38038124
chr1_6795	p2	(TC)8	16	38039868	38039883
chr1_6796	p2	(GT)8	16	38046574	38046589
chr1_6797	p2	(AC)8	16	38048621	38048636
chr1_6798	p3	(ATT)5	15	38052415	38052429
chr1_6799	p2	(TG)6	12	38066677	38066688
chr1_6800	p3	(TTA)6	18	38067164	38067181
chr1_6801	p2	(AC)9	18	38097957	38097974
chr1_6802	p2	(GA)6	12	38118352	38118363
chr1_6803	p3	(CTT)6	18	38120797	38120814
chr1_6804	p3	(CTT)6	18	38121680	38121697
chr1_6805	p2	(CT)10	20	38126214	38126233
chr1_6806	p3	(TAA)5	15	38126509	38126523
chr1_6807	p2	(GT)18	36	38133641	38133676
chr1_6808	p2	(GT)6	12	38143899	38143910

chr1_6809	p2	(AC)6	12	38153054	38153065
chr1_6810	p2	(GT)6	12	38163645	38163656
chr1_6811	p2	(AC)8	16	38170140	38170155
chr1_6812	c	:accaatcaacctaagctgcatgtctttggactgtgggaggaaaccggagt:	136	38175227	38175362
chr1_6813	p2	(CA)18	36	38175628	38175663
chr1_6814	p2	(CT)6	12	38182301	38182312
chr1_6815	p3	(TCT)5	15	38186663	38186677
chr1_6816	p2	(AC)11	22	38195990	38196011
chr1_6817	c	TTGA)5tttattctttcaaacatgcagaacagaacaaatgacagca(AC	77	38196777	38196853
chr1_6818	p2	(TC)6	12	38214684	38214695
chr1_6819	p2	(AC)9	18	38214921	38214938
chr1_6820	p2	(GA)6	12	38216863	38216874
chr1_6821	p2	(AG)8	16	38218023	38218038
chr1_6822	p3	(AAG)5	15	38218555	38218569
chr1_6823	p2	(CA)6	12	38224677	38224688
chr1_6824	p2	(GT)6	12	38230084	38230095
chr1_6825	p4	(TCCA)6	24	38239097	38239120
chr1_6826	p2	(TG)6	12	38244471	38244482
chr1_6827	p3	(CCT)8	24	38250805	38250828
chr1_6828	p3	(TAT)6	18	38256172	38256189
chr1_6829	p2	(AC)6	12	38261211	38261222
chr1_6830	p2	(AC)7	14	38266205	38266218
chr1_6831	p2	(TA)7	14	38266969	38266982
chr1_6832	p2	(AC)6	12	38276501	38276512
chr1_6833	p3	(TAT)5	15	38282685	38282699
chr1_6834	p5	(TTTGA)5	25	38293384	38293408
chr1_6835	p2	(AC)7	14	38293610	38293623
chr1_6836	p3	(AAT)6	18	38295258	38295275
chr1_6837	p3	(CTT)6	18	38298272	38298289
chr1_6838	p3	(TTG)5	15	38305859	38305873
chr1_6839	p2	(GA)6	12	38306781	38306792
chr1_6840	p2	(TC)7	14	38312401	38312414
chr1_6841	p2	(CA)6	12	38314156	38314167
chr1_6842	p2	(AC)8	16	38318847	38318862
chr1_6843	p2	(AC)13	26	38319682	38319707
chr1_6844	p2	(CA)6	12	38323845	38323856
chr1_6845	p2	(CA)11	22	38328982	38329003
chr1_6846	p2	(GT)6	12	38340202	38340213
chr1_6847	p2	(AC)6	12	38357083	38357094
chr1_6848	c	5gcgcgtttcagacactgataagagctcacaggggtttcaaggactcaac(	73	38382273	38382345
chr1_6849	p2	(AC)7	14	38448253	38448266
chr1_6850	p3	(TTA)5	15	38460826	38460840
chr1_6851	p2	(AC)40	80	38466723	38466802
chr1_6852	p2	(CA)16	32	38469993	38470024
chr1_6853	p2	(CA)8	16	38490314	38490329
chr1_6854	p2	(GT)17	34	38491046	38491079
chr1_6855	p3	(AAT)6	18	38496125	38496142
chr1_6856	p2	(AC)6	12	38517480	38517491
chr1_6857	p2	(CA)6	12	38525351	38525362
chr1_6858	p2	(GA)6	12	38541482	38541493
chr1_6859	p2	(TG)6	12	38541735	38541746
chr1_6860	p2	(AC)10	20	38544380	38544399

chr1_6861	p2	(TC)7	14	38547995	38548008
chr1_6862	p2	(TG)14	28	38553563	38553590
chr1_6863	p2	(AC)14	28	38565055	38565082
chr1_6864	p2	(AC)13	26	38571930	38571955
chr1_6865	p3	(CTT)6	18	38598284	38598301
chr1_6866	p2	(GT)6	12	38599057	38599068
chr1_6867	p2	(AG)7	14	38600828	38600841
chr1_6868	p5	(AATCT)7	35	38607610	38607644
chr1_6869	p4	(AAAT)5	20	38608158	38608177
chr1_6870	p2	(GT)9	18	38617655	38617672
chr1_6871	p2	(AC)7	14	38622731	38622744
chr1_6872	p2	(TG)7	14	38631170	38631183
chr1_6873	p2	(GT)9	18	38631441	38631458
chr1_6874	p2	(AC)20	40	38639290	38639329
chr1_6875	p2	(TG)6	12	38642652	38642663
chr1_6876	c	tgggtcacaccctcaaatgaggactgtcacagtttgtatactttgtgtgtgcat	170	38653811	38653980
chr1_6877	p2	(AC)10	20	38655771	38655790
chr1_6878	p2	(AC)11	22	38666327	38666348
chr1_6879	p4	(CATC)5	20	38667011	38667030
chr1_6880	p2	(TG)7	14	38670222	38670235
chr1_6881	p2	(CA)6	12	38672701	38672712
chr1_6882	p2	(AC)6	12	38675999	38676010
chr1_6883	p4	(AATC)8	32	38680820	38680851
chr1_6884	p2	(CA)6	12	38682552	38682563
chr1_6885	p2	(GT)6	12	38685422	38685433
chr1_6886	p2	(AC)6	12	38688357	38688368
chr1_6887	p2	(AT)6	12	38698619	38698630
chr1_6888	c	(GT)6ggacatgggatttttaacgtgaatgctttgtaggta(TG)7	62	38701287	38701348
chr1_6889	p2	(CA)17	34	38702131	38702164
chr1_6890	p2	(TG)8	16	38725334	38725349
chr1_6891	p2	(CA)10	20	38728672	38728691
chr1_6892	p2	(CA)21	42	38743938	38743979
chr1_6893	p2	(TG)32	64	38748253	38748316
chr1_6894	p2	(TG)9	18	38753429	38753446
chr1_6895	p3	(ATT)6	18	38758153	38758170
chr1_6896	p2	(AC)7	14	38763581	38763594
chr1_6897	p2	(TG)8	16	38776637	38776652
chr1_6898	p4	(TTTG)6	24	38779849	38779872
chr1_6899	p2	(TA)9	18	38781886	38781903
chr1_6900	p2	(TC)7	14	38786777	38786790
chr1_6901	p2	(CA)12	24	38788128	38788151
chr1_6902	p2	(CA)20	40	38788337	38788376
chr1_6903	p2	(AC)10	20	38788534	38788553
chr1_6904	p2	(AC)6	12	38789903	38789914
chr1_6905	p2	(TC)7	14	38791126	38791139
chr1_6906	p2	(AC)9	18	38792389	38792406
chr1_6907	p2	(AG)8	16	38794062	38794077
chr1_6908	p2	(TC)26	52	38794633	38794684
chr1_6909	p2	(CA)6	12	38795781	38795792
chr1_6910	p3	(ATA)6	18	38796996	38797013
chr1_6911	p2	(GT)6	12	38797633	38797644
chr1_6912	p2	(GA)6	12	38800576	38800587

chr1_6913	c	(GT)17c(TG)6	47	38809130	38809176
chr1_6914	p2	(CA)6	12	38811653	38811664
chr1_6915	p2	(TG)13	26	38812034	38812059
chr1_6916	p2	(AC)6	12	38822423	38822434
chr1_6917	p3	(TTA)6	18	38823912	38823929
chr1_6918	c	(TC)6tttctctctctctgtct(CA)7	43	38837301	38837343
chr1_6919	p2	(TG)6	12	38842726	38842737
chr1_6920	p2	(CA)31	62	38879575	38879636
chr1_6921	p2	(CA)7	14	38879982	38879995
chr1_6922	p3	(ATT)5	15	38884990	38885004
chr1_6923	p2	(AC)6	12	38888977	38888988
chr1_6924	p2	(AC)9	18	38889206	38889223
chr1_6925	p3	(CCT)5	15	38907951	38907965
chr1_6926	p2	(AC)11	22	38908229	38908250
chr1_6927	p3	(ATT)5	15	38909305	38909319
chr1_6928	p2	(AG)20	40	38914132	38914171
chr1_6929	p2	(TC)6	12	38970802	38970813
chr1_6930	p2	(TG)23	46	38975211	38975256
chr1_6931	p2	(TG)6	12	38975506	38975517
chr1_6932	p2	(CA)20	40	38976273	38976312
chr1_6933	p3	(TTA)5	15	38993015	38993029
chr1_6934	p2	(CA)14	28	38994810	38994837
chr1_6935	p2	(GT)14	28	39020185	39020212
chr1_6936	p3	(ATA)7	21	39033931	39033951
chr1_6937	p2	(CA)16	32	39038118	39038149
chr1_6938	c	(CT)6(AT)8	28	39040318	39040345
chr1_6939	p3	(CCT)7	21	39060258	39060278
chr1_6940	p2	(TG)7	14	39068600	39068613
chr1_6941	p3	(AGC)5	15	39070802	39070816
chr1_6942	p2	(AC)10	20	39074021	39074040
chr1_6943	p2	(TG)7	14	39076011	39076024
chr1_6944	p3	(CTT)7	21	39081047	39081067
chr1_6945	p3	(CCT)5	15	39083206	39083220
chr1_6946	p3	(TTA)5	15	39099955	39099969
chr1_6947	p4	(TCTA)7	28	39101896	39101923
chr1_6948	p2	(GT)7	14	39106532	39106545
chr1_6949	p3	(TTG)6	18	39110801	39110818
chr1_6950	p2	(GT)8	16	39136075	39136090
chr1_6951	p2	(AG)6	12	39138780	39138791
chr1_6952	p2	(GT)7	14	39148745	39148758
chr1_6953	p2	(TG)13	26	39161933	39161958
chr1_6954	p2	(AG)9	18	39163272	39163289
chr1_6955	p3	(CCT)10	30	39163491	39163520
chr1_6956	p2	(GT)6	12	39184275	39184286
chr1_6957	p2	(TC)8	16	39191803	39191818
chr1_6958	p4	(ATTG)5	20	39200442	39200461
chr1_6959	p2	(TG)6	12	39210087	39210098
chr1_6960	c	[illegible]gaaccctgctatatttagtcagattcgggaggtttcatagtgctataggttgac[illegible]	89	39212960	39213048
chr1_6961	c	(TCT)6tca(TCC)6	39	39217773	39217811
chr1_6962	p2	(GT)7	14	39219513	39219526
chr1_6963	p4	(GATG)5	20	39221186	39221205
chr1_6964	p2	(AT)6	12	39234544	39234555

chr1_6965	p2	(AT)6	12	39234693	39234704
chr1_6966	p2	(TA)6	12	39239440	39239451
chr1_6967	p4	(TTTG)7	28	39241270	39241297
chr1_6968	p2	(TG)14	28	39250874	39250901
chr1_6969	p5	(ATTCT)6	30	39252965	39252994
chr1_6970	c	(TG)10c(GT)14	49	39257245	39257293
chr1_6971	p2	(CT)9	18	39258047	39258064
chr1_6972	p2	(AC)9	18	39258744	39258761
chr1_6973	p2	(AC)7	14	39279330	39279343
chr1_6974	p3	(TAA)5	15	39280580	39280594
chr1_6975	p2	(TG)9	18	39283609	39283626
chr1_6976	p2	(AG)11	22	39284250	39284271
chr1_6977	p2	(TG)7	14	39287168	39287181
chr1_6978	p2	(CT)7	14	39293153	39293166
chr1_6979	p2	(AC)11	22	39294183	39294204
chr1_6980	p4	(ATTC)5	20	39296708	39296727
chr1_6981	p3	(TAT)5	15	39296863	39296877
chr1_6982	p2	(TC)11	22	39298938	39298959
chr1_6983	p2	(CT)7	14	39303026	39303039
chr1_6984	p2	(GT)13	26	39305459	39305484
chr1_6985	p2	(TG)7	14	39313356	39313369
chr1_6986	p2	(CA)8	16	39325696	39325711
chr1_6987	p2	(CA)6	12	39330471	39330482
chr1_6988	p2	(GT)6	12	39331308	39331319
chr1_6989	p2	(AC)18	36	39333034	39333069
chr1_6990	p2	(TG)14	28	39346795	39346822
chr1_6991	p2	(AT)15	30	39374354	39374383
chr1_6992	p3	(TTA)5	15	39380047	39380061
chr1_6993	p3	(TTA)5	15	39381735	39381749
chr1_6994	c	(TG)12tc(TG)10tctgtg(TC)6	64	39391156	39391219
chr1_6995	p2	(CA)9	18	39404934	39404951
chr1_6996	c	3tcatgaggatcacaaatgcgtgctgcgcaatctgcacgtataaagccatt(	100	39407639	39407738
chr1_6997	p2	(TG)9	18	39413268	39413285
chr1_6998	p2	(TG)11	22	39420428	39420449
chr1_6999	p2	(AC)12	24	39423064	39423087
chr1_7000	p2	(CA)12	24	39426495	39426518
chr1_7001	p2	(TG)8	16	39437268	39437283
chr1_7002	p2	(TG)17	34	39448140	39448173
chr1_7003	c*	(TA)19(TAGA)7*	64	39450507	39450570
chr1_7004	p3	(TGT)5	15	39453184	39453198
chr1_7005	p2	(TG)15	30	39463293	39463322
chr1_7006	p2	(CA)6	12	39469893	39469904
chr1_7007	p2	(CA)7	14	39473720	39473733
chr1_7008	p2	(AC)7	14	39478758	39478771
chr1_7009	c*	(GAGT)5(GT)6*	30	39479116	39479145
chr1_7010	c	(TC)6tgt(CA)6	27	39487608	39487634
chr1_7011	p2	(TC)6	12	39499172	39499183
chr1_7012	p2	(AC)7	14	39513251	39513264
chr1_7013	p3	(ATT)10	30	39538665	39538694
chr1_7014	c	(TAT)6gatgatactgataatgaagaagaagatgatgatactg(ATT)7	76	39551915	39551990
chr1_7015	p3	(CCT)7	21	39558301	39558321
chr1_7016	p4	(ATAA)5	20	39559355	39559374

chr1_7017	p2	(AC)37	74	39560950	39561023
chr1_7018	p2	(GT)7	14	39561668	39561681
chr1_7019	p2	(GA)8	16	39561839	39561854
chr1_7020	p2	(TG)13	26	39562424	39562449
chr1_7021	c	(CT)6(CA)6	24	39567909	39567932
chr1_7022	p2	(CA)12	24	39571371	39571394
chr1_7023	p2	(TG)10	20	39571761	39571780
chr1_7024	p2	(TC)10	20	39606678	39606697
chr1_7025	p2	(AT)7	14	39608468	39608481
chr1_7026	p2	(AT)7	14	39630000	39630013
chr1_7027	p2	(TG)6	12	39632528	39632539
chr1_7028	p2	(TC)7	14	39636972	39636985
chr1_7029	p2	(GT)7	14	39641996	39642009
chr1_7030	p2	(AC)17	34	39642896	39642929
chr1_7031	p2	(TA)7	14	39643296	39643309
chr1_7032	p2	(GT)9	18	39643510	39643527
chr1_7033	p2	(TG)6	12	39650153	39650164
chr1_7034	p3	(AAT)5	15	39652924	39652938
chr1_7035	p4	(AATA)5	20	39661708	39661727
chr1_7036	p2	(TG)6	12	39681871	39681882
chr1_7037	p2	(TG)10	20	39685570	39685589
chr1_7038	p2	(AT)9	18	39692358	39692375
chr1_7039	p2	(TG)8	16	39694214	39694229
chr1_7040	p2	(CA)14	28	39696792	39696819
chr1_7041	c	(AC)8at(AC)6	30	39696988	39697017
chr1_7042	p2	(AC)7	14	39707948	39707961
chr1_7043	p2	(AG)10	20	39716025	39716044
chr1_7044	p2	(GT)6	12	39716900	39716911
chr1_7045	p2	(AT)9	18	39718434	39718451
chr1_7046	p2	(AT)11	22	39720128	39720149
chr1_7047	p2	(TG)7	14	39723396	39723409
chr1_7048	p3	(CCA)5	15	39729353	39729367
chr1_7049	p2	(AC)16	32	39750275	39750306
chr1_7050	p2	(TG)8	16	39752648	39752663
chr1_7051	p2	(CA)17	34	39758659	39758692
chr1_7052	p2	(TG)12	24	39759632	39759655
chr1_7053	p2	(TA)6	12	39760057	39760068
chr1_7054	p5	(TTTGA)5	25	39763020	39763044
chr1_7055	p2	(AC)13	26	39788298	39788323
chr1_7056	p2	(CA)11	22	39789395	39789416
chr1_7057	p3	(AAT)5	15	39809755	39809769
chr1_7058	p2	(AT)6	12	39845077	39845088
chr1_7059	p2	(AT)6	12	39847102	39847113
chr1_7060	p2	(TA)6	12	39854763	39854774
chr1_7061	p3	(AAT)5	15	39881457	39881471
chr1_7062	p2	(CA)10	20	39893816	39893835
chr1_7063	p2	(TG)15	30	39899028	39899057
chr1_7064	p2	(TA)8	16	39904575	39904590
chr1_7065	p2	(AC)24	48	39905264	39905311
chr1_7066	p2	(AC)6	12	39908045	39908056
chr1_7067	p2	(TC)6	12	39918597	39918608
chr1_7068	p3	(TCC)5	15	39928909	39928923

chr1_7069	p2	(GA)6	12	39943087	39943098
chr1_7070	p3	(AAT)7	21	39944159	39944179
chr1_7071	p3	(ATT)13	39	39954949	39954987
chr1_7072	p3	(AAT)5	15	39968257	39968271
chr1_7073	p3	(CAC)7	21	39969209	39969229
chr1_7074	p2	(CA)6	12	39969823	39969834
chr1_7075	p2	(GT)11	22	39970038	39970059
chr1_7076	p2	(AC)11	22	39979422	39979443
chr1_7077	p2	(GT)11	22	39989868	39989889
chr1_7078	p2	(AC)9	18	40004901	40004918
chr1_7079	c	(AGG)5g(GA)6	28	40014780	40014807
chr1_7080	p2	(TA)6	12	40016820	40016831
chr1_7081	p3	(ATA)7	21	40026217	40026237
chr1_7082	p3	(AAG)6	18	40028189	40028206
chr1_7083	p2	(CA)8	16	40034639	40034654
chr1_7084	p2	(CA)7	14	40049860	40049873
chr1_7085	p2	(AC)7	14	40052062	40052075
chr1_7086	p3	(ATC)6	18	40055894	40055911
chr1_7087	p4	(AGAT)11	44	40061484	40061527
chr1_7088	p4	(TTAC)6	24	40075575	40075598
chr1_7089	p2	(TA)6	12	40086515	40086526
chr1_7090	p2	(TA)9	18	40087028	40087045
chr1_7091	p2	(TG)8	16	40090446	40090461
chr1_7092	p2	(GA)23	46	40090841	40090886
chr1_7093	p3	(ACT)5	15	40092099	40092113
chr1_7094	p2	(CA)7	14	40092622	40092635
chr1_7095	p2	(CA)6	12	40093817	40093828
chr1_7096	p4	(ATGG)5	20	40103803	40103822
chr1_7097	p2	(CT)6	12	40122128	40122139
chr1_7098	p2	(AC)6	12	40126182	40126193
chr1_7099	p2	(AC)19	38	40137120	40137157
chr1_7100	p2	(CA)6	12	40149209	40149220
chr1_7101	p2	(CT)7	14	40166160	40166173
chr1_7102	p2	(CA)15	30	40172197	40172226
chr1_7103	p2	(TG)22	44	40175605	40175648
chr1_7104	p3	(TGC)8	24	40176749	40176772
chr1_7105	p2	(AC)22	44	40177277	40177320
chr1_7106	p2	(TG)10	20	40186333	40186352
chr1_7107	p4	(TCTG)5	20	40188386	40188405
chr1_7108	p2	(CA)6	12	40188839	40188850
chr1_7109	p2	(TG)11	22	40192490	40192511
chr1_7110	p4	(TTTG)6	24	40194987	40195010
chr1_7111	p2	(CA)15	30	40197677	40197706
chr1_7112	p2	(TC)10	20	40201277	40201296
chr1_7113	p4	(ATTC)6	24	40204556	40204579
chr1_7114	p3	(CTC)7	21	40224198	40224218
chr1_7115	p2	(TG)6	12	40227221	40227232
chr1_7116	p2	(TA)6	12	40229487	40229498
chr1_7117	p2	(TG)16	32	40247560	40247591
chr1_7118	p2	(TC)6	12	40248368	40248379
chr1_7119	p2	(GT)7	14	40266314	40266327
chr1_7120	p2	(CA)6	12	40275577	40275588

chr1_7121	p3	(TTA)5	15	40283458	40283472
chr1_7122	p2	(TG)10	20	40285090	40285109
chr1_7123	p2	(CT)8	16	40309029	40309044
chr1_7124	p3	(AAT)5	15	40367291	40367305
chr1_7125	p2	(TA)7	14	40372912	40372925
chr1_7126	p2	(GT)13	26	40385024	40385049
chr1_7127	c	(TTC)5(TTA)5	30	40401662	40401691
chr1_7128	p2	(GT)7	14	40402364	40402377
chr1_7129	p3	(TTA)8	24	40403644	40403667
chr1_7130	p2	(TG)7	14	40410728	40410741
chr1_7131	p2	(GT)10	20	40411871	40411890
chr1_7132	p3	(AAG)5	15	40413793	40413807
chr1_7133	p2	(TG)10	20	40419476	40419495
chr1_7134	p2	(TG)11	22	40423116	40423137
chr1_7135	p2	(AC)16	32	40425494	40425525
chr1_7136	p2	(TG)14	28	40427717	40427744
chr1_7137	p2	(CA)24	48	40436411	40436458
chr1_7138	p2	(CA)27	54	40436840	40436893
chr1_7139	p2	(GT)8	16	40438075	40438090
chr1_7140	p2	(GT)17	34	40445460	40445493
chr1_7141	p2	(GT)7	14	40447187	40447200
chr1_7142	p3	(TGT)5	15	40468729	40468743
chr1_7143	c	(GT)6g(GT)30	73	40473755	40473827
chr1_7144	p2	(GA)7	14	40476637	40476650
chr1_7145	p4	(TTCA)6	24	40485838	40485861
chr1_7146	p4	(CATT)5	20	40492029	40492048
chr1_7147	p2	(GT)10	20	40494777	40494796
chr1_7148	p3	(AGA)5	15	40496747	40496761
chr1_7149	p2	(TG)9	18	40498902	40498919
chr1_7150	p4	(TTTA)5	20	40504495	40504514
chr1_7151	p3	(ATT)5	15	40509036	40509050
chr1_7152	p2	(CT)6	12	40510142	40510153
chr1_7153	c	(TC)14a(AT)6	41	40523235	40523275
chr1_7154	p2	(CA)13	26	40531467	40531492
chr1_7155	p2	(AC)13	26	40533539	40533564
chr1_7156	p2	(TG)6	12	40537134	40537145
chr1_7157	p2	(CT)7	14	40540224	40540237
chr1_7158	p2	(CT)7	14	40548073	40548086
chr1_7159	p2	(CA)6	12	40548291	40548302
chr1_7160	p4	(GGTA)5	20	40558718	40558737
chr1_7161	p2	(GT)19	38	40561614	40561651
chr1_7162	c	A)6ctgccaggagacctagctgtactgaaaatagaacagggaagtt(TC	71	40565473	40565543
chr1_7163	p3	(TTC)5	15	40574877	40574891
chr1_7164	p2	(TG)9	18	40583230	40583247
chr1_7165	p4	(GTAG)6	24	40597808	40597831
chr1_7166	p2	(AC)8	16	40631295	40631310
chr1_7167	p2	(CA)6	12	40631660	40631671
chr1_7168	p2	(AT)15	30	40643532	40643561
chr1_7169	p2	(AC)23	46	40646778	40646823
chr1_7170	p2	(GT)6	12	40650195	40650206
chr1_7171	c	(TC)6a(CT)6	25	40652451	40652475
chr1_7172	p4	(GATG)5	20	40670737	40670756

chr1_7173	c	(AC)6g(CA)6	25	40674293	40674317
chr1_7174	p2	(GT)7	14	40682539	40682552
chr1_7175	p2	(CA)6	12	40684551	40684562
chr1_7176	p2	(GA)6	12	40685742	40685753
chr1_7177	p2	(AC)13	26	40700022	40700047
chr1_7178	p3	(TGG)5	15	40705965	40705979
chr1_7179	p2	(CA)9	18	40706118	40706135
chr1_7180	p2	(TG)19	38	40710901	40710938
chr1_7181	p2	(GA)6	12	40715029	40715040
chr1_7182	c	(TG)29(AG)13	84	40730031	40730114
chr1_7183	c	(TG)10(AG)14	48	40730587	40730634
chr1_7184	p2	(AC)6	12	40731732	40731743
chr1_7185	p3	(TGA)15	45	40732629	40732673
chr1_7186	p2	(GT)13	26	40735360	40735385
chr1_7187	p2	(GA)8	16	40739274	40739289
chr1_7188	p2	(GT)9	18	40742472	40742489
chr1_7189	p2	(CA)7	14	40748320	40748333
chr1_7190	p2	(CA)6	12	40748497	40748508
chr1_7191	p2	(TG)10	20	40762441	40762460
chr1_7192	p2	(TG)14	28	40773392	40773419
chr1_7193	p3	(CTT)7	21	40787324	40787344
chr1_7194	p3	(TCT)6	18	40818041	40818058
chr1_7195	p2	(GT)6	12	40818673	40818684
chr1_7196	p3	(CCA)5	15	40828198	40828212
chr1_7197	p5	(TTGAT)5	25	40830485	40830509
chr1_7198	p2	(TG)7	14	40831986	40831999
chr1_7199	p2	(CA)7	14	40832994	40833007
chr1_7200	p3	(ATA)7	21	40835462	40835482
chr1_7201	p2	(TG)8	16	40864866	40864881
chr1_7202	p2	(AG)7	14	40865700	40865713
chr1_7203	p2	(CA)9	18	40867439	40867456
chr1_7204	p2	(GT)6	12	40881894	40881905
chr1_7205	p2	(TG)14	28	40882266	40882293
chr1_7206	p2	(TA)7	14	40882420	40882433
chr1_7207	p2	(TG)7	14	40886489	40886502
chr1_7208	p2	(AC)8	16	40887418	40887433
chr1_7209	c	(AC)16at(AC)6	46	40893077	40893122
chr1_7210	p2	(TG)19	38	40894625	40894662
chr1_7211	p3	(AAC)5	15	40897722	40897736
chr1_7212	p2	(GT)7	14	40903660	40903673
chr1_7213	p2	(TG)8	16	40912290	40912305
chr1_7214	p2	(CA)6	12	40951139	40951150
chr1_7215	p2	(CA)7	14	40960849	40960862
chr1_7216	c	ggggctgtgtgtggcgctgtctgtcctcagtcttcccccacagtgacccccg	134	41017871	41018004
chr1_7217	c	tacagatggcacgtaagcggcagccaacccaacacaatttctactcaaag	146	41019370	41019515
chr1_7218	c	(TG)7tatgtgtg(TA)7	36	41022063	41022098
chr1_7219	p2	(CA)6	12	41029565	41029576
chr1_7220	p3	(CTT)12	36	41032924	41032959
chr1_7221	p2	(GT)6	12	41038660	41038671
chr1_7222	p2	(CA)12	24	41041271	41041294
chr1_7223	p2	(AC)26	52	41046622	41046673
chr1_7224	c	agtggctggacccaatgcactgccacataaatgattataaaggaagttaata	101	41047077	41047177

chr1_7225	p2	(TG)13	26	41056011	41056036
chr1_7226	p2	(TG)14	28	41062488	41062515
chr1_7227	p3	(AAT)5	15	41070398	41070412
chr1_7228	p3	(AAT)6	18	41071343	41071360
chr1_7229	c	(TTA)7c(TAT)6	40	41075447	41075486
chr1_7230	p2	(TC)6	12	41077103	41077114
chr1_7231	p2	(CA)10	20	41078313	41078332
chr1_7232	p2	(TG)7	14	41081972	41081985
chr1_7233	p4	(TTTA)5	20	41084476	41084495
chr1_7234	p2	(CA)23	46	41115768	41115813
chr1_7235	p2	(CT)7	14	41118627	41118640
chr1_7236	p2	(AG)6	12	41145331	41145342
chr1_7237	p2	(GT)6	12	41148280	41148291
chr1_7238	p2	(TG)27	54	41149690	41149743
chr1_7239	p3	(TTA)5	15	41157878	41157892
chr1_7240	p2	(TG)6	12	41173992	41174003
chr1_7241	p5	(AAATC)6	30	41183444	41183473
chr1_7242	p4	(ATCC)9	36	41191104	41191139
chr1_7243	p3	(ATA)6	18	41199521	41199538
chr1_7244	p2	(AC)6	12	41200146	41200157
chr1_7245	p2	(TG)7	14	41204071	41204084
chr1_7246	p2	(GT)6	12	41207438	41207449
chr1_7247	p3	(ATT)5	15	41221505	41221519
chr1_7248	p2	(TG)8	16	41245073	41245088
chr1_7249	p2	(GT)15	30	41246109	41246138
chr1_7250	p3	(TTA)6	18	41249761	41249778
chr1_7251	p2	(GT)8	16	41280414	41280429
chr1_7252	p2	(AC)10	20	41290501	41290520
chr1_7253	p2	(TG)7	14	41307034	41307047
chr1_7254	p3	(ATT)5	15	41341280	41341294
chr1_7255	p2	(GA)6	12	41345302	41345313
chr1_7256	p2	(TG)6	12	41350393	41350404
chr1_7257	p2	(TG)6	12	41354258	41354269
chr1_7258	p3	(TGT)6	18	41358871	41358888
chr1_7259	p2	(AG)6	12	41372803	41372814
chr1_7260	c	[illegible]6aaatggctttatacgtgcagcttgcacagcacgcatttgtgatcctcatg([illegible]	74	41373614	41373687
chr1_7261	p2	(GA)7	14	41375842	41375855
chr1_7262	p3	(AAT)6	18	41376908	41376925
chr1_7263	p2	(CA)9	18	41379735	41379752
chr1_7264	p2	(GT)14	28	41391257	41391284
chr1_7265	c	(AC)11aaatat(AC)16	60	41422678	41422737
chr1_7266	p5	(TTTGA)5	25	41423467	41423491
chr1_7267	p4	(AATG)5	20	41423866	41423885
chr1_7268	p2	(GT)10	20	41459755	41459774
chr1_7269	c	(TG)7agagagt(GA)7	35	41465009	41465043
chr1_7270	p5	(ATTTG)5	25	41466992	41467016
chr1_7271	p3	(TAA)5	15	41467230	41467244
chr1_7272	p2	(GT)15	30	41479467	41479496
chr1_7273	p2	(TG)6	12	41482753	41482764
chr1_7274	p3	(TCA)5	15	41483986	41484000
chr1_7275	p3	(AAT)6	18	41497821	41497838
chr1_7276	c	(CG)6(CA)6	24	41506965	41506988

chr1_7277	p2	(AC)8	16	41514007	41514022
chr1_7278	p2	(GT)7	14	41527802	41527815
chr1_7279	c	(GC)6(AC)6	24	41535707	41535730
chr1_7280	p3	(TCC)6	18	41540562	41540579
chr1_7281	p3	(TGC)8	24	41543933	41543956
chr1_7282	p2	(AC)17	34	41548854	41548887
chr1_7283	p2	(AC)7	14	41556108	41556121
chr1_7284	c	(TCA)5g(CTT)8	40	41564718	41564757
chr1_7285	p4	(TCCA)7	28	41572491	41572518
chr1_7286	p3	(AGC)5	15	41579724	41579738
chr1_7287	p3	(ATG)6	18	41581736	41581753
chr1_7288	p2	(AC)9	18	41587770	41587787
chr1_7289	p3	(GAA)5	15	41588853	41588867
chr1_7290	p2	(GT)7	14	41606083	41606096
chr1_7291	p3	(TCC)10	30	41616685	41616714
chr1_7292	p3	(CTC)5	15	41617130	41617144
chr1_7293	p2	(TG)13	26	41617784	41617809
chr1_7294	p2	(GA)7	14	41619732	41619745
chr1_7295	c	gaagcagccacattgtgtgagagtgtgtttatactgtgcagcattgcatgac	123	41620956	41621078
chr1_7296	p2	(TA)10	20	41628769	41628788
chr1_7297	p2	(AG)6	12	41652840	41652851
chr1_7298	p3	(TTC)5	15	41661555	41661569
chr1_7299	p3	(TCC)8	24	41689456	41689479
chr1_7300	p3	(TCA)7	21	41707656	41707676
chr1_7301	p2	(CA)12	24	41717151	41717174
chr1_7302	p2	(AC)9	18	41749388	41749405
chr1_7303	c	(GT)17atg(TA)8cat(AC)17	90	41761118	41761207
chr1_7304	p4	(AAAT)5	20	41767255	41767274
chr1_7305	p4	(TAGA)8	32	41768863	41768894
chr1_7306	p2	(AC)20	40	41781874	41781913
chr1_7307	p2	(TG)23	46	41786794	41786839
chr1_7308	p2	(GA)6	12	41790446	41790457
chr1_7309	p2	(AT)6	12	41791435	41791446
chr1_7310	p3	(CCA)5	15	41793538	41793552
chr1_7311	p2	(GT)10	20	41798765	41798784
chr1_7312	p3	(TAA)5	15	41806936	41806950
chr1_7313	p2	(TG)7	14	41816343	41816356
chr1_7314	p2	(AC)16	32	41816733	41816764
chr1_7315	p2	(AC)20	40	41839895	41839934
chr1_7316	p2	(GT)6	12	41843635	41843646
chr1_7317	p2	(TG)6	12	41844639	41844650
chr1_7318	p2	(GT)6	12	41850834	41850845
chr1_7319	p2	(CA)6	12	41854033	41854044
chr1_7320	p2	(AC)6	12	41854379	41854390
chr1_7321	p2	(GA)10	20	41855013	41855032
chr1_7322	c	(TC)6ttgct(CA)30	77	41857925	41858001
chr1_7323	p2	(AC)29	58	41861447	41861504
chr1_7324	p2	(GT)6	12	41867920	41867931
chr1_7325	p2	(AC)7	14	41870209	41870222
chr1_7326	p2	(TG)6	12	41874890	41874901
chr1_7327	p5	(GATAT)5	25	41878336	41878360
chr1_7328	p2	(TG)16	32	41883307	41883338

chr1_7329	p2	(GT)8	16	41885087	41885102
chr1_7330	p2	(CT)7	14	41900600	41900613
chr1_7331	p2	(AC)6	12	41911636	41911647
chr1_7332	p4	(TATC)23	92	41913142	41913233
chr1_7333	p2	(TA)8	16	41913529	41913544
chr1_7334	p2	(GT)13	26	41921050	41921075
chr1_7335	p2	(TG)13	26	41922271	41922296
chr1_7336	p2	(GT)7	14	41922419	41922432
chr1_7337	p2	(TG)13	26	41923013	41923038
chr1_7338	c	(TG)11(AG)8	38	41933248	41933285
chr1_7339	c	cagcttgttccatcagttgtcactgaaagaagagcaccaaccagacagcaa	161	41937393	41937553
chr1_7340	p2	(GT)6	12	41943962	41943973
chr1_7341	p2	(TG)8	16	41947182	41947197
chr1_7342	p2	(AC)7	14	41952265	41952278
chr1_7343	p2	(AC)15	30	41953417	41953446
chr1_7344	p2	(TG)8	16	41970307	41970322
chr1_7345	p3	(TTG)6	18	41971335	41971352
chr1_7346	p2	(CA)9	18	41975229	41975246
chr1_7347	p3	(TAT)9	27	41977929	41977955
chr1_7348	p3	(AAT)5	15	41984493	41984507
chr1_7349	p3	(AGC)5	15	41986314	41986328
chr1_7350	p2	(GT)28	56	41990507	41990562
chr1_7351	p2	(CT)11	22	41990686	41990707
chr1_7352	p2	(TG)7	14	41991033	41991046
chr1_7353	p2	(TC)7	14	41992950	41992963
chr1_7354	p2	(GT)12	24	41996023	41996046
chr1_7355	p4	(ATCC)6	24	41999057	41999080
chr1_7356	p4	(TATC)15	60	42007911	42007970
chr1_7357	p3	(TGG)5	15	42008416	42008430
chr1_7358	p2	(TG)8	16	42011948	42011963
chr1_7359	p2	(TG)11	22	42012938	42012959
chr1_7360	p2	(AC)8	16	42024963	42024978
chr1_7361	p2	(TG)6	12	42025414	42025425
chr1_7362	c	(GT)16gagt(GA)7	50	42028038	42028087
chr1_7363	p2	(AC)6	12	42033705	42033716
chr1_7364	p2	(AC)10	20	42045456	42045475
chr1_7365	p5	(AAATC)5	25	42047318	42047342
chr1_7366	p2	(TC)6	12	42049868	42049879
chr1_7367	p2	(AC)7	14	42060890	42060903
chr1_7368	c	(AC)6at(AC)6	26	42061204	42061229
chr1_7369	p2	(AC)6	12	42061367	42061378
chr1_7370	p2	(TG)12	24	42062644	42062667
chr1_7371	p3	(AGG)5	15	42075250	42075264
chr1_7372	p2	(CA)15	30	42089998	42090027
chr1_7373	p2	(TG)8	16	42090534	42090549
chr1_7374	p2	(AC)7	14	42097411	42097424
chr1_7375	p2	(AT)6	12	42131748	42131759
chr1_7376	p3	(CAT)6	18	42134126	42134143
chr1_7377	p3	(GGA)11	33	42140921	42140953
chr1_7378	p2	(TA)6	12	42171873	42171884
chr1_7379	p3	(TTA)9	27	42180494	42180520
chr1_7380	p2	(TA)7	14	42190867	42190880

chr1_7381	p3	(ATT)5	15	42192327	42192341
chr1_7382	p2	(AC)6	12	42201790	42201801
chr1_7383	p2	(GT)6	12	42216493	42216504
chr1_7384	p3	(TTG)5	15	42216607	42216621
chr1_7385	c	(AC)7gcacg(CA)11	41	42233866	42233906
chr1_7386	p2	(GT)13	26	42245717	42245742
chr1_7387	p2	(TG)14	28	42247026	42247053
chr1_7388	p4	(TTTG)6	24	42256604	42256627
chr1_7389	p2	(AT)6	12	42257022	42257033
chr1_7390	p3	(CTT)5	15	42258189	42258203
chr1_7391	p4	(TAGA)15	60	42260081	42260140
chr1_7392	p2	(GT)21	42	42266557	42266598
chr1_7393	p2	(GT)12	24	42275376	42275399
chr1_7394	p3	(GAA)5	15	42282775	42282789
chr1_7395	p2	(TG)11	22	42284929	42284950
chr1_7396	p2	(AG)6	12	42287044	42287055
chr1_7397	p2	(TG)13	26	42295842	42295867
chr1_7398	p2	(AT)8	16	42297721	42297736
chr1_7399	p2	(TG)7	14	42300362	42300375
chr1_7400	p3	(ATA)6	18	42304999	42305016
chr1_7401	p2	(CA)6	12	42315256	42315267
chr1_7402	p2	(TG)6	12	42321670	42321681
chr1_7403	c	[illegible]1catgaggatcacaaatgcgtgctgcgcaatctgcacgtataaagccatt[illegible]	83	42333819	42333901
chr1_7404	p2	(GT)10	20	42336343	42336362
chr1_7405	p2	(AC)6	12	42339376	42339387
chr1_7406	p2	(CT)7	14	42345761	42345774
chr1_7407	p2	(CA)7	14	42356846	42356859
chr1_7408	p2	(CT)21	42	42359992	42360033
chr1_7409	p3	(AAT)7	21	42360245	42360265
chr1_7410	p2	(AG)6	12	42361408	42361419
chr1_7411	p2	(CA)17	34	42368438	42368471
chr1_7412	p2	(AC)33	66	42386059	42386124
chr1_7413	p2	(GA)6	12	42388718	42388729
chr1_7414	p3	(ATT)5	15	42389569	42389583
chr1_7415	p4	(TTGA)6	24	42392310	42392333
chr1_7416	p2	(AT)7	14	42399269	42399282
chr1_7417	p2	(CT)10	20	42403233	42403252
chr1_7418	p2	(TG)8	16	42407255	42407270
chr1_7419	p4	(TTTG)5	20	42417404	42417423
chr1_7420	p3	(ACA)8	24	42417709	42417732
chr1_7421	p2	(GT)6	12	42424220	42424231
chr1_7422	p2	(AT)6	12	42426677	42426688
chr1_7423	p2	(TG)6	12	42436884	42436895
chr1_7424	p2	(TG)12	24	42441960	42441983
chr1_7425	p2	(CA)7	14	42444282	42444295
chr1_7426	p4	(TTTG)5	20	42447407	42447426
chr1_7427	p2	(TG)14	28	42450753	42450780
chr1_7428	p2	(CA)7	14	42451633	42451646
chr1_7429	p3	(TAA)7	21	42459409	42459429
chr1_7430	p2	(TA)6	12	42462953	42462964
chr1_7431	p2	(AC)10	20	42463684	42463703
chr1_7432	p2	(CA)8	16	42463936	42463951

chr1_7433	p2	(AC)6	12	42475222	42475233
chr1_7434	p2	(AG)6	12	42476649	42476660
chr1_7435	p2	(TG)6	12	42476891	42476902
chr1_7436	p4	(AGTG)5	20	42487831	42487850
chr1_7437	p2	(AC)8	16	42489991	42490006
chr1_7438	c	(GT)8gc(GT)21	60	42491185	42491244
chr1_7439	p3	(GAG)11	33	42493933	42493965
chr1_7440	p3	(ACA)6	18	42494953	42494970
chr1_7441	p2	(TG)10	20	42495341	42495360
chr1_7442	p2	(AC)17	34	42497836	42497869
chr1_7443	p2	(AC)19	38	42498091	42498128
chr1_7444	p2	(TG)9	18	42499219	42499236
chr1_7445	p2	(TG)13	26	42501254	42501279
chr1_7446	p2	(AC)7	14	42502570	42502583
chr1_7447	p2	(TG)14	28	42508637	42508664
chr1_7448	c	)10agagctgatttgcataaacatggcagtgttcaagatatcacacatg(C	114	42508987	42509100
chr1_7449	c	(GT)6a(TG)9	31	42510459	42510489
chr1_7450	c	(CA)10cgc(AG)13	49	42510664	42510712
chr1_7451	p2	(CA)24	48	42511040	42511087
chr1_7452	p4	(ATCC)5	20	42515714	42515733
chr1_7453	c	(CT)7(CA)15	44	42516747	42516790
chr1_7454	p2	(AC)14	28	42517262	42517289
chr1_7455	p2	(AG)6	12	42518285	42518296
chr1_7456	p4	(TCTG)5	20	42518816	42518835
chr1_7457	p2	(GA)6	12	42521056	42521067
chr1_7458	p2	(CA)17	34	42525833	42525866
chr1_7459	p2	(TG)6	12	42529816	42529827
chr1_7460	p2	(AC)9	18	42530954	42530971
chr1_7461	p2	(GA)7	14	42535925	42535938
chr1_7462	c	(TC)6tt(TC)6	26	42539532	42539557
chr1_7463	p2	(AC)8	16	42542296	42542311
chr1_7464	p2	(GT)19	38	42550706	42550743
chr1_7465	p2	(AC)9	18	42554210	42554227
chr1_7466	p4	(CATA)5	20	42557930	42557949
chr1_7467	p2	(TC)6	12	42564625	42564636
chr1_7468	p2	(GA)15	30	42564822	42564851
chr1_7469	p2	(AC)6	12	42565366	42565377
chr1_7470	c	(TA)13(CA)6	38	42566485	42566522
chr1_7471	p2	(CA)9	18	42569825	42569842
chr1_7472	p2	(CA)17	34	42573790	42573823
chr1_7473	p2	(AC)10	20	42576768	42576787
chr1_7474	c	acgttattacaacctgcttctaacaaacatggacatgctgtagactgactaa	93	42577683	42577775
chr1_7475	p2	(TC)12	24	42578614	42578637
chr1_7476	p2	(CA)10	20	42582209	42582228
chr1_7477	p2	(GT)7	14	42583894	42583907
chr1_7478	p2	(CA)6	12	42586863	42586874
chr1_7479	p2	(AT)13	26	42587913	42587938
chr1_7480	p2	(AT)6	12	42589879	42589890
chr1_7481	p2	(GT)6	12	42594306	42594317
chr1_7482	p3	(TGT)5	15	42596293	42596307
chr1_7483	p2	(AC)8	16	42602029	42602044
chr1_7484	p2	(CA)13	26	42603518	42603543

chr1_7485	p2	(CA)17	34	42604114	42604147
chr1_7486	p4	(TTTG)5	20	42604490	42604509
chr1_7487	p2	(TG)6	12	42605326	42605337
chr1_7488	p2	(TG)8	16	42605577	42605592
chr1_7489	c	(TC)6catgtctctcttaccaacatgtc(CA)15	65	42609226	42609290
chr1_7490	p3	(CTT)5	15	42626284	42626298
chr1_7491	p4	(AACA)5	20	42627215	42627234
chr1_7492	p3	(ATT)5	15	42651694	42651708
chr1_7493	p3	(TGT)5	15	42652087	42652101
chr1_7494	p5	(GGAGG)5	25	42659494	42659518
chr1_7495	p2	(GT)14	28	42670008	42670035
chr1_7496	p2	(CA)17	34	42671548	42671581
chr1_7497	p2	(TG)6	12	42683228	42683239
chr1_7498	p2	(TG)11	22	42690634	42690655
chr1_7499	p3	(CAG)7	21	42695475	42695495
chr1_7500	p3	(TAA)5	15	42712074	42712088
chr1_7501	p2	(AC)12	24	42727548	42727571
chr1_7502	p3	(TGG)5	15	42733404	42733418
chr1_7503	p2	(CT)12	24	42736542	42736565
chr1_7504	p2	(TG)7	14	42738777	42738790
chr1_7505	p2	(TG)6	12	42740614	42740625
chr1_7506	p2	(AC)6	12	42751621	42751632
chr1_7507	p2	(CA)13	26	42754732	42754757
chr1_7508	p3	(TGT)6	18	42757045	42757062
chr1_7509	c	(TGT)16(TGC)7	69	42759944	42760012
chr1_7510	p2	(CA)13	26	42779527	42779552
chr1_7511	p2	(TC)15	30	42780638	42780667
chr1_7512	p3	(AAT)5	15	42784834	42784848
chr1_7513	p3	(TGT)5	15	42792689	42792703
chr1_7514	p3	(TAT)10	30	42795083	42795112
chr1_7515	p2	(AC)11	22	42803383	42803404
chr1_7516	p2	(AT)6	12	42825139	42825150
chr1_7517	p3	(TAA)7	21	42825278	42825298
chr1_7518	p2	(CA)11	22	42840889	42840910
chr1_7519	p2	(AG)9	18	42889141	42889158
chr1_7520	p2	(AC)33	66	42893836	42893901
chr1_7521	p2	(GT)10	20	42900842	42900861
chr1_7522	p3	(ATA)8	24	42902481	42902504
chr1_7523	p4	(TTTA)5	20	42905979	42905998
chr1_7524	p2	(CA)6	12	42926513	42926524
chr1_7525	c	(TG)6ttcattcattctgtatttgcttccactcactcact(CA)31	109	42942746	42942854
chr1_7526	p2	(TC)9	18	42952829	42952846
chr1_7527	p3	(AAT)5	15	42953856	42953870
chr1_7528	p3	(AAC)6	18	42956177	42956194
chr1_7529	p2	(GA)6	12	42966208	42966219
chr1_7530	p4	(TCTA)5	20	42967117	42967136
chr1_7531	p2	(AC)17	34	42972484	42972517
chr1_7532	p3	(ATT)5	15	42976453	42976467
chr1_7533	p2	(CA)12	24	42981400	42981423
chr1_7534	p2	(TA)6	12	42982381	42982392
chr1_7535	p2	(GT)13	26	42986484	42986509
chr1_7536	p2	(CA)7	14	42987707	42987720

chr1_7537	p5	(CCTCT)7	35	42994471	42994505
chr1_7538	p3	(TGC)5	15	42995722	42995736
chr1_7539	p2	(CA)6	12	43000437	43000448
chr1_7540	p2	(TG)9	18	43000625	43000642
chr1_7541	p2	(TG)7	14	43005083	43005096
chr1_7542	c	(CT)6tctct(CA)9	35	43005335	43005369
chr1_7543	p2	(TG)14	28	43009833	43009860
chr1_7544	p2	(TC)14	28	43033009	43033036
chr1_7545	p2	(AG)11	22	43034359	43034380
chr1_7546	p2	(GT)8	16	43034562	43034577
chr1_7547	p2	(CA)16	32	43039450	43039481
chr1_7548	c	(CT)8ca(CT)7	32	43039829	43039860
chr1_7549	p2	(TG)7	14	43042054	43042067
chr1_7550	p3	(CAC)5	15	43044394	43044408
chr1_7551	p4	(ATAG)11	44	43049386	43049429
chr1_7552	p2	(AC)14	28	43056812	43056839
chr1_7553	p2	(CA)14	28	43058646	43058673
chr1_7554	p3	(ACA)5	15	43064492	43064506
chr1_7555	p2	(AC)7	14	43074364	43074377
chr1_7556	p2	(GA)6	12	43088608	43088619
chr1_7557	p2	(CA)13	26	43110883	43110908
chr1_7558	p6	(AAATTA)5	30	43125078	43125107
chr1_7559	p2	(TA)6	12	43160490	43160501
chr1_7560	p2	(CT)9	18	43164951	43164968
chr1_7561	c	gatattgataaaatacaataaaaaaaactaattcccataaataacaagcagg	123	43165743	43165865
chr1_7562	p3	(GAG)5	15	43202266	43202280
chr1_7563	p2	(TG)7	14	43204954	43204967
chr1_7564	p2	(GT)9	18	43212812	43212829
chr1_7565	p2	(GT)7	14	43221994	43222007
chr1_7566	p4	(ATTA)5	20	43226580	43226599
chr1_7567	p2	(AG)22	44	43231287	43231330
chr1_7568	p3	(ATT)5	15	43240116	43240130
chr1_7569	c	TAT)6aagatcaactaactc(AAT)6gataacaacaacaata(ATT)	82	43240236	43240317
chr1_7570	p2	(TC)27	54	43249381	43249434
chr1_7571	c	(GAA)8gc(AGA)5	41	43262934	43262974
chr1_7572	c	(TGTGTA)7(TG)7	56	43271707	43271762
chr1_7573	p2	(CA)8	16	43286439	43286454
chr1_7574	c	(TC)12(AC)10c(CA)15	75	43288318	43288392
chr1_7575	p2	(GA)20	40	43293811	43293850
chr1_7576	c	ttgattcctacagtttcagttctggctggctccaactgtgtgtgtgtag(GT)	168	43296619	43296786
chr1_7577	c	(AGAC)6(AGAT)11	68	43306085	43306152
chr1_7578	p2	(TG)7	14	43310335	43310348
chr1_7579	p2	(TC)7	14	43327282	43327295
chr1_7580	p2	(AC)18	36	43327453	43327488
chr1_7581	p2	(AC)18	36	43328663	43328698
chr1_7582	p2	(TG)6	12	43329829	43329840
chr1_7583	p2	(AG)7	14	43332474	43332487
chr1_7584	p2	(AT)6	12	43334733	43334744
chr1_7585	p2	(TG)31	62	43340326	43340387
chr1_7586	p2	(TG)12	24	43349633	43349656
chr1_7587	p2	(CA)17	34	43361747	43361780
chr1_7588	p2	(GT)6	12	43366680	43366691

chr1_7589	c	(AT)6aaa(AC)7	29	43368156	43368184
chr1_7590	c	(TG)6aa(AG)11	36	43374315	43374350
chr1_7591	p2	(AT)9	18	43375156	43375173
chr1_7592	p3	(TGG)5	15	43377614	43377628
chr1_7593	p2	(TG)7	14	43385294	43385307
chr1_7594	p2	(TG)8	16	43387403	43387418
chr1_7595	p2	(GT)13	26	43395422	43395447
chr1_7596	p2	(AC)6	12	43414965	43414976
chr1_7597	p4	(TTTG)6	24	43417140	43417163
chr1_7598	p2	(AG)10	20	43417509	43417528
chr1_7599	p2	(TG)6	12	43424512	43424523
chr1_7600	p2	(GT)25	50	43426114	43426163
chr1_7601	p2	(GT)6	12	43469084	43469095
chr1_7602	p2	(TG)6	12	43470288	43470299
chr1_7603	p2	(GT)6	12	43472077	43472088
chr1_7604	p2	(CA)6	12	43484909	43484920
chr1_7605	p2	(TG)15	30	43492491	43492520
chr1_7606	p2	(GA)7	14	43497806	43497819
chr1_7607	c	(GT)11ctc(TG)16	57	43504946	43505002
chr1_7608	p2	(AC)6	12	43510058	43510069
chr1_7609	p2	(AC)8	16	43510292	43510307
chr1_7610	p2	(CT)6	12	43520360	43520371
chr1_7611	p3	(AAG)5	15	43555071	43555085
chr1_7612	p2	(GA)7	14	43557940	43557953
chr1_7613	p3	(CTG)5	15	43565369	43565383
chr1_7614	p2	(AC)11	22	43582509	43582530
chr1_7615	p5	(AAATC)6	30	43589082	43589111
chr1_7616	p2	(AC)15	30	43598458	43598487
chr1_7617	p4	(TTTG)5	20	43607194	43607213
chr1_7618	p2	(TG)18	36	43609632	43609667
chr1_7619	p2	(AC)19	38	43611404	43611441
chr1_7620	p4	(TGTA)6	24	43612825	43612848
chr1_7621	p2	(AC)28	56	43613028	43613083
chr1_7622	p2	(AG)8	16	43613671	43613686
chr1_7623	p2	(AC)8	16	43616676	43616691
chr1_7624	p2	(AC)9	18	43619758	43619775
chr1_7625	p3	(AAT)7	21	43623367	43623387
chr1_7626	c	(CT)7(CA)11	36	43650718	43650753
chr1_7627	p2	(GA)6	12	43653535	43653546
chr1_7628	p2	(GT)9	18	43660266	43660283
chr1_7629	p2	(TA)7	14	43706826	43706839
chr1_7630	p3	(AAC)5	15	43726113	43726127
chr1_7631	p3	(ATA)6	18	43743989	43744006
chr1_7632	p2	(GT)6	12	43744572	43744583
chr1_7633	p2	(TG)15	30	43759225	43759254
chr1_7634	p2	(TG)7	14	43773537	43773550
chr1_7635	p2	(TG)7	14	43791918	43791931
chr1_7636	p2	(AT)9	18	43798796	43798813
chr1_7637	c	(TG)11tatatatatgtacatatatgta(TG)6(TA)7(TG)7	84	43827748	43827831
chr1_7638	p2	(GT)14	28	43856934	43856961
chr1_7639	p2	(AC)10	20	43864231	43864250
chr1_7640	p2	(AT)6	12	43868682	43868693

chr1_7641	p2	(GT)7	14	43881492	43881505
chr1_7642	p3	(TTA)8	24	43909262	43909285
chr1_7643	p2	(AC)8	16	43914939	43914954
chr1_7644	p2	(AT)8	16	43918707	43918722
chr1_7645	p2	(TC)8	16	43920866	43920881
chr1_7646	p2	(TC)9	18	43923927	43923944
chr1_7647	p2	(TG)6	12	43925917	43925928
chr1_7648	c	(TG)7tatgtgtat(GA)7	37	43932717	43932753
chr1_7649	p2	(CA)21	42	43939425	43939466
chr1_7650	p2	(TG)7	14	43955731	43955744
chr1_7651	p5	(ATCTT)8	40	43956028	43956067
chr1_7652	p2	(GT)10	20	43958069	43958088
chr1_7653	p3	(AAT)5	15	43973827	43973841
chr1_7654	p2	(TA)7	14	43978977	43978990
chr1_7655	p3	(AAG)5	15	43981315	43981329
chr1_7656	p2	(AG)6	12	43983323	43983334
chr1_7657	p2	(CA)10	20	43989534	43989553
chr1_7658	p2	(TG)6	12	44008406	44008417
chr1_7659	p2	(CA)7	14	44015433	44015446
chr1_7660	p3	(CAG)5	15	44039591	44039605
chr1_7661	c*	6ttgtatggatgctgattcagcagccatacaatgttttccaa(TTAT)5(T	93	44057877	44057969
chr1_7662	p2	(TG)6	12	44058729	44058740
chr1_7663	p3	(CAT)6	18	44060404	44060421
chr1_7664	p2	(TC)10	20	44066770	44066789
chr1_7665	p2	(AC)13	26	44080335	44080360
chr1_7666	p2	(CA)7	14	44088236	44088249
chr1_7667	p4	(ATAG)9	36	44090665	44090700
chr1_7668	p4	(GTTT)5	20	44091678	44091697
chr1_7669	p3	(TTG)7	21	44106362	44106382
chr1_7670	p2	(TG)6	12	44107936	44107947
chr1_7671	p2	(TG)8	16	44131048	44131063
chr1_7672	p2	(AC)14	28	44132044	44132071
chr1_7673	p2	(TG)6	12	44139049	44139060
chr1_7674	p2	(AC)7	14	44146913	44146926
chr1_7675	c	(CA)10ccc(CA)30	83	44157215	44157297
chr1_7676	p3	(TAA)5	15	44157894	44157908
chr1_7677	p2	(AT)7	14	44162950	44162963
chr1_7678	p2	(AT)6	12	44165939	44165950
chr1_7679	p2	(GT)9	18	44176775	44176792
chr1_7680	c	(TATG)9(TAGA)5	56	44190576	44190631
chr1_7681	p2	(TG)8	16	44200559	44200574
chr1_7682	p2	(AC)8	16	44238078	44238093
chr1_7683	p2	(AC)6	12	44245114	44245125
chr1_7684	p2	(AC)15	30	44261000	44261029
chr1_7685	p2	(CA)18	36	44262269	44262304
chr1_7686	c	(TG)16c(GT)10	53	44265947	44265999
chr1_7687	p3	(TTA)7	21	44267975	44267995
chr1_7688	p3	(CAA)5	15	44269140	44269154
chr1_7689	p3	(TTC)10	30	44270349	44270378
chr1_7690	c	ıagcatattaccatttcaaccatgtgcattttgactttgtcttactttttttttttttgg	134	44273085	44273218
chr1_7691	p3	(TTC)10	30	44275239	44275268
chr1_7692	c	ıagcatattaccatttcaaacatgtgcattttgactttgtcttactttttttttttttgg	134	44277975	44278108

chr1_7693	p3	(GGA)6	18	44294071	44294088
chr1_7694	p4	(ACAA)7	28	44338557	44338584
chr1_7695	p3	(AGA)5	15	44341118	44341132
chr1_7696	p2	(GT)16	32	44343928	44343959
chr1_7697	p2	(CA)8	16	44352489	44352504
chr1_7698	p4	(AAAT)5	20	44364982	44365001
chr1_7699	p4	(TAAA)7	28	44365165	44365192
chr1_7700	p2	(AC)27	54	44383411	44383464
chr1_7701	p3	(ATC)6	18	44385332	44385349
chr1_7702	p4	(TAGA)7	28	44388681	44388708
chr1_7703	p3	(TGC)6	18	44400453	44400470
chr1_7704	p2	(TG)9	18	44407111	44407128
chr1_7705	p2	(AC)13	26	44410662	44410687
chr1_7706	p3	(TAA)5	15	44411405	44411419
chr1_7707	p2	(AT)6	12	44412651	44412662
chr1_7708	p2	(AC)16	32	44417190	44417221
chr1_7709	p2	(CA)6	12	44446989	44447000
chr1_7710	p2	(AG)6	12	44448243	44448254
chr1_7711	p2	(AC)16	32	44452291	44452322
chr1_7712	p2	(GA)7	14	44465900	44465913
chr1_7713	p2	(AT)6	12	44505362	44505373
chr1_7714	p2	(GT)9	18	44517529	44517546
chr1_7715	p2	(AG)6	12	44522522	44522533
chr1_7716	p2	(CT)20	40	44535128	44535167
chr1_7717	p2	(CA)17	34	44556461	44556494
chr1_7718	p2	(AC)13	26	44570426	44570451
chr1_7719	p2	(TG)13	26	44600360	44600385
chr1_7720	c	(TTA)5ttgc(TGT)6	37	44615964	44616000
chr1_7721	p2	(GA)19	38	44617557	44617594
chr1_7722	p4	(AATA)6	24	44619216	44619239
chr1_7723	p2	(AT)6	12	44621446	44621457
chr1_7724	p5	(AATAT)8	40	44623708	44623747
chr1_7725	p2	(TA)9	18	44626865	44626882
chr1_7726	p5	(AAATC)5	25	44639144	44639168
chr1_7727	c	(GT)12ag(GT)6	38	44644998	44645035
chr1_7728	p3	(CTT)6	18	44648231	44648248
chr1_7729	p2	(CA)6	12	44663753	44663764
chr1_7730	p2	(CA)6	12	44665945	44665956
chr1_7731	p2	(TG)7	14	44669496	44669509
chr1_7732	p2	(AT)6	12	44674119	44674130
chr1_7733	c	(GT)6gc(GT)8	30	44680460	44680489
chr1_7734	p3	(AAT)5	15	44687168	44687182
chr1_7735	p2	(CA)43	86	44690315	44690400
chr1_7736	p2	(AC)6	12	44690772	44690783
chr1_7737	p2	(GT)6	12	44720903	44720914
chr1_7738	p2	(TC)14	28	44738461	44738488
chr1_7739	p2	(AG)6	12	44738868	44738879
chr1_7740	p2	(CA)8	16	44742399	44742414
chr1_7741	p3	(AAC)6	18	44768549	44768566
chr1_7742	p3	(TAA)8	24	44783383	44783406
chr1_7743	p4	(TGGA)6	24	44790348	44790371
chr1_7744	p2	(AT)8	16	44792768	44792783

chr1_7745	p2	(AG)6	12	44803841	44803852
chr1_7746	c	(CT)11(CA)13	48	44804042	44804089
chr1_7747	p2	(TG)16	32	44804904	44804935
chr1_7748	p3	(CCT)5	15	44809459	44809473
chr1_7749	p2	(CA)7	14	44813764	44813777
chr1_7750	p2	(GT)12	24	44819670	44819693
chr1_7751	p2	(TC)6	12	44821775	44821786
chr1_7752	p3	(TTA)5	15	44826028	44826042
chr1_7753	p2	(AC)9	18	44831001	44831018
chr1_7754	p2	(AC)6	12	44833160	44833171
chr1_7755	p2	(TC)8	16	44834177	44834192
chr1_7756	c	(TC)12ag(AC)18	62	44838701	44838762
chr1_7757	p2	(GT)7	14	44840110	44840123
chr1_7758	p4	(TATT)5	20	44843507	44843526
chr1_7759	p4	(TTTG)5	20	44844813	44844832
chr1_7760	p3	(CAG)6	18	44862213	44862230
chr1_7761	p2	(GA)15	30	44862652	44862681
chr1_7762	p2	(TG)6	12	44863409	44863420
chr1_7763	p2	(GT)6	12	44880844	44880855
chr1_7764	p2	(TG)19	38	44885533	44885570
chr1_7765	p5	(AAATC)5	25	44893825	44893849
chr1_7766	p2	(AT)6	12	44901830	44901841
chr1_7767	p2	(GT)23	46	44906806	44906851
chr1_7768	p3	(GGA)5	15	44908338	44908352
chr1_7769	p3	(GGA)5	15	44908533	44908547
chr1_7770	p2	(CA)8	16	44916299	44916314
chr1_7771	p3	(ATT)6	18	44926831	44926848
chr1_7772	p2	(AG)11	22	44938236	44938257
chr1_7773	p5	(AGATA)8	40	44941507	44941546
chr1_7774	p3	(ATA)5	15	44942922	44942936
chr1_7775	p2	(CA)7	14	44946921	44946934
chr1_7776	p2	(TG)13	26	44948341	44948366
chr1_7777	p2	(CA)27	54	44948733	44948786
chr1_7778	p2	(GA)8	16	44950513	44950528
chr1_7779	p2	(TG)6	12	44954877	44954888
chr1_7780	p2	(TC)7	14	44955093	44955106
chr1_7781	p2	(AC)6	12	44955824	44955835
chr1_7782	p2	(GT)7	14	44959710	44959723
chr1_7783	p2	(TC)16	32	44960829	44960860
chr1_7784	p2	(TC)6	12	44976481	44976492
chr1_7785	c	(TG)33(AG)7	80	44976711	44976790
chr1_7786	p2	(AC)15	30	44977971	44978000
chr1_7787	p2	(CT)6	12	44979883	44979894
chr1_7788	p2	(TC)16	32	44981452	44981483
chr1_7789	p2	(AC)25	50	44982301	44982350
chr1_7790	p2	(AC)12	24	44986396	44986419
chr1_7791	p2	(CA)6	12	44987520	44987531
chr1_7792	p2	(AC)10	20	44991850	44991869
chr1_7793	p2	(TG)14	28	44992498	44992525
chr1_7794	c	)6gcgtgcgagtacagggatgagtgtgagagttcaggcacagctgg(CA	71	44997877	44997947
chr1_7795	p2	(AC)6	12	45008635	45008646
chr1_7796	p2	(TC)11	22	45022746	45022767

chr1_7797	p2	(AC)8	16	45026461	45026476
chr1_7798	p2	(TG)14	28	45038078	45038105
chr1_7799	p4	(ATGG)5	20	45055884	45055903
chr1_7800	p2	(TG)7	14	45056906	45056919
chr1_7801	p4	(AGTA)7	28	45061700	45061727
chr1_7802	p2	(TA)8	16	45062597	45062612
chr1_7803	p3	(TAA)5	15	45063285	45063299
chr1_7804	p2	(TG)7	14	45064119	45064132
chr1_7805	p2	(GT)14	28	45070419	45070446
chr1_7806	p3	(CAG)10	30	45073479	45073508
chr1_7807	p2	(GT)19	38	45081480	45081517
chr1_7808	p2	(AC)18	36	45081734	45081769
chr1_7809	p2	(CT)10	20	45088327	45088346
chr1_7810	p2	(CA)11	22	45093065	45093086
chr1_7811	p2	(TG)7	14	45095688	45095701
chr1_7812	p2	(AC)9	18	45105907	45105924
chr1_7813	p2	(AC)6	12	45108336	45108347
chr1_7814	p2	(AC)6	12	45109151	45109162
chr1_7815	p4	(ATGG)5	20	45113352	45113371
chr1_7816	p2	(CA)14	28	45115821	45115848
chr1_7817	p2	(TG)14	28	45122310	45122337
chr1_7818	p2	(TA)8	16	45130066	45130081
chr1_7819	p2	(GA)9	18	45153716	45153733
chr1_7820	p2	(CA)9	18	45154113	45154130
chr1_7821	p2	(AG)10	20	45156812	45156831
chr1_7822	p2	(TG)27	54	45159063	45159116
chr1_7823	p3	(TAT)5	15	45160989	45161003
chr1_7824	p2	(TG)16	32	45165818	45165849
chr1_7825	p2	(TC)6	12	45175046	45175057
chr1_7826	p2	(TG)21	42	45194498	45194539
chr1_7827	p2	(TA)9	18	45199839	45199856
chr1_7828	p4	(TAGG)5	20	45203347	45203366
chr1_7829	p3	(GAA)5	15	45210294	45210308
chr1_7830	p2	(AC)9	18	45216536	45216553
chr1_7831	p2	(TG)15	30	45245683	45245712
chr1_7832	p2	(AC)6	12	45251314	45251325
chr1_7833	p2	(AC)8	16	45265881	45265896
chr1_7834	p2	(GT)10	20	45309528	45309547
chr1_7835	p2	(TG)11	22	45314826	45314847
chr1_7836	p2	(GA)20	40	45317206	45317245
chr1_7837	p3	(AGC)5	15	45319954	45319968
chr1_7838	p3	(ATA)10	30	45336303	45336332
chr1_7839	p3	(CTT)5	15	45361893	45361907
chr1_7840	p2	(TA)9	18	45362462	45362479
chr1_7841	p2	(AC)6	12	45366152	45366163
chr1_7842	p2	(GT)10	20	45381967	45381986
chr1_7843	p2	(TG)11	22	45382344	45382365
chr1_7844	p2	(TG)12	24	45387036	45387059
chr1_7845	p4	(TCCA)6	24	45387604	45387627
chr1_7846	c	gttcaggacagtgattagataactgttgttaccaactgaaattcaaagcattcc	96	45388575	45388670
chr1_7847	p2	(TG)10	20	45397575	45397594
chr1_7848	p2	(TC)9	18	45400772	45400789

chr1_7849	p2	(CA)9	18	45402622	45402639
chr1_7850	p2	(CA)6	12	45412796	45412807
chr1_7851	p3	(TGT)7	21	45413104	45413124
chr1_7852	p2	(GT)20	40	45425225	45425264
chr1_7853	p2	(AC)6	12	45426737	45426748
chr1_7854	p2	(TG)8	16	45429219	45429234
chr1_7855	p5	(GATTT)8	40	45431892	45431931
chr1_7856	p2	(TG)6	12	45449604	45449615
chr1_7857	p4	(AAAG)5	20	45465602	45465621
chr1_7858	p4	(CATC)5	20	45467382	45467401
chr1_7859	p2	(GT)14	28	45476698	45476725
chr1_7860	p2	(GT)15	30	45492778	45492807
chr1_7861	p2	(GT)14	28	45494672	45494699
chr1_7862	c	aaataaactgaattttatactgtgtatctatgtcttcatgtactgtatgctttgttg	96	45500467	45500562
chr1_7863	p2	(AG)7	14	45505052	45505065
chr1_7864	p4	(GTTT)5	20	45519163	45519182
chr1_7865	p3	(GGA)6	18	45531597	45531614
chr1_7866	p2	(TG)11	22	45534131	45534152
chr1_7867	p2	(GT)21	42	45546371	45546412
chr1_7868	p2	(AC)8	16	45562420	45562435
chr1_7869	p2	(TG)6	12	45567558	45567569
chr1_7870	p3	(TAG)10	30	45571338	45571367
chr1_7871	p2	(GT)6	12	45571777	45571788
chr1_7872	p2	(TG)17	34	45571935	45571968
chr1_7873	p2	(AC)6	12	45572837	45572848
chr1_7874	p2	(CA)6	12	45573244	45573255
chr1_7875	p2	(TG)23	46	45577000	45577045
chr1_7876	p2	(TG)8	16	45584783	45584798
chr1_7877	p2	(TC)7	14	45587698	45587711
chr1_7878	c	(AC)8agatccacgtcagcatatgcgtg(CA)6	51	45588183	45588233
chr1_7879	c	gaaaatagatgttgggggggttagtttagagaaatggggaactgagagag	127	45594181	45594307
chr1_7880	p2	(AG)7	14	45598436	45598449
chr1_7881	p2	(GT)6	12	45601466	45601477
chr1_7882	p2	(TG)16	32	45604151	45604182
chr1_7883	p2	(TG)15	30	45605649	45605678
chr1_7884	p3	(TTA)7	21	45609391	45609411
chr1_7885	c	(TC)6(AC)14	40	45613471	45613510
chr1_7886	p2	(TC)6	12	45618182	45618193
chr1_7887	p2	(TG)25	50	45621099	45621148
chr1_7888	p2	(TG)15	30	45621501	45621530
chr1_7889	p2	(AG)6	12	45624079	45624090
chr1_7890	p2	(TG)9	18	45631561	45631578
chr1_7891	p2	(GT)9	18	45632905	45632922
chr1_7892	p3	(CTC)5	15	45643981	45643995
chr1_7893	p2	(TG)6	12	45652027	45652038
chr1_7894	p3	(ATC)5	15	45673217	45673231
chr1_7895	p2	(TA)10	20	45730615	45730634
chr1_7896	p2	(AC)12	24	45739436	45739459
chr1_7897	c	attcagctgtggtgtgctaatgtccctaatggacagttgccagagctgggga	124	45741796	45741919
chr1_7898	p2	(AG)9	18	45761981	45761998
chr1_7899	p2	(GA)6	12	45765441	45765452
chr1_7900	p2	(AC)23	46	45765780	45765825

chr1_7901	p2	(CT)15	30	45772436	45772465
chr1_7902	p2	(AC)9	18	45772793	45772810
chr1_7903	p2	(GT)19	38	45774819	45774856
chr1_7904	p2	(CA)7	14	45777738	45777751
chr1_7905	p2	(GT)10	20	45780280	45780299
chr1_7906	p2	(TA)8	16	45781636	45781651
chr1_7907	p2	(TG)7	14	45784424	45784437
chr1_7908	p2	(AC)10	20	45787058	45787077
chr1_7909	p3	(GAA)6	18	45788421	45788438
chr1_7910	p2	(AC)29	58	45795212	45795269
chr1_7911	p2	(CA)6	12	45799700	45799711
chr1_7912	p2	(TG)12	24	45804257	45804280
chr1_7913	p4	(AAAT)5	20	45806274	45806293
chr1_7914	p3	(CCT)5	15	45808180	45808194
chr1_7915	p3	(TTG)5	15	45828935	45828949
chr1_7916	p2	(GT)9	18	45830117	45830134
chr1_7917	p2	(AT)6	12	45831758	45831769
chr1_7918	p2	(TC)8	16	45833611	45833626
chr1_7919	c	tggatttgcattctgagtagcagggatgctgtcgctgtgtctttcagctactgt	115	45834000	45834114
chr1_7920	p2	(CA)6	12	45834532	45834543
chr1_7921	p2	(TG)14	28	45839557	45839584
chr1_7922	p2	(AG)6	12	45839988	45839999
chr1_7923	p2	(AC)7	14	45841399	45841412
chr1_7924	c	(CA)13cc(CCT)5	43	45847042	45847084
chr1_7925	p2	(AC)22	44	45850338	45850381
chr1_7926	p2	(GT)9	18	45850755	45850772
chr1_7927	c	(TG)9(AG)7aaagagagaga(GT)12	67	45852400	45852466
chr1_7928	p3	(AAT)20	60	45853787	45853846
chr1_7929	c	(GT)14gggtgg(GT)13	60	45854084	45854143
chr1_7930	p3	(AAT)10	30	45856625	45856654
chr1_7931	c	(TG)7tttgtgtgtgtgta(TG)7	42	45858520	45858561
chr1_7932	c	(AG)6aaggaggaggt(GGA)5	38	45858767	45858804
chr1_7933	p2	(GT)22	44	45859711	45859754
chr1_7934	p2	(CA)33	66	45861118	45861183
chr1_7935	p2	(TG)13	26	45862095	45862120
chr1_7936	p2	(CA)14	28	45862704	45862731
chr1_7937	p2	(GT)8	16	45863485	45863500
chr1_7938	p2	(GT)6	12	45864551	45864562
chr1_7939	p2	(GT)20	40	45871688	45871727
chr1_7940	p2	(AG)6	12	45878565	45878576
chr1_7941	p2	(CA)19	38	45882976	45883013
chr1_7942	p3	(GGA)5	15	45883880	45883894
chr1_7943	p3	(GAG)5	15	45886822	45886836
chr1_7944	p3	(CAC)7	21	45898261	45898281
chr1_7945	p2	(GT)7	14	45898553	45898566
chr1_7946	p3	(CTG)5	15	45901106	45901120
chr1_7947	p2	(AT)9	18	45901854	45901871
chr1_7948	p2	(GT)6	12	45902757	45902768
chr1_7949	p2	(GT)6	12	45905139	45905150
chr1_7950	p2	(TG)9	18	45905259	45905276
chr1_7951	p2	(TG)17	34	45907553	45907586
chr1_7952	c	tgtttactgcccttcttaacagaaatttaattcctctcctgaatatatttggctct	130	45912278	45912407

chr1_7953	p2	(CA)6	12	45918686	45918697
chr1_7954	p2	(GT)13	26	45921301	45921326
chr1_7955	p3	(CTG)6	18	45946598	45946615
chr1_7956	p2	(TG)6	12	45959626	45959637
chr1_7957	p2	(GT)15	30	45965891	45965920
chr1_7958	p2	(CA)9	18	45969450	45969467
chr1_7959	p2	(AC)11	22	45972017	45972038
chr1_7960	p2	(AT)9	18	45990086	45990103
chr1_7961	p2	(TG)25	50	45995969	45996018
chr1_7962	p2	(TG)7	14	46000044	46000057
chr1_7963	p2	(TG)6	12	46009872	46009883
chr1_7964	p3	(CTG)5	15	46015935	46015949
chr1_7965	p2	(TC)26	52	46034767	46034818
chr1_7966	p2	(CA)8	16	46037389	46037404
chr1_7967	p2	(CA)10	20	46037646	46037665
chr1_7968	p3	(AAT)6	18	46039250	46039267
chr1_7969	c	(AC)21(TC)7	56	46045530	46045585
chr1_7970	p2	(CA)16	32	46050212	46050243
chr1_7971	p2	(AC)19	38	46051931	46051968
chr1_7972	p2	(CA)7	14	46062104	46062117
chr1_7973	p2	(CA)6	12	46088889	46088900
chr1_7974	p2	(GA)7	14	46089312	46089325
chr1_7975	p2	(CA)14	28	46089437	46089464
chr1_7976	p3	(GAG)5	15	46089750	46089764
chr1_7977	p2	(TG)6	12	46095271	46095282
chr1_7978	p3	(CAC)5	15	46119587	46119601
chr1_7979	p2	(CA)6	12	46125322	46125333
chr1_7980	p5	(AGTGA)5	25	46125795	46125819
chr1_7981	p2	(TG)17	34	46127472	46127505
chr1_7982	p2	(TG)15	30	46135356	46135385
chr1_7983	p4	(TATT)26	104	46146054	46146157
chr1_7984	p2	(TG)7	14	46156044	46156057
chr1_7985	p2	(TA)7	14	46161722	46161735
chr1_7986	p2	(GT)6	12	46164917	46164928
chr1_7987	c	tctgtacagaggaatgtttatctttctcctgtccatgcaaacactgcatgttcat	102	46166541	46166642
chr1_7988	p2	(CA)22	44	46168273	46168316
chr1_7989	p3	(TTA)8	24	46177036	46177059
chr1_7990	p4	(TAGC)5	20	46198966	46198985
chr1_7991	p2	(TA)8	16	46201303	46201318
chr1_7992	p2	(TG)10	20	46210974	46210993
chr1_7993	p2	(CT)6	12	46215148	46215159
chr1_7994	p2	(GA)7	14	46245158	46245171
chr1_7995	p2	(AC)6	12	46263955	46263966
chr1_7996	p2	(TG)15	30	46267527	46267556
chr1_7997	p3	(TCC)5	15	46267933	46267947
chr1_7998	p2	(GT)7	14	46283688	46283701
chr1_7999	p2	(CT)10	20	46286343	46286362
chr1_8000	p2	(AC)6	12	46288490	46288501
chr1_8001	p2	(GT)6	12	46291249	46291260
chr1_8002	p2	(CT)11	22	46302031	46302052
chr1_8003	p2	(GA)7	14	46304506	46304519
chr1_8004	p3	(AAT)5	15	46307118	46307132

chr1_8005	p2	(AG)16	32	46310286	46310317
chr1_8006	p2	(GT)6	12	46321565	46321576
chr1_8007	p3	(TGT)6	18	46324058	46324075
chr1_8008	p3	(GCT)5	15	46327276	46327290
chr1_8009	p2	(CA)6	12	46331666	46331677
chr1_8010	p2	(AC)7	14	46332935	46332948
chr1_8011	p3	(AAC)5	15	46335811	46335825
chr1_8012	c	(AG)6ac(TG)7	28	46349712	46349739
chr1_8013	p2	(AC)6	12	46352580	46352591
chr1_8014	p4	(CATA)5	20	46357223	46357242
chr1_8015	p3	(AAG)5	15	46358923	46358937
chr1_8016	p2	(AG)7	14	46367009	46367022
chr1_8017	p3	(ATA)5	15	46388288	46388302
chr1_8018	p5	(TAAAA)6	30	46390106	46390135
chr1_8019	p2	(CT)7	14	46396765	46396778
chr1_8020	p2	(TG)11	22	46399437	46399458
chr1_8021	p2	(AG)7	14	46404945	46404958
chr1_8022	p2	(TC)7	14	46414194	46414207
chr1_8023	p3	(TAA)5	15	46418149	46418163
chr1_8024	p2	(TG)41	82	46424922	46425003
chr1_8025	c	(CA)7accccaccaaatagttcagaccccccccccccccc(AC)13	74	46427162	46427235
chr1_8026	p2	(CA)7	14	46444333	46444346
chr1_8027	p2	(TC)7	14	46445360	46445373
chr1_8028	p2	(CA)16	32	46452303	46452334
chr1_8029	p2	(GT)7	14	46452778	46452791
chr1_8030	p2	(AC)33	66	46456458	46456523
chr1_8031	p3	(GTT)5	15	46460283	46460297
chr1_8032	p2	(GA)8	16	46465549	46465564
chr1_8033	p2	(AC)12	24	46466167	46466190
chr1_8034	p2	(CA)6	12	46466542	46466553
chr1_8035	p2	(CA)21	42	46466766	46466807
chr1_8036	p2	(CA)6	12	46466959	46466970
chr1_8037	p2	(CA)19	38	46467183	46467220
chr1_8038	p2	(TG)17	34	46473796	46473829
chr1_8039	p3	(ACA)5	15	46474336	46474350
chr1_8040	p2	(CA)8	16	46477388	46477403
chr1_8041	p2	(CT)7	14	46484652	46484665
chr1_8042	p3	(TCA)5	15	46485834	46485848
chr1_8043	p2	(CA)17	34	46490257	46490290
chr1_8044	c	(TC)6tttctctcgcccaccccct(TC)9	49	46490525	46490573
chr1_8045	p2	(CA)6	12	46491639	46491650
chr1_8046	p2	(GA)11	22	46506765	46506786
chr1_8047	p4	(ATTT)5	20	46515785	46515804
chr1_8048	p2	(AG)8	16	46521303	46521318
chr1_8049	p2	(AG)6	12	46521624	46521635
chr1_8050	p2	(GT)9	18	46521844	46521861
chr1_8051	p2	(CA)26	52	46522425	46522476
chr1_8052	p2	(AC)7	14	46522646	46522659
chr1_8053	p2	(AG)10	20	46526208	46526227
chr1_8054	p2	(AC)6	12	46526329	46526340
chr1_8055	p2	(AC)12	24	46532671	46532694
chr1_8056	p2	(GT)11	22	46532903	46532924

chr1_8057	p2	(TA)7	14	46534327	46534340
chr1_8058	p2	(CA)6	12	46536004	46536015
chr1_8059	p2	(TC)6	12	46548698	46548709
chr1_8060	p2	(CA)7	14	46550563	46550576
chr1_8061	p2	(GT)8	16	46551819	46551834
chr1_8062	p2	(TG)18	36	46558911	46558946
chr1_8063	p2	(CT)13	26	46560106	46560131
chr1_8064	p2	(AG)6	12	46560709	46560720
chr1_8065	p2	(TG)26	52	46568633	46568684
chr1_8066	p2	(CA)7	14	46574604	46574617
chr1_8067	p3	(GAT)5	15	46575631	46575645
chr1_8068	p2	(CT)10	20	46580122	46580141
chr1_8069	p2	(CA)12	24	46583522	46583545
chr1_8070	p2	(CA)6	12	46586524	46586535
chr1_8071	c	gcatttcaccatttgtcagaaa(TC)6tttttgtttcacctcccttagaatagt	183	46586951	46587133
chr1_8072	p2	(GT)12	24	46587565	46587588
chr1_8073	p2	(AC)6	12	46597291	46597302
chr1_8074	p2	(AC)6	12	46605633	46605644
chr1_8075	p2	(CT)6	12	46607744	46607755
chr1_8076	p2	(AC)7	14	46621409	46621422
chr1_8077	p2	(CA)9	18	46622363	46622380
chr1_8078	p2	(TA)9	18	46623295	46623312
chr1_8079	p2	(CT)17	34	46624844	46624877
chr1_8080	p2	(AG)6	12	46625389	46625400
chr1_8081	p3	(CCT)5	15	46625574	46625588
chr1_8082	c	(AG)6c(GA)6	25	46625747	46625771
chr1_8083	p3	(AAT)6	18	46650450	46650467
chr1_8084	p2	(CA)10	20	46660000	46660019
chr1_8085	p2	(TC)13	26	46665660	46665685
chr1_8086	p2	(TA)6	12	46667577	46667588
chr1_8087	p2	(TG)12	24	46667945	46667968
chr1_8088	p2	(TG)7	14	46674217	46674230
chr1_8089	p2	(AC)7	14	46684329	46684342
chr1_8090	p2	(AT)7	14	46685107	46685120
chr1_8091	p2	(AC)6	12	46696052	46696063
chr1_8092	p2	(AT)6	12	46704676	46704687
chr1_8093	p4	(GATG)5	20	46719123	46719142
chr1_8094	p4	(AATG)6	24	46721137	46721160
chr1_8095	c	(TC)15c(CT)7	45	46735368	46735412
chr1_8096	p2	(TG)6	12	46738380	46738391
chr1_8097	p2	(AT)9	18	46739222	46739239
chr1_8098	p3	(AGT)5	15	46759378	46759392
chr1_8099	p3	(TTG)5	15	46764400	46764414
chr1_8100	p2	(CA)7	14	46768206	46768219
chr1_8101	p2	(AT)7	14	46772532	46772545
chr1_8102	p4	(CATC)5	20	46779735	46779754
chr1_8103	p2	(AC)6	12	46792870	46792881
chr1_8104	p2	(AC)9	18	46816512	46816529
chr1_8105	p3	(TTC)6	18	46825567	46825584
chr1_8106	p2	(TC)7	14	46834433	46834446
chr1_8107	p2	(TA)6	12	46839084	46839095
chr1_8108	p3	(CAT)6	18	46841047	46841064

chr1_8109	p4	(TCCA)6	24	46848256	46848279
chr1_8110	p2	(GT)7	14	46856214	46856227
chr1_8111	p2	(TA)6	12	46861054	46861065
chr1_8112	c	agagggagagannnnnnnnnnnnnnnnnnnnnnnnnnnnnnnnr	182	46867280	46867461
chr1_8113	p2	(GT)7	14	46876094	46876107
chr1_8114	p2	(GA)11	22	46886103	46886124
chr1_8115	p2	(TG)7	14	46891502	46891515
chr1_8116	p2	(GT)18	36	46898207	46898242
chr1_8117	p3	(ATG)11	33	46901031	46901063
chr1_8118	c	(AG)10c(GA)6	33	46903341	46903373
chr1_8119	p3	(TTC)5	15	46907941	46907955
chr1_8120	p2	(AC)20	40	46919645	46919684
chr1_8121	p2	(TG)6	12	46932504	46932515
chr1_8122	p2	(CA)6	12	46944847	46944858
chr1_8123	c	(ATAA)5atataaatactgattgtctaaaaacatcctaat(AC)14	81	46946369	46946449
chr1_8124	c	(TC)14(AC)11ag(GT)7(TG)12	90	46954385	46954474
chr1_8125	p2	(CA)7	14	46958828	46958841
chr1_8126	p2	(GT)6	12	46964929	46964940
chr1_8127	p2	(AC)14	28	46970765	46970792
chr1_8128	p2	(AG)7	14	46987263	46987276
chr1_8129	p2	(TG)22	44	46991642	46991685
chr1_8130	p2	(CA)32	64	46991832	46991895
chr1_8131	p2	(AC)15	30	46992607	46992636
chr1_8132	p3	(TGT)6	18	46994307	46994324
chr1_8133	p2	(TG)6	12	47015464	47015475
chr1_8134	p3	(ATA)8	24	47023115	47023138
chr1_8135	p2	(CA)6	12	47030666	47030677
chr1_8136	c	tgcgaaaatatacaccggaagatgtgcatttgatcgtgtctaacttgactatg	133	47034270	47034402
chr1_8137	p3	(TAA)13	39	47036251	47036289
chr1_8138	c	)8ccagcgccgtgcacagctgcaacgggagagaagaaaagcaaaacc(	73	47049889	47049961
chr1_8139	p2	(TG)9	18	47071823	47071840
chr1_8140	p3	(GAT)8	24	47074350	47074373
chr1_8141	p2	(TA)9	18	47074851	47074868
chr1_8142	p3	(GAT)8	24	47076631	47076654
chr1_8143	p2	(GA)12	24	47078383	47078406
chr1_8144	p2	(AC)6	12	47084531	47084542
chr1_8145	p4	(ATGG)5	20	47110310	47110329
chr1_8146	p2	(GT)8	16	47111267	47111282
chr1_8147	p5	(GACAG)5	25	47112712	47112736
chr1_8148	p2	(TG)6	12	47121491	47121502
chr1_8149	p2	(TG)19	38	47130317	47130354
chr1_8150	p3	(CTC)6	18	47131496	47131513
chr1_8151	p3	(TAT)5	15	47132809	47132823
chr1_8152	p2	(TG)8	16	47133382	47133397
chr1_8153	p2	(GT)6	12	47144428	47144439
chr1_8154	c	(AG)7tgtgtgtgtgtatatatatg(TA)7	48	47149959	47150006
chr1_8155	p2	(TG)8	16	47150234	47150249
chr1_8156	p2	(TC)7	14	47170019	47170032
chr1_8157	p2	(GT)9	18	47174168	47174185
chr1_8158	p2	(AC)16	32	47179461	47179492
chr1_8159	c	ıtaaatgaatctggttgacattgtctggaacaggtggtgaagtcagcaggga	134	47180636	47180769
chr1_8160	p2	(TC)6	12	47182083	47182094

chr1_8161	p2	(TC)6	12	47182530	47182541
chr1_8162	p2	(AG)6	12	47191206	47191217
chr1_8163	p2	(AC)9	18	47194267	47194284
chr1_8164	p2	(GT)6	12	47200329	47200340
chr1_8165	p2	(GT)9	18	47203250	47203267
chr1_8166	p2	(TG)6	12	47203486	47203497
chr1_8167	p2	(GT)15	30	47208432	47208461
chr1_8168	p2	(GT)12	24	47209706	47209729
chr1_8169	p2	(AC)31	62	47209869	47209930
chr1_8170	p2	(CA)6	12	47220978	47220989
chr1_8171	p2	(GT)14	28	47234455	47234482
chr1_8172	p2	(AC)6	12	47240297	47240308
chr1_8173	p2	(TG)11	22	47240688	47240709
chr1_8174	p2	(AC)6	12	47243848	47243859
chr1_8175	p2	(TG)24	48	47248966	47249013
chr1_8176	p2	(AC)7	14	47250961	47250974
chr1_8177	p2	(GT)10	20	47251948	47251967
chr1_8178	p2	(TA)9	18	47252361	47252378
chr1_8179	p2	(TG)7	14	47252836	47252849
chr1_8180	c	(GT)6atatg(TA)7	31	47253056	47253086
chr1_8181	p2	(GT)6	12	47254524	47254535
chr1_8182	c	(GT)6atatg(TA)7	31	47261965	47261995
chr1_8183	p2	(GT)6	12	47263433	47263444
chr1_8184	p2	(TA)6	12	47263943	47263954
chr1_8185	p2	(CA)19	38	47265558	47265595
chr1_8186	p2	(TG)9	18	47269250	47269267
chr1_8187	c	(TA)9(TG)14	46	47275964	47276009
chr1_8188	p2	(CT)8	16	47282815	47282830
chr1_8189	p2	(TG)12	24	47301412	47301435
chr1_8190	p3	(TTA)5	15	47311629	47311643
chr1_8191	p2	(AC)16	32	47312264	47312295
chr1_8192	p2	(CA)10	20	47318514	47318533
chr1_8193	p3	(ATC)8	24	47319946	47319969
chr1_8194	p2	(AC)6	12	47327294	47327305
chr1_8195	p2	(TG)9	18	47371611	47371628
chr1_8196	p3	(CTG)6	18	47374734	47374751
chr1_8197	p2	(TG)7	14	47393076	47393089
chr1_8198	p2	(CA)7	14	47402040	47402053
chr1_8199	p3	(TTC)6	18	47409417	47409434
chr1_8200	p3	(ATA)5	15	47423917	47423931
chr1_8201	p2	(GA)7	14	47449956	47449969
chr1_8202	p2	(GT)10	20	47463378	47463397
chr1_8203	p3	(CTC)5	15	47473627	47473641
chr1_8204	c	(TG)12(TA)6	36	47477604	47477639
chr1_8205	p2	(AC)17	34	47481654	47481687
chr1_8206	p2	(TG)13	26	47487794	47487819
chr1_8207	p2	(TG)11	22	47489537	47489558
chr1_8208	p2	(TG)7	14	47494478	47494491
chr1_8209	p2	(CA)9	18	47497985	47498002
chr1_8210	p2	(CA)15	30	47499430	47499459
chr1_8211	c	(TC)15acact(CA)18	71	47499626	47499696
chr1_8212	p3	(GGA)5	15	47501701	47501715

chr1_8213	p2	(TG)8	16	47501857	47501872
chr1_8214	p2	(GT)6	12	47509314	47509325
chr1_8215	p2	(AG)9	18	47545150	47545167
chr1_8216	p2	(TG)12	24	47548783	47548806
chr1_8217	p2	(CA)15	30	47555233	47555262
chr1_8218	p2	(AC)9	18	47555729	47555746
chr1_8219	p2	(CA)13	26	47555947	47555972
chr1_8220	p2	(TG)13	26	47563108	47563133
chr1_8221	p2	(AC)6	12	47564115	47564126
chr1_8222	p2	(CA)6	12	47569889	47569900
chr1_8223	p2	(AT)6	12	47574214	47574225
chr1_8224	p2	(TG)10	20	47576583	47576602
chr1_8225	p2	(TG)19	38	47579499	47579536
chr1_8226	p2	(TC)6	12	47581603	47581614
chr1_8227	p2	(TG)6	12	47590590	47590601
chr1_8228	p4	(TTCT)5	20	47609776	47609795
chr1_8229	p4	(ATGG)6	24	47613977	47614000
chr1_8230	p3	(TTA)7	21	47622401	47622421
chr1_8231	p2	(TG)21	42	47630652	47630693
chr1_8232	p2	(TA)7	14	47633752	47633765
chr1_8233	p2	(TG)10	20	47635794	47635813
chr1_8234	p3	(TTC)10	30	47638364	47638393
chr1_8235	p3	(TGA)5	15	47638631	47638645
chr1_8236	p2	(TG)6	12	47656302	47656313
chr1_8237	p2	(AT)10	20	47661027	47661046
chr1_8238	p2	(CA)10	20	47675107	47675126
chr1_8239	p2	(TG)20	40	47678598	47678637
chr1_8240	p2	(AC)16	32	47680553	47680584
chr1_8241	p2	(TG)6	12	47683855	47683866
chr1_8242	p3	(ATA)5	15	47688454	47688468
chr1_8243	p2	(TG)9	18	47698290	47698307
chr1_8244	p2	(CT)6	12	47700171	47700182
chr1_8245	p2	(GT)18	36	47707308	47707343
chr1_8246	p2	(AC)15	30	47709344	47709373
chr1_8247	p2	(TG)7	14	47710318	47710331
chr1_8248	p4	(ATTT)5	20	47712874	47712893
chr1_8249	p2	(TG)8	16	47722703	47722718
chr1_8250	c	(TA)8(TG)10	36	47725554	47725589
chr1_8251	p2	(CA)14	28	47738083	47738110
chr1_8252	p2	(AC)7	14	47742506	47742519
chr1_8253	p2	(AC)9	18	47756868	47756885
chr1_8254	p2	(AC)7	14	47757191	47757204
chr1_8255	p3	(TTG)5	15	47758459	47758473
chr1_8256	p2	(TG)6	12	47760007	47760018
chr1_8257	p2	(CT)6	12	47764979	47764990
chr1_8258	p3	(CAA)7	21	47765396	47765416
chr1_8259	p2	(TG)7	14	47771033	47771046
chr1_8260	p2	(TA)8	16	47788196	47788211
chr1_8261	p2	(TG)6	12	47807171	47807182
chr1_8262	p2	(TG)7	14	47809501	47809514
chr1_8263	p2	(CA)6	12	47818759	47818770
chr1_8264	p2	(TG)24	48	47824111	47824158

chr1_8265	p2	(TA)8	16	47832096	47832111
chr1_8266	p5	(TAGTA)5	25	47832404	47832428
chr1_8267	p2	(TC)6	12	47844313	47844324
chr1_8268	p3	(GAG)5	15	47849573	47849587
chr1_8269	p2	(AC)6	12	47873809	47873820
chr1_8270	c	(CA)8tccactctactatgacgcg(CA)6	47	47888965	47889011
chr1_8271	p2	(AC)12	24	47892248	47892271
chr1_8272	p2	(CA)11	22	47894401	47894422
chr1_8273	p2	(CA)11	22	47896869	47896890
chr1_8274	p2	(AT)7	14	47906736	47906749
chr1_8275	p2	(TG)17	34	47918221	47918254
chr1_8276	p2	(AC)6	12	47920802	47920813
chr1_8277	p2	(CA)10	20	47928088	47928107
chr1_8278	p3	(AAT)5	15	47929008	47929022
chr1_8279	p2	(TC)14	28	47946808	47946835
chr1_8280	p2	(GA)7	14	47952299	47952312
chr1_8281	p2	(TG)6	12	47954770	47954781
chr1_8282	p2	(GT)6	12	47965694	47965705
chr1_8283	p2	(AT)6	12	47970000	47970011
chr1_8284	c	(TC)18(AC)18	72	47975500	47975571
chr1_8285	p2	(TG)10	20	47978641	47978660
chr1_8286	p2	(GT)6	12	47987170	47987181
chr1_8287	p3	(TTA)6	18	48006938	48006955
chr1_8288	p3	(TTA)6	18	48010232	48010249
chr1_8289	p2	(GT)14	28	48024860	48024887
chr1_8290	p2	(AC)22	44	48025422	48025465
chr1_8291	p3	(TAA)5	15	48026948	48026962
chr1_8292	p3	(CCT)7	21	48029204	48029224
chr1_8293	p2	(CA)16	32	48041812	48041843
chr1_8294	p4	(TTAT)5	20	48067499	48067518
chr1_8295	p4	(ATGG)7	28	48094473	48094500
chr1_8296	p2	(TG)7	14	48115973	48115986
chr1_8297	p2	(GT)7	14	48116235	48116248
chr1_8298	p2	(CA)19	38	48119062	48119099
chr1_8299	p3	(CCT)5	15	48120885	48120899
chr1_8300	p3	(AGC)5	15	48124937	48124951
chr1_8301	p2	(CA)10	20	48133182	48133201
chr1_8302	p4	(AAAG)5	20	48139926	48139945
chr1_8303	p2	(AC)12	24	48151303	48151326
chr1_8304	p2	(AC)6	12	48154961	48154972
chr1_8305	p2	(AC)13	26	48157072	48157097
chr1_8306	p3	(AAC)7	21	48159375	48159395
chr1_8307	p2	(CA)8	16	48160486	48160501
chr1_8308	p2	(AC)6	12	48162890	48162901
chr1_8309	p2	(AG)6	12	48164770	48164781
chr1_8310	p2	(TG)6	12	48173266	48173277
chr1_8311	c	(CCT)5(CTT)6	33	48175855	48175887
chr1_8312	p2	(AC)6	12	48185704	48185715
chr1_8313	p2	(TG)9	18	48188073	48188090
chr1_8314	p2	(AC)8	16	48188916	48188931
chr1_8315	c	(ATA)8(AAT)6	42	48204616	48204657
chr1_8316	p3	(ATA)11	33	48205907	48205939

chr1_8317	p2	(GT)6	12	48222926	48222937
chr1_8318	p2	(TA)6	12	48241715	48241726
chr1_8319	p2	(CA)29	58	48261699	48261756
chr1_8320	p2	(CA)13	26	48261958	48261983
chr1_8321	p3	(CTC)6	18	48263751	48263768
chr1_8322	c	actagtctgggtatctgtctgttaatctgataagtgtatacatgcacatgtgtg	154	48267593	48267746
chr1_8323	p2	(CA)17	34	48269792	48269825
chr1_8324	p2	(AC)16	32	48281099	48281130
chr1_8325	p2	(GT)6	12	48294105	48294116
chr1_8326	p2	(CA)9	18	48298151	48298168
chr1_8327	p4	(TCTA)10	40	48307763	48307802
chr1_8328	p2	(CA)6	12	48311678	48311689
chr1_8329	p2	(TG)9	18	48321070	48321087
chr1_8330	p2	(TG)9	18	48322395	48322412
chr1_8331	p3	(TTA)7	21	48341036	48341056
chr1_8332	p3	(GAT)18	54	48341912	48341965
chr1_8333	p2	(AT)7	14	48350012	48350025
chr1_8334	p2	(TG)12	24	48361466	48361489
chr1_8335	p3	(CAG)5	15	48361724	48361738
chr1_8336	p4	(TAAT)5	20	48382966	48382985
chr1_8337	p2	(AC)17	34	48384616	48384649
chr1_8338	p2	(GT)26	52	48385764	48385815
chr1_8339	p2	(GA)8	16	48398661	48398676
chr1_8340	p2	(AC)16	32	48402467	48402498
chr1_8341	p2	(TG)13	26	48409405	48409430
chr1_8342	p4	(TTTG)5	20	48419539	48419558
chr1_8343	p2	(GT)13	26	48424252	48424277
chr1_8344	p5	(ATTTT)5	25	48442844	48442868
chr1_8345	p2	(AC)9	18	48487114	48487131
chr1_8346	p2	(GT)6	12	48488362	48488373
chr1_8347	p2	(GT)11	22	48496736	48496757
chr1_8348	p3	(TGT)5	15	48504537	48504551
chr1_8349	p4	(CAAA)5	20	48506653	48506672
chr1_8350	p2	(CA)8	16	48512301	48512316
chr1_8351	p2	(TG)8	16	48524153	48524168
chr1_8352	p3	(CTC)5	15	48528055	48528069
chr1_8353	p3	(ACA)7	21	48536394	48536414
chr1_8354	p2	(CT)7	14	48552003	48552016
chr1_8355	p2	(AT)8	16	48553430	48553445
chr1_8356	p5	(CATCC)6	30	48562912	48562941
chr1_8357	p2	(CA)6	12	48568895	48568906
chr1_8358	p3	(TAA)7	21	48576440	48576460
chr1_8359	p4	(ATTC)6	24	48579489	48579512
chr1_8360	p3	(GAA)5	15	48613061	48613075
chr1_8361	p2	(GA)6	12	48613567	48613578
chr1_8362	p2	(AC)7	14	48619683	48619696
chr1_8363	c	G)12catgcccagcattactcacactgtggggaccaaaacctgttt(AC	80	48619818	48619897
chr1_8364	p2	(AC)7	14	48622317	48622330
chr1_8365	p4	(TATG)5	20	48629054	48629073
chr1_8366	p2	(CA)11	22	48633250	48633271
chr1_8367	p2	(AC)11	22	48640076	48640097
chr1_8368	p3	(AAT)7	21	48640226	48640246

chr1_8369	p2	(GT)13	26	48648335	48648360
chr1_8370	p2	(TG)9	18	48656387	48656404
chr1_8371	p2	(TG)7	14	48670759	48670772
chr1_8372	p2	(AG)6	12	48679700	48679711
chr1_8373	p3	(ATT)6	18	48692340	48692357
chr1_8374	p4	(AAAC)5	20	48695957	48695976
chr1_8375	p2	(AT)8	16	48703120	48703135
chr1_8376	p3	(TTC)7	21	48721053	48721073
chr1_8377	p3	(GCA)6	18	48731949	48731966
chr1_8378	p2	(TC)6	12	48734421	48734432
chr1_8379	p5	(AAATC)6	30	48746647	48746676
chr1_8380	p2	(CA)7	14	48747843	48747856
chr1_8381	p2	(TA)6	12	48749770	48749781
chr1_8382	p3	(GGA)5	15	48752347	48752361
chr1_8383	p3	(ACA)5	15	48752659	48752673
chr1_8384	p2	(AG)16	32	48758860	48758891
chr1_8385	p4	(TTTA)5	20	48760462	48760481
chr1_8386	p2	(TC)7	14	48763620	48763633
chr1_8387	p2	(TG)7	14	48779261	48779274
chr1_8388	p2	(GT)6	12	48795059	48795070
chr1_8389	p2	(CA)6	12	48808750	48808761
chr1_8390	p2	(TG)7	14	48811716	48811729
chr1_8391	p3	(TTG)5	15	48818809	48818823
chr1_8392	p2	(AC)7	14	48822663	48822676
chr1_8393	p2	(TG)6	12	48823676	48823687
chr1_8394	c	tgttttactggaaagcaccttgtaacgcaggtttagaaaggtgctatataaat	101	48831552	48831652
chr1_8395	p2	(CA)10	20	48842970	48842989
chr1_8396	p2	(TG)17	34	48850480	48850513
chr1_8397	p2	(TG)9	18	48885081	48885098
chr1_8398	p2	(AT)6	12	48894408	48894419
chr1_8399	p2	(TG)19	38	48912605	48912642
chr1_8400	p2	(CA)13	26	48920762	48920787
chr1_8401	p2	(AC)6	12	48926413	48926424
chr1_8402	p2	(CT)8	16	48926581	48926596
chr1_8403	p2	(AC)19	38	48928160	48928197
chr1_8404	p3	(CTT)8	24	48929995	48930018
chr1_8405	p4	(TTTG)8	32	48930625	48930656
chr1_8406	p2	(TA)7	14	48932991	48933004
chr1_8407	p2	(TG)6	12	48940829	48940840
chr1_8408	p2	(CA)6	12	48941168	48941179
chr1_8409	p3	(CAG)5	15	48955850	48955864
chr1_8410	p2	(TG)7	14	48958287	48958300
chr1_8411	p5	(ATCAA)5	25	48963430	48963454
chr1_8412	p2	(TA)9	18	48974928	48974945
chr1_8413	p3	(TTA)8	24	49010666	49010689
chr1_8414	p3	(GTG)6	18	49028229	49028246
chr1_8415	p2	(TG)10	20	49037426	49037445
chr1_8416	p3	(AGC)6	18	49086117	49086134
chr1_8417	p2	(TG)11	22	49089927	49089948
chr1_8418	p2	(GT)8	16	49090183	49090198
chr1_8419	p3	(CTC)11	33	49092192	49092224
chr1_8420	p2	(AC)7	14	49093881	49093894

chr1_8421	p2	(GT)12	24	49098821	49098844
chr1_8422	p3	(TTG)5	15	49110117	49110131
chr1_8423	p3	(AAC)6	18	49127181	49127198
chr1_8424	p2	(AC)6	12	49149428	49149439
chr1_8425	p3	(CCT)5	15	49163467	49163481
chr1_8426	p2	(TG)17	34	49168126	49168159
chr1_8427	p2	(TG)6	12	49169992	49170003
chr1_8428	p2	(AC)8	16	49177304	49177319
chr1_8429	p4	(CTAT)10	40	49193627	49193666
chr1_8430	p2	(TG)10	20	49197146	49197165
chr1_8431	p3	(TCC)5	15	49200882	49200896
chr1_8432	p2	(CA)7	14	49202399	49202412
chr1_8433	p3	(TAA)5	15	49205737	49205751
chr1_8434	p2	(TG)20	40	49214126	49214165
chr1_8435	p2	(TG)6	12	49218543	49218554
chr1_8436	p2	(AC)8	16	49218837	49218852
chr1_8437	p2	(GT)9	18	49227325	49227342
chr1_8438	p2	(CA)6	12	49228158	49228169
chr1_8439	p2	(GT)12	24	49228613	49228636
chr1_8440	p2	(TG)12	24	49228794	49228817
chr1_8441	p2	(CA)7	14	49232889	49232902
chr1_8442	p2	(TG)15	30	49239651	49239680
chr1_8443	p2	(TG)24	48	49242840	49242887
chr1_8444	p2	(TG)10	20	49244729	49244748
chr1_8445	p2	(TC)11	22	49249185	49249206
chr1_8446	c	(AT)7c(AG)7	29	49249480	49249508
chr1_8447	p2	(TG)15	30	49249798	49249827
chr1_8448	p3	(GGT)5	15	49256776	49256790
chr1_8449	p2	(CA)6	12	49265001	49265012
chr1_8450	p2	(GT)6	12	49265227	49265238
chr1_8451	p2	(TC)6	12	49265654	49265665
chr1_8452	p2	(TG)13	26	49270460	49270485
chr1_8453	p2	(GT)7	14	49281663	49281676
chr1_8454	p2	(GA)7	14	49288469	49288482
chr1_8455	p3	(TTC)5	15	49292675	49292689
chr1_8456	p2	(TG)8	16	49293377	49293392
chr1_8457	p2	(TG)7	14	49298759	49298772
chr1_8458	p2	(GT)9	18	49304332	49304349
chr1_8459	p4	(CTAC)6	24	49308408	49308431
chr1_8460	p2	(TG)10	20	49311421	49311440
chr1_8461	p2	(CA)14	28	49311641	49311668
chr1_8462	p2	(TG)20	40	49312577	49312616
chr1_8463	p2	(TC)6	12	49316201	49316212
chr1_8464	p2	(TG)13	26	49325160	49325185
chr1_8465	p2	(GT)6	12	49328170	49328181
chr1_8466	c	(GT)26(GA)13	78	49336333	49336410
chr1_8467	c	(AAT)5t(ATG)5	31	49338907	49338937
chr1_8468	p2	(AC)34	68	49339444	49339511
chr1_8469	p2	(TG)6	12	49354608	49354619
chr1_8470	p2	(GA)6	12	49355857	49355868
chr1_8471	p3	(AAG)5	15	49356091	49356105
chr1_8472	p2	(GT)11	22	49357228	49357249

chr1_8473	p2	(TG)6	12	49359445	49359456
chr1_8474	p4	(AAAT)5	20	49360182	49360201
chr1_8475	p2	(TG)6	12	49380684	49380695
chr1_8476	p2	(TG)6	12	49383061	49383072
chr1_8477	p2	(CA)6	12	49391205	49391216
chr1_8478	p2	(CA)21	42	49392904	49392945
chr1_8479	p2	(TG)9	18	49395842	49395859
chr1_8480	p3	(CCT)6	18	49397381	49397398
chr1_8481	p2	(TG)14	28	49398983	49399010
chr1_8482	p2	(AC)10	20	49400210	49400229
chr1_8483	p3	(TTA)5	15	49401183	49401197
chr1_8484	p2	(GT)23	46	49401757	49401802
chr1_8485	p2	(CT)7	14	49404418	49404431
chr1_8486	p2	(TG)7	14	49409550	49409563
chr1_8487	p3	(TTC)5	15	49410995	49411009
chr1_8488	p2	(GT)6	12	49413522	49413533
chr1_8489	c	)11(CA)13cctttggtctgtgtgtgtttaaggacacgaccacatag(A	99	49414768	49414866
chr1_8490	p2	(GT)6	12	49415437	49415448
chr1_8491	p2	(TG)7	14	49422620	49422633
chr1_8492	p2	(AC)6	12	49425205	49425216
chr1_8493	p2	(TG)21	42	49427682	49427723
chr1_8494	p2	(CA)14	28	49430267	49430294
chr1_8495	p2	(AC)9	18	49442188	49442205
chr1_8496	p3	(CCT)5	15	49444026	49444040
chr1_8497	p2	(CT)6	12	49448456	49448467
chr1_8498	p2	(TG)6	12	49450225	49450236
chr1_8499	p2	(TG)11	22	49454220	49454241
chr1_8500	p3	(CCA)5	15	49460607	49460621
chr1_8501	p4	(AATA)5	20	49466506	49466525
chr1_8502	p2	(GT)9	18	49468162	49468179
chr1_8503	c	gcgcgtgcatgtgcaggtgtgcatgcataggcccactgccatgctttgtgta	90	49468556	49468645
chr1_8504	p3	(TCC)12	36	49501835	49501870
chr1_8505	p4	(TTTG)6	24	49509174	49509197
chr1_8506	p2	(AC)8	16	49510736	49510751
chr1_8507	p2	(TC)6	12	49515005	49515016
chr1_8508	p2	(AC)6	12	49542391	49542402
chr1_8509	p2	(GT)11	22	49585336	49585357
chr1_8510	p2	(CA)30	60	49600396	49600455
chr1_8511	p3	(ATG)6	18	49610767	49610784
chr1_8512	p4	(AAAT)5	20	49615912	49615931
chr1_8513	p2	(GT)7	14	49617886	49617899
chr1_8514	p2	(TG)6	12	49620403	49620414
chr1_8515	p2	(AC)29	58	49623970	49624027
chr1_8516	p2	(CT)8	16	49624695	49624710
chr1_8517	p2	(CA)14	28	49626055	49626082
chr1_8518	p2	(AC)17	34	49632407	49632440
chr1_8519	p2	(AC)6	12	49648552	49648563
chr1_8520	p3	(CTT)8	24	49657127	49657150
chr1_8521	p2	(TA)8	16	49663575	49663590
chr1_8522	p2	(TA)8	16	49665361	49665376
chr1_8523	p2	(AC)11	22	49671558	49671579
chr1_8524	p2	(CA)26	52	49697023	49697074

chr1_8525	p2	(GT)6	12	49697504	49697515
chr1_8526	p2	(TG)10	20	49717525	49717544
chr1_8527	p2	(AG)8	16	49726168	49726183
chr1_8528	p3	(TCC)5	15	49735759	49735773
chr1_8529	p2	(GT)7	14	49736507	49736520
chr1_8530	p2	(TG)15	30	49758580	49758609
chr1_8531	c	(TAC)10(TAA)7	51	49763965	49764015
chr1_8532	p2	(TG)15	30	49778005	49778034
chr1_8533	p2	(CT)23	46	49803511	49803556
chr1_8534	p2	(GA)6	12	49804716	49804727
chr1_8535	p2	(CA)6	12	49807050	49807061
chr1_8536	p2	(TA)7	14	49810960	49810973
chr1_8537	p2	(TG)6	12	49816179	49816190
chr1_8538	p4	(TTCA)5	20	49816915	49816934
chr1_8539	p2	(AC)9	18	49818796	49818813
chr1_8540	c	(TG)14(AG)25	78	49818937	49819014
chr1_8541	p2	(AG)6	12	49824061	49824072
chr1_8542	p2	(AC)6	12	49830833	49830844
chr1_8543	p2	(TG)7	14	49836244	49836257
chr1_8544	p2	(AC)6	12	49852846	49852857
chr1_8545	p2	(GT)17	34	49853561	49853594
chr1_8546	p4	(TGAT)8	32	49858221	49858252
chr1_8547	p2	(TC)6	12	49863310	49863321
chr1_8548	p2	(AC)6	12	49864231	49864242
chr1_8549	p2	(TG)8	16	49866351	49866366
chr1_8550	p2	(CA)20	40	49868755	49868794
chr1_8551	c	atatgctgtgcagcttaatgtttctggaactcaaaatcagctttgcctgtttgc	137	49870171	49870307
chr1_8552	p4	(TTCA)5	20	49873792	49873811
chr1_8553	p3	(TAT)5	15	49875994	49876008
chr1_8554	c	(AC)9(AT)9	36	49882923	49882958
chr1_8555	p2	(TG)9	18	49887905	49887922
chr1_8556	p2	(TG)6	12	49892515	49892526
chr1_8557	p2	(TG)6	12	49893701	49893712
chr1_8558	p2	(GT)7	14	49902176	49902189
chr1_8559	p2	(TC)10	20	49907339	49907358
chr1_8560	p2	(CA)11	22	49907605	49907626
chr1_8561	p2	(TA)7	14	49935443	49935456
chr1_8562	p2	(TG)20	40	49935563	49935602
chr1_8563	p2	(TG)7	14	49937611	49937624
chr1_8564	p2	(CT)13	26	49953762	49953787
chr1_8565	p2	(CA)8	16	49964530	49964545
chr1_8566	p2	(TG)8	16	49967974	49967989
chr1_8567	p2	(AG)9	18	49975362	49975379
chr1_8568	p2	(GT)16	32	49980697	49980728
chr1_8569	p2	(GT)6	12	49984207	49984218
chr1_8570	p2	(AG)7	14	49987013	49987026
chr1_8571	p3	(TAA)6	18	49992559	49992576
chr1_8572	p2	(AC)11	22	50004984	50005005
chr1_8573	p2	(TC)7	14	50006003	50006016
chr1_8574	p2	(TC)7	14	50010998	50011011
chr1_8575	p2	(GT)12	24	50012393	50012416
chr1_8576	c	(TC)10(AC)15	50	50016564	50016613

chr1_8577	p2	(GA)8	16	50019068	50019083
chr1_8578	p2	(AC)16	32	50019373	50019404
chr1_8579	c	(TG)14(AG)10	48	50019988	50020035
chr1_8580	c	(CA)7cg(CA)12	40	50028278	50028317
chr1_8581	p2	(TG)17	34	50029563	50029596
chr1_8582	p2	(TG)7	14	50041640	50041653
chr1_8583	p2	(CT)7	14	50045408	50045421
chr1_8584	p2	(GT)6	12	50046785	50046796
chr1_8585	p2	(TG)6	12	50048094	50048105
chr1_8586	p4	(ATGG)5	20	50053169	50053188
chr1_8587	p3	(TTG)6	18	50053520	50053537
chr1_8588	p2	(TG)6	12	50058895	50058906
chr1_8589	p2	(CA)13	26	50063010	50063035
chr1_8590	p2	(GT)11	22	50072694	50072715
chr1_8591	p3	(AAG)6	18	50073551	50073568
chr1_8592	p2	(AT)8	16	50073958	50073973
chr1_8593	p3	(CTT)5	15	50076945	50076959
chr1_8594	p3	(AGC)5	15	50080855	50080869
chr1_8595	p2	(CT)10	20	50087820	50087839
chr1_8596	c	(AC)10atatagctagc(TAGA)5	51	50089632	50089682
chr1_8597	p2	(AC)15	30	50094894	50094923
chr1_8598	p2	(CA)7	14	50102933	50102946
chr1_8599	p2	(AC)15	30	50103491	50103520
chr1_8600	p4	(GTCT)5	20	50104948	50104967
chr1_8601	p3	(AAC)5	15	50108981	50108995
chr1_8602	p3	(GAT)5	15	50112985	50112999
chr1_8603	p2	(GT)9	18	50124771	50124788
chr1_8604	p2	(CA)7	14	50125518	50125531
chr1_8605	p3	(TTG)6	18	50131953	50131970
chr1_8606	p2	(CA)6	12	50142098	50142109
chr1_8607	p2	(TG)10	20	50153888	50153907
chr1_8608	p3	(CAA)6	18	50156757	50156774
chr1_8609	p3	(TAC)5	15	50158014	50158028
chr1_8610	p4	(ATCC)5	20	50163225	50163244
chr1_8611	p2	(TG)6	12	50169129	50169140
chr1_8612	p3	(ATT)5	15	50171327	50171341
chr1_8613	p2	(TG)13	26	50183268	50183293
chr1_8614	p2	(AC)18	36	50183486	50183521
chr1_8615	p2	(AC)18	36	50186657	50186692
chr1_8616	p2	(AC)6	12	50192632	50192643
chr1_8617	p2	(AG)9	18	50195392	50195409
chr1_8618	p2	(GT)6	12	50205893	50205904
chr1_8619	p2	(TG)10	20	50207919	50207938
chr1_8620	p5	(TTGAT)6	30	50209444	50209473
chr1_8621	p2	(TG)9	18	50212028	50212045
chr1_8622	p2	(GT)15	30	50216435	50216464
chr1_8623	p2	(AC)11	22	50224281	50224302
chr1_8624	p2	(TG)7	14	50244532	50244545
chr1_8625	p2	(AC)7	14	50248168	50248181
chr1_8626	p2	(TG)13	26	50248328	50248353
chr1_8627	p2	(AC)7	14	50282779	50282792
chr1_8628	c	ggaggggctnnnnnnnnnnnnnnnnnnnnnnnnnnnnnnnnnn	180	50284359	50284538

chr1_8629	p2	(AC)6	12	50309744	50309755
chr1_8630	p2	(GT)28	56	50310685	50310740
chr1_8631	p2	(GT)17	34	50310938	50310971
chr1_8632	p2	(AC)6	12	50311101	50311112
chr1_8633	p2	(TG)6	12	50315035	50315046
chr1_8634	p2	(TG)6	12	50315319	50315330
chr1_8635	p2	(GT)7	14	50315701	50315714
chr1_8636	p2	(CT)6	12	50331644	50331655
chr1_8637	p2	(TG)8	16	50341174	50341189
chr1_8638	c	(TC)11g(CT)7(CA)8	53	50349621	50349673
chr1_8639	p2	(AT)8	16	50352753	50352768
chr1_8640	p2	(CA)15	30	50357850	50357879
chr1_8641	p2	(CA)18	36	50358162	50358197
chr1_8642	p2	(TG)20	40	50360415	50360454
chr1_8643	p3	(AGC)5	15	50361682	50361696
chr1_8644	p2	(CA)6	12	50363111	50363122
chr1_8645	p2	(TG)8	16	50387691	50387706
chr1_8646	p2	(TG)7	14	50401115	50401128
chr1_8647	p2	(GT)7	14	50405790	50405803
chr1_8648	p2	(AC)21	42	50411116	50411157
chr1_8649	p2	(AC)13	26	50411682	50411707
chr1_8650	p2	(GT)7	14	50412243	50412256
chr1_8651	p2	(CA)6	12	50415882	50415893
chr1_8652	p2	(GT)6	12	50416008	50416019
chr1_8653	c	(TG)11(TA)6	34	50418345	50418378
chr1_8654	p2	(AC)6	12	50418916	50418927
chr1_8655	p2	(AC)9	18	50439840	50439857
chr1_8656	p2	(AC)8	16	50443368	50443383
chr1_8657	p2	(GT)10	20	50446057	50446076
chr1_8658	p2	(GT)12	24	50446646	50446669
chr1_8659	p2	(AC)22	44	50452021	50452064
chr1_8660	p2	(GA)9	18	50458018	50458035
chr1_8661	p2	(TC)7	14	50463455	50463468
chr1_8662	p2	(TG)7	14	50463884	50463897
chr1_8663	c	(GT)6g(GT)30	73	50470793	50470865
chr1_8664	p3	(AAG)6	18	50478058	50478075
chr1_8665	p2	(GT)24	48	50483185	50483232
chr1_8666	p2	(AC)10	20	50487725	50487744
chr1_8667	p2	(TG)6	12	50492662	50492673
chr1_8668	p2	(TG)11	22	50498037	50498058
chr1_8669	p2	(TG)8	16	50498355	50498370
chr1_8670	p2	(CA)10	20	50498571	50498590
chr1_8671	p4	(TACC)7	28	50501187	50501214
chr1_8672	p2	(AG)6	12	50501808	50501819
chr1_8673	p2	(GT)8	16	50502290	50502305
chr1_8674	p2	(GT)12	24	50508352	50508375
chr1_8675	c	accctcctcttcctctattcccgtcatgccttgcagatatttctgctccccccc	103	50528605	50528707
chr1_8676	c	gaggatcggtgtgtctgacctcattcctatcagactccaccgaaaaacttatt	102	50532806	50532907
chr1_8677	p2	(TG)7	14	50534536	50534549
chr1_8678	p2	(GT)12	24	50534863	50534886
chr1_8679	p2	(GT)17	34	50537471	50537504
chr1_8680	c	(TG)10(AG)7	34	50540909	50540942

chr1_8681	p2	(TG)9	18	50542497	50542514
chr1_8682	p2	(TG)14	28	50542696	50542723
chr1_8683	c	(TG)8tatatata(TG)6	36	50544471	50544506
chr1_8684	p2	(TG)6	12	50544717	50544728
chr1_8685	p2	(AC)14	28	50550659	50550686
chr1_8686	p2	(TG)6	12	50551268	50551279
chr1_8687	c	(CT)10(CA)8	36	50553520	50553555
chr1_8688	p2	(AC)11	22	50555152	50555173
chr1_8689	p2	(CT)7	14	50556470	50556483
chr1_8690	p2	(CA)14	28	50556634	50556661
chr1_8691	c	(GT)6(GA)6	24	50572124	50572147
chr1_8692	p2	(TC)6	12	50575846	50575857
chr1_8693	p2	(TG)28	56	50593110	50593165
chr1_8694	p2	(AC)13	26	50600325	50600350
chr1_8695	p4	(CAGA)5	20	50604107	50604126
chr1_8696	p3	(ATA)6	18	50607777	50607794
chr1_8697	p3	(TTG)5	15	50609804	50609818
chr1_8698	p2	(AC)6	12	50610240	50610251
chr1_8699	p2	(CA)12	24	50615009	50615032
chr1_8700	p3	(AGT)5	15	50616482	50616496
chr1_8701	p3	(AAT)6	18	50623531	50623548
chr1_8702	c	(CT)21(CA)6	54	50624819	50624872
chr1_8703	p3	(AAT)5	15	50629016	50629030
chr1_8704	p3	(TCT)5	15	50631696	50631710
chr1_8705	p4	(CCTG)7	28	50637677	50637704
chr1_8706	p2	(GT)8	16	50640288	50640303
chr1_8707	p2	(AC)7	14	50640726	50640739
chr1_8708	p2	(CT)6	12	50643747	50643758
chr1_8709	p2	(TG)7	14	50656679	50656692
chr1_8710	p2	(AT)6	12	50656970	50656981
chr1_8711	p4	(TACC)6	24	50666065	50666088
chr1_8712	p2	(CA)6	12	50669848	50669859
chr1_8713	c	(CT)9cac(AG)15	51	50670717	50670767
chr1_8714	p2	(CA)11	22	50670940	50670961
chr1_8715	p2	(CA)18	36	50676805	50676840
chr1_8716	p2	(AC)7	14	50683159	50683172
chr1_8717	p2	(CA)7	14	50684121	50684134
chr1_8718	c	ggcttcagtctcaatgcagagtgtgaatgtgaggggaatgaaaggaacgg	150	50684270	50684419
chr1_8719	p2	(AG)6	12	50684876	50684887
chr1_8720	p2	(TC)6	12	50686694	50686705
chr1_8721	p4	(AATC)6	24	50694304	50694327
chr1_8722	c	(CT)10(CA)6	32	50699145	50699176
chr1_8723	p3	(TCA)5	15	50707832	50707846
chr1_8724	p3	(CTT)5	15	50708410	50708424
chr1_8725	p2	(AG)8	16	50712400	50712415
chr1_8726	p2	(AC)14	28	50713235	50713262
chr1_8727	p3	(ATA)5	15	50718978	50718992
chr1_8728	p2	(CA)15	30	50725158	50725187
chr1_8729	p2	(AC)19	38	50727714	50727751
chr1_8730	p2	(TA)6	12	50732706	50732717
chr1_8731	p2	(AC)6	12	50734672	50734683
chr1_8732	p2	(TG)14	28	50734970	50734997

chr1_8733	p2	(TG)6	12	50735961	50735972
chr1_8734	p4	(GATA)23	92	50739602	50739693
chr1_8735	p2	(CA)8	16	50756928	50756943
chr1_8736	p2	(TG)12	24	50758304	50758327
chr1_8737	c*	(CTAT)7(TA)10*	45	50767472	50767516
chr1_8738	p2	(GT)11	22	50775335	50775356
chr1_8739	p2	(TG)6	12	50785653	50785664
chr1_8740	p2	(TG)13	26	50795304	50795329
chr1_8741	p2	(AC)8	16	50807512	50807527
chr1_8742	c	(ACT)5(AAT)7	36	50829997	50830032
chr1_8743	p2	(AC)8	16	50848456	50848471
chr1_8744	p2	(TG)23	46	50863790	50863835
chr1_8745	p3	(AGT)5	15	50868284	50868298
chr1_8746	p3	(TTA)5	15	50879620	50879634
chr1_8747	c	(TC)8(TG)12	40	50881348	50881387
chr1_8748	c	(CA)14cc(CA)19	68	50883949	50884016
chr1_8749	p2	(GT)6	12	50884437	50884448
chr1_8750	c	(TG)7tataaattgatcctttgctcaat(TG)10	57	50885688	50885744
chr1_8751	p2	(TA)6	12	50887188	50887199
chr1_8752	p2	(AC)17	34	50896524	50896557
chr1_8753	p2	(AC)19	38	50899796	50899833
chr1_8754	p3	(TTA)16	48	50902170	50902217
chr1_8755	p2	(AC)9	18	50902628	50902645
chr1_8756	p2	(TC)7	14	50906174	50906187
chr1_8757	p2	(GA)10	20	50908859	50908878
chr1_8758	p2	(AC)11	22	50918179	50918200
chr1_8759	p2	(AT)9	18	50919505	50919522
chr1_8760	p3	(TAA)9	27	50919740	50919766
chr1_8761	p2	(GA)6	12	50923260	50923271
chr1_8762	c	(TC)7cctctctcttt(CA)38	101	50925452	50925552
chr1_8763	p2	(AC)7	14	50927825	50927838
chr1_8764	p3	(TTA)5	15	50932113	50932127
chr1_8765	c	(GT)20(GA)14	68	50934687	50934754
chr1_8766	p4	(TTTG)5	20	50935296	50935315
chr1_8767	p2	(TG)10	20	50937712	50937731
chr1_8768	p3	(CTG)5	15	50938553	50938567
chr1_8769	p3	(CAA)7	21	50939582	50939602
chr1_8770	p2	(TG)22	44	50940806	50940849
chr1_8771	p2	(CA)18	36	50941034	50941069
chr1_8772	p3	(AAG)13	39	50942460	50942498
chr1_8773	p3	(CAG)7	21	50943953	50943973
chr1_8774	p3	(AAG)5	15	50944559	50944573
chr1_8775	c	ıacagagataaagaaagagagggagagagcgcgcgcaggagagactcg	121	50944691	50944811
chr1_8776	p2	(TG)11	22	50946562	50946583
chr1_8777	c	(AG)9gggggg(GA)9	42	50946786	50946827
chr1_8778	p2	(GT)13	26	50950235	50950260
chr1_8779	p3	(CCT)5	15	50952249	50952263
chr1_8780	p2	(CA)7	14	50956815	50956828
chr1_8781	p2	(AC)13	26	50957051	50957076
chr1_8782	p2	(GT)8	16	50957802	50957817
chr1_8783	p4	(TAGA)13	52	50963387	50963438
chr1_8784	p4	(ATAG)8	32	50964521	50964552

chr1_8785	c	(TG)7ta(TG)9	34	50967224	50967257
chr1_8786	c	(CT)11(CA)8	38	50967532	50967569
chr1_8787	p3	(ATT)5	15	50967757	50967771
chr1_8788	p3	(ATT)10	30	50970185	50970214
chr1_8789	c	agtggtagcctaaatacaattgctccagcgataacatcatggacaacacac	95	50970417	50970511
chr1_8790	p2	(CA)10	20	50971634	50971653
chr1_8791	p2	(GA)6	12	50973704	50973715
chr1_8792	p2	(GT)29	58	50974220	50974277
chr1_8793	p2	(TG)6	12	50974482	50974493
chr1_8794	p2	(TA)8	16	50974729	50974744
chr1_8795	p2	(CA)6	12	50975682	50975693
chr1_8796	p3	(TCC)5	15	50976560	50976574
chr1_8797	p3	(ATT)10	30	50977799	50977828
chr1_8798	p2	(CA)10	20	50981093	50981112
chr1_8799	c	(AC)12t(CA)12	49	50981697	50981745
chr1_8800	p3	(TTA)6	18	50982389	50982406
chr1_8801	p2	(AG)6	12	50982829	50982840
chr1_8802	c	(CA)13gctccatccaacaa(AC)6	52	50983004	50983055
chr1_8803	p2	(GA)7	14	50987814	50987827
chr1_8804	p2	(AG)6	12	50988010	50988021
chr1_8805	p2	(TG)18	36	50988315	50988350
chr1_8806	p2	(AC)22	44	50988800	50988843
chr1_8807	c	cctctttcccagtatgacaaaaatgaatcttgtatttccggtgtgcgatagcg	117	50991365	50991481
chr1_8808	p2	(CA)6	12	50992348	50992359
chr1_8809	p2	(AG)7	14	50996166	50996179
chr1_8810	p2	(CA)6	12	50996401	50996412
chr1_8811	p2	(AG)8	16	50996826	50996841
chr1_8812	p2	(GT)20	40	50997117	50997156
chr1_8813	c*	(GATA)15(AG)7*	73	50997486	50997558
chr1_8814	p4	(TAGA)16	64	51000136	51000199
chr1_8815	p2	(TG)16	32	51001631	51001662
chr1_8816	p3	(ATA)7	21	51005191	51005211
chr1_8817	p2	(AC)13	26	51008609	51008634
chr1_8818	p3	(CCA)7	21	51009712	51009732
chr1_8819	p2	(CA)6	12	51010020	51010031
chr1_8820	p2	(GT)11	22	51010903	51010924
chr1_8821	c	gttcaatgaccaccacacaccaacaaacagctacagtaacaccgacctc	103	51014231	51014333
chr1_8822	p2	(GT)8	16	51014558	51014573
chr1_8823	p3	(ATA)11	33	51016484	51016516
chr1_8824	c	aaaaaacatacaagccccctatagcagcggagagtgggg(GA)6aacg	184	51018969	51019152
chr1_8825	p2	(GA)7	14	51019494	51019507
chr1_8826	p3	(GCA)8	24	51021473	51021496
chr1_8827	p3	(AAT)6	18	51022949	51022966
chr1_8828	p2	(AC)6	12	51024141	51024152
chr1_8829	p2	(TC)8	16	51026327	51026342
chr1_8830	c	tgagcattggtgcaaaatgctagctataaggtaaacatgcaaatataatgta	117	51026913	51027029
chr1_8831	c	(GTA)11(GTG)7	54	51032976	51033029
chr1_8832	p3	(TTA)5	15	51034660	51034674
chr1_8833	p2	(TG)16	32	51034858	51034889
chr1_8834	p2	(CA)8	16	51035790	51035805
chr1_8835	p4	(ATCT)5	20	51040107	51040126
chr1_8836	p2	(CA)9	18	51048763	51048780

chr1_8837	p2	(CT)8	16	51048972	51048987
chr1_8838	p2	(TG)6	12	51052905	51052916
chr1_8839	p3	(CCT)5	15	51057159	51057173
chr1_8840	p2	(AC)24	48	51058104	51058151
chr1_8841	c	gaaaggaacagaacaatgacgtgttcttaggtgacagattcagtacagattt	124	51059093	51059216
chr1_8842	p2	(TC)7	14	51062062	51062075
chr1_8843	p2	(TG)11	22	51062181	51062202
chr1_8844	p2	(AT)7	14	51066018	51066031
chr1_8845	p2	(GT)18	36	51066985	51067020
chr1_8846	p2	(AT)9	18	51069351	51069368
chr1_8847	p2	(TA)6	12	51069551	51069562
chr1_8848	p2	(AG)7	14	51072596	51072609
chr1_8849	p4	(ATCC)6	24	51073737	51073760
chr1_8850	p3	(TCC)5	15	51080551	51080565
chr1_8851	p3	(TGA)5	15	51081558	51081572
chr1_8852	p2	(CA)7	14	51085134	51085147
chr1_8853	p2	(CA)6	12	51085353	51085364
chr1_8854	p2	(TG)15	30	51086492	51086521
chr1_8855	p2	(GT)10	20	51088492	51088511
chr1_8856	c	acaggcctgaacagatatgactgtttgccttagcaaacgctgtgtactccat	128	51089030	51089157
chr1_8857	p2	(AC)6	12	51089353	51089364
chr1_8858	p2	(CA)8	16	51089532	51089547
chr1_8859	p2	(CA)8	16	51091230	51091245
chr1_8860	p2	(AC)6	12	51091441	51091452
chr1_8861	p2	(CA)8	16	51091620	51091635
chr1_8862	p2	(GT)25	50	51095171	51095220
chr1_8863	p2	(AC)8	16	51096005	51096020
chr1_8864	c	gtgtgtgtgagaatgactgcatcagtgtgcttacctgcaagtgtagtgtgtac	96	51099542	51099637
chr1_8865	p2	(AC)16	32	51101094	51101125
chr1_8866	c	catatgcacatactcagtgaacatctgtcaggcagtcagtgtctcttatttcct	87	51101278	51101364
chr1_8867	c	ataattaaatgccaataggggacaaaatctcatttgcagtgagggtctttata	136	51102057	51102192
chr1_8868	p2	(TG)7	14	51104769	51104782
chr1_8869	p2	(GT)7	14	51104904	51104917
chr1_8870	p2	(AT)6	12	51105837	51105848
chr1_8871	p4	(TAGA)15	60	51107431	51107490
chr1_8872	p2	(TG)6	12	51111910	51111921
chr1_8873	c	acaaggttgtaggagctcgttaaccccacacacaccatagctaaacccga	116	51113507	51113622
chr1_8874	p2	(TG)9	18	51113948	51113965
chr1_8875	p2	(TG)29	58	51115287	51115344
chr1_8876	p2	(TC)7	14	51117252	51117265
chr1_8877	p2	(CA)8	16	51124748	51124763
chr1_8878	p2	(AT)6	12	51127592	51127603
chr1_8879	c	(ATGCC)5(ATACC)46	255	51133574	51133828
chr1_8880	p2	(TA)8	16	51134023	51134038
chr1_8881	c	(CA)9(TA)8	34	51140399	51140432
chr1_8882	p2	(GT)19	38	51140547	51140584
chr1_8883	p2	(AC)9	18	51145030	51145047
chr1_8884	c	ggtcttagtgtggaatactcctcatatatcatcctgtgtgtgagagtgtgtctc	99	51148874	51148972
chr1_8885	p2	(GT)22	44	51149164	51149207
chr1_8886	p5	(TTTGA)5	25	51153298	51153322
chr1_8887	p2	(CA)10	20	51158004	51158023
chr1_8888	p2	(AC)10	20	51165663	51165682

chr1_8889	c	4agtgaggcatttacaaagtggagtcaaatggaattgtataataggagcca(	100	51166062	51166161
chr1_8890	p2	(AC)16	32	51172335	51172366
chr1_8891	p2	(AG)6	12	51178497	51178508
chr1_8892	p2	(CA)16	32	51190086	51190117
chr1_8893	p2	(GA)13	26	51190474	51190499
chr1_8894	c	cagctagcaagtaagccttccaaaacacaacctgctgtttggacattacac	171	51190626	51190796
chr1_8895	p2	(TG)12	24	51194413	51194436
chr1_8896	p2	(GT)8	16	51194586	51194601
chr1_8897	p2	(TG)9	18	51194753	51194770
chr1_8898	p2	(CA)17	34	51194880	51194913
chr1_8899	p3	(AAG)5	15	51195398	51195412
chr1_8900	p4	(CTAC)5	20	51203094	51203113
chr1_8901	p2	(GA)9	18	51204475	51204492
chr1_8902	p2	(TG)10	20	51208969	51208988
chr1_8903	p2	(CA)11	22	51210285	51210306
chr1_8904	p2	(AG)6	12	51211845	51211856
chr1_8905	p2	(CA)6	12	51211994	51212005
chr1_8906	p2	(AC)6	12	51212490	51212501
chr1_8907	p2	(CA)6	12	51215090	51215101
chr1_8908	p2	(CA)6	12	51215223	51215234
chr1_8909	p2	(AC)6	12	51216241	51216252
chr1_8910	p2	(CA)11	22	51216416	51216437
chr1_8911	p2	(AT)6	12	51217561	51217572
chr1_8912	p5	(TTAGA)9	45	51218460	51218504
chr1_8913	c	(TC)8(AC)17	50	51224184	51224233
chr1_8914	p2	(AC)6	12	51225444	51225455
chr1_8915	p4	(GACA)9	36	51231332	51231367
chr1_8916	p2	(TG)8	16	51255440	51255455
chr1_8917	p2	(AC)6	12	51256979	51256990
chr1_8918	p2	(GT)7	14	51257619	51257632
chr1_8919	p3	(ACA)5	15	51258318	51258332
chr1_8920	p2	(AT)6	12	51262055	51262066
chr1_8921	p2	(AC)15	30	51263908	51263937
chr1_8922	p2	(GA)6	12	51269369	51269380
chr1_8923	p2	(TC)6	12	51274178	51274189
chr1_8924	p2	(GA)7	14	51288415	51288428
chr1_8925	c	(GT)7atgtatgtatgtgc(AT)13	54	51290721	51290774
chr1_8926	p3	(TTG)5	15	51292803	51292817
chr1_8927	p2	(GT)23	46	51298724	51298769
chr1_8928	c	tgtgggagggagagaaagagagagagtgggtgggctaaaaactaaaga	169	51299268	51299436
chr1_8929	p2	(CA)11	22	51301359	51301380
chr1_8930	c	(CA)6ggcttgttgtcagtgcgcctttatttt(CA)6	51	51303272	51303322
chr1_8931	p2	(GT)15	30	51303541	51303570
chr1_8932	p3	(AAT)5	15	51304757	51304771
chr1_8933	p2	(GT)6	12	51304965	51304976
chr1_8934	p2	(GT)6	12	51305224	51305235
chr1_8935	p2	(TG)14	28	51305355	51305382
chr1_8936	p2	(GT)10	20	51311323	51311342
chr1_8937	c	(AC)11gcaa(AG)6aatc(CT)7	56	51314839	51314894
chr1_8938	p2	(AG)6	12	51315043	51315054
chr1_8939	p2	(TG)6	12	51315334	51315345
chr1_8940	p2	(TC)11	22	51316690	51316711

chr1_8941	p2	(AT)6	12	51317152	51317163
chr1_8942	p2	(CA)6	12	51318889	51318900
chr1_8943	p2	(AC)8	16	51321006	51321021
chr1_8944	p2	(CA)6	12	51321129	51321140
chr1_8945	c	ıtatctat(TG)8tctgtgtgtgtgcagctagctaatgatatctattttcttagtg	219	51325719	51325937
chr1_8946	c	(TG)10(AG)6	32	51327661	51327692
chr1_8947	p2	(TA)6	12	51328478	51328489
chr1_8948	p2	(GT)6	12	51329787	51329798
chr1_8949	c	cacatgcacaggttctggcagatacataaatactctccgttctgtgcctctct	124	51330020	51330143
chr1_8950	p2	(AC)7	14	51330889	51330902
chr1_8951	p2	(AC)12	24	51331226	51331249
chr1_8952	p2	(AC)11	22	51331426	51331447
chr1_8953	p3	(AAG)5	15	51331866	51331880
chr1_8954	p3	(TAT)5	15	51338851	51338865
chr1_8955	c	(GT)21(GA)7	56	51349446	51349501
chr1_8956	p2	(TG)6	12	51363513	51363524
chr1_8957	p2	(CA)8	16	51368278	51368293
chr1_8958	p3	(AAC)9	27	51368983	51369009
chr1_8959	p2	(TG)6	12	51374926	51374937
chr1_8960	p2	(TG)6	12	51375302	51375313
chr1_8961	p2	(TA)6	12	51381175	51381186
chr1_8962	p2	(GA)8	16	51395047	51395062
chr1_8963	p4	(AACA)5	20	51399645	51399664
chr1_8964	p2	(AC)13	26	51413138	51413163
chr1_8965	p2	(AC)12	24	51416268	51416291
chr1_8966	p2	(TG)11	22	51420554	51420575
chr1_8967	p3	(ACA)6	18	51420708	51420725
chr1_8968	p2	(AC)6	12	51421815	51421826
chr1_8969	p2	(CT)6	12	51425954	51425965
chr1_8970	p2	(CA)9	18	51427996	51428013
chr1_8971	p2	(TG)6	12	51429399	51429410
chr1_8972	p2	(GT)30	60	51430250	51430309
chr1_8973	p5	(ACAAA)5	25	51436367	51436391
chr1_8974	p3	(TAC)5	15	51441879	51441893
chr1_8975	p3	(CTG)6	18	51448013	51448030
chr1_8976	p2	(AC)7	14	51463219	51463232
chr1_8977	p2	(GT)15	30	51468004	51468033
chr1_8978	p4	(AGAC)6	24	51468159	51468182
chr1_8979	p2	(CA)7	14	51474001	51474014
chr1_8980	p3	(AGT)5	15	51476169	51476183
chr1_8981	p3	(GAT)5	15	51477797	51477811
chr1_8982	p2	(AT)8	16	51478298	51478313
chr1_8983	p2	(CA)7	14	51480764	51480777
chr1_8984	p2	(TG)13	26	51481386	51481411
chr1_8985	p4	(GAAA)6	24	51482858	51482881
chr1_8986	p3	(TGC)7	21	51490187	51490207
chr1_8987	p2	(AG)8	16	51490473	51490488
chr1_8988	p3	(CTT)5	15	51497564	51497578
chr1_8989	p3	(ATT)6	18	51508539	51508556
chr1_8990	p2	(TA)7	14	51509993	51510006
chr1_8991	p2	(AC)19	38	51514176	51514213
chr1_8992	p4	(TATC)5	20	51520360	51520379

chr1_8993	c	(TA)7(TG)10	34	51526955	51526988
chr1_8994	p2	(CA)14	28	51527805	51527832
chr1_8995	p2	(AC)6	12	51543924	51543935
chr1_8996	p2	(GT)9	18	51546456	51546473
chr1_8997	p2	(AT)13	26	51546989	51547014
chr1_8998	p2	(CA)14	28	51555547	51555574
chr1_8999	p2	(TC)7	14	51558187	51558200
chr1_9000	p2	(TG)10	20	51564298	51564317
chr1_9001	p2	(TG)8	16	51564546	51564561
chr1_9002	p2	(GT)19	38	51576795	51576832
chr1_9003	p2	(AT)7	14	51579669	51579682
chr1_9004	p4	(GCAC)5	20	51604671	51604690
chr1_9005	p2	(AC)17	34	51618453	51618486
chr1_9006	p3	(TTG)5	15	51645718	51645732
chr1_9007	p2	(TG)6	12	51652349	51652360
chr1_9008	p2	(GT)17	34	51658695	51658728
chr1_9009	p2	(GT)6	12	51659003	51659014
chr1_9010	p2	(TA)8	16	51662355	51662370
chr1_9011	p2	(CT)7	14	51663475	51663488
chr1_9012	c	(AAC)8attaataataata(ATT)6	55	51663923	51663977
chr1_9013	p2	(TA)10	20	51668846	51668865
chr1_9014	p4	(TGAC)6	24	51673152	51673175
chr1_9015	p2	(CT)6	12	51676282	51676293
chr1_9016	c	(AG)7(TG)10	34	51678239	51678272
chr1_9017	p2	(TG)9	18	51681259	51681276
chr1_9018	p3	(TAT)9	27	51693977	51694003
chr1_9019	p2	(AC)20	40	51694904	51694943
chr1_9020	p3	(ATA)5	15	51695711	51695725
chr1_9021	p2	(AC)11	22	51699179	51699200
chr1_9022	p2	(AC)6	12	51709481	51709492
chr1_9023	p2	(AC)9	18	51710580	51710597
chr1_9024	p2	(TG)16	32	51721786	51721817
chr1_9025	p3	(AAC)5	15	51731729	51731743
chr1_9026	p3	(TGT)5	15	51732255	51732269
chr1_9027	p2	(TC)14	28	51732522	51732549
chr1_9028	p2	(CA)6	12	51732801	51732812
chr1_9029	p2	(TG)6	12	51735369	51735380
chr1_9030	p4	(TGAT)6	24	51748642	51748665
chr1_9031	c	(GT)12(GA)8	40	51763497	51763536
chr1_9032	p3	(ATT)7	21	51779614	51779634
chr1_9033	p2	(TG)14	28	51780103	51780130
chr1_9034	p5	(CTATA)7	35	51786215	51786249
chr1_9035	p4	(ATGG)5	20	51812698	51812717
chr1_9036	c	(CA)18(GA)6	48	51814031	51814078
chr1_9037	p3	(GGA)5	15	51818019	51818033
chr1_9038	p2	(TC)6	12	51819957	51819968
chr1_9039	p3	(TCC)5	15	51820974	51820988
chr1_9040	p2	(TA)8	16	51824928	51824943
chr1_9041	p2	(AC)7	14	51826342	51826355
chr1_9042	p2	(CA)16	32	51840606	51840637
chr1_9043	p2	(TA)18	36	51841186	51841221
chr1_9044	p2	(AG)6	12	51851436	51851447

chr1_9045	c	(TA)10(CA)15	50	51856299	51856348
chr1_9046	p4	(AAAC)5	20	51860487	51860506
chr1_9047	p2	(AC)7	14	51863966	51863979
chr1_9048	c	(ATCCTC)5atcg(TCA)5	49	51868547	51868595
chr1_9049	p2	(TC)8	16	51872164	51872179
chr1_9050	c	(ATC)6gtcatcgtcg(TCA)5tcgtcatcatcgtcgtca(TCG)6	79	51872826	51872904
chr1_9051	p3	(TCA)9	27	51873047	51873073
chr1_9052	p2	(GT)13	26	51876522	51876547
chr1_9053	p2	(AG)12	24	51884648	51884671
chr1_9054	p2	(GT)13	26	51893781	51893806
chr1_9055	c	(AC)11(AG)7	36	51924482	51924517
chr1_9056	p2	(CA)12	24	51926037	51926060
chr1_9057	c	(TG)8tatatgtatatg(TA)7	42	51958671	51958712
chr1_9058	c	(CT)8(CA)17	50	51959631	51959680
chr1_9059	c	(TCT)5gttcgcgtcgtcttcttcttctg(TCT)5	53	51963128	51963180
chr1_9060	p2	(GT)6	12	51976118	51976129
chr1_9061	p2	(TG)21	42	51989870	51989911
chr1_9062	p2	(GT)11	22	52002175	52002196
chr1_9063	p3	(TGC)5	15	52003175	52003189
chr1_9064	p3	(TTG)6	18	52025000	52025017
chr1_9065	p2	(AC)22	44	52028047	52028090
chr1_9066	p2	(TG)12	24	52029644	52029667
chr1_9067	c	(TG)10(AG)14	48	52032292	52032339
chr1_9068	p2	(AC)10	20	52035199	52035218
chr1_9069	p3	(CAT)5	15	52051555	52051569
chr1_9070	p3	(AAT)5	15	52052677	52052691
chr1_9071	p2	(AG)6	12	52067462	52067473
chr1_9072	p2	(AG)6	12	52067616	52067627
chr1_9073	p2	(AG)8	16	52067981	52067996
chr1_9074	p2	(TG)8	16	52068370	52068385
chr1_9075	p3	(ATT)5	15	52070029	52070043
chr1_9076	p2	(GA)7	14	52093130	52093143
chr1_9077	p2	(GT)7	14	52098143	52098156
chr1_9078	p2	(AC)9	18	52104267	52104284
chr1_9079	p2	(GA)7	14	52125873	52125886
chr1_9080	p2	(GT)8	16	52126523	52126538
chr1_9081	p2	(AT)11	22	52128136	52128157
chr1_9082	p2	(TG)11	22	52135300	52135321
chr1_9083	p2	(TG)6	12	52141736	52141747
chr1_9084	p2	(AC)30	60	52144240	52144299
chr1_9085	p2	(AC)17	34	52146071	52146104
chr1_9086	p2	(TC)8	16	52149584	52149599
chr1_9087	p2	(GT)8	16	52151690	52151705
chr1_9088	p2	(CA)10	20	52151827	52151846
chr1_9089	p2	(TG)8	16	52164685	52164700
chr1_9090	p2	(TC)6	12	52167206	52167217
chr1_9091	p2	(AG)6	12	52168714	52168725
chr1_9092	p4	(CAAA)5	20	52170536	52170555
chr1_9093	p2	(GT)6	12	52171290	52171301
chr1_9094	p4	(ACAG)6	24	52181942	52181965
chr1_9095	p5	(AAGTC)5	25	52184290	52184314
chr1_9096	p2	(TG)6	12	52185347	52185358

chr1_9097	p3	(GTT)5	15	52189940	52189954
chr1_9098	p3	(ATT)7	21	52202415	52202435
chr1_9099	p2	(AC)20	40	52205291	52205330
chr1_9100	p2	(TG)9	18	52207168	52207185
chr1_9101	c	(TG)9(AG)9aa(AG)7	52	52207429	52207480
chr1_9102	p2	(TG)6	12	52212247	52212258
chr1_9103	p2	(TG)6	12	52212907	52212918
chr1_9104	p2	(CT)14	28	52214860	52214887
chr1_9105	p2	(AC)28	56	52216797	52216852
chr1_9106	p2	(TG)13	26	52217550	52217575
chr1_9107	p2	(GT)8	16	52222696	52222711
chr1_9108	p2	(CA)6	12	52222834	52222845
chr1_9109	p2	(GT)14	28	52227401	52227428
chr1_9110	p3	(CAA)5	15	52230022	52230036
chr1_9111	c	(GT)7a(TG)6	27	52247946	52247972
chr1_9112	p2	(TG)8	16	52252948	52252963
chr1_9113	p2	(CA)6	12	52256845	52256856
chr1_9114	c	)5ctctctttctgtgtgtcaccctatctccacttctattgaaatcagggagaat	87	52260110	52260196
chr1_9115	p2	(TG)15	30	52269653	52269682
chr1_9116	c	tgattcatcgagttctcagtgccaagttgtcagtgtcattcaatcctcttatag	123	52269809	52269931
chr1_9117	p2	(CA)6	12	52273685	52273696
chr1_9118	p2	(AC)6	12	52310410	52310421
chr1_9119	p2	(AC)10	20	52317480	52317499
chr1_9120	c	(AC)6agaagaaagaaaggaa(AG)9	46	52317609	52317654
chr1_9121	p2	(CA)6	12	52333310	52333321
chr1_9122	p3	(GGT)6	18	52334324	52334341
chr1_9123	p3	(TGA)5	15	52338813	52338827
chr1_9124	p2	(GT)8	16	52349150	52349165
chr1_9125	c	(TG)6tctctg(TC)6	30	52353987	52354016
chr1_9126	p2	(TG)8	16	52354491	52354506
chr1_9127	c	(CA)17aaaaatttagtgtgtatt(TG)23	98	52355480	52355577
chr1_9128	c	(GT)8ggtggtggtgggg(TA)6	41	52356679	52356719
chr1_9129	p2	(TG)6	12	52360641	52360652
chr1_9130	p3	(ATA)6	18	52370199	52370216
chr1_9131	p2	(GT)20	40	52374495	52374534
chr1_9132	p2	(TG)17	34	52387797	52387830
chr1_9133	p2	(GT)35	70	52403960	52404029
chr1_9134	p2	(GT)6	12	52427791	52427802
chr1_9135	p2	(AC)6	12	52427920	52427931
chr1_9136	p2	(TG)12	24	52431249	52431272
chr1_9137	p2	(CA)7	14	52431466	52431479
chr1_9138	p2	(TG)7	14	52432829	52432842
chr1_9139	c	agttattgagcttctggaacttctgaaagaggaactgcgactggcgatggca	151	52433582	52433732
chr1_9140	c	(TG)8tt(TG)6	30	52433837	52433866
chr1_9141	p2	(TG)22	44	52439158	52439201
chr1_9142	p2	(TG)6	12	52451653	52451664
chr1_9143	p2	(AC)11	22	52457063	52457084
chr1_9144	p2	(TA)8	16	52457685	52457700
chr1_9145	c	29taccgcagatgatcagccatgtgtgagatgcctaattagtgtacatgta(	119	52467055	52467173
chr1_9146	p2	(GT)15	30	52474538	52474567
chr1_9147	p4	(AATG)5	20	52491900	52491919
chr1_9148	c	(CT)6atatatatacatatctttg(TTC)5	46	52493534	52493579

chr1_9149	p2	(CA)6	12	52496178	52496189
chr1_9150	p3	(TCT)5	15	52501295	52501309
chr1_9151	p2	(TC)7	14	52504565	52504578
chr1_9152	p2	(GT)16	32	52510357	52510388
chr1_9153	p2	(GT)6	12	52511134	52511145
chr1_9154	p2	(TC)8	16	52517197	52517212
chr1_9155	p3	(ATT)7	21	52519653	52519673
chr1_9156	p2	(AC)18	36	52523945	52523980
chr1_9157	p2	(TC)10	20	52524859	52524878
chr1_9158	p2	(GA)9	18	52546233	52546250
chr1_9159	c	(CT)8(CA)6	28	52548217	52548244
chr1_9160	p2	(AC)23	46	52548639	52548684
chr1_9161	p2	(GT)6	12	52552924	52552935
chr1_9162	p2	(TG)22	44	52555620	52555663
chr1_9163	p4	(TCTT)6	24	52558769	52558792
chr1_9164	p3	(AAT)5	15	52566896	52566910
chr1_9165	p4	(GATT)26	104	52573266	52573369
chr1_9166	c	(CT)28t(TC)7(AC)6	83	52577044	52577126
chr1_9167	p2	(AC)15	30	52582622	52582651
chr1_9168	p4	(CCAT)5	20	52585573	52585592
chr1_9169	p2	(AC)8	16	52589212	52589227
chr1_9170	p2	(AC)14	28	52593034	52593061
chr1_9171	p2	(AC)8	16	52593269	52593284
chr1_9172	p2	(AC)26	52	52596165	52596216
chr1_9173	p2	(CT)7	14	52600565	52600578
chr1_9174	p2	(CT)6	12	52600726	52600737
chr1_9175	p2	(TG)8	16	52601490	52601505
chr1_9176	c	;ggagcttttgttttcagattgcagtattccagactgtgctaatgtgttggcatg	134	52604539	52604672
chr1_9177	p2	(TG)8	16	52604782	52604797
chr1_9178	p2	(AC)6	12	52604948	52604959
chr1_9179	p2	(TG)19	38	52605563	52605600
chr1_9180	p2	(AC)11	22	52610806	52610827
chr1_9181	p2	(TG)6	12	52617948	52617959
chr1_9182	p2	(TG)6	12	52624654	52624665
chr1_9183	p2	(CA)14	28	52625054	52625081
chr1_9184	p3	(TAC)9	27	52626887	52626913
chr1_9185	p2	(AC)24	48	52627158	52627205
chr1_9186	p2	(CA)6	12	52627357	52627368
chr1_9187	p2	(TG)18	36	52627791	52627826
chr1_9188	p3	(TAC)9	27	52629403	52629429
chr1_9189	p2	(AC)25	50	52629674	52629723
chr1_9190	p3	(TTA)6	18	52630657	52630674
chr1_9191	c	(CA)14g(AC)7	43	52631732	52631774
chr1_9192	p2	(AC)6	12	52637506	52637517
chr1_9193	p2	(GT)17	34	52640523	52640556
chr1_9194	p2	(TC)6	12	52641102	52641113
chr1_9195	c	(TG)6agagt(GA)8	33	52649030	52649062
chr1_9196	p2	(TG)15	30	52657041	52657070
chr1_9197	p2	(GT)19	38	52658490	52658527
chr1_9198	p2	(GT)8	16	52660557	52660572
chr1_9199	p2	(TC)7	14	52663989	52664002
chr1_9200	c	tgcttcagtcacaccctacctcccattgccccaagccatctgtcaagtacat;	135	52666978	52667112

chr1_9201	p5	(AGAAA)5	25	52667873	52667897
chr1_9202	p2	(AC)6	12	52672234	52672245
chr1_9203	c	(TG)17g(GT)18	71	52672499	52672569
chr1_9204	p2	(TG)25	50	52674593	52674642
chr1_9205	p4	(ATTC)5	20	52676082	52676101
chr1_9206	p2	(AC)8	16	52684460	52684475
chr1_9207	p2	(TG)11	22	52695282	52695303
chr1_9208	p2	(TC)18	36	52718737	52718772
chr1_9209	p2	(CA)7	14	52730440	52730453
chr1_9210	p2	(TA)7	14	52732994	52733007
chr1_9211	p2	(CA)12	24	52747965	52747988
chr1_9212	p2	(GT)14	28	52748407	52748434
chr1_9213	p2	(GT)15	30	52748733	52748762
chr1_9214	p2	(AC)10	20	52750718	52750737
chr1_9215	c	(CT)7(CA)14	42	52750915	52750956
chr1_9216	p2	(TC)10	20	52754234	52754253
chr1_9217	p2	(GT)6	12	52762762	52762773
chr1_9218	p2	(TA)6	12	52768202	52768213
chr1_9219	p2	(AG)6	12	52771341	52771352
chr1_9220	p5	(TTAGA)8	40	52771960	52771999
chr1_9221	p2	(AC)6	12	52773997	52774008
chr1_9222	p2	(AT)10	20	52777252	52777271
chr1_9223	p2	(TC)6	12	52783814	52783825
chr1_9224	p5	(GATTT)5	25	52794536	52794560
chr1_9225	p2	(TC)8	16	52799918	52799933
chr1_9226	p2	(AC)6	12	52802285	52802296
chr1_9227	p2	(CT)8	16	52812182	52812197
chr1_9228	p2	(GA)9	18	52826549	52826566
chr1_9229	p2	(CA)6	12	52835197	52835208
chr1_9230	p2	(TG)6	12	52837405	52837416
chr1_9231	p3	(TAT)6	18	52840511	52840528
chr1_9232	p2	(AC)26	52	52841003	52841054
chr1_9233	c	(TC)6cctcaccttc(TG)10(AG)13	68	52843725	52843792
chr1_9234	c	gaggcccatttagtgtgatctgctctcacctaacagcacatctttcactctcttt	146	52849926	52850071
chr1_9235	p2	(AC)23	46	52858645	52858690
chr1_9236	c	(TC)14acacacat(AC)8	52	52862909	52862960
chr1_9237	p2	(AC)11	22	52863099	52863120
chr1_9238	p2	(TA)12	24	52865743	52865766
chr1_9239	p2	(AC)16	32	52866184	52866215
chr1_9240	c	(TG)9tc(TG)21	62	52872652	52872713
chr1_9241	p2	(CA)6	12	52872874	52872885
chr1_9242	p2	(GT)23	46	52876256	52876301
chr1_9243	c	(GT)14gc(GT)12	54	52876616	52876669
chr1_9244	p2	(TC)7	14	52890930	52890943
chr1_9245	p3	(CAT)5	15	52896488	52896502
chr1_9246	p2	(GT)10	20	52901913	52901932
chr1_9247	p2	(AC)16	32	52903508	52903539
chr1_9248	p2	(TA)9	18	52907280	52907297
chr1_9249	p2	(AT)9	18	52917011	52917028
chr1_9250	p3	(TCT)5	15	52917852	52917866
chr1_9251	c	(CA)12cgcacg(CA)13	56	52925458	52925513
chr1_9252	p3	(ATT)5	15	52926044	52926058

chr1_9253	p3	(AAC)5	15	52950197	52950211
chr1_9254	p2	(GT)9	18	52959387	52959404
chr1_9255	p3	(TCC)5	15	52981030	52981044
chr1_9256	p5	(ACTCT)5	25	52983073	52983097
chr1_9257	p2	(AC)7	14	52987411	52987424
chr1_9258	p3	(ATA)5	15	52993700	52993714
chr1_9259	p2	(AC)13	26	52994294	52994319
chr1_9260	p4	(AAAT)7	28	52996533	52996560
chr1_9261	p2	(CT)7	14	53004687	53004700
chr1_9262	p3	(TTA)5	15	53006581	53006595
chr1_9263	p2	(TG)17	34	53041907	53041940
chr1_9264	c	(ATT)5aatagtaa(TAT)9	50	53042490	53042539
chr1_9265	p2	(TG)10	20	53045995	53046014
chr1_9266	p2	(GA)6	12	53046717	53046728
chr1_9267	p2	(AG)6	12	53060403	53060414
chr1_9268	p3	(GAT)5	15	53070635	53070649
chr1_9269	p2	(CA)13	26	53086435	53086460
chr1_9270	c	aactggttcagccagtgtagtccttgttgccacacccgccgaactacaaac	123	53087167	53087289
chr1_9271	p2	(GA)6	12	53091077	53091088
chr1_9272	c	(CA)8aacacacagatgtctccatattgacacctgaca(TC)6	61	53093432	53093492
chr1_9273	p2	(GT)6	12	53097811	53097822
chr1_9274	p2	(AT)10	20	53103054	53103073
chr1_9275	p3	(TTA)6	18	53129032	53129049
chr1_9276	p2	(TG)8	16	53131074	53131089
chr1_9277	p2	(TC)7	14	53135611	53135624
chr1_9278	p3	(ATG)6	18	53145276	53145293
chr1_9279	p2	(TA)11	22	53147671	53147692
chr1_9280	p2	(TC)19	38	53152127	53152164
chr1_9281	p4	(ATTT)6	24	53156843	53156866
chr1_9282	p2	(TA)6	12	53163909	53163920
chr1_9283	p2	(CT)12	24	53175837	53175860
chr1_9284	p4	(TCAA)5	20	53176988	53177007
chr1_9285	p2	(CA)9	18	53193315	53193332
chr1_9286	p2	(AT)8	16	53196684	53196699
chr1_9287	p3	(ATA)5	15	53203367	53203381
chr1_9288	p2	(AC)10	20	53207542	53207561
chr1_9289	p2	(AC)11	22	53211193	53211214
chr1_9290	p2	(GT)18	36	53226352	53226387
chr1_9291	p2	(CA)6	12	53237428	53237439
chr1_9292	p3	(AGC)6	18	53241815	53241832
chr1_9293	p3	(ACT)5	15	53242208	53242222
chr1_9294	p2	(TG)7	14	53242800	53242813
chr1_9295	c	(AC)7at(AC)18	52	53244507	53244558
chr1_9296	p2	(AC)6	12	53252168	53252179
chr1_9297	p2	(TG)54	108	53255836	53255943
chr1_9298	p3	(TTG)5	15	53261449	53261463
chr1_9299	p2	(CA)7	14	53266223	53266236
chr1_9300	p2	(GA)8	16	53284742	53284757
chr1_9301	p2	(TC)8	16	53286147	53286162
chr1_9302	p2	(TG)7	14	53287767	53287780
chr1_9303	p2	(AG)7	14	53288152	53288165
chr1_9304	p2	(CT)9	18	53320386	53320403

chr1_9305	p2	(AC)21	42	53327832	53327873
chr1_9306	p2	(AC)8	16	53354262	53354277
chr1_9307	p2	(AC)6	12	53366472	53366483
chr1_9308	p2	(TG)8	16	53367650	53367665
chr1_9309	p2	(CA)8	16	53368108	53368123
chr1_9310	p2	(AC)7	14	53372366	53372379
chr1_9311	p2	(TC)10	20	53376690	53376709
chr1_9312	c	(AC)11ccgtgactatgacg(AC)6	48	53376880	53376927
chr1_9313	p2	(AT)8	16	53380611	53380626
chr1_9314	p2	(TG)13	26	53381005	53381030
chr1_9315	p2	(CA)6	12	53387451	53387462
chr1_9316	p2	(AC)10	20	53390437	53390456
chr1_9317	c	ggatgtggatgtaaagtgtaaaggaatgtaaagtgacaaaaacaaggatga	85	53410319	53410403
chr1_9318	p2	(CA)9	18	53410915	53410932
chr1_9319	p2	(AC)6	12	53430424	53430435
chr1_9320	p2	(GT)10	20	53434447	53434466
chr1_9321	p4	(AATC)6	24	53437920	53437943
chr1_9322	p2	(AT)6	12	53441836	53441847
chr1_9323	p2	(TG)14	28	53445110	53445137
chr1_9324	p2	(TC)6	12	53445289	53445300
chr1_9325	p2	(TG)6	12	53446987	53446998
chr1_9326	p2	(TA)9	18	53459804	53459821
chr1_9327	p2	(TG)6	12	53465783	53465794
chr1_9328	p2	(TG)15	30	53472139	53472168
chr1_9329	p2	(GT)9	18	53476586	53476603
chr1_9330	p2	(TG)23	46	53477102	53477147
chr1_9331	p3	(ATC)6	18	53483765	53483782
chr1_9332	p2	(GA)6	12	53493993	53494004
chr1_9333	p3	(TTA)6	18	53518422	53518439
chr1_9334	p2	(TC)7	14	53525106	53525119
chr1_9335	c	(AG)6(GT)14	40	53525247	53525286
chr1_9336	p2	(CA)18	36	53534380	53534415
chr1_9337	p2	(CA)10	20	53536472	53536491
chr1_9338	p2	(CA)6	12	53538193	53538204
chr1_9339	c	aggatccctttac(TG)17gcacacattagctgtgatgggtttggcagttc	247	53551901	53552147
chr1_9340	p2	(GT)28	56	53555545	53555600
chr1_9341	p2	(AC)8	16	53559038	53559053
chr1_9342	p2	(TA)7	14	53566990	53567003
chr1_9343	c	(GC)6(GT)8	28	53570246	53570273
chr1_9344	p2	(GT)7	14	53570546	53570559
chr1_9345	p2	(TG)7	14	53571708	53571721
chr1_9346	p2	(TG)19	38	53571994	53572031
chr1_9347	p2	(CT)6	12	53577751	53577762
chr1_9348	p2	(AC)6	12	53587991	53588002
chr1_9349	c	(GT)8(AT)7	30	53592611	53592640
chr1_9350	p2	(TG)19	38	53601831	53601868
chr1_9351	p2	(CA)8	16	53601994	53602009
chr1_9352	p2	(CA)6	12	53605231	53605242
chr1_9353	p2	(TG)21	42	53620802	53620843
chr1_9354	p2	(AC)9	18	53620974	53620991
chr1_9355	p2	(AC)11	22	53624209	53624230
chr1_9356	p2	(AG)8	16	53626318	53626333

chr1_9357	c	(AC)26atac(AT)6(AC)7	82	53629743	53629824
chr1_9358	p2	(AC)10	20	53630117	53630136
chr1_9359	p2	(AC)7	14	53637616	53637629
chr1_9360	p2	(AC)11	22	53638574	53638595
chr1_9361	p2	(AC)6	12	53639634	53639645
chr1_9362	p3	(AGG)5	15	53641672	53641686
chr1_9363	p2	(AG)6	12	53648949	53648960
chr1_9364	p2	(AG)6	12	53649358	53649369
chr1_9365	p2	(TG)6	12	53654717	53654728
chr1_9366	p2	(AC)6	12	53660483	53660494
chr1_9367	p2	(AG)9	18	53674674	53674691
chr1_9368	p2	(CT)7	14	53677231	53677244
chr1_9369	p5	(AAATC)5	25	53682453	53682477
chr1_9370	p2	(AG)6	12	53690612	53690623
chr1_9371	p2	(TG)16	32	53712254	53712285
chr1_9372	p3	(AAT)5	15	53718428	53718442
chr1_9373	c	(GT)6gagtgtgagtgtgagt(GA)8	44	53741283	53741326
chr1_9374	p2	(AC)8	16	53743011	53743026
chr1_9375	p2	(TG)6	12	53747337	53747348
chr1_9376	p4	(TTTG)6	24	53750869	53750892
chr1_9377	p2	(TC)10	20	53760827	53760846
chr1_9378	p3	(TCA)5	15	53762899	53762913
chr1_9379	p2	(TA)6	12	53769069	53769080
chr1_9380	p4	(ATCT)10	40	53772705	53772744
chr1_9381	p4	(GATG)5	20	53779796	53779815
chr1_9382	p2	(AC)11	22	53781829	53781850
chr1_9383	c	tatatacagatagatagatagatagacagacagacaaatagacagatagat	120	53783820	53783939
chr1_9384	p2	(TC)7	14	53784511	53784524
chr1_9385	p2	(CA)7	14	53786535	53786548
chr1_9386	c*	(AGG)5(GA)8*	30	53799384	53799413
chr1_9387	p3	(AGT)5	15	53806519	53806533
chr1_9388	p4	(TACA)7	28	53809410	53809437
chr1_9389	p4	(CACT)5	20	53815757	53815776
chr1_9390	p2	(AC)15	30	53822000	53822029
chr1_9391	p2	(TG)11	22	53826355	53826376
chr1_9392	p2	(AC)11	22	53829229	53829250
chr1_9393	p2	(AT)11	22	53849374	53849395
chr1_9394	p2	(GT)17	34	53860063	53860096
chr1_9395	p2	(TG)7	14	53868195	53868208
chr1_9396	p2	(TG)7	14	53875099	53875112
chr1_9397	p3	(CCA)7	21	53883048	53883068
chr1_9398	p2	(TG)18	36	53906758	53906793
chr1_9399	p2	(GA)7	14	53907777	53907790
chr1_9400	p2	(CA)19	38	53918207	53918244
chr1_9401	p2	(AG)18	36	53918394	53918429
chr1_9402	p2	(TA)6	12	53924340	53924351
chr1_9403	p3	(TTG)5	15	53927499	53927513
chr1_9404	p4	(TCTA)5	20	53928064	53928083
chr1_9405	p2	(AC)18	36	53940794	53940829
chr1_9406	p4	(TACC)6	24	53949914	53949937
chr1_9407	p2	(AC)21	42	53952271	53952312
chr1_9408	p2	(AC)6	12	53978762	53978773

chr1_9409	p3	(AAT)5	15	53987007	53987021
chr1_9410	p4	(CATT)5	20	53987560	53987579
chr1_9411	p2	(CA)6	12	53991971	53991982
chr1_9412	p3	(ATT)6	18	54000281	54000298
chr1_9413	p2	(AT)7	14	54001627	54001640
chr1_9414	p2	(AT)8	16	54016718	54016733
chr1_9415	p3	(TAT)5	15	54035890	54035904
chr1_9416	p2	(AG)14	28	54040717	54040744
chr1_9417	p3	(TAT)5	15	54046723	54046737
chr1_9418	p2	(CA)18	36	54050821	54050856
chr1_9419	p3	(CTT)5	15	54065604	54065618
chr1_9420	p2	(CA)8	16	54066743	54066758
chr1_9421	p3	(TAT)5	15	54068840	54068854
chr1_9422	p2	(GT)10	20	54080707	54080726
chr1_9423	p2	(TC)7	14	54081402	54081415
chr1_9424	p2	(TG)12	24	54082750	54082773
chr1_9425	c	(TC)7(AC)7	28	54083512	54083539
chr1_9426	p2	(CT)7	14	54096223	54096236
chr1_9427	p2	(TG)6	12	54101519	54101530
chr1_9428	p4	(CACT)6	24	54103461	54103484
chr1_9429	p2	(CA)9	18	54135866	54135883
chr1_9430	p3	(AAC)5	15	54137562	54137576
chr1_9431	p2	(AC)24	48	54139397	54139444
chr1_9432	p2	(AC)6	12	54140343	54140354
chr1_9433	p2	(GT)14	28	54140724	54140751
chr1_9434	p2	(TC)22	44	54146065	54146108
chr1_9435	p5	(TTTGA)5	25	54157070	54157094
chr1_9436	p2	(GT)6	12	54162977	54162988
chr1_9437	p2	(AC)18	36	54171034	54171069
chr1_9438	p2	(TG)7	14	54177507	54177520
chr1_9439	p2	(AG)7	14	54191898	54191911
chr1_9440	p2	(AC)12	24	54220627	54220650
chr1_9441	p2	(GT)13	26	54233019	54233044
chr1_9442	p2	(AG)10	20	54267124	54267143
chr1_9443	p2	(TG)8	16	54282257	54282272
chr1_9444	p2	(CT)17	34	54295153	54295186
chr1_9445	p2	(AC)14	28	54299149	54299176
chr1_9446	p2	(TG)13	26	54305352	54305377
chr1_9447	p2	(TG)10	20	54311140	54311159
chr1_9448	p2	(TG)8	16	54311626	54311641
chr1_9449	p3	(TCA)5	15	54312088	54312102
chr1_9450	p2	(GT)10	20	54312238	54312257
chr1_9451	p3	(ATT)6	18	54312940	54312957
chr1_9452	p2	(AT)6	12	54319077	54319088
chr1_9453	p2	(TA)7	14	54329353	54329366
chr1_9454	p2	(TG)22	44	54329486	54329529
chr1_9455	c	tagttctaggttcttgtttgatggttaaaatcaaaacttcgaggacaaacttttc	140	54332134	54332273
chr1_9456	p2	(GT)7	14	54332750	54332763
chr1_9457	p2	(TA)6	12	54335854	54335865
chr1_9458	p3	(GAA)5	15	54340129	54340143
chr1_9459	p3	(TAA)6	18	54347765	54347782
chr1_9460	p2	(AC)7	14	54348452	54348465

chr1_9461	p2	(CA)6	12	54350847	54350858
chr1_9462	p2	(GT)19	38	54373900	54373937
chr1_9463	p2	(TG)7	14	54401074	54401087
chr1_9464	p2	(GA)7	14	54401198	54401211
chr1_9465	p2	(TG)9	18	54403076	54403093
chr1_9466	p2	(AT)9	18	54406372	54406389
chr1_9467	p2	(GT)6	12	54410313	54410324
chr1_9468	c	(GT)8atggtgtgggggggcgggaaccagacac(AT)7	59	54417081	54417139
chr1_9469	p2	(AC)6	12	54419972	54419983
chr1_9470	p2	(CA)6	12	54420237	54420248
chr1_9471	p2	(TG)8	16	54420811	54420826
chr1_9472	p2	(CA)7	14	54424231	54424244
chr1_9473	p2	(AT)6	12	54429626	54429637
chr1_9474	p2	(CA)6	12	54455786	54455797
chr1_9475	p2	(CA)6	12	54456023	54456034
chr1_9476	p2	(TG)6	12	54461101	54461112
chr1_9477	p3	(AAG)33	99	54477142	54477240
chr1_9478	p2	(CA)6	12	54482538	54482549
chr1_9479	p3	(ATT)6	18	54483934	54483951
chr1_9480	p2	(TG)11	22	54494440	54494461
chr1_9481	c	(TAA)8tta(ATT)5	42	54497504	54497545
chr1_9482	p2	(TG)9	18	54499036	54499053
chr1_9483	p2	(TA)10	20	54502009	54502028
chr1_9484	c	(TA)14tgtatgta(TG)10	56	54503515	54503570
chr1_9485	c	[illegible]aactttctataaatctttattcagatttgcacataaaccttaccacacagtttaa	120	54508197	54508316
chr1_9486	p2	(AC)7	14	54516729	54516742
chr1_9487	p2	(CA)8	16	54518682	54518697
chr1_9488	p3	(AAT)5	15	54527390	54527404
chr1_9489	p2	(TG)19	38	54533531	54533568
chr1_9490	p2	(AC)16	32	54535920	54535951
chr1_9491	p3	(TCT)5	15	54537315	54537329
chr1_9492	p3	(AAT)7	21	54540338	54540358
chr1_9493	p2	(CA)6	12	54547677	54547688
chr1_9494	p2	(AC)6	12	54550783	54550794
chr1_9495	p2	(AG)6	12	54573508	54573519
chr1_9496	p2	(AC)6	12	54588186	54588197
chr1_9497	p2	(CA)8	16	54588711	54588726
chr1_9498	p2	(TA)11	22	54594815	54594836
chr1_9499	p2	(GA)7	14	54596297	54596310
chr1_9500	p2	(CT)6	12	54597729	54597740
chr1_9501	p2	(CA)6	12	54610288	54610299
chr1_9502	p2	(CA)21	42	54611529	54611570
chr1_9503	p3	(TAA)6	18	54615571	54615588
chr1_9504	p2	(TG)6	12	54639383	54639394
chr1_9505	p2	(TA)15	30	54652820	54652849
chr1_9506	p2	(AT)6	12	54654024	54654035
chr1_9507	p2	(CA)7	14	54664012	54664025
chr1_9508	p2	(TA)7	14	54665759	54665772
chr1_9509	p2	(GT)11	22	54679436	54679457
chr1_9510	p2	(AC)15	30	54691512	54691541
chr1_9511	p4	(AATT)5	20	54692552	54692571
chr1_9512	p5	(ATATC)11	55	54702106	54702160

chr1_9513	p3	(AAT)5	15	54709106	54709120
chr1_9514	c	(ATGG)5atgttacttttaccctgctttgtatattaaaaacaat(TA)6	69	54718574	54718642
chr1_9515	p4	(TCTA)7	28	54754547	54754574
chr1_9516	c	(TC)10(TG)9	38	54757994	54758031
chr1_9517	p3	(TAA)5	15	54792301	54792315
chr1_9518	p2	(CT)12	24	54792960	54792983
chr1_9519	p2	(GA)6	12	54795759	54795770
chr1_9520	p2	(TG)6	12	54812631	54812642
chr1_9521	p2	(AC)18	36	54816483	54816518
chr1_9522	p2	(GT)16	32	54828628	54828659
chr1_9523	p3	(AAT)6	18	54832133	54832150
chr1_9524	p3	(TAT)8	24	54835908	54835931
chr1_9525	p2	(TG)6	12	54836394	54836405
chr1_9526	p4	(CATC)6	24	54838931	54838954
chr1_9527	p2	(CA)6	12	54875693	54875704
chr1_9528	p3	(ATA)6	18	54904211	54904228
chr1_9529	p3	(GGT)5	15	54904436	54904450
chr1_9530	p2	(AC)15	30	54910953	54910982
chr1_9531	p3	(TCT)6	18	54931689	54931706
chr1_9532	p2	(AT)6	12	54942387	54942398
chr1_9533	p2	(TG)10	20	54953149	54953168
chr1_9534	p3	(CTT)5	15	54967549	54967563
chr1_9535	p2	(TC)6	12	55004651	55004662
chr1_9536	p2	(GA)6	12	55006381	55006392
chr1_9537	p2	(TG)13	26	55007755	55007780
chr1_9538	p2	(GA)14	28	55009924	55009951
chr1_9539	p2	(TA)6	12	55010784	55010795
chr1_9540	p2	(CA)12	24	55020996	55021019
chr1_9541	p2	(CT)6	12	55027417	55027428
chr1_9542	p3	(TCA)6	18	55034453	55034470
chr1_9543	p3	(CAT)7	21	55037720	55037740
chr1_9544	p3	(TCC)7	21	55053004	55053024
chr1_9545	p2	(TG)10	20	55058960	55058979
chr1_9546	p4	(AATG)5	20	55069120	55069139
chr1_9547	p2	(CT)12	24	55075277	55075300
chr1_9548	p2	(GT)6	12	55079755	55079766
chr1_9549	p2	(TG)6	12	55081341	55081352
chr1_9550	p2	(TG)15	30	55099181	55099210
chr1_9551	p2	(AC)6	12	55106632	55106643
chr1_9552	p2	(AC)6	12	55106768	55106779
chr1_9553	c	cttgcacagtcatactcattctgtcatacatatgacatcatagtctcacacag	110	55133559	55133668
chr1_9554	p2	(AC)16	32	55151475	55151506
chr1_9555	p2	(GT)9	18	55153205	55153222
chr1_9556	p2	(GT)12	24	55156082	55156105
chr1_9557	p2	(GT)7	14	55158154	55158167
chr1_9558	c	(TG)9a(GT)6	31	55158795	55158825
chr1_9559	p2	(GA)11	22	55159064	55159085
chr1_9560	p2	(TG)19	38	55162462	55162499
chr1_9561	p3	(TTG)6	18	55165635	55165652
chr1_9562	p4	(TCTG)5	20	55167001	55167020
chr1_9563	p2	(TG)7	14	55176590	55176603
chr1_9564	p2	(AC)6	12	55181131	55181142

chr1_9565	p2	(TG)6	12	55186794	55186805
chr1_9566	p2	(AC)6	12	55221819	55221830
chr1_9567	c	(AT)7(AC)10	34	55225798	55225831
chr1_9568	p2	(GT)10	20	55242291	55242310
chr1_9569	p2	(CA)11	22	55242474	55242495
chr1_9570	c	(AG)7gaacacagaa(AG)6aatcagca(GT)6	56	55249573	55249628
chr1_9571	c	gagacagagatatggatggaaaagtgcaggtaggaaatgagtataaggtaa	184	55249801	55249984
chr1_9572	c	22ttcatatgtagagggatgtgtgtgagggagagaggggtatcttccattc(	105	55251271	55251375
chr1_9573	p2	(TG)6	12	55253225	55253236
chr1_9574	p2	(AC)7	14	55253966	55253979
chr1_9575	p2	(TG)14	28	55254304	55254331
chr1_9576	p2	(AC)9	18	55254980	55254997
chr1_9577	p2	(TG)15	30	55256449	55256478
chr1_9578	p2	(TG)7	14	55256914	55256927
chr1_9579	c	aaagccctgcagtttgtacagctgggcctgctgcttgcgttgggtaggaacc	112	55258009	55258120
chr1_9580	p2	(CT)6	12	55264210	55264221
chr1_9581	p2	(AC)21	42	55264470	55264511
chr1_9582	p2	(TG)11	22	55265851	55265872
chr1_9583	p2	(AG)6	12	55266089	55266100
chr1_9584	p2	(AC)6	12	55266480	55266491
chr1_9585	p2	(TA)7	14	55267653	55267666
chr1_9586	p2	(TC)6	12	55268245	55268256
chr1_9587	p2	(TG)7	14	55270290	55270303
chr1_9588	p2	(CT)10	20	55273093	55273112
chr1_9589	c	(AC)16g(CA)6	45	55273287	55273331
chr1_9590	p2	(GT)13	26	55273517	55273542
chr1_9591	p2	(AC)12	24	55273771	55273794
chr1_9592	p2	(TA)6	12	55274810	55274821
chr1_9593	p3	(TGG)7	21	55276447	55276467
chr1_9594	p2	(CA)8	16	55285484	55285499
chr1_9595	p2	(TG)8	16	55286276	55286291
chr1_9596	p2	(GT)7	14	55289929	55289942
chr1_9597	p2	(AC)14	28	55290238	55290265
chr1_9598	p2	(GT)13	26	55290998	55291023
chr1_9599	p3	(GAG)5	15	55292121	55292135
chr1_9600	c	(AC)6catttctgtctcca(TC)13	52	55293872	55293923
chr1_9601	p2	(TG)8	16	55296079	55296094
chr1_9602	c	(TC)6(AC)9	30	55296773	55296802
chr1_9603	p2	(CT)6	12	55297469	55297480
chr1_9604	p2	(GT)8	16	55299103	55299118
chr1_9605	p3	(TTA)8	24	55299453	55299476
chr1_9606	p2	(GT)7	14	55300624	55300637
chr1_9607	p2	(TG)11	22	55301946	55301967
chr1_9608	p2	(CA)6	12	55304569	55304580
chr1_9609	p2	(GT)10	20	55304697	55304716
chr1_9610	p2	(AC)27	54	55306430	55306483
chr1_9611	p2	(TG)18	36	55309039	55309074
chr1_9612	p2	(GT)34	68	55310486	55310553
chr1_9613	p2	(AC)17	34	55311358	55311391
chr1_9614	p2	(AC)18	36	55314704	55314739
chr1_9615	p2	(CA)7	14	55315817	55315830
chr1_9616	p2	(TG)6	12	55318998	55319009

chr1_9617	p2	(CA)6	12	55326296	55326307
chr1_9618	p3	(GGT)5	15	55330649	55330663
chr1_9619	p2	(CA)6	12	55333535	55333546
chr1_9620	p2	(AC)12	24	55335701	55335724
chr1_9621	p2	(AC)6	12	55335891	55335902
chr1_9622	p2	(CA)14	28	55336302	55336329
chr1_9623	p3	(TGC)5	15	55337936	55337950
chr1_9624	p2	(CA)6	12	55339928	55339939
chr1_9625	p2	(GT)14	28	55342650	55342677
chr1_9626	c	(TG)13agagagagagc(GA)10	57	55342919	55342975
chr1_9627	p2	(AC)15	30	55343086	55343115
chr1_9628	p2	(CT)10	20	55343290	55343309
chr1_9629	p2	(CA)6	12	55345428	55345439
chr1_9630	p2	(AC)8	16	55345677	55345692
chr1_9631	p2	(TC)6	12	55346160	55346171
chr1_9632	p2	(AC)7	14	55347055	55347068
chr1_9633	p2	(CT)6	12	55348093	55348104
chr1_9634	p2	(GT)8	16	55348454	55348469
chr1_9635	p2	(CA)11	22	55355624	55355645
chr1_9636	p2	(AC)7	14	55356237	55356250
chr1_9637	p2	(TG)6	12	55372025	55372036
chr1_9638	p2	(TC)8	16	55374432	55374447
chr1_9639	p2	(CA)6	12	55382879	55382890
chr1_9640	p2	(AT)7	14	55388423	55388436
chr1_9641	p3	(TGA)5	15	55390776	55390790
chr1_9642	p2	(TG)6	12	55397534	55397545
chr1_9643	p4	(AAAG)5	20	55400718	55400737
chr1_9644	p4	(AATA)5	20	55400872	55400891
chr1_9645	c	(AC)6at(AC)7	28	55402145	55402172
chr1_9646	p2	(TG)16	32	55403315	55403346
chr1_9647	c	(GT)6ctgtatatgtgggtgggtgatggcttgtgcctcgagtt(TG)10	70	55403479	55403548
chr1_9648	p2	(GT)6	12	55404678	55404689
chr1_9649	p2	(GT)7	14	55405227	55405240
chr1_9650	p2	(AT)6	12	55412282	55412293
chr1_9651	p2	(GT)12	24	55413789	55413812
chr1_9652	p2	(CA)6	12	55431428	55431439
chr1_9653	p2	(TG)9	18	55433765	55433782
chr1_9654	p2	(TG)6	12	55433938	55433949
chr1_9655	p3	(AGC)7	21	55435652	55435672
chr1_9656	p2	(CT)6	12	55435850	55435861
chr1_9657	p2	(AC)7	14	55437033	55437046
chr1_9658	p2	(AG)7	14	55445465	55445478
chr1_9659	p4	(CCAT)12	48	55448333	55448380
chr1_9660	p2	(CA)6	12	55452397	55452408
chr1_9661	p2	(GT)13	26	55456967	55456992
chr1_9662	p2	(CT)6	12	55464631	55464642
chr1_9663	p2	(AG)6	12	55465827	55465838
chr1_9664	p3	(CTT)5	15	55489278	55489292
chr1_9665	p4	(TATC)8	32	55497159	55497190
chr1_9666	p3	(TAG)7	21	55505354	55505374
chr1_9667	p3	(ATT)8	24	55507155	55507178
chr1_9668	p3	(CAC)6	18	55521916	55521933

chr1_9669	p3	(TCA)5	15	55525873	55525887
chr1_9670	p3	(ATA)5	15	55557301	55557315
chr1_9671	p2	(TG)33	66	55565882	55565947
chr1_9672	p3	(ATA)6	18	55611251	55611268
chr1_9673	p2	(AC)6	12	55620461	55620472
chr1_9674	p2	(CA)7	14	55621148	55621161
chr1_9675	p4	(ATGG)5	20	55626504	55626523
chr1_9676	p2	(AG)15	30	55641160	55641189
chr1_9677	p2	(TA)6	12	55649549	55649560
chr1_9678	p5	(GTAAT)5	25	55660503	55660527
chr1_9679	p2	(GT)6	12	55660927	55660938
chr1_9680	p2	(AC)8	16	55664872	55664887
chr1_9681	c	(GT)7gcacatagtttac(TG)8	43	55674187	55674229
chr1_9682	p3	(TTA)6	18	55675404	55675421
chr1_9683	p3	(TTA)6	18	55713460	55713477
chr1_9684	p2	(AC)7	14	55720047	55720060
chr1_9685	p2	(AC)8	16	55731006	55731021
chr1_9686	c	caacaacaatcgctactgacccctcatgcatacggtaagtttaaagaatcaa	135	55737041	55737175
chr1_9687	p3	(TCA)5	15	55747540	55747554
chr1_9688	p2	(GT)7	14	55752526	55752539
chr1_9689	p2	(TG)7	14	55760536	55760549
chr1_9690	p2	(AG)18	36	55761360	55761395
chr1_9691	p3	(ATT)5	15	55762165	55762179
chr1_9692	p4	(CATC)5	20	55765465	55765484
chr1_9693	p2	(GT)16	32	55809332	55809363
chr1_9694	p2	(TG)8	16	55810935	55810950
chr1_9695	p3	(AAG)5	15	55811215	55811229
chr1_9696	p2	(TG)7	14	55811613	55811626
chr1_9697	p2	(TG)15	30	55812771	55812800
chr1_9698	p2	(TG)36	72	55827267	55827338
chr1_9699	p2	(CA)6	12	55830463	55830474
chr1_9700	p2	(GT)6	12	55831588	55831599
chr1_9701	p2	(AC)29	58	55835455	55835512
chr1_9702	p2	(TG)6	12	55842056	55842067
chr1_9703	p2	(TG)11	22	55855660	55855681
chr1_9704	p2	(TG)7	14	55858260	55858273
chr1_9705	p2	(GT)10	20	55858524	55858543
chr1_9706	p2	(TG)8	16	55858769	55858784
chr1_9707	p2	(TG)14	28	55859009	55859036
chr1_9708	p2	(TG)13	26	55859278	55859303
chr1_9709	c	tttttttctgccagttcgtgatagtgtcacaaagtgtggctctcctacacacatc	213	55874972	55875184
chr1_9710	p2	(AT)8	16	55883006	55883021
chr1_9711	c	(AC)12aa(AC)10	46	55887392	55887437
chr1_9712	p2	(CA)8	16	55898130	55898145
chr1_9713	p5	(AAATC)6	30	55902509	55902538
chr1_9714	p2	(AT)7	14	55908361	55908374
chr1_9715	p4	(ATCC)5	20	55910918	55910937
chr1_9716	p2	(TG)10	20	55915820	55915839
chr1_9717	p2	(TG)6	12	55917958	55917969
chr1_9718	p2	(AC)7	14	55932383	55932396
chr1_9719	p5	(AACTC)5	25	55934546	55934570
chr1_9720	p2	(CA)20	40	55936975	55937014

chr1_9721	p2	(TG)23	46	55945867	55945912
chr1_9722	p2	(GT)6	12	55946022	55946033
chr1_9723	p2	(AC)23	46	55946553	55946598
chr1_9724	p2	(TG)16	32	55957379	55957410
chr1_9725	p2	(AC)13	26	55958683	55958708
chr1_9726	p2	(AT)12	24	55960075	55960098
chr1_9727	p2	(AT)8	16	55960394	55960409
chr1_9728	p2	(AC)9	18	55961361	55961378
chr1_9729	p4	(ATGG)5	20	55963934	55963953
chr1_9730	p3	(ATA)6	18	55964425	55964442
chr1_9731	p5	(CTTGA)5	25	55972722	55972746
chr1_9732	p2	(CT)9	18	55973380	55973397
chr1_9733	p5	(TTTGA)5	25	55984813	55984837
chr1_9734	p2	(TG)8	16	55996798	55996813
chr1_9735	p2	(TC)6	12	56002345	56002356
chr1_9736	p2	(TG)7	14	56002546	56002559
chr1_9737	p2	(TG)7	14	56007662	56007675
chr1_9738	p2	(AC)16	32	56009433	56009464
chr1_9739	p2	(TG)10	20	56013760	56013779
chr1_9740	p2	(TG)9	18	56054564	56054581
chr1_9741	p2	(AC)7	14	56057910	56057923
chr1_9742	p2	(TG)13	26	56058132	56058157
chr1_9743	p5	(CTTGA)7	35	56065932	56065966
chr1_9744	p2	(AC)8	16	56067628	56067643
chr1_9745	p2	(CA)7	14	56077606	56077619
chr1_9746	p2	(AG)6	12	56078903	56078914
chr1_9747	p2	(TG)6	12	56081274	56081285
chr1_9748	c	ggtcatacacaaatagaagtgtgccattggtgcagcttgcctcatagccctt(	108	56084738	56084845
chr1_9749	p2	(CT)9	18	56089652	56089669
chr1_9750	p2	(AC)8	16	56092430	56092445
chr1_9751	p2	(TG)8	16	56099353	56099368
chr1_9752	p2	(CA)10	20	56107104	56107123
chr1_9753	p2	(GT)6	12	56109111	56109122
chr1_9754	p2	(AC)6	12	56110684	56110695
chr1_9755	p2	(AC)6	12	56111421	56111432
chr1_9756	p2	(CA)7	14	56111875	56111888
chr1_9757	p2	(TG)16	32	56112231	56112262
chr1_9758	p2	(TG)9	18	56132248	56132265
chr1_9759	p2	(CA)7	14	56133280	56133293
chr1_9760	p2	(CA)8	16	56134412	56134427
chr1_9761	p2	(TG)9	18	56137690	56137707
chr1_9762	p2	(GT)21	42	56142166	56142207
chr1_9763	c	(ATA)5(ATT)10	45	56143299	56143343
chr1_9764	p2	(TC)6	12	56157049	56157060
chr1_9765	p2	(CA)13	26	56157697	56157722
chr1_9766	p2	(AC)11	22	56164559	56164580
chr1_9767	p3	(GAT)7	21	56169406	56169426
chr1_9768	c	tgtggccaagagtaacctccagcttctccaggtcattagcagaagattactc	114	56171353	56171466
chr1_9769	p2	(GA)12	24	56173614	56173637
chr1_9770	c	(TC)8(TG)6tatgtatatacata(TG)14(TA)7	84	56180121	56180204
chr1_9771	p2	(CA)23	46	56182188	56182233
chr1_9772	p2	(TG)21	42	56182491	56182532

chr1_9773	p2	(GT)18	36	56188625	56188660
chr1_9774	p2	(TG)14	28	56195293	56195320
chr1_9775	p2	(GT)9	18	56214363	56214380
chr1_9776	p2	(GT)8	16	56214632	56214647
chr1_9777	p2	(TC)8	16	56215329	56215344
chr1_9778	p2	(CA)19	38	56215791	56215828
chr1_9779	p2	(GT)11	22	56220536	56220557
chr1_9780	p2	(TA)6	12	56220844	56220855
chr1_9781	p2	(AC)13	26	56225635	56225660
chr1_9782	p2	(AT)6	12	56238616	56238627
chr1_9783	p2	(AC)6	12	56239682	56239693
chr1_9784	c	(TC)16(AC)7	46	56250260	56250305
chr1_9785	p2	(AC)7	14	56253743	56253756
chr1_9786	p4	(AAAC)5	20	56256922	56256941
chr1_9787	p2	(AG)6	12	56262286	56262297
chr1_9788	c*	(GT)6(GTG)5*	25	56268711	56268735
chr1_9789	p2	(GT)20	40	56269421	56269460
chr1_9790	p2	(TG)6	12	56269810	56269821
chr1_9791	p2	(CA)16	32	56272244	56272275
chr1_9792	p2	(GA)6	12	56274553	56274564
chr1_9793	c	(GT)10gagagagagac(AG)7	45	56286675	56286719
chr1_9794	p2	(TG)14	28	56290689	56290716
chr1_9795	p2	(AC)7	14	56293761	56293774
chr1_9796	p2	(TC)6	12	56303846	56303857
chr1_9797	p4	(CAAA)7	28	56319702	56319729
chr1_9798	c	(AC)13(AG)11	48	56342313	56342360
chr1_9799	p3	(TAA)5	15	56364233	56364247
chr1_9800	p3	(ATT)11	33	56398746	56398778
chr1_9801	p2	(AC)10	20	56404963	56404982
chr1_9802	p2	(GT)7	14	56405512	56405525
chr1_9803	c	(GT)21(GC)6	54	56412920	56412973
chr1_9804	p2	(TG)27	54	56421694	56421747
chr1_9805	p3	(TTA)7	21	56423074	56423094
chr1_9806	p3	(TTG)5	15	56423364	56423378
chr1_9807	p2	(CA)14	28	56428650	56428677
chr1_9808	p2	(TG)6	12	56429344	56429355
chr1_9809	p2	(AT)13	26	56430306	56430331
chr1_9810	p3	(ACA)5	15	56439167	56439181
chr1_9811	p4	(TCCA)6	24	56448393	56448416
chr1_9812	p2	(GT)9	18	56450458	56450475
chr1_9813	p2	(TG)7	14	56451648	56451661
chr1_9814	p2	(TA)6	12	56456378	56456389
chr1_9815	p2	(CA)9	18	56462541	56462558
chr1_9816	p2	(CA)8	16	56467605	56467620
chr1_9817	p2	(CA)21	42	56471311	56471352
chr1_9818	p2	(TG)9	18	56471498	56471515
chr1_9819	p2	(GT)23	46	56473680	56473725
chr1_9820	p2	(TG)7	14	56474243	56474256
chr1_9821	p2	(TG)8	16	56507038	56507053
chr1_9822	p3	(TAT)5	15	56526170	56526184
chr1_9823	p2	(TC)18	36	56528096	56528131
chr1_9824	p2	(CA)8	16	56533857	56533872

chr1_9825	p2	(CA)6	12	56536410	56536421
chr1_9826	p2	(GT)6	12	56538837	56538848
chr1_9827	p2	(GT)11	22	56548988	56549009
chr1_9828	c	A)7tctctatggtgatgaatgcagctgcaatcctcttctattagcctc(CA	73	56554991	56555063
chr1_9829	p2	(GT)6	12	56556020	56556031
chr1_9830	p2	(CA)9	18	56556194	56556211
chr1_9831	p2	(GT)9	18	56564020	56564037
chr1_9832	c	ttctattttttgtcctccctcttgattgcaaacatgacaggctcagaatattag	131	56564224	56564354
chr1_9833	p3	(ATT)5	15	56566041	56566055
chr1_9834	c	)6cacacacgtgtgtg(TA)6cacacacgtgtgtg(TA)6cacacac(	85	56570042	56570126
chr1_9835	p2	(GT)8	16	56581573	56581588
chr1_9836	p2	(TA)9	18	56602115	56602132
chr1_9837	p4	(CCAT)5	20	56634153	56634172
chr1_9838	p3	(ATT)5	15	56643091	56643105
chr1_9839	p2	(CA)6	12	56651135	56651146
chr1_9840	p4	(CATT)5	20	56652271	56652290
chr1_9841	p5	(GTAGA)5	25	56652842	56652866
chr1_9842	p2	(CT)7	14	56658924	56658937
chr1_9843	p3	(ATA)9	27	56659210	56659236
chr1_9844	c	(CT)11(CA)7tacacagacacatgtat(AC)15	83	56692230	56692312
chr1_9845	p2	(TG)6	12	56700496	56700507
chr1_9846	p2	(GT)20	40	56700686	56700725
chr1_9847	p2	(CA)16	32	56711239	56711270
chr1_9848	p2	(AC)6	12	56714406	56714417
chr1_9849	p2	(GT)18	36	56729062	56729097
chr1_9850	p2	(AC)15	30	56740346	56740375
chr1_9851	p2	(GT)6	12	56750806	56750817
chr1_9852	c*	(AT)7(TATG)5*	31	56763126	56763156
chr1_9853	c	(TA)8cataaaagaaatatagagac(AG)6	48	56763466	56763513
chr1_9854	p2	(TG)8	16	56766429	56766444
chr1_9855	p3	(TTA)6	18	56777852	56777869
chr1_9856	p2	(AC)6	12	56788151	56788162
chr1_9857	p2	(GA)6	12	56810898	56810909
chr1_9858	p3	(TAC)6	18	56822427	56822444
chr1_9859	c	(AG)7tc(AG)6	28	56827008	56827035
chr1_9860	p2	(AC)16	32	56870620	56870651
chr1_9861	p2	(AC)6	12	56874933	56874944
chr1_9862	p2	(CA)7	14	56878922	56878935
chr1_9863	p2	(TC)6	12	56881695	56881706
chr1_9864	p2	(TG)7	14	56884974	56884987
chr1_9865	p3	(TTA)5	15	56897943	56897957
chr1_9866	p2	(GT)8	16	56910794	56910809
chr1_9867	p2	(GT)6	12	56911328	56911339
chr1_9868	p2	(GA)33	66	56925752	56925817
chr1_9869	p2	(AC)12	24	56929852	56929875
chr1_9870	p2	(AC)10	20	56931630	56931649
chr1_9871	p2	(AG)7	14	56932299	56932312
chr1_9872	p2	(CA)12	24	56937352	56937375
chr1_9873	p2	(CA)6	12	56937628	56937639
chr1_9874	p2	(AC)9	18	56937876	56937893
chr1_9875	p4	(ATAG)6	24	56940904	56940927
chr1_9876	p2	(TG)6	12	56942371	56942382

chr1_9877	p2	(GA)7	14	56947488	56947501
chr1_9878	p4	(ATGG)5	20	56954348	56954367
chr1_9879	c	(TC)11actttttctttctctctctgtcttttt(TC)6	61	56959200	56959260
chr1_9880	c	(TG)19(AG)12	62	56959898	56959959
chr1_9881	p4	(ATCT)5	20	56967883	56967902
chr1_9882	p3	(TAA)6	18	56968522	56968539
chr1_9883	p2	(AC)8	16	56972421	56972436
chr1_9884	p2	(TC)9	18	56980814	56980831
chr1_9885	p2	(TC)8	16	56997328	56997343
chr1_9886	p2	(AC)6	12	57001213	57001224
chr1_9887	c	(CA)11tatataa(AT)6(GT)18	77	57001467	57001543
chr1_9888	p2	(GT)11	22	57006595	57006616
chr1_9889	p2	(GT)9	18	57006831	57006848
chr1_9890	p2	(GA)6	12	57011733	57011744
chr1_9891	p4	(ATAC)5	20	57013256	57013275
chr1_9892	p2	(GT)6	12	57024363	57024374
chr1_9893	p2	(GT)13	26	57029826	57029851
chr1_9894	p2	(CA)12	24	57032535	57032558
chr1_9895	p2	(CA)12	24	57032818	57032841
chr1_9896	p2	(TG)15	30	57033103	57033132
chr1_9897	p2	(CT)7	14	57037362	57037375
chr1_9898	p2	(CT)6	12	57045268	57045279
chr1_9899	p3	(AAG)6	18	57050019	57050036
chr1_9900	p2	(GA)7	14	57056851	57056864
chr1_9901	p4	(TGAT)5	20	57057191	57057210
chr1_9902	p2	(AG)9	18	57068021	57068038
chr1_9903	p2	(AG)11	22	57068962	57068983
chr1_9904	p2	(AG)7	14	57104240	57104253
chr1_9905	p2	(AC)13	26	57120267	57120292
chr1_9906	c	(GT)15(GA)11	52	57148845	57148896
chr1_9907	p3	(TTA)5	15	57150217	57150231
chr1_9908	c	actgaacctactagaaatgactgaacacatcaggaccagctttgctgcagtc	140	57151071	57151210
chr1_9909	p5	(AAATC)5	25	57159042	57159066
chr1_9910	p2	(CA)7	14	57165193	57165206
chr1_9911	p2	(CA)7	14	57173697	57173710
chr1_9912	p2	(TA)8	16	57176799	57176814
chr1_9913	p2	(TG)12	24	57182752	57182775
chr1_9914	c	(CT)8(CA)14	44	57190067	57190110
chr1_9915	p2	(CT)6	12	57198979	57198990
chr1_9916	p2	(GT)6	12	57200002	57200013
chr1_9917	p4	(CCAT)6	24	57217132	57217155
chr1_9918	p2	(TC)11	22	57231012	57231033
chr1_9919	p2	(AT)6	12	57247210	57247221
chr1_9920	p2	(AG)7	14	57247667	57247680
chr1_9921	p2	(GT)7	14	57260667	57260680
chr1_9922	p2	(GT)21	42	57265594	57265635
chr1_9923	p2	(GT)7	14	57301906	57301919
chr1_9924	p2	(TC)6	12	57303932	57303943
chr1_9925	p2	(TA)6	12	57305530	57305541
chr1_9926	p2	(AC)7	14	57310837	57310850
chr1_9927	p2	(AC)16	32	57321635	57321666
chr1_9928	p2	(AC)10	20	57324554	57324573

chr1_9929	p2	(AC)12	24	57331318	57331341
chr1_9930	p2	(TG)19	38	57335282	57335319
chr1_9931	p2	(AC)13	26	57340877	57340902
chr1_9932	p2	(AC)6	12	57342015	57342026
chr1_9933	p2	(AC)6	12	57347306	57347317
chr1_9934	c*	gagccagctgaatcatatggagataaaatactatcaaaaatgttacaaatg	153	57349008	57349160
chr1_9935	c	ggctttataagtgtagattgcgcagcacgcatgtgtgatcctcatgacacac	90	57349324	57349413
chr1_9936	p2	(AG)8	16	57350203	57350218
chr1_9937	p3	(TTC)5	15	57358604	57358618
chr1_9938	p3	(AAT)7	21	57361399	57361419
chr1_9939	p3	(TCT)5	15	57364174	57364188
chr1_9940	c	(TTA)6c(TAT)5	34	57367437	57367470
chr1_9941	c	(TTA)6c(TAT)5	34	57369748	57369781
chr1_9942	c	(TC)11(TG)16	54	57371980	57372033
chr1_9943	p2	(TG)12	24	57387250	57387273
chr1_9944	p2	(TG)7	14	57387775	57387788
chr1_9945	p2	(TC)7	14	57400831	57400844
chr1_9946	p2	(AG)6	12	57413808	57413819
chr1_9947	p2	(TG)18	36	57422252	57422287
chr1_9948	p2	(GA)6	12	57423845	57423856
chr1_9949	p3	(AAT)6	18	57426810	57426827
chr1_9950	p2	(AC)7	14	57429896	57429909
chr1_9951	p2	(CA)9	18	57434403	57434420
chr1_9952	p2	(CA)7	14	57438131	57438144
chr1_9953	p4	(TTTA)5	20	57439501	57439520
chr1_9954	c	(TC)8acacacacacat(AC)8	44	57444877	57444920
chr1_9955	p4	(ATCA)7	28	57445944	57445971
chr1_9956	p2	(AC)23	46	57452476	57452521
chr1_9957	p2	(AG)6	12	57455368	57455379
chr1_9958	p3	(TAA)8	24	57457187	57457210
chr1_9959	p4	(GTTT)5	20	57467228	57467247
chr1_9960	p2	(GA)6	12	57471750	57471761
chr1_9961	p2	(AC)7	14	57481397	57481410
chr1_9962	p2	(TC)6	12	57484459	57484470
chr1_9963	p4	(TTTG)6	24	57485716	57485739
chr1_9964	c	aaaaaactggatgcatgaagttatttcagaggctctggcagctcacaaagtt	79	57489015	57489093
chr1_9965	p2	(TG)27	54	57491910	57491963
chr1_9966	p2	(TA)6	12	57492751	57492762
chr1_9967	p2	(TG)7	14	57495086	57495099
chr1_9968	p2	(TG)12	24	57495304	57495327
chr1_9969	p2	(GT)8	16	57498252	57498267
chr1_9970	p2	(AC)22	44	57498599	57498642
chr1_9971	c	(TG)13gttag(GT)6	43	57499146	57499188
chr1_9972	p2	(AT)7	14	57518966	57518979
chr1_9973	p2	(AT)8	16	57520937	57520952
chr1_9974	p2	(AC)18	36	57521718	57521753
chr1_9975	p2	(TG)9	18	57524586	57524603
chr1_9976	c	(TG)11cga(GT)9	43	57525191	57525233
chr1_9977	p2	(TG)6	12	57536574	57536585
chr1_9978	p2	(AC)14	28	57541045	57541072
chr1_9979	p2	(GT)24	48	57544088	57544135
chr1_9980	p2	(CA)16	32	57545233	57545264

chr1_9981	p2	(TG)7	14	57548953	57548966
chr1_9982	p2	(GT)8	16	57550461	57550476
chr1_9983	p2	(GA)6	12	57551357	57551368
chr1_9984	c	(TG)7ta(TG)15	46	57557485	57557530
chr1_9985	p2	(TG)7	14	57559240	57559253
chr1_9986	p5	(CAACT)5	25	57561193	57561217
chr1_9987	p2	(TG)7	14	57578478	57578491
chr1_9988	p2	(CA)10	20	57596567	57596586
chr1_9989	p2	(CA)8	16	57596792	57596807
chr1_9990	p2	(CA)18	36	57597106	57597141
chr1_9991	p2	(CA)11	22	57597294	57597315
chr1_9992	p3	(GAG)7	21	57600433	57600453
chr1_9993	p2	(TG)18	36	57630921	57630956
chr1_9994	c	(GT)6g(GT)30	73	57632674	57632746
chr1_9995	p2	(CA)17	34	57647355	57647388
chr1_9996	p2	(CA)7	14	57648491	57648504
chr1_9997	c	(AC)14aaggtcaaatgtcgcagtgaaggaggagatatt(ATA)6	79	57653838	57653916
chr1_9998	p2	(CA)11	22	57661185	57661206
chr1_9999	p4	(TCTT)6	24	57665267	57665290
chr1_10000	p3	(AAG)5	15	57670768	57670782
chr1_10001	p3	(GGA)6	18	57676567	57676584
chr1_10002	p2	(AC)9	18	57692994	57693011
chr1_10003	p2	(CA)36	72	57699522	57699593
chr1_10004	p4	(AAAC)5	20	57703038	57703057
chr1_10005	p3	(TAA)6	18	57722638	57722655
chr1_10006	c	(GAA)6gaggaggaggat(GAG)5	45	57724059	57724103
chr1_10007	p2	(TG)6	12	57724538	57724549
chr1_10008	p2	(GT)11	22	57726682	57726703
chr1_10009	p5	(TTTGA)5	25	57730426	57730450
chr1_10010	p2	(TG)15	30	57737149	57737178
chr1_10011	p3	(TAA)5	15	57753473	57753487
chr1_10012	p2	(AC)9	18	57755586	57755603
chr1_10013	p3	(TAT)5	15	57778049	57778063
chr1_10014	p2	(AC)6	12	57800684	57800695
chr1_10015	p2	(CA)8	16	57804867	57804882
chr1_10016	p2	(TG)9	18	57808786	57808803
chr1_10017	p2	(TG)7	14	57810142	57810155
chr1_10018	p2	(GA)8	16	57810265	57810280
chr1_10019	p2	(AT)6	12	57811752	57811763
chr1_10020	p2	(AC)7	14	57814824	57814837
chr1_10021	c	(CT)8ccctctccctctccctctccc(CT)6	49	57818740	57818788
chr1_10022	p5	(AAGCA)5	25	57821355	57821379
chr1_10023	p2	(TC)6	12	57822326	57822337
chr1_10024	p2	(TG)6	12	57822565	57822576
chr1_10025	p2	(TC)12	24	57830149	57830172
chr1_10026	c	(CT)12ttt(TC)9	45	57831650	57831694
chr1_10027	c	(GT)12(GA)6	36	57833719	57833754
chr1_10028	p2	(AG)7	14	57834172	57834185
chr1_10029	p2	(GT)8	16	57834710	57834725
chr1_10030	p2	(TG)10	20	57838739	57838758
chr1_10031	p3	(TTA)8	24	57846104	57846127
chr1_10032	p2	(GA)6	12	57846405	57846416

chr1_10033	p2	(GT)9	18	57853474	57853491
chr1_10034	p3	(TGA)5	15	57861699	57861713
chr1_10035	p2	(GT)7	14	57862028	57862041
chr1_10036	p2	(TA)8	16	57862616	57862631
chr1_10037	p2	(TG)6	12	57869723	57869734
chr1_10038	p2	(GT)8	16	57878242	57878257
chr1_10039	p2	(CA)9	18	57880292	57880309
chr1_10040	p2	(GT)7	14	57880501	57880514
chr1_10041	p2	(AC)7	14	57893283	57893296
chr1_10042	p2	(TA)8	16	57893930	57893945
chr1_10043	p4	(TATC)5	20	57903473	57903492
chr1_10044	p2	(GA)6	12	57912255	57912266
chr1_10045	p2	(TC)16	32	57918078	57918109
chr1_10046	p2	(TA)14	28	57939434	57939461
chr1_10047	p2	(GT)6	12	57940035	57940046
chr1_10048	p2	(AT)6	12	57946711	57946722
chr1_10049	p2	(AG)6	12	57947126	57947137
chr1_10050	p4	(TTTG)5	20	57963910	57963929
chr1_10051	p4	(TTTG)5	20	57964266	57964285
chr1_10052	p2	(GT)6	12	57964425	57964436
chr1_10053	p2	(AT)7	14	57967357	57967370
chr1_10054	p3	(TCT)9	27	57980676	57980702
chr1_10055	p2	(CA)11	22	58001455	58001476
chr1_10056	p2	(TG)7	14	58015703	58015716
chr1_10057	p2	(TG)20	40	58018349	58018388
chr1_10058	p2	(CA)22	44	58018698	58018741
chr1_10059	c	(CA)6cg(CA)7	28	58019482	58019509
chr1_10060	p2	(TG)23	46	58019751	58019796
chr1_10061	p2	(CA)6	12	58021787	58021798
chr1_10062	p3	(ACA)6	18	58023128	58023145
chr1_10063	p3	(TAG)5	15	58039489	58039503
chr1_10064	p2	(GA)6	12	58040702	58040713
chr1_10065	p3	(CAG)5	15	58041004	58041018
chr1_10066	p2	(AT)6	12	58044428	58044439
chr1_10067	p3	(TTG)7	21	58052935	58052955
chr1_10068	p2	(TG)7	14	58057795	58057808
chr1_10069	p2	(AC)9	18	58060820	58060837
chr1_10070	c	(AC)15t(CA)7	45	58061906	58061950
chr1_10071	p2	(AC)23	46	58072831	58072876
chr1_10072	p2	(CA)6	12	58073155	58073166
chr1_10073	p2	(TA)9	18	58081662	58081679
chr1_10074	p3	(CAA)5	15	58084209	58084223
chr1_10075	c	(AT)8(AC)12	40	58085911	58085950
chr1_10076	p2	(CA)19	38	58086125	58086162
chr1_10077	p4	(ATGG)5	20	58088688	58088707
chr1_10078	c	(CA)16gtagcacagagacaca(AC)14	76	58090890	58090965
chr1_10079	p2	(CT)6	12	58094119	58094130
chr1_10080	p3	(TCA)5	15	58097232	58097246
chr1_10081	p2	(CA)6	12	58107749	58107760
chr1_10082	p2	(TG)9	18	58112665	58112682
chr1_10083	p2	(TA)9	18	58116272	58116289
chr1_10084	p2	(TG)8	16	58119075	58119090

chr1_10085	p2	(CA)6	12	58132150	58132161
chr1_10086	p2	(TG)8	16	58132298	58132313
chr1_10087	p2	(TG)18	36	58132633	58132668
chr1_10088	c	(CA)15cccctagtttttcctactatgtggggactgct(CA)7	76	58133782	58133857
chr1_10089	p2	(CA)12	24	58135058	58135081
chr1_10090	p2	(AC)6	12	58142908	58142919
chr1_10091	p4	(ATTC)5	20	58156480	58156499
chr1_10092	p5	(ATCAA)5	25	58158137	58158161
chr1_10093	p2	(AC)6	12	58182660	58182671
chr1_10094	p2	(GT)24	48	58186987	58187034
chr1_10095	p2	(GT)15	30	58191763	58191792
chr1_10096	p5	(TTGAA)5	25	58210913	58210937
chr1_10097	p3	(TTG)5	15	58218412	58218426
chr1_10098	p5	(GGAGA)9	45	58225108	58225152
chr1_10099	p2	(CA)6	12	58232705	58232716
chr1_10100	p3	(ATC)7	21	58244776	58244796
chr1_10101	p2	(GT)11	22	58261942	58261963
chr1_10102	p4	(GGCA)7	28	58265246	58265273
chr1_10103	p2	(TG)9	18	58269346	58269363
chr1_10104	p2	(CT)6	12	58278782	58278793
chr1_10105	p2	(CA)17	34	58280481	58280514
chr1_10106	c	(AG)12tatatacat(AC)10	53	58281991	58282043
chr1_10107	p2	(GT)9	18	58284661	58284678
chr1_10108	c	(GT)15atttataaaatgccccattcgccccgcacatatatacttt(TA)18	106	58286447	58286552
chr1_10109	p2	(TG)6	12	58293235	58293246
chr1_10110	p4	(TCCA)5	20	58294242	58294261
chr1_10111	p2	(AC)6	12	58297107	58297118
chr1_10112	c	(GAA)16caagaacaagaacaagaactgcaat(AAC)5	88	58333897	58333984
chr1_10113	p2	(GT)11	22	58335818	58335839
chr1_10114	p2	(AC)9	18	58355320	58355337
chr1_10115	p2	(GT)6	12	58358678	58358689
chr1_10116	p4	(AATG)5	20	58358830	58358849
chr1_10117	p3	(GAG)5	15	58372661	58372675
chr1_10118	c	(AC)15(AT)8	46	58373276	58373321
chr1_10119	p2	(AC)14	28	58375177	58375204
chr1_10120	p2	(TG)24	48	58377306	58377353
chr1_10121	p2	(TG)6	12	58378227	58378238
chr1_10122	p2	(TG)6	12	58380851	58380862
chr1_10123	p2	(CT)8	16	58385946	58385961
chr1_10124	p3	(GGT)5	15	58387246	58387260
chr1_10125	p3	(GGT)5	15	58387387	58387401
chr1_10126	p3	(AAG)6	18	58390824	58390841
chr1_10127	p2	(TG)13	26	58391909	58391934
chr1_10128	p2	(TG)7	14	58392610	58392623
chr1_10129	c	(CT)8(CA)7	30	58417664	58417693
chr1_10130	p2	(GT)7	14	58423716	58423729
chr1_10131	p2	(GT)7	14	58426264	58426277
chr1_10132	p2	(TC)10	20	58430210	58430229
chr1_10133	p2	(TG)7	14	58430723	58430736
chr1_10134	p2	(AT)6	12	58430989	58431000
chr1_10135	p2	(TG)6	12	58433458	58433469
chr1_10136	p2	(CT)9	18	58443523	58443540

chr1_10137	p2	(CA)16	32	58443868	58443899
chr1_10138	p2	(TG)7	14	58458929	58458942
chr1_10139	p2	(AC)6	12	58476760	58476771
chr1_10140	p2	(AC)12	24	58483690	58483713
chr1_10141	p2	(AC)9	18	58486874	58486891
chr1_10142	p2	(TG)7	14	58490393	58490406
chr1_10143	c	(AC)6(AT)6	24	58494813	58494836
chr1_10144	p2	(TA)10	20	58506186	58506205
chr1_10145	c	acaaaacacatgcatatgttacaaaaaaaaggattttaaaggattttatctct	126	58527301	58527426
chr1_10146	p2	(CA)12	24	58527825	58527848
chr1_10147	c	(CA)6t(AC)11	35	58530016	58530050
chr1_10148	p2	(GA)8	16	58530185	58530200
chr1_10149	p2	(TG)12	24	58531612	58531635
chr1_10150	p2	(GA)8	16	58535447	58535462
chr1_10151	c	(TC)11ttg(CT)12	49	58545934	58545982
chr1_10152	p2	(CA)9	18	58556813	58556830
chr1_10153	p3	(ACA)5	15	58560875	58560889
chr1_10154	p2	(TG)6	12	58583136	58583147
chr1_10155	c	(TC)14(AC)11	50	58590346	58590395
chr1_10156	p2	(AT)8	16	58594314	58594329
chr1_10157	p2	(CA)6	12	58607693	58607704
chr1_10158	p2	(AG)6	12	58614573	58614584
chr1_10159	p2	(CA)11	22	58614809	58614830
chr1_10160	p2	(TC)10	20	58619443	58619462
chr1_10161	p2	(CA)14	28	58619792	58619819
chr1_10162	p2	(AC)10	20	58621269	58621288
chr1_10163	c	(AC)7at(AC)15	46	58622735	58622780
chr1_10164	c	(TCAC)5tcaat(CA)6	37	58622892	58622928
chr1_10165	c	(TA)6ttcttgtgtacaccccccca(AC)8	47	58623347	58623393
chr1_10166	p2	(AC)6	12	58624262	58624273
chr1_10167	p2	(CA)18	36	58634847	58634882
chr1_10168	p2	(CA)11	22	58637283	58637304
chr1_10169	p2	(AG)6	12	58647604	58647615
chr1_10170	p2	(AT)6	12	58655074	58655085
chr1_10171	p3	(ATT)5	15	58660847	58660861
chr1_10172	p2	(AC)8	16	58660970	58660985
chr1_10173	p4	(TCAA)7	28	58663299	58663326
chr1_10174	p2	(GT)20	40	58679475	58679514
chr1_10175	p3	(ATT)5	15	58685686	58685700
chr1_10176	p2	(CA)6	12	58688108	58688119
chr1_10177	p2	(TG)15	30	58706246	58706275
chr1_10178	c	(AC)13atatatatacaatac(AT)9	59	58708907	58708965
chr1_10179	p2	(CT)11	22	58709099	58709120
chr1_10180	p3	(TTA)7	21	58711797	58711817
chr1_10181	c	(GT)10atatatt(TA)10	47	58713284	58713330
chr1_10182	p2	(GA)13	26	58736573	58736598
chr1_10183	p3	(ACA)7	21	58740716	58740736
chr1_10184	p2	(AC)6	12	58745655	58745666
chr1_10185	c	(AC)23(AG)11	68	58746146	58746213
chr1_10186	p2	(CA)6	12	58749504	58749515
chr1_10187	c	(AT)16acacatatat(AC)12	66	58750001	58750066
chr1_10188	p2	(AG)15	30	58764214	58764243

chr1_10189	p2	(TG)8	16	58773644	58773659
chr1_10190	p3	(AAT)7	21	58796720	58796740
chr1_10191	p3	(TTC)5	15	58799205	58799219
chr1_10192	p2	(AC)17	34	58805426	58805459
chr1_10193	c	(ATA)7atcataatactaat(AAG)5	50	58810558	58810607
chr1_10194	p2	(GT)19	38	58820151	58820188
chr1_10195	c	(AC)9g(CA)9	37	58822141	58822177
chr1_10196	p2	(TG)6	12	58840928	58840939
chr1_10197	c*	(GACA)5(AC)7*	31	58848773	58848803
chr1_10198	p3	(AAT)7	21	58855268	58855288
chr1_10199	p3	(AAT)7	21	58857291	58857311
chr1_10200	c	(ATGG)5aggtcac(TG)8	43	58860585	58860627
chr1_10201	p2	(AC)10	20	58869415	58869434
chr1_10202	p2	(TG)14	28	58878217	58878244
chr1_10203	p2	(AC)6	12	58896473	58896484
chr1_10204	p2	(TG)6	12	58913577	58913588
chr1_10205	p3	(TAA)5	15	58914724	58914738
chr1_10206	c	(TG)12ca(TG)7	40	58914994	58915033
chr1_10207	c	(GT)12gagagtgtgtgtgagagagaa(AG)9	63	58916296	58916358
chr1_10208	p2	(CA)10	20	58916617	58916636
chr1_10209	p2	(TG)8	16	58923644	58923659
chr1_10210	p3	(ATT)9	27	58925459	58925485
chr1_10211	p2	(TA)6	12	58937270	58937281
chr1_10212	p3	(ATA)5	15	58964281	58964295
chr1_10213	p2	(AC)10	20	58994294	58994313
chr1_10214	p2	(GA)17	34	58995485	58995518
chr1_10215	p2	(AG)8	16	58997100	58997115
chr1_10216	p2	(GT)6	12	59023663	59023674
chr1_10217	p2	(GT)7	14	59024731	59024744
chr1_10218	c	11(TA)8cacacagaggtcagaggtcacaggctgcaggttcaatata(T	100	59027582	59027681
chr1_10219	p2	(AC)11	22	59027805	59027826
chr1_10220	p2	(TG)19	38	59029822	59029859
chr1_10221	p2	(AC)29	58	59030703	59030760
chr1_10222	p2	(TG)7	14	59031058	59031071
chr1_10223	p2	(TC)14	28	59032651	59032678
chr1_10224	p2	(TG)6	12	59038180	59038191
chr1_10225	p3	(TTC)5	15	59043997	59044011
chr1_10226	p2	(GT)17	34	59044866	59044899
chr1_10227	p2	(GA)7	14	59045165	59045178
chr1_10228	p3	(CTG)5	15	59054731	59054745
chr1_10229	p2	(AC)13	26	59060595	59060620
chr1_10230	p2	(GT)6	12	59061405	59061416
chr1_10231	c	(AC)6g(CA)7	27	59064004	59064030
chr1_10232	p2	(AC)18	36	59069234	59069269
chr1_10233	p2	(CA)8	16	59072277	59072292
chr1_10234	c	(TC)6(AC)11	34	59075473	59075506
chr1_10235	p2	(GT)6	12	59082686	59082697
chr1_10236	p2	(CA)6	12	59083448	59083459
chr1_10237	p2	(AC)6	12	59091032	59091043
chr1_10238	p2	(TG)25	50	59096281	59096330
chr1_10239	p4	(AAAT)5	20	59106825	59106844
chr1_10240	p3	(CCA)5	15	59107639	59107653

chr1_10241	c	(AC)10agtc(GA)14	52	59112009	59112060
chr1_10242	p4	(CTAA)5	20	59113041	59113060
chr1_10243	p2	(CA)10	20	59132482	59132501
chr1_10244	c	(CA)17(GA)6	46	59147329	59147374
chr1_10245	p2	(CA)21	42	59157126	59157167
chr1_10246	p2	(TG)6	12	59158103	59158114
chr1_10247	p4	(ATTT)5	20	59160525	59160544
chr1_10248	p2	(GT)6	12	59168985	59168996
chr1_10249	p2	(AC)6	12	59176677	59176688
chr1_10250	p2	(TG)7	14	59178614	59178627
chr1_10251	c	[illegible])5attattttggactcggataatacaaacttcacatttaatgatgatgatta(T[illegible]	89	59180366	59180454
chr1_10252	p2	(GA)6	12	59191065	59191076
chr1_10253	p2	(GT)10	20	59205504	59205523
chr1_10254	p2	(TG)20	40	59205777	59205816
chr1_10255	p2	(TC)6	12	59217054	59217065
chr1_10256	p2	(GT)20	40	59223744	59223783
chr1_10257	c	(TG)20(AG)6	52	59241826	59241877
chr1_10258	p2	(TG)8	16	59275070	59275085
chr1_10259	p4	(TTGA)5	20	59287707	59287726
chr1_10260	p2	(CA)6	12	59295876	59295887
chr1_10261	p3	(GAT)5	15	59300984	59300998
chr1_10262	p3	(ATG)5	15	59305147	59305161
chr1_10263	p4	(GTGA)5	20	59309905	59309924
chr1_10264	p2	(AC)9	18	59318310	59318327
chr1_10265	p2	(TA)8	16	59325010	59325025
chr1_10266	p2	(TG)6	12	59327086	59327097
chr1_10267	p2	(CA)7	14	59335452	59335465
chr1_10268	c	(CT)9(CA)32	82	59335709	59335790
chr1_10269	p2	(AC)17	34	59358809	59358842
chr1_10270	p2	(GT)6	12	59362545	59362556
chr1_10271	p2	(AC)9	18	59409361	59409378
chr1_10272	p2	(CT)7	14	59419009	59419022
chr1_10273	p5	(CTTCT)19	95	59419678	59419772
chr1_10274	p2	(TC)8	16	59435324	59435339
chr1_10275	p3	(GTT)5	15	59441440	59441454
chr1_10276	p3	(TGA)6	18	59497284	59497301
chr1_10277	p2	(CT)6	12	59497970	59497981
chr1_10278	p2	(GT)7	14	59503395	59503408
chr1_10279	p2	(TG)6	12	59509454	59509465
chr1_10280	p2	(GT)7	14	59514354	59514367
chr1_10281	p2	(AC)8	16	59519994	59520009
chr1_10282	p2	(TG)43	86	59536729	59536814
chr1_10283	p3	(GAT)6	18	59545155	59545172
chr1_10284	p2	(AC)10	20	59547741	59547760
chr1_10285	p2	(GT)7	14	59552207	59552220
chr1_10286	p2	(TG)14	28	59557357	59557384
chr1_10287	p2	(TA)10	20	59559275	59559294
chr1_10288	p2	(CA)7	14	59567132	59567145
chr1_10289	c	[illegible]cctttatctatataca(GT)6gcgcatgtgtgtgtacacgcatgggtgcaa[illegible]	218	59575054	59575271
chr1_10290	p2	(TG)6	12	59578051	59578062
chr1_10291	c	(GT)6gagt(GA)34	84	59584918	59585001
chr1_10292	p5	(AAATC)6	30	59591364	59591393

chr1_10293	p2	(CT)6	12	59597732	59597743
chr1_10294	p4	(CCAT)7	28	59602785	59602812
chr1_10295	p4	(TTTC)6	24	59615439	59615462
chr1_10296	p2	(CA)6	12	59621582	59621593
chr1_10297	p2	(AC)9	18	59623546	59623563
chr1_10298	p2	(AC)16	32	59626308	59626339
chr1_10299	p2	(GT)8	16	59634350	59634365
chr1_10300	p3	(CCT)5	15	59634621	59634635
chr1_10301	p2	(TC)8	16	59641042	59641057
chr1_10302	p2	(TG)7	14	59642963	59642976
chr1_10303	p4	(CAAA)5	20	59643725	59643744
chr1_10304	p3	(TTA)8	24	59644186	59644209
chr1_10305	p2	(TG)9	18	59647022	59647039
chr1_10306	p2	(AC)28	56	59647330	59647385
chr1_10307	p2	(AT)6	12	59649739	59649750
chr1_10308	p3	(TTG)5	15	59651709	59651723
chr1_10309	c	(AC)10c(CA)10	41	59659337	59659377
chr1_10310	p3	(TGC)5	15	59661422	59661436
chr1_10311	p3	(ATT)5	15	59677549	59677563
chr1_10312	p2	(AG)7	14	59696108	59696121
chr1_10313	p2	(GT)8	16	59702174	59702189
chr1_10314	c	(AC)27aaaacgtaaa(CAT)6	82	59705822	59705903
chr1_10315	p2	(AC)14	28	59706669	59706696
chr1_10316	p2	(GT)6	12	59710477	59710488
chr1_10317	p2	(AC)34	68	59721661	59721728
chr1_10318	p2	(TG)8	16	59729244	59729259
chr1_10319	p2	(TG)6	12	59736287	59736298
chr1_10320	p2	(GT)8	16	59742059	59742074
chr1_10321	p3	(GGA)5	15	59750928	59750942
chr1_10322	p2	(GT)12	24	59754231	59754254
chr1_10323	p2	(GT)19	38	59755624	59755661
chr1_10324	p2	(TA)6	12	59756956	59756967
chr1_10325	p3	(AAC)9	27	59757370	59757396
chr1_10326	p2	(CA)14	28	59758159	59758186
chr1_10327	c	8gctcccagtgttccccaacacaactgtcaaaaaagttctctgtctctttc(C	88	59762379	59762466
chr1_10328	p3	(AAC)6	18	59763845	59763862
chr1_10329	p2	(CA)8	16	59767212	59767227
chr1_10330	p2	(TG)7	14	59773628	59773641
chr1_10331	p2	(TG)15	30	59773905	59773934
chr1_10332	p2	(GT)8	16	59782469	59782484
chr1_10333	p2	(AC)7	14	59782981	59782994
chr1_10334	p2	(CA)6	12	59788330	59788341
chr1_10335	p2	(CA)15	30	59797281	59797310
chr1_10336	p2	(GT)10	20	59799500	59799519
chr1_10337	p2	(AG)6	12	59800448	59800459
chr1_10338	c	(CT)14cacacacaca(CG)7(CA)13	78	59808956	59809033
chr1_10339	c	:actccggtcccccttgaagatagcaatgcatggatttttggccataaaagtat	109	59810857	59810965
chr1_10340	p2	(CA)8	16	59811108	59811123
chr1_10341	p2	(AT)6	12	59812851	59812862
chr1_10342	p4	(GGTA)6	24	59815873	59815896
chr1_10343	c	(CA)9c(CA)17	53	59820972	59821024
chr1_10344	p2	(GT)8	16	59823081	59823096

chr1_10345	p2	(GT)8	16	59824455	59824470
chr1_10346	p2	(GT)17	34	59826221	59826254
chr1_10347	p4	(CCAT)5	20	59831625	59831644
chr1_10348	p3	(AAT)5	15	59833399	59833413
chr1_10349	p2	(TG)6	12	59868512	59868523
chr1_10350	p2	(TA)7	14	59876068	59876081
chr1_10351	p4	(TCCA)5	20	59891437	59891456
chr1_10352	p2	(AC)7	14	59891941	59891954
chr1_10353	p2	(GT)6	12	59892787	59892798
chr1_10354	p3	(TTA)6	18	59917021	59917038
chr1_10355	p2	(TG)10	20	59926907	59926926
chr1_10356	p3	(ATT)7	21	59928581	59928601
chr1_10357	p3	(TAA)5	15	59931281	59931295
chr1_10358	p2	(AC)8	16	59933686	59933701
chr1_10359	p2	(CA)7	14	59934029	59934042
chr1_10360	p2	(TG)22	44	59935008	59935051
chr1_10361	p2	(AC)14	28	59938510	59938537
chr1_10362	c	(CA)12gtg(TA)19	65	59938904	59938968
chr1_10363	p2	(GT)18	36	59966262	59966297
chr1_10364	p3	(AAT)6	18	59970994	59971011
chr1_10365	p2	(CA)14	28	59975951	59975978
chr1_10366	p2	(GT)16	32	59984773	59984804
chr1_10367	p2	(TA)6	12	59992434	59992445
chr1_10368	p4	(TATT)5	20	59992944	59992963
chr1_10369	p2	(AG)6	12	59998615	59998626
chr1_10370	p3	(AGT)5	15	59999673	59999687
chr1_10371	p2	(TG)6	12	60013426	60013437
chr1_10372	p2	(AC)6	12	60014301	60014312
chr1_10373	p2	(CA)16	32	60025508	60025539
chr1_10374	p2	(TG)6	12	60027458	60027469
chr1_10375	p2	(TA)6	12	60066944	60066955
chr1_10376	p2	(TG)10	20	60103332	60103351
chr1_10377	p3	(TGC)5	15	60104818	60104832
chr1_10378	p2	(CA)10	20	60120065	60120084
chr1_10379	p2	(CT)6	12	60120256	60120267
chr1_10380	c	(GA)7ggag(GA)7	32	60133416	60133447
chr1_10381	p5	(AAATC)5	25	60139660	60139684
chr1_10382	p2	(GT)6	12	60147559	60147570
chr1_10383	p4	(GATA)6	24	60151531	60151554
chr1_10384	p2	(TG)13	26	60160157	60160182
chr1_10385	p2	(GT)16	32	60161070	60161101
chr1_10386	p2	(CT)27	54	60168076	60168129
chr1_10387	c	(TG)16tcattatgacatctgaggcctaccagcgaattctggcgc(GT)8	87	60179180	60179266
chr1_10388	p2	(TG)9	18	60190655	60190672
chr1_10389	p2	(GT)26	52	60193695	60193746
chr1_10390	p2	(TG)12	24	60207757	60207780
chr1_10391	p2	(CT)14	28	60212640	60212667
chr1_10392	p2	(AC)8	16	60231914	60231929
chr1_10393	p3	(TAT)7	21	60236917	60236937
chr1_10394	p2	(AC)26	52	60242952	60243003
chr1_10395	p2	(AC)6	12	60252543	60252554
chr1_10396	p4	(AAGG)5	20	60269380	60269399

chr1_10397	p2	(CA)22	44	60270071	60270114
chr1_10398	p2	(TC)6	12	60274216	60274227
chr1_10399	p4	(TCCA)6	24	60284336	60284359
chr1_10400	p3	(TCA)5	15	60286123	60286137
chr1_10401	p2	(AC)22	44	60289595	60289638
chr1_10402	p2	(CT)17	34	60302980	60303013
chr1_10403	p2	(CA)6	12	60306023	60306034
chr1_10404	p2	(AC)6	12	60323766	60323777
chr1_10405	p2	(GT)12	24	60326083	60326106
chr1_10406	p2	(AT)6	12	60328090	60328101
chr1_10407	p3	(AGT)5	15	60341672	60341686
chr1_10408	c	(AAT)5(TAT)5	30	60359473	60359502
chr1_10409	p5	(TTGAT)5	25	60365231	60365255
chr1_10410	p4	(AAAC)5	20	60366418	60366437
chr1_10411	p2	(GT)16	32	60370267	60370298
chr1_10412	p2	(AC)6	12	60382761	60382772
chr1_10413	p2	(GT)7	14	60385478	60385491
chr1_10414	p2	(TG)13	26	60394136	60394161
chr1_10415	p5	(GTATG)7	35	60397586	60397620
chr1_10416	p2	(AC)9	18	60406242	60406259
chr1_10417	p2	(AC)6	12	60409374	60409385
chr1_10418	p2	(TG)11	22	60410662	60410683
chr1_10419	p2	(AC)6	12	60429909	60429920
chr1_10420	c	(CA)8cgcacacacacg(CA)7	42	60440364	60440405
chr1_10421	p2	(GA)11	22	60448485	60448506
chr1_10422	p2	(TG)16	32	60470628	60470659
chr1_10423	p3	(TTA)5	15	60471024	60471038
chr1_10424	p2	(TA)7	14	60531205	60531218
chr1_10425	p2	(TG)15	30	60539190	60539219
chr1_10426	p2	(CT)7	14	60576325	60576338
chr1_10427	c	(TG)9a(GT)8	35	60578247	60578281
chr1_10428	p3	(TAC)26	78	60580940	60581017
chr1_10429	p4	(CATC)5	20	60592687	60592706
chr1_10430	p2	(TG)7	14	60603042	60603055
chr1_10431	p2	(TA)19	38	60603643	60603680
chr1_10432	p2	(CA)13	26	60610157	60610182
chr1_10433	p2	(AC)18	36	60610442	60610477
chr1_10434	p2	(CA)20	40	60615491	60615530
chr1_10435	c*	(CCA)5(CA)6*	25	60626453	60626477
chr1_10436	p5	(TTTGA)5	25	60630710	60630734
chr1_10437	p2	(TG)8	16	60638177	60638192
chr1_10438	c	gtgttcttgtctcggtcccctcggctggctcggactcggatcgttgcctgcct	115	60651781	60651895
chr1_10439	p2	(GT)6	12	60665406	60665417
chr1_10440	p2	(TG)7	14	60675937	60675950
chr1_10441	p2	(GT)15	30	60679739	60679768
chr1_10442	p4	(TTTG)7	28	60720924	60720951
chr1_10443	p5	(AAATC)8	40	60722466	60722505
chr1_10444	p2	(GT)14	28	60724877	60724904
chr1_10445	p3	(CTT)6	18	60746643	60746660
chr1_10446	p5	(AAATC)5	25	60750863	60750887
chr1_10447	p2	(GA)6	12	60752782	60752793
chr1_10448	c*	gcacacacattgagagctgatgtaccagagtcaaattcctcgtgtgtctga	160	60757489	60757648

chr1_10449	p3	(GAA)8	24	60779812	60779835
chr1_10450	p2	(AC)6	12	60791594	60791605
chr1_10451	c	(CCA)5gcaccacgagagatgccagaaaccctgcaatctaa(AC)8	66	60792343	60792408
chr1_10452	p4	(CATT)5	20	60806763	60806782
chr1_10453	p2	(GT)10	20	60815787	60815806
chr1_10454	p3	(GAA)5	15	60819478	60819492
chr1_10455	p3	(TTA)5	15	60820309	60820323
chr1_10456	p4	(AAAT)5	20	60834056	60834075
chr1_10457	p2	(TC)7	14	60844927	60844940
chr1_10458	p2	(AT)8	16	60845353	60845368
chr1_10459	p2	(CA)6	12	60845947	60845958
chr1_10460	p4	(TGTT)5	20	60869180	60869199
chr1_10461	p2	(AC)8	16	60888886	60888901
chr1_10462	p2	(AC)8	16	60890222	60890237
chr1_10463	p2	(AC)9	18	60891628	60891645
chr1_10464	p2	(CT)7	14	60892266	60892279
chr1_10465	p2	(AC)6	12	60893452	60893463
chr1_10466	p2	(AC)25	50	60894436	60894485
chr1_10467	p2	(GT)11	22	60894602	60894623
chr1_10468	p3	(ATA)5	15	60897208	60897222
chr1_10469	p3	(GGT)6	18	60914888	60914905
chr1_10470	c	(TG)8ttt(GA)7	33	60931594	60931626
chr1_10471	c	(AC)10ga(AC)20	62	60933180	60933241
chr1_10472	p2	(CA)6	12	60936338	60936349
chr1_10473	p2	(CA)10	20	60944769	60944788
chr1_10474	p2	(CA)9	18	60946659	60946676
chr1_10475	p3	(ATC)5	15	60947240	60947254
chr1_10476	p3	(AAG)5	15	60957519	60957533
chr1_10477	p2	(TA)8	16	60958475	60958490
chr1_10478	p2	(TG)6	12	60971374	60971385
chr1_10479	p3	(CTT)5	15	60975982	60975996
chr1_10480	p2	(AC)17	34	60995206	60995239
chr1_10481	p5	(CATAT)5	25	60999806	60999830
chr1_10482	p2	(AG)23	46	61001101	61001146
chr1_10483	p2	(AG)6	12	61002140	61002151
chr1_10484	p3	(TTG)5	15	61017561	61017575
chr1_10485	p2	(TG)16	32	61044174	61044205
chr1_10486	p3	(TTG)7	21	61052115	61052135
chr1_10487	p3	(AAT)5	15	61070386	61070400
chr1_10488	p5	(TGTTT)5	25	61076374	61076398
chr1_10489	p2	(TG)6	12	61097662	61097673
chr1_10490	p2	(TA)8	16	61107007	61107022
chr1_10491	p2	(TC)6	12	61114231	61114242
chr1_10492	p3	(ACA)5	15	61116143	61116157
chr1_10493	p2	(CA)13	26	61120864	61120889
chr1_10494	p2	(AG)7	14	61152442	61152455
chr1_10495	p3	(ATC)6	18	61153026	61153043
chr1_10496	p3	(TGT)21	63	61156755	61156817
chr1_10497	p2	(TA)6	12	61172218	61172229
chr1_10498	p2	(CA)6	12	61174552	61174563
chr1_10499	c	(ACAG)6(AC)10	44	61180173	61180216
chr1_10500	p2	(AC)6	12	61180697	61180708

chr1_10501	p2	(AG)6	12	61183859	61183870
chr1_10502	p2	(AC)7	14	61187144	61187157
chr1_10503	p4	(TGAA)6	24	61193871	61193894
chr1_10504	p3	(TGT)5	15	61203824	61203838
chr1_10505	p3	(TAA)5	15	61207008	61207022
chr1_10506	p3	(AAT)5	15	61209228	61209242
chr1_10507	p2	(CA)6	12	61215865	61215876
chr1_10508	p5	(TTTGA)5	25	61220714	61220738
chr1_10509	p2	(CA)10	20	61223754	61223773
chr1_10510	p2	(CA)6	12	61228008	61228019
chr1_10511	p3	(TGA)8	24	61233130	61233153
chr1_10512	p2	(GT)17	34	61243380	61243413
chr1_10513	p4	(ATTT)7	28	61268588	61268615
chr1_10514	p2	(CT)11	22	61271733	61271754
chr1_10515	p3	(ATA)5	15	61279127	61279141
chr1_10516	p2	(GT)9	18	61285537	61285554
chr1_10517	p2	(AC)9	18	61285831	61285848
chr1_10518	p2	(AC)11	22	61287363	61287384
chr1_10519	p2	(GT)7	14	61288184	61288197
chr1_10520	p2	(AC)9	18	61288484	61288501
chr1_10521	p3	(TCA)8	24	61291995	61292018
chr1_10522	p2	(AC)20	40	61294536	61294575
chr1_10523	p2	(AT)6	12	61296054	61296065
chr1_10524	c	gccaggttaacaacttggtacagagcacaaaaatgtattaaccaccgacac	142	61300945	61301086
chr1_10525	p2	(AC)30	60	61303101	61303160
chr1_10526	p3	(CTC)5	15	61305195	61305209
chr1_10527	p2	(TG)8	16	61307997	61308012
chr1_10528	p2	(CA)13	26	61308171	61308196
chr1_10529	p2	(CA)11	22	61316618	61316639
chr1_10530	p2	(TG)8	16	61318984	61318999
chr1_10531	p2	(AC)16	32	61321818	61321849
chr1_10532	p2	(CA)12	24	61322535	61322558
chr1_10533	c	(TG)20tt(TG)6	54	61326196	61326249
chr1_10534	p2	(TG)7	14	61330485	61330498
chr1_10535	p2	(GA)39	78	61344229	61344306
chr1_10536	c	(GT)8atgtatgtgtg(TA)8	43	61344965	61345007
chr1_10537	p3	(TAT)5	15	61345564	61345578
chr1_10538	p2	(TC)6	12	61346219	61346230
chr1_10539	p2	(GT)14	28	61364283	61364310
chr1_10540	p2	(AC)13	26	61381377	61381402
chr1_10541	p2	(GT)25	50	61381860	61381909
chr1_10542	p2	(AC)7	14	61382142	61382155
chr1_10543	p3	(GTG)12	36	61387850	61387885
chr1_10544	c	gatttgggggaagagtgtttctgtggtctccttggctgcgccgctgttgctca	111	61398799	61398909
chr1_10545	p2	(AG)7	14	61402154	61402167
chr1_10546	p2	(TG)19	38	61415609	61415646
chr1_10547	p4	(TCAA)5	20	61423396	61423415
chr1_10548	p2	(AC)20	40	61426888	61426927
chr1_10549	p3	(AAC)5	15	61434213	61434227
chr1_10550	p2	(TG)8	16	61441724	61441739
chr1_10551	p2	(AC)7	14	61448655	61448668
chr1_10552	p2	(TG)17	34	61449644	61449677

chr1_10553	p2	(CA)32	64	61449810	61449873
chr1_10554	p2	(GT)7	14	61450037	61450050
chr1_10555	p2	(TG)48	96	61451044	61451139
chr1_10556	p2	(CA)14	28	61453790	61453817
chr1_10557	c	(TA)12cacacatacacaatatatgta(TG)6	57	61454169	61454225
chr1_10558	p2	(TG)22	44	61454829	61454872
chr1_10559	p2	(GT)13	26	61455112	61455137
chr1_10560	p2	(GT)14	28	61456298	61456325
chr1_10561	p2	(CT)6	12	61462620	61462631
chr1_10562	p2	(AC)16	32	61465150	61465181
chr1_10563	p3	(TTA)6	18	61469044	61469061
chr1_10564	p4	(CTAC)6	24	61469238	61469261
chr1_10565	p2	(CA)16	32	61478138	61478169
chr1_10566	p2	(CA)10	20	61478306	61478325
chr1_10567	p2	(AC)20	40	61481099	61481138
chr1_10568	p2	(AC)9	18	61484627	61484644
chr1_10569	p2	(AT)9	18	61491778	61491795
chr1_10570	p2	(TG)7	14	61499724	61499737
chr1_10571	p2	(AT)7	14	61500284	61500297
chr1_10572	p2	(CA)8	16	61504364	61504379
chr1_10573	p2	(GT)6	12	61504664	61504675
chr1_10574	p4	(TTTC)5	20	61505263	61505282
chr1_10575	p2	(GT)16	32	61521555	61521586
chr1_10576	p2	(GT)8	16	61524520	61524535
chr1_10577	p2	(CA)7	14	61528609	61528622
chr1_10578	p2	(GA)6	12	61529095	61529106
chr1_10579	p2	(AC)7	14	61537585	61537598
chr1_10580	p4	(TATT)5	20	61539119	61539138
chr1_10581	p2	(TC)6	12	61540279	61540290
chr1_10582	p2	(AC)6	12	61542272	61542283
chr1_10583	p2	(TG)6	12	61542401	61542412
chr1_10584	p2	(GT)9	18	61542538	61542555
chr1_10585	p4	(GGTA)6	24	61551449	61551472
chr1_10586	p2	(TG)6	12	61562165	61562176
chr1_10587	p2	(AC)6	12	61572053	61572064
chr1_10588	p2	(TG)8	16	61575046	61575061
chr1_10589	p4	(TAAA)5	20	61576069	61576088
chr1_10590	p2	(AC)6	12	61580726	61580737
chr1_10591	p2	(AC)6	12	61583926	61583937
chr1_10592	c	(CT)16cacac(CA)17t(AC)17	106	61589517	61589622
chr1_10593	p2	(GT)6	12	61592975	61592986
chr1_10594	p2	(TG)13	26	61596247	61596272
chr1_10595	p2	(AG)6	12	61615396	61615407
chr1_10596	c	tgttggcgcctcataccaggtgtttttatcttagtcctccaggagtcctggcct	93	61620952	61621044
chr1_10597	p2	(CA)7	14	61629627	61629640
chr1_10598	p3	(AAT)9	27	61632778	61632804
chr1_10599	p2	(AC)6	12	61637277	61637288
chr1_10600	p2	(TG)21	42	61645764	61645805
chr1_10601	p2	(GT)12	24	61645990	61646013
chr1_10602	p2	(AC)6	12	61646907	61646918
chr1_10603	p2	(TG)23	46	61648038	61648083
chr1_10604	p2	(TG)7	14	61650000	61650013

chr1_10605	p2	(CA)8	16	61651067	61651082
chr1_10606	p2	(GT)6	12	61652449	61652460
chr1_10607	p2	(TG)6	12	61652592	61652603
chr1_10608	p4	(TCCA)5	20	61657485	61657504
chr1_10609	p2	(AG)6	12	61667808	61667819
chr1_10610	p3	(CCT)5	15	61679264	61679278
chr1_10611	p2	(TG)8	16	61681979	61681994
chr1_10612	p2	(TC)6	12	61682134	61682145
chr1_10613	p2	(AG)29	58	61682338	61682395
chr1_10614	p2	(TA)6	12	61685665	61685676
chr1_10615	p2	(AC)8	16	61687405	61687420
chr1_10616	c	cttacgagtgtatttgtttggctatatgttacacacagtaaataaacttcttttaa	137	61689146	61689282
chr1_10617	p2	(AC)11	22	61689490	61689511
chr1_10618	p2	(CT)6	12	61697602	61697613
chr1_10619	p2	(CA)22	44	61698780	61698823
chr1_10620	p2	(CA)7	14	61703617	61703630
chr1_10621	p2	(GT)6	12	61707688	61707699
chr1_10622	p2	(GA)6	12	61711722	61711733
chr1_10623	p2	(CT)6	12	61722369	61722380
chr1_10624	p2	(CT)8	16	61725142	61725157
chr1_10625	p2	(AC)6	12	61725686	61725697
chr1_10626	p2	(CT)15	30	61728390	61728419
chr1_10627	p2	(TG)6	12	61730805	61730816
chr1_10628	p2	(GT)17	34	61743200	61743233
chr1_10629	c	tgcccacttttgggttcctccatatgtggcataatggaaacctctcaacaggg	133	61747529	61747661
chr1_10630	p3	(TTA)6	18	61761160	61761177
chr1_10631	p3	(TTG)5	15	61769791	61769805
chr1_10632	p3	(ATT)5	15	61773141	61773155
chr1_10633	p3	(TTA)7	21	61774306	61774326
chr1_10634	p2	(AC)6	12	61788812	61788823
chr1_10635	p2	(TG)6	12	61794452	61794463
chr1_10636	p2	(TG)10	20	61800201	61800220
chr1_10637	p2	(AC)11	22	61803650	61803671
chr1_10638	p2	(TG)14	28	61804949	61804976
chr1_10639	p2	(TG)7	14	61810858	61810871
chr1_10640	p2	(GT)6	12	61811291	61811302
chr1_10641	p3	(ACC)5	15	61812156	61812170
chr1_10642	p4	(ATCC)5	20	61816124	61816143
chr1_10643	p2	(GT)16	32	61821011	61821042
chr1_10644	p2	(GT)18	36	61834640	61834675
chr1_10645	p2	(GT)18	36	61834868	61834903
chr1_10646	p2	(GT)17	34	61835790	61835823
chr1_10647	c	(TG)7(AG)8a(AG)6	43	61836006	61836048
chr1_10648	p2	(GT)12	24	61836182	61836205
chr1_10649	p2	(TG)14	28	61836400	61836427
chr1_10650	p2	(AC)7	14	61837736	61837749
chr1_10651	p2	(TG)6	12	61840737	61840748
chr1_10652	p2	(TG)21	42	61843829	61843870
chr1_10653	p2	(CA)9	18	61849791	61849808
chr1_10654	p2	(AC)10	20	61851535	61851554
chr1_10655	p3	(TAT)6	18	61855464	61855481
chr1_10656	p2	(TG)9	18	61864839	61864856

chr1_10657	p2	(TA)6	12	61871104	61871115
chr1_10658	c	(AC)11agacaatgcacaaaaacacacattaaacagt(CA)7	67	61884426	61884492
chr1_10659	p2	(GA)7	14	61885699	61885712
chr1_10660	c	(ATT)12aac(ATT)6	57	61886164	61886220
chr1_10661	p2	(TG)7	14	61888041	61888054
chr1_10662	p2	(CA)8	16	61903122	61903137
chr1_10663	p3	(AAG)6	18	61903936	61903953
chr1_10664	p2	(AC)7	14	61906667	61906680
chr1_10665	p2	(TG)6	12	61911510	61911521
chr1_10666	p3	(CTG)5	15	61916708	61916722
chr1_10667	p2	(AC)12	24	61920197	61920220
chr1_10668	p2	(CA)19	38	61920500	61920537
chr1_10669	p3	(GAG)5	15	61922759	61922773
chr1_10670	p2	(GT)6	12	61932220	61932231
chr1_10671	p2	(AG)7	14	61936112	61936125
chr1_10672	c	(TAT)16(TAG)8	72	61954177	61954248
chr1_10673	p2	(TG)8	16	61959461	61959476
chr1_10674	p3	(TTA)7	21	61962404	61962424
chr1_10675	p2	(AC)15	30	61966808	61966837
chr1_10676	p2	(GT)9	18	61974063	61974080
chr1_10677	c	(TAT)6cattattattattg(TTA)5	47	61983148	61983194
chr1_10678	p2	(TG)15	30	61984684	61984713
chr1_10679	p4	(ATTG)5	20	61986897	61986916
chr1_10680	p3	(AAT)5	15	61993688	61993702
chr1_10681	p2	(TA)10	20	62004496	62004515
chr1_10682	p2	(CA)15	30	62006690	62006719
chr1_10683	p3	(TAT)6	18	62015733	62015750
chr1_10684	p3	(TTA)5	15	62034772	62034786
chr1_10685	p2	(TG)6	12	62040707	62040718
chr1_10686	p2	(AT)6	12	62045926	62045937
chr1_10687	c	(AAC)6aataataatg(ATA)5	43	62056009	62056051
chr1_10688	p2	(CA)6	12	62074362	62074373
chr1_10689	p2	(CA)6	12	62082603	62082614
chr1_10690	p2	(CA)29	58	62089969	62090026
chr1_10691	p4	(TTCT)5	20	62091686	62091705
chr1_10692	p2	(TG)6	12	62095159	62095170
chr1_10693	p2	(TG)7	14	62099803	62099816
chr1_10694	p2	(AC)8	16	62102095	62102110
chr1_10695	c	ctctatctctctatctctctctctatctctctctatctctatctctctctctatctctc	106	62106195	62106300
chr1_10696	p2	(AC)7	14	62106602	62106615
chr1_10697	p4	(TACC)8	32	62106844	62106875
chr1_10698	p2	(CA)15	30	62107834	62107863
chr1_10699	p2	(TG)14	28	62109166	62109193
chr1_10700	p3	(CAT)5	15	62110400	62110414
chr1_10701	p3	(TCC)7	21	62112839	62112859
chr1_10702	p2	(CA)6	12	62116704	62116715
chr1_10703	p2	(AC)6	12	62117087	62117098
chr1_10704	p2	(AC)17	34	62117474	62117507
chr1_10705	p2	(AC)20	40	62121389	62121428
chr1_10706	p2	(AC)8	16	62126725	62126740
chr1_10707	p3	(TAT)5	15	62132987	62133001
chr1_10708	p2	(TG)9	18	62141229	62141246

chr1_10709	p2	(CA)14	28	62156839	62156866
chr1_10710	p4	(TTTA)6	24	62167952	62167975
chr1_10711	p3	(ATA)7	21	62204850	62204870
chr1_10712	p2	(TG)23	46	62207148	62207193
chr1_10713	p2	(TG)19	38	62207429	62207466
chr1_10714	p4	(CATT)5	20	62210171	62210190
chr1_10715	p2	(TA)6	12	62221905	62221916
chr1_10716	p3	(ATT)5	15	62222803	62222817
chr1_10717	p2	(GT)7	14	62241243	62241256
chr1_10718	p2	(TG)11	22	62254407	62254428
chr1_10719	c	(CA)25tacacct(AC)7	71	62255036	62255106
chr1_10720	p2	(AC)10	20	62267365	62267384
chr1_10721	p2	(TG)19	38	62268324	62268361
chr1_10722	p2	(TG)19	38	62269939	62269976
chr1_10723	p2	(GT)15	30	62284779	62284808
chr1_10724	p2	(TG)13	26	62284914	62284939
chr1_10725	p2	(AC)22	44	62285702	62285745
chr1_10726	p2	(GT)11	22	62287610	62287631
chr1_10727	c	caggctgtttggtacaaatgactaatgtcccggggtagtttgcctaagataa(	161	62288554	62288714
chr1_10728	p4	(ATTT)5	20	62295222	62295241
chr1_10729	p2	(CA)6	12	62296185	62296196
chr1_10730	p2	(TG)11	22	62310740	62310761
chr1_10731	p2	(GT)11	22	62329726	62329747
chr1_10732	p3	(TGA)5	15	62332697	62332711
chr1_10733	p2	(GT)19	38	62335647	62335684
chr1_10734	p2	(CA)9	18	62335825	62335842
chr1_10735	p3	(TAT)5	15	62337697	62337711
chr1_10736	p2	(TG)14	28	62338326	62338353
chr1_10737	c	(AC)10ag(AC)11	44	62344843	62344886
chr1_10738	p2	(TC)7	14	62360604	62360617
chr1_10739	c	(GT)20tattgtacttgctgtatag(TCCA)9	95	62365075	62365169
chr1_10740	p4	(CCAT)5	20	62371902	62371921
chr1_10741	p2	(CA)12	24	62377169	62377192
chr1_10742	p2	(AC)7	14	62390725	62390738
chr1_10743	p3	(GAT)5	15	62390965	62390979
chr1_10744	p3	(ATG)9	27	62391110	62391136
chr1_10745	p3	(GCT)6	18	62394355	62394372
chr1_10746	p4	(ATTT)5	20	62397876	62397895
chr1_10747	p2	(CT)7	14	62408870	62408883
chr1_10748	p2	(GA)6	12	62417279	62417290
chr1_10749	p2	(TC)6	12	62423217	62423228
chr1_10750	p2	(TG)6	12	62425027	62425038
chr1_10751	p2	(CA)17	34	62425380	62425413
chr1_10752	c	(TG)12(TA)6	36	62427159	62427194
chr1_10753	c	(TC)12(AC)8	40	62427965	62428004
chr1_10754	p2	(CT)13	26	62436327	62436352
chr1_10755	p2	(CT)6	12	62448575	62448586
chr1_10756	p2	(TA)6	12	62452115	62452126
chr1_10757	p2	(CA)7	14	62453113	62453126
chr1_10758	p3	(TTA)6	18	62466563	62466580
chr1_10759	p4	(ATTT)5	20	62476961	62476980
chr1_10760	p2	(CA)19	38	62477179	62477216

chr1_10761	p2	(AG)6	12	62485197	62485208
chr1_10762	p3	(ATA)8	24	62499100	62499123
chr1_10763	p2	(CA)18	36	62504570	62504605
chr1_10764	p2	(CT)6	12	62513949	62513960
chr1_10765	c	(AC)22atatacatac(AT)6	66	62516868	62516933
chr1_10766	p3	(AAG)7	21	62519038	62519058
chr1_10767	c	ctatgcttctatatgccaccattacacaccactaactttgtgtctcctctctccca	114	62535370	62535483
chr1_10768	p4	(ATTT)5	20	62542696	62542715
chr1_10769	p3	(CAC)6	18	62557684	62557701
chr1_10770	p4	(ACAA)5	20	62563303	62563322
chr1_10771	p3	(TAA)14	42	62563551	62563592
chr1_10772	c	(GT)10(GA)8	36	62584924	62584959
chr1_10773	p2	(TG)6	12	62599423	62599434
chr1_10774	p2	(AC)7	14	62600733	62600746
chr1_10775	p3	(ATA)5	15	62601553	62601567
chr1_10776	p2	(CA)6	12	62601930	62601941
chr1_10777	p2	(GT)9	18	62607628	62607645
chr1_10778	p2	(AC)7	14	62609300	62609313
chr1_10779	c	gaagggtgcatacttgaagagatatgttcatgtattttatgcaggctactgcag	95	62611307	62611401
chr1_10780	p2	(AC)7	14	62618243	62618256
chr1_10781	p2	(TG)12	24	62618424	62618447
chr1_10782	p2	(AC)8	16	62618759	62618774
chr1_10783	p2	(GT)6	12	62621122	62621133
chr1_10784	p2	(AC)18	36	62622216	62622251
chr1_10785	p2	(TG)9	18	62623723	62623740
chr1_10786	p2	(AC)15	30	62642543	62642572
chr1_10787	p2	(GT)6	12	62647016	62647027
chr1_10788	p4	(CATC)6	24	62647685	62647708
chr1_10789	p2	(TG)8	16	62651553	62651568
chr1_10790	p3	(ATA)5	15	62658441	62658455
chr1_10791	p2	(AC)15	30	62659418	62659447
chr1_10792	p2	(AC)6	12	62671296	62671307
chr1_10793	p2	(CA)8	16	62673753	62673768
chr1_10794	c	(TG)17g(GT)6	47	62676537	62676583
chr1_10795	p2	(GT)26	52	62684514	62684565
chr1_10796	c	(GT)14gagacttgcttcctatctgtatgtgcgccactg(GA)10	81	62686183	62686263
chr1_10797	p2	(TG)6	12	62690947	62690958
chr1_10798	p2	(GT)8	16	62694278	62694293
chr1_10799	p2	(AC)6	12	62698814	62698825
chr1_10800	p2	(TG)7	14	62705419	62705432
chr1_10801	p2	(AC)14	28	62708811	62708838
chr1_10802	p2	(AC)6	12	62710806	62710817
chr1_10803	p3	(TAT)5	15	62724805	62724819
chr1_10804	p2	(GT)28	56	62731924	62731979
chr1_10805	p2	(GA)7	14	62732753	62732766
chr1_10806	p5	(AGGCA)5	25	62735497	62735521
chr1_10807	p3	(TTA)8	24	62745493	62745516
chr1_10808	p4	(TCCA)6	24	62763890	62763913
chr1_10809	p2	(TG)6	12	62767516	62767527
chr1_10810	p2	(TG)21	42	62769225	62769266
chr1_10811	c	cgctgcagctgtgagccacaatcagggagccagcacctgggggagag	100	62787000	62787099
chr1_10812	p2	(CA)13	26	62789034	62789059

chr1_10813	c	gtcccctcgtagtaggaaaaacaagtaggggtgtgtgt(GA)6gcagtcc	204	62801313	62801516
chr1_10814	p2	(AG)7	14	62806019	62806032
chr1_10815	p2	(TG)6	12	62818475	62818486
chr1_10816	p2	(GT)25	50	62819493	62819542
chr1_10817	p2	(CT)6	12	62822199	62822210
chr1_10818	p2	(CA)19	38	62822718	62822755
chr1_10819	p2	(TG)6	12	62825234	62825245
chr1_10820	p2	(TC)19	38	62827780	62827817
chr1_10821	p2	(GT)19	38	62843611	62843648
chr1_10822	p2	(TG)10	20	62848411	62848430
chr1_10823	c	(GT)16(AT)8	48	62854854	62854901
chr1_10824	p2	(TG)17	34	62867460	62867493
chr1_10825	p3	(TTC)5	15	62870504	62870518
chr1_10826	p2	(TG)8	16	62877660	62877675
chr1_10827	p2	(CT)20	40	62883988	62884027
chr1_10828	p2	(GT)10	20	62893792	62893811
chr1_10829	p2	(AC)11	22	62910363	62910384
chr1_10830	p2	(GT)7	14	62923209	62923222
chr1_10831	p2	(AC)12	24	62959825	62959848
chr1_10832	p4	(ATGG)7	28	62983838	62983865
chr1_10833	p4	(ATGG)5	20	63009789	63009808
chr1_10834	p2	(TA)6	12	63027277	63027288
chr1_10835	p2	(AT)7	14	63041063	63041076
chr1_10836	p2	(AC)11	22	63046282	63046303
chr1_10837	p3	(CAC)5	15	63058288	63058302
chr1_10838	p2	(TG)6	12	63058461	63058472
chr1_10839	p3	(ATA)5	15	63064559	63064573
chr1_10840	p4	(TTGA)5	20	63071662	63071681
chr1_10841	p3	(TTG)9	27	63078180	63078206
chr1_10842	p5	(CTCCT)19	95	63080562	63080656
chr1_10843	c	A)6caaaaac(AAG)9t(AGA)14agtagtagtagaagaag(AG	244	63085691	63085934
chr1_10844	p2	(TG)6	12	63099645	63099656
chr1_10845	p2	(AT)8	16	63100516	63100531
chr1_10846	p3	(ATT)7	21	63103277	63103297
chr1_10847	p2	(TG)6	12	63108393	63108404
chr1_10848	p2	(TG)13	26	63108786	63108811
chr1_10849	p2	(CA)23	46	63109583	63109628
chr1_10850	p2	(TC)12	24	63111985	63112008
chr1_10851	p2	(TG)8	16	63112961	63112976
chr1_10852	p2	(AC)10	20	63115247	63115266
chr1_10853	p3	(AAT)5	15	63124676	63124690
chr1_10854	p2	(AC)6	12	63125414	63125425
chr1_10855	p2	(CA)6	12	63147383	63147394
chr1_10856	p2	(GA)6	12	63156270	63156281
chr1_10857	p2	(AC)6	12	63157676	63157687
chr1_10858	p2	(GA)6	12	63164694	63164705
chr1_10859	p2	(CA)15	30	63166416	63166445
chr1_10860	p2	(AC)14	28	63169903	63169930
chr1_10861	c	(AC)6atacatacac(AT)14(AC)8	66	63170498	63170563
chr1_10862	p2	(CA)11	22	63198929	63198950
chr1_10863	p3	(AGG)5	15	63210791	63210805
chr1_10864	p4	(TCCA)5	20	63215551	63215570

chr1_10865	p2	(AC)6	12	63220728	63220739
chr1_10866	p2	(TG)15	30	63226095	63226124
chr1_10867	p2	(GT)6	12	63230575	63230586
chr1_10868	p4	(AAAT)5	20	63241365	63241384
chr1_10869	p2	(AG)6	12	63248747	63248758
chr1_10870	p2	(GT)6	12	63251443	63251454
chr1_10871	p3	(CAG)7	21	63259035	63259055
chr1_10872	c	(AG)16aaggaaata(TC)13	67	63259585	63259651
chr1_10873	p3	(TTA)6	18	63270354	63270371
chr1_10874	p2	(AC)6	12	63279234	63279245
chr1_10875	p4	(ATGG)5	20	63285401	63285420
chr1_10876	p3	(ACA)5	15	63287666	63287680
chr1_10877	p3	(CTT)5	15	63290270	63290284
chr1_10878	p3	(TTG)5	15	63301603	63301617
chr1_10879	c	(CT)11cactcactcact(CA)15(GA)6	76	63302505	63302580
chr1_10880	c	(CT)11cactcactcact(CA)15(GA)6	76	63303954	63304029
chr1_10881	c*	(CT)8(CTCA)6*(CA)14*(GA)6	76	63305366	63305441
chr1_10882	p2	(CT)6	12	63307962	63307973
chr1_10883	p2	(TG)7	14	63319172	63319185
chr1_10884	p2	(AC)6	12	63323364	63323375
chr1_10885	p2	(GA)8	16	63325392	63325407
chr1_10886	p2	(AT)6	12	63326922	63326933
chr1_10887	p2	(TG)6	12	63335129	63335140
chr1_10888	p3	(TTA)7	21	63335652	63335672
chr1_10889	p2	(TG)14	28	63337568	63337595
chr1_10890	p2	(AC)7	14	63341504	63341517
chr1_10891	p2	(TG)8	16	63347711	63347726
chr1_10892	p2	(TG)24	48	63348323	63348370
chr1_10893	p2	(CA)6	12	63366162	63366173
chr1_10894	p3	(TTA)6	18	63368080	63368097
chr1_10895	p2	(GA)8	16	63370113	63370128
chr1_10896	c	(GCA)7acaaggg(CAA)5	43	63377342	63377384
chr1_10897	c	(GCA)7acaaggg(CAA)5	43	63378476	63378518
chr1_10898	p3	(GAG)9	27	63379569	63379595
chr1_10899	p3	(TTA)6	18	63404281	63404298
chr1_10900	p2	(TG)6	12	63415652	63415663
chr1_10901	p4	(GATG)5	20	63437684	63437703
chr1_10902	p2	(TG)15	30	63439635	63439664
chr1_10903	p2	(TG)6	12	63440149	63440160
chr1_10904	p2	(TG)6	12	63445517	63445528
chr1_10905	p3	(AGC)7	21	63451076	63451096
chr1_10906	p2	(TG)6	12	63454119	63454130
chr1_10907	p3	(ATT)5	15	63457437	63457451
chr1_10908	p3	(TTG)9	27	63464707	63464733
chr1_10909	p3	(CAG)5	15	63465924	63465938
chr1_10910	p3	(GGT)5	15	63483461	63483475
chr1_10911	p2	(CA)26	52	63492468	63492519
chr1_10912	p2	(TG)6	12	63495385	63495396
chr1_10913	p2	(TA)9	18	63497286	63497303
chr1_10914	c	(TG)10(AG)6	32	63501047	63501078
chr1_10915	p2	(CA)11	22	63546809	63546830
chr1_10916	p2	(GT)8	16	63560454	63560469

chr1_10917	p2	(AG)6	12	63561665	63561676
chr1_10918	p2	(CT)6	12	63572349	63572360
chr1_10919	p3	(CAA)6	18	63577165	63577182
chr1_10920	p2	(GT)12	24	63577548	63577571
chr1_10921	p3	(TCT)5	15	63584741	63584755
chr1_10922	p5	(ATTTG)5	25	63586063	63586087
chr1_10923	c	(GA)6aa(AG)16	46	63593979	63594024
chr1_10924	p2	(AC)13	26	63631210	63631235
chr1_10925	p2	(CA)20	40	63631438	63631477
chr1_10926	p2	(GT)6	12	63634688	63634699
chr1_10927	p2	(AC)10	20	63641982	63642001
chr1_10928	p2	(TG)22	44	63644922	63644965
chr1_10929	p2	(TA)6	12	63685060	63685071
chr1_10930	p2	(CA)6	12	63685347	63685358
chr1_10931	p3	(TGC)5	15	63690151	63690165
chr1_10932	p2	(CA)9	18	63711828	63711845
chr1_10933	p2	(CA)24	48	63721559	63721606
chr1_10934	p2	(CA)6	12	63728454	63728465
chr1_10935	p2	(AC)11	22	63744008	63744029
chr1_10936	p2	(AC)21	42	63748502	63748543
chr1_10937	p2	(TC)9	18	63770835	63770852
chr1_10938	p4	(ATGG)5	20	63787256	63787275
chr1_10939	c	ctccacctcaattactggcaattgagcaatggaatgtgggaaattagcacta	101	63788001	63788101
chr1_10940	p2	(CA)7	14	63796421	63796434
chr1_10941	p2	(TC)6	12	63796721	63796732
chr1_10942	p2	(AC)6	12	63803404	63803415
chr1_10943	p2	(AC)7	14	63808446	63808459
chr1_10944	p3	(TTA)5	15	63816531	63816545
chr1_10945	p3	(AGT)5	15	63834882	63834896
chr1_10946	p2	(TG)6	12	63836924	63836935
chr1_10947	p2	(GT)6	12	63837933	63837944
chr1_10948	p3	(CAC)5	15	63845117	63845131
chr1_10949	p2	(AC)17	34	63845807	63845840
chr1_10950	p2	(AT)6	12	63851312	63851323
chr1_10951	p2	(CA)18	36	63856208	63856243
chr1_10952	p2	(GT)7	14	63861554	63861567
chr1_10953	p3	(TTA)9	27	63895931	63895957
chr1_10954	p2	(CT)6	12	63896837	63896848
chr1_10955	p2	(AG)6	12	63908696	63908707
chr1_10956	p2	(GA)14	28	63909588	63909615
chr1_10957	p2	(CT)13	26	63912818	63912843
chr1_10958	p3	(TAT)6	18	63929349	63929366
chr1_10959	p2	(CA)6	12	63937430	63937441
chr1_10960	p2	(CA)18	36	63939186	63939221
chr1_10961	p2	(AC)6	12	63939861	63939872
chr1_10962	p2	(AC)6	12	63958090	63958101
chr1_10963	c	(AC)9atatatgtac(AT)6(GT)10	60	63964842	63964901
chr1_10964	c	(CT)6gactctcactttgtctcctacacatttaaatca(AC)7	59	63969560	63969618
chr1_10965	p3	(TAT)5	15	63971022	63971036
chr1_10966	p2	(GA)7	14	63981057	63981070
chr1_10967	p3	(GAG)6	18	63988024	63988041
chr1_10968	p2	(CA)7	14	63997437	63997450

chr1_10969	p2	(GT)8	16	63998252	63998267
chr1_10970	p2	(GT)6	12	64011104	64011115
chr1_10971	p3	(TTG)14	42	64027948	64027989
chr1_10972	p2	(AC)6	12	64030097	64030108
chr1_10973	p2	(TC)6	12	64030843	64030854
chr1_10974	p2	(TG)9	18	64036733	64036750
chr1_10975	c	(GT)7(GA)6gtgagtgtgtgt(GA)6	50	64044359	64044408
chr1_10976	c	(GT)12at(GA)6	38	64046128	64046165
chr1_10977	p2	(AC)30	60	64047055	64047114
chr1_10978	p2	(CA)9	18	64048127	64048144
chr1_10979	p2	(GT)8	16	64074449	64074464
chr1_10980	p2	(GT)10	20	64089669	64089688
chr1_10981	p2	(GT)9	18	64090355	64090372
chr1_10982	p2	(TA)11	22	64093107	64093128
chr1_10983	p2	(AC)6	12	64096213	64096224
chr1_10984	p2	(TG)6	12	64104478	64104489
chr1_10985	p2	(AT)6	12	64111222	64111233
chr1_10986	c	C)9(AC)17atacatacacacagacacaactacacacctacacat(AC	100	64117494	64117593
chr1_10987	c	(TC)10ttt(CA)16	55	64118002	64118056
chr1_10988	p2	(AC)6	12	64119002	64119013
chr1_10989	p2	(GT)16	32	64119962	64119993
chr1_10990	c	(CT)8(CA)7	30	64122267	64122296
chr1_10991	c	(TG)7(AG)7tgagagaaagagac(AG)6	54	64124785	64124838
chr1_10992	p2	(AT)6	12	64129101	64129112
chr1_10993	p3	(TAC)6	18	64130776	64130793
chr1_10994	p2	(TA)9	18	64133985	64134002
chr1_10995	c	:ataccgagtgtttatatatatacatatatatatacacacac(AT)17acaca	117	64149423	64149539
chr1_10996	p2	(GT)15	30	64160028	64160057
chr1_10997	p2	(CA)10	20	64162641	64162660
chr1_10998	c	gtagcttggggtgcctgacttgcacggtttgctaccagagggacagcaaac	131	64164661	64164791
chr1_10999	p3	(ACA)5	15	64179658	64179672
chr1_11000	p2	(AG)6	12	64183991	64184002
chr1_11001	p4	(AATC)5	20	64187923	64187942
chr1_11002	p2	(GT)10	20	64198936	64198955
chr1_11003	p3	(TAA)5	15	64200327	64200341
chr1_11004	p4	(AGCT)5	20	64201408	64201427
chr1_11005	p2	(TG)36	72	64204313	64204384
chr1_11006	p2	(GA)9	18	64206764	64206781
chr1_11007	p2	(TG)20	40	64213920	64213959
chr1_11008	p2	(AC)11	22	64214180	64214201
chr1_11009	p2	(TG)33	66	64218874	64218939
chr1_11010	p2	(TG)6	12	64223660	64223671
chr1_11011	p2	(AC)10	20	64223847	64223866
chr1_11012	p2	(GA)10	20	64225461	64225480
chr1_11013	p2	(AC)6	12	64238499	64238510
chr1_11014	p2	(CA)26	52	64243494	64243545
chr1_11015	p2	(AC)15	30	64245955	64245984
chr1_11016	c	(CA)7gagg(GA)6	30	64251509	64251538
chr1_11017	p2	(AC)9	18	64253881	64253898
chr1_11018	p2	(CA)19	38	64268789	64268826
chr1_11019	p3	(GCT)6	18	64273774	64273791
chr1_11020	c	ıcacctctcagctgctcccattaacaccacctgtttccactcctgcaatcacc:	113	64280100	64280212

chr1_11021	p2	(AC)11	22	64286726	64286747
chr1_11022	p3	(CTT)7	21	64287190	64287210
chr1_11023	p3	(ATT)5	15	64289449	64289463
chr1_11024	p2	(TC)6	12	64295151	64295162
chr1_11025	p2	(AC)27	54	64302828	64302881
chr1_11026	p2	(CA)26	52	64304475	64304526
chr1_11027	p3	(CTA)7	21	64308798	64308818
chr1_11028	p2	(CA)8	16	64308934	64308949
chr1_11029	p2	(TA)6	12	64312121	64312132
chr1_11030	p2	(GT)12	24	64318546	64318569
chr1_11031	p2	(TG)6	12	64320086	64320097
chr1_11032	p3	(TTA)5	15	64323683	64323697
chr1_11033	p2	(GT)17	34	64329599	64329632
chr1_11034	p2	(CA)16	32	64334567	64334598
chr1_11035	p2	(GT)6	12	64340687	64340698
chr1_11036	p2	(AC)7	14	64343470	64343483
chr1_11037	p2	(CA)6	12	64362992	64363003
chr1_11038	p2	(TG)10	20	64367341	64367360
chr1_11039	p2	(CA)6	12	64376565	64376576
chr1_11040	p2	(TG)8	16	64390698	64390713
chr1_11041	p2	(CA)14	28	64402676	64402703
chr1_11042	p2	(AG)14	28	64407602	64407629
chr1_11043	p3	(ATT)5	15	64415875	64415889
chr1_11044	p2	(TA)6	12	64417758	64417769
chr1_11045	p2	(TG)8	16	64427023	64427038
chr1_11046	p2	(CA)6	12	64432148	64432159
chr1_11047	p2	(TC)7	14	64439272	64439285
chr1_11048	p2	(TA)7	14	64450389	64450402
chr1_11049	p2	(AC)8	16	64451147	64451162
chr1_11050	p2	(GT)22	44	64454593	64454636
chr1_11051	p2	(CA)10	20	64468939	64468958
chr1_11052	p3	(AAG)9	27	64482994	64483020
chr1_11053	p2	(CA)19	38	64484916	64484953
chr1_11054	p2	(GT)17	34	64489305	64489338
chr1_11055	p2	(GA)7	14	64489915	64489928
chr1_11056	c	atgcacagaagcaccatttccctccaactctctctcccttctcagtttcttttac	118	64490070	64490187
chr1_11057	c	(TAC)12tatgac(TAA)6	60	64512261	64512320
chr1_11058	p3	(CAA)10	30	64512664	64512693
chr1_11059	p2	(GT)6	12	64520033	64520044
chr1_11060	p2	(GT)7	14	64520291	64520304
chr1_11061	p2	(AT)10	20	64520830	64520849
chr1_11062	p2	(AT)8	16	64521968	64521983
chr1_11063	p2	(GT)7	14	64523128	64523141
chr1_11064	p3	(GTT)5	15	64523644	64523658
chr1_11065	p2	(CT)6	12	64525021	64525032
chr1_11066	p2	(TG)18	36	64533673	64533708
chr1_11067	p2	(TC)8	16	64536991	64537006
chr1_11068	p2	(GT)12	24	64537247	64537270
chr1_11069	p2	(AC)6	12	64538091	64538102
chr1_11070	p2	(AT)6	12	64539677	64539688
chr1_11071	p5	(AAATC)6	30	64541046	64541075
chr1_11072	p2	(TG)10	20	64552780	64552799

chr1_11073	p2	(GT)9	18	64554275	64554292
chr1_11074	p2	(GA)17	34	64555633	64555666
chr1_11075	p2	(CA)7	14	64557430	64557443
chr1_11076	p2	(CA)9	18	64557825	64557842
chr1_11077	p2	(GA)6	12	64558080	64558091
chr1_11078	p2	(AT)9	18	64559771	64559788
chr1_11079	p2	(AC)9	18	64561569	64561586
chr1_11080	p2	(GT)6	12	64562969	64562980
chr1_11081	p2	(TG)15	30	64564133	64564162
chr1_11082	c	aggacgtccacttctattaaaacagtttttactggccaaagaagacacatgg	130	64565310	64565439
chr1_11083	p3	(CTC)5	15	64566249	64566263
chr1_11084	p2	(TC)13	26	64567036	64567061
chr1_11085	c	atgtgtgtgttggagcattgtacggaccagtttggattt(TG)9tcatagtag	193	64567469	64567661
chr1_11086	p2	(TG)10	20	64568358	64568377
chr1_11087	p2	(GT)7	14	64568975	64568988
chr1_11088	p2	(TG)14	28	64580058	64580085
chr1_11089	p2	(CA)12	24	64587018	64587041
chr1_11090	p2	(CA)6	12	64587182	64587193
chr1_11091	p2	(AC)13	26	64587638	64587663
chr1_11092	p2	(AC)7	14	64591195	64591208
chr1_11093	p2	(AC)20	40	64592314	64592353
chr1_11094	p2	(AT)6	12	64595767	64595778
chr1_11095	p2	(AG)7	14	64596475	64596488
chr1_11096	p2	(GT)22	44	64596767	64596810
chr1_11097	p2	(CA)6	12	64597774	64597785
chr1_11098	c	(CA)6ccctgttgttctgtc(TG)7	41	64601618	64601658
chr1_11099	p2	(CA)6	12	64602912	64602923
chr1_11100	c	(CAG)7ctgtgcggtggttaggatttactgagca(AAAC)6	73	64603079	64603151
chr1_11101	c	(GTT)5tactgccttcaatgagaacgagaataaaaaggacac(AG)10	71	64603763	64603833
chr1_11102	p3	(TAT)5	15	64604154	64604168
chr1_11103	p2	(TG)15	30	64605361	64605390
chr1_11104	p2	(AC)7	14	64605707	64605720
chr1_11105	p2	(CA)6	12	64608491	64608502
chr1_11106	p2	(CA)14	28	64610655	64610682
chr1_11107	p2	(AG)6	12	64611436	64611447
chr1_11108	p2	(CT)8	16	64612283	64612298
chr1_11109	p2	(TC)11	22	64615133	64615154
chr1_11110	p2	(GT)25	50	64619889	64619938
chr1_11111	p2	(GT)13	26	64626356	64626381
chr1_11112	p2	(CA)7	14	64632622	64632635
chr1_11113	p2	(AC)20	40	64632885	64632924
chr1_11114	p2	(CT)8	16	64640463	64640478
chr1_11115	p2	(GT)10	20	64678951	64678970
chr1_11116	p3	(GGT)5	15	64679652	64679666
chr1_11117	p2	(TG)13	26	64680339	64680364
chr1_11118	c	(GT)10gggtgg(GT)7	40	64680867	64680906
chr1_11119	p2	(GT)12	24	64682431	64682454
chr1_11120	p3	(CTG)6	18	64690635	64690652
chr1_11121	c	atgttcagcatatgcatttgtggatgtcctgtttttgttgtgtgataacagttga	125	64690908	64691032
chr1_11122	p2	(TG)6	12	64693460	64693471
chr1_11123	p2	(TG)6	12	64696086	64696097
chr1_11124	p2	(AG)10	20	64697987	64698006

chr1_11125	p2	(GT)21	42	64703748	64703789
chr1_11126	p2	(GA)6	12	64704321	64704332
chr1_11127	p3	(TGA)5	15	64708045	64708059
chr1_11128	p2	(AC)8	16	64709888	64709903
chr1_11129	p3	(AAT)6	18	64714750	64714767
chr1_11130	p2	(TG)6	12	64736161	64736172
chr1_11131	p2	(CA)10	20	64745942	64745961
chr1_11132	p2	(TG)9	18	64747965	64747982
chr1_11133	p2	(GT)7	14	64753970	64753983
chr1_11134	p2	(GT)15	30	64754121	64754150
chr1_11135	p2	(TG)10	20	64754274	64754293
chr1_11136	p2	(TA)8	16	64760801	64760816
chr1_11137	p2	(TA)6	12	64760951	64760962
chr1_11138	p2	(AC)11	22	64761194	64761215
chr1_11139	p2	(GA)7	14	64761606	64761619
chr1_11140	p2	(GA)7	14	64772430	64772443
chr1_11141	p2	(TG)11	22	64777530	64777551
chr1_11142	p4	(CATC)5	20	64783699	64783718
chr1_11143	c	(CT)9gcctgct(TC)6	37	64790391	64790427
chr1_11144	p2	(CA)20	40	64791187	64791226
chr1_11145	p2	(AC)8	16	64791539	64791554
chr1_11146	p2	(TG)7	14	64792709	64792722
chr1_11147	p2	(CA)7	14	64793351	64793364
chr1_11148	p2	(TA)9	18	64796012	64796029
chr1_11149	p2	(TG)6	12	64797555	64797566
chr1_11150	p2	(TG)6	12	64798264	64798275
chr1_11151	p2	(TG)18	36	64800260	64800295
chr1_11152	p3	(ATT)6	18	64802334	64802351
chr1_11153	p2	(GA)6	12	64803397	64803408
chr1_11154	p2	(AG)6	12	64807230	64807241
chr1_11155	p2	(AT)7	14	64808900	64808913
chr1_11156	p2	(TG)7	14	64816477	64816490
chr1_11157	p2	(CA)21	42	64818236	64818277
chr1_11158	p2	(CA)16	32	64819571	64819602
chr1_11159	p4	(AGGG)6	24	64830830	64830853
chr1_11160	p3	(TAA)5	15	64831462	64831476
chr1_11161	c	AC)10tacatacatgacctgtaacttgtccagcattgcagcgtcta(AC)	75	64837728	64837802
chr1_11162	p2	(CA)6	12	64840342	64840353
chr1_11163	p2	(AC)6	12	64850869	64850880
chr1_11164	p3	(AAG)5	15	64856862	64856876
chr1_11165	p3	(TTC)7	21	64859624	64859644
chr1_11166	p3	(ATT)5	15	64868030	64868044
chr1_11167	p2	(GT)21	42	64876507	64876548
chr1_11168	p2	(GA)11	22	64880931	64880952
chr1_11169	p2	(GT)6	12	64888223	64888234
chr1_11170	c	(TG)6(TA)10(CA)11	54	64891428	64891481
chr1_11171	c	(TG)11(TA)10	42	64896109	64896150
chr1_11172	p2	(AC)9	18	64898868	64898885
chr1_11173	p2	(CT)7	14	64901824	64901837
chr1_11174	p2	(AC)8	16	64904015	64904030
chr1_11175	p2	(TG)6	12	64905008	64905019
chr1_11176	p2	(TG)7	14	64960188	64960201

chr1_11177	c	(AG)7ctagatg(GT)11	43	64966441	64966483
chr1_11178	p2	(TA)10	20	64973551	64973570
chr1_11179	p2	(GA)7	14	64978658	64978671
chr1_11180	p2	(GT)6	12	64980749	64980760
chr1_11181	p2	(CT)6	12	64988619	64988630
chr1_11182	c	(AC)6g(CA)7	27	64989633	64989659
chr1_11183	p2	(AC)10	20	64999199	64999218
chr1_11184	p2	(GT)10	20	65025154	65025173
chr1_11185	p4	(GATG)7	28	65027716	65027743
chr1_11186	p2	(GA)7	14	65028248	65028261
chr1_11187	c	agatgctgccctgaatgtgatgcataggattgctgcattcaatgggggctgg	99	65030743	65030841
chr1_11188	p5	(TGATT)5	25	65038581	65038605
chr1_11189	p3	(TTA)8	24	65061843	65061866
chr1_11190	p3	(TAT)7	21	65073788	65073808
chr1_11191	p2	(TG)11	22	65079598	65079619
chr1_11192	p2	(AC)17	34	65086816	65086849
chr1_11193	c	(ATA)6atcatgat(AAC)6	44	65092959	65093002
chr1_11194	p2	(AT)7	14	65120077	65120090
chr1_11195	c	(AAT)6(TAT)6	36	65121521	65121556
chr1_11196	p2	(TG)8	16	65133574	65133589
chr1_11197	p2	(AC)6	12	65155434	65155445
chr1_11198	p4	(TATC)16	64	65167105	65167168
chr1_11199	p2	(AT)6	12	65190855	65190866
chr1_11200	p2	(TG)7	14	65195097	65195110
chr1_11201	p2	(GT)8	16	65197540	65197555
chr1_11202	p4	(GATA)5	20	65208804	65208823
chr1_11203	p4	(TAGG)5	20	65218520	65218539
chr1_11204	p2	(AT)6	12	65234755	65234766
chr1_11205	p2	(CA)6	12	65236754	65236765
chr1_11206	p2	(AT)8	16	65259458	65259473
chr1_11207	p2	(TG)18	36	65272762	65272797
chr1_11208	c	nnnnnnnnnnnnnnnnnnnnnnnnnnnnnnnnnnnnnnnnnnn	143	65274359	65274501
chr1_11209	c	16tatatatatgtatatatatgtgtatgtatgtatgtgtatatacttatatgtgta(	98	65285982	65286079
chr1_11210	p2	(TG)15	30	65287627	65287656
chr1_11211	p2	(GT)10	20	65290844	65290863
chr1_11212	p2	(GT)11	22	65291801	65291822
chr1_11213	p2	(GT)11	22	65292695	65292716
chr1_11214	p2	(TG)7	14	65294806	65294819
chr1_11215	p2	(TG)22	44	65296213	65296256
chr1_11216	c	cc(TG)15gcacttgagagggtaaatctgtcagaaatggaaagtggtctc	170	65296397	65296566
chr1_11217	p2	(GA)9	18	65297007	65297024
chr1_11218	p2	(AC)12	24	65299602	65299625
chr1_11219	p4	(AAAC)6	24	65307026	65307049
chr1_11220	p2	(AC)16	32	65309788	65309819
chr1_11221	p2	(TG)17	34	65311302	65311335
chr1_11222	c	(AC)6atgcacactg(CT)6	34	65312574	65312607
chr1_11223	p2	(CT)8	16	65312878	65312893
chr1_11224	p2	(TG)8	16	65313663	65313678
chr1_11225	p2	(TG)7	14	65320980	65320993
chr1_11226	p2	(TG)10	20	65321402	65321421
chr1_11227	p4	(GAAA)7	28	65326913	65326940
chr1_11228	p3	(CTC)6	18	65327637	65327654

chr1_11229	c	(GT)7ga(GT)8	32	65334520	65334551
chr1_11230	p2	(AC)9	18	65344742	65344759
chr1_11231	p2	(TG)7	14	65362186	65362199
chr1_11232	p3	(CCT)5	15	65368090	65368104
chr1_11233	p4	(TTGA)6	24	65378107	65378130
chr1_11234	p3	(TTG)5	15	65380213	65380227
chr1_11235	p2	(CA)7	14	65382226	65382239
chr1_11236	p2	(TC)8	16	65388963	65388978
chr1_11237	p2	(TG)6	12	65389109	65389120
chr1_11238	p2	(TG)6	12	65389586	65389597
chr1_11239	p2	(AC)8	16	65393866	65393881
chr1_11240	p2	(AC)8	16	65396995	65397010
chr1_11241	p2	(AC)7	14	65397316	65397329
chr1_11242	p2	(TG)9	18	65399347	65399364
chr1_11243	p2	(AC)6	12	65402701	65402712
chr1_11244	p2	(AC)6	12	65403094	65403105
chr1_11245	p2	(TC)7	14	65407928	65407941
chr1_11246	p2	(GA)10	20	65409272	65409291
chr1_11247	c	(TG)16(AG)10	52	65412552	65412603
chr1_11248	p2	(TG)16	32	65420138	65420169
chr1_11249	p2	(CT)9	18	65421282	65421299
chr1_11250	p2	(TG)14	28	65424902	65424929
chr1_11251	c	gtatatgtatgtgggtacaaaaggcagagagtcacaaaattcaaaggatgc	130	65431108	65431237
chr1_11252	p2	(TC)7	14	65431400	65431413
chr1_11253	p2	(TA)6	12	65451505	65451516
chr1_11254	p2	(TG)21	42	65459439	65459480
chr1_11255	p2	(GT)26	52	65460141	65460192
chr1_11256	p2	(GA)7	14	65466637	65466650
chr1_11257	p2	(TG)8	16	65466798	65466813
chr1_11258	p2	(AC)12	24	65476354	65476377
chr1_11259	p2	(GA)11	22	65478829	65478850
chr1_11260	p2	(AT)8	16	65482809	65482824
chr1_11261	p2	(TG)8	16	65507863	65507878
chr1_11262	p2	(CA)12	24	65519491	65519514
chr1_11263	p2	(AC)7	14	65521453	65521466
chr1_11264	p2	(CA)14	28	65523408	65523435
chr1_11265	p3	(CAA)5	15	65524933	65524947
chr1_11266	p2	(TG)12	24	65531547	65531570
chr1_11267	p2	(GT)7	14	65534625	65534638
chr1_11268	p2	(AC)16	32	65542608	65542639
chr1_11269	p2	(GT)22	44	65542923	65542966
chr1_11270	p2	(AC)11	22	65543669	65543690
chr1_11271	p2	(TG)13	26	65545435	65545460
chr1_11272	p2	(CT)6	12	65549359	65549370
chr1_11273	p2	(AC)6	12	65556817	65556828
chr1_11274	p2	(TG)21	42	65558800	65558841
chr1_11275	c	(TC)8(AC)24	64	65564739	65564802
chr1_11276	p2	(CA)22	44	65566279	65566322
chr1_11277	p2	(AC)15	30	65570760	65570789
chr1_11278	p2	(AC)16	32	65570974	65571005
chr1_11279	p2	(TC)7	14	65581699	65581712
chr1_11280	c	cagtatttatgatggcggttgatttgactcctcggttcagaagaaggagcaga	196	65597497	65597692

chr1_11281	c	(GT)10t(TG)10	41	65607473	65607513
chr1_11282	p3	(GAG)8	24	65623847	65623870
chr1_11283	p3	(AGT)5	15	65625602	65625616
chr1_11284	p2	(GT)7	14	65644123	65644136
chr1_11285	p2	(TG)8	16	65656465	65656480
chr1_11286	p2	(AG)6	12	65656692	65656703
chr1_11287	p2	(AC)33	66	65657107	65657172
chr1_11288	p2	(CA)8	16	65678896	65678911
chr1_11289	p2	(GT)8	16	65684229	65684244
chr1_11290	p2	(AT)6	12	65686992	65687003
chr1_11291	p2	(AC)21	42	65692684	65692725
chr1_11292	p2	(AG)9	18	65694090	65694107
chr1_11293	p2	(AC)6	12	65694359	65694370
chr1_11294	p2	(TG)6	12	65709936	65709947
chr1_11295	p2	(TG)6	12	65710355	65710366
chr1_11296	c	(CTT)6cactcccctatcctct(CTC)5	49	65711711	65711759
chr1_11297	p3	(AAT)5	15	65715760	65715774
chr1_11298	p2	(TG)20	40	65725389	65725428
chr1_11299	p2	(AC)15	30	65728892	65728921
chr1_11300	c	(GT)13(GA)10	46	65733561	65733606
chr1_11301	p2	(TG)12	24	65734175	65734198
chr1_11302	p2	(TG)6	12	65737525	65737536
chr1_11303	p2	(TG)7	14	65738599	65738612
chr1_11304	p3	(CAC)5	15	65742383	65742397
chr1_11305	p2	(TG)15	30	65745411	65745440
chr1_11306	p2	(AT)7	14	65749471	65749484
chr1_11307	p2	(TG)8	16	65751241	65751256
chr1_11308	p2	(GT)7	14	65751526	65751539
chr1_11309	p2	(TC)7	14	65753774	65753787
chr1_11310	p2	(GT)6	12	65757279	65757290
chr1_11311	p2	(TG)8	16	65761799	65761814
chr1_11312	p3	(GAG)5	15	65768723	65768737
chr1_11313	p2	(TG)6	12	65768904	65768915
chr1_11314	p2	(TG)18	36	65769429	65769464
chr1_11315	p2	(AC)6	12	65770351	65770362
chr1_11316	p3	(CCT)9	27	65774059	65774085
chr1_11317	p2	(TG)14	28	65776814	65776841
chr1_11318	p2	(GT)10	20	65776963	65776982
chr1_11319	p2	(AC)7	14	65799007	65799020
chr1_11320	p2	(GT)6	12	65801040	65801051
chr1_11321	p2	(CT)7	14	65802305	65802318
chr1_11322	c	(AAAAG)6aaaacaa(AAC)5	52	65807142	65807193
chr1_11323	p2	(AT)13	26	65835961	65835986
chr1_11324	p3	(TCT)5	15	65836926	65836940
chr1_11325	p2	(TG)8	16	65848376	65848391
chr1_11326	p2	(GT)9	18	65854250	65854267
chr1_11327	p4	(TAGA)5	20	65862505	65862524
chr1_11328	p2	(AG)7	14	65880253	65880266
chr1_11329	p3	(TTG)5	15	65885558	65885572
chr1_11330	c	(GT)6t(AG)6	25	65892729	65892753
chr1_11331	p3	(ATT)7	21	65897583	65897603
chr1_11332	p2	(TG)7	14	65898682	65898695

chr1_11333	p2	(CA)9	18	65909030	65909047
chr1_11334	c	caggcagaggaaaaatgtcagctgcttacctacagatggaaggaataagat	114	65909356	65909469
chr1_11335	p2	(TG)14	28	65912099	65912126
chr1_11336	p2	(GT)26	52	65918381	65918432
chr1_11337	p2	(GT)9	18	65919148	65919165
chr1_11338	p4	(CATT)5	20	65936531	65936550
chr1_11339	p2	(AT)7	14	65949156	65949169
chr1_11340	p2	(GT)6	12	65953319	65953330
chr1_11341	p2	(TG)6	12	65953903	65953914
chr1_11342	p2	(TG)24	48	65954638	65954685
chr1_11343	p2	(GT)8	16	65967480	65967495
chr1_11344	p2	(AC)7	14	65970732	65970745
chr1_11345	p2	(TG)14	28	65973830	65973857
chr1_11346	p2	(GT)7	14	65978554	65978567
chr1_11347	p2	(TG)12	24	65978781	65978804
chr1_11348	p2	(TC)6	12	65999988	65999999
chr1_11349	p2	(TG)13	26	66000879	66000904
chr1_11350	c	(CT)9cc(CT)7ttctcact(CA)14	70	66001488	66001557
chr1_11351	p2	(TG)18	36	66005277	66005312
chr1_11352	p2	(TG)18	36	66011408	66011443
chr1_11353	p2	(CA)6	12	66016655	66016666
chr1_11354	p4	(TTGA)5	20	66035011	66035030
chr1_11355	p3	(AAG)11	33	66038684	66038716
chr1_11356	p2	(GT)11	22	66043016	66043037
chr1_11357	p3	(TAA)8	24	66044733	66044756
chr1_11358	p2	(GT)18	36	66055088	66055123
chr1_11359	p2	(TG)10	20	66055281	66055300
chr1_11360	p2	(AG)7	14	66057876	66057889
chr1_11361	p2	(TG)6	12	66059223	66059234
chr1_11362	p2	(GT)6	12	66064757	66064768
chr1_11363	p2	(AC)6	12	66066488	66066499
chr1_11364	c	(GT)17(GA)11	56	66066936	66066991
chr1_11365	c	ccattttgtgctctgtacaccgaggagactggactgcagccaccagtaagg	105	66070145	66070249
chr1_11366	p2	(GT)8	16	66073113	66073128
chr1_11367	p3	(TAT)6	18	66074085	66074102
chr1_11368	p2	(AT)13	26	66090084	66090109
chr1_11369	p2	(TG)6	12	66103005	66103016
chr1_11370	p2	(TG)19	38	66107132	66107169
chr1_11371	p2	(TG)15	30	66108957	66108986
chr1_11372	c	(TG)21tttgtgtatatatactgtatatatg(TA)6	79	66110656	66110734
chr1_11373	p2	(CA)11	22	66128196	66128217
chr1_11374	c	(AC)6ttgttcactttg(CT)12	48	66153476	66153523
chr1_11375	p2	(TG)6	12	66168857	66168868
chr1_11376	p2	(GT)6	12	66168998	66169009
chr1_11377	p2	(TG)22	44	66170181	66170224
chr1_11378	p2	(TG)15	30	66189811	66189840
chr1_11379	p3	(TTA)7	21	66202449	66202469
chr1_11380	p3	(AAT)6	18	66202921	66202938
chr1_11381	p2	(GA)9	18	66210673	66210690
chr1_11382	p2	(AG)10	20	66215497	66215516
chr1_11383	p2	(AC)19	38	66216722	66216759
chr1_11384	p2	(GA)6	12	66218531	66218542

chr1_11385	p2	(TG)7	14	66222645	66222658
chr1_11386	p2	(GT)6	12	66222908	66222919
chr1_11387	p2	(CA)16	32	66234807	66234838
chr1_11388	p3	(AAT)5	15	66247856	66247870
chr1_11389	p4	(AAAT)7	28	66248681	66248708
chr1_11390	p2	(TG)27	54	66255744	66255797
chr1_11391	p2	(AC)9	18	66260866	66260883
chr1_11392	p2	(AG)10	20	66273389	66273408
chr1_11393	p2	(AC)18	36	66274993	66275028
chr1_11394	p3	(AAT)7	21	66277665	66277685
chr1_11395	p2	(CA)6	12	66278662	66278673
chr1_11396	p2	(CA)11	22	66280153	66280174
chr1_11397	p2	(TG)8	16	66280291	66280306
chr1_11398	p2	(TC)6	12	66281561	66281572
chr1_11399	p2	(GA)22	44	66282918	66282961
chr1_11400	p2	(TG)19	38	66300189	66300226
chr1_11401	p5	(AAAAT)7	35	66306837	66306871
chr1_11402	p3	(TAA)5	15	66311701	66311715
chr1_11403	p2	(TA)7	14	66312188	66312201
chr1_11404	p2	(AC)8	16	66314180	66314195
chr1_11405	p2	(TG)24	48	66319259	66319306
chr1_11406	p2	(GT)11	22	66332037	66332058
chr1_11407	p2	(AC)8	16	66336288	66336303
chr1_11408	p2	(CA)7	14	66336472	66336485
chr1_11409	p2	(GT)15	30	66337165	66337194
chr1_11410	p2	(GT)9	18	66337349	66337366
chr1_11411	p2	(CA)14	28	66339431	66339458
chr1_11412	p2	(GT)12	24	66339819	66339842
chr1_11413	p2	(CT)10	20	66343278	66343297
chr1_11414	p3	(CAG)7	21	66351229	66351249
chr1_11415	p2	(CA)7	14	66351722	66351735
chr1_11416	p2	(AC)24	48	66357376	66357423
chr1_11417	p2	(AG)6	12	66361071	66361082
chr1_11418	p2	(GT)6	12	66361672	66361683
chr1_11419	p2	(AC)7	14	66363715	66363728
chr1_11420	p4	(ATGG)6	24	66366729	66366752
chr1_11421	p3	(ATT)6	18	66372558	66372575
chr1_11422	p2	(CA)11	22	66372858	66372879
chr1_11423	p2	(AC)6	12	66375394	66375405
chr1_11424	p2	(AC)27	54	66379822	66379875
chr1_11425	p2	(AT)7	14	66384017	66384030
chr1_11426	p2	(CA)20	40	66384472	66384511
chr1_11427	p2	(AC)7	14	66387785	66387798
chr1_11428	p2	(TG)8	16	66414190	66414205
chr1_11429	p4	(AATG)5	20	66424624	66424643
chr1_11430	p2	(AC)6	12	66430808	66430819
chr1_11431	p3	(TCT)5	15	66433118	66433132
chr1_11432	p4	(TCAT)5	20	66439969	66439988
chr1_11433	p4	(AATA)5	20	66445434	66445453
chr1_11434	p2	(AC)6	12	66453875	66453886
chr1_11435	p2	(TG)8	16	66454697	66454712
chr1_11436	p2	(CA)7	14	66454895	66454908

chr1_11437	p2	(CA)18	36	66463919	66463954
chr1_11438	p2	(AC)15	30	66468661	66468690
chr1_11439	p2	(CA)8	16	66479644	66479659
chr1_11440	c	(AG)8g(GA)8	33	66481733	66481765
chr1_11441	p2	(AC)35	70	66487850	66487919
chr1_11442	p2	(CA)9	18	66489347	66489364
chr1_11443	c	acacaagcacataaagagatgtacatgttcacagaagtggagaacacagt	83	66494338	66494420
chr1_11444	p2	(TG)19	38	66494845	66494882
chr1_11445	p2	(CA)28	56	66495662	66495717
chr1_11446	p2	(AC)17	34	66496004	66496037
chr1_11447	p4	(AAAC)5	20	66508100	66508119
chr1_11448	p3	(TAA)5	15	66511578	66511592
chr1_11449	p2	(AC)32	64	66518349	66518412
chr1_11450	p2	(AC)28	56	66518972	66519027
chr1_11451	p2	(CA)6	12	66519439	66519450
chr1_11452	p2	(TG)12	24	66527009	66527032
chr1_11453	p2	(GA)17	34	66527369	66527402
chr1_11454	p2	(TG)12	24	66530215	66530238
chr1_11455	p2	(CA)9	18	66530439	66530456
chr1_11456	p2	(TG)6	12	66530721	66530732
chr1_11457	p2	(CA)6	12	66530984	66530995
chr1_11458	p2	(GT)6	12	66538435	66538446
chr1_11459	p2	(AC)6	12	66539683	66539694
chr1_11460	p2	(AC)16	32	66544709	66544740
chr1_11461	p3	(TTA)5	15	66566935	66566949
chr1_11462	p2	(CA)21	42	66577485	66577526
chr1_11463	c	(AC)6(AT)11	34	66593166	66593199
chr1_11464	p2	(AT)11	22	66595252	66595273
chr1_11465	p4	(TAGA)6	24	66612608	66612631
chr1_11466	p2	(GA)7	14	66614806	66614819
chr1_11467	p2	(GA)7	14	66615759	66615772
chr1_11468	p2	(GA)7	14	66618394	66618407
chr1_11469	p2	(AC)15	30	66628312	66628341
chr1_11470	p2	(AC)9	18	66629006	66629023
chr1_11471	p2	(CA)6	12	66630543	66630554
chr1_11472	p3	(ATT)6	18	66635315	66635332
chr1_11473	p2	(CT)15	30	66638365	66638394
chr1_11474	p2	(AC)7	14	66638594	66638607
chr1_11475	p2	(AC)15	30	66643688	66643717
chr1_11476	p2	(AC)6	12	66648710	66648721
chr1_11477	p2	(AC)15	30	66667113	66667142
chr1_11478	p2	(AC)18	36	66670366	66670401
chr1_11479	p2	(AC)10	20	66674764	66674783
chr1_11480	p2	(GA)7	14	66677598	66677611
chr1_11481	p2	(TG)9	18	66677994	66678011
chr1_11482	p3	(TCT)7	21	66681892	66681912
chr1_11483	c	(TC)8(AC)8	32	66693386	66693417
chr1_11484	p4	(AATG)5	20	66694560	66694579
chr1_11485	p2	(TG)24	48	66700137	66700184
chr1_11486	c	(CT)11(CA)27	76	66703240	66703315
chr1_11487	p2	(TA)6	12	66705762	66705773
chr1_11488	p2	(AC)11	22	66723862	66723883

chr1_11489	p4	(ATTG)7	28	66730290	66730317
chr1_11490	p2	(CA)18	36	66740418	66740453
chr1_11491	p2	(GT)7	14	66742185	66742198
chr1_11492	p2	(AC)6	12	66777775	66777786
chr1_11493	p2	(AC)8	16	66790651	66790666
chr1_11494	p2	(AC)6	12	66794985	66794996
chr1_11495	p2	(TG)6	12	66805331	66805342
chr1_11496	p2	(TG)6	12	66806966	66806977
chr1_11497	p2	(GT)7	14	66819204	66819217
chr1_11498	c	aattcctacctgctaatgcctcttttcggtctctgatgggaattgctcagatcc	92	66841426	66841517
chr1_11499	p2	(GA)6	12	66844509	66844520
chr1_11500	p2	(AC)8	16	66845957	66845972
chr1_11501	p2	(GT)37	74	66846536	66846609
chr1_11502	p3	(ATA)7	21	66894113	66894133
chr1_11503	p4	(AATG)5	20	66899426	66899445
chr1_11504	p3	(ATT)8	24	66917566	66917589
chr1_11505	p2	(TG)7	14	66921056	66921069
chr1_11506	p2	(CT)7	14	66926677	66926690
chr1_11507	p2	(GT)6	12	66929515	66929526
chr1_11508	p3	(GTG)5	15	66929767	66929781
chr1_11509	p2	(AC)7	14	66933351	66933364
chr1_11510	p2	(CA)6	12	66933593	66933604
chr1_11511	p2	(TA)6	12	66935155	66935166
chr1_11512	p2	(TG)6	12	66941500	66941511
chr1_11513	p2	(TG)6	12	66951085	66951096
chr1_11514	p3	(AAT)7	21	66959156	66959176
chr1_11515	p2	(GT)12	24	66960955	66960978
chr1_11516	p3	(GCA)5	15	66968008	66968022
chr1_11517	p2	(CA)6	12	66979782	66979793
chr1_11518	p3	(GTA)5	15	66980042	66980056
chr1_11519	p3	(TGT)6	18	66992877	66992894
chr1_11520	p3	(TAT)5	15	67000919	67000933
chr1_11521	p2	(CA)6	12	67005927	67005938
chr1_11522	p2	(AG)10	20	67006919	67006938
chr1_11523	p2	(AC)7	14	67008078	67008091
chr1_11524	p2	(CA)10	20	67008372	67008391
chr1_11525	p2	(TG)7	14	67010842	67010855
chr1_11526	p2	(TG)6	12	67015157	67015168
chr1_11527	c	(TG)21tac(GT)10	65	67017472	67017536
chr1_11528	p2	(TG)7	14	67017722	67017735
chr1_11529	p2	(AC)6	12	67020497	67020508
chr1_11530	c	gccgcagctttgttgctcccctggggctgatggagatgggccgtcgcctgg	135	67023448	67023582
chr1_11531	p2	(TG)16	32	67032036	67032067
chr1_11532	p2	(TG)6	12	67056479	67056490
chr1_11533	p2	(CA)6	12	67060266	67060277
chr1_11534	p2	(TG)6	12	67069057	67069068
chr1_11535	p2	(TC)8	16	67072301	67072316
chr1_11536	p4	(CATA)6	24	67076284	67076307
chr1_11537	p2	(TG)6	12	67089188	67089199
chr1_11538	p2	(AC)9	18	67089304	67089321
chr1_11539	p3	(TAT)5	15	67097647	67097661
chr1_11540	p2	(TA)6	12	67097799	67097810

chr1_11541	p3	(ATT)5	15	67101436	67101450
chr1_11542	p2	(AC)15	30	67101893	67101922
chr1_11543	p2	(CA)6	12	67103094	67103105
chr1_11544	p4	(TTTG)5	20	67103318	67103337
chr1_11545	p2	(TG)6	12	67105777	67105788
chr1_11546	p2	(AC)6	12	67115102	67115113
chr1_11547	p2	(AC)6	12	67122553	67122564
chr1_11548	p2	(AG)9	18	67124194	67124211
chr1_11549	p2	(AC)6	12	67124789	67124800
chr1_11550	p3	(TTA)5	15	67131208	67131222
chr1_11551	p2	(TG)6	12	67132344	67132355
chr1_11552	p2	(CA)7	14	67134000	67134013
chr1_11553	p2	(GT)8	16	67134538	67134553
chr1_11554	p2	(TG)17	34	67138893	67138926
chr1_11555	p3	(TAT)5	15	67140017	67140031
chr1_11556	p2	(TG)17	34	67140695	67140728
chr1_11557	p2	(TG)6	12	67142272	67142283
chr1_11558	p4	(CATT)5	20	67152946	67152965
chr1_11559	p2	(AC)8	16	67164005	67164020
chr1_11560	p2	(GT)9	18	67177511	67177528
chr1_11561	p2	(TG)14	28	67189186	67189213
chr1_11562	p2	(GA)6	12	67189486	67189497
chr1_11563	p3	(TCT)5	15	67199819	67199833
chr1_11564	p3	(AAT)5	15	67201981	67201995
chr1_11565	p2	(CA)9	18	67211303	67211320
chr1_11566	p2	(CA)12	24	67212381	67212404
chr1_11567	p3	(CCA)7	21	67231026	67231046
chr1_11568	p2	(GA)8	16	67234792	67234807
chr1_11569	p2	(TC)7	14	67243853	67243866
chr1_11570	p4	(TCCA)5	20	67253691	67253710
chr1_11571	p2	(CT)6	12	67256547	67256558
chr1_11572	p2	(CT)7	14	67261653	67261666
chr1_11573	p3	(TAT)5	15	67263243	67263257
chr1_11574	p4	(ATGG)5	20	67270457	67270476
chr1_11575	c	(AC)8atatatatacatat(AC)8atatatatacatat(AC)8	76	67276104	67276179
chr1_11576	p2	(AC)7	14	67281335	67281348
chr1_11577	p2	(AC)7	14	67284800	67284813
chr1_11578	p4	(AAAC)5	20	67292713	67292732
chr1_11579	p2	(AG)9	18	67293924	67293941
chr1_11580	p2	(TG)12	24	67295685	67295708
chr1_11581	c	(TC)7gcacgcacacg(CA)9cg(CA)15	75	67299992	67300066
chr1_11582	c	(AT)7t(TG)6	27	67301252	67301278
chr1_11583	p2	(CT)7	14	67306441	67306454
chr1_11584	p2	(AC)25	50	67317048	67317097
chr1_11585	p3	(ATT)6	18	67328185	67328202
chr1_11586	p3	(GGA)6	18	67329550	67329567
chr1_11587	p3	(AAT)5	15	67333893	67333907
chr1_11588	p2	(CT)14	28	67334368	67334395
chr1_11589	p2	(AC)11	22	67336434	67336455
chr1_11590	p2	(CA)8	16	67340742	67340757
chr1_11591	p2	(TG)9	18	67343767	67343784
chr1_11592	p4	(AAAT)5	20	67350142	67350161

chr1_11593	p2	(GA)6	12	67352060	67352071
chr1_11594	p2	(GT)10	20	67352709	67352728
chr1_11595	p2	(AC)16	32	67358470	67358501
chr1_11596	p2	(GA)6	12	67361155	67361166
chr1_11597	p2	(GT)7	14	67361414	67361427
chr1_11598	p2	(CA)9	18	67368110	67368127
chr1_11599	p2	(CA)6	12	67369491	67369502
chr1_11600	p2	(AC)14	28	67374072	67374099
chr1_11601	p2	(AC)13	26	67374909	67374934
chr1_11602	p2	(AT)8	16	67375995	67376010
chr1_11603	p2	(GT)9	18	67378097	67378114
chr1_11604	p2	(GT)14	28	67388472	67388499
chr1_11605	p2	(AC)9	18	67388793	67388810
chr1_11606	p2	(CA)6	12	67390371	67390382
chr1_11607	p2	(CA)6	12	67393898	67393909
chr1_11608	p2	(AC)6	12	67394087	67394098
chr1_11609	p2	(TG)28	56	67394729	67394784
chr1_11610	p2	(TG)7	14	67398321	67398334
chr1_11611	p2	(CA)6	12	67402667	67402678
chr1_11612	p2	(GT)6	12	67414680	67414691
chr1_11613	c	(TG)7(AG)9	32	67421622	67421653
chr1_11614	p2	(AC)7	14	67436610	67436623
chr1_11615	p2	(AT)6	12	67438112	67438123
chr1_11616	p2	(CA)11	22	67439796	67439817
chr1_11617	p2	(TG)23	46	67445264	67445309
chr1_11618	p2	(AC)13	26	67446015	67446040
chr1_11619	p2	(TG)7	14	67446791	67446804
chr1_11620	p2	(TA)7	14	67449020	67449033
chr1_11621	p2	(CA)18	36	67450201	67450236
chr1_11622	p2	(TG)6	12	67450348	67450359
chr1_11623	p2	(CA)6	12	67459838	67459849
chr1_11624	p2	(TG)11	22	67460780	67460801
chr1_11625	p2	(GT)9	18	67461330	67461347
chr1_11626	p2	(CA)16	32	67461947	67461978
chr1_11627	p2	(TA)9	18	67462141	67462158
chr1_11628	p2	(GA)7	14	67466604	67466617
chr1_11629	p2	(GT)15	30	67469121	67469150
chr1_11630	p2	(TC)9	18	67470879	67470896
chr1_11631	p2	(GT)10	20	67476139	67476158
chr1_11632	p2	(CA)6	12	67478231	67478242
chr1_11633	p2	(CA)15	30	67499327	67499356
chr1_11634	p2	(CA)12	24	67502505	67502528
chr1_11635	p2	(TG)6	12	67505944	67505955
chr1_11636	p2	(GT)6	12	67512422	67512433
chr1_11637	p3	(CCT)11	33	67523740	67523772
chr1_11638	p2	(TG)12	24	67530948	67530971
chr1_11639	p4	(ATTC)5	20	67536809	67536828
chr1_11640	p2	(CA)6	12	67542657	67542668
chr1_11641	p3	(GGT)5	15	67550588	67550602
chr1_11642	p2	(AC)13	26	67560530	67560555
chr1_11643	p2	(CT)6	12	67562043	67562054
chr1_11644	p2	(AC)19	38	67576684	67576721

chr1_11645	p2	(GT)18	36	67578977	67579012
chr1_11646	p2	(GT)6	12	67579367	67579378
chr1_11647	p2	(TG)16	32	67581111	67581142
chr1_11648	p2	(TC)7	14	67585259	67585272
chr1_11649	p2	(AG)11	22	67601559	67601580
chr1_11650	p2	(TG)6	12	67605692	67605703
chr1_11651	p2	(CA)6	12	67612945	67612956
chr1_11652	c	(TG)8ta(TG)28	74	67613909	67613982
chr1_11653	p2	(TG)6	12	67617650	67617661
chr1_11654	p2	(AC)7	14	67621919	67621932
chr1_11655	p2	(TA)7	14	67625528	67625541
chr1_11656	p2	(GT)11	22	67626581	67626602
chr1_11657	p2	(TG)11	22	67630947	67630968
chr1_11658	p2	(TG)10	20	67632532	67632551
chr1_11659	p2	(TG)6	12	67632678	67632689
chr1_11660	p2	(CA)15	30	67632795	67632824
chr1_11661	p2	(CA)12	24	67633959	67633982
chr1_11662	p2	(TG)7	14	67635857	67635870
chr1_11663	p2	(TG)19	38	67638746	67638783
chr1_11664	p2	(AC)15	30	67639815	67639844
chr1_11665	p2	(TA)6	12	67640222	67640233
chr1_11666	p2	(AC)14	28	67642309	67642336
chr1_11667	c*	(TC)12tttctctctctatctctctctt(TC)6(CTCC)6*	79	67642544	67642622
chr1_11668	p2	(TC)6	12	67646123	67646134
chr1_11669	p2	(GT)6	12	67649403	67649414
chr1_11670	c	(TC)7g(CT)7	29	67650122	67650150
chr1_11671	c	(GT)6gcgcgcgtgtg(TA)6(TG)12	59	67650979	67651037
chr1_11672	p2	(GT)20	40	67652670	67652709
chr1_11673	p2	(TG)16	32	67654132	67654163
chr1_11674	p2	(AC)12	24	67661750	67661773
chr1_11675	p2	(AC)29	58	67665172	67665229
chr1_11676	p2	(TG)15	30	67668290	67668319
chr1_11677	p2	(TG)10	20	67668618	67668637
chr1_11678	p2	(TC)7	14	67668984	67668997
chr1_11679	p2	(TA)7	14	67671394	67671407
chr1_11680	p2	(TG)13	26	67672820	67672845
chr1_11681	p2	(TG)19	38	67674698	67674735
chr1_11682	p3	(TTA)6	18	67703340	67703357
chr1_11683	p2	(TC)14	28	67703826	67703853
chr1_11684	p2	(GT)8	16	67719660	67719675
chr1_11685	p2	(GT)8	16	67722172	67722187
chr1_11686	c	(TC)7at(AC)16	48	67726074	67726121
chr1_11687	p2	(AC)7	14	67741833	67741846
chr1_11688	c	acagtggctcttgttctctctacaggcagtctaatgaacacacaaatatggc	113	67745697	67745809
chr1_11689	c	(TG)8(AG)8(TG)13	58	67747768	67747825
chr1_11690	p3	(TGC)6	18	67749735	67749752
chr1_11691	c	(TC)10tgtctctttct(CA)9	49	67759127	67759175
chr1_11692	p2	(GT)23	46	67760159	67760204
chr1_11693	p2	(CA)11	22	67762754	67762775
chr1_11694	p2	(CA)6	12	67770442	67770453
chr1_11695	c	(CACG)6(CA)20	64	67770949	67771012
chr1_11696	c	ttgcacatttgtttgaggtgacttcccaagggaacatcagcagggtgtattt	113	67772435	67772547

chr1_11697	p2	(GT)10	20	67785040	67785059
chr1_11698	p2	(GT)18	36	67789556	67789591
chr1_11699	p2	(CA)18	36	67790725	67790760
chr1_11700	p2	(CA)9	18	67795985	67796002
chr1_11701	p2	(AT)6	12	67800252	67800263
chr1_11702	p2	(GT)15	30	67820343	67820372
chr1_11703	p2	(CA)6	12	67824316	67824327
chr1_11704	p2	(GT)8	16	67825851	67825866
chr1_11705	p2	(TG)6	12	67828733	67828744
chr1_11706	p2	(TC)16	32	67831500	67831531
chr1_11707	p2	(TG)25	50	67832931	67832980
chr1_11708	p2	(CA)9	18	67835713	67835730
chr1_11709	p2	(TG)14	28	67843443	67843470
chr1_11710	p2	(GT)9	18	67844376	67844393
chr1_11711	p2	(TG)8	16	67847280	67847295
chr1_11712	p2	(TG)9	18	67852249	67852266
chr1_11713	p5	(AAATC)5	25	67854478	67854502
chr1_11714	p2	(GA)12	24	67862506	67862529
chr1_11715	p4	(ATTT)6	24	67864771	67864794
chr1_11716	p2	(CA)8	16	67872533	67872548
chr1_11717	p4	(TAAA)5	20	67872717	67872736
chr1_11718	p2	(TC)6	12	67875405	67875416
chr1_11719	p2	(TG)22	44	67876989	67877032
chr1_11720	p2	(TG)15	30	67885454	67885483
chr1_11721	p2	(AC)6	12	67890303	67890314
chr1_11722	c	(TG)26tatgtatgattgccatagtttgtgcatgtacgctgtt(TG)22	133	67903403	67903535
chr1_11723	p2	(GT)30	60	67904310	67904369
chr1_11724	p2	(CT)6	12	67905234	67905245
chr1_11725	p2	(TG)6	12	67906979	67906990
chr1_11726	p2	(AC)11	22	67907944	67907965
chr1_11727	p2	(AC)6	12	67910673	67910684
chr1_11728	p2	(AC)8	16	67918471	67918486
chr1_11729	p2	(TG)11	22	67929397	67929418
chr1_11730	p3	(TAA)5	15	67940046	67940060
chr1_11731	p2	(AC)6	12	67954027	67954038
chr1_11732	p2	(CT)6	12	67957412	67957423
chr1_11733	p4	(ATGG)5	20	67978891	67978910
chr1_11734	p3	(CTT)7	21	67991782	67991802
chr1_11735	p3	(TTA)5	15	67994018	67994032
chr1_11736	p2	(GT)6	12	68010930	68010941
chr1_11737	c	(TA)8(TG)10a(GT)6	49	68011413	68011461
chr1_11738	c	(TG)10(AG)8	36	68012848	68012883
chr1_11739	p3	(AAT)5	15	68013745	68013759
chr1_11740	p2	(CA)7	14	68014563	68014576
chr1_11741	p2	(CA)10	20	68029229	68029248
chr1_11742	p2	(AG)6	12	68029364	68029375
chr1_11743	p2	(GT)8	16	68043285	68043300
chr1_11744	p5	(TTGAT)5	25	68045555	68045579
chr1_11745	p2	(GA)6	12	68053130	68053141
chr1_11746	p3	(TAC)5	15	68059719	68059733
chr1_11747	p2	(GT)30	60	68059998	68060057
chr1_11748	p3	(ATA)7	21	68061294	68061314

chr1_11749	p2	(AT)6	12	68065182	68065193
chr1_11750	p3	(TGT)5	15	68071540	68071554
chr1_11751	p3	(ATA)6	18	68074173	68074190
chr1_11752	c	(CA)7tatacag(TA)6	33	68080517	68080549
chr1_11753	p2	(TC)6	12	68092283	68092294
chr1_11754	p2	(GA)6	12	68108615	68108626
chr1_11755	p2	(GA)6	12	68115843	68115854
chr1_11756	p2	(AG)21	42	68118046	68118087
chr1_11757	p2	(TA)12	24	68119612	68119635
chr1_11758	p2	(AC)14	28	68124361	68124388
chr1_11759	p2	(TG)12	24	68128985	68129008
chr1_11760	c	(CT)14(CA)24	76	68131909	68131984
chr1_11761	p2	(AC)6	12	68139094	68139105
chr1_11762	p2	(CA)13	26	68150985	68151010
chr1_11763	p3	(ATA)10	30	68151725	68151754
chr1_11764	c	(TA)7(CA)11gaat(GA)8	56	68155266	68155321
chr1_11765	p2	(TG)6	12	68157884	68157895
chr1_11766	p2	(GT)6	12	68172136	68172147
chr1_11767	p2	(AC)6	12	68179450	68179461
chr1_11768	p2	(CA)6	12	68187995	68188006
chr1_11769	p2	(TG)7	14	68200161	68200174
chr1_11770	p2	(TG)6	12	68207731	68207742
chr1_11771	p2	(AC)12	24	68224504	68224527
chr1_11772	p2	(GT)6	12	68224715	68224726
chr1_11773	p3	(CCT)8	24	68224946	68224969
chr1_11774	p2	(CA)8	16	68228594	68228609
chr1_11775	p2	(GT)6	12	68230239	68230250
chr1_11776	p2	(TC)14	28	68230544	68230571
chr1_11777	p2	(TG)6	12	68231636	68231647
chr1_11778	p2	(GA)8	16	68240720	68240735
chr1_11779	p2	(AC)12	24	68246113	68246136
chr1_11780	p2	(CA)18	36	68246263	68246298
chr1_11781	p2	(CA)11	22	68250041	68250062
chr1_11782	p2	(AC)24	48	68250187	68250234
chr1_11783	p3	(GAG)5	15	68252449	68252463
chr1_11784	p2	(TG)7	14	68252590	68252603
chr1_11785	p2	(AG)7	14	68255591	68255604
chr1_11786	p3	(CAA)10	30	68260492	68260521
chr1_11787	p2	(AG)6	12	68260922	68260933
chr1_11788	p3	(GAG)5	15	68261170	68261184
chr1_11789	p2	(AC)6	12	68266326	68266337
chr1_11790	p3	(CTT)11	33	68268519	68268551
chr1_11791	p2	(GA)8	16	68276818	68276833
chr1_11792	p2	(TC)9	18	68277981	68277998
chr1_11793	p5	(GAGAA)9	45	68279321	68279365
chr1_11794	p2	(GT)6	12	68282449	68282460
chr1_11795	p2	(CA)6	12	68287257	68287268
chr1_11796	p2	(TC)29	58	68288508	68288565
chr1_11797	p2	(AC)11	22	68289116	68289137
chr1_11798	p2	(CA)6	12	68292489	68292500
chr1_11799	p2	(CA)7	14	68292789	68292802
chr1_11800	p2	(TG)12	24	68295062	68295085

chr1_11801	c	(CT)8(CA)10	36	68299329	68299364
chr1_11802	p2	(AC)9	18	68303972	68303989
chr1_11803	p2	(CA)14	28	68306481	68306508
chr1_11804	p2	(GT)8	16	68309272	68309287
chr1_11805	p2	(GT)7	14	68323302	68323315
chr1_11806	p2	(GT)9	18	68330269	68330286
chr1_11807	p2	(AC)12	24	68332845	68332868
chr1_11808	p2	(TC)7	14	68333622	68333635
chr1_11809	p2	(GT)8	16	68333779	68333794
chr1_11810	p2	(AC)6	12	68335350	68335361
chr1_11811	p2	(CA)7	14	68338235	68338248
chr1_11812	p2	(AT)7	14	68345024	68345037
chr1_11813	p3	(ATA)6	18	68345238	68345255
chr1_11814	p3	(AGA)8	24	68378901	68378924
chr1_11815	p2	(GA)8	16	68380124	68380139
chr1_11816	p2	(CA)6	12	68388676	68388687
chr1_11817	p5	(GTAGA)12	60	68392674	68392733
chr1_11818	p2	(CA)17	34	68394943	68394976
chr1_11819	p2	(CA)7	14	68398784	68398797
chr1_11820	p2	(AC)6	12	68404101	68404112
chr1_11821	p3	(ATA)6	18	68406001	68406018
chr1_11822	p2	(AC)6	12	68411234	68411245
chr1_11823	p2	(TC)6	12	68411622	68411633
chr1_11824	p2	(CA)6	12	68415710	68415721
chr1_11825	p2	(AC)8	16	68417678	68417693
chr1_11826	p2	(GT)6	12	68418603	68418614
chr1_11827	c	(AT)8gtt(TA)8	35	68426053	68426087
chr1_11828	p4	(AATG)6	24	68438895	68438918
chr1_11829	p3	(CAG)5	15	68444151	68444165
chr1_11830	p2	(CA)6	12	68445263	68445274
chr1_11831	p2	(CT)6	12	68452761	68452772
chr1_11832	p2	(AG)7	14	68452893	68452906
chr1_11833	p2	(TG)7	14	68453049	68453062
chr1_11834	p2	(AC)8	16	68453867	68453882
chr1_11835	c	(AC)8aggtaatacgtgttgctacacgcatttcc(AT)6	57	68455404	68455460
chr1_11836	c	[illegible]cctttattaagtgcatacagtcgcatatatgtgtggtaacacatggtaaacg[illegible]	100	68457364	68457463
chr1_11837	p2	(CT)55	110	68463851	68463960
chr1_11838	p2	(CT)19	38	68467081	68467118
chr1_11839	p3	(CCT)44	132	68476380	68476511
chr1_11840	p2	(TG)39	78	68479865	68479942
chr1_11841	p2	(TG)6	12	68480236	68480247
chr1_11842	p2	(AG)6	12	68483046	68483057
chr1_11843	c	[illegible])9ggtagccccccttcccgtgcatgcagacactgactatttccagatgtt(T[illegible]	99	68483918	68484016
chr1_11844	p2	(AC)13	26	68494459	68494484
chr1_11845	p2	(CT)7	14	68524489	68524502
chr1_11846	c	(TG)12g(GT)6	37	68526955	68526991
chr1_11847	p3	(TCA)5	15	68538293	68538307
chr1_11848	p2	(TG)6	12	68547742	68547753
chr1_11849	p2	(TG)7	14	68548016	68548029
chr1_11850	p3	(CTG)5	15	68552728	68552742
chr1_11851	p2	(CA)7	14	68553817	68553830
chr1_11852	p2	(CT)6	12	68554004	68554015

chr1_11853	c	(AG)6atgat(AG)7	31	68563310	68563340
chr1_11854	p2	(TG)6	12	68563683	68563694
chr1_11855	p2	(AG)6	12	68563811	68563822
chr1_11856	p2	(GT)10	20	68566904	68566923
chr1_11857	p2	(GT)13	26	68570822	68570847
chr1_11858	p3	(TAT)6	18	68571543	68571560
chr1_11859	p3	(TAA)7	21	68574296	68574316
chr1_11860	p2	(GA)8	16	68576105	68576120
chr1_11861	p2	(TG)10	20	68593696	68593715
chr1_11862	p2	(TG)6	12	68593819	68593830
chr1_11863	p2	(CA)6	12	68594150	68594161
chr1_11864	p2	(AC)13	26	68595807	68595832
chr1_11865	p3	(CTC)5	15	68596692	68596706
chr1_11866	p2	(TG)20	40	68596933	68596972
chr1_11867	p2	(TG)16	32	68604332	68604363
chr1_11868	p2	(AC)24	48	68605784	68605831
chr1_11869	p2	(TG)17	34	68607624	68607657
chr1_11870	p3	(ATC)6	18	68611869	68611886
chr1_11871	p2	(CA)9	18	68618676	68618693
chr1_11872	p2	(AC)7	14	68619558	68619571
chr1_11873	c	(TG)17(CG)7	48	68630143	68630190
chr1_11874	p4	(ATAC)5	20	68655831	68655850
chr1_11875	p3	(ATA)7	21	68659961	68659981
chr1_11876	p3	(ATT)8	24	68661051	68661074
chr1_11877	p2	(CA)10	20	68665065	68665084
chr1_11878	p2	(CA)10	20	68666142	68666161
chr1_11879	p2	(CA)10	20	68667268	68667287
chr1_11880	p2	(TG)9	18	68667487	68667504
chr1_11881	p2	(TG)15	30	68671545	68671574
chr1_11882	p2	(GA)8	16	68673746	68673761
chr1_11883	p2	(TG)13	26	68674402	68674427
chr1_11884	p2	(AC)6	12	68692812	68692823
chr1_11885	p2	(AC)14	28	68704296	68704323
chr1_11886	p2	(AC)8	16	68705575	68705590
chr1_11887	p2	(TA)8	16	68705805	68705820
chr1_11888	p2	(GT)6	12	68712183	68712194
chr1_11889	p2	(AC)14	28	68714089	68714116
chr1_11890	p2	(TG)16	32	68716372	68716403
chr1_11891	p2	(AG)7	14	68720261	68720274
chr1_11892	p3	(ATA)6	18	68721037	68721054
chr1_11893	c	(CA)6g(AC)6	25	68727580	68727604
chr1_11894	p2	(CA)17	34	68739066	68739099
chr1_11895	p2	(AC)13	26	68739315	68739340
chr1_11896	p2	(TC)7	14	68746830	68746843
chr1_11897	p3	(AGC)9	27	68758213	68758239
chr1_11898	p2	(AC)8	16	68763567	68763582
chr1_11899	p2	(GA)6	12	68764849	68764860
chr1_11900	p2	(AC)9	18	68766865	68766882
chr1_11901	p2	(TG)18	36	68768834	68768869
chr1_11902	p5	(TAACG)8	40	68770211	68770250
chr1_11903	p2	(CA)15	30	68773584	68773613
chr1_11904	p2	(CT)22	44	68774209	68774252

chr1_11905	p2	(AC)8	16	68774630	68774645
chr1_11906	c	(CA)11a(AC)12	47	68781555	68781601
chr1_11907	p3	(CTC)9	27	68782052	68782078
chr1_11908	p2	(CA)7	14	68782836	68782849
chr1_11909	p2	(GT)11	22	68786631	68786652
chr1_11910	p2	(AC)6	12	68787600	68787611
chr1_11911	p2	(CA)8	16	68801228	68801243
chr1_11912	p2	(TG)6	12	68802493	68802504
chr1_11913	p3	(TAA)5	15	68804482	68804496
chr1_11914	p2	(TG)16	32	68807060	68807091
chr1_11915	p2	(TG)13	26	68819779	68819804
chr1_11916	p2	(TG)6	12	68824115	68824126
chr1_11917	p2	(TG)7	14	68824436	68824449
chr1_11918	p2	(AG)7	14	68828350	68828363
chr1_11919	p2	(AC)6	12	68830818	68830829
chr1_11920	p2	(CA)6	12	68831575	68831586
chr1_11921	p2	(TG)6	12	68840685	68840696
chr1_11922	p2	(GT)20	40	68847132	68847171
chr1_11923	p2	(AC)6	12	68854086	68854097
chr1_11924	c	(GT)12t(TG)15	55	68861747	68861801
chr1_11925	p2	(TG)7	14	68863733	68863746
chr1_11926	p2	(TG)6	12	68869615	68869626
chr1_11927	p2	(GT)8	16	68880593	68880608
chr1_11928	p2	(GT)19	38	68899954	68899991
chr1_11929	p2	(AT)12	24	68903875	68903898
chr1_11930	p2	(TG)9	18	68905396	68905413
chr1_11931	p2	(GT)7	14	68905576	68905589
chr1_11932	p2	(TG)17	34	68906005	68906038
chr1_11933	c*	(AT)10(GT)13(GTAT)5*	64	68907201	68907264
chr1_11934	p2	(TG)8	16	68907847	68907862
chr1_11935	p2	(CT)7	14	68908007	68908020
chr1_11936	p2	(TG)16	32	68914751	68914782
chr1_11937	p2	(TA)9	18	68915593	68915610
chr1_11938	p2	(TG)12	24	68919786	68919809
chr1_11939	p2	(CA)7	14	68920311	68920324
chr1_11940	p3	(CTC)8	24	68923170	68923193
chr1_11941	p2	(TG)7	14	68925177	68925190
chr1_11942	p2	(TC)6	12	68935564	68935575
chr1_11943	p3	(CAC)5	15	68937665	68937679
chr1_11944	p2	(TC)6	12	68938673	68938684
chr1_11945	p2	(GT)6	12	68944691	68944702
chr1_11946	p2	(TG)8	16	68945905	68945920
chr1_11947	p2	(AC)18	36	68946283	68946318
chr1_11948	c	(GT)10ga(GT)13	48	68946988	68947035
chr1_11949	p2	(TG)9	18	68948672	68948689
chr1_11950	p2	(GT)10	20	68952109	68952128
chr1_11951	p2	(TG)14	28	68954803	68954830
chr1_11952	p2	(CA)7	14	68959411	68959424
chr1_11953	p2	(GT)26	52	68965408	68965459
chr1_11954	p2	(TG)17	34	68967710	68967743
chr1_11955	p2	(AC)8	16	68968516	68968531
chr1_11956	p2	(AC)6	12	68970973	68970984

chr1_11957	c	gcaaaccccccacggtccatggataatgcatttttacacctttcttattagtcac	117	68973079	68973195
chr1_11958	c	tgtctttctttaagcctctctgatatacaacagttccttcaggggagtttttcttc	135	68973527	68973661
chr1_11959	p2	(AC)6	12	68975665	68975676
chr1_11960	p2	(TG)14	28	68978487	68978514
chr1_11961	p2	(TG)15	30	68979071	68979100
chr1_11962	p2	(AC)15	30	68980750	68980779
chr1_11963	p2	(CA)6	12	68981007	68981018
chr1_11964	p2	(TC)6	12	68982195	68982206
chr1_11965	p3	(AGG)5	15	68985406	68985420
chr1_11966	c	cttgtttatagacacacatgcttatatggtccgacatacacatcgaaacacat	135	68988553	68988687
chr1_11967	p2	(TG)6	12	68989088	68989099
chr1_11968	p2	(GT)11	22	68994160	68994181
chr1_11969	p2	(TG)10	20	69003100	69003119
chr1_11970	c	gagagagggatggggggagagagacaaagagaggggggaggaggaag	100	69005698	69005797
chr1_11971	p2	(AC)10	20	69012070	69012089
chr1_11972	c	ttcatt(TG)30tccaggtccccaataaaccagataatgatccatcatgata	254	69020115	69020368
chr1_11973	p5	(ATTTG)5	25	69021815	69021839
chr1_11974	p5	(TATTC)9	45	69029651	69029695
chr1_11975	p2	(CA)14	28	69033767	69033794
chr1_11976	p2	(TG)7	14	69034005	69034018
chr1_11977	p2	(CA)6	12	69034661	69034672
chr1_11978	p2	(TA)11	22	69035802	69035823
chr1_11979	p2	(CA)6	12	69048425	69048436
chr1_11980	p2	(TG)6	12	69050116	69050127
chr1_11981	p2	(TG)7	14	69050932	69050945
chr1_11982	p2	(GT)15	30	69051118	69051147
chr1_11983	p2	(TG)7	14	69055945	69055958
chr1_11984	p2	(CA)8	16	69056080	69056095
chr1_11985	p2	(TG)24	48	69056381	69056428
chr1_11986	p2	(GT)12	24	69062926	69062949
chr1_11987	p2	(TG)22	44	69064117	69064160
chr1_11988	p2	(TG)12	24	69066308	69066331
chr1_11989	p4	(AGGT)6	24	69067108	69067131
chr1_11990	c	agattcagcgagcatgacggagaatgacaggttgatttatggcaagactat	121	69070967	69071087
chr1_11991	p2	(TG)10	20	69074199	69074218
chr1_11992	p2	(TG)6	12	69074579	69074590
chr1_11993	p2	(TC)7	14	69074699	69074712
chr1_11994	p2	(CT)7	14	69074859	69074872
chr1_11995	p2	(AG)6	12	69077143	69077154
chr1_11996	p2	(GT)8	16	69077911	69077926
chr1_11997	p2	(CA)11	22	69085090	69085111
chr1_11998	c	(CA)7(CG)7	28	69085261	69085288
chr1_11999	p2	(TG)24	48	69090585	69090632
chr1_12000	p2	(GT)19	38	69091661	69091698
chr1_12001	p2	(CT)6	12	69092840	69092851
chr1_12002	p4	(ATGG)6	24	69094778	69094801
chr1_12003	p3	(TTA)5	15	69095115	69095129
chr1_12004	p2	(AC)6	12	69099609	69099620
chr1_12005	p2	(TG)14	28	69101913	69101940
chr1_12006	p2	(CT)11	22	69103032	69103053
chr1_12007	p4	(ATTC)5	20	69108556	69108575
chr1_12008	c	acttgctcagcatgcattttttctgtttttctgtctccctccatctgtgtcatatcc	94	69116632	69116725

chr1_12009	p3	(ACC)5	15	69125412	69125426
chr1_12010	p2	(GC)6	12	69127873	69127884
chr1_12011	p2	(TG)23	46	69135626	69135671
chr1_12012	p2	(GT)6	12	69136086	69136097
chr1_12013	c	ctaactcctaaccctctctgagcagaacctattgtgcgtgacacagagaccg	148	69137021	69137168
chr1_12014	p2	(GC)6	12	69143347	69143358
chr1_12015	p2	(TG)6	12	69153821	69153832
chr1_12016	p2	(GT)19	38	69171223	69171260
chr1_12017	p2	(GT)8	16	69177569	69177584
chr1_12018	p2	(GT)7	14	69178108	69178121
chr1_12019	p2	(AC)25	50	69180070	69180119
chr1_12020	p2	(TC)9	18	69182837	69182854
chr1_12021	p2	(GA)6	12	69183959	69183970
chr1_12022	p2	(GT)10	20	69184285	69184304
chr1_12023	p2	(CA)6	12	69184954	69184965
chr1_12024	p2	(AT)6	12	69186090	69186101
chr1_12025	p2	(TG)10	20	69202967	69202986
chr1_12026	p2	(AC)7	14	69204738	69204751
chr1_12027	p3	(TCT)5	15	69207822	69207836
chr1_12028	p3	(TAT)5	15	69221832	69221846
chr1_12029	p2	(CA)17	34	69223587	69223620
chr1_12030	p2	(AC)33	66	69224019	69224084
chr1_12031	p2	(TC)6	12	69225801	69225812
chr1_12032	p3	(AAT)7	21	69230337	69230357
chr1_12033	p4	(GAGT)5	20	69230554	69230573
chr1_12034	p2	(AC)7	14	69236671	69236684
chr1_12035	p2	(TG)10	20	69238368	69238387
chr1_12036	p2	(CA)36	72	69243058	69243129
chr1_12037	p2	(CA)19	38	69243297	69243334
chr1_12038	p2	(TG)20	40	69249736	69249775
chr1_12039	p2	(GT)22	44	69251492	69251535
chr1_12040	p2	(GT)8	16	69266168	69266183
chr1_12041	p2	(TG)7	14	69277130	69277143
chr1_12042	p3	(ACT)5	15	69277614	69277628
chr1_12043	p2	(GT)8	16	69284995	69285010
chr1_12044	p2	(GT)28	56	69287967	69288022
chr1_12045	p2	(AG)6	12	69288140	69288151
chr1_12046	p2	(TG)9	18	69291654	69291671
chr1_12047	p2	(AC)7	14	69294976	69294989
chr1_12048	p2	(AC)10	20	69298117	69298136
chr1_12049	p4	(AAAC)5	20	69311597	69311616
chr1_12050	c	caccctgatcttgatcaaggtgcacagaacaatggggtggcctgagttgcag	94	69316446	69316539
chr1_12051	p2	(CA)9	18	69319579	69319596
chr1_12052	p4	(CTGT)6	24	69322502	69322525
chr1_12053	p2	(AC)17	34	69325538	69325571
chr1_12054	p3	(AGT)5	15	69336941	69336955
chr1_12055	p2	(TG)22	44	69353524	69353567
chr1_12056	p2	(TG)14	28	69357908	69357935
chr1_12057	p2	(AC)9	18	69358039	69358056
chr1_12058	p2	(TG)16	32	69363860	69363891
chr1_12059	p2	(GT)9	18	69366229	69366246
chr1_12060	c	(CT)20(CA)8	56	69366767	69366822

chr1_12061	p2	(CA)28	56	69371366	69371421
chr1_12062	p2	(AC)8	16	69375532	69375547
chr1_12063	p2	(AC)21	42	69395304	69395345
chr1_12064	c	(CA)15cctgtttgtttttctactaggtggggactgcccccc(CA)6	79	69405589	69405667
chr1_12065	p2	(AC)8	16	69406555	69406570
chr1_12066	p2	(AC)10	20	69410591	69410610
chr1_12067	p2	(AC)6	12	69430515	69430526
chr1_12068	p2	(TG)6	12	69444475	69444486
chr1_12069	c	tgtctgagagtgtgtgtctgtgcatgcatgtggtaattactttttttctgtgtgtg	82	69457051	69457132
chr1_12070	p3	(TAT)5	15	69472886	69472900
chr1_12071	c	(AT)8gta(TG)6	31	69473007	69473037
chr1_12072	p2	(TG)7	14	69473700	69473713
chr1_12073	p2	(TA)6	12	69475109	69475120
chr1_12074	p2	(TG)18	36	69493263	69493298
chr1_12075	p2	(CA)6	12	69495089	69495100
chr1_12076	p3	(AAT)7	21	69500724	69500744
chr1_12077	p2	(AC)14	28	69507217	69507244
chr1_12078	p2	(AC)10	20	69514713	69514732
chr1_12079	c	(CA)11tatatgtatgtg(TA)6	46	69532780	69532825
chr1_12080	p2	(GT)19	38	69538317	69538354
chr1_12081	p2	(CA)8	16	69538516	69538531
chr1_12082	p3	(ATA)5	15	69546932	69546946
chr1_12083	p2	(TG)8	16	69551094	69551109
chr1_12084	p2	(TG)6	12	69558262	69558273
chr1_12085	p2	(AC)8	16	69561801	69561816
chr1_12086	p2	(GT)9	18	69565221	69565238
chr1_12087	p2	(AG)6	12	69583816	69583827
chr1_12088	p3	(AAC)10	30	69596018	69596047
chr1_12089	p2	(CT)11	22	69597209	69597230
chr1_12090	p2	(TG)9	18	69604255	69604272
chr1_12091	c	(AC)14(AT)8	44	69605273	69605316
chr1_12092	p2	(AC)10	20	69605863	69605882
chr1_12093	p2	(AT)9	18	69609464	69609481
chr1_12094	p2	(CA)7	14	69616725	69616738
chr1_12095	p4	(AAAC)5	20	69621174	69621193
chr1_12096	p2	(TG)8	16	69624773	69624788
chr1_12097	p2	(CA)12	24	69636412	69636435
chr1_12098	p3	(TCC)6	18	69639793	69639810
chr1_12099	p2	(AC)8	16	69646012	69646027
chr1_12100	p2	(GT)16	32	69659551	69659582
chr1_12101	p2	(TG)6	12	69660764	69660775
chr1_12102	p2	(GT)9	18	69664261	69664278
chr1_12103	p2	(GT)6	12	69700247	69700258
chr1_12104	p3	(CAA)5	15	69704787	69704801
chr1_12105	p2	(TA)9	18	69714434	69714451
chr1_12106	p2	(AC)7	14	69719025	69719038
chr1_12107	p2	(AT)13	26	69719208	69719233
chr1_12108	p2	(TG)7	14	69726423	69726436
chr1_12109	p2	(CA)6	12	69728686	69728697
chr1_12110	p3	(GAA)6	18	69734576	69734593
chr1_12111	p2	(AG)6	12	69739298	69739309
chr1_12112	p2	(AC)16	32	69740377	69740408

chr1_12113	p2	(TA)6	12	69741236	69741247
chr1_12114	c	:agtagtttgagcctcattattctcgtcactataacaaaactcctgattggccc:	100	69742493	69742592
chr1_12115	p2	(AG)10	20	69747671	69747690
chr1_12116	p3	(TCA)6	18	69755906	69755923
chr1_12117	c	(TC)12(AC)11	46	69762406	69762451
chr1_12118	p2	(CA)25	50	69765416	69765465
chr1_12119	p2	(GT)11	22	69777293	69777314
chr1_12120	p2	(TG)16	32	69787819	69787850
chr1_12121	p2	(TA)8	16	69792356	69792371
chr1_12122	p2	(TG)14	28	69794927	69794954
chr1_12123	p2	(TC)7	14	69799949	69799962
chr1_12124	p2	(GA)6	12	69800457	69800468
chr1_12125	p3	(AAG)7	21	69800954	69800974
chr1_12126	p2	(TA)6	12	69807214	69807225
chr1_12127	p2	(GT)7	14	69809346	69809359
chr1_12128	c	(CA)10c(CA)13	47	69816657	69816703
chr1_12129	p3	(CAC)6	18	69831335	69831352
chr1_12130	p2	(GT)14	28	69841112	69841139
chr1_12131	p2	(CA)7	14	69841749	69841762
chr1_12132	p2	(TG)16	32	69846079	69846110
chr1_12133	c	(TG)11g(GT)6	35	69847364	69847398
chr1_12134	p2	(AC)12	24	69874151	69874174
chr1_12135	p2	(GA)7	14	69875265	69875278
chr1_12136	p2	(TG)10	20	69883103	69883122
chr1_12137	c	gtcaatgcggtgcagtccgggtaaagcccagtgatgcacagaggcagtg:	102	69887831	69887932
chr1_12138	p2	(AC)6	12	69888502	69888513
chr1_12139	p2	(AC)8	16	69888677	69888692
chr1_12140	p2	(AC)6	12	69910696	69910707
chr1_12141	c	(GT)9g(GT)20gc(GT)7	75	69924686	69924760
chr1_12142	p2	(GA)7	14	69932038	69932051
chr1_12143	p2	(TC)6	12	69935367	69935378
chr1_12144	p2	(TG)6	12	69941294	69941305
chr1_12145	c	(GT)9(GA)8	34	69944849	69944882
chr1_12146	p3	(ATA)5	15	69949323	69949337
chr1_12147	p3	(TTG)5	15	69949535	69949549
chr1_12148	p2	(TG)6	12	69958542	69958553
chr1_12149	p2	(CT)8	16	69969459	69969474
chr1_12150	p2	(AG)7	14	69969603	69969616
chr1_12151	p2	(CA)9	18	69981445	69981462
chr1_12152	p2	(CA)13	26	69983887	69983912
chr1_12153	p2	(AG)7	14	69984817	69984830
chr1_12154	c	(GT)11(GA)14	50	69987408	69987457
chr1_12155	p2	(CA)20	40	69988629	69988668
chr1_12156	p2	(GT)6	12	69989189	69989200
chr1_12157	p2	(CA)17	34	69991595	69991628
chr1_12158	p2	(GA)8	16	70005859	70005874
chr1_12159	p5	(CTCTC)5	25	70015472	70015496
chr1_12160	p3	(AAG)5	15	70019921	70019935
chr1_12161	p2	(TG)9	18	70021430	70021447
chr1_12162	p3	(ATA)5	15	70029559	70029573
chr1_12163	p3	(GAG)5	15	70034138	70034152
chr1_12164	p2	(AC)16	32	70040881	70040912

chr1_12165	p3	(TAA)8	24	70046769	70046792
chr1_12166	p4	(CTAC)5	20	70076144	70076163
chr1_12167	p2	(TA)6	12	70081892	70081903
chr1_12168	p3	(TAA)5	15	70089330	70089344
chr1_12169	p2	(AT)8	16	70092909	70092924
chr1_12170	p2	(AC)6	12	70103814	70103825
chr1_12171	p4	(TCCA)5	20	70117102	70117121
chr1_12172	p2	(GT)6	12	70127530	70127541
chr1_12173	p2	(TG)11	22	70127665	70127686
chr1_12174	p2	(CA)6	12	70129414	70129425
chr1_12175	p2	(AC)6	12	70134813	70134824
chr1_12176	p3	(ATT)6	18	70141321	70141338
chr1_12177	p2	(AC)16	32	70142891	70142922
chr1_12178	p2	(GT)6	12	70146415	70146426
chr1_12179	p2	(AT)8	16	70147708	70147723
chr1_12180	p2	(TG)32	64	70148301	70148364
chr1_12181	p2	(TC)6	12	70170497	70170508
chr1_12182	p2	(GA)6	12	70171922	70171933
chr1_12183	c	[illegible]TGG)5ataaaaaaatatgtgcttaccttagttaacacacacatag(AC)	84	70192299	70192382
chr1_12184	p2	(GT)14	28	70206215	70206242
chr1_12185	p3	(ATT)6	18	70208389	70208406
chr1_12186	p2	(AC)7	14	70208512	70208525
chr1_12187	p2	(GT)6	12	70211637	70211648
chr1_12188	p2	(CA)7	14	70215356	70215369
chr1_12189	p2	(AC)6	12	70221389	70221400
chr1_12190	p2	(CA)8	16	70225554	70225569
chr1_12191	c	(CT)9t(TC)9(AC)8	53	70231198	70231250
chr1_12192	p3	(TCA)7	21	70236908	70236928
chr1_12193	p2	(GA)6	12	70242007	70242018
chr1_12194	p2	(AC)16	32	70257936	70257967
chr1_12195	p2	(CA)6	12	70268936	70268947
chr1_12196	p2	(AC)11	22	70271601	70271622
chr1_12197	p2	(AC)15	30	70301127	70301156
chr1_12198	p2	(GT)6	12	70315450	70315461
chr1_12199	p2	(TG)13	26	70338963	70338988
chr1_12200	p2	(CA)15	30	70341476	70341505
chr1_12201	p2	(AC)9	18	70344760	70344777
chr1_12202	p2	(CA)10	20	70349440	70349459
chr1_12203	p2	(TG)7	14	70357901	70357914
chr1_12204	p3	(TAT)5	15	70361184	70361198
chr1_12205	p2	(TC)7	14	70368430	70368443
chr1_12206	p2	(AC)15	30	70372867	70372896
chr1_12207	c	(CT)18(CA)15	66	70373046	70373111
chr1_12208	p2	(CA)13	26	70373356	70373381
chr1_12209	p2	(GT)6	12	70374324	70374335
chr1_12210	p2	(CA)7	14	70375171	70375184
chr1_12211	p3	(AAT)9	27	70377601	70377627
chr1_12212	p2	(TG)6	12	70389083	70389094
chr1_12213	p2	(TG)6	12	70389957	70389968
chr1_12214	p2	(TG)9	18	70393165	70393182
chr1_12215	p2	(TA)7	14	70399910	70399923
chr1_12216	c	(CA)8tatatatgtatgtat(ATCC)5	51	70400046	70400096

chr1_12217	c	(AT)13(GT)7(AT)6	52	70400252	70400303
chr1_12218	p3	(TTA)6	18	70416811	70416828
chr1_12219	p4	(TCTA)6	24	70426585	70426608
chr1_12220	p2	(GT)12	24	70460751	70460774
chr1_12221	p2	(TG)9	18	70465712	70465729
chr1_12222	p2	(AT)9	18	70466717	70466734
chr1_12223	p2	(TG)12	24	70467022	70467045
chr1_12224	p2	(AC)10	20	70474011	70474030
chr1_12225	p2	(AC)6	12	70480995	70481006
chr1_12226	p4	(GATG)7	28	70485012	70485039
chr1_12227	p2	(AC)11	22	70489348	70489369
chr1_12228	p2	(GT)6	12	70492784	70492795
chr1_12229	p2	(CA)6	12	70495213	70495224
chr1_12230	p2	(GT)9	18	70498518	70498535
chr1_12231	p2	(TG)11	22	70498638	70498659
chr1_12232	p5	(TTTCA)5	25	70499679	70499703
chr1_12233	p2	(AC)18	36	70500596	70500631
chr1_12234	p2	(TG)6	12	70502629	70502640
chr1_12235	p2	(GT)7	14	70502982	70502995
chr1_12236	p2	(TG)8	16	70504569	70504584
chr1_12237	p4	(ATGG)5	20	70507089	70507108
chr1_12238	p2	(AC)7	14	70507576	70507589
chr1_12239	p2	(AC)6	12	70513614	70513625
chr1_12240	p2	(TG)6	12	70547939	70547950
chr1_12241	p5	(AAATC)5	25	70548137	70548161
chr1_12242	p2	(AC)14	28	70564772	70564799
chr1_12243	p2	(GT)7	14	70565901	70565914
chr1_12244	p2	(TG)7	14	70567009	70567022
chr1_12245	p2	(TG)6	12	70579469	70579480
chr1_12246	p4	(ATGG)6	24	70590396	70590419
chr1_12247	c	(AC)9atatatatannn(TG)8	46	70593849	70593894
chr1_12248	p2	(CA)6	12	70595103	70595114
chr1_12249	p2	(CA)9	18	70597083	70597100
chr1_12250	p2	(GA)9	18	70604785	70604802
chr1_12251	p2	(GT)7	14	70607697	70607710
chr1_12252	p2	(TC)6	12	70610968	70610979
chr1_12253	c	[illegible]atcattcgtcacctgtgctgcaatcataactgcactatggggtcaacaggag[illegible]	110	70612961	70613070
chr1_12254	p4	(ATGG)6	24	70615779	70615802
chr1_12255	p3	(AGG)5	15	70626355	70626369
chr1_12256	p2	(GA)7	14	70626979	70626992
chr1_12257	p3	(ACA)5	15	70628298	70628312
chr1_12258	p2	(GT)8	16	70637242	70637257
chr1_12259	p2	(TA)6	12	70645662	70645673
chr1_12260	p2	(CA)6	12	70645809	70645820
chr1_12261	p2	(CA)13	26	70649458	70649483
chr1_12262	p2	(AC)12	24	70653019	70653042
chr1_12263	p2	(TG)6	12	70668718	70668729
chr1_12264	p2	(AC)8	16	70671635	70671650
chr1_12265	p2	(AC)11	22	70672274	70672295
chr1_12266	p2	(CA)6	12	70673272	70673283
chr1_12267	p2	(GT)16	32	70673560	70673591
chr1_12268	p2	(AC)11	22	70674635	70674656

chr1_12269	p2	(CA)8	16	70675017	70675032
chr1_12270	p2	(AC)17	34	70677338	70677371
chr1_12271	p2	(CA)6	12	70677484	70677495
chr1_12272	p2	(AC)7	14	70677687	70677700
chr1_12273	c	(TG)15g(GT)9	49	70683827	70683875
chr1_12274	p2	(CA)19	38	70689486	70689523
chr1_12275	c	(CT)7aacacacct(AC)12	47	70690013	70690059
chr1_12276	p2	(AC)7	14	70690164	70690177
chr1_12277	p2	(AC)8	16	70696226	70696241
chr1_12278	p2	(AC)6	12	70701208	70701219
chr1_12279	p2	(AG)8	16	70708730	70708745
chr1_12280	p2	(AG)6	12	70708881	70708892
chr1_12281	p3	(CAG)5	15	70711461	70711475
chr1_12282	p2	(TC)9	18	70718586	70718603
chr1_12283	p2	(CA)7	14	70718865	70718878
chr1_12284	p5	(GAGAG)10	50	70719938	70719987
chr1_12285	p2	(AG)6	12	70721458	70721469
chr1_12286	c	(CA)13aacacacagaa(AG)6	49	70721682	70721730
chr1_12287	p2	(CT)7	14	70724457	70724470
chr1_12288	p2	(AG)28	56	70726065	70726120
chr1_12289	p2	(TA)6	12	70729653	70729664
chr1_12290	p2	(CA)12	24	70733146	70733169
chr1_12291	p2	(TA)6	12	70737116	70737127
chr1_12292	p2	(CA)6	12	70745584	70745595
chr1_12293	p2	(AT)6	12	70749662	70749673
chr1_12294	p2	(AC)12	24	70750565	70750588
chr1_12295	p2	(CA)13	26	70760222	70760247
chr1_12296	p3	(ACA)8	24	70760787	70760810
chr1_12297	p2	(CA)6	12	70770891	70770902
chr1_12298	p2	(TC)9	18	70771797	70771814
chr1_12299	p2	(TG)8	16	70783084	70783099
chr1_12300	p4	(TAGA)5	20	70789065	70789084
chr1_12301	p3	(CTG)7	21	70797561	70797581
chr1_12302	p2	(TG)6	12	70803564	70803575
chr1_12303	p3	(TTA)11	33	70804950	70804982
chr1_12304	p2	(AG)10	20	70818773	70818792
chr1_12305	c	(AC)8attttatatacatatacat(AC)6	47	70823471	70823517
chr1_12306	p2	(TG)12	24	70826755	70826778
chr1_12307	p2	(TG)8	16	70826900	70826915
chr1_12308	p2	(AC)8	16	70828139	70828154
chr1_12309	p2	(CT)11	22	70828497	70828518
chr1_12310	p2	(GT)12	24	70833705	70833728
chr1_12311	p3	(TGC)6	18	70849241	70849258
chr1_12312	p3	(ATA)5	15	70851257	70851271
chr1_12313	p2	(CA)7	14	70853887	70853900
chr1_12314	p2	(TG)27	54	70857542	70857595
chr1_12315	p2	(CA)9	18	70859325	70859342
chr1_12316	p2	(TG)19	38	70860652	70860689
chr1_12317	p2	(AC)10	20	70869341	70869360
chr1_12318	p2	(GT)7	14	70888373	70888386
chr1_12319	p2	(TC)7	14	70891677	70891690
chr1_12320	p2	(TG)8	16	70902622	70902637

chr1_12321	p2	(CA)16	32	70902843	70902874
chr1_12322	p2	(AC)23	46	70909384	70909429
chr1_12323	p2	(TG)10	20	70912229	70912248
chr1_12324	p2	(AC)6	12	70919574	70919585
chr1_12325	p3	(TGG)7	21	70919789	70919809
chr1_12326	p2	(CA)6	12	70924381	70924392
chr1_12327	p2	(TG)6	12	70927542	70927553
chr1_12328	p2	(AC)6	12	70928238	70928249
chr1_12329	p2	(GT)6	12	70931566	70931577
chr1_12330	p2	(AC)6	12	70948799	70948810
chr1_12331	p3	(AAT)8	24	70949205	70949228
chr1_12332	c	caaatatcaaccaaacatttttcaatattgtagcagcttttcttcttacgctttga	98	70951247	70951344
chr1_12333	p2	(TG)6	12	70959894	70959905
chr1_12334	p2	(TG)14	28	70963690	70963717
chr1_12335	p2	(CA)6	12	70965449	70965460
chr1_12336	p3	(TAA)5	15	70974890	70974904
chr1_12337	p2	(TC)6	12	70976536	70976547
chr1_12338	p5	(AAATC)5	25	70996459	70996483
chr1_12339	p2	(CA)27	54	71001336	71001389
chr1_12340	p4	(AAGA)5	20	71004010	71004029
chr1_12341	p2	(AC)29	58	71005682	71005739
chr1_12342	c	(CA)16(TA)6	44	71014179	71014222
chr1_12343	p2	(TG)7	14	71015995	71016008
chr1_12344	c	(GA)9gg(GA)6	32	71018037	71018068
chr1_12345	p2	(TG)8	16	71026878	71026893
chr1_12346	c	tggcagacctatgggccattccttttgtctctgatcacacaagagacccatg	172	71029424	71029595
chr1_12347	p2	(TG)18	36	71031352	71031387
chr1_12348	p2	(CA)8	16	71038712	71038727
chr1_12349	p2	(TG)14	28	71048056	71048083
chr1_12350	p2	(AT)6	12	71051016	71051027
chr1_12351	p2	(AC)16	32	71060008	71060039
chr1_12352	p3	(TAG)5	15	71063441	71063455
chr1_12353	c	(CT)6(CA)10	32	71075873	71075904
chr1_12354	p2	(GT)12	24	71079258	71079281
chr1_12355	c	(AGG)5aaactgatgtcatgaaacta(GAT)6	53	71088150	71088202
chr1_12356	p2	(TG)6	12	71091681	71091692
chr1_12357	p2	(TG)7	14	71092277	71092290
chr1_12358	p2	(AG)11	22	71097489	71097510
chr1_12359	c	(CT)15(CA)14	58	71127039	71127096
chr1_12360	p2	(CA)6	12	71129123	71129134
chr1_12361	p2	(GT)6	12	71143611	71143622
chr1_12362	p2	(TG)35	70	71147674	71147743
chr1_12363	p2	(CA)6	12	71170172	71170183
chr1_12364	p2	(CA)6	12	71175484	71175495
chr1_12365	p2	(TC)7	14	71185291	71185304
chr1_12366	p2	(CA)12	24	71187324	71187347
chr1_12367	p3	(ATG)7	21	71189101	71189121
chr1_12368	c	(TG)11(CG)7	36	71203301	71203336
chr1_12369	p2	(TG)7	14	71217186	71217199
chr1_12370	p2	(TG)6	12	71221238	71221249
chr1_12371	p2	(GT)15	30	71224107	71224136
chr1_12372	p2	(AC)31	62	71243104	71243165

chr1_12373	p2	(TG)6	12	71246234	71246245
chr1_12374	p2	(TG)15	30	71246420	71246449
chr1_12375	p2	(CA)8	16	71248124	71248139
chr1_12376	p5	(TACCC)6	30	71255392	71255421
chr1_12377	p2	(TA)6	12	71263871	71263882
chr1_12378	p2	(CA)22	44	71265074	71265117
chr1_12379	p2	(TG)20	40	71281616	71281655
chr1_12380	p3	(TAA)7	21	71284881	71284901
chr1_12381	p2	(AT)7	14	71286675	71286688
chr1_12382	p3	(TCT)5	15	71323515	71323529
chr1_12383	p2	(TG)16	32	71329470	71329501
chr1_12384	p2	(AC)7	14	71338618	71338631
chr1_12385	p4	(TACC)6	24	71347384	71347407
chr1_12386	p2	(TG)6	12	71347908	71347919
chr1_12387	p2	(TG)20	40	71356483	71356522
chr1_12388	p2	(TG)16	32	71362603	71362634
chr1_12389	p2	(TC)18	36	71369005	71369040
chr1_12390	p2	(CA)9	18	71369688	71369705
chr1_12391	p2	(TG)6	12	71379166	71379177
chr1_12392	p2	(AC)6	12	71381138	71381149
chr1_12393	p2	(TG)16	32	71383264	71383295
chr1_12394	p3	(TTA)7	21	71387510	71387530
chr1_12395	p5	(TTGAT)6	30	71387865	71387894
chr1_12396	p2	(GT)18	36	71389401	71389436
chr1_12397	p2	(TG)11	22	71389818	71389839
chr1_12398	p2	(TG)13	26	71396878	71396903
chr1_12399	p3	(ATT)5	15	71412998	71413012
chr1_12400	p2	(CA)6	12	71422570	71422581
chr1_12401	p2	(AC)8	16	71422930	71422945
chr1_12402	c	(CA)10tatctatatc(TA)7	44	71424072	71424115
chr1_12403	p4	(GGAG)5	20	71427525	71427544
chr1_12404	p2	(AC)6	12	71427788	71427799
chr1_12405	p2	(CT)6	12	71444650	71444661
chr1_12406	p3	(TGA)5	15	71447900	71447914
chr1_12407	p3	(ATA)5	15	71451280	71451294
chr1_12408	p2	(CA)8	16	71453329	71453344
chr1_12409	p3	(ATT)5	15	71454988	71455002
chr1_12410	p2	(GT)6	12	71465797	71465808
chr1_12411	p4	(GATG)11	44	71468895	71468938
chr1_12412	p2	(CA)7	14	71489224	71489237
chr1_12413	p2	(GT)9	18	71489780	71489797
chr1_12414	p2	(CA)7	14	71498654	71498667
chr1_12415	p2	(GT)8	16	71499202	71499217
chr1_12416	p4	(ATGG)5	20	71502802	71502821
chr1_12417	p3	(GAT)5	15	71509273	71509287
chr1_12418	c	(CA)7ggggggcctacaggagaa(AC)7	46	71513369	71513414
chr1_12419	p4	(ATGG)5	20	71525268	71525287
chr1_12420	p2	(TG)6	12	71530139	71530150
chr1_12421	p3	(CTG)7	21	71540637	71540657
chr1_12422	p3	(ATA)5	15	71549413	71549427
chr1_12423	p2	(GT)7	14	71601458	71601471
chr1_12424	p2	(AC)9	18	71601843	71601860

chr1_12425	p2	(TG)9	18	71606713	71606730
chr1_12426	p2	(TA)7	14	71614972	71614985
chr1_12427	p3	(ATA)5	15	71640129	71640143
chr1_12428	p4	(ATCC)5	20	71667605	71667624
chr1_12429	p2	(AC)6	12	71668456	71668467
chr1_12430	p3	(ATT)6	18	71685525	71685542
chr1_12431	p2	(TA)8	16	71688444	71688459
chr1_12432	p2	(AC)17	34	71689459	71689492
chr1_12433	p3	(CAA)5	15	71689714	71689728
chr1_12434	p2	(CA)11	22	71696587	71696608
chr1_12435	p2	(TG)6	12	71723170	71723181
chr1_12436	p2	(GT)7	14	71725597	71725610
chr1_12437	c	(GT)7gcgggcgtgtgtcg(GT)13	54	71732845	71732898
chr1_12438	p2	(AT)7	14	71741065	71741078
chr1_12439	p2	(AC)6	12	71745084	71745095
chr1_12440	p2	(TG)10	20	71747020	71747039
chr1_12441	p3	(ATC)26	78	71752359	71752436
chr1_12442	p2	(CA)6	12	71784931	71784942
chr1_12443	p3	(TTA)5	15	71791170	71791184
chr1_12444	p2	(AC)10	20	71795318	71795337
chr1_12445	p2	(CA)20	40	71802290	71802329
chr1_12446	p2	(TC)7	14	71806265	71806278
chr1_12447	p2	(CT)14	28	71808973	71809000
chr1_12448	p2	(AC)9	18	71812949	71812966
chr1_12449	p3	(TCC)5	15	71818411	71818425
chr1_12450	p2	(TG)8	16	71818998	71819013
chr1_12451	p3	(AAT)5	15	71822120	71822134
chr1_12452	p2	(CT)7	14	71842680	71842693
chr1_12453	p2	(GA)6	12	71843279	71843290
chr1_12454	p2	(GT)15	30	71875957	71875986
chr1_12455	p2	(CA)7	14	71893672	71893685
chr1_12456	p3	(ATA)5	15	71914231	71914245
chr1_12457	p2	(TG)36	72	71920369	71920440
chr1_12458	p2	(GT)6	12	71921503	71921514
chr1_12459	p2	(CA)12	24	71923295	71923318
chr1_12460	p4	(AATG)5	20	71932057	71932076
chr1_12461	p3	(AAT)5	15	71933088	71933102
chr1_12462	p2	(TG)10	20	71963304	71963323
chr1_12463	c	(GT)7(GA)7	28	71980560	71980587
chr1_12464	p2	(CT)7	14	71985668	71985681
chr1_12465	p2	(GT)20	40	71989288	71989327
chr1_12466	p3	(ATA)7	21	71989600	71989620
chr1_12467	p2	(TG)14	28	71991195	71991222
chr1_12468	p3	(GAT)6	18	72004783	72004800
chr1_12469	c*	(CTCA)9(CA)14*	62	72005879	72005940
chr1_12470	p2	(TG)6	12	72008680	72008691
chr1_12471	p2	(CT)16	32	72018124	72018155
chr1_12472	c	(TGT)6acagttgtttatgataaga(TG)8	53	72020920	72020972
chr1_12473	p2	(TG)17	34	72022343	72022376
chr1_12474	p3	(GAG)6	18	72037753	72037770
chr1_12475	p2	(TC)6	12	72051585	72051596
chr1_12476	p3	(ATA)5	15	72063050	72063064

chr1_12477	p3	(AGG)5	15	72065138	72065152
chr1_12478	p2	(TG)11	22	72068597	72068618
chr1_12479	p2	(AC)6	12	72074990	72075001
chr1_12480	p2	(CA)11	22	72076219	72076240
chr1_12481	p2	(GT)18	36	72079536	72079571
chr1_12482	p2	(GT)7	14	72087753	72087766
chr1_12483	p2	(GT)7	14	72120877	72120890
chr1_12484	p2	(AT)6	12	72126717	72126728
chr1_12485	p2	(AT)6	12	72126846	72126857
chr1_12486	p2	(CA)6	12	72131110	72131121
chr1_12487	p3	(ATA)10	30	72134462	72134491
chr1_12488	p2	(TG)9	18	72138137	72138154
chr1_12489	p2	(AC)6	12	72150467	72150478
chr1_12490	p2	(CA)6	12	72150594	72150605
chr1_12491	p2	(CA)7	14	72155561	72155574
chr1_12492	p2	(GT)19	38	72165834	72165871
chr1_12493	p2	(GT)10	20	72171690	72171709
chr1_12494	p2	(TG)10	20	72172464	72172483
chr1_12495	p4	(ATGG)5	20	72173314	72173333
chr1_12496	p2	(TG)7	14	72175092	72175105
chr1_12497	p3	(CAG)5	15	72175601	72175615
chr1_12498	p2	(CA)6	12	72180183	72180194
chr1_12499	p2	(GT)17	34	72181008	72181041
chr1_12500	p2	(TC)6	12	72182726	72182737
chr1_12501	c	(TG)7tact(TG)6	30	72189407	72189436
chr1_12502	p2	(CA)12	24	72201531	72201554
chr1_12503	p2	(AC)17	34	72201995	72202028
chr1_12504	p2	(GA)6	12	72206473	72206484
chr1_12505	p2	(GT)11	22	72227983	72228004
chr1_12506	p2	(GT)6	12	72230338	72230349
chr1_12507	p2	(TG)6	12	72241322	72241333
chr1_12508	c	(TG)7cgtgc(GT)8	35	72242155	72242189
chr1_12509	p2	(TG)25	50	72243676	72243725
chr1_12510	p2	(AC)9	18	72256936	72256953
chr1_12511	p2	(GT)17	34	72291451	72291484
chr1_12512	p2	(AC)19	38	72304896	72304933
chr1_12513	p2	(TG)10	20	72321774	72321793
chr1_12514	p2	(AC)13	26	72332208	72332233
chr1_12515	p3	(CTC)5	15	72340310	72340324
chr1_12516	p3	(CTC)5	15	72348956	72348970
chr1_12517	p2	(AC)6	12	72359612	72359623
chr1_12518	p2	(GT)8	16	72403331	72403346
chr1_12519	p2	(GA)6	12	72405905	72405916
chr1_12520	p2	(GA)7	14	72419184	72419197
chr1_12521	p2	(GT)6	12	72431262	72431273
chr1_12522	p2	(GA)10	20	72448462	72448481
chr1_12523	p2	(CA)11	22	72475923	72475944
chr1_12524	p2	(TA)13	26	72490696	72490721
chr1_12525	p2	(CA)7	14	72495478	72495491
chr1_12526	p2	(AC)13	26	72499184	72499209
chr1_12527	p2	(GT)7	14	72501683	72501696
chr1_12528	p2	(CA)6	12	72508169	72508180

chr1_12529	p2	(AT)6	12	72527004	72527015
chr1_12530	p4	(CTAT)6	24	72527128	72527151
chr1_12531	p3	(TTA)5	15	72528218	72528232
chr1_12532	p3	(ATC)6	18	72538277	72538294
chr1_12533	p2	(CA)12	24	72557624	72557647
chr1_12534	p2	(CA)10	20	72570775	72570794
chr1_12535	p2	(CA)6	12	72571005	72571016
chr1_12536	p2	(TG)8	16	72582666	72582681
chr1_12537	p2	(CA)6	12	72598437	72598448
chr1_12538	p2	(CA)6	12	72607010	72607021
chr1_12539	p2	(GT)7	14	72620509	72620522
chr1_12540	p2	(CA)18	36	72625067	72625102
chr1_12541	p2	(GT)9	18	72626072	72626089
chr1_12542	c	(ATA)7attt(TA)7	39	72629172	72629210
chr1_12543	p3	(TTA)5	15	72633090	72633104
chr1_12544	p3	(TAA)5	15	72644470	72644484
chr1_12545	p2	(CA)21	42	72645229	72645270
chr1_12546	p2	(TG)10	20	72651581	72651600
chr1_12547	p2	(TG)7	14	72658124	72658137
chr1_12548	p3	(ACC)6	18	72658351	72658368
chr1_12549	p5	(TTTGA)5	25	72662304	72662328
chr1_12550	p2	(AC)7	14	72668801	72668814
chr1_12551	p2	(TA)7	14	72683381	72683394
chr1_12552	p2	(TG)6	12	72685035	72685046
chr1_12553	c	[illegible]C)8ctctgcatcagtattcctttaacttttcttttttgtggctccctttgt(AC)[illegible]	104	72690011	72690114
chr1_12554	p2	(AC)7	14	72694958	72694971
chr1_12555	p2	(AT)8	16	72699961	72699976
chr1_12556	c	(CT)6ccctctccc(CT)23(CA)9	85	72724323	72724407
chr1_12557	p2	(TG)7	14	72731733	72731746
chr1_12558	p3	(CAG)6	18	72737718	72737735
chr1_12559	p2	(TG)20	40	72738424	72738463
chr1_12560	p2	(GT)9	18	72768122	72768139
chr1_12561	p2	(CT)6	12	72783667	72783678
chr1_12562	p2	(AC)6	12	72784415	72784426
chr1_12563	p2	(TG)32	64	72785046	72785109
chr1_12564	p2	(TA)7	14	72814323	72814336
chr1_12565	p2	(GT)9	18	72818335	72818352
chr1_12566	p4	(TTAT)8	32	72820307	72820338
chr1_12567	p2	(AC)6	12	72828609	72828620
chr1_12568	p3	(TGT)9	27	72832409	72832435
chr1_12569	p2	(TG)6	12	72850134	72850145
chr1_12570	p2	(CT)45	90	72855804	72855893
chr1_12571	p3	(TCT)5	15	72861863	72861877
chr1_12572	p3	(TAT)5	15	72863849	72863863
chr1_12573	p2	(AC)7	14	72863972	72863985
chr1_12574	p2	(GT)9	18	72875279	72875296
chr1_12575	p3	(TGT)7	21	72886452	72886472
chr1_12576	p2	(TG)24	48	72904528	72904575
chr1_12577	p2	(TA)8	16	72925646	72925661
chr1_12578	p2	(TG)6	12	72929365	72929376
chr1_12579	p2	(CA)19	38	72931125	72931162
chr1_12580	p2	(AT)9	18	72942753	72942770

chr1_12581	p2	(AC)6	12	72943117	72943128
chr1_12582	p2	(AC)6	12	72955305	72955316
chr1_12583	p2	(TG)14	28	72966431	72966458
chr1_12584	p2	(TG)6	12	72966878	72966889
chr1_12585	c	(CT)7gtctctctcaatctctctgagccccacgtgcat(AC)14	77	72967025	72967101
chr1_12586	c	(TA)7(TG)6	26	72992203	72992228
chr1_12587	p2	(TG)11	22	73007389	73007410
chr1_12588	c	(AC)10(AT)7	34	73013148	73013181
chr1_12589	p2	(AG)7	14	73041799	73041812
chr1_12590	p2	(TC)8	16	73049590	73049605
chr1_12591	p3	(TAT)6	18	73056538	73056555
chr1_12592	c	(GA)6a(AG)6	25	73060955	73060979
chr1_12593	p2	(TG)15	30	73064267	73064296
chr1_12594	p2	(TC)9	18	73066858	73066875
chr1_12595	p2	(GA)6	12	73070911	73070922
chr1_12596	p2	(TA)7	14	73072934	73072947
chr1_12597	p2	(CA)20	40	73077183	73077222
chr1_12598	p3	(TAG)6	18	73089193	73089210
chr1_12599	p2	(TC)7	14	73101998	73102011
chr1_12600	p3	(TCA)6	18	73104862	73104879
chr1_12601	p2	(TG)6	12	73120576	73120587
chr1_12602	p2	(CA)21	42	73127858	73127899
chr1_12603	p2	(GA)8	16	73147278	73147293
chr1_12604	c	tgagtgtaaactctttattattgttttaataaatgattttgatactggatgtttaatg	114	73153035	73153148
chr1_12605	p2	(TG)18	36	73154008	73154043
chr1_12606	p2	(AC)6	12	73156310	73156321
chr1_12607	p2	(AC)7	14	73161318	73161331
chr1_12608	p2	(AC)26	52	73169919	73169970
chr1_12609	c	CT)8caacacctcccattccaactttccacactaagttcagcagc(CT)1	89	73172845	73172933
chr1_12610	p2	(CA)8	16	73174427	73174442
chr1_12611	p6	(TCTTCA)5	30	73186744	73186773
chr1_12612	p2	(AC)6	12	73191690	73191701
chr1_12613	p4	(AATA)5	20	73198062	73198081
chr1_12614	p2	(AC)8	16	73198635	73198650
chr1_12615	p2	(AT)7	14	73214840	73214853
chr1_12616	p3	(TTA)5	15	73215977	73215991
chr1_12617	p2	(GT)14	28	73235855	73235882
chr1_12618	p2	(TG)8	16	73242550	73242565
chr1_12619	p2	(CA)7	14	73268243	73268256
chr1_12620	p2	(TG)8	16	73294927	73294942
chr1_12621	p2	(TG)10	20	73295518	73295537
chr1_12622	p2	(AG)7	14	73322268	73322281
chr1_12623	p2	(TA)6	12	73326313	73326324
chr1_12624	p2	(CA)8	16	73326868	73326883
chr1_12625	p2	(TC)6	12	73337026	73337037
chr1_12626	p2	(GT)9	18	73339750	73339767
chr1_12627	p2	(TC)7	14	73360262	73360275
chr1_12628	p2	(CA)14	28	73384537	73384564
chr1_12629	p2	(TG)6	12	73397076	73397087
chr1_12630	p5	(ATTTG)5	25	73401123	73401147
chr1_12631	p2	(TG)6	12	73405167	73405178
chr1_12632	p4	(AATC)5	20	73420949	73420968

chr1_12633	p2	(TG)14	28	73422044	73422071
chr1_12634	p2	(CA)6	12	73427303	73427314
chr1_12635	c	(AT)7acag(TA)6	30	73439519	73439548
chr1_12636	p3	(TTG)6	18	73450761	73450778
chr1_12637	c	(CT)7ggtgatgctctgctattaaag(CT)7	49	73460325	73460373
chr1_12638	p4	(AAGA)5	20	73474334	73474353
chr1_12639	p3	(TTG)5	15	73478017	73478031
chr1_12640	p2	(CA)40	80	73492045	73492124
chr1_12641	c	tctgttctgttaatggtgcagaaaatcatgactgtgtttgcccagtgaaacgtg	129	73511028	73511156
chr1_12642	p2	(CT)13	26	73519816	73519841
chr1_12643	c	(TC)9actctctctcc(CT)7(CA)15	73	73530966	73531038
chr1_12644	p2	(AC)21	42	73535023	73535064
chr1_12645	p2	(GT)8	16	73536128	73536143
chr1_12646	p2	(AC)13	26	73567877	73567902
chr1_12647	p2	(CA)11	22	73568034	73568055
chr1_12648	p2	(CA)17	34	73570800	73570833
chr1_12649	p4	(TTTC)6	24	73575598	73575621
chr1_12650	p2	(CA)12	24	73583929	73583952
chr1_12651	p2	(TG)6	12	73584143	73584154
chr1_12652	p2	(CA)14	28	73609624	73609651
chr1_12653	p2	(CA)15	30	73610013	73610042
chr1_12654	p2	(GT)13	26	73622769	73622794
chr1_12655	p2	(GT)22	44	73630352	73630395
chr1_12656	p2	(AG)6	12	73634019	73634030
chr1_12657	p2	(GT)15	30	73641321	73641350
chr1_12658	p2	(TG)7	14	73643727	73643740
chr1_12659	p2	(AC)7	14	73644087	73644100
chr1_12660	p2	(AC)8	16	73648873	73648888
chr1_12661	p2	(AC)8	16	73649034	73649049
chr1_12662	p2	(TG)17	34	73649956	73649989
chr1_12663	p3	(TTA)8	24	73650731	73650754
chr1_12664	p2	(AC)9	18	73654693	73654710
chr1_12665	c	caatcag(CA)9aaacaacataaatcagcacacacacaaaacaaaatca	113	73656633	73656745
chr1_12666	p3	(AGC)5	15	73658701	73658715
chr1_12667	p3	(AGA)6	18	73668488	73668505
chr1_12668	p2	(TG)12	24	73673025	73673048
chr1_12669	p2	(CA)6	12	73685138	73685149
chr1_12670	c	(TG)9ta(TG)10	40	73686272	73686311
chr1_12671	p3	(CAA)6	18	73688539	73688556
chr1_12672	p2	(CA)18	36	73689120	73689155
chr1_12673	p2	(AC)6	12	73692874	73692885
chr1_12674	p2	(CA)6	12	73702123	73702134
chr1_12675	p2	(AC)11	22	73702444	73702465
chr1_12676	c	G)6aaagagagagagagaatacagtcctgaaggcagagatatgagt(GA)	83	73714819	73714901
chr1_12677	p2	(TG)18	36	73720195	73720230
chr1_12678	p3	(TAT)7	21	73761097	73761117
chr1_12679	p4	(ACAG)7	28	73764691	73764718
chr1_12680	p2	(TG)7	14	73775830	73775843
chr1_12681	p2	(TG)10	20	73784274	73784293
chr1_12682	p2	(AC)6	12	73784853	73784864
chr1_12683	p2	(AC)10	20	73785939	73785958
chr1_12684	p2	(TG)6	12	73786802	73786813

chr1_12685	p3	(CAA)5	15	73791063	73791077
chr1_12686	p2	(AC)6	12	73795948	73795959
chr1_12687	p2	(TG)12	24	73799854	73799877
chr1_12688	p2	(GT)7	14	73808290	73808303
chr1_12689	p2	(AC)12	24	73810710	73810733
chr1_12690	c	(AC)6aagcatacatgagcagtgatacaa(AC)8	52	73811828	73811879
chr1_12691	p3	(ATA)6	18	73835257	73835274
chr1_12692	p2	(AG)6	12	73859758	73859769
chr1_12693	p3	(AAG)5	15	73864436	73864450
chr1_12694	p2	(TG)8	16	73869600	73869615
chr1_12695	p3	(TCT)6	18	73870906	73870923
chr1_12696	p2	(AC)10	20	73880583	73880602
chr1_12697	p2	(GA)7	14	73885470	73885483
chr1_12698	p2	(TG)19	38	73900142	73900179
chr1_12699	p2	(TG)23	46	73905375	73905420
chr1_12700	p3	(AAT)5	15	73908473	73908487
chr1_12701	p2	(CA)26	52	73910560	73910611
chr1_12702	p3	(AAC)5	15	73910980	73910994
chr1_12703	p2	(AC)7	14	73917711	73917724
chr1_12704	p2	(GT)6	12	73921175	73921186
chr1_12705	p2	(GT)7	14	73921420	73921433
chr1_12706	p2	(AC)8	16	73923513	73923528
chr1_12707	c	(TG)6caggcccgagaggaatcactgtttcaatttgtccagcc(AG)7	64	73928313	73928376
chr1_12708	c	(GA)12(GT)26	76	73932504	73932579
chr1_12709	p2	(AC)6	12	73932867	73932878
chr1_12710	p5	(GGAGA)7	35	73956372	73956406
chr1_12711	p3	(CTG)5	15	73976707	73976721
chr1_12712	p2	(TG)8	16	73977673	73977688
chr1_12713	p2	(CA)9	18	73984021	73984038
chr1_12714	p3	(CTC)5	15	73999934	73999948
chr1_12715	p2	(GT)6	12	74001821	74001832
chr1_12716	p2	(TG)10	20	74001989	74002008
chr1_12717	p2	(TG)16	32	74003433	74003464
chr1_12718	p2	(TG)16	32	74004070	74004101
chr1_12719	p2	(TG)6	12	74005858	74005869
chr1_12720	p2	(GT)8	16	74007874	74007889
chr1_12721	p2	(AC)16	32	74011497	74011528
chr1_12722	p2	(CA)6	12	74017463	74017474
chr1_12723	p2	(TG)6	12	74018877	74018888
chr1_12724	p2	(TG)6	12	74019007	74019018
chr1_12725	p2	(TG)11	22	74028995	74029016
chr1_12726	p2	(TG)27	54	74029159	74029212
chr1_12727	p2	(GT)27	54	74031505	74031558
chr1_12728	p2	(TG)8	16	74043426	74043441
chr1_12729	p3	(GAT)15	45	74049723	74049767
chr1_12730	p2	(AC)26	52	74053780	74053831
chr1_12731	p2	(CA)8	16	74054521	74054536
chr1_12732	p2	(AC)9	18	74057883	74057900
chr1_12733	p3	(CCT)6	18	74062274	74062291
chr1_12734	p2	(AC)7	14	74062735	74062748
chr1_12735	c	(TAA)8tttttaaattagtg(TAT)6	56	74062887	74062942
chr1_12736	p2	(TG)11	22	74064393	74064414

chr1_12737	p3	(TAA)6	18	74067391	74067408
chr1_12738	p2	(AC)9	18	74068718	74068735
chr1_12739	p3	(CTT)5	15	74069787	74069801
chr1_12740	p2	(AC)21	42	74070205	74070246
chr1_12741	p2	(GT)12	24	74072371	74072394
chr1_12742	p2	(TC)6	12	74072611	74072622
chr1_12743	p2	(TC)14	28	74089154	74089181
chr1_12744	p2	(GT)6	12	74094673	74094684
chr1_12745	p2	(TG)9	18	74095211	74095228
chr1_12746	p2	(CA)6	12	74096373	74096384
chr1_12747	p2	(AC)9	18	74096494	74096511
chr1_12748	p3	(TAA)5	15	74103812	74103826
chr1_12749	p2	(TG)15	30	74104810	74104839
chr1_12750	p2	(TG)24	48	74110003	74110050
chr1_12751	p2	(CA)7	14	74113367	74113380
chr1_12752	p2	(CA)16	32	74115659	74115690
chr1_12753	p2	(TC)18	36	74118421	74118456
chr1_12754	p2	(CA)31	62	74124054	74124115
chr1_12755	p2	(CA)13	26	74126857	74126882
chr1_12756	c*	tatgtaatttctccttgttcactagaatctggaca(CT)8(CTCA)5*ctc	235	74131469	74131703
chr1_12757	p2	(GT)13	26	74131872	74131897
chr1_12758	p2	(TG)14	28	74132186	74132213
chr1_12759	p3	(AGA)10	30	74137381	74137410
chr1_12760	p2	(TG)14	28	74140616	74140643
chr1_12761	p2	(CA)18	36	74141358	74141393
chr1_12762	p2	(GA)8	16	74149798	74149813
chr1_12763	p2	(TC)6	12	74150058	74150069
chr1_12764	p2	(AC)8	16	74154039	74154054
chr1_12765	p2	(AC)22	44	74155217	74155260
chr1_12766	p4	(TCCA)6	24	74157240	74157263
chr1_12767	p2	(TG)11	22	74157724	74157745
chr1_12768	p2	(TG)8	16	74159892	74159907
chr1_12769	p2	(TG)23	46	74165392	74165437
chr1_12770	p2	(GT)10	20	74166511	74166530
chr1_12771	p2	(AC)8	16	74174432	74174447
chr1_12772	p2	(TG)10	20	74177398	74177417
chr1_12773	p2	(AC)15	30	74177718	74177747
chr1_12774	p2	(AG)7	14	74189117	74189130
chr1_12775	p2	(TA)6	12	74193067	74193078
chr1_12776	p2	(GT)16	32	74195401	74195432
chr1_12777	p2	(CA)10	20	74196408	74196427
chr1_12778	p2	(TC)7	14	74197100	74197113
chr1_12779	p2	(GA)6	12	74208882	74208893
chr1_12780	p2	(TG)6	12	74215329	74215340
chr1_12781	c	tg(TATC)8tatatatnnnnnnnnnnnnnnnnnnnnnnnnnnn	102	74215591	74215692
chr1_12782	p2	(GT)13	26	74216471	74216496
chr1_12783	p3	(AGC)5	15	74222881	74222895
chr1_12784	p2	(AT)10	20	74237452	74237471
chr1_12785	p2	(AT)10	20	74238351	74238370
chr1_12786	c	CA)9ctccaaacttaatataacatggcctcaactacccccgtaaat(AC)1	80	74239253	74239332
chr1_12787	p2	(TG)10	20	74241657	74241676
chr1_12788	p2	(GT)14	28	74244345	74244372

chr1_12789	p2	(AC)9	18	74251334	74251351
chr1_12790	p3	(TAT)6	18	74252063	74252080
chr1_12791	p2	(AC)6	12	74254226	74254237
chr1_12792	p3	(CAT)7	21	74264697	74264717
chr1_12793	p2	(CA)9	18	74264900	74264917
chr1_12794	p2	(CA)6	12	74265926	74265937
chr1_12795	p2	(AC)17	34	74268164	74268197
chr1_12796	p2	(CT)6	12	74268535	74268546
chr1_12797	p2	(CT)8	16	74270136	74270151
chr1_12798	p2	(TG)7	14	74280596	74280609
chr1_12799	c	(CT)7ttctctctttctatgtct(CA)10	52	74281437	74281488
chr1_12800	p2	(TA)6	12	74291624	74291635
chr1_12801	p2	(GT)14	28	74298780	74298807
chr1_12802	p2	(TG)17	34	74305068	74305101
chr1_12803	p2	(AC)8	16	74316587	74316602
chr1_12804	c	(AC)7aa(AT)9gtat(AC)10	58	74330015	74330072
chr1_12805	c	(GT)7(GA)13	40	74344475	74344514
chr1_12806	p5	(GATTT)5	25	74344660	74344684
chr1_12807	c*	(CTG)5(TG)14*	41	74363941	74363981
chr1_12808	p2	(CT)9	18	74365962	74365979
chr1_12809	p2	(CA)12	24	74371884	74371907
chr1_12810	p2	(GT)17	34	74375646	74375679
chr1_12811	p2	(GT)9	18	74381246	74381263
chr1_12812	p2	(TG)9	18	74381426	74381443
chr1_12813	p2	(TG)6	12	74381589	74381600
chr1_12814	p2	(TC)8	16	74382013	74382028
chr1_12815	p2	(TG)6	12	74385824	74385835
chr1_12816	p2	(TG)6	12	74406647	74406658
chr1_12817	p2	(TC)7	14	74411607	74411620
chr1_12818	p2	(GT)11	22	74416335	74416356
chr1_12819	p2	(CA)26	52	74420574	74420625
chr1_12820	p2	(TG)11	22	74422471	74422492
chr1_12821	c	(TTG)5tatgtgcagaaggactttttgtg(CA)6	50	74433894	74433943
chr1_12822	c	(CA)11cgcg(CA)8	42	74443403	74443444
chr1_12823	p2	(AC)7	14	74445724	74445737
chr1_12824	p2	(GA)6	12	74454621	74454632
chr1_12825	p4	(TCAT)5	20	74457258	74457277
chr1_12826	p2	(TG)6	12	74466847	74466858
chr1_12827	p3	(ATT)5	15	74469384	74469398
chr1_12828	p3	(TTG)6	18	74475861	74475878
chr1_12829	p2	(TG)6	12	74482455	74482466
chr1_12830	p2	(TG)6	12	74482968	74482979
chr1_12831	p3	(GAA)5	15	74489779	74489793
chr1_12832	p2	(TG)16	32	74508707	74508738
chr1_12833	c	(GT)6a(TG)16	45	74510250	74510294
chr1_12834	p2	(GT)6	12	74516473	74516484
chr1_12835	p2	(CA)6	12	74520962	74520973
chr1_12836	p3	(ATT)6	18	74532550	74532567
chr1_12837	p3	(GGA)5	15	74551439	74551453
chr1_12838	p2	(GT)6	12	74568899	74568910
chr1_12839	p3	(TAT)7	21	74577616	74577636
chr1_12840	c	tcgcccccacagacacaaactgacggagaagttgattagaagcagctcta	143	74583603	74583745

chr1_12841	p2	(AC)25	50	74584102	74584151
chr1_12842	c	(GA)6gggagagagaaagagaa(AG)7	43	74589033	74589075
chr1_12843	p2	(CA)22	44	74590394	74590437
chr1_12844	p2	(CA)15	30	74591797	74591826
chr1_12845	p2	(CA)19	38	74594032	74594069
chr1_12846	p2	(CA)13	26	74594554	74594579
chr1_12847	p3	(ACT)6	18	74601747	74601764
chr1_12848	p2	(GT)9	18	74602602	74602619
chr1_12849	p3	(CTC)5	15	74612964	74612978
chr1_12850	p2	(CA)6	12	74613083	74613094
chr1_12851	p2	(GT)6	12	74618088	74618099
chr1_12852	p3	(AAT)8	24	74623455	74623478
chr1_12853	p4	(GTCA)5	20	74624793	74624812
chr1_12854	c	(AC)13aa(AC)6	40	74632538	74632577
chr1_12855	p2	(AC)15	30	74632685	74632714
chr1_12856	p2	(AC)9	18	74634379	74634396
chr1_12857	p2	(TG)17	34	74634897	74634930
chr1_12858	p2	(TC)7	14	74635530	74635543
chr1_12859	p2	(CA)9	18	74640449	74640466
chr1_12860	p2	(GT)6	12	74640578	74640589
chr1_12861	p2	(TG)9	18	74643631	74643648
chr1_12862	c	(TG)14(AG)8	44	74646287	74646330
chr1_12863	p2	(AC)20	40	74648218	74648257
chr1_12864	p2	(GT)10	20	74656327	74656346
chr1_12865	p2	(AT)6	12	74656972	74656983
chr1_12866	p2	(GT)9	18	74662903	74662920
chr1_12867	p2	(TG)13	26	74668617	74668642
chr1_12868	p2	(GT)9	18	74670481	74670498
chr1_12869	p2	(TG)12	24	74670637	74670660
chr1_12870	p2	(TG)7	14	74679439	74679452
chr1_12871	c	(CA)6cctcaaactatctctgaaacacagacatt(CA)8	57	74679838	74679894
chr1_12872	p2	(TG)20	40	74683337	74683376
chr1_12873	p2	(CA)13	26	74684911	74684936
chr1_12874	p2	(TG)49	98	74686838	74686935
chr1_12875	p3	(CTT)6	18	74690809	74690826
chr1_12876	c	(TG)7agcccag(GT)6	33	74691504	74691536
chr1_12877	p3	(CTC)5	15	74705644	74705658
chr1_12878	p2	(GT)6	12	74710536	74710547
chr1_12879	p2	(TG)13	26	74715505	74715530
chr1_12880	p2	(CA)9	18	74720729	74720746
chr1_12881	p2	(AC)12	24	74720859	74720882
chr1_12882	p2	(AC)6	12	74721000	74721011
chr1_12883	p2	(CA)6	12	74721661	74721672
chr1_12884	p2	(TG)8	16	74725008	74725023
chr1_12885	p2	(GT)12	24	74726635	74726658
chr1_12886	p2	(TG)6	12	74726782	74726793
chr1_12887	c	(TG)16gc(TG)6	46	74729796	74729841
chr1_12888	p2	(CT)6	12	74732132	74732143
chr1_12889	p2	(AC)9	18	74732951	74732968
chr1_12890	p2	(AC)16	32	74755905	74755936
chr1_12891	p2	(CA)6	12	74757439	74757450
chr1_12892	c	(AC)14(AT)10	48	74762175	74762222

chr1_12893	c	cgtcactggctctctcaggtctgtgtgtgttagacagttcattcatctcacatg	101	74763383	74763483
chr1_12894	c	(CT)6tacatacactattacacagacatga(AC)7	51	74763622	74763672
chr1_12895	p2	(GT)24	48	74763821	74763868
chr1_12896	p2	(TG)20	40	74763979	74764018
chr1_12897	p2	(TG)7	14	74765391	74765404
chr1_12898	p2	(TC)6	12	74767478	74767489
chr1_12899	p2	(TC)12	24	74768333	74768356
chr1_12900	c	(GT)6a(TG)8	29	74770278	74770306
chr1_12901	p3	(CCT)6	18	74773700	74773717
chr1_12902	p2	(CT)9	18	74774083	74774100
chr1_12903	p2	(AC)11	22	74787897	74787918
chr1_12904	p3	(CTC)5	15	74788741	74788755
chr1_12905	c	(AT)6ctatatacatgcacacagacacag(AC)12	60	74790169	74790228
chr1_12906	p2	(AG)6	12	74794661	74794672
chr1_12907	p2	(TG)7	14	74797412	74797425
chr1_12908	p3	(TCT)5	15	74799363	74799377
chr1_12909	p2	(CA)25	50	74811430	74811479
chr1_12910	p2	(CA)10	20	74811680	74811699
chr1_12911	p2	(TG)14	28	74814796	74814823
chr1_12912	p2	(AC)6	12	74818552	74818563
chr1_12913	p2	(CA)12	24	74822121	74822144
chr1_12914	p2	(CA)8	16	74829163	74829178
chr1_12915	p2	(CA)8	16	74831393	74831408
chr1_12916	p2	(TG)6	12	74839293	74839304
chr1_12917	p3	(AGA)6	18	74843464	74843481
chr1_12918	p2	(TG)6	12	74848958	74848969
chr1_12919	p2	(TG)6	12	74874321	74874332
chr1_12920	c	(AC)10aatgttagaacagcatcttc(CT)8(CA)10	76	74893390	74893465
chr1_12921	p2	(AC)7	14	74912141	74912154
chr1_12922	p2	(TC)7	14	74912859	74912872
chr1_12923	p2	(TG)18	36	74915931	74915966
chr1_12924	p2	(AC)11	22	74923458	74923479
chr1_12925	p2	(TG)6	12	74924360	74924371
chr1_12926	p2	(TG)6	12	74931933	74931944
chr1_12927	p2	(CA)10	20	74952691	74952710
chr1_12928	p2	(TG)6	12	74955707	74955718
chr1_12929	p2	(AC)24	48	74960342	74960389
chr1_12930	p2	(TG)6	12	74982758	74982769
chr1_12931	p3	(TTC)5	15	74988265	74988279
chr1_12932	p2	(AC)15	30	75019807	75019836
chr1_12933	p2	(CA)6	12	75020889	75020900
chr1_12934	p2	(AC)13	26	75049432	75049457
chr1_12935	p2	(CA)6	12	75050666	75050677
chr1_12936	p2	(CA)8	16	75054933	75054948
chr1_12937	p2	(AC)16	32	75056409	75056440
chr1_12938	p2	(AC)17	34	75056549	75056582
chr1_12939	p3	(GAG)5	15	75058698	75058712
chr1_12940	p2	(AC)7	14	75066616	75066629
chr1_12941	p2	(AT)6	12	75067899	75067910
chr1_12942	p2	(CA)16	32	75074186	75074217
chr1_12943	p2	(TG)12	24	75075038	75075061
chr1_12944	p2	(TC)7	14	75078821	75078834

chr1_12945	p2	(CT)14	28	75084643	75084670
chr1_12946	p2	(GT)13	26	75088409	75088434
chr1_12947	p2	(TG)6	12	75092207	75092218
chr1_12948	p2	(GT)26	52	75098675	75098726
chr1_12949	p2	(TG)21	42	75099118	75099159
chr1_12950	p2	(TG)23	46	75099296	75099341
chr1_12951	p2	(TG)10	20	75103770	75103789
chr1_12952	p2	(AC)11	22	75106156	75106177
chr1_12953	p2	(TG)23	46	75106661	75106706
chr1_12954	p2	(TG)6	12	75110020	75110031
chr1_12955	p2	(CA)13	26	75110357	75110382
chr1_12956	p2	(AC)6	12	75116101	75116112
chr1_12957	p2	(TG)18	36	75116686	75116721
chr1_12958	p2	(GT)21	42	75118801	75118842
chr1_12959	p2	(TG)19	38	75123579	75123616
chr1_12960	p2	(TG)9	18	75123785	75123802
chr1_12961	c	ttgtagctgtcatttgtcctcataattttcaggcagatcatcctgaatccctgtg	190	75132554	75132743
chr1_12962	p4	(TTAT)5	20	75135866	75135885
chr1_12963	c	})7gagtaaccttttccttcagcgacttccataatttgatcttcactttctgc(T	76	75139436	75139511
chr1_12964	p3	(CTT)5	15	75144508	75144522
chr1_12965	p2	(CA)16	32	75145043	75145074
chr1_12966	c	GT)7gcgcctttcagtgtgtttgtgcacacttcagctcactgtacag(GT)	71	75146872	75146942
chr1_12967	p2	(TG)12	24	75158017	75158040
chr1_12968	p2	(CA)6	12	75177063	75177074
chr1_12969	p2	(GT)15	30	75180049	75180078
chr1_12970	p2	(GT)26	52	75189232	75189283
chr1_12971	p2	(AC)11	22	75197696	75197717
chr1_12972	p2	(TG)23	46	75198125	75198170
chr1_12973	p2	(TG)7	14	75200237	75200250
chr1_12974	c	(CA)9c(CA)9	37	75200451	75200487
chr1_12975	p2	(CA)6	12	75201643	75201654
chr1_12976	p2	(TG)6	12	75203237	75203248
chr1_12977	p2	(GT)6	12	75209293	75209304
chr1_12978	p2	(CA)6	12	75213592	75213603
chr1_12979	p3	(AAT)5	15	75215375	75215389
chr1_12980	p2	(AC)19	38	75215590	75215627
chr1_12981	p3	(GCT)5	15	75221651	75221665
chr1_12982	p2	(TC)6	12	75223677	75223688
chr1_12983	p2	(TG)21	42	75227730	75227771
chr1_12984	c	(CA)10cg(CA)10	42	75228036	75228077
chr1_12985	p2	(CA)9	18	75231155	75231172
chr1_12986	p2	(TC)6	12	75239308	75239319
chr1_12987	p2	(AC)15	30	75245585	75245614
chr1_12988	c	(TG)8(TC)9	34	75280522	75280555
chr1_12989	c	(AC)9ag(AC)7	34	75283403	75283436
chr1_12990	p2	(TG)12	24	75284187	75284210
chr1_12991	p2	(TG)17	34	75285883	75285916
chr1_12992	p2	(CA)19	38	75287841	75287878
chr1_12993	p2	(CA)6	12	75288422	75288433
chr1_12994	p2	(GT)10	20	75290236	75290255
chr1_12995	p2	(TG)16	32	75292890	75292921
chr1_12996	p2	(CA)6	12	75300358	75300369

chr1_12997	p2	(CA)11	22	75309513	75309534
chr1_12998	p2	(CA)8	16	75314539	75314554
chr1_12999	p3	(CTG)5	15	75327773	75327787
chr1_13000	p2	(GT)7	14	75334211	75334224
chr1_13001	p3	(GAG)5	15	75355135	75355149
chr1_13002	p2	(CA)12	24	75355255	75355278
chr1_13003	p2	(TG)6	12	75367653	75367664
chr1_13004	p2	(GT)18	36	75374744	75374779
chr1_13005	p2	(CT)13	26	75391257	75391282
chr1_13006	p2	(CT)13	26	75393766	75393791
chr1_13007	p2	(AC)8	16	75395522	75395537
chr1_13008	c	(CT)14(CA)7	42	75395933	75395974
chr1_13009	p2	(AC)8	16	75397813	75397828
chr1_13010	p2	(GT)17	34	75403475	75403508
chr1_13011	p3	(CAT)6	18	75407900	75407917
chr1_13012	p3	(TCC)6	18	75408562	75408579
chr1_13013	c	;tggtgttaggcgtcccaagcgaaatcgtacggctggaagtcgtctctcatc	111	75424117	75424227
chr1_13014	p3	(TTC)5	15	75433765	75433779
chr1_13015	p2	(GT)25	50	75437002	75437051
chr1_13016	p2	(AC)6	12	75437744	75437755
chr1_13017	p3	(CCT)6	18	75438325	75438342
chr1_13018	p2	(AC)7	14	75439453	75439466
chr1_13019	p2	(TG)9	18	75445748	75445765
chr1_13020	p5	(CTGCT)5	25	75459981	75460005
chr1_13021	p2	(TG)6	12	75484556	75484567
chr1_13022	p4	(CCAT)5	20	75486359	75486378
chr1_13023	p2	(GT)6	12	75500272	75500283
chr1_13024	p2	(GT)7	14	75507547	75507560
chr1_13025	p2	(GA)11	22	75509977	75509998
chr1_13026	p2	(GT)6	12	75512170	75512181
chr1_13027	p2	(AC)6	12	75518758	75518769
chr1_13028	p3	(AGC)6	18	75518895	75518912
chr1_13029	p3	(CAA)5	15	75521240	75521254
chr1_13030	p5	(TAAAA)5	25	75527747	75527771
chr1_13031	p2	(CT)6	12	75532237	75532248
chr1_13032	p2	(TC)6	12	75538604	75538615
chr1_13033	p3	(AAT)6	18	75546461	75546478
chr1_13034	p2	(AC)8	16	75555786	75555801
chr1_13035	p5	(TGCTC)5	25	75558019	75558043
chr1_13036	p3	(TTG)8	24	75580766	75580789
chr1_13037	p2	(GT)6	12	75582940	75582951
chr1_13038	p2	(TG)11	22	75585055	75585076
chr1_13039	p2	(CA)10	20	75586847	75586866
chr1_13040	c	;ggagagagggacacactacgg(GA)6ggacacattatgg(GA)6aca	401	75591344	75591744
chr1_13041	p3	(TGA)7	21	75592068	75592088
chr1_13042	p3	(AGG)5	15	75593539	75593553
chr1_13043	p2	(TG)7	14	75603690	75603703
chr1_13044	p2	(TA)7	14	75620017	75620030
chr1_13045	p2	(TA)6	12	75621172	75621183
chr1_13046	c	(TG)8ta(TG)7	32	75625556	75625587
chr1_13047	p3	(GAG)9	27	75631967	75631993
chr1_13048	p2	(CT)8	16	75635467	75635482

chr1_13049	p2	(GT)20	40	75636539	75636578
chr1_13050	p2	(GT)16	32	75637091	75637122
chr1_13051	c	(CT)9cccatactgtgtcctctt(TC)6	48	75647365	75647412
chr1_13052	p2	(GT)8	16	75657605	75657620
chr1_13053	p2	(TG)13	26	75658815	75658840
chr1_13054	p3	(ATT)5	15	75666216	75666230
chr1_13055	p3	(ATA)8	24	75671461	75671484
chr1_13056	p2	(CA)6	12	75683080	75683091
chr1_13057	p3	(CTC)5	15	75687827	75687841
chr1_13058	p3	(ATA)7	21	75702726	75702746
chr1_13059	c	(TAT)8tg(TTA)6	44	75705584	75705627
chr1_13060	p3	(TTA)5	15	75709128	75709142
chr1_13061	p2	(GT)6	12	75724384	75724395
chr1_13062	p2	(AC)13	26	75731096	75731121
chr1_13063	p2	(GT)19	38	75734009	75734046
chr1_13064	p4	(TCCA)5	20	75747967	75747986
chr1_13065	p2	(AG)6	12	75764933	75764944
chr1_13066	p3	(GAG)5	15	75768914	75768928
chr1_13067	p2	(TG)8	16	75769281	75769296
chr1_13068	p2	(AC)15	30	75786643	75786672
chr1_13069	p2	(GT)17	34	75787753	75787786
chr1_13070	p2	(TA)6	12	75830957	75830968
chr1_13071	p2	(TG)8	16	75831514	75831529
chr1_13072	p5	(TCAAA)5	25	75838026	75838050
chr1_13073	p2	(TG)21	42	75843689	75843730
chr1_13074	p2	(TG)6	12	75846183	75846194
chr1_13075	p2	(AC)15	30	75850970	75850999
chr1_13076	p3	(CAG)5	15	75861906	75861920
chr1_13077	p2	(CA)8	16	75891263	75891278
chr1_13078	p2	(TA)10	20	75895953	75895972
chr1_13079	p3	(AGC)5	15	75903877	75903891
chr10_1	p3	(TTG)7	21	22196	22216
chr10_2	p3	(GAA)6	18	63142	63159
chr10_3	p2	(AT)7	14	68735	68748
chr10_4	p2	(AC)15	30	73056	73085
chr10_5	p2	(AT)7	14	79348	79361
chr10_6	p3	(GCT)5	15	83054	83068
chr10_7	p4	(TAGA)7	28	131486	131513
chr10_8	p2	(TG)6	12	136246	136257
chr10_9	p2	(AC)8	16	147500	147515
chr10_10	p2	(TG)9	18	151057	151074
chr10_11	p2	(GT)6	12	156053	156064
chr10_12	p3	(GAA)5	15	156260	156274
chr10_13	p2	(TG)6	12	156384	156395
chr10_14	p2	(GT)14	28	161394	161421
chr10_15	p2	(GT)8	16	168694	168709
chr10_16	p2	(AC)19	38	173880	173917
chr10_17	p2	(AC)13	26	183118	183143
chr10_18	p4	(TATC)14	56	186487	186542
chr10_19	p2	(AG)6	12	191688	191699
chr10_20	p2	(AT)7	14	192444	192457
chr10_21	p2	(AT)6	12	211842	211853

chr10_22	p2	(AG)6	12	214326	214337
chr10_23	p2	(GT)15	30	249713	249742
chr10_24	p2	(TC)8	16	250303	250318
chr10_25	p2	(TG)11	22	250802	250823
chr10_26	p2	(CA)17	34	253148	253181
chr10_27	p2	(TG)8	16	253539	253554
chr10_28	c	(GT)8(GA)6	28	260023	260050
chr10_29	p2	(AT)7	14	292047	292060
chr10_30	p2	(AC)6	12	292325	292336
chr10_31	p2	(GA)7	14	300233	300246
chr10_32	p4	(GAGG)5	20	340576	340595
chr10_33	p3	(AAC)5	15	393138	393152
chr10_34	p2	(TG)8	16	395033	395048
chr10_35	p2	(TC)7	14	396718	396731
chr10_36	p2	(CA)11	22	398039	398060
chr10_37	p2	(TG)17	34	405242	405275
chr10_38	p2	(TA)8	16	417235	417250
chr10_39	p4	(ATGG)5	20	444403	444422
chr10_40	p3	(AGC)5	15	460999	461013
chr10_41	p2	(CT)15	30	475090	475119
chr10_42	p2	(CA)7	14	489011	489024
chr10_43	p4	(AGAT)7	28	493108	493135
chr10_44	p2	(CT)6	12	497717	497728
chr10_45	p2	(TC)6	12	566564	566575
chr10_46	p2	(AC)8	16	568048	568063
chr10_47	p2	(TA)9	18	579183	579200
chr10_48	p2	(CT)6	12	583033	583044
chr10_49	p5	(ATTTG)5	25	583400	583424
chr10_50	p4	(ATTT)5	20	588329	588348
chr10_51	p4	(AAAC)6	24	591893	591916
chr10_52	p2	(AC)12	24	606233	606256
chr10_53	p2	(CA)13	26	630708	630733
chr10_54	p2	(AC)14	28	635734	635761
chr10_55	p2	(TG)12	24	649571	649594
chr10_56	c	ΓG)6agttgtgttcacatctgagaagagagatgtctgtaagagga(GT)1	81	655733	655813
chr10_57	p2	(GT)10	20	697956	697975
chr10_58	p2	(AC)6	12	705250	705261
chr10_59	p2	(GT)6	12	710700	710711
chr10_60	p2	(CA)6	12	729175	729186
chr10_61	p2	(AC)10	20	738726	738745
chr10_62	c	(TC)9(AC)20	58	751666	751723
chr10_63	p3	(GCA)6	18	763590	763607
chr10_64	c	(GA)13gc(GA)9(GT)7	60	767266	767325
chr10_65	p2	(GT)10	20	776493	776512
chr10_66	p2	(GT)7	14	785625	785638
chr10_67	p2	(AC)6	12	792422	792433
chr10_68	p2	(CA)9	18	794442	794459
chr10_69	p2	(GA)9	18	814892	814909
chr10_70	p2	(TG)12	24	820662	820685
chr10_71	p2	(AC)14	28	823224	823251
chr10_72	p2	(CA)13	26	828231	828256
chr10_73	p2	(GT)6	12	830601	830612

chr10_74	c	(GT)6g(GT)30	73	830735	830807
chr10_75	p2	(GT)12	24	840037	840060
chr10_76	c	(AC)8at(AC)19	56	842098	842153
chr10_77	p2	(AC)14	28	843661	843688
chr10_78	c	(AC)8atttattt(TA)8	40	849443	849482
chr10_79	c	(AC)12atatacatatatacacacatatacatatata(TG)6	67	850031	850097
chr10_80	p2	(TG)6	12	863826	863837
chr10_81	p2	(GT)8	16	867198	867213
chr10_82	p3	(CAC)5	15	883454	883468
chr10_83	p2	(GT)7	14	892396	892409
chr10_84	p2	(AT)6	12	902140	902151
chr10_85	p2	(GT)6	12	907806	907817
chr10_86	p2	(TG)6	12	924470	924481
chr10_87	p2	(TC)9	18	928129	928146
chr10_88	p2	(GT)11	22	928493	928514
chr10_89	p3	(TGT)5	15	929079	929093
chr10_90	p3	(TCC)6	18	934389	934406
chr10_91	p2	(AG)6	12	935492	935503
chr10_92	p3	(AAC)5	15	968240	968254
chr10_93	p2	(AC)6	12	976930	976941
chr10_94	p2	(TG)10	20	991654	991673
chr10_95	p2	(GT)20	40	1001665	1001704
chr10_96	p3	(ATA)6	18	1031144	1031161
chr10_97	p3	(ATT)6	18	1034934	1034951
chr10_98	p2	(GT)6	12	1048339	1048350
chr10_99	p2	(AC)6	12	1049942	1049953
chr10_100	p3	(ATC)8	24	1066502	1066525
chr10_101	p2	(AC)17	34	1067569	1067602
chr10_102	p2	(TC)21	42	1070074	1070115
chr10_103	p2	(TG)6	12	1074019	1074030
chr10_104	p2	(AC)10	20	1082485	1082504
chr10_105	p3	(ATG)6	18	1090154	1090171
chr10_106	p2	(GA)6	12	1112773	1112784
chr10_107	p3	(ATT)5	15	1113852	1113866
chr10_108	p2	(GT)7	14	1114018	1114031
chr10_109	p3	(AAC)6	18	1128142	1128159
chr10_110	p3	(AGG)8	24	1129550	1129573
chr10_111	p2	(AC)6	12	1130396	1130407
chr10_112	p2	(CA)6	12	1132363	1132374
chr10_113	p2	(TG)11	22	1134493	1134514
chr10_114	p2	(CT)6	12	1138165	1138176
chr10_115	p2	(TA)7	14	1145831	1145844
chr10_116	p2	(AC)6	12	1147720	1147731
chr10_117	p2	(CA)13	26	1148661	1148686
chr10_118	p2	(GT)17	34	1153297	1153330
chr10_119	p2	(CA)10	20	1161078	1161097
chr10_120	p2	(AC)6	12	1163928	1163939
chr10_121	p2	(TC)6	12	1164394	1164405
chr10_122	p2	(GT)7	14	1186818	1186831
chr10_123	p3	(AAT)5	15	1188917	1188931
chr10_124	c	2catctgtgtgagtgtgcatgcacaataacacaattgtatgagaatgactc(	104	1189580	1189683
chr10_125	p2	(TG)7	14	1197793	1197806

chr10_126	p2	(AC)11	22	1218300	1218321
chr10_127	p3	(CCA)5	15	1235382	1235396
chr10_128	p2	(AG)9	18	1239707	1239724
chr10_129	p2	(AG)8	16	1247224	1247239
chr10_130	p2	(AC)6	12	1255219	1255230
chr10_131	p2	(CA)6	12	1256454	1256465
chr10_132	p2	(AC)8	16	1275021	1275036
chr10_133	p2	(AG)8	16	1275727	1275742
chr10_134	c	(GT)7g(GT)10	35	1277957	1277991
chr10_135	p2	(GT)7	14	1279653	1279666
chr10_136	p2	(AC)8	16	1285520	1285535
chr10_137	p2	(CA)6	12	1286331	1286342
chr10_138	p2	(CA)10	20	1288494	1288513
chr10_139	p2	(AC)38	76	1306612	1306687
chr10_140	p2	(CA)6	12	1307704	1307715
chr10_141	p2	(GT)30	60	1321206	1321265
chr10_142	p3	(CCT)5	15	1345676	1345690
chr10_143	p2	(CA)11	22	1348698	1348719
chr10_144	p2	(CA)7	14	1349080	1349093
chr10_145	p2	(GA)6	12	1349599	1349610
chr10_146	p3	(AGT)5	15	1354269	1354283
chr10_147	c	(TC)6tt(TC)7	28	1359929	1359956
chr10_148	p5	(ATTTG)5	25	1382879	1382903
chr10_149	p2	(AC)10	20	1388168	1388187
chr10_150	p2	(AT)7	14	1401165	1401178
chr10_151	p2	(TA)9	18	1418501	1418518
chr10_152	p2	(GT)6	12	1419748	1419759
chr10_153	p4	(CATC)5	20	1421034	1421053
chr10_154	p2	(CT)10	20	1430048	1430067
chr10_155	p3	(AGC)6	18	1435233	1435250
chr10_156	p4	(AATG)5	20	1446478	1446497
chr10_157	p2	(CA)7	14	1451271	1451284
chr10_158	p2	(AC)9	18	1483729	1483746
chr10_159	p2	(GT)10	20	1489379	1489398
chr10_160	p2	(AC)11	22	1489540	1489561
chr10_161	p2	(GT)15	30	1489686	1489715
chr10_162	p2	(TG)11	22	1491543	1491564
chr10_163	p2	(AG)6	12	1492535	1492546
chr10_164	p2	(TG)6	12	1495752	1495763
chr10_165	p3	(AAT)5	15	1497199	1497213
chr10_166	p4	(TCCA)5	20	1500553	1500572
chr10_167	p2	(AC)15	30	1505163	1505192
chr10_168	p4	(AAAT)5	20	1506189	1506208
chr10_169	p2	(AC)9	18	1507177	1507194
chr10_170	p3	(CTC)5	15	1523825	1523839
chr10_171	p3	(CTG)6	18	1539557	1539574
chr10_172	p2	(CA)7	14	1545888	1545901
chr10_173	p2	(CA)8	16	1554073	1554088
chr10_174	p2	(AC)9	18	1566460	1566477
chr10_175	p2	(CA)15	30	1576321	1576350
chr10_176	p3	(AAC)6	18	1607893	1607910
chr10_177	p2	(TG)26	52	1615792	1615843

chr10_178	p2	(CA)7	14	1618024	1618037
chr10_179	p3	(ACA)5	15	1622380	1622394
chr10_180	p2	(AG)7	14	1623225	1623238
chr10_181	p2	(AC)13	26	1637232	1637257
chr10_182	p2	(AC)10	20	1638882	1638901
chr10_183	p2	(TC)13	26	1641108	1641133
chr10_184	c	(AAT)5gatgaggcatttatggtacctctgctccactgg(ACA)5	63	1644414	1644476
chr10_185	p2	(CA)7	14	1649096	1649109
chr10_186	p3	(CAG)6	18	1651014	1651031
chr10_187	p2	(GA)6	12	1662268	1662279
chr10_188	p3	(GAG)5	15	1667600	1667614
chr10_189	p2	(CA)7	14	1683900	1683913
chr10_190	c	(AC)9at(AC)6	32	1690352	1690383
chr10_191	p2	(CA)9	18	1691229	1691246
chr10_192	p4	(AGAC)5	20	1704093	1704112
chr10_193	c	gtataaatgaaaaaacacaactgtacacttccccacacagtccatgacctc[illegible]	78	1713194	1713271
chr10_194	p4	(CTAC)6	24	1714671	1714694
chr10_195	p3	(ATT)5	15	1722045	1722059
chr10_196	p2	(CA)14	28	1740097	1740124
chr10_197	p2	(AG)6	12	1740782	1740793
chr10_198	p2	(TC)9	18	1741994	1742011
chr10_199	p2	(CA)12	24	1743355	1743378
chr10_200	p2	(AC)12	24	1745676	1745699
chr10_201	p2	(CA)11	22	1753944	1753965
chr10_202	p2	(AC)6	12	1762697	1762708
chr10_203	p3	(ATG)15	45	1769354	1769398
chr10_204	p2	(TG)21	42	1777620	1777661
chr10_205	p2	(GT)12	24	1779747	1779770
chr10_206	p2	(CT)7	14	1782635	1782648
chr10_207	p4	(ACCA)5	20	1791539	1791558
chr10_208	p2	(AC)6	12	1791921	1791932
chr10_209	p2	(AC)14	28	1792838	1792865
chr10_210	p3	(CTT)5	15	1793365	1793379
chr10_211	c	(CAA)6cagcaacaacaacag(CAA)5	48	1797549	1797596
chr10_212	p2	(CA)7	14	1801959	1801972
chr10_213	p2	(AC)10	20	1802108	1802127
chr10_214	p2	(AC)11	22	1804304	1804325
chr10_215	p2	(GT)15	30	1818439	1818468
chr10_216	p2	(CA)7	14	1821286	1821299
chr10_217	p2	(GT)13	26	1823721	1823746
chr10_218	p3	(GCT)6	18	1833465	1833482
chr10_219	p2	(GT)8	16	1845445	1845460
chr10_220	p2	(GT)7	14	1847642	1847655
chr10_221	p2	(TG)22	44	1848930	1848973
chr10_222	p2	(CA)9	18	1849111	1849128
chr10_223	p2	(GA)7	14	1859526	1859539
chr10_224	p2	(TG)9	18	1866783	1866800
chr10_225	p2	(CA)17	34	1876365	1876398
chr10_226	p2	(AC)8	16	1880633	1880648
chr10_227	p2	(AC)6	12	1881278	1881289
chr10_228	c	(AC)6agccagaggagggggcattttgttcacatgg(CA)8	59	1881839	1881897
chr10_229	p2	(GT)6	12	1883209	1883220

chr10_230	p2	(AG)6	12	1891667	1891678
chr10_231	p2	(AC)10	20	1893245	1893264
chr10_232	p2	(AC)12	24	1893395	1893418
chr10_233	p2	(GT)8	16	1893775	1893790
chr10_234	p2	(AC)8	16	1900505	1900520
chr10_235	p2	(GT)7	14	1901514	1901527
chr10_236	p2	(TG)6	12	1911026	1911037
chr10_237	p4	(TCCA)8	32	1911483	1911514
chr10_238	p2	(TG)7	14	1915655	1915668
chr10_239	p2	(GA)6	12	1915801	1915812
chr10_240	p2	(TC)15	30	1920878	1920907
chr10_241	p3	(CAA)7	21	1925150	1925170
chr10_242	p2	(CT)6	12	1939268	1939279
chr10_243	p2	(CA)9	18	1942297	1942314
chr10_244	p2	(AC)20	40	1942572	1942611
chr10_245	p2	(GT)12	24	1945835	1945858
chr10_246	p2	(CA)7	14	1947867	1947880
chr10_247	c	(AC)10gtgcg(CA)6	37	1954647	1954683
chr10_248	p2	(CA)6	12	1966838	1966849
chr10_249	p2	(CA)14	28	1984325	1984352
chr10_250	p2	(CA)16	32	1996715	1996746
chr10_251	p2	(AT)6	12	2018392	2018403
chr10_252	p2	(AG)6	12	2020310	2020321
chr10_253	p2	(GA)6	12	2020441	2020452
chr10_254	p2	(CA)6	12	2023995	2024006
chr10_255	p3	(AAC)5	15	2033936	2033950
chr10_256	p2	(GT)30	60	2038278	2038337
chr10_257	p3	(CAA)5	15	2039708	2039722
chr10_258	c	(CA)30c(CA)6	73	2042318	2042390
chr10_259	p2	(TG)20	40	2062881	2062920
chr10_260	p2	(TG)9	18	2069747	2069764
chr10_261	p2	(AC)10	20	2074226	2074245
chr10_262	p2	(CA)14	28	2075873	2075900
chr10_263	c	(TGC)5(TGA)5	30	2085761	2085790
chr10_264	p3	(TAA)5	15	2092992	2093006
chr10_265	p2	(AC)17	34	2097705	2097738
chr10_266	p2	(TC)7	14	2100435	2100448
chr10_267	p2	(AT)6	12	2119695	2119706
chr10_268	p4	(TCCA)5	20	2122062	2122081
chr10_269	p3	(CTG)6	18	2128684	2128701
chr10_270	p4	(CATT)7	28	2132686	2132713
chr10_271	c	(AC)6tgcacactgcaatgaagaacagcttgcttgtacta(AC)8	63	2133688	2133750
chr10_272	c	(TG)20tc(TG)9	60	2145111	2145170
chr10_273	p3	(AAT)6	18	2155320	2155337
chr10_274	p2	(TG)9	18	2158837	2158854
chr10_275	p2	(GT)21	42	2159962	2160003
chr10_276	p3	(TTG)5	15	2166721	2166735
chr10_277	p3	(TCT)5	15	2167173	2167187
chr10_278	p2	(TG)7	14	2182895	2182908
chr10_279	p2	(TA)6	12	2188496	2188507
chr10_280	c	(CT)14cctcagtcacacaacacatgcgctt(AC)10	73	2196575	2196647
chr10_281	c	(TG)12agaaagagagagagaa(AG)8	56	2199576	2199631

chr10_282	p2	(GT)7	14	2206291	2206304
chr10_283	p2	(CA)29	58	2208610	2208667
chr10_284	c	:ctacttcaacaggcacagaaatactaaacacacacacgcacacacacac;	75	2209151	2209225
chr10_285	p2	(CT)9	18	2225522	2225539
chr10_286	p2	(TG)8	16	2235198	2235213
chr10_287	c	(TG)6agatggagtgataaggaggaagc(AG)6	47	2235343	2235389
chr10_288	p2	(AG)8	16	2238269	2238284
chr10_289	p2	(TG)6	12	2241124	2241135
chr10_290	p3	(TCC)5	15	2244433	2244447
chr10_291	p4	(ATTT)5	20	2273257	2273276
chr10_292	p2	(TG)21	42	2278930	2278971
chr10_293	p3	(TAA)6	18	2279321	2279338
chr10_294	p2	(CA)8	16	2280271	2280286
chr10_295	p2	(TG)8	16	2286714	2286729
chr10_296	p2	(GT)6	12	2290399	2290410
chr10_297	p2	(TG)9	18	2293308	2293325
chr10_298	p2	(AC)6	12	2297504	2297515
chr10_299	p3	(ATA)8	24	2300305	2300328
chr10_300	p2	(TG)7	14	2315634	2315647
chr10_301	p2	(AT)7	14	2316696	2316709
chr10_302	p2	(CA)17	34	2320387	2320420
chr10_303	p2	(GT)7	14	2330272	2330285
chr10_304	p4	(AATC)6	24	2332693	2332716
chr10_305	c	(TG)9a(GT)8ga(GT)8ga(GT)8ga(GT)9	91	2335907	2335997
chr10_306	p2	(GT)11	22	2336240	2336261
chr10_307	p2	(GT)7	14	2356230	2356243
chr10_308	p4	(ATCC)5	20	2360054	2360073
chr10_309	p2	(GT)7	14	2364355	2364368
chr10_310	p4	(ATCC)5	20	2368179	2368198
chr10_311	p3	(ATA)7	21	2371555	2371575
chr10_312	p6	(ACTTTG)5	30	2397919	2397948
chr10_313	c	agcatgcatgtgtggattttgctcagtgctgacaggcaatggttaggca(G	114	2401368	2401481
chr10_314	p2	(AC)15	30	2403393	2403422
chr10_315	p3	(AGG)5	15	2403761	2403775
chr10_316	p3	(AGG)5	15	2405239	2405253
chr10_317	p2	(TG)6	12	2409012	2409023
chr10_318	p2	(GT)11	22	2412355	2412376
chr10_319	p3	(GCT)5	15	2418360	2418374
chr10_320	c	(TG)14tcaggctgtgctttcatctggctaggtttctcttgtgta(TG)8	83	2421356	2421438
chr10_321	p2	(CA)20	40	2426787	2426826
chr10_322	p4	(CATC)6	24	2428356	2428379
chr10_323	p2	(GT)15	30	2430681	2430710
chr10_324	p2	(TG)6	12	2438971	2438982
chr10_325	p2	(TG)6	12	2441618	2441629
chr10_326	p2	(GA)6	12	2454290	2454301
chr10_327	p2	(GT)6	12	2457771	2457782
chr10_328	p5	(TTTTA)5	25	2462838	2462862
chr10_329	p2	(GT)6	12	2481543	2481554
chr10_330	p3	(TCT)5	15	2488859	2488873
chr10_331	p2	(TG)13	26	2490898	2490923
chr10_332	p3	(AAT)7	21	2501545	2501565
chr10_333	p2	(AC)14	28	2513658	2513685

chr10_334	p2	(CA)6	12	2515564	2515575
chr10_335	p2	(AC)23	46	2522155	2522200
chr10_336	p2	(AC)11	22	2533706	2533727
chr10_337	p3	(CTC)6	18	2544832	2544849
chr10_338	c	(CA)6gacacatttgtgtgagctggtaaagtt(TG)7	53	2547994	2548046
chr10_339	p4	(AAAT)5	20	2564707	2564726
chr10_340	p2	(AC)7	14	2566119	2566132
chr10_341	p2	(AC)6	12	2569752	2569763
chr10_342	p2	(GT)9	18	2573465	2573482
chr10_343	p2	(TG)8	16	2587224	2587239
chr10_344	p2	(GT)12	24	2592057	2592080
chr10_345	p2	(TG)14	28	2592748	2592775
chr10_346	p2	(TG)8	16	2592915	2592930
chr10_347	p2	(AC)12	24	2594426	2594449
chr10_348	p3	(ATT)6	18	2594969	2594986
chr10_349	p4	(GGTA)5	20	2598902	2598921
chr10_350	p2	(AC)6	12	2601235	2601246
chr10_351	p2	(AC)6	12	2603061	2603072
chr10_352	p2	(CA)15	30	2604596	2604625
chr10_353	c	:ctctcaactcactcttcacttcagatataaccaggactgtacggacaaacac	107	2615939	2616045
chr10_354	p3	(TAA)7	21	2618957	2618977
chr10_355	p3	(TAA)7	21	2619697	2619717
chr10_356	p3	(CCA)5	15	2633328	2633342
chr10_357	p2	(TG)6	12	2633976	2633987
chr10_358	p2	(AC)9	18	2648354	2648371
chr10_359	p2	(TG)7	14	2661534	2661547
chr10_360	p2	(AC)10	20	2669264	2669283
chr10_361	p3	(TCC)5	15	2670347	2670361
chr10_362	c	gaatatattgaatgaatcttgagaatatataatgaaagtctgaatatatatataa	103	2677547	2677649
chr10_363	p2	(TC)10	20	2679175	2679194
chr10_364	p2	(TG)13	26	2682296	2682321
chr10_365	p2	(GA)6	12	2685462	2685473
chr10_366	p2	(GT)8	16	2689222	2689237
chr10_367	p3	(GCT)9	27	2693501	2693527
chr10_368	p2	(CA)6	12	2710497	2710508
chr10_369	p2	(CA)7	14	2710622	2710635
chr10_370	p2	(CA)6	12	2719460	2719471
chr10_371	p2	(TG)10	20	2725440	2725459
chr10_372	p2	(CT)6	12	2728241	2728252
chr10_373	c	atagagaggagcaagtatggggctttaattaaatgcatgtgttttgtgtgtaa	158	2735588	2735745
chr10_374	p4	(ATGT)6	24	2736738	2736761
chr10_375	p2	(GT)11	22	2736882	2736903
chr10_376	c	;tgacagagcaaaagagagatggaagaattatagggt(TG)15tactgta	221	2737822	2738042
chr10_377	p2	(TG)6	12	2738189	2738200
chr10_378	p2	(CA)16	32	2744988	2745019
chr10_379	p2	(GT)29	58	2759271	2759328
chr10_380	c	(GT)6(GA)6	24	2764051	2764074
chr10_381	p3	(CAG)5	15	2770187	2770201
chr10_382	p2	(AC)6	12	2779177	2779188
chr10_383	p3	(ATG)5	15	2798405	2798419
chr10_384	p2	(GT)6	12	2827120	2827131
chr10_385	p4	(CAGA)5	20	2854766	2854785

chr10_386	p2	(TC)7	14	2861201	2861214
chr10_387	c	taattttgagcatgcggcatgatgattttaatatatatacagtatgtgtgtatgta	151	2863880	2864030
chr10_388	p2	(GT)14	28	2868074	2868101
chr10_389	p2	(GA)8	16	2868670	2868685
chr10_390	p2	(GT)8	16	2881290	2881305
chr10_391	c	3cctttatatgcaacttttcaagaaaacttgcaacacagataagcaaacag(A	96	2886636	2886731
chr10_392	p2	(GT)7	14	2907285	2907298
chr10_393	p2	(TA)6	12	2909898	2909909
chr10_394	p3	(CCT)5	15	2942627	2942641
chr10_395	p2	(TG)6	12	2945799	2945810
chr10_396	p3	(TGA)6	18	2954848	2954865
chr10_397	p2	(AG)6	12	2962550	2962561
chr10_398	p3	(GAA)7	21	2965423	2965443
chr10_399	p4	(TTTG)5	20	2969325	2969344
chr10_400	p3	(CTC)8	24	2970502	2970525
chr10_401	c	(TC)7(AC)16	46	2992512	2992557
chr10_402	p2	(AC)11	22	3028531	3028552
chr10_403	p2	(GA)16	32	3031694	3031725
chr10_404	p2	(AC)11	22	3039985	3040006
chr10_405	p2	(GT)6	12	3062935	3062946
chr10_406	p3	(CAC)5	15	3069233	3069247
chr10_407	p2	(CA)24	48	3078883	3078930
chr10_408	p2	(AC)15	30	3081800	3081829
chr10_409	p2	(GA)21	42	3084076	3084117
chr10_410	p2	(TG)12	24	3091661	3091684
chr10_411	p2	(TG)32	64	3094373	3094436
chr10_412	p2	(CA)9	18	3094544	3094561
chr10_413	p5	(ATTTG)5	25	3098569	3098593
chr10_414	p2	(AC)10	20	3104393	3104412
chr10_415	p2	(GA)20	40	3104808	3104847
chr10_416	p2	(AC)7	14	3111376	3111389
chr10_417	p2	(TG)9	18	3112944	3112961
chr10_418	p2	(AC)8	16	3115614	3115629
chr10_419	p2	(AC)6	12	3124078	3124089
chr10_420	p2	(AT)14	28	3129795	3129822
chr10_421	p2	(AC)23	46	3137782	3137827
chr10_422	p2	(CA)9	18	3139955	3139972
chr10_423	p2	(GT)10	20	3140295	3140314
chr10_424	p2	(GT)8	16	3155602	3155617
chr10_425	p3	(TAA)5	15	3167858	3167872
chr10_426	p2	(AC)7	14	3168677	3168690
chr10_427	p2	(TG)13	26	3172214	3172239
chr10_428	p3	(ATC)5	15	3175161	3175175
chr10_429	p2	(CA)6	12	3204926	3204937
chr10_430	p2	(TG)8	16	3205381	3205396
chr10_431	p3	(AAG)5	15	3210573	3210587
chr10_432	p4	(TAGG)5	20	3217573	3217592
chr10_433	p2	(AC)13	26	3240310	3240335
chr10_434	p4	(ATAG)8	32	3240604	3240635
chr10_435	c	(CA)20accacacaaacnnnnnnnncacacacacc(CA)22	113	3252775	3252887
chr10_436	p3	(TAG)11	33	3253216	3253248
chr10_437	c	(TG)6ta(TG)19	52	3260615	3260666

chr10_438	p2	(TA)6	12	3277762	3277773
chr10_439	p2	(TA)6	12	3286598	3286609
chr10_440	p2	(TA)6	12	3310746	3310757
chr10_441	p2	(CA)13	26	3328885	3328910
chr10_442	p3	(CAG)8	24	3335359	3335382
chr10_443	p3	(ATA)10	30	3352404	3352433
chr10_444	p2	(AC)23	46	3368095	3368140
chr10_445	p2	(AT)6	12	3372209	3372220
chr10_446	p3	(TTC)6	18	3383949	3383966
chr10_447	p2	(TA)12	24	3407056	3407079
chr10_448	c	(TG)7ta(TG)6	28	3434287	3434314
chr10_449	p3	(TCA)5	15	3441179	3441193
chr10_450	p2	(CA)8	16	3444791	3444806
chr10_451	p2	(AT)7	14	3445210	3445223
chr10_452	p3	(TAA)5	15	3450792	3450806
chr10_453	c	AC)13ataatatatatataaaattgctcacacatcacaatatatatat(AC)	81	3464950	3465030
chr10_454	p2	(AC)10	20	3480942	3480961
chr10_455	p3	(TTA)6	18	3485469	3485486
chr10_456	p2	(AC)6	12	3494223	3494234
chr10_457	p2	(AC)13	26	3495708	3495733
chr10_458	p2	(GA)10	20	3497987	3498006
chr10_459	p3	(AAT)5	15	3499548	3499562
chr10_460	c	(CA)6cgcg(CA)13c(TA)6	55	3507109	3507163
chr10_461	p5	(CACTA)5	25	3513891	3513915
chr10_462	p3	(AAT)7	21	3517480	3517500
chr10_463	p3	(CCT)5	15	3518520	3518534
chr10_464	p4	(AAAC)6	24	3524715	3524738
chr10_465	p2	(TG)17	34	3553313	3553346
chr10_466	c	[)7gagagagataaattactcgtggagcaggaggccacatacacac(TG	85	3561071	3561155
chr10_467	p2	(GT)7	14	3583040	3583053
chr10_468	p3	(CAA)5	15	3590323	3590337
chr10_469	p2	(AG)10	20	3593044	3593063
chr10_470	p3	(AAC)6	18	3593341	3593358
chr10_471	p2	(TG)9	18	3602378	3602395
chr10_472	p2	(AC)6	12	3609581	3609592
chr10_473	p2	(CA)18	36	3616438	3616473
chr10_474	p2	(TG)6	12	3617203	3617214
chr10_475	c	(ACT)15(GCT)5	60	3620275	3620334
chr10_476	p2	(GA)6	12	3649359	3649370
chr10_477	p2	(TG)6	12	3657273	3657284
chr10_478	p2	(AC)17	34	3657508	3657541
chr10_479	c	(AC)6aa(AC)9	32	3693734	3693765
chr10_480	p2	(CA)9	18	3696272	3696289
chr10_481	p2	(TG)10	20	3696413	3696432
chr10_482	p2	(CA)6	12	3700922	3700933
chr10_483	p4	(AATG)5	20	3707987	3708006
chr10_484	p2	(AG)6	12	3757968	3757979
chr10_485	p2	(GT)12	24	3762497	3762520
chr10_486	p2	(AC)7	14	3763690	3763703
chr10_487	p2	(AC)6	12	3768633	3768644
chr10_488	p3	(CAA)6	18	3778653	3778670
chr10_489	p2	(TA)12	24	3784860	3784883

chr10_490	p2	(TG)8	16	3796006	3796021
chr10_491	p2	(CA)14	28	3796771	3796798
chr10_492	p4	(CCAT)5	20	3809317	3809336
chr10_493	p2	(GT)16	32	3818247	3818278
chr10_494	p2	(CA)28	56	3820838	3820893
chr10_495	p2	(CA)9	18	3824849	3824866
chr10_496	p2	(GT)19	38	3836426	3836463
chr10_497	p4	(ACAA)5	20	3863122	3863141
chr10_498	p2	(GT)15	30	3905713	3905742
chr10_499	p2	(AG)6	12	3909296	3909307
chr10_500	p2	(CA)9	18	3912499	3912516
chr10_501	c	(TTA)7(GTA)6	39	3917219	3917257
chr10_502	p2	(TA)7	14	3919124	3919137
chr10_503	p2	(CA)29	58	3935705	3935762
chr10_504	p3	(CCA)7	21	3937698	3937718
chr10_505	c	(GT)9ctgt(GA)12	46	3942795	3942840
chr10_506	p2	(GT)8	16	3981526	3981541
chr10_507	p2	(TC)6	12	3984735	3984746
chr10_508	p2	(TG)9	18	3985370	3985387
chr10_509	p3	(TAA)5	15	3986709	3986723
chr10_510	p3	(AGT)8	24	3986916	3986939
chr10_511	p2	(GT)6	12	3994134	3994145
chr10_512	p2	(TG)23	46	4004365	4004410
chr10_513	p2	(GA)7	14	4007664	4007677
chr10_514	p2	(AC)8	16	4008711	4008726
chr10_515	p4	(AAAC)5	20	4009113	4009132
chr10_516	p2	(AT)11	22	4009612	4009633
chr10_517	p2	(AC)6	12	4011892	4011903
chr10_518	p2	(AC)18	36	4023577	4023612
chr10_519	p2	(TG)6	12	4037532	4037543
chr10_520	p3	(CTC)5	15	4065774	4065788
chr10_521	p2	(TA)6	12	4071944	4071955
chr10_522	p2	(AC)20	40	4072473	4072512
chr10_523	p2	(AC)6	12	4082180	4082191
chr10_524	p2	(GA)6	12	4089489	4089500
chr10_525	c	(TC)8(AC)12	40	4103780	4103819
chr10_526	c	(TC)8(AC)12	40	4104131	4104170
chr10_527	p5	(TGTTG)5	25	4110111	4110135
chr10_528	p3	(GAT)6	18	4112589	4112606
chr10_529	p2	(TG)12	24	4117040	4117063
chr10_530	p2	(GT)27	54	4122832	4122885
chr10_531	p2	(TA)7	14	4132228	4132241
chr10_532	p2	(AC)16	32	4137199	4137230
chr10_533	p2	(CT)6	12	4148420	4148431
chr10_534	p2	(TG)14	28	4149087	4149114
chr10_535	p2	(AT)7	14	4149783	4149796
chr10_536	p3	(ATT)6	18	4150097	4150114
chr10_537	p2	(CA)15	30	4155337	4155366
chr10_538	p3	(TTA)5	15	4157948	4157962
chr10_539	p2	(AC)9	18	4159644	4159661
chr10_540	p3	(TTA)5	15	4165283	4165297
chr10_541	p3	(CTC)5	15	4168984	4168998

chr10_542	p2	(GT)7	14	4173140	4173153
chr10_543	p2	(AC)6	12	4193716	4193727
chr10_544	p2	(AT)6	12	4198022	4198033
chr10_545	p2	(TC)6	12	4210642	4210653
chr10_546	p2	(GT)6	12	4215205	4215216
chr10_547	p3	(CCT)5	15	4221115	4221129
chr10_548	p2	(AG)8	16	4245869	4245884
chr10_549	p2	(TA)6	12	4267213	4267224
chr10_550	p3	(GAA)5	15	4271277	4271291
chr10_551	p3	(GAA)5	15	4273089	4273103
chr10_552	p2	(TG)13	26	4282307	4282332
chr10_553	p2	(TA)6	12	4289875	4289886
chr10_554	p2	(AC)11	22	4290360	4290381
chr10_555	p2	(CA)7	14	4295480	4295493
chr10_556	p2	(CA)7	14	4296326	4296339
chr10_557	p2	(GT)6	12	4301192	4301203
chr10_558	p2	(CA)12	24	4301326	4301349
chr10_559	p2	(GT)6	12	4305587	4305598
chr10_560	c	(CA)6a(AC)6	25	4313750	4313774
chr10_561	p5	(CTCTC)5	25	4321409	4321433
chr10_562	p3	(ATA)5	15	4321681	4321695
chr10_563	p3	(CAA)5	15	4322011	4322025
chr10_564	p2	(AG)10	20	4331598	4331617
chr10_565	c	(TC)9(AC)6	30	4338649	4338678
chr10_566	p2	(CT)17	34	4343227	4343260
chr10_567	p2	(TG)7	14	4369307	4369320
chr10_568	p4	(ATTC)7	28	4369778	4369805
chr10_569	p4	(AAAT)5	20	4370388	4370407
chr10_570	p2	(CA)6	12	4372727	4372738
chr10_571	p2	(AC)7	14	4378456	4378469
chr10_572	p3	(ATA)5	15	4380022	4380036
chr10_573	p2	(TA)6	12	4383199	4383210
chr10_574	p2	(TG)7	14	4388038	4388051
chr10_575	p2	(TG)6	12	4402002	4402013
chr10_576	p2	(AC)18	36	4409149	4409184
chr10_577	p2	(AT)6	12	4411758	4411769
chr10_578	p2	(TG)7	14	4421289	4421302
chr10_579	p2	(TG)23	46	4425593	4425638
chr10_580	p2	(GT)7	14	4425788	4425801
chr10_581	p2	(CT)6	12	4447925	4447936
chr10_582	p3	(AAT)8	24	4452980	4453003
chr10_583	p2	(TA)7	14	4473415	4473428
chr10_584	p2	(CA)8	16	4478796	4478811
chr10_585	p5	(GATTT)5	25	4485708	4485732
chr10_586	p2	(TG)6	12	4495118	4495129
chr10_587	p3	(AAT)5	15	4524738	4524752
chr10_588	p4	(AATG)5	20	4531667	4531686
chr10_589	p2	(CA)9	18	4532454	4532471
chr10_590	p3	(CAA)5	15	4533589	4533603
chr10_591	p2	(CA)13	26	4563681	4563706
chr10_592	p2	(TG)6	12	4567602	4567613
chr10_593	p4	(GTTT)5	20	4576232	4576251

chr10_594	p3	(TGA)6	18	4576405	4576422
chr10_595	p2	(CT)6	12	4581598	4581609
chr10_596	p3	(ACC)5	15	4594362	4594376
chr10_597	p3	(AAC)5	15	4605517	4605531
chr10_598	c	(TG)7(TA)14	42	4610015	4610056
chr10_599	p3	(AAT)8	24	4619303	4619326
chr10_600	c	(AT)8(AC)15	46	4634718	4634763
chr10_601	c	(AC)6t(CA)19	51	4636723	4636773
chr10_602	p2	(AC)13	26	4639633	4639658
chr10_603	p2	(TG)14	28	4641067	4641094
chr10_604	p2	(GT)9	18	4641770	4641787
chr10_605	p2	(GT)19	38	4643138	4643175
chr10_606	p2	(GT)19	38	4644572	4644609
chr10_607	p4	(AATC)6	24	4647046	4647069
chr10_608	p3	(ATC)7	21	4650962	4650982
chr10_609	p2	(TG)25	50	4653200	4653249
chr10_610	p2	(GA)6	12	4660968	4660979
chr10_611	p2	(TG)8	16	4678501	4678516
chr10_612	p2	(CT)9	18	4679368	4679385
chr10_613	c	catattagcagcagtcgtcgtcgtcttcctctactcatattagtagcagtcgtc	109	4682834	4682942
chr10_614	p3	(ACA)5	15	4696319	4696333
chr10_615	p3	(ACA)5	15	4697801	4697815
chr10_616	p2	(AG)10	20	4712687	4712706
chr10_617	p3	(CTC)6	18	4714278	4714295
chr10_618	p2	(TG)8	16	4719828	4719843
chr10_619	p2	(CA)7	14	4721989	4722002
chr10_620	c	(AC)9(AG)6	30	4723915	4723944
chr10_621	c	(AC)9(AG)6	30	4725652	4725681
chr10_622	p2	(GT)7	14	4727494	4727507
chr10_623	c	(AC)9(AG)6	30	4733755	4733784
chr10_624	p2	(GT)6	12	4736414	4736425
chr10_625	p2	(GT)9	18	4743565	4743582
chr10_626	p3	(CAA)6	18	4751773	4751790
chr10_627	p2	(AT)6	12	4762985	4762996
chr10_628	p3	(TTA)5	15	4763635	4763649
chr10_629	p4	(TTCC)6	24	4767172	4767195
chr10_630	p3	(TCT)7	21	4777773	4777793
chr10_631	c	(GA)7ggagcacggagaggagaatggctgctgtaacagagg(GA)1[illegible]	76	4781655	4781730
chr10_632	c	(AC)7(AT)9	32	4797221	4797252
chr10_633	p3	(CTT)5	15	4800781	4800795
chr10_634	p2	(GA)8	16	4809645	4809660
chr10_635	c	(AC)7atatatatatac(AT)7(AC)14	68	4814044	4814111
chr10_636	p2	(TC)6	12	4835290	4835301
chr10_637	p2	(GT)7	14	4839244	4839257
chr10_638	p2	(AC)14	28	4840339	4840366
chr10_639	p2	(TA)6	12	4843863	4843874
chr10_640	p2	(AC)10	20	4850341	4850360
chr10_641	c	(TC)8a(CT)6	29	4866906	4866934
chr10_642	p5	(AATCA)5	25	4868633	4868657
chr10_643	p2	(AT)9	18	4875720	4875737
chr10_644	p2	(AT)6	12	4887542	4887553
chr10_645	p3	(CTA)5	15	4894583	4894597

chr10_646	p2	(GT)15	30	4901542	4901571
chr10_647	p2	(TG)6	12	4914736	4914747
chr10_648	p2	(GT)6	12	4930492	4930503
chr10_649	p4	(AAAT)6	24	4937072	4937095
chr10_650	c	(AG)6ac(AG)9	32	4941353	4941384
chr10_651	p2	(TG)7	14	4947711	4947724
chr10_652	p2	(TG)8	16	4961099	4961114
chr10_653	p2	(GT)11	22	4961380	4961401
chr10_654	p4	(ATGG)7	28	4969279	4969306
chr10_655	p2	(GT)7	14	4975227	4975240
chr10_656	p3	(TGC)7	21	4986968	4986988
chr10_657	p2	(AC)13	26	4991290	4991315
chr10_658	p3	(ATC)5	15	5000107	5000121
chr10_659	p2	(AC)12	24	5004727	5004750
chr10_660	p2	(GA)7	14	5019138	5019151
chr10_661	p2	(AC)13	26	5019620	5019645
chr10_662	p2	(TA)6	12	5037243	5037254
chr10_663	p2	(TA)6	12	5045313	5045324
chr10_664	p2	(CT)6	12	5048722	5048733
chr10_665	p2	(AC)7	14	5050568	5050581
chr10_666	p2	(CA)6	12	5056553	5056564
chr10_667	p3	(TTA)6	18	5057432	5057449
chr10_668	p3	(TGT)11	33	5064732	5064764
chr10_669	p3	(AAT)8	24	5068122	5068145
chr10_670	p2	(CA)8	16	5069278	5069293
chr10_671	c	(AT)8acactgtatatgtatc(TG)9	50	5078076	5078125
chr10_672	p2	(CT)8	16	5086894	5086909
chr10_673	p2	(CA)8	16	5097877	5097892
chr10_674	c	(GT)9(GA)7	32	5098145	5098176
chr10_675	p2	(GT)6	12	5101510	5101521
chr10_676	p2	(GT)8	16	5102871	5102886
chr10_677	p3	(ATT)5	15	5103071	5103085
chr10_678	p4	(AAAT)5	20	5107675	5107694
chr10_679	p3	(CTT)5	15	5114506	5114520
chr10_680	p4	(ATGG)5	20	5117015	5117034
chr10_681	p2	(AT)7	14	5118158	5118171
chr10_682	p2	(AT)7	14	5119780	5119793
chr10_683	p2	(AC)9	18	5125567	5125584
chr10_684	c	[illegible]ggtgtaatatacaccgcctaccccgccaatggcttgtgtttaaaagggtca[illegible]	123	5141082	5141204
chr10_685	p2	(AC)19	38	5143044	5143081
chr10_686	p2	(AC)11	22	5149070	5149091
chr10_687	p3	(AAC)7	21	5156653	5156673
chr10_688	p3	(AAC)6	18	5159072	5159089
chr10_689	p2	(CA)6	12	5164423	5164434
chr10_690	p2	(CA)7	14	5165827	5165840
chr10_691	p2	(AG)8	16	5172635	5172650
chr10_692	p3	(TTA)5	15	5173588	5173602
chr10_693	p2	(GT)7	14	5210060	5210073
chr10_694	p2	(AC)9	18	5210952	5210969
chr10_695	p2	(AC)14	28	5216180	5216207
chr10_696	p2	(TG)22	44	5219916	5219959
chr10_697	p3	(TTA)5	15	5221150	5221164

chr10_698	c	(TA)7(CA)6	26	5244314	5244339
chr10_699	p3	(GAG)5	15	5255120	5255134
chr10_700	p2	(AC)7	14	5263010	5263023
chr10_701	p2	(GT)6	12	5268800	5268811
chr10_702	p2	(AG)7	14	5276871	5276884
chr10_703	c	(TC)9(AC)7	32	5282223	5282254
chr10_704	p2	(CT)18	36	5293537	5293572
chr10_705	p3	(ATT)6	18	5294541	5294558
chr10_706	c	ggcactgagactaacctgcagcactaatgtgtgttggcatgtgtatgcata	110	5304410	5304519
chr10_707	p2	(CA)6	12	5309536	5309547
chr10_708	p3	(AAC)6	18	5310675	5310692
chr10_709	p2	(GT)8	16	5318422	5318437
chr10_710	p2	(AC)7	14	5322984	5322997
chr10_711	p2	(GT)6	12	5336305	5336316
chr10_712	p2	(TG)8	16	5342050	5342065
chr10_713	p2	(GT)12	24	5345818	5345841
chr10_714	p2	(TG)29	58	5350148	5350205
chr10_715	p2	(AC)7	14	5350914	5350927
chr10_716	c	(CT)8ccctct(CA)7	36	5351055	5351090
chr10_717	p2	(CA)10	20	5352688	5352707
chr10_718	p2	(AC)8	16	5357659	5357674
chr10_719	p4	(TAGG)5	20	5358632	5358651
chr10_720	p2	(AC)17	34	5362572	5362605
chr10_721	c	(AT)9annnnnnnnnnntgaggctatgtg(TA)11tt(AC)21	108	5365284	5365391
chr10_722	p2	(CA)6	12	5375997	5376008
chr10_723	p2	(AC)20	40	5378334	5378373
chr10_724	p3	(AAT)18	54	5379171	5379224
chr10_725	p2	(GT)7	14	5382201	5382214
chr10_726	c	(AC)8atatatatat(AC)6	38	5413658	5413695
chr10_727	p2	(TG)6	12	5416724	5416735
chr10_728	p2	(AG)8	16	5421710	5421725
chr10_729	p2	(GT)17	34	5454456	5454489
chr10_730	p2	(CA)14	28	5454592	5454619
chr10_731	p2	(CA)7	14	5456280	5456293
chr10_732	c	(TG)12(AG)6	36	5458249	5458284
chr10_733	p2	(TG)11	22	5479428	5479449
chr10_734	p2	(CA)8	16	5481448	5481463
chr10_735	c	(CA)12a(ACT)5	40	5486573	5486612
chr10_736	p2	(TG)6	12	5487641	5487652
chr10_737	p2	(CA)6	12	5491672	5491683
chr10_738	p2	(TC)6	12	5498853	5498864
chr10_739	p2	(AC)7	14	5500530	5500543
chr10_740	p2	(GA)6	12	5500767	5500778
chr10_741	p2	(CA)16	32	5501314	5501345
chr10_742	p2	(AC)10	20	5502849	5502868
chr10_743	p2	(CA)7	14	5503558	5503571
chr10_744	p2	(GA)6	12	5503948	5503959
chr10_745	p2	(AC)10	20	5505193	5505212
chr10_746	p2	(GT)13	26	5513147	5513172
chr10_747	p2	(CA)6	12	5514511	5514522
chr10_748	p2	(CA)9	18	5514887	5514904
chr10_749	p2	(AC)13	26	5518316	5518341

chr10_750	p4	(ACAG)5	20	5525697	5525716
chr10_751	p3	(GTG)5	15	5525867	5525881
chr10_752	p3	(AAT)10	30	5529535	5529564
chr10_753	p2	(CT)6	12	5532174	5532185
chr10_754	p2	(GA)8	16	5536910	5536925
chr10_755	c	(TG)10cgcactatgtaa(GT)9	50	5548294	5548343
chr10_756	p2	(AC)10	20	5548506	5548525
chr10_757	p2	(TG)6	12	5548672	5548683
chr10_758	p3	(CTG)5	15	5559837	5559851
chr10_759	p2	(AC)6	12	5561973	5561984
chr10_760	c	(GT)8a(TG)48	113	5576978	5577090
chr10_761	p2	(AC)9	18	5582869	5582886
chr10_762	p2	(GT)19	38	5583995	5584032
chr10_763	p2	(GT)25	50	5586576	5586625
chr10_764	p2	(CA)24	48	5588468	5588515
chr10_765	p2	(AT)6	12	5593354	5593365
chr10_766	p3	(ATT)5	15	5599610	5599624
chr10_767	p2	(CA)10	20	5601464	5601483
chr10_768	p2	(TG)11	22	5602588	5602609
chr10_769	p2	(CA)14	28	5610396	5610423
chr10_770	p2	(TG)10	20	5618943	5618962
chr10_771	p3	(AAC)5	15	5622973	5622987
chr10_772	p2	(AC)8	16	5623717	5623732
chr10_773	p2	(AC)6	12	5624946	5624957
chr10_774	p2	(GT)8	16	5626124	5626139
chr10_775	p2	(TG)12	24	5651508	5651531
chr10_776	c	(GT)6t(TG)30	73	5656182	5656254
chr10_777	p2	(CA)6	12	5656674	5656685
chr10_778	p2	(CT)6	12	5658204	5658215
chr10_779	p2	(GT)6	12	5658957	5658968
chr10_780	p2	(CA)9	18	5669883	5669900
chr10_781	p2	(AT)6	12	5679748	5679759
chr10_782	p2	(AC)11	22	5685996	5686017
chr10_783	p2	(AC)6	12	5700968	5700979
chr10_784	p2	(AC)6	12	5710919	5710930
chr10_785	p3	(TCC)7	21	5719044	5719064
chr10_786	p5	(AGAGC)6	30	5725838	5725867
chr10_787	p3	(GAG)9	27	5734557	5734583
chr10_788	p2	(GA)8	16	5759597	5759612
chr10_789	p2	(AG)6	12	5761537	5761548
chr10_790	p2	(GT)6	12	5771071	5771082
chr10_791	p2	(TG)7	14	5771458	5771471
chr10_792	p2	(CA)28	56	5781681	5781736
chr10_793	p2	(AC)13	26	5784450	5784475
chr10_794	p3	(AAT)11	33	5789069	5789101
chr10_795	p2	(GA)16	32	5789351	5789382
chr10_796	p2	(AC)13	26	5805330	5805355
chr10_797	p3	(AAT)11	33	5809949	5809981
chr10_798	p2	(GA)16	32	5810231	5810262
chr10_799	p3	(TGC)9	27	5811314	5811340
chr10_800	p2	(TA)6	12	5813396	5813407
chr10_801	c	(CT)14(CA)12	52	5814902	5814953

chr10_802	p2	(AC)6	12	5819309	5819320
chr10_803	p4	(GTCA)6	24	5832292	5832315
chr10_804	p4	(TCTA)7	28	5864171	5864198
chr10_805	p4	(TTTA)6	24	5871955	5871978
chr10_806	p2	(GT)11	22	5877731	5877752
chr10_807	p3	(CAG)5	15	5885618	5885632
chr10_808	p2	(GT)7	14	5888890	5888903
chr10_809	p3	(GAT)7	21	5902347	5902367
chr10_810	p3	(AGC)5	15	5911340	5911354
chr10_811	p3	(TCC)6	18	5928365	5928382
chr10_812	p2	(TG)6	12	5949122	5949133
chr10_813	p2	(TA)6	12	5949813	5949824
chr10_814	p2	(TG)10	20	5952635	5952654
chr10_815	p2	(GT)26	52	5954630	5954681
chr10_816	p3	(GCT)5	15	5958557	5958571
chr10_817	p2	(TG)7	14	5959924	5959937
chr10_818	p2	(GT)6	12	5961143	5961154
chr10_819	p3	(ATT)5	15	5964109	5964123
chr10_820	p2	(GT)6	12	5974563	5974574
chr10_821	p2	(AC)6	12	5974752	5974763
chr10_822	p2	(TG)8	16	5993014	5993029
chr10_823	p2	(GT)9	18	6001497	6001514
chr10_824	p2	(AT)9	18	6003483	6003500
chr10_825	p2	(TA)7	14	6062088	6062101
chr10_826	p2	(AC)6	12	6072662	6072673
chr10_827	p3	(TAA)6	18	6098394	6098411
chr10_828	p3	(ATA)6	18	6103533	6103550
chr10_829	p2	(TG)17	34	6157897	6157930
chr10_830	p2	(CA)6	12	6158882	6158893
chr10_831	p2	(AT)6	12	6160142	6160153
chr10_832	c	gcgagctggctgcctgaatcgcaaagctgaaaagaagatgaacggacca	120	6164434	6164553
chr10_833	p2	(TA)12	24	6165447	6165470
chr10_834	p2	(GA)7	14	6169391	6169404
chr10_835	p2	(TG)23	46	6174171	6174216
chr10_836	p2	(TG)8	16	6198045	6198060
chr10_837	p2	(GA)6	12	6203160	6203171
chr10_838	p2	(TA)7	14	6210925	6210938
chr10_839	p2	(GA)8	16	6211395	6211410
chr10_840	p2	(CA)10	20	6217106	6217125
chr10_841	p2	(CA)9	18	6239331	6239348
chr10_842	p2	(GT)6	12	6260152	6260163
chr10_843	p2	(GT)9	18	6265591	6265608
chr10_844	c	(TC)6(TG)25	62	6274323	6274384
chr10_845	p2	(AC)10	20	6299736	6299755
chr10_846	p2	(AC)27	54	6300075	6300128
chr10_847	p3	(TAA)7	21	6301128	6301148
chr10_848	p2	(GA)6	12	6313162	6313173
chr10_849	p4	(TTTG)5	20	6315844	6315863
chr10_850	p2	(AC)14	28	6319471	6319498
chr10_851	p5	(TTTGA)5	25	6326774	6326798
chr10_852	p3	(TTA)5	15	6342336	6342350
chr10_853	p2	(AG)7	14	6354089	6354102

chr10_854	p2	(AC)6	12	6357229	6357240
chr10_855	p2	(AC)6	12	6368831	6368842
chr10_856	p3	(TAT)5	15	6380370	6380384
chr10_857	p2	(GT)6	12	6382754	6382765
chr10_858	c	(CA)9agggagcctttattgaccccc(CA)6	51	6397115	6397165
chr10_859	p2	(TG)10	20	6397503	6397522
chr10_860	p4	(ATAC)5	20	6409651	6409670
chr10_861	p2	(AC)6	12	6420422	6420433
chr10_862	p2	(GT)13	26	6420923	6420948
chr10_863	p3	(GAG)5	15	6432813	6432827
chr10_864	p4	(CATC)6	24	6456135	6456158
chr10_865	p5	(AAAAT)7	35	6459740	6459774
chr10_866	p2	(TG)8	16	6464577	6464592
chr10_867	p2	(AC)15	30	6469060	6469089
chr10_868	p2	(GT)10	20	6469237	6469256
chr10_869	p2	(GT)6	12	6473332	6473343
chr10_870	p2	(CT)7	14	6473597	6473610
chr10_871	p2	(TG)6	12	6474921	6474932
chr10_872	p2	(GT)6	12	6483987	6483998
chr10_873	p2	(AC)6	12	6500482	6500493
chr10_874	p2	(AG)6	12	6505980	6505991
chr10_875	p3	(GAG)5	15	6506252	6506266
chr10_876	p2	(GT)13	26	6525631	6525656
chr10_877	p2	(CA)7	14	6528708	6528721
chr10_878	p2	(GT)8	16	6529936	6529951
chr10_879	p2	(CT)8	16	6548713	6548728
chr10_880	p2	(TG)10	20	6554677	6554696
chr10_881	p2	(CA)8	16	6565341	6565356
chr10_882	p2	(CA)6	12	6588471	6588482
chr10_883	p2	(AC)10	20	6590865	6590884
chr10_884	p2	(CA)16	32	6610826	6610857
chr10_885	c	(GT)12gc(GT)9	44	6612102	6612145
chr10_886	p3	(TAT)5	15	6616588	6616602
chr10_887	p2	(AC)9	18	6617568	6617585
chr10_888	p2	(AC)6	12	6621775	6621786
chr10_889	p2	(CA)9	18	6626355	6626372
chr10_890	p2	(TG)18	36	6651426	6651461
chr10_891	p2	(TG)8	16	6681840	6681855
chr10_892	p2	(AT)6	12	6706168	6706179
chr10_893	p2	(GT)9	18	6745139	6745156
chr10_894	p3	(CAT)5	15	6751510	6751524
chr10_895	p2	(AC)9	18	6765971	6765988
chr10_896	p3	(GAT)5	15	6767217	6767231
chr10_897	p2	(AC)8	16	6770795	6770810
chr10_898	p5	(AAACA)5	25	6781107	6781131
chr10_899	p3	(CAT)6	18	6794671	6794688
chr10_900	p3	(GCT)6	18	6810521	6810538
chr10_901	p2	(AT)6	12	6817954	6817965
chr10_902	p3	(GTG)6	18	6824278	6824295
chr10_903	p2	(TG)9	18	6832235	6832252
chr10_904	p2	(TG)6	12	6837747	6837758
chr10_905	p2	(CA)6	12	6851437	6851448

chr10_906	c	(CA)22(GACA)6	68	6852721	6852788
chr10_907	p3	(TCT)11	33	6854665	6854697
chr10_908	p3	(CTT)5	15	6872550	6872564
chr10_909	p2	(CA)12	24	6876625	6876648
chr10_910	p2	(GT)6	12	6881612	6881623
chr10_911	p2	(TG)6	12	6884388	6884399
chr10_912	p2	(AG)6	12	6888111	6888122
chr10_913	p2	(AC)17	34	6889360	6889393
chr10_914	p2	(TC)6	12	6894382	6894393
chr10_915	p2	(TA)7	14	6908246	6908259
chr10_916	p2	(TG)7	14	6910892	6910905
chr10_917	p2	(AC)18	36	6911115	6911150
chr10_918	p3	(AAT)5	15	6939521	6939535
chr10_919	p4	(AAAC)5	20	6948007	6948026
chr10_920	p2	(CA)15	30	6951888	6951917
chr10_921	p4	(TGGA)6	24	6957419	6957442
chr10_922	p2	(TA)10	20	6964987	6965006
chr10_923	p2	(CT)9	18	6980807	6980824
chr10_924	p2	(AC)6	12	6990409	6990420
chr10_925	p2	(AG)6	12	6992121	6992132
chr10_926	p3	(CAG)6	18	6992730	6992747
chr10_927	p2	(CA)6	12	6995816	6995827
chr10_928	p3	(TCC)10	30	7004070	7004099
chr10_929	p2	(AC)14	28	7007302	7007329
chr10_930	p4	(TTGA)5	20	7008248	7008267
chr10_931	p2	(GT)6	12	7014593	7014604
chr10_932	p3	(GTT)6	18	7018370	7018387
chr10_933	p2	(GT)12	24	7023254	7023277
chr10_934	p2	(GT)14	28	7025459	7025486
chr10_935	p2	(GA)6	12	7027415	7027426
chr10_936	p2	(GT)15	30	7027825	7027854
chr10_937	p2	(GT)20	40	7052850	7052889
chr10_938	p2	(AC)6	12	7053953	7053964
chr10_939	p3	(AGG)8	24	7059487	7059510
chr10_940	p2	(AC)16	32	7062648	7062679
chr10_941	p3	(AGG)5	15	7069681	7069695
chr10_942	p2	(TG)6	12	7071313	7071324
chr10_943	p2	(CA)6	12	7077903	7077914
chr10_944	p3	(CAC)6	18	7082750	7082767
chr10_945	p2	(CA)24	48	7089476	7089523
chr10_946	p2	(CA)22	44	7095009	7095052
chr10_947	c	(AC)16(AG)8	48	7101929	7101976
chr10_948	p2	(CA)8	16	7105210	7105225
chr10_949	p2	(TG)6	12	7105644	7105655
chr10_950	c	(AAC)6ata(AAC)5	36	7117802	7117837
chr10_951	p2	(AC)12	24	7118384	7118407
chr10_952	p2	(CA)9	18	7120849	7120866
chr10_953	p2	(AC)7	14	7123208	7123221
chr10_954	p2	(CA)11	22	7127437	7127458
chr10_955	p2	(CA)14	28	7140681	7140708
chr10_956	p2	(AC)7	14	7162768	7162781
chr10_957	p2	(GT)6	12	7163844	7163855

chr10_958	p2	(CA)7	14	7169589	7169602
chr10_959	p2	(GT)7	14	7171866	7171879
chr10_960	c	(TC)13c(CA)16	59	7172519	7172577
chr10_961	p2	(GT)17	34	7178268	7178301
chr10_962	p3	(GCT)5	15	7179324	7179338
chr10_963	p2	(AC)20	40	7186430	7186469
chr10_964	c	ıttactctgtctcatatctccaacatttgtacagttacactgctgtactaacttctį	93	7186607	7186699
chr10_965	p2	(TG)20	40	7187217	7187256
chr10_966	p2	(CA)7	14	7189141	7189154
chr10_967	c	acaccaaactctacataacacacatgtacagacagagcagtgcactcagat	157	7191765	7191921
chr10_968	c	(CA)7(CT)10	34	7192865	7192898
chr10_969	c	(CTCTT)13(CTCCT)6	95	7194979	7195073
chr10_970	p2	(AC)7	14	7196716	7196729
chr10_971	p2	(CA)21	42	7196907	7196948
chr10_972	p2	(GT)26	52	7197052	7197103
chr10_973	p2	(CA)23	46	7197835	7197880
chr10_974	p2	(GT)12	24	7199704	7199727
chr10_975	p2	(GT)16	32	7210580	7210611
chr10_976	p2	(TG)9	18	7220269	7220286
chr10_977	p2	(GT)10	20	7222776	7222795
chr10_978	p3	(TTG)7	21	7224586	7224606
chr10_979	p2	(AC)8	16	7224755	7224770
chr10_980	p2	(TC)7	14	7225211	7225224
chr10_981	p2	(AC)11	22	7226101	7226122
chr10_982	p2	(TG)7	14	7234455	7234468
chr10_983	p2	(TG)12	24	7234580	7234603
chr10_984	p3	(TGT)5	15	7236389	7236403
chr10_985	p2	(AC)7	14	7238525	7238538
chr10_986	p2	(TG)9	18	7241340	7241357
chr10_987	p2	(TG)9	18	7241897	7241914
chr10_988	p2	(AC)23	46	7243676	7243721
chr10_989	p2	(TA)10	20	7246096	7246115
chr10_990	p2	(GT)9	18	7247814	7247831
chr10_991	p2	(GT)34	68	7250525	7250592
chr10_992	p2	(TG)14	28	7250757	7250784
chr10_993	p2	(GT)8	16	7257735	7257750
chr10_994	p2	(GT)6	12	7261842	7261853
chr10_995	c	(AG)6cgcgcgtc(CCT)6	38	7265750	7265787
chr10_996	p3	(CCT)5	15	7266739	7266753
chr10_997	p2	(TG)6	12	7283971	7283982
chr10_998	p2	(AG)7	14	7288506	7288519
chr10_999	p2	(GT)23	46	7290316	7290361
chr10_1000	p2	(CA)6	12	7292573	7292584
chr10_1001	p3	(ATT)5	15	7298114	7298128
chr10_1002	c	tacagagaaatggacacattctcacacacacactcttgtgtgtatagtagtg	93	7309799	7309891
chr10_1003	p2	(GT)6	12	7316817	7316828
chr10_1004	p2	(AC)9	18	7318417	7318434
chr10_1005	p2	(AC)19	38	7318780	7318817
chr10_1006	p2	(AC)10	20	7340797	7340816
chr10_1007	p2	(GT)6	12	7341245	7341256
chr10_1008	p2	(CA)9	18	7343242	7343259
chr10_1009	p2	(AT)6	12	7345499	7345510

chr10_1010	p2	(AC)6	12	7345934	7345945
chr10_1011	p2	(AC)14	28	7346261	7346288
chr10_1012	p2	(AC)11	22	7348286	7348307
chr10_1013	c	tgcaaggacgaacagacagtgaacacaagcataggatgaaagcagggg:	142	7350690	7350831
chr10_1014	p2	(GT)16	32	7351734	7351765
chr10_1015	p2	(TG)19	38	7361739	7361776
chr10_1016	p2	(TG)17	34	7365876	7365909
chr10_1017	p2	(GT)6	12	7366870	7366881
chr10_1018	p2	(GT)6	12	7370246	7370257
chr10_1019	p2	(CA)6	12	7370641	7370652
chr10_1020	p2	(GT)18	36	7370834	7370869
chr10_1021	p2	(CA)8	16	7377753	7377768
chr10_1022	p2	(TG)18	36	7382167	7382202
chr10_1023	p2	(TG)21	42	7393509	7393550
chr10_1024	p3	(CTC)5	15	7407431	7407445
chr10_1025	p3	(CTC)8	24	7423620	7423643
chr10_1026	p2	(AC)16	32	7423786	7423817
chr10_1027	p2	(CA)16	32	7423947	7423978
chr10_1028	p2	(AC)6	12	7424108	7424119
chr10_1029	p2	(CA)10	20	7438363	7438382
chr10_1030	p2	(GT)6	12	7440831	7440842
chr10_1031	p3	(CTC)5	15	7449008	7449022
chr10_1032	p2	(CA)14	28	7450787	7450814
chr10_1033	p3	(AGC)9	27	7452288	7452314
chr10_1034	p2	(CA)18	36	7455279	7455314
chr10_1035	p2	(AC)9	18	7455449	7455466
chr10_1036	p2	(AC)6	12	7461614	7461625
chr10_1037	p2	(CA)12	24	7462955	7462978
chr10_1038	p2	(TG)9	18	7463396	7463413
chr10_1039	p2	(GT)9	18	7466085	7466102
chr10_1040	p2	(CA)15	30	7470529	7470558
chr10_1041	p2	(AC)16	32	7472824	7472855
chr10_1042	p2	(GT)7	14	7473322	7473335
chr10_1043	p2	(GT)7	14	7486715	7486728
chr10_1044	p2	(GT)6	12	7487835	7487846
chr10_1045	p2	(AC)12	24	7487950	7487973
chr10_1046	p2	(GT)8	16	7491269	7491284
chr10_1047	p2	(AG)6	12	7495024	7495035
chr10_1048	p2	(TG)9	18	7497562	7497579
chr10_1049	p3	(GAG)5	15	7515165	7515179
chr10_1050	p2	(GA)6	12	7532848	7532859
chr10_1051	p2	(AG)9	18	7537752	7537769
chr10_1052	c	(TG)9tctc(TG)13	48	7544357	7544404
chr10_1053	p2	(TG)6	12	7549250	7549261
chr10_1054	c	(CA)13atcttcctgc(TG)10	56	7553986	7554041
chr10_1055	p2	(GT)6	12	7558337	7558348
chr10_1056	c	ıttggccacaggagactgcaagtgcctattaaagagtgggaggctttggca(	113	7571737	7571849
chr10_1057	p2	(AC)14	28	7576191	7576218
chr10_1058	p3	(AGC)6	18	7589692	7589709
chr10_1059	p2	(TG)9	18	7594334	7594351
chr10_1060	c	ttatcagtgtccatgtagcctctttatgtctctgcatgcaaacagctgcatggt:	104	7594544	7594647
chr10_1061	p2	(CT)6	12	7598507	7598518

chr10_1062	p2	(CA)10	20	7600724	7600743
chr10_1063	p2	(CA)10	20	7605412	7605431
chr10_1064	p2	(CA)6	12	7611603	7611614
chr10_1065	c	(CT)7cactcact(CA)6	34	7614322	7614355
chr10_1066	p3	(TTG)7	21	7628381	7628401
chr10_1067	p2	(AC)10	20	7631075	7631094
chr10_1068	c	[illegible]cagagcaggagagagtggagaacaaacaggtggagagagcaattgtgc[illegible]	92	7638178	7638269
chr10_1069	c	(GT)10gc(GT)7	36	7640198	7640233
chr10_1070	p3	(CAG)5	15	7646079	7646093
chr10_1071	p2	(TG)22	44	7652410	7652453
chr10_1072	p4	(GAGG)5	20	7653497	7653516
chr10_1073	p2	(AC)6	12	7654251	7654262
chr10_1074	p2	(CA)6	12	7655561	7655572
chr10_1075	p2	(CA)16	32	7659176	7659207
chr10_1076	p2	(TG)15	30	7659392	7659421
chr10_1077	p2	(GT)19	38	7666059	7666096
chr10_1078	p2	(TG)7	14	7676696	7676709
chr10_1079	p2	(AT)7	14	7702257	7702270
chr10_1080	p3	(TCC)5	15	7707399	7707413
chr10_1081	p2	(TG)6	12	7719852	7719863
chr10_1082	p2	(TG)13	26	7724609	7724634
chr10_1083	p4	(ATCC)5	20	7730403	7730422
chr10_1084	p3	(ATA)10	30	7730674	7730703
chr10_1085	p2	(CT)6	12	7734327	7734338
chr10_1086	p3	(CCT)5	15	7734501	7734515
chr10_1087	p3	(CCT)5	15	7737075	7737089
chr10_1088	p3	(TTA)5	15	7739319	7739333
chr10_1089	p2	(TG)6	12	7748042	7748053
chr10_1090	c	(AG)8acagagaga(GT)9	43	7751113	7751155
chr10_1091	p3	(GAG)5	15	7757026	7757040
chr10_1092	p2	(AT)6	12	7773883	7773894
chr10_1093	p2	(TG)15	30	7783683	7783712
chr10_1094	p2	(GT)14	28	7783830	7783857
chr10_1095	p2	(GA)12	24	7784240	7784263
chr10_1096	p2	(CA)6	12	7784429	7784440
chr10_1097	p2	(CA)6	12	7784596	7784607
chr10_1098	p2	(CA)16	32	7784731	7784762
chr10_1099	p3	(AGC)5	15	7787942	7787956
chr10_1100	p3	(ATG)5	15	7793877	7793891
chr10_1101	p2	(AC)15	30	7794493	7794522
chr10_1102	p2	(CA)17	34	7796195	7796228
chr10_1103	p3	(ACA)6	18	7798711	7798728
chr10_1104	p2	(CA)7	14	7804924	7804937
chr10_1105	p3	(AAC)5	15	7810476	7810490
chr10_1106	p2	(CA)9	18	7814460	7814477
chr10_1107	c	(CA)20(GA)11	62	7819511	7819572
chr10_1108	p2	(AC)8	16	7827536	7827551
chr10_1109	p2	(CA)6	12	7835716	7835727
chr10_1110	p2	(CA)6	12	7841022	7841033
chr10_1111	p4	(CCTA)5	20	7842265	7842284
chr10_1112	p2	(CA)6	12	7849418	7849429
chr10_1113	p2	(TG)20	40	7850525	7850564

chr10_1114	c	(GC)9(AC)6	30	7866706	7866735
chr10_1115	p2	(AC)7	14	7867588	7867601
chr10_1116	c	(GT)15gc(GT)15	62	7872005	7872066
chr10_1117	p2	(GA)6	12	7902863	7902874
chr10_1118	p4	(AATG)5	20	7904633	7904652
chr10_1119	p2	(GA)6	12	7909508	7909519
chr10_1120	c	;tgtgttagctggcagctcctgtcggtgtcagtgctgttagcatctctacagc(	113	7910996	7911108
chr10_1121	p3	(TTC)10	30	7911731	7911760
chr10_1122	p2	(CT)11	22	7920838	7920859
chr10_1123	c	(TC)6(TG)26	64	7924933	7924996
chr10_1124	p2	(CT)8	16	7925904	7925919
chr10_1125	p2	(TG)17	34	7928671	7928704
chr10_1126	p3	(ACC)6	18	7930430	7930447
chr10_1127	p2	(GT)7	14	7936538	7936551
chr10_1128	p2	(TC)14	28	7940859	7940886
chr10_1129	p2	(TA)7	14	7954011	7954024
chr10_1130	p3	(TTC)6	18	7955686	7955703
chr10_1131	p6	(TACATA)7	42	7986114	7986155
chr10_1132	p2	(AG)6	12	7999446	7999457
chr10_1133	p2	(TG)24	48	8005074	8005121
chr10_1134	p2	(AC)10	20	8005421	8005440
chr10_1135	p2	(AG)6	12	8011698	8011709
chr10_1136	p2	(AC)6	12	8011848	8011859
chr10_1137	p3	(CCA)6	18	8023329	8023346
chr10_1138	c	(GT)11gctgtcctgcaggagaaacagccg(CT)7	60	8029245	8029304
chr10_1139	c	(GA)13gc(GA)16(GT)9	78	8031721	8031798
chr10_1140	p2	(AC)58	116	8034792	8034907
chr10_1141	p3	(GAT)6	18	8038987	8039004
chr10_1142	p2	(AC)21	42	8039942	8039983
chr10_1143	p2	(TG)6	12	8042595	8042606
chr10_1144	p2	(AC)7	14	8042962	8042975
chr10_1145	p2	(AC)6	12	8045280	8045291
chr10_1146	c	taca(GT)6tacagtgtgtgtgacgatgaagtgagtcttaattagcctcctc	84	8045544	8045627
chr10_1147	p2	(TG)8	16	8046683	8046698
chr10_1148	p2	(TG)6	12	8049557	8049568
chr10_1149	p2	(CA)21	42	8049688	8049729
chr10_1150	p2	(AC)8	16	8055342	8055357
chr10_1151	p2	(AC)17	34	8055846	8055879
chr10_1152	p2	(GT)8	16	8056445	8056460
chr10_1153	p2	(AC)11	22	8060743	8060764
chr10_1154	p2	(CA)8	16	8064529	8064544
chr10_1155	p2	(CA)8	16	8065325	8065340
chr10_1156	p3	(TAA)6	18	8078638	8078655
chr10_1157	p2	(AC)11	22	8079016	8079037
chr10_1158	p2	(TG)18	36	8080443	8080478
chr10_1159	c	tcaatgctgaaccacttcacttcaatgagcattcagtgtcaaacaaacagct(	173	8080601	8080773
chr10_1160	p2	(AC)6	12	8081739	8081750
chr10_1161	p2	(AC)10	20	8082977	8082996
chr10_1162	p2	(GT)6	12	8086816	8086827
chr10_1163	p2	(GT)15	30	8087518	8087547
chr10_1164	c	agtact(AC)8ccacagaacacactctgccggtaatgactacttctccctc	204	8090739	8090942
chr10_1165	p2	(TC)7	14	8094292	8094305

chr10_1166	p2	(AC)15	30	8094410	8094439
chr10_1167	p3	(CTC)6	18	8097863	8097880
chr10_1168	p2	(AC)12	24	8098023	8098046
chr10_1169	p2	(AC)6	12	8100014	8100025
chr10_1170	p2	(AC)6	12	8100163	8100174
chr10_1171	p2	(TG)6	12	8101478	8101489
chr10_1172	c	gccttttatagctggagacaaaatgtgtgattttgcatacttatgaaaagtcat	99	8101614	8101712
chr10_1173	p2	(CA)16	32	8102937	8102968
chr10_1174	p2	(AG)7	14	8104985	8104998
chr10_1175	p2	(AC)14	28	8105141	8105168
chr10_1176	p3	(AAT)6	18	8113123	8113140
chr10_1177	p2	(GA)7	14	8116846	8116859
chr10_1178	p2	(GT)6	12	8121059	8121070
chr10_1179	p2	(TG)7	14	8121293	8121306
chr10_1180	p2	(AG)10	20	8122803	8122822
chr10_1181	p2	(AC)9	18	8126170	8126187
chr10_1182	p2	(CA)9	18	8126523	8126540
chr10_1183	p3	(CTC)5	15	8126654	8126668
chr10_1184	c	(TA)9(CA)14	46	8143737	8143782
chr10_1185	p2	(AC)7	14	8149365	8149378
chr10_1186	p2	(CA)17	34	8149881	8149914
chr10_1187	p2	(TG)6	12	8150246	8150257
chr10_1188	p2	(CA)8	16	8150359	8150374
chr10_1189	p3	(TCT)15	45	8152379	8152423
chr10_1190	p3	(GAG)6	18	8165834	8165851
chr10_1191	p2	(CA)6	12	8169262	8169273
chr10_1192	p2	(TG)15	30	8170274	8170303
chr10_1193	p2	(GT)6	12	8171578	8171589
chr10_1194	p2	(AC)8	16	8176320	8176335
chr10_1195	c	(TAT)5(TAC)6	33	8180834	8180866
chr10_1196	p3	(GAG)5	15	8189128	8189142
chr10_1197	p2	(CA)9	18	8192384	8192401
chr10_1198	c	(GT)18a(TG)6	49	8194787	8194835
chr10_1199	p2	(AT)7	14	8202771	8202784
chr10_1200	p2	(AG)6	12	8204241	8204252
chr10_1201	c	(CA)6agacggaaaac(AG)6	35	8207137	8207171
chr10_1202	p2	(AC)28	56	8211128	8211183
chr10_1203	p2	(AC)13	26	8213029	8213054
chr10_1204	p2	(AC)8	16	8226759	8226774
chr10_1205	p2	(AC)6	12	8229243	8229254
chr10_1206	c	gatgtcatctacagcccatctgattggctggcaagggacagcgcccatttcc	193	8237243	8237435
chr10_1207	p4	(ATAC)6	24	8248072	8248095
chr10_1208	p2	(AC)6	12	8252531	8252542
chr10_1209	p2	(CT)6	12	8256870	8256881
chr10_1210	p3	(CTG)6	18	8260440	8260457
chr10_1211	p3	(TGA)5	15	8265235	8265249
chr10_1212	p2	(AC)19	38	8268972	8269009
chr10_1213	p2	(AC)6	12	8275566	8275577
chr10_1214	p2	(GT)13	26	8278094	8278119
chr10_1215	c	(GACA)10(GATA)9	76	8290097	8290172
chr10_1216	p2	(GT)8	16	8294832	8294847
chr10_1217	p2	(AC)17	34	8295756	8295789

chr10_1218	p2	(AC)8	16	8308903	8308918
chr10_1219	p2	(AC)7	14	8309130	8309143
chr10_1220	p2	(CA)12	24	8309543	8309566
chr10_1221	p2	(TG)6	12	8310768	8310779
chr10_1222	p2	(CA)7	14	8313908	8313921
chr10_1223	p3	(ACA)5	15	8316844	8316858
chr10_1224	p2	(GT)6	12	8317516	8317527
chr10_1225	p2	(CA)11	22	8319080	8319101
chr10_1226	p2	(AC)7	14	8322489	8322502
chr10_1227	p2	(AC)6	12	8324089	8324100
chr10_1228	p2	(AC)8	16	8332025	8332040
chr10_1229	p3	(TAC)6	18	8338461	8338478
chr10_1230	p2	(AC)13	26	8371748	8371773
chr10_1231	p3	(GAG)5	15	8372781	8372795
chr10_1232	p2	(GT)6	12	8374503	8374514
chr10_1233	p2	(TG)18	36	8375456	8375491
chr10_1234	p3	(TAA)6	18	8390150	8390167
chr10_1235	p2	(GT)14	28	8419623	8419650
chr10_1236	p2	(GT)14	28	8419773	8419800
chr10_1237	p2	(AC)6	12	8424354	8424365
chr10_1238	p2	(CA)6	12	8424785	8424796
chr10_1239	p2	(AG)26	52	8426263	8426314
chr10_1240	p2	(AC)21	42	8426668	8426709
chr10_1241	c	tctgtttttagggatcacttcctcttcctgtgacagaccttgttatatttagccac	123	8430327	8430449
chr10_1242	p2	(CA)6	12	8432557	8432568
chr10_1243	p5	(AATCT)5	25	8435200	8435224
chr10_1244	p2	(AG)6	12	8440130	8440141
chr10_1245	p2	(CA)6	12	8447172	8447183
chr10_1246	p2	(CA)8	16	8447315	8447330
chr10_1247	p2	(AG)16	32	8447460	8447491
chr10_1248	p2	(TG)20	40	8469311	8469350
chr10_1249	p2	(GT)6	12	8473928	8473939
chr10_1250	p2	(CA)8	16	8475969	8475984
chr10_1251	p2	(GA)6	12	8477223	8477234
chr10_1252	p3	(TCC)6	18	8477814	8477831
chr10_1253	p2	(AC)6	12	8478969	8478980
chr10_1254	c	(AG)7acaggg(GA)16	52	8493181	8493232
chr10_1255	p3	(TTA)7	21	8501534	8501554
chr10_1256	p2	(GT)7	14	8505598	8505611
chr10_1257	p2	(AC)16	32	8516348	8516379
chr10_1258	p3	(AAG)7	21	8533031	8533051
chr10_1259	p2	(AC)6	12	8553361	8553372
chr10_1260	p2	(CA)6	12	8558951	8558962
chr10_1261	c	cactggagacatgacaaggagccagtcactctgcactgcatttacatgcac	132	8560534	8560665
chr10_1262	p2	(CT)6	12	8561204	8561215
chr10_1263	p2	(GT)9	18	8561821	8561838
chr10_1264	p2	(CA)22	44	8562364	8562407
chr10_1265	p2	(AC)6	12	8571527	8571538
chr10_1266	p2	(CA)12	24	8572091	8572114
chr10_1267	p2	(GT)19	38	8572278	8572315
chr10_1268	p2	(CA)6	12	8573307	8573318
chr10_1269	p2	(AC)11	22	8578540	8578561

chr10_1270	p2	(AC)6	12	8581808	8581819
chr10_1271	p2	(CA)40	80	8583409	8583488
chr10_1272	c	5g(CA)17gtatgatctaactatca(AC)10gcacacacacgcacacg	135	8588238	8588372
chr10_1273	p2	(TG)7	14	8593344	8593357
chr10_1274	c	(GT)6ac(GT)9	32	8595564	8595595
chr10_1275	c	(CA)8cgcgcg(CA)6	34	8595744	8595777
chr10_1276	p2	(AG)6	12	8596810	8596821
chr10_1277	p3	(AGG)5	15	8600665	8600679
chr10_1278	p2	(GT)12	24	8604881	8604904
chr10_1279	p2	(CA)22	44	8607851	8607894
chr10_1280	p2	(AC)7	14	8624589	8624602
chr10_1281	p2	(AC)17	34	8625322	8625355
chr10_1282	p2	(GT)6	12	8626371	8626382
chr10_1283	p2	(AC)16	32	8627614	8627645
chr10_1284	p2	(AC)11	22	8629957	8629978
chr10_1285	c	cggaggtgagttggagtagtggctggacgtgtgtgaggaggtggaggaa	91	8632575	8632665
chr10_1286	p2	(AG)7	14	8638422	8638435
chr10_1287	p2	(CA)7	14	8644821	8644834
chr10_1288	p2	(TG)6	12	8644983	8644994
chr10_1289	p3	(ATA)7	21	8645291	8645311
chr10_1290	p2	(CT)6	12	8662763	8662774
chr10_1291	p2	(GT)8	16	8674780	8674795
chr10_1292	p2	(TG)13	26	8675437	8675462
chr10_1293	c	(GT)7ac(GT)11	38	8680734	8680771
chr10_1294	p2	(CA)9	18	8684484	8684501
chr10_1295	p3	(GTG)6	18	8685801	8685818
chr10_1296	p2	(CT)9	18	8688149	8688166
chr10_1297	p2	(GA)6	12	8689741	8689752
chr10_1298	p2	(GT)12	24	8702977	8703000
chr10_1299	p2	(AC)7	14	8706637	8706650
chr10_1300	p2	(CA)13	26	8709110	8709135
chr10_1301	p2	(CA)15	30	8716036	8716065
chr10_1302	p3	(TTC)6	18	8725892	8725909
chr10_1303	p3	(TTA)5	15	8726430	8726444
chr10_1304	p2	(GT)8	16	8731971	8731986
chr10_1305	p2	(TG)6	12	8762998	8763009
chr10_1306	p3	(GTG)6	18	8770040	8770057
chr10_1307	p3	(CTG)5	15	8786399	8786413
chr10_1308	p2	(TG)31	62	8787319	8787380
chr10_1309	p3	(TAA)5	15	8790166	8790180
chr10_1310	p2	(AC)7	14	8800931	8800944
chr10_1311	p2	(AT)8	16	8805916	8805931
chr10_1312	p2	(GT)7	14	8806313	8806326
chr10_1313	p2	(AC)10	20	8818744	8818763
chr10_1314	p2	(AC)7	14	8819270	8819283
chr10_1315	c	(TC)6(AC)6	24	8840447	8840470
chr10_1316	p2	(TG)15	30	8863526	8863555
chr10_1317	p2	(TG)16	32	8869747	8869778
chr10_1318	p2	(CA)6	12	8897267	8897278
chr10_1319	p3	(AGG)6	18	8897764	8897781
chr10_1320	p3	(AGG)6	18	8902231	8902248
chr10_1321	p3	(CTA)5	15	8907983	8907997

chr10_1322	p3	(AGG)6	18	8909980	8909997
chr10_1323	p2	(CA)12	24	8917085	8917108
chr10_1324	p3	(TAT)5	15	8927702	8927716
chr10_1325	c	(CA)14t(AC)7	43	8930794	8930836
chr10_1326	p2	(CA)9	18	8945998	8946015
chr10_1327	p2	(TA)7	14	8955165	8955178
chr10_1328	p2	(GT)11	22	8957882	8957903
chr10_1329	c	(TG)14(AG)6	40	8958406	8958445
chr10_1330	p3	(ACA)8	24	8963140	8963163
chr10_1331	c	(AG)8tgagagaa(TG)6	36	8965678	8965713
chr10_1332	p4	(CATC)8	32	8973442	8973473
chr10_1333	p2	(AG)8	16	8985502	8985517
chr10_1334	p3	(ATT)8	24	8990385	8990408
chr10_1335	p2	(AG)8	16	8991227	8991242
chr10_1336	p2	(CA)9	18	8992999	8993016
chr10_1337	p2	(AC)10	20	9006591	9006610
chr10_1338	p2	(TG)7	14	9026075	9026088
chr10_1339	p2	(TG)6	12	9034483	9034494
chr10_1340	p2	(CT)16	32	9046739	9046770
chr10_1341	p2	(CA)8	16	9051788	9051803
chr10_1342	p2	(TG)6	12	9051948	9051959
chr10_1343	p2	(AT)7	14	9058190	9058203
chr10_1344	p2	(TG)11	22	9059732	9059753
chr10_1345	p2	(TC)6	12	9078581	9078592
chr10_1346	p2	(GT)12	24	9082722	9082745
chr10_1347	p2	(GT)26	52	9089878	9089929
chr10_1348	p4	(ATCC)5	20	9099297	9099316
chr10_1349	p2	(GT)7	14	9108051	9108064
chr10_1350	p2	(TG)7	14	9108815	9108828
chr10_1351	p2	(CT)6	12	9115445	9115456
chr10_1352	p2	(TG)10	20	9130667	9130686
chr10_1353	p2	(TG)7	14	9133192	9133205
chr10_1354	p4	(TCCA)5	20	9147502	9147521
chr10_1355	p2	(AC)6	12	9150608	9150619
chr10_1356	c	(CAA)5aaaaacaaatgtcctaataagt(CA)6ggggaac(AT)8	72	9151618	9151689
chr10_1357	p2	(TG)11	22	9156758	9156779
chr10_1358	p2	(TG)6	12	9156997	9157008
chr10_1359	p2	(GT)6	12	9160886	9160897
chr10_1360	p2	(CT)6	12	9161659	9161670
chr10_1361	p2	(CT)9	18	9161847	9161864
chr10_1362	p2	(TG)9	18	9164868	9164885
chr10_1363	p5	(AAATC)6	30	9171833	9171862
chr10_1364	p2	(TC)6	12	9184332	9184343
chr10_1365	p2	(AC)8	16	9190788	9190803
chr10_1366	p2	(GT)15	30	9209223	9209252
chr10_1367	p2	(TG)6	12	9212089	9212100
chr10_1368	p4	(GATG)6	24	9233096	9233119
chr10_1369	p3	(AAC)5	15	9236206	9236220
chr10_1370	p2	(TC)8	16	9236910	9236925
chr10_1371	p2	(AG)23	46	9240823	9240868
chr10_1372	p2	(AC)6	12	9242248	9242259
chr10_1373	c	(CT)12cacacacg(CA)7	46	9247982	9248027

chr10_1374	p2	(TG)15	30	9251047	9251076
chr10_1375	p2	(TC)10	20	9264384	9264403
chr10_1376	p2	(TG)9	18	9265260	9265277
chr10_1377	p2	(GT)7	14	9265985	9265998
chr10_1378	p2	(CA)6	12	9267680	9267691
chr10_1379	p2	(CA)8	16	9300356	9300371
chr10_1380	c	(TG)22(AG)7	58	9310020	9310077
chr10_1381	p2	(GT)10	20	9317010	9317029
chr10_1382	c	(AC)9atacatacacacatacat(AC)9at(AC)13	82	9332413	9332494
chr10_1383	c	(CT)8ccactacattcactaatacattcattattcc(CT)6	59	9337485	9337543
chr10_1384	p2	(TG)6	12	9338978	9338989
chr10_1385	p2	(GA)6	12	9339408	9339419
chr10_1386	p2	(TG)8	16	9339573	9339588
chr10_1387	p2	(GT)13	26	9340089	9340114
chr10_1388	p2	(CA)12	24	9343719	9343742
chr10_1389	p3	(ATA)5	15	9348950	9348964
chr10_1390	p2	(CA)12	24	9352776	9352799
chr10_1391	p2	(TG)8	16	9359331	9359346
chr10_1392	p2	(AC)7	14	9363601	9363614
chr10_1393	p2	(GT)11	22	9369608	9369629
chr10_1394	p2	(CT)7	14	9373189	9373202
chr10_1395	p2	(AT)15	30	9394693	9394722
chr10_1396	p2	(TA)8	16	9396156	9396171
chr10_1397	p2	(TG)8	16	9407083	9407098
chr10_1398	p2	(AG)6	12	9408767	9408778
chr10_1399	p2	(TA)6	12	9426970	9426981
chr10_1400	p2	(AC)6	12	9428387	9428398
chr10_1401	p2	(AT)7	14	9435373	9435386
chr10_1402	p2	(TC)14	28	9439362	9439389
chr10_1403	p2	(GA)14	28	9444894	9444921
chr10_1404	c	(GT)9a(TG)6	31	9446491	9446521
chr10_1405	p2	(AC)6	12	9457462	9457473
chr10_1406	p2	(AT)8	16	9462207	9462222
chr10_1407	p2	(AT)8	16	9463495	9463510
chr10_1408	p2	(CA)11	22	9495586	9495607
chr10_1409	p2	(GA)6	12	9496853	9496864
chr10_1410	p2	(AC)8	16	9514673	9514688
chr10_1411	p2	(TG)9	18	9515058	9515075
chr10_1412	p2	(CA)6	12	9516331	9516342
chr10_1413	p3	(CAA)5	15	9523897	9523911
chr10_1414	p2	(AC)6	12	9528108	9528119
chr10_1415	p2	(GT)7	14	9542573	9542586
chr10_1416	p2	(TG)6	12	9554321	9554332
chr10_1417	p4	(ATCT)5	20	9559889	9559908
chr10_1418	p2	(AC)10	20	9574858	9574877
chr10_1419	p2	(TC)6	12	9587623	9587634
chr10_1420	p2	(CA)15	30	9590272	9590301
chr10_1421	p2	(TG)6	12	9591497	9591508
chr10_1422	p3	(TTA)5	15	9599546	9599560
chr10_1423	p2	(CT)11	22	9600958	9600979
chr10_1424	p2	(TG)7	14	9605806	9605819
chr10_1425	c	(AC)9(AG)9	36	9624434	9624469

chr10_1426	p4	(AATC)6	24	9634445	9634468
chr10_1427	p2	(AC)6	12	9645468	9645479
chr10_1428	p2	(CT)6	12	9645789	9645800
chr10_1429	p2	(AC)21	42	9647274	9647315
chr10_1430	p2	(CA)8	16	9647426	9647441
chr10_1431	p2	(GT)26	52	9649397	9649448
chr10_1432	c	TG)7ta(TG)6cgtatacagtatgtacatgtaatcatgtatatgtata(TG)	81	9649802	9649882
chr10_1433	p3	(TTA)6	18	9662741	9662758
chr10_1434	p2	(CG)6	12	9668300	9668311
chr10_1435	p2	(TG)7	14	9680922	9680935
chr10_1436	p2	(CA)6	12	9693307	9693318
chr10_1437	p2	(AG)8	16	9699167	9699182
chr10_1438	p4	(TATC)5	20	9700356	9700375
chr10_1439	p2	(CA)6	12	9725477	9725488
chr10_1440	p3	(TGT)6	18	9730489	9730506
chr10_1441	p2	(CA)13	26	9749907	9749932
chr10_1442	p2	(CT)7	14	9750833	9750846
chr10_1443	p3	(TGC)5	15	9759318	9759332
chr10_1444	p2	(TG)8	16	9781742	9781757
chr10_1445	p2	(CA)9	18	9783607	9783624
chr10_1446	p2	(GT)12	24	9786024	9786047
chr10_1447	p3	(AAT)6	18	9788964	9788981
chr10_1448	p2	(TG)18	36	9793014	9793049
chr10_1449	p2	(AC)8	16	9804365	9804380
chr10_1450	p2	(GT)18	36	9806229	9806264
chr10_1451	p2	(AC)9	18	9808484	9808501
chr10_1452	p2	(GT)14	28	9822537	9822564
chr10_1453	p2	(AC)6	12	9822680	9822691
chr10_1454	p2	(CA)6	12	9838775	9838786
chr10_1455	p2	(TG)6	12	9863810	9863821
chr10_1456	p2	(CA)11	22	9866283	9866304
chr10_1457	p2	(CA)6	12	9882949	9882960
chr10_1458	p2	(TG)9	18	9883136	9883153
chr10_1459	p2	(GA)8	16	9883872	9883887
chr10_1460	p2	(CA)7	14	9884064	9884077
chr10_1461	p2	(TG)6	12	9893444	9893455
chr10_1462	p2	(AG)6	12	9907683	9907694
chr10_1463	p2	(TG)7	14	9910339	9910352
chr10_1464	p2	(TG)11	22	9929243	9929264
chr10_1465	p2	(GT)6	12	9933297	9933308
chr10_1466	p2	(CA)6	12	9933732	9933743
chr10_1467	c	(AGA)5aaaagaagaagaag(AAC)5	44	9939421	9939464
chr10_1468	p2	(CA)7	14	9954189	9954202
chr10_1469	p2	(TG)6	12	9979021	9979032
chr10_1470	p2	(AT)11	22	9981911	9981932
chr10_1471	p2	(AT)11	22	9986915	9986936
chr10_1472	p4	(AGTC)6	24	9990351	9990374
chr10_1473	p2	(AC)7	14	9991371	9991384
chr10_1474	p2	(CT)6	12	9998545	9998556
chr10_1475	p2	(AC)8	16	10007652	10007667
chr10_1476	p3	(AGC)9	27	10015844	10015870
chr10_1477	p2	(CA)30	60	10022577	10022636

chr10_1478	p2	(AC)6	12	10023033	10023044
chr10_1479	p2	(CA)6	12	10023780	10023791
chr10_1480	p2	(CA)8	16	10027872	10027887
chr10_1481	p2	(CT)13	26	10027997	10028022
chr10_1482	p2	(CA)17	34	10028405	10028438
chr10_1483	p2	(GT)6	12	10034705	10034716
chr10_1484	p2	(GT)20	40	10040436	10040475
chr10_1485	p2	(CA)6	12	10041121	10041132
chr10_1486	p3	(CTC)7	21	10041951	10041971
chr10_1487	p4	(CTTT)7	28	10051511	10051538
chr10_1488	p2	(CA)7	14	10057962	10057975
chr10_1489	c	(GA)8cagagagac(AG)11	47	10062183	10062229
chr10_1490	p2	(AC)8	16	10065236	10065251
chr10_1491	p2	(AC)17	34	10069413	10069446
chr10_1492	p2	(TC)7	14	10070969	10070982
chr10_1493	p2	(TG)6	12	10071418	10071429
chr10_1494	p2	(AC)20	40	10079970	10080009
chr10_1495	p2	(TG)13	26	10086655	10086680
chr10_1496	p4	(TTTA)5	20	10099090	10099109
chr10_1497	p2	(GT)9	18	10121935	10121952
chr10_1498	p3	(TTA)10	30	10122855	10122884
chr10_1499	p2	(TG)36	72	10135199	10135270
chr10_1500	p2	(CA)22	44	10164759	10164802
chr10_1501	p2	(TA)6	12	10167960	10167971
chr10_1502	p2	(TG)6	12	10168407	10168418
chr10_1503	p2	(TA)7	14	10177172	10177185
chr10_1504	p2	(TG)6	12	10183303	10183314
chr10_1505	p2	(CA)14	28	10188376	10188403
chr10_1506	c	(TC)16(AC)18	68	10189002	10189069
chr10_1507	p2	(CA)9	18	10196445	10196462
chr10_1508	p2	(GT)8	16	10201094	10201109
chr10_1509	p3	(CTC)5	15	10206993	10207007
chr10_1510	p3	(TTG)5	15	10218515	10218529
chr10_1511	p2	(CA)9	18	10219009	10219026
chr10_1512	c	nnnnnnnnnnnnnnnnnnnnnnnnnnnnnnnnnnnnnnnnnnnnnn	127	10225495	10225621
chr10_1513	p3	(CTC)5	15	10226310	10226324
chr10_1514	p2	(GT)13	26	10227503	10227528
chr10_1515	p2	(CA)6	12	10260570	10260581
chr10_1516	p4	(TCTA)5	20	10268254	10268273
chr10_1517	p2	(AC)6	12	10270795	10270806
chr10_1518	p2	(AC)9	18	10281256	10281273
chr10_1519	p2	(AC)6	12	10282300	10282311
chr10_1520	p4	(TATG)7	28	10286281	10286308
chr10_1521	p2	(AC)9	18	10288874	10288891
chr10_1522	p2	(CA)7	14	10290508	10290521
chr10_1523	p2	(TC)9	18	10329267	10329284
chr10_1524	p3	(CTT)6	18	10332291	10332308
chr10_1525	p2	(AC)7	14	10333639	10333652
chr10_1526	p2	(AC)11	22	10339338	10339359
chr10_1527	p4	(TCAA)6	24	10348417	10348440
chr10_1528	p2	(TG)16	32	10349045	10349076
chr10_1529	p2	(AC)16	32	10349833	10349864

chr10_1530	p4	(TAGG)5	20	10350458	10350477
chr10_1531	p2	(GA)6	12	10351789	10351800
chr10_1532	p2	(AC)9	18	10361605	10361622
chr10_1533	p2	(CG)6	12	10362370	10362381
chr10_1534	p2	(TG)7	14	10366792	10366805
chr10_1535	p3	(ATA)5	15	10375733	10375747
chr10_1536	p2	(AC)11	22	10392129	10392150
chr10_1537	p2	(TG)6	12	10397126	10397137
chr10_1538	c	(AC)14a(AC)9	47	10408437	10408483
chr10_1539	p2	(GT)8	16	10428511	10428526
chr10_1540	p2	(AC)7	14	10436324	10436337
chr10_1541	p2	(AC)10	20	10439223	10439242
chr10_1542	p2	(CT)6	12	10444531	10444542
chr10_1543	c	(TC)6(AC)6g(CA)6	37	10447125	10447161
chr10_1544	p2	(AC)8	16	10450284	10450299
chr10_1545	p2	(CT)6	12	10453047	10453058
chr10_1546	p2	(CT)6	12	10457369	10457380
chr10_1547	p3	(GGA)5	15	10459647	10459661
chr10_1548	p2	(AC)6	12	10467998	10468009
chr10_1549	p4	(TAAA)6	24	10472432	10472455
chr10_1550	p4	(ATCT)16	64	10482780	10482843
chr10_1551	p2	(AG)6	12	10488874	10488885
chr10_1552	p3	(CCT)5	15	10491874	10491888
chr10_1553	p2	(TG)7	14	10498299	10498312
chr10_1554	p4	(AAAT)5	20	10499713	10499732
chr10_1555	p3	(TGA)7	21	10501529	10501549
chr10_1556	p2	(CA)15	30	10503974	10504003
chr10_1557	p2	(CA)10	20	10507770	10507789
chr10_1558	p5	(AAGAG)13	65	10508864	10508928
chr10_1559	p2	(TG)6	12	10509823	10509834
chr10_1560	p2	(TG)9	18	10511900	10511917
chr10_1561	p2	(GT)12	24	10512975	10512998
chr10_1562	p2	(TG)6	12	10516055	10516066
chr10_1563	p2	(TG)6	12	10517010	10517021
chr10_1564	p3	(ATA)5	15	10518145	10518159
chr10_1565	p2	(GT)16	32	10519374	10519405
chr10_1566	p2	(TA)6	12	10520916	10520927
chr10_1567	p3	(TCG)8	24	10522604	10522627
chr10_1568	c	(AC)22g(CA)16	77	10524381	10524457
chr10_1569	p4	(TCTG)5	20	10524979	10524998
chr10_1570	p2	(AC)10	20	10534343	10534362
chr10_1571	p2	(GT)7	14	10536471	10536484
chr10_1572	p2	(AC)10	20	10560028	10560047
chr10_1573	p2	(GT)8	16	10563742	10563757
chr10_1574	p2	(AC)13	26	10567089	10567114
chr10_1575	p2	(CA)16	32	10574941	10574972
chr10_1576	p2	(TC)7	14	10582636	10582649
chr10_1577	p3	(ATT)5	15	10583597	10583611
chr10_1578	p2	(AG)6	12	10584072	10584083
chr10_1579	p2	(TG)11	22	10595381	10595402
chr10_1580	p3	(TTA)5	15	10597390	10597404
chr10_1581	p3	(GAT)5	15	10606216	10606230

chr10_1582	p2	(GT)26	52	10606455	10606506
chr10_1583	p3	(TTC)6	18	10618588	10618605
chr10_1584	p2	(GT)15	30	10620892	10620921
chr10_1585	p4	(GAAT)6	24	10628148	10628171
chr10_1586	p2	(CA)15	30	10629367	10629396
chr10_1587	p2	(CA)13	26	10629617	10629642
chr10_1588	p2	(TG)9	18	10631280	10631297
chr10_1589	c	(CA)7t(TA)9cacacacacatatagacacacg(CA)10	75	10639557	10639631
chr10_1590	p2	(TG)17	34	10646136	10646169
chr10_1591	p2	(CA)11	22	10646343	10646364
chr10_1592	p2	(AC)7	14	10646531	10646544
chr10_1593	p2	(AC)23	46	10655882	10655927
chr10_1594	p2	(CT)9	18	10657276	10657293
chr10_1595	c	(AC)9at(AC)8	36	10659650	10659685
chr10_1596	p2	(TC)7	14	10664424	10664437
chr10_1597	p2	(CA)7	14	10665067	10665080
chr10_1598	p2	(CA)15	30	10671713	10671742
chr10_1599	p2	(CA)10	20	10672081	10672100
chr10_1600	p2	(CA)20	40	10672415	10672454
chr10_1601	p2	(TG)25	50	10673138	10673187
chr10_1602	p2	(AG)6	12	10674329	10674340
chr10_1603	p3	(CCA)5	15	10674658	10674672
chr10_1604	p2	(AC)7	14	10675029	10675042
chr10_1605	p2	(CA)20	40	10678239	10678278
chr10_1606	p2	(AG)8	16	10681515	10681530
chr10_1607	p2	(TG)6	12	10681703	10681714
chr10_1608	p3	(CTA)5	15	10683948	10683962
chr10_1609	p2	(CA)17	34	10687056	10687089
chr10_1610	p2	(AC)14	28	10691864	10691891
chr10_1611	p2	(TA)6	12	10692494	10692505
chr10_1612	p2	(GT)12	24	10692980	10693003
chr10_1613	p2	(GT)10	20	10695192	10695211
chr10_1614	p2	(AC)6	12	10701234	10701245
chr10_1615	p2	(AC)10	20	10706103	10706122
chr10_1616	p2	(TG)7	14	10712464	10712477
chr10_1617	p2	(GA)6	12	10715815	10715826
chr10_1618	p2	(GT)9	18	10716945	10716962
chr10_1619	p2	(GT)6	12	10718464	10718475
chr10_1620	p2	(AG)8	16	10718602	10718617
chr10_1621	p2	(CA)6	12	10719162	10719173
chr10_1622	p2	(GT)7	14	10721398	10721411
chr10_1623	p2	(GA)6	12	10721799	10721810
chr10_1624	p2	(AG)6	12	10721983	10721994
chr10_1625	c	[illegible]tg(AC)7gtgtgcgcccacaccaaacacacaagctacacactgaaaca[illegible]	211	10722248	10722458
chr10_1626	p2	(CA)9	18	10722944	10722961
chr10_1627	p2	(TC)8	16	10727896	10727911
chr10_1628	p2	(TG)15	30	10753213	10753242
chr10_1629	p2	(AG)8	16	10755087	10755102
chr10_1630	p2	(AC)25	50	10765616	10765665
chr10_1631	p2	(GT)7	14	10772437	10772450
chr10_1632	p2	(TG)12	24	10773449	10773472
chr10_1633	p2	(TG)6	12	10773989	10774000

chr10_1634	p2	(CA)10	20	10779792	10779811
chr10_1635	p2	(CA)7	14	10785721	10785734
chr10_1636	p2	(TC)7	14	10794217	10794230
chr10_1637	p2	(TG)6	12	10794339	10794350
chr10_1638	p2	(TG)30	60	10801895	10801954
chr10_1639	p2	(AC)18	36	10802886	10802921
chr10_1640	c	(AC)10gcccatctaaccagctcagagaaaaagaa(AC)10	69	10806573	10806641
chr10_1641	p2	(TG)6	12	10809519	10809530
chr10_1642	p2	(GT)23	46	10817460	10817505
chr10_1643	p3	(TGT)6	18	10828862	10828879
chr10_1644	p2	(TA)6	12	10846375	10846386
chr10_1645	p5	(CTCTC)12	60	10866923	10866982
chr10_1646	p3	(GCT)8	24	10870821	10870844
chr10_1647	p2	(TG)23	46	10881024	10881069
chr10_1648	p2	(AG)8	16	10891939	10891954
chr10_1649	p2	(TC)6	12	10907919	10907930
chr10_1650	p2	(AG)8	16	10908630	10908645
chr10_1651	p2	(AG)7	14	10909156	10909169
chr10_1652	p2	(AC)16	32	10940976	10941007
chr10_1653	p2	(CT)7	14	10943988	10944001
chr10_1654	p2	(TG)6	12	10948998	10949009
chr10_1655	p2	(CA)10	20	10950175	10950194
chr10_1656	p2	(TC)7	14	10953817	10953830
chr10_1657	p2	(GT)11	22	10954054	10954075
chr10_1658	p5	(CTGTA)5	25	10961628	10961652
chr10_1659	p2	(GT)6	12	10966364	10966375
chr10_1660	p2	(AG)8	16	10966612	10966627
chr10_1661	c	gtaatggtgggggctctaatgatttttccaggacaagctgaagtgggtgtac	89	10977711	10977799
chr10_1662	p2	(TG)15	30	10998455	10998484
chr10_1663	p2	(CT)15	30	11018341	11018370
chr10_1664	p3	(GTG)6	18	11021748	11021765
chr10_1665	p2	(TG)6	12	11030596	11030607
chr10_1666	p3	(TGA)5	15	11033633	11033647
chr10_1667	p2	(TA)9	18	11060891	11060908
chr10_1668	p3	(AGA)15	45	11066519	11066563
chr10_1669	p3	(AGA)8	24	11067606	11067629
chr10_1670	p4	(TTTA)5	20	11075966	11075985
chr10_1671	p2	(AC)10	20	11094530	11094549
chr10_1672	p3	(ATT)9	27	11098558	11098584
chr10_1673	p2	(CT)6	12	11130788	11130799
chr10_1674	p2	(AC)7	14	11139756	11139769
chr10_1675	p2	(GT)6	12	11147235	11147246
chr10_1676	p2	(AG)6	12	11149631	11149642
chr10_1677	p2	(AC)7	14	11167004	11167017
chr10_1678	p2	(TG)28	56	11178842	11178897
chr10_1679	p2	(AG)6	12	11194289	11194300
chr10_1680	p2	(GT)10	20	11207278	11207297
chr10_1681	p2	(TC)6	12	11207938	11207949
chr10_1682	p2	(TC)9	18	11233645	11233662
chr10_1683	p2	(TG)13	26	11254258	11254283
chr10_1684	p4	(ATGG)5	20	11268455	11268474
chr10_1685	p3	(GAG)10	30	11270076	11270105

chr10_1686	c	(AC)23c(CA)9	65	11273367	11273431
chr10_1687	p4	(AACA)5	20	11290019	11290038
chr10_1688	p5	(AAAAG)6	30	11298438	11298467
chr10_1689	p2	(TG)6	12	11309668	11309679
chr10_1690	p2	(AC)10	20	11317699	11317718
chr10_1691	p2	(AC)7	14	11324832	11324845
chr10_1692	c	(AC)11(AT)7	36	11332742	11332777
chr10_1693	c	(AT)9a(GT)12	43	11333041	11333083
chr10_1694	p2	(AC)15	30	11343179	11343208
chr10_1695	p2	(TC)6	12	11351195	11351206
chr10_1696	p3	(GCT)8	24	11366766	11366789
chr10_1697	p2	(AG)7	14	11372384	11372397
chr10_1698	p2	(AG)12	24	11374140	11374163
chr10_1699	p2	(GT)8	16	11387076	11387091
chr10_1700	p2	(AC)10	20	11400566	11400585
chr10_1701	p2	(AC)7	14	11404750	11404763
chr10_1702	p2	(CA)13	26	11405658	11405683
chr10_1703	p3	(TGA)5	15	11406559	11406573
chr10_1704	p2	(AC)22	44	11410028	11410071
chr10_1705	p2	(TA)6	12	11414264	11414275
chr10_1706	p2	(CA)6	12	11418938	11418949
chr10_1707	p2	(TG)6	12	11419348	11419359
chr10_1708	p3	(TCA)6	18	11421431	11421448
chr10_1709	p2	(CA)13	26	11423368	11423393
chr10_1710	p2	(TG)6	12	11432087	11432098
chr10_1711	p3	(TAT)5	15	11432508	11432522
chr10_1712	p2	(TC)12	24	11434264	11434287
chr10_1713	p2	(AC)12	24	11436581	11436604
chr10_1714	p3	(GAG)6	18	11443174	11443191
chr10_1715	p2	(AC)8	16	11444089	11444104
chr10_1716	p2	(AC)9	18	11451479	11451496
chr10_1717	p2	(TG)13	26	11463141	11463166
chr10_1718	p2	(CA)6	12	11464037	11464048
chr10_1719	p2	(AC)11	22	11466446	11466467
chr10_1720	p2	(TG)23	46	11483928	11483973
chr10_1721	p2	(TG)8	16	11484155	11484170
chr10_1722	c	(TG)19ta(TG)7(AG)7	68	11489802	11489869
chr10_1723	p2	(TC)10	20	11491980	11491999
chr10_1724	p2	(AC)6	12	11497521	11497532
chr10_1725	p2	(GT)54	108	11502603	11502710
chr10_1726	p2	(AT)6	12	11506432	11506443
chr10_1727	p2	(CA)8	16	11541091	11541106
chr10_1728	p2	(CT)7	14	11542568	11542581
chr10_1729	p2	(AC)10	20	11553057	11553076
chr10_1730	p2	(CA)7	14	11556657	11556670
chr10_1731	p3	(TAT)6	18	11558051	11558068
chr10_1732	p2	(TC)6	12	11562881	11562892
chr10_1733	p2	(TG)7	14	11581382	11581395
chr10_1734	p2	(AG)6	12	11587360	11587371
chr10_1735	c	(TC)11(AC)12	46	11596536	11596581
chr10_1736	p2	(AC)8	16	11598490	11598505
chr10_1737	p2	(CA)14	28	11604005	11604032

chr10_1738	p2	(CA)7	14	11604817	11604830
chr10_1739	p2	(AC)9	18	11608097	11608114
chr10_1740	p2	(AT)6	12	11635378	11635389
chr10_1741	p2	(AC)6	12	11637903	11637914
chr10_1742	p2	(AG)7	14	11641940	11641953
chr10_1743	p2	(AG)10	20	11654971	11654990
chr10_1744	p3	(TTG)6	18	11665565	11665582
chr10_1745	p2	(AC)8	16	11666358	11666373
chr10_1746	p2	(AC)11	22	11670376	11670397
chr10_1747	p2	(AC)7	14	11674478	11674491
chr10_1748	p2	(AC)6	12	11674603	11674614
chr10_1749	p2	(GA)7	14	11686361	11686374
chr10_1750	p3	(ATA)5	15	11690272	11690286
chr10_1751	p2	(CA)22	44	11695607	11695650
chr10_1752	p2	(AT)6	12	11702261	11702272
chr10_1753	p3	(AGA)7	21	11705363	11705383
chr10_1754	p2	(AC)10	20	11744559	11744578
chr10_1755	p2	(TG)11	22	11755263	11755284
chr10_1756	p3	(ATT)8	24	11759434	11759457
chr10_1757	p4	(TACA)8	32	11772201	11772232
chr10_1758	p3	(ACA)5	15	11777907	11777921
chr10_1759	p4	(ATTT)5	20	11778118	11778137
chr10_1760	p3	(CCA)5	15	11779589	11779603
chr10_1761	p3	(TAA)10	30	11782473	11782502
chr10_1762	p3	(CAC)5	15	11787882	11787896
chr10_1763	p2	(TG)8	16	11792803	11792818
chr10_1764	p2	(AC)17	34	11796308	11796341
chr10_1765	p2	(AG)11	22	11796855	11796876
chr10_1766	p2	(GT)7	14	11801960	11801973
chr10_1767	p2	(TG)58	116	11808465	11808580
chr10_1768	p2	(AT)9	18	11814678	11814695
chr10_1769	p3	(GGA)5	15	11818567	11818581
chr10_1770	p2	(AC)29	58	11819561	11819618
chr10_1771	p2	(AC)38	76	11820517	11820592
chr10_1772	p2	(AT)6	12	11824094	11824105
chr10_1773	p3	(CTT)11	33	11836801	11836833
chr10_1774	p2	(AG)7	14	11855646	11855659
chr10_1775	p2	(GA)7	14	11857807	11857820
chr10_1776	p2	(AT)9	18	11873587	11873604
chr10_1777	p3	(TTA)5	15	11875172	11875186
chr10_1778	p2	(CT)8	16	11876070	11876085
chr10_1779	p2	(AG)12	24	11877178	11877201
chr10_1780	p2	(GT)6	12	11888458	11888469
chr10_1781	p2	(TG)6	12	11888996	11889007
chr10_1782	p2	(CT)7	14	11894470	11894483
chr10_1783	p2	(AT)7	14	11898567	11898580
chr10_1784	p3	(TAT)44	132	11938679	11938810
chr10_1785	p4	(ATGT)5	20	11943841	11943860
chr10_1786	p3	(ATC)19	57	11971945	11972001
chr10_1787	p2	(GT)7	14	11974284	11974297
chr10_1788	p2	(TC)6	12	11991265	11991276
chr10_1789	p2	(CA)7	14	11992709	11992722

chr10_1790	p5	(AAATC)5	25	12013466	12013490
chr10_1791	p3	(AAC)8	24	12019858	12019881
chr10_1792	p3	(TTG)5	15	12022532	12022546
chr10_1793	c	(TG)21a(GAGT)9	79	12026139	12026217
chr10_1794	c	[illegible]T)5ctgctgaagtgtccttgagcaagacgcagtctcaatacacccccac[illegible]	138	12046659	12046796
chr10_1795	c	(GA)7a(AG)7	29	12048096	12048124
chr10_1796	p2	(CA)9	18	12063591	12063608
chr10_1797	p3	(CTT)5	15	12064608	12064622
chr10_1798	p3	(AAT)9	27	12069312	12069338
chr10_1799	p2	(TA)6	12	12079882	12079893
chr10_1800	p2	(TG)6	12	12080835	12080846
chr10_1801	p2	(CA)16	32	12091773	12091804
chr10_1802	p3	(GCT)6	18	12107273	12107290
chr10_1803	p2	(AC)9	18	12120075	12120092
chr10_1804	p4	(AATT)6	24	12129595	12129618
chr10_1805	p2	(GT)9	18	12142885	12142902
chr10_1806	p2	(CA)8	16	12167971	12167986
chr10_1807	c	(CA)6cgcgcacacacactcacaca(CT)10	52	12169578	12169629
chr10_1808	p3	(ATC)5	15	12172223	12172237
chr10_1809	p3	(ATG)5	15	12172513	12172527
chr10_1810	p2	(GT)9	18	12184369	12184386
chr10_1811	c	(AC)25a(AC)9	69	12186522	12186590
chr10_1812	p2	(TC)8	16	12187515	12187530
chr10_1813	p2	(TG)19	38	12189203	12189240
chr10_1814	p2	(CA)6	12	12193633	12193644
chr10_1815	p2	(AC)9	18	12206542	12206559
chr10_1816	p2	(GA)10	20	12218439	12218458
chr10_1817	p2	(AC)10	20	12218758	12218777
chr10_1818	p2	(CA)9	18	12232897	12232914
chr10_1819	p2	(AC)8	16	12235501	12235516
chr10_1820	p3	(AGG)8	24	12252679	12252702
chr10_1821	p2	(AT)6	12	12280465	12280476
chr10_1822	p2	(AG)6	12	12280987	12280998
chr10_1823	p2	(TG)21	42	12285922	12285963
chr10_1824	p2	(CT)6	12	12288073	12288084
chr10_1825	p2	(TG)7	14	12291201	12291214
chr10_1826	p2	(CA)13	26	12291360	12291385
chr10_1827	p2	(GT)6	12	12299334	12299345
chr10_1828	p4	(GACA)6	24	12300025	12300048
chr10_1829	p2	(TG)6	12	12302892	12302903
chr10_1830	p2	(AC)12	24	12305230	12305253
chr10_1831	p2	(TC)6	12	12306442	12306453
chr10_1832	p2	(TC)9	18	12306797	12306814
chr10_1833	c*	(CCA)6(CA)18*	52	12307042	12307093
chr10_1834	p2	(TG)10	20	12307622	12307641
chr10_1835	p2	(CA)8	16	12307800	12307815
chr10_1836	p2	(CA)8	16	12314079	12314094
chr10_1837	p2	(AC)8	16	12314938	12314953
chr10_1838	c	(TG)22(AG)7	58	12326197	12326254
chr10_1839	p2	(CA)6	12	12329961	12329972
chr10_1840	p2	(CA)28	56	12333576	12333631
chr10_1841	p2	(CA)7	14	12337849	12337862

chr10_1842	p2	(GT)27	54	12340401	12340454
chr10_1843	p2	(GT)8	16	12342152	12342167
chr10_1844	p2	(CA)18	36	12345602	12345637
chr10_1845	p2	(GA)7	14	12347836	12347849
chr10_1846	p3	(CCT)5	15	12352348	12352362
chr10_1847	c	(GA)19gtgca(TG)9	61	12354969	12355029
chr10_1848	c	(GT)12gagtgt(GA)9	48	12357381	12357428
chr10_1849	p2	(AC)10	20	12363254	12363273
chr10_1850	p3	(TCC)5	15	12364000	12364014
chr10_1851	p2	(GT)7	14	12364892	12364905
chr10_1852	p3	(AGC)6	18	12383604	12383621
chr10_1853	p3	(GCA)5	15	12385926	12385940
chr10_1854	c	(AC)12(AG)16	56	12389038	12389093
chr10_1855	p6	(TCAGAA)5	30	12390815	12390844
chr10_1856	p3	(ATA)5	15	12393259	12393273
chr10_1857	p3	(CAA)5	15	12393767	12393781
chr10_1858	p4	(TAGC)5	20	12402950	12402969
chr10_1859	p2	(TG)22	44	12426834	12426877
chr10_1860	p2	(GT)7	14	12431119	12431132
chr10_1861	p3	(AGC)5	15	12434007	12434021
chr10_1862	p2	(AC)7	14	12442920	12442933
chr10_1863	p2	(GT)6	12	12464695	12464706
chr10_1864	p2	(CA)11	22	12468270	12468291
chr10_1865	p3	(TTC)5	15	12470526	12470540
chr10_1866	p2	(AC)6	12	12477509	12477520
chr10_1867	p2	(CA)7	14	12482637	12482650
chr10_1868	p2	(CA)10	20	12484912	12484931
chr10_1869	p2	(TC)6	12	12485851	12485862
chr10_1870	p2	(AC)27	54	12491791	12491844
chr10_1871	p4	(CAGT)5	20	12493399	12493418
chr10_1872	p2	(GT)7	14	12498097	12498110
chr10_1873	p2	(AC)6	12	12504938	12504949
chr10_1874	p2	(AC)8	16	12515226	12515241
chr10_1875	c	(CA)30c(CA)6	73	12517103	12517175
chr10_1876	p2	(GT)8	16	12519527	12519542
chr10_1877	p2	(TG)7	14	12519975	12519988
chr10_1878	p4	(AATC)5	20	12525402	12525421
chr10_1879	p2	(AC)12	24	12532169	12532192
chr10_1880	p3	(TGA)6	18	12534068	12534085
chr10_1881	p2	(GT)8	16	12540311	12540326
chr10_1882	p2	(CA)7	14	12550148	12550161
chr10_1883	p2	(TC)6	12	12553659	12553670
chr10_1884	p2	(CA)22	44	12564518	12564561
chr10_1885	c	(TG)18tatgaatatgtatgctcacatac(TG)16	91	12566608	12566698
chr10_1886	p2	(AT)7	14	12579956	12579969
chr10_1887	c	(TG)15(TA)7	44	12580369	12580412
chr10_1888	p2	(TG)8	16	12582066	12582081
chr10_1889	p2	(TA)7	14	12585654	12585667
chr10_1890	p2	(CT)6	12	12589326	12589337
chr10_1891	p4	(CACT)6	24	12608855	12608878
chr10_1892	c	aagaaagagagagacagcgagattaagtgagagggggagggagaatgg	85	12609037	12609121
chr10_1893	p3	(ATT)5	15	12610387	12610401

chr10_1894	p2	(TA)8	16	12619593	12619608
chr10_1895	p2	(CA)12	24	12624481	12624504
chr10_1896	p2	(TA)7	14	12626952	12626965
chr10_1897	p3	(TCA)5	15	12631967	12631981
chr10_1898	p2	(AT)9	18	12638788	12638805
chr10_1899	p2	(GT)19	38	12669383	12669420
chr10_1900	p4	(TTTA)5	20	12676225	12676244
chr10_1901	p2	(GT)6	12	12676370	12676381
chr10_1902	p3	(AGA)5	15	12677830	12677844
chr10_1903	p2	(TC)7	14	12687426	12687439
chr10_1904	p2	(TG)6	12	12688620	12688631
chr10_1905	p2	(TA)9	18	12689908	12689925
chr10_1906	p3	(TTC)5	15	12694281	12694295
chr10_1907	c	(TAC)5(TAA)6	33	12696250	12696282
chr10_1908	p2	(CA)6	12	12711128	12711139
chr10_1909	c	(TC)6tttcactcaggga(GT)10	45	12730754	12730798
chr10_1910	p2	(AG)11	22	12732648	12732669
chr10_1911	p3	(CTC)5	15	12736234	12736248
chr10_1912	p2	(TG)18	36	12738010	12738045
chr10_1913	p2	(CA)12	24	12742895	12742918
chr10_1914	p2	(AC)18	36	12763602	12763637
chr10_1915	p2	(AG)6	12	12764231	12764242
chr10_1916	p2	(AG)6	12	12765394	12765405
chr10_1917	c	gagcattgtgttgctgctgaggaagagcccatgtctgcacaaagaggtag	120	12765612	12765731
chr10_1918	p2	(CA)15	30	12772496	12772525
chr10_1919	p2	(TG)15	30	12773174	12773203
chr10_1920	p2	(TG)15	30	12774361	12774390
chr10_1921	c	(CA)6cg(CA)15	44	12774909	12774952
chr10_1922	p2	(TG)10	20	12775951	12775970
chr10_1923	p3	(AAG)5	15	12796205	12796219
chr10_1924	p2	(TG)17	34	12800815	12800848
chr10_1925	p2	(GT)23	46	12801871	12801916
chr10_1926	p2	(TG)6	12	12804408	12804419
chr10_1927	p2	(AC)17	34	12805667	12805700
chr10_1928	p2	(AC)11	22	12807469	12807490
chr10_1929	p3	(ATG)6	18	12808326	12808343
chr10_1930	p2	(AC)16	32	12810550	12810581
chr10_1931	p2	(TG)18	36	12814034	12814069
chr10_1932	p2	(TG)6	12	12821374	12821385
chr10_1933	p2	(TG)6	12	12825956	12825967
chr10_1934	p2	(AC)8	16	12826107	12826122
chr10_1935	p2	(TG)8	16	12840983	12840998
chr10_1936	p2	(CA)12	24	12841465	12841488
chr10_1937	c	(AG)7aaag(AGC)5	33	12844687	12844719
chr10_1938	c	(TC)6cct(CA)13	41	12847300	12847340
chr10_1939	c	(GA)7ccttgagtcagacagagagagagggagcgagggct(AG)12	73	12847444	12847516
chr10_1940	p2	(GA)17	34	12848699	12848732
chr10_1941	p2	(CA)7	14	12854544	12854557
chr10_1942	p2	(GT)24	48	12854719	12854766
chr10_1943	p2	(TG)9	18	12854916	12854933
chr10_1944	p2	(TC)6	12	12855554	12855565
chr10_1945	p2	(TG)6	12	12858441	12858452

chr10_1946	p2	(AC)11	22	12859680	12859701
chr10_1947	p2	(GT)7	14	12861641	12861654
chr10_1948	p2	(GT)8	16	12862547	12862562
chr10_1949	p2	(TC)7	14	12863024	12863037
chr10_1950	p2	(AC)17	34	12863195	12863228
chr10_1951	c	[illegible])7tcagtgtgtatgtgtgattgtgttagcatttgtgactgttctgtgtgtgta(T	81	12864928	12865008
chr10_1952	p3	(ATG)7	21	12869479	12869499
chr10_1953	p2	(TG)14	28	12869734	12869761
chr10_1954	p2	(TG)14	28	12870201	12870228
chr10_1955	p3	(GTG)5	15	12877244	12877258
chr10_1956	p3	(AAC)5	15	12878798	12878812
chr10_1957	p2	(TG)7	14	12881646	12881659
chr10_1958	p2	(GT)9	18	12884305	12884322
chr10_1959	p2	(GA)6	12	12885133	12885144
chr10_1960	p2	(TG)18	36	12885747	12885782
chr10_1961	c	ttgattgacgtcccatcataaattatgcgtgcgtgtctgtctgcctgcctgcat	131	12886048	12886178
chr10_1962	p2	(TG)18	36	12889875	12889910
chr10_1963	p2	(GT)9	18	12896902	12896919
chr10_1964	p2	(TC)12	24	12899011	12899034
chr10_1965	p2	(CA)7	14	12901761	12901774
chr10_1966	p2	(AC)8	16	12905798	12905813
chr10_1967	p2	(AC)10	20	12908843	12908862
chr10_1968	p2	(AC)8	16	12913054	12913069
chr10_1969	p2	(TG)12	24	12913707	12913730
chr10_1970	p2	(AC)14	28	12919465	12919492
chr10_1971	p2	(GA)8	16	12920068	12920083
chr10_1972	p2	(GT)7	14	12923785	12923798
chr10_1973	p2	(GT)22	44	12924233	12924276
chr10_1974	p2	(AC)11	22	12927148	12927169
chr10_1975	p3	(CTG)5	15	12930867	12930881
chr10_1976	c	(AC)8ccacacacacaccttacat(AC)6	47	12933475	12933521
chr10_1977	p2	(TG)7	14	12933921	12933934
chr10_1978	p2	(TG)14	28	12934234	12934261
chr10_1979	p3	(AAT)5	15	12939287	12939301
chr10_1980	p2	(AT)14	28	12944652	12944679
chr10_1981	p2	(CA)6	12	12945636	12945647
chr10_1982	p2	(GA)6	12	12949404	12949415
chr10_1983	p2	(CA)24	48	12951914	12951961
chr10_1984	p2	(TA)7	14	12960397	12960410
chr10_1985	p2	(TG)22	44	12974515	12974558
chr10_1986	p2	(TG)9	18	12980940	12980957
chr10_1987	p2	(AC)6	12	12981416	12981427
chr10_1988	p2	(TG)34	68	12984023	12984090
chr10_1989	p2	(TG)6	12	12988444	12988455
chr10_1990	p2	(TG)13	26	12992184	12992209
chr10_1991	p2	(CA)22	44	12992401	12992444
chr10_1992	p3	(AAT)10	30	12994138	12994167
chr10_1993	p3	(ATC)7	21	12995312	12995332
chr10_1994	p2	(TG)13	26	13001553	13001578
chr10_1995	p3	(TTA)6	18	13009001	13009018
chr10_1996	p2	(CT)6	12	13012065	13012076
chr10_1997	p2	(TG)9	18	13015233	13015250

chr10_1998	p2	(GT)17	34	13015494	13015527
chr10_1999	p2	(AC)8	16	13015637	13015652
chr10_2000	p2	(TG)12	24	13017917	13017940
chr10_2001	p2	(AC)6	12	13021070	13021081
chr10_2002	p2	(TG)18	36	13025558	13025593
chr10_2003	p2	(TG)13	26	13027256	13027281
chr10_2004	p2	(TG)8	16	13037487	13037502
chr10_2005	c	(GT)6ttttatcagtagta(TG)7	40	13037821	13037860
chr10_2006	p2	(TG)7	14	13037994	13038007
chr10_2007	p2	(GT)11	22	13050068	13050089
chr10_2008	p2	(GT)22	44	13051858	13051901
chr10_2009	p2	(CA)8	16	13052036	13052051
chr10_2010	p2	(AC)13	26	13052804	13052829
chr10_2011	p2	(TG)13	26	13061839	13061864
chr10_2012	p6	(AATATG)5	30	13062942	13062971
chr10_2013	p2	(CA)9	18	13064029	13064046
chr10_2014	p2	(TG)9	18	13067267	13067284
chr10_2015	p2	(TG)8	16	13069950	13069965
chr10_2016	p2	(AC)8	16	13070572	13070587
chr10_2017	p2	(AC)19	38	13070946	13070983
chr10_2018	p4	(CATT)5	20	13073667	13073686
chr10_2019	p2	(CA)9	18	13073841	13073858
chr10_2020	p2	(CA)24	48	13079879	13079926
chr10_2021	p2	(TG)12	24	13083031	13083054
chr10_2022	p2	(AC)12	24	13084581	13084604
chr10_2023	p2	(CA)6	12	13085132	13085143
chr10_2024	p2	(TA)7	14	13114117	13114130
chr10_2025	p3	(CAG)5	15	13114334	13114348
chr10_2026	p2	(AC)10	20	13117518	13117537
chr10_2027	p2	(CA)12	24	13118794	13118817
chr10_2028	p2	(AC)10	20	13136333	13136352
chr10_2029	p2	(AG)9	18	13137550	13137567
chr10_2030	p2	(AC)8	16	13139047	13139062
chr10_2031	p2	(TC)10	20	13139407	13139426
chr10_2032	p2	(AC)7	14	13145759	13145772
chr10_2033	c	(CA)10t(AC)13	47	13148974	13149020
chr10_2034	c	(AC)12atat(ATAC)7ataa(ATAC)5	80	13154303	13154382
chr10_2035	p2	(AT)6	12	13163955	13163966
chr10_2036	p2	(TG)14	28	13167917	13167944
chr10_2037	p2	(GT)6	12	13168375	13168386
chr10_2038	p2	(GT)11	22	13169333	13169354
chr10_2039	p2	(AC)17	34	13173404	13173437
chr10_2040	p2	(AC)6	12	13177281	13177292
chr10_2041	p2	(GT)7	14	13179000	13179013
chr10_2042	p2	(CA)6	12	13179927	13179938
chr10_2043	p2	(TG)11	22	13182118	13182139
chr10_2044	p2	(TG)6	12	13189596	13189607
chr10_2045	p2	(TG)19	38	13189737	13189774
chr10_2046	p2	(CA)12	24	13190355	13190378
chr10_2047	p2	(AC)17	34	13191980	13192013
chr10_2048	p2	(GT)18	36	13195272	13195307
chr10_2049	p2	(TG)6	12	13196174	13196185

chr10_2050	p2	(GT)10	20	13196297	13196316
chr10_2051	p2	(CT)6	12	13200201	13200212
chr10_2052	c	(TC)6gctttcaagccagtagtcaagaggcatatatatgta(TG)11	70	13205043	13205112
chr10_2053	p3	(GGA)5	15	13207033	13207047
chr10_2054	p2	(TA)9	18	13208819	13208836
chr10_2055	p2	(GT)6	12	13208969	13208980
chr10_2056	p2	(AC)6	12	13209299	13209310
chr10_2057	p2	(CA)11	22	13210798	13210819
chr10_2058	p2	(TG)9	18	13210979	13210996
chr10_2059	c	5tctaagctaaaataagaagtgtgggtattgcccagtaacccgctgcaaat(	93	13212334	13212426
chr10_2060	p2	(TG)7	14	13215786	13215799
chr10_2061	p2	(GT)29	58	13224517	13224574
chr10_2062	p2	(AC)11	22	13230821	13230842
chr10_2063	p2	(AC)6	12	13235553	13235564
chr10_2064	p2	(GT)7	14	13256437	13256450
chr10_2065	p3	(CTC)5	15	13267201	13267215
chr10_2066	p3	(CAG)6	18	13268336	13268353
chr10_2067	p2	(GT)6	12	13269477	13269488
chr10_2068	p4	(GAAG)5	20	13271356	13271375
chr10_2069	p2	(AC)13	26	13273684	13273709
chr10_2070	p2	(CA)16	32	13274983	13275014
chr10_2071	p2	(CT)10	20	13278534	13278553
chr10_2072	p2	(GT)24	48	13285229	13285276
chr10_2073	p2	(AC)8	16	13287035	13287050
chr10_2074	c	(TG)8ta(TG)11	40	13288648	13288687
chr10_2075	p2	(TG)6	12	13297192	13297203
chr10_2076	p2	(AG)16	32	13304000	13304031
chr10_2077	p2	(TG)7	14	13310204	13310217
chr10_2078	c	cttcg(AC)6agcttggccggaacgttatccacacagcgtgaccctgtgc	126	13320908	13321033
chr10_2079	p2	(CA)6	12	13324914	13324925
chr10_2080	p2	(CA)22	44	13332094	13332137
chr10_2081	p2	(CA)8	16	13339498	13339513
chr10_2082	p2	(TG)22	44	13339755	13339798
chr10_2083	p2	(GT)8	16	13377636	13377651
chr10_2084	p3	(AAT)5	15	13386059	13386073
chr10_2085	p3	(TCA)11	33	13390756	13390788
chr10_2086	p2	(CA)7	14	13394909	13394922
chr10_2087	p2	(AC)13	26	13398953	13398978
chr10_2088	p2	(TC)14	28	13400393	13400420
chr10_2089	p2	(GT)14	28	13400625	13400652
chr10_2090	p2	(AC)6	12	13404237	13404248
chr10_2091	p2	(TG)6	12	13404634	13404645
chr10_2092	p2	(TG)9	18	13406731	13406748
chr10_2093	p2	(AC)11	22	13420228	13420249
chr10_2094	p2	(CA)6	12	13427687	13427698
chr10_2095	p4	(TATT)5	20	13434135	13434154
chr10_2096	p2	(AC)13	26	13434566	13434591
chr10_2097	c	nnnnnnnnnnnnnnnnnnnnnnnnnnnnnnnccctctctcc(CT)3	164	13447104	13447267
chr10_2098	p2	(CA)7	14	13452933	13452946
chr10_2099	c	(GT)8(GC)6	28	13468363	13468390
chr10_2100	p2	(CA)17	34	13468661	13468694
chr10_2101	c	)12aagggctaggtaaacaacaacaagggctaggtaaacaacaacaa(C	101	13469119	13469219

chr10_2102	p2	(TA)7	14	13469816	13469829
chr10_2103	p2	(GT)6	12	13478021	13478032
chr10_2104	c	(AC)6at(AC)9	32	13481320	13481351
chr10_2105	p2	(TG)6	12	13487327	13487338
chr10_2106	p2	(AC)7	14	13488713	13488726
chr10_2107	c	(TC)6cct(CA)6	27	13494080	13494106
chr10_2108	p2	(CA)12	24	13497640	13497663
chr10_2109	p2	(AT)6	12	13502164	13502175
chr10_2110	p2	(GT)7	14	13518638	13518651
chr10_2111	p3	(TTG)5	15	13534271	13534285
chr10_2112	p2	(AG)6	12	13547536	13547547
chr10_2113	p2	(AC)7	14	13561853	13561866
chr10_2114	p2	(AC)15	30	13567294	13567323
chr10_2115	p2	(TG)7	14	13570518	13570531
chr10_2116	p2	(GT)15	30	13571977	13572006
chr10_2117	p2	(GT)18	36	13574682	13574717
chr10_2118	p2	(AC)11	22	13575505	13575526
chr10_2119	p2	(AC)7	14	13575631	13575644
chr10_2120	p2	(AC)14	28	13576776	13576803
chr10_2121	c	)6gcaagacacataaatacttacaaaactgagtgcatgtctg(TC)15(A	108	13578193	13578300
chr10_2122	p3	(AAC)6	18	13580057	13580074
chr10_2123	p3	(GTG)5	15	13580608	13580622
chr10_2124	c	)10gcagaccctgccagcacaaagctagatgtgtgcagcttgtgtgggaa	117	13583506	13583622
chr10_2125	c	tgctttccactctaatgttaagataaaagatgaaagtatggtgtctgtctggct	185	13585369	13585553
chr10_2126	p2	(TG)20	40	13586640	13586679
chr10_2127	p2	(AC)12	24	13587168	13587191
chr10_2128	p2	(AC)8	16	13587368	13587383
chr10_2129	p2	(TG)25	50	13589064	13589113
chr10_2130	p2	(TG)12	24	13589438	13589461
chr10_2131	p2	(TG)6	12	13590632	13590643
chr10_2132	p4	(TAGA)10	40	13604669	13604708
chr10_2133	c	atagggtgcaaaaaacagtgttcccagaatgtcagcacccacacatgtgca	128	13604986	13605113
chr10_2134	c	(CT)20(CA)7	54	13605264	13605317
chr10_2135	p2	(CT)6	12	13606831	13606842
chr10_2136	p2	(TG)29	58	13610398	13610455
chr10_2137	p2	(AC)7	14	13612429	13612442
chr10_2138	p2	(AC)8	16	13613389	13613404
chr10_2139	p2	(TG)31	62	13614702	13614763
chr10_2140	p2	(GT)30	60	13615200	13615259
chr10_2141	p2	(CA)19	38	13618342	13618379
chr10_2142	p2	(TG)6	12	13624612	13624623
chr10_2143	p2	(CA)6	12	13626377	13626388
chr10_2144	p4	(AAGA)12	48	13627679	13627726
chr10_2145	p5	(TTTGA)5	25	13627929	13627953
chr10_2146	p2	(CA)9	18	13630939	13630956
chr10_2147	p2	(AC)8	16	13639310	13639325
chr10_2148	p2	(TG)7	14	13641785	13641798
chr10_2149	c	(TG)35tt(TG)10	92	13644550	13644641
chr10_2150	p2	(AC)9	18	13647588	13647605
chr10_2151	p2	(CA)7	14	13650892	13650905
chr10_2152	c	)6atgctggactgtgtggatttaactgtgctgctgtgacacgtagtacac(C	84	13651265	13651348
chr10_2153	p2	(CA)10	20	13653370	13653389

chr10_2154	p2	(CA)11	22	13655095	13655116
chr10_2155	p2	(TG)6	12	13656116	13656127
chr10_2156	p2	(TG)6	12	13660108	13660119
chr10_2157	p2	(AC)9	18	13666087	13666104
chr10_2158	p2	(TG)18	36	13672502	13672537
chr10_2159	p2	(TG)20	40	13673900	13673939
chr10_2160	p2	(TG)6	12	13675257	13675268
chr10_2161	p2	(TC)7	14	13683160	13683173
chr10_2162	p3	(CAT)6	18	13687225	13687242
chr10_2163	p2	(AC)7	14	13694536	13694549
chr10_2164	p2	(TG)13	26	13704469	13704494
chr10_2165	p2	(TC)11	22	13707012	13707033
chr10_2166	p2	(AC)6	12	13707172	13707183
chr10_2167	c	gagaggaacatgagacaaaggctctctaattaatattcctgtcgaagatcac	85	13711185	13711269
chr10_2168	c	(AG)7g(GT)11	37	13713047	13713083
chr10_2169	p2	(GT)28	56	13716303	13716358
chr10_2170	p2	(AC)6	12	13717066	13717077
chr10_2171	p2	(GT)13	26	13729513	13729538
chr10_2172	p2	(AT)7	14	13735463	13735476
chr10_2173	p2	(AC)7	14	13742252	13742265
chr10_2174	c	gattcagcaagcgacaaatctccatggcaacacagcaatcacgacccaga	84	13745075	13745158
chr10_2175	p2	(CA)13	26	13750610	13750635
chr10_2176	p2	(GT)6	12	13753223	13753234
chr10_2177	p2	(TC)9	18	13753794	13753811
chr10_2178	p2	(CT)9	18	13754007	13754024
chr10_2179	p2	(TC)7	14	13767117	13767130
chr10_2180	c	(CT)6ccctctctctctt(TC)7	39	13768205	13768243
chr10_2181	p2	(CT)6	12	13768610	13768621
chr10_2182	c	acaagtccctgtcctcctccaatcatccatgcccctctgacctgcacgcacc	129	13769647	13769775
chr10_2183	p2	(GT)11	22	13774215	13774236
chr10_2184	p2	(AC)6	12	13774383	13774394
chr10_2185	p2	(AT)10	20	13775283	13775302
chr10_2186	c	gaatgacactccatgcacacagacacatatgtgcacttatacacccagacat	92	13776320	13776411
chr10_2187	p2	(GT)6	12	13778931	13778942
chr10_2188	p2	(AC)22	44	13782753	13782796
chr10_2189	c	igttgggctgctccactttgagcccgaccagcgaaggaggcatctgcccgg	111	13787183	13787293
chr10_2190	p2	(GT)7	14	13792014	13792027
chr10_2191	p2	(AC)6	12	13795724	13795735
chr10_2192	p2	(TC)6	12	13801876	13801887
chr10_2193	c	(TG)13(AG)8	42	13803951	13803992
chr10_2194	p4	(TGGA)6	24	13815335	13815358
chr10_2195	p2	(TA)8	16	13816764	13816779
chr10_2196	p2	(AT)6	12	13816981	13816992
chr10_2197	p2	(CA)9	18	13823181	13823198
chr10_2198	p2	(CA)10	20	13823563	13823582
chr10_2199	p3	(TAA)9	27	13825860	13825886
chr10_2200	p2	(AC)7	14	13826434	13826447
chr10_2201	p2	(TG)7	14	13839207	13839220
chr10_2202	p2	(CA)20	40	13850598	13850637
chr10_2203	c	caaaaattaaacctgcagtttactgtgctgcatctagcttggttgaagatgttt	142	13851388	13851529
chr10_2204	p3	(GGA)5	15	13856337	13856351
chr10_2205	c	(GT)7atgtgtgt(GA)6	34	13856521	13856554

chr10_2206	p2	(GT)6	12	13859421	13859432
chr10_2207	p2	(TG)11	22	13871263	13871284
chr10_2208	c	)6gaggagaggaagggaaggatggagggaggtacctaatgatggaaag	88	13874394	13874481
chr10_2209	p2	(CT)7	14	13874761	13874774
chr10_2210	p2	(CA)9	18	13877612	13877629
chr10_2211	c	(TG)11(AG)6	34	13877942	13877975
chr10_2212	c	(TG)6gacagtagtcc(GT)9	41	13879563	13879603
chr10_2213	p2	(TG)6	12	13890519	13890530
chr10_2214	p2	(GT)22	44	13890701	13890744
chr10_2215	p2	(GT)24	48	13892829	13892876
chr10_2216	p2	(AC)9	18	13892979	13892996
chr10_2217	p2	(CA)21	42	13893115	13893156
chr10_2218	p2	(TG)9	18	13893297	13893314
chr10_2219	p2	(AC)23	46	13898314	13898359
chr10_2220	p2	(TA)8	16	13898694	13898709
chr10_2221	p2	(TG)14	28	13900056	13900083
chr10_2222	p2	(TC)7	14	13900187	13900200
chr10_2223	p2	(CA)7	14	13906047	13906060
chr10_2224	p2	(TG)7	14	13908836	13908849
chr10_2225	p2	(AC)8	16	13910280	13910295
chr10_2226	p2	(CA)10	20	13910863	13910882
chr10_2227	p2	(TG)6	12	13911343	13911354
chr10_2228	p2	(AC)6	12	13911520	13911531
chr10_2229	p3	(AAT)10	30	13919232	13919261
chr10_2230	p4	(CCTA)5	20	13920028	13920047
chr10_2231	p3	(TAT)5	15	13932402	13932416
chr10_2232	p2	(TG)17	34	13934921	13934954
chr10_2233	c	atgaagctgtgtttatctagtttacacaaattgggctggaattgttcagtgttgg	83	13942169	13942251
chr10_2234	p2	(TC)9	18	13943513	13943530
chr10_2235	p3	(TGA)5	15	13944681	13944695
chr10_2236	p2	(TC)7	14	13945501	13945514
chr10_2237	p2	(AC)40	80	13953886	13953965
chr10_2238	c	(TC)13(AC)22	70	13955288	13955357
chr10_2239	p2	(TG)17	34	13955544	13955577
chr10_2240	c	(TG)12tttt(TG)15	58	13955706	13955763
chr10_2241	p2	(GA)6	12	13958059	13958070
chr10_2242	p2	(AC)28	56	13962619	13962674
chr10_2243	p2	(TG)6	12	13963885	13963896
chr10_2244	p2	(GT)42	84	13964036	13964119
chr10_2245	p2	(CA)8	16	13964285	13964300
chr10_2246	p2	(TG)7	14	13966250	13966263
chr10_2247	p2	(TG)8	16	13968735	13968750
chr10_2248	p2	(CA)6	12	13968983	13968994
chr10_2249	p2	(AC)6	12	13969866	13969877
chr10_2250	p2	(GT)6	12	13971097	13971108
chr10_2251	p2	(AC)7	14	13974300	13974313
chr10_2252	c	aagcagagctgatgtgatatgaatcagcagccatggtcgatgtgcttaatgc	127	13975332	13975458
chr10_2253	c	(GT)8gagaa(AG)6	33	13976371	13976403
chr10_2254	p2	(AC)10	20	13976732	13976751
chr10_2255	p2	(GT)25	50	13983262	13983311
chr10_2256	p2	(GT)18	36	13983472	13983507
chr10_2257	p2	(TG)9	18	13987861	13987878

chr10_2258	p2	(TG)15	30	13988135	13988164
chr10_2259	p2	(CT)10	20	13988751	13988770
chr10_2260	p2	(TG)6	12	13991177	13991188
chr10_2261	p2	(CA)8	16	13993475	13993490
chr10_2262	p2	(AC)25	50	13997752	13997801
chr10_2263	p2	(GT)9	18	14003151	14003168
chr10_2264	p2	(CA)6	12	14003829	14003840
chr10_2265	p2	(CA)13	26	14004282	14004307
chr10_2266	p2	(GT)8	16	14008026	14008041
chr10_2267	p2	(TG)6	12	14011076	14011087
chr10_2268	p2	(CA)8	16	14011877	14011892
chr10_2269	p2	(GT)14	28	14018197	14018224
chr10_2270	p2	(GT)13	26	14018712	14018737
chr10_2271	p2	(AT)6	12	14035435	14035446
chr10_2272	c	(TG)6(TA)8(TG)14	56	14036502	14036557
chr10_2273	c	(CT)15(CA)13	56	14036701	14036756
chr10_2274	p2	(AC)12	24	14049108	14049131
chr10_2275	c	gggatcaaacagggttgtttgactgttcgcctgatgtcaaacctaagtgaaa	143	14050110	14050252
chr10_2276	p2	(GT)6	12	14053671	14053682
chr10_2277	p2	(AG)6	12	14056020	14056031
chr10_2278	p2	(AC)15	30	14056583	14056612
chr10_2279	p2	(GT)16	32	14057993	14058024
chr10_2280	p2	(CA)13	26	14058170	14058195
chr10_2281	p2	(AG)6	12	14059801	14059812
chr10_2282	p2	(TG)14	28	14061338	14061365
chr10_2283	p2	(CA)20	40	14063469	14063508
chr10_2284	p2	(GT)17	34	14063756	14063789
chr10_2285	c	(TC)6g(CA)17	47	14064711	14064757
chr10_2286	p2	(AC)22	44	14069037	14069080
chr10_2287	p2	(CA)16	32	14069465	14069496
chr10_2288	p2	(AC)9	18	14069809	14069826
chr10_2289	p2	(TG)7	14	14070061	14070074
chr10_2290	p2	(GT)15	30	14070775	14070804
chr10_2291	p2	(AG)9	18	14071458	14071475
chr10_2292	p2	(CA)9	18	14073220	14073237
chr10_2293	p5	(AAATA)6	30	14073885	14073914
chr10_2294	p2	(CA)6	12	14075687	14075698
chr10_2295	p2	(AC)7	14	14075814	14075827
chr10_2296	p2	(GT)12	24	14078930	14078953
chr10_2297	p3	(TTC)6	18	14079243	14079260
chr10_2298	p2	(CA)11	22	14079815	14079836
chr10_2299	p2	(GA)6	12	14083653	14083664
chr10_2300	p2	(AC)9	18	14087359	14087376
chr10_2301	p2	(AC)10	20	14093208	14093227
chr10_2302	p2	(AC)11	22	14095503	14095524
chr10_2303	p2	(TG)8	16	14096866	14096881
chr10_2304	p2	(AC)6	12	14097190	14097201
chr10_2305	p2	(TG)6	12	14098658	14098669
chr10_2306	p2	(CA)20	40	14100471	14100510
chr10_2307	p2	(CA)12	24	14100923	14100946
chr10_2308	p2	(AC)7	14	14101167	14101180
chr10_2309	p2	(CA)13	26	14105524	14105549

chr10_2310	p2	(CA)7	14	14106583	14106596
chr10_2311	p2	(CA)6	12	14115841	14115852
chr10_2312	c	(TG)7ta(TG)6	28	14117443	14117470
chr10_2313	p2	(TG)8	16	14118131	14118146
chr10_2314	p2	(AG)6	12	14125044	14125055
chr10_2315	p2	(AC)9	18	14126401	14126418
chr10_2316	p4	(TAGA)10	40	14126776	14126815
chr10_2317	p4	(ATCT)13	52	14127247	14127298
chr10_2318	p2	(GT)6	12	14129501	14129512
chr10_2319	p2	(TG)8	16	14129634	14129649
chr10_2320	p2	(AG)6	12	14129788	14129799
chr10_2321	c	(AC)12(AT)6	36	14130710	14130745
chr10_2322	p2	(TG)10	20	14131530	14131549
chr10_2323	p2	(CA)10	20	14131886	14131905
chr10_2324	p2	(AC)9	18	14132059	14132076
chr10_2325	p2	(GA)7	14	14132668	14132681
chr10_2326	p2	(GT)6	12	14140759	14140770
chr10_2327	p2	(GT)11	22	14141032	14141053
chr10_2328	p2	(TG)6	12	14141474	14141485
chr10_2329	p3	(ATT)6	18	14144525	14144542
chr10_2330	c	(TGTT)7gacta(AG)7	47	14144661	14144707
chr10_2331	p3	(ATT)5	15	14150357	14150371
chr10_2332	p3	(TTG)6	18	14156759	14156776
chr10_2333	c	gggaggaaagcacccactcagactgaaagtgtgtgta(TG)6aagctga	196	14157825	14158020
chr10_2334	p3	(TAA)13	39	14162746	14162784
chr10_2335	p2	(AC)22	44	14165940	14165983
chr10_2336	p2	(TG)7	14	14168036	14168049
chr10_2337	p2	(TG)7	14	14171276	14171289
chr10_2338	p2	(AC)12	24	14179239	14179262
chr10_2339	p2	(TG)15	30	14179385	14179414
chr10_2340	p2	(AG)6	12	14180985	14180996
chr10_2341	p2	(GT)10	20	14189424	14189443
chr10_2342	p2	(GT)17	34	14197702	14197735
chr10_2343	p2	(TG)11	22	14198218	14198239
chr10_2344	p2	(TC)7	14	14198946	14198959
chr10_2345	p2	(GT)15	30	14211636	14211665
chr10_2346	p2	(GT)7	14	14216610	14216623
chr10_2347	p2	(TG)6	12	14225135	14225146
chr10_2348	p2	(TG)11	22	14229643	14229664
chr10_2349	p3	(TGT)6	18	14229882	14229899
chr10_2350	p2	(GT)6	12	14232892	14232903
chr10_2351	p2	(GT)11	22	14238847	14238868
chr10_2352	p2	(GT)12	24	14242821	14242844
chr10_2353	p2	(TG)7	14	14244553	14244566
chr10_2354	p2	(CT)6	12	14261387	14261398
chr10_2355	p2	(AG)6	12	14276595	14276606
chr10_2356	p2	(GT)7	14	14276733	14276746
chr10_2357	p3	(CCA)7	21	14279545	14279565
chr10_2358	p2	(TG)6	12	14294787	14294798
chr10_2359	p2	(GT)10	20	14295133	14295152
chr10_2360	p2	(AC)6	12	14295919	14295930
chr10_2361	p2	(TG)6	12	14298226	14298237

chr10_2362	p2	(TG)13	26	14300600	14300625
chr10_2363	p2	(TG)15	30	14300776	14300805
chr10_2364	p2	(GA)8	16	14314333	14314348
chr10_2365	p2	(TG)7	14	14314543	14314556
chr10_2366	p4	(TTTA)6	24	14318060	14318083
chr10_2367	p2	(GT)6	12	14325486	14325497
chr10_2368	c	(TG)10tagacgagctaatatgtttttatattatctgtcttgac(TG)21	100	14326728	14326827
chr10_2369	p2	(GT)6	12	14327036	14327047
chr10_2370	p4	(AATC)5	20	14332409	14332428
chr10_2371	p2	(AC)7	14	14334266	14334279
chr10_2372	p2	(CA)6	12	14337093	14337104
chr10_2373	p2	(GT)16	32	14355473	14355504
chr10_2374	c	tgtctccctctgtctgcgttaccaagtctgctctgacaccaaacaagattggg	159	14359026	14359184
chr10_2375	p2	(AG)7	14	14372204	14372217
chr10_2376	p2	(TC)14	28	14386372	14386399
chr10_2377	p2	(GT)6	12	14387278	14387289
chr10_2378	p2	(CA)7	14	14388700	14388713
chr10_2379	p2	(TC)13	26	14389257	14389282
chr10_2380	p2	(AG)13	26	14389673	14389698
chr10_2381	p2	(TG)16	32	14393744	14393775
chr10_2382	p2	(TG)17	34	14401163	14401196
chr10_2383	p2	(TG)12	24	14401630	14401653
chr10_2384	p2	(TG)6	12	14405451	14405462
chr10_2385	p3	(CAA)5	15	14425312	14425326
chr10_2386	p3	(CAA)5	15	14427977	14427991
chr10_2387	p2	(TG)6	12	14432258	14432269
chr10_2388	p2	(AC)6	12	14451094	14451105
chr10_2389	c	(CT)7cgctcgctctctctcg(CT)9	48	14457263	14457310
chr10_2390	p2	(CA)6	12	14482022	14482033
chr10_2391	p2	(GA)6	12	14492731	14492742
chr10_2392	c	15gcctgtagacactggttaagctcagactgatcttttcaaatgaaattctc(	94	14506703	14506796
chr10_2393	p2	(CA)12	24	14507186	14507209
chr10_2394	p2	(GA)14	28	14509282	14509309
chr10_2395	p3	(AGA)5	15	14509467	14509481
chr10_2396	p2	(AG)6	12	14510465	14510476
chr10_2397	p2	(AC)28	56	14510874	14510929
chr10_2398	p3	(CTC)6	18	14514735	14514752
chr10_2399	p2	(GA)6	12	14519415	14519426
chr10_2400	p2	(GT)12	24	14521476	14521499
chr10_2401	p2	(AC)6	12	14531247	14531258
chr10_2402	p2	(TG)7	14	14531440	14531453
chr10_2403	p2	(TG)6	12	14531734	14531745
chr10_2404	p2	(TG)12	24	14536550	14536573
chr10_2405	p2	(AC)21	42	14538819	14538860
chr10_2406	p3	(AAT)5	15	14545248	14545262
chr10_2407	p2	(TG)14	28	14546252	14546279
chr10_2408	p2	(AT)9	18	14550742	14550759
chr10_2409	p2	(TA)6	12	14557914	14557925
chr10_2410	p2	(GT)7	14	14561788	14561801
chr10_2411	p2	(TG)10	20	14567122	14567141
chr10_2412	p2	(AG)6	12	14567451	14567462
chr10_2413	p2	(GT)6	12	14571385	14571396

chr10_2414	p2	(GA)7	14	14574081	14574094
chr10_2415	p2	(GT)6	12	14575665	14575676
chr10_2416	p2	(CA)8	16	14576391	14576406
chr10_2417	p2	(CA)16	32	14577039	14577070
chr10_2418	p2	(GT)13	26	14578490	14578515
chr10_2419	c	gaaggtttgggggggacaggaaaattgacaacaggagagcaggtttgag	113	14581041	14581153
chr10_2420	p2	(CA)8	16	14586697	14586712
chr10_2421	p2	(TG)13	26	14588098	14588123
chr10_2422	p2	(AC)11	22	14595185	14595206
chr10_2423	p2	(AT)6	12	14597741	14597752
chr10_2424	p2	(TG)25	50	14599205	14599254
chr10_2425	p2	(GT)7	14	14600579	14600592
chr10_2426	p2	(TC)7	14	14602704	14602717
chr10_2427	p2	(AC)23	46	14605032	14605077
chr10_2428	p2	(TC)6	12	14608068	14608079
chr10_2429	p2	(CA)11	22	14610041	14610062
chr10_2430	p2	(GT)6	12	14614193	14614204
chr10_2431	p2	(TG)9	18	14616182	14616199
chr10_2432	p3	(GGA)5	15	14617512	14617526
chr10_2433	p2	(TG)6	12	14622245	14622256
chr10_2434	c	tgctgtataaaaccagctttcatctt(TC)7attttttctctttacatcaacctc	257	14626569	14626825
chr10_2435	c	(TC)7at(AC)9	34	14629965	14629998
chr10_2436	p2	(AC)9	18	14632429	14632446
chr10_2437	p2	(GT)15	30	14635087	14635116
chr10_2438	c	atttcaatccatcagctttttctgtcttcagcatcctttcaacaatcaaatccac	131	14640763	14640893
chr10_2439	p2	(GT)6	12	14641039	14641050
chr10_2440	p2	(GT)13	26	14642263	14642288
chr10_2441	p2	(TG)7	14	14643541	14643554
chr10_2442	c	(TC)13c(CT)6	39	14646545	14646583
chr10_2443	p2	(TG)11	22	14660389	14660410
chr10_2444	p2	(TG)27	54	14661690	14661743
chr10_2445	p2	(GT)7	14	14664562	14664575
chr10_2446	p3	(CCT)6	18	14669476	14669493
chr10_2447	p2	(GT)7	14	14673599	14673612
chr10_2448	p2	(AC)6	12	14676625	14676636
chr10_2449	p2	(TG)13	26	14678350	14678375
chr10_2450	c	(GT)13actcactgttagccagta(GCT)6	62	14681978	14682039
chr10_2451	c	tgggcctcatcccctctgccatttacaccggggaagaaaagaagtataaa	131	14683144	14683274
chr10_2452	p2	(AC)6	12	14689450	14689461
chr10_2453	p2	(TG)14	28	14689754	14689781
chr10_2454	p3	(AAC)5	15	14692455	14692469
chr10_2455	p2	(AC)6	12	14692885	14692896
chr10_2456	p2	(TG)13	26	14697243	14697268
chr10_2457	p2	(TG)6	12	14697487	14697498
chr10_2458	c	tgagcaaatgttttctctgggttccactaatgttcaacaaacacatacacttct	144	14698842	14698985
chr10_2459	p2	(GA)13	26	14702371	14702396
chr10_2460	p2	(AC)6	12	14704043	14704054
chr10_2461	c	aagaaacgaaaacaagaaccttcacttttcctctgaactctgcttcacctctc	122	14705136	14705257
chr10_2462	c	(GT)6a(TG)7	27	14705396	14705422
chr10_2463	p2	(GT)15	30	14705562	14705591
chr10_2464	c	tgaataagcacggacttgctaacacgtttgacagattgatttagctcagaat	125	14714989	14715113
chr10_2465	p2	(TA)8	16	14716093	14716108

chr10_2466	p2	(CT)6	12	14717067	14717078
chr10_2467	p2	(CT)6	12	14717278	14717289
chr10_2468	p2	(CT)7	14	14721286	14721299
chr10_2469	c	ctgaacaggaatgtcactggagaaaaaaagagacgaagaaatacaa(A(	185	14722297	14722481
chr10_2470	c	;ctctttcctttcctaa(AC)6aatgaatcttccagaatatattattattgcaga	116	14724940	14725055
chr10_2471	p2	(CA)6	12	14725191	14725202
chr10_2472	c	:tttcatcctccaaggcaggaaatgctgcttttaagctctgtgtgtttgagttaa	136	14732764	14732899
chr10_2473	p3	(GGA)6	18	14733374	14733391
chr10_2474	p3	(ATA)5	15	14734647	14734661
chr10_2475	p3	(TCT)5	15	14735466	14735480
chr10_2476	p2	(TG)9	18	14739531	14739548
chr10_2477	p2	(GT)19	38	14740033	14740070
chr10_2478	p2	(TG)9	18	14740216	14740233
chr10_2479	p2	(AG)9	18	14740384	14740401
chr10_2480	p2	(CA)13	26	14744968	14744993
chr10_2481	p2	(GT)9	18	14746790	14746807
chr10_2482	p2	(TG)9	18	14750283	14750300
chr10_2483	p2	(GT)9	18	14750430	14750447
chr10_2484	c	(TG)13agat(AG)7ggagagagagggg(GA)8	73	14754128	14754200
chr10_2485	p2	(AC)19	38	14755886	14755923
chr10_2486	p2	(AC)31	62	14756087	14756148
chr10_2487	p2	(GT)21	42	14757942	14757983
chr10_2488	c	(TG)7c(GT)16	47	14758499	14758545
chr10_2489	p2	(AC)7	14	14765427	14765440
chr10_2490	c	(TC)6tgtgtct(CA)20	59	14772715	14772773
chr10_2491	p2	(TC)8	16	14776704	14776719
chr10_2492	p4	(ATGG)5	20	14785079	14785098
chr10_2493	p2	(CA)8	16	14785556	14785571
chr10_2494	p2	(GT)6	12	14786244	14786255
chr10_2495	p2	(AC)12	24	14788187	14788210
chr10_2496	p2	(TG)11	22	14788316	14788337
chr10_2497	p2	(TG)16	32	14789351	14789382
chr10_2498	p2	(TG)8	16	14789524	14789539
chr10_2499	c	(CT)6t(TC)9(AC)11	53	14790524	14790576
chr10_2500	p2	(CA)10	20	14790708	14790727
chr10_2501	p3	(CTT)8	24	14792209	14792232
chr10_2502	p3	(GAT)7	21	14793074	14793094
chr10_2503	p2	(GT)6	12	14793732	14793743
chr10_2504	p2	(TG)7	14	14807525	14807538
chr10_2505	p2	(AC)7	14	14809906	14809919
chr10_2506	p2	(CA)6	12	14811886	14811897
chr10_2507	p2	(AC)6	12	14812528	14812539
chr10_2508	p2	(AC)6	12	14816371	14816382
chr10_2509	p2	(CA)11	22	14816855	14816876
chr10_2510	p2	(TG)6	12	14817668	14817679
chr10_2511	p4	(TAAA)7	28	14818262	14818289
chr10_2512	p2	(CA)17	34	14820475	14820508
chr10_2513	p3	(TTC)11	33	14829113	14829145
chr10_2514	p2	(TG)14	28	14834271	14834298
chr10_2515	p3	(GTC)5	15	14851674	14851688
chr10_2516	p2	(CA)6	12	14853720	14853731
chr10_2517	p3	(AGT)7	21	14856198	14856218

chr10_2518	p2	(AG)10	20	14858073	14858092
chr10_2519	p2	(AG)6	12	14860272	14860283
chr10_2520	p2	(TA)7	14	14873921	14873934
chr10_2521	p2	(GT)7	14	14896659	14896672
chr10_2522	p2	(GT)7	14	14898066	14898079
chr10_2523	p2	(AT)7	14	14898369	14898382
chr10_2524	p2	(TC)7	14	14926267	14926280
chr10_2525	c	(CA)16(CT)9cacactcactcact(CA)12ct(CA)6	102	14928277	14928378
chr10_2526	p2	(GA)7	14	14928722	14928735
chr10_2527	p3	(GAC)6	18	14929058	14929075
chr10_2528	p3	(GAA)5	15	14936621	14936635
chr10_2529	c	(GT)6(CT)6(GT)7	38	14945715	14945752
chr10_2530	p2	(AC)6	12	14948210	14948221
chr10_2531	p2	(GT)16	32	14950707	14950738
chr10_2532	p3	(AGC)5	15	14964933	14964947
chr10_2533	p2	(CA)9	18	14965498	14965515
chr10_2534	p2	(TG)15	30	14966809	14966838
chr10_2535	p2	(AC)7	14	14967508	14967521
chr10_2536	c	tcagattcaggaagtgaaaatttagatggctgctggtttgtgcatgtgtgtgc	125	14971525	14971649
chr10_2537	p2	(CA)14	28	14975249	14975276
chr10_2538	p2	(AG)8	16	14980029	14980044
chr10_2539	p3	(TGA)5	15	14982214	14982228
chr10_2540	p2	(GT)6	12	14987304	14987315
chr10_2541	p2	(TG)6	12	14999830	14999841
chr10_2542	p2	(GT)7	14	15001552	15001565
chr10_2543	p2	(CA)16	32	15003584	15003615
chr10_2544	p2	(AC)6	12	15005411	15005422
chr10_2545	p3	(ATA)6	18	15005786	15005803
chr10_2546	p2	(GT)6	12	15017015	15017026
chr10_2547	p2	(TC)6	12	15024584	15024595
chr10_2548	p2	(AC)8	16	15025344	15025359
chr10_2549	p2	(TG)6	12	15031297	15031308
chr10_2550	p2	(TA)6	12	15032578	15032589
chr10_2551	p2	(TG)8	16	15044284	15044299
chr10_2552	p2	(GT)15	30	15052321	15052350
chr10_2553	p2	(GT)12	24	15053330	15053353
chr10_2554	c	atggagacagacaaaactcctcttcacatagagcttccttttcctctgttgatc	131	15055741	15055871
chr10_2555	p2	(AT)14	28	15057715	15057742
chr10_2556	p2	(TG)6	12	15060503	15060514
chr10_2557	p2	(TG)7	14	15061903	15061916
chr10_2558	p3	(AAC)5	15	15063047	15063061
chr10_2559	p2	(CA)44	88	15079296	15079383
chr10_2560	p3	(CAA)6	18	15079967	15079984
chr10_2561	p2	(TC)8	16	15086512	15086527
chr10_2562	p2	(TG)31	62	15089690	15089751
chr10_2563	p2	(TG)7	14	15091792	15091805
chr10_2564	p2	(TG)15	30	15101751	15101780
chr10_2565	p2	(TG)20	40	15102467	15102506
chr10_2566	p2	(TG)8	16	15108424	15108439
chr10_2567	p2	(CT)7	14	15117977	15117990
chr10_2568	p3	(TCC)7	21	15118191	15118211
chr10_2569	p4	(ATGG)5	20	15119401	15119420

chr10_2570	p2	(TG)6	12	15122686	15122697
chr10_2571	p2	(GT)6	12	15128633	15128644
chr10_2572	c	(GT)6atgtgtat(CA)8	36	15129009	15129044
chr10_2573	p2	(CA)7	14	15137366	15137379
chr10_2574	p2	(GT)11	22	15137602	15137623
chr10_2575	p2	(TG)9	18	15163510	15163527
chr10_2576	p3	(AAT)5	15	15182529	15182543
chr10_2577	p2	(GT)8	16	15214301	15214316
chr10_2578	p2	(GT)23	46	15238522	15238567
chr10_2579	p2	(GA)7	14	15243123	15243136
chr10_2580	p2	(GA)6	12	15247114	15247125
chr10_2581	p4	(TTTA)6	24	15249020	15249043
chr10_2582	p2	(TC)7	14	15255736	15255749
chr10_2583	p2	(TG)7	14	15259062	15259075
chr10_2584	p4	(GAAT)5	20	15289602	15289621
chr10_2585	p2	(AC)8	16	15291986	15292001
chr10_2586	p2	(CT)6	12	15309982	15309993
chr10_2587	p3	(TGC)5	15	15323896	15323910
chr10_2588	c	(TC)6(AC)14	40	15326267	15326306
chr10_2589	p3	(AAT)6	18	15342237	15342254
chr10_2590	p2	(GA)13	26	15361192	15361217
chr10_2591	c*	(AGA)5(AG)6*	24	15366016	15366039
chr10_2592	p2	(AT)6	12	15381109	15381120
chr10_2593	p2	(GT)6	12	15394397	15394408
chr10_2594	p4	(ATAA)5	20	15398251	15398270
chr10_2595	p4	(ATGG)6	24	15399138	15399161
chr10_2596	p2	(AG)7	14	15402594	15402607
chr10_2597	c	(TG)19tctgccacagtctggaagctttgtcctaacagatggtgg(GA)7	91	15409920	15410010
chr10_2598	p2	(CA)8	16	15410153	15410168
chr10_2599	p2	(CA)15	30	15417467	15417496
chr10_2600	p2	(AC)10	20	15421250	15421269
chr10_2601	p3	(GTG)5	15	15436008	15436022
chr10_2602	p3	(ACA)8	24	15445808	15445831
chr10_2603	p2	(TG)11	22	15446425	15446446
chr10_2604	p2	(AG)16	32	15447531	15447562
chr10_2605	p2	(AC)6	12	15457601	15457612
chr10_2606	p2	(GA)9	18	15461937	15461954
chr10_2607	p2	(GT)14	28	15472233	15472260
chr10_2608	p2	(GT)12	24	15476192	15476215
chr10_2609	p2	(GT)14	28	15476898	15476925
chr10_2610	p2	(AC)16	32	15484937	15484968
chr10_2611	p2	(CT)6	12	15485498	15485509
chr10_2612	p2	(AC)11	22	15488897	15488918
chr10_2613	p2	(CA)6	12	15489142	15489153
chr10_2614	p2	(TC)6	12	15492028	15492039
chr10_2615	p2	(TG)13	26	15492230	15492255
chr10_2616	p2	(CA)6	12	15507262	15507273
chr10_2617	p2	(CA)6	12	15508145	15508156
chr10_2618	p2	(GT)9	18	15511572	15511589
chr10_2619	p2	(GT)6	12	15514505	15514516
chr10_2620	p2	(GT)9	18	15519485	15519502
chr10_2621	p2	(AC)6	12	15519916	15519927

chr10_2622	p2	(AG)9	18	15522061	15522078
chr10_2623	p2	(AC)12	24	15523603	15523626
chr10_2624	p3	(TTA)5	15	15523845	15523859
chr10_2625	p2	(AC)6	12	15524562	15524573
chr10_2626	p2	(GT)6	12	15529377	15529388
chr10_2627	p4	(GAGG)5	20	15531901	15531920
chr10_2628	p2	(TG)9	18	15536178	15536195
chr10_2629	p2	(GT)6	12	15546202	15546213
chr10_2630	p2	(TC)6	12	15548544	15548555
chr10_2631	c	(GT)8(GA)14	44	15555512	15555555
chr10_2632	p2	(TC)7	14	15557411	15557424
chr10_2633	p3	(CCT)6	18	15562829	15562846
chr10_2634	p2	(GT)10	20	15564409	15564428
chr10_2635	p2	(CA)6	12	15566467	15566478
chr10_2636	p2	(GT)6	12	15571258	15571269
chr10_2637	p2	(CA)6	12	15573650	15573661
chr10_2638	p2	(CA)9	18	15573918	15573935
chr10_2639	p2	(CT)7	14	15582634	15582647
chr10_2640	p2	(GA)7	14	15591872	15591885
chr10_2641	p2	(TC)9	18	15593425	15593442
chr10_2642	p2	(CA)13	26	15600258	15600283
chr10_2643	p2	(GT)6	12	15604820	15604831
chr10_2644	p2	(TG)10	20	15619427	15619446
chr10_2645	p2	(GA)8	16	15625192	15625207
chr10_2646	p2	(TG)13	26	15632395	15632420
chr10_2647	p3	(TAT)6	18	15632767	15632784
chr10_2648	p2	(AC)19	38	15638396	15638433
chr10_2649	p2	(AC)6	12	15652109	15652120
chr10_2650	p2	(AC)13	26	15652814	15652839
chr10_2651	p2	(CA)8	16	15672095	15672110
chr10_2652	p2	(AC)11	22	15689283	15689304
chr10_2653	p2	(AT)7	14	15697090	15697103
chr10_2654	p2	(CA)23	46	15698109	15698154
chr10_2655	c	(CT)9gtatcatattttatcctcc(GT)8	53	15699208	15699260
chr10_2656	p2	(AC)19	38	15701333	15701370
chr10_2657	p2	(CA)14	28	15703075	15703102
chr10_2658	p2	(GT)12	24	15710038	15710061
chr10_2659	p2	(GT)15	30	15710215	15710244
chr10_2660	p2	(CT)7	14	15711899	15711912
chr10_2661	p2	(CA)6	12	15712547	15712558
chr10_2662	p2	(TA)9	18	15712708	15712725
chr10_2663	p2	(AC)15	30	15715986	15716015
chr10_2664	p2	(AC)11	22	15725953	15725974
chr10_2665	p2	(TG)9	18	15729058	15729075
chr10_2666	p2	(TC)7	14	15736170	15736183
chr10_2667	p2	(CA)7	14	15737967	15737980
chr10_2668	p2	(TG)18	36	15743288	15743323
chr10_2669	p2	(AT)6	12	15755674	15755685
chr10_2670	p2	(TG)6	12	15758715	15758726
chr10_2671	c	(TC)7(AC)14	42	15762887	15762928
chr10_2672	p2	(CA)19	38	15763147	15763184
chr10_2673	p3	(AAC)8	24	15764273	15764296

chr10_2674	p3	(GTG)5	15	15767716	15767730
chr10_2675	p2	(AC)10	20	15770656	15770675
chr10_2676	p2	(AC)11	22	15771749	15771770
chr10_2677	p2	(AT)6	12	15775576	15775587
chr10_2678	p2	(GT)13	26	15779957	15779982
chr10_2679	p2	(GT)12	24	15782228	15782251
chr10_2680	p2	(AG)7	14	15783037	15783050
chr10_2681	p2	(TG)8	16	15788535	15788550
chr10_2682	p2	(AC)13	26	15793166	15793191
chr10_2683	p2	(CA)8	16	15805022	15805037
chr10_2684	p2	(TC)6	12	15809499	15809510
chr10_2685	p2	(CA)8	16	15809941	15809956
chr10_2686	p2	(AC)11	22	15810101	15810122
chr10_2687	p4	(TTTG)5	20	15813840	15813859
chr10_2688	p2	(TG)8	16	15816174	15816189
chr10_2689	p3	(AAG)5	15	15823021	15823035
chr10_2690	p3	(CCT)5	15	15824666	15824680
chr10_2691	p3	(TGC)5	15	15825704	15825718
chr10_2692	p2	(TA)7	14	15828998	15829011
chr10_2693	p2	(CA)7	14	15842532	15842545
chr10_2694	p2	(AC)13	26	15851875	15851900
chr10_2695	p2	(AC)14	28	15859672	15859699
chr10_2696	p2	(TG)12	24	15863032	15863055
chr10_2697	p2	(CA)11	22	15865390	15865411
chr10_2698	p2	(CA)11	22	15868437	15868458
chr10_2699	p2	(GT)14	28	15870633	15870660
chr10_2700	p2	(GT)13	26	15875464	15875489
chr10_2701	p2	(CA)6	12	15893943	15893954
chr10_2702	p2	(CA)6	12	15907500	15907511
chr10_2703	p2	(AC)10	20	15911609	15911628
chr10_2704	p2	(TC)12	24	15914376	15914399
chr10_2705	p3	(GCA)5	15	15917218	15917232
chr10_2706	p2	(CA)6	12	15920519	15920530
chr10_2707	p2	(GT)7	14	15926988	15927001
chr10_2708	p2	(AC)6	12	15931272	15931283
chr10_2709	p2	(AC)25	50	15938462	15938511
chr10_2710	p2	(CA)6	12	15941188	15941199
chr10_2711	p2	(TA)9	18	15941642	15941659
chr10_2712	p2	(AC)23	46	15942169	15942214
chr10_2713	p2	(TG)8	16	15944467	15944482
chr10_2714	p2	(CA)9	18	15964356	15964373
chr10_2715	p2	(CA)12	24	15965441	15965464
chr10_2716	p3	(TGC)5	15	15965728	15965742
chr10_2717	p2	(AC)9	18	15970275	15970292
chr10_2718	p2	(TG)14	28	15972723	15972750
chr10_2719	c	CTG)7ttgttgttgtttacctagcccttgttgttgtttacctagccc(TTG)[illegible]	84	15973261	15973344
chr10_2720	p2	(AC)7	14	15975896	15975909
chr10_2721	p2	(AC)7	14	15987517	15987530
chr10_2722	p2	(AG)6	12	15988491	15988502
chr10_2723	p2	(CA)7	14	15989525	15989538
chr10_2724	p3	(GCT)8	24	15993642	15993665
chr10_2725	p2	(CA)10	20	15995177	15995196

chr10_2726	p2	(AC)7	14	15998738	15998751
chr10_2727	p2	(AC)11	22	16005088	16005109
chr10_2728	p2	(AC)13	26	16005375	16005400
chr10_2729	c	(TC)6at(TC)6(AC)7ag(AC)8	58	16007921	16007978
chr10_2730	p2	(TC)6	12	16018808	16018819
chr10_2731	p2	(AC)6	12	16022489	16022500
chr10_2732	p2	(AG)6	12	16024742	16024753
chr10_2733	p2	(GT)10	20	16028456	16028475
chr10_2734	p2	(TG)6	12	16029632	16029643
chr10_2735	p2	(CA)19	38	16029789	16029826
chr10_2736	p2	(TG)7	14	16032659	16032672
chr10_2737	p2	(AC)13	26	16035241	16035266
chr10_2738	p2	(AC)17	34	16041738	16041771
chr10_2739	p2	(GT)14	28	16042895	16042922
chr10_2740	p3	(TCC)5	15	16058535	16058549
chr10_2741	p2	(CA)6	12	16060545	16060556
chr10_2742	p2	(AC)12	24	16063018	16063041
chr10_2743	p2	(AC)6	12	16070733	16070744
chr10_2744	p2	(TC)7	14	16071568	16071581
chr10_2745	p2	(TA)8	16	16075829	16075844
chr10_2746	p2	(TG)6	12	16078640	16078651
chr10_2747	p2	(CA)10	20	16089116	16089135
chr10_2748	c	gcaggtaaacatcatccaagatcaagctgtggctaccattttaatttcccagtg	143	16089300	16089442
chr10_2749	p3	(GAG)5	15	16094810	16094824
chr10_2750	p3	(AGG)5	15	16108679	16108693
chr10_2751	p4	(TAGC)5	20	16125403	16125422
chr10_2752	p2	(CA)15	30	16128790	16128819
chr10_2753	p2	(AC)12	24	16129184	16129207
chr10_2754	c	ACA)7aactgcacaccattaacgctgcattactatgttctgctat(TTG)	76	16151290	16151365
chr10_2755	p2	(CA)15	30	16152555	16152584
chr10_2756	p2	(TG)9	18	16171241	16171258
chr10_2757	p2	(TC)7	14	16177499	16177512
chr10_2758	p2	(GT)7	14	16179478	16179491
chr10_2759	p2	(CA)23	46	16218517	16218562
chr10_2760	p2	(AC)6	12	16219527	16219538
chr10_2761	c	(TG)8attctgagtctctgtgagcacaccaggttca(GT)11	69	16220560	16220628
chr10_2762	p2	(GT)13	26	16222682	16222707
chr10_2763	p3	(TTC)5	15	16223359	16223373
chr10_2764	p3	(AGG)7	21	16225833	16225853
chr10_2765	p2	(GT)6	12	16229106	16229117
chr10_2766	p2	(AC)8	16	16231423	16231438
chr10_2767	p3	(CCT)5	15	16233165	16233179
chr10_2768	p2	(TG)14	28	16240683	16240710
chr10_2769	p2	(AC)6	12	16242273	16242284
chr10_2770	p2	(CA)14	28	16248686	16248713
chr10_2771	p3	(TGG)5	15	16249781	16249795
chr10_2772	p3	(GAG)5	15	16249975	16249989
chr10_2773	p2	(GT)51	102	16250818	16250919
chr10_2774	c	gtggggtacttttacaagaggtgaaacctcctgcgctgcagcctgagcaaa	98	16251248	16251345
chr10_2775	p2	(AC)12	24	16259364	16259387
chr10_2776	p3	(TGC)6	18	16264226	16264243
chr10_2777	p2	(CA)12	24	16266018	16266041

chr10_2778	p3	(ACA)5	15	16279441	16279455
chr10_2779	p3	(TTC)6	18	16281824	16281841
chr10_2780	p3	(TTA)5	15	16282091	16282105
chr10_2781	c	(GT)11tc(GA)7	38	16285106	16285143
chr10_2782	p4	(AAAC)6	24	16294069	16294092
chr10_2783	p2	(CA)6	12	16295334	16295345
chr10_2784	p2	(CA)8	16	16315099	16315114
chr10_2785	p2	(TA)7	14	16317143	16317156
chr10_2786	p2	(CA)7	14	16318585	16318598
chr10_2787	p2	(TG)6	12	16336238	16336249
chr10_2788	p2	(CA)8	16	16337759	16337774
chr10_2789	p2	(TG)6	12	16340105	16340116
chr10_2790	p2	(TG)16	32	16350102	16350133
chr10_2791	p2	(TG)11	22	16350256	16350277
chr10_2792	p2	(TG)6	12	16352566	16352577
chr10_2793	p2	(AC)16	32	16354298	16354329
chr10_2794	p2	(CA)9	18	16355916	16355933
chr10_2795	p2	(AC)15	30	16358770	16358799
chr10_2796	p2	(TG)15	30	16360439	16360468
chr10_2797	p2	(AC)7	14	16361947	16361960
chr10_2798	p2	(CA)12	24	16363458	16363481
chr10_2799	p3	(GCT)6	18	16366368	16366385
chr10_2800	p2	(AG)7	14	16373483	16373496
chr10_2801	p2	(TG)13	26	16377641	16377666
chr10_2802	p2	(GT)8	16	16380373	16380388
chr10_2803	p2	(AC)16	32	16382183	16382214
chr10_2804	p2	(CA)6	12	16383931	16383942
chr10_2805	p2	(GT)6	12	16385756	16385767
chr10_2806	p2	(AC)14	28	16388165	16388192
chr10_2807	p2	(CA)7	14	16388466	16388479
chr10_2808	p2	(GA)7	14	16393265	16393278
chr10_2809	p2	(CA)14	28	16394760	16394787
chr10_2810	p2	(GT)6	12	16400036	16400047
chr10_2811	p2	(GT)6	12	16400252	16400263
chr10_2812	p2	(GT)6	12	16402264	16402275
chr10_2813	p2	(CA)10	20	16406423	16406442
chr10_2814	p3	(TTA)5	15	16408043	16408057
chr10_2815	p4	(TGTT)8	32	16411296	16411327
chr10_2816	p2	(AT)7	14	16421029	16421042
chr10_2817	p2	(CA)9	18	16432957	16432974
chr10_2818	p3	(AAC)5	15	16449106	16449120
chr10_2819	p2	(TG)9	18	16454726	16454743
chr10_2820	p2	(TG)19	38	16465985	16466022
chr10_2821	p2	(AC)16	32	16471062	16471093
chr10_2822	p2	(AT)6	12	16474589	16474600
chr10_2823	c	(TG)21ttatgcttggctcagaccgcgagatcag(CA)8	86	16476025	16476110
chr10_2824	p2	(TG)13	26	16487616	16487641
chr10_2825	p2	(CA)24	48	16499084	16499131
chr10_2826	p2	(TA)8	16	16500854	16500869
chr10_2827	p2	(TG)14	28	16503339	16503366
chr10_2828	p2	(GT)7	14	16508632	16508645
chr10_2829	p2	(AC)21	42	16509015	16509056

chr10_2830	p2	(AC)12	24	16510901	16510924
chr10_2831	p2	(GT)23	46	16513321	16513366
chr10_2832	p2	(CT)12	24	16515099	16515122
chr10_2833	p2	(GT)6	12	16528600	16528611
chr10_2834	p2	(GT)8	16	16539281	16539296
chr10_2835	c	ggggggggggggggtcctgtaacctcaaaaagttttgggacattctcctt(	89	16543269	16543357
chr10_2836	p5	(TGATT)5	25	16544595	16544619
chr10_2837	p4	(TATT)5	20	16554299	16554318
chr10_2838	p4	(TTTA)5	20	16556769	16556788
chr10_2839	p2	(CT)6	12	16561354	16561365
chr10_2840	p3	(AGT)5	15	16563534	16563548
chr10_2841	p2	(TG)11	22	16564081	16564102
chr10_2842	p3	(TTA)5	15	16568098	16568112
chr10_2843	p2	(TG)6	12	16570968	16570979
chr10_2844	p3	(TTA)5	15	16576394	16576408
chr10_2845	p2	(TG)18	36	16577227	16577262
chr10_2846	p2	(AC)14	28	16583342	16583369
chr10_2847	p2	(GA)6	12	16585034	16585045
chr10_2848	p2	(GT)7	14	16587325	16587338
chr10_2849	p3	(GGA)5	15	16599499	16599513
chr10_2850	p4	(AATG)6	24	16602113	16602136
chr10_2851	p2	(CA)6	12	16624877	16624888
chr10_2852	p4	(ATGG)5	20	16637894	16637913
chr10_2853	p2	(TG)8	16	16655711	16655726
chr10_2854	p2	(TG)16	32	16656102	16656133
chr10_2855	p2	(TG)17	34	16658323	16658356
chr10_2856	p2	(CA)6	12	16660173	16660184
chr10_2857	p2	(CA)6	12	16668088	16668099
chr10_2858	p2	(CT)12	24	16676574	16676597
chr10_2859	p2	(CA)14	28	16679830	16679857
chr10_2860	p2	(TG)16	32	16680738	16680769
chr10_2861	p3	(TTA)7	21	16681003	16681023
chr10_2862	c	atgcacagtttcctgctatgagtatggtgtcagtattaattaaacacgttgtct	113	16686400	16686512
chr10_2863	p2	(TG)15	30	16687014	16687043
chr10_2864	p2	(AC)8	16	16703433	16703448
chr10_2865	c	C)6aaatactagacaagtacagtacagaaacacgcacacagag(GT)	76	16703708	16703783
chr10_2866	p2	(CA)8	16	16710613	16710628
chr10_2867	p2	(CA)7	14	16710826	16710839
chr10_2868	p2	(AG)10	20	16716167	16716186
chr10_2869	p2	(AC)7	14	16718833	16718846
chr10_2870	p2	(CA)18	36	16718972	16719007
chr10_2871	p2	(CA)7	14	16728298	16728311
chr10_2872	p2	(AC)9	18	16730347	16730364
chr10_2873	p2	(CA)6	12	16730653	16730664
chr10_2874	p2	(CA)7	14	16732292	16732305
chr10_2875	p2	(CA)20	40	16734955	16734994
chr10_2876	c	(CA)6g(AC)6	25	16740449	16740473
chr10_2877	p2	(AC)6	12	16746674	16746685
chr10_2878	p4	(GTAT)5	20	16750998	16751017
chr10_2879	p2	(GT)6	12	16753241	16753252
chr10_2880	p2	(TG)18	36	16771950	16771985
chr10_2881	p2	(GT)6	12	16775964	16775975

chr10_2882	p2	(CA)18	36	16776206	16776241
chr10_2883	p5	(GAGAG)7	35	16780511	16780545
chr10_2884	p2	(AC)12	24	16782950	16782973
chr10_2885	p2	(CA)6	12	16787947	16787958
chr10_2886	p2	(AC)7	14	16790029	16790042
chr10_2887	p2	(GT)11	22	16800501	16800522
chr10_2888	p2	(AC)6	12	16801014	16801025
chr10_2889	p2	(AC)12	24	16806219	16806242
chr10_2890	p2	(TC)6	12	16807197	16807208
chr10_2891	p2	(GT)8	16	16807461	16807476
chr10_2892	p3	(AGC)5	15	16824565	16824579
chr10_2893	p2	(GT)16	32	16830779	16830810
chr10_2894	p2	(CA)22	44	16831661	16831704
chr10_2895	p2	(CA)11	22	16831962	16831983
chr10_2896	p2	(TG)6	12	16859011	16859022
chr10_2897	p2	(TG)13	26	16863540	16863565
chr10_2898	p2	(AC)6	12	16871059	16871070
chr10_2899	p2	(CA)44	88	16873347	16873434
chr10_2900	p2	(AC)16	32	16881201	16881232
chr10_2901	p2	(GA)15	30	16882707	16882736
chr10_2902	p3	(CAT)5	15	16885137	16885151
chr10_2903	p2	(CA)9	18	16886882	16886899
chr10_2904	p2	(GA)6	12	16887358	16887369
chr10_2905	c	(AC)20tcc(CA)8	59	16889408	16889466
chr10_2906	p2	(TG)10	20	16895456	16895475
chr10_2907	p2	(TA)6	12	16896155	16896166
chr10_2908	p3	(CCT)10	30	16918089	16918118
chr10_2909	p4	(ATGT)6	24	16923981	16924004
chr10_2910	p2	(AC)7	14	16939648	16939661
chr10_2911	p2	(CT)6	12	16948295	16948306
chr10_2912	p2	(TA)6	12	16956523	16956534
chr10_2913	p2	(TG)6	12	16963049	16963060
chr10_2914	p3	(CAC)5	15	16965250	16965264
chr10_2915	p4	(ATGG)5	20	16966531	16966550
chr10_2916	p2	(AC)19	38	16972769	16972806
chr10_2917	c	(TC)14(AC)6	40	16974885	16974924
chr10_2918	p4	(CATC)5	20	16976855	16976874
chr10_2919	p2	(AC)13	26	16978797	16978822
chr10_2920	p2	(GT)9	18	16985210	16985227
chr10_2921	p2	(AC)7	14	16985570	16985583
chr10_2922	p2	(AC)9	18	16995201	16995218
chr10_2923	p2	(AT)8	16	16998974	16998989
chr10_2924	p3	(AAC)7	21	17000514	17000534
chr10_2925	c	(CT)7ttctcttctccctctctcttctt(TC)8	55	17002720	17002774
chr10_2926	p3	(TTA)5	15	17005939	17005953
chr10_2927	p2	(AC)12	24	17006275	17006298
chr10_2928	p2	(TC)6	12	17010829	17010840
chr10_2929	p4	(TCCA)5	20	17015866	17015885
chr10_2930	p2	(TG)21	42	17046055	17046096
chr10_2931	p3	(TTG)5	15	17047985	17047999
chr10_2932	p2	(CA)6	12	17051843	17051854
chr10_2933	p2	(AG)7	14	17058805	17058818

chr10_2934	p3	(TAA)5	15	17059043	17059057
chr10_2935	p2	(GA)10	20	17065337	17065356
chr10_2936	p4	(AGAT)5	20	17077933	17077952
chr10_2937	p2	(TC)6	12	17081560	17081571
chr10_2938	c	(CA)11cg(CA)6	36	17083443	17083478
chr10_2939	p2	(AC)8	16	17084300	17084315
chr10_2940	p2	(TG)6	12	17096370	17096381
chr10_2941	p2	(GT)11	22	17096597	17096618
chr10_2942	p2	(CA)8	16	17099073	17099088
chr10_2943	p2	(AC)8	16	17101105	17101120
chr10_2944	p2	(TA)8	16	17117422	17117437
chr10_2945	p2	(CA)6	12	17121788	17121799
chr10_2946	p2	(AG)8	16	17124914	17124929
chr10_2947	p2	(CT)8	16	17132475	17132490
chr10_2948	p4	(TATT)10	40	17133874	17133913
chr10_2949	p2	(CT)6	12	17143154	17143165
chr10_2950	p5	(TCAAA)5	25	17143272	17143296
chr10_2951	p2	(TC)7	14	17154791	17154804
chr10_2952	p2	(TA)6	12	17155377	17155388
chr10_2953	p2	(AC)7	14	17162240	17162253
chr10_2954	p2	(TG)11	22	17162414	17162435
chr10_2955	p2	(GT)7	14	17185522	17185535
chr10_2956	p4	(TTTG)6	24	17187208	17187231
chr10_2957	c	(TG)7ta(TG)12	40	17197705	17197744
chr10_2958	p2	(TG)18	36	17224448	17224483
chr10_2959	p2	(AC)7	14	17234230	17234243
chr10_2960	p2	(GT)6	12	17243925	17243936
chr10_2961	c	(GT)23(GA)12	70	17246749	17246818
chr10_2962	p2	(GT)10	20	17247357	17247376
chr10_2963	c	(TG)16(AG)6	44	17252727	17252770
chr10_2964	p2	(GA)6	12	17267416	17267427
chr10_2965	p2	(TA)12	24	17270810	17270833
chr10_2966	p2	(CA)6	12	17272665	17272676
chr10_2967	p2	(GT)10	20	17276000	17276019
chr10_2968	p2	(GA)6	12	17285808	17285819
chr10_2969	p3	(ATA)5	15	17294648	17294662
chr10_2970	p3	(ATC)5	15	17306311	17306325
chr10_2971	c	(GA)8(GT)7	30	17307772	17307801
chr10_2972	p3	(TAG)9	27	17313094	17313120
chr10_2973	p2	(AC)14	28	17329769	17329796
chr10_2974	p2	(GT)11	22	17337777	17337798
chr10_2975	p2	(TC)7	14	17350765	17350778
chr10_2976	p2	(GT)11	22	17362635	17362656
chr10_2977	p2	(CT)6	12	17367743	17367754
chr10_2978	p2	(TC)6	12	17378463	17378474
chr10_2979	p4	(CCAT)5	20	17405332	17405351
chr10_2980	p2	(GA)8	16	17406331	17406346
chr10_2981	p2	(CT)6	12	17408789	17408800
chr10_2982	p2	(CA)9	18	17410541	17410558
chr10_2983	p2	(CA)19	38	17415578	17415615
chr10_2984	p2	(CA)7	14	17429311	17429324
chr10_2985	p2	(AG)6	12	17438547	17438558

chr10_2986	p2	(GT)11	22	17440381	17440402
chr10_2987	p2	(AC)6	12	17448698	17448709
chr10_2988	p2	(TG)7	14	17450575	17450588
chr10_2989	p2	(TG)7	14	17451601	17451614
chr10_2990	p2	(CT)6	12	17466143	17466154
chr10_2991	p2	(TG)6	12	17468449	17468460
chr10_2992	p2	(GA)10	20	17474837	17474856
chr10_2993	p2	(TA)8	16	17475423	17475438
chr10_2994	p2	(TG)11	22	17478445	17478466
chr10_2995	p2	(TG)9	18	17483153	17483170
chr10_2996	p2	(CA)6	12	17491481	17491492
chr10_2997	p2	(TC)6	12	17502655	17502666
chr10_2998	p2	(AC)7	14	17520532	17520545
chr10_2999	c	(GT)13gc(GT)6	40	17540679	17540718
chr10_3000	p3	(AAT)6	18	17542330	17542347
chr10_3001	p4	(TCCA)5	20	17544975	17544994
chr10_3002	p2	(CA)9	18	17550955	17550972
chr10_3003	p2	(AC)14	28	17551092	17551119
chr10_3004	p3	(TGC)5	15	17553306	17553320
chr10_3005	p2	(AT)8	16	17557196	17557211
chr10_3006	p2	(AC)22	44	17573843	17573886
chr10_3007	p4	(AATA)6	24	17577859	17577882
chr10_3008	p2	(AC)10	20	17581171	17581190
chr10_3009	p2	(AC)10	20	17581472	17581491
chr10_3010	p2	(CA)6	12	17582076	17582087
chr10_3011	p2	(TC)7	14	17584380	17584393
chr10_3012	p2	(TA)9	18	17585125	17585142
chr10_3013	p2	(AG)6	12	17586183	17586194
chr10_3014	p2	(GT)7	14	17588495	17588508
chr10_3015	p2	(CA)10	20	17590587	17590606
chr10_3016	p2	(AC)9	18	17592110	17592127
chr10_3017	p2	(TA)6	12	17593109	17593120
chr10_3018	p2	(AC)25	50	17597633	17597682
chr10_3019	p2	(GA)10	20	17612481	17612500
chr10_3020	c	(CT)13ttct(CA)6	42	17639394	17639435
chr10_3021	p2	(GT)6	12	17639763	17639774
chr10_3022	p4	(ATCC)5	20	17639958	17639977
chr10_3023	p3	(AAT)5	15	17645365	17645379
chr10_3024	p2	(GT)24	48	17647349	17647396
chr10_3025	p2	(TC)8	16	17647603	17647618
chr10_3026	c	gtttgtgtgctccaggctctgccttaacttaaccatatgatgaaaggggatcc	144	17647757	17647900
chr10_3027	p2	(GT)8	16	17651831	17651846
chr10_3028	p2	(AC)6	12	17668927	17668938
chr10_3029	p2	(TG)13	26	17673196	17673221
chr10_3030	p2	(TG)11	22	17673944	17673965
chr10_3031	c	(GT)7(GA)11	36	17682554	17682589
chr10_3032	p2	(GT)9	18	17698623	17698640
chr10_3033	p2	(TG)12	24	17701950	17701973
chr10_3034	p2	(TC)6	12	17708410	17708421
chr10_3035	p2	(AC)28	56	17709366	17709421
chr10_3036	p3	(TGT)6	18	17724840	17724857
chr10_3037	p3	(TGT)8	24	17724973	17724996

chr10_3038	c	(TG)6c(GT)7a(TG)9	46	17744514	17744559
chr10_3039	p2	(AT)7	14	17757114	17757127
chr10_3040	p2	(AG)14	28	17760692	17760719
chr10_3041	p2	(GT)13	26	17767809	17767834
chr10_3042	p2	(TG)10	20	17772440	17772459
chr10_3043	p2	(CA)8	16	17778515	17778530
chr10_3044	p3	(CAT)5	15	17783841	17783855
chr10_3045	p3	(TCC)6	18	17791054	17791071
chr10_3046	p2	(GT)6	12	17793018	17793029
chr10_3047	p2	(AC)7	14	17805838	17805851
chr10_3048	p3	(CTC)6	18	17810487	17810504
chr10_3049	p2	(CA)6	12	17816623	17816634
chr10_3050	p2	(GT)8	16	17824090	17824105
chr10_3051	c	(AC)14(AT)7	42	17826005	17826046
chr10_3052	p2	(AG)6	12	17829700	17829711
chr10_3053	p2	(AG)6	12	17830743	17830754
chr10_3054	p3	(AAG)5	15	17834010	17834024
chr10_3055	p3	(AAT)5	15	17836768	17836782
chr10_3056	p4	(ATCC)5	20	17838673	17838692
chr10_3057	p2	(TG)17	34	17858357	17858390
chr10_3058	p2	(TC)7	14	17863115	17863128
chr10_3059	c	aggtgctatattgagtgaacaactgagagcaatgctgaactggatgtctgct	115	17863608	17863722
chr10_3060	p2	(GT)18	36	17868428	17868463
chr10_3061	c	(GT)7gcgcgtgtgtgc(GA)6	38	17870627	17870664
chr10_3062	p2	(TG)17	34	17871008	17871041
chr10_3063	p2	(TG)6	12	17871391	17871402
chr10_3064	p2	(CA)17	34	17875648	17875681
chr10_3065	p2	(GT)15	30	17881794	17881823
chr10_3066	p2	(CA)11	22	17882570	17882591
chr10_3067	p2	(TG)9	18	17892688	17892705
chr10_3068	p2	(AC)12	24	17894224	17894247
chr10_3069	p3	(CTC)5	15	17899062	17899076
chr10_3070	p3	(CCT)7	21	17903010	17903030
chr10_3071	p2	(CA)19	38	17903900	17903937
chr10_3072	p2	(CT)6	12	17905500	17905511
chr10_3073	p2	(CA)19	38	17906288	17906325
chr10_3074	p2	(CT)7	14	17907979	17907992
chr10_3075	p2	(CT)6	12	17908331	17908342
chr10_3076	p2	(CT)7	14	17910349	17910362
chr10_3077	p2	(CT)6	12	17910701	17910712
chr10_3078	p2	(CT)7	14	17913033	17913046
chr10_3079	p2	(CT)6	12	17913385	17913396
chr10_3080	p3	(TTG)5	15	17916993	17917007
chr10_3081	p2	(GT)15	30	17919009	17919038
chr10_3082	p2	(TG)6	12	17919357	17919368
chr10_3083	p2	(TG)6	12	17919473	17919484
chr10_3084	p2	(CA)17	34	17926610	17926643
chr10_3085	p2	(GT)8	16	17927695	17927710
chr10_3086	p2	(GT)16	32	17946423	17946454
chr10_3087	p2	(CA)6	12	17960806	17960817
chr10_3088	p2	(TG)9	18	17966498	17966515
chr10_3089	p2	(GT)9	18	17973314	17973331

chr10_3090	p2	(TG)33	66	17973804	17973869
chr10_3091	c	(TC)10(AC)21	62	17975703	17975764
chr10_3092	p2	(CA)6	12	17976012	17976023
chr10_3093	p2	(AG)7	14	17976488	17976501
chr10_3094	p2	(GT)8	16	17982815	17982830
chr10_3095	p2	(TA)7	14	17983220	17983233
chr10_3096	p2	(CA)10	20	17985328	17985347
chr10_3097	p2	(AC)6	12	17986608	17986619
chr10_3098	p2	(CA)17	34	17989354	17989387
chr10_3099	p2	(AC)7	14	17990183	17990196
chr10_3100	p2	(AC)13	26	17990331	17990356
chr10_3101	c	(CT)7gtcactctgtcaaagacatacc(CA)12	60	17990487	17990546
chr10_3102	p2	(TG)9	18	17990769	17990786
chr10_3103	p2	(GT)11	22	17990995	17991016
chr10_3104	c	tgtcaacgtgttcgctcattagtgctgtcaa(AC)15atctgctcctggctg	167	17992068	17992234
chr10_3105	c	cactggccatcccacacatgtatgcactctcctcgagccatttctgtttaca	96	17994789	17994884
chr10_3106	p2	(CA)16	32	17995489	17995520
chr10_3107	p3	(TGA)5	15	18000464	18000478
chr10_3108	p2	(AG)11	22	18001425	18001446
chr10_3109	p2	(CT)6	12	18002523	18002534
chr10_3110	p2	(AC)6	12	18003524	18003535
chr10_3111	p2	(AC)19	38	18004539	18004576
chr10_3112	p2	(TG)9	18	18007841	18007858
chr10_3113	p2	(AC)11	22	18008505	18008526
chr10_3114	p3	(AAT)8	24	18014189	18014212
chr10_3115	p2	(TG)8	16	18015562	18015577
chr10_3116	p2	(AG)6	12	18024698	18024709
chr10_3117	c	(CA)6(TA)6	24	18025003	18025026
chr10_3118	p2	(TG)8	16	18025749	18025764
chr10_3119	p2	(CA)11	22	18038378	18038399
chr10_3120	c	(CT)6ttccacacacaccttattccctctgtctccc(CA)17	77	18043168	18043244
chr10_3121	p2	(GT)9	18	18044687	18044704
chr10_3122	p2	(TC)6	12	18045100	18045111
chr10_3123	p2	(AG)9	18	18046138	18046155
chr10_3124	p2	(GA)8	16	18048043	18048058
chr10_3125	p2	(TC)35	70	18048554	18048623
chr10_3126	p2	(CA)14	28	18051984	18052011
chr10_3127	p2	(AC)20	40	18053529	18053568
chr10_3128	c	ccacacacaagacacccccctctctcaacaacnnnnnnnnnnnnnnntct	137	18055324	18055460
chr10_3129	c	(CA)13(CT)12(CA)7	64	18061346	18061409
chr10_3130	p5	(TTTGA)5	25	18068966	18068990
chr10_3131	p2	(CA)8	16	18071758	18071773
chr10_3132	p2	(GT)6	12	18079858	18079869
chr10_3133	p2	(GT)8	16	18084868	18084883
chr10_3134	p2	(GT)9	18	18085094	18085111
chr10_3135	c	tcattatgtttcctgaaaggccacttggctattaatcagaaacagatcagcta	186	18090365	18090550
chr10_3136	p2	(AG)6	12	18092225	18092236
chr10_3137	p2	(CA)10	20	18094268	18094287
chr10_3138	p2	(CA)12	24	18100699	18100722
chr10_3139	p2	(AC)6	12	18100826	18100837
chr10_3140	p2	(TG)6	12	18105423	18105434
chr10_3141	p2	(TG)16	32	18112568	18112599

chr10_3142	c	(AC)6aaaacta(AC)9	37	18117764	18117800
chr10_3143	p2	(TG)6	12	18119912	18119923
chr10_3144	p2	(GA)6	12	18120125	18120136
chr10_3145	p3	(GGA)7	21	18120949	18120969
chr10_3146	p2	(AT)6	12	18127923	18127934
chr10_3147	p2	(AC)6	12	18138246	18138257
chr10_3148	p2	(CA)23	46	18140836	18140881
chr10_3149	p3	(TAA)5	15	18141060	18141074
chr10_3150	p2	(GT)8	16	18149493	18149508
chr10_3151	p3	(CTT)5	15	18151268	18151282
chr10_3152	p2	(GA)6	12	18155919	18155930
chr10_3153	c	gcagcagtggaacagacaaacccccaaagcacatcaccttcaaaaagaaa	128	18156046	18156173
chr10_3154	p2	(CA)8	16	18157224	18157239
chr10_3155	p2	(GA)9	18	18164463	18164480
chr10_3156	p2	(GC)7	14	18165153	18165166
chr10_3157	p4	(ATGG)5	20	18167399	18167418
chr10_3158	p2	(CA)13	26	18176158	18176183
chr10_3159	p2	(TC)6	12	18176656	18176667
chr10_3160	p2	(CA)10	20	18180066	18180085
chr10_3161	p2	(GT)7	14	18180791	18180804
chr10_3162	p2	(TG)18	36	18186085	18186120
chr10_3163	p2	(GT)24	48	18197270	18197317
chr10_3164	p4	(TACC)5	20	18203570	18203589
chr10_3165	p2	(AC)10	20	18205961	18205980
chr10_3166	p2	(TG)8	16	18207014	18207029
chr10_3167	p2	(AC)7	14	18208753	18208766
chr10_3168	p2	(CA)16	32	18209057	18209088
chr10_3169	p2	(AG)7	14	18215705	18215718
chr10_3170	p2	(TG)7	14	18218185	18218198
chr10_3171	p2	(CT)7	14	18221091	18221104
chr10_3172	p2	(AC)16	32	18223398	18223429
chr10_3173	p2	(GT)15	30	18223566	18223595
chr10_3174	p2	(TC)6	12	18225752	18225763
chr10_3175	p3	(ATA)5	15	18227590	18227604
chr10_3176	p2	(GT)18	36	18231070	18231105
chr10_3177	p2	(GT)23	46	18231692	18231737
chr10_3178	p2	(GT)13	26	18233518	18233543
chr10_3179	p2	(AC)15	30	18237740	18237769
chr10_3180	c	gcagtgctctcaggtcgactatggaggtcaggagaggactccgcctggca	121	18242063	18242183
chr10_3181	p2	(GT)19	38	18242450	18242487
chr10_3182	p2	(GT)21	42	18245394	18245435
chr10_3183	p2	(TG)12	24	18245639	18245662
chr10_3184	p2	(AC)6	12	18245839	18245850
chr10_3185	p2	(CA)7	14	18245952	18245965
chr10_3186	p2	(TG)11	22	18253390	18253411
chr10_3187	p2	(GA)6	12	18253639	18253650
chr10_3188	p2	(AC)13	26	18253752	18253777
chr10_3189	p2	(CA)14	28	18260627	18260654
chr10_3190	p2	(CA)7	14	18265628	18265641
chr10_3191	c	(AC)22g(CA)6	57	18267086	18267142
chr10_3192	p2	(TG)7	14	18267273	18267286
chr10_3193	p2	(TG)7	14	18286875	18286888

chr10_3194	p2	(GT)20	40	18289927	18289966
chr10_3195	p2	(AC)17	34	18292991	18293024
chr10_3196	p2	(CA)29	58	18297292	18297349
chr10_3197	p2	(CA)23	46	18297497	18297542
chr10_3198	p2	(CA)19	38	18297662	18297699
chr10_3199	c	(AC)10atgcacgtaataagtatgtacattacatgcact(CA)6	65	18297825	18297889
chr10_3200	p2	(TA)7	14	18304542	18304555
chr10_3201	p2	(CA)12	24	18306269	18306292
chr10_3202	p2	(AC)12	24	18309795	18309818
chr10_3203	p4	(AAAT)6	24	18313149	18313172
chr10_3204	p2	(AC)20	40	18315401	18315440
chr10_3205	p2	(AC)7	14	18316986	18316999
chr10_3206	p2	(AC)20	40	18318539	18318578
chr10_3207	p2	(AC)6	12	18328926	18328937
chr10_3208	p2	(AC)15	30	18332655	18332684
chr10_3209	p2	(AC)10	20	18333195	18333214
chr10_3210	p3	(CTC)7	21	18335737	18335757
chr10_3211	p2	(GT)12	24	18342400	18342423
chr10_3212	p3	(CTG)5	15	18344071	18344085
chr10_3213	p2	(GA)6	12	18347955	18347966
chr10_3214	p3	(TGG)5	15	18368394	18368408
chr10_3215	p2	(AC)14	28	18370144	18370171
chr10_3216	p2	(AC)14	28	18377618	18377645
chr10_3217	p2	(GT)9	18	18379476	18379493
chr10_3218	p2	(TG)8	16	18380299	18380314
chr10_3219	p2	(TC)6	12	18382539	18382550
chr10_3220	p2	(TG)10	20	18383604	18383623
chr10_3221	p2	(AC)21	42	18383804	18383845
chr10_3222	p2	(TG)7	14	18383986	18383999
chr10_3223	p2	(GT)10	20	18385694	18385713
chr10_3224	p2	(GT)16	32	18385881	18385912
chr10_3225	p2	(AC)19	38	18387206	18387243
chr10_3226	p2	(AC)11	22	18387381	18387402
chr10_3227	p2	(AG)6	12	18388847	18388858
chr10_3228	p2	(AC)7	14	18389526	18389539
chr10_3229	c	(AG)6(TG)12	36	18391336	18391371
chr10_3230	c	gtgcacacatgaata(TG)8cagtggcacggtgcctgtcattgtgccctgg	501	18392100	18392600
chr10_3231	p2	(CA)9	18	18392892	18392909
chr10_3232	p2	(CA)22	44	18396898	18396941
chr10_3233	p2	(CA)6	12	18397510	18397521
chr10_3234	p2	(TG)6	12	18397794	18397805
chr10_3235	p2	(AC)6	12	18400086	18400097
chr10_3236	p2	(AC)6	12	18401285	18401296
chr10_3237	p2	(AC)7	14	18413063	18413076
chr10_3238	p2	(TG)8	16	18422452	18422467
chr10_3239	p2	(GT)6	12	18422663	18422674
chr10_3240	p2	(AT)7	14	18432370	18432383
chr10_3241	p2	(CA)6	12	18441354	18441365
chr10_3242	p2	(CA)8	16	18444963	18444978
chr10_3243	p4	(AAAC)5	20	18450735	18450754
chr10_3244	p4	(AATC)6	24	18452160	18452183
chr10_3245	p2	(TG)14	28	18459755	18459782

chr10_3246	c	)14agagacagtaaataatcatttccaaataatcattttcaatactaccag(T	90	18463286	18463375
chr10_3247	p2	(TG)8	16	18487247	18487262
chr10_3248	p4	(TGAG)5	20	18488283	18488302
chr10_3249	p2	(AT)6	12	18493372	18493383
chr10_3250	p3	(GAG)5	15	18499068	18499082
chr10_3251	p2	(GT)6	12	18500295	18500306
chr10_3252	p2	(TG)6	12	18512543	18512554
chr10_3253	p2	(CA)11	22	18513854	18513875
chr10_3254	p2	(CA)18	36	18518607	18518642
chr10_3255	p2	(CA)11	22	18520106	18520127
chr10_3256	p3	(GAG)8	24	18529241	18529264
chr10_3257	p2	(TG)13	26	18529769	18529794
chr10_3258	p2	(AT)8	16	18534795	18534810
chr10_3259	p2	(GT)7	14	18536544	18536557
chr10_3260	p2	(AC)13	26	18537671	18537696
chr10_3261	p2	(CA)10	20	18541178	18541197
chr10_3262	p2	(TG)11	22	18542500	18542521
chr10_3263	p2	(CA)17	34	18542675	18542708
chr10_3264	p2	(AC)9	18	18546636	18546653
chr10_3265	p2	(AC)27	54	18546887	18546940
chr10_3266	p2	(TG)16	32	18547276	18547307
chr10_3267	p3	(AGG)7	21	18548423	18548443
chr10_3268	p3	(GCT)5	15	18550735	18550749
chr10_3269	p2	(CA)15	30	18554071	18554100
chr10_3270	c	(CA)9(CT)9	36	18554216	18554251
chr10_3271	p2	(GT)10	20	18563766	18563785
chr10_3272	p2	(AC)9	18	18567018	18567035
chr10_3273	p2	(TG)10	20	18568766	18568785
chr10_3274	p2	(CA)8	16	18568965	18568980
chr10_3275	p3	(TGT)5	15	18571364	18571378
chr10_3276	p2	(TG)7	14	18572911	18572924
chr10_3277	p2	(TG)15	30	18573100	18573129
chr10_3278	p2	(TG)12	24	18574409	18574432
chr10_3279	p2	(AC)8	16	18574640	18574655
chr10_3280	p2	(CT)8	16	18575303	18575318
chr10_3281	p2	(AC)7	14	18575862	18575875
chr10_3282	p2	(GT)21	42	18580081	18580122
chr10_3283	p4	(CTTT)5	20	18590895	18590914
chr10_3284	p2	(AT)7	14	18593498	18593511
chr10_3285	p2	(CA)11	22	18594387	18594408
chr10_3286	p2	(TC)13	26	18595056	18595081
chr10_3287	p2	(CA)6	12	18596904	18596915
chr10_3288	p2	(TG)22	44	18597576	18597619
chr10_3289	p2	(GT)11	22	18619357	18619378
chr10_3290	c	(AC)10(AT)7	34	18623545	18623578
chr10_3291	p2	(AT)6	12	18623907	18623918
chr10_3292	p2	(GT)17	34	18626106	18626139
chr10_3293	p3	(GCA)5	15	18634173	18634187
chr10_3294	p2	(AC)7	14	18657580	18657593
chr10_3295	p2	(GA)20	40	18664260	18664299
chr10_3296	p2	(CA)8	16	18665727	18665742
chr10_3297	p2	(AC)11	22	18666248	18666269

chr10_3298	p2	(AC)9	18	18667584	18667601
chr10_3299	p2	(AC)6	12	18673658	18673669
chr10_3300	p2	(GA)9	18	18675452	18675469
chr10_3301	c	(AC)8gt(AC)8gt(AC)8gt(AC)8	70	18676060	18676129
chr10_3302	p2	(GT)6	12	18701588	18701599
chr10_3303	p2	(GA)11	22	18702813	18702834
chr10_3304	p2	(TC)9	18	18703156	18703173
chr10_3305	p2	(CA)9	18	18704423	18704440
chr10_3306	p2	(CA)18	36	18704550	18704585
chr10_3307	p2	(TG)8	16	18704925	18704940
chr10_3308	c	(ATT)5(ACT)5	30	18708538	18708567
chr10_3309	p2	(TG)13	26	18710151	18710176
chr10_3310	p2	(CA)47	94	18710497	18710590
chr10_3311	p3	(ATT)5	15	18713072	18713086
chr10_3312	p2	(AC)14	28	18713192	18713219
chr10_3313	p2	(CA)6	12	18716129	18716140
chr10_3314	c	(TC)7tttt(AC)12	42	18721618	18721659
chr10_3315	p4	(CATC)5	20	18724806	18724825
chr10_3316	p2	(TG)7	14	18728625	18728638
chr10_3317	p2	(TG)6	12	18730229	18730240
chr10_3318	p2	(GT)12	24	18738600	18738623
chr10_3319	p3	(TGC)5	15	18739789	18739803
chr10_3320	p3	(GTA)5	15	18741390	18741404
chr10_3321	p2	(CA)8	16	18742156	18742171
chr10_3322	p2	(GT)20	40	18742570	18742609
chr10_3323	p2	(GT)14	28	18742736	18742763
chr10_3324	c*	(TC)6(CTC)5*	24	18743233	18743256
chr10_3325	p3	(GGA)5	15	18743716	18743730
chr10_3326	p2	(GT)8	16	18745189	18745204
chr10_3327	p2	(CA)17	34	18756374	18756407
chr10_3328	p2	(AC)7	14	18757037	18757050
chr10_3329	p2	(TG)7	14	18757187	18757200
chr10_3330	p2	(CA)6	12	18758684	18758695
chr10_3331	p2	(TA)7	14	18777861	18777874
chr10_3332	p5	(AGCCT)5	25	18784836	18784860
chr10_3333	c	5acacagcattaagtcctcagggttaaggcatgtagtcatattgtggcacttt	98	18785503	18785600
chr10_3334	p2	(AC)14	28	18790392	18790419
chr10_3335	p2	(AG)12	24	18792045	18792068
chr10_3336	p2	(TG)25	50	18798108	18798157
chr10_3337	p3	(AGG)8	24	18806663	18806686
chr10_3338	p2	(CA)6	12	18806986	18806997
chr10_3339	p2	(AC)8	16	18807177	18807192
chr10_3340	c	(GT)20(GA)13	66	18807323	18807388
chr10_3341	p2	(AG)8	16	18807492	18807507
chr10_3342	p2	(TC)7	14	18820168	18820181
chr10_3343	p4	(TATT)5	20	18820927	18820946
chr10_3344	p3	(ATA)6	18	18824180	18824197
chr10_3345	p2	(CA)17	34	18850168	18850201
chr10_3346	p2	(TG)9	18	18853281	18853298
chr10_3347	p2	(CA)6	12	18854109	18854120
chr10_3348	p2	(TG)12	24	18859387	18859410
chr10_3349	p2	(CA)7	14	18867707	18867720

chr10_3350	p2	(CA)7	14	18868883	18868896
chr10_3351	p2	(TC)9	18	18871740	18871757
chr10_3352	p4	(CATT)5	20	18877949	18877968
chr10_3353	p3	(CAT)5	15	18884636	18884650
chr10_3354	p2	(AC)21	42	18895835	18895876
chr10_3355	p3	(AGT)5	15	18916136	18916150
chr10_3356	p2	(TG)16	32	18921154	18921185
chr10_3357	p2	(TG)24	48	18922240	18922287
chr10_3358	p3	(TAA)5	15	18938138	18938152
chr10_3359	p2	(AC)6	12	18949757	18949768
chr10_3360	p4	(AAAC)6	24	18952807	18952830
chr10_3361	c	(GT)11aaatgtgatctgtggcctgtcagccaaaggtgtgta(TG)6	70	18955601	18955670
chr10_3362	p2	(CA)6	12	18964013	18964024
chr10_3363	p2	(AC)13	26	18966376	18966401
chr10_3364	p2	(CA)7	14	18967330	18967343
chr10_3365	p2	(CA)9	18	18981959	18981976
chr10_3366	p3	(TCC)8	24	18983481	18983504
chr10_3367	p2	(GT)9	18	18983609	18983626
chr10_3368	p3	(ACA)7	21	18991745	18991765
chr10_3369	p2	(GA)6	12	18999681	18999692
chr10_3370	p3	(ATA)5	15	19002242	19002256
chr10_3371	p2	(AC)6	12	19007717	19007728
chr10_3372	p2	(GT)9	18	19009558	19009575
chr10_3373	p2	(AC)10	20	19009743	19009762
chr10_3374	p2	(CA)8	16	19013393	19013408
chr10_3375	p2	(AG)11	22	19015742	19015763
chr10_3376	p2	(CA)9	18	19017151	19017168
chr10_3377	p2	(AC)11	22	19019431	19019452
chr10_3378	p2	(AG)6	12	19023092	19023103
chr10_3379	p2	(GT)20	40	19024950	19024989
chr10_3380	p2	(CA)22	44	19051822	19051865
chr10_3381	p2	(TC)11	22	19053716	19053737
chr10_3382	p2	(AT)6	12	19068977	19068988
chr10_3383	p2	(AC)7	14	19076644	19076657
chr10_3384	p3	(CAA)6	18	19082392	19082409
chr10_3385	p2	(GT)9	18	19082876	19082893
chr10_3386	p2	(GT)8	16	19084699	19084714
chr10_3387	p2	(GT)9	18	19088335	19088352
chr10_3388	p2	(TG)17	34	19088932	19088965
chr10_3389	p2	(AC)8	16	19091732	19091747
chr10_3390	p2	(CA)21	42	19101298	19101339
chr10_3391	p3	(AGG)6	18	19106422	19106439
chr10_3392	p2	(GT)6	12	19111112	19111123
chr10_3393	p2	(CA)30	60	19115172	19115231
chr10_3394	p2	(AC)6	12	19117024	19117035
chr10_3395	p2	(CA)12	24	19117244	19117267
chr10_3396	p2	(GT)15	30	19117644	19117673
chr10_3397	p2	(AC)8	16	19123244	19123259
chr10_3398	p2	(CA)9	18	19138670	19138687
chr10_3399	p2	(TA)7	14	19148838	19148851
chr10_3400	p3	(TTA)6	18	19149428	19149445
chr10_3401	p2	(CA)9	18	19151465	19151482

chr10_3402	p2	(CA)6	12	19152832	19152843
chr10_3403	p2	(AC)14	28	19157322	19157349
chr10_3404	p2	(AC)13	26	19171717	19171742
chr10_3405	p2	(AC)12	24	19172119	19172142
chr10_3406	c	(TA)9ttcactatggcgacccctaacatgaacagctgtag(GTA)7	74	19176686	19176759
chr10_3407	p2	(GA)6	12	19177507	19177518
chr10_3408	p3	(TCA)5	15	19180917	19180931
chr10_3409	c	aaagaatttgtgtccagtacagaaccaaggtagccatctgtggcgccaact(	89	19182842	19182930
chr10_3410	p2	(CA)6	12	19200668	19200679
chr10_3411	p3	(GCA)6	18	19214194	19214211
chr10_3412	c	(TC)7tt(TC)8	32	19214972	19215003
chr10_3413	p2	(GT)7	14	19217817	19217830
chr10_3414	p2	(GT)10	20	19221500	19221519
chr10_3415	p2	(TG)9	18	19221997	19222014
chr10_3416	p2	(TG)8	16	19235500	19235515
chr10_3417	p2	(CA)6	12	19240220	19240231
chr10_3418	c	(GT)9(GAG)5	33	19243660	19243692
chr10_3419	p2	(TG)11	22	19244054	19244075
chr10_3420	p2	(GT)7	14	19248102	19248115
chr10_3421	p2	(GT)14	28	19252347	19252374
chr10_3422	p2	(TG)13	26	19255289	19255314
chr10_3423	c	(GT)6acacagctgagcattgtaacacgtccagtctctgag(GT)6	60	19258540	19258599
chr10_3424	p2	(GT)8	16	19259133	19259148
chr10_3425	c	ctcacacttgttctatctctttgtcccacctgtcctgttatttctcacctagacaa	131	19259544	19259674
chr10_3426	p3	(GCA)9	27	19262655	19262681
chr10_3427	p2	(TG)6	12	19263232	19263243
chr10_3428	p2	(TG)14	28	19264564	19264591
chr10_3429	p2	(TA)6	12	19267781	19267792
chr10_3430	c	igttattaatagaaaattagtggggtcgggtggagaaaagggcacccggttg	101	19268672	19268772
chr10_3431	p2	(CA)9	18	19269299	19269316
chr10_3432	p2	(AC)14	28	19270015	19270042
chr10_3433	p2	(CA)6	12	19270829	19270840
chr10_3434	p2	(GT)17	34	19272362	19272395
chr10_3435	p2	(TG)16	32	19273202	19273233
chr10_3436	p2	(TG)10	20	19273974	19273993
chr10_3437	c	(GT)6gagt(GA)6	28	19274154	19274181
chr10_3438	p2	(CT)8	16	19274289	19274304
chr10_3439	p3	(CCT)5	15	19278225	19278239
chr10_3440	p2	(TG)7	14	19279290	19279303
chr10_3441	p2	(TG)13	26	19279669	19279694
chr10_3442	p2	(TG)17	34	19283431	19283464
chr10_3443	p2	(AG)9	18	19284322	19284339
chr10_3444	p2	(CA)19	38	19285550	19285587
chr10_3445	c	(AC)6tgcctgaatgcctgaataat(CA)6	44	19286699	19286742
chr10_3446	p2	(AC)6	12	19287801	19287812
chr10_3447	p2	(CA)23	46	19287971	19288016
chr10_3448	p2	(GT)8	16	19292360	19292375
chr10_3449	p2	(CA)8	16	19295253	19295268
chr10_3450	p2	(TG)6	12	19298048	19298059
chr10_3451	p2	(GT)6	12	19299140	19299151
chr10_3452	p2	(CA)9	18	19300112	19300129
chr10_3453	p2	(CA)9	18	19300244	19300261

chr10_3454	c	(CA)13t(AC)6	39	19300574	19300612
chr10_3455	p2	(CA)9	18	19300923	19300940
chr10_3456	p2	(GT)12	24	19301116	19301139
chr10_3457	p2	(CA)7	14	19305202	19305215
chr10_3458	p2	(CA)13	26	19307317	19307342
chr10_3459	c	(TC)7tatcc(CT)6	31	19311310	19311340
chr10_3460	p2	(GA)6	12	19313586	19313597
chr10_3461	p2	(CA)18	36	19314327	19314362
chr10_3462	p2	(AC)9	18	19315574	19315591
chr10_3463	p2	(AC)6	12	19315736	19315747
chr10_3464	p2	(AC)17	34	19316820	19316853
chr10_3465	p2	(CA)8	16	19316960	19316975
chr10_3466	p2	(TC)6	12	19317875	19317886
chr10_3467	p2	(TG)15	30	19323085	19323114
chr10_3468	p2	(AG)9	18	19323873	19323890
chr10_3469	p2	(CA)9	18	19324225	19324242
chr10_3470	p2	(TG)17	34	19325900	19325933
chr10_3471	p2	(CT)6	12	19327050	19327061
chr10_3472	p2	(GT)7	14	19329149	19329162
chr10_3473	p2	(CT)6	12	19333694	19333705
chr10_3474	c	ggtttgatgataggatgttttgctttgacagcaaacggtggggatggtgagc	127	19336357	19336483
chr10_3475	p2	(TG)12	24	19339431	19339454
chr10_3476	p2	(CA)10	20	19341037	19341056
chr10_3477	p2	(AC)13	26	19345150	19345175
chr10_3478	p2	(TG)10	20	19346987	19347006
chr10_3479	p2	(TG)10	20	19347146	19347165
chr10_3480	p2	(TG)9	18	19347331	19347348
chr10_3481	p2	(TC)6	12	19347748	19347759
chr10_3482	p2	(GT)6	12	19348113	19348124
chr10_3483	p2	(TG)18	36	19349272	19349307
chr10_3484	p2	(AC)10	20	19349467	19349486
chr10_3485	p2	(GT)24	48	19349600	19349647
chr10_3486	p2	(TG)6	12	19358105	19358116
chr10_3487	c	tacgatcatctctctccctgtctctgtgtttcagtatctgtcacaacacttatta	142	19359287	19359428
chr10_3488	c	atatatatagtgtatatattttagatgtccaatttctatgcttgtcttaaccatttg	139	19362090	19362228
chr10_3489	c	tatgaagatggacatatctggtataaatctgttgcacatcttttaactcacctt	125	19362341	19362465
chr10_3490	p2	(GT)12	24	19363293	19363316
chr10_3491	p2	(TG)6	12	19363532	19363543
chr10_3492	p2	(GT)15	30	19364448	19364477
chr10_3493	p2	(TG)6	12	19364587	19364598
chr10_3494	p2	(GT)16	32	19370752	19370783
chr10_3495	p2	(CA)8	16	19370901	19370916
chr10_3496	p2	(CA)16	32	19375958	19375989
chr10_3497	p2	(TG)8	16	19376151	19376166
chr10_3498	p2	(AC)6	12	19376360	19376371
chr10_3499	p2	(CA)9	18	19382737	19382754
chr10_3500	p2	(CT)16	32	19384173	19384204
chr10_3501	p2	(AC)8	16	19384402	19384417
chr10_3502	p2	(TC)8	16	19384739	19384754
chr10_3503	c	gcaagaggggagagggagagaaaaaagagagaacaagtgactgaaatg	104	19385003	19385106
chr10_3504	p2	(TG)24	48	19386399	19386446
chr10_3505	p2	(TG)7	14	19386548	19386561

chr10_3506	p2	(CA)7	14	19388630	19388643
chr10_3507	p2	(GT)20	40	19390492	19390531
chr10_3508	p2	(CA)7	14	19390659	19390672
chr10_3509	p2	(TG)15	30	19391203	19391232
chr10_3510	p2	(TG)17	34	19391457	19391490
chr10_3511	p2	(TG)6	12	19393399	19393410
chr10_3512	p2	(TG)8	16	19393787	19393802
chr10_3513	p2	(GT)14	28	19396266	19396293
chr10_3514	p2	(GA)7	14	19396638	19396651
chr10_3515	p2	(GT)18	36	19397223	19397258
chr10_3516	p2	(GT)13	26	19402433	19402458
chr10_3517	p2	(TG)6	12	19404818	19404829
chr10_3518	p2	(AC)12	24	19415782	19415805
chr10_3519	p2	(TC)6	12	19418007	19418018
chr10_3520	p2	(CA)21	42	19418158	19418199
chr10_3521	p2	(CA)13	26	19418921	19418946
chr10_3522	p2	(TC)16	32	19419135	19419166
chr10_3523	p2	(AC)12	24	19427664	19427687
chr10_3524	c	(TG)12c(GT)6	37	19431570	19431606
chr10_3525	p2	(GT)20	40	19446788	19446827
chr10_3526	p2	(CA)7	14	19453055	19453068
chr10_3527	p2	(AC)11	22	19453180	19453201
chr10_3528	p2	(GT)7	14	19453415	19453428
chr10_3529	p4	(TTCA)5	20	19455093	19455112
chr10_3530	p2	(AC)9	18	19466827	19466844
chr10_3531	p2	(TG)6	12	19470186	19470197
chr10_3532	c	(TC)12g(CT)8cact(CA)19cc(CA)7	99	19483711	19483809
chr10_3533	p2	(CA)10	20	19486970	19486989
chr10_3534	p2	(AC)16	32	19487282	19487313
chr10_3535	p2	(TG)6	12	19488346	19488357
chr10_3536	p2	(AC)13	26	19494060	19494085
chr10_3537	p2	(GT)7	14	19494479	19494492
chr10_3538	p2	(GT)7	14	19494704	19494717
chr10_3539	p2	(GT)11	22	19496182	19496203
chr10_3540	p3	(TCC)7	21	19501169	19501189
chr10_3541	p2	(AG)6	12	19501402	19501413
chr10_3542	p2	(TG)21	42	19513052	19513093
chr10_3543	p2	(AC)13	26	19515783	19515808
chr10_3544	c	(CA)11cg(CA)16	56	19526237	19526292
chr10_3545	c	ctacacgtgatgataatagtggcttactcactccaaaacacagcattatacaa	87	19534810	19534896
chr10_3546	p2	(CA)7	14	19535427	19535440
chr10_3547	p2	(GT)6	12	19535825	19535836
chr10_3548	p2	(TG)28	56	19538549	19538604
chr10_3549	p2	(GT)6	12	19538824	19538835
chr10_3550	p2	(TG)12	24	19539486	19539509
chr10_3551	p2	(TG)10	20	19539670	19539689
chr10_3552	c	(TG)6tttaata(AG)6	31	19546145	19546175
chr10_3553	p2	(CA)8	16	19556009	19556024
chr10_3554	p2	(AC)7	14	19558663	19558676
chr10_3555	p2	(CA)12	24	19561956	19561979
chr10_3556	p3	(TCC)5	15	19564004	19564018
chr10_3557	c	taaatgcatgaacagtgtttatgcgccttactcttgtttcatggcccttgagga	90	19564170	19564259

chr10_3558	p2	(GT)16	32	19566409	19566440
chr10_3559	p2	(TC)10	20	19571994	19572013
chr10_3560	p2	(CT)6	12	19574483	19574494
chr10_3561	p2	(TG)6	12	19578192	19578203
chr10_3562	p3	(CAG)5	15	19583863	19583877
chr10_3563	p2	(AC)10	20	19584747	19584766
chr10_3564	p2	(GT)33	66	19588042	19588107
chr10_3565	p2	(TA)6	12	19588398	19588409
chr10_3566	c	(GT)18gggtgt(TG)8	58	19604821	19604878
chr10_3567	p2	(TG)9	18	19616565	19616582
chr10_3568	p2	(TA)6	12	19617526	19617537
chr10_3569	p2	(AC)7	14	19631771	19631784
chr10_3570	p2	(TG)8	16	19636609	19636624
chr10_3571	p3	(TGA)5	15	19637252	19637266
chr10_3572	c	(TG)23tt(TG)10	68	19647497	19647564
chr10_3573	p3	(TTA)5	15	19648892	19648906
chr10_3574	p2	(TG)11	22	19650484	19650505
chr10_3575	p2	(AC)8	16	19655884	19655899
chr10_3576	p2	(GT)28	56	19656705	19656760
chr10_3577	p2	(TG)11	22	19659636	19659657
chr10_3578	p2	(CT)6	12	19668636	19668647
chr10_3579	p2	(GT)6	12	19675506	19675517
chr10_3580	p2	(AG)6	12	19680983	19680994
chr10_3581	p2	(GT)15	30	19681240	19681269
chr10_3582	p2	(TG)6	12	19695794	19695805
chr10_3583	p2	(CT)26	52	19696038	19696089
chr10_3584	p2	(GT)9	18	19696340	19696357
chr10_3585	p2	(GT)9	18	19702908	19702925
chr10_3586	p2	(TG)17	34	19703184	19703217
chr10_3587	p2	(AC)6	12	19703479	19703490
chr10_3588	c	(TA)6(AT)9	30	19704147	19704176
chr10_3589	p2	(GT)21	42	19705591	19705632
chr10_3590	p2	(CA)7	14	19706184	19706197
chr10_3591	p2	(AC)7	14	19712440	19712453
chr10_3592	c	aggcagatgatgaacagctgtcatgtggtttgcattgatgtatctgagagcg	135	19712733	19712867
chr10_3593	p2	(TG)6	12	19714379	19714390
chr10_3594	p3	(TGT)5	15	19717768	19717782
chr10_3595	p2	(GT)15	30	19722662	19722691
chr10_3596	p2	(TG)13	26	19723344	19723369
chr10_3597	p2	(GT)12	24	19723492	19723515
chr10_3598	p2	(TC)6	12	19727422	19727433
chr10_3599	p2	(TC)16	32	19728335	19728366
chr10_3600	p2	(TC)18	36	19733078	19733113
chr10_3601	p2	(TG)7	14	19733225	19733238
chr10_3602	p2	(TG)9	18	19736209	19736226
chr10_3603	p2	(CA)10	20	19737022	19737041
chr10_3604	c	(CA)6tgtgagtgccctgcctgcacatgtg(CA)7	51	19737469	19737519
chr10_3605	p2	(TG)22	44	19737662	19737705
chr10_3606	c	acactgtgatcaggttggccttggtcgcagagataaaaagaagggagaag	104	19742742	19742845
chr10_3607	p2	(GT)9	18	19744274	19744291
chr10_3608	p3	(AAT)7	21	19746629	19746649
chr10_3609	c	(TC)19ttgt(TC)7	56	19746926	19746981

chr10_3610	p2	(TG)13	26	19747805	19747830
chr10_3611	p2	(TG)6	12	19754403	19754414
chr10_3612	p2	(AC)9	18	19755947	19755964
chr10_3613	p2	(TG)8	16	19756298	19756313
chr10_3614	p2	(GT)6	12	19757234	19757245
chr10_3615	p2	(TC)11	22	19759984	19760005
chr10_3616	p2	(CA)7	14	19761227	19761240
chr10_3617	p3	(CTG)5	15	19761680	19761694
chr10_3618	p2	(AC)6	12	19764300	19764311
chr10_3619	p2	(TG)8	16	19764480	19764495
chr10_3620	p2	(AT)7	14	19764605	19764618
chr10_3621	p2	(GT)6	12	19766071	19766082
chr10_3622	p2	(GT)9	18	19768926	19768943
chr10_3623	p2	(AC)9	18	19774626	19774643
chr10_3624	c	(TG)15tt(TG)13(AG)8	74	19775934	19776007
chr10_3625	p2	(AC)9	18	19777396	19777413
chr10_3626	p2	(GT)8	16	19778741	19778756
chr10_3627	p2	(CA)10	20	19779865	19779884
chr10_3628	p2	(CT)8	16	19780385	19780400
chr10_3629	p2	(AC)7	14	19794167	19794180
chr10_3630	p3	(CAC)6	18	19811293	19811310
chr10_3631	p2	(AC)8	16	19818124	19818139
chr10_3632	p2	(GT)6	12	19818465	19818476
chr10_3633	p2	(TG)9	18	19819986	19820003
chr10_3634	p2	(AC)8	16	19820812	19820827
chr10_3635	p2	(GT)6	12	19823816	19823827
chr10_3636	p2	(TG)6	12	19836562	19836573
chr10_3637	p2	(TG)25	50	19851403	19851452
chr10_3638	p3	(GAG)5	15	19853384	19853398
chr10_3639	p2	(TG)6	12	19855355	19855366
chr10_3640	p3	(ATA)5	15	19856220	19856234
chr10_3641	p3	(TCA)8	24	19856856	19856879
chr10_3642	p3	(AGG)5	15	19874268	19874282
chr10_3643	p3	(AGA)5	15	19881676	19881690
chr10_3644	p2	(GT)13	26	19884132	19884157
chr10_3645	p2	(TG)18	36	19886111	19886146
chr10_3646	p2	(TG)18	36	19889009	19889044
chr10_3647	p2	(CT)6	12	19889838	19889849
chr10_3648	p2	(TG)6	12	19896103	19896114
chr10_3649	p2	(TG)13	26	19897207	19897232
chr10_3650	p2	(GT)12	24	19898299	19898322
chr10_3651	p3	(CAT)5	15	19901364	19901378
chr10_3652	p2	(CA)7	14	19903364	19903377
chr10_3653	p3	(TCT)5	15	19905076	19905090
chr10_3654	p2	(GT)13	26	19913533	19913558
chr10_3655	p3	(TAT)6	18	19918862	19918879
chr10_3656	p3	(GAA)6	18	19923507	19923524
chr10_3657	p2	(GT)6	12	19927033	19927044
chr10_3658	p2	(GT)7	14	19927371	19927384
chr10_3659	p2	(GT)11	22	19928502	19928523
chr10_3660	p2	(GT)7	14	19928824	19928837
chr10_3661	p2	(GT)12	24	19929582	19929605

chr10_3662	p3	(TTA)5	15	19932258	19932272
chr10_3663	p2	(AC)9	18	19935082	19935099
chr10_3664	p2	(TG)6	12	19935241	19935252
chr10_3665	p2	(TG)6	12	19943030	19943041
chr10_3666	p2	(GT)12	24	19947103	19947126
chr10_3667	p3	(GAG)5	15	19949957	19949971
chr10_3668	p2	(AC)13	26	19956939	19956964
chr10_3669	p2	(GT)13	26	19962929	19962954
chr10_3670	p3	(CTC)5	15	19971590	19971604
chr10_3671	p2	(TC)11	22	19974763	19974784
chr10_3672	p2	(GT)10	20	19982130	19982149
chr10_3673	p2	(TG)6	12	19986748	19986759
chr10_3674	c	(CATC)9tgtgcccacacagcgt(TC)6	64	19990907	19990970
chr10_3675	p2	(TC)7	14	19991345	19991358
chr10_3676	p2	(TG)16	32	19998469	19998500
chr10_3677	p3	(CAT)5	15	20006684	20006698
chr10_3678	p2	(TG)9	18	20012428	20012445
chr10_3679	p3	(TGT)5	15	20013248	20013262
chr10_3680	p2	(AC)17	34	20021078	20021111
chr10_3681	p2	(GT)18	36	20025793	20025828
chr10_3682	p2	(TG)12	24	20046666	20046689
chr10_3683	p3	(ATA)6	18	20048840	20048857
chr10_3684	p3	(TGA)10	30	20049205	20049234
chr10_3685	p2	(GT)11	22	20059135	20059156
chr10_3686	p3	(ACT)5	15	20062956	20062970
chr10_3687	p2	(GT)20	40	20066352	20066391
chr10_3688	p2	(AC)7	14	20070468	20070481
chr10_3689	p2	(GT)11	22	20075102	20075123
chr10_3690	p2	(TC)6	12	20079161	20079172
chr10_3691	p2	(TA)8	16	20084277	20084292
chr10_3692	p4	(TGAT)6	24	20085503	20085526
chr10_3693	p3	(TTA)5	15	20112476	20112490
chr10_3694	p2	(GT)15	30	20113943	20113972
chr10_3695	p2	(AC)14	28	20115610	20115637
chr10_3696	p2	(CT)10	20	20120184	20120203
chr10_3697	p3	(GAG)5	15	20128336	20128350
chr10_3698	p2	(TG)27	54	20136275	20136328
chr10_3699	p3	(GAG)8	24	20141413	20141436
chr10_3700	p2	(AT)7	14	20160303	20160316
chr10_3701	p2	(AT)6	12	20162575	20162586
chr10_3702	c	tctaacagacaaaaactacagttcctctggtttgtggttctttgttttcagtgac	103	20169018	20169120
chr10_3703	p3	(TGA)6	18	20172681	20172698
chr10_3704	p2	(AG)19	38	20174616	20174653
chr10_3705	p2	(AC)12	24	20175942	20175965
chr10_3706	p2	(TG)7	14	20185499	20185512
chr10_3707	p2	(CA)6	12	20200923	20200934
chr10_3708	p4	(GACA)5	20	20203602	20203621
chr10_3709	p2	(CA)24	48	20215599	20215646
chr10_3710	p2	(GT)9	18	20216795	20216812
chr10_3711	p2	(AC)6	12	20217875	20217886
chr10_3712	p2	(GT)7	14	20218511	20218524
chr10_3713	p2	(TG)7	14	20220611	20220624

chr10_3714	p2	(GA)7	14	20223838	20223851
chr10_3715	c	(CA)6g(AC)10	33	20238532	20238564
chr10_3716	p3	(ATA)5	15	20247558	20247572
chr10_3717	p2	(CA)14	28	20259897	20259924
chr10_3718	p3	(AGG)7	21	20261506	20261526
chr10_3719	p2	(AC)11	22	20263194	20263215
chr10_3720	p2	(AC)9	18	20263350	20263367
chr10_3721	c	gtagatgacatattctgcctttcctctcccacagcagctcgttttatcactttga	159	20263582	20263740
chr10_3722	c	(AC)6at(AC)13	40	20263887	20263926
chr10_3723	c	(GT)13(GA)9	44	20266607	20266650
chr10_3724	p3	(GAG)5	15	20267114	20267128
chr10_3725	p2	(GA)6	12	20267754	20267765
chr10_3726	p3	(ATT)5	15	20277172	20277186
chr10_3727	p2	(AC)17	34	20290854	20290887
chr10_3728	p2	(AC)8	16	20303497	20303512
chr10_3729	p2	(GT)12	24	20311440	20311463
chr10_3730	p2	(GT)9	18	20313090	20313107
chr10_3731	c	tgtggccaccatcttgtctgtgtgtgtttctgtttgccgtctttgtgactcgagg	149	20314313	20314461
chr10_3732	p2	(AG)6	12	20316202	20316213
chr10_3733	p3	(AGG)5	15	20316411	20316425
chr10_3734	p2	(GA)14	28	20317129	20317156
chr10_3735	p2	(AC)13	26	20317805	20317830
chr10_3736	p2	(CA)8	16	20318933	20318948
chr10_3737	p2	(GT)7	14	20321105	20321118
chr10_3738	c	(TG)8aga(GT)6	31	20322853	20322883
chr10_3739	p2	(TG)13	26	20325546	20325571
chr10_3740	p2	(TG)9	18	20325682	20325699
chr10_3741	p3	(AGG)5	15	20325950	20325964
chr10_3742	p2	(TG)9	18	20326656	20326673
chr10_3743	p2	(CA)6	12	20327290	20327301
chr10_3744	p3	(TCC)6	18	20327929	20327946
chr10_3745	c	acttttactacactgatcggcccttcacacaccatctaacatcacacttcttgt	141	20329480	20329620
chr10_3746	p2	(CA)14	28	20334882	20334909
chr10_3747	p3	(CCT)7	21	20337142	20337162
chr10_3748	p3	(AGG)5	15	20337918	20337932
chr10_3749	p2	(AC)10	20	20343780	20343799
chr10_3750	p3	(TTC)5	15	20346043	20346057
chr10_3751	p2	(TG)7	14	20352990	20353003
chr10_3752	p2	(TC)6	12	20353790	20353801
chr10_3753	p3	(TAT)6	18	20360588	20360605
chr10_3754	p2	(CA)7	14	20361829	20361842
chr10_3755	p2	(TG)17	34	20364170	20364203
chr10_3756	p2	(TG)17	34	20364523	20364556
chr10_3757	p2	(GT)7	14	20364681	20364694
chr10_3758	p2	(TG)6	12	20369224	20369235
chr10_3759	p2	(AC)14	28	20369380	20369407
chr10_3760	c	(TG)7(AG)14	42	20371117	20371158
chr10_3761	p2	(TG)10	20	20372004	20372023
chr10_3762	p2	(CA)9	18	20374863	20374880
chr10_3763	p2	(TG)12	24	20375839	20375862
chr10_3764	p2	(AC)9	18	20376077	20376094
chr10_3765	c	(CA)7cg(CA)6	28	20377264	20377291

chr10_3766	p2	(AC)15	30	20383303	20383332
chr10_3767	p2	(GT)6	12	20386188	20386199
chr10_3768	p2	(GT)9	18	20386439	20386456
chr10_3769	p2	(GT)6	12	20386674	20386685
chr10_3770	c	tgaactgtgtgtaatataaaccagctcagggactttaacatgagcctctagg	162	20386800	20386961
chr10_3771	p3	(CTC)6	18	20388222	20388239
chr10_3772	p2	(TG)7	14	20390808	20390821
chr10_3773	p2	(CA)7	14	20402229	20402242
chr10_3774	p3	(ACA)8	24	20403143	20403166
chr10_3775	p2	(AC)6	12	20403554	20403565
chr10_3776	p3	(TTA)5	15	20407203	20407217
chr10_3777	p2	(GT)16	32	20409879	20409910
chr10_3778	c	(TG)15(AG)12	54	20410058	20410111
chr10_3779	p2	(TG)11	22	20413672	20413693
chr10_3780	p2	(TG)17	34	20414829	20414862
chr10_3781	p2	(GT)6	12	20415159	20415170
chr10_3782	p2	(TG)10	20	20415778	20415797
chr10_3783	p4	(GAGG)5	20	20416135	20416154
chr10_3784	p2	(GT)21	42	20416385	20416426
chr10_3785	p2	(CT)6	12	20424526	20424537
chr10_3786	p2	(TG)6	12	20425353	20425364
chr10_3787	p2	(TG)8	16	20425684	20425699
chr10_3788	p2	(CA)14	28	20436099	20436126
chr10_3789	p2	(CA)23	46	20436292	20436337
chr10_3790	c	(CT)12cacact(CA)16	62	20443043	20443104
chr10_3791	p2	(GT)6	12	20443412	20443423
chr10_3792	p2	(TG)6	12	20445619	20445630
chr10_3793	p2	(AG)6	12	20446886	20446897
chr10_3794	p3	(GAA)5	15	20447053	20447067
chr10_3795	p2	(CA)12	24	20451948	20451971
chr10_3796	p2	(AC)6	12	20452082	20452093
chr10_3797	p2	(GT)10	20	20454832	20454851
chr10_3798	p2	(TG)6	12	20457610	20457621
chr10_3799	p2	(TG)6	12	20457775	20457786
chr10_3800	p3	(GGA)5	15	20458503	20458517
chr10_3801	p2	(GT)9	18	20458803	20458820
chr10_3802	p2	(TG)19	38	20459175	20459212
chr10_3803	p2	(AT)7	14	20460489	20460502
chr10_3804	p2	(CA)7	14	20461458	20461471
chr10_3805	p2	(TG)12	24	20461743	20461766
chr10_3806	p2	(CT)22	44	20463493	20463536
chr10_3807	p2	(CT)6	12	20467163	20467174
chr10_3808	p2	(TG)9	18	20468432	20468449
chr10_3809	c	agttgaattatgtggggccattcatcttttctgtcgtctgtgatcatttatgtctct	101	20471750	20471850
chr10_3810	p2	(TG)6	12	20478481	20478492
chr10_3811	p2	(AC)20	40	20486862	20486901
chr10_3812	p2	(AG)10	20	20487530	20487549
chr10_3813	p3	(GAG)5	15	20491422	20491436
chr10_3814	c	(TC)7(AC)23	60	20495731	20495790
chr10_3815	p2	(CA)11	22	20500150	20500171
chr10_3816	p2	(AC)10	20	20500419	20500438
chr10_3817	p4	(TTGT)5	20	20502753	20502772

chr10_3818	p2	(AC)16	32	20505143	20505174
chr10_3819	p2	(TG)17	34	20515729	20515762
chr10_3820	p2	(AC)27	54	20517643	20517696
chr10_3821	p2	(CT)6	12	20520039	20520050
chr10_3822	p2	(AG)9	18	20523089	20523106
chr10_3823	p2	(TG)16	32	20527025	20527056
chr10_3824	p2	(CT)6	12	20527576	20527587
chr10_3825	p2	(GA)6	12	20529571	20529582
chr10_3826	p2	(AC)7	14	20536648	20536661
chr10_3827	p2	(GT)7	14	20540382	20540395
chr10_3828	p3	(AGA)5	15	20542659	20542673
chr10_3829	p2	(CA)6	12	20549100	20549111
chr10_3830	p3	(ATT)5	15	20549831	20549845
chr10_3831	p3	(TCC)5	15	20555883	20555897
chr10_3832	p2	(CA)7	14	20568870	20568883
chr10_3833	c	gtgaagagacattcttcagcatggagaatttcttggtcactgaactgccgctc	95	20575664	20575758
chr10_3834	p2	(GT)11	22	20575879	20575900
chr10_3835	p2	(TG)14	28	20577082	20577109
chr10_3836	p4	(ATGG)5	20	20578899	20578918
chr10_3837	p2	(AG)6	12	20584851	20584862
chr10_3838	p3	(TGT)10	30	20600747	20600776
chr10_3839	p3	(AGC)7	21	20605983	20606003
chr10_3840	p3	(TCA)5	15	20607579	20607593
chr10_3841	p3	(CTC)6	18	20607867	20607884
chr10_3842	p3	(TCC)5	15	20609523	20609537
chr10_3843	c	gtgctgaatggagatgaaagggc(GT)25ttgttacagtgttgtatgtgtgc	245	20611136	20611380
chr10_3844	p2	(GT)18	36	20611587	20611622
chr10_3845	p2	(TC)6	12	20613719	20613730
chr10_3846	p2	(TG)12	24	20616679	20616702
chr10_3847	p2	(GT)7	14	20617072	20617085
chr10_3848	p3	(TCC)6	18	20617679	20617696
chr10_3849	p2	(GT)10	20	20626433	20626452
chr10_3850	p3	(GTT)5	15	20649677	20649691
chr10_3851	p3	(TAT)16	48	20664266	20664313
chr10_3852	p2	(TG)6	12	20677302	20677313
chr10_3853	p2	(GT)6	12	20677663	20677674
chr10_3854	p3	(CAT)7	21	20680002	20680022
chr10_3855	p2	(AC)11	22	20680675	20680696
chr10_3856	p2	(AC)22	44	20680879	20680922
chr10_3857	c	(TG)9(AG)10	38	20681370	20681407
chr10_3858	p2	(AC)6	12	20685903	20685914
chr10_3859	p2	(CA)8	16	20686511	20686526
chr10_3860	p2	(AG)8	16	20686667	20686682
chr10_3861	p3	(GAG)5	15	20689475	20689489
chr10_3862	p2	(AC)9	18	20690244	20690261
chr10_3863	c	(GT)7gagagt(GA)6	32	20690632	20690663
chr10_3864	p2	(TG)6	12	20701810	20701821
chr10_3865	p2	(AC)14	28	20703498	20703525
chr10_3866	c	gccttctcctgtttaatacctgttgctcctgggagaaaggcctgttccgcatac	158	20712080	20712237
chr10_3867	p2	(CA)19	38	20712590	20712627
chr10_3868	p2	(AC)6	12	20717451	20717462
chr10_3869	p2	(TG)6	12	20721532	20721543

chr10_3870	p2	(TG)10	20	20722839	20722858
chr10_3871	p2	(TC)6	12	20723024	20723035
chr10_3872	p2	(TG)18	36	20724079	20724114
chr10_3873	p2	(CA)8	16	20726299	20726314
chr10_3874	p2	(AC)16	32	20733805	20733836
chr10_3875	p2	(AC)23	46	20734316	20734361
chr10_3876	p2	(AG)11	22	20737232	20737253
chr10_3877	p2	(GT)8	16	20738769	20738784
chr10_3878	p2	(TC)6	12	20740472	20740483
chr10_3879	p2	(TG)25	50	20742741	20742790
chr10_3880	p2	(GT)9	18	20743952	20743969
chr10_3881	p2	(AC)22	44	20744454	20744497
chr10_3882	p2	(CT)6	12	20749342	20749353
chr10_3883	p3	(AAT)5	15	20751403	20751417
chr10_3884	p2	(TC)7	14	20758593	20758606
chr10_3885	p2	(AC)6	12	20761018	20761029
chr10_3886	p2	(TG)9	18	20763693	20763710
chr10_3887	p2	(TC)17	34	20767633	20767666
chr10_3888	p2	(TA)6	12	20784122	20784133
chr10_3889	p2	(TG)6	12	20796760	20796771
chr10_3890	p2	(AC)19	38	20797741	20797778
chr10_3891	c	(CTC)7ctacgactacgactactg(CTA)5ctccta(CTC)5	75	20809078	20809152
chr10_3892	p2	(TG)6	12	20817966	20817977
chr10_3893	p2	(GT)6	12	20823002	20823013
chr10_3894	p2	(GT)10	20	20823360	20823379
chr10_3895	p2	(GA)6	12	20824428	20824439
chr10_3896	p3	(TAA)9	27	20827749	20827775
chr10_3897	c	tgggtgtgcacatttagccttttgttttgtgtaacttatttttgcatgtagacatgt	122	20831592	20831713
chr10_3898	p2	(GT)9	18	20831995	20832012
chr10_3899	p2	(GT)8	16	20833592	20833607
chr10_3900	p2	(TG)13	26	20835008	20835033
chr10_3901	p2	(CT)6	12	20837139	20837150
chr10_3902	p2	(AC)6	12	20837255	20837266
chr10_3903	p3	(ATA)5	15	20842784	20842798
chr10_3904	c	(AAG)5aggcagaaaacaaca(AAG)7	51	20843418	20843468
chr10_3905	c	tgggtgtgcacgtttagccttttgttttgtgtaacttatttttgcatgtagacatgt	100	20853574	20853673
chr10_3906	p2	(TG)22	44	20854319	20854362
chr10_3907	p3	(ATT)6	18	20860767	20860784
chr10_3908	p4	(CCAT)5	20	20881858	20881877
chr10_3909	p2	(GT)7	14	20892529	20892542
chr10_3910	p2	(AC)20	40	20892755	20892794
chr10_3911	p2	(CA)13	26	20893711	20893736
chr10_3912	p2	(TC)6	12	20894453	20894464
chr10_3913	p2	(TG)6	12	20894611	20894622
chr10_3914	p3	(GAT)8	24	20916929	20916952
chr10_3915	p2	(GT)6	12	20919434	20919445
chr10_3916	p3	(ATG)10	30	20922131	20922160
chr10_3917	p2	(TG)8	16	20922852	20922867
chr10_3918	p2	(CA)13	26	20923904	20923929
chr10_3919	c	tttggaggggaaagaacacaagaagaactgtgatcactgtttcagagaag	111	20925566	20925676
chr10_3920	p3	(TTA)5	15	20929840	20929854
chr10_3921	p2	(GT)6	12	20930201	20930212

chr10_3922	p2	(TG)10	20	20930501	20930520
chr10_3923	p2	(GT)7	14	20931291	20931304
chr10_3924	p2	(TG)25	50	20933832	20933881
chr10_3925	p2	(TC)8	16	20936518	20936533
chr10_3926	p2	(AC)6	12	20941274	20941285
chr10_3927	p2	(TG)25	50	20945474	20945523
chr10_3928	p2	(TG)15	30	20945632	20945661
chr10_3929	p2	(AC)12	24	20955228	20955251
chr10_3930	c	(AC)17tcacactcacactcaca(CT)9(CA)12	93	20955470	20955562
chr10_3931	p2	(TG)8	16	20957504	20957519
chr10_3932	p2	(TG)6	12	20958863	20958874
chr10_3933	p2	(TG)20	40	20960260	20960299
chr10_3934	c	(TG)9cttgacctctgctgacctttt(TG)10	59	20960514	20960572
chr10_3935	p2	(GT)7	14	20962965	20962978
chr10_3936	p2	(GT)8	16	20963806	20963821
chr10_3937	p2	(TG)7	14	20969684	20969697
chr10_3938	p2	(TG)17	34	20976587	20976620
chr10_3939	p2	(GT)10	20	20977469	20977488
chr10_3940	p2	(AC)7	14	20977634	20977647
chr10_3941	p2	(GT)16	32	20978267	20978298
chr10_3942	p2	(AC)10	20	20983012	20983031
chr10_3943	p2	(AC)12	24	20983238	20983261
chr10_3944	p2	(AC)8	16	20983664	20983679
chr10_3945	p2	(GT)17	34	20983881	20983914
chr10_3946	p2	(TC)7	14	20984901	20984914
chr10_3947	p2	(GT)21	42	20989182	20989223
chr10_3948	p3	(CTC)6	18	20990195	20990212
chr10_3949	p2	(GT)8	16	20990626	20990641
chr10_3950	p2	(TC)6	12	20992125	20992136
chr10_3951	p2	(TG)14	28	20992358	20992385
chr10_3952	p2	(AC)7	14	20996734	20996747
chr10_3953	p2	(TG)6	12	20999363	20999374
chr10_3954	p2	(AC)6	12	21011895	21011906
chr10_3955	p2	(AC)7	14	21012233	21012246
chr10_3956	p2	(TC)19	38	21019081	21019118
chr10_3957	c	[illegible]AC)8atatatatatactgagggtgacttgtataggcagccagtggg(GA)[illegible]	88	21019475	21019562
chr10_3958	p3	(TAT)5	15	21025336	21025350
chr10_3959	p2	(CT)8	16	21028760	21028775
chr10_3960	p2	(TG)10	20	21030475	21030494
chr10_3961	p2	(GA)8	16	21030715	21030730
chr10_3962	p2	(GT)7	14	21034592	21034605
chr10_3963	c	(TC)8acact(CA)10	41	21037036	21037076
chr10_3964	p2	(TG)6	12	21040647	21040658
chr10_3965	p2	(GT)6	12	21040910	21040921
chr10_3966	p2	(TG)6	12	21041048	21041059
chr10_3967	p2	(GA)9	18	21043354	21043371
chr10_3968	p2	(AC)8	16	21046068	21046083
chr10_3969	p2	(GT)13	26	21057876	21057901
chr10_3970	p2	(GT)7	14	21059915	21059928
chr10_3971	p2	(AC)6	12	21063741	21063752
chr10_3972	p2	(GA)7	14	21064895	21064908
chr10_3973	p2	(TC)7	14	21066496	21066509

chr10_3974	p2	(TG)6	12	21072549	21072560
chr10_3975	p2	(AC)6	12	21088639	21088650
chr10_3976	p2	(CA)8	16	21089086	21089101
chr10_3977	p2	(GT)17	34	21089281	21089314
chr10_3978	p2	(TC)8	16	21092606	21092621
chr10_3979	p2	(GT)9	18	21092806	21092823
chr10_3980	p2	(AC)6	12	21093052	21093063
chr10_3981	p2	(GT)20	40	21094078	21094117
chr10_3982	p2	(AC)6	12	21096844	21096855
chr10_3983	p2	(TG)6	12	21097944	21097955
chr10_3984	p2	(AC)27	54	21098153	21098206
chr10_3985	p2	(AC)6	12	21102230	21102241
chr10_3986	p2	(GA)7	14	21107802	21107815
chr10_3987	c	(TCTA)5t(AG)10aa(AG)7	57	21109576	21109632
chr10_3988	c	(CG)7(CA)20	54	21110490	21110543
chr10_3989	c	(TA)6(TG)9	30	21112041	21112070
chr10_3990	p2	(TG)7	14	21112355	21112368
chr10_3991	p2	(AC)33	66	21117479	21117544
chr10_3992	p2	(TG)7	14	21119323	21119336
chr10_3993	p2	(TG)6	12	21119523	21119534
chr10_3994	p2	(CA)17	34	21120382	21120415
chr10_3995	p2	(AC)6	12	21124416	21124427
chr10_3996	p2	(TG)6	12	21131847	21131858
chr10_3997	p2	(CA)10	20	21133874	21133893
chr10_3998	p2	(AC)7	14	21135004	21135017
chr10_3999	p2	(TC)6	12	21136683	21136694
chr10_4000	c	(TG)28cgcaagacgc(AG)6	78	21139744	21139821
chr10_4001	c	(AC)10g(CA)7	35	21139936	21139970
chr10_4002	p2	(CA)19	38	21142363	21142400
chr10_4003	p2	(CT)6	12	21142746	21142757
chr10_4004	c	gttcacctccagaagtgcctttatgctgctgacagactggaaacagttgtct	98	21145949	21146046
chr10_4005	p2	(TG)7	14	21147070	21147083
chr10_4006	p3	(CAT)7	21	21147872	21147892
chr10_4007	p2	(TG)8	16	21152669	21152684
chr10_4008	p2	(AC)7	14	21155473	21155486
chr10_4009	p2	(CA)20	40	21159049	21159088
chr10_4010	p2	(TG)10	20	21161567	21161586
chr10_4011	c	ttcagggggctaaggcaaagagagggggaaactcttttccaaagacgtca	129	21163566	21163694
chr10_4012	p2	(GT)9	18	21171342	21171359
chr10_4013	p2	(CA)13	26	21172856	21172881
chr10_4014	p2	(AC)15	30	21177106	21177135
chr10_4015	p2	(CA)14	28	21182866	21182893
chr10_4016	p2	(AC)10	20	21186855	21186874
chr10_4017	p2	(GT)13	26	21189024	21189049
chr10_4018	p2	(TG)14	28	21189495	21189522
chr10_4019	p2	(TG)8	16	21192456	21192471
chr10_4020	p2	(AC)14	28	21196183	21196210
chr10_4021	c	(CA)6ctcct(CA)33	83	21197125	21197207
chr10_4022	p2	(AC)6	12	21201583	21201594
chr10_4023	p2	(GT)7	14	21205609	21205622
chr10_4024	p2	(AC)13	26	21205901	21205926
chr10_4025	p2	(GT)8	16	21211000	21211015

chr10_4026	p2	(GT)16	32	21224187	21224218
chr10_4027	c	(AC)15(AG)29	88	21224504	21224591
chr10_4028	p2	(CA)18	36	21228468	21228503
chr10_4029	p2	(AC)6	12	21228711	21228722
chr10_4030	p2	(AG)24	48	21229142	21229189
chr10_4031	p2	(AC)9	18	21230477	21230494
chr10_4032	p2	(AC)17	34	21230681	21230714
chr10_4033	p2	(CA)8	16	21232967	21232982
chr10_4034	p4	(ATAG)14	56	21242888	21242943
chr10_4035	p2	(AC)17	34	21243106	21243139
chr10_4036	p2	(TG)12	24	21243354	21243377
chr10_4037	p2	(GT)8	16	21243644	21243659
chr10_4038	p2	(TG)6	12	21247049	21247060
chr10_4039	p2	(CA)11	22	21250730	21250751
chr10_4040	p2	(CA)17	34	21266344	21266377
chr10_4041	c	(GT)12atat(AC)6	40	21268731	21268770
chr10_4042	p4	(AAAT)6	24	21271169	21271192
chr10_4043	p2	(AC)7	14	21271751	21271764
chr10_4044	p2	(TC)6	12	21287640	21287651
chr10_4045	p3	(AAT)5	15	21289177	21289191
chr10_4046	p3	(TTG)10	30	21290607	21290636
chr10_4047	p2	(GT)10	20	21295601	21295620
chr10_4048	p2	(AC)11	22	21298445	21298466
chr10_4049	p2	(CA)13	26	21305201	21305226
chr10_4050	p2	(CA)11	22	21306454	21306475
chr10_4051	p2	(CA)6	12	21306686	21306697
chr10_4052	p3	(ATA)5	15	21335299	21335313
chr10_4053	p3	(ATA)5	15	21336143	21336157
chr10_4054	p4	(CAGA)6	24	21348048	21348071
chr10_4055	p2	(TG)6	12	21349896	21349907
chr10_4056	p2	(GT)13	26	21351547	21351572
chr10_4057	c*	(AG)6(GAG)6*	27	21358962	21358988
chr10_4058	p3	(ATT)9	27	21375075	21375101
chr10_4059	p2	(TG)17	34	21375750	21375783
chr10_4060	p2	(AC)10	20	21376850	21376869
chr10_4061	p2	(GA)6	12	21381702	21381713
chr10_4062	p2	(GT)15	30	21398408	21398437
chr10_4063	p2	(CA)9	18	21443335	21443352
chr10_4064	p2	(GT)16	32	21447349	21447380
chr10_4065	p2	(TG)21	42	21462047	21462088
chr10_4066	p3	(CCT)5	15	21492314	21492328
chr10_4067	p2	(GA)6	12	21498417	21498428
chr10_4068	p2	(CA)11	22	21498578	21498599
chr10_4069	p2	(AC)7	14	21498712	21498725
chr10_4070	p4	(ATCC)5	20	21504263	21504282
chr10_4071	p2	(TG)11	22	21508511	21508532
chr10_4072	p2	(TG)8	16	21513235	21513250
chr10_4073	p2	(AC)10	20	21523851	21523870
chr10_4074	p2	(GT)11	22	21537254	21537275
chr10_4075	p2	(CA)7	14	21544629	21544642
chr10_4076	p2	(AG)6	12	21545177	21545188
chr10_4077	p2	(CA)8	16	21556718	21556733

chr10_4078	p2	(TG)6	12	21559802	21559813
chr10_4079	p2	(GT)8	16	21566047	21566062
chr10_4080	p2	(AC)6	12	21577422	21577433
chr10_4081	p4	(TCCA)5	20	21590258	21590277
chr10_4082	p3	(GCT)6	18	21594804	21594821
chr10_4083	p2	(CA)6	12	21603531	21603542
chr10_4084	p2	(GT)13	26	21617838	21617863
chr10_4085	p2	(AC)11	22	21623241	21623262
chr10_4086	c	6tctgaaagagagagcgagggatgcagggactgtgtgtgttgcctgtata(	75	21625727	21625801
chr10_4087	p2	(GT)9	18	21633188	21633205
chr10_4088	p5	(TTAGC)6	30	21649137	21649166
chr10_4089	p2	(CA)6	12	21655141	21655152
chr10_4090	p2	(GT)6	12	21656618	21656629
chr10_4091	p2	(AC)6	12	21666998	21667009
chr10_4092	p3	(TGG)5	15	21678128	21678142
chr10_4093	p2	(GT)6	12	21685249	21685260
chr10_4094	p2	(GT)7	14	21692045	21692058
chr10_4095	p3	(TAT)5	15	21698107	21698121
chr10_4096	p2	(TG)10	20	21705372	21705391
chr10_4097	p3	(ATT)5	15	21710816	21710830
chr10_4098	p2	(TC)6	12	21718175	21718186
chr10_4099	p2	(AG)8	16	21754026	21754041
chr10_4100	p2	(CA)6	12	21760260	21760271
chr10_4101	p2	(CA)7	14	21764645	21764658
chr10_4102	p3	(TTA)9	27	21767957	21767983
chr10_4103	p2	(AC)13	26	21772901	21772926
chr10_4104	p3	(ATG)5	15	21778475	21778489
chr10_4105	p5	(GAGAG)6	30	21784778	21784807
chr10_4106	p4	(AATG)5	20	21799294	21799313
chr10_4107	p2	(TA)7	14	21830432	21830445
chr10_4108	p3	(CAA)8	24	21834914	21834937
chr10_4109	c	(TC)13(AC)7	40	21844223	21844262
chr10_4110	p3	(CCA)6	18	21844588	21844605
chr10_4111	p2	(TG)13	26	21850727	21850752
chr10_4112	p2	(TA)7	14	21852237	21852250
chr10_4113	p2	(GT)12	24	21853762	21853785
chr10_4114	p2	(CA)9	18	21854588	21854605
chr10_4115	p2	(CA)6	12	21855078	21855089
chr10_4116	p2	(AG)9	18	21860497	21860514
chr10_4117	p2	(AC)14	28	21869460	21869487
chr10_4118	p2	(CA)12	24	21879329	21879352
chr10_4119	p3	(GAG)6	18	21882157	21882174
chr10_4120	p2	(CA)17	34	21894551	21894584
chr10_4121	p2	(AC)7	14	21902735	21902748
chr10_4122	p3	(ATA)5	15	21929079	21929093
chr10_4123	p4	(ATAG)5	20	21940201	21940220
chr10_4124	c	ıgatggctacctcggttctgtaccagagacaaattccctgtgtgtgaaacata	129	21950090	21950218
chr10_4125	p2	(AC)14	28	21992777	21992804
chr10_4126	p3	(ATA)6	18	21996534	21996551
chr10_4127	p2	(GT)6	12	22007175	22007186
chr10_4128	p2	(TG)7	14	22007780	22007793
chr10_4129	p3	(AAT)6	18	22008340	22008357

chr10_4130	p3	(AAT)6	18	22010323	22010340
chr10_4131	p2	(AG)9	18	22011088	22011105
chr10_4132	p2	(TG)6	12	22012719	22012730
chr10_4133	p2	(CT)6	12	22016496	22016507
chr10_4134	p3	(TAA)5	15	22023261	22023275
chr10_4135	p2	(AC)8	16	22025105	22025120
chr10_4136	p4	(TAGA)7	28	22032665	22032692
chr10_4137	p2	(GA)8	16	22036541	22036556
chr10_4138	p2	(AC)7	14	22038175	22038188
chr10_4139	p2	(GA)11	22	22038874	22038895
chr10_4140	p3	(TGA)8	24	22042769	22042792
chr10_4141	p2	(CA)6	12	22043193	22043204
chr10_4142	p2	(GT)14	28	22047621	22047648
chr10_4143	p2	(TG)7	14	22051478	22051491
chr10_4144	c	(AGAT)18at(AC)11	96	22053288	22053383
chr10_4145	p2	(AT)6	12	22062824	22062835
chr10_4146	p2	(GT)10	20	22066464	22066483
chr10_4147	p3	(CTT)12	36	22076349	22076384
chr10_4148	p2	(TC)6	12	22081032	22081043
chr10_4149	p2	(CA)6	12	22091133	22091144
chr10_4150	p2	(TG)6	12	22091561	22091572
chr10_4151	p2	(TG)12	24	22098959	22098982
chr10_4152	p2	(GT)6	12	22121179	22121190
chr10_4153	p2	(CA)7	14	22123226	22123239
chr10_4154	p2	(TC)6	12	22132514	22132525
chr10_4155	p2	(AG)6	12	22140826	22140837
chr10_4156	p2	(TG)14	28	22146801	22146828
chr10_4157	p2	(AC)6	12	22150288	22150299
chr10_4158	c	(AT)6g(TA)6	25	22156910	22156934
chr10_4159	p2	(CA)31	62	22160633	22160694
chr10_4160	p2	(GT)6	12	22163589	22163600
chr10_4161	p2	(GT)11	22	22169297	22169318
chr10_4162	p2	(TG)7	14	22173464	22173477
chr10_4163	p2	(AC)19	38	22174179	22174216
chr10_4164	c	(GT)16gcgc(GT)6	48	22176941	22176988
chr10_4165	p2	(CT)6	12	22180074	22180085
chr10_4166	p2	(AC)7	14	22182351	22182364
chr10_4167	p2	(TG)7	14	22187057	22187070
chr10_4168	p2	(GT)6	12	22219253	22219264
chr10_4169	p3	(TCT)5	15	22228751	22228765
chr10_4170	p2	(TG)6	12	22255947	22255958
chr10_4171	p2	(CT)6	12	22257305	22257316
chr10_4172	p2	(CA)9	18	22260175	22260192
chr10_4173	p2	(TA)8	16	22263547	22263562
chr10_4174	p2	(AC)7	14	22273238	22273251
chr10_4175	c	(CCAT)5acatacat(AC)14	56	22273803	22273858
chr10_4176	p3	(CCT)7	21	22275347	22275367
chr10_4177	p2	(AC)6	12	22275861	22275872
chr10_4178	p2	(TC)11	22	22283441	22283462
chr10_4179	p3	(CCT)6	18	22291320	22291337
chr10_4180	c	(GT)10(TG)20	60	22297405	22297464
chr10_4181	p2	(GA)6	12	22303239	22303250

chr10_4182	p2	(GT)27	54	22311963	22312016
chr10_4183	p2	(GT)24	48	22316826	22316873
chr10_4184	p3	(TCT)6	18	22318271	22318288
chr10_4185	p2	(GT)26	52	22323914	22323965
chr10_4186	p4	(CCAT)7	28	22325791	22325818
chr10_4187	c	(AT)6(GT)18	48	22331932	22331979
chr10_4188	p3	(ATG)9	27	22333319	22333345
chr10_4189	p2	(AC)6	12	22337718	22337729
chr10_4190	p3	(ATT)5	15	22373238	22373252
chr10_4191	p2	(AC)6	12	22374330	22374341
chr10_4192	p3	(CCA)5	15	22387777	22387791
chr10_4193	p2	(AC)10	20	22390160	22390179
chr10_4194	p2	(CA)10	20	22391654	22391673
chr10_4195	p2	(GA)6	12	22394447	22394458
chr10_4196	p3	(CAG)6	18	22402692	22402709
chr10_4197	p2	(TG)13	26	22407557	22407582
chr10_4198	p3	(ACT)5	15	22412811	22412825
chr10_4199	p4	(TAGA)8	32	22446763	22446794
chr10_4200	p3	(ACA)7	21	22448749	22448769
chr10_4201	p2	(GT)7	14	22449108	22449121
chr10_4202	p2	(TA)6	12	22468076	22468087
chr10_4203	p2	(TA)7	14	22502347	22502360
chr10_4204	p2	(AC)6	12	22508680	22508691
chr10_4205	p2	(TG)7	14	22510672	22510685
chr10_4206	p2	(GT)14	28	22516394	22516421
chr10_4207	c	(CT)6cc(CT)7	28	22516702	22516729
chr10_4208	p2	(TG)14	28	22517103	22517130
chr10_4209	p2	(AC)8	16	22518688	22518703
chr10_4210	c	(AG)6aaagggagagggacatggaaat(AG)8	50	22527058	22527107
chr10_4211	p2	(CA)7	14	22532380	22532393
chr10_4212	p2	(GT)17	34	22538326	22538359
chr10_4213	c	(GAG)7gaagatgaggaggag(GAA)8	60	22545242	22545301
chr10_4214	p2	(AC)7	14	22551328	22551341
chr10_4215	p2	(TC)7	14	22551737	22551750
chr10_4216	p3	(TAT)6	18	22561383	22561400
chr10_4217	p2	(TG)16	32	22567009	22567040
chr10_4218	p2	(TG)7	14	22576052	22576065
chr10_4219	p2	(AC)6	12	22576180	22576191
chr10_4220	p2	(AC)22	44	22588508	22588551
chr10_4221	p3	(AGC)5	15	22625629	22625643
chr10_4222	p2	(GT)6	12	22628260	22628271
chr10_4223	p2	(TG)13	26	22632202	22632227
chr10_4224	p2	(CT)6	12	22638046	22638057
chr10_4225	p2	(CT)8	16	22663885	22663900
chr10_4226	p3	(ATG)5	15	22664788	22664802
chr10_4227	p2	(GT)6	12	22665684	22665695
chr10_4228	p2	(TG)14	28	22668887	22668914
chr10_4229	p2	(CA)9	18	22673979	22673996
chr10_4230	p2	(GT)10	20	22679668	22679687
chr10_4231	p3	(GCT)5	15	22679936	22679950
chr10_4232	p2	(TG)8	16	22680377	22680392
chr10_4233	p2	(AG)8	16	22680579	22680594

chr10_4234	p3	(CTC)5	15	22682064	22682078
chr10_4235	p2	(GA)7	14	22684562	22684575
chr10_4236	p3	(TTC)5	15	22693087	22693101
chr10_4237	p2	(GT)8	16	22695283	22695298
chr10_4238	p2	(AT)7	14	22696923	22696936
chr10_4239	p2	(TA)9	18	22704626	22704643
chr10_4240	c	(TC)10actctct(CA)8	43	22730970	22731012
chr10_4241	p2	(TG)7	14	22734370	22734383
chr10_4242	p2	(AC)7	14	22735086	22735099
chr10_4243	p2	(AC)8	16	22738818	22738833
chr10_4244	p2	(TG)11	22	22752318	22752339
chr10_4245	p2	(CA)11	22	22759791	22759812
chr10_4246	p2	(AC)7	14	22771976	22771989
chr10_4247	p2	(AC)6	12	22773175	22773186
chr10_4248	p2	(GT)7	14	22775206	22775219
chr10_4249	p3	(CAT)5	15	22789185	22789199
chr10_4250	p2	(GT)12	24	22793172	22793195
chr10_4251	c	(TG)13agtgtgt(GA)7	47	22793585	22793631
chr10_4252	p4	(ATGG)7	28	22795012	22795039
chr10_4253	p2	(AC)15	30	22798022	22798051
chr10_4254	p2	(CA)7	14	22802101	22802114
chr10_4255	p2	(TG)6	12	22805127	22805138
chr10_4256	c	ınnnnnnnnnnnnnnnnnnnnnnnnnnnnnnnnnnnggagggg(C	113	22806192	22806304
chr10_4257	p3	(AAG)5	15	22807052	22807066
chr10_4258	p2	(GT)15	30	22811288	22811317
chr10_4259	p3	(CTA)11	33	22811823	22811855
chr10_4260	p2	(CA)16	32	22814490	22814521
chr10_4261	p3	(CCT)5	15	22816164	22816178
chr10_4262	p3	(CCT)8	24	22820784	22820807
chr10_4263	c	(TA)6ctgtatatatacat(AC)6	38	22824788	22824825
chr10_4264	p2	(GA)6	12	22829945	22829956
chr10_4265	p2	(AG)6	12	22840082	22840093
chr10_4266	p2	(AT)6	12	22846606	22846617
chr10_4267	p2	(CA)7	14	22855422	22855435
chr10_4268	p3	(TCC)5	15	22860956	22860970
chr10_4269	p2	(AC)8	16	22890030	22890045
chr10_4270	p3	(GGA)5	15	22896589	22896603
chr10_4271	p2	(CA)9	18	22896940	22896957
chr10_4272	p2	(TG)6	12	22898657	22898668
chr10_4273	p2	(CA)7	14	22901211	22901224
chr10_4274	p2	(AC)16	32	22907092	22907123
chr10_4275	p4	(CATC)5	20	22907783	22907802
chr10_4276	p2	(TG)13	26	22907974	22907999
chr10_4277	p3	(CAT)5	15	22908358	22908372
chr10_4278	p2	(AC)6	12	22953214	22953225
chr10_4279	p2	(TG)28	56	22955112	22955167
chr10_4280	c	(GT)6gc(GT)11	36	22956849	22956884
chr10_4281	p3	(AAT)5	15	22957143	22957157
chr10_4282	p2	(AG)12	24	22958661	22958684
chr10_4283	p3	(AAT)6	18	22973401	22973418
chr10_4284	p3	(CCT)7	21	22995763	22995783
chr10_4285	p3	(TCC)5	15	22998809	22998823

chr10_4286	p2	(CA)8	16	22999435	22999450
chr10_4287	p3	(CCT)7	21	23002418	23002438
chr10_4288	p2	(TG)18	36	23006271	23006306
chr10_4289	p2	(AC)6	12	23006411	23006422
chr10_4290	p3	(TCC)6	18	23016029	23016046
chr10_4291	p3	(TCC)8	24	23017243	23017266
chr10_4292	p4	(AATA)6	24	23023167	23023190
chr10_4293	c	aatacacacgtacgtggaagcacatcatgcacacacacaaaatgagttcac	121	23034763	23034883
chr10_4294	p2	(TG)11	22	23034990	23035011
chr10_4295	p2	(AG)6	12	23036009	23036020
chr10_4296	p2	(GT)8	16	23037175	23037190
chr10_4297	p2	(GA)8	16	23037426	23037441
chr10_4298	p2	(GA)6	12	23038883	23038894
chr10_4299	p2	(TC)6	12	23040020	23040031
chr10_4300	p3	(GTA)5	15	23042355	23042369
chr10_4301	p2	(CA)8	16	23043921	23043936
chr10_4302	p2	(GT)6	12	23066891	23066902
chr10_4303	c	(TG)7agagtgtgt(GA)29	81	23068648	23068728
chr10_4304	c	(GT)12a(TG)30	85	23073839	23073923
chr10_4305	p2	(TC)13	26	23076486	23076511
chr10_4306	p2	(TG)6	12	23089277	23089288
chr10_4307	p3	(TGA)5	15	23094173	23094187
chr10_4308	p2	(AC)13	26	23097151	23097176
chr10_4309	p2	(GT)20	40	23105574	23105613
chr10_4310	c	(GT)18gc(GT)8	54	23112193	23112246
chr10_4311	p2	(AT)9	18	23116154	23116171
chr10_4312	p2	(TG)6	12	23116615	23116626
chr10_4313	p3	(AAT)5	15	23117555	23117569
chr10_4314	p2	(GT)6	12	23124632	23124643
chr10_4315	p2	(GT)13	26	23126436	23126461
chr10_4316	p3	(TCA)18	54	23129591	23129644
chr10_4317	p2	(TG)6	12	23162984	23162995
chr10_4318	p2	(GT)15	30	23173617	23173646
chr10_4319	p2	(GT)10	20	23175634	23175653
chr10_4320	p2	(TG)6	12	23175804	23175815
chr10_4321	p4	(TCTT)5	20	23186642	23186661
chr10_4322	p4	(TGAA)5	20	23195082	23195101
chr10_4323	p2	(TG)6	12	23205019	23205030
chr10_4324	p2	(GT)6	12	23205683	23205694
chr10_4325	c	(TC)7acactctcacact(CA)13	53	23212045	23212097
chr10_4326	p2	(TG)11	22	23212348	23212369
chr10_4327	p2	(CA)14	28	23212670	23212697
chr10_4328	p2	(AC)7	14	23214786	23214799
chr10_4329	p4	(ATGG)5	20	23238382	23238401
chr10_4330	p2	(CA)7	14	23248659	23248672
chr10_4331	c	(AC)15atacacccaggctgggaaagagag(AC)7	68	23269164	23269231
chr10_4332	p2	(AG)7	14	23294965	23294978
chr10_4333	p2	(GA)6	12	23295157	23295168
chr10_4334	p2	(AT)7	14	23295840	23295853
chr10_4335	p2	(TG)22	44	23298887	23298930
chr10_4336	p2	(GT)9	18	23303907	23303924
chr10_4337	p2	(TG)6	12	23304108	23304119

chr10_4338	p2	(TG)9	18	23312690	23312707
chr10_4339	p2	(GT)6	12	23322475	23322486
chr10_4340	p2	(TA)8	16	23324425	23324440
chr10_4341	p2	(TC)11	22	23335866	23335887
chr10_4342	p2	(CT)12	24	23336726	23336749
chr10_4343	p2	(TG)7	14	23339127	23339140
chr10_4344	p2	(CA)9	18	23347829	23347846
chr10_4345	p2	(AC)10	20	23351992	23352011
chr10_4346	c	(TG)24tatgtatgt(ATAG)11	101	23354341	23354441
chr10_4347	p3	(TCC)6	18	23355545	23355562
chr10_4348	p2	(CT)11	22	23357897	23357918
chr10_4349	p2	(TC)7	14	23358091	23358104
chr10_4350	p4	(GTTT)5	20	23358473	23358492
chr10_4351	p2	(AC)7	14	23377356	23377369
chr10_4352	p2	(TA)6	12	23385436	23385447
chr10_4353	p2	(AC)6	12	23399589	23399600
chr10_4354	p2	(GT)10	20	23405182	23405201
chr10_4355	p2	(TG)7	14	23407966	23407979
chr10_4356	p5	(TATGC)5	25	23408825	23408849
chr10_4357	p2	(CA)7	14	23409843	23409856
chr10_4358	c	(TG)6ta(TG)12	38	23411696	23411733
chr10_4359	p2	(CT)6	12	23455311	23455322
chr10_4360	p2	(AT)10	20	23490623	23490642
chr10_4361	p3	(AAT)5	15	23497606	23497620
chr10_4362	p2	(TG)21	42	23513448	23513489
chr10_4363	c	(TA)6(CA)22	56	23533004	23533059
chr10_4364	p4	(TATT)6	24	23535281	23535304
chr10_4365	p5	(ATTTG)5	25	23566377	23566401
chr10_4366	p2	(GA)14	28	23581402	23581429
chr10_4367	p3	(TGC)7	21	23592614	23592634
chr10_4368	p2	(TG)7	14	23609982	23609995
chr10_4369	p2	(AT)7	14	23613185	23613198
chr10_4370	p2	(TG)7	14	23622543	23622556
chr10_4371	p3	(GAT)7	21	23624876	23624896
chr10_4372	p3	(TTA)10	30	23626039	23626068
chr10_4373	p2	(GT)6	12	23642595	23642606
chr10_4374	p2	(CA)6	12	23659040	23659051
chr10_4375	p2	(TG)6	12	23663044	23663055
chr10_4376	p2	(CA)16	32	23672576	23672607
chr10_4377	p2	(TG)8	16	23690613	23690628
chr10_4378	p2	(TG)7	14	23692555	23692568
chr10_4379	p2	(TG)7	14	23692741	23692754
chr10_4380	p2	(TG)7	14	23692865	23692878
chr10_4381	p2	(TG)7	14	23692989	23693002
chr10_4382	p2	(TG)7	14	23693113	23693126
chr10_4383	p2	(TG)15	30	23716973	23717002
chr10_4384	p2	(CT)7	14	23724929	23724942
chr10_4385	p3	(TGG)5	15	23726802	23726816
chr10_4386	p2	(CA)6	12	23726931	23726942
chr10_4387	c	(AG)6aa(AG)7	28	23728487	23728514
chr10_4388	p3	(AAT)5	15	23729059	23729073
chr10_4389	p2	(AC)6	12	23730451	23730462

chr10_4390	p2	(AG)6	12	23739064	23739075
chr10_4391	p2	(GT)8	16	23739458	23739473
chr10_4392	p2	(TC)8	16	23740881	23740896
chr10_4393	p2	(TC)15	30	23767426	23767455
chr10_4394	p2	(TG)6	12	23782040	23782051
chr10_4395	p2	(AG)8	16	23783059	23783074
chr10_4396	p2	(GT)6	12	23785672	23785683
chr10_4397	p4	(TTAT)5	20	23787046	23787065
chr10_4398	p3	(TTA)6	18	23800608	23800625
chr10_4399	p2	(TG)8	16	23806168	23806183
chr10_4400	p2	(AC)13	26	23817027	23817052
chr10_4401	p3	(TTA)9	27	23819138	23819164
chr10_4402	p3	(AGT)6	18	23819444	23819461
chr10_4403	p2	(AG)7	14	23820374	23820387
chr10_4404	p2	(AG)7	14	23820924	23820937
chr10_4405	p3	(CCT)7	21	23821140	23821160
chr10_4406	p2	(AC)10	20	23821936	23821955
chr10_4407	p4	(GATA)5	20	23825443	23825462
chr10_4408	p3	(TGG)7	21	23831978	23831998
chr10_4409	p3	(TAA)5	15	23833919	23833933
chr10_4410	p5	(TAAGC)5	25	23836571	23836595
chr10_4411	p3	(AAC)6	18	23839904	23839921
chr10_4412	p4	(AAAC)5	20	23841051	23841070
chr10_4413	p2	(TA)11	22	23842342	23842363
chr10_4414	p4	(TTTA)5	20	23846578	23846597
chr10_4415	p2	(AC)6	12	23856022	23856033
chr10_4416	p2	(TC)9	18	23902267	23902284
chr10_4417	p2	(CA)8	16	23903377	23903392
chr10_4418	p2	(TC)7	14	23904429	23904442
chr10_4419	p2	(AG)7	14	23919598	23919611
chr10_4420	p2	(TG)10	20	23922753	23922772
chr10_4421	p4	(TAGA)18	72	23929191	23929262
chr10_4422	p2	(TC)12	24	23936671	23936694
chr10_4423	p2	(CA)6	12	23941814	23941825
chr10_4424	p2	(TA)8	16	23946363	23946378
chr10_4425	p3	(TTG)7	21	23975486	23975506
chr10_4426	p3	(CAT)5	15	23978548	23978562
chr10_4427	p2	(AG)7	14	23979117	23979130
chr10_4428	p2	(AT)9	18	23986783	23986800
chr10_4429	p2	(AC)8	16	23992144	23992159
chr10_4430	p3	(AAC)5	15	23992552	23992566
chr10_4431	p2	(GA)6	12	23998966	23998977
chr10_4432	p2	(CA)8	16	24002251	24002266
chr10_4433	c	5aagtctggcccaattaaatatgcactgtgaatgcatcatgactgcgcgcg	127	24008927	24009053
chr10_4434	p4	(TAAA)5	20	24010093	24010112
chr10_4435	p2	(TG)6	12	24026793	24026804
chr10_4436	p2	(CA)8	16	24027286	24027301
chr10_4437	p2	(CA)6	12	24029763	24029774
chr10_4438	p2	(AC)15	30	24034436	24034465
chr10_4439	c	(TG)9(AG)8aa(AG)10	56	24036418	24036473
chr10_4440	p3	(ATG)8	24	24040625	24040648
chr10_4441	p2	(AG)6	12	24041094	24041105

chr10_4442	p2	(CA)6	12	24041215	24041226
chr10_4443	p2	(GT)22	44	24041676	24041719
chr10_4444	c	tctatatgccaccattacttgtcattaaatttgtgtctcctctctcctgcagtgtg	122	24049351	24049472
chr10_4445	p3	(TAC)5	15	24049659	24049673
chr10_4446	p2	(TG)8	16	24060418	24060433
chr10_4447	p2	(CA)26	52	24061342	24061393
chr10_4448	p2	(TG)6	12	24061585	24061596
chr10_4449	p2	(CA)9	18	24063039	24063056
chr10_4450	p2	(CA)16	32	24064941	24064972
chr10_4451	p2	(CA)26	52	24066114	24066165
chr10_4452	p2	(CA)17	34	24068901	24068934
chr10_4453	p2	(CA)13	26	24078380	24078405
chr10_4454	p2	(GT)12	24	24078706	24078729
chr10_4455	p2	(TG)9	18	24083300	24083317
chr10_4456	p2	(CA)13	26	24083426	24083451
chr10_4457	c	gagtcatgatcaaagtatatactgacagacaaat(AC)6tggccacccca	180	24086742	24086921
chr10_4458	p2	(TC)11	22	24087167	24087188
chr10_4459	p2	(CA)22	44	24091903	24091946
chr10_4460	p2	(TG)9	18	24092467	24092484
chr10_4461	p2	(GT)10	20	24095337	24095356
chr10_4462	c	gcttcattttgagtactcgaaggctgttgtcctttgaagcttggccttttccattc	129	24096956	24097084
chr10_4463	p2	(TG)17	34	24098490	24098523
chr10_4464	p2	(AG)10	20	24099550	24099569
chr10_4465	p2	(AC)7	14	24112724	24112737
chr10_4466	p2	(AC)6	12	24114736	24114747
chr10_4467	p2	(TG)6	12	24114857	24114868
chr10_4468	p2	(TG)9	18	24116135	24116152
chr10_4469	p2	(TG)12	24	24118808	24118831
chr10_4470	p2	(CA)15	30	24126461	24126490
chr10_4471	p2	(GT)12	24	24127539	24127562
chr10_4472	p2	(AC)7	14	24128889	24128902
chr10_4473	p2	(GA)6	12	24137970	24137981
chr10_4474	c	(AC)11gcg(CA)20	65	24144769	24144833
chr10_4475	p2	(CA)9	18	24146591	24146608
chr10_4476	p2	(CA)7	14	24147031	24147044
chr10_4477	p2	(GT)11	22	24155936	24155957
chr10_4478	c	caccgcagataatacccggcattctgtcagaccaccagccctggcttgtgg	112	24160455	24160566
chr10_4479	p2	(AC)8	16	24162067	24162082
chr10_4480	p2	(TG)9	18	24166920	24166937
chr10_4481	p2	(TC)7	14	24169736	24169749
chr10_4482	p2	(TG)10	20	24172041	24172060
chr10_4483	p2	(TG)6	12	24172326	24172337
chr10_4484	p2	(CT)9	18	24175811	24175828
chr10_4485	p2	(AC)7	14	24179625	24179638
chr10_4486	p2	(CT)6	12	24185122	24185133
chr10_4487	p2	(CA)7	14	24188399	24188412
chr10_4488	p2	(CA)6	12	24202109	24202120
chr10_4489	p2	(AC)6	12	24210745	24210756
chr10_4490	p2	(TG)6	12	24210923	24210934
chr10_4491	p2	(TC)8	16	24211223	24211238
chr10_4492	c	(TG)12c(GT)8	41	24215720	24215760
chr10_4493	p2	(CA)6	12	24225895	24225906

chr10_4494	p2	(AC)6	12	24227399	24227410
chr10_4495	p2	(TA)6	12	24238538	24238549
chr10_4496	p2	(AC)11	22	24239783	24239804
chr10_4497	p2	(AC)9	18	24259001	24259018
chr10_4498	p3	(AAT)7	21	24285188	24285208
chr10_4499	p2	(AT)7	14	24289961	24289974
chr10_4500	p2	(CA)11	22	24293518	24293539
chr10_4501	p2	(GT)28	56	24293668	24293723
chr10_4502	c	(GA)9gg(GA)9	38	24309363	24309400
chr10_4503	p2	(AC)6	12	24317609	24317620
chr10_4504	p3	(CTC)5	15	24328737	24328751
chr10_4505	p5	(TTTTG)5	25	24331386	24331410
chr10_4506	p2	(TG)6	12	24339258	24339269
chr10_4507	p2	(CA)15	30	24352637	24352666
chr10_4508	p2	(TA)10	20	24356672	24356691
chr10_4509	p3	(CTC)6	18	24369782	24369799
chr10_4510	p2	(AC)8	16	24384470	24384485
chr10_4511	p2	(AC)21	42	24390223	24390264
chr10_4512	c	catctcaaaccacaaaagtatttactggaacagcatctatttccaaggttctg	120	24392379	24392498
chr10_4513	c	(TG)6(AG)8	28	24398384	24398411
chr10_4514	p2	(TG)6	12	24404899	24404910
chr10_4515	p4	(AGAT)14	56	24408137	24408192
chr10_4516	p2	(TG)7	14	24414051	24414064
chr10_4517	p2	(TG)7	14	24414766	24414779
chr10_4518	p2	(TG)8	16	24419035	24419050
chr10_4519	p3	(TGA)9	27	24426641	24426667
chr10_4520	p2	(CA)6	12	24428912	24428923
chr10_4521	p2	(AC)13	26	24448898	24448923
chr10_4522	p2	(AC)6	12	24452934	24452945
chr10_4523	p2	(TA)6	12	24468916	24468927
chr10_4524	p2	(TG)14	28	24470716	24470743
chr10_4525	p5	(AGGTA)5	25	24471009	24471033
chr10_4526	p2	(CA)12	24	24481151	24481174
chr10_4527	p2	(AC)11	22	24486183	24486204
chr10_4528	p3	(ATA)5	15	24496704	24496718
chr10_4529	p2	(TG)21	42	24500695	24500736
chr10_4530	p2	(AC)29	58	24500884	24500941
chr10_4531	p2	(TA)12	24	24507396	24507419
chr10_4532	p2	(AC)7	14	24510347	24510360
chr10_4533	p2	(CA)6	12	24542486	24542497
chr10_4534	p2	(GA)7	14	24551688	24551701
chr10_4535	p3	(TAA)5	15	24552712	24552726
chr10_4536	c	(CA)14cgcagagtcacacatc(CA)6	56	24552887	24552942
chr10_4537	p2	(AG)10	20	24553103	24553122
chr10_4538	p2	(CA)11	22	24556263	24556284
chr10_4539	p2	(TG)6	12	24568457	24568468
chr10_4540	p2	(GT)6	12	24573667	24573678
chr10_4541	p2	(TG)15	30	24579472	24579501
chr10_4542	p4	(TAGG)6	24	24581150	24581173
chr10_4543	p2	(TG)26	52	24582235	24582286
chr10_4544	p2	(AC)7	14	24589568	24589581
chr10_4545	p2	(TA)6	12	24595926	24595937

chr10_4546	p2	(TA)7	14	24599189	24599202
chr10_4547	p2	(TA)7	14	24601355	24601368
chr10_4548	p3	(TTC)5	15	24605364	24605378
chr10_4549	p4	(ATGG)6	24	24605982	24606005
chr10_4550	p2	(AT)6	12	24622360	24622371
chr10_4551	c	(GA)12cagcagtaagtgcaatgggctagaggagagcggc(GAG)5	73	24629326	24629398
chr10_4552	p2	(AT)6	12	24631094	24631105
chr10_4553	p2	(CA)7	14	24640933	24640946
chr10_4554	p2	(AC)22	44	24642760	24642803
chr10_4555	p3	(ATC)5	15	24645211	24645225
chr10_4556	p2	(CT)6	12	24649257	24649268
chr10_4557	p4	(CTCC)6	24	24659735	24659758
chr10_4558	c	tcgttgagatcagtcacttcctgattctgtcagtcaacatggacaaacctaag	118	24666132	24666249
chr10_4559	p3	(AAC)5	15	24676435	24676449
chr10_4560	p3	(AGC)5	15	24700202	24700216
chr10_4561	p2	(TA)8	16	24714955	24714970
chr10_4562	p4	(ATGT)5	20	24741801	24741820
chr10_4563	p2	(GT)14	28	24742274	24742301
chr10_4564	p2	(AC)6	12	24758323	24758334
chr10_4565	p3	(GCA)5	15	24766487	24766501
chr10_4566	p3	(AGG)5	15	24770342	24770356
chr10_4567	p2	(AC)6	12	24792211	24792222
chr10_4568	p3	(AGC)6	18	24795578	24795595
chr10_4569	p2	(GT)18	36	24798541	24798576
chr10_4570	p4	(GTAT)5	20	24799379	24799398
chr10_4571	p2	(TA)6	12	24806538	24806549
chr10_4572	p2	(GA)7	14	24827446	24827459
chr10_4573	p3	(GAA)5	15	24827661	24827675
chr10_4574	p2	(CA)8	16	24829549	24829564
chr10_4575	p2	(CA)20	40	24830682	24830721
chr10_4576	p2	(AG)7	14	24838866	24838879
chr10_4577	c	;ttgtatagtatgtgtgtgtctgtgcgatcctatggggatgtaactgtgcagtgt	131	24844024	24844154
chr10_4578	p2	(AC)19	38	24849922	24849959
chr10_4579	c	gtataactgcctctctcgctccctctatgctgcacacacattcatgtgctctca	121	24857882	24858002
chr10_4580	p2	(GT)6	12	24881226	24881237
chr10_4581	p3	(TGG)5	15	24896307	24896321
chr10_4582	p3	(ACT)6	18	24898388	24898405
chr10_4583	p2	(AT)7	14	24922452	24922465
chr10_4584	p2	(TG)7	14	24937660	24937673
chr10_4585	p2	(CA)26	52	24939326	24939377
chr10_4586	p2	(AC)8	16	24946077	24946092
chr10_4587	p2	(AC)8	16	24946307	24946322
chr10_4588	c	(TA)6c(AT)6	25	24949096	24949120
chr10_4589	p2	(GT)32	64	24960587	24960650
chr10_4590	p2	(TA)6	12	24968778	24968789
chr10_4591	p2	(TG)6	12	24969565	24969576
chr10_4592	p2	(GT)8	16	24971249	24971264
chr10_4593	p3	(CCA)5	15	24976739	24976753
chr10_4594	p3	(CCA)5	15	24977850	24977864
chr10_4595	p3	(TCA)10	30	24984605	24984634
chr10_4596	p4	(ATAG)6	24	24999875	24999898
chr10_4597	c	(CT)6t(TC)9	31	25008188	25008218

chr10_4598	p3	(AGC)5	15	25009569	25009583
chr10_4599	p2	(TA)6	12	25021644	25021655
chr10_4600	p2	(AC)13	26	25022562	25022587
chr10_4601	p3	(CTT)5	15	25024133	25024147
chr10_4602	p2	(AC)9	18	25028010	25028027
chr10_4603	p4	(ATCC)5	20	25028650	25028669
chr10_4604	p2	(GT)6	12	25033406	25033417
chr10_4605	p2	(TG)6	12	25040503	25040514
chr10_4606	p2	(CA)6	12	25045129	25045140
chr10_4607	p3	(TTA)13	39	25056138	25056176
chr10_4608	p3	(TGA)7	21	25061141	25061161
chr10_4609	p4	(ATTT)5	20	25062625	25062644
chr10_4610	p2	(AC)7	14	25065575	25065588
chr10_4611	p2	(TG)17	34	25072661	25072694
chr10_4612	p2	(GT)31	62	25075030	25075091
chr10_4613	p2	(CA)6	12	25075640	25075651
chr10_4614	p2	(AC)7	14	25097236	25097249
chr10_4615	p2	(AT)6	12	25106790	25106801
chr10_4616	p2	(CT)8	16	25109320	25109335
chr10_4617	p2	(GT)13	26	25111732	25111757
chr10_4618	p2	(AC)10	20	25111880	25111899
chr10_4619	p2	(GT)16	32	25114052	25114083
chr10_4620	p2	(AC)10	20	25114205	25114224
chr10_4621	c	(CA)7tgtgcagtattcactcaaccacaacatacatgctt(AC)8	65	25120099	25120163
chr10_4622	p2	(TG)10	20	25126178	25126197
chr10_4623	p2	(TG)11	22	25129629	25129650
chr10_4624	c	[illegible]tgtctctctcttcctctttaatggagcactggcctggggcactgaaccagcc	118	25131267	25131384
chr10_4625	p2	(TG)8	16	25133778	25133793
chr10_4626	p2	(TG)7	14	25134364	25134377
chr10_4627	p2	(CA)12	24	25135067	25135090
chr10_4628	p2	(GA)9	18	25135236	25135253
chr10_4629	p2	(GT)14	28	25135567	25135594
chr10_4630	c	(CA)9t(AC)8at(AC)8at(AC)8ag(AC)7	87	25135725	25135811
chr10_4631	p2	(CT)8	16	25136080	25136095
chr10_4632	p2	(TG)10	20	25138693	25138712
chr10_4633	c	(GT)11t(TG)11	45	25140134	25140178
chr10_4634	p3	(CAA)5	15	25140714	25140728
chr10_4635	p2	(AT)6	12	25142404	25142415
chr10_4636	p2	(TG)8	16	25143559	25143574
chr10_4637	p2	(GT)7	14	25148560	25148573
chr10_4638	p2	(AC)9	18	25150545	25150562
chr10_4639	p2	(TG)6	12	25152699	25152710
chr10_4640	p2	(CA)8	16	25156595	25156610
chr10_4641	c	(TC)6(TG)16	44	25157727	25157770
chr10_4642	c	(TC)6cctcttgctcaagcaccttttt(TC)6(AC)7	60	25158128	25158187
chr10_4643	p2	(TG)15	30	25158403	25158432
chr10_4644	p2	(CA)11	22	25160154	25160175
chr10_4645	p2	(AC)11	22	25165878	25165899
chr10_4646	p2	(TG)6	12	25166903	25166914
chr10_4647	p2	(CA)20	40	25168174	25168213
chr10_4648	p2	(TG)12	24	25170450	25170473
chr10_4649	p2	(TG)13	26	25172774	25172799

chr10_4650	p2	(TG)22	44	25173878	25173921
chr10_4651	c	aatatcaacggtgtcagccactgggggtcattgttccccactggtccagctc	166	25174130	25174295
chr10_4652	p2	(AG)13	26	25175422	25175447
chr10_4653	p2	(GT)20	40	25200068	25200107
chr10_4654	p2	(GA)6	12	25204271	25204282
chr10_4655	c	tcccccccaggctacattgaaaggtcagggatccatgcaggaaattagtgtc	115	25207531	25207645
chr10_4656	p2	(CA)6	12	25210204	25210215
chr10_4657	p2	(TG)13	26	25210353	25210378
chr10_4658	p2	(AC)6	12	25212214	25212225
chr10_4659	p2	(GT)6	12	25213827	25213838
chr10_4660	p2	(AG)6	12	25220256	25220267
chr10_4661	p2	(GT)9	18	25220822	25220839
chr10_4662	p2	(TC)7	14	25223952	25223965
chr10_4663	p2	(CA)6	12	25224870	25224881
chr10_4664	p2	(AC)21	42	25225430	25225471
chr10_4665	c	(CA)19gg(GA)6	52	25225823	25225874
chr10_4666	p2	(TG)12	24	25227871	25227894
chr10_4667	p2	(TA)7	14	25264919	25264932
chr10_4668	p2	(GT)11	22	25268551	25268572
chr10_4669	p2	(AC)7	14	25269001	25269014
chr10_4670	p3	(TTG)9	27	25271090	25271116
chr10_4671	c	(TG)6aaagagagacaaggaaaagaattaagagaa(AG)15	72	25274747	25274818
chr10_4672	p2	(TG)8	16	25281559	25281574
chr10_4673	c	(TG)12cttgctgcagccat(TG)9ttgtgtt(TG)12	87	25287314	25287400
chr10_4674	p2	(GT)6	12	25291084	25291095
chr10_4675	p2	(CT)6	12	25291242	25291253
chr10_4676	p3	(GGA)5	15	25308476	25308490
chr10_4677	p2	(CA)6	12	25313364	25313375
chr10_4678	p2	(CT)7	14	25317685	25317698
chr10_4679	p3	(TGC)5	15	25318274	25318288
chr10_4680	p3	(TGT)5	15	25320250	25320264
chr10_4681	p2	(TG)12	24	25333144	25333167
chr10_4682	p2	(TG)6	12	25336619	25336630
chr10_4683	p5	(AAATC)5	25	25347290	25347314
chr10_4684	p2	(GT)12	24	25352511	25352534
chr10_4685	p2	(AC)8	16	25357780	25357795
chr10_4686	c	caagctgtctaccgaaccgacaggctacaactctatgtatacaaagaggaa	115	25358096	25358210
chr10_4687	p2	(AC)6	12	25368149	25368160
chr10_4688	p5	(ATTTG)5	25	25371414	25371438
chr10_4689	c	(GC)7(AC)10	34	25377471	25377504
chr10_4690	p2	(CA)7	14	25397986	25397999
chr10_4691	p2	(AG)6	12	25401053	25401064
chr10_4692	p2	(AT)8	16	25405682	25405697
chr10_4693	p2	(AC)8	16	25416718	25416733
chr10_4694	p2	(CA)10	20	25418058	25418077
chr10_4695	p2	(CA)11	22	25418516	25418537
chr10_4696	p2	(AC)7	14	25440865	25440878
chr10_4697	p3	(GAG)7	21	25443201	25443221
chr10_4698	p4	(TTGT)7	28	25449782	25449809
chr10_4699	p2	(AC)19	38	25470287	25470324
chr10_4700	p4	(CATA)5	20	25474124	25474143
chr10_4701	p2	(CA)6	12	25474262	25474273

chr10_4702	c*	(AAC)5(AC)15*	43	25481875	25481917
chr10_4703	p2	(CA)10	20	25484366	25484385
chr10_4704	p2	(AC)15	30	25484748	25484777
chr10_4705	p4	(TTTA)5	20	25499594	25499613
chr10_4706	p2	(CA)10	20	25507376	25507395
chr10_4707	p2	(AC)8	16	25507857	25507872
chr10_4708	p2	(AC)6	12	25510578	25510589
chr10_4709	p2	(AC)7	14	25511531	25511544
chr10_4710	c	(AGG)7(AGA)5	36	25511652	25511687
chr10_4711	p2	(TG)12	24	25521231	25521254
chr10_4712	p2	(TG)12	24	25526455	25526478
chr10_4713	p2	(TG)7	14	25534309	25534322
chr10_4714	p2	(TA)6	12	25536346	25536357
chr10_4715	p2	(TG)9	18	25541493	25541510
chr10_4716	p2	(TA)6	12	25549436	25549447
chr10_4717	p3	(ATT)5	15	25551358	25551372
chr10_4718	p4	(AAAT)5	20	25552837	25552856
chr10_4719	p2	(CA)20	40	25572404	25572443
chr10_4720	c	(TG)8agtgt(GA)6	33	25584104	25584136
chr10_4721	p2	(GT)16	32	25587906	25587937
chr10_4722	c	(AT)6(GT)12	36	25588183	25588218
chr10_4723	p3	(CAC)11	33	25589194	25589226
chr10_4724	p2	(AC)8	16	25591171	25591186
chr10_4725	c	(TG)9(AG)17	52	25598244	25598295
chr10_4726	p2	(GT)14	28	25598683	25598710
chr10_4727	p2	(AG)6	12	25599135	25599146
chr10_4728	p2	(GT)6	12	25599750	25599761
chr10_4729	p2	(TG)11	22	25601052	25601073
chr10_4730	p3	(CCT)5	15	25602718	25602732
chr10_4731	p2	(CA)13	26	25604142	25604167
chr10_4732	p3	(CCT)5	15	25604794	25604808
chr10_4733	p2	(TG)7	14	25605059	25605072
chr10_4734	p2	(CA)7	14	25605286	25605299
chr10_4735	p2	(AC)13	26	25605455	25605480
chr10_4736	c	(CT)14t(TC)6	41	25607904	25607944
chr10_4737	c	(AC)7c(CA)10	35	25609193	25609227
chr10_4738	c	(AC)10(AT)6	32	25609687	25609718
chr10_4739	p2	(AC)10	20	25610104	25610123
chr10_4740	p2	(AC)9	18	25610775	25610792
chr10_4741	p2	(TC)7	14	25612504	25612517
chr10_4742	p2	(TG)9	18	25612957	25612974
chr10_4743	p2	(CA)6	12	25613112	25613123
chr10_4744	p2	(AC)11	22	25613297	25613318
chr10_4745	c	(CT)6tt(CA)6t(AC)16	59	25618973	25619031
chr10_4746	p2	(TG)12	24	25619346	25619369
chr10_4747	p2	(AC)6	12	25621007	25621018
chr10_4748	p2	(CA)11	22	25621160	25621181
chr10_4749	p2	(TC)6	12	25623932	25623943
chr10_4750	c	(TG)12(AG)8	40	25624491	25624530
chr10_4751	p2	(TA)6	12	25626109	25626120
chr10_4752	p2	(AC)23	46	25626810	25626855
chr10_4753	p2	(CA)6	12	25628179	25628190

chr10_4754	p5	(TTTTG)5	25	25629375	25629399
chr10_4755	c	(GT)10gcatgtgtgcgtgtgtttaa(AG)6	52	25631479	25631530
chr10_4756	p2	(GT)13	26	25632764	25632789
chr10_4757	p2	(TG)6	12	25634401	25634412
chr10_4758	p3	(CCA)5	15	25636039	25636053
chr10_4759	p2	(GT)21	42	25636394	25636435
chr10_4760	c	aaaaaacaagaaaagagagtgcag(GA)8aaaaaaacaacaaagatc	177	25637255	25637431
chr10_4761	p2	(TG)8	16	25637775	25637790
chr10_4762	c	(CA)6ctggcatacggtgactgtctgacgcc(CA)12	62	25639286	25639347
chr10_4763	p2	(AC)23	46	25639472	25639517
chr10_4764	p2	(GT)19	38	25641560	25641597
chr10_4765	p2	(GT)6	12	25642927	25642938
chr10_4766	c	(AC)6atgcacacattcagacgcacaca(CT)9(CA)6	65	25643048	25643112
chr10_4767	p2	(AC)13	26	25643237	25643262
chr10_4768	p2	(GT)13	26	25643439	25643464
chr10_4769	c	(GT)9(GA)23	64	25645511	25645574
chr10_4770	p2	(GA)7	14	25646000	25646013
chr10_4771	c	ccccgaccgcagccccaccctcagggccccttctgggctaaccgacctc	121	25647974	25648094
chr10_4772	p2	(TG)6	12	25648209	25648220
chr10_4773	c	gacatattcatgaagaaatacacagttgtcagaaaaacagcatacattct(T	130	25649492	25649621
chr10_4774	p2	(CA)26	52	25650169	25650220
chr10_4775	p2	(GT)11	22	25650512	25650533
chr10_4776	p3	(GAT)5	15	25654563	25654577
chr10_4777	p2	(GT)29	58	25655586	25655643
chr10_4778	p2	(GT)12	24	25657350	25657373
chr10_4779	p2	(CA)6	12	25659394	25659405
chr10_4780	p2	(TG)23	46	25659591	25659636
chr10_4781	p2	(GT)12	24	25660498	25660521
chr10_4782	p3	(CCT)5	15	25661158	25661172
chr10_4783	p2	(TG)10	20	25661300	25661319
chr10_4784	p2	(TG)6	12	25661421	25661432
chr10_4785	p2	(AC)17	34	25662680	25662713
chr10_4786	p2	(TC)6	12	25662885	25662896
chr10_4787	p2	(AC)9	18	25663781	25663798
chr10_4788	p2	(GT)18	36	25665170	25665205
chr10_4789	p2	(AC)6	12	25665823	25665834
chr10_4790	p2	(TG)15	30	25666659	25666688
chr10_4791	p2	(AC)15	30	25669294	25669323
chr10_4792	p2	(AC)12	24	25669435	25669458
chr10_4793	p2	(TG)6	12	25678247	25678258
chr10_4794	c	aaaccttccatttagacatagcctgagtatgtcaatgaagtgaatgaatggc	129	25679947	25680075
chr10_4795	c	(TG)6cata(TG)7	30	25680197	25680226
chr10_4796	p3	(TAT)9	27	25680769	25680795
chr10_4797	p2	(CA)17	34	25681408	25681441
chr10_4798	c	(TG)6(AG)6	24	25681724	25681747
chr10_4799	p2	(TG)10	20	25685267	25685286
chr10_4800	p2	(TG)17	34	25685396	25685429
chr10_4801	p2	(AC)8	16	25686000	25686015
chr10_4802	p3	(TGG)6	18	25687017	25687034
chr10_4803	p2	(AC)10	20	25688459	25688478
chr10_4804	p2	(TG)19	38	25692481	25692518
chr10_4805	p2	(AC)6	12	25692943	25692954

chr10_4806	p2	(CA)6	12	25693190	25693201
chr10_4807	p2	(AT)6	12	25694227	25694238
chr10_4808	p2	(AC)8	16	25695911	25695926
chr10_4809	p2	(AC)15	30	25697573	25697602
chr10_4810	p2	(CA)6	12	25699898	25699909
chr10_4811	p2	(GT)9	18	25703590	25703607
chr10_4812	p2	(GT)6	12	25705632	25705643
chr10_4813	p2	(TG)6	12	25705760	25705771
chr10_4814	c	(AGAC)6(AGAT)19	100	25707613	25707712
chr10_4815	p2	(GT)6	12	25707831	25707842
chr10_4816	p2	(GT)15	30	25708674	25708703
chr10_4817	p2	(TG)6	12	25712395	25712406
chr10_4818	p2	(CA)21	42	25712614	25712655
chr10_4819	p3	(TCA)5	15	25721309	25721323
chr10_4820	c	(TG)9agagtga(GAGT)5	45	25727499	25727543
chr10_4821	p2	(GT)24	48	25728497	25728544
chr10_4822	p2	(TG)8	16	25738484	25738499
chr10_4823	p2	(CA)13	26	25747352	25747377
chr10_4824	p2	(CA)12	24	25753787	25753810
chr10_4825	p2	(TG)18	36	25762080	25762115
chr10_4826	p2	(AC)7	14	25762510	25762523
chr10_4827	p2	(AC)10	20	25771642	25771661
chr10_4828	p2	(TG)13	26	25775688	25775713
chr10_4829	p2	(CA)14	28	25778703	25778730
chr10_4830	p2	(CA)12	24	25782794	25782817
chr10_4831	p2	(CA)9	18	25783374	25783391
chr10_4832	p2	(CA)7	14	25786080	25786093
chr10_4833	p2	(CA)7	14	25786196	25786209
chr10_4834	p3	(TGT)6	18	25819712	25819729
chr10_4835	p2	(AC)10	20	25820976	25820995
chr10_4836	p2	(TG)7	14	25824680	25824693
chr10_4837	p2	(TG)17	34	25825941	25825974
chr10_4838	p2	(GT)12	24	25847099	25847122
chr10_4839	p4	(TTTA)5	20	25848925	25848944
chr10_4840	p2	(AC)7	14	25856404	25856417
chr10_4841	p2	(TG)12	24	25859597	25859620
chr10_4842	p3	(TGA)8	24	25869134	25869157
chr10_4843	p3	(CAG)5	15	25882090	25882104
chr10_4844	p3	(TGT)5	15	25903209	25903223
chr10_4845	p2	(TG)16	32	25903381	25903412
chr10_4846	c	(TC)6cctccctct(CA)7	35	25903586	25903620
chr10_4847	p2	(CA)7	14	25903991	25904004
chr10_4848	p2	(TG)6	12	25906618	25906629
chr10_4849	p2	(GT)6	12	25908155	25908166
chr10_4850	p2	(TG)6	12	25935893	25935904
chr10_4851	p2	(TA)7	14	25948566	25948579
chr10_4852	p2	(TG)6	12	25957559	25957570
chr10_4853	p2	(TC)7	14	25959472	25959485
chr10_4854	c	agagcttagaccattagatgctcacactgatgttgcttggtgccttggatc(C	161	25963908	25964068
chr10_4855	p2	(CA)7	14	25965401	25965414
chr10_4856	p2	(GT)13	26	25965732	25965757
chr10_4857	p2	(CA)6	12	25970365	25970376

chr10_4858	p2	(AC)7	14	25972469	25972482
chr10_4859	c	(TC)7tt(TC)9(AC)8	50	25972663	25972712
chr10_4860	p2	(GT)6	12	25977492	25977503
chr10_4861	c	(CT)6cactcacacacgcg(CA)9	44	25977638	25977681
chr10_4862	c	(GT)10(GA)12	44	25984054	25984097
chr10_4863	p2	(AT)7	14	25984419	25984432
chr10_4864	p2	(AC)6	12	25985907	25985918
chr10_4865	p2	(AC)12	24	25986068	25986091
chr10_4866	p2	(TG)10	20	25994206	25994225
chr10_4867	p2	(TG)8	16	26007829	26007844
chr10_4868	p2	(GT)8	16	26011074	26011089
chr10_4869	p2	(TG)20	40	26011213	26011252
chr10_4870	p2	(GA)6	12	26011958	26011969
chr10_4871	p2	(AC)6	12	26014522	26014533
chr10_4872	c	:agaaccatt(TC)8gctcact(CA)7ctgtgcatctttcagtttttcttatttt	192	26014917	26015108
chr10_4873	p2	(TC)9	18	26019028	26019045
chr10_4874	p2	(TG)11	22	26020741	26020762
chr10_4875	p2	(GT)8	16	26026106	26026121
chr10_4876	c	nnnnnnnnnnnnnnnnnnnnnnnnnnnnnnnnnnnnnnnnnnnn	164	26033317	26033480
chr10_4877	p2	(GT)7	14	26034717	26034730
chr10_4878	p3	(ACT)9	27	26036946	26036972
chr10_4879	p2	(AC)6	12	26037452	26037463
chr10_4880	p2	(CA)22	44	26039627	26039670
chr10_4881	p2	(TC)7	14	26040261	26040274
chr10_4882	p2	(TG)6	12	26042551	26042562
chr10_4883	c	(AC)6c(CA)11	35	26048267	26048301
chr10_4884	p2	(AC)7	14	26051804	26051817
chr10_4885	p2	(GT)9	18	26056829	26056846
chr10_4886	p2	(AC)10	20	26071816	26071835
chr10_4887	p2	(AC)12	24	26079476	26079499
chr10_4888	p2	(TG)9	18	26087295	26087312
chr10_4889	p2	(GT)10	20	26102260	26102279
chr10_4890	p4	(TTGA)5	20	26106565	26106584
chr10_4891	p2	(TC)10	20	26108735	26108754
chr10_4892	c	(TG)7tt(TG)6	28	26109698	26109725
chr10_4893	p2	(GT)12	24	26110017	26110040
chr10_4894	p2	(CA)9	18	26121904	26121921
chr10_4895	p3	(ACA)6	18	26122754	26122771
chr10_4896	c	(GT)14gc(GT)6	42	26138441	26138482
chr10_4897	c	actatttaaaaagaaaaaaaactaaatggcaactgccactgtgtggatgca	128	26143642	26143769
chr10_4898	p2	(TG)6	12	26143990	26144001
chr10_4899	p2	(TG)6	12	26144151	26144162
chr10_4900	p2	(GT)10	20	26172097	26172116
chr10_4901	p3	(GCA)5	15	26176563	26176577
chr10_4902	p2	(GT)13	26	26188752	26188777
chr10_4903	p2	(GA)12	24	26193457	26193480
chr10_4904	p2	(CA)10	20	26212057	26212076
chr10_4905	p2	(CA)10	20	26224571	26224590
chr10_4906	p2	(TG)9	18	26231468	26231485
chr10_4907	p2	(CA)17	34	26238578	26238611
chr10_4908	p2	(TG)9	18	26255991	26256008
chr10_4909	p2	(AC)6	12	26262368	26262379

chr10_4910	p2	(TG)6	12	26262706	26262717
chr10_4911	p2	(CA)6	12	26266039	26266050
chr10_4912	p2	(CT)19	38	26270718	26270755
chr10_4913	p3	(CTT)5	15	26276334	26276348
chr10_4914	p2	(AC)15	30	26277075	26277104
chr10_4915	p2	(TG)7	14	26281639	26281652
chr10_4916	p2	(TG)6	12	26282918	26282929
chr10_4917	p3	(CCT)6	18	26283189	26283206
chr10_4918	p3	(GAA)5	15	26294691	26294705
chr10_4919	p2	(AC)19	38	26304122	26304159
chr10_4920	p2	(AC)7	14	26305080	26305093
chr10_4921	p4	(TCAA)5	20	26305765	26305784
chr10_4922	p2	(CA)20	40	26306698	26306737
chr10_4923	p4	(TTTA)5	20	26307107	26307126
chr10_4924	c	(CT)15cact(CA)17	68	26320731	26320798
chr10_4925	p2	(TG)14	28	26322818	26322845
chr10_4926	p2	(AC)14	28	26324093	26324120
chr10_4927	p3	(TCT)5	15	26327680	26327694
chr10_4928	p3	(GAC)5	15	26331102	26331116
chr10_4929	p2	(CA)12	24	26335874	26335897
chr10_4930	p2	(GT)21	42	26336090	26336131
chr10_4931	p2	(AC)9	18	26336516	26336533
chr10_4932	p2	(AC)10	20	26343395	26343414
chr10_4933	p3	(GAG)5	15	26348538	26348552
chr10_4934	p2	(AT)6	12	26351101	26351112
chr10_4935	p2	(CA)6	12	26352006	26352017
chr10_4936	p2	(AG)6	12	26353214	26353225
chr10_4937	p2	(TG)7	14	26354654	26354667
chr10_4938	p2	(GT)10	20	26387909	26387928
chr10_4939	c	[illegible])23(AG)8attgtgtgagtgggtatgtgtgagggagtgagggtgaa(A[illegible]	112	26395122	26395233
chr10_4940	p2	(GT)6	12	26398933	26398944
chr10_4941	p2	(TG)17	34	26401801	26401834
chr10_4942	p3	(GTT)6	18	26409441	26409458
chr10_4943	p2	(TG)21	42	26424060	26424101
chr10_4944	p2	(CA)6	12	26424471	26424482
chr10_4945	p2	(AC)24	48	26429484	26429531
chr10_4946	p2	(AG)6	12	26440528	26440539
chr10_4947	p2	(TC)7	14	26444054	26444067
chr10_4948	p2	(TA)6	12	26444440	26444451
chr10_4949	p2	(AT)6	12	26453733	26453744
chr10_4950	p3	(TTC)5	15	26454621	26454635
chr10_4951	p3	(CAT)5	15	26467522	26467536
chr10_4952	p2	(CA)7	14	26478193	26478206
chr10_4953	p3	(GGA)5	15	26481858	26481872
chr10_4954	p2	(GT)8	16	26484499	26484514
chr10_4955	p4	(CCAT)5	20	26488807	26488826
chr10_4956	p2	(TA)6	12	26502633	26502644
chr10_4957	p2	(AT)6	12	26504110	26504121
chr10_4958	p2	(CA)6	12	26506051	26506062
chr10_4959	p3	(ATT)6	18	26535003	26535020
chr10_4960	c	(TG)26agtgt(GA)8	73	26538666	26538738
chr10_4961	p3	(ATT)8	24	26556007	26556030

chr10_4962	p2	(GT)6	12	26558938	26558949
chr10_4963	p3	(GTA)6	18	26563093	26563110
chr10_4964	p3	(TGT)5	15	26564452	26564466
chr10_4965	p3	(GTT)5	15	26568902	26568916
chr10_4966	p2	(TG)9	18	26570069	26570086
chr10_4967	p2	(AT)6	12	26577082	26577093
chr10_4968	p2	(TG)6	12	26578421	26578432
chr10_4969	p2	(TA)7	14	26578546	26578559
chr10_4970	p2	(CA)7	14	26586210	26586223
chr10_4971	c	(TG)9c(GT)6	31	26589748	26589778
chr10_4972	p2	(CA)14	28	26589884	26589911
chr10_4973	p2	(CA)10	20	26598893	26598912
chr10_4974	p3	(GTA)5	15	26607693	26607707
chr10_4975	p2	(TG)7	14	26615289	26615302
chr10_4976	p2	(GT)11	22	26661763	26661784
chr10_4977	p2	(GT)12	24	26670058	26670081
chr10_4978	p2	(GA)9	18	26670457	26670474
chr10_4979	p2	(CA)7	14	26685120	26685133
chr10_4980	p2	(GT)6	12	26690418	26690429
chr10_4981	p2	(AC)8	16	26691234	26691249
chr10_4982	p3	(GGA)8	24	26695356	26695379
chr10_4983	p3	(CTT)8	24	26729900	26729923
chr10_4984	p4	(AAAT)6	24	26734505	26734528
chr10_4985	p2	(AC)8	16	26748773	26748788
chr10_4986	p3	(GAG)5	15	26756447	26756461
chr10_4987	p3	(ATC)6	18	26775052	26775069
chr10_4988	p3	(CTT)7	21	26780720	26780740
chr10_4989	p2	(TA)10	20	26811653	26811672
chr10_4990	p3	(CTT)5	15	26816891	26816905
chr10_4991	c	[illegible](CA)9t(AC)7aaaccagggaaggacagaaggggaatggagagac[illegible]	149	26823746	26823894
chr10_4992	p2	(AT)6	12	26827182	26827193
chr10_4993	p2	(TG)15	30	26849202	26849231
chr10_4994	p2	(AC)7	14	26849979	26849992
chr10_4995	p4	(TTCA)7	28	26864281	26864308
chr10_4996	c	(GA)9(GT)6	30	26867760	26867789
chr10_4997	p2	(AC)8	16	26868595	26868610
chr10_4998	p2	(AC)13	26	26872226	26872251
chr10_4999	p2	(TG)16	32	26894792	26894823
chr10_5000	p2	(TA)6	12	26895374	26895385
chr10_5001	p2	(TC)10	20	26895641	26895660
chr10_5002	p2	(TG)10	20	26920457	26920476
chr10_5003	p2	(TG)15	30	26920693	26920722
chr10_5004	p2	(GT)18	36	26920836	26920871
chr10_5005	p2	(TG)12	24	26979432	26979455
chr10_5006	p5	(TTTTG)5	25	26983073	26983097
chr10_5007	p2	(AC)10	20	26995565	26995584
chr10_5008	p2	(AC)7	14	27005073	27005086
chr10_5009	p3	(TTA)5	15	27005789	27005803
chr10_5010	p2	(CA)13	26	27020330	27020355
chr10_5011	p2	(CT)7	14	27028584	27028597
chr10_5012	p2	(CA)11	22	27030877	27030898
chr10_5013	p2	(CA)9	18	27037743	27037760

chr10_5014	p2	(CA)17	34	27043472	27043505
chr10_5015	p2	(TG)6	12	27047206	27047217
chr10_5016	p2	(TG)11	22	27049298	27049319
chr10_5017	c	5cacattggccttctcttgtattcgacctcttcatctcacatggttggcta(GC	82	27050298	27050379
chr10_5018	p3	(GTT)5	15	27059615	27059629
chr10_5019	p2	(TC)8	16	27060007	27060022
chr10_5020	p2	(GT)6	12	27081217	27081228
chr10_5021	p4	(CCAT)5	20	27087635	27087654
chr10_5022	p2	(TG)6	12	27088592	27088603
chr10_5023	p2	(AC)11	22	27116616	27116637
chr10_5024	p2	(CA)11	22	27125336	27125357
chr10_5025	p3	(TTA)5	15	27130122	27130136
chr10_5026	p2	(TA)11	22	27130494	27130515
chr10_5027	p2	(AT)7	14	27145326	27145339
chr10_5028	p2	(CT)10	20	27146837	27146856
chr10_5029	p2	(AC)8	16	27151801	27151816
chr10_5030	p2	(AT)6	12	27177226	27177237
chr10_5031	p2	(CA)6	12	27199955	27199966
chr10_5032	p2	(AC)10	20	27210046	27210065
chr10_5033	p2	(TG)10	20	27210905	27210924
chr10_5034	p2	(CT)16	32	27212392	27212423
chr10_5035	p2	(GA)6	12	27214019	27214030
chr10_5036	p2	(TG)6	12	27223257	27223268
chr10_5037	p2	(CA)9	18	27231708	27231725
chr10_5038	p2	(CA)30	60	27232542	27232601
chr10_5039	p3	(ACA)5	15	27240396	27240410
chr10_5040	p2	(TA)8	16	27240958	27240973
chr10_5041	p2	(AC)9	18	27244129	27244146
chr10_5042	p3	(TTC)6	18	27250942	27250959
chr10_5043	p2	(GA)7	14	27265556	27265569
chr10_5044	p2	(CA)8	16	27272454	27272469
chr10_5045	p3	(TAT)5	15	27272789	27272803
chr10_5046	p5	(GCTTT)5	25	27278157	27278181
chr10_5047	c	(AGA)10ac(AAG)7	53	27279137	27279189
chr10_5048	c	(AGAC)5(AGAT)16	84	27290030	27290113
chr10_5049	p2	(CA)12	24	27298112	27298135
chr10_5050	p4	(TTTC)5	20	27300086	27300105
chr10_5051	p2	(CA)15	30	27303466	27303495
chr10_5052	p2	(GT)27	54	27320994	27321047
chr10_5053	c	aggagtgcagttgtttctgcatgtgtgtgcgagtctatcaaccatgcatgtaca	100	27321196	27321295
chr10_5054	p2	(AT)6	12	27321600	27321611
chr10_5055	p2	(AC)6	12	27324675	27324686
chr10_5056	p2	(TG)8	16	27326239	27326254
chr10_5057	p4	(TTTG)6	24	27329376	27329399
chr10_5058	p2	(CA)8	16	27330533	27330548
chr10_5059	p2	(TG)6	12	27339472	27339483
chr10_5060	p2	(GT)12	24	27349344	27349367
chr10_5061	p3	(AAT)5	15	27350017	27350031
chr10_5062	p2	(TC)6	12	27353203	27353214
chr10_5063	p2	(TC)6	12	27362162	27362173
chr10_5064	p2	(GT)6	12	27363316	27363327
chr10_5065	p2	(GT)22	44	27365353	27365396

chr10_5066	p2	(AC)10	20	27370945	27370964
chr10_5067	p3	(TAA)5	15	27373338	27373352
chr10_5068	p2	(GT)11	22	27415026	27415047
chr10_5069	p2	(CA)12	24	27420796	27420819
chr10_5070	p2	(GT)7	14	27421519	27421532
chr10_5071	p2	(TG)6	12	27423647	27423658
chr10_5072	p2	(TG)9	18	27439591	27439608
chr10_5073	p2	(CA)8	16	27442527	27442542
chr10_5074	p2	(AC)15	30	27442660	27442689
chr10_5075	p2	(TG)10	20	27443175	27443194
chr10_5076	p2	(AC)17	34	27467904	27467937
chr10_5077	p2	(TG)24	48	27476336	27476383
chr10_5078	p2	(TG)9	18	27478187	27478204
chr10_5079	p2	(AG)10	20	27480808	27480827
chr10_5080	p2	(CA)10	20	27481263	27481282
chr10_5081	p2	(GA)11	22	27483284	27483305
chr10_5082	p2	(AT)8	16	27486311	27486326
chr10_5083	p2	(AC)7	14	27499764	27499777
chr10_5084	p5	(AAATC)5	25	27501935	27501959
chr10_5085	p2	(TA)6	12	27507687	27507698
chr10_5086	p2	(AC)8	16	27511082	27511097
chr10_5087	p3	(TAA)8	24	27517826	27517849
chr10_5088	p2	(AC)8	16	27519233	27519248
chr10_5089	p2	(CA)15	30	27522345	27522374
chr10_5090	c*	(ATAC)5(AC)19*	56	27531148	27531203
chr10_5091	p2	(AC)7	14	27534775	27534788
chr10_5092	p3	(AGA)5	15	27537740	27537754
chr10_5093	c*	(GA)6(AGAA)6*	33	27542380	27542412
chr10_5094	p2	(TG)10	20	27552506	27552525
chr10_5095	p3	(AAC)5	15	27563359	27563373
chr10_5096	p2	(AC)6	12	27563720	27563731
chr10_5097	c	(TG)9agaa(AG)9	40	27575575	27575614
chr10_5098	p3	(AAC)5	15	27575803	27575817
chr10_5099	p2	(AC)7	14	27578391	27578404
chr10_5100	p2	(CT)16	32	27580179	27580210
chr10_5101	p3	(TTG)5	15	27581769	27581783
chr10_5102	c	(TC)9tttct(CA)9	41	27581973	27582013
chr10_5103	p2	(GT)6	12	27582493	27582504
chr10_5104	c	(TG)9agaa(AG)9	40	27588197	27588236
chr10_5105	p3	(AAC)5	15	27588425	27588439
chr10_5106	p2	(CA)17	34	27591909	27591942
chr10_5107	p2	(GT)6	12	27600663	27600674
chr10_5108	p2	(TG)7	14	27601158	27601171
chr10_5109	p2	(TG)7	14	27621283	27621296
chr10_5110	p2	(CA)15	30	27628800	27628829
chr10_5111	p4	(TATC)5	20	27635938	27635957
chr10_5112	p4	(TTAT)5	20	27640008	27640027
chr10_5113	p2	(AC)10	20	27646810	27646829
chr10_5114	c*	(TC)12actcg(CTCA)7(CA)6*	67	27653307	27653373
chr10_5115	p2	(TG)29	58	27667493	27667550
chr10_5116	p2	(TC)8	16	27672388	27672403
chr10_5117	p2	(TC)9	18	27694655	27694672

chr10_5118	c	(GA)6ctgtagat(GA)7	34	27710560	27710593
chr10_5119	p2	(AG)6	12	27712303	27712314
chr10_5120	p2	(CA)6	12	27712697	27712708
chr10_5121	p2	(GT)28	56	27715151	27715206
chr10_5122	p3	(AGT)11	33	27715903	27715935
chr10_5123	p2	(TG)6	12	27719158	27719169
chr10_5124	p2	(TG)9	18	27720921	27720938
chr10_5125	p2	(CA)7	14	27731229	27731242
chr10_5126	p3	(AGC)6	18	27737484	27737501
chr10_5127	p2	(AC)6	12	27744003	27744014
chr10_5128	p2	(AC)20	40	27745324	27745363
chr10_5129	p2	(AC)22	44	27751907	27751950
chr10_5130	p2	(GT)19	38	27753407	27753444
chr10_5131	p2	(AC)10	20	27759542	27759561
chr10_5132	p2	(AC)7	14	27766855	27766868
chr10_5133	p2	(TG)9	18	27783145	27783162
chr10_5134	p3	(ATC)5	15	27783350	27783364
chr10_5135	p4	(AATC)5	20	27783930	27783949
chr10_5136	p2	(AC)35	70	27794130	27794199
chr10_5137	p4	(AATA)5	20	27802761	27802780
chr10_5138	p2	(AG)6	12	27808845	27808856
chr10_5139	p3	(ATG)5	15	27815126	27815140
chr10_5140	p2	(AG)6	12	27816974	27816985
chr10_5141	p2	(CA)6	12	27844461	27844472
chr10_5142	p2	(CA)9	18	27845710	27845727
chr10_5143	c	[illegible]caccctggccatcagttgaaggagcccaaccaaggtaggtgggggggg[illegible]	107	27846430	27846536
chr10_5144	p2	(AC)8	16	27847135	27847150
chr10_5145	p3	(ATC)18	54	27849813	27849866
chr10_5146	p4	(TGTT)5	20	27851148	27851167
chr10_5147	p3	(CTC)9	27	27856262	27856288
chr10_5148	p2	(CA)6	12	27857125	27857136
chr10_5149	p3	(TCT)5	15	27859018	27859032
chr10_5150	c	gccagctgctgttatacaacattagttcttgtctttacacacaaacacctcact	131	27863799	27863929
chr10_5151	p2	(GA)6	12	27864524	27864535
chr10_5152	p2	(AC)11	22	27866132	27866153
chr10_5153	p2	(GT)13	26	27867596	27867621
chr10_5154	p2	(AC)7	14	27873152	27873165
chr10_5155	p3	(GCT)7	21	27877432	27877452
chr10_5156	p2	(AC)12	24	27877904	27877927
chr10_5157	p2	(TC)10	20	27879516	27879535
chr10_5158	c	(GT)12(GC)6	36	27887541	27887576
chr10_5159	p2	(AT)7	14	27887772	27887785
chr10_5160	p3	(TAT)7	21	27897180	27897200
chr10_5161	c	(ATG)9tattattattattctgctttt(GTA)9	75	27897343	27897417
chr10_5162	p3	(GAT)7	21	27902863	27902883
chr10_5163	p2	(AT)8	16	27904989	27905004
chr10_5164	p2	(AT)8	16	27905347	27905362
chr10_5165	p2	(AC)10	20	27918988	27919007
chr10_5166	p3	(TTC)5	15	27922451	27922465
chr10_5167	p2	(AC)9	18	27928593	27928610
chr10_5168	p2	(TA)7	14	27932357	27932370
chr10_5169	p2	(CA)12	24	27934697	27934720

chr10_5170	p3	(GTT)5	15	27952865	27952879
chr10_5171	p2	(AC)14	28	27962077	27962104
chr10_5172	p2	(AT)7	14	27962263	27962276
chr10_5173	p2	(TG)7	14	27967103	27967116
chr10_5174	p3	(AAT)6	18	27973337	27973354
chr10_5175	p2	(AC)6	12	27977160	27977171
chr10_5176	p2	(GT)6	12	27990980	27990991
chr10_5177	p2	(AC)30	60	28005667	28005726
chr10_5178	p2	(CA)6	12	28023757	28023768
chr10_5179	p5	(ATTTG)5	25	28032095	28032119
chr10_5180	p2	(AG)6	12	28040428	28040439
chr10_5181	p2	(CT)6	12	28053128	28053139
chr10_5182	p2	(AC)14	28	28053923	28053950
chr10_5183	p2	(AT)7	14	28056114	28056127
chr10_5184	p3	(TTA)5	15	28088782	28088796
chr10_5185	p2	(TG)6	12	28093123	28093134
chr10_5186	c	atttgtcatcttgatgagtacaacatataaccttgtaaaatatggagggctgta	117	28093343	28093459
chr10_5187	p2	(CA)6	12	28093723	28093734
chr10_5188	p2	(GA)6	12	28107771	28107782
chr10_5189	p2	(CA)14	28	28111101	28111128
chr10_5190	p2	(TG)13	26	28117851	28117876
chr10_5191	p2	(AC)8	16	28130950	28130965
chr10_5192	p2	(AC)11	22	28134997	28135018
chr10_5193	p2	(AC)7	14	28136158	28136171
chr10_5194	p3	(AGG)7	21	28140880	28140900
chr10_5195	p2	(AC)10	20	28163542	28163561
chr10_5196	p2	(TG)8	16	28163816	28163831
chr10_5197	p2	(TG)8	16	28169892	28169907
chr10_5198	p2	(TG)7	14	28174000	28174013
chr10_5199	p2	(TG)14	28	28174578	28174605
chr10_5200	p2	(AG)6	12	28176698	28176709
chr10_5201	p2	(CA)6	12	28182372	28182383
chr10_5202	p2	(AC)8	16	28187951	28187966
chr10_5203	p2	(CT)7	14	28193978	28193991
chr10_5204	p2	(CA)17	34	28197641	28197674
chr10_5205	p2	(AC)6	12	28200190	28200201
chr10_5206	c	(GA)11c(AG)7	37	28200363	28200399
chr10_5207	p2	(TG)6	12	28201207	28201218
chr10_5208	p2	(GT)10	20	28204017	28204036
chr10_5209	p2	(CT)10	20	28213676	28213695
chr10_5210	p2	(AC)6	12	28221900	28221911
chr10_5211	p2	(CA)19	38	28244840	28244877
chr10_5212	c	(AC)6tctctcct(TC)10	40	28256220	28256259
chr10_5213	p2	(AC)9	18	28259483	28259500
chr10_5214	p2	(TG)8	16	28260088	28260103
chr10_5215	c	ttccaggttgacgtggagacgaatgagtgtatgtgtgaggtgtgtttttcca	129	28272851	28272979
chr10_5216	p2	(AC)9	18	28276839	28276856
chr10_5217	p2	(CA)11	22	28278377	28278398
chr10_5218	p2	(CA)14	28	28278647	28278674
chr10_5219	p4	(TCCA)5	20	28283837	28283856
chr10_5220	p2	(CT)6	12	28292557	28292568
chr10_5221	p2	(AC)6	12	28293072	28293083

chr10_5222	c	(AC)11g(CA)13	49	28293236	28293284
chr10_5223	p2	(AC)6	12	28298490	28298501
chr10_5224	p2	(CA)13	26	28304913	28304938
chr10_5225	p2	(TG)8	16	28308207	28308222
chr10_5226	c	(GT)14gagt(GA)6	44	28310015	28310058
chr10_5227	p2	(TG)6	12	28310803	28310814
chr10_5228	c	(CTG)5ttgtgtgtgttgtgtcta(TG)14	61	28312308	28312368
chr10_5229	p2	(CA)12	24	28312499	28312522
chr10_5230	p2	(AC)7	14	28312793	28312806
chr10_5231	p2	(CA)8	16	28313894	28313909
chr10_5232	p2	(GT)6	12	28328798	28328809
chr10_5233	p2	(AC)9	18	28331083	28331100
chr10_5234	p2	(TG)6	12	28332790	28332801
chr10_5235	p2	(TG)11	22	28342674	28342695
chr10_5236	p3	(TTC)5	15	28343104	28343118
chr10_5237	c	ccatacacacccctctcactggttacccactgattattacacaccttacacat	85	28350829	28350913
chr10_5238	p2	(CA)6	12	28351040	28351051
chr10_5239	p2	(CA)12	24	28353783	28353806
chr10_5240	p2	(TG)11	22	28355468	28355489
chr10_5241	p2	(AC)19	38	28356062	28356099
chr10_5242	p2	(AC)13	26	28357919	28357944
chr10_5243	p2	(AC)6	12	28360947	28360958
chr10_5244	p2	(CA)7	14	28361066	28361079
chr10_5245	p2	(TC)6	12	28369381	28369392
chr10_5246	p2	(GT)6	12	28371454	28371465
chr10_5247	p2	(GT)11	22	28374945	28374966
chr10_5248	p2	(GT)19	38	28377818	28377855
chr10_5249	c	tacac(AT)6acatacatacac(AT)6acatacatacacatatatacata	421	28379799	28380219
chr10_5250	p2	(AG)7	14	28380986	28380999
chr10_5251	p2	(AG)6	12	28392319	28392330
chr10_5252	p2	(AC)6	12	28393197	28393208
chr10_5253	p2	(CA)14	28	28395107	28395134
chr10_5254	p2	(TG)6	12	28398152	28398163
chr10_5255	p2	(GT)11	22	28404352	28404373
chr10_5256	p2	(TG)16	32	28408120	28408151
chr10_5257	p2	(CA)12	24	28428569	28428592
chr10_5258	p2	(GA)6	12	28428701	28428712
chr10_5259	c	nnnnnnnnnnnnnnnnnnnnnnnnnnnnnnnnnnnnnnnnnnnnnn	155	28433007	28433161
chr10_5260	p2	(GA)6	12	28441385	28441396
chr10_5261	p3	(CAG)5	15	28456752	28456766
chr10_5262	p2	(TG)7	14	28462105	28462118
chr10_5263	p3	(CTA)8	24	28470196	28470219
chr10_5264	p4	(AAAT)5	20	28482964	28482983
chr10_5265	p3	(GAG)6	18	28486000	28486017
chr10_5266	p2	(AC)8	16	28492670	28492685
chr10_5267	p3	(AAG)5	15	28496864	28496878
chr10_5268	p2	(AC)12	24	28514637	28514660
chr10_5269	p2	(AC)6	12	28517874	28517885
chr10_5270	p3	(TAA)6	18	28519918	28519935
chr10_5271	p2	(TA)10	20	28527920	28527939
chr10_5272	p2	(AT)6	12	28532549	28532560
chr10_5273	p2	(GA)9	18	28551522	28551539

chr10_5274	p2	(GT)10	20	28552466	28552485
chr10_5275	p2	(CA)6	12	28557901	28557912
chr10_5276	p2	(AC)15	30	28558286	28558315
chr10_5277	p2	(AC)18	36	28561683	28561718
chr10_5278	c	(GT)16gaga(GT)7	50	28577183	28577232
chr10_5279	p2	(CA)16	32	28582766	28582797
chr10_5280	c	(GT)17t(TG)9tttgtccatctgtatctgaatc(TG)7	89	28587818	28587906
chr10_5281	c	(CA)11tacat(AC)7	41	28588023	28588063
chr10_5282	p2	(GA)6	12	28590771	28590782
chr10_5283	p2	(GT)13	26	28606719	28606744
chr10_5284	p2	(TC)11	22	28611702	28611723
chr10_5285	p2	(CA)6	12	28615515	28615526
chr10_5286	c	(ATAG)9atc(GATA)7	67	28616458	28616524
chr10_5287	p2	(TC)12	24	28624498	28624521
chr10_5288	p2	(CA)6	12	28634153	28634164
chr10_5289	p2	(AC)14	28	28634302	28634329
chr10_5290	p2	(TG)6	12	28634643	28634654
chr10_5291	p2	(GT)6	12	28667616	28667627
chr10_5292	p2	(AC)6	12	28667929	28667940
chr10_5293	p2	(TG)22	44	28668150	28668193
chr10_5294	p2	(AC)6	12	28669045	28669056
chr10_5295	p2	(TC)6	12	28675375	28675386
chr10_5296	p3	(AAT)6	18	28693551	28693568
chr10_5297	p2	(AC)10	20	28697172	28697191
chr10_5298	p3	(AAT)5	15	28707311	28707325
chr10_5299	p2	(GT)6	12	28710922	28710933
chr10_5300	p2	(TG)6	12	28718851	28718862
chr10_5301	p2	(GT)7	14	28719036	28719049
chr10_5302	p4	(CCAT)5	20	28734786	28734805
chr10_5303	p2	(TG)6	12	28751347	28751358
chr10_5304	p2	(CA)20	40	28759590	28759629
chr10_5305	p2	(AC)6	12	28759783	28759794
chr10_5306	p4	(AAAT)5	20	28769356	28769375
chr10_5307	p2	(TG)12	24	28782606	28782629
chr10_5308	p2	(AC)8	16	28783929	28783944
chr10_5309	p2	(AC)10	20	28784520	28784539
chr10_5310	p2	(AC)12	24	28794884	28794907
chr10_5311	p2	(AC)6	12	28798487	28798498
chr10_5312	p5	(TCCTC)7	35	28805400	28805434
chr10_5313	p2	(AC)13	26	28806530	28806555
chr10_5314	p2	(TG)6	12	28809207	28809218
chr10_5315	p2	(TG)13	26	28816247	28816272
chr10_5316	c	ggttccaggtcactttagcccaatagagtggactgcttggcaactagcgat	125	28822429	28822553
chr10_5317	p3	(TGC)7	21	28825614	28825634
chr10_5318	p2	(TG)7	14	28842784	28842797
chr10_5319	p2	(TG)6	12	28848279	28848290
chr10_5320	c	gttttgggctgttggtgcgaaatagggcctgataatagcagg(GT)9ggg	148	28856671	28856818
chr10_5321	p2	(AC)14	28	28857297	28857324
chr10_5322	c	accatgcgctagctgttccctgctgtggcgtacatgctaaacccacaccaat	124	28858769	28858892
chr10_5323	p2	(AG)8	16	28861042	28861057
chr10_5324	p2	(TG)6	12	28863654	28863665
chr10_5325	p2	(TG)22	44	28890451	28890494

chr10_5326	p2	(TC)10	20	28891187	28891206
chr10_5327	p2	(GA)6	12	28892207	28892218
chr10_5328	p2	(TG)6	12	28897276	28897287
chr10_5329	p2	(GT)6	12	28898336	28898347
chr10_5330	c	tgannnnnnnnnnnnnnnnnnnnnnntacgtgtgttagctccccc	100	28898678	28898777
chr10_5331	p2	(AC)19	38	28900198	28900235
chr10_5332	p3	(TTA)5	15	28901439	28901453
chr10_5333	p2	(CA)6	12	28902543	28902554
chr10_5334	p4	(ATTG)6	24	28904924	28904947
chr10_5335	c	gcctgcagtctcagccagcgctacatctaagcagctaaactgctgctacct	125	28911823	28911947
chr10_5336	p2	(TC)7	14	28912217	28912230
chr10_5337	p5	(AGTAG)11	55	28914740	28914794
chr10_5338	c	tcttcagtgtttaccaaggatcatcgctactggagggtgtgtgtaaatgcag	124	28920495	28920618
chr10_5339	p2	(AC)9	18	28920873	28920890
chr10_5340	p2	(TG)7	14	28924271	28924284
chr10_5341	p2	(GT)6	12	28935155	28935166
chr10_5342	p3	(GAT)11	33	28944830	28944862
chr10_5343	p2	(GA)6	12	28956762	28956773
chr10_5344	p2	(AG)6	12	28961746	28961757
chr10_5345	p2	(TG)12	24	28961886	28961909
chr10_5346	p2	(GT)10	20	28963060	28963079
chr10_5347	p2	(TG)13	26	28977821	28977846
chr10_5348	p3	(AAG)7	21	28987972	28987992
chr10_5349	p2	(TG)7	14	28990805	28990818
chr10_5350	p2	(AC)16	32	28993794	28993825
chr10_5351	p2	(AT)7	14	28998805	28998818
chr10_5352	c	(TG)10taattcccaaaata(TG)7	48	28998963	28999010
chr10_5353	p3	(TAT)5	15	29000447	29000461
chr10_5354	p2	(CA)8	16	29002394	29002409
chr10_5355	p2	(GT)15	30	29011039	29011068
chr10_5356	p2	(AC)16	32	29013685	29013716
chr10_5357	p2	(TG)6	12	29015875	29015886
chr10_5358	p2	(TA)8	16	29019115	29019130
chr10_5359	p2	(AC)6	12	29028617	29028628
chr10_5360	p2	(AC)12	24	29031807	29031830
chr10_5361	p2	(TG)17	34	29041778	29041811
chr10_5362	p2	(TG)6	12	29042020	29042031
chr10_5363	p3	(TTA)6	18	29043301	29043318
chr10_5364	p4	(CCAT)5	20	29046510	29046529
chr10_5365	c	(TG)9tc(TG)18	56	29048112	29048167
chr10_5366	p2	(CA)14	28	29048419	29048446
chr10_5367	p2	(AC)12	24	29048557	29048580
chr10_5368	p2	(GT)6	12	29052038	29052049
chr10_5369	c	(AG)6actatttata(TG)16	54	29055146	29055199
chr10_5370	p2	(TG)7	14	29065797	29065810
chr10_5371	p2	(AC)8	16	29067755	29067770
chr10_5372	p2	(AC)6	12	29070024	29070035
chr10_5373	p2	(AG)6	12	29074584	29074595
chr10_5374	c	(TG)14agt(GA)11	53	29079908	29079960
chr10_5375	p2	(CA)6	12	29081534	29081545
chr10_5376	p2	(GA)24	48	29083302	29083349
chr10_5377	p2	(TG)6	12	29092632	29092643

chr10_5378	p2	(GT)9	18	29093867	29093884
chr10_5379	p4	(AATG)5	20	29096399	29096418
chr10_5380	p2	(AC)11	22	29098222	29098243
chr10_5381	p2	(GA)7	14	29106845	29106858
chr10_5382	p2	(AC)11	22	29107049	29107070
chr10_5383	p2	(AC)21	42	29115315	29115356
chr10_5384	p4	(CAAC)5	20	29115458	29115477
chr10_5385	p2	(AC)8	16	29124581	29124596
chr10_5386	p4	(ATTG)6	24	29126002	29126025
chr10_5387	p2	(GA)7	14	29129480	29129493
chr10_5388	c	)7ttcacttatctcatcatccttctccttttacctaataccagctctctctcaac(	79	29141683	29141761
chr10_5389	p2	(GT)6	12	29144520	29144531
chr10_5390	p2	(CT)7	14	29162569	29162582
chr10_5391	p2	(AC)8	16	29163683	29163698
chr10_5392	c	(GT)6atactgtacttgtagtgtga(TG)7	46	29163823	29163868
chr10_5393	p3	(TTA)6	18	29168000	29168017
chr10_5394	p2	(TG)7	14	29170557	29170570
chr10_5395	p2	(GT)12	24	29177791	29177814
chr10_5396	p2	(TG)6	12	29193485	29193496
chr10_5397	p2	(GA)13	26	29197213	29197238
chr10_5398	p2	(TG)23	46	29209795	29209840
chr10_5399	p2	(TG)9	18	29210615	29210632
chr10_5400	p2	(GT)6	12	29225311	29225322
chr10_5401	p2	(AT)8	16	29243705	29243720
chr10_5402	p2	(AC)8	16	29244941	29244956
chr10_5403	p2	(TG)10	20	29248798	29248817
chr10_5404	p2	(TA)6	12	29252459	29252470
chr10_5405	p2	(CA)7	14	29255199	29255212
chr10_5406	c	(CT)6atttcaa(AC)7	33	29256151	29256183
chr10_5407	p2	(AC)9	18	29260501	29260518
chr10_5408	p2	(CA)8	16	29261221	29261236
chr10_5409	p2	(CA)8	16	29263341	29263356
chr10_5410	p2	(TG)9	18	29265662	29265679
chr10_5411	c	10ctgtgtgtgtctgtgtgtatatatgtatatgtcagtttttgaacaattcctt(T	90	29271111	29271200
chr10_5412	p2	(TC)6	12	29271639	29271650
chr10_5413	p2	(AC)8	16	29272130	29272145
chr10_5414	p2	(AC)6	12	29272569	29272580
chr10_5415	p2	(TG)6	12	29282252	29282263
chr10_5416	p3	(CTT)5	15	29284065	29284079
chr10_5417	p2	(TA)7	14	29287750	29287763
chr10_5418	p2	(TG)12	24	29292359	29292382
chr10_5419	p2	(TG)10	20	29300804	29300823
chr10_5420	p2	(CA)10	20	29314973	29314992
chr10_5421	p4	(CATT)5	20	29332020	29332039
chr10_5422	p2	(TG)6	12	29332408	29332419
chr10_5423	p2	(TG)6	12	29335225	29335236
chr10_5424	p3	(AGA)5	15	29351238	29351252
chr10_5425	p2	(TG)21	42	29354902	29354943
chr10_5426	p2	(AC)6	12	29356487	29356498
chr10_5427	p2	(CA)9	18	29363138	29363155
chr10_5428	p2	(TG)7	14	29364199	29364212
chr10_5429	c	(TG)6ta(TG)10	34	29377888	29377921

chr10_5430	p2	(CA)13	26	29378243	29378268
chr10_5431	p2	(AC)15	30	29380802	29380831
chr10_5432	p2	(AG)6	12	29382620	29382631
chr10_5433	p4	(CCAT)5	20	29389872	29389891
chr10_5434	p4	(CCAT)5	20	29399644	29399663
chr10_5435	p2	(AT)7	14	29404214	29404227
chr10_5436	c	(TG)8(AG)10	36	29404332	29404367
chr10_5437	p2	(TG)13	26	29405398	29405423
chr10_5438	p3	(GTT)8	24	29410669	29410692
chr10_5439	c	(CT)35(CA)13	96	29414944	29415039
chr10_5440	p2	(GT)6	12	29420388	29420399
chr10_5441	c	(AG)8aa(AG)8	34	29425830	29425863
chr10_5442	p2	(AC)12	24	29429278	29429301
chr10_5443	c	(TG)8(AG)13	42	29431499	29431540
chr10_5444	p2	(CA)7	14	29434708	29434721
chr10_5445	p2	(AC)6	12	29443895	29443906
chr10_5446	p2	(TG)8	16	29456948	29456963
chr10_5447	p2	(AG)6	12	29457867	29457878
chr10_5448	p2	(CA)12	24	29460631	29460654
chr10_5449	p2	(TG)7	14	29461045	29461058
chr10_5450	c	(GT)7gagt(GA)9	36	29461164	29461199
chr10_5451	p2	(TG)6	12	29461469	29461480
chr10_5452	p2	(CA)6	12	29483025	29483036
chr10_5453	p2	(TG)16	32	29485988	29486019
chr10_5454	p2	(TA)12	24	29489748	29489771
chr10_5455	p3	(CAT)6	18	29501990	29502007
chr10_5456	p2	(CA)8	16	29504261	29504276
chr10_5457	p2	(CA)7	14	29504413	29504426
chr10_5458	c	(TG)6agtgagaa(AG)8	36	29510497	29510532
chr10_5459	p2	(CA)20	40	29519194	29519233
chr10_5460	c	(TA)6atgtattttttagaag(GT)6	41	29522885	29522925
chr10_5461	p2	(TG)12	24	29523855	29523878
chr10_5462	p4	(TGAT)7	28	29527112	29527139
chr10_5463	p2	(AC)6	12	29530613	29530624
chr10_5464	p3	(ATT)8	24	29533211	29533234
chr10_5465	p2	(GT)6	12	29539725	29539736
chr10_5466	p2	(TG)10	20	29539990	29540009
chr10_5467	p2	(CA)8	16	29544946	29544961
chr10_5468	p2	(TG)12	24	29546728	29546751
chr10_5469	c	[illegible]catggcagtacattgtgagaattactttgatttggcgtgaggatattgccag[illegible]	98	29548814	29548911
chr10_5470	p2	(TG)6	12	29549335	29549346
chr10_5471	p2	(AG)6	12	29551518	29551529
chr10_5472	c	[illegible]6atgcacgcatgcatacatatacacacagagcatgcaccagcaggggt[illegible]	92	29553010	29553101
chr10_5473	p2	(AG)13	26	29555434	29555459
chr10_5474	c	(AG)7c(GA)7	29	29556258	29556286
chr10_5475	p2	(TG)6	12	29560147	29560158
chr10_5476	p2	(CA)14	28	29570393	29570420
chr10_5477	p2	(CA)7	14	29573194	29573207
chr10_5478	p2	(GA)7	14	29579475	29579488
chr10_5479	c	(CA)11cg(CA)6	36	29584363	29584398
chr10_5480	c	(TG)8cgcgcgcg(CA)15	54	29585300	29585353
chr10_5481	p2	(AC)15	30	29587374	29587403

chr10_5482	p6	(ACATTT)5	30	29590421	29590450
chr10_5483	p2	(CA)6	12	29606899	29606910
chr10_5484	c	(GT)6g(GT)21	55	29607146	29607200
chr10_5485	p2	(GT)9	18	29607436	29607453
chr10_5486	p2	(TG)26	52	29621118	29621169
chr10_5487	p2	(TG)8	16	29623446	29623461
chr10_5488	p2	(TG)9	18	29624793	29624810
chr10_5489	p2	(TG)6	12	29625313	29625324
chr10_5490	p3	(TTA)5	15	29632965	29632979
chr10_5491	p2	(TG)7	14	29665761	29665774
chr10_5492	p2	(AC)8	16	29665961	29665976
chr10_5493	p5	(ATAAA)5	25	29671853	29671877
chr10_5494	p2	(CA)17	34	29678739	29678772
chr10_5495	p2	(AC)14	28	29681664	29681691
chr10_5496	p2	(GT)9	18	29682306	29682323
chr10_5497	p2	(TG)6	12	29683343	29683354
chr10_5498	p2	(AG)13	26	29689178	29689203
chr10_5499	p2	(GT)13	26	29690193	29690218
chr10_5500	p3	(ATT)5	15	29694137	29694151
chr10_5501	p3	(GGT)5	15	29694262	29694276
chr10_5502	p2	(AG)13	26	29697488	29697513
chr10_5503	p2	(TG)15	30	29700047	29700076
chr10_5504	p2	(CA)20	40	29748468	29748507
chr10_5505	p2	(TG)8	16	29756308	29756323
chr10_5506	p4	(TGTC)5	20	29757980	29757999
chr10_5507	p2	(TG)15	30	29790068	29790097
chr10_5508	p2	(AT)9	18	29805352	29805369
chr10_5509	p3	(ATA)7	21	29808341	29808361
chr10_5510	p3	(TAA)5	15	29826598	29826612
chr10_5511	p2	(AT)6	12	29833541	29833552
chr10_5512	p2	(CA)14	28	29838715	29838742
chr10_5513	p2	(TA)7	14	29848204	29848217
chr10_5514	p2	(AC)8	16	29850204	29850219
chr10_5515	p3	(TAA)6	18	29863603	29863620
chr10_5516	p2	(AC)6	12	29867920	29867931
chr10_5517	c	(AT)6ac(AT)9	32	29873732	29873763
chr10_5518	p2	(AC)7	14	29876529	29876542
chr10_5519	p2	(AC)6	12	29887843	29887854
chr10_5520	c	(AT)14annnnnnnnnnnntacat(AC)17	80	29890918	29890997
chr10_5521	p2	(TG)12	24	29903655	29903678
chr10_5522	p2	(CA)25	50	29907353	29907402
chr10_5523	p2	(AC)12	24	29910106	29910129
chr10_5524	p2	(AC)11	22	29929071	29929092
chr10_5525	c	(CA)6cggactcacaggtccccagagagatgag(CA)9	58	29932656	29932713
chr10_5526	p2	(GA)8	16	29944637	29944652
chr10_5527	p4	(ATGG)5	20	29962330	29962349
chr10_5528	p3	(CTG)5	15	29973301	29973315
chr10_5529	p2	(AG)9	18	29976422	29976439
chr10_5530	p2	(CA)17	34	29977758	29977791
chr10_5531	p2	(TG)9	18	29980678	29980695
chr10_5532	p2	(CA)8	16	29984151	29984166
chr10_5533	p2	(TC)6	12	29992649	29992660

chr10_5534	p2	(TC)6	12	29997202	29997213
chr10_5535	p2	(TA)6	12	30004982	30004993
chr10_5536	p2	(GC)6	12	30008581	30008592
chr10_5537	p2	(TA)9	18	30012339	30012356
chr10_5538	p2	(CA)13	26	30012936	30012961
chr10_5539	p2	(TA)10	20	30014854	30014873
chr10_5540	p2	(AT)6	12	30019113	30019124
chr10_5541	p2	(TG)6	12	30039113	30039124
chr10_5542	p2	(CA)30	60	30040094	30040153
chr10_5543	p3	(AAT)5	15	30040675	30040689
chr10_5544	p2	(GT)8	16	30056839	30056854
chr10_5545	p2	(GT)18	36	30066764	30066799
chr10_5546	p2	(TG)15	30	30066999	30067028
chr10_5547	p5	(GATTT)5	25	30069739	30069763
chr10_5548	p2	(AG)6	12	30071513	30071524
chr10_5549	c	ataacacatacagggctaactctgtgaacctcccccccatcactctcattca	114	30071701	30071814
chr10_5550	p3	(CTC)6	18	30076577	30076594
chr10_5551	p2	(GT)6	12	30079824	30079835
chr10_5552	p3	(AAT)6	18	30106864	30106881
chr10_5553	p3	(GAG)5	15	30160014	30160028
chr10_5554	p2	(AC)9	18	30175217	30175234
chr10_5555	p4	(AGAT)32	128	30189681	30189808
chr10_5556	p2	(GT)6	12	30195553	30195564
chr10_5557	p4	(AATA)5	20	30197533	30197552
chr10_5558	p2	(CA)9	18	30198477	30198494
chr10_5559	p2	(CA)8	16	30217098	30217113
chr10_5560	p2	(AT)9	18	30225680	30225697
chr10_5561	p2	(AT)6	12	30233808	30233819
chr10_5562	p2	(TG)12	24	30238621	30238644
chr10_5563	p3	(TTA)5	15	30249004	30249018
chr10_5564	p2	(TG)6	12	30292259	30292270
chr10_5565	p2	(CA)11	22	30296198	30296219
chr10_5566	p3	(TAT)6	18	30299530	30299547
chr10_5567	p2	(AC)6	12	30299847	30299858
chr10_5568	p2	(GA)9	18	30303293	30303310
chr10_5569	p2	(CA)14	28	30306745	30306772
chr10_5570	p2	(TG)20	40	30313789	30313828
chr10_5571	p2	(GT)11	22	30316058	30316079
chr10_5572	p2	(GT)7	14	30317349	30317362
chr10_5573	p2	(GT)17	34	30317643	30317676
chr10_5574	p4	(CTGA)6	24	30321260	30321283
chr10_5575	p2	(CA)6	12	30321770	30321781
chr10_5576	p2	(GT)7	14	30322570	30322583
chr10_5577	p2	(AC)7	14	30326640	30326653
chr10_5578	c	cgtgaccctgtgttgttctttggagagttggtttgagattaagactggagttct	120	30330757	30330876
chr10_5579	p2	(TA)6	12	30331410	30331421
chr10_5580	p2	(TG)6	12	30334378	30334389
chr10_5581	p2	(AC)8	16	30337753	30337768
chr10_5582	p2	(TG)10	20	30345074	30345093
chr10_5583	p2	(TG)8	16	30351563	30351578
chr10_5584	p2	(CA)8	16	30361403	30361418
chr10_5585	p2	(TG)15	30	30374190	30374219

chr10_5586	p3	(CCT)5	15	30391326	30391340
chr10_5587	p2	(TG)6	12	30399597	30399608
chr10_5588	p2	(TG)7	14	30400158	30400171
chr10_5589	p2	(AC)6	12	30409604	30409615
chr10_5590	p2	(GT)11	22	30416479	30416500
chr10_5591	p2	(GT)10	20	30418740	30418759
chr10_5592	p5	(CACAG)5	25	30421252	30421276
chr10_5593	p2	(TA)7	14	30436004	30436017
chr10_5594	p2	(TG)19	38	30450117	30450154
chr10_5595	p2	(GT)8	16	30451829	30451844
chr10_5596	p2	(TG)13	26	30457307	30457332
chr10_5597	p2	(TG)11	22	30460038	30460059
chr10_5598	p4	(TTTA)5	20	30463168	30463187
chr10_5599	c	ggctgaagggagt(GGA)5aaaagaggcggccagtggtaaaagcttc	228	30466951	30467178
chr10_5600	p2	(CA)6	12	30471365	30471376
chr10_5601	p2	(TA)6	12	30483717	30483728
chr10_5602	p3	(TAT)6	18	30492792	30492809
chr10_5603	p3	(TGA)6	18	30495941	30495958
chr10_5604	p3	(TTG)6	18	30499284	30499301
chr10_5605	p2	(AC)16	32	30503691	30503722
chr10_5606	p2	(AG)7	14	30526044	30526057
chr10_5607	p2	(AC)6	12	30539901	30539912
chr10_5608	p2	(GT)6	12	30540620	30540631
chr10_5609	c	(GT)15c(TG)6	43	30560559	30560601
chr10_5610	p2	(TG)11	22	30560854	30560875
chr10_5611	p2	(TG)6	12	30563269	30563280
chr10_5612	c	(CA)18cg(CA)6	50	30577303	30577352
chr10_5613	p3	(GCT)6	18	30578671	30578688
chr10_5614	p2	(CA)6	12	30603822	30603833
chr10_5615	p2	(TG)20	40	30609737	30609776
chr10_5616	p2	(CA)14	28	30626041	30626068
chr10_5617	p2	(AG)10	20	30628679	30628698
chr10_5618	p3	(ATT)7	21	30631694	30631714
chr10_5619	p2	(AC)13	26	30641077	30641102
chr10_5620	p2	(GT)6	12	30641699	30641710
chr10_5621	p2	(AG)7	14	30643208	30643221
chr10_5622	p2	(GT)6	12	30648473	30648484
chr10_5623	p2	(GT)6	12	30663857	30663868
chr10_5624	p3	(GAG)5	15	30710534	30710548
chr10_5625	p2	(GT)14	28	30711928	30711955
chr10_5626	p2	(AC)14	28	30712332	30712359
chr10_5627	p2	(CA)7	14	30715555	30715568
chr10_5628	p2	(GT)17	34	30721430	30721463
chr10_5629	p2	(AC)6	12	30721869	30721880
chr10_5630	p2	(AC)7	14	30726472	30726485
chr10_5631	p3	(CAG)9	27	30728730	30728756
chr10_5632	p2	(TG)15	30	30731082	30731111
chr10_5633	p2	(CA)8	16	30741806	30741821
chr10_5634	p3	(TTC)5	15	30749722	30749736
chr10_5635	p2	(GA)7	14	30751443	30751456
chr10_5636	p2	(AC)6	12	30755656	30755667
chr10_5637	p2	(TG)12	24	30777957	30777980

chr10_5638	c	ggattcagcataatcatgcattaagaagtagtggatgtctgtaaagatgtggc	103	30790086	30790188
chr10_5639	p2	(AC)6	12	30793139	30793150
chr10_5640	p2	(AC)6	12	30798826	30798837
chr10_5641	p3	(ATC)6	18	30803552	30803569
chr10_5642	p2	(GT)27	54	30808199	30808252
chr10_5643	p2	(TA)12	24	30808395	30808418
chr10_5644	p2	(TG)12	24	30816802	30816825
chr10_5645	p2	(AC)6	12	30845821	30845832
chr10_5646	c	(AAC)7aa(AAC)7	44	30856287	30856330
chr10_5647	c	(CT)7t(TC)8	31	30859062	30859092
chr10_5648	p4	(ATGG)6	24	30863034	30863057
chr10_5649	p2	(AC)6	12	30870575	30870586
chr10_5650	p2	(AC)6	12	30875220	30875231
chr10_5651	p3	(TCT)7	21	30876357	30876377
chr10_5652	p2	(AC)12	24	30882616	30882639
chr10_5653	p2	(GT)8	16	30891544	30891559
chr10_5654	p2	(AC)12	24	30891754	30891777
chr10_5655	p3	(CTG)5	15	30892253	30892267
chr10_5656	c	(AGC)6aataataataaata(AAT)5	47	30893517	30893563
chr10_5657	p2	(AC)6	12	30899257	30899268
chr10_5658	p3	(GAA)10	30	30904626	30904655
chr10_5659	p2	(TG)10	20	30913806	30913825
chr10_5660	p2	(GT)6	12	30935875	30935886
chr10_5661	p2	(AC)20	40	30936075	30936114
chr10_5662	p2	(TG)25	50	30938099	30938148
chr10_5663	p3	(TCT)5	15	30979340	30979354
chr10_5664	p2	(TG)11	22	30984941	30984962
chr10_5665	p2	(TA)9	18	30990186	30990203
chr10_5666	p2	(CA)6	12	30998041	30998052
chr10_5667	p4	(TCTG)5	20	31003573	31003592
chr10_5668	c	.TT)5tactgagcagagactgggagaatgaacacaggtgaaaaattta(A	83	31013728	31013810
chr10_5669	p4	(GATT)7	28	31015915	31015942
chr10_5670	p2	(TC)6	12	31025830	31025841
chr10_5671	p2	(AC)8	16	31044374	31044389
chr10_5672	c	5acatatcaggcaatatttaaaaaaacagtttcagatgacttgtaaatgcttt(	82	31046431	31046512
chr10_5673	p4	(TTTC)5	20	31052445	31052464
chr10_5674	p3	(CTG)6	18	31063466	31063483
chr10_5675	p2	(CA)26	52	31069706	31069757
chr10_5676	p5	(GTCAA)5	25	31070988	31071012
chr10_5677	p4	(TCCA)5	20	31075354	31075373
chr10_5678	p2	(AC)6	12	31077976	31077987
chr10_5679	c	acatgcaggcaggcatgcggaaccatcacacaagaacatgtcgaaccatt	107	31078537	31078643
chr10_5680	p5	(TTTGA)6	30	31083706	31083735
chr10_5681	p2	(GA)7	14	31088441	31088454
chr10_5682	p3	(TCT)5	15	31088784	31088798
chr10_5683	p2	(AC)9	18	31089721	31089738
chr10_5684	p2	(CA)22	44	31089934	31089977
chr10_5685	p2	(CT)8	16	31091751	31091766
chr10_5686	p2	(AG)7	14	31093526	31093539
chr10_5687	p2	(TG)9	18	31096785	31096802
chr10_5688	p2	(GT)8	16	31108233	31108248
chr10_5689	p3	(AAT)5	15	31117622	31117636

chr10_5690	p2	(CT)6	12	31122962	31122973
chr10_5691	p2	(TG)6	12	31139613	31139624
chr10_5692	p2	(GA)8	16	31141947	31141962
chr10_5693	c	(TC)12tg(TC)11	48	31154859	31154906
chr10_5694	p2	(TG)6	12	31158180	31158191
chr10_5695	p2	(CA)8	16	31158342	31158357
chr10_5696	p2	(CT)15	30	31171196	31171225
chr10_5697	p4	(AATC)7	28	31184908	31184935
chr10_5698	p3	(TAT)7	21	31185774	31185794
chr10_5699	p2	(TG)10	20	31189180	31189199
chr10_5700	p2	(AC)6	12	31196238	31196249
chr10_5701	p2	(GA)7	14	31223571	31223584
chr10_5702	p4	(GATG)7	28	31234473	31234500
chr10_5703	c	(AGA)6ggaagaagaagcaacaac(AGA)6	54	31238263	31238316
chr10_5704	p2	(AC)15	30	31262129	31262158
chr10_5705	p2	(TG)16	32	31279423	31279454
chr10_5706	p2	(TG)7	14	31281356	31281369
chr10_5707	c	(TG)6catacaggctcata(TG)6	38	31286011	31286048
chr10_5708	p2	(AC)8	16	31292738	31292753
chr10_5709	p2	(TG)18	36	31296771	31296806
chr10_5710	p2	(CA)10	20	31297928	31297947
chr10_5711	p4	(GATA)6	24	31299998	31300021
chr10_5712	p2	(AG)17	34	31322648	31322681
chr10_5713	p2	(CA)10	20	31329774	31329793
chr10_5714	p2	(CA)6	12	31330366	31330377
chr10_5715	p2	(AC)23	46	31335783	31335828
chr10_5716	p3	(ATT)5	15	31336732	31336746
chr10_5717	p2	(AG)7	14	31337056	31337069
chr10_5718	p4	(CAGC)6	24	31351375	31351398
chr10_5719	p2	(AG)11	22	31354824	31354845
chr10_5720	p2	(GA)12	24	31356843	31356866
chr10_5721	p3	(AGG)5	15	31360764	31360778
chr10_5722	p3	(GTG)7	21	31360886	31360906
chr10_5723	p5	(AAATC)5	25	31363857	31363881
chr10_5724	p3	(TGC)5	15	31366478	31366492
chr10_5725	p2	(AT)6	12	31367678	31367689
chr10_5726	p2	(AC)7	14	31373228	31373241
chr10_5727	p4	(GATA)9	36	31381207	31381242
chr10_5728	p2	(TA)6	12	31390530	31390541
chr10_5729	p3	(TAA)10	30	31416083	31416112
chr10_5730	p2	(TA)9	18	31424302	31424319
chr10_5731	p3	(ACA)6	18	31442609	31442626
chr10_5732	p2	(CA)6	12	31444441	31444452
chr10_5733	p2	(CA)10	20	31452653	31452672
chr10_5734	p2	(CA)6	12	31490014	31490025
chr10_5735	p3	(TAA)6	18	31492652	31492669
chr10_5736	c	(AAT)6agtttatataaattaac(TAT)7	56	31499465	31499520
chr10_5737	p2	(AC)14	28	31505256	31505283
chr10_5738	p3	(TAT)8	24	31512945	31512968
chr10_5739	p4	(TCCA)5	20	31514925	31514944
chr10_5740	p2	(TC)6	12	31517097	31517108
chr10_5741	p2	(TA)8	16	31525384	31525399

chr10_5742	p2	(AG)8	16	31527010	31527025
chr10_5743	p3	(CTT)6	18	31532743	31532760
chr10_5744	c	[illegible]ttgtgtggaccaaaacattgttactcactctggggactcggctcagggttga	162	31537895	31538056
chr10_5745	p2	(AC)6	12	31544077	31544088
chr10_5746	p3	(GAA)5	15	31545071	31545085
chr10_5747	p2	(AG)7	14	31561342	31561355
chr10_5748	p2	(TA)7	14	31565172	31565185
chr10_5749	p5	(ATTTG)5	25	31574165	31574189
chr10_5750	p3	(TAA)6	18	31580709	31580726
chr10_5751	p2	(AC)7	14	31584728	31584741
chr10_5752	p2	(GA)7	14	31587831	31587844
chr10_5753	p2	(GT)21	42	31592007	31592048
chr10_5754	p2	(AC)7	14	31598382	31598395
chr10_5755	p2	(AT)6	12	31599419	31599430
chr10_5756	p2	(CA)6	12	31611951	31611962
chr10_5757	p2	(CA)6	12	31612234	31612245
chr10_5758	p2	(GT)6	12	31614742	31614753
chr10_5759	p2	(TG)6	12	31615028	31615039
chr10_5760	p2	(TG)9	18	31615922	31615939
chr10_5761	p4	(TAGG)7	28	31617529	31617556
chr10_5762	p3	(CAA)5	15	31631397	31631411
chr10_5763	p2	(CA)6	12	31632447	31632458
chr10_5764	p3	(AAC)8	24	31635534	31635557
chr10_5765	p2	(TG)18	36	31639884	31639919
chr10_5766	p2	(CA)21	42	31655194	31655235
chr10_5767	p2	(CA)8	16	31658738	31658753
chr10_5768	p2	(AC)9	18	31660734	31660751
chr10_5769	p3	(ATG)6	18	31661374	31661391
chr10_5770	p2	(TC)6	12	31684665	31684676
chr10_5771	p2	(AC)6	12	31700206	31700217
chr10_5772	p2	(AC)13	26	31704644	31704669
chr10_5773	p2	(CA)14	28	31708450	31708477
chr10_5774	p2	(AG)6	12	31717139	31717150
chr10_5775	p2	(AT)10	20	31735809	31735828
chr10_5776	p3	(TAG)6	18	31741644	31741661
chr10_5777	p2	(AC)14	28	31741947	31741974
chr10_5778	p2	(GT)9	18	31743197	31743214
chr10_5779	p2	(AC)7	14	31743948	31743961
chr10_5780	p4	(CCAT)6	24	31751235	31751258
chr10_5781	c	(CT)15(CA)15	60	31776381	31776440
chr10_5782	p4	(ATCC)6	24	31779889	31779912
chr10_5783	p2	(CA)16	32	31783316	31783347
chr10_5784	c	(AC)6g(CA)8	29	31790462	31790490
chr10_5785	p2	(GT)6	12	31796889	31796900
chr10_5786	p2	(CT)12	24	31798506	31798529
chr10_5787	p3	(AAG)7	21	31801123	31801143
chr10_5788	p2	(AC)9	18	31809767	31809784
chr10_5789	p2	(TC)7	14	31815293	31815306
chr10_5790	p2	(GA)7	14	31817105	31817118
chr10_5791	p4	(AATA)5	20	31824140	31824159
chr10_5792	p5	(AATCA)5	25	31830107	31830131
chr10_5793	p4	(ATAG)11	44	31840954	31840997

chr10_5794	p4	(AATC)5	20	31841854	31841873
chr10_5795	p5	(ATTTT)5	25	31845852	31845876
chr10_5796	p2	(GT)10	20	31848793	31848812
chr10_5797	p2	(TG)11	22	31851036	31851057
chr10_5798	p2	(TC)12	24	31855884	31855907
chr10_5799	p3	(AGC)6	18	31856743	31856760
chr10_5800	p3	(TGA)5	15	31857020	31857034
chr10_5801	p2	(AC)9	18	31858839	31858856
chr10_5802	p2	(GT)6	12	31861058	31861069
chr10_5803	p2	(AC)11	22	31861503	31861524
chr10_5804	p2	(GT)6	12	31867774	31867785
chr10_5805	p3	(AAC)5	15	31876267	31876281
chr10_5806	p2	(AC)7	14	31882619	31882632
chr10_5807	p2	(TG)13	26	31884207	31884232
chr10_5808	p2	(AC)17	34	31886556	31886589
chr10_5809	p5	(GATTT)5	25	31896885	31896909
chr10_5810	p2	(TG)6	12	31922552	31922563
chr10_5811	p2	(AC)7	14	31925826	31925839
chr10_5812	p3	(CTA)6	18	31929508	31929525
chr10_5813	p2	(GA)6	12	31931382	31931393
chr10_5814	p2	(AG)10	20	31931517	31931536
chr10_5815	p2	(GT)15	30	31938298	31938327
chr10_5816	p2	(AC)6	12	31949447	31949458
chr10_5817	p2	(TG)8	16	31949581	31949596
chr10_5818	p2	(GT)21	42	31957747	31957788
chr10_5819	c	(TC)7tttctctctctc(TG)12	50	31963564	31963613
chr10_5820	c	(GT)6caaaacctg(AT)8	37	31966035	31966071
chr10_5821	p2	(GT)16	32	31968751	31968782
chr10_5822	p2	(TG)19	38	31972792	31972829
chr10_5823	p2	(TG)6	12	31975455	31975466
chr10_5824	p2	(TG)13	26	31978056	31978081
chr10_5825	p2	(TG)7	14	31979642	31979655
chr10_5826	p2	(CA)6	12	31981034	31981045
chr10_5827	p2	(GT)10	20	31981297	31981316
chr10_5828	p2	(TG)9	18	31986150	31986167
chr10_5829	c	(CA)7t(AC)7	29	31990909	31990937
chr10_5830	c	(CA)12(GA)11(GT)11(GA)11	90	31993552	31993641
chr10_5831	p2	(AC)7	14	31998377	31998390
chr10_5832	c	(TG)12c(GT)19	63	32006519	32006581
chr10_5833	p2	(GT)17	34	32008077	32008110
chr10_5834	p3	(TAT)8	24	32017434	32017457
chr10_5835	p2	(AC)7	14	32020552	32020565
chr10_5836	p2	(CA)24	48	32020671	32020718
chr10_5837	p2	(TC)7	14	32021603	32021616
chr10_5838	p2	(CA)17	34	32026772	32026805
chr10_5839	p2	(AC)8	16	32032337	32032352
chr10_5840	c	(TA)6g(AT)6(AG)7	39	32036272	32036310
chr10_5841	p3	(GGA)5	15	32036863	32036877
chr10_5842	p2	(GT)7	14	32038007	32038020
chr10_5843	p2	(AC)18	36	32064430	32064465
chr10_5844	p2	(GT)7	14	32095248	32095261
chr10_5845	p2	(TG)6	12	32095679	32095690

chr10_5846	p2	(TG)12	24	32096934	32096957
chr10_5847	p4	(ATGG)6	24	32104422	32104445
chr10_5848	p3	(ACA)5	15	32105576	32105590
chr10_5849	p2	(AC)6	12	32130323	32130334
chr10_5850	p2	(AC)11	22	32132642	32132663
chr10_5851	p3	(AAT)12	36	32134962	32134997
chr10_5852	p3	(TAT)5	15	32138323	32138337
chr10_5853	p4	(ATGG)6	24	32156733	32156756
chr10_5854	p5	(CAGAG)6	30	32161945	32161974
chr10_5855	p2	(GT)16	32	32162157	32162188
chr10_5856	p2	(TA)7	14	32178090	32178103
chr10_5857	p2	(GT)12	24	32186524	32186547
chr10_5858	p2	(TG)19	38	32189835	32189872
chr10_5859	p2	(TG)6	12	32190035	32190046
chr10_5860	p2	(TG)6	12	32190676	32190687
chr10_5861	c	(CA)7aa(CTTT)5	36	32190882	32190917
chr10_5862	p4	(AACA)6	24	32193340	32193363
chr10_5863	p2	(GA)7	14	32194272	32194285
chr10_5864	p3	(TTA)5	15	32207152	32207166
chr10_5865	p2	(TA)6	12	32211071	32211082
chr10_5866	p2	(GA)14	28	32219989	32220016
chr10_5867	p3	(TGA)6	18	32224505	32224522
chr10_5868	p3	(GTA)5	15	32264855	32264869
chr10_5869	p2	(TG)6	12	32266005	32266016
chr10_5870	p2	(TG)13	26	32271438	32271463
chr10_5871	p2	(GA)6	12	32272326	32272337
chr10_5872	p2	(GA)6	12	32272450	32272461
chr10_5873	p3	(GTA)5	15	32280015	32280029
chr10_5874	p2	(GT)18	36	32283850	32283885
chr10_5875	p2	(GT)10	20	32288747	32288766
chr10_5876	p2	(AC)9	18	32305906	32305923
chr10_5877	p2	(GT)18	36	32307120	32307155
chr10_5878	p2	(TA)7	14	32309076	32309089
chr10_5879	p2	(AT)7	14	32309438	32309451
chr10_5880	p2	(AC)14	28	32312984	32313011
chr10_5881	p2	(CA)17	34	32319120	32319153
chr10_5882	p2	(AC)26	52	32319487	32319538
chr10_5883	p2	(AC)10	20	32328659	32328678
chr10_5884	p2	(AC)13	26	32337529	32337554
chr10_5885	p2	(AC)6	12	32340550	32340561
chr10_5886	p3	(AGC)5	15	32361331	32361345
chr10_5887	p2	(AC)10	20	32371058	32371077
chr10_5888	p2	(AG)9	18	32373117	32373134
chr10_5889	p3	(TAT)5	15	32374912	32374926
chr10_5890	p2	(AC)10	20	32379613	32379632
chr10_5891	p2	(AC)14	28	32381566	32381593
chr10_5892	p2	(AC)10	20	32384178	32384197
chr10_5893	p4	(GATA)5	20	32386045	32386064
chr10_5894	p2	(TG)6	12	32387150	32387161
chr10_5895	p3	(GCT)5	15	32388649	32388663
chr10_5896	p2	(TG)12	24	32389322	32389345
chr10_5897	p2	(TG)7	14	32389560	32389573

chr10_5898	p2	(TA)6	12	32390750	32390761
chr10_5899	p2	(TG)21	42	32391162	32391203
chr10_5900	p2	(GA)8	16	32399670	32399685
chr10_5901	p2	(GT)6	12	32401015	32401026
chr10_5902	p2	(TG)16	32	32401377	32401408
chr10_5903	p2	(TG)8	16	32403313	32403328
chr10_5904	p2	(TG)6	12	32403479	32403490
chr10_5905	p4	(TCTG)7	28	32406986	32407013
chr10_5906	p2	(TG)6	12	32419488	32419499
chr10_5907	p2	(AC)7	14	32426502	32426515
chr10_5908	p2	(AC)20	40	32431561	32431600
chr10_5909	p2	(TG)10	20	32432304	32432323
chr10_5910	p2	(CA)6	12	32448033	32448044
chr10_5911	p3	(TGG)5	15	32454842	32454856
chr10_5912	p2	(CA)6	12	32456740	32456751
chr10_5913	p3	(TAT)5	15	32457776	32457790
chr10_5914	p2	(CA)13	26	32463043	32463068
chr10_5915	p2	(TG)6	12	32463890	32463901
chr10_5916	p2	(TA)9	18	32465677	32465694
chr10_5917	p5	(CATAT)9	45	32469817	32469861
chr10_5918	p2	(AC)15	30	32480112	32480141
chr10_5919	p2	(AC)11	22	32492472	32492493
chr10_5920	p3	(TAT)5	15	32495903	32495917
chr10_5921	p2	(CA)19	38	32501593	32501630
chr10_5922	p3	(CCT)5	15	32501819	32501833
chr10_5923	c	agtagagggggtcgttcagaggacagaaacacacaggcagtctctcgtct	121	32507151	32507271
chr10_5924	p2	(AT)7	14	32509330	32509343
chr10_5925	p2	(AC)6	12	32515439	32515450
chr10_5926	p2	(CT)6	12	32530113	32530124
chr10_5927	c	(AT)8(GT)11	38	32541485	32541522
chr10_5928	p3	(ATT)5	15	32545258	32545272
chr10_5929	p2	(TA)16	32	32562945	32562976
chr10_5930	p2	(CA)6	12	32580228	32580239
chr10_5931	p2	(GT)14	28	32593236	32593263
chr10_5932	p2	(CA)6	12	32613570	32613581
chr10_5933	p2	(AT)10	20	32615042	32615061
chr10_5934	p4	(GCTA)5	20	32618824	32618843
chr10_5935	p4	(GCTA)5	20	32627064	32627083
chr10_5936	p2	(CT)6	12	32632204	32632215
chr10_5937	p2	(AC)7	14	32634070	32634083
chr10_5938	p2	(CT)6	12	32638068	32638079
chr10_5939	p3	(ATC)5	15	32643397	32643411
chr10_5940	p2	(GT)29	58	32648918	32648975
chr10_5941	c	(GT)22gg(GT)6	58	32654708	32654765
chr10_5942	p5	(CCTCT)9	45	32665363	32665407
chr10_5943	p2	(AG)12	24	32673764	32673787
chr10_5944	p3	(TCC)5	15	32685000	32685014
chr10_5945	p3	(GGA)9	27	32686830	32686856
chr10_5946	p2	(TG)6	12	32689921	32689932
chr10_5947	p3	(CCA)6	18	32692865	32692882
chr10_5948	p3	(GGA)6	18	32697646	32697663
chr10_5949	p2	(AC)9	18	32701392	32701409

chr10_5950	p2	(TG)6	12	32725187	32725198
chr10_5951	p3	(CTT)9	27	32750353	32750379
chr10_5952	p2	(AC)6	12	32761146	32761157
chr10_5953	p3	(TCT)8	24	32766515	32766538
chr10_5954	p2	(AC)10	20	32768591	32768610
chr10_5955	c	ıtctctctgtctgtctctcggtctctctctgtctttctgactgtctgtctgtctgtct	112	32769806	32769917
chr10_5956	c	)9tcttcatcgtggacacaccgttcattgtggacacacactgttcactctga(/	80	32776483	32776562
chr10_5957	p2	(AG)15	30	32783281	32783310
chr10_5958	p3	(ACA)6	18	32792945	32792962
chr10_5959	p3	(TTC)5	15	32794188	32794202
chr10_5960	p2	(AG)6	12	32803691	32803702
chr10_5961	p6	(TACATA)5	30	32805725	32805754
chr10_5962	p3	(TGA)6	18	32813669	32813686
chr10_5963	p3	(AAT)5	15	32813864	32813878
chr10_5964	p4	(TATT)5	20	32840442	32840461
chr10_5965	p2	(TG)8	16	32840859	32840874
chr10_5966	p2	(AC)6	12	32865251	32865262
chr10_5967	p2	(TG)6	12	32885181	32885192
chr10_5968	c	(TG)12cgtgtgtgtgtccacaaagaccttctgcacat(ACA)5	71	32886193	32886263
chr10_5969	p2	(AC)8	16	32893369	32893384
chr10_5970	p2	(AC)7	14	32896632	32896645
chr10_5971	p2	(TG)20	40	32909212	32909251
chr10_5972	p4	(TTTG)6	24	32922670	32922693
chr10_5973	p2	(CT)6	12	32955382	32955393
chr10_5974	p4	(ATTC)7	28	32968088	32968115
chr10_5975	p3	(ATA)5	15	32975567	32975581
chr10_5976	p4	(TCTT)5	20	32993490	32993509
chr10_5977	p2	(AT)12	24	33011734	33011757
chr10_5978	p3	(ATG)9	27	33021029	33021055
chr10_5979	p2	(TG)6	12	33034126	33034137
chr10_5980	p3	(AAT)6	18	33053419	33053436
chr10_5981	c	ıcgtatgcgcannnnnnnnnnnnnnnnnnnnnnnnnnnnntgtgtc(	179	33067550	33067728
chr10_5982	p2	(AC)7	14	33124024	33124037
chr10_5983	c	(TC)6tatctatctatatttatata(TC)6	44	33133758	33133801
chr10_5984	p2	(CA)29	58	33134455	33134512
chr10_5985	p2	(AC)6	12	33134871	33134882
chr10_5986	p2	(GT)6	12	33136779	33136790
chr10_5987	p2	(TG)7	14	33138204	33138217
chr10_5988	p2	(AC)8	16	33140443	33140458
chr10_5989	p3	(CAA)5	15	33142734	33142748
chr10_5990	p3	(GAA)6	18	33162515	33162532
chr10_5991	p2	(AG)7	14	33165015	33165028
chr10_5992	p2	(TG)6	12	33165310	33165321
chr10_5993	p4	(AATC)5	20	33168097	33168116
chr10_5994	p2	(TG)7	14	33169195	33169208
chr10_5995	p2	(TG)9	18	33169992	33170009
chr10_5996	p3	(GGA)6	18	33173933	33173950
chr10_5997	p2	(GT)30	60	33179730	33179789
chr10_5998	p2	(TC)12	24	33180629	33180652
chr10_5999	p2	(CA)7	14	33180815	33180828
chr10_6000	p2	(TG)11	22	33184834	33184855
chr10_6001	p5	(TTTGA)5	25	33186204	33186228

chr10_6002	p2	(AC)11	22	33186999	33187020
chr10_6003	c	(AT)7acatatatacgtataagtatatc(AT)6	49	33195415	33195463
chr10_6004	p2	(TG)6	12	33197281	33197292
chr10_6005	p2	(TG)6	12	33205723	33205734
chr10_6006	p3	(GAG)12	36	33205994	33206029
chr10_6007	p2	(CA)6	12	33206666	33206677
chr10_6008	p4	(TTTC)5	20	33211444	33211463
chr10_6009	p2	(CA)15	30	33214515	33214544
chr10_6010	p2	(TC)9	18	33222987	33223004
chr10_6011	p5	(AATCT)5	25	33235738	33235762
chr10_6012	p2	(AC)18	36	33236241	33236276
chr10_6013	p2	(TC)6	12	33255064	33255075
chr10_6014	p2	(GT)6	12	33257024	33257035
chr10_6015	p2	(TC)10	20	33258231	33258250
chr10_6016	p2	(GT)11	22	33263979	33264000
chr10_6017	p2	(AC)7	14	33273401	33273414
chr10_6018	c	(TG)18ta(TG)6	50	33276083	33276132
chr10_6019	p2	(AT)10	20	33280686	33280705
chr10_6020	p3	(ATT)5	15	33290132	33290146
chr10_6021	p5	(TGTTT)5	25	33338693	33338717
chr10_6022	p2	(GT)6	12	33391921	33391932
chr10_6023	p3	(AGG)5	15	33394331	33394345
chr10_6024	p4	(CATC)5	20	33416440	33416459
chr10_6025	p3	(CCT)5	15	33426234	33426248
chr10_6026	c	(TCTG)6tcttcttt(TTC)6ta(CTT)5	67	33430990	33431056
chr10_6027	c	(CT)6cccgtagtgtgtc(CT)8	41	33433737	33433777
chr10_6028	p2	(GT)13	26	33439122	33439147
chr10_6029	p3	(GAG)6	18	33441890	33441907
chr10_6030	p2	(AC)16	32	33446349	33446380
chr10_6031	p3	(TCC)6	18	33447292	33447309
chr10_6032	p2	(CA)6	12	33457529	33457540
chr10_6033	p3	(CCT)5	15	33462623	33462637
chr10_6034	p2	(GT)18	36	33463957	33463992
chr10_6035	p4	(ATTC)5	20	33468059	33468078
chr10_6036	p2	(AC)13	26	33479682	33479707
chr10_6037	p3	(TAA)6	18	33505157	33505174
chr10_6038	p2	(AC)7	14	33506167	33506180
chr10_6039	p2	(CA)9	18	33509337	33509354
chr10_6040	p2	(CA)9	18	33514497	33514514
chr10_6041	c	(AC)12aa(AAC)6	44	33516449	33516492
chr10_6042	p3	(GAG)5	15	33525900	33525914
chr10_6043	p2	(TG)6	12	33526615	33526626
chr10_6044	p5	(CCGAC)6	30	33527335	33527364
chr10_6045	p2	(AC)7	14	33528375	33528388
chr10_6046	p2	(TA)14	28	33534917	33534944
chr10_6047	p3	(TCT)6	18	33537172	33537189
chr10_6048	p2	(TA)7	14	33537540	33537553
chr10_6049	p2	(TG)7	14	33549654	33549667
chr10_6050	p3	(ATT)5	15	33567056	33567070
chr10_6051	c	(GT)27gagagagc(GA)12	86	33589086	33589171
chr10_6052	p2	(CA)7	14	33590251	33590264
chr10_6053	p4	(ATGG)5	20	33593148	33593167

chr10_6054	p2	(AT)8	16	33593779	33593794
chr10_6055	p3	(CTC)5	15	33603149	33603163
chr10_6056	p2	(AT)8	16	33603851	33603866
chr10_6057	p2	(CG)6	12	33604618	33604629
chr10_6058	c	(ATA)7atg(ATA)6	42	33605557	33605598
chr10_6059	c	(AGA)5aaggaggagagagggaggggggtgatcaaag(GA)8	61	33606646	33606706
chr10_6060	p5	(CAGAA)5	25	33609471	33609495
chr10_6061	p3	(GAG)7	21	33611714	33611734
chr10_6062	p2	(TC)6	12	33618449	33618460
chr10_6063	p2	(TG)7	14	33624677	33624690
chr10_6064	c	catatatggtccctcataaacagtactgtggccccatatcaattaccccctca	126	33638685	33638810
chr10_6065	c	)6atagttaaactagcagctgccttaccacggctgtggttttaattacac(T	88	33641876	33641963
chr10_6066	p2	(AC)6	12	33644073	33644084
chr10_6067	c	)5agcagtagtggtagtgataccagtacc(AGT)5agcagtagtggtag	222	33645957	33646178
chr10_6068	p2	(AT)6	12	33649468	33649479
chr10_6069	p3	(ATG)6	18	33657211	33657228
chr10_6070	p2	(AC)7	14	33663346	33663359
chr10_6071	p2	(CA)35	70	33665059	33665128
chr10_6072	c	(CA)8cc(CA)6	30	33667660	33667689
chr10_6073	p3	(TCT)5	15	33673308	33673322
chr10_6074	p2	(GA)6	12	33674162	33674173
chr10_6075	p3	(AAT)5	15	33677600	33677614
chr10_6076	p2	(GT)6	12	33695554	33695565
chr10_6077	p2	(CA)7	14	33696482	33696495
chr10_6078	p2	(AC)12	24	33702673	33702696
chr10_6079	p2	(AC)11	22	33703394	33703415
chr10_6080	p3	(TCC)5	15	33704491	33704505
chr10_6081	p2	(TC)6	12	33708133	33708144
chr10_6082	p3	(TCC)6	18	33709181	33709198
chr10_6083	p2	(TG)8	16	33711294	33711309
chr10_6084	p2	(AG)7	14	33734196	33734209
chr10_6085	p2	(CT)8	16	33740466	33740481
chr10_6086	p2	(TC)8	16	33742945	33742960
chr10_6087	p2	(CA)13	26	33753052	33753077
chr10_6088	p2	(CA)8	16	33759612	33759627
chr10_6089	p2	(AC)8	16	33774715	33774730
chr10_6090	p3	(TAA)6	18	33776849	33776866
chr10_6091	p3	(AAG)6	18	33781575	33781592
chr10_6092	p3	(GCT)5	15	33809105	33809119
chr10_6093	p2	(AC)15	30	33815815	33815844
chr10_6094	p2	(CA)10	20	33831058	33831077
chr10_6095	p4	(TCCA)5	20	33843363	33843382
chr10_6096	p2	(CA)7	14	33846515	33846528
chr10_6097	p2	(TG)13	26	33849870	33849895
chr10_6098	c	(GT)14t(TG)6	41	33859500	33859540
chr10_6099	p2	(TG)12	24	33865394	33865417
chr10_6100	p4	(ATGG)5	20	33867957	33867976
chr10_6101	p2	(AG)10	20	33873619	33873638
chr10_6102	p2	(GA)12	24	33879515	33879538
chr10_6103	p2	(TG)11	22	33886406	33886427
chr10_6104	c	26tttatatttttgtgtgctccaatgaccacacattgcaaccccccccacat(	120	33912937	33913056
chr10_6105	p2	(TG)7	14	33915459	33915472

chr10_6106	p2	(GA)7	14	33923832	33923845
chr10_6107	p2	(GT)15	30	33933288	33933317
chr10_6108	p3	(AGC)8	24	33940343	33940366
chr10_6109	p2	(TC)9	18	33941141	33941158
chr10_6110	p2	(AC)23	46	33943352	33943397
chr10_6111	p2	(TG)6	12	33951945	33951956
chr10_6112	p2	(AT)8	16	33964046	33964061
chr10_6113	p2	(AC)7	14	33986199	33986212
chr10_6114	p2	(AC)6	12	33988963	33988974
chr10_6115	p2	(AC)12	24	33990277	33990300
chr10_6116	p4	(TACC)7	28	33990647	33990674
chr10_6117	p2	(TC)10	20	33990783	33990802
chr10_6118	p2	(AG)7	14	33996383	33996396
chr10_6119	p2	(TG)18	36	33999953	33999988
chr10_6120	p2	(AT)13	26	34005206	34005231
chr10_6121	p2	(TA)8	16	34006776	34006791
chr10_6122	p2	(TA)6	12	34007495	34007506
chr10_6123	p2	(TG)32	64	34015349	34015412
chr10_6124	p2	(TG)22	44	34017668	34017711
chr10_6125	p3	(TTA)6	18	34021932	34021949
chr10_6126	p3	(TTA)6	18	34024786	34024803
chr10_6127	p2	(GT)18	36	34041263	34041298
chr10_6128	p2	(GT)11	22	34044295	34044316
chr10_6129	p2	(AC)7	14	34057610	34057623
chr10_6130	p2	(CA)8	16	34067489	34067504
chr10_6131	p2	(TG)8	16	34070701	34070716
chr10_6132	p3	(TTG)5	15	34074336	34074350
chr10_6133	p2	(AT)8	16	34074653	34074668
chr10_6134	p2	(TC)7	14	34080135	34080148
chr10_6135	p2	(GT)7	14	34085045	34085058
chr10_6136	p2	(AC)26	52	34086854	34086905
chr10_6137	p3	(AGG)6	18	34089716	34089733
chr10_6138	p2	(TG)9	18	34112021	34112038
chr10_6139	p2	(AC)19	38	34113849	34113886
chr10_6140	p5	(GAGAG)6	30	34116940	34116969
chr10_6141	p3	(CCA)7	21	34122324	34122344
chr10_6142	p4	(GGTA)5	20	34135000	34135019
chr10_6143	p3	(ATC)5	15	34143631	34143645
chr10_6144	c	gcttcattctctggaatttagtttataccctcctcatatggacatgtctgtacag	113	34147564	34147676
chr10_6145	p2	(GA)6	12	34166459	34166470
chr10_6146	c	(GT)17gcgtgcgtgc(GT)14	72	34195146	34195217
chr10_6147	p2	(AG)9	18	34200760	34200777
chr10_6148	p3	(CCT)5	15	34216677	34216691
chr10_6149	p2	(GT)6	12	34223016	34223027
chr10_6150	p2	(CA)13	26	34225828	34225853
chr10_6151	p4	(TTGA)8	32	34229160	34229191
chr10_6152	p3	(TTA)11	33	34256067	34256099
chr10_6153	c	tgcaacctagagattcctgtacagattctctagcagcaaagaattcaaaagt	138	34266759	34266896
chr10_6154	p2	(AC)7	14	34282033	34282046
chr10_6155	p2	(GT)6	12	34292787	34292798
chr10_6156	p4	(TCAT)5	20	34300443	34300462
chr10_6157	p3	(AAC)5	15	34302869	34302883

chr10_6158	p2	(AC)10	20	34319256	34319275
chr10_6159	c	(AC)12atata(TATC)12	77	34331515	34331591
chr10_6160	p3	(TGA)5	15	34344581	34344595
chr10_6161	p2	(AC)7	14	34354060	34354073
chr10_6162	c	(TG)7cgtgtgc(GT)10	41	34357326	34357366
chr10_6163	c	(TTA)5(TTG)5	30	34364242	34364271
chr10_6164	p2	(AG)6	12	34379143	34379154
chr10_6165	p2	(AG)16	32	34400792	34400823
chr10_6166	p3	(GTT)7	21	34416491	34416511
chr10_6167	p2	(AC)10	20	34425622	34425641
chr10_6168	p2	(AC)21	42	34427239	34427280
chr10_6169	p4	(CCTA)15	60	34443390	34443449
chr10_6170	p2	(AT)7	14	34451178	34451191
chr10_6171	p2	(GT)6	12	34469844	34469855
chr10_6172	p2	(AC)11	22	34491159	34491180
chr10_6173	p2	(AC)6	12	34491771	34491782
chr10_6174	p3	(TAA)6	18	34509989	34510006
chr10_6175	p2	(AC)20	40	34514966	34515005
chr10_6176	p2	(CA)16	32	34520439	34520470
chr10_6177	c	(TA)13ga(GT)8	44	34560720	34560763
chr10_6178	p2	(TG)9	18	34561598	34561615
chr10_6179	p2	(CA)7	14	34567110	34567123
chr10_6180	p2	(AC)7	14	34567358	34567371
chr10_6181	p2	(TG)19	38	34570821	34570858
chr10_6182	c*	(GT)9(GTGA)5*(GA)10*	54	34571640	34571693
chr10_6183	p2	(AC)6	12	34572436	34572447
chr10_6184	c	(GT)9(GA)6	30	34582615	34582644
chr10_6185	c	(AAT)5aaataaag(AAT)5	38	34588056	34588093
chr10_6186	p3	(TTG)5	15	34590451	34590465
chr10_6187	p3	(TAA)7	21	34614955	34614975
chr10_6188	p2	(AC)10	20	34616336	34616355
chr10_6189	p2	(TG)7	14	34621613	34621626
chr10_6190	p2	(CT)17	34	34652076	34652109
chr10_6191	p3	(TTA)5	15	34662368	34662382
chr10_6192	p2	(AC)6	12	34679400	34679411
chr10_6193	c	(TG)6tatatacacacacacaaacacatatacatac(AT)9(GT)9	79	34689175	34689253
chr10_6194	c	gccatttggcatgagcaggtgaacccctgcctgcacctgcttgctgaaat	112	34707845	34707956
chr10_6195	p2	(TG)9	18	34711441	34711458
chr10_6196	p2	(CA)11	22	34715471	34715492
chr11_1	p3	(TTA)7	21	12304	12324
chr11_2	p2	(AG)6	12	22700	22711
chr11_3	p2	(GT)7	14	32778	32791
chr11_4	p2	(TC)8	16	51956	51971
chr11_5	p2	(AT)11	22	52829	52850
chr11_6	p4	(GGTA)7	28	107032	107059
chr11_7	p3	(AAT)7	21	127923	127943
chr11_8	p2	(GT)6	12	137341	137352
chr11_9	p2	(AC)14	28	140495	140522
chr11_10	p2	(TG)19	38	143573	143610
chr11_11	p2	(AC)9	18	176964	176981
chr11_12	p3	(GCC)6	18	178268	178285
chr11_13	p2	(GT)16	32	196682	196713

chr11_14	p4	(AGGT)7	28	198920	198947
chr11_15	p2	(AT)7	14	202713	202726
chr11_16	p2	(AG)6	12	219321	219332
chr11_17	p2	(AC)7	14	224370	224383
chr11_18	c	(AC)6atgcacacacatg(CA)6	37	224559	224595
chr11_19	p2	(TC)7	14	232156	232169
chr11_20	p3	(ATT)6	18	259637	259654
chr11_21	p2	(CA)7	14	260576	260589
chr11_22	p2	(TC)8	16	270727	270742
chr11_23	p2	(AG)6	12	273414	273425
chr11_24	p2	(TG)9	18	283519	283536
chr11_25	p2	(GT)6	12	299129	299140
chr11_26	c	(AC)7g(CA)7	29	314754	314782
chr11_27	p2	(TG)9	18	317301	317318
chr11_28	p2	(TC)6	12	318338	318349
chr11_29	p2	(AC)6	12	319279	319290
chr11_30	p3	(TCA)5	15	335038	335052
chr11_31	p2	(GT)7	14	360531	360544
chr11_32	p2	(TA)6	12	365366	365377
chr11_33	p3	(CTC)5	15	365531	365545
chr11_34	p2	(AC)6	12	372894	372905
chr11_35	p2	(GT)6	12	380005	380016
chr11_36	p2	(AC)14	28	382037	382064
chr11_37	p2	(AT)8	16	398775	398790
chr11_38	p2	(CA)6	12	408591	408602
chr11_39	p2	(TG)11	22	412301	412322
chr11_40	p2	(CA)15	30	412792	412821
chr11_41	c	(AT)18(GT)6	48	417581	417628
chr11_42	p2	(AC)6	12	419481	419492
chr11_43	p2	(AC)7	14	427375	427388
chr11_44	p2	(CA)7	14	430681	430694
chr11_45	p2	(GT)11	22	433255	433276
chr11_46	p2	(CA)9	18	449247	449264
chr11_47	p2	(CA)15	30	453376	453405
chr11_48	p2	(TG)6	12	453560	453571
chr11_49	p2	(AC)8	16	455677	455692
chr11_50	p2	(CA)7	14	463331	463344
chr11_51	c	(TA)7(TG)11	36	481348	481383
chr11_52	p2	(GT)10	20	482392	482411
chr11_53	p5	(AAATC)5	25	489132	489156
chr11_54	p2	(TG)6	12	489757	489768
chr11_55	p3	(TTA)5	15	500857	500871
chr11_56	p3	(GCT)6	18	501022	501039
chr11_57	p2	(TG)7	14	505386	505399
chr11_58	p2	(AC)17	34	510130	510163
chr11_59	p2	(TC)12	24	565998	566021
chr11_60	p3	(ACA)5	15	579082	579096
chr11_61	p2	(TG)15	30	584158	584187
chr11_62	p2	(GT)11	22	605800	605821
chr11_63	p2	(CA)11	22	617303	617324
chr11_64	c	(CA)26ctgccacccctgctctcaccctaagacacaacggtga(AC)2[illegible]	131	617848	617978
chr11_65	p2	(AC)33	66	642828	642893

chr11_66	p2	(GT)6	12	643228	643239
chr11_67	p2	(AC)7	14	648328	648341
chr11_68	p2	(GT)24	48	659672	659719
chr11_69	p2	(TG)12	24	665747	665770
chr11_70	p2	(GT)13	26	668503	668528
chr11_71	p2	(TG)6	12	671905	671916
chr11_72	p2	(AC)6	12	675251	675262
chr11_73	c	ttcatacattttcggggcactaaggccaatagacaaatgatatgcataggcta	96	681780	681875
chr11_74	p4	(ATGG)5	20	687232	687251
chr11_75	p2	(CA)14	28	690450	690477
chr11_76	p3	(GCA)5	15	703343	703357
chr11_77	p2	(TG)6	12	703953	703964
chr11_78	p2	(TG)11	22	712035	712056
chr11_79	p2	(GT)6	12	746152	746163
chr11_80	p5	(TTTGA)6	30	747844	747873
chr11_81	p2	(GT)7	14	757759	757772
chr11_82	p2	(TG)6	12	765147	765158
chr11_83	p2	(CA)15	30	771842	771871
chr11_84	p2	(CA)6	12	775940	775951
chr11_85	p2	(CA)9	18	794997	795014
chr11_86	p2	(GA)6	12	795546	795557
chr11_87	c	(AC)8at(AC)8	34	800333	800366
chr11_88	c	ctctccttaattcttggcagcatgtgtattccctcaacagggcatgattagttaa	105	807717	807821
chr11_89	p2	(CA)6	12	807956	807967
chr11_90	p2	(AC)8	16	809318	809333
chr11_91	p2	(CA)9	18	814803	814820
chr11_92	p2	(TA)9	18	830700	830717
chr11_93	p2	(TA)10	20	833063	833082
chr11_94	p2	(TG)6	12	833522	833533
chr11_95	p2	(AG)6	12	845726	845737
chr11_96	p2	(TG)6	12	866731	866742
chr11_97	p3	(CAG)7	21	871432	871452
chr11_98	p2	(TC)9	18	874038	874055
chr11_99	p2	(TA)7	14	905974	905987
chr11_100	p3	(CTT)7	21	909763	909783
chr11_101	p2	(AC)7	14	967072	967085
chr11_102	p4	(TATT)5	20	968332	968351
chr11_103	p2	(CA)8	16	971381	971396
chr11_104	p2	(TA)6	12	972939	972950
chr11_105	p4	(TTTG)5	20	1010360	1010379
chr11_106	p3	(TGA)7	21	1019457	1019477
chr11_107	p2	(AC)10	20	1027289	1027308
chr11_108	p2	(TG)31	62	1027474	1027535
chr11_109	p2	(CT)6	12	1031521	1031532
chr11_110	p2	(GT)6	12	1046130	1046141
chr11_111	p3	(ATA)5	15	1046847	1046861
chr11_112	p2	(CA)10	20	1051824	1051843
chr11_113	p2	(CT)6	12	1079626	1079637
chr11_114	p2	(AC)31	62	1083107	1083168
chr11_115	p2	(AC)9	18	1083344	1083361
chr11_116	p2	(CA)8	16	1085835	1085850
chr11_117	p3	(TAT)8	24	1088745	1088768

chr11_118	p3	(ACT)5	15	1094694	1094708
chr11_119	p2	(GA)9	18	1099880	1099897
chr11_120	p3	(ATA)8	24	1100317	1100340
chr11_121	p4	(AGAT)5	20	1101490	1101509
chr11_122	p2	(TC)7	14	1103663	1103676
chr11_123	p2	(AC)6	12	1107968	1107979
chr11_124	p2	(TA)7	14	1109611	1109624
chr11_125	p2	(AC)6	12	1109757	1109768
chr11_126	p3	(TGA)6	18	1117443	1117460
chr11_127	p2	(AG)8	16	1160975	1160990
chr11_128	p2	(TA)6	12	1190869	1190880
chr11_129	p2	(TG)6	12	1196648	1196659
chr11_130	p4	(AATC)5	20	1207545	1207564
chr11_131	p2	(TG)7	14	1208275	1208288
chr11_132	p3	(ATT)6	18	1217342	1217359
chr11_133	p2	(CA)8	16	1218955	1218970
chr11_134	p3	(CAT)9	27	1228237	1228263
chr11_135	p2	(AG)7	14	1239348	1239361
chr11_136	p2	(CA)6	12	1242697	1242708
chr11_137	p2	(AC)19	38	1242993	1243030
chr11_138	p2	(TG)6	12	1266800	1266811
chr11_139	p2	(CA)7	14	1268723	1268736
chr11_140	p2	(TG)7	14	1270606	1270619
chr11_141	p2	(TC)8	16	1273198	1273213
chr11_142	p2	(CA)10	20	1276017	1276036
chr11_143	c	(CT)6(CA)14	40	1277344	1277383
chr11_144	p2	(TG)7	14	1279904	1279917
chr11_145	p2	(TC)7	14	1292879	1292892
chr11_146	p2	(TA)7	14	1303171	1303184
chr11_147	p2	(AT)6	12	1303439	1303450
chr11_148	p2	(GT)27	54	1307628	1307681
chr11_149	p2	(CA)6	12	1331558	1331569
chr11_150	p2	(CT)6	12	1346195	1346206
chr11_151	p2	(AC)7	14	1362274	1362287
chr11_152	p2	(TC)6	12	1365145	1365156
chr11_153	p2	(AC)7	14	1366560	1366573
chr11_154	p4	(AACC)5	20	1379229	1379248
chr11_155	p4	(TCCA)5	20	1380072	1380091
chr11_156	p2	(AT)9	18	1385348	1385365
chr11_157	p2	(GT)6	12	1416538	1416549
chr11_158	p2	(CA)6	12	1418646	1418657
chr11_159	p2	(GT)8	16	1418946	1418961
chr11_160	p2	(TG)7	14	1420788	1420801
chr11_161	p2	(GT)6	12	1427634	1427645
chr11_162	p3	(TTA)5	15	1430095	1430109
chr11_163	p3	(TAA)6	18	1436751	1436768
chr11_164	p3	(AAT)7	21	1437568	1437588
chr11_165	p2	(AT)6	12	1450715	1450726
chr11_166	p2	(GT)6	12	1456473	1456484
chr11_167	p2	(CA)10	20	1458282	1458301
chr11_168	p2	(CT)11	22	1465803	1465824
chr11_169	p2	(TC)6	12	1469348	1469359

chr11_170	p2	(TG)13	26	1471878	1471903
chr11_171	p3	(ATA)5	15	1475779	1475793
chr11_172	p2	(AG)12	24	1507025	1507048
chr11_173	p3	(TGT)5	15	1517068	1517082
chr11_174	p2	(AT)8	16	1521883	1521898
chr11_175	p2	(TC)6	12	1533430	1533441
chr11_176	p3	(TTG)5	15	1537539	1537553
chr11_177	p3	(GCA)6	18	1538855	1538872
chr11_178	p4	(TAGG)5	20	1540765	1540784
chr11_179	p2	(GT)14	28	1548516	1548543
chr11_180	p2	(AC)13	26	1549244	1549269
chr11_181	p2	(GT)9	18	1557659	1557676
chr11_182	p2	(TG)18	36	1569507	1569542
chr11_183	p2	(AC)7	14	1570982	1570995
chr11_184	p2	(TC)6	12	1572327	1572338
chr11_185	p2	(TG)16	32	1587675	1587706
chr11_186	p2	(AC)6	12	1587848	1587859
chr11_187	p2	(AC)15	30	1592300	1592329
chr11_188	p2	(AT)10	20	1600451	1600470
chr11_189	p2	(CA)14	28	1606198	1606225
chr11_190	p2	(CA)7	14	1611172	1611185
chr11_191	p2	(TG)6	12	1611354	1611365
chr11_192	p2	(TG)6	12	1613429	1613440
chr11_193	p2	(CA)10	20	1616168	1616187
chr11_194	p2	(AC)16	32	1638265	1638296
chr11_195	p2	(TC)9	18	1640318	1640335
chr11_196	p2	(TC)6	12	1646854	1646865
chr11_197	c	(CCA)6cccacc(CA)13	50	1648053	1648102
chr11_198	p2	(AT)8	16	1648510	1648525
chr11_199	p2	(TG)10	20	1657363	1657382
chr11_200	c	tcctttttcacctctaattggtcttctgggagttgtcaggggtctcactggcta	175	1657707	1657881
chr11_201	c	(TA)7cag(TA)15	47	1660427	1660473
chr11_202	p2	(AC)6	12	1667176	1667187
chr11_203	p3	(TTG)6	18	1677531	1677548
chr11_204	p2	(TG)15	30	1678973	1679002
chr11_205	p5	(TTAAA)5	25	1679889	1679913
chr11_206	p3	(CAG)6	18	1698352	1698369
chr11_207	p2	(AC)7	14	1702779	1702792
chr11_208	p2	(AC)14	28	1709934	1709961
chr11_209	p3	(TTG)6	18	1711374	1711391
chr11_210	p2	(CA)8	16	1716127	1716142
chr11_211	p2	(CA)7	14	1732211	1732224
chr11_212	p2	(AC)17	34	1732342	1732375
chr11_213	p2	(CA)13	26	1747025	1747050
chr11_214	p2	(TG)8	16	1759679	1759694
chr11_215	c	gagaaggtatagcatttattaacattgtttaaataaaaactgtgagtgtgctc(	80	1792058	1792137
chr11_216	c	acacctcaaaaaaccactaactcctagaagcagatatgttagctggtttaac	142	1801051	1801192
chr11_217	p3	(TGG)5	15	1807095	1807109
chr11_218	p2	(GT)7	14	1807246	1807259
chr11_219	p2	(TG)6	12	1807987	1807998
chr11_220	p3	(TGA)5	15	1823051	1823065
chr11_221	p2	(GT)6	12	1836030	1836041

chr11_222	p2	(TG)7	14	1836630	1836643
chr11_223	p3	(AGG)6	18	1843553	1843570
chr11_224	p2	(CA)7	14	1852817	1852830
chr11_225	p4	(TATT)5	20	1865616	1865635
chr11_226	p2	(AC)6	12	1868793	1868804
chr11_227	p3	(AGT)5	15	1875403	1875417
chr11_228	p3	(AAG)7	21	1876244	1876264
chr11_229	p2	(GA)7	14	1879870	1879883
chr11_230	p2	(AC)6	12	1882687	1882698
chr11_231	p2	(TG)15	30	1896546	1896575
chr11_232	c	agaaaaagaaaaggaaa(AAG)6aaaaagaaaaagaaaaagaagaa	567	1913967	1914533
chr11_233	p2	(GT)7	14	1915458	1915471
chr11_234	p4	(TCCA)5	20	1934711	1934730
chr11_235	p2	(AG)12	24	1942971	1942994
chr11_236	p2	(GT)7	14	1965525	1965538
chr11_237	p2	(CA)10	20	1969342	1969361
chr11_238	c	(TTCCTC)7ttc(TCCT)11	89	1971761	1971849
chr11_239	p2	(AG)21	42	1974900	1974941
chr11_240	p5	(TTAGA)5	25	1979772	1979796
chr11_241	p2	(TG)8	16	1989264	1989279
chr11_242	p2	(AG)8	16	2005059	2005074
chr11_243	p2	(TG)12	24	2007569	2007592
chr11_244	p4	(ATGG)5	20	2013882	2013901
chr11_245	p2	(CA)7	14	2018449	2018462
chr11_246	p2	(GA)7	14	2023562	2023575
chr11_247	p2	(TG)6	12	2024268	2024279
chr11_248	p3	(AGG)5	15	2029953	2029967
chr11_249	p2	(TG)13	26	2032334	2032359
chr11_250	c	(CA)14atactataattgcccgcctttt(TG)9	68	2032752	2032819
chr11_251	p3	(TAG)8	24	2042311	2042334
chr11_252	p2	(GT)10	20	2043337	2043356
chr11_253	p2	(AT)10	20	2052821	2052840
chr11_254	p2	(AC)10	20	2057053	2057072
chr11_255	p2	(AC)7	14	2068282	2068295
chr11_256	p3	(AAT)5	15	2076778	2076792
chr11_257	p2	(TG)7	14	2078929	2078942
chr11_258	p2	(CA)16	32	2079238	2079269
chr11_259	p2	(AC)28	56	2086524	2086579
chr11_260	c	(AC)11(TA)15cacgcgcgc(GT)7	75	2088686	2088760
chr11_261	p3	(TTG)5	15	2102045	2102059
chr11_262	p2	(AG)6	12	2130566	2130577
chr11_263	c	(GA)10gc(GA)8	38	2133400	2133437
chr11_264	p2	(GA)9	18	2164513	2164530
chr11_265	p2	(GT)8	16	2166214	2166229
chr11_266	p2	(AC)10	20	2166707	2166726
chr11_267	p2	(TG)17	34	2173739	2173772
chr11_268	p2	(AC)12	24	2173882	2173905
chr11_269	p2	(AC)20	40	2193039	2193078
chr11_270	p3	(AAT)5	15	2202684	2202698
chr11_271	p2	(TG)6	12	2203369	2203380
chr11_272	c	(TG)14(CG)7	42	2203626	2203667
chr11_273	c	(TG)12tatacatactgtatac(AT)7	54	2204896	2204949

chr11_274	p2	(AT)6	12	2206752	2206763
chr11_275	p2	(AC)6	12	2207285	2207296
chr11_276	p4	(GTAG)9	36	2210432	2210467
chr11_277	p2	(CT)8	16	2215752	2215767
chr11_278	p2	(GT)23	46	2217082	2217127
chr11_279	p2	(TG)6	12	2219410	2219421
chr11_280	p2	(AT)7	14	2233201	2233214
chr11_281	p3	(GAT)5	15	2236763	2236777
chr11_282	p3	(ATT)7	21	2263135	2263155
chr11_283	p4	(AATC)5	20	2268001	2268020
chr11_284	p6	(ATTAGT)6	36	2270611	2270646
chr11_285	c	(CA)6cgcaaacagaa(AG)7	37	2274992	2275028
chr11_286	p2	(TG)10	20	2278318	2278337
chr11_287	p2	(TG)8	16	2296332	2296347
chr11_288	p2	(AC)8	16	2298234	2298249
chr11_289	p2	(TA)7	14	2302855	2302868
chr11_290	p2	(CA)7	14	2304261	2304274
chr11_291	p2	(CA)8	16	2322939	2322954
chr11_292	p2	(CA)27	54	2323254	2323307
chr11_293	p3	(ATT)5	15	2333032	2333046
chr11_294	p2	(GA)7	14	2335678	2335691
chr11_295	p4	(AACA)5	20	2336404	2336423
chr11_296	p5	(CCTCT)5	25	2342670	2342694
chr11_297	p2	(TG)19	38	2346617	2346654
chr11_298	c	(AC)8a(AC)15	47	2348342	2348388
chr11_299	p2	(GA)7	14	2352893	2352906
chr11_300	p3	(GAT)7	21	2366654	2366674
chr11_301	p2	(TG)6	12	2378502	2378513
chr11_302	p2	(AC)11	22	2381752	2381773
chr11_303	p2	(CA)8	16	2383341	2383356
chr11_304	p3	(TTA)5	15	2386553	2386567
chr11_305	p2	(GT)6	12	2405590	2405601
chr11_306	p3	(AAG)5	15	2416371	2416385
chr11_307	p2	(GT)13	26	2428214	2428239
chr11_308	p4	(TATG)5	20	2433474	2433493
chr11_309	p2	(TG)13	26	2441752	2441777
chr11_310	c*	(CCA)7(CA)6*	31	2463125	2463155
chr11_311	p2	(CA)8	16	2465466	2465481
chr11_312	p4	(ATCC)6	24	2483417	2483440
chr11_313	p2	(GT)10	20	2488751	2488770
chr11_314	p4	(AGGT)9	36	2498921	2498956
chr11_315	p5	(CCTCT)8	40	2499622	2499661
chr11_316	p2	(AC)6	12	2520814	2520825
chr11_317	p2	(AC)27	54	2528646	2528699
chr11_318	p3	(ATT)7	21	2538501	2538521
chr11_319	p2	(AC)13	26	2541818	2541843
chr11_320	p4	(TTTA)5	20	2542478	2542497
chr11_321	p2	(AT)7	14	2554526	2554539
chr11_322	p3	(AGA)5	15	2563115	2563129
chr11_323	p4	(TCTA)7	28	2571126	2571153
chr11_324	p2	(GA)6	12	2572809	2572820
chr11_325	p2	(TA)7	14	2578793	2578806

chr11_326	c	(TC)9atgtacagtg(TA)8	44	2582873	2582916
chr11_327	p3	(TAA)5	15	2598976	2598990
chr11_328	p3	(ATT)5	15	2599274	2599288
chr11_329	p2	(TG)7	14	2603038	2603051
chr11_330	p3	(TCT)5	15	2628396	2628410
chr11_331	p4	(AAGC)5	20	2630101	2630120
chr11_332	c	(TATC)5(CATC)9	56	2631570	2631625
chr11_333	p2	(TG)6	12	2636265	2636276
chr11_334	p4	(TACC)5	20	2636447	2636466
chr11_335	p3	(AGC)5	15	2658433	2658447
chr11_336	p2	(TC)6	12	2664995	2665006
chr11_337	c	gaatatattgaatgaatcttgagaatatataatgaaagtctgaatatatatata	103	2675175	2675277
chr11_338	p2	(TA)10	20	2675929	2675948
chr11_339	p2	(TC)6	12	2682381	2682392
chr11_340	p2	(AC)8	16	2691829	2691844
chr11_341	p3	(AAG)5	15	2708496	2708510
chr11_342	p2	(CT)6	12	2709745	2709756
chr11_343	p2	(TA)6	12	2715160	2715171
chr11_344	c	(AT)7acacaa(AG)6t(GA)16	65	2716835	2716899
chr11_345	c	ttctttgtttcatctatgtatgtgtgtgtag(GT)16ctgcatacccgtgctcc	250	2723016	2723265
chr11_346	p2	(GT)6	12	2727423	2727434
chr11_347	p2	(AC)6	12	2730582	2730593
chr11_348	p2	(TG)14	28	2733349	2733376
chr11_349	p2	(GA)7	14	2734972	2734985
chr11_350	p2	(AC)12	24	2740763	2740786
chr11_351	p2	(CA)6	12	2743746	2743757
chr11_352	p2	(CA)8	16	2746303	2746318
chr11_353	p3	(TTG)5	15	2755380	2755394
chr11_354	p2	(AC)7	14	2769307	2769320
chr11_355	p2	(CA)16	32	2797515	2797546
chr11_356	p2	(AT)7	14	2798930	2798943
chr11_357	p2	(TG)9	18	2799618	2799635
chr11_358	p2	(CA)6	12	2810057	2810068
chr11_359	p2	(CA)13	26	2811669	2811694
chr11_360	p2	(GT)12	24	2811834	2811857
chr11_361	p2	(TG)9	18	2816345	2816362
chr11_362	p4	(ATCT)5	20	2817012	2817031
chr11_363	p2	(GT)6	12	2823520	2823531
chr11_364	p2	(CA)14	28	2825817	2825844
chr11_365	p2	(CA)8	16	2826128	2826143
chr11_366	p2	(TG)7	14	2831152	2831165
chr11_367	p2	(CA)7	14	2841473	2841486
chr11_368	c	(TC)9(AC)8	34	2849940	2849973
chr11_369	c	ggagccaatcccggctggcaatcgggcaaaggcaggtcaccaatccat	132	2857991	2858122
chr11_370	c	(GT)9g(GT)6(GA)18	67	2861058	2861124
chr11_371	p2	(AC)8	16	2869917	2869932
chr11_372	p2	(TG)9	18	2879201	2879218
chr11_373	p2	(CT)7	14	2884863	2884876
chr11_374	p2	(GT)9	18	2889426	2889443
chr11_375	p2	(CT)7	14	2896060	2896073
chr11_376	p3	(TTA)12	36	2896190	2896225
chr11_377	p2	(AG)13	26	2897041	2897066

chr11_378	c	(AC)6tgctttgtagagttgagaaggga(GT)11	57	2924436	2924492
chr11_379	p2	(AC)6	12	2941425	2941436
chr11_380	c	(AT)23(AC)10	66	2942721	2942786
chr11_381	c	(TC)6cctg(TC)7	30	2968525	2968554
chr11_382	p2	(AT)7	14	2969122	2969135
chr11_383	p2	(CA)18	36	2979761	2979796
chr11_384	p4	(CCAT)5	20	2988135	2988154
chr11_385	p3	(AAT)6	18	2989325	2989342
chr11_386	p2	(TG)6	12	2995540	2995551
chr11_387	p4	(TCCA)6	24	3006608	3006631
chr11_388	p2	(TA)8	16	3027471	3027486
chr11_389	p2	(CA)7	14	3027912	3027925
chr11_390	p2	(TG)8	16	3029845	3029860
chr11_391	p2	(CA)8	16	3032661	3032676
chr11_392	p2	(AC)7	14	3033898	3033911
chr11_393	p2	(GT)12	24	3039361	3039384
chr11_394	p2	(GT)7	14	3042891	3042904
chr11_395	p2	(CA)16	32	3043047	3043078
chr11_396	p2	(AC)30	60	3044121	3044180
chr11_397	p2	(CA)7	14	3051528	3051541
chr11_398	p4	(TTGC)6	24	3056411	3056434
chr11_399	p2	(TC)7	14	3060033	3060046
chr11_400	p2	(GT)11	22	3069554	3069575
chr11_401	p2	(TG)18	36	3074760	3074795
chr11_402	p2	(TG)7	14	3085943	3085956
chr11_403	p2	(AC)7	14	3089987	3090000
chr11_404	p2	(AC)6	12	3093289	3093300
chr11_405	p2	(AC)6	12	3112179	3112190
chr11_406	p2	(TG)14	28	3125389	3125416
chr11_407	p2	(AT)6	12	3133709	3133720
chr11_408	c	[illegible]acgtgcgcctatggagataaaatactatcaaaaatgttacaaatgcgtgttt[illegible]	128	3135579	3135706
chr11_409	p2	(AC)6	12	3138422	3138433
chr11_410	p2	(GA)8	16	3141370	3141385
chr11_411	p2	(AC)10	20	3165103	3165122
chr11_412	p2	(AG)6	12	3174547	3174558
chr11_413	c	[illegible]C)7aaactggcaatttatttagccaagaatgtgcctacttgttcactg(TC[illegible]	71	3179960	3180030
chr11_414	c*	(CA)19(CACT)5*	56	3180621	3180676
chr11_415	p3	(TAT)5	15	3186922	3186936
chr11_416	p2	(GC)7	14	3192080	3192093
chr11_417	p2	(CA)7	14	3203619	3203632
chr11_418	p2	(AC)6	12	3203829	3203840
chr11_419	p2	(TG)6	12	3204036	3204047
chr11_420	p5	(TTGTT)5	25	3215978	3216002
chr11_421	p3	(TTC)5	15	3222211	3222225
chr11_422	p2	(CA)16	32	3226161	3226192
chr11_423	p3	(AAG)5	15	3236995	3237009
chr11_424	p2	(AT)6	12	3241725	3241736
chr11_425	p3	(TAT)7	21	3242206	3242226
chr11_426	p2	(TG)7	14	3261872	3261885
chr11_427	p2	(TG)6	12	3267855	3267866
chr11_428	p2	(AT)20	40	3270772	3270811
chr11_429	p2	(AT)10	20	3274105	3274124

chr11_430	p3	(TTG)5	15	3275700	3275714
chr11_431	p2	(CA)18	36	3276962	3276997
chr11_432	p4	(TCCA)6	24	3283512	3283535
chr11_433	p3	(TTG)5	15	3307608	3307622
chr11_434	p2	(AC)11	22	3317939	3317960
chr11_435	p2	(TC)8	16	3330589	3330604
chr11_436	p2	(CA)6	12	3345917	3345928
chr11_437	c	aacagtcatcatcactatgcatctaataataataacagttatacaataatcata	84	3347239	3347322
chr11_438	p5	(TAATC)7	35	3349246	3349280
chr11_439	p2	(GA)9	18	3355205	3355222
chr11_440	p2	(GT)6	12	3356165	3356176
chr11_441	p2	(GT)18	36	3379984	3380019
chr11_442	p2	(CA)6	12	3380416	3380427
chr11_443	p2	(AC)9	18	3383312	3383329
chr11_444	p2	(CA)6	12	3384811	3384822
chr11_445	p2	(AC)8	16	3385958	3385973
chr11_446	p2	(TG)9	18	3386122	3386139
chr11_447	p2	(CA)7	14	3387647	3387660
chr11_448	p2	(TG)16	32	3387979	3388010
chr11_449	p2	(TG)9	18	3396740	3396757
chr11_450	p4	(TCAT)5	20	3407589	3407608
chr11_451	p2	(GT)6	12	3413961	3413972
chr11_452	p2	(AC)6	12	3430126	3430137
chr11_453	c*	aaaaaaataaagggaacaccacatagttgaatgtcttatttttgtgttccctttta	133	3431297	3431429
chr11_454	p5	(TCAAG)6	30	3431640	3431669
chr11_455	c	(CT)12ttttcctttctccc(CT)6	51	3448024	3448074
chr11_456	p3	(GTT)5	15	3448349	3448363
chr11_457	p2	(CA)21	42	3454761	3454802
chr11_458	p2	(TG)9	18	3456786	3456803
chr11_459	p3	(AGG)7	21	3458970	3458990
chr11_460	p4	(TTTG)5	20	3467555	3467574
chr11_461	p2	(GT)9	18	3470871	3470888
chr11_462	p3	(AAT)5	15	3496053	3496067
chr11_463	p2	(CA)6	12	3496458	3496469
chr11_464	p3	(TAA)5	15	3499479	3499493
chr11_465	p3	(TAT)5	15	3514810	3514824
chr11_466	p2	(GA)11	22	3517002	3517023
chr11_467	p2	(TG)7	14	3521830	3521843
chr11_468	p2	(GT)6	12	3528203	3528214
chr11_469	p2	(TA)6	12	3537043	3537054
chr11_470	p2	(GT)7	14	3543314	3543327
chr11_471	p2	(AC)6	12	3544084	3544095
chr11_472	p2	(TG)10	20	3545926	3545945
chr11_473	p2	(TG)9	18	3550266	3550283
chr11_474	p2	(AC)10	20	3557401	3557420
chr11_475	p3	(TTA)5	15	3558621	3558635
chr11_476	p2	(AC)8	16	3575205	3575220
chr11_477	p2	(TA)6	12	3577361	3577372
chr11_478	p2	(TG)7	14	3604365	3604378
chr11_479	p2	(TG)15	30	3606547	3606576
chr11_480	p3	(GCA)5	15	3611323	3611337
chr11_481	p3	(TAT)5	15	3611921	3611935

chr11_482	p2	(CA)7	14	3624810	3624823
chr11_483	p2	(AC)18	36	3627356	3627391
chr11_484	p2	(AC)16	32	3627673	3627704
chr11_485	p2	(TG)14	28	3635360	3635387
chr11_486	p2	(TG)7	14	3637120	3637133
chr11_487	p2	(TG)16	32	3646261	3646292
chr11_488	c	(CCA)5(CCT)9	42	3651291	3651332
chr11_489	p2	(CT)7	14	3660728	3660741
chr11_490	p2	(AT)6	12	3666504	3666515
chr11_491	p3	(ACC)7	21	3674891	3674911
chr11_492	p3	(AAT)6	18	3678826	3678843
chr11_493	p2	(TG)8	16	3686761	3686776
chr11_494	p2	(GT)27	54	3688294	3688347
chr11_495	p2	(TG)6	12	3691682	3691693
chr11_496	p3	(GAG)6	18	3693937	3693954
chr11_497	p2	(TG)7	14	3694364	3694377
chr11_498	p2	(TG)10	20	3696304	3696323
chr11_499	p2	(GA)6	12	3701049	3701060
chr11_500	p2	(TG)14	28	3702105	3702132
chr11_501	p2	(AC)6	12	3703394	3703405
chr11_502	p2	(GT)6	12	3716155	3716166
chr11_503	p2	(TG)7	14	3716412	3716425
chr11_504	p2	(TG)8	16	3721906	3721921
chr11_505	p2	(GA)18	36	3724620	3724655
chr11_506	p2	(GT)6	12	3740510	3740521
chr11_507	p3	(AAT)5	15	3758008	3758022
chr11_508	p2	(GA)11	22	3760135	3760156
chr11_509	p2	(TG)23	46	3783679	3783724
chr11_510	p2	(GT)14	28	3795123	3795150
chr11_511	p2	(CA)13	26	3796679	3796704
chr11_512	p2	(AT)6	12	3801295	3801306
chr11_513	p2	(AC)9	18	3803700	3803717
chr11_514	p2	(CA)8	16	3804074	3804089
chr11_515	p2	(CA)8	16	3805168	3805183
chr11_516	p2	(AC)28	56	3806411	3806466
chr11_517	p2	(TG)10	20	3806864	3806883
chr11_518	p2	(TC)11	22	3809621	3809642
chr11_519	p2	(AT)7	14	3822259	3822272
chr11_520	p2	(TG)14	28	3827527	3827554
chr11_521	p4	(AATA)5	20	3833092	3833111
chr11_522	p4	(TCAT)8	32	3836502	3836533
chr11_523	p2	(AC)11	22	3839078	3839099
chr11_524	c	(TG)8(TC)7	30	3859820	3859849
chr11_525	p2	(GT)11	22	3861150	3861171
chr11_526	c	(CT)6ccctctctcacactcgcgcgcg(CA)8	50	3861274	3861323
chr11_527	c	(TG)7tttaagggcagggct(AC)9	47	3864336	3864382
chr11_528	p2	(AC)6	12	3903905	3903916
chr11_529	p2	(TA)6	12	3912374	3912385
chr11_530	p3	(AAC)5	15	3914838	3914852
chr11_531	p2	(TC)6	12	3923958	3923969
chr11_532	p2	(CT)6	12	3925334	3925345
chr11_533	p2	(GT)9	18	3934970	3934987

chr11_534	p2	(AC)7	14	3955909	3955922
chr11_535	p2	(GT)11	22	3964310	3964331
chr11_536	p4	(ATGG)5	20	3964988	3965007
chr11_537	c	(TA)7gttatatatat(AC)7	39	3966719	3966757
chr11_538	p2	(AC)6	12	3970061	3970072
chr11_539	p2	(TA)6	12	3970639	3970650
chr11_540	p2	(TG)6	12	3991172	3991183
chr11_541	c	(GT)13gg(GT)9	46	3991383	3991428
chr11_542	p2	(TG)6	12	4000193	4000204
chr11_543	p2	(AC)6	12	4013592	4013603
chr11_544	p4	(AATG)5	20	4014390	4014409
chr11_545	p2	(AC)18	36	4015047	4015082
chr11_546	p2	(GT)8	16	4025498	4025513
chr11_547	p4	(TATC)7	28	4026487	4026514
chr11_548	p2	(CA)6	12	4030272	4030283
chr11_549	p2	(TG)10	20	4038733	4038752
chr11_550	p2	(TC)10	20	4039561	4039580
chr11_551	p2	(GA)13	26	4051994	4052019
chr11_552	p2	(TG)33	66	4052225	4052290
chr11_553	p2	(TG)7	14	4056281	4056294
chr11_554	p3	(TAT)5	15	4060670	4060684
chr11_555	p3	(AGC)5	15	4063578	4063592
chr11_556	p2	(CA)7	14	4064691	4064704
chr11_557	p2	(GA)7	14	4064939	4064952
chr11_558	p3	(TGA)5	15	4072741	4072755
chr11_559	p2	(AC)19	38	4075729	4075766
chr11_560	p2	(AC)9	18	4098970	4098987
chr11_561	p2	(AC)10	20	4099619	4099638
chr11_562	p4	(AGTA)5	20	4099786	4099805
chr11_563	p3	(ATA)6	18	4103344	4103361
chr11_564	p3	(TTA)5	15	4104961	4104975
chr11_565	p2	(AC)18	36	4108419	4108454
chr11_566	p2	(TG)6	12	4119147	4119158
chr11_567	p2	(AT)6	12	4119406	4119417
chr11_568	p3	(TAA)5	15	4128569	4128583
chr11_569	p5	(ATCAA)5	25	4131989	4132013
chr11_570	p4	(CCAT)5	20	4181546	4181565
chr11_571	p2	(AT)16	32	4184763	4184794
chr11_572	p2	(TG)19	38	4186652	4186689
chr11_573	p3	(ATA)5	15	4190382	4190396
chr11_574	p5	(CTACT)6	30	4200281	4200310
chr11_575	p2	(TG)19	38	4207338	4207375
chr11_576	p2	(CA)13	26	4207622	4207647
chr11_577	p2	(CA)6	12	4207851	4207862
chr11_578	p2	(AC)16	32	4212432	4212463
chr11_579	p3	(AAG)7	21	4217291	4217311
chr11_580	p2	(AC)6	12	4220833	4220844
chr11_581	p2	(GT)6	12	4240371	4240382
chr11_582	p3	(TCC)6	18	4242402	4242419
chr11_583	p2	(AC)6	12	4244767	4244778
chr11_584	p3	(AAT)5	15	4245963	4245977
chr11_585	p3	(GCA)5	15	4255377	4255391

chr11_586	p2	(CA)6	12	4262119	4262130
chr11_587	p2	(TA)6	12	4276266	4276277
chr11_588	p2	(TG)6	12	4289429	4289440
chr11_589	p2	(TG)6	12	4289668	4289679
chr11_590	p2	(AC)9	18	4291678	4291695
chr11_591	p2	(AC)13	26	4301812	4301837
chr11_592	p3	(TTG)5	15	4318066	4318080
chr11_593	p3	(AGC)5	15	4318462	4318476
chr11_594	p2	(GT)7	14	4320483	4320496
chr11_595	c	(TG)6(AG)6	24	4321595	4321618
chr11_596	c	TG)17gcagtttgagactgggtgtgagcgtgtgtatgaagtgtgca(TG)	89	4324228	4324316
chr11_597	p3	(TAT)5	15	4327919	4327933
chr11_598	p3	(ATT)5	15	4349527	4349541
chr11_599	p2	(GT)6	12	4351183	4351194
chr11_600	p2	(AG)6	12	4358090	4358101
chr11_601	p2	(AC)9	18	4373400	4373417
chr11_602	c	(TG)6agagggagaaaaa(GT)6	37	4380297	4380333
chr11_603	p3	(CAT)5	15	4386757	4386771
chr11_604	p3	(TGC)5	15	4389261	4389275
chr11_605	p3	(CTG)5	15	4389545	4389559
chr11_606	p3	(TGC)6	18	4389774	4389791
chr11_607	p2	(CT)6	12	4392752	4392763
chr11_608	p2	(AC)18	36	4398825	4398860
chr11_609	c	(TA)10cacac(ACAT)5	45	4401422	4401466
chr11_610	c	(TA)8catatatacacacacat(AC)14	61	4402794	4402854
chr11_611	p2	(AC)6	12	4423271	4423282
chr11_612	p2	(GT)20	40	4425836	4425875
chr11_613	p2	(CA)20	40	4426877	4426916
chr11_614	p2	(CA)25	50	4436208	4436257
chr11_615	p2	(AC)27	54	4437069	4437122
chr11_616	p3	(AAC)5	15	4449536	4449550
chr11_617	c	(AC)6atatacatacaaaattgttggtacccttctgtt(TA)8	61	4465854	4465914
chr11_618	p2	(TA)8	16	4467703	4467718
chr11_619	p2	(TG)10	20	4471541	4471560
chr11_620	p4	(ATGG)5	20	4472389	4472408
chr11_621	p2	(TA)6	12	4486093	4486104
chr11_622	p2	(GT)6	12	4492711	4492722
chr11_623	p4	(CCAC)6	24	4497058	4497081
chr11_624	p2	(AT)7	14	4498131	4498144
chr11_625	p4	(GAAT)6	24	4499478	4499501
chr11_626	p4	(ATCC)5	20	4500315	4500334
chr11_627	p2	(AG)6	12	4500940	4500951
chr11_628	p2	(AC)6	12	4515514	4515525
chr11_629	p3	(ATT)5	15	4515904	4515918
chr11_630	c	(CCTT)5ctttttc(TCTT)5	47	4541926	4541972
chr11_631	p3	(TCT)9	27	4542714	4542740
chr11_632	p2	(AC)11	22	4543746	4543767
chr11_633	p2	(AC)20	40	4543959	4543998
chr11_634	p2	(GT)11	22	4545084	4545105
chr11_635	p4	(TATC)17	68	4548416	4548483
chr11_636	p2	(CA)24	48	4548820	4548867
chr11_637	p2	(AC)6	12	4551643	4551654

chr11_638	p2	(CA)7	14	4556605	4556618
chr11_639	p2	(AC)14	28	4557615	4557642
chr11_640	p2	(CA)7	14	4557824	4557837
chr11_641	p2	(TG)20	40	4560046	4560085
chr11_642	p2	(GT)8	16	4560883	4560898
chr11_643	p2	(AC)9	18	4575720	4575737
chr11_644	p3	(AAC)5	15	4591780	4591794
chr11_645	p2	(TA)11	22	4630319	4630340
chr11_646	p2	(TG)6	12	4631891	4631902
chr11_647	p3	(CTG)6	18	4634847	4634864
chr11_648	p3	(TGG)7	21	4640522	4640542
chr11_649	c	(ACC)5tcct(CCA)6	37	4640647	4640683
chr11_650	p3	(TTG)5	15	4640807	4640821
chr11_651	p3	(TAA)6	18	4646195	4646212
chr11_652	p2	(GT)11	22	4647773	4647794
chr11_653	p2	(TG)17	34	4653243	4653276
chr11_654	p3	(AAT)5	15	4658805	4658819
chr11_655	p2	(AC)6	12	4658940	4658951
chr11_656	p2	(TG)13	26	4660588	4660613
chr11_657	p2	(TG)13	26	4674649	4674674
chr11_658	p2	(TA)7	14	4675948	4675961
chr11_659	p3	(TAA)6	18	4701764	4701781
chr11_660	p2	(AC)6	12	4707032	4707043
chr11_661	p3	(AAT)6	18	4711154	4711171
chr11_662	p2	(AC)10	20	4719567	4719586
chr11_663	p2	(CA)6	12	4728448	4728459
chr11_664	p2	(TG)23	46	4729290	4729335
chr11_665	p3	(TAT)6	18	4739756	4739773
chr11_666	c	(GT)6atatatactgtatactgtagctaggtaatagtt(TA)6	57	4742058	4742114
chr11_667	p2	(CA)14	28	4745696	4745723
chr11_668	p4	(TCCA)5	20	4749938	4749957
chr11_669	p2	(TG)17	34	4774938	4774971
chr11_670	p3	(TTG)5	15	4776035	4776049
chr11_671	p4	(ATTT)5	20	4782257	4782276
chr11_672	p2	(GT)6	12	4794598	4794609
chr11_673	p4	(AATG)6	24	4803420	4803443
chr11_674	c	(CA)12(TA)6	36	4806243	4806278
chr11_675	p2	(CA)7	14	4813327	4813340
chr11_676	p4	(ATGG)5	20	4814183	4814202
chr11_677	p2	(AT)8	16	4825079	4825094
chr11_678	c	(AGA)7agtagt(AGA)5	42	4828095	4828136
chr11_679	p2	(TG)7	14	4829302	4829315
chr11_680	p3	(CAT)5	15	4830743	4830757
chr11_681	p2	(CA)6	12	4839327	4839338
chr11_682	p2	(TG)25	50	4845219	4845268
chr11_683	p2	(TA)10	20	4848695	4848714
chr11_684	p2	(TG)10	20	4849200	4849219
chr11_685	p2	(GT)8	16	4852724	4852739
chr11_686	p3	(GTG)5	15	4854353	4854367
chr11_687	p3	(GGT)9	27	4857140	4857166
chr11_688	c	[illegible])8cctccctctctctctgtctgc(CT)7ccctccctctctctctccctctt(T	86	4862125	4862210
chr11_689	p2	(TG)7	14	4866601	4866614

chr11_690	p2	(CA)9	18	4870770	4870787
chr11_691	p2	(TG)19	38	4877273	4877310
chr11_692	p3	(TAC)7	21	4892318	4892338
chr11_693	p2	(TG)10	20	4894855	4894874
chr11_694	p4	(AAAT)6	24	4895541	4895564
chr11_695	p3	(AAT)5	15	4901393	4901407
chr11_696	p2	(AT)6	12	4907724	4907735
chr11_697	p2	(TG)6	12	4930491	4930502
chr11_698	p2	(TC)6	12	4931286	4931297
chr11_699	c	(CT)18cccc(CT)11	62	4951121	4951182
chr11_700	c	(CT)12(CA)32	88	4956306	4956393
chr11_701	p2	(AC)13	26	4956617	4956642
chr11_702	p2	(CA)13	26	4959011	4959036
chr11_703	p2	(CA)6	12	4962510	4962521
chr11_704	p2	(AG)6	12	4967339	4967350
chr11_705	p3	(AAT)5	15	4968958	4968972
chr11_706	p3	(TAT)6	18	4975139	4975156
chr11_707	p3	(TTA)6	18	4979592	4979609
chr11_708	p3	(AGG)5	15	4984899	4984913
chr11_709	p4	(TGAT)5	20	4987737	4987756
chr11_710	p3	(TTA)5	15	5001194	5001208
chr11_711	p2	(TG)9	18	5007579	5007596
chr11_712	p2	(TC)7	14	5007727	5007740
chr11_713	p2	(AC)8	16	5010567	5010582
chr11_714	c*	(GTG)6(GT)12*	39	5017605	5017643
chr11_715	p2	(GT)22	44	5020655	5020698
chr11_716	p2	(AT)7	14	5023109	5023122
chr11_717	p3	(TAT)5	15	5035527	5035541
chr11_718	p2	(AC)6	12	5044219	5044230
chr11_719	p3	(TTA)6	18	5044633	5044650
chr11_720	p2	(TG)9	18	5045764	5045781
chr11_721	p3	(GGA)7	21	5049593	5049613
chr11_722	p2	(AC)6	12	5054009	5054020
chr11_723	p2	(AT)9	18	5066779	5066796
chr11_724	p2	(CA)13	26	5071134	5071159
chr11_725	p2	(GT)18	36	5073770	5073805
chr11_726	p2	(TC)16	32	5073985	5074016
chr11_727	p2	(TG)6	12	5076844	5076855
chr11_728	p2	(AC)16	32	5091415	5091446
chr11_729	p2	(TG)7	14	5092325	5092338
chr11_730	p2	(CA)12	24	5092613	5092636
chr11_731	p2	(TG)6	12	5094511	5094522
chr11_732	p2	(AC)12	24	5098725	5098748
chr11_733	p2	(TC)6	12	5111039	5111050
chr11_734	p2	(CT)6	12	5115642	5115653
chr11_735	p2	(AT)7	14	5117689	5117702
chr11_736	p4	(ATAG)6	24	5118340	5118363
chr11_737	p2	(CA)8	16	5120517	5120532
chr11_738	p2	(AC)8	16	5122946	5122961
chr11_739	p2	(TA)8	16	5125587	5125602
chr11_740	p2	(CA)8	16	5136218	5136233
chr11_741	p4	(ATTC)5	20	5139496	5139515

chr11_742	p2	(TG)16	32	5151542	5151573
chr11_743	p4	(TTTG)5	20	5159045	5159064
chr11_744	p2	(CA)6	12	5160002	5160013
chr11_745	c	(GT)7ataaa(AG)6	31	5165501	5165531
chr11_746	p3	(CTC)5	15	5173675	5173689
chr11_747	p2	(CA)9	18	5176955	5176972
chr11_748	p3	(GTT)7	21	5179407	5179427
chr11_749	p2	(AG)12	24	5189406	5189429
chr11_750	p2	(GT)10	20	5192495	5192514
chr11_751	p2	(AC)16	32	5196081	5196112
chr11_752	p2	(TG)17	34	5198175	5198208
chr11_753	p4	(CATT)5	20	5212882	5212901
chr11_754	p2	(GT)6	12	5213286	5213297
chr11_755	c	(AG)10acaagaggcccgac(CA)22	78	5217793	5217870
chr11_756	p2	(AC)6	12	5218414	5218425
chr11_757	p2	(TG)11	22	5222740	5222761
chr11_758	p2	(TG)10	20	5225361	5225380
chr11_759	p2	(TC)8	16	5227668	5227683
chr11_760	p2	(TG)7	14	5233815	5233828
chr11_761	p2	(TG)6	12	5234149	5234160
chr11_762	p2	(AT)9	18	5246679	5246696
chr11_763	p2	(AC)8	16	5250948	5250963
chr11_764	c	gatagtgtggtctggaatgggatggcaagtgtgcctgaggaaataggata	135	5251676	5251810
chr11_765	p2	(TC)25	50	5252034	5252083
chr11_766	p2	(CA)24	48	5255846	5255893
chr11_767	p3	(AAG)6	18	5257454	5257471
chr11_768	p2	(GA)6	12	5261705	5261716
chr11_769	p2	(CT)10	20	5273708	5273727
chr11_770	p2	(CA)7	14	5273890	5273903
chr11_771	p2	(TG)7	14	5275879	5275892
chr11_772	p2	(GT)21	42	5276216	5276257
chr11_773	p2	(AC)6	12	5279901	5279912
chr11_774	p2	(GT)9	18	5281804	5281821
chr11_775	c	(AT)6(AC)7	26	5285217	5285242
chr11_776	p2	(CA)6	12	5288757	5288768
chr11_777	p2	(AC)6	12	5292267	5292278
chr11_778	p2	(TG)8	16	5297583	5297598
chr11_779	c	agtgatggcctatacaggccccagtgattaacatttgactgcgggcgtaca	83	5299039	5299121
chr11_780	p2	(CA)6	12	5299468	5299479
chr11_781	p3	(GAG)6	18	5305820	5305837
chr11_782	p3	(GAG)6	18	5308277	5308294
chr11_783	p2	(AC)10	20	5308905	5308924
chr11_784	p2	(TG)6	12	5312178	5312189
chr11_785	c	accttacctctccagccaactgccagaaatggcctctctcccactggaact	157	5312378	5312534
chr11_786	c	tttccaagggagacaaatgttagaaacgtgacacgaagagccagtgtatc	106	5317848	5317953
chr11_787	c	(TG)14(AG)8	44	5320719	5320762
chr11_788	p2	(TG)6	12	5324527	5324538
chr11_789	p2	(AC)7	14	5342735	5342748
chr11_790	p2	(AC)10	20	5343037	5343056
chr11_791	p2	(AG)12	24	5346738	5346761
chr11_792	p3	(AAT)5	15	5357236	5357250
chr11_793	p5	(TTTGA)5	25	5358956	5358980

chr11_794	p2	(AC)7	14	5374184	5374197
chr11_795	p2	(TG)6	12	5379019	5379030
chr11_796	p2	(TG)6	12	5381177	5381188
chr11_797	p2	(TG)17	34	5390914	5390947
chr11_798	p2	(CA)8	16	5394544	5394559
chr11_799	p2	(GT)6	12	5400295	5400306
chr11_800	p2	(TG)9	18	5401838	5401855
chr11_801	p2	(GA)6	12	5420980	5420991
chr11_802	p3	(CAT)6	18	5421599	5421616
chr11_803	p2	(AC)9	18	5422455	5422472
chr11_804	p2	(AT)16	32	5430150	5430181
chr11_805	p2	(GT)8	16	5435116	5435131
chr11_806	p2	(TG)7	14	5439061	5439074
chr11_807	p3	(AAG)5	15	5449017	5449031
chr11_808	p2	(TA)10	20	5449785	5449804
chr11_809	p3	(CCA)5	15	5458573	5458587
chr11_810	p2	(TG)6	12	5459756	5459767
chr11_811	p2	(AC)6	12	5460733	5460744
chr11_812	p2	(CA)20	40	5463822	5463861
chr11_813	p3	(TAT)6	18	5464495	5464512
chr11_814	p2	(TG)8	16	5472610	5472625
chr11_815	p2	(AC)20	40	5472782	5472821
chr11_816	p2	(TG)6	12	5473160	5473171
chr11_817	p2	(CA)12	24	5474899	5474922
chr11_818	p2	(AC)9	18	5475326	5475343
chr11_819	p3	(TTA)5	15	5486641	5486655
chr11_820	p2	(CA)6	12	5495031	5495042
chr11_821	p2	(TG)6	12	5499998	5500009
chr11_822	p2	(CT)6	12	5502979	5502990
chr11_823	p2	(TG)14	28	5511106	5511133
chr11_824	p2	(TG)12	24	5511298	5511321
chr11_825	p3	(TGT)6	18	5512840	5512857
chr11_826	p2	(GT)6	12	5513158	5513169
chr11_827	p2	(TG)7	14	5516238	5516251
chr11_828	p2	(AC)8	16	5517118	5517133
chr11_829	p2	(AC)8	16	5517702	5517717
chr11_830	p2	(GT)14	28	5518813	5518840
chr11_831	p5	(AAATC)5	25	5526017	5526041
chr11_832	p2	(AC)7	14	5526252	5526265
chr11_833	p2	(AC)12	24	5526471	5526494
chr11_834	p2	(GT)6	12	5529524	5529535
chr11_835	p2	(CT)23	46	5535893	5535938
chr11_836	p3	(TGC)5	15	5579829	5579843
chr11_837	p3	(TGC)5	15	5596709	5596723
chr11_838	p3	(TTA)5	15	5606776	5606790
chr11_839	c	(ACCT)7(AC)12	52	5611429	5611480
chr11_840	p3	(TGC)5	15	5626759	5626773
chr11_841	p2	(TG)7	14	5648993	5649006
chr11_842	p2	(CA)27	54	5653533	5653586
chr11_843	p3	(GCT)6	18	5660675	5660692
chr11_844	p2	(GA)7	14	5661663	5661676
chr11_845	p5	(AAATC)5	25	5664554	5664578

chr11_846	p2	(TG)22	44	5670852	5670895
chr11_847	p2	(CA)9	18	5678724	5678741
chr11_848	p4	(AGAT)13	52	5684611	5684662
chr11_849	p2	(TC)8	16	5687714	5687729
chr11_850	p2	(AC)7	14	5707043	5707056
chr11_851	p2	(AC)6	12	5740955	5740966
chr11_852	p6	(ATTACA)5	30	5744682	5744711
chr11_853	p2	(GA)6	12	5753457	5753468
chr11_854	p2	(TG)11	22	5758294	5758315
chr11_855	p2	(AC)14	28	5760454	5760481
chr11_856	p2	(AC)19	38	5766113	5766150
chr11_857	p3	(AAG)12	36	5780543	5780578
chr11_858	p2	(TC)6	12	5780979	5780990
chr11_859	p2	(TG)8	16	5797772	5797787
chr11_860	p4	(AATG)7	28	5803923	5803950
chr11_861	p2	(AG)6	12	5828385	5828396
chr11_862	p2	(TG)11	22	5840689	5840710
chr11_863	p2	(TG)9	18	5841423	5841440
chr11_864	p5	(TAAAC)5	25	5857872	5857896
chr11_865	p2	(GA)9	18	5877095	5877112
chr11_866	p2	(AC)7	14	5880068	5880081
chr11_867	p3	(AAT)6	18	5891464	5891481
chr11_868	p3	(TGA)7	21	5893667	5893687
chr11_869	p3	(CTG)5	15	5894779	5894793
chr11_870	c	atacacatatatacatatatatac(AT)7acatacatatatatacatacacac	301	5905579	5905879
chr11_871	c	(GT)7c(TG)25	65	5907825	5907889
chr11_872	p5	(CTCTC)5	25	5970607	5970631
chr11_873	p3	(TAT)6	18	5972056	5972073
chr11_874	c	(CA)8agcacacacacaagcatgtgttccctcagt(CA)17	80	5973765	5973844
chr11_875	p2	(CA)8	16	5974041	5974056
chr11_876	p2	(TG)16	32	5979577	5979608
chr11_877	p3	(GAG)5	15	5989425	5989439
chr11_878	p3	(TTA)11	33	5994170	5994202
chr11_879	p2	(AC)6	12	6002042	6002053
chr11_880	c	(TA)7cacatata(TG)14	50	6005289	6005338
chr11_881	p2	(TG)8	16	6009363	6009378
chr11_882	p2	(GT)10	20	6024858	6024877
chr11_883	p2	(AC)22	44	6030252	6030295
chr11_884	p2	(TA)6	12	6030526	6030537
chr11_885	p2	(AC)13	26	6042930	6042955
chr11_886	p5	(AAGGG)6	30	6046305	6046334
chr11_887	p2	(CA)19	38	6046449	6046486
chr11_888	p3	(TGA)5	15	6049116	6049130
chr11_889	p2	(TA)6	12	6049873	6049884
chr11_890	p2	(AC)23	46	6064811	6064856
chr11_891	p2	(TG)16	32	6066892	6066923
chr11_892	p2	(AC)6	12	6069416	6069427
chr11_893	p2	(TG)6	12	6079702	6079713
chr11_894	p2	(AC)17	34	6080212	6080245
chr11_895	p2	(AC)25	50	6080461	6080510
chr11_896	p2	(AC)14	28	6087058	6087085
chr11_897	p2	(TG)14	28	6087599	6087626

chr11_898	p2	(CA)6	12	6089998	6090009
chr11_899	p2	(TG)7	14	6090316	6090329
chr11_900	p5	(AAATT)9	45	6119573	6119617
chr11_901	p2	(TG)22	44	6126711	6126754
chr11_902	p4	(ATCT)6	24	6127464	6127487
chr11_903	p2	(AC)7	14	6133850	6133863
chr11_904	p2	(TG)12	24	6135558	6135581
chr11_905	p2	(GT)8	16	6136224	6136239
chr11_906	p2	(AT)6	12	6137304	6137315
chr11_907	p4	(TTTG)5	20	6137958	6137977
chr11_908	p2	(AC)22	44	6156178	6156221
chr11_909	p2	(TC)12	24	6156595	6156618
chr11_910	p2	(CT)8	16	6157790	6157805
chr11_911	p4	(CCAT)5	20	6161282	6161301
chr11_912	c	(AATG)5(AT)7(GT)33	100	6170591	6170690
chr11_913	p2	(CT)7	14	6172220	6172233
chr11_914	p2	(CA)14	28	6179674	6179701
chr11_915	p2	(TG)7	14	6181316	6181329
chr11_916	p2	(CA)34	68	6189715	6189782
chr11_917	p3	(TAA)7	21	6190592	6190612
chr11_918	p4	(AACA)5	20	6200528	6200547
chr11_919	p2	(CA)7	14	6202839	6202852
chr11_920	p2	(CA)6	12	6203232	6203243
chr11_921	p2	(TG)10	20	6205829	6205848
chr11_922	p2	(CA)30	60	6214235	6214294
chr11_923	c	TGAT)5ttattctttcaaacatgcagaacagaacaaatgacagca(AC	76	6224475	6224550
chr11_924	p2	(TG)7	14	6226245	6226258
chr11_925	p2	(TG)6	12	6226760	6226771
chr11_926	c	C)6tatccacagtattaagtattcactcaacctataccaccccccc(CA)	81	6226965	6227045
chr11_927	p2	(AC)8	16	6233587	6233602
chr11_928	p2	(TC)10	20	6255454	6255473
chr11_929	p3	(GTT)6	18	6271484	6271501
chr11_930	p4	(GATA)6	24	6294489	6294512
chr11_931	p2	(GT)13	26	6297331	6297356
chr11_932	p2	(GT)6	12	6313702	6313713
chr11_933	p3	(CAA)6	18	6320750	6320767
chr11_934	p2	(AG)18	36	6323494	6323529
chr11_935	p2	(TG)11	22	6323820	6323841
chr11_936	p2	(AC)6	12	6323978	6323989
chr11_937	p2	(CA)11	22	6328376	6328397
chr11_938	p4	(GGAA)5	20	6339973	6339992
chr11_939	p2	(GT)6	12	6341106	6341117
chr11_940	c	(AC)7c(CA)11	37	6341276	6341312
chr11_941	p2	(CA)16	32	6341480	6341511
chr11_942	p2	(CA)8	16	6341748	6341763
chr11_943	p2	(TG)8	16	6343974	6343989
chr11_944	c	gctccagctctgtgctttatttcaaaaacattttaattcaccacaaactgtgag	157	6344191	6344347
chr11_945	c	ctgtatctcaccaaaccatagcaacccaaacactgctttcacagctaaaaa	133	6357224	6357356
chr11_946	p2	(GT)7	14	6359125	6359138
chr11_947	p2	(CA)25	50	6359344	6359393
chr11_948	c	(AC)6t(CA)6	25	6361451	6361475
chr11_949	p2	(CA)9	18	6361655	6361672

chr11_950	p2	(AC)6	12	6363080	6363091
chr11_951	p2	(TC)8	16	6365525	6365540
chr11_952	p2	(TG)8	16	6367242	6367257
chr11_953	p2	(CA)6	12	6368491	6368502
chr11_954	p2	(AC)6	12	6373368	6373379
chr11_955	p2	(AC)6	12	6375229	6375240
chr11_956	p2	(TA)7	14	6377319	6377332
chr11_957	p2	(TG)10	20	6377949	6377968
chr11_958	p2	(GT)10	20	6379824	6379843
chr11_959	p2	(CA)13	26	6380905	6380930
chr11_960	p2	(TG)8	16	6381311	6381326
chr11_961	p2	(TG)6	12	6381540	6381551
chr11_962	p2	(AC)15	30	6382876	6382905
chr11_963	c	3T)18atggggacaaaactgagactgcaccaaatcccaagtaga(GT)	97	6383106	6383202
chr11_964	p2	(AC)24	48	6389429	6389476
chr11_965	c	gtatggcagtggagaaagcacctaatcctgttacatgatggcggctactgga	142	6390283	6390424
chr11_966	p2	(TG)8	16	6391250	6391265
chr11_967	p2	(GT)8	16	6401190	6401205
chr11_968	p2	(CA)12	24	6403967	6403990
chr11_969	p2	(CA)11	22	6411120	6411141
chr11_970	p4	(TATC)5	20	6416294	6416313
chr11_971	p2	(AG)7	14	6416562	6416575
chr11_972	p2	(TA)8	16	6436231	6436246
chr11_973	p2	(GT)7	14	6438753	6438766
chr11_974	p3	(TCT)5	15	6439392	6439406
chr11_975	p2	(TG)13	26	6441632	6441657
chr11_976	p2	(AC)6	12	6447705	6447716
chr11_977	p2	(CA)8	16	6447969	6447984
chr11_978	p2	(AC)10	20	6450458	6450477
chr11_979	p3	(CTC)5	15	6450927	6450941
chr11_980	p4	(ATTC)5	20	6457051	6457070
chr11_981	p2	(GT)9	18	6461081	6461098
chr11_982	p3	(AAT)5	15	6462606	6462620
chr11_983	p3	(TGG)5	15	6476425	6476439
chr11_984	p3	(TTA)5	15	6480213	6480227
chr11_985	c	(CT)8ccctc(TG)11	43	6481831	6481873
chr11_986	c	(AGC)6atcaagagtgcttcaatatc(CT)6	50	6489715	6489764
chr11_987	p2	(GT)7	14	6492344	6492357
chr11_988	p2	(CA)21	42	6494797	6494838
chr11_989	p2	(CA)12	24	6494983	6495006
chr11_990	p2	(CA)10	20	6496097	6496116
chr11_991	p4	(ATCT)11	44	6500650	6500693
chr11_992	p3	(AAT)5	15	6505161	6505175
chr11_993	p2	(AG)6	12	6505356	6505367
chr11_994	p2	(CA)7	14	6506301	6506314
chr11_995	p3	(TAA)10	30	6511040	6511069
chr11_996	p2	(TA)8	16	6525457	6525472
chr11_997	p3	(AAT)6	18	6529784	6529801
chr11_998	c	(CA)6g(AC)6	25	6529998	6530022
chr11_999	p2	(AC)6	12	6530249	6530260
chr11_1000	p2	(TG)8	16	6530713	6530728
chr11_1001	p2	(GT)6	12	6530844	6530855

chr11_1002	p2	(TG)22	44	6531048	6531091
chr11_1003	c	tgcacacacacttacaccttgctgagtataatatagaaaagcacacctctac	213	6532935	6533147
chr11_1004	p2	(AG)31	62	6539208	6539269
chr11_1005	p2	(GA)19	38	6540906	6540943
chr11_1006	c	(ATG)13(GT)6	51	6543678	6543728
chr11_1007	p3	(AAT)9	27	6544436	6544462
chr11_1008	p2	(CT)6	12	6544742	6544753
chr11_1009	p2	(CA)8	16	6545427	6545442
chr11_1010	p2	(GT)16	32	6546576	6546607
chr11_1011	p2	(GA)9	18	6546901	6546918
chr11_1012	p2	(TG)9	18	6547134	6547151
chr11_1013	p2	(TG)18	36	6548222	6548257
chr11_1014	p4	(TATC)6	24	6551108	6551131
chr11_1015	p2	(TG)17	34	6553642	6553675
chr11_1016	p2	(TC)6	12	6565735	6565746
chr11_1017	p2	(AC)7	14	6572540	6572553
chr11_1018	p2	(CA)16	32	6573507	6573538
chr11_1019	p2	(GT)15	30	6578263	6578292
chr11_1020	p2	(AT)6	12	6580651	6580662
chr11_1021	p2	(TG)6	12	6588492	6588503
chr11_1022	p2	(GA)9	18	6590491	6590508
chr11_1023	p3	(GTT)7	21	6591978	6591998
chr11_1024	p2	(CA)10	20	6596186	6596205
chr11_1025	p3	(AAC)5	15	6598500	6598514
chr11_1026	p2	(TG)6	12	6599412	6599423
chr11_1027	c	(AC)8a(AC)9	35	6601538	6601572
chr11_1028	p2	(TC)8	16	6601816	6601831
chr11_1029	p2	(GT)6	12	6609953	6609964
chr11_1030	p2	(CA)15	30	6611803	6611832
chr11_1031	p2	(AC)13	26	6614713	6614738
chr11_1032	c	tgttgtggcctgacagaggcccaggtcatagcacgtttactcactgtcatga	146	6623106	6623251
chr11_1033	p2	(TG)7	14	6629615	6629628
chr11_1034	p2	(AC)9	18	6629979	6629996
chr11_1035	c	(CT)6cacag(AC)11	39	6633754	6633792
chr11_1036	p2	(AC)7	14	6640589	6640602
chr11_1037	p2	(TG)6	12	6640721	6640732
chr11_1038	p2	(TG)7	14	6642935	6642948
chr11_1039	c	(TG)7c(GT)7	29	6643292	6643320
chr11_1040	p2	(TG)17	34	6643877	6643910
chr11_1041	p2	(AG)8	16	6646848	6646863
chr11_1042	p2	(GT)6	12	6651696	6651707
chr11_1043	p3	(GAA)5	15	6659761	6659775
chr11_1044	p2	(TG)15	30	6662108	6662137
chr11_1045	p2	(TG)7	14	6670323	6670336
chr11_1046	c	(GT)8gcc(TG)6	31	6673021	6673051
chr11_1047	p2	(TG)22	44	6683565	6683608
chr11_1048	p2	(AC)6	12	6683724	6683735
chr11_1049	p2	(AC)13	26	6695372	6695397
chr11_1050	p2	(AC)7	14	6705447	6705460
chr11_1051	p2	(AC)20	40	6706300	6706339
chr11_1052	p2	(GT)7	14	6708484	6708497
chr11_1053	p2	(CA)15	30	6714620	6714649

chr11_1054	p2	(CA)12	24	6715161	6715184
chr11_1055	p2	(TG)18	36	6715572	6715607
chr11_1056	p4	(TTCT)5	20	6717922	6717941
chr11_1057	p2	(CA)6	12	6719350	6719361
chr11_1058	p2	(TG)7	14	6719887	6719900
chr11_1059	p2	(CA)12	24	6721123	6721146
chr11_1060	p2	(GT)6	12	6721292	6721303
chr11_1061	p2	(TG)7	14	6721708	6721721
chr11_1062	p2	(TG)7	14	6724907	6724920
chr11_1063	p2	(CA)23	46	6725303	6725348
chr11_1064	p2	(AC)6	12	6726920	6726931
chr11_1065	p2	(TG)6	12	6727102	6727113
chr11_1066	p2	(AC)21	42	6728841	6728882
chr11_1067	c	gtataaagcacttgggcatacggaatattagaacacaacaatgtgtgtgtg	106	6729041	6729146
chr11_1068	p2	(AC)7	14	6729272	6729285
chr11_1069	p2	(GA)10	20	6731599	6731618
chr11_1070	p2	(GT)6	12	6732211	6732222
chr11_1071	p2	(AC)10	20	6733085	6733104
chr11_1072	p2	(GT)7	14	6733218	6733231
chr11_1073	p2	(TG)7	14	6733414	6733427
chr11_1074	p2	(AC)16	32	6736668	6736699
chr11_1075	p2	(CA)10	20	6737395	6737414
chr11_1076	p2	(CA)6	12	6737548	6737559
chr11_1077	p2	(AC)6	12	6738070	6738081
chr11_1078	c	6aacactcatctgtttcaagccacagatggtcttttgaatggagtgggggtt	95	6741194	6741288
chr11_1079	p2	(TG)21	42	6741915	6741956
chr11_1080	p2	(AC)8	16	6747668	6747683
chr11_1081	p2	(TG)7	14	6748319	6748332
chr11_1082	p2	(TG)7	14	6748497	6748510
chr11_1083	p2	(CA)10	20	6749418	6749437
chr11_1084	p2	(TG)8	16	6750207	6750222
chr11_1085	p2	(AC)6	12	6750348	6750359
chr11_1086	p2	(GT)14	28	6751670	6751697
chr11_1087	c	(TG)11(AG)10	42	6752230	6752271
chr11_1088	p2	(AC)6	12	6752387	6752398
chr11_1089	p4	(TTTA)5	20	6754383	6754402
chr11_1090	c	(TC)10acacacg(CA)8	43	6756803	6756845
chr11_1091	c	(CT)11(CA)13	48	6756973	6757020
chr11_1092	p2	(AC)15	30	6757748	6757777
chr11_1093	c	gtgtctagggttgctggctgaatggaggagaaaattagactgatatgatag	114	6758533	6758646
chr11_1094	p2	(AC)6	12	6762505	6762516
chr11_1095	p2	(CA)9	18	6762962	6762979
chr11_1096	p2	(TG)16	32	6763843	6763874
chr11_1097	p2	(GT)12	24	6764022	6764045
chr11_1098	p2	(GT)12	24	6764768	6764791
chr11_1099	p4	(ATCA)5	20	6765062	6765081
chr11_1100	p2	(CA)26	52	6768239	6768290
chr11_1101	p2	(AC)9	18	6769201	6769218
chr11_1102	p2	(CA)6	12	6771737	6771748
chr11_1103	c	gagagcaggtggtcacctcagcagagtatggggggttgttagcttttacgg	94	6772003	6772096
chr11_1104	p2	(TG)16	32	6773569	6773600
chr11_1105	p2	(CA)7	14	6774618	6774631

chr11_1106	p2	(TG)9	18	6778901	6778918
chr11_1107	p2	(TG)7	14	6780501	6780514
chr11_1108	p2	(GT)18	36	6780760	6780795
chr11_1109	p2	(GT)11	22	6786853	6786874
chr11_1110	p2	(TG)18	36	6796853	6796888
chr11_1111	p2	(AG)9	18	6798664	6798681
chr11_1112	p2	(AC)8	16	6800579	6800594
chr11_1113	p2	(TC)12	24	6801374	6801397
chr11_1114	p2	(TG)6	12	6803825	6803836
chr11_1115	p2	(CA)18	36	6805631	6805666
chr11_1116	p2	(GT)6	12	6807163	6807174
chr11_1117	p2	(TG)8	16	6807305	6807320
chr11_1118	p2	(TG)9	18	6809630	6809647
chr11_1119	p2	(TG)13	26	6809820	6809845
chr11_1120	p2	(TG)18	36	6813178	6813213
chr11_1121	p2	(GT)6	12	6814608	6814619
chr11_1122	p2	(TA)6	12	6817734	6817745
chr11_1123	p2	(AC)11	22	6819324	6819345
chr11_1124	p2	(TG)9	18	6822217	6822234
chr11_1125	p2	(TG)13	26	6822339	6822364
chr11_1126	c	(CA)6aacaagt(TG)8	35	6823154	6823188
chr11_1127	c	caaggtgattgattgaataaacagctgtcagtagttggagataacctcactag	184	6824015	6824198
chr11_1128	p2	(CA)6	12	6824564	6824575
chr11_1129	p2	(CA)8	16	6824781	6824796
chr11_1130	c	ctagcctgcttaaatcacagctcacttaagaaaaagccatatcacctctctag	105	6824938	6825042
chr11_1131	p2	(TA)7	14	6830652	6830665
chr11_1132	p2	(AC)17	34	6835042	6835075
chr11_1133	p2	(CA)18	36	6837564	6837599
chr11_1134	p2	(CA)6	12	6838318	6838329
chr11_1135	p3	(TCC)5	15	6838438	6838452
chr11_1136	p2	(AG)6	12	6841528	6841539
chr11_1137	p2	(TG)6	12	6841968	6841979
chr11_1138	p2	(AC)9	18	6844660	6844677
chr11_1139	c	acacacattgctatagctctgtcaggactaagccttgtaaaatgtgatattgc	86	6844901	6844986
chr11_1140	p4	(TTAA)6	24	6845345	6845368
chr11_1141	p2	(GT)11	22	6851061	6851082
chr11_1142	p2	(TG)16	32	6855314	6855345
chr11_1143	p2	(AC)9	18	6859405	6859422
chr11_1144	p2	(CA)17	34	6860481	6860514
chr11_1145	c	ctgaagggagcagtgaggcagcaggaagggtttgtctgagcgttcgtcact	143	6860743	6860885
chr11_1146	p2	(TG)8	16	6863498	6863513
chr11_1147	p4	(TTTA)5	20	6863815	6863834
chr11_1148	c	(CA)12ctgccatactgctcttatcccttagaatagcc(TG)20	96	6864269	6864364
chr11_1149	c	(GT)6gactttgcacagggtagacttagtaaaaa(TC)8	57	6867976	6868032
chr11_1150	p2	(CA)8	16	6868138	6868153
chr11_1151	p2	(TG)6	12	6874723	6874734
chr11_1152	c	tgccgctcacctccatccagtgctggcatgtccattgtgaagaatgctggct	141	6877310	6877450
chr11_1153	p2	(AC)10	20	6883531	6883550
chr11_1154	c*	(TG)7(GTGC)5*	31	6883927	6883957
chr11_1155	p2	(TC)10	20	6884494	6884513
chr11_1156	p2	(CA)8	16	6885248	6885263
chr11_1157	p4	(TCTA)5	20	6890022	6890041

chr11_1158	p2	(TG)7	14	6892370	6892383
chr11_1159	p2	(GT)12	24	6894346	6894369
chr11_1160	p2	(GT)8	16	6898432	6898447
chr11_1161	p2	(GT)11	22	6902459	6902480
chr11_1162	p2	(TG)6	12	6903221	6903232
chr11_1163	p2	(TG)16	32	6903618	6903649
chr11_1164	p2	(TG)6	12	6909387	6909398
chr11_1165	p2	(CA)6	12	6931139	6931150
chr11_1166	p2	(AC)6	12	6933175	6933186
chr11_1167	p2	(AC)6	12	6933536	6933547
chr11_1168	p2	(CA)8	16	6939077	6939092
chr11_1169	p2	(TC)6	12	6945344	6945355
chr11_1170	p2	(TC)6	12	6946787	6946798
chr11_1171	p2	(AC)6	12	6946942	6946953
chr11_1172	p2	(CA)13	26	6952095	6952120
chr11_1173	p2	(TG)6	12	6955043	6955054
chr11_1174	p2	(AC)14	28	6964179	6964206
chr11_1175	c	(CT)6ttctttcctctcttttgtcag(AC)8	49	6975893	6975941
chr11_1176	p5	(AAATC)5	25	6987415	6987439
chr11_1177	c	(GT)12ggga(GT)7	42	6987851	6987892
chr11_1178	p2	(GA)6	12	6989150	6989161
chr11_1179	p2	(TC)6	12	6989858	6989869
chr11_1180	c	(GT)7(GA)6	26	6992820	6992845
chr11_1181	p2	(TG)8	16	6994777	6994792
chr11_1182	p2	(TG)6	12	7004179	7004190
chr11_1183	p2	(AC)7	14	7008826	7008839
chr11_1184	p2	(GA)6	12	7009563	7009574
chr11_1185	p2	(GA)9	18	7010001	7010018
chr11_1186	p2	(TG)17	34	7014651	7014684
chr11_1187	p2	(AT)11	22	7018238	7018259
chr11_1188	p2	(GT)6	12	7022368	7022379
chr11_1189	p2	(GA)6	12	7024240	7024251
chr11_1190	p3	(AGC)5	15	7026461	7026475
chr11_1191	p2	(GT)6	12	7029044	7029055
chr11_1192	p2	(CA)8	16	7032402	7032417
chr11_1193	p2	(CA)7	14	7036720	7036733
chr11_1194	p2	(GT)16	32	7038342	7038373
chr11_1195	p2	(GT)13	26	7052983	7053008
chr11_1196	p2	(TG)12	24	7056590	7056613
chr11_1197	p2	(AT)10	20	7064397	7064416
chr11_1198	p2	(GT)13	26	7070614	7070639
chr11_1199	p2	(AC)6	12	7071982	7071993
chr11_1200	p2	(AG)10	20	7073613	7073632
chr11_1201	p2	(TG)11	22	7081884	7081905
chr11_1202	p2	(TG)6	12	7087183	7087194
chr11_1203	p2	(CT)6	12	7100175	7100186
chr11_1204	p3	(ATC)5	15	7100477	7100491
chr11_1205	c	(ATA)6ca(TAT)5	35	7100755	7100789
chr11_1206	p2	(TA)6	12	7102854	7102865
chr11_1207	p4	(AACA)5	20	7105008	7105027
chr11_1208	p2	(AT)9	18	7115162	7115179
chr11_1209	p2	(GA)11	22	7144367	7144388

chr11_1210	p2	(GA)6	12	7156466	7156477
chr11_1211	p3	(AGT)7	21	7179683	7179703
chr11_1212	p2	(TA)7	14	7180575	7180588
chr11_1213	p2	(AC)15	30	7183680	7183709
chr11_1214	p4	(TTTG)5	20	7186759	7186778
chr11_1215	p2	(AC)6	12	7188592	7188603
chr11_1216	p2	(AG)8	16	7191296	7191311
chr11_1217	p2	(AG)6	12	7195802	7195813
chr11_1218	p3	(GTT)5	15	7198076	7198090
chr11_1219	p3	(TTA)8	24	7204003	7204026
chr11_1220	p2	(AC)13	26	7210009	7210034
chr11_1221	p2	(AC)6	12	7214245	7214256
chr11_1222	p2	(AC)12	24	7218293	7218316
chr11_1223	p2	(AC)20	40	7226652	7226691
chr11_1224	p2	(GA)7	14	7241663	7241676
chr11_1225	p2	(AC)7	14	7246687	7246700
chr11_1226	p2	(AC)6	12	7258547	7258558
chr11_1227	c	)7ccacacatcttgtagcattatatgaatcattgcagtgtaaaagtcataa(A	79	7259722	7259800
chr11_1228	p2	(AC)9	18	7259943	7259960
chr11_1229	p2	(TC)8	16	7262124	7262139
chr11_1230	p2	(GT)6	12	7266314	7266325
chr11_1231	p3	(CCT)5	15	7273211	7273225
chr11_1232	p2	(CA)11	22	7274226	7274247
chr11_1233	p2	(TG)11	22	7274797	7274818
chr11_1234	p2	(AC)16	32	7275722	7275753
chr11_1235	c	(AG)7acagagagac(AG)6	36	7281843	7281878
chr11_1236	p2	(AC)10	20	7287844	7287863
chr11_1237	p2	(AC)20	40	7294995	7295034
chr11_1238	p2	(CA)20	40	7297371	7297410
chr11_1239	p4	(AAAT)5	20	7299495	7299514
chr11_1240	p2	(GT)17	34	7304220	7304253
chr11_1241	p2	(TG)18	36	7304629	7304664
chr11_1242	p3	(TAA)6	18	7305099	7305116
chr11_1243	p2	(GT)6	12	7310283	7310294
chr11_1244	p3	(CAG)8	24	7310905	7310928
chr11_1245	p2	(CA)6	12	7311500	7311511
chr11_1246	p2	(AC)7	14	7319031	7319044
chr11_1247	p2	(AC)8	16	7330318	7330333
chr11_1248	p2	(CA)24	48	7340976	7341023
chr11_1249	p2	(CA)11	22	7341409	7341430
chr11_1250	p2	(CA)6	12	7343201	7343212
chr11_1251	c	(CA)6aacccgtgtgccctgtgatgcagtctga(GT)9	58	7345016	7345073
chr11_1252	p2	(CA)22	44	7346062	7346105
chr11_1253	p2	(GA)6	12	7347093	7347104
chr11_1254	p2	(AC)6	12	7361397	7361408
chr11_1255	p5	(TTTAC)5	25	7363025	7363049
chr11_1256	p2	(AC)11	22	7365001	7365022
chr11_1257	p2	(GT)7	14	7365360	7365373
chr11_1258	p2	(CA)6	12	7365701	7365712
chr11_1259	p2	(CA)7	14	7371541	7371554
chr11_1260	p2	(AC)10	20	7373631	7373650
chr11_1261	p2	(GT)14	28	7374918	7374945

chr11_1262	p2	(TG)15	30	7375462	7375491
chr11_1263	p2	(CA)22	44	7382427	7382470
chr11_1264	p2	(TG)8	16	7387474	7387489
chr11_1265	p2	(CA)14	28	7389976	7390003
chr11_1266	p2	(TG)7	14	7398226	7398239
chr11_1267	p2	(AC)8	16	7409836	7409851
chr11_1268	p2	(GT)28	56	7410865	7410920
chr11_1269	p4	(AATA)5	20	7421948	7421967
chr11_1270	p2	(GT)7	14	7428488	7428501
chr11_1271	p2	(TG)10	20	7430997	7431016
chr11_1272	p2	(CA)19	38	7431281	7431318
chr11_1273	p2	(GT)8	16	7431548	7431563
chr11_1274	p2	(AC)7	14	7437935	7437948
chr11_1275	p2	(CA)8	16	7438415	7438430
chr11_1276	p2	(TC)6	12	7443510	7443521
chr11_1277	p2	(TC)11	22	7443756	7443777
chr11_1278	p2	(AC)12	24	7445518	7445541
chr11_1279	p2	(GT)10	20	7449565	7449584
chr11_1280	p4	(CATC)5	20	7452227	7452246
chr11_1281	p2	(TC)6	12	7457557	7457568
chr11_1282	p3	(CTC)6	18	7460617	7460634
chr11_1283	p2	(GT)6	12	7461330	7461341
chr11_1284	p3	(CCT)5	15	7461864	7461878
chr11_1285	p2	(TG)14	28	7465783	7465810
chr11_1286	p2	(GT)11	22	7466094	7466115
chr11_1287	p2	(TC)7	14	7466333	7466346
chr11_1288	c	aaatcactcccatcagaagctcatcattgatgactaaacagctcacttgagta	107	7470307	7470413
chr11_1289	p2	(AG)6	12	7472926	7472937
chr11_1290	p2	(CA)12	24	7475039	7475062
chr11_1291	p2	(CA)6	12	7477506	7477517
chr11_1292	p2	(CA)22	44	7478120	7478163
chr11_1293	p3	(AAC)5	15	7478281	7478295
chr11_1294	p2	(CA)6	12	7484898	7484909
chr11_1295	p2	(CT)6	12	7489213	7489224
chr11_1296	p2	(GA)6	12	7489382	7489393
chr11_1297	p2	(AC)12	24	7489595	7489618
chr11_1298	c	(CT)6t(TC)6	25	7491561	7491585
chr11_1299	p2	(TG)6	12	7492477	7492488
chr11_1300	p2	(TG)6	12	7503788	7503799
chr11_1301	p2	(AG)6	12	7506163	7506174
chr11_1302	p2	(AC)10	20	7507274	7507293
chr11_1303	p2	(CA)6	12	7509962	7509973
chr11_1304	p3	(ATC)5	15	7519229	7519243
chr11_1305	p2	(TG)14	28	7519907	7519934
chr11_1306	p2	(AC)7	14	7520238	7520251
chr11_1307	p2	(GT)14	28	7520627	7520654
chr11_1308	p2	(TG)7	14	7521719	7521732
chr11_1309	p2	(CA)7	14	7523276	7523289
chr11_1310	p2	(TA)6	12	7524644	7524655
chr11_1311	p2	(TA)6	12	7524916	7524927
chr11_1312	p3	(AAT)5	15	7525329	7525343
chr11_1313	c	(AT)6acacatatacatatatatacatatacatatatatacat(AC)8	66	7529764	7529829

chr11_1314	p2	(TA)6	12	7540537	7540548
chr11_1315	p2	(GT)7	14	7544239	7544252
chr11_1316	p2	(TG)14	28	7550520	7550547
chr11_1317	p2	(GT)8	16	7552845	7552860
chr11_1318	p2	(AT)7	14	7555357	7555370
chr11_1319	p3	(GTA)6	18	7555488	7555505
chr11_1320	p2	(GT)14	28	7560480	7560507
chr11_1321	p4	(TTGT)5	20	7572744	7572763
chr11_1322	p2	(AC)18	36	7582755	7582790
chr11_1323	p2	(AC)19	38	7583951	7583988
chr11_1324	p3	(TTC)5	15	7585065	7585079
chr11_1325	p4	(ATAG)6	24	7590084	7590107
chr11_1326	p2	(GA)6	12	7615291	7615302
chr11_1327	p2	(TG)6	12	7620084	7620095
chr11_1328	p3	(AAG)5	15	7624632	7624646
chr11_1329	p2	(TG)17	34	7626752	7626785
chr11_1330	p2	(TG)10	20	7635510	7635529
chr11_1331	p2	(AT)13	26	7636780	7636805
chr11_1332	p3	(TTG)7	21	7654605	7654625
chr11_1333	p2	(CT)6	12	7660079	7660090
chr11_1334	p2	(CA)26	52	7661806	7661857
chr11_1335	p4	(CTGT)5	20	7663621	7663640
chr11_1336	p2	(GA)6	12	7674459	7674470
chr11_1337	p4	(AAAC)5	20	7680988	7681007
chr11_1338	p3	(CAC)5	15	7705167	7705181
chr11_1339	p2	(CT)13	26	7709549	7709574
chr11_1340	c	(AG)6ttaaagttgattttataaaa(TG)11	54	7715080	7715133
chr11_1341	p4	(TAAA)5	20	7720922	7720941
chr11_1342	p3	(TCA)5	15	7730180	7730194
chr11_1343	p2	(CA)6	12	7736654	7736665
chr11_1344	p2	(GA)6	12	7738153	7738164
chr11_1345	p3	(ATA)5	15	7752155	7752169
chr11_1346	p2	(TG)12	24	7775655	7775678
chr11_1347	c	(AT)6(AC)6	24	7780402	7780425
chr11_1348	p2	(GT)6	12	7785964	7785975
chr11_1349	p4	(TAGA)12	48	7792252	7792299
chr11_1350	p2	(CA)16	32	7792695	7792726
chr11_1351	p2	(GT)9	18	7793497	7793514
chr11_1352	p3	(AGC)6	18	7814621	7814638
chr11_1353	p2	(TG)8	16	7823997	7824012
chr11_1354	p2	(GT)10	20	7849797	7849816
chr11_1355	c	(TG)12tacat(AC)6	41	7855050	7855090
chr11_1356	p2	(TC)6	12	7879486	7879497
chr11_1357	p2	(GT)18	36	7885487	7885522
chr11_1358	p3	(TTC)5	15	7894504	7894518
chr11_1359	p2	(AC)6	12	7904220	7904231
chr11_1360	p2	(CT)6	12	7910133	7910144
chr11_1361	p4	(TTCA)6	24	7917740	7917763
chr11_1362	p4	(TTCA)6	24	7919671	7919694
chr11_1363	p2	(TG)10	20	7924429	7924448
chr11_1364	c	(TATC)9tg(TCTA)6	62	7928390	7928451
chr11_1365	p2	(TA)7	14	7935697	7935710

chr11_1366	p2	(GA)6	12	7941173	7941184
chr11_1367	p4	(TCTA)12	48	7942892	7942939
chr11_1368	p2	(AC)20	40	7949752	7949791
chr11_1369	c	(AC)6aaaccttcacagctcacttga(GT)12	57	7956851	7956907
chr11_1370	p3	(TAA)8	24	7963261	7963284
chr11_1371	p2	(GT)9	18	7963951	7963968
chr11_1372	p3	(ATC)5	15	7987887	7987901
chr11_1373	p2	(TG)11	22	7993955	7993976
chr11_1374	p2	(AT)6	12	8009411	8009422
chr11_1375	p3	(AAT)8	24	8015143	8015166
chr11_1376	c	(TG)7c(GT)21	57	8032621	8032677
chr11_1377	p2	(AC)9	18	8041471	8041488
chr11_1378	p2	(AC)9	18	8046206	8046223
chr11_1379	p2	(TG)7	14	8047864	8047877
chr11_1380	p3	(AAG)5	15	8066486	8066500
chr11_1381	p2	(TG)26	52	8067176	8067227
chr11_1382	p2	(AC)6	12	8067398	8067409
chr11_1383	p2	(TG)6	12	8068330	8068341
chr11_1384	p4	(ATGG)5	20	8069351	8069370
chr11_1385	p2	(TA)9	18	8077014	8077031
chr11_1386	p3	(AAG)9	27	8098229	8098255
chr11_1387	p2	(TC)7	14	8110021	8110034
chr11_1388	p2	(GA)7	14	8110794	8110807
chr11_1389	p2	(TA)7	14	8117649	8117662
chr11_1390	p2	(GT)6	12	8125390	8125401
chr11_1391	p3	(AGA)5	15	8132523	8132537
chr11_1392	p2	(TA)6	12	8138443	8138454
chr11_1393	p2	(AC)10	20	8143189	8143208
chr11_1394	p2	(AC)16	32	8150670	8150701
chr11_1395	p2	(CA)6	12	8154475	8154486
chr11_1396	p2	(AT)6	12	8159204	8159215
chr11_1397	p2	(AC)8	16	8166321	8166336
chr11_1398	p2	(TG)18	36	8173408	8173443
chr11_1399	p2	(AT)8	16	8177039	8177054
chr11_1400	p4	(AAGA)5	20	8203085	8203104
chr11_1401	p2	(AG)7	14	8208959	8208972
chr11_1402	p4	(TTGA)5	20	8214277	8214296
chr11_1403	p2	(TG)10	20	8245136	8245155
chr11_1404	p2	(AG)11	22	8250477	8250498
chr11_1405	p2	(TC)7	14	8251311	8251324
chr11_1406	p3	(GGT)6	18	8261159	8261176
chr11_1407	p3	(CCA)5	15	8262831	8262845
chr11_1408	c	(ATTT)5(TG)7	34	8268857	8268890
chr11_1409	p2	(TG)23	46	8273628	8273673
chr11_1410	p2	(TC)7	14	8280415	8280428
chr11_1411	p2	(GT)8	16	8296278	8296293
chr11_1412	p2	(GA)13	26	8301921	8301946
chr11_1413	p2	(CA)18	36	8302455	8302490
chr11_1414	p2	(AC)13	26	8302609	8302634
chr11_1415	p2	(GT)6	12	8311705	8311716
chr11_1416	p2	(GA)6	12	8314264	8314275
chr11_1417	p2	(AC)10	20	8318521	8318540

chr11_1418	p2	(TG)12	24	8328854	8328877
chr11_1419	p2	(AC)17	34	8331261	8331294
chr11_1420	p2	(GA)6	12	8335717	8335728
chr11_1421	p2	(CA)8	16	8339259	8339274
chr11_1422	p2	(TG)21	42	8343076	8343117
chr11_1423	p3	(ATA)5	15	8344390	8344404
chr11_1424	p2	(TG)7	14	8347719	8347732
chr11_1425	p2	(GT)7	14	8347873	8347886
chr11_1426	p2	(TG)6	12	8348094	8348105
chr11_1427	p2	(GT)8	16	8352444	8352459
chr11_1428	p2	(CT)7	14	8357553	8357566
chr11_1429	p2	(TG)6	12	8359862	8359873
chr11_1430	p2	(AC)8	16	8366264	8366279
chr11_1431	p2	(CT)6	12	8367721	8367732
chr11_1432	p2	(AC)9	18	8369457	8369474
chr11_1433	p4	(TCCA)5	20	8373167	8373186
chr11_1434	p2	(GA)8	16	8376913	8376928
chr11_1435	c	gcagcataggccacgctttttcttgtcaccacaaggggttgcagtgtcactg	144	8377547	8377690
chr11_1436	c	(TG)6attgctgccatttttgttttgaccttctgtcagttatataaa(TG)29	112	8377995	8378106
chr11_1437	p2	(AC)14	28	8386730	8386757
chr11_1438	p3	(ATG)5	15	8387669	8387683
chr11_1439	p3	(ATT)6	18	8392471	8392488
chr11_1440	p2	(AC)7	14	8395757	8395770
chr11_1441	p2	(CA)15	30	8397919	8397948
chr11_1442	p2	(AG)6	12	8407493	8407504
chr11_1443	p2	(GT)8	16	8409672	8409687
chr11_1444	p2	(CA)14	28	8413020	8413047
chr11_1445	p2	(TG)7	14	8413489	8413502
chr11_1446	p2	(AC)9	18	8415257	8415274
chr11_1447	c	(TC)8(AC)10	36	8419382	8419417
chr11_1448	p2	(CA)12	24	8433178	8433201
chr11_1449	p2	(TG)7	14	8446413	8446426
chr11_1450	p5	(TTGAC)5	25	8448177	8448201
chr11_1451	p2	(GT)8	16	8452819	8452834
chr11_1452	p2	(TA)10	20	8455285	8455304
chr11_1453	p2	(GT)6	12	8455782	8455793
chr11_1454	p2	(GT)6	12	8456727	8456738
chr11_1455	p2	(GT)6	12	8457005	8457016
chr11_1456	p2	(GT)16	32	8459914	8459945
chr11_1457	p2	(AT)9	18	8463190	8463207
chr11_1458	p2	(TG)12	24	8466592	8466615
chr11_1459	p2	(TG)10	20	8467358	8467377
chr11_1460	p2	(CA)16	32	8467531	8467562
chr11_1461	p2	(AG)6	12	8467743	8467754
chr11_1462	p2	(TG)8	16	8477166	8477181
chr11_1463	p2	(GT)12	24	8480718	8480741
chr11_1464	p2	(CA)12	24	8483035	8483058
chr11_1465	p5	(AGGCA)5	25	8485771	8485795
chr11_1466	p2	(TG)10	20	8487880	8487899
chr11_1467	p2	(TG)13	26	8491012	8491037
chr11_1468	p2	(TG)6	12	8491802	8491813
chr11_1469	p2	(TG)17	34	8493857	8493890

chr11_1470	p4	(ATAG)5	20	8498739	8498758
chr11_1471	c	(TG)15tat(GA)10	53	8503041	8503093
chr11_1472	p2	(TG)7	14	8518148	8518161
chr11_1473	p2	(GT)10	20	8520911	8520930
chr11_1474	p2	(TG)13	26	8521056	8521081
chr11_1475	p2	(TG)7	14	8521518	8521531
chr11_1476	p2	(CA)6	12	8527753	8527764
chr11_1477	c	(CTAT)5ctg(TCTA)5(TCTG)5	63	8529861	8529923
chr11_1478	c	(TG)6cgcatg(TGCA)5	38	8533179	8533216
chr11_1479	p4	(TGTC)7	28	8559403	8559430
chr11_1480	p2	(TG)9	18	8562938	8562955
chr11_1481	p2	(GA)11	22	8564072	8564093
chr11_1482	p2	(CA)8	16	8567009	8567024
chr11_1483	p2	(TC)6	12	8572797	8572808
chr11_1484	p2	(AG)7	14	8576912	8576925
chr11_1485	p2	(TG)7	14	8583554	8583567
chr11_1486	p2	(CT)11	22	8585072	8585093
chr11_1487	p2	(CA)6	12	8586509	8586520
chr11_1488	c	(TG)11(AG)10	42	8593660	8593701
chr11_1489	p2	(TG)10	20	8603795	8603814
chr11_1490	p2	(CA)13	26	8606280	8606305
chr11_1491	p2	(GT)18	36	8609477	8609512
chr11_1492	p2	(GT)7	14	8612110	8612123
chr11_1493	p2	(TG)10	20	8612226	8612245
chr11_1494	p2	(AC)6	12	8615296	8615307
chr11_1495	p2	(AT)7	14	8618268	8618281
chr11_1496	p4	(TCCA)6	24	8621423	8621446
chr11_1497	p3	(ATT)6	18	8629230	8629247
chr11_1498	p2	(TG)9	18	8646990	8647007
chr11_1499	p2	(CA)8	16	8653614	8653629
chr11_1500	p2	(TG)6	12	8665767	8665778
chr11_1501	p4	(AAAT)7	28	8672668	8672695
chr11_1502	p2	(CA)25	50	8678373	8678422
chr11_1503	p2	(GT)20	40	8695599	8695638
chr11_1504	p2	(TC)6	12	8710268	8710279
chr11_1505	p2	(CA)10	20	8713296	8713315
chr11_1506	p2	(CA)6	12	8716989	8717000
chr11_1507	p2	(GT)6	12	8725184	8725195
chr11_1508	p2	(TG)8	16	8727166	8727181
chr11_1509	p2	(TG)6	12	8739980	8739991
chr11_1510	p4	(TTTG)6	24	8754906	8754929
chr11_1511	p2	(GT)6	12	8757080	8757091
chr11_1512	c	(TC)11(AC)9	40	8758329	8758368
chr11_1513	p2	(CA)23	46	8764180	8764225
chr11_1514	p2	(AC)11	22	8772114	8772135
chr11_1515	c	(TGT)5aatgccagtgtttgcacaaatgcaggaatgtgtgtgtg(TA)1[illegible]	77	8773881	8773957
chr11_1516	p3	(TAT)20	60	8777421	8777480
chr11_1517	p2	(GT)14	28	8797402	8797429
chr11_1518	p2	(AC)6	12	8804925	8804936
chr11_1519	p3	(GAG)6	18	8815059	8815076
chr11_1520	p2	(AG)7	14	8817262	8817275
chr11_1521	p2	(TG)17	34	8817686	8817719

chr11_1522	p2	(TA)7	14	8829044	8829057
chr11_1523	p2	(AT)6	12	8840766	8840777
chr11_1524	p2	(AC)8	16	8841121	8841136
chr11_1525	p2	(TA)6	12	8842885	8842896
chr11_1526	p2	(AG)9	18	8843536	8843553
chr11_1527	p2	(CT)14	28	8844256	8844283
chr11_1528	p2	(CA)6	12	8844484	8844495
chr11_1529	c	(TG)7ttggg(GT)13	45	8844668	8844712
chr11_1530	p2	(TG)8	16	8845315	8845330
chr11_1531	p2	(TG)7	14	8847241	8847254
chr11_1532	p2	(TC)9	18	8850641	8850658
chr11_1533	p2	(GT)11	22	8850999	8851020
chr11_1534	p2	(AC)9	18	8854727	8854744
chr11_1535	p2	(TG)6	12	8860677	8860688
chr11_1536	p2	(TC)6	12	8864230	8864241
chr11_1537	p2	(CT)6	12	8870986	8870997
chr11_1538	p2	(CT)26	52	8873125	8873176
chr11_1539	p3	(CTT)6	18	8878679	8878696
chr11_1540	p2	(GT)10	20	8888113	8888132
chr11_1541	p2	(GT)19	38	8891065	8891102
chr11_1542	p2	(GA)6	12	8895307	8895318
chr11_1543	c	[illegible]9ttatatcaatcagctccctgtatttccattcaaaagatatttctctctctattc([illegible]	84	8897222	8897305
chr11_1544	p3	(CTC)6	18	8897491	8897508
chr11_1545	p2	(AC)12	24	8898714	8898737
chr11_1546	p2	(CA)12	24	8903825	8903848
chr11_1547	p2	(GT)12	24	8905268	8905291
chr11_1548	p2	(GT)31	62	8909624	8909685
chr11_1549	p2	(AC)6	12	8912967	8912978
chr11_1550	p2	(CA)10	20	8926331	8926350
chr11_1551	p3	(ATT)5	15	8928380	8928394
chr11_1552	p2	(AC)6	12	8947205	8947216
chr11_1553	p2	(AT)6	12	8951543	8951554
chr11_1554	p2	(AC)12	24	8964986	8965009
chr11_1555	c	(TA)15gcctaacatttatatc(TA)13	72	8966160	8966231
chr11_1556	p2	(TG)17	34	8969732	8969765
chr11_1557	c	(CA)6cg(CA)11	36	8978999	8979034
chr11_1558	p3	(TTA)9	27	9002859	9002885
chr11_1559	p3	(CAG)5	15	9004487	9004501
chr11_1560	p2	(GT)6	12	9012575	9012586
chr11_1561	p2	(TG)7	14	9024570	9024583
chr11_1562	p2	(AC)13	26	9028448	9028473
chr11_1563	c	(AT)6(TG)8	28	9028724	9028751
chr11_1564	p2	(CA)19	38	9028922	9028959
chr11_1565	p3	(GAG)9	27	9030253	9030279
chr11_1566	p2	(AC)13	26	9031188	9031213
chr11_1567	p2	(AG)6	12	9031333	9031344
chr11_1568	p2	(CA)7	14	9034254	9034267
chr11_1569	p2	(AG)6	12	9034526	9034537
chr11_1570	p2	(AG)7	14	9037199	9037212
chr11_1571	c	(TG)20(AG)12	64	9037965	9038028
chr11_1572	p2	(GT)6	12	9038818	9038829
chr11_1573	p2	(CT)11	22	9039466	9039487

chr11_1574	p2	(GT)17	34	9041367	9041400
chr11_1575	p2	(AC)6	12	9043182	9043193
chr11_1576	c	(TG)6a(GT)9	31	9052205	9052235
chr11_1577	p2	(TG)6	12	9052902	9052913
chr11_1578	p2	(GT)12	24	9055636	9055659
chr11_1579	p2	(AC)6	12	9056487	9056498
chr11_1580	p2	(AC)11	22	9059169	9059190
chr11_1581	p2	(TG)10	20	9060301	9060320
chr11_1582	p2	(CA)13	26	9062134	9062159
chr11_1583	p2	(AC)7	14	9066470	9066483
chr11_1584	p2	(TG)7	14	9075959	9075972
chr11_1585	p2	(TG)12	24	9084744	9084767
chr11_1586	p2	(GT)24	48	9091012	9091059
chr11_1587	c	caaatgaatcaccgtgctgagtcactcaccccctccccatctctccccttga	151	9092639	9092789
chr11_1588	p2	(AG)8	16	9093166	9093181
chr11_1589	p3	(TAA)5	15	9093621	9093635
chr11_1590	p2	(TG)8	16	9101381	9101396
chr11_1591	p2	(CA)6	12	9102293	9102304
chr11_1592	p3	(ATA)5	15	9122714	9122728
chr11_1593	p3	(TAG)5	15	9143788	9143802
chr11_1594	p4	(TTTA)6	24	9147978	9148001
chr11_1595	p2	(CA)12	24	9148583	9148606
chr11_1596	p3	(CAG)5	15	9149071	9149085
chr11_1597	p2	(TG)7	14	9154997	9155010
chr11_1598	c	tacatacataaccacagatgggcctaaaaacaagcaaccaaataacatttt	104	9155229	9155332
chr11_1599	p2	(AC)35	70	9160522	9160591
chr11_1600	p2	(AC)6	12	9164346	9164357
chr11_1601	p3	(TAC)5	15	9164659	9164673
chr11_1602	p2	(GT)6	12	9165245	9165256
chr11_1603	p2	(AC)10	20	9168102	9168121
chr11_1604	p2	(AC)19	38	9202024	9202061
chr11_1605	p3	(ATT)6	18	9252536	9252553
chr11_1606	p3	(TCC)5	15	9258286	9258300
chr11_1607	p2	(TG)7	14	9258411	9258424
chr11_1608	p2	(TG)6	12	9269032	9269043
chr11_1609	p2	(TG)9	18	9270235	9270252
chr11_1610	p2	(GT)6	12	9272642	9272653
chr11_1611	p2	(CA)9	18	9275879	9275896
chr11_1612	c	gtcctgaaattcgctttacaagggacctgaggagtgatggaattcagacaa	117	9277631	9277747
chr11_1613	p4	(CTGC)6	24	9278213	9278236
chr11_1614	p2	(CA)14	28	9285497	9285524
chr11_1615	p2	(TG)13	26	9285925	9285950
chr11_1616	p2	(AC)14	28	9286099	9286126
chr11_1617	p2	(GT)7	14	9286996	9287009
chr11_1618	p2	(AC)6	12	9288774	9288785
chr11_1619	c	(TG)18ta(TG)6	50	9289280	9289329
chr11_1620	p2	(AC)12	24	9289954	9289977
chr11_1621	p3	(GAG)6	18	9290561	9290578
chr11_1622	p2	(GT)17	34	9294202	9294235
chr11_1623	p2	(GT)6	12	9298734	9298745
chr11_1624	p2	(TG)10	20	9298898	9298917
chr11_1625	p2	(CT)6	12	9299100	9299111

chr11_1626	p2	(GT)12	24	9303909	9303932
chr11_1627	p2	(TG)8	16	9304356	9304371
chr11_1628	p2	(AC)12	24	9305534	9305557
chr11_1629	p2	(CA)12	24	9306579	9306602
chr11_1630	p3	(GGT)5	15	9307176	9307190
chr11_1631	p2	(AC)8	16	9308885	9308900
chr11_1632	p2	(TC)6	12	9313922	9313933
chr11_1633	p2	(CA)7	14	9314187	9314200
chr11_1634	p2	(GA)7	14	9314375	9314388
chr11_1635	p2	(AC)17	34	9318976	9319009
chr11_1636	p2	(CA)17	34	9319293	9319326
chr11_1637	p2	(GT)7	14	9320069	9320082
chr11_1638	p2	(GT)6	12	9322523	9322534
chr11_1639	p3	(GTT)7	21	9328166	9328186
chr11_1640	p2	(AC)10	20	9328370	9328389
chr11_1641	p2	(TG)25	50	9330344	9330393
chr11_1642	p2	(GA)7	14	9334721	9334734
chr11_1643	p2	(AC)6	12	9334841	9334852
chr11_1644	p4	(ATGG)5	20	9344649	9344668
chr11_1645	p3	(TAA)5	15	9347890	9347904
chr11_1646	p2	(CA)10	20	9349811	9349830
chr11_1647	p2	(CA)8	16	9349980	9349995
chr11_1648	p2	(GA)8	16	9350552	9350567
chr11_1649	p2	(TG)6	12	9353166	9353177
chr11_1650	c	(CA)7cg(CA)7	30	9360018	9360047
chr11_1651	p2	(AC)7	14	9360262	9360275
chr11_1652	p2	(GT)6	12	9360450	9360461
chr11_1653	p2	(AC)6	12	9367123	9367134
chr11_1654	p3	(TGC)5	15	9371682	9371696
chr11_1655	p2	(GT)6	12	9372592	9372603
chr11_1656	p2	(CT)7	14	9372775	9372788
chr11_1657	c	(CT)7gtctt(TC)8	35	9373516	9373550
chr11_1658	p2	(TC)9	18	9373663	9373680
chr11_1659	c	(CT)8cc(CT)8	34	9378296	9378329
chr11_1660	p3	(AAT)5	15	9386193	9386207
chr11_1661	p4	(AAAC)7	28	9386598	9386625
chr11_1662	c	(GA)31gtgggagaaac(AG)13	99	9388111	9388209
chr11_1663	c	(TCT)8tcg(GCT)5	42	9393220	9393261
chr11_1664	p3	(ATT)7	21	9411133	9411153
chr11_1665	p2	(AG)8	16	9411404	9411419
chr11_1666	p4	(CTTT)5	20	9412200	9412219
chr11_1667	p3	(ATT)8	24	9417935	9417958
chr11_1668	p2	(TA)12	24	9447754	9447777
chr11_1669	p5	(ATTTG)5	25	9451174	9451198
chr11_1670	c	tactcacacatacatatgctcctgtctgcatatttattgaaagtacaattagtta	145	9456656	9456800
chr11_1671	p2	(CA)6	12	9460124	9460135
chr11_1672	p2	(CT)9	18	9460924	9460941
chr11_1673	p2	(CA)16	32	9461409	9461440
chr11_1674	p2	(AG)6	12	9479511	9479522
chr11_1675	p2	(CA)18	36	9504570	9504605
chr11_1676	p2	(AG)11	22	9508015	9508036
chr11_1677	p2	(TA)6	12	9518362	9518373

chr11_1678	p2	(CA)10	20	9522427	9522446
chr11_1679	p4	(TCTA)5	20	9523007	9523026
chr11_1680	p2	(GA)6	12	9536356	9536367
chr11_1681	p2	(TG)12	24	9553362	9553385
chr11_1682	p2	(GA)6	12	9554291	9554302
chr11_1683	p2	(AC)16	32	9555271	9555302
chr11_1684	p2	(TG)12	24	9558893	9558916
chr11_1685	p2	(AC)26	52	9563055	9563106
chr11_1686	c	(GA)6ttaaaaggcata(CT)6	36	9566316	9566351
chr11_1687	p2	(AT)6	12	9571310	9571321
chr11_1688	p2	(AT)7	14	9571455	9571468
chr11_1689	p2	(TG)6	12	9582973	9582984
chr11_1690	p2	(GT)16	32	9584084	9584115
chr11_1691	p2	(TC)6	12	9588793	9588804
chr11_1692	p2	(AC)10	20	9591873	9591892
chr11_1693	c	atcagcgaaaagtgggcaggcactgtagataagaaattaaaaatcattttaa	115	9594236	9594350
chr11_1694	p3	(GGA)7	21	9599639	9599659
chr11_1695	p3	(ATA)5	15	9600782	9600796
chr11_1696	p2	(GT)9	18	9602006	9602023
chr11_1697	p2	(TC)7	14	9602393	9602406
chr11_1698	p3	(GAG)5	15	9614299	9614313
chr11_1699	p2	(AC)10	20	9623369	9623388
chr11_1700	p2	(GA)7	14	9626201	9626214
chr11_1701	p3	(ATA)5	15	9626868	9626882
chr11_1702	p2	(AC)17	34	9629476	9629509
chr11_1703	p2	(AG)8	16	9632089	9632104
chr11_1704	p2	(CA)15	30	9633488	9633517
chr11_1705	p2	(GT)13	26	9633660	9633685
chr11_1706	p2	(CT)17	34	9633873	9633906
chr11_1707	p2	(TA)6	12	9637841	9637852
chr11_1708	p2	(CA)7	14	9657756	9657769
chr11_1709	p3	(AGT)5	15	9658478	9658492
chr11_1710	p2	(GT)11	22	9661733	9661754
chr11_1711	p2	(AC)9	18	9661891	9661908
chr11_1712	p2	(AC)6	12	9668357	9668368
chr11_1713	p2	(AC)11	22	9676484	9676505
chr11_1714	p2	(AG)13	26	9683751	9683776
chr11_1715	p3	(ATC)8	24	9693236	9693259
chr11_1716	p2	(TG)13	26	9703427	9703452
chr11_1717	p3	(AAT)6	18	9704684	9704701
chr11_1718	p2	(CA)10	20	9709664	9709683
chr11_1719	p2	(GT)8	16	9713934	9713949
chr11_1720	p2	(TG)8	16	9720449	9720464
chr11_1721	c	(CA)9(GA)13	44	9723982	9724025
chr11_1722	p2	(AC)18	36	9748043	9748078
chr11_1723	p2	(AC)21	42	9762455	9762496
chr11_1724	p2	(CT)6	12	9764578	9764589
chr11_1725	p3	(CAT)5	15	9776816	9776830
chr11_1726	p5	(TTGAG)10	50	9781803	9781852
chr11_1727	p2	(TG)15	30	9786756	9786785
chr11_1728	p2	(GT)6	12	9788026	9788037
chr11_1729	p2	(GT)22	44	9795513	9795556

chr11_1730	p3	(TTA)5	15	9797070	9797084
chr11_1731	p2	(AC)6	12	9803110	9803121
chr11_1732	p2	(TC)7	14	9821207	9821220
chr11_1733	p2	(CA)7	14	9835002	9835015
chr11_1734	p2	(AC)6	12	9843415	9843426
chr11_1735	p2	(GT)11	22	9846021	9846042
chr11_1736	p3	(GAA)5	15	9847652	9847666
chr11_1737	p3	(GGA)11	33	9847942	9847974
chr11_1738	p4	(TTTA)5	20	9851125	9851144
chr11_1739	p2	(AT)6	12	9857058	9857069
chr11_1740	p3	(CAG)6	18	9862862	9862879
chr11_1741	p2	(CA)11	22	9865044	9865065
chr11_1742	p2	(AC)6	12	9866479	9866490
chr11_1743	p2	(TC)14	28	9868047	9868074
chr11_1744	p2	(AG)6	12	9872073	9872084
chr11_1745	p2	(AC)22	44	9877036	9877079
chr11_1746	p2	(CA)6	12	9887710	9887721
chr11_1747	p2	(CA)6	12	9895335	9895346
chr11_1748	p2	(GT)19	38	9897192	9897229
chr11_1749	p2	(AC)6	12	9905720	9905731
chr11_1750	p3	(GAA)5	15	9908726	9908740
chr11_1751	p4	(TCAT)5	20	9911937	9911956
chr11_1752	p2	(CT)6	12	9916758	9916769
chr11_1753	p2	(CT)7	14	9923463	9923476
chr11_1754	p3	(AGT)6	18	9947583	9947600
chr11_1755	p2	(GT)7	14	9952914	9952927
chr11_1756	p2	(GT)7	14	9955317	9955330
chr11_1757	p2	(TG)15	30	9969495	9969524
chr11_1758	p2	(TA)6	12	9974712	9974723
chr11_1759	p3	(TGC)8	24	9976573	9976596
chr11_1760	p3	(GCT)6	18	9977161	9977178
chr11_1761	p3	(TGC)6	18	9978680	9978697
chr11_1762	p2	(CT)7	14	9994767	9994780
chr11_1763	p2	(TG)6	12	10029212	10029223
chr11_1764	p2	(TA)11	22	10032752	10032773
chr11_1765	p4	(TATC)7	28	10035860	10035887
chr11_1766	p3	(AGA)5	15	10046056	10046070
chr11_1767	p2	(TG)7	14	10059708	10059721
chr11_1768	p2	(AG)6	12	10061424	10061435
chr11_1769	p2	(TG)6	12	10066266	10066277
chr11_1770	p2	(TG)20	40	10068552	10068591
chr11_1771	p2	(GA)20	40	10073218	10073257
chr11_1772	p2	(CA)15	30	10080297	10080326
chr11_1773	p2	(TG)13	26	10082086	10082111
chr11_1774	p3	(CTG)5	15	10092848	10092862
chr11_1775	p3	(CCA)5	15	10103237	10103251
chr11_1776	p2	(CA)6	12	10104862	10104873
chr11_1777	p5	(TGAGT)9	45	10111339	10111383
chr11_1778	p3	(CTG)7	21	10120032	10120052
chr11_1779	c	(TG)27tc(TG)6	68	10145720	10145787
chr11_1780	p2	(GA)14	28	10147191	10147218
chr11_1781	p2	(GT)6	12	10148743	10148754

chr11_1782	p5	(TGTTT)5	25	10169212	10169236
chr11_1783	p3	(ATC)5	15	10171199	10171213
chr11_1784	p2	(CA)6	12	10174057	10174068
chr11_1785	p2	(AC)9	18	10181321	10181338
chr11_1786	p2	(TG)14	28	10183763	10183790
chr11_1787	p4	(AATG)6	24	10183906	10183929
chr11_1788	p2	(CA)9	18	10191089	10191106
chr11_1789	p2	(AC)8	16	10192552	10192567
chr11_1790	p3	(CAG)5	15	10195928	10195942
chr11_1791	p2	(AC)19	38	10202158	10202195
chr11_1792	p2	(GT)15	30	10202358	10202387
chr11_1793	p2	(GA)7	14	10210834	10210847
chr11_1794	p2	(CA)12	24	10212919	10212942
chr11_1795	p2	(AC)6	12	10219086	10219097
chr11_1796	p2	(GT)18	36	10221735	10221770
chr11_1797	p4	(GATG)5	20	10226566	10226585
chr11_1798	p2	(TG)8	16	10226939	10226954
chr11_1799	p2	(TG)8	16	10228988	10229003
chr11_1800	p2	(AC)9	18	10239822	10239839
chr11_1801	c	(GT)10gagt(GA)8	40	10245705	10245744
chr11_1802	p2	(CA)6	12	10251492	10251503
chr11_1803	p2	(GT)13	26	10255810	10255835
chr11_1804	p3	(GCT)6	18	10266025	10266042
chr11_1805	p3	(GCT)6	18	10267938	10267955
chr11_1806	p2	(TA)7	14	10276291	10276304
chr11_1807	p2	(TG)21	42	10277715	10277756
chr11_1808	p2	(GT)15	30	10286642	10286671
chr11_1809	p2	(AG)8	16	10286826	10286841
chr11_1810	p2	(GT)14	28	10287083	10287110
chr11_1811	p2	(TG)35	70	10304652	10304721
chr11_1812	p2	(TG)22	44	10305076	10305119
chr11_1813	p4	(TTTC)7	28	10311370	10311397
chr11_1814	c	(AC)11atagatatgtgtatata(TG)8	55	10316002	10316056
chr11_1815	p3	(CAG)5	15	10316907	10316921
chr11_1816	p3	(TAA)6	18	10319209	10319226
chr11_1817	p2	(CT)6	12	10319440	10319451
chr11_1818	p2	(TG)31	62	10322653	10322714
chr11_1819	p2	(TG)12	24	10360485	10360508
chr11_1820	p2	(TG)7	14	10367859	10367872
chr11_1821	p3	(AGG)5	15	10370945	10370959
chr11_1822	p2	(AT)12	24	10381679	10381702
chr11_1823	p2	(AC)11	22	10383535	10383556
chr11_1824	c	(TC)8(AC)24	64	10408195	10408258
chr11_1825	p2	(TG)9	18	10418666	10418683
chr11_1826	p2	(GT)7	14	10435320	10435333
chr11_1827	p2	(AG)6	12	10435735	10435746
chr11_1828	p3	(TTG)8	24	10450256	10450279
chr11_1829	p2	(GT)7	14	10453870	10453883
chr11_1830	p2	(CA)9	18	10472168	10472185
chr11_1831	p3	(ATT)6	18	10484005	10484022
chr11_1832	p2	(AC)9	18	10489139	10489156
chr11_1833	p2	(TG)8	16	10492323	10492338

chr11_1834	c	(AG)6cgagagggagg(GA)11	45	10505149	10505193
chr11_1835	p2	(TG)6	12	10514115	10514126
chr11_1836	p2	(TG)8	16	10518424	10518439
chr11_1837	p2	(AG)9	18	10519521	10519538
chr11_1838	p2	(CA)6	12	10522968	10522979
chr11_1839	p2	(TA)11	22	10527645	10527666
chr11_1840	p2	(GA)6	12	10529965	10529976
chr11_1841	p2	(TG)16	32	10534749	10534780
chr11_1842	p2	(CT)11	22	10536115	10536136
chr11_1843	p2	(CT)6	12	10539028	10539039
chr11_1844	p2	(TG)14	28	10551334	10551361
chr11_1845	p2	(TG)8	16	10555138	10555153
chr11_1846	p2	(TG)6	12	10570082	10570093
chr11_1847	p2	(TG)7	14	10572150	10572163
chr11_1848	p2	(TC)6	12	10586869	10586880
chr11_1849	c	actgccatacatagtcactcttactatgaacatgcatgcatttacagaatgca	144	10587922	10588065
chr11_1850	p2	(CA)7	14	10589467	10589480
chr11_1851	p2	(TG)12	24	10599380	10599403
chr11_1852	p2	(CA)9	18	10602273	10602290
chr11_1853	p2	(CT)6	12	10602815	10602826
chr11_1854	p6	(TTCCTA)19	114	10613917	10614030
chr11_1855	p3	(AAT)5	15	10621787	10621801
chr11_1856	p2	(CA)8	16	10626056	10626071
chr11_1857	p2	(GA)8	16	10626763	10626778
chr11_1858	p2	(AC)6	12	10627511	10627522
chr11_1859	c	(CCT)5ctctctcctgctttggctatata(CCT)5	53	10648119	10648171
chr11_1860	p2	(TG)6	12	10648776	10648787
chr11_1861	p2	(AC)6	12	10653180	10653191
chr11_1862	p2	(TG)7	14	10653300	10653313
chr11_1863	p2	(TG)8	16	10654613	10654628
chr11_1864	c	agtgagtttgggagggagcgctagggggtggatgaaactggtgcaggag	103	10654983	10655085
chr11_1865	p2	(GT)8	16	10668673	10668688
chr11_1866	p3	(AAT)6	18	10671049	10671066
chr11_1867	p2	(CA)12	24	10673533	10673556
chr11_1868	p3	(AGG)7	21	10675456	10675476
chr11_1869	p2	(CA)6	12	10685569	10685580
chr11_1870	p2	(CA)6	12	10688938	10688949
chr11_1871	p2	(TG)23	46	10734408	10734453
chr11_1872	p2	(TG)33	66	10741731	10741796
chr11_1873	p3	(GCA)7	21	10744726	10744746
chr11_1874	c	(TTC)5tg(CTT)5	32	10747996	10748027
chr11_1875	p2	(TA)15	30	10748869	10748898
chr11_1876	p2	(GT)6	12	10758986	10758997
chr11_1877	p2	(GT)9	18	10775999	10776016
chr11_1878	p2	(AC)15	30	10782083	10782112
chr11_1879	p2	(TG)11	22	10783804	10783825
chr11_1880	p2	(TG)9	18	10800350	10800367
chr11_1881	p2	(AC)9	18	10803542	10803559
chr11_1882	c	(AT)9acacacat(AC)6	38	10806238	10806275
chr11_1883	p2	(CA)7	14	10816851	10816864
chr11_1884	p2	(CA)10	20	10825379	10825398
chr11_1885	p2	(TG)12	24	10830238	10830261

chr11_1886	p2	(AC)9	18	10831245	10831262
chr11_1887	p2	(AC)7	14	10831923	10831936
chr11_1888	p3	(TTC)6	18	10835012	10835029
chr11_1889	p2	(TC)8	16	10850417	10850432
chr11_1890	p3	(TTG)6	18	10853466	10853483
chr11_1891	p2	(GT)7	14	10867116	10867129
chr11_1892	p3	(TAT)5	15	10872410	10872424
chr11_1893	p2	(AC)7	14	10873520	10873533
chr11_1894	p2	(TG)11	22	10875707	10875728
chr11_1895	p2	(AC)6	12	10876625	10876636
chr11_1896	p2	(AT)9	18	10885322	10885339
chr11_1897	p4	(AATG)5	20	10902655	10902674
chr11_1898	p2	(GA)8	16	10912100	10912115
chr11_1899	p2	(TG)14	28	10932417	10932444
chr11_1900	p2	(AT)7	14	10933932	10933945
chr11_1901	p2	(AC)7	14	10935882	10935895
chr11_1902	p2	(AC)11	22	10938509	10938530
chr11_1903	p3	(TTA)5	15	10955489	10955503
chr11_1904	p3	(ATG)14	42	10969565	10969606
chr11_1905	p2	(AC)10	20	10970384	10970403
chr11_1906	p3	(AAT)5	15	10974920	10974934
chr11_1907	p2	(AT)7	14	10977623	10977636
chr11_1908	p2	(CA)17	34	10980849	10980882
chr11_1909	p4	(TTTG)6	24	10983717	10983740
chr11_1910	p2	(GT)6	12	10987178	10987189
chr11_1911	p2	(AG)7	14	10993317	10993330
chr11_1912	p2	(CA)7	14	10996599	10996612
chr11_1913	p2	(CA)24	48	11012542	11012589
chr11_1914	p3	(GAG)5	15	11015001	11015015
chr11_1915	p2	(GT)14	28	11015252	11015279
chr11_1916	p2	(CA)11	22	11018960	11018981
chr11_1917	p2	(TG)9	18	11029203	11029220
chr11_1918	c	[illegible]T)6tttttttgtttatgtatggagtgtatatatctatctatctatagatag(AT[illegible]	79	11029572	11029650
chr11_1919	p2	(CA)12	24	11034209	11034232
chr11_1920	p2	(TC)7	14	11038718	11038731
chr11_1921	p3	(CCA)6	18	11040389	11040406
chr11_1922	p3	(TTG)7	21	11046637	11046657
chr11_1923	p2	(CA)6	12	11057816	11057827
chr11_1924	p2	(TC)6	12	11061152	11061163
chr11_1925	p2	(GT)9	18	11072529	11072546
chr11_1926	p2	(CA)8	16	11072790	11072805
chr11_1927	p2	(CA)13	26	11074232	11074257
chr11_1928	p2	(GT)23	46	11079455	11079500
chr11_1929	c	(GT)7a(TG)18	51	11081692	11081742
chr11_1930	p2	(GT)7	14	11081895	11081908
chr11_1931	p4	(TTTG)5	20	11085524	11085543
chr11_1932	c	(CA)6(GA)6	24	11088116	11088139
chr11_1933	p2	(TA)8	16	11089775	11089790
chr11_1934	p2	(TG)6	12	11090733	11090744
chr11_1935	p4	(AAAC)5	20	11091111	11091130
chr11_1936	p2	(AC)18	36	11097168	11097203
chr11_1937	p2	(GT)9	18	11098378	11098395

chr11_1938	p3	(TCG)5	15	11100211	11100225
chr11_1939	p4	(TCCA)6	24	11100825	11100848
chr11_1940	p2	(TC)9	18	11106538	11106555
chr11_1941	p2	(GT)6	12	11121162	11121173
chr11_1942	p2	(AC)9	18	11136047	11136064
chr11_1943	p2	(CT)7	14	11136433	11136446
chr11_1944	p2	(CA)6	12	11149785	11149796
chr11_1945	p2	(AC)12	24	11150350	11150373
chr11_1946	p2	(GT)19	38	11150785	11150822
chr11_1947	p2	(TG)14	28	11160165	11160192
chr11_1948	p2	(CA)8	16	11160319	11160334
chr11_1949	p2	(AG)6	12	11160731	11160742
chr11_1950	p2	(TA)6	12	11162905	11162916
chr11_1951	p2	(AC)16	32	11163208	11163239
chr11_1952	p2	(AC)20	40	11168879	11168918
chr11_1953	p3	(GAG)5	15	11169483	11169497
chr11_1954	p2	(GT)16	32	11178463	11178494
chr11_1955	p2	(TG)19	38	11179601	11179638
chr11_1956	p2	(TC)6	12	11183573	11183584
chr11_1957	p2	(CA)19	38	11189360	11189397
chr11_1958	p2	(CA)12	24	11189529	11189552
chr11_1959	p2	(TA)8	16	11196668	11196683
chr11_1960	p2	(TG)6	12	11200920	11200931
chr11_1961	p2	(CA)11	22	11227827	11227848
chr11_1962	c	(AC)11(TC)8	38	11243241	11243278
chr11_1963	p3	(TAA)10	30	11252247	11252276
chr11_1964	p2	(AT)8	16	11254957	11254972
chr11_1965	p3	(ATT)5	15	11255982	11255996
chr11_1966	p4	(AAAG)5	20	11257385	11257404
chr11_1967	p2	(AG)10	20	11265472	11265491
chr11_1968	p2	(TG)14	28	11267841	11267868
chr11_1969	p2	(AC)7	14	11269072	11269085
chr11_1970	p3	(ACA)6	18	11275064	11275081
chr11_1971	p2	(AC)9	18	11293035	11293052
chr11_1972	p2	(TC)6	12	11314747	11314758
chr11_1973	p2	(TG)7	14	11316867	11316880
chr11_1974	p2	(AC)24	48	11318099	11318146
chr11_1975	p4	(TCCA)5	20	11322783	11322802
chr11_1976	p2	(CA)7	14	11328949	11328962
chr11_1977	p2	(AC)6	12	11329085	11329096
chr11_1978	c	(GT)6ctgtgcagagaaagagagagtgaggggga(AG)6	52	11335115	11335166
chr11_1979	p2	(TG)11	22	11341825	11341846
chr11_1980	p3	(ATT)9	27	11342574	11342600
chr11_1981	p2	(CT)6	12	11353682	11353693
chr11_1982	p2	(AG)6	12	11356292	11356303
chr11_1983	p2	(AC)6	12	11359011	11359022
chr11_1984	p2	(AC)7	14	11360033	11360046
chr11_1985	c	(CA)6t(AC)6	25	11360272	11360296
chr11_1986	p2	(AC)7	14	11361180	11361193
chr11_1987	p2	(AT)6	12	11375199	11375210
chr11_1988	p2	(TG)6	12	11380541	11380552
chr11_1989	p2	(TC)6	12	11382755	11382766

chr11_1990	p2	(AC)7	14	11398422	11398435
chr11_1991	p2	(AC)14	28	11404272	11404299
chr11_1992	p2	(CA)15	30	11405934	11405963
chr11_1993	p2	(TG)7	14	11406268	11406281
chr11_1994	c	AT)5cccacacactcggtttattttctcaggtggacaaactgtatttg(CA)	83	11407930	11408012
chr11_1995	p3	(ATC)5	15	11412828	11412842
chr11_1996	p2	(TG)6	12	11419772	11419783
chr11_1997	p2	(AT)7	14	11420610	11420623
chr11_1998	c	'G)10(TA)9(TG)10(TA)9(TG)10(TA)9(TG)10(TA)9	550	11430935	11431484
chr11_1999	p3	(CTA)5	15	11439514	11439528
chr11_2000	p2	(AG)7	14	11444106	11444119
chr11_2001	p2	(GA)6	12	11450730	11450741
chr11_2002	p2	(TG)9	18	11456775	11456792
chr11_2003	p2	(TG)9	18	11461508	11461525
chr11_2004	p3	(TTC)5	15	11477793	11477807
chr11_2005	c	ttcttcgtcgtcctttgccatttaagggcggtttgcatgccacgccaccgcct	129	11478013	11478141
chr11_2006	p2	(CT)16	32	11485315	11485346
chr11_2007	p2	(TA)7	14	11501979	11501992
chr11_2008	p2	(GT)6	12	11509781	11509792
chr11_2009	p2	(AC)16	32	11513774	11513805
chr11_2010	p2	(CA)6	12	11519113	11519124
chr11_2011	p2	(CA)6	12	11520517	11520528
chr11_2012	p3	(TAA)6	18	11521785	11521802
chr11_2013	p2	(AG)15	30	11522867	11522896
chr11_2014	p2	(AG)7	14	11547614	11547627
chr11_2015	c	ıtgcctgcacaaacatacagccaaggacatccacacttctgtcaactaacat	121	11575569	11575689
chr11_2016	p2	(AC)6	12	11589602	11589613
chr11_2017	c	AC)11aaccaacttcagtgcccagcagtcctgtgagtctcccccaccac(	105	11591800	11591904
chr11_2018	p2	(TG)22	44	11593102	11593145
chr11_2019	p3	(AAT)5	15	11605874	11605888
chr11_2020	c	(TG)9(AG)9	36	11606293	11606328
chr11_2021	c	(AT)7(AC)10	34	11616769	11616802
chr11_2022	p3	(AGC)6	18	11621695	11621712
chr11_2023	p4	(ATGA)5	20	11622967	11622986
chr11_2024	p2	(TA)8	16	11623609	11623624
chr11_2025	p2	(CA)8	16	11628532	11628547
chr11_2026	p2	(TA)7	14	11652817	11652830
chr11_2027	p3	(TTA)7	21	11656527	11656547
chr11_2028	p3	(CAA)6	18	11658720	11658737
chr11_2029	c	(GT)8(AT)7	30	11664928	11664957
chr11_2030	p2	(CA)14	28	11673208	11673235
chr11_2031	p3	(AAT)6	18	11686379	11686396
chr11_2032	p2	(GT)6	12	11691064	11691075
chr11_2033	p2	(TG)7	14	11691224	11691237
chr11_2034	p5	(CTCTC)5	25	11693018	11693042
chr11_2035	p2	(CT)6	12	11714107	11714118
chr11_2036	p2	(TG)13	26	11718988	11719013
chr11_2037	p3	(CAA)5	15	11725685	11725699
chr11_2038	p2	(TG)15	30	11732277	11732306
chr11_2039	p4	(TATC)10	40	11750710	11750749
chr11_2040	p3	(AAG)5	15	11752143	11752157
chr11_2041	p3	(AGA)5	15	11757926	11757940

chr11_2042	p2	(TG)7	14	11761541	11761554
chr11_2043	p2	(TG)23	46	11776985	11777030
chr11_2044	p3	(TTG)5	15	11788286	11788300
chr11_2045	p2	(AC)6	12	11789570	11789581
chr11_2046	p2	(TG)24	48	11830905	11830952
chr11_2047	p2	(AC)6	12	11831339	11831350
chr11_2048	p5	(TGTTT)5	25	11862550	11862574
chr11_2049	p2	(TG)15	30	11867622	11867651
chr11_2050	p2	(AT)6	12	11872309	11872320
chr11_2051	p2	(CA)6	12	11878865	11878876
chr11_2052	p2	(AT)6	12	11894252	11894263
chr11_2053	p2	(GT)6	12	11894901	11894912
chr11_2054	p2	(CA)6	12	11895227	11895238
chr11_2055	p2	(AC)31	62	11895592	11895653
chr11_2056	p2	(GT)6	12	11899354	11899365
chr11_2057	p2	(CA)23	46	11902358	11902403
chr11_2058	p2	(CT)7	14	11904957	11904970
chr11_2059	p2	(TG)14	28	11947926	11947953
chr11_2060	p2	(TG)14	28	11950761	11950788
chr11_2061	p4	(ATGG)5	20	11952798	11952817
chr11_2062	p3	(AAT)6	18	11964134	11964151
chr11_2063	p2	(GT)7	14	11967954	11967967
chr11_2064	p3	(AAC)5	15	11968121	11968135
chr11_2065	p2	(TA)6	12	11968866	11968877
chr11_2066	c	(TCC)5acttccttgtgttcttcttcattgtg(CTC)6	59	11970349	11970407
chr11_2067	p2	(AC)12	24	11975911	11975934
chr11_2068	c	tcaaaatatgtgccatgaaagagctatttagctcataaaaatgttaaatgtaa	120	11978195	11978314
chr11_2069	p4	(TTGA)6	24	11984553	11984576
chr11_2070	c	gtagtttttcagtgacagctgtgttatctggtttcgaggacacacaaccaaga	107	11997947	11998053
chr11_2071	p2	(TG)16	32	12001635	12001666
chr11_2072	p2	(CA)6	12	12005455	12005466
chr11_2073	p2	(CA)9	18	12016093	12016110
chr11_2074	c	T)6ctgtgtgcatggacactgtgatattcacccgaaacgctaag(CTTT	77	12017170	12017246
chr11_2075	p2	(AT)7	14	12023887	12023900
chr11_2076	p5	(TTTTA)5	25	12051038	12051062
chr11_2077	p4	(GATA)5	20	12051688	12051707
chr11_2078	p2	(AC)9	18	12052963	12052980
chr11_2079	p2	(GT)17	34	12065460	12065493
chr11_2080	p2	(TC)6	12	12067782	12067793
chr11_2081	p4	(CATC)5	20	12069652	12069671
chr11_2082	p2	(TG)7	14	12072569	12072582
chr11_2083	p2	(CT)6	12	12084912	12084923
chr11_2084	p2	(TG)23	46	12085444	12085489
chr11_2085	p2	(AT)6	12	12086024	12086035
chr11_2086	p2	(GA)6	12	12089159	12089170
chr11_2087	p2	(AG)7	14	12090467	12090480
chr11_2088	p2	(GT)6	12	12092387	12092398
chr11_2089	p2	(CA)10	20	12096221	12096240
chr11_2090	p2	(TG)7	14	12097856	12097869
chr11_2091	p2	(TG)15	30	12107080	12107109
chr11_2092	p4	(TTTA)5	20	12109213	12109232
chr11_2093	p2	(TG)15	30	12110444	12110473

chr11_2094	p2	(TG)9	18	12125627	12125644
chr11_2095	p2	(CA)15	30	12127155	12127184
chr11_2096	c	(GT)15gc(GT)17	66	12127413	12127478
chr11_2097	p2	(AC)19	38	12128516	12128553
chr11_2098	p3	(TCC)8	24	12136017	12136040
chr11_2099	p2	(TC)7	14	12140848	12140861
chr11_2100	p2	(TG)6	12	12144387	12144398
chr11_2101	p3	(ATA)5	15	12144504	12144518
chr11_2102	p2	(AG)6	12	12146133	12146144
chr11_2103	p2	(GT)11	22	12149252	12149273
chr11_2104	p4	(TCTG)5	20	12155641	12155660
chr11_2105	p2	(AC)6	12	12160359	12160370
chr11_2106	p2	(CT)9	18	12160926	12160943
chr11_2107	p2	(CT)9	18	12177872	12177889
chr11_2108	p2	(TG)10	20	12197886	12197905
chr11_2109	p2	(TG)9	18	12235351	12235368
chr11_2110	p2	(TA)6	12	12236434	12236445
chr11_2111	p2	(AT)14	28	12255101	12255128
chr11_2112	p2	(AG)6	12	12265416	12265427
chr11_2113	p3	(GCT)5	15	12279271	12279285
chr11_2114	p3	(TCT)5	15	12296215	12296229
chr11_2115	p2	(AT)6	12	12301425	12301436
chr11_2116	p2	(TG)12	24	12339992	12340015
chr11_2117	p2	(TG)10	20	12368029	12368048
chr11_2118	p3	(AAT)6	18	12385417	12385434
chr11_2119	p4	(TATT)6	24	12387968	12387991
chr11_2120	p2	(AT)6	12	12400514	12400525
chr11_2121	c	(TG)7atggggggaagcgtcctgtgaactcatcactatct(CA)12	74	12418192	12418265
chr11_2122	p2	(TG)11	22	12419666	12419687
chr11_2123	p2	(AC)8	16	12424977	12424992
chr11_2124	p2	(TG)7	14	12425406	12425419
chr11_2125	p2	(GA)6	12	12432119	12432130
chr11_2126	p3	(AAT)7	21	12432630	12432650
chr11_2127	p2	(GT)11	22	12450005	12450026
chr11_2128	p2	(AC)7	14	12454766	12454779
chr11_2129	p2	(AC)11	22	12457791	12457812
chr11_2130	p2	(AG)6	12	12462789	12462800
chr11_2131	p4	(GTTA)6	24	12469850	12469873
chr11_2132	p2	(TG)15	30	12471935	12471964
chr11_2133	p4	(TGTT)6	24	12476434	12476457
chr11_2134	p3	(GAG)5	15	12477578	12477592
chr11_2135	p2	(TG)9	18	12486987	12487004
chr11_2136	p2	(CA)8	16	12488294	12488309
chr11_2137	p2	(TG)14	28	12490078	12490105
chr11_2138	p2	(TC)6	12	12491713	12491724
chr11_2139	p2	(AC)6	12	12494612	12494623
chr11_2140	c	(TG)9tc(TG)23	66	12498289	12498354
chr11_2141	p2	(TG)6	12	12513885	12513896
chr11_2142	p4	(AAAC)5	20	12537262	12537281
chr11_2143	p2	(GA)7	14	12538104	12538117
chr11_2144	p5	(AAAGA)5	25	12543554	12543578
chr11_2145	p2	(TG)11	22	12544314	12544335

chr11_2146	p2	(TG)11	22	12544494	12544515
chr11_2147	p2	(TC)9	18	12564822	12564839
chr11_2148	p6	(TTATAT)5	30	12571882	12571911
chr11_2149	p2	(AC)9	18	12579376	12579393
chr11_2150	p3	(ATA)5	15	12585868	12585882
chr11_2151	p2	(GT)16	32	12630415	12630446
chr11_2152	p2	(TC)8	16	12643128	12643143
chr11_2153	p3	(TAA)5	15	12653929	12653943
chr11_2154	p2	(GT)17	34	12658474	12658507
chr11_2155	p2	(CA)9	18	12661407	12661424
chr11_2156	p3	(ACA)6	18	12679833	12679850
chr11_2157	p3	(TGG)6	18	12682382	12682399
chr11_2158	c	(CA)9t(AC)7	33	12685111	12685143
chr11_2159	p2	(GT)9	18	12701618	12701635
chr11_2160	p2	(CA)8	16	12702355	12702370
chr11_2161	p3	(ATC)5	15	12703235	12703249
chr11_2162	p3	(TCT)7	21	12703921	12703941
chr11_2163	p4	(ATCT)26	104	12709845	12709948
chr11_2164	p4	(CTAT)7	28	12710155	12710182
chr11_2165	p2	(TC)8	16	12712765	12712780
chr11_2166	p3	(CTC)6	18	12715569	12715586
chr11_2167	p4	(TCCA)6	24	12728200	12728223
chr11_2168	p2	(GT)19	38	12733229	12733266
chr11_2169	p2	(AC)7	14	12739338	12739351
chr11_2170	p2	(TC)6	12	12741617	12741628
chr11_2171	c	(GT)6a(TG)7	27	12743153	12743179
chr11_2172	p2	(CA)11	22	12753060	12753081
chr11_2173	p2	(CA)50	100	12764000	12764099
chr11_2174	c	tacatgctaaattaagcactgattttccttggcagttaaaattcaggtcactag	131	12769951	12770081
chr11_2175	p4	(TCTA)24	96	12770951	12771046
chr11_2176	c	(TG)21ta(TG)6	56	12782981	12783036
chr11_2177	p2	(GT)34	68	12786995	12787062
chr11_2178	c	(GGA)8tgaggaggaagaggaggaa(GAG)5	58	12796303	12796360
chr11_2179	p2	(CA)12	24	12796649	12796672
chr11_2180	p3	(ATT)7	21	12797157	12797177
chr11_2181	c	(AC)9(AG)6aagagagaa(AG)7	53	12797468	12797520
chr11_2182	c	(TC)6tttctctctctctt(TC)23	72	12798669	12798740
chr11_2183	p3	(TTA)5	15	12799463	12799477
chr11_2184	p2	(TG)8	16	12804715	12804730
chr11_2185	p2	(TG)18	36	12805640	12805675
chr11_2186	p2	(CA)6	12	12806007	12806018
chr11_2187	p2	(TG)12	24	12806924	12806947
chr11_2188	p2	(GA)7	14	12809672	12809685
chr11_2189	p2	(GT)16	32	12813582	12813613
chr11_2190	p3	(AGC)5	15	12813759	12813773
chr11_2191	p2	(TG)6	12	12816095	12816106
chr11_2192	p2	(AC)16	32	12817750	12817781
chr11_2193	p2	(GA)12	24	12819682	12819705
chr11_2194	p2	(AC)14	28	12820196	12820223
chr11_2195	p2	(GT)6	12	12820396	12820407
chr11_2196	p2	(TG)9	18	12821552	12821569
chr11_2197	p2	(GA)9	18	12824147	12824164

chr11_2198	p2	(TG)7	14	12825200	12825213
chr11_2199	c	(ATT)5(AGT)5	30	12825343	12825372
chr11_2200	p2	(AC)7	14	12848222	12848235
chr11_2201	p2	(CA)6	12	12869591	12869602
chr11_2202	p2	(CA)22	44	12874693	12874736
chr11_2203	p2	(AC)9	18	12880424	12880441
chr11_2204	p3	(CAG)6	18	12883232	12883249
chr11_2205	p2	(TA)8	16	12892266	12892281
chr11_2206	p3	(TGC)6	18	12901613	12901630
chr11_2207	p2	(TC)9	18	12904853	12904870
chr11_2208	p2	(CA)6	12	12905472	12905483
chr11_2209	p2	(CA)8	16	12905821	12905836
chr11_2210	p3	(GCT)5	15	12909361	12909375
chr11_2211	p3	(TCC)5	15	12912558	12912572
chr11_2212	p2	(TG)10	20	12912745	12912764
chr11_2213	p2	(TA)10	20	12913746	12913765
chr11_2214	c	gtgctgttgtgtatctggcaaaaaggaagagcaactctgtaaaaaaaacaa	169	12919197	12919365
chr11_2215	p3	(TCC)5	15	12921843	12921857
chr11_2216	c	(CA)12cc(CA)15	56	12925063	12925118
chr11_2217	p2	(CA)6	12	12926291	12926302
chr11_2218	p2	(AC)7	14	12926989	12927002
chr11_2219	p4	(CATC)5	20	12929798	12929817
chr11_2220	c	T)7g(CA)8ctcatctagtgcagggcaccctgcactaga(TG)8tatgt	139	12930723	12930861
chr11_2221	p2	(CA)18	36	12940190	12940225
chr11_2222	p3	(CCT)6	18	12954047	12954064
chr11_2223	p2	(TC)7	14	12957773	12957786
chr11_2224	p3	(ATT)5	15	12961232	12961246
chr11_2225	c	(GT)7(TG)7	28	12973819	12973846
chr11_2226	p3	(AGC)5	15	12982519	12982533
chr11_2227	p2	(TG)7	14	12985752	12985765
chr11_2228	p2	(TG)9	18	12986967	12986984
chr11_2229	p2	(TG)9	18	12988019	12988036
chr11_2230	p2	(GT)8	16	12988176	12988191
chr11_2231	p2	(AC)9	18	12988303	12988320
chr11_2232	c	(CA)9gagtg(TC)6	35	12988561	12988595
chr11_2233	p4	(TTTC)6	24	12990749	12990772
chr11_2234	p2	(CA)10	20	12992472	12992491
chr11_2235	p2	(AC)10	20	12994455	12994474
chr11_2236	p2	(AC)7	14	13001257	13001270
chr11_2237	p3	(AGG)6	18	13014169	13014186
chr11_2238	p2	(AG)7	14	13014374	13014387
chr11_2239	p3	(TTA)12	36	13018142	13018177
chr11_2240	p2	(CT)7	14	13022304	13022317
chr11_2241	p2	(AT)9	18	13025352	13025369
chr11_2242	c	(CG)9(CA)27	72	13037915	13037986
chr11_2243	p3	(TAA)7	21	13042786	13042806
chr11_2244	p2	(CA)16	32	13052971	13053002
chr11_2245	p2	(CA)27	54	13067218	13067271
chr11_2246	p2	(TG)7	14	13073193	13073206
chr11_2247	p2	(TG)11	22	13080157	13080178
chr11_2248	p2	(GA)6	12	13081471	13081482
chr11_2249	p2	(CA)7	14	13081998	13082011

chr11_2250	p2	(TG)7	14	13083379	13083392
chr11_2251	p3	(CTC)7	21	13086726	13086746
chr11_2252	p2	(CA)9	18	13086961	13086978
chr11_2253	p2	(TG)22	44	13090170	13090213
chr11_2254	c	(AGAT)5agacagacagc(CAGA)6	55	13092413	13092467
chr11_2255	p2	(CA)7	14	13094007	13094020
chr11_2256	p2	(GT)6	12	13094136	13094147
chr11_2257	p2	(AG)7	14	13096446	13096459
chr11_2258	p2	(TG)15	30	13097127	13097156
chr11_2259	p2	(AC)15	30	13097868	13097897
chr11_2260	p2	(AC)17	34	13105370	13105403
chr11_2261	p2	(CT)6	12	13105686	13105697
chr11_2262	p4	(AATC)5	20	13106385	13106404
chr11_2263	p2	(AC)6	12	13114802	13114813
chr11_2264	p2	(TG)19	38	13115079	13115116
chr11_2265	p2	(CA)6	12	13115387	13115398
chr11_2266	p2	(GT)7	14	13116650	13116663
chr11_2267	p2	(AC)12	24	13116902	13116925
chr11_2268	p2	(TG)11	22	13119480	13119501
chr11_2269	p2	(TG)9	18	13121785	13121802
chr11_2270	p2	(AC)6	12	13122914	13122925
chr11_2271	p2	(GA)12	24	13125250	13125273
chr11_2272	c	ΓAC)6taagaataataataataggtcctgtatgctttattgacattt(GAA)	78	13130616	13130693
chr11_2273	p2	(GT)28	56	13132474	13132529
chr11_2274	p2	(AT)7	14	13133096	13133109
chr11_2275	p2	(GT)15	30	13134314	13134343
chr11_2276	p3	(GAA)5	15	13135547	13135561
chr11_2277	p2	(TG)17	34	13158947	13158980
chr11_2278	p2	(TA)7	14	13195519	13195532
chr11_2279	p3	(CTC)6	18	13199155	13199172
chr11_2280	p3	(TAA)6	18	13203436	13203453
chr11_2281	p2	(AC)7	14	13219832	13219845
chr11_2282	c	ınnnnnnnnnnnnnnnnnnnnnnnnnnnnnnnnnnnnnnnnnnnr	215	13220915	13221129
chr11_2283	p2	(CA)11	22	13222187	13222208
chr11_2284	p2	(CA)7	14	13223303	13223316
chr11_2285	p2	(TG)7	14	13228490	13228503
chr11_2286	p2	(CA)12	24	13229640	13229663
chr11_2287	p2	(AC)9	18	13229791	13229808
chr11_2288	p2	(CA)11	22	13240533	13240554
chr11_2289	p2	(AC)14	28	13243875	13243902
chr11_2290	p2	(AT)7	14	13257850	13257863
chr11_2291	p4	(TGTT)5	20	13284016	13284035
chr11_2292	p2	(TC)9	18	13289551	13289568
chr11_2293	p4	(GTAA)8	32	13293350	13293381
chr11_2294	p2	(AC)17	34	13296392	13296425
chr11_2295	p3	(TAG)5	15	13301210	13301224
chr11_2296	p4	(TGTT)7	28	13305838	13305865
chr11_2297	p2	(AC)6	12	13310987	13310998
chr11_2298	p2	(AC)7	14	13315388	13315401
chr11_2299	p2	(CA)9	18	13322505	13322522
chr11_2300	p2	(AT)10	20	13323573	13323592
chr11_2301	p3	(AAC)8	24	13326299	13326322

chr11_2302	c	(TG)8ta(TG)6	30	13339111	13339140
chr11_2303	p2	(GT)13	26	13341977	13342002
chr11_2304	p2	(AC)6	12	13344110	13344121
chr11_2305	p2	(AC)8	16	13356188	13356203
chr11_2306	p4	(TTTG)6	24	13359879	13359902
chr11_2307	p2	(CA)8	16	13363577	13363592
chr11_2308	p2	(TG)7	14	13375366	13375379
chr11_2309	c	:aggaaggagcaggtgctgcctctggtccagccgcagtaaggatccctag	189	13383018	13383206
chr11_2310	p2	(GA)10	20	13385201	13385220
chr11_2311	p2	(TG)11	22	13389801	13389822
chr11_2312	p4	(ATGG)6	24	13397264	13397287
chr11_2313	p3	(AAT)7	21	13398218	13398238
chr11_2314	p2	(CA)6	12	13402114	13402125
chr11_2315	p2	(AC)14	28	13402765	13402792
chr11_2316	p2	(AG)6	12	13404974	13404985
chr11_2317	p2	(CA)6	12	13405640	13405651
chr11_2318	p2	(CA)7	14	13406331	13406344
chr11_2319	c	(TC)10at(AC)8	38	13411048	13411085
chr11_2320	p2	(CA)6	12	13411840	13411851
chr11_2321	p2	(TG)6	12	13420246	13420257
chr11_2322	p3	(CCT)5	15	13420436	13420450
chr11_2323	p3	(AAT)6	18	13427092	13427109
chr11_2324	p2	(CA)6	12	13427647	13427658
chr11_2325	c	aaaaactgaatagcttgttgaccgacagtaatgcagctttaaccaacactgt	82	13439040	13439121
chr11_2326	p2	(AC)9	18	13440088	13440105
chr11_2327	p3	(TAA)6	18	13447542	13447559
chr11_2328	p4	(CAAA)9	36	13449478	13449513
chr11_2329	p2	(TA)6	12	13450939	13450950
chr11_2330	c	(CA)6cg(CA)7	28	13451857	13451884
chr11_2331	p2	(CA)28	56	13453415	13453470
chr11_2332	c	(CA)10cg(CA)6	34	13464379	13464412
chr11_2333	p2	(CA)13	26	13465461	13465486
chr11_2334	p2	(CT)7	14	13475325	13475338
chr11_2335	p2	(GT)10	20	13476240	13476259
chr11_2336	p2	(AG)8	16	13477069	13477084
chr11_2337	p2	(CA)25	50	13477582	13477631
chr11_2338	c	(AC)6t(CA)8	29	13477762	13477790
chr11_2339	p2	(CA)14	28	13478655	13478682
chr11_2340	p2	(AG)7	14	13478877	13478890
chr11_2341	p2	(TG)7	14	13480887	13480900
chr11_2342	p2	(TC)7	14	13489931	13489944
chr11_2343	p2	(TC)18	36	13518462	13518497
chr11_2344	p2	(TG)6	12	13518706	13518717
chr11_2345	c	ι)6ctggggttgtgctgtcagctctttggacaaaggctaattaagaaattt(T	72	13525566	13525637
chr11_2346	p2	(TG)10	20	13528257	13528276
chr11_2347	p2	(TC)13	26	13536808	13536833
chr11_2348	p2	(GT)7	14	13537724	13537737
chr11_2349	p2	(GT)20	40	13538207	13538246
chr11_2350	p2	(AC)8	16	13545489	13545504
chr11_2351	p4	(AAAC)5	20	13554823	13554842
chr11_2352	p2	(TG)14	28	13556049	13556076
chr11_2353	p2	(AC)27	54	13556244	13556297

chr11_2354	c	(AC)6atgctgggcaaacagagact(CA)6	44	13559651	13559694
chr11_2355	p2	(GT)11	22	13561487	13561508
chr11_2356	p2	(AC)6	12	13563861	13563872
chr11_2357	p2	(AC)9	18	13568029	13568046
chr11_2358	p3	(GGA)5	15	13568281	13568295
chr11_2359	p2	(GT)6	12	13569115	13569126
chr11_2360	p2	(AC)13	26	13569278	13569303
chr11_2361	p2	(AG)10	20	13572522	13572541
chr11_2362	p2	(TG)6	12	13572753	13572764
chr11_2363	p2	(CA)8	16	13574579	13574594
chr11_2364	p4	(AGAA)7	28	13575796	13575823
chr11_2365	p2	(AG)6	12	13576179	13576190
chr11_2366	p3	(CAG)7	21	13577013	13577033
chr11_2367	p2	(AC)8	16	13578474	13578489
chr11_2368	p2	(TG)20	40	13585688	13585727
chr11_2369	p2	(GT)6	12	13587042	13587053
chr11_2370	p2	(GT)6	12	13590632	13590643
chr11_2371	p2	(GA)8	16	13590838	13590853
chr11_2372	p2	(CA)7	14	13593386	13593399
chr11_2373	p2	(AC)6	12	13593931	13593942
chr11_2374	p2	(TG)12	24	13599114	13599137
chr11_2375	p2	(AC)6	12	13612701	13612712
chr11_2376	p2	(CA)12	24	13626665	13626688
chr11_2377	p2	(TG)11	22	13629826	13629847
chr11_2378	p2	(TG)8	16	13630461	13630476
chr11_2379	p2	(TG)18	36	13630744	13630779
chr11_2380	p4	(AATG)5	20	13631144	13631163
chr11_2381	p2	(TG)15	30	13637853	13637882
chr11_2382	c	)5ctctctctcctctcctgtcgtacccccctgctctctctcc(CT)6cgcgcg	84	13641077	13641160
chr11_2383	p2	(TG)6	12	13642957	13642968
chr11_2384	p2	(TG)16	32	13644853	13644884
chr11_2385	p2	(TG)12	24	13646709	13646732
chr11_2386	p2	(TG)8	16	13647267	13647282
chr11_2387	p2	(CA)9	18	13647417	13647434
chr11_2388	p2	(AC)7	14	13651736	13651749
chr11_2389	p2	(TG)6	12	13652437	13652448
chr11_2390	c	gactccatgtcagccttagccgacctgggctgggaggcttaccctaatgag	123	13656409	13656531
chr11_2391	p2	(AC)8	16	13656767	13656782
chr11_2392	p2	(TG)6	12	13660440	13660451
chr11_2393	p3	(TCT)5	15	13660773	13660787
chr11_2394	p2	(TG)9	18	13661474	13661491
chr11_2395	p2	(TC)6	12	13669170	13669181
chr11_2396	p2	(TC)10	20	13670853	13670872
chr11_2397	p2	(GT)6	12	13671147	13671158
chr11_2398	c	(CT)6t(TC)11	35	13677614	13677648
chr11_2399	p2	(TG)7	14	13677836	13677849
chr11_2400	p2	(TG)12	24	13678119	13678142
chr11_2401	p2	(AC)6	12	13678257	13678268
chr11_2402	p2	(GT)7	14	13678540	13678553
chr11_2403	c	(AC)13ag(AC)6	40	13682193	13682232
chr11_2404	p2	(TG)16	32	13682441	13682472
chr11_2405	p2	(CA)6	12	13682578	13682589

chr11_2406	p2	(AC)8	16	13682720	13682735
chr11_2407	p2	(CA)7	14	13685289	13685302
chr11_2408	p2	(CA)13	26	13685616	13685641
chr11_2409	p2	(TG)15	30	13692844	13692873
chr11_2410	p2	(AC)12	24	13698417	13698440
chr11_2411	p2	(TG)6	12	13698757	13698768
chr11_2412	p2	(GT)14	28	13700096	13700123
chr11_2413	p3	(ATG)5	15	13702527	13702541
chr11_2414	p2	(CA)7	14	13702847	13702860
chr11_2415	c	(TCT)5ttttcatcttcttcttcatcatcttcttcttcttattaataa(TAT)5	72	13705192	13705263
chr11_2416	p2	(AC)12	24	13708547	13708570
chr11_2417	p2	(TG)6	12	13708692	13708703
chr11_2418	p2	(GT)9	18	13708980	13708997
chr11_2419	p2	(AC)8	16	13710097	13710112
chr11_2420	p2	(CA)21	42	13710582	13710623
chr11_2421	p2	(TG)14	28	13711775	13711802
chr11_2422	c	(TAA)5tgtttctttttctgtatgtgtactgtagtga(GT)10	66	13712048	13712113
chr11_2423	p2	(TG)6	12	13715255	13715266
chr11_2424	c	(CA)10aacacacaa(AC)17	63	13715629	13715691
chr11_2425	p2	(AC)11	22	13716536	13716557
chr11_2426	p2	(CA)10	20	13716727	13716746
chr11_2427	p2	(TG)18	36	13716919	13716954
chr11_2428	p2	(TG)6	12	13717200	13717211
chr11_2429	p2	(CA)6	12	13717748	13717759
chr11_2430	p4	(GTTT)6	24	13718571	13718594
chr11_2431	p2	(CA)10	20	13718801	13718820
chr11_2432	p2	(AC)7	14	13721278	13721291
chr11_2433	p2	(AG)6	12	13724828	13724839
chr11_2434	p2	(TG)7	14	13733074	13733087
chr11_2435	p2	(CT)6	12	13736543	13736554
chr11_2436	p2	(TC)7	14	13736739	13736752
chr11_2437	p2	(TG)6	12	13737114	13737125
chr11_2438	c	(TC)7tttctaat(AC)9	40	13739342	13739381
chr11_2439	p2	(CA)7	14	13745295	13745308
chr11_2440	p2	(AC)15	30	13750359	13750388
chr11_2441	p2	(AC)7	14	13754613	13754626
chr11_2442	p2	(GT)6	12	13754821	13754832
chr11_2443	p2	(GT)6	12	13755064	13755075
chr11_2444	p2	(TG)16	32	13764892	13764923
chr11_2445	p2	(CA)6	12	13766691	13766702
chr11_2446	p2	(CT)6	12	13767301	13767312
chr11_2447	p2	(GT)19	38	13768585	13768622
chr11_2448	p2	(GA)6	12	13768761	13768772
chr11_2449	p2	(GT)30	60	13770834	13770893
chr11_2450	p2	(TG)6	12	13771880	13771891
chr11_2451	p2	(AC)8	16	13773209	13773224
chr11_2452	p5	(ATCAA)5	25	13781839	13781863
chr11_2453	p2	(CA)17	34	13794183	13794216
chr11_2454	p2	(GA)6	12	13798124	13798135
chr11_2455	p2	(GT)22	44	13801547	13801590
chr11_2456	p2	(GT)6	12	13802473	13802484
chr11_2457	p2	(AC)8	16	13806838	13806853

chr11_2458	c	(CT)10(CA)14	48	13812902	13812949
chr11_2459	p2	(GT)24	48	13814885	13814932
chr11_2460	c	(AC)16at(AC)15	64	13815049	13815112
chr11_2461	p2	(AC)7	14	13819958	13819971
chr11_2462	p2	(GA)6	12	13821747	13821758
chr11_2463	p2	(TG)20	40	13829133	13829172
chr11_2464	c	:tgcaccttcacatatatatgtaactgagttgcatgacacacagaaattgaat;	157	13833737	13833893
chr11_2465	p2	(TG)6	12	13834035	13834046
chr11_2466	p2	(AT)8	16	13834849	13834864
chr11_2467	p3	(TTC)6	18	13837970	13837987
chr11_2468	p2	(TG)17	34	13840671	13840704
chr11_2469	p2	(TG)8	16	13851911	13851926
chr11_2470	p4	(GTTT)5	20	13858974	13858993
chr11_2471	p2	(TG)21	42	13864654	13864695
chr11_2472	p2	(CA)11	22	13871949	13871970
chr11_2473	p2	(GT)11	22	13882417	13882438
chr11_2474	p2	(AC)6	12	13888624	13888635
chr11_2475	p2	(TG)14	28	13890262	13890289
chr11_2476	p2	(AC)6	12	13890486	13890497
chr11_2477	p2	(CA)12	24	13890778	13890801
chr11_2478	p3	(CTA)10	30	13901980	13902009
chr11_2479	p2	(AC)8	16	13902299	13902314
chr11_2480	p2	(AT)6	12	13902761	13902772
chr11_2481	p2	(AC)6	12	13906284	13906295
chr11_2482	c	gtgctcaaaacaaagccctttgtacaagcaagatcatttacatgcagaacaa	106	13908304	13908409
chr11_2483	p2	(AC)6	12	13908616	13908627
chr11_2484	c	(AC)6aa(AC)8	30	13914649	13914678
chr11_2485	p2	(CA)7	14	13915102	13915115
chr11_2486	p2	(CA)8	16	13915223	13915238
chr11_2487	p2	(AC)12	24	13916455	13916478
chr11_2488	p2	(GT)8	16	13919893	13919908
chr11_2489	p2	(GT)6	12	13936922	13936933
chr11_2490	p2	(GT)6	12	13937250	13937261
chr11_2491	p2	(AC)26	52	13941505	13941556
chr11_2492	c	:catgtcctcacagttccactgagtcaaagaccccctttcaaatgcctgctgtg	235	13943538	13943772
chr11_2493	c	(GACA)10(GATA)6	64	13943944	13944007
chr11_2494	p2	(CA)6	12	13950664	13950675
chr11_2495	p2	(CA)11	22	13951055	13951076
chr11_2496	p2	(TG)15	30	13958279	13958308
chr11_2497	p4	(AGAT)5	20	13958690	13958709
chr11_2498	p2	(CA)9	18	13959750	13959767
chr11_2499	p2	(CA)10	20	13960075	13960094
chr11_2500	p2	(GT)10	20	13976159	13976178
chr11_2501	p2	(CA)7	14	13977330	13977343
chr11_2502	p2	(AC)7	14	13981921	13981934
chr11_2503	p2	(TC)7	14	13988919	13988932
chr11_2504	p2	(TG)9	18	13996129	13996146
chr11_2505	p2	(TG)12	24	14002612	14002635
chr11_2506	p2	(AC)6	12	14002802	14002813
chr11_2507	p2	(AG)10	20	14008024	14008043
chr11_2508	p3	(GAA)5	15	14010143	14010157
chr11_2509	p3	(TTA)12	36	14012017	14012052

chr11_2510	p2	(CA)6	12	14012776	14012787
chr11_2511	p3	(AAC)6	18	14019134	14019151
chr11_2512	p2	(CA)9	18	14023681	14023698
chr11_2513	p2	(AC)12	24	14030788	14030811
chr11_2514	p3	(TGA)12	36	14055936	14055971
chr11_2515	p2	(CA)8	16	14071246	14071261
chr11_2516	p2	(GT)7	14	14072668	14072681
chr11_2517	p2	(AT)7	14	14074993	14075006
chr11_2518	p2	(TG)6	12	14079915	14079926
chr11_2519	p2	(TG)11	22	14084640	14084661
chr11_2520	p2	(CA)22	44	14093256	14093299
chr11_2521	p2	(TC)6	12	14105417	14105428
chr11_2522	p2	(CA)30	60	14107774	14107833
chr11_2523	p2	(TG)6	12	14109109	14109120
chr11_2524	p2	(CA)19	38	14109727	14109764
chr11_2525	p2	(AG)7	14	14115838	14115851
chr11_2526	p2	(CA)18	36	14116306	14116341
chr11_2527	p2	(TG)9	18	14144209	14144226
chr11_2528	c	(GA)6a(AG)7	27	14151304	14151330
chr11_2529	p2	(AG)6	12	14156281	14156292
chr11_2530	p2	(AT)6	12	14164305	14164316
chr11_2531	p2	(TG)6	12	14167135	14167146
chr11_2532	p2	(AC)6	12	14180032	14180043
chr11_2533	p5	(AGAAA)6	30	14188672	14188701
chr11_2534	p2	(AT)7	14	14196292	14196305
chr11_2535	p2	(GT)7	14	14197980	14197993
chr11_2536	p2	(TG)7	14	14202348	14202361
chr11_2537	p2	(GA)8	16	14202520	14202535
chr11_2538	p3	(CTG)6	18	14203705	14203722
chr11_2539	p2	(CA)6	12	14204013	14204024
chr11_2540	c	aacatatgcctaagcagcagaagagtgcagctgatggaatctactaatga	103	14212148	14212250
chr11_2541	p2	(CA)6	12	14220449	14220460
chr11_2542	p2	(AC)7	14	14221431	14221444
chr11_2543	p2	(CA)15	30	14226343	14226372
chr11_2544	p2	(AC)8	16	14227457	14227472
chr11_2545	p2	(GT)15	30	14229839	14229868
chr11_2546	p3	(ATA)6	18	14230405	14230422
chr11_2547	p2	(AC)7	14	14230821	14230834
chr11_2548	p3	(TGC)5	15	14231317	14231331
chr11_2549	c	(AAT)5aa(AAT)6	35	14235033	14235067
chr11_2550	p2	(CA)6	12	14239690	14239701
chr11_2551	p2	(TG)6	12	14240889	14240900
chr11_2552	p2	(GA)7	14	14248720	14248733
chr11_2553	p2	(GA)8	16	14259696	14259711
chr11_2554	p2	(GT)10	20	14262151	14262170
chr11_2555	p2	(GT)11	22	14264097	14264118
chr11_2556	p2	(TG)25	50	14273850	14273899
chr11_2557	p2	(TG)15	30	14274193	14274222
chr11_2558	p2	(GA)6	12	14279164	14279175
chr11_2559	p2	(AC)14	28	14280317	14280344
chr11_2560	p2	(AG)8	16	14282894	14282909
chr11_2561	p2	(CA)6	12	14286731	14286742

chr11_2562	p2	(GT)6	12	14288066	14288077
chr11_2563	p2	(AC)6	12	14290639	14290650
chr11_2564	p2	(AC)6	12	14291951	14291962
chr11_2565	p2	(AG)6	12	14292781	14292792
chr11_2566	p2	(TG)14	28	14297567	14297594
chr11_2567	p2	(CA)18	36	14302382	14302417
chr11_2568	p2	(TC)6	12	14304972	14304983
chr11_2569	c	(TC)10(AC)33	86	14307059	14307144
chr11_2570	p2	(GT)17	34	14307999	14308032
chr11_2571	p2	(AC)7	14	14309016	14309029
chr11_2572	p2	(CA)14	28	14311207	14311234
chr11_2573	p3	(ACT)23	69	14312479	14312547
chr11_2574	p2	(TA)6	12	14326662	14326673
chr11_2575	p2	(GA)8	16	14329629	14329644
chr11_2576	p2	(TG)8	16	14333471	14333486
chr11_2577	p3	(GCT)6	18	14335616	14335633
chr11_2578	p3	(TAA)5	15	14340535	14340549
chr11_2579	p2	(GA)6	12	14353208	14353219
chr11_2580	p2	(CT)9	18	14374504	14374521
chr11_2581	p2	(CT)9	18	14380703	14380720
chr11_2582	p3	(CTC)5	15	14391030	14391044
chr11_2583	p3	(ATT)9	27	14398393	14398419
chr11_2584	p2	(GT)18	36	14404901	14404936
chr11_2585	p2	(CA)14	28	14405229	14405256
chr11_2586	p2	(GT)17	34	14410231	14410264
chr11_2587	p2	(TG)8	16	14410894	14410909
chr11_2588	p2	(TC)7	14	14414623	14414636
chr11_2589	c	(AC)7(AT)6ac(AT)7(AC)12	66	14420184	14420249
chr11_2590	p3	(AAC)6	18	14425012	14425029
chr11_2591	p2	(CA)19	38	14437751	14437788
chr11_2592	p2	(AT)6	12	14442583	14442594
chr11_2593	p2	(GT)9	18	14443087	14443104
chr11_2594	p2	(TG)6	12	14461640	14461651
chr11_2595	p2	(AG)9	18	14465855	14465872
chr11_2596	p2	(AC)12	24	14470738	14470761
chr11_2597	p2	(AT)7	14	14475645	14475658
chr11_2598	p2	(TG)7	14	14476689	14476702
chr11_2599	p2	(TG)6	12	14482427	14482438
chr11_2600	p2	(GA)8	16	14485175	14485190
chr11_2601	c	(AC)32atatatactgtata(TG)6	90	14493946	14494035
chr11_2602	p2	(CA)6	12	14500534	14500545
chr11_2603	p4	(ACAA)7	28	14506294	14506321
chr11_2604	p2	(AC)11	22	14508819	14508840
chr11_2605	p2	(CT)17	34	14520927	14520960
chr11_2606	p2	(TC)6	12	14522655	14522666
chr11_2607	p2	(GA)6	12	14526053	14526064
chr11_2608	p2	(TG)16	32	14553672	14553703
chr11_2609	p3	(ACA)10	30	14561586	14561615
chr11_2610	p2	(AC)14	28	14599164	14599191
chr11_2611	c	(CCA)8(CCT)8	48	14608935	14608982
chr11_2612	p3	(CAC)5	15	14624982	14624996
chr11_2613	p3	(GCG)6	18	14627257	14627274

chr11_2614	p3	(ATT)5	15	14630664	14630678
chr11_2615	p2	(CT)7	14	14633447	14633460
chr11_2616	p2	(TG)6	12	14636575	14636586
chr11_2617	c	(TG)10(CGTG)5	40	14636992	14637031
chr11_2618	p2	(TC)13	26	14656711	14656736
chr11_2619	p2	(GT)22	44	14668313	14668356
chr11_2620	p4	(CAGA)6	24	14699487	14699510
chr11_2621	p3	(AGC)5	15	14720917	14720931
chr11_2622	p2	(AC)6	12	14721927	14721938
chr11_2623	p2	(AG)8	16	14722154	14722169
chr11_2624	p2	(TG)20	40	14725146	14725185
chr11_2625	p2	(CA)6	12	14731778	14731789
chr11_2626	p2	(TC)6	12	14737777	14737788
chr11_2627	p2	(CA)7	14	14743887	14743900
chr11_2628	p3	(GCA)37	111	14745452	14745562
chr11_2629	p2	(TG)7	14	14745967	14745980
chr11_2630	p2	(AC)6	12	14746179	14746190
chr11_2631	p2	(AC)9	18	14751152	14751169
chr11_2632	p2	(CT)7	14	14754690	14754703
chr11_2633	p2	(TG)12	24	14757776	14757799
chr11_2634	p2	(TC)6	12	14759008	14759019
chr11_2635	p3	(TTG)5	15	14766527	14766541
chr11_2636	p2	(GT)18	36	14772828	14772863
chr11_2637	p2	(AC)6	12	14788609	14788620
chr11_2638	p2	(CA)17	34	14791270	14791303
chr11_2639	p2	(TA)7	14	14791881	14791894
chr11_2640	p2	(AC)6	12	14800818	14800829
chr11_2641	p3	(ATT)6	18	14803167	14803184
chr11_2642	p3	(GAG)6	18	14831309	14831326
chr11_2643	c	ccggaccagcgtttgcccgctcctttatcctgttgtctgttttcctccagccta	190	14832473	14832662
chr11_2644	p2	(TG)14	28	14833671	14833698
chr11_2645	p2	(GT)6	12	14839260	14839271
chr11_2646	p2	(GT)7	14	14840718	14840731
chr11_2647	p2	(TG)22	44	14841651	14841694
chr11_2648	p4	(TTAT)6	24	14846462	14846485
chr11_2649	p3	(TCC)6	18	14852462	14852479
chr11_2650	p2	(TC)8	16	14865314	14865329
chr11_2651	p2	(AC)7	14	14868025	14868038
chr11_2652	p2	(GT)6	12	14910014	14910025
chr11_2653	c	(AT)10ccagt(AC)6	37	14911969	14912005
chr11_2654	p2	(TG)6	12	14912352	14912363
chr11_2655	p2	(TG)7	14	14921638	14921651
chr11_2656	p4	(TACC)5	20	14924136	14924155
chr11_2657	p2	(TG)11	22	14949597	14949618
chr11_2658	p2	(AC)17	34	14951413	14951446
chr11_2659	p2	(CA)12	24	14954871	14954894
chr11_2660	p2	(CT)43	86	14957784	14957869
chr11_2661	p2	(GT)9	18	14959066	14959083
chr11_2662	p2	(AC)6	12	14972303	14972314
chr11_2663	p2	(AC)14	28	14975424	14975451
chr11_2664	p2	(GT)12	24	14983330	14983353
chr11_2665	c	(AT)9g(TA)11	41	14985279	14985319

chr11_2666	p2	(AT)9	18	14988995	14989012
chr11_2667	p2	(TG)8	16	14989190	14989205
chr11_2668	p2	(TG)11	22	14990247	14990268
chr11_2669	p5	(GTGGG)5	25	14992556	14992580
chr11_2670	p2	(GT)8	16	15005104	15005119
chr11_2671	p2	(AC)7	14	15005226	15005239
chr11_2672	p2	(TG)10	20	15005550	15005569
chr11_2673	p2	(AC)8	16	15009740	15009755
chr11_2674	p2	(TG)9	18	15009900	15009917
chr11_2675	p2	(GA)7	14	15010639	15010652
chr11_2676	p2	(TA)8	16	15014204	15014219
chr11_2677	p2	(AC)8	16	15014326	15014341
chr11_2678	p3	(TTG)5	15	15016970	15016984
chr11_2679	p3	(TCC)6	18	15032415	15032432
chr11_2680	p2	(GT)6	12	15033909	15033920
chr11_2681	p2	(CA)7	14	15035545	15035558
chr11_2682	p2	(GT)10	20	15037373	15037392
chr11_2683	c	(TG)26(AG)11	74	15040628	15040701
chr11_2684	p2	(GT)13	26	15041082	15041107
chr11_2685	p2	(AC)6	12	15041479	15041490
chr11_2686	p2	(GT)6	12	15047294	15047305
chr11_2687	p2	(GT)35	70	15048765	15048834
chr11_2688	p2	(TA)8	16	15054291	15054306
chr11_2689	p2	(AC)16	32	15059025	15059056
chr11_2690	p4	(TTTA)7	28	15059741	15059768
chr11_2691	p4	(TTTG)5	20	15059988	15060007
chr11_2692	p2	(CA)24	48	15061032	15061079
chr11_2693	p2	(GT)6	12	15069221	15069232
chr11_2694	p2	(AC)33	66	15081838	15081903
chr11_2695	p4	(ATGG)5	20	15087835	15087854
chr11_2696	p2	(GT)9	18	15093354	15093371
chr11_2697	c	(TG)6tctgtgtgtgt(GA)7	37	15094381	15094417
chr11_2698	p2	(TG)7	14	15101294	15101307
chr11_2699	c	(GT)36(GA)7	86	15102299	15102384
chr11_2700	p3	(GAT)5	15	15107368	15107382
chr11_2701	p3	(TAA)5	15	15128703	15128717
chr11_2702	p2	(AC)8	16	15131191	15131206
chr11_2703	p2	(CA)14	28	15133116	15133143
chr11_2704	p2	(CA)6	12	15134876	15134887
chr11_2705	p2	(GT)6	12	15136211	15136222
chr11_2706	p2	(TG)21	42	15136806	15136847
chr11_2707	p2	(CA)6	12	15139676	15139687
chr11_2708	p2	(CA)18	36	15140608	15140643
chr11_2709	c	(AC)6t(CA)7	27	15145195	15145221
chr11_2710	p5	(TACTG)5	25	15180389	15180413
chr11_2711	p2	(GT)17	34	15180605	15180638
chr11_2712	p2	(TG)24	48	15189867	15189914
chr11_2713	p3	(GAG)5	15	15192279	15192293
chr11_2714	p2	(AC)25	50	15199317	15199366
chr11_2715	p2	(AC)6	12	15205122	15205133
chr11_2716	p2	(CT)6	12	15205508	15205519
chr11_2717	p2	(GA)6	12	15211603	15211614

chr11_2718	c	catatat(AC)6atacatatacacatatat(AC)7atat(AC)6atacatat	248	15226660	15226907
chr11_2719	p3	(ATC)8	24	15240244	15240267
chr11_2720	p2	(AC)6	12	15248975	15248986
chr11_2721	p2	(GT)16	32	15250801	15250832
chr11_2722	p2	(TG)14	28	15255693	15255720
chr11_2723	p2	(CA)16	32	15256188	15256219
chr11_2724	p2	(AC)6	12	15257963	15257974
chr11_2725	p2	(AC)7	14	15260763	15260776
chr11_2726	p2	(AC)6	12	15268705	15268716
chr11_2727	p2	(GA)9	18	15278226	15278243
chr11_2728	p2	(GA)6	12	15285336	15285347
chr11_2729	p4	(AGAA)6	24	15295302	15295325
chr11_2730	p3	(CTT)5	15	15314725	15314739
chr11_2731	p2	(AT)9	18	15323056	15323073
chr11_2732	c	(AT)6(GT)26	64	15327036	15327099
chr11_2733	p3	(AGC)8	24	15335633	15335656
chr11_2734	p2	(GT)9	18	15336184	15336201
chr11_2735	p2	(AG)6	12	15344408	15344419
chr11_2736	p2	(AC)11	22	15350793	15350814
chr11_2737	c	(TC)45(AC)8	106	15351064	15351169
chr11_2738	p2	(CA)15	30	15361670	15361699
chr11_2739	p2	(TA)6	12	15382114	15382125
chr11_2740	p2	(GT)9	18	15386863	15386880
chr11_2741	p3	(TTC)8	24	15391964	15391987
chr11_2742	p5	(ATTTG)6	30	15414407	15414436
chr11_2743	p3	(AAC)10	30	15433447	15433476
chr11_2744	p3	(TTA)6	18	15448323	15448340
chr11_2745	p2	(AC)6	12	15468661	15468672
chr11_2746	p2	(AC)23	46	15476111	15476156
chr11_2747	p2	(AT)6	12	15477254	15477265
chr11_2748	p2	(CA)7	14	15492688	15492701
chr11_2749	p4	(CCAT)5	20	15493903	15493922
chr11_2750	p2	(AC)16	32	15513944	15513975
chr11_2751	p3	(TCC)5	15	15533507	15533521
chr11_2752	p2	(AC)6	12	15541316	15541327
chr11_2753	p2	(TG)10	20	15543157	15543176
chr11_2754	p2	(AC)18	36	15548614	15548649
chr11_2755	p2	(CT)7	14	15578947	15578960
chr11_2756	p2	(CA)7	14	15587246	15587259
chr11_2757	p4	(AATG)5	20	15602981	15603000
chr11_2758	p2	(TG)10	20	15605645	15605664
chr11_2759	p2	(AT)6	12	15612142	15612153
chr11_2760	p2	(AC)7	14	15613057	15613070
chr11_2761	p2	(AC)8	16	15613471	15613486
chr11_2762	p2	(TG)15	30	15614585	15614614
chr11_2763	p2	(TC)6	12	15670677	15670688
chr11_2764	c	(TG)12tatgtgtgtata(TG)9(AG)9	72	15671185	15671256
chr11_2765	c	(TG)15cgcgcgc(GT)8	53	15680285	15680337
chr11_2766	p2	(GT)15	30	15681409	15681438
chr11_2767	p2	(TA)7	14	15684706	15684719
chr11_2768	p2	(AC)9	18	15686486	15686503
chr11_2769	p2	(CA)17	34	15696371	15696404

chr11_2770	p2	(CT)6	12	15702547	15702558
chr11_2771	p2	(GT)7	14	15703913	15703926
chr11_2772	c	(AC)12g(CA)7	39	15704948	15704986
chr11_2773	p4	(ATGG)5	20	15720854	15720873
chr11_2774	p2	(CA)6	12	15723503	15723514
chr11_2775	p2	(CT)6	12	15723748	15723759
chr11_2776	p2	(GT)18	36	15731788	15731823
chr11_2777	p2	(AC)12	24	15760372	15760395
chr11_2778	p2	(GA)6	12	15764340	15764351
chr11_2779	p5	(ATCAA)5	25	15768407	15768431
chr11_2780	p2	(GA)6	12	15768794	15768805
chr11_2781	p3	(GGA)6	18	15781065	15781082
chr11_2782	p2	(GT)6	12	15782631	15782642
chr11_2783	p2	(AC)8	16	15789001	15789016
chr11_2784	p3	(AAC)11	33	15794849	15794881
chr11_2785	p4	(ACAT)5	20	15808719	15808738
chr11_2786	p2	(TA)6	12	15812167	15812178
chr11_2787	p2	(AC)9	18	15827593	15827610
chr11_2788	p2	(TG)12	24	15836728	15836751
chr11_2789	p2	(GT)9	18	15842277	15842294
chr11_2790	p2	(TC)8	16	15842788	15842803
chr11_2791	p3	(TGT)6	18	15846127	15846144
chr11_2792	c	(CA)6g(AC)6	25	15846283	15846307
chr11_2793	p3	(TCC)7	21	15847167	15847187
chr11_2794	p2	(GT)7	14	15847302	15847315
chr11_2795	p2	(TG)6	12	15850384	15850395
chr11_2796	p2	(CA)7	14	15850767	15850780
chr11_2797	p2	(AC)6	12	15851030	15851041
chr11_2798	p2	(AC)9	18	15851589	15851606
chr11_2799	c	(CA)8cttaa(AC)7	35	15859452	15859486
chr11_2800	p2	(TG)14	28	15859806	15859833
chr11_2801	p5	(GAGAG)7	35	15860186	15860220
chr11_2802	p2	(CA)7	14	15870500	15870513
chr11_2803	p2	(TG)9	18	15874036	15874053
chr11_2804	p2	(GT)9	18	15874558	15874575
chr11_2805	p2	(CA)55	110	15878991	15879100
chr11_2806	c	(GA)7gt(GA)6	28	15880665	15880692
chr11_2807	p2	(TG)6	12	15882507	15882518
chr11_2808	p2	(CA)11	22	15884609	15884630
chr11_2809	p2	(TG)7	14	15895653	15895666
chr11_2810	p2	(AC)10	20	15895778	15895797
chr11_2811	p2	(GT)10	20	15896866	15896885
chr11_2812	p2	(GT)9	18	15901692	15901709
chr11_2813	p2	(CA)6	12	15901960	15901971
chr11_2814	p3	(AAC)5	15	15902368	15902382
chr11_2815	c	ıggcagagaaagggggcacagtgtgctaatcagataaggagctccatgat	130	15913338	15913467
chr11_2816	p2	(AC)6	12	15919850	15919861
chr11_2817	p2	(TG)6	12	15922052	15922063
chr11_2818	p2	(GT)11	22	15923034	15923055
chr11_2819	p2	(TA)6	12	15926379	15926390
chr11_2820	p4	(TCTA)5	20	15939663	15939682
chr11_2821	p2	(GT)25	50	15946900	15946949

chr11_2822	p2	(GA)6	12	15947170	15947181
chr11_2823	p2	(GA)6	12	15955653	15955664
chr11_2824	p2	(TA)6	12	15957366	15957377
chr11_2825	p3	(TAA)6	18	15961855	15961872
chr11_2826	p2	(TC)6	12	15961997	15962008
chr11_2827	c	(TC)7(AC)11	36	15972704	15972739
chr11_2828	p2	(GT)10	20	15972865	15972884
chr11_2829	p2	(CA)6	12	15975456	15975467
chr11_2830	p2	(TG)15	30	15976115	15976144
chr11_2831	p2	(AG)7	14	15982128	15982141
chr11_2832	p2	(AC)12	24	15982907	15982930
chr11_2833	p2	(AC)6	12	15985445	15985456
chr11_2834	p2	(GT)21	42	15985635	15985676
chr11_2835	p2	(CA)13	26	15986805	15986830
chr11_2836	p2	(TG)8	16	15989637	15989652
chr11_2837	p2	(GT)6	12	15998458	15998469
chr11_2838	p3	(CCA)6	18	16004422	16004439
chr11_2839	p2	(AC)6	12	16006873	16006884
chr11_2840	p2	(GT)18	36	16009680	16009715
chr11_2841	p2	(CA)18	36	16011087	16011122
chr11_2842	p2	(CA)17	34	16016212	16016245
chr11_2843	p2	(GT)13	26	16016411	16016436
chr11_2844	p2	(GT)10	20	16017585	16017604
chr11_2845	p2	(GT)8	16	16018101	16018116
chr11_2846	p2	(AC)18	36	16024815	16024850
chr11_2847	p2	(GT)8	16	16027045	16027060
chr11_2848	p3	(AGT)5	15	16034660	16034674
chr11_2849	p2	(CG)6	12	16037223	16037234
chr11_2850	p2	(AC)7	14	16042577	16042590
chr11_2851	c	(AC)9aattcttaaacatc(CA)10	52	16063037	16063088
chr11_2852	p2	(GT)6	12	16079662	16079673
chr11_2853	p5	(AAGTT)6	30	16080529	16080558
chr11_2854	p2	(CA)6	12	16083226	16083237
chr11_2855	p3	(CAC)5	15	16084044	16084058
chr11_2856	p2	(TG)8	16	16091354	16091369
chr11_2857	p2	(TG)6	12	16091757	16091768
chr11_2858	p2	(GT)6	12	16092197	16092208
chr11_2859	p3	(CTT)5	15	16094900	16094914
chr11_2860	p2	(TG)7	14	16095180	16095193
chr11_2861	p2	(AG)6	12	16096357	16096368
chr11_2862	p2	(AT)7	14	16106146	16106159
chr11_2863	p3	(TCC)5	15	16109443	16109457
chr11_2864	p2	(AG)8	16	16127397	16127412
chr11_2865	p2	(TG)7	14	16127665	16127678
chr11_2866	p2	(TG)12	24	16130517	16130540
chr11_2867	p2	(CA)7	14	16130671	16130684
chr11_2868	p2	(AC)8	16	16132724	16132739
chr11_2869	p2	(CA)8	16	16146462	16146477
chr11_2870	p2	(GT)12	24	16149088	16149111
chr11_2871	p2	(AC)19	38	16153857	16153894
chr11_2872	p3	(TTC)6	18	16159700	16159717
chr11_2873	p3	(CCT)5	15	16178479	16178493

chr11_2874	p2	(TG)13	26	16183862	16183887
chr11_2875	p2	(AC)6	12	16191293	16191304
chr11_2876	p2	(TG)12	24	16192188	16192211
chr11_2877	p2	(AC)6	12	16194738	16194749
chr11_2878	p2	(AC)7	14	16213675	16213688
chr11_2879	p2	(TG)9	18	16215920	16215937
chr11_2880	c	(TAA)5tatt(AAG)5	34	16218000	16218033
chr11_2881	p2	(GT)18	36	16218223	16218258
chr11_2882	p2	(TG)21	42	16231321	16231362
chr11_2883	p2	(GT)23	46	16250767	16250812
chr11_2884	p5	(GATTT)5	25	16265130	16265154
chr11_2885	p3	(TCC)5	15	16268132	16268146
chr11_2886	p2	(TG)18	36	16287126	16287161
chr11_2887	p2	(GT)12	24	16288394	16288417
chr11_2888	p2	(TG)13	26	16292545	16292570
chr11_2889	p2	(TG)6	12	16318282	16318293
chr11_2890	p2	(AC)6	12	16320781	16320792
chr11_2891	p2	(TG)14	28	16328230	16328257
chr11_2892	p2	(GA)7	14	16341744	16341757
chr11_2893	p2	(CA)8	16	16343987	16344002
chr11_2894	p2	(AG)6	12	16363840	16363851
chr11_2895	p2	(GT)7	14	16370944	16370957
chr11_2896	p3	(CCT)5	15	16375995	16376009
chr11_2897	c	(TC)6(AC)6	24	16378543	16378566
chr11_2898	p2	(TC)6	12	16393055	16393066
chr11_2899	p3	(ATT)6	18	16405137	16405154
chr11_2900	p2	(TG)6	12	16421226	16421237
chr11_2901	p2	(AG)6	12	16421818	16421829
chr11_2902	p2	(GT)7	14	16424138	16424151
chr11_2903	c	(GT)6g(GT)30	73	16427716	16427788
chr11_2904	p2	(AC)13	26	16429358	16429383
chr11_2905	p3	(CAT)5	15	16452671	16452685
chr11_2906	p2	(TG)6	12	16464245	16464256
chr11_2907	p2	(AC)14	28	16466086	16466113
chr11_2908	p2	(CA)9	18	16466343	16466360
chr11_2909	p2	(AC)6	12	16466479	16466490
chr11_2910	p2	(AC)7	14	16466905	16466918
chr11_2911	p4	(AGGG)5	20	16467598	16467617
chr11_2912	p4	(AAAC)5	20	16474130	16474149
chr11_2913	p2	(TC)6	12	16474678	16474689
chr11_2914	p2	(CA)12	24	16482511	16482534
chr11_2915	p2	(TA)7	14	16482989	16483002
chr11_2916	p2	(TG)6	12	16495556	16495567
chr11_2917	p2	(GT)9	18	16505261	16505278
chr11_2918	c	.aagttaaatttacagagtaggaggtagagagctgccctcaggcttagtatct	132	16505433	16505564
chr11_2919	p2	(TC)8	16	16522374	16522389
chr11_2920	p2	(AC)21	42	16528264	16528305
chr11_2921	p3	(TAC)6	18	16529106	16529123
chr11_2922	p2	(TG)8	16	16534397	16534412
chr11_2923	p2	(GT)16	32	16540633	16540664
chr11_2924	p2	(CA)23	46	16546868	16546913
chr11_2925	c	(CA)18cttgtgtgtgtgcatata(GT)8	70	16554675	16554744

chr11_2926	p3	(ATA)5	15	16562844	16562858
chr11_2927	p2	(TC)9	18	16575770	16575787
chr11_2928	p2	(AC)10	20	16584894	16584913
chr11_2929	p2	(CA)15	30	16586244	16586273
chr11_2930	p2	(AT)6	12	16587361	16587372
chr11_2931	p3	(ACA)6	18	16593697	16593714
chr11_2932	p2	(TG)8	16	16595412	16595427
chr11_2933	p2	(AG)6	12	16596131	16596142
chr11_2934	p2	(AG)8	16	16597492	16597507
chr11_2935	p5	(ATCAA)5	25	16603094	16603118
chr11_2936	c	gtctgcctcttcgcagctgcttaaaaataaagcccccccctcaaaaacctcaa	97	16607722	16607818
chr11_2937	p2	(AC)37	74	16610464	16610537
chr11_2938	p2	(TG)25	50	16613858	16613907
chr11_2939	p2	(CA)18	36	16619000	16619035
chr11_2940	p2	(GT)6	12	16622473	16622484
chr11_2941	p2	(CA)6	12	16622901	16622912
chr11_2942	p2	(TG)14	28	16624414	16624441
chr11_2943	p2	(AC)6	12	16643723	16643734
chr11_2944	p2	(CA)12	24	16643919	16643942
chr11_2945	c	(TTC)12ttag(TAT)9(TAG)11	100	16644906	16645005
chr11_2946	p2	(TG)9	18	16654732	16654749
chr11_2947	p2	(GA)7	14	16659788	16659801
chr11_2948	p2	(TG)8	16	16669452	16669467
chr11_2949	p4	(TTTG)5	20	16676019	16676038
chr11_2950	p3	(GCA)6	18	16676256	16676273
chr11_2951	p2	(GT)6	12	16678959	16678970
chr11_2952	p2	(CA)6	12	16683037	16683048
chr11_2953	p2	(AC)9	18	16695560	16695577
chr11_2954	c	(TC)9tgtct(CA)8	39	16697275	16697313
chr11_2955	c	(CT)7ttcttcctt(TC)7	37	16705339	16705375
chr11_2956	p2	(TG)7	14	16710741	16710754
chr11_2957	p2	(TG)17	34	16713629	16713662
chr11_2958	p3	(AAT)5	15	16752703	16752717
chr11_2959	p4	(TTGA)6	24	16762724	16762747
chr11_2960	p3	(ATA)5	15	16783616	16783630
chr11_2961	p2	(TG)8	16	16790042	16790057
chr11_2962	p3	(AGC)6	18	16792832	16792849
chr11_2963	p2	(GT)16	32	16793970	16794001
chr11_2964	p3	(CCA)6	18	16816045	16816062
chr11_2965	p2	(CA)6	12	16816646	16816657
chr11_2966	p2	(AG)6	12	16818164	16818175
chr11_2967	c	aaatcatctaaaatattgttcttgaaattattatttcttttagtgctgtcaattgatt	145	16819659	16819803
chr11_2968	p2	(CA)6	12	16827540	16827551
chr11_2969	p3	(ATA)8	24	16834780	16834803
chr11_2970	p2	(GT)6	12	16841796	16841807
chr11_2971	p4	(CATC)6	24	16845209	16845232
chr11_2972	p2	(AC)6	12	16847212	16847223
chr11_2973	p5	(ATAGA)6	30	16847351	16847380
chr11_2974	p2	(AC)28	56	16852404	16852459
chr11_2975	p2	(GT)12	24	16856797	16856820
chr11_2976	p2	(TG)10	20	16856992	16857011
chr11_2977	p3	(TCT)8	24	16861058	16861081

chr11_2978	p2	(AT)6	12	16871878	16871889
chr11_2979	p5	(TCTCT)11	55	16883662	16883716
chr11_2980	p3	(TCA)5	15	16884909	16884923
chr11_2981	p2	(AC)8	16	16886281	16886296
chr11_2982	p2	(AG)10	20	16886731	16886750
chr11_2983	p2	(TG)16	32	16891977	16892008
chr11_2984	p3	(ACA)6	18	16906457	16906474
chr11_2985	p3	(GCA)5	15	16909563	16909577
chr11_2986	p3	(AAT)5	15	16918939	16918953
chr11_2987	p3	(TTA)5	15	16930958	16930972
chr11_2988	p2	(CA)7	14	16931912	16931925
chr11_2989	p4	(CCAT)5	20	16932480	16932499
chr11_2990	p3	(TTA)5	15	16933249	16933263
chr11_2991	p2	(CA)7	14	16934203	16934216
chr11_2992	p4	(CCAT)5	20	16934771	16934790
chr11_2993	p2	(GT)15	30	16940347	16940376
chr11_2994	p2	(TG)14	28	16943643	16943670
chr11_2995	p2	(TG)6	12	16966289	16966300
chr11_2996	p3	(TGT)5	15	16967645	16967659
chr11_2997	p2	(GT)14	28	16973119	16973146
chr11_2998	p2	(CA)23	46	16988419	16988464
chr11_2999	p4	(TTTG)5	20	17008730	17008749
chr11_3000	p2	(TG)9	18	17023165	17023182
chr11_3001	p2	(TG)7	14	17024529	17024542
chr11_3002	p2	(TG)6	12	17032293	17032304
chr11_3003	c	(TG)6tc(TG)7	28	17047937	17047964
chr11_3004	p2	(CA)6	12	17054949	17054960
chr11_3005	p2	(GT)16	32	17056268	17056299
chr11_3006	p4	(AAAT)5	20	17080158	17080177
chr11_3007	p3	(CAC)5	15	17081997	17082011
chr11_3008	p3	(TAA)5	15	17084452	17084466
chr11_3009	p2	(AC)15	30	17090545	17090574
chr11_3010	p2	(GT)6	12	17093063	17093074
chr11_3011	p2	(AC)16	32	17093607	17093638
chr11_3012	c	(CCA)5caacagacacacat(AC)13	55	17098570	17098624
chr11_3013	p2	(AC)10	20	17100269	17100288
chr11_3014	p2	(TG)11	22	17100915	17100936
chr11_3015	p2	(TG)7	14	17106082	17106095
chr11_3016	p2	(AC)16	32	17107363	17107394
chr11_3017	p2	(TG)6	12	17109124	17109135
chr11_3018	p3	(TTC)5	15	17113498	17113512
chr11_3019	p2	(GT)6	12	17115800	17115811
chr11_3020	p5	(ATTTG)5	25	17118767	17118791
chr11_3021	c	(TA)9catatatatatcatggatttgtttt(TA)6	55	17134667	17134721
chr11_3022	p2	(CA)9	18	17143761	17143778
chr11_3023	p2	(GT)8	16	17149508	17149523
chr11_3024	p2	(CA)7	14	17150068	17150081
chr11_3025	p2	(AC)6	12	17152825	17152836
chr11_3026	p4	(TTTA)5	20	17158374	17158393
chr11_3027	c	tttgttgtcattttacacatttg(AC)7agggaaatgaaatattgttcctaatg	190	17159540	17159729
chr11_3028	p3	(TAA)7	21	17163629	17163649
chr11_3029	p2	(AT)9	18	17165942	17165959

chr11_3030	p2	(AG)13	26	17174621	17174646
chr11_3031	p3	(ATG)5	15	17177220	17177234
chr11_3032	p2	(CA)11	22	17177750	17177771
chr11_3033	p2	(AG)6	12	17178066	17178077
chr11_3034	p2	(CA)25	50	17190401	17190450
chr11_3035	p2	(TG)6	12	17192708	17192719
chr11_3036	p2	(CT)6	12	17206055	17206066
chr11_3037	p5	(TAGCT)5	25	17211026	17211050
chr11_3038	p2	(AG)7	14	17213941	17213954
chr11_3039	p2	(AC)16	32	17215226	17215257
chr11_3040	p2	(CA)22	44	17215361	17215404
chr11_3041	p2	(AC)12	24	17219091	17219114
chr11_3042	p2	(AC)6	12	17220695	17220706
chr11_3043	p2	(AC)12	24	17221814	17221837
chr11_3044	p2	(AG)9	18	17224840	17224857
chr11_3045	p2	(CA)9	18	17226516	17226533
chr11_3046	p2	(GT)7	14	17230274	17230287
chr11_3047	p2	(GA)7	14	17231098	17231111
chr11_3048	p2	(AC)10	20	17243307	17243326
chr11_3049	p2	(AC)26	52	17268372	17268423
chr11_3050	p2	(AG)7	14	17279325	17279338
chr11_3051	p2	(CA)8	16	17280274	17280289
chr11_3052	p5	(GAAGA)5	25	17280794	17280818
chr11_3053	p2	(GA)6	12	17284902	17284913
chr11_3054	p2	(AG)6	12	17288419	17288430
chr11_3055	p2	(AT)6	12	17291192	17291203
chr11_3056	c	(TGGA)5(TAGA)14	76	17295255	17295330
chr11_3057	p3	(TCC)5	15	17298031	17298045
chr11_3058	p2	(AC)7	14	17302201	17302214
chr11_3059	p2	(TG)11	22	17335547	17335568
chr11_3060	p4	(TTTG)5	20	17338097	17338116
chr11_3061	p2	(TG)6	12	17346229	17346240
chr11_3062	p4	(TCCA)5	20	17346462	17346481
chr11_3063	p2	(GA)6	12	17373340	17373351
chr11_3064	p2	(AC)11	22	17376721	17376742
chr11_3065	c	(GT)11(TG)11	44	17381412	17381455
chr11_3066	p2	(TG)10	20	17384273	17384292
chr11_3067	p2	(TC)7	14	17405952	17405965
chr11_3068	p2	(AC)10	20	17407527	17407546
chr11_3069	p2	(AC)16	32	17413054	17413085
chr11_3070	p3	(ATG)5	15	17414565	17414579
chr11_3071	p2	(AC)7	14	17414942	17414955
chr11_3072	c	(CA)6t(AC)7	27	17418337	17418363
chr11_3073	p2	(CA)6	12	17420299	17420310
chr11_3074	c	(TG)7ta(TG)22	60	17420565	17420624
chr11_3075	p2	(GT)7	14	17425043	17425056
chr11_3076	p2	(AC)6	12	17426464	17426475
chr11_3077	p2	(CT)6	12	17427116	17427127
chr11_3078	p2	(AC)14	28	17427607	17427634
chr11_3079	p3	(AAC)8	24	17446762	17446785
chr11_3080	p2	(AC)7	14	17447470	17447483
chr11_3081	p2	(TA)6	12	17454605	17454616

chr11_3082	p2	(CA)21	42	17455076	17455117
chr11_3083	p2	(TC)8	16	17455497	17455512
chr11_3084	p2	(CA)13	26	17456461	17456486
chr11_3085	p3	(AAT)6	18	17460133	17460150
chr11_3086	p2	(CA)7	14	17466598	17466611
chr11_3087	p3	(AAT)5	15	17468512	17468526
chr11_3088	p2	(TG)14	28	17483514	17483541
chr11_3089	p2	(GT)7	14	17483828	17483841
chr11_3090	p4	(AGAT)12	48	17484644	17484691
chr11_3091	p2	(GT)11	22	17491763	17491784
chr11_3092	p2	(AC)8	16	17491972	17491987
chr11_3093	p2	(TC)6	12	17494392	17494403
chr11_3094	p4	(ATTC)5	20	17495289	17495308
chr11_3095	c	(TA)6(TG)7	26	17508550	17508575
chr11_3096	p2	(AC)13	26	17511353	17511378
chr11_3097	p3	(CTC)5	15	17512280	17512294
chr11_3098	p2	(TG)6	12	17513242	17513253
chr11_3099	p2	(CA)20	40	17524752	17524791
chr11_3100	p2	(TG)6	12	17537596	17537607
chr11_3101	p2	(AT)6	12	17542354	17542365
chr11_3102	p2	(AC)7	14	17543012	17543025
chr11_3103	p2	(CA)10	20	17546317	17546336
chr11_3104	p2	(TC)8	16	17563572	17563587
chr11_3105	p2	(CA)6	12	17566728	17566739
chr11_3106	p2	(AT)6	12	17571584	17571595
chr11_3107	p5	(AAATC)5	25	17580679	17580703
chr11_3108	p2	(GT)6	12	17581918	17581929
chr11_3109	p2	(GT)30	60	17596643	17596702
chr11_3110	p2	(AC)13	26	17598345	17598370
chr11_3111	p2	(CA)6	12	17599155	17599166
chr11_3112	p3	(GAG)7	21	17600436	17600456
chr11_3113	p4	(TAGA)13	52	17603844	17603895
chr11_3114	p2	(TC)7	14	17604446	17604459
chr11_3115	p2	(TC)12	24	17612132	17612155
chr11_3116	p2	(CA)11	22	17618129	17618150
chr11_3117	p3	(CTT)9	27	17619351	17619377
chr11_3118	p2	(CA)6	12	17625734	17625745
chr11_3119	p2	(TC)7	14	17626115	17626128
chr11_3120	c	(TG)24(AG)6	60	17631659	17631718
chr11_3121	p2	(AC)7	14	17654044	17654057
chr11_3122	p2	(AC)6	12	17657973	17657984
chr11_3123	p2	(GT)25	50	17662310	17662359
chr11_3124	c	(TG)7(AG)26	66	17665533	17665598
chr11_3125	p2	(GT)7	14	17665712	17665725
chr11_3126	p2	(AC)16	32	17669326	17669357
chr11_3127	p3	(TCA)6	18	17673304	17673321
chr11_3128	p2	(AC)9	18	17676831	17676848
chr11_3129	p2	(AT)7	14	17685404	17685417
chr11_3130	p4	(CATA)5	20	17697746	17697765
chr11_3131	p2	(AC)9	18	17706408	17706425
chr11_3132	p3	(GAG)5	15	17708530	17708544
chr11_3133	p3	(CAA)5	15	17712445	17712459

chr11_3134	p2	(AC)10	20	17722843	17722862
chr11_3135	p3	(AGC)5	15	17726746	17726760
chr11_3136	p2	(CA)6	12	17730430	17730441
chr11_3137	p2	(AT)6	12	17750189	17750200
chr11_3138	p2	(TC)8	16	17757796	17757811
chr11_3139	p2	(CA)14	28	17760006	17760033
chr11_3140	p2	(AC)39	78	17760745	17760822
chr11_3141	p4	(TATT)5	20	17761541	17761560
chr11_3142	p2	(CA)6	12	17763301	17763312
chr11_3143	p2	(TA)9	18	17766481	17766498
chr11_3144	p2	(TC)6	12	17769191	17769202
chr11_3145	p3	(CTG)6	18	17771577	17771594
chr11_3146	p2	(CT)10	20	17778923	17778942
chr11_3147	c	(TAA)5tattaataagaataattaagatttaaaacaatacc(TAA)5	65	17779443	17779507
chr11_3148	p4	(ATAG)8	32	17781591	17781622
chr11_3149	p2	(GT)9	18	17782595	17782612
chr11_3150	p2	(AC)6	12	17783873	17783884
chr11_3151	c	(AAC)5acagtttctttgaattaa(GT)6	45	17793996	17794040
chr11_3152	p2	(AC)16	32	17811479	17811510
chr11_3153	p2	(GT)15	30	17819456	17819485
chr11_3154	p2	(TG)9	18	17839057	17839074
chr11_3155	p2	(TG)6	12	17851444	17851455
chr11_3156	p3	(TTA)6	18	17858187	17858204
chr11_3157	c	(GGA)5agac(GAG)5	34	17859349	17859382
chr11_3158	p2	(TG)8	16	17869846	17869861
chr11_3159	p2	(CA)12	24	17886523	17886546
chr11_3160	p2	(TA)8	16	17892067	17892082
chr11_3161	p2	(GT)17	34	17892531	17892564
chr11_3162	p3	(TGT)5	15	17900575	17900589
chr11_3163	p3	(TTG)5	15	17924257	17924271
chr11_3164	p2	(GT)8	16	17931372	17931387
chr11_3165	p2	(GT)7	14	17938983	17938996
chr11_3166	c	(GAA)7ggaagcggagt(AGA)9	59	17942061	17942119
chr11_3167	p3	(AGA)7	21	17961730	17961750
chr11_3168	p2	(CA)6	12	17971668	17971679
chr11_3169	p3	(TCA)5	15	17973505	17973519
chr11_3170	p2	(GT)24	48	17992280	17992327
chr11_3171	p2	(TG)6	12	17999960	17999971
chr11_3172	p4	(TTTG)5	20	18005036	18005055
chr11_3173	p3	(GAT)7	21	18046547	18046567
chr11_3174	p2	(TC)9	18	18060381	18060398
chr11_3175	p2	(TG)14	28	18065368	18065395
chr11_3176	p2	(GA)6	12	18068214	18068225
chr11_3177	p2	(CT)6	12	18068333	18068344
chr11_3178	p2	(TG)15	30	18072111	18072140
chr11_3179	p2	(GT)12	24	18079613	18079636
chr11_3180	p2	(TG)8	16	18089984	18089999
chr11_3181	p2	(CA)8	16	18096863	18096878
chr11_3182	p2	(TG)7	14	18102261	18102274
chr11_3183	p2	(GA)6	12	18104884	18104895
chr11_3184	p2	(TC)7	14	18117397	18117410
chr11_3185	c	(CA)21cg(CA)6	56	18117565	18117620

chr11_3186	p2	(TG)9	18	18121329	18121346
chr11_3187	p5	(AAATC)5	25	18137991	18138015
chr11_3188	p2	(TC)6	12	18163273	18163284
chr11_3189	p2	(TG)6	12	18167706	18167717
chr11_3190	c	[illegible]CA)8gctcagcactggtggagcctacttcattaggaagagctga(AC)6	178	18167819	18167996
chr11_3191	p2	(AC)9	18	18173807	18173824
chr11_3192	p4	(CCTG)7	28	18183136	18183163
chr11_3193	p2	(AC)21	42	18187849	18187890
chr11_3194	p3	(TCC)5	15	18192128	18192142
chr11_3195	p2	(CA)6	12	18200829	18200840
chr11_3196	p2	(CA)16	32	18202082	18202113
chr11_3197	p2	(AC)8	16	18206535	18206550
chr11_3198	p3	(GCT)6	18	18237807	18237824
chr11_3199	p3	(AGT)9	27	18267917	18267943
chr11_3200	p2	(CA)9	18	18271363	18271380
chr11_3201	p2	(AC)9	18	18290074	18290091
chr11_3202	p2	(TA)6	12	18309960	18309971
chr11_3203	p2	(GT)8	16	18313077	18313092
chr11_3204	p2	(GT)14	28	18314354	18314381
chr11_3205	p2	(AT)6	12	18315316	18315327
chr11_3206	p3	(ATG)5	15	18318811	18318825
chr11_3207	p2	(TC)13	26	18321142	18321167
chr11_3208	p2	(TA)6	12	18344965	18344976
chr11_3209	p5	(ATAGT)7	35	18352534	18352568
chr11_3210	p2	(TA)6	12	18353835	18353846
chr11_3211	p2	(TG)8	16	18360700	18360715
chr11_3212	p2	(GA)12	24	18365746	18365769
chr11_3213	p2	(TC)6	12	18366939	18366950
chr11_3214	p2	(AT)12	24	18370523	18370546
chr11_3215	c	(AC)6atat(AC)6(AT)10	48	18371732	18371779
chr11_3216	p2	(TG)7	14	18374961	18374974
chr11_3217	p2	(TG)19	38	18376806	18376843
chr11_3218	p3	(ACC)5	15	18381515	18381529
chr11_3219	p2	(TG)13	26	18381645	18381670
chr11_3220	p3	(ATT)6	18	18382904	18382921
chr11_3221	p2	(GT)6	12	18383237	18383248
chr11_3222	p2	(CA)6	12	18383690	18383701
chr11_3223	p2	(AT)6	12	18385369	18385380
chr11_3224	p2	(TG)17	34	18392114	18392147
chr11_3225	p2	(TG)7	14	18399768	18399781
chr11_3226	c	(GT)7ga(GT)8	32	18402257	18402288
chr11_3227	p3	(CAG)5	15	18406025	18406039
chr11_3228	p4	(CCTA)7	28	18413458	18413485
chr11_3229	p3	(GAG)10	30	18441225	18441254
chr11_3230	p2	(TG)11	22	18442529	18442550
chr11_3231	p2	(CA)13	26	18455149	18455174
chr11_3232	p4	(AAAT)5	20	18457456	18457475
chr11_3233	p3	(GAG)5	15	18466617	18466631
chr11_3234	p3	(ACA)5	15	18474293	18474307
chr11_3235	p3	(GAT)6	18	18479456	18479473
chr11_3236	p3	(ATA)5	15	18499132	18499146
chr11_3237	p2	(AC)9	18	18503669	18503686

chr11_3238	p3	(ATG)8	24	18514332	18514355
chr11_3239	p2	(AC)6	12	18531643	18531654
chr11_3240	p2	(AC)6	12	18532789	18532800
chr11_3241	p2	(CA)17	34	18542380	18542413
chr11_3242	p4	(TTTG)5	20	18547277	18547296
chr11_3243	p2	(CA)12	24	18570141	18570164
chr11_3244	p3	(TCA)5	15	18577401	18577415
chr11_3245	p2	(GT)10	20	18578440	18578459
chr11_3246	p5	(AAATC)6	30	18588642	18588671
chr11_3247	p2	(GT)9	18	18592060	18592077
chr11_3248	p2	(GT)18	36	18604763	18604798
chr11_3249	c	TG)10agagagagagacagcaggcagtactctagtggagtaa(TGC)	78	18614238	18614315
chr11_3250	p2	(TA)8	16	18615367	18615382
chr11_3251	p2	(TG)6	12	18615938	18615949
chr11_3252	p3	(TTA)12	36	18619982	18620017
chr11_3253	p2	(TA)7	14	18622764	18622777
chr11_3254	p2	(TG)17	34	18626125	18626158
chr11_3255	p2	(TG)12	24	18646360	18646383
chr11_3256	p2	(GT)6	12	18648558	18648569
chr11_3257	p2	(GT)6	12	18648835	18648846
chr11_3258	p2	(GA)6	12	18656169	18656180
chr11_3259	p2	(TG)9	18	18667628	18667645
chr11_3260	p4	(ATTT)5	20	18671492	18671511
chr11_3261	p3	(AGA)6	18	18677948	18677965
chr11_3262	p2	(AC)7	14	18682674	18682687
chr11_3263	p2	(AT)8	16	18683129	18683144
chr11_3264	p2	(TG)12	24	18689250	18689273
chr11_3265	p3	(AAT)11	33	18694142	18694174
chr11_3266	p4	(AGGT)6	24	18698861	18698884
chr11_3267	p2	(AG)8	16	18701431	18701446
chr11_3268	p2	(AG)6	12	18705429	18705440
chr11_3269	p2	(TA)7	14	18718482	18718495
chr11_3270	p2	(AC)8	16	18736040	18736055
chr11_3271	p2	(AT)7	14	18737554	18737567
chr11_3272	p2	(GT)14	28	18740212	18740239
chr11_3273	p2	(TG)13	26	18741931	18741956
chr11_3274	p2	(GT)6	12	18747305	18747316
chr11_3275	p2	(TG)6	12	18751569	18751580
chr11_3276	p2	(GT)36	72	18755862	18755933
chr11_3277	p2	(CA)7	14	18792396	18792409
chr11_3278	p3	(GAA)5	15	18803920	18803934
chr11_3279	p2	(GT)6	12	18810399	18810410
chr11_3280	p3	(GAA)5	15	18825768	18825782
chr11_3281	p4	(ACCA)5	20	18827305	18827324
chr11_3282	p2	(TG)11	22	18834582	18834603
chr11_3283	p2	(TG)6	12	18841810	18841821
chr11_3284	p2	(AC)9	18	18849999	18850016
chr11_3285	p2	(GT)15	30	18867174	18867203
chr11_3286	c	acgggagaaaggagacacaaagttagtaatgtgtaatggtggcatatagaa	95	18867960	18868054
chr11_3287	p2	(AC)6	12	18886145	18886156
chr11_3288	p4	(TTTG)5	20	18898144	18898163
chr11_3289	p3	(GGA)8	24	18923513	18923536

chr11_3290	p3	(ATT)5	15	18935648	18935662
chr11_3291	p2	(TG)6	12	18945114	18945125
chr11_3292	p2	(TA)6	12	18955480	18955491
chr11_3293	p3	(ACA)5	15	18963768	18963782
chr11_3294	p3	(ACA)7	21	18979140	18979160
chr11_3295	p2	(TG)10	20	18984945	18984964
chr11_3296	p3	(TAA)16	48	18994882	18994929
chr11_3297	p2	(CA)18	36	18997162	18997197
chr11_3298	p2	(GT)12	24	19001305	19001328
chr11_3299	p5	(TGGGG)5	25	19011226	19011250
chr11_3300	p2	(AC)9	18	19015451	19015468
chr11_3301	p2	(AG)12	24	19017836	19017859
chr11_3302	p2	(AC)8	16	19018012	19018027
chr11_3303	p2	(TG)9	18	19026121	19026138
chr11_3304	p2	(GA)10	20	19033399	19033418
chr11_3305	p2	(TG)6	12	19035445	19035456
chr11_3306	p2	(GT)6	12	19047067	19047078
chr11_3307	p2	(CA)17	34	19050701	19050734
chr11_3308	p2	(AC)9	18	19056033	19056050
chr11_3309	p2	(TG)13	26	19078051	19078076
chr11_3310	p5	(TGTAA)5	25	19079075	19079099
chr11_3311	p2	(TA)6	12	19096236	19096247
chr11_3312	p2	(AT)8	16	19100272	19100287
chr11_3313	p2	(AC)8	16	19114072	19114087
chr11_3314	p2	(CA)6	12	19119406	19119417
chr11_3315	p3	(AAT)5	15	19123959	19123973
chr11_3316	p2	(CA)13	26	19143947	19143972
chr11_3317	p2	(AC)10	20	19167060	19167079
chr11_3318	p2	(AC)19	38	19175102	19175139
chr11_3319	p2	(AC)7	14	19179803	19179816
chr11_3320	p3	(GAG)8	24	19190581	19190604
chr11_3321	p2	(TA)8	16	19190811	19190826
chr11_3322	p2	(CA)18	36	19210284	19210319
chr11_3323	p2	(TC)10	20	19210742	19210761
chr11_3324	c	(GT)7gc(GT)10	36	19213705	19213740
chr11_3325	p2	(AG)8	16	19215006	19215021
chr11_3326	p5	(AATCA)5	25	19217472	19217496
chr11_3327	p4	(ATCC)5	20	19220085	19220104
chr11_3328	p2	(AG)6	12	19226765	19226776
chr11_3329	p2	(GA)6	12	19272920	19272931
chr11_3330	p3	(ATA)6	18	19310019	19310036
chr11_3331	p5	(TTGAT)5	25	19344455	19344479
chr11_3332	p2	(AC)6	12	19346389	19346400
chr11_3333	p3	(AGT)5	15	19348984	19348998
chr11_3334	c	(TCA)9tctttctgttcttcttcttcatcc(TCA)5	66	19366069	19366134
chr11_3335	p3	(AAT)8	24	19367726	19367749
chr11_3336	p3	(GTG)5	15	19375745	19375759
chr11_3337	p2	(GT)10	20	19376773	19376792
chr11_3338	p3	(GTG)5	15	19378992	19379006
chr11_3339	c	(GT)21(GA)6	54	19380020	19380073
chr11_3340	c	(GT)19(GA)6	50	19382365	19382414
chr11_3341	p2	(TG)6	12	19390291	19390302

chr11_3342	p2	(CA)6	12	19390440	19390451
chr11_3343	p2	(TG)6	12	19392173	19392184
chr11_3344	c	(GT)13(GA)7c(AG)10	61	19398024	19398084
chr11_3345	p2	(TG)10	20	19403474	19403493
chr11_3346	p2	(TA)8	16	19424292	19424307
chr11_3347	p2	(CA)13	26	19425749	19425774
chr11_3348	p4	(TATG)6	24	19431529	19431552
chr11_3349	p2	(AG)6	12	19433721	19433732
chr11_3350	p2	(AC)6	12	19449550	19449561
chr11_3351	p3	(TAA)5	15	19451503	19451517
chr11_3352	p2	(AG)7	14	19477636	19477649
chr11_3353	p2	(AC)7	14	19479960	19479973
chr11_3354	p2	(AC)12	24	19487223	19487246
chr11_3355	p2	(TA)8	16	19501127	19501142
chr11_3356	p4	(TAAT)6	24	19503750	19503773
chr11_3357	c	(TC)11(AC)9	40	19504432	19504471
chr11_3358	p2	(CA)23	46	19531348	19531393
chr11_3359	p2	(CA)7	14	19533203	19533216
chr11_3360	p2	(CA)7	14	19535179	19535192
chr11_3361	p2	(GA)7	14	19540995	19541008
chr11_3362	p2	(AG)6	12	19568513	19568524
chr11_3363	p5	(ATTTG)5	25	19575774	19575798
chr11_3364	p2	(AC)22	44	19576948	19576991
chr11_3365	p2	(CT)15	30	19582970	19582999
chr11_3366	p2	(AT)7	14	19595282	19595295
chr11_3367	p2	(TG)6	12	19598747	19598758
chr11_3368	p2	(AC)6	12	19601905	19601916
chr11_3369	p2	(TG)6	12	19606827	19606838
chr11_3370	p2	(CA)6	12	19610490	19610501
chr11_3371	p3	(TAA)5	15	19659607	19659621
chr11_3372	p2	(CA)6	12	19660094	19660105
chr11_3373	p2	(TG)6	12	19675522	19675533
chr11_3374	c	8ccttgcgagcaggttggcacactaggaacacagtttggtgctgtgcgtgc	98	19675728	19675825
chr11_3375	p2	(AT)6	12	19679105	19679116
chr11_3376	p4	(TTGT)5	20	19707421	19707440
chr11_3377	c	gatttttagagctgtccactgctgcagtgcatgcccaaaggtagctatcagc	129	19719939	19720067
chr11_3378	p3	(CAG)6	18	19724076	19724093
chr11_3379	c	(GT)6atgtat(TC)6	30	19735333	19735362
chr11_3380	p2	(AC)14	28	19748044	19748071
chr11_3381	p2	(TC)6	12	19757735	19757746
chr11_3382	p2	(AG)11	22	19760164	19760185
chr11_3383	p3	(GGT)6	18	19771325	19771342
chr11_3384	p2	(TA)8	16	19782469	19782484
chr11_3385	p3	(CTA)15	45	19782658	19782702
chr11_3386	p2	(TA)6	12	19789383	19789394
chr11_3387	p2	(GT)6	12	19794030	19794041
chr11_3388	p2	(GT)6	12	19802599	19802610
chr11_3389	c	gcaacttcagaaactgcacaggagcaaatgtcaaaagcatctttccacag	99	19806925	19807023
chr11_3390	p3	(AAC)6	18	19809361	19809378
chr11_3391	c	(TC)8acacacacacat(AC)10	48	19822735	19822782
chr11_3392	p2	(AC)12	24	19825194	19825217
chr11_3393	p4	(AGAC)7	28	19832619	19832646

chr11_3394	p4	(AATA)5	20	19858223	19858242
chr11_3395	p2	(AC)9	18	19859231	19859248
chr11_3396	p2	(TG)6	12	19861064	19861075
chr11_3397	p2	(AT)6	12	19863375	19863386
chr11_3398	c	gagcgtgcacttggatgtgtgttgtcgcaggcatgcaaggttgtctgtgtacg	173	19876125	19876297
chr11_3399	p2	(CA)25	50	19880631	19880680
chr11_3400	p4	(CTAA)5	20	19886240	19886259
chr11_3401	p2	(CA)7	14	19896865	19896878
chr11_3402	c	(TC)7(AC)6	26	19915639	19915664
chr11_3403	p2	(TG)8	16	19923189	19923204
chr11_3404	p2	(GA)6	12	19935010	19935021
chr11_3405	p2	(TG)6	12	19937502	19937513
chr11_3406	p4	(TCCA)5	20	19972138	19972157
chr11_3407	p2	(CA)9	18	19974524	19974541
chr11_3408	p2	(CA)12	24	19993727	19993750
chr11_3409	p2	(GT)29	58	19994692	19994749
chr11_3410	p2	(TG)8	16	20002535	20002550
chr11_3411	p4	(AATA)5	20	20026487	20026506
chr11_3412	p2	(AG)6	12	20031285	20031296
chr11_3413	p2	(TA)8	16	20034976	20034991
chr11_3414	p3	(TAT)5	15	20040044	20040058
chr11_3415	p5	(GATTT)6	30	20047539	20047568
chr11_3416	p3	(CTG)6	18	20049719	20049736
chr11_3417	p4	(GTTT)6	24	20051161	20051184
chr11_3418	p2	(AC)7	14	20053887	20053900
chr11_3419	p2	(GT)26	52	20067390	20067441
chr11_3420	p2	(TG)12	24	20068950	20068973
chr11_3421	p2	(CA)12	24	20070230	20070253
chr11_3422	p2	(TC)7	14	20073887	20073900
chr11_3423	p2	(TC)17	34	20074701	20074734
chr11_3424	p2	(TG)6	12	20083088	20083099
chr11_3425	p2	(GT)8	16	20090437	20090452
chr11_3426	p2	(TG)19	38	20100645	20100682
chr11_3427	c	(AT)8acacatac(AT)7(AC)6atac(AT)7	68	20119782	20119849
chr11_3428	p2	(AC)8	16	20120088	20120103
chr11_3429	p3	(ATT)5	15	20138475	20138489
chr11_3430	p2	(AT)7	14	20146118	20146131
chr11_3431	p3	(CTC)5	15	20150811	20150825
chr11_3432	p2	(CA)47	94	20166758	20166851
chr11_3433	p2	(CA)6	12	20182340	20182351
chr11_3434	p2	(TG)20	40	20198764	20198803
chr11_3435	p2	(AC)11	22	20199744	20199765
chr11_3436	p2	(CA)8	16	20204798	20204813
chr11_3437	p2	(GT)9	18	20207153	20207170
chr11_3438	p2	(TG)10	20	20207936	20207955
chr11_3439	p3	(AGG)5	15	20210968	20210982
chr11_3440	p2	(AC)10	20	20216900	20216919
chr11_3441	p2	(TG)12	24	20221156	20221179
chr11_3442	p2	(TG)9	18	20229844	20229861
chr11_3443	p2	(CA)7	14	20238612	20238625
chr11_3444	p4	(TTAT)5	20	20239111	20239130
chr11_3445	p2	(GA)6	12	20251654	20251665

chr11_3446	p2	(GA)6	12	20254280	20254291
chr11_3447	p2	(TA)6	12	20255460	20255471
chr11_3448	p2	(GT)6	12	20255600	20255611
chr11_3449	p4	(TTAT)6	24	20259441	20259464
chr11_3450	p2	(CA)6	12	20262398	20262409
chr11_3451	p2	(AC)9	18	20265578	20265595
chr11_3452	p2	(GT)7	14	20288539	20288552
chr11_3453	p3	(GAT)5	15	20295950	20295964
chr11_3454	p2	(TG)12	24	20296135	20296158
chr11_3455	p2	(AC)15	30	20297238	20297267
chr11_3456	p2	(CA)6	12	20297558	20297569
chr11_3457	p2	(GA)13	26	20298299	20298324
chr11_3458	p2	(TG)8	16	20298890	20298905
chr11_3459	p2	(GT)8	16	20300092	20300107
chr11_3460	p3	(GGA)6	18	20300280	20300297
chr11_3461	p2	(TG)6	12	20302022	20302033
chr11_3462	p2	(CA)8	16	20305025	20305040
chr11_3463	p2	(TC)6	12	20307206	20307217
chr11_3464	p2	(AT)10	20	20310743	20310762
chr11_3465	p2	(AC)19	38	20314395	20314432
chr11_3466	p2	(GT)6	12	20315567	20315578
chr11_3467	c	(TGTA)6(TG)13	50	20323667	20323716
chr11_3468	p2	(TC)7	14	20330245	20330258
chr11_3469	p3	(AGC)5	15	20334934	20334948
chr11_3470	p2	(TA)6	12	20335633	20335644
chr11_3471	p3	(TCC)8	24	20337600	20337623
chr11_3472	p2	(GC)6	12	20343676	20343687
chr11_3473	p4	(TCCA)6	24	20359530	20359553
chr11_3474	p2	(GT)9	18	20366197	20366214
chr11_3475	p2	(TG)8	16	20372194	20372209
chr11_3476	p3	(CAG)5	15	20372878	20372892
chr11_3477	p2	(AG)6	12	20383195	20383206
chr11_3478	p3	(CAT)13	39	20404297	20404335
chr11_3479	p2	(TC)6	12	20407429	20407440
chr11_3480	p3	(GTG)6	18	20422084	20422101
chr11_3481	p2	(TG)6	12	20422222	20422233
chr11_3482	p2	(AC)9	18	20426290	20426307
chr11_3483	p2	(AT)6	12	20439286	20439297
chr11_3484	p2	(CT)13	26	20465122	20465147
chr11_3485	p3	(CTC)5	15	20468152	20468166
chr11_3486	p2	(AG)8	16	20472040	20472055
chr11_3487	p2	(AG)17	34	20475706	20475739
chr11_3488	c	(GT)23(GA)6	58	20478887	20478944
chr11_3489	p2	(TG)6	12	20479466	20479477
chr11_3490	p2	(AT)9	18	20484282	20484299
chr11_3491	p2	(GT)6	12	20490042	20490053
chr11_3492	p2	(TG)6	12	20519798	20519809
chr11_3493	p3	(TAT)7	21	20540546	20540566
chr11_3494	p2	(AC)6	12	20552827	20552838
chr11_3495	p2	(AC)16	32	20601138	20601169
chr11_3496	p2	(GT)16	32	20610965	20610996
chr11_3497	p2	(TG)10	20	20617505	20617524

chr11_3498	p2	(GA)11	22	20620323	20620344
chr11_3499	p2	(GT)18	36	20627440	20627475
chr11_3500	p2	(AC)6	12	20629222	20629233
chr11_3501	c	(TG)10(AG)11(TG)6agcgagagag(AGA)5	79	20630649	20630727
chr11_3502	p2	(AC)6	12	20632367	20632378
chr11_3503	p2	(CT)15	30	20635972	20636001
chr11_3504	p4	(AAAC)5	20	20645622	20645641
chr11_3505	p2	(AC)14	28	20660520	20660547
chr11_3506	p3	(ACA)6	18	20683409	20683426
chr11_3507	p2	(CA)9	18	20690156	20690173
chr11_3508	p2	(GA)8	16	20710805	20710820
chr11_3509	p2	(TG)31	62	20710991	20711052
chr11_3510	p2	(TG)10	20	20716842	20716861
chr11_3511	p2	(CA)7	14	20722547	20722560
chr11_3512	p2	(TG)10	20	20725262	20725281
chr11_3513	p2	(TG)12	24	20725636	20725659
chr11_3514	p3	(GGA)5	15	20730483	20730497
chr11_3515	c	(CT)6(CA)8	28	20730757	20730784
chr11_3516	c	atgcatggagaagaagagaaggcaggagacagtgagacaatgtgtgcatg	104	20735248	20735351
chr11_3517	p2	(TG)9	18	20735870	20735887
chr11_3518	p2	(GT)11	22	20737445	20737466
chr11_3519	p2	(AC)15	30	20737722	20737751
chr11_3520	p3	(GGT)7	21	20738958	20738978
chr11_3521	p3	(GGT)7	21	20740155	20740175
chr11_3522	p2	(CA)15	30	20745846	20745875
chr11_3523	p2	(TG)10	20	20750302	20750321
chr11_3524	p2	(GT)9	18	20751243	20751260
chr11_3525	p2	(TG)10	20	20753742	20753761
chr11_3526	p2	(TG)13	26	20766119	20766144
chr11_3527	p2	(GT)7	14	20767061	20767074
chr11_3528	p4	(ATGG)5	20	20772070	20772089
chr11_3529	p2	(CT)6	12	20796579	20796590
chr11_3530	p2	(CT)7	14	20798420	20798433
chr11_3531	p2	(TG)7	14	20800196	20800209
chr11_3532	p2	(AC)22	44	20809914	20809957
chr11_3533	p3	(AAC)5	15	20844800	20844814
chr11_3534	c	(TC)14(AC)17	62	20891082	20891143
chr11_3535	p2	(TG)20	40	20896138	20896177
chr11_3536	p3	(CAA)5	15	20897224	20897238
chr11_3537	p2	(CA)7	14	20924954	20924967
chr11_3538	p2	(TA)11	22	20932260	20932281
chr11_3539	p4	(TTTA)6	24	20933397	20933420
chr11_3540	p2	(TA)7	14	20940844	20940857
chr11_3541	p2	(AT)9	18	20944033	20944050
chr11_3542	p2	(TG)7	14	20945536	20945549
chr11_3543	p2	(CA)10	20	20952480	20952499
chr11_3544	p2	(TC)7	14	20967226	20967239
chr11_3545	p2	(AC)9	18	20978500	20978517
chr11_3546	p2	(GT)6	12	20982712	20982723
chr11_3547	p2	(TC)10	20	20987904	20987923
chr11_3548	p2	(TC)6	12	21001404	21001415
chr11_3549	c	agatgaggaaaaagaagtagaaaacctagttgaagaggcagatgaggag	124	21021141	21021264

chr11_3550	p3	(TCA)5	15	21055433	21055447
chr11_3551	p2	(GA)6	12	21063695	21063706
chr11_3552	p2	(GT)7	14	21065908	21065921
chr11_3553	p2	(GT)7	14	21068734	21068747
chr11_3554	p2	(GT)6	12	21068913	21068924
chr11_3555	p2	(GT)7	14	21070731	21070744
chr11_3556	p2	(GT)6	12	21070910	21070921
chr11_3557	p3	(TAC)6	18	21076217	21076234
chr11_3558	p2	(AC)11	22	21078210	21078231
chr11_3559	p4	(CACT)5	20	21078588	21078607
chr11_3560	p2	(AC)11	22	21097632	21097653
chr11_3561	p2	(GT)7	14	21107974	21107987
chr11_3562	p3	(ATT)5	15	21118593	21118607
chr11_3563	p2	(CA)9	18	21130415	21130432
chr11_3564	p2	(TC)6	12	21132050	21132061
chr11_3565	p2	(TG)6	12	21132210	21132221
chr11_3566	p2	(AG)7	14	21134559	21134572
chr11_3567	p2	(CA)20	40	21137474	21137513
chr11_3568	p2	(GT)11	22	21140377	21140398
chr11_3569	c	(GATA)5gag(AGAT)10	63	21141280	21141342
chr11_3570	p2	(CT)8	16	21142104	21142119
chr11_3571	p2	(TG)11	22	21144716	21144737
chr11_3572	p2	(AC)6	12	21148713	21148724
chr11_3573	p3	(CAA)7	21	21150789	21150809
chr11_3574	p2	(TC)6	12	21160362	21160373
chr11_3575	p2	(GT)14	28	21179709	21179736
chr11_3576	p2	(GA)8	16	21186766	21186781
chr11_3577	p2	(TA)8	16	21190673	21190688
chr11_3578	p3	(GCA)7	21	21197145	21197165
chr11_3579	p2	(TG)37	74	21198160	21198233
chr11_3580	p2	(TG)24	48	21202034	21202081
chr11_3581	p2	(TG)33	66	21202399	21202464
chr11_3582	p3	(CTC)15	45	21216940	21216984
chr11_3583	p2	(TG)6	12	21229332	21229343
chr11_3584	p3	(TTG)7	21	21229532	21229552
chr11_3585	p2	(TG)6	12	21235687	21235698
chr11_3586	p2	(CA)7	14	21245976	21245989
chr11_3587	p2	(CT)10	20	21247483	21247502
chr11_3588	p3	(TGA)5	15	21254958	21254972
chr11_3589	p3	(ATA)6	18	21263689	21263706
chr11_3590	p2	(TG)8	16	21271930	21271945
chr11_3591	p2	(TG)6	12	21278832	21278843
chr11_3592	p2	(CA)8	16	21288236	21288251
chr11_3593	p2	(TG)6	12	21300010	21300021
chr11_3594	p2	(AC)9	18	21301608	21301625
chr11_3595	p3	(AAT)5	15	21304218	21304232
chr11_3596	p5	(CAAAT)5	25	21305739	21305763
chr11_3597	p2	(AC)17	34	21309464	21309497
chr11_3598	p2	(AC)11	22	21313928	21313949
chr11_3599	p2	(TG)14	28	21316845	21316872
chr11_3600	p2	(TG)7	14	21317148	21317161
chr11_3601	p3	(AAT)5	15	21324801	21324815

chr11_3602	c	ıtatcaccagataaatccattgctgctccctctgtgaatgtgca(TG)8atga	172	21325129	21325300
chr11_3603	p2	(AC)8	16	21327302	21327317
chr11_3604	p2	(TC)8	16	21328452	21328467
chr11_3605	p2	(TC)10	20	21329419	21329438
chr11_3606	p5	(AAATC)5	25	21335728	21335752
chr11_3607	p3	(TAT)5	15	21338461	21338475
chr11_3608	p2	(AC)12	24	21344535	21344558
chr11_3609	p2	(CA)11	22	21353822	21353843
chr11_3610	p2	(TG)7	14	21358368	21358381
chr11_3611	p2	(AC)18	36	21360146	21360181
chr11_3612	c	(TG)6cctttgtattaccaaaacctgggc(GT)11	58	21368788	21368845
chr11_3613	p3	(AAC)5	15	21369694	21369708
chr11_3614	p3	(AGG)5	15	21373600	21373614
chr11_3615	p2	(TC)6	12	21373821	21373832
chr11_3616	p3	(ACA)5	15	21386952	21386966
chr11_3617	c	;)23tctttgcaattggaactcacactgtctgtgtcaggctcacacacaa(A	104	21388999	21389102
chr11_3618	p2	(GT)9	18	21392054	21392071
chr11_3619	p2	(AC)6	12	21395926	21395937
chr11_3620	p4	(ATAG)6	24	21401042	21401065
chr11_3621	p2	(GT)10	20	21404812	21404831
chr11_3622	c	(TC)7ccaccccatttc(TA)9(TG)19	82	21406269	21406350
chr11_3623	p2	(TC)9	18	21406467	21406484
chr11_3624	p3	(AAT)8	24	21407044	21407067
chr11_3625	p3	(ATA)5	15	21407405	21407419
chr11_3626	p3	(CAT)5	15	21413713	21413727
chr11_3627	p2	(CA)13	26	21414930	21414955
chr11_3628	p2	(AG)10	20	21422470	21422489
chr11_3629	p2	(AC)7	14	21424197	21424210
chr11_3630	p2	(AC)16	32	21428419	21428450
chr11_3631	p3	(CCT)7	21	21430590	21430610
chr11_3632	p2	(AC)11	22	21434626	21434647
chr11_3633	p2	(TA)6	12	21443042	21443053
chr11_3634	p3	(ATA)5	15	21453627	21453641
chr11_3635	p3	(TGG)7	21	21457312	21457332
chr11_3636	p2	(TG)13	26	21458505	21458530
chr11_3637	p2	(TG)9	18	21462631	21462648
chr11_3638	c	(AAT)7(TAT)5	36	21484537	21484572
chr11_3639	p2	(AC)6	12	21487944	21487955
chr11_3640	p2	(AT)6	12	21490261	21490272
chr11_3641	p2	(TG)6	12	21498304	21498315
chr11_3642	p3	(GGT)5	15	21499850	21499864
chr11_3643	p2	(GT)12	24	21503397	21503420
chr11_3644	p2	(AC)15	30	21506385	21506414
chr11_3645	p2	(CA)9	18	21507453	21507470
chr11_3646	p2	(CT)6	12	21509042	21509053
chr11_3647	p2	(AC)16	32	21510362	21510393
chr11_3648	p2	(TA)6	12	21511076	21511087
chr11_3649	p5	(AAAGC)5	25	21511484	21511508
chr11_3650	c	(GT)8atgt(GA)6	32	21512153	21512184
chr11_3651	p2	(TG)6	12	21521009	21521020
chr11_3652	p2	(GA)6	12	21529310	21529321
chr11_3653	p2	(CA)10	20	21548526	21548545

chr11_3654	p4	(ATTG)5	20	21551007	21551026
chr11_3655	p2	(TG)15	30	21582693	21582722
chr11_3656	p5	(TGATT)5	25	21585233	21585257
chr11_3657	p3	(CAT)6	18	21618976	21618993
chr11_3658	p3	(ATT)6	18	21619866	21619883
chr11_3659	p2	(TG)17	34	21625899	21625932
chr11_3660	p2	(TG)17	34	21629356	21629389
chr11_3661	p3	(GAT)6	18	21633703	21633720
chr11_3662	p2	(CA)14	28	21650731	21650758
chr11_3663	p3	(AAT)5	15	21663649	21663663
chr11_3664	c	(TA)11cacacatat(AC)11	53	21667314	21667366
chr11_3665	p2	(TC)7	14	21668637	21668650
chr11_3666	p3	(AAC)5	15	21724330	21724344
chr11_3667	p2	(TG)6	12	21737406	21737417
chr11_3668	p2	(AC)11	22	21760801	21760822
chr11_3669	p2	(CT)7	14	21765526	21765539
chr11_3670	p3	(AGC)6	18	21767705	21767722
chr11_3671	p3	(CAT)11	33	21771782	21771814
chr11_3672	p2	(TC)7	14	21782497	21782510
chr11_3673	c	(TG)10tatatg(TA)6	38	21782683	21782720
chr11_3674	p2	(TG)6	12	21790114	21790125
chr11_3675	p3	(GCA)5	15	21790794	21790808
chr11_3676	p2	(CT)6	12	21791003	21791014
chr11_3677	p2	(GT)10	20	21795822	21795841
chr11_3678	p2	(TG)11	22	21810664	21810685
chr11_3679	p2	(GA)8	16	21814437	21814452
chr11_3680	p2	(TG)6	12	21827277	21827288
chr11_3681	p2	(GA)8	16	21827583	21827598
chr11_3682	p2	(AC)7	14	21859155	21859168
chr11_3683	p2	(AC)8	16	21860744	21860759
chr11_3684	p2	(AG)11	22	21867913	21867934
chr11_3685	p2	(CA)20	40	21870657	21870696
chr11_3686	p2	(AC)8	16	21870963	21870978
chr11_3687	p4	(AGAT)15	60	21877201	21877260
chr11_3688	p3	(TTC)5	15	21885268	21885282
chr11_3689	p2	(TC)11	22	21891661	21891682
chr11_3690	p2	(CA)6	12	21892097	21892108
chr11_3691	p3	(TCT)13	39	21923631	21923669
chr11_3692	p3	(TTG)6	18	21934340	21934357
chr11_3693	p2	(TG)7	14	21942319	21942332
chr11_3694	p4	(ATCC)6	24	21965123	21965146
chr11_3695	p3	(ACT)10	30	21983021	21983050
chr11_3696	p2	(CA)18	36	21990160	21990195
chr11_3697	p2	(TA)8	16	22006203	22006218
chr11_3698	p2	(CA)13	26	22007485	22007510
chr11_3699	p2	(TG)8	16	22016484	22016499
chr11_3700	p2	(AC)8	16	22038525	22038540
chr11_3701	p4	(GTCT)9	36	22046107	22046142
chr11_3702	p2	(GT)6	12	22048949	22048960
chr11_3703	p5	(AAATC)6	30	22049881	22049910
chr11_3704	p2	(TG)8	16	22059856	22059871
chr11_3705	p2	(TG)6	12	22065909	22065920

chr11_3706	p3	(TAT)5	15	22067353	22067367
chr11_3707	c	A)6ggggaggagagggaagggaagtgaagagaagagaagagaat(GA	101	22080338	22080438
chr11_3708	p2	(TG)6	12	22116266	22116277
chr11_3709	p3	(ATA)5	15	22116387	22116401
chr11_3710	p2	(TG)7	14	22126130	22126143
chr11_3711	p2	(TG)6	12	22128552	22128563
chr11_3712	p2	(AG)6	12	22141883	22141894
chr11_3713	p4	(AACA)7	28	22148423	22148450
chr11_3714	p2	(CA)8	16	22149926	22149941
chr11_3715	p2	(TG)7	14	22158692	22158705
chr11_3716	p2	(CA)12	24	22166192	22166215
chr11_3717	p3	(CAG)11	33	22180359	22180391
chr11_3718	p2	(GT)13	26	22181119	22181144
chr11_3719	p2	(GT)10	20	22191034	22191053
chr11_3720	p2	(TC)6	12	22198600	22198611
chr11_3721	p3	(ATA)5	15	22198802	22198816
chr11_3722	p2	(TG)9	18	22214559	22214576
chr11_3723	p2	(AC)11	22	22217885	22217906
chr11_3724	p2	(AC)7	14	22218287	22218300
chr11_3725	p2	(GT)10	20	22219712	22219731
chr11_3726	p2	(AT)9	18	22222372	22222389
chr11_3727	c	(CT)8(CA)12	40	22248282	22248321
chr11_3728	p2	(CA)10	20	22258992	22259011
chr11_3729	p3	(AAG)5	15	22265078	22265092
chr11_3730	p5	(AAATC)5	25	22273591	22273615
chr11_3731	p2	(CT)6	12	22275889	22275900
chr11_3732	p3	(TAA)6	18	22276850	22276867
chr11_3733	p2	(CA)28	56	22299078	22299133
chr11_3734	p2	(TG)12	24	22322324	22322347
chr11_3735	p2	(TA)7	14	22331336	22331349
chr11_3736	p2	(AT)8	16	22338875	22338890
chr11_3737	p2	(TG)8	16	22350688	22350703
chr11_3738	p2	(TG)6	12	22351548	22351559
chr11_3739	p2	(GT)16	32	22370033	22370064
chr11_3740	c	gggagagaggagacacaaagttagtaatgtgtaatggtggcatatagaagc	106	22370946	22371051
chr11_3741	p2	(GA)8	16	22373778	22373793
chr11_3742	p3	(CAA)7	21	22383370	22383390
chr11_3743	p2	(AC)7	14	22422530	22422543
chr11_3744	p4	(TCCA)6	24	22430169	22430192
chr11_3745	p2	(AC)16	32	22442407	22442438
chr11_3746	p2	(TC)6	12	22447697	22447708
chr11_3747	p2	(AC)9	18	22452265	22452282
chr11_3748	p2	(TG)10	20	22454487	22454506
chr11_3749	p2	(TG)6	12	22459976	22459987
chr11_3750	p2	(AC)10	20	22463720	22463739
chr11_3751	p2	(AC)29	58	22463846	22463903
chr11_3752	p4	(TCCA)7	28	22469061	22469088
chr11_3753	p2	(AC)14	28	22476072	22476099
chr11_3754	p2	(CT)6	12	22480243	22480254
chr11_3755	p3	(GAA)5	15	22497642	22497656
chr11_3756	p3	(AAT)5	15	22502094	22502108
chr11_3757	c	(CA)6aactttaccggctcacattagtgagtta(GT)11	62	22510977	22511038

chr11_3758	p2	(AC)17	34	22530078	22530111
chr11_3759	p2	(GT)6	12	22533286	22533297
chr11_3760	p4	(CCAT)6	24	22576459	22576482
chr11_3761	p3	(ATG)5	15	22578265	22578279
chr11_3762	p4	(CCTA)5	20	22590414	22590433
chr11_3763	p2	(AC)14	28	22595146	22595173
chr11_3764	p2	(CA)13	26	22607514	22607539
chr11_3765	p2	(TG)18	36	22618023	22618058
chr11_3766	p2	(CA)18	36	22618219	22618254
chr11_3767	p2	(TG)9	18	22630231	22630248
chr11_3768	p2	(CA)24	48	22635986	22636033
chr11_3769	p2	(CA)7	14	22647716	22647729
chr11_3770	p2	(GT)18	36	22648818	22648853
chr11_3771	p2	(TG)17	34	22649116	22649149
chr11_3772	p5	(TCAAA)5	25	22651019	22651043
chr11_3773	p2	(AC)34	68	22654230	22654297
chr11_3774	p2	(AC)7	14	22654409	22654422
chr11_3775	c	cat(CA)11agtccatccctccctgttcaccaagaaatcatccctctctcg	190	22664110	22664299
chr11_3776	p2	(CA)21	42	22670757	22670798
chr11_3777	p2	(TG)13	26	22676054	22676079
chr11_3778	p3	(GAT)10	30	22678801	22678830
chr11_3779	p2	(GA)6	12	22679591	22679602
chr11_3780	p2	(CA)15	30	22684727	22684756
chr11_3781	p2	(AC)8	16	22686395	22686410
chr11_3782	p2	(GT)8	16	22689309	22689324
chr11_3783	p2	(TC)6	12	22693052	22693063
chr11_3784	p2	(GT)6	12	22693629	22693640
chr11_3785	p2	(CA)10	20	22695269	22695288
chr11_3786	p2	(CA)10	20	22696613	22696632
chr11_3787	c	(GT)12gcgc(GT)6	40	22701902	22701941
chr11_3788	p2	(CA)6	12	22717735	22717746
chr11_3789	p2	(CA)15	30	22723964	22723993
chr11_3790	p2	(AC)6	12	22735639	22735650
chr11_3791	p2	(TC)8	16	22743431	22743446
chr11_3792	p2	(CA)7	14	22744075	22744088
chr11_3793	p2	(AC)13	26	22752325	22752350
chr11_3794	p2	(TG)11	22	22754045	22754066
chr11_3795	p2	(AC)16	32	22754884	22754915
chr11_3796	p2	(AC)9	18	22768164	22768181
chr11_3797	p2	(AC)16	32	22768682	22768713
chr11_3798	p2	(TA)7	14	22791373	22791386
chr11_3799	p2	(CA)8	16	22803918	22803933
chr11_3800	p2	(CT)6	12	22815279	22815290
chr11_3801	p4	(AAAC)5	20	22817498	22817517
chr11_3802	p2	(GT)8	16	22822016	22822031
chr11_3803	p2	(GT)21	42	22822171	22822212
chr11_3804	p2	(AC)15	30	22823803	22823832
chr11_3805	p2	(AC)25	50	22824823	22824872
chr11_3806	p2	(AG)14	28	22824980	22825007
chr11_3807	p2	(CT)9	18	22825288	22825305
chr11_3808	p2	(TG)6	12	22830027	22830038
chr11_3809	p2	(TG)6	12	22844636	22844647

chr11_3810	p2	(CA)20	40	22847064	22847103
chr11_3811	p2	(TG)10	20	22854108	22854127
chr11_3812	p2	(TA)6	12	22871391	22871402
chr11_3813	p3	(AAC)8	24	22875735	22875758
chr11_3814	p3	(AAT)5	15	22877093	22877107
chr11_3815	p2	(CA)13	26	22878353	22878378
chr11_3816	p5	(AGAAG)5	25	22880301	22880325
chr11_3817	p2	(TG)7	14	22888736	22888749
chr11_3818	p2	(AC)14	28	22890180	22890207
chr11_3819	p2	(TG)12	24	22890655	22890678
chr11_3820	p2	(TG)7	14	22890869	22890882
chr11_3821	p2	(GT)6	12	22901657	22901668
chr11_3822	c	(AG)8acatccacacaatccattaaa(AC)16	69	22905672	22905740
chr11_3823	p2	(AT)6	12	22909305	22909316
chr11_3824	c	(TC)9(AC)19	56	22919731	22919786
chr11_3825	p2	(CA)8	16	22927957	22927972
chr11_3826	c	(GT)7gagagagagac(AG)12acagagacagaa(AG)6	73	22930109	22930181
chr11_3827	p2	(CA)7	14	22933107	22933120
chr11_3828	p2	(AC)16	32	22935667	22935698
chr11_3829	p2	(AC)6	12	22936075	22936086
chr11_3830	p2	(GT)8	16	22936222	22936237
chr11_3831	p2	(TG)21	42	22942780	22942821
chr11_3832	p2	(TG)14	28	22949205	22949232
chr11_3833	p2	(CA)13	26	22951385	22951410
chr11_3834	p4	(AAAT)5	20	22952626	22952645
chr11_3835	p2	(CA)15	30	22954261	22954290
chr11_3836	p3	(AAT)7	21	22956414	22956434
chr11_3837	p2	(CT)6	12	22969929	22969940
chr11_3838	p3	(TAT)6	18	22982637	22982654
chr11_3839	c	(TG)9tacatttgctgtgtctcattttgaatgta(TG)9	65	22983804	22983868
chr11_3840	p2	(CA)18	36	22988808	22988843
chr11_3841	p2	(GT)7	14	22997136	22997149
chr11_3842	p3	(AAG)5	15	23001038	23001052
chr11_3843	p2	(AC)8	16	23015540	23015555
chr11_3844	p2	(GT)18	36	23016571	23016606
chr11_3845	p2	(AC)9	18	23017868	23017885
chr11_3846	p3	(CTC)7	21	23019221	23019241
chr11_3847	p2	(AC)17	34	23021133	23021166
chr11_3848	p2	(AC)44	88	23021986	23022073
chr11_3849	p2	(GT)7	14	23024786	23024799
chr11_3850	p2	(GT)8	16	23029289	23029304
chr11_3851	p2	(GT)20	40	23033251	23033290
chr11_3852	p2	(CA)6	12	23035892	23035903
chr11_3853	p2	(AT)6	12	23048422	23048433
chr11_3854	p2	(CA)21	42	23048587	23048628
chr11_3855	p2	(TG)11	22	23050667	23050688
chr11_3856	p2	(CA)6	12	23059677	23059688
chr11_3857	p3	(TTG)10	30	23061091	23061120
chr11_3858	c	[illegible]T)7tagtgatacaaataaagcatgaaaaatgtgtatgtgtatatag(GT)[illegible]	87	23061426	23061512
chr11_3859	p2	(CA)13	26	23065943	23065968
chr11_3860	p4	(TGTT)5	20	23069489	23069508
chr11_3861	p4	(TTTG)6	24	23081229	23081252

chr11_3862	c	(GT)16(GA)9	50	23086907	23086956
chr11_3863	p2	(TG)6	12	23091240	23091251
chr11_3864	p3	(AAC)5	15	23097707	23097721
chr11_3865	p2	(CA)39	78	23098613	23098690
chr11_3866	p2	(AC)8	16	23099540	23099555
chr11_3867	p2	(AC)18	36	23101186	23101221
chr11_3868	p3	(TGT)5	15	23102033	23102047
chr11_3869	p3	(GCT)5	15	23103785	23103799
chr11_3870	p2	(TG)30	60	23106894	23106953
chr11_3871	p2	(AC)7	14	23133424	23133437
chr11_3872	p2	(GT)15	30	23137975	23138004
chr11_3873	p2	(CT)6	12	23140391	23140402
chr11_3874	p2	(CA)9	18	23143561	23143578
chr11_3875	c	(CT)8(CA)18	52	23143840	23143891
chr11_3876	p2	(TG)10	20	23164103	23164122
chr11_3877	c	(GT)14t(TG)7	43	23166653	23166695
chr11_3878	p2	(GT)12	24	23181716	23181739
chr11_3879	c	ctcaaatg(CA)18tacagagagaagacaaatgaccatgtttgtaatagtg	297	23181857	23182153
chr11_3880	p2	(CA)6	12	23183214	23183225
chr11_3881	p5	(AAATC)5	25	23186203	23186227
chr11_3882	p3	(AAT)5	15	23191099	23191113
chr11_3883	p2	(CA)20	40	23191393	23191432
chr11_3884	p2	(TG)6	12	23196446	23196457
chr11_3885	p2	(CA)21	42	23198286	23198327
chr11_3886	p2	(TG)14	28	23207685	23207712
chr11_3887	p3	(AAT)5	15	23218830	23218844
chr11_3888	p3	(ATA)6	18	23218956	23218973
chr11_3889	p2	(GT)10	20	23219156	23219175
chr11_3890	p2	(AT)6	12	23220435	23220446
chr11_3891	c	lagtatgtgttgaattgctgagcaggttcatcgagcaggttcataaatcttgca	109	23223835	23223943
chr11_3892	p2	(GA)6	12	23226730	23226741
chr11_3893	p2	(GT)9	18	23228101	23228118
chr11_3894	p2	(AC)6	12	23234210	23234221
chr11_3895	c	(CA)8t(AC)6	29	23235740	23235768
chr11_3896	p2	(CA)7	14	23248886	23248899
chr11_3897	c	cga(AC)7cctcc(CT)7tccctgctcttgctctgtcacaaacacacaca	109	23250541	23250649
chr11_3898	p2	(GA)12	24	23253124	23253147
chr11_3899	p2	(AC)11	22	23255888	23255909
chr11_3900	p4	(TTAT)5	20	23259507	23259526
chr11_3901	p2	(AC)6	12	23262229	23262240
chr11_3902	p2	(CA)6	12	23265106	23265117
chr11_3903	p2	(TG)6	12	23266171	23266182
chr11_3904	p2	(TG)18	36	23269051	23269086
chr11_3905	c	ggggaaatgtcagtgtcattctattatgtatcaaggcaaaacacagaagggt	99	23276339	23276437
chr11_3906	p2	(CA)6	12	23291197	23291208
chr11_3907	p2	(GT)18	36	23297598	23297633
chr11_3908	p2	(GT)15	30	23308213	23308242
chr11_3909	p2	(TG)8	16	23315561	23315576
chr11_3910	p2	(GT)25	50	23318758	23318807
chr11_3911	p2	(AG)6	12	23319556	23319567
chr11_3912	p2	(TG)15	30	23322213	23322242
chr11_3913	p2	(CT)6	12	23322455	23322466

chr11_3914	p2	(CA)12	24	23322924	23322947
chr11_3915	p2	(GA)10	20	23325580	23325599
chr11_3916	p3	(TAT)5	15	23346068	23346082
chr11_3917	p3	(TAT)5	15	23348243	23348257
chr11_3918	p2	(TA)9	18	23349495	23349512
chr11_3919	p2	(AC)10	20	23354711	23354730
chr11_3920	p2	(TG)7	14	23357084	23357097
chr11_3921	p2	(GT)7	14	23361591	23361604
chr11_3922	p2	(GT)8	16	23362143	23362158
chr11_3923	p2	(GT)18	36	23387263	23387298
chr11_3924	p2	(GT)24	48	23390624	23390671
chr11_3925	p2	(TG)6	12	23401834	23401845
chr11_3926	p3	(TGC)6	18	23404003	23404020
chr11_3927	p3	(AGT)5	15	23410324	23410338
chr11_3928	p2	(AC)11	22	23411266	23411287
chr11_3929	p2	(CA)6	12	23411408	23411419
chr11_3930	p2	(CA)17	34	23412474	23412507
chr11_3931	p2	(AC)15	30	23417916	23417945
chr11_3932	p2	(TG)6	12	23418968	23418979
chr11_3933	p2	(GT)19	38	23421535	23421572
chr11_3934	p2	(CA)6	12	23425362	23425373
chr11_3935	p2	(CA)9	18	23425689	23425706
chr11_3936	p3	(TTA)5	15	23426264	23426278
chr11_3937	p2	(TG)8	16	23430131	23430146
chr11_3938	p3	(ACA)5	15	23431914	23431928
chr11_3939	p2	(TA)7	14	23436088	23436101
chr11_3940	p2	(CA)7	14	23437469	23437482
chr11_3941	c	7cccctcccccgtaatgtagacacacactcctccctgtctcctcccaca(A	112	23438179	23438290
chr11_3942	p2	(AC)11	22	23439144	23439165
chr11_3943	p2	(AC)17	34	23443763	23443796
chr11_3944	p2	(CA)28	56	23444408	23444463
chr11_3945	p2	(GT)6	12	23446995	23447006
chr11_3946	p2	(CA)22	44	23447172	23447215
chr11_3947	p2	(AC)11	22	23465188	23465209
chr11_3948	c	ggacgagatcaccagagaggaagccagcaaaatatgacaaggtgtgaca	138	23465833	23465970
chr11_3949	p3	(TCC)5	15	23468860	23468874
chr11_3950	c	(TG)11tatatacac(AT)6	43	23472860	23472902
chr11_3951	c	acaagacaactgacaggttctaatagctctcgaacacctccatctgaacatg	102	23473336	23473437
chr11_3952	p2	(CA)10	20	23481172	23481191
chr11_3953	p2	(CA)10	20	23485208	23485227
chr11_3954	p2	(TG)10	20	23485614	23485633
chr11_3955	c	gtgggaacgtggatgaaggaaacaggactgattttgtatctgagagggagg	85	23487149	23487233
chr11_3956	p4	(AGAC)10	40	23487907	23487946
chr11_3957	p2	(CA)6	12	23488237	23488248
chr11_3958	p2	(AC)10	20	23490414	23490433
chr11_3959	c	igctggtatgcatccatacttcctgaatttttcattttcgctggggctttggttatt	113	23492830	23492942
chr11_3960	p2	(GT)6	12	23493365	23493376
chr11_3961	p2	(GA)8	16	23497887	23497902
chr11_3962	p2	(TG)7	14	23506645	23506658
chr11_3963	c	(TG)7ta(TG)6	28	23506799	23506826
chr11_3964	c	ıtctgactgtgcgtaggcatgagtgagtggtacgtgagtgactgattaggaa	77	23507521	23507597
chr11_3965	p2	(TG)14	28	23509050	23509077

chr11_3966	p2	(GT)9	18	23509274	23509291
chr11_3967	p2	(TC)7	14	23509419	23509432
chr11_3968	p2	(AC)11	22	23513653	23513674
chr11_3969	p2	(CA)10	20	23513841	23513860
chr11_3970	p2	(AC)28	56	23514022	23514077
chr11_3971	p2	(CA)7	14	23514212	23514225
chr11_3972	p2	(CA)15	30	23524394	23524423
chr11_3973	p2	(TG)7	14	23528994	23529007
chr11_3974	c	gctgcagtagtaaggccgtcaggcagcctgccgcttcctgtctgctttaattg	155	23529495	23529649
chr11_3975	c	ctgcagtagtaaggccgtcaggcagcctgccgcttcctgtctgctttaattgt	147	23530132	23530278
chr11_3976	p2	(TG)6	12	23530761	23530772
chr11_3977	p2	(AC)18	36	23535048	23535083
chr11_3978	p2	(GA)7	14	23537942	23537955
chr11_3979	p3	(ATT)6	18	23538718	23538735
chr11_3980	p2	(AC)15	30	23539278	23539307
chr11_3981	p2	(GT)14	28	23545955	23545982
chr11_3982	p2	(TG)6	12	23546158	23546169
chr11_3983	p2	(CA)9	18	23572670	23572687
chr11_3984	p2	(TG)12	24	23574958	23574981
chr11_3985	p2	(TG)14	28	23586369	23586396
chr11_3986	p3	(CTG)5	15	23586929	23586943
chr11_3987	p2	(GT)14	28	23588074	23588101
chr11_3988	p2	(CA)18	36	23588577	23588612
chr11_3989	p2	(CT)6	12	23599855	23599866
chr11_3990	p2	(AT)8	16	23603283	23603298
chr11_3991	p2	(CA)15	30	23603595	23603624
chr11_3992	p2	(TC)9	18	23611999	23612016
chr11_3993	p4	(AAAT)59	236	23625423	23625658
chr11_3994	p2	(GT)12	24	23634464	23634487
chr11_3995	p2	(AC)26	52	23635932	23635983
chr11_3996	p2	(AC)9	18	23644699	23644716
chr11_3997	p2	(GT)16	32	23646351	23646382
chr11_3998	p2	(CA)9	18	23656070	23656087
chr11_3999	p2	(GT)21	42	23676971	23677012
chr11_4000	p3	(GGA)6	18	23686123	23686140
chr11_4001	p2	(AG)6	12	23691212	23691223
chr11_4002	p2	(TG)7	14	23693658	23693671
chr11_4003	p2	(AC)15	30	23694246	23694275
chr11_4004	p2	(CA)11	22	23695415	23695436
chr11_4005	p2	(TG)9	18	23697000	23697017
chr11_4006	p3	(AGT)6	18	23699250	23699267
chr11_4007	p2	(TG)23	46	23706755	23706800
chr11_4008	c	(TA)16catatacatatatatacat(AC)8	67	23707226	23707292
chr11_4009	p2	(TC)6	12	23709125	23709136
chr11_4010	p2	(CA)31	62	23714710	23714771
chr11_4011	p3	(TTG)7	21	23731302	23731322
chr11_4012	p3	(ATT)8	24	23731867	23731890
chr11_4013	p6	(TCTTTT)5	30	23739450	23739479
chr11_4014	p2	(TG)6	12	23752372	23752383
chr11_4015	p2	(AC)7	14	23786525	23786538
chr11_4016	p2	(CA)7	14	23787613	23787626
chr11_4017	p2	(AC)15	30	23787790	23787819

chr11_4018	p4	(TGTA)14	56	23791253	23791308
chr11_4019	p4	(ATAA)6	24	23825429	23825452
chr11_4020	p2	(TA)6	12	23842484	23842495
chr11_4021	p2	(TA)6	12	23849138	23849149
chr11_4022	p2	(GT)7	14	23850220	23850233
chr11_4023	p2	(TG)9	18	23856675	23856692
chr11_4024	p2	(AC)7	14	23878716	23878729
chr11_4025	p2	(TG)10	20	23893390	23893409
chr11_4026	p2	(AC)6	12	23898733	23898744
chr11_4027	p2	(TG)6	12	23899206	23899217
chr11_4028	p3	(TTC)5	15	23899338	23899352
chr11_4029	c	(TG)8c(GT)20	57	23906183	23906239
chr11_4030	p4	(TTTG)6	24	23914761	23914784
chr11_4031	p2	(TG)23	46	23915065	23915110
chr11_4032	p2	(TG)15	30	23917836	23917865
chr11_4033	p2	(CA)6	12	23921230	23921241
chr11_4034	p2	(AT)14	28	23940797	23940824
chr11_4035	c	(GC)6(AC)13	38	23950251	23950288
chr11_4036	p2	(TC)6	12	23955401	23955412
chr11_4037	p2	(GT)6	12	23981009	23981020
chr11_4038	p2	(CA)6	12	23989381	23989392
chr11_4039	p2	(TG)7	14	23989683	23989696
chr11_4040	c	(AC)9aacctttctattgtgtgga(TG)6	49	23992099	23992147
chr11_4041	p2	(TG)15	30	23994525	23994554
chr11_4042	c	(CA)8ctgcaaagctataaatacactgcctc(CA)8	58	23995342	23995399
chr11_4043	c	caggactttccagctgcttcctgctcggcccaggtgctcaatcagaccttat	97	23996189	23996285
chr11_4044	p2	(AG)8	16	24001592	24001607
chr11_4045	p2	(GA)12	24	24002097	24002120
chr11_4046	p2	(AC)6	12	24002421	24002432
chr11_4047	p2	(TG)6	12	24003468	24003479
chr11_4048	p2	(TG)11	22	24003968	24003989
chr11_4049	p2	(AG)6	12	24009242	24009253
chr11_4050	p2	(TG)8	16	24013878	24013893
chr11_4051	p2	(TG)7	14	24014252	24014265
chr11_4052	p2	(AC)6	12	24019332	24019343
chr11_4053	p5	(ATCTT)5	25	24024791	24024815
chr11_4054	p2	(CA)19	38	24025010	24025047
chr11_4055	p2	(CA)6	12	24025204	24025215
chr11_4056	p3	(ATG)5	15	24028854	24028868
chr11_4057	p3	(TAT)7	21	24035507	24035527
chr11_4058	p2	(GT)7	14	24043442	24043455
chr11_4059	p2	(CA)33	66	24046084	24046149
chr11_4060	p2	(CA)6	12	24077122	24077133
chr11_4061	c	(CA)6t(TA)10	33	24079926	24079958
chr11_4062	p4	(ATAG)5	20	24088545	24088564
chr11_4063	p4	(ATGG)7	28	24097402	24097429
chr11_4064	p2	(TA)6	12	24100010	24100021
chr11_4065	p2	(CA)7	14	24101154	24101167
chr11_4066	p2	(GT)13	26	24101844	24101869
chr11_4067	p2	(AT)6	12	24106961	24106972
chr11_4068	p3	(CTC)5	15	24107631	24107645
chr11_4069	p3	(CAG)5	15	24109930	24109944

chr11_4070	p3	(CTG)5	15	24118926	24118940
chr11_4071	p3	(GTG)5	15	24129335	24129349
chr11_4072	p2	(CA)21	42	24139231	24139272
chr11_4073	p2	(TA)6	12	24164548	24164559
chr11_4074	p2	(AC)25	50	24165442	24165491
chr11_4075	p4	(AAGA)5	20	24170485	24170504
chr11_4076	p3	(CTG)7	21	24174650	24174670
chr11_4077	p2	(AC)10	20	24190687	24190706
chr11_4078	p3	(GCA)5	15	24198277	24198291
chr11_4079	p3	(GAT)7	21	24208978	24208998
chr11_4080	p3	(AGA)9	27	24209256	24209282
chr11_4081	p2	(TG)9	18	24217940	24217957
chr11_4082	p2	(TG)8	16	24219607	24219622
chr11_4083	p2	(CA)6	12	24221793	24221804
chr11_4084	p2	(TC)14	28	24225808	24225835
chr11_4085	p2	(GT)7	14	24228243	24228256
chr11_4086	p3	(AGC)5	15	24233670	24233684
chr11_4087	c	(GT)14gg(GT)11	52	24234015	24234066
chr11_4088	p2	(GT)7	14	24236952	24236965
chr11_4089	p3	(CCA)5	15	24238911	24238925
chr11_4090	p4	(CATC)5	20	24239949	24239968
chr11_4091	p2	(GT)10	20	24244567	24244586
chr11_4092	p2	(TG)20	40	24248135	24248174
chr11_4093	p2	(CA)7	14	24255187	24255200
chr11_4094	p2	(CT)7	14	24257050	24257063
chr11_4095	p2	(TC)6	12	24259549	24259560
chr11_4096	p2	(GA)8	16	24262468	24262483
chr11_4097	p3	(ATT)8	24	24264250	24264273
chr11_4098	p2	(TG)12	24	24271680	24271703
chr11_4099	p2	(TG)14	28	24271925	24271952
chr11_4100	c	(GTA)12(ATG)5	51	24273596	24273646
chr11_4101	p2	(CA)7	14	24276963	24276976
chr11_4102	p2	(AC)9	18	24281090	24281107
chr11_4103	p2	(AT)6	12	24284949	24284960
chr11_4104	p2	(CA)24	48	24287844	24287891
chr11_4105	p2	(AG)7	14	24297735	24297748
chr11_4106	p2	(CA)13	26	24300492	24300517
chr11_4107	p3	(AAT)5	15	24302687	24302701
chr11_4108	p3	(CAA)5	15	24305646	24305660
chr11_4109	p2	(CT)7	14	24305773	24305786
chr11_4110	p2	(AC)9	18	24321769	24321786
chr11_4111	p2	(CA)10	20	24331295	24331314
chr11_4112	p2	(TG)7	14	24333977	24333990
chr11_4113	p2	(GT)12	24	24339436	24339459
chr11_4114	c	(GT)9(GA)6	30	24340083	24340112
chr11_4115	p2	(GT)6	12	24340377	24340388
chr11_4116	p2	(TG)6	12	24358684	24358695
chr11_4117	c	(GT)9(GA)14	46	24362163	24362208
chr11_4118	p2	(TG)6	12	24365939	24365950
chr11_4119	p2	(CA)9	18	24372214	24372231
chr11_4120	p2	(CA)9	18	24392460	24392477
chr11_4121	p2	(AC)6	12	24392963	24392974

chr11_4122	p2	(CA)8	16	24409872	24409887
chr11_4123	p2	(CA)9	18	24413058	24413075
chr11_4124	c	(TG)6gtaagaaagtgcatgggcgtggtta(TG)6	49	24420780	24420828
chr11_4125	p2	(TG)22	44	24421222	24421265
chr11_4126	p2	(TG)7	14	24421391	24421404
chr11_4127	p2	(CA)7	14	24424333	24424346
chr11_4128	p2	(AG)10	20	24424618	24424637
chr11_4129	p2	(AG)19	38	24429553	24429590
chr11_4130	p2	(GT)14	28	24429915	24429942
chr11_4131	c	(AC)15(CA)6	42	24434892	24434933
chr11_4132	p2	(TC)6	12	24442722	24442733
chr11_4133	p2	(CA)12	24	24445406	24445429
chr11_4134	p2	(AG)16	32	24450849	24450880
chr11_4135	c	(TG)11(CG)6	34	24451458	24451491
chr11_4136	c	(CA)7cg(CA)13	42	24451648	24451689
chr11_4137	c	agagagagagactggagcgcaaagaccagcgcaagtgtccctcgagag	122	24451834	24451955
chr11_4138	p2	(GT)7	14	24452313	24452326
chr11_4139	p2	(CA)7	14	24453327	24453340
chr11_4140	p3	(GAG)6	18	24454785	24454802
chr11_4141	p2	(TG)9	18	24456364	24456381
chr11_4142	p3	(AGA)11	33	24458302	24458334
chr11_4143	p4	(CTGA)5	20	24466478	24466497
chr11_4144	p2	(GT)14	28	24472540	24472567
chr11_4145	p2	(CA)6	12	24472735	24472746
chr11_4146	p2	(GT)13	26	24473022	24473047
chr11_4147	p2	(TG)9	18	24474490	24474507
chr11_4148	p2	(TG)7	14	24475074	24475087
chr11_4149	p3	(CTG)6	18	24476714	24476731
chr11_4150	p2	(CA)8	16	24479495	24479510
chr11_4151	p2	(TG)27	54	24480136	24480189
chr11_4152	c	(AG)9(TG)8	34	24486513	24486546
chr11_4153	p2	(CA)11	22	24486659	24486680
chr11_4154	p2	(CA)17	34	24492374	24492407
chr11_4155	p2	(CA)6	12	24493024	24493035
chr11_4156	p3	(TTG)5	15	24496914	24496928
chr11_4157	c	(TC)8tgtggagtgggaaatggtgcccaaatttcagtgcctgct(AG)8	71	24501492	24501562
chr11_4158	p2	(AG)8	16	24501982	24501997
chr11_4159	p3	(AAC)6	18	24507571	24507588
chr11_4160	p2	(AC)21	42	24513910	24513951
chr11_4161	p2	(AC)9	18	24517083	24517100
chr11_4162	p2	(TC)6	12	24525232	24525243
chr11_4163	p4	(AAAT)7	28	24541473	24541500
chr11_4164	p2	(CA)11	22	24551287	24551308
chr11_4165	c	TG)8tcagggtcagcaagatgcgtgcatgcgtgcatgcgtgcgca(TG)	71	24555661	24555731
chr11_4166	p2	(CA)6	12	24557794	24557805
chr11_4167	p2	(CA)10	20	24561649	24561668
chr11_4168	p3	(CAA)5	15	24565558	24565572
chr11_4169	p2	(AT)9	18	24566109	24566126
chr11_4170	p2	(AC)10	20	24569155	24569174
chr11_4171	p3	(CTG)5	15	24587245	24587259
chr11_4172	p2	(GT)30	60	24592425	24592484
chr11_4173	p2	(TG)6	12	24606770	24606781

chr11_4174	p3	(CCT)5	15	24607413	24607427
chr11_4175	p2	(AC)9	18	24616335	24616352
chr11_4176	p2	(TG)8	16	24621959	24621974
chr11_4177	c	(CA)6agcacaa(AC)6	31	24622524	24622554
chr11_4178	p2	(TG)6	12	24623647	24623658
chr11_4179	p3	(CAG)5	15	24623971	24623985
chr11_4180	p3	(ACC)5	15	24635594	24635608
chr11_4181	p2	(GT)6	12	24642278	24642289
chr11_4182	p2	(AG)6	12	24667569	24667580
chr11_4183	p2	(CA)21	42	24672381	24672422
chr11_4184	p2	(CA)9	18	24676032	24676049
chr11_4185	p3	(GTA)5	15	24676398	24676412
chr11_4186	p2	(AC)7	14	24679217	24679230
chr11_4187	p2	(CA)11	22	24686873	24686894
chr11_4188	p2	(CA)8	16	24690678	24690693
chr11_4189	p2	(AC)12	24	24690971	24690994
chr11_4190	p2	(AT)8	16	24705897	24705912
chr11_4191	p3	(GCT)5	15	24717710	24717724
chr11_4192	p2	(TC)7	14	24724685	24724698
chr11_4193	p2	(TA)10	20	24726073	24726092
chr11_4194	p2	(TC)7	14	24727935	24727948
chr11_4195	p2	(TA)10	20	24729323	24729342
chr11_4196	p3	(TGC)6	18	24731998	24732015
chr11_4197	p2	(AT)7	14	24741320	24741333
chr11_4198	p2	(GT)7	14	24743881	24743894
chr11_4199	p2	(AC)8	16	24744245	24744260
chr11_4200	p2	(CA)7	14	24745345	24745358
chr11_4201	p2	(AC)8	16	24782519	24782534
chr11_4202	p4	(GATG)5	20	24798846	24798865
chr11_4203	p2	(AC)7	14	24831050	24831063
chr11_4204	p2	(AC)9	18	24850585	24850602
chr11_4205	p3	(GAA)6	18	24851204	24851221
chr11_4206	p2	(CA)17	34	24853021	24853054
chr11_4207	p2	(TG)6	12	24853822	24853833
chr11_4208	p2	(AT)12	24	24854807	24854830
chr11_4209	p4	(ATGG)5	20	24907662	24907681
chr11_4210	p3	(GTG)5	15	24908959	24908973
chr11_4211	c	(TA)6(CA)13	38	24913544	24913581
chr11_4212	p2	(TG)8	16	24916574	24916589
chr11_4213	p2	(GT)15	30	24917683	24917712
chr11_4214	p2	(TG)24	48	24938586	24938633
chr11_4215	p3	(GCT)5	15	24947428	24947442
chr11_4216	p2	(AC)6	12	24954111	24954122
chr11_4217	p2	(AC)9	18	24955159	24955176
chr11_4218	p2	(AC)20	40	24956368	24956407
chr11_4219	c	(TG)29(AG)11	80	24961408	24961487
chr11_4220	c	(CT)7(CA)9	32	24962276	24962307
chr11_4221	p3	(TAT)7	21	24963624	24963644
chr11_4222	p2	(GT)15	30	24974065	24974094
chr11_4223	p2	(CA)16	32	24978802	24978833
chr11_4224	p3	(ATA)5	15	24979808	24979822
chr11_4225	p2	(TG)12	24	24983528	24983551

chr11_4226	p3	(GTT)8	24	24990200	24990223
chr11_4227	p3	(TTC)5	15	25010192	25010206
chr11_4228	p2	(AC)7	14	25010329	25010342
chr11_4229	c	caggggactggagttgtaaattattcactgatatgcacggatgcacgcata	90	25019619	25019708
chr11_4230	p4	(AAAC)6	24	25022600	25022623
chr11_4231	c	(TC)8actcact(CA)19	61	25034061	25034121
chr11_4232	p2	(CA)6	12	25052627	25052638
chr11_4233	c	(GT)6gg(GT)8	30	25065982	25066011
chr11_4234	p5	(ATCAA)5	25	25083861	25083885
chr11_4235	p2	(TG)13	26	25091396	25091421
chr11_4236	c	tgagtgtgtgtgagagcgtgagtggtgaagaagcaccgtaatgccaggtc	104	25091845	25091948
chr11_4237	p3	(TGA)8	24	25093987	25094010
chr11_4238	p2	(AG)6	12	25097792	25097803
chr11_4239	p2	(TG)8	16	25107752	25107767
chr11_4240	p2	(CA)6	12	25111932	25111943
chr11_4241	p2	(GA)6	12	25118432	25118443
chr11_4242	p2	(GT)16	32	25120661	25120692
chr11_4243	p2	(AC)10	20	25131885	25131904
chr11_4244	p2	(CA)6	12	25145487	25145498
chr11_4245	p2	(TG)6	12	25146074	25146085
chr11_4246	p3	(TGA)7	21	25176068	25176088
chr11_4247	p2	(TG)8	16	25178659	25178674
chr11_4248	p3	(CCT)6	18	25198576	25198593
chr11_4249	p2	(AC)12	24	25200416	25200439
chr11_4250	p5	(AAATC)6	30	25221235	25221264
chr11_4251	p2	(AG)6	12	25228176	25228187
chr11_4252	p2	(CA)23	46	25236071	25236116
chr11_4253	p2	(CA)54	108	25253422	25253529
chr11_4254	c	(GC)6acg(CA)20	55	25263318	25263372
chr11_4255	p2	(AG)6	12	25266681	25266692
chr11_4256	c	cccggctcgttgttttctgaatccatcctgttggaggggaagcgagggtgg	162	25270472	25270633
chr11_4257	p2	(GT)19	38	25270968	25271005
chr11_4258	p2	(AC)7	14	25275562	25275575
chr11_4259	p2	(AC)19	38	25277631	25277668
chr11_4260	p2	(CT)6	12	25286510	25286521
chr11_4261	p2	(CA)15	30	25287761	25287790
chr11_4262	p2	(AC)9	18	25290693	25290710
chr11_4263	p2	(CA)19	38	25297814	25297851
chr11_4264	p3	(GCA)8	24	25311990	25312013
chr11_4265	p2	(TC)12	24	25319676	25319699
chr11_4266	p3	(GAT)5	15	25321174	25321188
chr11_4267	c	ccacttatcactgacggcactgttgctcatcgatgatggcatgggaggtgc	117	25328947	25329063
chr11_4268	c	(TA)6c(AT)6	25	25329611	25329635
chr11_4269	c	gtgg(TC)6ttgtagtgcc(CT)8cccctgtagtgtgg(TC)6ctgtag	1037	25331497	25332533
chr11_4270	p2	(AC)6	12	25333254	25333265
chr11_4271	p4	(TGAT)8	32	25335950	25335981
chr11_4272	p3	(AGG)5	15	25338048	25338062
chr11_4273	p2	(AC)12	24	25340482	25340505
chr11_4274	p2	(GT)10	20	25341100	25341119
chr11_4275	p2	(GA)6	12	25343219	25343230
chr11_4276	p2	(GT)7	14	25356403	25356416
chr11_4277	p2	(AC)15	30	25372709	25372738

chr11_4278	p2	(TG)6	12	25379036	25379047
chr11_4279	p2	(AG)11	22	25386343	25386364
chr11_4280	p2	(CA)7	14	25394216	25394229
chr11_4281	p2	(TG)8	16	25401812	25401827
chr11_4282	p2	(AC)13	26	25408608	25408633
chr11_4283	p2	(CT)7	14	25409625	25409638
chr11_4284	p3	(GAT)8	24	25410345	25410368
chr11_4285	p2	(GT)14	28	25413739	25413766
chr11_4286	p2	(CT)7	14	25415851	25415864
chr11_4287	p2	(GT)18	36	25416521	25416556
chr11_4288	p5	(GGGGC)5	25	25425204	25425228
chr11_4289	p2	(AC)9	18	25425954	25425971
chr11_4290	p2	(CA)9	18	25434048	25434065
chr11_4291	p2	(AC)17	34	25435157	25435190
chr11_4292	c	(GT)7atagt(GA)6	31	25435794	25435824
chr11_4293	c	agaatagttggcagtgtgtctgagaaatatgtgacctctgaccacacacagc	103	25441734	25441836
chr11_4294	p2	(TA)7	14	25448350	25448363
chr11_4295	p3	(TTA)9	27	25449105	25449131
chr11_4296	p2	(CA)10	20	25452203	25452222
chr11_4297	p2	(AC)6	12	25460254	25460265
chr11_4298	c	(CT)11(CA)13	48	25462327	25462374
chr11_4299	p2	(AG)12	24	25471917	25471940
chr11_4300	p2	(CA)17	34	25479924	25479957
chr11_4301	c	cgcgactccatgtgtgtcgtctgttggcctgtttaagattgtgataaccaatct	106	25486318	25486423
chr11_4302	p2	(GT)7	14	25489101	25489114
chr11_4303	p2	(GT)12	24	25489389	25489412
chr11_4304	p2	(GT)6	12	25497020	25497031
chr11_4305	p2	(TG)13	26	25504639	25504664
chr11_4306	p2	(GT)12	24	25505992	25506015
chr11_4307	p2	(CA)11	22	25506148	25506169
chr11_4308	p2	(GT)8	16	25516396	25516411
chr11_4309	p2	(TG)15	30	25517992	25518021
chr11_4310	p2	(AC)9	18	25528977	25528994
chr11_4311	p2	(AC)7	14	25529832	25529845
chr11_4312	p2	(TG)9	18	25540514	25540531
chr11_4313	p2	(TG)6	12	25541085	25541096
chr11_4314	p2	(TG)7	14	25545193	25545206
chr11_4315	p2	(CA)10	20	25550258	25550277
chr11_4316	p3	(TGA)5	15	25551021	25551035
chr11_4317	p2	(TG)10	20	25560309	25560328
chr11_4318	p3	(GAG)7	21	25564924	25564944
chr11_4319	c	(AC)6(AG)34	80	25582719	25582798
chr11_4320	c	(TCC)5atc(TCA)5	33	25585213	25585245
chr11_4321	p3	(CCA)5	15	25608351	25608365
chr11_4322	p2	(CA)8	16	25618631	25618646
chr11_4323	p2	(AT)7	14	25620290	25620303
chr11_4324	p3	(GCA)5	15	25647216	25647230
chr11_4325	p3	(ATA)6	18	25650580	25650597
chr11_4326	p2	(CA)17	34	25668787	25668820
chr11_4327	p2	(AC)26	52	25675042	25675093
chr11_4328	p4	(CATC)5	20	25676933	25676952
chr11_4329	p3	(AGA)5	15	25678119	25678133

chr11_4330	p4	(TTTG)5	20	25680778	25680797
chr11_4331	p2	(CA)9	18	25685069	25685086
chr11_4332	p2	(CT)6	12	25686930	25686941
chr11_4333	p2	(GT)7	14	25688197	25688210
chr11_4334	p2	(AC)7	14	25689134	25689147
chr11_4335	p2	(AC)6	12	25692207	25692218
chr11_4336	p2	(TC)8	16	25693003	25693018
chr11_4337	p2	(TA)11	22	25693357	25693378
chr11_4338	p2	(TA)12	24	25693529	25693552
chr11_4339	c	)10atctgtttaccactcctttctatctgaacccatgtgtgtgcttttgtgttt(G	93	25696463	25696555
chr11_4340	p2	(AC)6	12	25698357	25698368
chr11_4341	p2	(TG)6	12	25698883	25698894
chr11_4342	p2	(AC)6	12	25700646	25700657
chr11_4343	p2	(CT)11	22	25706211	25706232
chr11_4344	p2	(TC)6	12	25707657	25707668
chr11_4345	p2	(AC)9	18	25715612	25715629
chr11_4346	p2	(TC)7	14	25723655	25723668
chr11_4347	p2	(AG)8	16	25729918	25729933
chr11_4348	p2	(TG)6	12	25730526	25730537
chr11_4349	p2	(TG)6	12	25732204	25732215
chr11_4350	p2	(GT)15	30	25741465	25741494
chr11_4351	p2	(AC)29	58	25745380	25745437
chr11_4352	p2	(CA)16	32	25746745	25746776
chr11_4353	c	(AC)9g(CA)19	57	25748587	25748643
chr11_4354	p2	(AC)6	12	25752212	25752223
chr11_4355	p2	(AC)10	20	25752358	25752377
chr11_4356	p2	(TG)12	24	25753358	25753381
chr11_4357	p2	(AC)39	78	25754491	25754568
chr11_4358	c	gtataacacgctccacacacaacaccaaaaaaacacttctccacctaaac	92	25755431	25755522
chr11_4359	p2	(AT)6	12	25758949	25758960
chr11_4360	p2	(AG)20	40	25759343	25759382
chr11_4361	p2	(GT)9	18	25762862	25762879
chr11_4362	p2	(GT)15	30	25764584	25764613
chr11_4363	p2	(TG)7	14	25764939	25764952
chr11_4364	p2	(TG)16	32	25765059	25765090
chr11_4365	p2	(AC)6	12	25766250	25766261
chr11_4366	p2	(AC)14	28	25769184	25769211
chr11_4367	p2	(AC)19	38	25770102	25770139
chr11_4368	p2	(GT)6	12	25777161	25777172
chr11_4369	p2	(TG)7	14	25777364	25777377
chr11_4370	p2	(CA)37	74	25780330	25780403
chr11_4371	p2	(CT)6	12	25787595	25787606
chr11_4372	c	gcgccacagatggctgccttggacgtgtgtgtaacacgctttgccaataaag	96	25794594	25794689
chr11_4373	p2	(CA)15	30	25799598	25799627
chr11_4374	p2	(CA)10	20	25800912	25800931
chr11_4375	p2	(AC)8	16	25809445	25809460
chr11_4376	p2	(AG)6	12	25811943	25811954
chr11_4377	p2	(TG)6	12	25813447	25813458
chr11_4378	p2	(TG)14	28	25814642	25814669
chr11_4379	p2	(TG)8	16	25820888	25820903
chr11_4380	p2	(CA)37	74	25825777	25825850
chr11_4381	c	(TC)6actct(CA)8	33	25848889	25848921

chr11_4382	p2	(TG)6	12	25852558	25852569
chr11_4383	c	ıagcttgcccg(AC)13agttaaaagatcgatc(GT)14cttagtctctgı	202	25860179	25860380
chr11_4384	p2	(TC)7	14	25862106	25862119
chr11_4385	c	(TC)7(AC)25	64	25865883	25865946
chr11_4386	c	(TC)7(AC)23	60	25867012	25867071
chr11_4387	p2	(GT)12	24	25870576	25870599
chr11_4388	p2	(AC)6	12	25872024	25872035
chr11_4389	p2	(GT)30	60	25877062	25877121
chr11_4390	p2	(AC)21	42	25881440	25881481
chr11_4391	c	;tttggacactgggcagaaacgtcaggggccaaactggcctttctgccagc	132	25883374	25883505
chr11_4392	p2	(GT)23	46	25884233	25884278
chr11_4393	p2	(GT)6	12	25884581	25884592
chr11_4394	p2	(AC)7	14	25886990	25887003
chr11_4395	p2	(AC)6	12	25887754	25887765
chr11_4396	p2	(AC)7	14	25897880	25897893
chr11_4397	c	(TG)19ta(TG)7	54	25899824	25899877
chr11_4398	c	gaaagcagaaagaatttgatccttgccgtctccgtcgtcacataactctaca	99	25903330	25903428
chr11_4399	p2	(TG)6	12	25910793	25910804
chr11_4400	c	(TA)6g(AT)6ctatatatagatatag(AT)7	55	25924173	25924227
chr11_4401	p2	(GT)17	34	25931516	25931549
chr11_4402	c	(ATT)5actgttattca(ATT)5	41	25935811	25935851
chr11_4403	p2	(AC)15	30	25954888	25954917
chr11_4404	c	(GT)8(AT)8	32	25959967	25959998
chr11_4405	p2	(TG)6	12	25965063	25965074
chr11_4406	p3	(CAG)5	15	25966929	25966943
chr11_4407	c	(ATAG)6ag(AGAT)5agag(AGAT)5agag(AGAT)5	94	25976878	25976971
chr11_4408	c	(GT)19gg(GT)9	58	25985334	25985391
chr11_4409	p6	(GAGAGT)5	30	25999339	25999368
chr11_4410	p2	(TG)12	24	26004639	26004662
chr11_4411	p3	(TCC)6	18	26017735	26017752
chr11_4412	p3	(GAA)10	30	26044698	26044727
chr11_4413	p3	(TGC)5	15	26045060	26045074
chr11_4414	p3	(CAG)6	18	26052812	26052829
chr11_4415	p2	(AC)14	28	26059899	26059926
chr11_4416	p2	(TG)11	22	26061993	26062014
chr11_4417	p2	(AC)13	26	26075747	26075772
chr11_4418	p2	(GT)7	14	26077298	26077311
chr11_4419	p4	(TAAC)7	28	26081808	26081835
chr11_4420	p2	(TC)6	12	26082510	26082521
chr11_4421	p2	(CA)18	36	26082731	26082766
chr11_4422	p2	(AC)6	12	26085219	26085230
chr11_4423	p2	(TG)8	16	26088862	26088877
chr11_4424	c	:acacacccatgcatctacatatgcagttaaaatactcgcatt(CA)7gtga	208	26092067	26092274
chr11_4425	p2	(GA)12	24	26107488	26107511
chr11_4426	p2	(AC)19	38	26109979	26110016
chr11_4427	p2	(AC)6	12	26110302	26110313
chr11_4428	p3	(CTG)5	15	26137346	26137360
chr11_4429	p2	(AC)11	22	26148045	26148066
chr11_4430	p2	(CT)6	12	26148670	26148681
chr11_4431	p2	(AC)7	14	26148801	26148814
chr11_4432	p2	(AC)15	30	26149691	26149720
chr11_4433	p2	(GT)7	14	26154396	26154409

chr11_4434	p2	(TG)14	28	26160811	26160838
chr11_4435	c	(CT)6(CA)6	24	26170544	26170567
chr11_4436	p3	(ATA)5	15	26172553	26172567
chr11_4437	p2	(TG)6	12	26174388	26174399
chr11_4438	p3	(TGT)9	27	26177217	26177243
chr11_4439	c	(TC)9(AC)9	36	26189982	26190017
chr11_4440	p2	(TG)18	36	26190612	26190647
chr11_4441	p3	(GAG)6	18	26193236	26193253
chr11_4442	p2	(TC)6	12	26193598	26193609
chr11_4443	p2	(GT)8	16	26195798	26195813
chr11_4444	p2	(TC)8	16	26196541	26196556
chr11_4445	p2	(TG)9	18	26208484	26208501
chr11_4446	p2	(GT)12	24	26209347	26209370
chr11_4447	p2	(GT)12	24	26211716	26211739
chr11_4448	p2	(GT)6	12	26211896	26211907
chr11_4449	p2	(TG)12	24	26215116	26215139
chr11_4450	p2	(TG)9	18	26223288	26223305
chr11_4451	p2	(TG)14	28	26226957	26226984
chr11_4452	p4	(AAAC)5	20	26233362	26233381
chr11_4453	p2	(TA)8	16	26236496	26236511
chr11_4454	p2	(CT)7	14	26237072	26237085
chr11_4455	p3	(GAT)6	18	26244442	26244459
chr11_4456	p2	(AC)7	14	26264411	26264424
chr11_4457	p3	(AAG)5	15	26266429	26266443
chr11_4458	p3	(AAT)6	18	26270105	26270122
chr11_4459	p5	(AGTTG)5	25	26278025	26278049
chr11_4460	p2	(AC)7	14	26288293	26288306
chr11_4461	p2	(TG)6	12	26312867	26312878
chr11_4462	p2	(TG)7	14	26320239	26320252
chr11_4463	p2	(GT)8	16	26323420	26323435
chr11_4464	p2	(TG)8	16	26326491	26326506
chr11_4465	p3	(ATT)7	21	26330464	26330484
chr11_4466	p2	(GT)14	28	26338428	26338455
chr11_4467	p2	(AC)8	16	26340986	26341001
chr11_4468	p2	(CA)8	16	26343974	26343989
chr11_4469	p4	(ATAG)6	24	26348304	26348327
chr11_4470	p2	(AC)14	28	26348628	26348655
chr11_4471	p2	(CA)26	52	26348943	26348994
chr11_4472	p2	(TC)12	24	26375192	26375215
chr11_4473	p2	(TG)24	48	26375462	26375509
chr11_4474	p2	(TG)12	24	26376285	26376308
chr11_4475	p2	(AT)7	14	26380239	26380252
chr11_4476	c	agcaccaaccatcagggaactgcctcttgctttgtcatatgctcatttgtcag	151	26383681	26383831
chr11_4477	p2	(TG)21	42	26385941	26385982
chr11_4478	p2	(TG)11	22	26388114	26388135
chr11_4479	p2	(AT)7	14	26388656	26388669
chr11_4480	c	ccatttttcagatcaatgattctgtccagcacacagtaactgtgagaggcctt	109	26389813	26389921
chr11_4481	p2	(CT)6	12	26390269	26390280
chr11_4482	p3	(TGT)5	15	26394918	26394932
chr11_4483	p2	(AC)8	16	26399848	26399863
chr11_4484	p2	(TG)14	28	26400084	26400111
chr11_4485	p2	(AC)9	18	26400222	26400239

chr11_4486	p2	(GA)6	12	26401669	26401680
chr11_4487	p4	(CATT)5	20	26402347	26402366
chr11_4488	p2	(TG)28	56	26406864	26406919
chr11_4489	p2	(AC)7	14	26412662	26412675
chr11_4490	p2	(AC)10	20	26412790	26412809
chr11_4491	p4	(ATTT)5	20	26417600	26417619
chr11_4492	c	ttcagtcatgagtcatcctgtcgcta(AC)21agtatgcagctgttgatctg	265	26419350	26419614
chr11_4493	p2	(TG)21	42	26421655	26421696
chr11_4494	p3	(AAT)5	15	26422371	26422385
chr11_4495	p2	(TG)7	14	26423356	26423369
chr11_4496	p2	(CT)8	16	26424256	26424271
chr11_4497	p2	(GA)7	14	26430009	26430022
chr11_4498	p2	(CA)19	38	26431910	26431947
chr11_4499	p2	(TG)6	12	26439736	26439747
chr11_4500	p2	(AC)6	12	26440852	26440863
chr11_4501	c	(TG)6cgtgcgcttgttctctttcacctaattcctgtgc(GT)7	60	26443732	26443791
chr11_4502	p2	(CT)13	26	26449082	26449107
chr11_4503	p2	(AC)6	12	26449319	26449330
chr11_4504	p2	(TG)7	14	26453036	26453049
chr11_4505	c	acacactttggtcacttgagattatgcatgtgcacattgttgtacatgtatcca	91	26453644	26453734
chr11_4506	p2	(TC)6	12	26454068	26454079
chr11_4507	p2	(CT)9	18	26457927	26457944
chr11_4508	p3	(ATG)5	15	26459406	26459420
chr11_4509	p2	(TG)7	14	26467249	26467262
chr11_4510	p2	(CA)6	12	26469699	26469710
chr11_4511	p2	(GT)6	12	26470621	26470632
chr11_4512	p3	(TCA)7	21	26472243	26472263
chr11_4513	p2	(TG)6	12	26473099	26473110
chr11_4514	p2	(CA)10	20	26475013	26475032
chr11_4515	c	(GT)12gagggagagagagaa(AG)8	55	26476807	26476861
chr11_4516	p2	(TC)6	12	26478294	26478305
chr11_4517	p2	(GT)6	12	26478425	26478436
chr11_4518	p2	(CA)6	12	26486185	26486196
chr11_4519	p2	(CA)11	22	26493028	26493049
chr11_4520	p2	(TG)17	34	26497240	26497273
chr11_4521	p2	(GT)11	22	26503629	26503650
chr11_4522	p2	(TC)7	14	26522136	26522149
chr11_4523	p2	(AT)7	14	26522582	26522595
chr11_4524	p2	(CA)12	24	26531075	26531098
chr11_4525	p2	(CA)6	12	26543306	26543317
chr11_4526	p2	(GT)8	16	26543893	26543908
chr11_4527	p2	(AC)8	16	26544477	26544492
chr11_4528	p2	(AG)6	12	26544657	26544668
chr11_4529	p2	(TG)7	14	26552567	26552580
chr11_4530	p2	(AG)6	12	26554460	26554471
chr11_4531	p2	(CT)8	16	26557204	26557219
chr11_4532	p2	(AC)6	12	26557335	26557346
chr11_4533	p2	(TA)6	12	26568616	26568627
chr11_4534	c	(CA)6t(AC)6	25	26575778	26575802
chr11_4535	p2	(CA)37	74	26578229	26578302
chr11_4536	p3	(ATT)8	24	26582446	26582469
chr11_4537	p3	(ATT)5	15	26589194	26589208

chr11_4538	p3	(ATT)7	21	26594851	26594871
chr11_4539	c	(CT)7ccctccacccggaccacaggagccca(CT)6	52	26602336	26602387
chr11_4540	p2	(GT)9	18	26605039	26605056
chr11_4541	p2	(CA)7	14	26608111	26608124
chr11_4542	p2	(AG)8	16	26611115	26611130
chr11_4543	p2	(AC)8	16	26612216	26612231
chr11_4544	p2	(CA)12	24	26621084	26621107
chr11_4545	p2	(AC)6	12	26624327	26624338
chr11_4546	p3	(ATT)5	15	26637012	26637026
chr11_4547	p3	(TGC)6	18	26641632	26641649
chr11_4548	p2	(AC)19	38	26647537	26647574
chr11_4549	c	(GA)7(GT)19	52	26648147	26648198
chr11_4550	p5	(CTGCT)5	25	26656055	26656079
chr11_4551	p3	(AAT)5	15	26683855	26683869
chr11_4552	p2	(TG)6	12	26715457	26715468
chr11_4553	p2	(TG)7	14	26716277	26716290
chr11_4554	p2	(GT)27	54	26717489	26717542
chr11_4555	p2	(CA)9	18	26718705	26718722
chr11_4556	p2	(CA)16	32	26722431	26722462
chr11_4557	p2	(AC)7	14	26723344	26723357
chr11_4558	p2	(GT)14	28	26723962	26723989
chr11_4559	p2	(GT)6	12	26725663	26725674
chr11_4560	p2	(AG)8	16	26739996	26740011
chr11_4561	p2	(AC)6	12	26740114	26740125
chr11_4562	p2	(TC)6	12	26744278	26744289
chr11_4563	p2	(GA)7	14	26747703	26747716
chr11_4564	p2	(GT)6	12	26749190	26749201
chr11_4565	c	tccatcccctactgggtcagtctcctgtgcctgctgtgnnnnnnnnnnnn	112	26753877	26753988
chr11_4566	p2	(CA)10	20	26758291	26758310
chr11_4567	p2	(AG)12	24	26779291	26779314
chr11_4568	p2	(AC)9	18	26781430	26781447
chr11_4569	p2	(AC)10	20	26785765	26785784
chr11_4570	p2	(TG)6	12	26805606	26805617
chr11_4571	p3	(GAG)5	15	26817522	26817536
chr11_4572	p2	(GT)12	24	26818383	26818406
chr11_4573	p2	(TC)6	12	26824723	26824734
chr11_4574	p5	(TTGAT)5	25	26831999	26832023
chr11_4575	p4	(TATT)8	32	26840594	26840625
chr11_4576	p4	(ATGG)5	20	26858629	26858648
chr11_4577	p2	(CA)20	40	26861826	26861865
chr11_4578	p2	(TG)6	12	26880104	26880115
chr11_4579	p2	(TG)10	20	26903781	26903800
chr11_4580	p3	(CTT)6	18	26924442	26924459
chr11_4581	p2	(GA)9	18	26936299	26936316
chr11_4582	c	(TA)7ctgtatatatata(TG)11	49	26948353	26948401
chr11_4583	p3	(CTG)6	18	26954940	26954957
chr11_4584	p2	(GA)6	12	26956372	26956383
chr11_4585	p2	(AC)6	12	26958396	26958407
chr11_4586	c	(CT)11(CA)23	68	26964818	26964885
chr11_4587	p2	(AG)10	20	26986297	26986316
chr11_4588	p2	(CA)11	22	26986554	26986575
chr11_4589	p2	(CA)6	12	26991616	26991627

chr11_4590	p2	(CA)8	16	26993560	26993575
chr11_4591	p2	(GT)7	14	26996908	26996921
chr11_4592	p3	(TAT)5	15	26998088	26998102
chr11_4593	p2	(AC)6	12	26999320	26999331
chr11_4594	p2	(CA)11	22	27001088	27001109
chr11_4595	p2	(AC)10	20	27001351	27001370
chr11_4596	p2	(TG)6	12	27008635	27008646
chr11_4597	p2	(GT)15	30	27010008	27010037
chr11_4598	p2	(TG)10	20	27012365	27012384
chr11_4599	p2	(CA)6	12	27018261	27018272
chr11_4600	p2	(CA)17	34	27018380	27018413
chr11_4601	p2	(TG)7	14	27023426	27023439
chr11_4602	c	(GT)7(GA)14	42	27026159	27026200
chr11_4603	c	(TG)11(AG)10	42	27059021	27059062
chr11_4604	p2	(CA)10	20	27059762	27059781
chr11_4605	p2	(GT)10	20	27067056	27067075
chr11_4606	p2	(CA)19	38	27078498	27078535
chr11_4607	p2	(CT)9	18	27080087	27080104
chr11_4608	p2	(AC)15	30	27081243	27081272
chr11_4609	p2	(CT)7	14	27082367	27082380
chr11_4610	c	(CA)7tacacg(CA)6	32	27083918	27083949
chr11_4611	p2	(AC)7	14	27084118	27084131
chr11_4612	p2	(TG)8	16	27086778	27086793
chr11_4613	p2	(AC)10	20	27090032	27090051
chr11_4614	p2	(AC)6	12	27094232	27094243
chr11_4615	p2	(GA)7	14	27094513	27094526
chr11_4616	p2	(CT)7	14	27095495	27095508
chr11_4617	c	(AGT)6(AAT)5	33	27095911	27095943
chr11_4618	p2	(TG)15	30	27100814	27100843
chr11_4619	p2	(CA)6	12	27103765	27103776
chr11_4620	p2	(CA)6	12	27126921	27126932
chr11_4621	p2	(TG)6	12	27130637	27130648
chr11_4622	p2	(GT)15	30	27131536	27131565
chr11_4623	c	(TG)6(AG)8	28	27139620	27139647
chr11_4624	p2	(AC)12	24	27142622	27142645
chr11_4625	p2	(AT)6	12	27142892	27142903
chr11_4626	p3	(TTG)5	15	27144143	27144157
chr11_4627	p3	(TAT)6	18	27151558	27151575
chr11_4628	p2	(AC)8	16	27157788	27157803
chr11_4629	p2	(GT)10	20	27157933	27157952
chr11_4630	c	(CA)12t(AC)7	39	27161693	27161731
chr11_4631	c	(GT)7gcgtgcgc(GT)6	34	27167675	27167708
chr11_4632	p2	(GA)20	40	27172842	27172881
chr11_4633	p2	(CA)7	14	27173710	27173723
chr11_4634	p2	(CA)9	18	27184632	27184649
chr11_4635	p2	(AC)7	14	27188889	27188902
chr11_4636	p2	(TC)6	12	27191518	27191529
chr11_4637	p3	(GGT)5	15	27205925	27205939
chr11_4638	p2	(TG)6	12	27209249	27209260
chr11_4639	p2	(TG)14	28	27210308	27210335
chr11_4640	p2	(CT)6	12	27212565	27212576
chr11_4641	p4	(AAAC)5	20	27216923	27216942

chr11_4642	p2	(AG)10	20	27219989	27220008
chr11_4643	c	(GT)10a(TG)11	43	27225301	27225343
chr11_4644	p2	(TG)10	20	27233566	27233585
chr11_4645	p3	(TTC)5	15	27241011	27241025
chr11_4646	p2	(TG)6	12	27246467	27246478
chr11_4647	c	(TC)7c(CT)6	27	27250833	27250859
chr11_4648	p2	(GT)7	14	27252198	27252211
chr11_4649	p2	(TG)10	20	27258152	27258171
chr11_4650	p2	(AT)6	12	27259475	27259486
chr11_4651	p2	(AG)12	24	27259899	27259922
chr11_4652	p2	(GT)11	22	27304815	27304836
chr11_4653	p2	(GT)13	26	27312350	27312375
chr11_4654	p4	(CTGT)5	20	27319479	27319498
chr11_4655	p2	(TG)19	38	27324114	27324151
chr11_4656	p2	(TA)6	12	27330599	27330610
chr11_4657	p3	(ACA)5	15	27356250	27356264
chr11_4658	p2	(GA)7	14	27365480	27365493
chr11_4659	p2	(AC)6	12	27368645	27368656
chr11_4660	p2	(TG)9	18	27375104	27375121
chr11_4661	p2	(CA)6	12	27388454	27388465
chr11_4662	p2	(GT)15	30	27390481	27390510
chr11_4663	p2	(TG)22	44	27392744	27392787
chr11_4664	p2	(GT)10	20	27393610	27393629
chr11_4665	p2	(GA)6	12	27397342	27397353
chr11_4666	c	(TG)22(AG)12	68	27397625	27397692
chr11_4667	p2	(AC)12	24	27399840	27399863
chr11_4668	p2	(TG)11	22	27401973	27401994
chr11_4669	c	(GT)11gagagg(GA)6	40	27402799	27402838
chr11_4670	p2	(TG)22	44	27408635	27408678
chr11_4671	c	(CA)30c(CA)6	73	27410641	27410713
chr11_4672	p2	(AC)6	12	27418482	27418493
chr11_4673	p4	(AAAT)5	20	27423625	27423644
chr11_4674	p2	(TG)17	34	27423947	27423980
chr11_4675	p2	(AT)6	12	27425245	27425256
chr11_4676	p3	(TGC)5	15	27426617	27426631
chr11_4677	p2	(CA)16	32	27432632	27432663
chr11_4678	p4	(AATC)5	20	27437533	27437552
chr11_4679	p2	(CA)7	14	27454566	27454579
chr11_4680	p2	(AG)10	20	27454922	27454941
chr11_4681	p3	(TTG)6	18	27461392	27461409
chr11_4682	p2	(CA)18	36	27464176	27464211
chr11_4683	p2	(AC)6	12	27464488	27464499
chr11_4684	p3	(TTA)6	18	27475278	27475295
chr11_4685	p2	(TG)12	24	27482407	27482430
chr11_4686	p2	(TG)10	20	27485952	27485971
chr11_4687	p2	(CA)7	14	27491594	27491607
chr11_4688	p2	(CA)7	14	27498226	27498239
chr11_4689	p2	(TG)12	24	27502897	27502920
chr11_4690	p2	(CA)9	18	27509259	27509276
chr11_4691	p2	(CA)10	20	27511332	27511351
chr11_4692	p2	(TG)14	28	27515749	27515776
chr11_4693	p2	(GT)8	16	27516268	27516283

chr11_4694	p2	(CA)13	26	27519925	27519950
chr11_4695	p3	(GAG)6	18	27523556	27523573
chr11_4696	p2	(GT)14	28	27525244	27525271
chr11_4697	p2	(TG)9	18	27526293	27526310
chr11_4698	p2	(GT)26	52	27541023	27541074
chr11_4699	p2	(CA)6	12	27543506	27543517
chr11_4700	c	acagagataacgcagagagggtgcaaagaggtgtcagggacagaaaac	125	27544118	27544242
chr11_4701	p2	(TG)24	48	27544426	27544473
chr11_4702	p2	(TG)21	42	27544640	27544681
chr11_4703	p2	(AG)15	30	27545534	27545563
chr11_4704	c	(TG)16ttcatactgtacaagagaaa(GT)6	64	27546822	27546885
chr11_4705	p2	(AG)11	22	27548615	27548636
chr11_4706	p2	(CA)12	24	27550496	27550519
chr11_4707	p2	(AC)6	12	27551724	27551735
chr11_4708	p2	(AC)10	20	27553506	27553525
chr11_4709	p2	(CA)9	18	27554114	27554131
chr11_4710	c	gcatggactgtcactgtaagtctgacagcgtcaatagcaagatgacgact	173	27556201	27556373
chr11_4711	p2	(GA)8	16	27556935	27556950
chr11_4712	p2	(CA)14	28	27557228	27557255
chr11_4713	p2	(AC)7	14	27558357	27558370
chr11_4714	p2	(AC)15	30	27558741	27558770
chr11_4715	p2	(TC)6	12	27559369	27559380
chr11_4716	p2	(AG)13	26	27561387	27561412
chr11_4717	c	A)10cgtgcaacactgatgcattcacacagacacgcaaaccaac(CA)	90	27562857	27562946
chr11_4718	p2	(TG)9	18	27563592	27563609
chr11_4719	p2	(AG)11	22	27563831	27563852
chr11_4720	p3	(GGA)9	27	27564339	27564365
chr11_4721	p2	(AC)7	14	27564625	27564638
chr11_4722	p2	(GT)6	12	27565708	27565719
chr11_4723	p2	(AC)6	12	27567970	27567981
chr11_4724	c	GT)10cctagcagaggctgtgtctgtaagagagcgacaggaacgaggc	417	27569553	27569969
chr11_4725	p2	(AC)12	24	27570522	27570545
chr11_4726	p2	(CA)8	16	27571803	27571818
chr11_4727	p3	(ACT)16	48	27572367	27572414
chr11_4728	p2	(CA)12	24	27573115	27573138
chr11_4729	p2	(GT)10	20	27573402	27573421
chr11_4730	p2	(TG)6	12	27573592	27573603
chr11_4731	p2	(AC)7	14	27574187	27574200
chr11_4732	p2	(CA)8	16	27575441	27575456
chr11_4733	p2	(AG)6	12	27576063	27576074
chr11_4734	p3	(GGT)5	15	27577238	27577252
chr11_4735	p4	(GAAA)7	28	27578536	27578563
chr11_4736	p2	(AC)6	12	27590254	27590265
chr11_4737	p2	(TG)6	12	27591682	27591693
chr11_4738	p2	(GT)26	52	27595816	27595867
chr11_4739	p2	(AC)11	22	27597195	27597216
chr11_4740	c	gtgtgggcaatttcctgtgtttacaattttacactcacacatgtgggcacatct	125	27597335	27597459
chr11_4741	p2	(GT)12	24	27597786	27597809
chr11_4742	p2	(AG)9	18	27608522	27608539
chr11_4743	p2	(AC)11	22	27614463	27614484
chr11_4744	p2	(CA)7	14	27614887	27614900
chr11_4745	p2	(TG)23	46	27616744	27616789

chr11_4746	p5	(AAATC)5	25	27629658	27629682
chr11_4747	p2	(GT)18	36	27642075	27642110
chr11_4748	p2	(TG)6	12	27646344	27646355
chr11_4749	p2	(GT)6	12	27653470	27653481
chr11_4750	p2	(AG)10	20	27654243	27654262
chr11_4751	p3	(ATG)7	21	27659713	27659733
chr11_4752	p2	(CA)6	12	27661170	27661181
chr11_4753	p2	(TG)6	12	27671252	27671263
chr11_4754	p3	(GTG)5	15	27697517	27697531
chr11_4755	p2	(TG)6	12	27701538	27701549
chr11_4756	p2	(AC)8	16	27714314	27714329
chr11_4757	p2	(TA)6	12	27715619	27715630
chr11_4758	c	(CA)8g(AC)12	41	27717362	27717402
chr11_4759	p2	(TA)6	12	27732408	27732419
chr11_4760	p2	(AC)20	40	27734932	27734971
chr11_4761	p3	(AAT)5	15	27735347	27735361
chr11_4762	p2	(GA)8	16	27744581	27744596
chr11_4763	p2	(AG)8	16	27753393	27753408
chr11_4764	p2	(CA)7	14	27753886	27753899
chr11_4765	p2	(TG)11	22	27757190	27757211
chr11_4766	p2	(AT)7	14	27764631	27764644
chr11_4767	p2	(AC)23	46	27766696	27766741
chr11_4768	p2	(TA)7	14	27786439	27786452
chr11_4769	p2	(CA)11	22	27786978	27786999
chr11_4770	p2	(AC)13	26	27789143	27789168
chr11_4771	p2	(CA)6	12	27796483	27796494
chr11_4772	p2	(TG)6	12	27803459	27803470
chr11_4773	c	(TC)6tgtctg(TC)9	36	27806121	27806156
chr11_4774	p2	(GA)25	50	27808448	27808497
chr11_4775	p2	(AC)6	12	27809789	27809800
chr11_4776	p2	(GT)15	30	27810834	27810863
chr11_4777	p2	(CA)20	40	27810993	27811032
chr11_4778	p2	(TG)8	16	27813750	27813765
chr11_4779	p2	(CT)18	36	27821982	27822017
chr11_4780	c	(ATT)8atgatc(ATT)6	48	27828486	27828533
chr11_4781	p2	(AG)6	12	27829844	27829855
chr11_4782	p4	(AAAG)5	20	27832348	27832367
chr11_4783	p2	(TG)14	28	27833744	27833771
chr11_4784	p2	(TG)23	46	27839095	27839140
chr11_4785	p4	(AAAT)5	20	27839342	27839361
chr11_4786	p2	(GT)6	12	27843936	27843947
chr11_4787	p2	(GT)6	12	27848128	27848139
chr11_4788	p2	(AC)9	18	27849052	27849069
chr11_4789	p2	(CA)10	20	27849250	27849269
chr11_4790	p2	(AG)8	16	27852066	27852081
chr11_4791	p2	(TC)9	18	27852975	27852992
chr11_4792	p2	(CA)13	26	27853564	27853589
chr11_4793	p2	(AC)13	26	27855120	27855145
chr11_4794	p2	(CA)19	38	27859492	27859529
chr11_4795	p2	(AC)8	16	27859954	27859969
chr11_4796	p2	(GT)8	16	27861812	27861827
chr11_4797	p2	(AC)7	14	27863565	27863578

chr11_4798	p3	(GAG)5	15	27865285	27865299
chr11_4799	p4	(AAAC)6	24	27870077	27870100
chr11_4800	p3	(ACC)5	15	27872639	27872653
chr11_4801	p2	(CT)12	24	27877303	27877326
chr11_4802	p2	(TG)6	12	27878588	27878599
chr11_4803	p2	(AC)7	14	27881039	27881052
chr11_4804	p2	(CA)10	20	27881201	27881220
chr11_4805	p2	(CA)8	16	27885094	27885109
chr11_4806	p2	(TA)7	14	27900130	27900143
chr11_4807	p2	(AT)8	16	27903694	27903709
chr11_4808	p3	(GGA)5	15	27962339	27962353
chr11_4809	p2	(AC)12	24	27977049	27977072
chr11_4810	p2	(GT)6	12	27977210	27977221
chr11_4811	p2	(GT)15	30	27982848	27982877
chr11_4812	c	(GAT)5(TAT)7	36	27997553	27997588
chr11_4813	p2	(CT)6	12	27997938	27997949
chr11_4814	p4	(AGAT)18	72	28004431	28004502
chr11_4815	p2	(TG)12	24	28005976	28005999
chr11_4816	p2	(TG)12	24	28008505	28008528
chr11_4817	p2	(GT)6	12	28027621	28027632
chr11_4818	p2	(AC)7	14	28028483	28028496
chr11_4819	p2	(AT)6	12	28051139	28051150
chr11_4820	p2	(TA)6	12	28052976	28052987
chr11_4821	p2	(AG)10	20	28065787	28065806
chr11_4822	p2	(CA)10	20	28068084	28068103
chr11_4823	p4	(TTCA)5	20	28077780	28077799
chr11_4824	p2	(GT)8	16	28080961	28080976
chr11_4825	p2	(GT)6	12	28108767	28108778
chr11_4826	p2	(CA)7	14	28127691	28127704
chr11_4827	p4	(CATC)6	24	28132934	28132957
chr11_4828	p2	(CT)6	12	28168995	28169006
chr11_4829	p4	(TGTT)5	20	28221149	28221168
chr11_4830	p2	(AC)6	12	28229143	28229154
chr11_4831	p2	(AC)6	12	28230235	28230246
chr11_4832	p5	(AAATC)5	25	28236956	28236980
chr11_4833	p2	(TG)8	16	28244914	28244929
chr11_4834	p2	(AC)10	20	28263727	28263746
chr11_4835	p4	(AATA)5	20	28283256	28283275
chr11_4836	p4	(TGTT)5	20	28284038	28284057
chr11_4837	p2	(AT)7	14	28288034	28288047
chr11_4838	p2	(TG)11	22	28290644	28290665
chr11_4839	p2	(TG)10	20	28294037	28294056
chr11_4840	p2	(CA)14	28	28295365	28295392
chr11_4841	p2	(AC)12	24	28302889	28302912
chr11_4842	p2	(AC)7	14	28309984	28309997
chr11_4843	p5	(TTTGA)5	25	28317513	28317537
chr11_4844	p2	(AC)7	14	28317722	28317735
chr11_4845	p2	(AC)6	12	28327205	28327216
chr11_4846	p2	(AC)6	12	28337472	28337483
chr11_4847	p2	(GT)6	12	28340812	28340823
chr11_4848	p2	(CA)11	22	28340931	28340952
chr11_4849	p2	(CA)17	34	28342008	28342041

chr11_4850	p2	(TG)10	20	28345134	28345153
chr11_4851	p2	(AC)7	14	28348983	28348996
chr11_4852	p5	(AAATC)5	25	28356080	28356104
chr11_4853	p3	(GAG)5	15	28359591	28359605
chr11_4854	p2	(GT)18	36	28363097	28363132
chr11_4855	p2	(GT)6	12	28368687	28368698
chr11_4856	p2	(GT)10	20	28375640	28375659
chr11_4857	p2	(GT)17	34	28379442	28379475
chr11_4858	p4	(ATGG)5	20	28383471	28383490
chr11_4859	p2	(AC)6	12	28400195	28400206
chr11_4860	p2	(GT)16	32	28404031	28404062
chr11_4861	p3	(CTC)6	18	28407868	28407885
chr11_4862	p2	(AC)7	14	28415148	28415161
chr11_4863	p2	(TC)6	12	28423702	28423713
chr11_4864	p2	(AG)10	20	28431515	28431534
chr11_4865	p2	(AC)12	24	28442794	28442817
chr11_4866	p2	(AC)9	18	28467763	28467780
chr11_4867	p2	(CA)6	12	28479114	28479125
chr11_4868	p3	(AAT)5	15	28494856	28494870
chr11_4869	p2	(TC)6	12	28497853	28497864
chr11_4870	c	(AT)8(AC)9	34	28501327	28501360
chr11_4871	p2	(AT)8	16	28502931	28502946
chr11_4872	p2	(CT)7	14	28509935	28509948
chr11_4873	p2	(AG)6	12	28525629	28525640
chr11_4874	p2	(TC)6	12	28526430	28526441
chr11_4875	p2	(CA)19	38	28547212	28547249
chr11_4876	p4	(AACA)6	24	28549729	28549752
chr11_4877	p2	(GA)20	40	28551464	28551503
chr11_4878	p2	(TG)13	26	28558209	28558234
chr11_4879	p2	(TG)17	34	28563869	28563902
chr11_4880	p2	(AC)13	26	28564709	28564734
chr11_4881	p2	(TG)6	12	28565228	28565239
chr11_4882	p2	(GT)7	14	28583597	28583610
chr11_4883	c	(CA)14cctgccaaaacataaacaaaatacatgcac(GT)6	70	28597761	28597830
chr11_4884	p2	(TA)7	14	28604124	28604137
chr11_4885	p2	(AC)6	12	28608735	28608746
chr11_4886	p2	(GA)7	14	28621524	28621537
chr11_4887	p3	(ATT)7	21	28632984	28633004
chr11_4888	p2	(TG)13	26	28633199	28633224
chr11_4889	p2	(GA)9	18	28640837	28640854
chr11_4890	p2	(AT)7	14	28668968	28668981
chr11_4891	p2	(TA)7	14	28675611	28675624
chr11_4892	p2	(GT)7	14	28676043	28676056
chr11_4893	p2	(TC)7	14	28677466	28677479
chr11_4894	c	[illegible]T)5gaaaaaacacacagtcatccataataagcctgatagtgtggata(T[illegible]	83	28684523	28684605
chr11_4895	p2	(TG)6	12	28684748	28684759
chr11_4896	p2	(AG)9	18	28687992	28688009
chr11_4897	p3	(CTT)5	15	28702391	28702405
chr11_4898	p2	(CA)6	12	28704921	28704932
chr11_4899	p2	(CA)28	56	28712987	28713042
chr11_4900	p3	(GCT)6	18	28729955	28729972
chr11_4901	p2	(GA)6	12	28744613	28744624

chr11_4902	p3	(ATA)5	15	28765870	28765884
chr11_4903	p3	(TGA)5	15	28779368	28779382
chr11_4904	c	(CA)6tacatcaaatattcgcagcctggatatttctgttt(CA)11	69	28789097	28789165
chr11_4905	p2	(CA)15	30	28796742	28796771
chr11_4906	p2	(TA)8	16	28798379	28798394
chr11_4907	c	[illegible]ctcgcctctatattccgctattacagatcactaactttgtgtctcctctctcccg[illegible]	111	28815097	28815207
chr11_4908	p3	(ATA)6	18	28815510	28815527
chr11_4909	p2	(GT)16	32	28824193	28824224
chr11_4910	p2	(AC)6	12	28824452	28824463
chr11_4911	p2	(TG)6	12	28833067	28833078
chr11_4912	p2	(CA)22	44	28834493	28834536
chr11_4913	p2	(TG)6	12	28841300	28841311
chr11_4914	p2	(TA)7	14	28850210	28850223
chr11_4915	p2	(TG)14	28	28866356	28866383
chr11_4916	p3	(AAG)5	15	28873926	28873940
chr11_4917	p2	(AC)6	12	28874166	28874177
chr11_4918	p2	(TG)6	12	28875499	28875510
chr11_4919	p2	(TG)13	26	28888945	28888970
chr11_4920	p2	(GT)12	24	28890924	28890947
chr11_4921	p2	(TG)10	20	28907546	28907565
chr11_4922	p4	(TGTC)6	24	28909793	28909816
chr11_4923	p2	(GT)7	14	28926654	28926667
chr11_4924	p2	(GT)11	22	28942490	28942511
chr11_4925	p2	(GT)20	40	28945838	28945877
chr11_4926	p2	(CA)6	12	28946026	28946037
chr11_4927	p4	(CTAC)6	24	28953892	28953915
chr11_4928	p2	(AC)6	12	28955318	28955329
chr11_4929	p2	(AC)6	12	28962131	28962142
chr11_4930	p2	(CA)11	22	28962584	28962605
chr11_4931	p2	(TG)8	16	28965442	28965457
chr11_4932	p2	(CA)14	28	28975106	28975133
chr11_4933	p2	(GT)14	28	28976467	28976494
chr11_4934	p3	(ATT)6	18	28983824	28983841
chr11_4935	p4	(TAAA)5	20	28997397	28997416
chr11_4936	c	(GT)6ctctctctct(CA)12	46	29006707	29006752
chr11_4937	p2	(TA)6	12	29007959	29007970
chr11_4938	p3	(AGC)7	21	29009654	29009674
chr11_4939	p2	(AT)6	12	29022976	29022987
chr11_4940	p3	(TAT)5	15	29042160	29042174
chr11_4941	p2	(TC)8	16	29063000	29063015
chr11_4942	p3	(TTA)6	18	29070958	29070975
chr11_4943	p2	(AG)7	14	29071081	29071094
chr11_4944	c	(AT)8(AC)7	30	29075014	29075043
chr11_4945	p2	(CA)12	24	29079606	29079629
chr11_4946	p2	(AC)8	16	29081449	29081464
chr11_4947	p2	(CT)6	12	29095158	29095169
chr11_4948	p2	(CA)21	42	29098874	29098915
chr11_4949	p2	(AC)9	18	29102141	29102158
chr11_4950	p2	(AC)6	12	29103644	29103655
chr11_4951	p2	(CA)6	12	29107673	29107684
chr11_4952	p2	(GT)9	18	29110280	29110297
chr11_4953	p2	(AC)6	12	29110449	29110460

chr11_4954	p3	(GTT)5	15	29112839	29112853
chr11_4955	p3	(TAT)7	21	29120035	29120055
chr11_4956	p2	(AC)8	16	29121561	29121576
chr11_4957	p2	(TG)13	26	29126242	29126267
chr11_4958	p3	(TAT)5	15	29130555	29130569
chr11_4959	p2	(AC)15	30	29131761	29131790
chr11_4960	p2	(GT)14	28	29134248	29134275
chr11_4961	p4	(ATGG)8	32	29143692	29143723
chr11_4962	p2	(GT)12	24	29149680	29149703
chr11_4963	p2	(AC)17	34	29151113	29151146
chr11_4964	p2	(TG)12	24	29153154	29153177
chr11_4965	p2	(CA)12	24	29154271	29154294
chr11_4966	p2	(GT)10	20	29163529	29163548
chr11_4967	p2	(AC)8	16	29170166	29170181
chr11_4968	c	(CA)21ccacttcttttcttgactgaggcatccctcggctttt(TTG)8	103	29174114	29174216
chr11_4969	p2	(AC)7	14	29176583	29176596
chr11_4970	p2	(AG)8	16	29181672	29181687
chr11_4971	p2	(AC)7	14	29181937	29181950
chr11_4972	p2	(GA)6	12	29186276	29186287
chr11_4973	p2	(GA)6	12	29191984	29191995
chr11_4974	p2	(CA)6	12	29193506	29193517
chr11_4975	p2	(CA)23	46	29201366	29201411
chr11_4976	c	ctgaatatgagcaactgtttgagtggggcattgaacaatataagcactt(TC	166	29201736	29201901
chr11_4977	p2	(AC)8	16	29209896	29209911
chr11_4978	p4	(AGGC)6	24	29213958	29213981
chr11_4979	p2	(CA)9	18	29214197	29214214
chr11_4980	p2	(CA)12	24	29219727	29219750
chr11_4981	p2	(AC)8	16	29221124	29221139
chr11_4982	p2	(CA)7	14	29224601	29224614
chr11_4983	p2	(CA)8	16	29228915	29228930
chr11_4984	p2	(AC)10	20	29235117	29235136
chr11_4985	p2	(AC)22	44	29238018	29238061
chr11_4986	p2	(GT)7	14	29238520	29238533
chr11_4987	p2	(AC)8	16	29238896	29238911
chr11_4988	p2	(CT)8	16	29241015	29241030
chr11_4989	p3	(TCT)7	21	29250478	29250498
chr11_4990	c	(TC)6(AC)20	52	29256229	29256280
chr11_4991	p2	(AG)13	26	29259939	29259964
chr11_4992	p2	(GT)15	30	29264469	29264498
chr12_1	p3	(TTA)7	21	6215	6235
chr12_2	p3	(TCC)6	18	9946	9963
chr12_3	p2	(TC)6	12	16727	16738
chr12_4	p2	(TG)7	14	51860	51873
chr12_5	p2	(AC)7	14	54531	54544
chr12_6	p3	(AAG)5	15	59867	59881
chr12_7	p2	(AT)10	20	63101	63120
chr12_8	p2	(GT)9	18	73783	73800
chr12_9	p2	(GT)6	12	83138	83149
chr12_10	p2	(GT)6	12	90992	91003
chr12_11	p2	(GT)6	12	91939	91950
chr12_12	p2	(AC)6	12	100936	100947
chr12_13	p2	(GA)28	56	110474	110529

chr12_14	p4	(AATG)6	24	115468	115491
chr12_15	p2	(GT)19	38	121444	121481
chr12_16	p2	(GA)9	18	124638	124655
chr12_17	c	(TC)12(AC)11	46	129246	129291
chr12_18	p2	(TG)32	64	178775	178838
chr12_19	p2	(GT)6	12	180699	180710
chr12_20	p3	(AAG)6	18	182512	182529
chr12_21	p2	(CT)6	12	184674	184685
chr12_22	p3	(TGC)6	18	189711	189728
chr12_23	p2	(CT)15	30	191908	191937
chr12_24	p3	(TAT)5	15	224682	224696
chr12_25	p2	(CA)7	14	233506	233519
chr12_26	p3	(GGT)6	18	248157	248174
chr12_27	p2	(TC)6	12	251416	251427
chr12_28	p2	(AC)6	12	254798	254809
chr12_29	p4	(AATC)5	20	258612	258631
chr12_30	p2	(CA)8	16	265948	265963
chr12_31	p2	(CA)8	16	269368	269383
chr12_32	p2	(CA)8	16	272536	272551
chr12_33	p2	(AG)6	12	279967	279978
chr12_34	p2	(CA)18	36	284168	284203
chr12_35	p2	(CA)20	40	286828	286867
chr12_36	p2	(GA)14	28	292501	292528
chr12_37	p5	(AATCA)5	25	312663	312687
chr12_38	p2	(TG)13	26	324527	324552
chr12_39	p2	(GT)11	22	337113	337134
chr12_40	p2	(AC)6	12	348783	348794
chr12_41	c	(TG)8(GT)19	54	355510	355563
chr12_42	p3	(GCA)5	15	356964	356978
chr12_43	p3	(TTA)11	33	359590	359622
chr12_44	p2	(TA)6	12	382654	382665
chr12_45	p2	(AC)15	30	403748	403777
chr12_46	p3	(GTT)6	18	404109	404126
chr12_47	p2	(TG)6	12	405966	405977
chr12_48	p2	(CA)10	20	409061	409080
chr12_49	p2	(GT)25	50	414830	414879
chr12_50	p2	(GT)13	26	416635	416660
chr12_51	p2	(CA)12	24	423818	423841
chr12_52	c	(CT)9g(TC)7	33	425351	425383
chr12_53	p2	(CT)6	12	447022	447033
chr12_54	p2	(AT)6	12	462753	462764
chr12_55	p2	(AC)6	12	490448	490459
chr12_56	p2	(TG)6	12	503152	503163
chr12_57	p3	(TTG)6	18	512384	512401
chr12_58	p4	(AATG)6	24	512792	512815
chr12_59	p2	(AT)12	24	518839	518862
chr12_60	p3	(ATT)17	51	529735	529785
chr12_61	p2	(CA)6	12	534171	534182
chr12_62	c	(TG)8(AG)26	68	535765	535832
chr12_63	p3	(TTG)5	15	538103	538117
chr12_64	p2	(TG)7	14	557728	557741
chr12_65	p2	(TG)6	12	558685	558696

chr12_66	p3	(ATG)5	15	558864	558878
chr12_67	p3	(ATA)5	15	563971	563985
chr12_68	p2	(CA)7	14	564487	564500
chr12_69	p3	(AAT)6	18	568338	568355
chr12_70	p2	(CA)28	56	592907	592962
chr12_71	p2	(GA)6	12	599285	599296
chr12_72	p2	(CA)13	26	610298	610323
chr12_73	p4	(AAAC)5	20	614872	614891
chr12_74	p2	(TG)8	16	615642	615657
chr12_75	p2	(TG)14	28	624466	624493
chr12_76	p2	(CA)18	36	626355	626390
chr12_77	p5	(AAATC)6	30	633586	633615
chr12_78	p2	(AC)6	12	634275	634286
chr12_79	p2	(TG)6	12	634596	634607
chr12_80	p2	(GT)10	20	638596	638615
chr12_81	c	aaggtagcaccagggggactaaggtcagccaatgttctacatgggatcta	126	638882	639007
chr12_82	c	(AC)11at(AC)7	38	640999	641036
chr12_83	p2	(CA)9	18	643759	643776
chr12_84	p2	(TC)8	16	648795	648810
chr12_85	c	(GA)7gg(GA)7	30	651384	651413
chr12_86	p2	(CA)10	20	659514	659533
chr12_87	p2	(AC)6	12	661888	661899
chr12_88	p4	(AAAG)5	20	665889	665908
chr12_89	p3	(ACT)6	18	668044	668061
chr12_90	p3	(TAC)7	21	668239	668259
chr12_91	p2	(TG)15	30	669174	669203
chr12_92	p2	(AC)8	16	669316	669331
chr12_93	p2	(AC)7	14	681113	681126
chr12_94	p2	(CT)6	12	683288	683299
chr12_95	p2	(AC)9	18	690604	690621
chr12_96	p2	(AC)25	50	699915	699964
chr12_97	p2	(CA)7	14	701614	701627
chr12_98	p2	(AC)16	32	702151	702182
chr12_99	p2	(GT)12	24	704543	704566
chr12_100	p2	(CA)13	26	705001	705026
chr12_101	p3	(TCA)5	15	710522	710536
chr12_102	p2	(TG)6	12	711317	711328
chr12_103	p3	(TCA)6	18	711651	711668
chr12_104	p2	(CA)18	36	712061	712096
chr12_105	c	catgacaacattaccacttgcagctgctctgcattctcacggtctgccctgac	127	712329	712455
chr12_106	p3	(TTA)5	15	719113	719127
chr12_107	p2	(CA)26	52	727980	728031
chr12_108	p3	(AAT)8	24	728997	729020
chr12_109	p2	(TA)6	12	732375	732386
chr12_110	p2	(GT)6	12	736384	736395
chr12_111	p3	(AAG)7	21	738125	738145
chr12_112	p2	(TG)6	12	740851	740862
chr12_113	p2	(AC)13	26	747030	747055
chr12_114	p2	(AC)9	18	750000	750017
chr12_115	p2	(GT)16	32	756638	756669
chr12_116	p2	(GT)17	34	759147	759180
chr12_117	p2	(TG)6	12	759415	759426

chr12_118	c	(AAC)8tctttcacttacactaaagcacacagacacacg(CA)7	71	761070	761140
chr12_119	p2	(CA)11	22	767531	767552
chr12_120	p3	(TAC)12	36	780279	780314
chr12_121	p4	(CTCC)5	20	785592	785611
chr12_122	p2	(AC)6	12	787742	787753
chr12_123	p2	(GT)10	20	807681	807700
chr12_124	p2	(AC)6	12	811398	811409
chr12_125	p2	(TC)7	14	811999	812012
chr12_126	c	(TA)6c(AT)9	31	820413	820443
chr12_127	p2	(TA)8	16	822055	822070
chr12_128	p2	(CA)8	16	825827	825842
chr12_129	p2	(AC)8	16	836822	836837
chr12_130	p2	(CA)6	12	853126	853137
chr12_131	p2	(TG)10	20	859281	859300
chr12_132	p3	(TGA)6	18	862091	862108
chr12_133	p2	(GT)13	26	869485	869510
chr12_134	p2	(TG)17	34	869643	869676
chr12_135	p3	(AAT)8	24	870548	870571
chr12_136	p2	(GT)9	18	870795	870812
chr12_137	p2	(CT)7	14	871288	871301
chr12_138	p2	(CT)11	22	872201	872222
chr12_139	p2	(TG)11	22	874038	874059
chr12_140	p2	(GA)9	18	875522	875539
chr12_141	p2	(TG)10	20	878747	878766
chr12_142	p3	(TTC)5	15	882270	882284
chr12_143	p2	(AG)6	12	882614	882625
chr12_144	p2	(GT)11	22	883790	883811
chr12_145	p2	(GT)6	12	884064	884075
chr12_146	c	gcacagcacagcctgtgccggcttctctaattggctgctgatggtttatgaa	122	889135	889256
chr12_147	c	gaatgagtggagagggttaagataacagcgaggggcagagagacaccta	83	892711	892793
chr12_148	p2	(GT)9	18	895143	895160
chr12_149	p2	(AC)9	18	901174	901191
chr12_150	p2	(TC)6	12	901322	901333
chr12_151	p4	(CTGG)6	24	908683	908706
chr12_152	p2	(TG)15	30	909730	909759
chr12_153	p2	(TG)6	12	909912	909923
chr12_154	c	(AC)7tctcttattcaggtgtgcg(CA)17	67	914226	914292
chr12_155	p2	(GT)8	16	922103	922118
chr12_156	p2	(CT)9	18	928545	928562
chr12_157	p2	(TG)6	12	931997	932008
chr12_158	p2	(CA)6	12	932313	932324
chr12_159	p2	(AC)16	32	932938	932969
chr12_160	c	(AC)8t(CA)11	39	933106	933144
chr12_161	c	(TG)6tccgtga(GT)6	31	940260	940290
chr12_162	p2	(TC)6	12	957432	957443
chr12_163	p2	(AC)7	14	963304	963317
chr12_164	c	(CA)6ggcctagagaaactat(AC)18	64	964608	964671
chr12_165	p2	(AC)6	12	967918	967929
chr12_166	p2	(CA)7	14	969443	969456
chr12_167	p2	(CA)17	34	969599	969632
chr12_168	p2	(CA)6	12	972206	972217
chr12_169	p2	(AC)6	12	972616	972627

chr12_170	p2	(CT)8	16	972958	972973
chr12_171	p2	(TG)6	12	976662	976673
chr12_172	p2	(GA)16	32	984729	984760
chr12_173	p2	(TG)11	22	984862	984883
chr12_174	p2	(TG)13	26	986971	986996
chr12_175	c	)8aggagagactgatctatatatatggatctgtgtgcatgtgtagtgtgtct(T	84	987100	987183
chr12_176	p2	(CA)9	18	987314	987331
chr12_177	p2	(CA)6	12	987702	987713
chr12_178	p2	(AC)7	14	989728	989741
chr12_179	p2	(GT)15	30	993383	993412
chr12_180	p2	(TC)6	12	993685	993696
chr12_181	p2	(TC)7	14	993949	993962
chr12_182	p2	(TG)12	24	995952	995975
chr12_183	p2	(CA)13	26	997694	997719
chr12_184	p2	(AC)6	12	997864	997875
chr12_185	p2	(CA)8	16	998094	998109
chr12_186	p2	(GT)26	52	1000196	1000247
chr12_187	p2	(AC)9	18	1002110	1002127
chr12_188	p2	(TG)9	18	1003259	1003276
chr12_189	c	(TG)9tcagg(GA)8	39	1005122	1005160
chr12_190	p2	(GT)6	12	1005466	1005477
chr12_191	p2	(CA)17	34	1005893	1005926
chr12_192	p2	(AC)17	34	1006246	1006279
chr12_193	p3	(TAA)5	15	1006433	1006447
chr12_194	p2	(CA)9	18	1007571	1007588
chr12_195	p2	(CA)14	28	1007784	1007811
chr12_196	c	(CA)6cttgcaagctcatgcacactgtctttcttgcttccaca(AC)16	82	1009810	1009891
chr12_197	p2	(AG)6	12	1011503	1011514
chr12_198	p2	(CA)14	28	1014154	1014181
chr12_199	c	ıtatggatccatgtgtccgtctctctgtctgttcctgtctgtacatatgtggggg	126	1018158	1018283
chr12_200	p2	(AC)10	20	1019844	1019863
chr12_201	p2	(GA)6	12	1020173	1020184
chr12_202	p2	(AC)10	20	1022207	1022226
chr12_203	p2	(AC)6	12	1022463	1022474
chr12_204	c	(AC)7atacatgcc(TG)7	37	1023817	1023853
chr12_205	p2	(TG)6	12	1024127	1024138
chr12_206	c	(TG)6tcagt(GA)9gttagcttatctgcttactggcc(CA)9	76	1028809	1028884
chr12_207	c	(GT)16atgtatgtat(GA)6	54	1029660	1029713
chr12_208	p2	(CA)15	30	1029850	1029879
chr12_209	p2	(CA)6	12	1032386	1032397
chr12_210	p2	(GT)9	18	1046861	1046878
chr12_211	p2	(GT)10	20	1048740	1048759
chr12_212	p2	(CA)14	28	1050272	1050299
chr12_213	p2	(CA)13	26	1055688	1055713
chr12_214	p2	(AC)7	14	1059542	1059555
chr12_215	p4	(TGAT)7	28	1070068	1070095
chr12_216	p2	(TA)6	12	1071677	1071688
chr12_217	p3	(TAT)5	15	1076574	1076588
chr12_218	p2	(AT)8	16	1089051	1089066
chr12_219	p3	(ATT)6	18	1108158	1108175
chr12_220	c	(GT)7ga(GT)10	36	1108898	1108933
chr12_221	p2	(TG)6	12	1114167	1114178

chr12_222	p2	(AC)15	30	1126695	1126724
chr12_223	p2	(TG)9	18	1131291	1131308
chr12_224	p2	(GT)9	18	1131440	1131457
chr12_225	p2	(CA)6	12	1132664	1132675
chr12_226	p2	(CA)6	12	1137710	1137721
chr12_227	p2	(TG)9	18	1144102	1144119
chr12_228	p2	(AC)19	38	1151219	1151256
chr12_229	p3	(GAA)6	18	1156726	1156743
chr12_230	p2	(TC)7	14	1165215	1165228
chr12_231	p2	(CA)23	46	1172868	1172913
chr12_232	p2	(TG)8	16	1173885	1173900
chr12_233	p2	(AC)14	28	1177403	1177430
chr12_234	p2	(AC)14	28	1183607	1183634
chr12_235	p2	(CA)7	14	1202827	1202840
chr12_236	p2	(AC)8	16	1207422	1207437
chr12_237	p2	(CA)15	30	1210453	1210482
chr12_238	p2	(AC)14	28	1215773	1215800
chr12_239	p2	(AC)9	18	1216840	1216857
chr12_240	p3	(AAC)9	27	1224172	1224198
chr12_241	p2	(AG)28	56	1225361	1225416
chr12_242	p2	(AC)24	48	1238724	1238771
chr12_243	p2	(TG)6	12	1239442	1239453
chr12_244	p2	(GA)6	12	1245936	1245947
chr12_245	c	(TG)7c(GT)9	33	1251900	1251932
chr12_246	p2	(TG)10	20	1262286	1262305
chr12_247	p2	(TG)8	16	1270611	1270626
chr12_248	c	4cgagggga(AG)14cgaggggga(AG)14cggagc(GA)10a	141	1274640	1274780
chr12_249	p4	(AATC)5	20	1279758	1279777
chr12_250	p4	(TCAT)5	20	1297738	1297757
chr12_251	p2	(CT)6	12	1311526	1311537
chr12_252	p3	(CTG)6	18	1312523	1312540
chr12_253	p2	(AT)11	22	1314089	1314110
chr12_254	p3	(ATA)5	15	1315296	1315310
chr12_255	c	(TC)9tg(TC)9	38	1337623	1337660
chr12_256	p2	(GT)8	16	1342551	1342566
chr12_257	p2	(TG)6	12	1343792	1343803
chr12_258	c	(AC)6(ATT)6	30	1350517	1350546
chr12_259	p3	(TCA)6	18	1350695	1350712
chr12_260	p3	(TAA)6	18	1353368	1353385
chr12_261	p2	(CA)6	12	1354670	1354681
chr12_262	p2	(TG)6	12	1359083	1359094
chr12_263	p2	(TG)13	26	1381314	1381339
chr12_264	c	gaagttattgtgaatgaaagcctggcatgttaaacaacacagtaaactgaaa	181	1381503	1381683
chr12_265	c	gaagttattgtgaatgaaagcctggcatgttaaacaacacagtaaactgaaa	173	1383057	1383229
chr12_266	p2	(GA)6	12	1390395	1390406
chr12_267	p3	(TTG)5	15	1404217	1404231
chr12_268	p2	(TG)11	22	1423984	1424005
chr12_269	p2	(AG)7	14	1426281	1426294
chr12_270	p2	(AT)8	16	1430681	1430696
chr12_271	p4	(GTAT)5	20	1435876	1435895
chr12_272	p2	(GT)28	56	1441745	1441800
chr12_273	p3	(TAA)5	15	1485694	1485708

chr12_274	p3	(ATA)7	21	1493014	1493034
chr12_275	p4	(ATTT)5	20	1495947	1495966
chr12_276	p3	(TGT)9	27	1526514	1526540
chr12_277	p3	(CTG)5	15	1526710	1526724
chr12_278	p2	(AC)10	20	1529192	1529211
chr12_279	p2	(TG)10	20	1539938	1539957
chr12_280	p2	(AC)9	18	1573747	1573764
chr12_281	p2	(TC)10	20	1604034	1604053
chr12_282	p2	(GT)13	26	1608823	1608848
chr12_283	p2	(AC)8	16	1615085	1615100
chr12_284	p2	(CA)13	26	1617094	1617119
chr12_285	c	(TC)7c(CT)11	37	1631569	1631605
chr12_286	c	(CT)8(GT)13	42	1633887	1633928
chr12_287	p2	(AT)7	14	1649798	1649811
chr12_288	p4	(GATG)5	20	1676079	1676098
chr12_289	p2	(CA)7	14	1699191	1699204
chr12_290	p2	(TG)7	14	1716005	1716018
chr12_291	p2	(GT)11	22	1719366	1719387
chr12_292	p2	(GT)12	24	1719946	1719969
chr12_293	p2	(AC)6	12	1723700	1723711
chr12_294	p2	(TG)7	14	1725584	1725597
chr12_295	p2	(TG)8	16	1727116	1727131
chr12_296	p4	(AAAT)7	28	1734766	1734793
chr12_297	p2	(CT)6	12	1738868	1738879
chr12_298	p2	(GT)9	18	1769091	1769108
chr12_299	p3	(AAT)5	15	1778085	1778099
chr12_300	p2	(TG)11	22	1780609	1780630
chr12_301	p2	(AG)8	16	1781633	1781648
chr12_302	p2	(TG)6	12	1788762	1788773
chr12_303	p2	(GT)6	12	1795604	1795615
chr12_304	p4	(ATCC)5	20	1798016	1798035
chr12_305	p2	(GT)6	12	1805829	1805840
chr12_306	p2	(GT)24	48	1810677	1810724
chr12_307	p2	(TC)10	20	1815329	1815348
chr12_308	p2	(TC)6	12	1816470	1816481
chr12_309	p3	(AGC)5	15	1816794	1816808
chr12_310	p2	(TA)6	12	1821772	1821783
chr12_311	p2	(CA)6	12	1822322	1822333
chr12_312	p2	(CA)6	12	1829623	1829634
chr12_313	p2	(GT)22	44	1831752	1831795
chr12_314	p2	(GT)6	12	1835625	1835636
chr12_315	c	(CA)15ctcg(CT)9	52	1843626	1843677
chr12_316	p3	(AAC)7	21	1845485	1845505
chr12_317	p2	(AC)14	28	1848195	1848222
chr12_318	c	(CT)14gtg(TC)10tgtgg(CT)10gtgg(CT)14gtgg(CT)10	132	1849033	1849164
chr12_319	p2	(TG)11	22	1858915	1858936
chr12_320	p2	(TG)13	26	1867721	1867746
chr12_321	p2	(GT)7	14	1875113	1875126
chr12_322	p2	(CA)17	34	1875559	1875592
chr12_323	p2	(CT)14	28	1877612	1877639
chr12_324	p2	(AG)8	16	1877948	1877963
chr12_325	c	(TG)11ttgctatggggtcatacattgttgaatgtgcataggttttttt(TG)[illegible]	81	1884193	1884273

chr12_326	p2	(AT)9	18	1886646	1886663
chr12_327	p2	(TA)6	12	1888217	1888228
chr12_328	p2	(GT)11	22	1890089	1890110
chr12_329	p2	(CA)7	14	1906813	1906826
chr12_330	p2	(TC)6	12	1928589	1928600
chr12_331	p2	(AG)6	12	1957380	1957391
chr12_332	p2	(AC)7	14	1964746	1964759
chr12_333	p2	(AC)7	14	1965807	1965820
chr12_334	p2	(AG)9	18	1966856	1966873
chr12_335	p2	(CA)7	14	1967079	1967092
chr12_336	p2	(TC)9	18	1968138	1968155
chr12_337	p2	(TG)10	20	1968614	1968633
chr12_338	p2	(GT)20	40	1968806	1968845
chr12_339	p4	(CATA)5	20	1977028	1977047
chr12_340	p2	(CA)7	14	1978690	1978703
chr12_341	p3	(ATA)6	18	1979439	1979456
chr12_342	p2	(CT)9	18	1983379	1983396
chr12_343	p2	(CA)10	20	1983577	1983596
chr12_344	p2	(AC)10	20	1985851	1985870
chr12_345	p2	(TG)6	12	1988610	1988621
chr12_346	p2	(GT)10	20	1991855	1991874
chr12_347	p2	(GT)9	18	1992492	1992509
chr12_348	p2	(AC)11	22	1995327	1995348
chr12_349	p2	(CA)6	12	2008602	2008613
chr12_350	p2	(CA)6	12	2009868	2009879
chr12_351	p2	(AC)9	18	2010029	2010046
chr12_352	c	acctcactccgttcaccagaagctacctggcatatggctggatgagggtct	121	2011115	2011235
chr12_353	p2	(CA)10	20	2012489	2012508
chr12_354	p2	(TG)9	18	2014256	2014273
chr12_355	p2	(AC)27	54	2014920	2014973
chr12_356	p2	(AC)7	14	2021640	2021653
chr12_357	c	(TA)6catataca(TG)11	42	2025280	2025321
chr12_358	p2	(GT)16	32	2045350	2045381
chr12_359	p2	(AG)10	20	2053216	2053235
chr12_360	p4	(TTCC)5	20	2055343	2055362
chr12_361	p2	(GT)9	18	2063863	2063880
chr12_362	p2	(TG)8	16	2066567	2066582
chr12_363	p2	(GT)9	18	2067692	2067709
chr12_364	p2	(GT)9	18	2068047	2068064
chr12_365	p4	(ACAA)7	28	2069304	2069331
chr12_366	p2	(AG)6	12	2073901	2073912
chr12_367	p3	(AAT)5	15	2074702	2074716
chr12_368	p2	(CA)9	18	2076516	2076533
chr12_369	p2	(TG)13	26	2076827	2076852
chr12_370	p2	(AG)8	16	2078608	2078623
chr12_371	p2	(GT)16	32	2079459	2079490
chr12_372	c	gtaatatggtgacagctttgggacagctaggcagctccagcagctaaacat	195	2079610	2079804
chr12_373	p2	(AC)6	12	2082759	2082770
chr12_374	p2	(CA)6	12	2082908	2082919
chr12_375	p2	(GT)6	12	2090324	2090335
chr12_376	p2	(TG)11	22	2091834	2091855
chr12_377	c	(TC)6(AC)12	36	2095566	2095601

chr12_378	p2	(CA)11	22	2097350	2097371
chr12_379	p2	(CA)6	12	2098068	2098079
chr12_380	p2	(TG)18	36	2098815	2098850
chr12_381	p2	(AC)18	36	2099128	2099163
chr12_382	p2	(AC)17	34	2103292	2103325
chr12_383	p2	(CA)6	12	2106163	2106174
chr12_384	p2	(AG)7	14	2106888	2106901
chr12_385	p2	(CA)7	14	2107483	2107496
chr12_386	p2	(GT)8	16	2113591	2113606
chr12_387	p2	(AC)7	14	2115435	2115448
chr12_388	p3	(CCT)5	15	2118031	2118045
chr12_389	p2	(CA)7	14	2119417	2119430
chr12_390	c	(CT)10g(TC)8	37	2120038	2120074
chr12_391	p2	(TG)6	12	2121369	2121380
chr12_392	p3	(TGG)5	15	2137679	2137693
chr12_393	p2	(TG)21	42	2138816	2138857
chr12_394	c	;tgagtttcatatggtgacatgtcatgtgcacagcatgtcagtctgtcaggtat	124	2143705	2143828
chr12_395	p2	(TG)10	20	2153402	2153421
chr12_396	p2	(GT)23	46	2159850	2159895
chr12_397	p2	(CT)7	14	2160921	2160934
chr12_398	p2	(TG)8	16	2161970	2161985
chr12_399	p3	(AAT)6	18	2164063	2164080
chr12_400	p3	(ATC)13	39	2166813	2166851
chr12_401	p3	(TTG)6	18	2169320	2169337
chr12_402	p2	(TG)7	14	2171904	2171917
chr12_403	p2	(CA)13	26	2176145	2176170
chr12_404	c	:T)8gtctgacacatatcctctctctccttctccctctgtctctctctcctt(CA	76	2177899	2177974
chr12_405	p3	(TCC)5	15	2182587	2182601
chr12_406	p2	(TG)6	12	2184950	2184961
chr12_407	p2	(AG)8	16	2185081	2185096
chr12_408	p3	(TAG)7	21	2185356	2185376
chr12_409	p2	(CA)18	36	2188157	2188192
chr12_410	p2	(CA)18	36	2189447	2189482
chr12_411	c	gaggaatggagtgctgcagttaggacatggtgtgttcgtgcgtatatgtgtg	127	2191059	2191185
chr12_412	c	:attgaaacacacctctgtggaacagctgcaatgatgagacagagagtagg	125	2193111	2193235
chr12_413	p2	(GA)7	14	2193337	2193350
chr12_414	p2	(AC)11	22	2195019	2195040
chr12_415	p2	(TG)6	12	2196179	2196190
chr12_416	p2	(CA)8	16	2197870	2197885
chr12_417	c	;tgctctttaacctgaatgtgagtcagaaaccagtttgattcatatcctgcctg	126	2198948	2199073
chr12_418	p2	(CT)6	12	2199383	2199394
chr12_419	p2	(AC)6	12	2202651	2202662
chr12_420	c	tctgcctctctgatgtcaccttgtatcatttcagactaatttcacctattctcaga	95	2204781	2204875
chr12_421	p2	(TG)14	28	2207590	2207617
chr12_422	c	tggcagaacagtgaggaaagggagaagtgttaactgaaaacgtgggtga	109	2209018	2209126
chr12_423	p4	(GTAA)5	20	2219217	2219236
chr12_424	p2	(AC)6	12	2223299	2223310
chr12_425	p2	(AC)6	12	2225579	2225590
chr12_426	c	(TG)6(TA)6(TG)9	42	2226959	2227000
chr12_427	p2	(CA)16	32	2229158	2229189
chr12_428	p2	(TG)10	20	2230803	2230822
chr12_429	p2	(AT)6	12	2233276	2233287

chr12_430	c	gatgaagtagcttttaatccaaatcctgaattttctgctgaaaacctcttggtca	106	2234912	2235017
chr12_431	p2	(TG)6	12	2235409	2235420
chr12_432	p2	(CA)7	14	2236642	2236655
chr12_433	p2	(TG)19	38	2237836	2237873
chr12_434	c	ggctatatgttgtgaggatcagtttgagttttatactatgggagtgaggatgtt	112	2238377	2238488
chr12_435	p2	(TG)6	12	2241533	2241544
chr12_436	p2	(AT)6	12	2245035	2245046
chr12_437	p2	(TG)6	12	2247284	2247295
chr12_438	p2	(CT)7	14	2252660	2252673
chr12_439	p4	(CTTT)7	28	2264489	2264516
chr12_440	p2	(AT)10	20	2270816	2270835
chr12_441	p3	(GAG)5	15	2273280	2273294
chr12_442	p3	(AAG)5	15	2276900	2276914
chr12_443	p5	(TTTTG)5	25	2282281	2282305
chr12_444	p2	(TG)8	16	2285234	2285249
chr12_445	p2	(AG)6	12	2288505	2288516
chr12_446	c	(TC)7(AC)10	34	2293263	2293296
chr12_447	p2	(TG)7	14	2303295	2303308
chr12_448	p2	(GT)13	26	2307129	2307154
chr12_449	p3	(TTG)5	15	2307571	2307585
chr12_450	p4	(TTTA)6	24	2334841	2334864
chr12_451	p2	(GA)12	24	2337808	2337831
chr12_452	p2	(AC)6	12	2341664	2341675
chr12_453	p2	(CA)8	16	2351783	2351798
chr12_454	p2	(CA)17	34	2352532	2352565
chr12_455	p3	(GCA)9	27	2354073	2354099
chr12_456	p2	(TG)10	20	2355850	2355869
chr12_457	p2	(CA)10	20	2357709	2357728
chr12_458	p2	(CA)6	12	2357978	2357989
chr12_459	p2	(AC)7	14	2360497	2360510
chr12_460	p2	(CA)12	24	2360778	2360801
chr12_461	p2	(AC)6	12	2365521	2365532
chr12_462	p2	(TG)6	12	2368488	2368499
chr12_463	p4	(CCCT)5	20	2370789	2370808
chr12_464	p2	(AC)9	18	2372943	2372960
chr12_465	c	aggctgtgagacagaaatcttgggctgtgtgtgacagagcaggggcgtaac	113	2373700	2373812
chr12_466	p2	(GT)6	12	2375153	2375164
chr12_467	p2	(GT)7	14	2378128	2378141
chr12_468	p2	(AT)7	14	2414472	2414485
chr12_469	p2	(TG)6	12	2414707	2414718
chr12_470	p2	(TG)6	12	2416481	2416492
chr12_471	p2	(TA)6	12	2433760	2433771
chr12_472	p4	(AAAT)6	24	2447095	2447118
chr12_473	p3	(ATT)8	24	2460077	2460100
chr12_474	p2	(AC)6	12	2471387	2471398
chr12_475	p2	(AC)12	24	2479434	2479457
chr12_476	p2	(AT)9	18	2494013	2494030
chr12_477	p2	(TC)6	12	2516505	2516516
chr12_478	p2	(AC)13	26	2520711	2520736
chr12_479	p2	(TG)8	16	2527079	2527094
chr12_480	p3	(GCT)7	21	2537611	2537631
chr12_481	p2	(CA)6	12	2539137	2539148

chr12_482	p2	(TG)8	16	2541278	2541293
chr12_483	p3	(CTT)5	15	2544763	2544777
chr12_484	p5	(GATTT)5	25	2554404	2554428
chr12_485	p2	(TG)6	12	2570416	2570427
chr12_486	p2	(CA)15	30	2572674	2572703
chr12_487	p2	(CA)7	14	2596065	2596078
chr12_488	p3	(ATC)5	15	2638600	2638614
chr12_489	p2	(TG)15	30	2674583	2674612
chr12_490	p2	(GT)14	28	2676840	2676867
chr12_491	p2	(GT)12	24	2678722	2678745
chr12_492	p2	(GT)16	32	2681034	2681065
chr12_493	p3	(TTG)5	15	2690777	2690791
chr12_494	p2	(AC)8	16	2696754	2696769
chr12_495	p2	(CA)6	12	2703919	2703930
chr12_496	p2	(AG)7	14	2716299	2716312
chr12_497	p2	(AC)7	14	2717961	2717974
chr12_498	p2	(AC)6	12	2728656	2728667
chr12_499	p2	(TG)7	14	2736557	2736570
chr12_500	p2	(GT)10	20	2739536	2739555
chr12_501	p2	(CT)6	12	2739957	2739968
chr12_502	p2	(CT)13	26	2740437	2740462
chr12_503	c	(TC)6c(CT)9(CA)27	85	2740779	2740863
chr12_504	p2	(TG)17	34	2745377	2745410
chr12_505	p2	(AC)12	24	2752225	2752248
chr12_506	p2	(AC)6	12	2757485	2757496
chr12_507	p2	(TG)17	34	2783201	2783234
chr12_508	p2	(TC)7	14	2784126	2784139
chr12_509	p4	(GAGT)6	24	2785060	2785083
chr12_510	p2	(GT)10	20	2785799	2785818
chr12_511	p2	(AG)7	14	2786050	2786063
chr12_512	p3	(CCA)5	15	2786410	2786424
chr12_513	p2	(GT)7	14	2787731	2787744
chr12_514	c	;ggattttgttcagggattgtcgctacagatgtcagtgtgtgagtgcatgtgta	155	2796038	2796192
chr12_515	p2	(TG)21	42	2796326	2796367
chr12_516	p2	(AG)8	16	2798284	2798299
chr12_517	p2	(TG)11	22	2823023	2823044
chr12_518	p2	(TG)16	32	2823246	2823277
chr12_519	p2	(TG)6	12	2827952	2827963
chr12_520	p2	(CA)9	18	2829103	2829120
chr12_521	p2	(TG)7	14	2831981	2831994
chr12_522	p2	(TG)16	32	2834977	2835008
chr12_523	p2	(CA)12	24	2835913	2835936
chr12_524	p3	(CCA)5	15	2847163	2847177
chr12_525	p2	(AC)6	12	2847749	2847760
chr12_526	p2	(GT)11	22	2853384	2853405
chr12_527	p2	(TG)12	24	2854081	2854104
chr12_528	c	(GT)6t(TG)10	33	2860572	2860604
chr12_529	p2	(TG)6	12	2860860	2860871
chr12_530	p2	(TG)17	34	2863025	2863058
chr12_531	p3	(CCT)6	18	2863231	2863248
chr12_532	c	cgcatactgatatgcagtatgtgtatgcagacaaaatcta(AC)7atactg	270	2874355	2874624
chr12_533	p2	(TG)21	42	2890260	2890301

chr12_534	p2	(CT)12	24	2893172	2893195
chr12_535	p2	(CA)7	14	2893839	2893852
chr12_536	p2	(CA)8	16	2901769	2901784
chr12_537	p2	(GT)8	16	2902081	2902096
chr12_538	p2	(AC)9	18	2905868	2905885
chr12_539	p2	(TC)7	14	2908319	2908332
chr12_540	p2	(TG)7	14	2912754	2912767
chr12_541	p2	(CA)8	16	2912980	2912995
chr12_542	p2	(TG)11	22	2923254	2923275
chr12_543	p2	(CA)8	16	2926596	2926611
chr12_544	p2	(TG)11	22	2929777	2929798
chr12_545	p2	(CA)8	16	2933119	2933134
chr12_546	p3	(GTT)5	15	2943989	2944003
chr12_547	p2	(AC)7	14	2950661	2950674
chr12_548	c	gcatatgtgtatgcaaccatactgtcaacagtaggaaatgggaaacagtaat	121	2962517	2962637
chr12_549	p3	(TCC)5	15	2970021	2970035
chr12_550	p3	(TGA)5	15	2970687	2970701
chr12_551	p5	(GGTTT)6	30	2970818	2970847
chr12_552	p2	(TG)7	14	2971917	2971930
chr12_553	p2	(AC)7	14	2972886	2972899
chr12_554	p2	(CA)11	22	2975566	2975587
chr12_555	c	)6cttgtaaaactctgcatcctgggaaataagcagagtcgagtggttcatg((	73	2981573	2981645
chr12_556	p2	(TG)6	12	2984576	2984587
chr12_557	p2	(TG)8	16	2984796	2984811
chr12_558	p2	(TG)7	14	2987289	2987302
chr12_559	p2	(CA)8	16	2990322	2990337
chr12_560	p4	(CCAT)5	20	2993411	2993430
chr12_561	p2	(TG)12	24	2999982	3000005
chr12_562	p2	(TG)6	12	3002344	3002355
chr12_563	p4	(TTAT)5	20	3003386	3003405
chr12_564	p2	(TC)6	12	3004462	3004473
chr12_565	p2	(TG)8	16	3007169	3007184
chr12_566	p2	(TC)6	12	3009120	3009131
chr12_567	p2	(TC)6	12	3012858	3012869
chr12_568	p2	(CT)7	14	3013031	3013044
chr12_569	p2	(TG)7	14	3014795	3014808
chr12_570	p2	(AT)6	12	3019930	3019941
chr12_571	p2	(TG)11	22	3022844	3022865
chr12_572	p4	(TACA)5	20	3029236	3029255
chr12_573	p2	(CA)6	12	3032345	3032356
chr12_574	p3	(TGC)6	18	3044524	3044541
chr12_575	p2	(TG)6	12	3051388	3051399
chr12_576	p3	(CTC)6	18	3077548	3077565
chr12_577	p2	(AT)6	12	3081724	3081735
chr12_578	p2	(TA)7	14	3083877	3083890
chr12_579	p2	(AC)6	12	3084591	3084602
chr12_580	p3	(GTT)5	15	3088639	3088653
chr12_581	p4	(CAAA)6	24	3129228	3129251
chr12_582	p3	(TAA)5	15	3143795	3143809
chr12_583	p2	(AC)53	106	3150780	3150885
chr12_584	p3	(GCA)8	24	3169772	3169795
chr12_585	p3	(CAG)6	18	3169995	3170012

chr12_586	p2	(AC)9	18	3177060	3177077
chr12_587	p4	(ATGG)5	20	3182859	3182878
chr12_588	p2	(TG)19	38	3202166	3202203
chr12_589	p2	(TG)6	12	3204199	3204210
chr12_590	p3	(AAC)5	15	3230825	3230839
chr12_591	p2	(GT)7	14	3235652	3235665
chr12_592	p2	(AC)52	104	3239611	3239714
chr12_593	p2	(AT)6	12	3247304	3247315
chr12_594	p4	(AAAT)5	20	3250257	3250276
chr12_595	p4	(TCTA)8	32	3257574	3257605
chr12_596	p5	(ATAGT)6	30	3258053	3258082
chr12_597	p2	(AC)21	42	3289819	3289860
chr12_598	p3	(CAG)5	15	3292989	3293003
chr12_599	p2	(TC)11	22	3297148	3297169
chr12_600	p2	(AC)9	18	3298076	3298093
chr12_601	p2	(AC)26	52	3329476	3329527
chr12_602	p2	(TG)12	24	3333432	3333455
chr12_603	p3	(CAT)10	30	3335649	3335678
chr12_604	p2	(TG)7	14	3342938	3342951
chr12_605	p2	(AG)6	12	3352023	3352034
chr12_606	p3	(ATA)6	18	3357290	3357307
chr12_607	p2	(TG)7	14	3358048	3358061
chr12_608	p3	(GAG)5	15	3365262	3365276
chr12_609	p2	(CA)16	32	3365892	3365923
chr12_610	p2	(CT)7	14	3373285	3373298
chr12_611	p2	(TG)7	14	3376504	3376517
chr12_612	p2	(GA)6	12	3376950	3376961
chr12_613	p2	(TG)9	18	3390821	3390838
chr12_614	p2	(AG)6	12	3406446	3406457
chr12_615	p2	(CT)10	20	3413913	3413932
chr12_616	p2	(GA)6	12	3437605	3437616
chr12_617	p2	(TG)18	36	3440866	3440901
chr12_618	p2	(TG)6	12	3451238	3451249
chr12_619	p2	(GT)6	12	3451475	3451486
chr12_620	c	(GT)11ga(GT)14	52	3459424	3459475
chr12_621	p2	(AC)7	14	3462983	3462996
chr12_622	p2	(CA)6	12	3468316	3468327
chr12_623	p3	(TTA)5	15	3471398	3471412
chr12_624	p2	(GT)7	14	3475386	3475399
chr12_625	p2	(AC)12	24	3478474	3478497
chr12_626	p2	(AC)7	14	3478853	3478866
chr12_627	p2	(TG)10	20	3483158	3483177
chr12_628	p2	(TG)6	12	3490920	3490931
chr12_629	p3	(CCT)8	24	3492365	3492388
chr12_630	p5	(AGAGG)6	30	3507614	3507643
chr12_631	p2	(AC)13	26	3510391	3510416
chr12_632	c	[illegible]GT)8tgggataactatatcaacaactataaataaatatttttatgtgc(AT[illegible]	82	3510671	3510752
chr12_633	p3	(TTA)5	15	3521471	3521485
chr12_634	p3	(GAG)6	18	3522971	3522988
chr12_635	p2	(TG)7	14	3528552	3528565
chr12_636	c	(CTCA)6ctaactcact(CA)9	52	3530869	3530920
chr12_637	p2	(GT)11	22	3532197	3532218

chr12_638	p2	(TG)8	16	3550054	3550069
chr12_639	p2	(CA)18	36	3550242	3550277
chr12_640	p2	(CA)11	22	3553113	3553134
chr12_641	p2	(TC)8	16	3554021	3554036
chr12_642	p2	(TA)6	12	3560185	3560196
chr12_643	c	(GT)8ttgtag(GT)14	50	3570731	3570780
chr12_644	p2	(AC)7	14	3579059	3579072
chr12_645	p4	(AAAC)5	20	3613450	3613469
chr12_646	p2	(TC)8	16	3614356	3614371
chr12_647	p2	(TA)6	12	3614881	3614892
chr12_648	p2	(TG)14	28	3615309	3615336
chr12_649	c	[illegible]6aaatggctttatacgtgcagattgcgcagcacgcatttgtgatcctcatg([illegible]	74	3616255	3616328
chr12_650	p2	(GT)7	14	3621526	3621539
chr12_651	p2	(AG)12	24	3623873	3623896
chr12_652	p2	(CA)7	14	3626434	3626447
chr12_653	p2	(CA)11	22	3626783	3626804
chr12_654	p2	(CA)8	16	3627040	3627055
chr12_655	p2	(GT)6	12	3629510	3629521
chr12_656	p2	(TC)7	14	3630899	3630912
chr12_657	p4	(AGAC)7	28	3636816	3636843
chr12_658	p2	(AC)6	12	3641423	3641434
chr12_659	p2	(CA)19	38	3642019	3642056
chr12_660	c	(TG)7aggc(AG)7	32	3657986	3658017
chr12_661	p2	(GT)6	12	3658264	3658275
chr12_662	p2	(AC)10	20	3661466	3661485
chr12_663	p2	(CA)10	20	3666626	3666645
chr12_664	p2	(GT)11	22	3672988	3673009
chr12_665	p2	(AC)10	20	3683481	3683500
chr12_666	p5	(AAATC)5	25	3690161	3690185
chr12_667	p2	(TG)6	12	3695066	3695077
chr12_668	p2	(AC)10	20	3699483	3699502
chr12_669	p2	(TG)13	26	3700319	3700344
chr12_670	p2	(CA)7	14	3703050	3703063
chr12_671	p2	(CA)6	12	3705134	3705145
chr12_672	p2	(AC)10	20	3707451	3707470
chr12_673	p2	(GA)16	32	3710512	3710543
chr12_674	p2	(AC)6	12	3711363	3711374
chr12_675	p2	(AC)6	12	3714983	3714994
chr12_676	p2	(GT)24	48	3715600	3715647
chr12_677	p2	(AC)22	44	3720466	3720509
chr12_678	p2	(CA)17	34	3720779	3720812
chr12_679	p2	(AC)15	30	3722087	3722116
chr12_680	p2	(GT)6	12	3722335	3722346
chr12_681	p2	(AC)6	12	3723207	3723218
chr12_682	p2	(CA)15	30	3728791	3728820
chr12_683	p2	(CA)7	14	3736294	3736307
chr12_684	p2	(GT)7	14	3746575	3746588
chr12_685	p2	(CA)11	22	3746700	3746721
chr12_686	p2	(TG)8	16	3747397	3747412
chr12_687	p2	(GT)10	20	3749460	3749479
chr12_688	p2	(CA)13	26	3750115	3750140
chr12_689	p2	(CA)7	14	3752170	3752183

chr12_690	p2	(GT)10	20	3752932	3752951
chr12_691	p2	(AG)6	12	3754167	3754178
chr12_692	c	ccctgtgctaactctctatctgtcttgctccatccttcctttcactgtcccacac	97	3754357	3754453
chr12_693	p2	(TG)36	72	3757614	3757685
chr12_694	p2	(TC)7	14	3758058	3758071
chr12_695	p2	(AC)11	22	3758219	3758240
chr12_696	p2	(AC)6	12	3758454	3758465
chr12_697	p2	(GT)6	12	3759515	3759526
chr12_698	p2	(TG)7	14	3767179	3767192
chr12_699	p2	(AC)6	12	3767451	3767462
chr12_700	p2	(CA)11	22	3774475	3774496
chr12_701	p2	(AC)7	14	3784667	3784680
chr12_702	p2	(TA)6	12	3785212	3785223
chr12_703	p3	(GAG)5	15	3786134	3786148
chr12_704	p2	(CA)11	22	3786564	3786585
chr12_705	c	agcttgttcatatgactggtccagaatcggattacctcgttacattccacacac	153	3796681	3796833
chr12_706	p2	(CA)7	14	3801048	3801061
chr12_707	p2	(TG)6	12	3801241	3801252
chr12_708	p2	(AC)7	14	3802181	3802194
chr12_709	p2	(AC)9	18	3802477	3802494
chr12_710	p4	(ATTT)5	20	3804771	3804790
chr12_711	p3	(ATT)5	15	3806684	3806698
chr12_712	p2	(AC)8	16	3808258	3808273
chr12_713	p2	(CT)6	12	3808380	3808391
chr12_714	p2	(AC)13	26	3808693	3808718
chr12_715	p2	(AG)6	12	3823368	3823379
chr12_716	p2	(TG)12	24	3823780	3823803
chr12_717	p2	(AC)13	26	3824818	3824843
chr12_718	p2	(TG)16	32	3827548	3827579
chr12_719	p2	(TG)21	42	3829791	3829832
chr12_720	p2	(CA)14	28	3830969	3830996
chr12_721	p2	(CA)8	16	3833281	3833296
chr12_722	p2	(TG)14	28	3833814	3833841
chr12_723	p2	(GA)12	24	3836906	3836929
chr12_724	p2	(AG)6	12	3837781	3837792
chr12_725	p2	(CA)6	12	3838287	3838298
chr12_726	p2	(AC)6	12	3844014	3844025
chr12_727	p2	(AC)7	14	3844637	3844650
chr12_728	p2	(AC)17	34	3844894	3844927
chr12_729	p2	(TC)8	16	3852267	3852282
chr12_730	p2	(AC)9	18	3855054	3855071
chr12_731	p2	(CA)8	16	3860808	3860823
chr12_732	p2	(AT)6	12	3861113	3861124
chr12_733	c	(CA)8t(AC)13	43	3866565	3866607
chr12_734	p2	(CA)13	26	3878580	3878605
chr12_735	p3	(TCA)5	15	3880876	3880890
chr12_736	p2	(AT)7	14	3894125	3894138
chr12_737	c	ggatactaacattaaggtccattaccgccacctactatgcttgagtgttgacc	140	3895774	3895913
chr12_738	c	(TAA)5(TTA)6	33	3896182	3896214
chr12_739	p2	(AT)7	14	3898129	3898142
chr12_740	p3	(ATT)5	15	3904085	3904099
chr12_741	p2	(TA)8	16	3909412	3909427

chr12_742	p2	(GT)15	30	3912814	3912843
chr12_743	c	(TG)6ta(TG)8	30	3914396	3914425
chr12_744	p2	(TG)13	26	3922484	3922509
chr12_745	p2	(TG)13	26	3922681	3922706
chr12_746	p2	(CA)6	12	3924280	3924291
chr12_747	c	(AT)7(GT)9	32	3925613	3925644
chr12_748	p4	(TAAA)6	24	3930851	3930874
chr12_749	p2	(TG)9	18	3934684	3934701
chr12_750	p2	(AC)7	14	3939908	3939921
chr12_751	c	(TG)10ttc(AT)6c(TA)8gatatatatctcatataca(GT)17	105	3940261	3940365
chr12_752	p2	(GT)9	18	3940657	3940674
chr12_753	p2	(CA)11	22	3945214	3945235
chr12_754	p2	(TG)17	34	3947820	3947853
chr12_755	p2	(GT)11	22	3949472	3949493
chr12_756	p2	(GT)9	18	3950752	3950769
chr12_757	p5	(TCCTT)5	25	3950904	3950928
chr12_758	p2	(AC)8	16	3953335	3953350
chr12_759	p2	(TG)23	46	3954498	3954543
chr12_760	p3	(ATA)8	24	3962933	3962956
chr12_761	p2	(GA)6	12	3967246	3967257
chr12_762	c	0aaagaaataattggaaaacatagtatggatgctgaatcagcatccatgca	99	3973128	3973226
chr12_763	p2	(TG)6	12	4001328	4001339
chr12_764	p4	(CAGC)5	20	4003020	4003039
chr12_765	p3	(GTG)6	18	4015662	4015679
chr12_766	p2	(AC)17	34	4017583	4017616
chr12_767	p3	(TGG)6	18	4017932	4017949
chr12_768	p2	(GT)9	18	4040300	4040317
chr12_769	p2	(CA)7	14	4047190	4047203
chr12_770	p2	(GA)9	18	4048104	4048121
chr12_771	c	(GT)6atatatacagtagtttatgtat(AC)19	72	4048754	4048825
chr12_772	p2	(TG)6	12	4060638	4060649
chr12_773	p4	(AATC)5	20	4060790	4060809
chr12_774	p2	(GA)6	12	4061176	4061187
chr12_775	p2	(TG)21	42	4062879	4062920
chr12_776	p2	(GT)6	12	4063897	4063908
chr12_777	p2	(TG)7	14	4067743	4067756
chr12_778	p2	(GA)9	18	4069967	4069984
chr12_779	p2	(TG)7	14	4070139	4070152
chr12_780	p2	(GT)6	12	4073822	4073833
chr12_781	p3	(AGA)5	15	4082421	4082435
chr12_782	p2	(TG)7	14	4087803	4087816
chr12_783	p3	(AAG)6	18	4089211	4089228
chr12_784	p2	(CA)9	18	4114513	4114530
chr12_785	p3	(TAA)10	30	4120999	4121028
chr12_786	p3	(AAT)7	21	4122407	4122427
chr12_787	p2	(TG)13	26	4130138	4130163
chr12_788	p2	(CA)10	20	4132423	4132442
chr12_789	p2	(TG)17	34	4137011	4137044
chr12_790	p2	(AC)8	16	4142867	4142882
chr12_791	p2	(CT)6	12	4150216	4150227
chr12_792	c	(AC)8at(AC)13	44	4150421	4150464
chr12_793	p2	(AC)6	12	4161659	4161670

chr12_794	c	cnnnnnnnnnnnnnnnnnnnnnnnnnnnagatacaccccccgctcact	159	4169028	4169186
chr12_795	p2	(CA)15	30	4173142	4173171
chr12_796	c	(TG)18cgcgcgcgcgcacatgtgtgtacgtaaa(TG)6	76	4180452	4180527
chr12_797	p2	(AC)7	14	4181919	4181932
chr12_798	p2	(TC)13	26	4186270	4186295
chr12_799	p2	(GT)10	20	4189380	4189399
chr12_800	p2	(TG)15	30	4191824	4191853
chr12_801	p2	(AC)13	26	4196803	4196828
chr12_802	p2	(CA)18	36	4200706	4200741
chr12_803	p2	(GT)6	12	4201613	4201624
chr12_804	p2	(TC)6	12	4203184	4203195
chr12_805	p2	(TG)13	26	4208209	4208234
chr12_806	p2	(GT)23	46	4216367	4216412
chr12_807	p2	(CA)20	40	4226924	4226963
chr12_808	p2	(TG)6	12	4227344	4227355
chr12_809	c	ctgatcctcacatccacatagctcctctgcactggcctaaatggagggtagg	143	4228191	4228333
chr12_810	p2	(GT)8	16	4228909	4228924
chr12_811	p2	(GT)6	12	4232256	4232267
chr12_812	p2	(TC)12	24	4232540	4232563
chr12_813	p4	(AACA)5	20	4236125	4236144
chr12_814	p2	(CA)11	22	4241302	4241323
chr12_815	p2	(TG)10	20	4243714	4243733
chr12_816	p2	(GT)9	18	4245399	4245416
chr12_817	p2	(TG)8	16	4248697	4248712
chr12_818	p2	(AC)16	32	4250549	4250580
chr12_819	p3	(TAT)7	21	4251296	4251316
chr12_820	p4	(GTAT)5	20	4251703	4251722
chr12_821	p3	(CAT)5	15	4252951	4252965
chr12_822	p2	(AC)11	22	4260080	4260101
chr12_823	p2	(GT)12	24	4266869	4266892
chr12_824	p2	(CA)12	24	4272656	4272679
chr12_825	p2	(GA)10	20	4276740	4276759
chr12_826	p2	(CA)6	12	4279500	4279511
chr12_827	c	(AC)7atacacacat(AC)10	44	4280325	4280368
chr12_828	p2	(TG)24	48	4280659	4280706
chr12_829	p2	(TG)6	12	4284430	4284441
chr12_830	p2	(TG)10	20	4286320	4286339
chr12_831	p4	(CATT)5	20	4291805	4291824
chr12_832	p2	(CA)9	18	4292882	4292899
chr12_833	c	(TG)10tc(TG)9tc(TG)9tc(TG)9tc(TG)8	98	4293374	4293471
chr12_834	p2	(CT)14	28	4296522	4296549
chr12_835	p2	(GT)9	18	4311140	4311157
chr12_836	p3	(GGT)5	15	4315293	4315307
chr12_837	p2	(TC)8	16	4371550	4371565
chr12_838	p2	(CA)6	12	4373491	4373502
chr12_839	p4	(TATT)5	20	4374389	4374408
chr12_840	p2	(CT)8	16	4394367	4394382
chr12_841	p3	(GAT)10	30	4420767	4420796
chr12_842	p3	(TAT)8	24	4423080	4423103
chr12_843	p2	(GT)6	12	4424936	4424947
chr12_844	p2	(AG)9	18	4430863	4430880
chr12_845	p2	(AG)7	14	4431143	4431156

chr12_846	p2	(TG)9	18	4432677	4432694
chr12_847	p4	(TCCA)5	20	4435065	4435084
chr12_848	p2	(GT)12	24	4447378	4447401
chr12_849	p2	(CA)6	12	4449491	4449502
chr12_850	p2	(GT)25	50	4455779	4455828
chr12_851	p2	(GT)8	16	4460017	4460032
chr12_852	p2	(GT)6	12	4470341	4470352
chr12_853	p3	(ACT)5	15	4471504	4471518
chr12_854	p2	(TC)6	12	4474774	4474785
chr12_855	p2	(TG)8	16	4475274	4475289
chr12_856	c	(GT)11(GA)8	38	4480593	4480630
chr12_857	p2	(GT)11	22	4499395	4499416
chr12_858	p2	(GT)15	30	4530093	4530122
chr12_859	p2	(TG)8	16	4530305	4530320
chr12_860	c	(AC)6ctacacacct(AC)9	40	4547807	4547846
chr12_861	c	(AC)6aatgtgtacaatgctcttatgttttcacagcagca(AC)6	59	4550639	4550697
chr12_862	p2	(TG)22	44	4556968	4557011
chr12_863	p2	(AT)10	20	4557880	4557899
chr12_864	p3	(CTG)5	15	4559198	4559212
chr12_865	p2	(TG)6	12	4563213	4563224
chr12_866	p2	(TG)6	12	4577976	4577987
chr12_867	p2	(TG)9	18	4595426	4595443
chr12_868	p2	(TC)6	12	4597453	4597464
chr12_869	p3	(GTA)5	15	4598791	4598805
chr12_870	p2	(AC)6	12	4608800	4608811
chr12_871	p2	(AC)9	18	4617044	4617061
chr12_872	p2	(CA)7	14	4621041	4621054
chr12_873	p2	(TC)10	20	4630946	4630965
chr12_874	p2	(CA)22	44	4631244	4631287
chr12_875	p2	(TG)11	22	4632543	4632564
chr12_876	c	(AG)6aa(AG)17	48	4633512	4633559
chr12_877	p2	(CT)7	14	4648046	4648059
chr12_878	p2	(GT)7	14	4659987	4660000
chr12_879	p2	(AC)6	12	4664266	4664277
chr12_880	p2	(TG)6	12	4665009	4665020
chr12_881	p2	(GT)6	12	4667769	4667780
chr12_882	c	(GT)12gc(GT)6	38	4669403	4669440
chr12_883	p2	(GT)7	14	4671974	4671987
chr12_884	p2	(GT)6	12	4676689	4676700
chr12_885	p2	(GT)6	12	4682493	4682504
chr12_886	p2	(CA)6	12	4685097	4685108
chr12_887	c	(CT)17(CA)6	46	4688787	4688832
chr12_888	c	(CT)25(CA)6	62	4688974	4689035
chr12_889	p2	(GT)6	12	4696865	4696876
chr12_890	p2	(AC)23	46	4702458	4702503
chr12_891	p2	(AC)6	12	4710282	4710293
chr12_892	p2	(TG)7	14	4716936	4716949
chr12_893	p2	(GT)6	12	4718244	4718255
chr12_894	p2	(TG)11	22	4721366	4721387
chr12_895	c	(TC)6cttctcttg(CT)6	33	4721586	4721618
chr12_896	p2	(TG)8	16	4722135	4722150
chr12_897	p2	(GT)14	28	4724958	4724985

chr12_898	p2	(TG)14	28	4726049	4726076
chr12_899	p2	(GT)6	12	4729030	4729041
chr12_900	p2	(TG)8	16	4729536	4729551
chr12_901	p2	(CT)10	20	4735123	4735142
chr12_902	p2	(TG)12	24	4740982	4741005
chr12_903	p2	(CA)27	54	4766485	4766538
chr12_904	p2	(CA)7	14	4771328	4771341
chr12_905	p2	(AC)9	18	4783292	4783309
chr12_906	c	(TG)9cagtta(TG)8	40	4783894	4783933
chr12_907	p2	(AC)8	16	4787490	4787505
chr12_908	p2	(AC)6	12	4790176	4790187
chr12_909	p2	(GT)10	20	4794013	4794032
chr12_910	p2	(CA)10	20	4794375	4794394
chr12_911	p2	(TG)8	16	4797863	4797878
chr12_912	p2	(CA)8	16	4802902	4802917
chr12_913	p2	(CA)9	18	4807138	4807155
chr12_914	p2	(AC)6	12	4815474	4815485
chr12_915	p2	(TG)9	18	4819547	4819564
chr12_916	p2	(GA)6	12	4832318	4832329
chr12_917	p2	(CT)17	34	4833931	4833964
chr12_918	p2	(GT)9	18	4834095	4834112
chr12_919	c	[illegible]tttttccaatgtgggtgtgtgtaacttttactgtagtgggtggctgcatgctag[illegible]	95	4834283	4834377
chr12_920	p2	(TG)7	14	4835292	4835305
chr12_921	p2	(TG)12	24	4837571	4837594
chr12_922	p2	(AC)30	60	4842784	4842843
chr12_923	p2	(GT)11	22	4843858	4843879
chr12_924	p3	(GAA)7	21	4865032	4865052
chr12_925	c	(CTTTTT)5tccttttccttttccttttcctcctccgcc(TCT)12	96	4866462	4866557
chr12_926	p2	(CA)15	30	4868982	4869011
chr12_927	c	(GA)6gggagagagagaaagagg(GA)9	48	4871202	4871249
chr12_928	p2	(GA)6	12	4879585	4879596
chr12_929	p2	(AT)6	12	4881470	4881481
chr12_930	p2	(AC)29	58	4887391	4887448
chr12_931	p2	(AC)6	12	4893644	4893655
chr12_932	c	(TG)16(AG)9	50	4915884	4915933
chr12_933	c	(TC)7tatacacacacacc(CA)11	50	4926204	4926253
chr12_934	p2	(CA)17	34	4933833	4933866
chr12_935	p2	(AC)6	12	4935714	4935725
chr12_936	p2	(AC)6	12	4937292	4937303
chr12_937	c	(TG)6g(GT)6	25	4940961	4940985
chr12_938	p2	(TG)10	20	4948708	4948727
chr12_939	p2	(AC)34	68	4953318	4953385
chr12_940	p2	(AC)9	18	4959064	4959081
chr12_941	p2	(AC)17	34	4959656	4959689
chr12_942	c	(AC)9atg(CA)7	35	4961162	4961196
chr12_943	p2	(TG)7	14	4961569	4961582
chr12_944	p2	(CA)16	32	4961721	4961752
chr12_945	p2	(CA)8	16	4972050	4972065
chr12_946	p2	(GT)31	62	4977409	4977470
chr12_947	c	agaggttctcagggtaccaggccatgaggaattgcttgtcactgtgtttgtg[illegible]	131	4978907	4979037
chr12_948	p2	(CT)9	18	4979228	4979245
chr12_949	p2	(TG)21	42	4992904	4992945

chr12_950	p3	(TTA)5	15	4997532	4997546
chr12_951	p2	(AT)8	16	4999172	4999187
chr12_952	p2	(CA)6	12	5004815	5004826
chr12_953	c	gtgtgtttgtgtgttaggacagagcattaaggcagagggggaa(GT)11g	97	5007497	5007593
chr12_954	p2	(TC)9	18	5011112	5011129
chr12_955	p4	(ATCC)5	20	5012286	5012305
chr12_956	p2	(TG)18	36	5013012	5013047
chr12_957	p2	(GT)25	50	5013772	5013821
chr12_958	p2	(AC)16	32	5013981	5014012
chr12_959	c	(TC)8a(CT)6	29	5016582	5016610
chr12_960	p2	(GT)9	18	5020393	5020410
chr12_961	p2	(GT)6	12	5023860	5023871
chr12_962	c	agggaaggaagacttaaaaggaatggaatatttcagtgttgaaggacctctc	127	5034306	5034432
chr12_963	p2	(CA)6	12	5036383	5036394
chr12_964	p2	(TG)7	14	5040379	5040392
chr12_965	p3	(ACT)9	27	5051564	5051590
chr12_966	p2	(GT)21	42	5073914	5073955
chr12_967	p2	(TG)11	22	5074091	5074112
chr12_968	p2	(CT)6	12	5078117	5078128
chr12_969	p2	(AT)8	16	5090691	5090706
chr12_970	p2	(AC)6	12	5094347	5094358
chr12_971	p2	(AT)6	12	5109971	5109982
chr12_972	p2	(TG)6	12	5113425	5113436
chr12_973	p2	(AC)19	38	5117824	5117861
chr12_974	p2	(TG)6	12	5118984	5118995
chr12_975	p2	(AG)16	32	5141332	5141363
chr12_976	p2	(TG)13	26	5164537	5164562
chr12_977	p2	(GT)6	12	5166712	5166723
chr12_978	p3	(TTA)5	15	5169165	5169179
chr12_979	p2	(AC)6	12	5169530	5169541
chr12_980	p2	(AC)6	12	5172478	5172489
chr12_981	p2	(GT)7	14	5173453	5173466
chr12_982	p2	(CA)7	14	5178067	5178080
chr12_983	p2	(GT)20	40	5184067	5184106
chr12_984	p2	(AC)11	22	5185165	5185186
chr12_985	p2	(TC)7	14	5185314	5185327
chr12_986	p2	(GA)9	18	5185435	5185452
chr12_987	p2	(AC)12	24	5199132	5199155
chr12_988	p2	(GT)8	16	5199320	5199335
chr12_989	p2	(AC)9	18	5199492	5199509
chr12_990	p2	(TG)16	32	5201554	5201585
chr12_991	p2	(AC)19	38	5210879	5210916
chr12_992	p2	(GT)7	14	5215200	5215213
chr12_993	p2	(CA)14	28	5219208	5219235
chr12_994	p2	(AG)7	14	5222531	5222544
chr12_995	p2	(AC)6	12	5229370	5229381
chr12_996	p2	(CA)8	16	5229681	5229696
chr12_997	c	aactccagaccaaagattcaggattctggggaaagcagggtgtaagcactt	83	5231622	5231704
chr12_998	p2	(GA)6	12	5234577	5234588
chr12_999	p2	(AC)6	12	5239538	5239549
chr12_1000	p2	(CT)7	14	5242612	5242625
chr12_1001	p2	(TG)6	12	5251861	5251872

chr12_1002	p2	(AT)8	16	5252426	5252441
chr12_1003	p3	(AGC)5	15	5253961	5253975
chr12_1004	p2	(GT)9	18	5270861	5270878
chr12_1005	p2	(GT)6	12	5273813	5273824
chr12_1006	p2	(AC)13	26	5273950	5273975
chr12_1007	p3	(GAA)5	15	5279079	5279093
chr12_1008	p2	(CA)6	12	5284848	5284859
chr12_1009	p2	(TG)8	16	5287292	5287307
chr12_1010	p2	(GT)7	14	5287452	5287465
chr12_1011	p2	(CA)15	30	5288442	5288471
chr12_1012	p3	(TGT)6	18	5290903	5290920
chr12_1013	p2	(TG)7	14	5291643	5291656
chr12_1014	p2	(TG)13	26	5292450	5292475
chr12_1015	p2	(TG)6	12	5292809	5292820
chr12_1016	p2	(TG)6	12	5295939	5295950
chr12_1017	p2	(AC)7	14	5299211	5299224
chr12_1018	p2	(CA)6	12	5318883	5318894
chr12_1019	p2	(AC)13	26	5321362	5321387
chr12_1020	p3	(ATA)10	30	5322192	5322221
chr12_1021	p2	(TC)6	12	5323554	5323565
chr12_1022	p2	(AG)18	36	5323777	5323812
chr12_1023	c	T)11atgcgatgtaagccagatagaaaaaaaaaaatagagggac(GT)	82	5333380	5333461
chr12_1024	p2	(TG)6	12	5333913	5333924
chr12_1025	p2	(TG)7	14	5336456	5336469
chr12_1026	c	(TG)18tccctgagtgaacgaaa(AG)11	75	5338857	5338931
chr12_1027	p2	(CA)7	14	5344080	5344093
chr12_1028	p5	(TTGAT)5	25	5345248	5345272
chr12_1029	p2	(AG)8	16	5353171	5353186
chr12_1030	p2	(GT)6	12	5353439	5353450
chr12_1031	p2	(AG)7	14	5353958	5353971
chr12_1032	p2	(GT)11	22	5354805	5354826
chr12_1033	p2	(CA)8	16	5355575	5355590
chr12_1034	p2	(AC)6	12	5355978	5355989
chr12_1035	p2	(AC)15	30	5358876	5358905
chr12_1036	c	(TG)8tt(TG)7	32	5376878	5376909
chr12_1037	p2	(TG)10	20	5379227	5379246
chr12_1038	p2	(TA)6	12	5391112	5391123
chr12_1039	p2	(CA)12	24	5393888	5393911
chr12_1040	p3	(AAT)5	15	5394203	5394217
chr12_1041	p3	(CAG)7	21	5402992	5403012
chr12_1042	p2	(CA)15	30	5410350	5410379
chr12_1043	p2	(GT)10	20	5425678	5425697
chr12_1044	p2	(TG)7	14	5426036	5426049
chr12_1045	p2	(TG)7	14	5427921	5427934
chr12_1046	c	(TG)6c(GT)8	29	5429230	5429258
chr12_1047	c	acatgagcctcaaatgtacacagagtggataccatgcataattatgtacac	160	5430038	5430197
chr12_1048	p2	(AC)6	12	5432724	5432735
chr12_1049	p2	(CA)9	18	5435815	5435832
chr12_1050	p2	(CA)18	36	5436859	5436894
chr12_1051	p2	(TG)6	12	5438004	5438015
chr12_1052	p2	(CA)16	32	5458854	5458885
chr12_1053	p2	(CT)11	22	5461881	5461902

chr12_1054	p2	(TG)9	18	5479095	5479112
chr12_1055	p2	(AC)14	28	5481103	5481130
chr12_1056	p4	(AAAT)6	24	5483809	5483832
chr12_1057	p4	(AAAC)5	20	5484009	5484028
chr12_1058	p2	(AC)11	22	5486357	5486378
chr12_1059	p2	(AC)7	14	5492028	5492041
chr12_1060	p2	(TA)8	16	5548494	5548509
chr12_1061	p2	(AG)10	20	5554030	5554049
chr12_1062	p2	(AC)9	18	5559800	5559817
chr12_1063	c	(TG)9(TA)6	30	5564593	5564622
chr12_1064	c	(TG)9(TA)6	30	5567138	5567167
chr12_1065	c	(TG)9(TA)6	30	5569157	5569186
chr12_1066	p2	(AC)7	14	5585179	5585192
chr12_1067	p2	(GT)7	14	5585915	5585928
chr12_1068	p2	(TG)11	22	5593205	5593226
chr12_1069	p2	(TA)7	14	5600845	5600858
chr12_1070	p2	(GT)6	12	5606229	5606240
chr12_1071	p5	(TGATT)6	30	5608692	5608721
chr12_1072	p2	(TG)27	54	5615015	5615068
chr12_1073	p2	(TG)6	12	5615668	5615679
chr12_1074	p2	(GA)8	16	5622381	5622396
chr12_1075	p2	(TG)47	94	5629700	5629793
chr12_1076	p3	(GGA)5	15	5631116	5631130
chr12_1077	c	aaaccgagcataaactgtctttctcctttgctagaagccggagtgacagctt	112	5634131	5634242
chr12_1078	p2	(CA)6	12	5635792	5635803
chr12_1079	p2	(TG)6	12	5639423	5639434
chr12_1080	p2	(GT)16	32	5641689	5641720
chr12_1081	p2	(GT)13	26	5644065	5644090
chr12_1082	p2	(CA)9	18	5645133	5645150
chr12_1083	p2	(CA)9	18	5645347	5645364
chr12_1084	p2	(CT)8	16	5646632	5646647
chr12_1085	p2	(CT)13	26	5646838	5646863
chr12_1086	c	AC)6tccagcacccaccaattcaaatacatgtcaacatgtgcgtg(CA)	69	5647490	5647558
chr12_1087	p2	(CA)6	12	5648515	5648526
chr12_1088	p2	(AC)26	52	5649885	5649936
chr12_1089	p2	(CA)10	20	5650267	5650286
chr12_1090	p2	(AC)7	14	5651350	5651363
chr12_1091	c	(GT)6g(GT)30	73	5657542	5657614
chr12_1092	p2	(TG)7	14	5662166	5662179
chr12_1093	p2	(AC)16	32	5663678	5663709
chr12_1094	p2	(CT)7	14	5664482	5664495
chr12_1095	p2	(AC)12	24	5665311	5665334
chr12_1096	p2	(GT)10	20	5666111	5666130
chr12_1097	p2	(AC)16	32	5666374	5666405
chr12_1098	p2	(TG)6	12	5666823	5666834
chr12_1099	p2	(TG)18	36	5666962	5666997
chr12_1100	p2	(GT)21	42	5671428	5671469
chr12_1101	p2	(AC)7	14	5676533	5676546
chr12_1102	p2	(TG)6	12	5679063	5679074
chr12_1103	p2	(AC)7	14	5679420	5679433
chr12_1104	p2	(TG)7	14	5682228	5682241
chr12_1105	p2	(CA)12	24	5682886	5682909

chr12_1106	p2	(AC)9	18	5685640	5685657
chr12_1107	p2	(CA)12	24	5686021	5686044
chr12_1108	p2	(CA)6	12	5688735	5688746
chr12_1109	p2	(CA)8	16	5692366	5692381
chr12_1110	p2	(TG)11	22	5693776	5693797
chr12_1111	p2	(CA)16	32	5693973	5694004
chr12_1112	p2	(CA)7	14	5694561	5694574
chr12_1113	p2	(GT)15	30	5695027	5695056
chr12_1114	p2	(GT)6	12	5697709	5697720
chr12_1115	p2	(AC)10	20	5699804	5699823
chr12_1116	p2	(AC)9	18	5700004	5700021
chr12_1117	p2	(AC)19	38	5701311	5701348
chr12_1118	c	(CA)8cgcgcg(CA)20	62	5704982	5705043
chr12_1119	c	taaacatgatagcatgctacagatgacagatcatgaggttaaaacttaaag	135	5705927	5706061
chr12_1120	p2	(TG)15	30	5708111	5708140
chr12_1121	p2	(AT)11	22	5717080	5717101
chr12_1122	p2	(CA)17	34	5718970	5719003
chr12_1123	p2	(CA)14	28	5719802	5719829
chr12_1124	p4	(TATC)13	52	5725548	5725599
chr12_1125	p2	(TA)8	16	5745915	5745930
chr12_1126	p2	(AG)13	26	5757061	5757086
chr12_1127	p2	(TG)8	16	5759176	5759191
chr12_1128	p2	(GT)6	12	5764572	5764583
chr12_1129	p2	(CT)7	14	5776817	5776830
chr12_1130	p2	(TG)6	12	5780117	5780128
chr12_1131	p2	(TG)15	30	5789319	5789348
chr12_1132	p2	(TA)6	12	5792633	5792644
chr12_1133	p2	(TA)6	12	5796913	5796924
chr12_1134	p3	(ATT)5	15	5798643	5798657
chr12_1135	p2	(GT)30	60	5802475	5802534
chr12_1136	p2	(TC)13	26	5806677	5806702
chr12_1137	p2	(GT)19	38	5807305	5807342
chr12_1138	c	(GT)6gc(GT)21	56	5807503	5807558
chr12_1139	p2	(TG)8	16	5807700	5807715
chr12_1140	p2	(TG)6	12	5809368	5809379
chr12_1141	p2	(CA)6	12	5809546	5809557
chr12_1142	p2	(CA)17	34	5813649	5813682
chr12_1143	p2	(AC)25	50	5817546	5817595
chr12_1144	p2	(TG)23	46	5819346	5819391
chr12_1145	p3	(GGA)5	15	5822952	5822966
chr12_1146	p3	(CCT)5	15	5837545	5837559
chr12_1147	c	(TA)8(AT)6	28	5865004	5865031
chr12_1148	p4	(TTTA)6	24	5868100	5868123
chr12_1149	p2	(AG)7	14	5870525	5870538
chr12_1150	p2	(CA)10	20	5878095	5878114
chr12_1151	p2	(TC)6	12	5881586	5881597
chr12_1152	c	(TG)13tctgagagaa(AG)6	48	5881819	5881866
chr12_1153	p2	(TG)6	12	5882539	5882550
chr12_1154	c	cgtgcacacacactgtgcaggagtggaaagcacagatgtggtgtgtgtat	85	5884318	5884402
chr12_1155	p2	(AC)6	12	5884970	5884981
chr12_1156	p2	(AC)9	18	5886340	5886357
chr12_1157	p2	(CA)6	12	5886523	5886534

chr12_1158	p2	(CA)9	18	5887892	5887909
chr12_1159	p2	(TG)9	18	5888381	5888398
chr12_1160	p2	(TG)6	12	5889267	5889278
chr12_1161	p2	(CA)6	12	5891406	5891417
chr12_1162	p2	(AC)8	16	5891767	5891782
chr12_1163	c	:catatggccttctgaaaatgaactgttttgctctgacagaaggtgtgagaaa:	144	5893563	5893706
chr12_1164	p2	(TC)9	18	5896253	5896270
chr12_1165	p2	(CA)23	46	5899154	5899199
chr12_1166	p2	(GT)6	12	5899788	5899799
chr12_1167	p2	(TG)13	26	5899912	5899937
chr12_1168	p2	(CA)17	34	5900116	5900149
chr12_1169	p2	(GT)7	14	5901855	5901868
chr12_1170	p2	(AC)11	22	5902254	5902275
chr12_1171	p2	(AC)7	14	5902422	5902435
chr12_1172	p2	(AC)15	30	5902539	5902568
chr12_1173	c	(CT)8(CA)7	30	5902739	5902768
chr12_1174	p2	(AC)22	44	5904576	5904619
chr12_1175	c	(TG)12ttgcagtcagcacactgcatttgagcgtatga(GT)16	88	5905936	5906023
chr12_1176	p2	(TC)6	12	5906521	5906532
chr12_1177	p2	(CA)19	38	5907734	5907771
chr12_1178	c	(CA)10t(AC)6	33	5916316	5916348
chr12_1179	p2	(TC)15	30	5916711	5916740
chr12_1180	p2	(GA)7	14	5917850	5917863
chr12_1181	p2	(AG)7	14	5918093	5918106
chr12_1182	p3	(TTG)6	18	5919593	5919610
chr12_1183	p3	(TAT)5	15	5920456	5920470
chr12_1184	c	(GT)19gcgtgagagagaa(AG)7	65	5921284	5921348
chr12_1185	p2	(CA)7	14	5922061	5922074
chr12_1186	p2	(AC)13	26	5922239	5922264
chr12_1187	p2	(CA)9	18	5924883	5924900
chr12_1188	p2	(CA)24	48	5926005	5926052
chr12_1189	p2	(AC)16	32	5926221	5926252
chr12_1190	p2	(GT)18	36	5926381	5926416
chr12_1191	p2	(CA)14	28	5926974	5927001
chr12_1192	p2	(GA)13	26	5928974	5928999
chr12_1193	p2	(CA)7	14	5931313	5931326
chr12_1194	p2	(TG)6	12	5931440	5931451
chr12_1195	c	agagggagtgtgtgtagatataaatatgtgtgcaggatacatgactaaaaca	103	5932050	5932152
chr12_1196	p2	(TG)12	24	5932563	5932586
chr12_1197	p2	(CA)8	16	5932742	5932757
chr12_1198	p2	(AC)6	12	5936193	5936204
chr12_1199	p2	(TC)6	12	5942158	5942169
chr12_1200	p2	(CA)9	18	5942548	5942565
chr12_1201	p2	(CA)9	18	5942735	5942752
chr12_1202	p2	(TG)10	20	5947049	5947068
chr12_1203	p2	(TC)6	12	5949089	5949100
chr12_1204	p2	(AC)12	24	5950877	5950900
chr12_1205	p2	(TG)6	12	5953171	5953182
chr12_1206	p4	(TCCA)5	20	5953578	5953597
chr12_1207	p2	(CT)6	12	5957607	5957618
chr12_1208	p2	(AC)14	28	5961527	5961554
chr12_1209	p2	(AC)7	14	5961679	5961692

chr12_1210	c	gttgaaactgttgatctggagcaaacaggaccaatgtttcagttttgttgtgttg	104	5964974	5965077
chr12_1211	p2	(CA)6	12	5967916	5967927
chr12_1212	p2	(TG)6	12	5969483	5969494
chr12_1213	p3	(TAA)5	15	5970424	5970438
chr12_1214	p2	(CA)6	12	5971330	5971341
chr12_1215	p2	(TG)7	14	5971972	5971985
chr12_1216	p2	(TG)6	12	5974077	5974088
chr12_1217	p2	(CA)18	36	5980159	5980194
chr12_1218	p2	(CA)7	14	5980693	5980706
chr12_1219	p2	(AC)6	12	5980820	5980831
chr12_1220	p2	(TG)6	12	5981399	5981410
chr12_1221	p2	(CA)10	20	5981674	5981693
chr12_1222	c	(CA)6t(AC)11	35	5981824	5981858
chr12_1223	p2	(CA)6	12	5984767	5984778
chr12_1224	p2	(CA)6	12	5989389	5989400
chr12_1225	p3	(TAA)6	18	5997631	5997648
chr12_1226	p2	(CA)8	16	6005059	6005074
chr12_1227	p3	(TAT)7	21	6007543	6007563
chr12_1228	p2	(TG)15	30	6011543	6011572
chr12_1229	p2	(TG)19	38	6018985	6019022
chr12_1230	p3	(TAC)6	18	6019348	6019365
chr12_1231	p2	(TC)7	14	6020388	6020401
chr12_1232	p2	(CA)6	12	6022468	6022479
chr12_1233	p2	(CA)7	14	6023194	6023207
chr12_1234	p4	(TAGA)17	68	6039862	6039929
chr12_1235	p5	(TGATT)6	30	6042514	6042543
chr12_1236	p2	(CT)7	14	6046019	6046032
chr12_1237	p2	(TG)6	12	6049322	6049333
chr12_1238	p2	(GT)11	22	6058806	6058827
chr12_1239	p2	(CA)10	20	6068844	6068863
chr12_1240	p2	(TG)10	20	6074033	6074052
chr12_1241	p2	(CT)8	16	6076654	6076669
chr12_1242	p2	(TG)11	22	6088762	6088783
chr12_1243	p2	(CA)7	14	6090590	6090603
chr12_1244	p2	(AC)6	12	6091049	6091060
chr12_1245	c	(GT)6g(GT)30	73	6096505	6096577
chr12_1246	p2	(TG)6	12	6099829	6099840
chr12_1247	p3	(TTA)7	21	6102841	6102861
chr12_1248	p5	(AAGCA)7	35	6106189	6106223
chr12_1249	c	(TC)11ttcatatctatctctcc(CT)6	51	6106332	6106382
chr12_1250	p2	(TG)14	28	6108777	6108804
chr12_1251	p2	(TG)17	34	6108948	6108981
chr12_1252	p2	(TG)16	32	6109125	6109156
chr12_1253	p2	(CA)9	18	6109403	6109420
chr12_1254	p2	(TG)9	18	6115790	6115807
chr12_1255	p2	(TG)20	40	6117164	6117203
chr12_1256	p2	(TG)6	12	6127760	6127771
chr12_1257	p2	(TG)7	14	6129121	6129134
chr12_1258	p2	(GT)6	12	6129322	6129333
chr12_1259	p2	(TG)8	16	6134484	6134499
chr12_1260	p2	(TA)7	14	6141716	6141729
chr12_1261	p2	(CT)9	18	6151128	6151145

chr12_1262	c	(GT)13gct(TG)6	41	6158343	6158383
chr12_1263	p3	(ACA)5	15	6167288	6167302
chr12_1264	c	caggtgtgtgtaaggaaaaggagtgcaggagctgaagccggctatctgct	197	6188443	6188639
chr12_1265	p2	(CA)23	46	6207565	6207610
chr12_1266	p2	(TG)7	14	6207817	6207830
chr12_1267	p3	(TAA)10	30	6228626	6228655
chr12_1268	p2	(GT)9	18	6252732	6252749
chr12_1269	c	(GT)9gagagag(AC)9	43	6253685	6253727
chr12_1270	p3	(GAT)5	15	6270827	6270841
chr12_1271	p2	(GT)7	14	6277383	6277396
chr12_1272	p2	(CA)13	26	6279318	6279343
chr12_1273	p3	(AGG)5	15	6286625	6286639
chr12_1274	c	(TG)6acctcgagattcctctgagccgttatcagtatctgtgagac(GT)6	65	6297223	6297287
chr12_1275	p2	(GT)13	26	6350306	6350331
chr12_1276	p5	(AAATC)15	75	6368235	6368309
chr12_1277	p2	(CA)6	12	6372035	6372046
chr12_1278	p2	(AC)32	64	6377834	6377897
chr12_1279	p2	(TG)6	12	6399270	6399281
chr12_1280	p2	(AC)9	18	6401489	6401506
chr12_1281	p2	(AG)6	12	6408268	6408279
chr12_1282	p2	(TG)6	12	6409257	6409268
chr12_1283	c	(TG)6tctgctgctcgctccttgggg(GT)9	51	6412610	6412660
chr12_1284	p2	(GT)9	18	6416632	6416649
chr12_1285	p2	(AG)6	12	6417971	6417982
chr12_1286	p2	(AC)6	12	6419668	6419679
chr12_1287	p2	(TA)7	14	6429847	6429860
chr12_1288	c	(TG)11aa(AG)6g(GA)7	51	6433774	6433824
chr12_1289	p2	(CA)13	26	6435195	6435220
chr12_1290	p2	(CA)8	16	6448301	6448316
chr12_1291	p2	(TA)9	18	6451306	6451323
chr12_1292	p3	(CTT)7	21	6461999	6462019
chr12_1293	c*	(ACAT)10(AT)8*	54	6463247	6463300
chr12_1294	p2	(GT)6	12	6465194	6465205
chr12_1295	p2	(AC)6	12	6476988	6476999
chr12_1296	p3	(ATA)5	15	6478913	6478927
chr12_1297	p2	(GT)10	20	6480897	6480916
chr12_1298	p2	(AT)14	28	6501202	6501229
chr12_1299	p2	(AC)7	14	6506286	6506299
chr12_1300	p4	(CCAT)5	20	6537491	6537510
chr12_1301	p2	(AC)21	42	6553313	6553354
chr12_1302	c	(AC)8gg(AC)7	32	6570973	6571004
chr12_1303	c	(AGA)5cgat(GAC)5	34	6581859	6581892
chr12_1304	p3	(TTG)5	15	6589490	6589504
chr12_1305	p5	(AAATC)5	25	6602647	6602671
chr12_1306	p2	(TG)14	28	6621206	6621233
chr12_1307	p2	(TG)14	28	6639570	6639597
chr12_1308	c	(TG)11ta(TG)24	72	6660732	6660803
chr12_1309	p3	(TTA)5	15	6669281	6669295
chr12_1310	p2	(TG)6	12	6675861	6675872
chr12_1311	p3	(TAT)5	15	6681034	6681048
chr12_1312	p2	(TG)19	38	6691197	6691234
chr12_1313	p2	(AC)8	16	6693317	6693332

chr12_1314	p2	(AC)6	12	6698207	6698218
chr12_1315	p2	(TC)6	12	6727900	6727911
chr12_1316	p2	(CT)8	16	6742569	6742584
chr12_1317	p2	(TG)7	14	6748274	6748287
chr12_1318	p4	(CATC)5	20	6788956	6788975
chr12_1319	p3	(CCT)5	15	6803607	6803621
chr12_1320	p2	(AC)21	42	6804313	6804354
chr12_1321	p2	(TG)6	12	6815026	6815037
chr12_1322	p2	(TG)11	22	6828838	6828859
chr12_1323	p3	(ATA)6	18	6828964	6828981
chr12_1324	p2	(TG)6	12	6838982	6838993
chr12_1325	p2	(GT)6	12	6839126	6839137
chr12_1326	c	(TC)7tt(TC)6c(AT)9	47	6840439	6840485
chr12_1327	p2	(GT)7	14	6841821	6841834
chr12_1328	p2	(TG)6	12	6842208	6842219
chr12_1329	p2	(GA)6	12	6843747	6843758
chr12_1330	p2	(GT)16	32	6857116	6857147
chr12_1331	p2	(AC)7	14	6858918	6858931
chr12_1332	p2	(GT)8	16	6869603	6869618
chr12_1333	p2	(GT)16	32	6883905	6883936
chr12_1334	p2	(AC)9	18	6886938	6886955
chr12_1335	p5	(ACAGG)13	65	6895605	6895669
chr12_1336	p4	(TTCA)7	28	6917942	6917969
chr12_1337	p2	(TA)8	16	6936946	6936961
chr12_1338	p2	(CA)16	32	6937283	6937314
chr12_1339	p2	(TA)6	12	6940138	6940149
chr12_1340	p2	(AC)13	26	6951435	6951460
chr12_1341	p2	(AG)12	24	6955537	6955560
chr12_1342	p3	(TAT)7	21	6957477	6957497
chr12_1343	p2	(AG)6	12	6968244	6968255
chr12_1344	p2	(AC)6	12	6969508	6969519
chr12_1345	p4	(AACA)6	24	6995858	6995881
chr12_1346	p2	(CA)6	12	7006935	7006946
chr12_1347	p2	(AT)6	12	7014432	7014443
chr12_1348	p2	(CA)21	42	7015822	7015863
chr12_1349	p2	(AG)11	22	7021255	7021276
chr12_1350	p2	(AC)23	46	7037822	7037867
chr12_1351	p2	(CA)33	66	7038707	7038772
chr12_1352	p2	(AC)11	22	7044241	7044262
chr12_1353	p2	(AC)11	22	7071077	7071098
chr12_1354	p2	(GA)8	16	7071454	7071469
chr12_1355	p2	(CA)16	32	7086467	7086498
chr12_1356	p2	(TG)10	20	7087297	7087316
chr12_1357	p3	(TAC)10	30	7094993	7095022
chr12_1358	p3	(CCT)5	15	7095878	7095892
chr12_1359	p2	(TG)26	52	7102444	7102495
chr12_1360	p3	(ATA)5	15	7110049	7110063
chr12_1361	p2	(GT)6	12	7112122	7112133
chr12_1362	p2	(GA)11	22	7114250	7114271
chr12_1363	c	(TA)12cacacat(AC)8	47	7116120	7116166
chr12_1364	p3	(TTA)6	18	7117169	7117186
chr12_1365	p2	(GA)9	18	7117460	7117477

chr12_1366	c	(GAA)10g(GAA)5	46	7120702	7120747
chr12_1367	p3	(TAG)15	45	7130553	7130597
chr12_1368	p2	(TG)6	12	7143002	7143013
chr12_1369	p3	(TCA)7	21	7154864	7154884
chr12_1370	p3	(TAC)6	18	7156844	7156861
chr12_1371	p2	(CA)9	18	7162026	7162043
chr12_1372	p2	(GA)6	12	7187043	7187054
chr12_1373	p3	(CTT)11	33	7189901	7189933
chr12_1374	p2	(AT)8	16	7197974	7197989
chr12_1375	p2	(GT)8	16	7201700	7201715
chr12_1376	p3	(TTG)5	15	7216333	7216347
chr12_1377	p2	(CA)6	12	7220397	7220408
chr12_1378	p2	(TG)6	12	7221403	7221414
chr12_1379	p3	(TTA)5	15	7250030	7250044
chr12_1380	p2	(CA)22	44	7250433	7250476
chr12_1381	c	(TCTA)207(TCTT)11	872	7261426	7262297
chr12_1382	p3	(TTA)5	15	7279332	7279346
chr12_1383	p2	(CA)6	12	7280618	7280629
chr12_1384	p4	(AGCT)6	24	7282596	7282619
chr12_1385	p2	(TG)15	30	7285024	7285053
chr12_1386	p2	(GT)11	22	7286674	7286695
chr12_1387	p3	(ATA)5	15	7289995	7290009
chr12_1388	p2	(AG)6	12	7297563	7297574
chr12_1389	p2	(TG)24	48	7298256	7298303
chr12_1390	p3	(AGT)8	24	7300779	7300802
chr12_1391	p2	(GT)7	14	7302315	7302328
chr12_1392	p2	(TG)14	28	7307687	7307714
chr12_1393	p2	(TA)7	14	7307819	7307832
chr12_1394	p2	(TG)9	18	7317996	7318013
chr12_1395	c	(CAA)6(TAA)5	33	7324864	7324896
chr12_1396	p3	(TTA)9	27	7325612	7325638
chr12_1397	p2	(TC)6	12	7336184	7336195
chr12_1398	p3	(ATA)6	18	7336341	7336358
chr12_1399	p2	(TG)20	40	7336777	7336816
chr12_1400	p2	(CA)6	12	7338863	7338874
chr12_1401	p2	(AC)9	18	7340032	7340049
chr12_1402	p2	(GT)8	16	7355469	7355484
chr12_1403	p2	(TC)12	24	7361476	7361499
chr12_1404	p2	(CT)8	16	7363129	7363144
chr12_1405	p2	(CA)9	18	7366444	7366461
chr12_1406	p2	(TC)8	16	7382598	7382613
chr12_1407	p2	(AT)8	16	7391124	7391139
chr12_1408	p2	(GT)11	22	7393666	7393687
chr12_1409	p2	(AC)6	12	7402723	7402734
chr12_1410	c	(TG)20c(GT)10	61	7405602	7405662
chr12_1411	p2	(AC)14	28	7408817	7408844
chr12_1412	p2	(AC)25	50	7416051	7416100
chr12_1413	p3	(AAC)5	15	7436053	7436067
chr12_1414	p3	(ATT)8	24	7456526	7456549
chr12_1415	p2	(TG)6	12	7473187	7473198
chr12_1416	p3	(TTG)8	24	7473858	7473881
chr12_1417	p2	(TG)8	16	7508866	7508881

chr12_1418	p4	(ATCT)5	20	7509290	7509309
chr12_1419	p5	(ATTTG)11	55	7520305	7520359
chr12_1420	p3	(ACA)5	15	7533189	7533203
chr12_1421	p2	(CT)6	12	7538996	7539007
chr12_1422	p3	(TTA)5	15	7543877	7543891
chr12_1423	p3	(AAT)5	15	7547076	7547090
chr12_1424	p2	(CT)6	12	7569484	7569495
chr12_1425	p2	(TC)9	18	7582312	7582329
chr12_1426	p2	(TG)22	44	7589531	7589574
chr12_1427	p2	(TC)6	12	7595614	7595625
chr12_1428	p2	(AT)6	12	7620067	7620078
chr12_1429	p4	(GGTA)5	20	7632632	7632651
chr12_1430	p4	(TTTA)5	20	7634936	7634955
chr12_1431	p4	(AATG)5	20	7638405	7638424
chr12_1432	p2	(GA)6	12	7656533	7656544
chr12_1433	p3	(ATT)8	24	7658313	7658336
chr12_1434	p2	(TG)13	26	7662097	7662122
chr12_1435	p2	(TC)11	22	7664828	7664849
chr12_1436	p2	(AC)6	12	7706569	7706580
chr12_1437	p2	(TA)7	14	7728515	7728528
chr12_1438	c	(CA)8cg(CA)9(TA)6	48	7733322	7733369
chr12_1439	p2	(CT)7	14	7736228	7736241
chr12_1440	p2	(GT)10	20	7743221	7743240
chr12_1441	p2	(AT)9	18	7768540	7768557
chr12_1442	p2	(AT)9	18	7770822	7770839
chr12_1443	p2	(GT)10	20	7783780	7783799
chr12_1444	p3	(ATA)5	15	7800002	7800016
chr12_1445	p2	(GT)11	22	7804765	7804786
chr12_1446	p2	(CA)10	20	7812863	7812882
chr12_1447	p2	(GT)10	20	7813662	7813681
chr12_1448	p3	(TTG)5	15	7824660	7824674
chr12_1449	p5	(AAATA)5	25	7833290	7833314
chr12_1450	p3	(TTA)8	24	7836771	7836794
chr12_1451	p2	(AG)6	12	7841286	7841297
chr12_1452	p2	(TG)6	12	7843058	7843069
chr12_1453	c	(AC)8ag(AC)10	38	7861816	7861853
chr12_1454	c	(CT)7cg(CT)18	52	7884873	7884924
chr12_1455	p2	(TA)8	16	7888851	7888866
chr12_1456	p2	(CA)6	12	7889232	7889243
chr12_1457	p2	(CA)16	32	7908557	7908588
chr12_1458	p4	(ATCC)5	20	7927558	7927577
chr12_1459	p3	(ATA)5	15	7928656	7928670
chr12_1460	p2	(TA)7	14	7948747	7948760
chr12_1461	p2	(AC)15	30	7961178	7961207
chr12_1462	p3	(ATC)5	15	7964952	7964966
chr12_1463	p2	(TG)8	16	7982891	7982906
chr12_1464	p2	(CT)6	12	7984885	7984896
chr12_1465	p2	(TG)9	18	7988172	7988189
chr12_1466	p3	(CAA)5	15	7998476	7998490
chr12_1467	p2	(GT)6	12	7999825	7999836
chr12_1468	c	(CT)8(CA)7	30	8009611	8009640
chr12_1469	p2	(AG)6	12	8022284	8022295

chr12_1470	p2	(TG)21	42	8025280	8025321
chr12_1471	p5	(AAATC)5	25	8072383	8072407
chr12_1472	p2	(AC)17	34	8080389	8080422
chr12_1473	p3	(CAG)5	15	8082526	8082540
chr12_1474	p2	(GT)6	12	8089810	8089821
chr12_1475	p2	(CA)13	26	8097001	8097026
chr12_1476	p2	(CA)6	12	8097996	8098007
chr12_1477	p2	(TG)10	20	8116872	8116891
chr12_1478	p2	(CT)8	16	8118013	8118028
chr12_1479	p2	(TC)7	14	8130827	8130840
chr12_1480	p2	(TG)11	22	8134636	8134657
chr12_1481	p2	(CA)6	12	8143925	8143936
chr12_1482	p2	(AG)6	12	8146513	8146524
chr12_1483	p2	(AC)7	14	8172421	8172434
chr12_1484	p3	(CCT)5	15	8181130	8181144
chr12_1485	p3	(TCC)6	18	8181467	8181484
chr12_1486	p3	(ATT)8	24	8181675	8181698
chr12_1487	p2	(AT)6	12	8184382	8184393
chr12_1488	p2	(TC)8	16	8186423	8186438
chr12_1489	p2	(AC)8	16	8197844	8197859
chr12_1490	p2	(AC)15	30	8209048	8209077
chr12_1491	p2	(CA)6	12	8210931	8210942
chr12_1492	p2	(AG)7	14	8211540	8211553
chr12_1493	p2	(GA)7	14	8218410	8218423
chr12_1494	p2	(TG)20	40	8219304	8219343
chr12_1495	p2	(AC)10	20	8219454	8219473
chr12_1496	p2	(GT)8	16	8224947	8224962
chr12_1497	p2	(TG)10	20	8229812	8229831
chr12_1498	p2	(GT)6	12	8244138	8244149
chr12_1499	p2	(GT)10	20	8252621	8252640
chr12_1500	p3	(TTA)6	18	8254980	8254997
chr12_1501	p2	(CA)6	12	8268827	8268838
chr12_1502	c	(TATG)13(GA)9	70	8269589	8269658
chr12_1503	p2	(TG)7	14	8272902	8272915
chr12_1504	p2	(AC)14	28	8275554	8275581
chr12_1505	p4	(TGAA)5	20	8316543	8316562
chr12_1506	p2	(TA)6	12	8320176	8320187
chr12_1507	p2	(CA)13	26	8343342	8343367
chr12_1508	p2	(GT)8	16	8344863	8344878
chr12_1509	p2	(AC)13	26	8358703	8358728
chr12_1510	p4	(CATT)5	20	8363211	8363230
chr12_1511	p2	(CA)13	26	8363357	8363382
chr12_1512	c	(CA)8g(AC)7	31	8364762	8364792
chr12_1513	p2	(TG)6	12	8373816	8373827
chr12_1514	p2	(TG)9	18	8389224	8389241
chr12_1515	p2	(GA)7	14	8400774	8400787
chr12_1516	p2	(TC)7	14	8414644	8414657
chr12_1517	p2	(AG)7	14	8422349	8422362
chr12_1518	p2	(AC)7	14	8423213	8423226
chr12_1519	p3	(TAA)8	24	8432321	8432344
chr12_1520	p2	(GT)9	18	8454022	8454039
chr12_1521	p2	(GT)8	16	8454982	8454997

chr12_1522	p2	(CA)16	32	8457205	8457236
chr12_1523	p2	(AC)10	20	8463138	8463157
chr12_1524	p3	(AAT)6	18	8472223	8472240
chr12_1525	p3	(TCT)5	15	8509186	8509200
chr12_1526	p3	(TAT)8	24	8512767	8512790
chr12_1527	p2	(AT)7	14	8518968	8518981
chr12_1528	p3	(AGC)5	15	8519740	8519754
chr12_1529	p2	(GT)6	12	8528607	8528618
chr12_1530	p2	(TG)6	12	8534797	8534808
chr12_1531	p2	(CA)14	28	8539520	8539547
chr12_1532	p2	(TG)7	14	8541373	8541386
chr12_1533	p2	(GT)6	12	8563041	8563052
chr12_1534	p3	(ATG)5	15	8565289	8565303
chr12_1535	p3	(TTG)5	15	8573464	8573478
chr12_1536	p2	(CT)8	16	8603104	8603119
chr12_1537	p3	(TAA)5	15	8604469	8604483
chr12_1538	p2	(TG)17	34	8609483	8609516
chr12_1539	c	ttctggttaaggttagtcaacaggaaatgcatgtaaatcaatatggacaacaa	134	8651585	8651718
chr12_1540	p2	(AC)8	16	8653741	8653756
chr12_1541	c*	(AG)6(GAGAG)5*	32	8679167	8679198
chr12_1542	p3	(TCT)5	15	8686472	8686486
chr12_1543	p2	(GT)6	12	8705755	8705766
chr12_1544	p2	(TG)6	12	8713377	8713388
chr12_1545	p2	(TA)7	14	8715697	8715710
chr12_1546	p2	(TA)7	14	8723916	8723929
chr12_1547	c	cagatggttcagcacagagagcgtaattgaaaagaggggtcagggtgga	99	8730474	8730572
chr12_1548	p2	(CA)7	14	8760071	8760084
chr12_1549	p2	(CA)6	12	8768123	8768134
chr12_1550	p2	(CA)16	32	8771206	8771237
chr12_1551	p4	(AATC)7	28	8787705	8787732
chr12_1552	p6	(TCTAGG)6	36	8840545	8840580
chr12_1553	p2	(AC)12	24	8852600	8852623
chr12_1554	p2	(AC)8	16	8856055	8856070
chr12_1555	p3	(TAA)5	15	8877397	8877411
chr12_1556	p2	(GA)10	20	8878072	8878091
chr12_1557	p3	(TAA)5	15	8881918	8881932
chr12_1558	p3	(TAA)5	15	8884383	8884397
chr12_1559	p2	(AC)6	12	8885129	8885140
chr12_1560	p4	(AAAT)6	24	8896747	8896770
chr12_1561	p2	(TG)13	26	8903420	8903445
chr12_1562	p3	(ATA)5	15	8921993	8922007
chr12_1563	p2	(AC)7	14	8924452	8924465
chr12_1564	p3	(TCT)6	18	8943762	8943779
chr12_1565	p2	(GT)7	14	8950031	8950044
chr12_1566	c	(TG)8c(GT)11	39	8960121	8960159
chr12_1567	p2	(CA)6	12	8976280	8976291
chr12_1568	p2	(CA)10	20	8992006	8992025
chr12_1569	p3	(TTA)5	15	8992223	8992237
chr12_1570	p2	(CA)6	12	8999592	8999603
chr12_1571	p2	(TG)15	30	8999899	8999928
chr12_1572	p4	(GATG)7	28	9021751	9021778
chr12_1573	p3	(AAG)5	15	9024789	9024803

chr12_1574	p2	(CA)14	28	9028325	9028352
chr12_1575	p2	(GT)6	12	9031984	9031995
chr12_1576	p3	(CTC)7	21	9044407	9044427
chr12_1577	p2	(AC)7	14	9061635	9061648
chr12_1578	p5	(ATTTG)5	25	9065878	9065902
chr12_1579	p4	(ATGG)6	24	9070650	9070673
chr12_1580	p3	(ATA)5	15	9079014	9079028
chr12_1581	p3	(TTA)7	21	9088296	9088316
chr12_1582	p3	(GTG)6	18	9092155	9092172
chr12_1583	p3	(CTT)5	15	9095356	9095370
chr12_1584	p2	(TG)6	12	9118526	9118537
chr12_1585	p2	(GA)6	12	9119231	9119242
chr12_1586	p3	(CTT)5	15	9125509	9125523
chr12_1587	p2	(TG)6	12	9134209	9134220
chr12_1588	p2	(TG)6	12	9141703	9141714
chr12_1589	p3	(AAT)5	15	9144650	9144664
chr12_1590	p2	(GT)6	12	9152407	9152418
chr12_1591	p3	(ATT)5	15	9157114	9157128
chr12_1592	p2	(TA)7	14	9178051	9178064
chr12_1593	p2	(TG)6	12	9178811	9178822
chr12_1594	p3	(CTT)5	15	9183492	9183506
chr12_1595	p3	(CCA)5	15	9185354	9185368
chr12_1596	p4	(ATGG)5	20	9194415	9194434
chr12_1597	p2	(TA)6	12	9206336	9206347
chr12_1598	p2	(AC)6	12	9216854	9216865
chr12_1599	c	(TG)12(TA)10gatagataga(TG)6	66	9230076	9230141
chr12_1600	p3	(AAT)6	18	9234816	9234833
chr12_1601	p2	(AC)13	26	9258837	9258862
chr12_1602	p3	(TGT)6	18	9264261	9264278
chr12_1603	p3	(TTG)6	18	9265746	9265763
chr12_1604	p2	(AC)7	14	9276575	9276588
chr12_1605	p2	(GT)16	32	9281652	9281683
chr12_1606	p2	(TG)6	12	9282315	9282326
chr12_1607	p2	(AT)9	18	9291365	9291382
chr12_1608	p2	(TC)9	18	9296111	9296128
chr12_1609	p2	(GT)7	14	9296588	9296601
chr12_1610	p3	(TGT)5	15	9299663	9299677
chr12_1611	c	(CT)10(CA)7	34	9314730	9314763
chr12_1612	p4	(GTTT)5	20	9325966	9325985
chr12_1613	p2	(CA)6	12	9328758	9328769
chr12_1614	p2	(TG)23	46	9331846	9331891
chr12_1615	c	(TA)6(TG)18	48	9335554	9335601
chr12_1616	p2	(TC)8	16	9350517	9350532
chr12_1617	p3	(ACA)5	15	9351775	9351789
chr12_1618	p4	(TTTC)6	24	9354904	9354927
chr12_1619	p3	(GAT)5	15	9357234	9357248
chr12_1620	p3	(ATC)5	15	9357729	9357743
chr12_1621	p2	(CA)6	12	9365558	9365569
chr12_1622	p2	(AG)6	12	9374778	9374789
chr12_1623	p2	(TG)7	14	9376703	9376716
chr12_1624	p2	(GT)18	36	9376896	9376931
chr12_1625	p3	(CCA)6	18	9389218	9389235

chr12_1626	p3	(TCT)9	27	9390123	9390149
chr12_1627	p2	(TG)8	16	9396046	9396061
chr12_1628	p2	(CT)8	16	9397038	9397053
chr12_1629	p2	(CA)13	26	9403782	9403807
chr12_1630	p2	(TG)6	12	9409105	9409116
chr12_1631	p2	(TG)25	50	9409791	9409840
chr12_1632	p2	(GA)8	16	9415742	9415757
chr12_1633	p4	(TTTA)5	20	9430520	9430539
chr12_1634	p2	(CA)6	12	9446753	9446764
chr12_1635	p2	(AT)16	32	9459327	9459358
chr12_1636	p2	(GT)14	28	9461776	9461803
chr12_1637	p2	(TG)15	30	9462765	9462794
chr12_1638	p3	(TAT)5	15	9463359	9463373
chr12_1639	p2	(AT)6	12	9468965	9468976
chr12_1640	p2	(AC)12	24	9475605	9475628
chr12_1641	p3	(CTG)5	15	9480650	9480664
chr12_1642	p2	(AC)7	14	9487514	9487527
chr12_1643	p3	(TAA)7	21	9494798	9494818
chr12_1644	p3	(TTA)6	18	9495529	9495546
chr12_1645	p3	(ACA)6	18	9495789	9495806
chr12_1646	p3	(CCT)5	15	9510109	9510123
chr12_1647	p3	(TTA)7	21	9510579	9510599
chr12_1648	p2	(TC)6	12	9550095	9550106
chr12_1649	p2	(AC)12	24	9552278	9552301
chr12_1650	p2	(CA)24	48	9554331	9554378
chr12_1651	c	ggaaggcatatccccacaatgtcagtgtgtaaacaggttttagtcttcacaat	123	9565242	9565364
chr12_1652	c	(TG)6ttagagg(GA)7	33	9574627	9574659
chr12_1653	p2	(GT)6	12	9578547	9578558
chr12_1654	p3	(AAT)5	15	9604986	9605000
chr12_1655	p3	(TCT)8	24	9615485	9615508
chr12_1656	p2	(CA)15	30	9637703	9637732
chr12_1657	p3	(TGT)6	18	9642642	9642659
chr12_1658	p3	(TAA)7	21	9643688	9643708
chr12_1659	p2	(GT)8	16	9679414	9679429
chr12_1660	p4	(TGTA)5	20	9682496	9682515
chr12_1661	p3	(TTG)5	15	9699528	9699542
chr12_1662	p2	(TC)6	12	9738503	9738514
chr12_1663	p2	(TC)11	22	9739203	9739224
chr12_1664	p5	(GATTT)5	25	9741698	9741722
chr12_1665	p2	(AC)8	16	9758503	9758518
chr12_1666	p2	(CA)13	26	9771618	9771643
chr12_1667	p2	(AG)23	46	9784245	9784290
chr12_1668	p4	(AAAC)5	20	9789298	9789317
chr12_1669	p2	(AC)9	18	9798576	9798593
chr12_1670	p2	(CA)20	40	9801971	9802010
chr12_1671	p4	(TGAC)7	28	9810176	9810203
chr12_1672	p2	(GT)20	40	9811762	9811801
chr12_1673	p2	(GT)25	50	9813912	9813961
chr12_1674	p3	(TCA)7	21	9836254	9836274
chr12_1675	p3	(GTC)5	15	9837257	9837271
chr12_1676	p2	(CA)12	24	9861700	9861723
chr12_1677	c	atgtttttgttgtccatattgatttacatgcatttcctgttgactaaccttaaccag	156	9862562	9862717

chr12_1678	p4	(TTTA)6	24	9896500	9896523
chr12_1679	p2	(CA)11	22	9905245	9905266
chr12_1680	p2	(AC)6	12	9939510	9939521
chr12_1681	p3	(GCA)5	15	9940671	9940685
chr12_1682	p4	(TACA)7	28	9944850	9944877
chr12_1683	p2	(AC)8	16	9945126	9945141
chr12_1684	p4	(TACA)5	20	9947911	9947930
chr12_1685	p2	(AC)8	16	9948179	9948194
chr12_1686	p2	(CA)13	26	9951267	9951292
chr12_1687	p2	(TG)21	42	9970193	9970234
chr12_1688	p2	(GT)6	12	9971320	9971331
chr12_1689	p2	(TG)15	30	9972766	9972795
chr12_1690	p3	(TTA)5	15	9992156	9992170
chr12_1691	p2	(GT)6	12	9996126	9996137
chr12_1692	p2	(AC)16	32	10005682	10005713
chr12_1693	p3	(ATA)5	15	10019972	10019986
chr12_1694	p2	(CA)6	12	10042141	10042152
chr12_1695	p4	(ATAA)5	20	10046518	10046537
chr12_1696	p2	(TA)10	20	10055217	10055236
chr12_1697	p3	(AAT)5	15	10061880	10061894
chr12_1698	p2	(CA)8	16	10065646	10065661
chr12_1699	p2	(CT)9	18	10088224	10088241
chr12_1700	p2	(CT)6	12	10117924	10117935
chr12_1701	p2	(GT)8	16	10120477	10120492
chr12_1702	p2	(AC)8	16	10123931	10123946
chr12_1703	p2	(TG)7	14	10129104	10129117
chr12_1704	p2	(TG)14	28	10133781	10133808
chr12_1705	p4	(ATCC)5	20	10135003	10135022
chr12_1706	p2	(GT)6	12	10138502	10138513
chr12_1707	p3	(ATT)5	15	10141983	10141997
chr12_1708	p2	(AC)10	20	10152921	10152940
chr12_1709	p2	(TG)8	16	10153815	10153830
chr12_1710	p2	(AC)6	12	10160557	10160568
chr12_1711	p3	(AGA)5	15	10162900	10162914
chr12_1712	p2	(TG)7	14	10170048	10170061
chr12_1713	p2	(TG)12	24	10170262	10170285
chr12_1714	c	tacccgtctggctcacccatgcatgcaacagattttctttccaccaatcaga[illegible]	123	10173506	10173628
chr12_1715	p2	(AT)6	12	10181283	10181294
chr12_1716	p2	(CA)6	12	10182185	10182196
chr12_1717	p3	(TAA)8	24	10191864	10191887
chr12_1718	p2	(CA)8	16	10196385	10196400
chr12_1719	p3	(ATC)9	27	10197181	10197207
chr12_1720	p2	(CA)6	12	10197602	10197613
chr12_1721	c	(AT)8gcagtcagtcatgcatatatgtatgcagt(TG)6	57	10199671	10199727
chr12_1722	p2	(CA)6	12	10201110	10201121
chr12_1723	p2	(AC)10	20	10207963	10207982
chr12_1724	p2	(CT)10	20	10210137	10210156
chr12_1725	p4	(AAAT)5	20	10216860	10216879
chr12_1726	p2	(AG)8	16	10217055	10217070
chr12_1727	p2	(GT)9	18	10219353	10219370
chr12_1728	p2	(TG)13	26	10223861	10223886
chr12_1729	c	[illegible]g(GT)11gctggtttgtatttctgctatnnnnnnnnnnnnaggtgtgttc[illegible]	144	10229354	10229497

chr12_1730	p2	(TG)6	12	10235441	10235452
chr12_1731	p2	(TG)6	12	10235611	10235622
chr12_1732	p2	(GT)7	14	10236088	10236101
chr12_1733	p2	(TC)7	14	10238083	10238096
chr12_1734	c	(TC)6atgcact(CA)6	31	10241195	10241225
chr12_1735	p2	(TG)8	16	10251477	10251492
chr12_1736	p2	(TG)6	12	10264258	10264269
chr12_1737	p2	(AC)6	12	10274074	10274085
chr12_1738	p2	(AC)6	12	10277518	10277529
chr12_1739	p2	(TC)9	18	10277648	10277665
chr12_1740	p2	(CA)7	14	10282322	10282335
chr12_1741	p2	(CA)6	12	10284001	10284012
chr12_1742	p2	(AC)8	16	10289306	10289321
chr12_1743	p2	(AG)6	12	10292321	10292332
chr12_1744	p2	(CA)7	14	10293026	10293039
chr12_1745	p2	(CA)26	52	10294787	10294838
chr12_1746	p3	(AAT)6	18	10295150	10295167
chr12_1747	p2	(GT)6	12	10295297	10295308
chr12_1748	p3	(AAT)15	45	10300602	10300646
chr12_1749	p2	(AC)10	20	10302460	10302479
chr12_1750	p2	(TG)14	28	10305695	10305722
chr12_1751	p2	(GT)6	12	10305866	10305877
chr12_1752	p2	(AG)6	12	10306557	10306568
chr12_1753	p2	(AC)9	18	10306828	10306845
chr12_1754	p2	(AG)7	14	10310716	10310729
chr12_1755	p2	(CA)8	16	10312108	10312123
chr12_1756	p2	(CA)21	42	10312265	10312306
chr12_1757	p3	(TTC)5	15	10319006	10319020
chr12_1758	p2	(TG)14	28	10332405	10332432
chr12_1759	p2	(CA)6	12	10350702	10350713
chr12_1760	p5	(TCTCG)5	25	10362716	10362740
chr12_1761	p2	(CA)19	38	10367208	10367245
chr12_1762	p2	(GT)7	14	10371101	10371114
chr12_1763	p2	(AG)6	12	10376166	10376177
chr12_1764	p2	(TG)9	18	10377631	10377648
chr12_1765	p2	(CA)15	30	10385560	10385589
chr12_1766	p3	(GCT)6	18	10387576	10387593
chr12_1767	p3	(AGC)5	15	10389650	10389664
chr12_1768	p2	(TG)10	20	10394542	10394561
chr12_1769	c	(AC)13(GC)7	40	10401171	10401210
chr12_1770	c	(GT)6a(TG)17	47	10402394	10402440
chr12_1771	p2	(GT)6	12	10403678	10403689
chr12_1772	p3	(TTG)5	15	10419071	10419085
chr12_1773	p5	(CCTCT)16	80	10419218	10419297
chr12_1774	p2	(AC)7	14	10420304	10420317
chr12_1775	p2	(GT)7	14	10421306	10421319
chr12_1776	p3	(ACA)5	15	10421830	10421844
chr12_1777	p2	(CA)16	32	10425594	10425625
chr12_1778	p4	(AAAC)7	28	10426867	10426894
chr12_1779	c	(GT)6gcgcgcgcgcac(GT)6	36	10431297	10431332
chr12_1780	p4	(ATAG)9	36	10434822	10434857
chr12_1781	p2	(TG)18	36	10438044	10438079

chr12_1782	p2	(TG)8	16	10441740	10441755
chr12_1783	p2	(CA)14	28	10443220	10443247
chr12_1784	p2	(GT)7	14	10444168	10444181
chr12_1785	p2	(GT)8	16	10444943	10444958
chr12_1786	p2	(AC)8	16	10447410	10447425
chr12_1787	p2	(AG)9	18	10449772	10449789
chr12_1788	p2	(AC)7	14	10450410	10450423
chr12_1789	p2	(GT)18	36	10450910	10450945
chr12_1790	p2	(AC)8	16	10455397	10455412
chr12_1791	p2	(AC)14	28	10458202	10458229
chr12_1792	c	(TG)27gggggggagtgagcgcgggggggct(TG)27	133	10464164	10464296
chr12_1793	c	agattgctattggatgctcatttcagacctgatcaatgatttgcctgagtttcag	188	10468870	10469057
chr12_1794	p2	(CA)18	36	10472521	10472556
chr12_1795	p3	(CTC)7	21	10478190	10478210
chr12_1796	p2	(CA)16	32	10478408	10478439
chr12_1797	p3	(TGA)7	21	10506280	10506300
chr12_1798	p2	(AG)9	18	10508846	10508863
chr12_1799	p2	(GT)9	18	10509422	10509439
chr12_1800	p2	(TG)7	14	10517904	10517917
chr12_1801	p2	(AC)7	14	10520263	10520276
chr12_1802	p2	(GT)7	14	10520901	10520914
chr12_1803	p4	(TCCA)6	24	10525659	10525682
chr12_1804	p5	(ACCTC)8	40	10530864	10530903
chr12_1805	c	acagacatacacaagaatgtaaacatgcaaagtgcatacactcctgctctta	115	10533248	10533362
chr12_1806	p2	(TG)11	22	10538403	10538424
chr12_1807	p2	(AC)6	12	10538564	10538575
chr12_1808	p2	(GT)7	14	10542491	10542504
chr12_1809	p2	(TG)6	12	10550530	10550541
chr12_1810	p2	(TG)19	38	10555774	10555811
chr12_1811	p2	(GT)10	20	10559235	10559254
chr12_1812	p4	(GGGA)5	20	10576412	10576431
chr12_1813	p2	(TG)8	16	10582905	10582920
chr12_1814	p2	(AC)9	18	10604394	10604411
chr12_1815	p2	(CA)12	24	10610320	10610343
chr12_1816	p2	(TA)15	30	10634461	10634490
chr12_1817	p3	(GCT)5	15	10636523	10636537
chr12_1818	p2	(CT)9	18	10665384	10665401
chr12_1819	p3	(AAT)5	15	10680027	10680041
chr12_1820	p2	(TG)6	12	10680878	10680889
chr12_1821	p2	(CT)8	16	10681700	10681715
chr12_1822	p4	(TCAA)7	28	10682845	10682872
chr12_1823	p2	(TA)8	16	10694700	10694715
chr12_1824	p2	(CA)7	14	10719589	10719602
chr12_1825	p2	(TC)15	30	10721976	10722005
chr12_1826	p2	(AC)11	22	10724781	10724802
chr12_1827	p5	(AAAAC)5	25	10726180	10726204
chr12_1828	c*	(GTGA)7(GA)26*	78	10745685	10745762
chr12_1829	p2	(AT)6	12	10747860	10747871
chr12_1830	c	(AAC)5agcaac(AAT)8	45	10749529	10749573
chr12_1831	p2	(CA)6	12	10749979	10749990
chr12_1832	p2	(AC)8	16	10762892	10762907
chr12_1833	c	gagagggaaagaaaatgtgtgtcagtgtgtgtaaatgcatacggagagcat	118	10773091	10773208

chr12_1834	p2	(CA)8	16	10773852	10773867
chr12_1835	p2	(AG)7	14	10777329	10777342
chr12_1836	p2	(AC)7	14	10781892	10781905
chr12_1837	p2	(TA)6	12	10785967	10785978
chr12_1838	p2	(CA)7	14	10789718	10789731
chr12_1839	c	(CA)7cg(CA)9	34	10795419	10795452
chr12_1840	p2	(TG)8	16	10795824	10795839
chr12_1841	p2	(CT)6	12	10798337	10798348
chr12_1842	p2	(AC)36	72	10801063	10801134
chr12_1843	p3	(TCA)7	21	10817182	10817202
chr12_1844	p2	(AC)7	14	10823148	10823161
chr12_1845	p2	(CA)14	28	10823531	10823558
chr12_1846	p2	(AC)7	14	10827178	10827191
chr12_1847	p2	(TC)8	16	10831124	10831139
chr12_1848	p2	(GT)13	26	10836177	10836202
chr12_1849	p2	(GT)11	22	10836648	10836669
chr12_1850	p3	(GAA)5	15	10851336	10851350
chr12_1851	p2	(GA)9	18	10856671	10856688
chr12_1852	p2	(CA)7	14	10856791	10856804
chr12_1853	p2	(TC)9	18	10861885	10861902
chr12_1854	p4	(TCCA)5	20	10862949	10862968
chr12_1855	p3	(CAG)5	15	10874705	10874719
chr12_1856	p2	(TG)10	20	10886033	10886052
chr12_1857	p2	(AC)8	16	10900055	10900070
chr12_1858	p4	(ATGG)7	28	10904726	10904753
chr12_1859	p2	(TG)8	16	10905913	10905928
chr12_1860	p2	(CA)14	28	10907550	10907577
chr12_1861	c	(GT)7(GA)7	28	10919280	10919307
chr12_1862	p2	(AC)6	12	10935837	10935848
chr12_1863	p2	(TG)6	12	10937232	10937243
chr12_1864	p4	(TATC)15	60	10949888	10949947
chr12_1865	p2	(AT)6	12	10950238	10950249
chr12_1866	p3	(CTA)5	15	10959951	10959965
chr12_1867	p2	(GT)6	12	10974953	10974964
chr12_1868	p2	(TG)6	12	10975241	10975252
chr12_1869	p2	(TG)7	14	10995765	10995778
chr12_1870	p2	(TC)6	12	11001803	11001814
chr12_1871	c	(GA)17a(AG)6	47	11010395	11010441
chr12_1872	p3	(AGC)5	15	11014119	11014133
chr12_1873	p4	(TAAA)5	20	11014545	11014564
chr12_1874	p2	(AC)22	44	11019543	11019586
chr12_1875	p2	(AC)6	12	11022293	11022304
chr12_1876	p2	(CA)6	12	11024134	11024145
chr12_1877	p2	(GT)16	32	11026501	11026532
chr12_1878	p2	(CA)13	26	11027284	11027309
chr12_1879	p2	(AC)10	20	11030766	11030785
chr12_1880	p3	(ACA)5	15	11033696	11033710
chr12_1881	p2	(AT)6	12	11043191	11043202
chr12_1882	p2	(GT)6	12	11046524	11046535
chr12_1883	p2	(AG)8	16	11053890	11053905
chr12_1884	p2	(TC)9	18	11060153	11060170
chr12_1885	p3	(ATC)5	15	11062173	11062187

chr12_1886	p3	(GAT)5	15	11065299	11065313
chr12_1887	p2	(AC)7	14	11066328	11066341
chr12_1888	p2	(GT)21	42	11081766	11081807
chr12_1889	p2	(GA)13	26	11122043	11122068
chr12_1890	p2	(GT)15	30	11146663	11146692
chr12_1891	p2	(GT)8	16	11148540	11148555
chr12_1892	p2	(TG)8	16	11150812	11150827
chr12_1893	p2	(TG)6	12	11152632	11152643
chr12_1894	p2	(CA)6	12	11160147	11160158
chr12_1895	p2	(TG)17	34	11163932	11163965
chr12_1896	c	(GAT)15catg(ATC)7	70	11167922	11167991
chr12_1897	p3	(GAG)5	15	11173279	11173293
chr12_1898	p2	(TC)7	14	11189213	11189226
chr12_1899	p3	(TTG)8	24	11192616	11192639
chr12_1900	p2	(GT)7	14	11194360	11194373
chr12_1901	c	(TG)7tatatatgtatgtatg(TA)10	50	11198471	11198520
chr12_1902	c	(TG)16tatgtatgtg(TA)8(TG)13tcacatcatattaa(AT)11	120	11201478	11201597
chr12_1903	p2	(GT)22	44	11204079	11204122
chr12_1904	p2	(GT)21	42	11205229	11205270
chr12_1905	p2	(GT)11	22	11209725	11209746
chr12_1906	p2	(CT)33	66	11209958	11210023
chr12_1907	p2	(GT)6	12	11211618	11211629
chr12_1908	p2	(GA)8	16	11218425	11218440
chr12_1909	c*	(GT)17atgtgta(TATG)7(TG)6*	79	11219387	11219465
chr12_1910	p2	(CT)9	18	11222954	11222971
chr12_1911	p2	(CT)9	18	11223398	11223415
chr12_1912	p2	(CT)12	24	11227627	11227650
chr12_1913	p2	(GT)9	18	11233768	11233785
chr12_1914	p2	(CT)6	12	11235792	11235803
chr12_1915	p2	(CA)16	32	11253852	11253883
chr12_1916	p5	(CAAAT)5	25	11274740	11274764
chr12_1917	c	(GT)6g(GT)30	73	11275679	11275751
chr12_1918	p2	(TG)6	12	11288474	11288485
chr12_1919	p2	(AC)15	30	11292193	11292222
chr12_1920	p2	(AT)6	12	11293725	11293736
chr12_1921	p3	(TAA)10	30	11305567	11305596
chr12_1922	c	(AG)6(TG)11	34	11306167	11306200
chr12_1923	p3	(TTC)5	15	11311027	11311041
chr12_1924	c	(GTAT)5gtgtg(TA)9(CA)6	55	11312150	11312204
chr12_1925	p3	(CTC)7	21	11316161	11316181
chr12_1926	p2	(GT)9	18	11323641	11323658
chr12_1927	p2	(AC)14	28	11329335	11329362
chr12_1928	p2	(GT)6	12	11348204	11348215
chr12_1929	c	(GT)6gc(GT)19	52	11363326	11363377
chr12_1930	p4	(GAGT)5	20	11367601	11367620
chr12_1931	p2	(TG)16	32	11369021	11369052
chr12_1932	p4	(ATGG)9	36	11369723	11369758
chr12_1933	p2	(AC)7	14	11374623	11374636
chr12_1934	p2	(CA)12	24	11380052	11380075
chr12_1935	p2	(GT)8	16	11396110	11396125
chr12_1936	p2	(CA)7	14	11396346	11396359
chr12_1937	p2	(AC)6	12	11403041	11403052

chr12_1938	p3	(ATT)5	15	11406544	11406558
chr12_1939	p2	(AG)7	14	11407859	11407872
chr12_1940	p2	(GT)6	12	11408270	11408281
chr12_1941	p3	(ATC)5	15	11408386	11408400
chr12_1942	p3	(TAA)6	18	11411145	11411162
chr12_1943	p2	(TG)9	18	11413779	11413796
chr12_1944	p4	(AATG)6	24	11416018	11416041
chr12_1945	p2	(TG)20	40	11423322	11423361
chr12_1946	p2	(AT)8	16	11425458	11425473
chr12_1947	p2	(GT)8	16	11466951	11466966
chr12_1948	p2	(GT)8	16	11473606	11473621
chr12_1949	p3	(GGA)8	24	11511018	11511041
chr12_1950	p2	(TG)6	12	11536816	11536827
chr12_1951	p2	(AC)7	14	11549985	11549998
chr12_1952	p4	(AATG)6	24	11574886	11574909
chr12_1953	p2	(TA)7	14	11577444	11577457
chr12_1954	p4	(ATCA)5	20	11581186	11581205
chr12_1955	p2	(GA)18	36	11582031	11582066
chr12_1956	p2	(GT)10	20	11594490	11594509
chr12_1957	p2	(CA)16	32	11598324	11598355
chr12_1958	p2	(TG)16	32	11609551	11609582
chr12_1959	p2	(AC)21	42	11609687	11609728
chr12_1960	p3	(GGA)5	15	11630364	11630378
chr12_1961	p2	(TG)13	26	11651205	11651230
chr12_1962	p2	(GT)16	32	11653254	11653285
chr12_1963	c	(AC)9agtggatttttcatttcacg(AC)15	68	11653413	11653480
chr12_1964	p2	(TC)10	20	11660403	11660422
chr12_1965	p2	(CA)12	24	11669444	11669467
chr12_1966	p2	(GT)6	12	11670373	11670384
chr12_1967	p2	(TG)8	16	11689419	11689434
chr12_1968	p2	(AG)14	28	11691957	11691984
chr12_1969	p2	(AG)14	28	11694348	11694375
chr12_1970	p3	(ATA)5	15	11697397	11697411
chr12_1971	p2	(CA)8	16	11702594	11702609
chr12_1972	p3	(ATC)5	15	11703424	11703438
chr12_1973	c	(AC)9aagccaatcgtgtgtgaactcgtg(CA)6	54	11705428	11705481
chr12_1974	p2	(AC)18	36	11707744	11707779
chr12_1975	p3	(GAT)5	15	11711228	11711242
chr12_1976	p2	(TC)6	12	11714965	11714976
chr12_1977	p2	(AT)7	14	11717394	11717407
chr12_1978	p3	(CTC)10	30	11718998	11719027
chr12_1979	p2	(CT)15	30	11719244	11719273
chr12_1980	p2	(CA)7	14	11723131	11723144
chr12_1981	p2	(AC)7	14	11730821	11730834
chr12_1982	p2	(GT)22	44	11751962	11752005
chr12_1983	p2	(CA)9	18	11752505	11752522
chr12_1984	p2	(AC)11	22	11758417	11758438
chr12_1985	p2	(AC)12	24	11766899	11766922
chr12_1986	p3	(TTG)5	15	11773830	11773844
chr12_1987	p3	(CTC)5	15	11779301	11779315
chr12_1988	p3	(TGT)7	21	11806497	11806517
chr12_1989	p2	(TG)7	14	11823242	11823255

chr12_1990	p3	(CTC)5	15	11837817	11837831
chr12_1991	p2	(AG)6	12	11841467	11841478
chr12_1992	p3	(CAT)7	21	11841824	11841844
chr12_1993	p3	(GCT)5	15	11844343	11844357
chr12_1994	p2	(TA)10	20	11849575	11849594
chr12_1995	p4	(TTCA)5	20	11884531	11884550
chr12_1996	p3	(CAT)7	21	11888295	11888315
chr12_1997	p3	(ATA)5	15	11899774	11899788
chr12_1998	p2	(GT)9	18	11916360	11916377
chr12_1999	p2	(CA)6	12	11942405	11942416
chr12_2000	p4	(CATT)6	24	11963252	11963275
chr12_2001	p4	(TAGC)6	24	11965637	11965660
chr12_2002	p2	(TG)6	12	11973246	11973257
chr12_2003	c	(GA)8c(AG)6	29	11996913	11996941
chr12_2004	p2	(AC)6	12	12040310	12040321
chr12_2005	p3	(AAG)5	15	12045594	12045608
chr12_2006	p3	(AAC)6	18	12050546	12050563
chr12_2007	p2	(GT)15	30	12051247	12051276
chr12_2008	p2	(CA)6	12	12070994	12071005
chr12_2009	p2	(AC)6	12	12071299	12071310
chr12_2010	p2	(GT)6	12	12084473	12084484
chr12_2011	c	(GAG)7gcggagaggagtta(GT)15	65	12088132	12088196
chr12_2012	p2	(AC)8	16	12092872	12092887
chr12_2013	p2	(TG)8	16	12093998	12094013
chr12_2014	p2	(TG)15	30	12094284	12094313
chr12_2015	p2	(GT)22	44	12103551	12103594
chr12_2016	p3	(TTG)5	15	12113948	12113962
chr12_2017	p3	(GGA)6	18	12114947	12114964
chr12_2018	p2	(CA)6	12	12122323	12122334
chr12_2019	c	gttgctttcatgggggacttgcattcatttcctccagacttaagtgcccacaat	136	12127036	12127171
chr12_2020	p2	(AC)6	12	12133728	12133739
chr12_2021	p2	(TA)13	26	12134011	12134036
chr12_2022	p2	(AT)6	12	12135464	12135475
chr12_2023	p2	(TG)13	26	12140307	12140332
chr12_2024	p3	(TTA)5	15	12168115	12168129
chr12_2025	p3	(TGG)5	15	12171951	12171965
chr12_2026	p2	(CA)6	12	12173728	12173739
chr12_2027	p3	(AGG)8	24	12176955	12176978
chr12_2028	p2	(GT)7	14	12179557	12179570
chr12_2029	p2	(AC)9	18	12193874	12193891
chr12_2030	p2	(TG)6	12	12194073	12194084
chr12_2031	c	(TATC)12tttctatct(ATCC)5	77	12195820	12195896
chr12_2032	p2	(TG)7	14	12196153	12196166
chr12_2033	p2	(GT)11	22	12198988	12199009
chr12_2034	p2	(CA)7	14	12199370	12199383
chr12_2035	p2	(AC)11	22	12201539	12201560
chr12_2036	p2	(AC)12	24	12202708	12202731
chr12_2037	c	16actgaatgcatacacatgctctcatatgaggccctctctgtctcacatg(	95	12209053	12209147
chr12_2038	p2	(CT)7	14	12211724	12211737
chr12_2039	p2	(CA)7	14	12213115	12213128
chr12_2040	p2	(GA)9	18	12213509	12213526
chr12_2041	p2	(CT)10	20	12215225	12215244

chr12_2042	p2	(TG)9	18	12228068	12228085
chr12_2043	p2	(AC)13	26	12230938	12230963
chr12_2044	p2	(TG)23	46	12240486	12240531
chr12_2045	p2	(GA)8	16	12240696	12240711
chr12_2046	p2	(AC)8	16	12241875	12241890
chr12_2047	p2	(AC)9	18	12256239	12256256
chr12_2048	p2	(GT)11	22	12256412	12256433
chr12_2049	p2	(CT)7	14	12256731	12256744
chr12_2050	p2	(AC)6	12	12258404	12258415
chr12_2051	p2	(CA)11	22	12267801	12267822
chr12_2052	p2	(TG)6	12	12270889	12270900
chr12_2053	p2	(GT)6	12	12273568	12273579
chr12_2054	p2	(TC)7	14	12273898	12273911
chr12_2055	p2	(TG)6	12	12282203	12282214
chr12_2056	p2	(TG)16	32	12283062	12283093
chr12_2057	p2	(GA)9	18	12284664	12284681
chr12_2058	p2	(TG)10	20	12286639	12286658
chr12_2059	p2	(CA)10	20	12286763	12286782
chr12_2060	p2	(AC)9	18	12290116	12290133
chr12_2061	p3	(CTA)5	15	12290688	12290702
chr12_2062	c	(TG)15ac(AG)8	48	12294522	12294569
chr12_2063	p2	(AC)9	18	12295782	12295799
chr12_2064	p2	(AC)10	20	12298314	12298333
chr12_2065	p2	(GT)9	18	12301437	12301454
chr12_2066	p2	(TG)9	18	12302015	12302032
chr12_2067	p2	(CA)6	12	12307098	12307109
chr12_2068	p3	(TTA)6	18	12307254	12307271
chr12_2069	p2	(TG)10	20	12315348	12315367
chr12_2070	p2	(GT)7	14	12316298	12316311
chr12_2071	p2	(GT)17	34	12318920	12318953
chr12_2072	p2	(TG)8	16	12319982	12319997
chr12_2073	p2	(TG)52	104	12320552	12320655
chr12_2074	p2	(AC)7	14	12323499	12323512
chr12_2075	p2	(GT)12	24	12330022	12330045
chr12_2076	p2	(AC)7	14	12343718	12343731
chr12_2077	p2	(AC)6	12	12349682	12349693
chr12_2078	p2	(GT)10	20	12349871	12349890
chr12_2079	p2	(CA)12	24	12350073	12350096
chr12_2080	p3	(TTG)5	15	12351568	12351582
chr12_2081	p2	(CA)6	12	12356906	12356917
chr12_2082	p2	(CA)23	46	12379436	12379481
chr12_2083	c	(CA)11ct(CA)12	48	12379771	12379818
chr12_2084	p2	(TG)18	36	12380076	12380111
chr12_2085	p2	(AC)8	16	12380301	12380316
chr12_2086	p2	(CA)8	16	12384220	12384235
chr12_2087	p2	(CA)9	18	12387096	12387113
chr12_2088	p2	(CA)6	12	12387445	12387456
chr12_2089	p3	(TAC)5	15	12393428	12393442
chr12_2090	c	(AC)16a(AG)18	69	12397467	12397535
chr12_2091	p2	(AC)8	16	12398333	12398348
chr12_2092	p2	(TG)9	18	12398777	12398794
chr12_2093	c	(GA)7caaagccacaagcgcg(CA)20	70	12400226	12400295

chr12_2094	p2	(TC)7	14	12401133	12401146
chr12_2095	p2	(CA)7	14	12403127	12403140
chr12_2096	p2	(AC)6	12	12406271	12406282
chr12_2097	p2	(CA)7	14	12410304	12410317
chr12_2098	p2	(CA)18	36	12412286	12412321
chr12_2099	p2	(CA)14	28	12413326	12413353
chr12_2100	p2	(CA)8	16	12416972	12416987
chr12_2101	p2	(CA)15	30	12419766	12419795
chr12_2102	p2	(CA)16	32	12420975	12421006
chr12_2103	p3	(TAT)5	15	12440046	12440060
chr12_2104	p2	(CT)6	12	12441398	12441409
chr12_2105	p2	(GA)6	12	12442988	12442999
chr12_2106	p2	(TG)9	18	12451084	12451101
chr12_2107	p2	(CA)9	18	12452620	12452637
chr12_2108	p2	(TG)10	20	12454540	12454559
chr12_2109	p2	(AC)18	36	12455567	12455602
chr12_2110	p2	(AC)8	16	12455847	12455862
chr12_2111	c	(TG)10agtgtgt(GA)16(GT)7	73	12459036	12459108
chr12_2112	p2	(GT)7	14	12460505	12460518
chr12_2113	p2	(AC)20	40	12461145	12461184
chr12_2114	p2	(TG)7	14	12466144	12466157
chr12_2115	c	(TG)10tttgt(GA)6	37	12466396	12466432
chr12_2116	p2	(CT)7	14	12466616	12466629
chr12_2117	p2	(AT)10	20	12472400	12472419
chr12_2118	p2	(AC)9	18	12476323	12476340
chr12_2119	c	(TC)12acacact(CA)11	53	12484946	12484998
chr12_2120	p2	(CA)10	20	12486382	12486401
chr12_2121	p2	(AC)9	18	12490072	12490089
chr12_2122	p2	(CA)9	18	12492146	12492163
chr12_2123	p2	(TG)6	12	12496609	12496620
chr12_2124	p2	(TG)9	18	12496723	12496740
chr12_2125	p3	(AAT)5	15	12507222	12507236
chr12_2126	p2	(AC)16	32	12510589	12510620
chr12_2127	p4	(ATCC)5	20	12517379	12517398
chr12_2128	p2	(TG)6	12	12519238	12519249
chr12_2129	p2	(TG)13	26	12519705	12519730
chr12_2130	p2	(CA)6	12	12523627	12523638
chr12_2131	p2	(TA)9	18	12524501	12524518
chr12_2132	p2	(TG)23	46	12542241	12542286
chr12_2133	p2	(TG)6	12	12542954	12542965
chr12_2134	p2	(CA)7	14	12547221	12547234
chr12_2135	c	ctctctaaaggttcaaacaacacaccttgcctgtcttttcagtgaagagaaaaca	130	12548345	12548474
chr12_2136	p2	(GA)11	22	12552139	12552160
chr12_2137	p2	(AC)16	32	12555115	12555146
chr12_2138	p2	(CA)8	16	12558497	12558512
chr12_2139	p2	(GT)8	16	12562660	12562675
chr12_2140	p2	(AC)8	16	12567827	12567842
chr12_2141	p2	(TC)7	14	12571898	12571911
chr12_2142	p2	(GT)12	24	12574390	12574413
chr12_2143	p2	(TG)10	20	12578367	12578386
chr12_2144	p2	(TG)15	30	12579691	12579720
chr12_2145	c	(TG)15(CG)6	42	12580331	12580372

chr12_2146	p2	(AT)6	12	12583005	12583016
chr12_2147	p2	(AC)22	44	12584611	12584654
chr12_2148	p2	(AC)10	20	12591333	12591352
chr12_2149	p2	(GT)11	22	12600065	12600086
chr12_2150	p2	(TC)7	14	12601026	12601039
chr12_2151	p2	(AC)7	14	12603683	12603696
chr12_2152	p2	(CA)7	14	12605946	12605959
chr12_2153	c	AC)11agaatagataaccctgactttaaagcctacagcatttctgct(TG)	78	12612040	12612117
chr12_2154	p3	(AAT)6	18	12619610	12619627
chr12_2155	c	(CT)8cat(AC)20	59	12625104	12625162
chr12_2156	p2	(CA)6	12	12625361	12625372
chr12_2157	p2	(GT)16	32	12637605	12637636
chr12_2158	p2	(GT)15	30	12639968	12639997
chr12_2159	p2	(GT)6	12	12642323	12642334
chr12_2160	p3	(TCA)5	15	12642666	12642680
chr12_2161	p2	(GA)6	12	12642792	12642803
chr12_2162	p2	(TG)6	12	12643578	12643589
chr12_2163	p2	(GT)12	24	12645967	12645990
chr12_2164	p2	(CT)7	14	12648876	12648889
chr12_2165	p2	(GA)6	12	12653002	12653013
chr12_2166	p2	(GT)17	34	12656758	12656791
chr12_2167	p2	(AG)7	14	12657011	12657024
chr12_2168	p2	(CA)6	12	12658630	12658641
chr12_2169	p2	(AC)10	20	12661151	12661170
chr12_2170	p2	(CA)6	12	12661308	12661319
chr12_2171	p2	(AG)6	12	12661570	12661581
chr12_2172	p2	(TC)9	18	12665363	12665380
chr12_2173	p2	(AT)6	12	12666007	12666018
chr12_2174	p3	(TAT)6	18	12666517	12666534
chr12_2175	p2	(TC)9	18	12670293	12670310
chr12_2176	p2	(AC)7	14	12671934	12671947
chr12_2177	p2	(GA)6	12	12674251	12674262
chr12_2178	p2	(CA)8	16	12679443	12679458
chr12_2179	p2	(TG)8	16	12679873	12679888
chr12_2180	p2	(CA)10	20	12680789	12680808
chr12_2181	p2	(AC)7	14	12681643	12681656
chr12_2182	p2	(TG)9	18	12683333	12683350
chr12_2183	p2	(AT)6	12	12702449	12702460
chr12_2184	p2	(TG)16	32	12728983	12729014
chr12_2185	p2	(AC)7	14	12729545	12729558
chr12_2186	p2	(TC)8	16	12732396	12732411
chr12_2187	p2	(TG)8	16	12732538	12732553
chr12_2188	p2	(TA)6	12	12737516	12737527
chr12_2189	p2	(AC)13	26	12742369	12742394
chr12_2190	p3	(GAG)5	15	12747090	12747104
chr12_2191	p4	(GATT)7	28	12747606	12747633
chr12_2192	p2	(AC)9	18	12750924	12750941
chr12_2193	p2	(AG)6	12	12761980	12761991
chr12_2194	p2	(GT)9	18	12762283	12762300
chr12_2195	p3	(CAA)8	24	12762905	12762928
chr12_2196	c	(GT)11(GA)7	36	12777773	12777808
chr12_2197	p2	(AC)6	12	12779413	12779424

chr12_2198	p2	(CT)19	38	12786810	12786847
chr12_2199	p2	(GT)17	34	12787754	12787787
chr12_2200	p2	(GT)11	22	12794206	12794227
chr12_2201	p3	(CAA)5	15	12800352	12800366
chr12_2202	p2	(TC)10	20	12801982	12802001
chr12_2203	c	(TG)6tttatgtatttgtgtgtgttggga(GT)6atgtgtg(TGC)5	70	12802115	12802184
chr12_2204	p2	(TG)7	14	12802460	12802473
chr12_2205	p2	(TG)9	18	12802635	12802652
chr12_2206	p2	(TG)9	18	12805096	12805113
chr12_2207	c	(TG)7tatgggga(TGG)5	37	12805511	12805547
chr12_2208	p3	(TGG)5	15	12806002	12806016
chr12_2209	p2	(CA)15	30	12807891	12807920
chr12_2210	p2	(TG)14	28	12812847	12812874
chr12_2211	p2	(AC)17	34	12814673	12814706
chr12_2212	p2	(TG)8	16	12817954	12817969
chr12_2213	p2	(CT)7	14	12823942	12823955
chr12_2214	p2	(AC)8	16	12824675	12824690
chr12_2215	p2	(CA)7	14	12827070	12827083
chr12_2216	p2	(AG)10	20	12851976	12851995
chr12_2217	p2	(GA)10	20	12855360	12855379
chr12_2218	p2	(AC)11	22	12856798	12856819
chr12_2219	p3	(CCA)5	15	12860523	12860537
chr12_2220	p3	(AAT)5	15	12865090	12865104
chr12_2221	p4	(TAAA)6	24	12866883	12866906
chr12_2222	p2	(AC)10	20	12878118	12878137
chr12_2223	p3	(ATA)7	21	12878336	12878356
chr12_2224	p2	(AG)9	18	12879437	12879454
chr12_2225	p2	(AC)7	14	12886963	12886976
chr12_2226	p3	(TCT)7	21	12889419	12889439
chr12_2227	p2	(CA)10	20	12892860	12892879
chr12_2228	p2	(TG)7	14	12899055	12899068
chr12_2229	p2	(GT)25	50	12899351	12899400
chr12_2230	p2	(TG)8	16	12902722	12902737
chr12_2231	p3	(CTG)5	15	12902842	12902856
chr12_2232	p2	(TG)12	24	12904520	12904543
chr12_2233	p2	(AC)6	12	12909016	12909027
chr12_2234	p2	(GT)20	40	12912147	12912186
chr12_2235	p2	(AC)29	58	12914064	12914121
chr12_2236	p2	(GA)12	24	12915559	12915582
chr12_2237	p2	(AC)16	32	12920904	12920935
chr12_2238	p3	(AAC)6	18	12924679	12924696
chr12_2239	p2	(AC)8	16	12930671	12930686
chr12_2240	p2	(CA)9	18	12943540	12943557
chr12_2241	p2	(TC)10	20	12946661	12946680
chr12_2242	p2	(TG)6	12	12948764	12948775
chr12_2243	p2	(AC)6	12	12951797	12951808
chr12_2244	p2	(GT)8	16	12953867	12953882
chr12_2245	p4	(TTTA)6	24	12961182	12961205
chr12_2246	p2	(GT)12	24	12964502	12964525
chr12_2247	p2	(AC)7	14	12964646	12964659
chr12_2248	p2	(CA)11	22	12965700	12965721
chr12_2249	p5	(ACACC)7	35	12966191	12966225

chr12_2250	p3	(AAC)5	15	12969338	12969352
chr12_2251	p3	(AAT)5	15	12973386	12973400
chr12_2252	p2	(AC)6	12	12981140	12981151
chr12_2253	p2	(GT)8	16	12987088	12987103
chr12_2254	p2	(AC)7	14	12993754	12993767
chr12_2255	p2	(GT)17	34	12999154	12999187
chr12_2256	p2	(AC)13	26	13001019	13001044
chr12_2257	c	(CT)10(CA)17	54	13001394	13001447
chr12_2258	p2	(TG)15	30	13020890	13020919
chr12_2259	c	tgcatcagatcttcaactgtttctctttctgttgctcacaaacacacacactccc	126	13022715	13022840
chr12_2260	p2	(TG)7	14	13024965	13024978
chr12_2261	p2	(AG)11	22	13025122	13025143
chr12_2262	p2	(CT)15	30	13030817	13030846
chr12_2263	p2	(TG)10	20	13030998	13031017
chr12_2264	p2	(AC)13	26	13031215	13031240
chr12_2265	p2	(CA)9	18	13031392	13031409
chr12_2266	p2	(GT)7	14	13033733	13033746
chr12_2267	p2	(CA)7	14	13034290	13034303
chr12_2268	p2	(GT)6	12	13034415	13034426
chr12_2269	c	(CA)6cctttcctcactccttcacctttaacaaacagtatgactg(GT)21	94	13034548	13034641
chr12_2270	p2	(AC)6	12	13034826	13034837
chr12_2271	p2	(CA)9	18	13035166	13035183
chr12_2272	p2	(TG)6	12	13036461	13036472
chr12_2273	p2	(AG)6	12	13036862	13036873
chr12_2274	p3	(CCT)9	27	13037459	13037485
chr12_2275	p2	(TC)10	20	13037752	13037771
chr12_2276	p2	(GA)13	26	13038709	13038734
chr12_2277	p2	(TC)6	12	13041915	13041926
chr12_2278	p2	(TG)15	30	13043514	13043543
chr12_2279	c	(CA)6t(AC)6	25	13043697	13043721
chr12_2280	p2	(TG)8	16	13044792	13044807
chr12_2281	p3	(TTA)8	24	13049581	13049604
chr12_2282	c	(ATA)7acaataataataatg(ATA)6	54	13049739	13049792
chr12_2283	c	(AAC)5tgaacataccagctctggccgcagtggcta(TAC)6	63	13050015	13050077
chr12_2284	p2	(TG)12	24	13052555	13052578
chr12_2285	p2	(AC)6	12	13052808	13052819
chr12_2286	p2	(TG)7	14	13060318	13060331
chr12_2287	p2	(TA)8	16	13062249	13062264
chr12_2288	p2	(AC)10	20	13066144	13066163
chr12_2289	c	(TC)6cctg(TC)6	28	13075120	13075147
chr12_2290	p2	(GT)7	14	13083926	13083939
chr12_2291	p2	(CA)9	18	13094957	13094974
chr12_2292	p2	(CA)16	32	13095242	13095273
chr12_2293	p2	(TG)14	28	13095658	13095685
chr12_2294	p2	(CA)8	16	13100278	13100293
chr12_2295	p3	(GCT)5	15	13103319	13103333
chr12_2296	p2	(GT)18	36	13103732	13103767
chr12_2297	p2	(CT)7	14	13106098	13106111
chr12_2298	p4	(TTTA)5	20	13115607	13115626
chr12_2299	p4	(CCTA)5	20	13120119	13120138
chr12_2300	p3	(CTT)5	15	13120857	13120871
chr12_2301	p2	(TC)9	18	13122933	13122950

chr12_2302	p2	(GT)8	16	13125437	13125452
chr12_2303	p2	(TG)12	24	13127580	13127603
chr12_2304	p2	(GT)7	14	13139637	13139650
chr12_2305	p5	(AAGAG)5	25	13148046	13148070
chr12_2306	p4	(GATA)6	24	13150099	13150122
chr12_2307	p3	(AAT)5	15	13152773	13152787
chr12_2308	c	(TG)6agtgagaa(AG)9	38	13156555	13156592
chr12_2309	p2	(GT)6	12	13164118	13164129
chr12_2310	p2	(CA)27	54	13171078	13171131
chr12_2311	p2	(AC)6	12	13180422	13180433
chr12_2312	p2	(TG)6	12	13180843	13180854
chr12_2313	p2	(CA)15	30	13181915	13181944
chr12_2314	p2	(AC)6	12	13182763	13182774
chr12_2315	p2	(AC)7	14	13187063	13187076
chr12_2316	p2	(CA)19	38	13192768	13192805
chr12_2317	p2	(CA)8	16	13192966	13192981
chr12_2318	p2	(AC)6	12	13193298	13193309
chr12_2319	p2	(GT)9	18	13205904	13205921
chr12_2320	p5	(CTCTC)5	25	13217388	13217412
chr12_2321	p2	(TG)7	14	13219418	13219431
chr12_2322	p2	(CA)9	18	13220366	13220383
chr12_2323	p2	(TG)15	30	13223672	13223701
chr12_2324	c	ttcaaaggcatgaatgcagatttttctcttgcatgcatcttcatgtgtgtattca[illegible]	102	13229787	13229888
chr12_2325	p2	(CA)7	14	13230590	13230603
chr12_2326	p2	(TG)14	28	13233845	13233872
chr12_2327	c	ttcaaaggcatgaatgcagatttttctcttgcatgcatcttcatgtgtgtattca[illegible]	102	13239784	13239885
chr12_2328	p2	(CA)7	14	13240587	13240600
chr12_2329	p2	(TG)22	44	13251541	13251584
chr12_2330	c	(AG)6tggggataa(TGG)5	36	13251974	13252009
chr12_2331	p2	(CA)6	12	13252959	13252970
chr12_2332	p3	(AAG)5	15	13254061	13254075
chr12_2333	p2	(TG)24	48	13256330	13256377
chr12_2334	p2	(TG)24	48	13277095	13277142
chr12_2335	p2	(GT)10	20	13287180	13287199
chr12_2336	p2	(TC)6	12	13296252	13296263
chr12_2337	p2	(CA)18	36	13297214	13297249
chr12_2338	p2	(GT)8	16	13298807	13298822
chr12_2339	p2	(GT)10	20	13299580	13299599
chr12_2340	p2	(AC)8	16	13303087	13303102
chr12_2341	p2	(TG)10	20	13313307	13313326
chr12_2342	c	(CT)19catatg(CA)7	58	13317353	13317410
chr12_2343	p2	(GT)7	14	13328402	13328415
chr12_2344	p3	(GGA)5	15	13328620	13328634
chr12_2345	p2	(AC)8	16	13329134	13329149
chr12_2346	p2	(AC)6	12	13330720	13330731
chr12_2347	p2	(TC)19	38	13332092	13332129
chr12_2348	p2	(AC)9	18	13332377	13332394
chr12_2349	p2	(AC)11	22	13333739	13333760
chr12_2350	p2	(GT)11	22	13333964	13333985
chr12_2351	p2	(TG)11	22	13336483	13336504
chr12_2352	p2	(TG)12	24	13342647	13342670
chr12_2353	p2	(TG)19	38	13342806	13342843

chr12_2354	p2	(TG)8	16	13343684	13343699
chr12_2355	c	(GT)7ttgagccctgtgtgtgttcaaga(GT)7	51	13344704	13344754
chr12_2356	p2	(GA)6	12	13347100	13347111
chr12_2357	p4	(TTTC)6	24	13357495	13357518
chr12_2358	p2	(AC)23	46	13358102	13358147
chr12_2359	p3	(TTA)5	15	13373300	13373314
chr12_2360	p2	(GT)6	12	13387409	13387420
chr12_2361	p5	(CTAAG)6	30	13388454	13388483
chr12_2362	p2	(CT)28	56	13401009	13401064
chr12_2363	p2	(TG)6	12	13430320	13430331
chr12_2364	p3	(TAA)5	15	13431180	13431194
chr12_2365	p2	(TG)12	24	13434406	13434429
chr12_2366	p2	(AT)7	14	13453596	13453609
chr12_2367	p2	(GT)13	26	13459245	13459270
chr12_2368	p3	(TTA)6	18	13462027	13462044
chr12_2369	p2	(GT)6	12	13485322	13485333
chr12_2370	p2	(TG)6	12	13495356	13495367
chr12_2371	p3	(CTC)5	15	13497304	13497318
chr12_2372	p3	(AGA)5	15	13501831	13501845
chr12_2373	p3	(GGA)5	15	13502513	13502527
chr12_2374	p2	(TG)10	20	13503251	13503270
chr12_2375	p2	(CA)21	42	13508517	13508558
chr12_2376	p3	(ATA)8	24	13511305	13511328
chr12_2377	p3	(TCA)5	15	13511555	13511569
chr12_2378	p2	(AC)8	16	13525424	13525439
chr12_2379	p3	(GAG)7	21	13527412	13527432
chr12_2380	p3	(GGA)5	15	13527564	13527578
chr12_2381	p4	(TTTA)5	20	13533385	13533404
chr12_2382	p4	(ATTG)5	20	13540423	13540442
chr12_2383	p2	(GT)6	12	13553903	13553914
chr12_2384	p3	(CTA)6	18	13554166	13554183
chr12_2385	c	(CT)8(CA)19	54	13559685	13559738
chr12_2386	p6	(ATATAC)10	60	13565005	13565064
chr12_2387	p2	(TC)6	12	13605312	13605323
chr12_2388	p2	(AC)9	18	13612086	13612103
chr12_2389	p2	(GT)6	12	13613399	13613410
chr12_2390	p2	(TG)7	14	13615565	13615578
chr12_2391	p3	(AAC)5	15	13620363	13620377
chr12_2392	p2	(AT)7	14	13622515	13622528
chr12_2393	p2	(AT)7	14	13623355	13623368
chr12_2394	p2	(AT)7	14	13624782	13624795
chr12_2395	c	(GT)7gggtgcgc(GT)8	38	13634870	13634907
chr12_2396	p3	(ATA)5	15	13639224	13639238
chr12_2397	p2	(AC)7	14	13645611	13645624
chr12_2398	p2	(AC)11	22	13653015	13653036
chr12_2399	c	(GAG)5gtggagctcccataccaggtaag(GGA)5	53	13654745	13654797
chr12_2400	p2	(AC)9	18	13663205	13663222
chr12_2401	p5	(CTAAG)5	25	13664629	13664653
chr12_2402	p2	(AC)8	16	13665685	13665700
chr12_2403	p3	(CCT)6	18	13672923	13672940
chr12_2404	p2	(TG)9	18	13674704	13674721
chr12_2405	p5	(ATTCT)5	25	13676026	13676050

chr12_2406	p3	(GAG)6	18	13691848	13691865
chr12_2407	p2	(CA)11	22	13695931	13695952
chr12_2408	p2	(AC)16	32	13697024	13697055
chr12_2409	p3	(GTC)5	15	13704887	13704901
chr12_2410	p2	(AC)14	28	13714104	13714131
chr12_2411	c	(CTA)5gtagtac(TAG)5	37	13715695	13715731
chr12_2412	p2	(GT)6	12	13724711	13724722
chr12_2413	p2	(AC)14	28	13729905	13729932
chr12_2414	p3	(CTC)5	15	13732653	13732667
chr12_2415	p2	(GA)7	14	13732843	13732856
chr12_2416	p2	(CA)11	22	13733503	13733524
chr12_2417	p2	(TG)8	16	13734859	13734874
chr12_2418	p2	(AC)9	18	13737505	13737522
chr12_2419	p2	(AC)6	12	13737734	13737745
chr12_2420	p3	(TTC)6	18	13737985	13738002
chr12_2421	p2	(CA)12	24	13741312	13741335
chr12_2422	p2	(CA)14	28	13749984	13750011
chr12_2423	p2	(AC)6	12	13761682	13761693
chr12_2424	p3	(CAT)8	24	13774027	13774050
chr12_2425	p3	(AAG)5	15	13778666	13778680
chr12_2426	p2	(GT)9	18	13785023	13785040
chr12_2427	c	(CA)12gctataac(TA)7	46	13785158	13785203
chr12_2428	p2	(GA)7	14	13788344	13788357
chr12_2429	p2	(AC)11	22	13790180	13790201
chr12_2430	p2	(AC)15	30	13816073	13816102
chr12_2431	p2	(CA)10	20	13819610	13819629
chr12_2432	p2	(TG)9	18	13831410	13831427
chr12_2433	p3	(TAA)5	15	13838439	13838453
chr12_2434	p2	(GT)10	20	13851709	13851728
chr12_2435	c	(AC)7g(CA)22	59	13853330	13853388
chr12_2436	p2	(AC)6	12	13854028	13854039
chr12_2437	p2	(AC)10	20	13854748	13854767
chr12_2438	p2	(AC)9	18	13855207	13855224
chr12_2439	p2	(AC)7	14	13863754	13863767
chr12_2440	p3	(AAC)6	18	13863976	13863993
chr12_2441	p2	(AG)6	12	13879881	13879892
chr12_2442	p2	(GT)11	22	13880666	13880687
chr12_2443	p3	(ATA)7	21	13884112	13884132
chr12_2444	p2	(AC)7	14	13887272	13887285
chr12_2445	p2	(CA)13	26	13888831	13888856
chr12_2446	p2	(CA)13	26	13889342	13889367
chr12_2447	p2	(AG)6	12	13906348	13906359
chr12_2448	p2	(TG)13	26	13906987	13907012
chr12_2449	p3	(AGG)7	21	13909131	13909151
chr12_2450	p3	(ATA)5	15	13911146	13911160
chr12_2451	p2	(AC)10	20	13916821	13916840
chr12_2452	p2	(CA)6	12	13919303	13919314
chr12_2453	p5	(GAGGA)5	25	13927146	13927170
chr12_2454	p2	(CA)6	12	13928482	13928493
chr12_2455	p3	(TCA)5	15	13929897	13929911
chr12_2456	p2	(TG)15	30	13931059	13931088
chr12_2457	p3	(TAT)10	30	13933271	13933300

chr12_2458	c	(CCT)6(CAT)5	33	13939359	13939391
chr12_2459	p4	(TTTA)5	20	13940643	13940662
chr12_2460	p2	(AG)6	12	13941007	13941018
chr12_2461	p2	(GT)7	14	13941817	13941830
chr12_2462	p2	(TG)6	12	13945114	13945125
chr12_2463	p3	(TGA)8	24	13958636	13958659
chr12_2464	p3	(CTC)5	15	13962544	13962558
chr12_2465	p2	(TG)6	12	13964116	13964127
chr12_2466	p3	(CTC)5	15	13965273	13965287
chr12_2467	p3	(TCC)6	18	13969582	13969599
chr12_2468	p2	(AC)6	12	13969706	13969717
chr12_2469	p2	(GT)11	22	13975808	13975829
chr12_2470	p2	(TG)9	18	13979095	13979112
chr12_2471	p4	(CAAA)5	20	13979642	13979661
chr12_2472	p2	(TG)17	34	13987489	13987522
chr12_2473	p2	(AC)7	14	13997887	13997900
chr12_2474	p2	(TG)12	24	14000025	14000048
chr12_2475	p2	(CA)25	50	14004641	14004690
chr12_2476	p2	(TG)9	18	14010949	14010966
chr12_2477	p2	(GT)6	12	14012589	14012600
chr12_2478	p2	(GT)6	12	14024158	14024169
chr12_2479	p2	(AC)11	22	14025126	14025147
chr12_2480	p2	(TG)9	18	14027039	14027056
chr12_2481	c	[illegible]acaggctgtgatggcaaaacatttatcaccaaccacaaaacaaaccacag	98	14028786	14028883
chr12_2482	p3	(TCC)5	15	14030040	14030054
chr12_2483	p2	(GT)26	52	14032265	14032316
chr12_2484	c	(GT)10(GA)7	34	14047570	14047603
chr12_2485	p2	(CA)10	20	14047987	14048006
chr12_2486	p3	(TGC)5	15	14051760	14051774
chr12_2487	p2	(TA)17	34	14055091	14055124
chr12_2488	p2	(AT)6	12	14055278	14055289
chr12_2489	p2	(TG)6	12	14067054	14067065
chr12_2490	p2	(AC)6	12	14067220	14067231
chr12_2491	p2	(GT)17	34	14068240	14068273
chr12_2492	p2	(GT)6	12	14069602	14069613
chr12_2493	p2	(GT)10	20	14078656	14078675
chr12_2494	p3	(TGA)5	15	14082453	14082467
chr12_2495	p3	(TAA)5	15	14083555	14083569
chr12_2496	p3	(AGC)5	15	14090287	14090301
chr12_2497	p2	(AG)10	20	14097492	14097511
chr12_2498	p2	(GT)9	18	14098379	14098396
chr12_2499	p2	(CA)9	18	14098557	14098574
chr12_2500	p2	(GT)25	50	14108128	14108177
chr12_2501	p2	(GT)6	12	14111524	14111535
chr12_2502	p2	(GT)7	14	14112636	14112649
chr12_2503	p2	(AC)10	20	14113219	14113238
chr12_2504	p2	(CA)8	16	14116440	14116455
chr12_2505	p2	(TC)10	20	14126915	14126934
chr12_2506	p2	(CT)6	12	14127045	14127056
chr12_2507	p2	(CA)17	34	14127555	14127588
chr12_2508	p2	(CA)12	24	14130555	14130578
chr12_2509	p2	(AC)18	36	14133899	14133934

chr12_2510	p2	(CA)17	34	14160419	14160452
chr12_2511	p3	(GGA)5	15	14163298	14163312
chr12_2512	p2	(CT)6	12	14169365	14169376
chr12_2513	p3	(GGA)6	18	14169514	14169531
chr12_2514	p2	(TG)21	42	14172767	14172808
chr12_2515	p2	(CT)15	30	14177529	14177558
chr12_2516	c	(TGTC)5cacatttcattgagttatggttc(TGTT)5	63	14180574	14180636
chr12_2517	p2	(GT)9	18	14182998	14183015
chr12_2518	p3	(TGA)5	15	14184206	14184220
chr12_2519	c	(GT)6g(GT)30	73	14186432	14186504
chr12_2520	p2	(CA)6	12	14194933	14194944
chr12_2521	p2	(AC)7	14	14205869	14205882
chr12_2522	p2	(AC)10	20	14208500	14208519
chr12_2523	p3	(TCT)8	24	14222030	14222053
chr12_2524	p2	(TG)15	30	14222375	14222404
chr12_2525	p4	(ATGG)5	20	14230114	14230133
chr12_2526	p2	(TG)6	12	14240534	14240545
chr12_2527	p2	(AC)7	14	14244189	14244202
chr12_2528	p4	(CATT)5	20	14258072	14258091
chr12_2529	p2	(TG)8	16	14259120	14259135
chr12_2530	p2	(TG)6	12	14261642	14261653
chr12_2531	p2	(TA)6	12	14262353	14262364
chr12_2532	p2	(CA)22	44	14263297	14263340
chr12_2533	p2	(TG)9	18	14277320	14277337
chr12_2534	p3	(CCT)6	18	14282724	14282741
chr12_2535	p2	(AC)6	12	14291771	14291782
chr12_2536	p2	(AC)6	12	14296863	14296874
chr12_2537	p2	(CA)13	26	14300124	14300149
chr12_2538	p3	(CTT)5	15	14300539	14300553
chr12_2539	p2	(CA)6	12	14321430	14321441
chr12_2540	p2	(CA)8	16	14328431	14328446
chr12_2541	p2	(CA)12	24	14334627	14334650
chr12_2542	p2	(CA)10	20	14334780	14334799
chr12_2543	p2	(AC)15	30	14368230	14368259
chr12_2544	c	(CT)6cc(CT)8	30	14368576	14368605
chr12_2545	p2	(GA)6	12	14369802	14369813
chr12_2546	p2	(GT)6	12	14370129	14370140
chr12_2547	p2	(CA)9	18	14375168	14375185
chr12_2548	c	(GC)7(AC)10	34	14376150	14376183
chr12_2549	p3	(ATG)6	18	14377553	14377570
chr12_2550	p2	(TG)6	12	14378126	14378137
chr12_2551	p2	(CA)12	24	14381459	14381482
chr12_2552	p3	(TAT)5	15	14382266	14382280
chr12_2553	p2	(GT)6	12	14382822	14382833
chr12_2554	p2	(AG)6	12	14384965	14384976
chr12_2555	p2	(AC)6	12	14386552	14386563
chr12_2556	c	(TG)10(GT)7	34	14387215	14387248
chr12_2557	p2	(TG)10	20	14406190	14406209
chr12_2558	p3	(GAG)6	18	14417505	14417522
chr12_2559	p3	(ATG)5	15	14418631	14418645
chr12_2560	p3	(GGA)6	18	14423823	14423840
chr12_2561	p2	(TG)6	12	14424924	14424935

chr12_2562	p3	(TGA)5	15	14442754	14442768
chr12_2563	p4	(CCTA)5	20	14450104	14450123
chr12_2564	p6	(TAAATG)5	30	14452360	14452389
chr12_2565	p4	(GATG)5	20	14456550	14456569
chr12_2566	p3	(AGG)5	15	14470763	14470777
chr12_2567	p2	(AG)6	12	14476420	14476431
chr12_2568	p2	(AT)14	28	14486113	14486140
chr12_2569	p2	(CA)13	26	14487061	14487086
chr12_2570	p2	(CA)7	14	14488437	14488450
chr12_2571	p4	(TTAC)5	20	14490841	14490860
chr12_2572	p2	(TG)9	18	14492252	14492269
chr12_2573	p2	(AC)6	12	14496611	14496622
chr12_2574	p3	(GAT)5	15	14499490	14499504
chr12_2575	p2	(CA)17	34	14500064	14500097
chr12_2576	p2	(CA)13	26	14502910	14502935
chr12_2577	p2	(GT)13	26	14510609	14510634
chr12_2578	p3	(ATC)6	18	14511587	14511604
chr12_2579	p3	(GGA)5	15	14522472	14522486
chr12_2580	p2	(AT)6	12	14524929	14524940
chr12_2581	p2	(CA)21	42	14531847	14531888
chr12_2582	p2	(AC)18	36	14533261	14533296
chr12_2583	p2	(CA)10	20	14535071	14535090
chr12_2584	p4	(GTCT)5	20	14540355	14540374
chr12_2585	p3	(GGA)5	15	14546161	14546175
chr12_2586	p4	(TGAT)5	20	14546566	14546585
chr12_2587	p3	(ATT)8	24	14550403	14550426
chr12_2588	p2	(AC)6	12	14550536	14550547
chr12_2589	p2	(CA)6	12	14558338	14558349
chr12_2590	p2	(CA)6	12	14560324	14560335
chr12_2591	p5	(TACTC)5	25	14562153	14562177
chr12_2592	p2	(GA)8	16	14562863	14562878
chr12_2593	c	gagagaggagacacaaagttagtgatgtgtaatggtggcatatagaagca	110	14572851	14572960
chr12_2594	p4	(AATC)6	24	14584231	14584254
chr12_2595	p2	(GT)6	12	14590773	14590784
chr12_2596	c	(TC)7gcatg(CA)13	45	14617234	14617278
chr12_2597	p2	(CT)14	28	14619003	14619030
chr12_2598	p3	(GGA)5	15	14622151	14622165
chr12_2599	c	(AC)11(TC)11	44	14629867	14629910
chr12_2600	p3	(TCC)6	18	14630276	14630293
chr12_2601	p3	(CCT)6	18	14633891	14633908
chr12_2602	p3	(TCC)6	18	14635590	14635607
chr12_2603	p3	(AGA)6	18	14644472	14644489
chr12_2604	p4	(AAAT)5	20	14647332	14647351
chr12_2605	p2	(TG)7	14	14654655	14654668
chr12_2606	p2	(TG)13	26	14655851	14655876
chr12_2607	p2	(GT)13	26	14659584	14659609
chr12_2608	p2	(TA)6	12	14664342	14664353
chr12_2609	p2	(GT)25	50	14666198	14666247
chr12_2610	p3	(ATA)5	15	14667680	14667694
chr12_2611	p2	(GT)35	70	14668570	14668639
chr12_2612	p4	(ATGG)5	20	14673003	14673022
chr12_2613	p2	(CA)10	20	14679636	14679655

chr12_2614	p3	(GAG)6	18	14684117	14684134
chr12_2615	p2	(GT)11	22	14685092	14685113
chr12_2616	p2	(CA)20	40	14685262	14685301
chr12_2617	p2	(GT)12	24	14685542	14685565
chr12_2618	p2	(TG)19	38	14686327	14686364
chr12_2619	p3	(GAG)7	21	14691746	14691766
chr12_2620	p3	(AGG)5	15	14692362	14692376
chr12_2621	p2	(CA)9	18	14695109	14695126
chr12_2622	p2	(TG)14	28	14704550	14704577
chr12_2623	p2	(TG)10	20	14713330	14713349
chr12_2624	p2	(GT)12	24	14717209	14717232
chr12_2625	p2	(GT)8	16	14718724	14718739
chr12_2626	p2	(AC)10	20	14726285	14726304
chr12_2627	p2	(GT)9	18	14731334	14731351
chr12_2628	p3	(GAG)6	18	14733539	14733556
chr12_2629	p2	(CA)15	30	14734745	14734774
chr12_2630	c	(TG)6ta(TG)6ttgcacagttcagctgccagtcaggctcctgaagtcca	105	14736344	14736448
chr12_2631	p2	(CA)17	34	14762256	14762289
chr12_2632	p2	(CT)6	12	14764647	14764658
chr12_2633	p2	(TG)16	32	14803377	14803408
chr12_2634	p2	(AG)7	14	14810122	14810135
chr12_2635	p2	(CA)6	12	14820659	14820670
chr12_2636	p2	(TG)10	20	14822846	14822865
chr12_2637	p2	(AC)6	12	14830344	14830355
chr12_2638	c	(GT)6ttcgttgtggtgtttgatcttcccacacttcaccgac(GT)9	67	14835149	14835215
chr12_2639	c	(GT)6(GA)6	24	14841629	14841652
chr12_2640	c	(AC)10agatgccagtaaactcggacaatcaccacgctg(AC)16	85	14845802	14845886
chr12_2641	p2	(AC)13	26	14846236	14846261
chr12_2642	p2	(TG)7	14	14847921	14847934
chr12_2643	p2	(GT)17	34	14848052	14848085
chr12_2644	p2	(CT)6	12	14851027	14851038
chr12_2645	p2	(TA)7	14	14856548	14856561
chr12_2646	p2	(GA)14	28	14878718	14878745
chr12_2647	p2	(GT)6	12	14883890	14883901
chr12_2648	p2	(GT)8	16	14899923	14899938
chr12_2649	p3	(CAT)5	15	14900732	14900746
chr12_2650	p2	(CA)14	28	14904460	14904487
chr12_2651	p2	(AC)9	18	14906817	14906834
chr12_2652	p2	(CT)8	16	14911906	14911921
chr12_2653	p2	(TC)6	12	14913243	14913254
chr12_2654	p3	(AGC)5	15	14914178	14914192
chr12_2655	p2	(TG)14	28	14919927	14919954
chr12_2656	p2	(CA)6	12	14920286	14920297
chr12_2657	p2	(CA)14	28	14933447	14933474
chr12_2658	p2	(TC)7	14	14937624	14937637
chr12_2659	p2	(TG)9	18	14942110	14942127
chr12_2660	p2	(GT)18	36	14944077	14944112
chr12_2661	p2	(TG)6	12	14949916	14949927
chr12_2662	p2	(TG)16	32	14953551	14953582
chr12_2663	p2	(GT)6	12	14954075	14954086
chr12_2664	p2	(CA)19	38	14958833	14958870
chr12_2665	p2	(CT)6	12	14959078	14959089

chr12_2666	p2	(CT)6	12	14961095	14961106
chr12_2667	p2	(TG)8	16	14965856	14965871
chr12_2668	p2	(TG)10	20	14968093	14968112
chr12_2669	p2	(GA)6	12	15001965	15001976
chr12_2670	p2	(TG)9	18	15017494	15017511
chr12_2671	p3	(ATT)5	15	15019681	15019695
chr12_2672	p2	(GT)15	30	15025366	15025395
chr12_2673	p2	(TG)10	20	15036292	15036311
chr12_2674	p3	(GAG)6	18	15048169	15048186
chr12_2675	p3	(AGA)5	15	15049699	15049713
chr12_2676	p3	(GAG)6	18	15050234	15050251
chr12_2677	p2	(TG)7	14	15050908	15050921
chr12_2678	p2	(CG)6	12	15073266	15073277
chr12_2679	p3	(AGC)9	27	15079128	15079154
chr12_2680	p2	(GT)7	14	15082829	15082842
chr12_2681	p2	(AC)7	14	15085543	15085556
chr12_2682	p2	(CA)8	16	15092111	15092126
chr12_2683	p2	(TG)8	16	15093686	15093701
chr12_2684	p2	(TC)6	12	15095549	15095560
chr12_2685	p3	(AAT)7	21	15098917	15098937
chr12_2686	p2	(CA)19	38	15101346	15101383
chr12_2687	p2	(TG)6	12	15102487	15102498
chr12_2688	p3	(GAG)6	18	15110850	15110867
chr12_2689	p2	(CA)15	30	15114293	15114322
chr12_2690	p2	(TG)17	34	15121479	15121512
chr12_2691	p2	(AC)16	32	15123471	15123502
chr12_2692	p2	(GT)16	32	15123707	15123738
chr12_2693	p3	(ATG)6	18	15124628	15124645
chr12_2694	p2	(TC)10	20	15125257	15125276
chr12_2695	p4	(ATAG)6	24	15129955	15129978
chr12_2696	p2	(GT)6	12	15132783	15132794
chr12_2697	p3	(TTC)5	15	15138967	15138981
chr12_2698	p2	(AC)18	36	15141771	15141806
chr12_2699	p3	(TCT)5	15	15142574	15142588
chr12_2700	p3	(TTC)7	21	15144978	15144998
chr12_2701	p4	(ATGG)5	20	15156056	15156075
chr12_2702	p2	(AC)12	24	15162025	15162048
chr12_2703	p2	(TG)14	28	15169057	15169084
chr12_2704	p2	(GT)15	30	15169231	15169260
chr12_2705	p2	(AC)12	24	15172306	15172329
chr12_2706	p2	(GA)7	14	15173695	15173708
chr12_2707	p2	(CA)13	26	15174347	15174372
chr12_2708	p2	(GA)6	12	15185087	15185098
chr12_2709	p3	(TTA)8	24	15186760	15186783
chr12_2710	p3	(ATA)5	15	15193983	15193997
chr12_2711	p2	(AC)10	20	15201439	15201458
chr12_2712	p2	(CA)8	16	15207983	15207998
chr12_2713	p2	(GT)6	12	15218459	15218470
chr12_2714	p2	(CA)10	20	15231362	15231381
chr12_2715	p4	(ATGG)6	24	15244363	15244386
chr12_2716	c	(AC)9t(CA)6	31	15276144	15276174
chr12_2717	p2	(CT)6	12	15284402	15284413

chr12_2718	p2	(CA)9	18	15284714	15284731
chr12_2719	p2	(CA)13	26	15288266	15288291
chr12_2720	p4	(ATGG)6	24	15292025	15292048
chr12_2721	p3	(CAA)5	15	15313079	15313093
chr12_2722	p3	(ACA)5	15	15324609	15324623
chr12_2723	p4	(CAAA)5	20	15334700	15334719
chr12_2724	p2	(AC)9	18	15339041	15339058
chr12_2725	p4	(TTCA)5	20	15341266	15341285
chr12_2726	p2	(CA)7	14	15363495	15363508
chr12_2727	p2	(AC)16	32	15366876	15366907
chr12_2728	p2	(CA)11	22	15369943	15369964
chr12_2729	c*	(GCT)5(TG)20*	54	15378280	15378333
chr12_2730	p4	(TTGT)5	20	15380501	15380520
chr12_2731	p2	(TG)9	18	15389083	15389100
chr12_2732	p3	(GAT)5	15	15389236	15389250
chr12_2733	p4	(ATGG)5	20	15397027	15397046
chr12_2734	p2	(CA)12	24	15399113	15399136
chr12_2735	p2	(CA)7	14	15400684	15400697
chr12_2736	p2	(GT)15	30	15408235	15408264
chr12_2737	p2	(AT)7	14	15424671	15424684
chr12_2738	p2	(GT)12	24	15433041	15433064
chr12_2739	p3	(CAG)5	15	15436953	15436967
chr12_2740	p3	(AAG)5	15	15438100	15438114
chr12_2741	p2	(AG)6	12	15440927	15440938
chr12_2742	p3	(TAT)8	24	15446875	15446898
chr12_2743	p2	(GT)9	18	15448979	15448996
chr12_2744	p3	(GTG)5	15	15450425	15450439
chr12_2745	p2	(CT)6	12	15452919	15452930
chr12_2746	p2	(GT)6	12	15454624	15454635
chr12_2747	p2	(TG)6	12	15469249	15469260
chr12_2748	p2	(GT)8	16	15474510	15474525
chr12_2749	p2	(GT)14	28	15477509	15477536
chr12_2750	p2	(CA)20	40	15481832	15481871
chr12_2751	p3	(CCT)5	15	15485202	15485216
chr12_2752	p2	(GT)28	56	15498126	15498181
chr12_2753	p2	(TG)12	24	15498475	15498498
chr12_2754	p4	(CTTC)5	20	15504012	15504031
chr12_2755	p2	(TA)6	12	15511082	15511093
chr12_2756	p3	(ATT)5	15	15519480	15519494
chr12_2757	p2	(TG)8	16	15540800	15540815
chr12_2758	p3	(TGC)7	21	15548960	15548980
chr12_2759	p3	(TAT)6	18	15567523	15567540
chr12_2760	p2	(GA)7	14	15572866	15572879
chr12_2761	p2	(TG)6	12	15576757	15576768
chr12_2762	p3	(GCC)5	15	15579683	15579697
chr12_2763	p3	(TAA)5	15	15580907	15580921
chr12_2764	p3	(AGG)6	18	15581224	15581241
chr12_2765	p3	(CCT)6	18	15586624	15586641
chr12_2766	p2	(TC)9	18	15588245	15588262
chr12_2767	p2	(CA)12	24	15592312	15592335
chr12_2768	p3	(CTG)6	18	15594577	15594594
chr12_2769	c	(TGT)6(TGC)7	39	15595439	15595477

chr12_2770	p3	(TGC)6	18	15596014	15596031
chr12_2771	p4	(TAGG)8	32	15601742	15601773
chr12_2772	p3	(AGC)8	24	15610236	15610259
chr12_2773	p3	(GCT)7	21	15626857	15626877
chr12_2774	p2	(TA)6	12	15639703	15639714
chr12_2775	p3	(AGC)5	15	15657218	15657232
chr12_2776	p2	(TG)9	18	15674327	15674344
chr12_2777	p3	(AAT)6	18	15675453	15675470
chr12_2778	p2	(GT)17	34	15676892	15676925
chr12_2779	c	(TG)8ta(TG)8	34	15680653	15680686
chr12_2780	p3	(ATA)5	15	15688332	15688346
chr12_2781	p3	(TTA)6	18	15703502	15703519
chr12_2782	p3	(GAT)7	21	15705789	15705809
chr12_2783	p3	(CTA)6	18	15711186	15711203
chr12_2784	p2	(GT)6	12	15716728	15716739
chr12_2785	p2	(AC)6	12	15718021	15718032
chr12_2786	p2	(TC)6	12	15719726	15719737
chr12_2787	p2	(GA)6	12	15747961	15747972
chr12_2788	p3	(TAG)10	30	15748684	15748713
chr12_2789	p2	(CA)8	16	15754768	15754783
chr12_2790	c	(CA)7tacacccacacacaacactgtcatggtc(CA)13	68	15762525	15762592
chr12_2791	c	(TC)11(AC)11	44	15762752	15762795
chr12_2792	p4	(ATTG)6	24	15772322	15772345
chr12_2793	p2	(TG)8	16	15778397	15778412
chr12_2794	p2	(TG)8	16	15780364	15780379
chr12_2795	p2	(TG)10	20	15780956	15780975
chr12_2796	p2	(CA)12	24	15786576	15786599
chr12_2797	p2	(TG)11	22	15786893	15786914
chr12_2798	p3	(CTG)5	15	15804171	15804185
chr12_2799	p3	(CCT)7	21	15807853	15807873
chr12_2800	p2	(AG)6	12	15820886	15820897
chr12_2801	c	(AGG)5gggagggggagggggat(GTG)6	51	15825206	15825256
chr12_2802	p2	(TG)7	14	15825646	15825659
chr12_2803	p2	(TG)12	24	15825935	15825958
chr12_2804	p2	(TG)13	26	15828061	15828086
chr12_2805	p3	(CTA)5	15	15858020	15858034
chr12_2806	p3	(AGC)7	21	15859192	15859212
chr12_2807	p2	(TC)15	30	15862811	15862840
chr12_2808	c	(GC)6(AC)6	24	15877495	15877518
chr12_2809	p2	(TG)8	16	15878012	15878027
chr12_2810	p2	(GT)15	30	15882176	15882205
chr12_2811	p2	(TG)17	34	15883499	15883532
chr12_2812	c	(GT)13acga(AGG)5	45	15887955	15887999
chr12_2813	p2	(CA)11	22	15888253	15888274
chr12_2814	p2	(GT)12	24	15889652	15889675
chr12_2815	p2	(CA)16	32	15894853	15894884
chr12_2816	p3	(AAC)5	15	15896912	15896926
chr12_2817	p2	(AC)9	18	15897891	15897908
chr12_2818	p2	(AC)7	14	15925415	15925428
chr12_2819	c*	(CT)9(TCTA)24*	111	15933532	15933642
chr12_2820	p2	(TG)6	12	15933788	15933799
chr12_2821	p2	(AT)7	14	15936829	15936842

chr12_2822	p2	(AC)17	34	15937115	15937148
chr12_2823	p2	(TG)21	42	15938281	15938322
chr12_2824	p2	(TG)6	12	15952163	15952174
chr12_2825	p3	(CTG)5	15	15959321	15959335
chr12_2826	p3	(GCA)5	15	15963126	15963140
chr12_2827	p3	(ATA)5	15	15969040	15969054
chr12_2828	p2	(TG)7	14	15969945	15969958
chr12_2829	p2	(TA)15	30	15980319	15980348
chr12_2830	p2	(AC)16	32	15987779	15987810
chr12_2831	p2	(AC)12	24	15990986	15991009
chr12_2832	c	(GT)7(GA)11	36	15993040	15993075
chr12_2833	p2	(GA)7	14	15995413	15995426
chr12_2834	p2	(AC)10	20	15996438	15996457
chr12_2835	p2	(TG)6	12	15999268	15999279
chr12_2836	p2	(GT)7	14	16000774	16000787
chr12_2837	p2	(TG)7	14	16004454	16004467
chr12_2838	p2	(GT)7	14	16011596	16011609
chr12_2839	p2	(TG)11	22	16012928	16012949
chr12_2840	p2	(TG)13	26	16015361	16015386
chr12_2841	p3	(TGA)6	18	16022723	16022740
chr12_2842	p2	(GA)6	12	16024241	16024252
chr12_2843	p2	(AC)24	48	16026337	16026384
chr12_2844	p2	(TG)14	28	16035755	16035782
chr12_2845	p2	(TG)14	28	16036408	16036435
chr12_2846	p4	(TTTA)5	20	16057701	16057720
chr12_2847	p2	(TG)6	12	16059472	16059483
chr12_2848	p2	(CA)13	26	16061152	16061177
chr12_2849	p2	(AC)6	12	16061476	16061487
chr12_2850	p3	(ATT)5	15	16064008	16064022
chr12_2851	p2	(TG)14	28	16073656	16073683
chr12_2852	p3	(TTC)5	15	16074997	16075011
chr12_2853	p3	(AAT)5	15	16079752	16079766
chr12_2854	p2	(AC)7	14	16098556	16098569
chr12_2855	p3	(AGT)5	15	16105386	16105400
chr12_2856	p2	(AT)6	12	16111859	16111870
chr12_2857	p3	(TCA)6	18	16114495	16114512
chr12_2858	p2	(AG)14	28	16115076	16115103
chr12_2859	p2	(GT)11	22	16116922	16116943
chr12_2860	p4	(TAAA)5	20	16117675	16117694
chr12_2861	p2	(TC)6	12	16120660	16120671
chr12_2862	p4	(CATC)6	24	16123909	16123932
chr12_2863	p2	(AC)8	16	16130053	16130068
chr12_2864	p2	(CT)6	12	16139703	16139714
chr12_2865	p2	(GA)7	14	16139990	16140003
chr12_2866	p3	(CTC)6	18	16140493	16140510
chr12_2867	p2	(GT)14	28	16142635	16142662
chr12_2868	c	(AC)10g(CA)6	33	16144717	16144749
chr12_2869	p2	(CA)17	34	16151829	16151862
chr12_2870	p2	(GT)9	18	16154027	16154044
chr12_2871	c	nnnnnnnnnnnnnnnnnnnnnnnnnnnnnnnnnnnnnnnnn	119	16155267	16155385
chr12_2872	p2	(AC)24	48	16155678	16155725
chr12_2873	p3	(TCC)5	15	16167991	16168005

chr12_2874	c	(AT)7actgtatagagagaaagagagagt(GA)8	54	16169894	16169947
chr12_2875	p2	(TG)7	14	16173610	16173623
chr12_2876	p2	(CA)9	18	16179475	16179492
chr12_2877	p2	(GA)6	12	16187542	16187553
chr12_2878	p2	(CA)13	26	16193052	16193077
chr12_2879	p2	(TG)15	30	16200648	16200677
chr12_2880	p4	(AATC)5	20	16202350	16202369
chr12_2881	c	tgtgtagtcattcctcagttgatttgccattttctgttgtgtgtgtttgagggaga	140	16203247	16203386
chr12_2882	p2	(TG)7	14	16204114	16204127
chr12_2883	p4	(GTCT)5	20	16205041	16205060
chr12_2884	p2	(AG)9	18	16210448	16210465
chr12_2885	p2	(CA)19	38	16211904	16211941
chr12_2886	p2	(AC)9	18	16216867	16216884
chr12_2887	p2	(AC)17	34	16217100	16217133
chr12_2888	p2	(AC)17	34	16225998	16226031
chr12_2889	p2	(CA)6	12	16226232	16226243
chr12_2890	p3	(TTA)5	15	16228075	16228089
chr12_2891	p2	(CA)6	12	16228820	16228831
chr12_2892	p2	(GT)20	40	16233730	16233769
chr12_2893	p3	(CTC)7	21	16245582	16245602
chr12_2894	p2	(AC)14	28	16254306	16254333
chr12_2895	p2	(TG)9	18	16259071	16259088
chr12_2896	p3	(CTC)5	15	16261604	16261618
chr12_2897	p2	(TC)9	18	16276320	16276337
chr12_2898	c	(GT)6gcgtgtgtgc(GT)7	36	16279684	16279719
chr12_2899	p2	(TG)7	14	16291168	16291181
chr12_2900	p2	(CA)20	40	16291562	16291601
chr12_2901	p2	(AC)8	16	16292274	16292289
chr12_2902	p2	(CA)12	24	16299945	16299968
chr12_2903	p4	(AGAT)11	44	16300666	16300709
chr12_2904	p3	(ATC)6	18	16304064	16304081
chr12_2905	p3	(TAC)5	15	16306387	16306401
chr12_2906	p2	(TG)13	26	16312214	16312239
chr12_2907	p2	(AG)8	16	16312776	16312791
chr12_2908	p2	(CA)8	16	16320573	16320588
chr12_2909	p2	(TG)6	12	16326504	16326515
chr12_2910	p2	(AG)6	12	16333972	16333983
chr12_2911	p2	(TG)14	28	16334740	16334767
chr12_2912	c	(TA)6tc(TA)7	28	16335504	16335531
chr12_2913	p4	(AATG)7	28	16337982	16338009
chr12_2914	c	cacacattcattcacacaccggcagagcgacagcagcggtgccctgagg	110	16344388	16344497
chr12_2915	c	(TA)7tgtcatggatgtaacatgc(TA)8	49	16345048	16345096
chr12_2916	p2	(GA)7	14	16347495	16347508
chr12_2917	p3	(TCC)5	15	16355141	16355155
chr12_2918	p2	(AG)9	18	16357323	16357340
chr12_2919	p2	(TC)7	14	16358269	16358282
chr12_2920	p2	(TG)8	16	16359840	16359855
chr12_2921	p2	(CA)17	34	16362222	16362255
chr12_2922	p3	(GAT)5	15	16367042	16367056
chr12_2923	p2	(GT)6	12	16374510	16374521
chr12_2924	p2	(TC)13	26	16381055	16381080
chr12_2925	p2	(AC)25	50	16381605	16381654

chr12_2926	c	[illegible]nnnnnnnnnnnnnnnnnnnnnnnnnnnnccctccccacacccaac	121	16383720	16383840
chr12_2927	p2	(AC)8	16	16384253	16384268
chr12_2928	p2	(CT)6	12	16384666	16384677
chr12_2929	p2	(CA)7	14	16386696	16386709
chr12_2930	p2	(AC)7	14	16392312	16392325
chr12_2931	p3	(TAA)7	21	16398615	16398635
chr12_2932	p4	(CCAT)5	20	16408328	16408347
chr12_2933	p2	(TG)6	12	16417749	16417760
chr12_2934	p2	(AC)13	26	16424648	16424673
chr12_2935	p2	(GT)7	14	16424831	16424844
chr12_2936	p2	(GT)12	24	16425065	16425088
chr12_2937	p3	(TGA)9	27	16431507	16431533
chr12_2938	p3	(TGA)9	27	16433249	16433275
chr12_2939	p3	(CAT)6	18	16440001	16440018
chr12_2940	p3	(CTA)5	15	16443742	16443756
chr12_2941	p2	(TC)6	12	16457818	16457829
chr12_2942	p4	(GCCT)5	20	16463328	16463347
chr12_2943	p2	(TG)6	12	16464248	16464259
chr12_2944	p3	(TAG)5	15	16487466	16487480
chr12_2945	p2	(TG)15	30	16492195	16492224
chr12_2946	p2	(TG)13	26	16495926	16495951
chr12_2947	p3	(TGC)5	15	16497824	16497838
chr12_2948	p3	(GAG)10	30	16499230	16499259
chr12_2949	p2	(TG)7	14	16499472	16499485
chr12_2950	p2	(GA)6	12	16501714	16501725
chr12_2951	p2	(CT)7	14	16501890	16501903
chr12_2952	p2	(GT)18	36	16514567	16514602
chr12_2953	p2	(CA)17	34	16518409	16518442
chr12_2954	p2	(TG)9	18	16520796	16520813
chr12_2955	p2	(GT)6	12	16521260	16521271
chr12_2956	p2	(TG)6	12	16522791	16522802
chr12_2957	p3	(CCA)5	15	16534542	16534556
chr12_2958	p2	(TC)6	12	16538193	16538204
chr12_2959	p2	(TG)8	16	16546530	16546545
chr12_2960	p2	(TC)45	90	16558363	16558452
chr12_2961	p3	(TCA)5	15	16558794	16558808
chr12_2962	p3	(AAG)5	15	16604267	16604281
chr12_2963	p4	(ATAG)17	68	16609480	16609547
chr12_2964	p2	(GT)10	20	16615528	16615547
chr12_2965	p3	(AAT)5	15	16615939	16615953
chr12_2966	p2	(CA)6	12	16617953	16617964
chr12_2967	p3	(TAC)5	15	16619211	16619225
chr12_2968	p2	(TG)16	32	16627344	16627375
chr12_2969	p2	(GT)15	30	16628211	16628240
chr12_2970	p2	(TG)12	24	16634711	16634734
chr12_2971	p2	(TG)10	20	16635680	16635699
chr12_2972	p2	(TG)12	24	16636045	16636068
chr12_2973	p2	(GA)9	18	16646444	16646461
chr12_2974	c	(CT)12cccattctctcttatactctctcttccattc(CT)6	67	16649957	16650023
chr12_2975	p3	(ATC)8	24	16651360	16651383
chr12_2976	p2	(AC)6	12	16653299	16653310
chr12_2977	p3	(TCT)5	15	16654778	16654792

chr12_2978	c	gaaagtgagaaaaa(GT)9tcgcccctttctcgctccgtttcaactgaaaa	227	16658015	16658241
chr12_2979	p2	(TG)18	36	16658425	16658460
chr12_2980	c	(CA)16acacacg(CA)8	55	16662641	16662695
chr12_2981	p2	(CA)6	12	16664425	16664436
chr12_2982	p3	(TTC)5	15	16669793	16669807
chr12_2983	p2	(GT)6	12	16677537	16677548
chr12_2984	c	(CA)6gcatatg(CA)6	31	16677941	16677971
chr12_2985	p2	(CT)6	12	16680139	16680150
chr12_2986	p2	(AC)13	26	16681649	16681674
chr12_2987	c	:aaatcaataccaacacgagcttctcagtaaaccacaagcaagaggcacac	94	16685853	16685946
chr12_2988	p2	(CA)15	30	16686163	16686192
chr12_2989	p2	(CA)26	52	16687372	16687423
chr12_2990	c	(TC)6(AC)14	40	16687585	16687624
chr12_2991	p2	(AC)16	32	16687767	16687798
chr12_2992	p2	(CA)8	16	16693925	16693940
chr12_2993	p2	(GT)6	12	16694130	16694141
chr12_2994	p2	(TG)10	20	16694283	16694302
chr12_2995	p2	(AC)8	16	16694493	16694508
chr12_2996	c	(CT)7(CA)15	44	16695807	16695850
chr12_2997	p2	(GT)10	20	16696344	16696363
chr12_2998	p2	(AC)6	12	16697065	16697076
chr12_2999	p2	(CA)12	24	16697612	16697635
chr12_3000	p2	(GT)12	24	16697821	16697844
chr12_3001	p3	(GCT)5	15	16698244	16698258
chr12_3002	p2	(CA)16	32	16705100	16705131
chr12_3003	p2	(AC)9	18	16712076	16712093
chr12_3004	p2	(CA)10	20	16718981	16719000
chr12_3005	p2	(TG)8	16	16722145	16722160
chr12_3006	p2	(TG)8	16	16723058	16723073
chr12_3007	p2	(CA)11	22	16725149	16725170
chr12_3008	p2	(TG)9	18	16725360	16725377
chr12_3009	c	:gtgggcggcaaacagaagaacatctgatgacatgtacagctgcttcagac	118	16726646	16726763
chr12_3010	c	:gtctgt(CA)27ccgtcttccattatcagttcccaatcaccagttattcacac	163	16728471	16728633
chr12_3011	p2	(TG)7	14	16728809	16728822
chr12_3012	p2	(AC)29	58	16730010	16730067
chr12_3013	p2	(CA)14	28	16730178	16730205
chr12_3014	p2	(GT)18	36	16735245	16735280
chr12_3015	p2	(AC)18	36	16735516	16735551
chr12_3016	p2	(TA)6	12	16747824	16747835
chr12_3017	p2	(CA)13	26	16748291	16748316
chr12_3018	p3	(CTC)6	18	16748515	16748532
chr12_3019	p2	(TG)10	20	16752620	16752639
chr12_3020	p2	(AT)7	14	16757997	16758010
chr12_3021	p3	(AGA)5	15	16761209	16761223
chr12_3022	c	aacagaagaagg(GT)12tgagtcagactgactgtgaatctcttgatgtg	230	16764423	16764652
chr12_3023	p2	(AC)8	16	16764885	16764900
chr12_3024	p2	(AC)9	18	16765700	16765717
chr12_3025	p2	(TG)15	30	16766559	16766588
chr12_3026	p2	(AC)6	12	16776401	16776412
chr12_3027	c	(CT)8gtct(CA)8	36	16776647	16776682
chr12_3028	p2	(TG)41	82	16779621	16779702
chr12_3029	p2	(TG)8	16	16786506	16786521

chr12_3030	p2	(AC)12	24	16790808	16790831
chr12_3031	c	gagatcagctgagtgtgttcacaactaatttcaatcatagagacaaacttagtt	117	16793628	16793744
chr12_3032	p2	(CT)7	14	16800227	16800240
chr12_3033	p3	(TCT)5	15	16803004	16803018
chr12_3034	p3	(GAG)5	15	16804836	16804850
chr12_3035	p2	(TG)14	28	16805281	16805308
chr12_3036	p2	(AC)18	36	16805491	16805526
chr12_3037	p2	(TG)9	18	16805673	16805690
chr12_3038	p2	(AC)14	28	16813716	16813743
chr12_3039	p2	(AC)8	16	16817522	16817537
chr12_3040	p3	(ATA)5	15	16820411	16820425
chr12_3041	p2	(GC)6	12	16823340	16823351
chr12_3042	p2	(GT)11	22	16823491	16823512
chr12_3043	p2	(AC)11	22	16824348	16824369
chr12_3044	p2	(AC)7	14	16826828	16826841
chr12_3045	p2	(AC)6	12	16827641	16827652
chr12_3046	p2	(TG)6	12	16830224	16830235
chr12_3047	p2	(CT)7	14	16830511	16830524
chr12_3048	p2	(CA)8	16	16841873	16841888
chr12_3049	p2	(AC)9	18	16844141	16844158
chr12_3050	p2	(CA)11	22	16846413	16846434
chr12_3051	p4	(TCCA)6	24	16847766	16847789
chr12_3052	p3	(GAA)5	15	16849552	16849566
chr12_3053	p2	(CT)7	14	16850283	16850296
chr12_3054	p2	(GT)22	44	16854480	16854523
chr12_3055	p2	(GT)6	12	16859436	16859447
chr12_3056	p2	(CA)12	24	16861999	16862022
chr12_3057	p2	(GT)17	34	16862458	16862491
chr12_3058	p2	(AC)19	38	16866132	16866169
chr12_3059	p2	(TG)6	12	16866317	16866328
chr12_3060	p3	(ATA)5	15	16892008	16892022
chr12_3061	p2	(GA)22	44	16892920	16892963
chr12_3062	p2	(TC)7	14	16895433	16895446
chr12_3063	p2	(AC)7	14	16899954	16899967
chr12_3064	p2	(CT)9	18	16903753	16903770
chr12_3065	p3	(CAG)6	18	16903995	16904012
chr12_3066	p2	(TA)6	12	16905452	16905463
chr12_3067	p2	(TG)7	14	16923730	16923743
chr12_3068	c	g(AC)6aaatggctttatacgtgcagattgcgcagcacgcatttgtgatcct	212	16924496	16924707
chr12_3069	p3	(GTA)5	15	16925667	16925681
chr12_3070	p2	(AG)6	12	16956601	16956612
chr12_3071	p5	(AACAC)7	35	16959652	16959686
chr12_3072	p2	(TG)7	14	16967900	16967913
chr12_3073	p2	(AC)6	12	16973513	16973524
chr12_3074	p3	(AAT)5	15	16979790	16979804
chr12_3075	p3	(CCT)5	15	16980089	16980103
chr12_3076	p2	(CA)6	12	16983964	16983975
chr12_3077	p2	(CA)15	30	16986638	16986667
chr12_3078	p3	(TAC)5	15	16996248	16996262
chr12_3079	p2	(TG)8	16	17001999	17002014
chr12_3080	p2	(AC)8	16	17007376	17007391
chr12_3081	p2	(GA)6	12	17017231	17017242

chr12_3082	p2	(TG)15	30	17022280	17022309
chr12_3083	c	(TG)9(AG)6	30	17022592	17022621
chr12_3084	p2	(GT)13	26	17029373	17029398
chr12_3085	c	(GT)6t(TG)10	33	17034095	17034127
chr12_3086	p2	(GT)6	12	17038536	17038547
chr12_3087	p2	(GT)8	16	17048574	17048589
chr12_3088	p2	(TC)6	12	17058695	17058706
chr12_3089	p2	(AC)12	24	17067795	17067818
chr12_3090	p2	(CA)9	18	17068487	17068504
chr12_3091	p3	(CCT)7	21	17070228	17070248
chr12_3092	p3	(CCT)6	18	17070367	17070384
chr12_3093	p2	(TG)14	28	17075266	17075293
chr12_3094	p4	(ATGG)5	20	17078452	17078471
chr12_3095	p2	(TG)6	12	17085968	17085979
chr12_3096	p4	(CCTC)6	24	17086553	17086576
chr12_3097	p2	(TG)13	26	17087675	17087700
chr12_3098	p2	(AC)22	44	17097349	17097392
chr12_3099	p2	(TA)6	12	17100239	17100250
chr12_3100	p2	(CA)15	30	17105967	17105996
chr12_3101	p2	(AC)6	12	17107519	17107530
chr12_3102	p3	(GTG)6	18	17108190	17108207
chr12_3103	p2	(AC)9	18	17115260	17115277
chr12_3104	p2	(TG)28	56	17115869	17115924
chr12_3105	p2	(TG)6	12	17118528	17118539
chr12_3106	p4	(TTGA)5	20	17122733	17122752
chr12_3107	p2	(CA)9	18	17127188	17127205
chr12_3108	p2	(TG)9	18	17133544	17133561
chr12_3109	p2	(TA)12	24	17140232	17140255
chr12_3110	p2	(CA)11	22	17141694	17141715
chr12_3111	p2	(CT)7	14	17145687	17145700
chr12_3112	p2	(CT)8	16	17146842	17146857
chr12_3113	p2	(TG)7	14	17148378	17148391
chr12_3114	p2	(CA)9	18	17183166	17183183
chr12_3115	p2	(AC)22	44	17192503	17192546
chr12_3116	p2	(AC)12	24	17196833	17196856
chr12_3117	p2	(AC)6	12	17199387	17199398
chr12_3118	p2	(AG)6	12	17200804	17200815
chr12_3119	p2	(GT)6	12	17202360	17202371
chr12_3120	c	(CG)7(CA)6	26	17210309	17210334
chr12_3121	p2	(AG)8	16	17216433	17216448
chr12_3122	p2	(AT)8	16	17233689	17233704
chr12_3123	p2	(AT)8	16	17238634	17238649
chr12_3124	p2	(TG)18	36	17245988	17246023
chr12_3125	p2	(TC)6	12	17247093	17247104
chr12_3126	p2	(AC)13	26	17252986	17253011
chr12_3127	p3	(TAT)7	21	17256267	17256287
chr12_3128	p2	(TG)8	16	17261741	17261756
chr12_3129	p2	(TG)6	12	17268507	17268518
chr12_3130	p2	(TG)7	14	17268733	17268746
chr12_3131	p2	(TG)20	40	17319880	17319919
chr12_3132	p5	(AGTAG)5	25	17333006	17333030
chr12_3133	p3	(AAG)5	15	17346879	17346893

chr12_3134	p2	(TC)6	12	17356558	17356569
chr12_3135	p2	(AC)7	14	17359627	17359640
chr12_3136	c	gagagagagtaggagccagaaaagggaatgaccactgggtggagcctc	122	17370317	17370438
chr12_3137	p2	(TG)9	18	17384555	17384572
chr12_3138	p3	(TGG)5	15	17395491	17395505
chr12_3139	p4	(AATA)6	24	17414379	17414402
chr12_3140	p2	(AC)6	12	17431684	17431695
chr12_3141	p4	(TTTA)5	20	17435584	17435603
chr12_3142	c	acagaaaaaaagacataagaaacagcaacagacagagga(AG)6gga	119	17435778	17435896
chr12_3143	p3	(GTT)8	24	17445863	17445886
chr12_3144	p2	(TG)15	30	17449586	17449615
chr12_3145	p4	(AAAC)6	24	17456900	17456923
chr12_3146	p2	(AC)9	18	17457413	17457430
chr12_3147	p2	(TC)6	12	17466819	17466830
chr12_3148	p2	(GT)7	14	17494394	17494407
chr12_3149	p2	(AG)9	18	17496651	17496668
chr12_3150	p2	(TG)9	18	17505379	17505396
chr12_3151	p3	(TGC)6	18	17506184	17506201
chr12_3152	p2	(TG)7	14	17507126	17507139
chr12_3153	p3	(GCT)6	18	17508493	17508510
chr12_3154	p2	(CA)7	14	17508816	17508829
chr12_3155	p2	(TG)6	12	17516007	17516018
chr12_3156	p2	(TG)6	12	17516223	17516234
chr12_3157	p2	(TG)17	34	17521222	17521255
chr12_3158	p2	(GA)6	12	17521667	17521678
chr12_3159	p2	(CA)22	44	17522877	17522920
chr12_3160	p2	(AG)10	20	17523877	17523896
chr12_3161	p2	(AC)22	44	17525183	17525226
chr12_3162	p2	(CA)19	38	17528623	17528660
chr12_3163	p2	(CA)6	12	17529690	17529701
chr12_3164	c	(AC)12(GC)6	36	17538024	17538059
chr12_3165	p2	(AC)6	12	17538580	17538591
chr12_3166	p2	(AC)9	18	17538830	17538847
chr12_3167	p2	(TC)10	20	17540252	17540271
chr12_3168	p2	(TG)21	42	17540406	17540447
chr12_3169	p2	(AG)6	12	17541080	17541091
chr12_3170	p2	(AC)17	34	17541877	17541910
chr12_3171	c	(GT)7a(TG)10	35	17546116	17546150
chr12_3172	p2	(CA)6	12	17548082	17548093
chr12_3173	c	(CT)10gcgtgtggtgtgtgtgtatgtgtatttgcccgctaccc(TG)6	70	17548738	17548807
chr12_3174	c	(TAA)5tactaataccactactactaataataatat(TAA)5	60	17550398	17550457
chr12_3175	p3	(TAA)7	21	17552119	17552139
chr12_3176	p2	(CA)11	22	17553084	17553105
chr12_3177	p2	(GT)8	16	17553884	17553899
chr12_3178	c	(TAA)5tactaataccactactactaataataatat(TAA)5	60	17570322	17570381
chr12_3179	p3	(TAA)7	21	17572043	17572063
chr12_3180	p2	(CA)11	22	17573008	17573029
chr12_3181	p2	(GT)8	16	17573808	17573823
chr12_3182	p2	(CA)9	18	17582049	17582066
chr12_3183	p3	(TCT)5	15	17605259	17605273
chr12_3184	p2	(CA)20	40	17612973	17613012
chr12_3185	p2	(GA)6	12	17615606	17615617

chr12_3186	p2	(CA)6	12	17619494	17619505
chr12_3187	p4	(AATG)6	24	17630083	17630106
chr12_3188	p2	(GA)7	14	17633396	17633409
chr12_3189	p3	(ATT)5	15	17643975	17643989
chr12_3190	p3	(ATT)5	15	17646393	17646407
chr12_3191	p2	(AC)9	18	17686986	17687003
chr12_3192	p2	(AT)8	16	17729661	17729676
chr12_3193	p2	(CA)8	16	17733066	17733081
chr12_3194	p2	(AG)7	14	17736603	17736616
chr12_3195	p3	(ATA)5	15	17738242	17738256
chr12_3196	p2	(CA)23	46	17754082	17754127
chr12_3197	p2	(TG)12	24	17759885	17759908
chr12_3198	c	(AC)13(TG)17	60	17760842	17760901
chr12_3199	p2	(TG)6	12	17775782	17775793
chr12_3200	p2	(CA)8	16	17778671	17778686
chr12_3201	p2	(TG)24	48	17779041	17779088
chr12_3202	p2	(AG)11	22	17782195	17782216
chr12_3203	p2	(GT)6	12	17790322	17790333
chr12_3204	p2	(AC)8	16	17791557	17791572
chr12_3205	c	(TG)11tatatatataatatgtgtatatgcca(TG)9(TA)8	82	17792639	17792720
chr12_3206	p4	(TTCA)6	24	17806461	17806484
chr12_3207	p2	(TG)6	12	17822956	17822967
chr12_3208	p2	(TG)6	12	17823069	17823080
chr12_3209	p2	(AG)6	12	17829268	17829279
chr12_3210	p2	(TG)8	16	17839161	17839176
chr12_3211	p2	(GT)9	18	17857080	17857097
chr12_3212	p3	(CAT)5	15	17868820	17868834
chr12_3213	p2	(CA)24	48	17875169	17875216
chr12_3214	c	(CA)6t(AC)8	29	17875667	17875695
chr12_3215	c	(GTG)5gggtgggtgcagggcgttttttgtgtca(GT)7	57	17880742	17880798
chr12_3216	p2	(CA)8	16	17881703	17881718
chr12_3217	p2	(AC)6	12	17882218	17882229
chr12_3218	p3	(AGC)9	27	17904606	17904632
chr12_3219	c	(AG)7aaagagac(AG)7	36	17914323	17914358
chr12_3220	c	(AG)7aaagagac(AG)7	36	17916469	17916504
chr12_3221	p2	(CA)6	12	17932949	17932960
chr12_3222	p4	(GCCT)6	24	17955432	17955455
chr12_3223	p2	(AC)6	12	17960870	17960881
chr12_3224	p2	(TG)17	34	17968828	17968861
chr12_3225	p4	(TGTT)5	20	17974893	17974912
chr12_3226	p2	(TG)10	20	17979470	17979489
chr12_3227	p2	(AC)12	24	17982606	17982629
chr12_3228	p2	(CA)36	72	17994683	17994754
chr12_3229	p2	(CA)6	12	17997478	17997489
chr12_3230	p3	(TAA)9	27	17997682	17997708
chr12_3231	p2	(GT)10	20	17998531	17998550
chr12_3232	p4	(TAAA)5	20	18006104	18006123
chr12_3233	c	(TG)9caaatatctgcatgtgtgtga(GT)7	53	18012697	18012749
chr12_3234	p2	(CT)6	12	18014996	18015007
chr12_3235	p2	(TG)7	14	18016474	18016487
chr12_3236	p2	(TG)18	36	18023091	18023126
chr12_3237	p2	(AG)6	12	18031463	18031474

chr12_3238	p2	(TG)12	24	18032828	18032851
chr12_3239	p2	(GT)12	24	18038917	18038940
chr12_3240	p2	(TG)21	42	18052585	18052626
chr12_3241	p2	(TG)11	22	18067572	18067593
chr12_3242	p2	(AC)14	28	18067878	18067905
chr12_3243	p3	(GTA)5	15	18074124	18074138
chr12_3244	c	(TG)15g(GT)20gagt(GA)11	97	18077893	18077989
chr12_3245	p2	(TG)11	22	18078263	18078284
chr12_3246	p2	(GT)6	12	18079478	18079489
chr12_3247	p2	(TC)7	14	18101541	18101554
chr12_3248	p3	(ATT)5	15	18106317	18106331
chr12_3249	p2	(AC)8	16	18108710	18108725
chr12_3250	p5	(AAATC)7	35	18111655	18111689
chr12_3251	p2	(TG)6	12	18115180	18115191
chr12_3252	p2	(TA)7	14	18130909	18130922
chr12_3253	p2	(TG)20	40	18138365	18138404
chr12_3254	p2	(TG)7	14	18139228	18139241
chr12_3255	p2	(TA)6	12	18144789	18144800
chr12_3256	c	(TA)7(TG)15	44	18145361	18145404
chr12_3257	p2	(CA)12	24	18145715	18145738
chr12_3258	p2	(TG)27	54	18154125	18154178
chr12_3259	p2	(TG)6	12	18160329	18160340
chr12_3260	p3	(TAA)5	15	18161286	18161300
chr12_3261	p4	(CATT)6	24	18161825	18161848
chr12_3262	p2	(GT)6	12	18162886	18162897
chr12_3263	p2	(AC)6	12	18167636	18167647
chr12_3264	p2	(TG)17	34	18174848	18174881
chr12_3265	p2	(TA)7	14	18178588	18178601
chr12_3266	p4	(TTTG)5	20	18180610	18180629
chr12_3267	p4	(AAAC)5	20	18185524	18185543
chr12_3268	p4	(ATCC)5	20	18185781	18185800
chr12_3269	p2	(AC)15	30	18191561	18191590
chr12_3270	p3	(GAT)6	18	18192306	18192323
chr12_3271	p2	(GT)17	34	18194834	18194867
chr12_3272	p2	(CA)12	24	18196399	18196422
chr12_3273	p3	(TAT)5	15	18202112	18202126
chr12_3274	p4	(AATG)5	20	18213435	18213454
chr12_3275	p2	(TG)8	16	18213916	18213931
chr12_3276	p2	(AC)29	58	18228670	18228727
chr12_3277	p2	(AC)20	40	18229013	18229052
chr12_3278	p2	(AC)6	12	18234166	18234177
chr12_3279	p2	(AT)6	12	18239624	18239635
chr12_3280	p2	(CA)6	12	18240114	18240125
chr12_3281	p2	(GT)8	16	18241766	18241781
chr12_3282	p2	(AC)11	22	18244269	18244290
chr12_3283	p3	(CTG)5	15	18245850	18245864
chr12_3284	p3	(AAT)5	15	18254007	18254021
chr12_3285	p4	(ATTG)6	24	18254956	18254979
chr12_3286	c	(CA)30c(CA)6	73	18282499	18282571
chr12_3287	p4	(TGAT)5	20	18299390	18299409
chr12_3288	p2	(AC)7	14	18300667	18300680
chr12_3289	p2	(GT)14	28	18303622	18303649

chr12_3290	p2	(GA)6	12	18307319	18307330
chr12_3291	p2	(AC)6	12	18315152	18315163
chr12_3292	p2	(GT)7	14	18316435	18316448
chr12_3293	p2	(CA)7	14	18318432	18318445
chr12_3294	p2	(GT)11	22	18322123	18322144
chr12_3295	p2	(AG)6	12	18326210	18326221
chr12_3296	p2	(AT)6	12	18330694	18330705
chr12_3297	p2	(AC)10	20	18343198	18343217
chr12_3298	p2	(CA)10	20	18348261	18348280
chr12_3299	p3	(GTT)5	15	18348545	18348559
chr12_3300	c	(GT)9ta(GT)14	48	18355037	18355084
chr12_3301	p2	(CT)8	16	18356815	18356830
chr12_3302	p2	(AC)6	12	18357433	18357444
chr12_3303	p2	(GT)7	14	18357736	18357749
chr12_3304	p2	(CA)6	12	18373405	18373416
chr12_3305	p2	(CA)9	18	18374274	18374291
chr12_3306	p3	(ATA)5	15	18380692	18380706
chr12_3307	p2	(TG)13	26	18385229	18385254
chr12_3308	p2	(AG)6	12	18387006	18387017
chr12_3309	p2	(GT)23	46	18391949	18391994
chr12_3310	p2	(CA)22	44	18392245	18392288
chr12_3311	p2	(AC)14	28	18397623	18397650
chr12_3312	p2	(GT)8	16	18398426	18398441
chr12_3313	p2	(CA)27	54	18414655	18414708
chr12_3314	p2	(AC)6	12	18419108	18419119
chr12_3315	p2	(TG)6	12	18423244	18423255
chr12_3316	p2	(CA)8	16	18426486	18426501
chr12_3317	p2	(TG)31	62	18428908	18428969
chr12_3318	p2	(TG)32	64	18429211	18429274
chr12_3319	p2	(AC)6	12	18430754	18430765
chr12_3320	c	gtgtgtgcgcgcactcaggtttgagtgtgtgtgcaagaaggcagagaagc	90	18435204	18435293
chr12_3321	p2	(CA)7	14	18435601	18435614
chr12_3322	p2	(TA)10	20	18443525	18443544
chr12_3323	p2	(TA)10	20	18443710	18443729
chr12_3324	p3	(TCT)5	15	18447024	18447038
chr12_3325	p2	(CT)7	14	18451819	18451832
chr12_3326	p2	(CT)7	14	18451941	18451954
chr12_3327	p2	(GT)16	32	18454968	18454999
chr12_3328	p2	(TG)32	64	18457123	18457186
chr12_3329	p2	(GT)8	16	18457528	18457543
chr12_3330	p2	(AC)11	22	18459754	18459775
chr12_3331	p2	(TG)47	94	18462695	18462788
chr12_3332	p2	(GT)6	12	18464112	18464123
chr12_3333	p2	(CA)6	12	18484839	18484850
chr12_3334	p3	(CTG)8	24	18495058	18495081
chr12_3335	p2	(AC)6	12	18528180	18528191
chr12_3336	p3	(ATG)5	15	18528967	18528981
chr12_3337	p2	(AC)8	16	18532517	18532532
chr12_3338	c	tgaacacctgctacctccctttttgatcaagatgttatttccatcactcctacaa	115	18546307	18546421
chr12_3339	p2	(CA)10	20	18549821	18549840
chr12_3340	p2	(AC)7	14	18552471	18552484
chr12_3341	p2	(CA)12	24	18553042	18553065

chr12_3342	p4	(ATGG)5	20	18554798	18554817
chr12_3343	p2	(AC)9	18	18563099	18563116
chr12_3344	p2	(TG)7	14	18568729	18568742
chr12_3345	p2	(GT)13	26	18570699	18570724
chr12_3346	p2	(TG)6	12	18598289	18598300
chr12_3347	p2	(CA)7	14	18602142	18602155
chr12_3348	p2	(TG)11	22	18603486	18603507
chr12_3349	p2	(GT)10	20	18612895	18612914
chr12_3350	p2	(GT)8	16	18614975	18614990
chr12_3351	p2	(AC)12	24	18624807	18624830
chr12_3352	p3	(AGA)5	15	18632988	18633002
chr12_3353	p2	(GT)6	12	18636049	18636060
chr12_3354	p2	(CA)6	12	18638760	18638771
chr12_3355	p2	(AC)8	16	18642771	18642786
chr12_3356	p2	(GT)7	14	18643498	18643511
chr12_3357	p2	(TG)6	12	18646930	18646941
chr12_3358	p2	(AG)8	16	18653198	18653213
chr12_3359	p2	(AC)6	12	18657427	18657438
chr12_3360	p3	(TCC)6	18	18658594	18658611
chr12_3361	p2	(AG)7	14	18661673	18661686
chr12_3362	p2	(GT)8	16	18672255	18672270
chr12_3363	p2	(TG)6	12	18676692	18676703
chr12_3364	p2	(TA)6	12	18681232	18681243
chr12_3365	p2	(AC)6	12	18681392	18681403
chr12_3366	p2	(AC)6	12	18682272	18682283
chr12_3367	p3	(GAG)8	24	18684571	18684594
chr12_3368	p2	(TA)6	12	18703171	18703182
chr12_3369	p2	(CA)7	14	18709289	18709302
chr12_3370	p2	(AC)7	14	18711500	18711513
chr12_3371	p2	(TG)17	34	18719876	18719909
chr12_3372	p3	(AAT)5	15	18724143	18724157
chr12_3373	p3	(ATA)6	18	18727397	18727414
chr12_3374	c	(TG)12ta(TG)7	40	18730943	18730982
chr12_3375	p3	(TTA)6	18	18735900	18735917
chr12_3376	p2	(AC)6	12	18760840	18760851
chr12_3377	p2	(CT)6	12	18765605	18765616
chr12_3378	p2	(TG)6	12	18766442	18766453
chr12_3379	p2	(CT)16	32	18778199	18778230
chr12_3380	p3	(TTA)6	18	18789256	18789273
chr12_3381	p2	(AC)22	44	18792347	18792390
chr12_3382	p2	(TG)12	24	18793341	18793364
chr12_3383	p3	(TTA)9	27	18803449	18803475
chr12_3384	p3	(ATA)8	24	18805478	18805501
chr12_3385	p3	(AGA)5	15	18829780	18829794
chr12_3386	p3	(TCC)6	18	18837141	18837158
chr12_3387	p2	(TG)6	12	18844134	18844145
chr12_3388	p2	(TG)6	12	18844376	18844387
chr12_3389	p2	(GT)10	20	18852739	18852758
chr12_3390	p2	(GT)6	12	18858037	18858048
chr12_3391	p3	(GCA)7	21	18873161	18873181
chr12_3392	p2	(TG)6	12	18875986	18875997
chr12_3393	p2	(TG)7	14	18883143	18883156

chr12_3394	p2	(GT)13	26	18883328	18883353
chr12_3395	p2	(TG)11	22	18883502	18883523
chr12_3396	p2	(AC)6	12	18883697	18883708
chr12_3397	p2	(AC)12	24	18883887	18883910
chr12_3398	p2	(GT)9	18	18888806	18888823
chr12_3399	p2	(GT)10	20	18894726	18894745
chr12_3400	p2	(TG)9	18	18897815	18897832
chr12_3401	p2	(TG)6	12	18911352	18911363
chr12_3402	p2	(TG)6	12	18929563	18929574
chr12_3403	p2	(AC)13	26	18932240	18932265
chr12_3404	p2	(TG)6	12	18935980	18935991
chr12_3405	p2	(TG)20	40	18936104	18936143
chr12_3406	p2	(TG)11	22	18936866	18936887
chr12_3407	c	(TATG)6gaaggattttttggttgttcatat(TG)25	99	18945454	18945552
chr12_3408	p2	(AG)6	12	18945720	18945731
chr12_3409	p2	(TA)6	12	18948836	18948847
chr12_3410	p2	(CA)6	12	18955435	18955446
chr12_3411	c	actcctgcatacacaaatgggagcacagtgtataaaaataacacactcact	103	18955704	18955806
chr12_3412	p2	(AG)6	12	18958412	18958423
chr12_3413	p2	(GT)9	18	18960933	18960950
chr12_3414	p2	(AC)12	24	18966274	18966297
chr12_3415	p2	(AC)6	12	18972094	18972105
chr12_3416	p2	(TG)6	12	18973776	18973787
chr12_3417	p2	(CA)7	14	18978615	18978628
chr12_3418	p2	(AC)16	32	18979953	18979984
chr12_3419	p2	(GT)9	18	18982161	18982178
chr12_3420	p2	(GT)9	18	18987100	18987117
chr12_3421	p2	(TC)6	12	18987253	18987264
chr12_3422	p3	(ACA)5	15	18994241	18994255
chr12_3423	p2	(GT)14	28	18998889	18998916
chr12_3424	p4	(TTTG)5	20	19000946	19000965
chr12_3425	p2	(GT)12	24	19002030	19002053
chr12_3426	p5	(AAATC)5	25	19009956	19009980
chr12_3427	p3	(GAG)8	24	19017232	19017255
chr12_3428	p2	(CT)6	12	19023147	19023158
chr12_3429	p2	(AT)8	16	19025069	19025084
chr12_3430	p3	(ATA)5	15	19026019	19026033
chr12_3431	p3	(AGA)5	15	19035128	19035142
chr12_3432	p2	(GT)9	18	19035850	19035867
chr12_3433	p2	(GT)15	30	19044435	19044464
chr12_3434	p2	(CA)12	24	19047423	19047446
chr12_3435	c	AC)8ag(AC)8ag(AC)8ag(AC)8ag(AC)8ag(AC)8ag(A	355	19048180	19048534
chr12_3436	p2	(TG)7	14	19050761	19050774
chr12_3437	p2	(AC)12	24	19054324	19054347
chr12_3438	p2	(CA)33	66	19056592	19056657
chr12_3439	c	cagtctttctctccaaaagctgcatctcaaagagttgattctccactctgatg	124	19058810	19058933
chr12_3440	p2	(GT)13	26	19061190	19061215
chr12_3441	p3	(GGA)7	21	19063986	19064006
chr12_3442	p2	(CA)17	34	19066364	19066397
chr12_3443	p2	(GT)16	32	19070891	19070922
chr12_3444	p2	(AC)14	28	19072955	19072982
chr12_3445	p2	(TG)11	22	19074636	19074657

chr12_3446	p2	(AC)9	18	19074794	19074811
chr12_3447	p2	(GA)6	12	19078759	19078770
chr12_3448	p2	(CA)11	22	19078898	19078919
chr12_3449	p3	(CTG)6	18	19079763	19079780
chr12_3450	p5	(ATTTG)6	30	19088049	19088078
chr12_3451	p2	(CA)10	20	19091362	19091381
chr12_3452	p2	(TG)7	14	19094966	19094979
chr12_3453	p2	(AC)7	14	19096216	19096229
chr12_3454	p2	(GT)6	12	19097714	19097725
chr12_3455	p3	(TCA)8	24	19104355	19104378
chr12_3456	p5	(TTTGA)5	25	19107323	19107347
chr12_3457	p2	(CA)22	44	19109797	19109840
chr12_3458	p2	(AC)19	38	19110008	19110045
chr12_3459	p2	(TG)6	12	19118469	19118480
chr12_3460	p2	(AC)17	34	19124508	19124541
chr12_3461	p3	(TGG)7	21	19128355	19128375
chr12_3462	p2	(TG)6	12	19130123	19130134
chr12_3463	p2	(CA)11	22	19141788	19141809
chr12_3464	p2	(CT)8	16	19152326	19152341
chr12_3465	p3	(ATT)6	18	19162706	19162723
chr12_3466	p2	(AC)17	34	19164519	19164552
chr12_3467	p2	(TG)24	48	19165516	19165563
chr12_3468	p2	(AC)7	14	19169724	19169737
chr12_3469	p3	(CTC)6	18	19175373	19175390
chr12_3470	c	ggggaaaccccagggctgctccaccatctgtagctgctcagtaatactgca	130	19176089	19176218
chr12_3471	p2	(AC)16	32	19176546	19176577
chr12_3472	c	ggcagggagagtatccttgggtgtgggtgtggacgagatggggatgtgtg	91	19177029	19177119
chr12_3473	p4	(CCCT)6	24	19177469	19177492
chr12_3474	p2	(CT)7	14	19180003	19180016
chr12_3475	c	(AC)11aa(AC)6	36	19192007	19192042
chr12_3476	p2	(AC)11	22	19195994	19196015
chr12_3477	p2	(TA)7	14	19212090	19212103
chr12_3478	p2	(CA)9	18	19218187	19218204
chr12_3479	p3	(CAT)6	18	19224922	19224939
chr12_3480	p2	(AT)6	12	19230040	19230051
chr12_3481	c	(TC)7(TG)8	30	19235994	19236023
chr12_3482	p2	(AC)7	14	19240960	19240973
chr12_3483	p4	(AAAT)5	20	19247159	19247178
chr12_3484	p4	(AATA)6	24	19250393	19250416
chr12_3485	p2	(TG)6	12	19250911	19250922
chr12_3486	p3	(TGC)7	21	19254654	19254674
chr12_3487	c	(TC)10tt(TA)7(TG)8(TA)6	64	19256507	19256570
chr12_3488	p4	(TAGC)5	20	19268527	19268546
chr12_3489	p3	(TGG)8	24	19275425	19275448
chr12_3490	c	(AC)9tttctttacaagccgatttttgcatcttcaacac(CA)15	82	19277190	19277271
chr12_3491	p2	(AC)8	16	19278175	19278190
chr12_3492	p2	(AT)9	18	19278392	19278409
chr12_3493	p2	(CA)10	20	19279360	19279379
chr12_3494	p2	(AG)6	12	19280304	19280315
chr12_3495	p2	(AC)6	12	19283272	19283283
chr12_3496	p3	(CTG)7	21	19284354	19284374
chr12_3497	p2	(GT)6	12	19285183	19285194

chr12_3498	p2	(GT)6	12	19289968	19289979
chr12_3499	c	(CT)6cact(CA)8	32	19290868	19290899
chr12_3500	p2	(TG)32	64	19306185	19306248
chr12_3501	p2	(TC)6	12	19307844	19307855
chr12_3502	p2	(AC)6	12	19309588	19309599
chr12_3503	p2	(AC)14	28	19311829	19311856
chr12_3504	p2	(CT)6	12	19316911	19316922
chr12_3505	p2	(AT)8	16	19317320	19317335
chr12_3506	p2	(AG)11	22	19319089	19319110
chr12_3507	p2	(AC)6	12	19321424	19321435
chr12_3508	p3	(ATA)5	15	19323610	19323624
chr12_3509	p2	(AC)7	14	19326234	19326247
chr12_3510	p4	(TATT)6	24	19327491	19327514
chr12_3511	p2	(GT)16	32	19328781	19328812
chr12_3512	c	(AG)6aacgaa(AG)6	30	19328951	19328980
chr12_3513	p2	(AC)14	28	19329453	19329480
chr12_3514	p2	(TG)6	12	19330740	19330751
chr12_3515	p2	(CA)15	30	19330949	19330978
chr12_3516	c	attgttattctgcacacagctgtggtcacagacagggcaaggcaggagtgc	150	19331757	19331906
chr12_3517	c	agagcaacatctgccgttctttgagactctttctttcaaagcagacttcctcct	135	19332029	19332163
chr12_3518	p2	(AC)15	30	19342329	19342358
chr12_3519	p2	(TG)15	30	19342627	19342656
chr12_3520	p3	(ATT)9	27	19353989	19354015
chr12_3521	p2	(GT)18	36	19354220	19354255
chr12_3522	p2	(AC)15	30	19355008	19355037
chr12_3523	p2	(GA)7	14	19355249	19355262
chr12_3524	p2	(TG)9	18	19355390	19355407
chr12_3525	c	gtttgctacatgtcgttggtgaatacacacacacatacagtagatgggttggg	158	19357090	19357247
chr12_3526	p2	(TG)6	12	19360638	19360649
chr12_3527	p2	(GT)6	12	19362842	19362853
chr12_3528	p2	(AG)8	16	19367052	19367067
chr12_3529	p2	(GT)6	12	19369876	19369887
chr12_3530	p2	(AC)6	12	19370782	19370793
chr12_3531	p2	(AC)8	16	19370947	19370962
chr12_3532	p2	(GA)20	40	19372654	19372693
chr12_3533	p2	(AG)7	14	19375445	19375458
chr12_3534	c	ccactgaagagatg(CA)10aagaatggggaggagagaacagagatct	230	19378452	19378681
chr12_3535	p2	(TA)12	24	19378902	19378925
chr12_3536	p2	(CA)6	12	19379163	19379174
chr12_3537	p2	(GT)8	16	19383467	19383482
chr12_3538	c	gtgttaggcagacagaggcaaacaggagagacgggaagtgtgtgttaaa	95	19384423	19384517
chr12_3539	p3	(TCC)5	15	19386153	19386167
chr12_3540	p2	(GA)9	18	19386776	19386793
chr12_3541	p2	(GT)18	36	19388186	19388221
chr12_3542	p2	(CA)8	16	19395746	19395761
chr12_3543	p2	(AG)6	12	19398632	19398643
chr12_3544	c	(TG)7(TA)7a(AT)6(AG)8	57	19407387	19407443
chr12_3545	p2	(TG)6	12	19407675	19407686
chr12_3546	c	tgtcacaatcaccgctatcgacattgctagcatcgttgttatcaccacacatt	108	19408095	19408202
chr12_3547	p2	(CA)19	38	19411098	19411135
chr12_3548	p2	(GT)13	26	19411280	19411305
chr12_3549	p3	(AAT)5	15	19411910	19411924

chr12_3550	p2	(TG)15	30	19412152	19412181
chr12_3551	p2	(TG)21	42	19412479	19412520
chr12_3552	p2	(AC)6	12	19413125	19413136
chr12_3553	p2	(AC)7	14	19414476	19414489
chr12_3554	p2	(GT)14	28	19415389	19415416
chr12_3555	p2	(TG)7	14	19416071	19416084
chr12_3556	p2	(GA)20	40	19417911	19417950
chr12_3557	p3	(TGC)9	27	19418211	19418237
chr12_3558	p2	(TA)7	14	19419561	19419574
chr12_3559	p2	(TG)8	16	19423070	19423085
chr12_3560	p2	(AC)15	30	19423938	19423967
chr12_3561	c	(CA)8cgtg(CA)36	92	19424082	19424173
chr12_3562	c	(AT)7(GT)12	38	19426680	19426717
chr12_3563	p2	(GT)7	14	19428102	19428115
chr12_3564	p2	(GT)14	28	19433011	19433038
chr12_3565	p2	(AC)15	30	19433189	19433218
chr12_3566	p2	(AG)11	22	19433647	19433668
chr12_3567	p2	(CA)14	28	19434219	19434246
chr12_3568	p2	(GT)7	14	19434895	19434908
chr12_3569	p2	(GT)6	12	19439296	19439307
chr12_3570	p2	(AG)6	12	19447964	19447975
chr12_3571	p2	(CA)6	12	19449812	19449823
chr12_3572	p2	(CA)19	38	19449967	19450004
chr12_3573	p2	(TG)15	30	19453560	19453589
chr12_3574	p2	(TG)6	12	19457802	19457813
chr12_3575	p2	(CA)11	22	19457924	19457945
chr12_3576	p2	(AC)21	42	19458306	19458347
chr12_3577	p2	(CT)6	12	19459186	19459197
chr12_3578	c	tcagacagtgtgaaagtagaccagagcaaaatgagctggccccttactgaca	122	19462171	19462292
chr12_3579	p2	(AT)7	14	19467801	19467814
chr12_3580	c*	(CA)7gacag(AC)8(CAC)7*	53	19468181	19468233
chr12_3581	p2	(TG)10	20	19471035	19471054
chr12_3582	c	(AC)20t(GA)6	53	19471166	19471218
chr12_3583	p2	(CA)14	28	19471469	19471496
chr12_3584	p2	(GA)11	22	19473022	19473043
chr12_3585	p4	(TCAA)5	20	19473991	19474010
chr12_3586	p2	(CA)10	20	19475615	19475634
chr12_3587	p2	(GT)9	18	19476267	19476284
chr12_3588	p2	(CA)6	12	19480729	19480740
chr12_3589	p2	(AC)6	12	19480933	19480944
chr12_3590	p3	(TCC)5	15	19496342	19496356
chr12_3591	p2	(AC)10	20	19498033	19498052
chr12_3592	p2	(AC)7	14	19500755	19500768
chr12_3593	p2	(GA)6	12	19503480	19503491
chr12_3594	p2	(GT)6	12	19504968	19504979
chr12_3595	p2	(GT)20	40	19508140	19508179
chr12_3596	p2	(AC)26	52	19519880	19519931
chr12_3597	p2	(AC)17	34	19521933	19521966
chr12_3598	p2	(AG)8	16	19522633	19522648
chr12_3599	c	tgctttcattcattctctcccccagtttaacagttcagtgttcagaaggagcata	113	19523762	19523874
chr12_3600	p2	(CA)6	12	19526148	19526159
chr12_3601	c	(AC)7gcacacacc(CA)6	35	19528902	19528936

chr12_3602	p2	(TC)8	16	19529490	19529505
chr12_3603	c	(AC)13gcg(CA)11	51	19532535	19532585
chr12_3604	p2	(AG)6	12	19536287	19536298
chr12_3605	c	(GGT)6(GAT)6	36	19543406	19543441
chr12_3606	p3	(TCA)7	21	19549913	19549933
chr12_3607	p2	(TG)12	24	19550130	19550153
chr12_3608	p5	(AAATC)5	25	19557573	19557597
chr12_3609	c	(AC)9agagtcaa(TG)6	38	19565513	19565550
chr12_3610	p3	(TTA)6	18	19568117	19568134
chr12_3611	p3	(TTC)5	15	19575352	19575366
chr12_3612	p2	(AC)6	12	19575606	19575617
chr12_3613	c	(GAACA)14(AAACA)27	205	19578509	19578713
chr12_3614	p2	(GT)8	16	19580220	19580235
chr12_3615	p2	(TG)6	12	19581738	19581749
chr12_3616	p2	(GT)6	12	19589824	19589835
chr12_3617	c	(AGT)5(AAT)6	33	19596115	19596147
chr12_3618	p2	(CT)12	24	19597959	19597982
chr12_3619	p2	(TG)6	12	19601401	19601412
chr12_3620	p2	(CA)9	18	19603938	19603955
chr12_3621	c	(CA)7cg(CA)8	32	19604155	19604186
chr12_3622	p2	(AC)18	36	19606169	19606204
chr12_3623	p2	(AC)8	16	19608159	19608174
chr12_3624	p2	(GT)7	14	19608726	19608739
chr12_3625	p2	(AC)6	12	19610813	19610824
chr12_3626	p2	(TC)6	12	19615144	19615155
chr12_3627	p2	(GT)6	12	19632969	19632980
chr12_3628	p3	(TAA)6	18	19633958	19633975
chr12_3629	p2	(TA)6	12	19636682	19636693
chr12_3630	p2	(CA)6	12	19637074	19637085
chr12_3631	p2	(CA)17	34	19645599	19645632
chr12_3632	p2	(TA)8	16	19646497	19646512
chr12_3633	p2	(TA)6	12	19651377	19651388
chr12_3634	p3	(TGG)5	15	19652509	19652523
chr12_3635	p2	(TG)6	12	19655202	19655213
chr12_3636	p2	(CA)10	20	19655912	19655931
chr12_3637	p2	(AC)15	30	19656057	19656086
chr12_3638	p2	(AC)8	16	19656692	19656707
chr12_3639	c	(CA)6cg(CA)6	26	19661533	19661558
chr12_3640	p2	(CA)9	18	19667616	19667633
chr12_3641	p2	(GT)22	44	19669377	19669420
chr12_3642	p2	(TG)6	12	19673059	19673070
chr12_3643	c	cacatgttcagtcttaacctctaacagtcccttgtgaactccctgatgatgcat	132	19674765	19674896
chr12_3644	p2	(CA)15	30	19677272	19677301
chr12_3645	p2	(AC)11	22	19684176	19684197
chr12_3646	p2	(GT)19	38	19688648	19688685
chr12_3647	p2	(CT)7	14	19691451	19691464
chr12_3648	c	gtaaatgttgttgcaggaatgaagtcatctgacatttaagaatcagccatcca	123	19693569	19693691
chr12_3649	p2	(AC)6	12	19701742	19701753
chr12_3650	p2	(TG)18	36	19706291	19706326
chr12_3651	p2	(GT)12	24	19707953	19707976
chr12_3652	p2	(TG)13	26	19708331	19708356
chr12_3653	p2	(TG)13	26	19708512	19708537

chr12_3654	p2	(AC)27	54	19711592	19711645
chr12_3655	p2	(AT)6	12	19713731	19713742
chr12_3656	p2	(GT)6	12	19714600	19714611
chr12_3657	p2	(CA)13	26	19716197	19716222
chr12_3658	p2	(GT)14	28	19724018	19724045
chr12_3659	c	(CA)10cg(CA)11	44	19728080	19728123
chr12_3660	c	(GA)6ggacaatggaggc(AG)9	43	19730225	19730267
chr12_3661	p2	(AC)9	18	19732491	19732508
chr12_3662	p2	(TG)7	14	19748684	19748697
chr12_3663	p3	(ATA)5	15	19755314	19755328
chr12_3664	p2	(CT)6	12	19757097	19757108
chr12_3665	p2	(AC)6	12	19757896	19757907
chr12_3666	p2	(AC)6	12	19760138	19760149
chr12_3667	p3	(TTG)6	18	19761248	19761265
chr12_3668	p2	(AG)17	34	19761934	19761967
chr12_3669	p2	(AC)6	12	19765122	19765133
chr12_3670	p2	(TC)7	14	19765942	19765955
chr12_3671	p2	(GA)7	14	19770724	19770737
chr12_3672	p3	(AAT)5	15	19771689	19771703
chr12_3673	p2	(TC)7	14	19780429	19780442
chr12_3674	p2	(CA)7	14	19782507	19782520
chr12_3675	p2	(CA)7	14	19782633	19782646
chr12_3676	p2	(TC)8	16	19785397	19785412
chr12_3677	p3	(CAA)11	33	19792067	19792099
chr12_3678	p2	(CA)9	18	19793804	19793821
chr12_3679	p2	(CA)17	34	19795995	19796028
chr12_3680	c	(GA)12aagg(GA)8	44	19796228	19796271
chr12_3681	p2	(AC)18	36	19800231	19800266
chr12_3682	p3	(CAA)5	15	19809932	19809946
chr12_3683	p3	(GAG)9	27	19816160	19816186
chr12_3684	p2	(AC)23	46	19816650	19816695
chr12_3685	p2	(AC)17	34	19819052	19819085
chr12_3686	p2	(AC)16	32	19821310	19821341
chr12_3687	p2	(AC)8	16	19823526	19823541
chr12_3688	p2	(TG)10	20	19824142	19824161
chr12_3689	p2	(TG)7	14	19825037	19825050
chr12_3690	p2	(CA)13	26	19826931	19826956
chr12_3691	p2	(AC)6	12	19828280	19828291
chr12_3692	p2	(GT)12	24	19829969	19829992
chr12_3693	p2	(CA)9	18	19833433	19833450
chr12_3694	c	ttctgtcaggactttttaacaagcagccgagtccccacagggaaacactga	112	19833568	19833679
chr12_3695	p2	(CA)9	18	19840266	19840283
chr12_3696	p2	(AC)19	38	19840392	19840429
chr12_3697	p2	(AG)6	12	19840878	19840889
chr12_3698	p2	(CT)6	12	19845958	19845969
chr12_3699	c	tcaacataattatagagggatacgaagaatcaccttgacttggcac(TG)2	257	19847450	19847706
chr12_3700	p2	(AC)12	24	19848477	19848500
chr12_3701	p2	(AC)7	14	19848890	19848903
chr12_3702	c	(GT)11cagagagaa(AG)6g(GA)6	56	19849098	19849153
chr12_3703	p2	(TC)7	14	19849716	19849729
chr12_3704	p2	(AC)6	12	19851166	19851177
chr12_3705	p2	(AC)6	12	19852016	19852027

chr12_3706	p3	(TAT)7	21	19853053	19853073
chr12_3707	p2	(GA)6	12	19854474	19854485
chr12_3708	c	(TC)6(AC)12	36	19854675	19854710
chr12_3709	p2	(CA)7	14	19854850	19854863
chr12_3710	p2	(GT)9	18	19858077	19858094
chr12_3711	p2	(TG)9	18	19864446	19864463
chr12_3712	p2	(GT)11	22	19864622	19864643
chr12_3713	p2	(AT)17	34	19866401	19866434
chr12_3714	p2	(AG)8	16	19867355	19867370
chr12_3715	p2	(GT)10	20	19871320	19871339
chr12_3716	p2	(CA)11	22	19878995	19879016
chr12_3717	p2	(CA)13	26	19881048	19881073
chr12_3718	p2	(AC)12	24	19881188	19881211
chr12_3719	p2	(CA)6	12	19881502	19881513
chr12_3720	p2	(AC)10	20	19882044	19882063
chr12_3721	p2	(CA)11	22	19883845	19883866
chr12_3722	c	acaacgcacaccacatctgaaagaataggagtaggaaccttgaaaacaac	124	19884020	19884143
chr12_3723	p2	(TG)6	12	19886423	19886434
chr12_3724	p2	(TG)15	30	19891428	19891457
chr12_3725	p2	(TG)9	18	19891599	19891616
chr12_3726	p2	(AC)15	30	19892949	19892978
chr12_3727	p2	(TG)6	12	19894957	19894968
chr12_3728	p2	(AC)8	16	19896019	19896034
chr12_3729	c	(GT)7ata(TG)18	53	19898098	19898150
chr12_3730	p3	(CTG)5	15	19902422	19902436
chr12_3731	c	(AC)6(AG)16	44	19903012	19903055
chr12_3732	p2	(CA)7	14	19904783	19904796
chr12_3733	c	(AC)8gc(AG)6	30	19904989	19905018
chr12_3734	p2	(CA)7	14	19909862	19909875
chr12_3735	p2	(CA)15	30	19910074	19910103
chr12_3736	p2	(GT)10	20	19924693	19924712
chr12_3737	p2	(TG)10	20	19925804	19925823
chr12_3738	p2	(AC)11	22	19932632	19932653
chr12_3739	p2	(TG)7	14	19946760	19946773
chr12_3740	p2	(TG)11	22	19951276	19951297
chr12_3741	p2	(AG)6	12	19959908	19959919
chr12_3742	p2	(TG)9	18	19960802	19960819
chr12_3743	p2	(CA)6	12	19964191	19964202
chr12_3744	p2	(AC)21	42	19965747	19965788
chr12_3745	p2	(AC)6	12	19966003	19966014
chr12_3746	p2	(GT)6	12	19977472	19977483
chr12_3747	p2	(CT)6	12	19984211	19984222
chr12_3748	p2	(GT)11	22	19988876	19988897
chr12_3749	p2	(CA)6	12	19989164	19989175
chr12_3750	p2	(TG)12	24	19990866	19990889
chr12_3751	p2	(CA)16	32	19994639	19994670
chr12_3752	p2	(AC)15	30	19997570	19997599
chr12_3753	p2	(TG)7	14	19997973	19997986
chr12_3754	c	cattactcctaaagagaggaactgcaagagggctgtcatttcattaacctca	138	19998949	19999086
chr12_3755	p2	(AC)7	14	20000582	20000595
chr12_3756	p2	(CT)6	12	20001305	20001316
chr12_3757	p3	(AAC)5	15	20001880	20001894

chr12_3758	p2	(CA)7	14	20006027	20006040
chr12_3759	p2	(AC)12	24	20006242	20006265
chr12_3760	p2	(CA)8	16	20006930	20006945
chr12_3761	p4	(CATC)7	28	20018618	20018645
chr12_3762	p3	(AAT)6	18	20021161	20021178
chr12_3763	c	[illegible]cagctataggatgaaatctgtcatgctgagtccactcagtcatactcactgg	148	20024748	20024895
chr12_3764	p2	(CA)18	36	20026882	20026917
chr12_3765	p4	(AGAT)18	72	20028037	20028108
chr12_3766	c	(CT)6tacattttggcatcaaacaca(CT)6	45	20028430	20028474
chr12_3767	p2	(GT)6	12	20028598	20028609
chr12_3768	p2	(GT)6	12	20029082	20029093
chr12_3769	p2	(AC)25	50	20029228	20029277
chr12_3770	p2	(TG)6	12	20030905	20030916
chr12_3771	p2	(AC)9	18	20036855	20036872
chr12_3772	p2	(AC)12	24	20044318	20044341
chr12_3773	p2	(AC)10	20	20045767	20045786
chr12_3774	p3	(AGA)6	18	20048649	20048666
chr12_3775	p2	(TC)6	12	20048854	20048865
chr12_3776	p2	(GT)10	20	20052697	20052716
chr12_3777	p2	(CA)23	46	20053316	20053361
chr12_3778	p2	(CA)7	14	20056262	20056275
chr12_3779	p2	(GA)8	16	20056401	20056416
chr12_3780	p2	(TG)11	22	20057720	20057741
chr12_3781	p2	(TC)7	14	20057893	20057906
chr12_3782	p2	(CA)11	22	20065656	20065677
chr12_3783	p2	(TG)6	12	20066080	20066091
chr12_3784	c	(AC)8g(CT)10	37	20069277	20069313
chr12_3785	p2	(CA)6	12	20070122	20070133
chr12_3786	p2	(AC)7	14	20071996	20072009
chr12_3787	p2	(CA)12	24	20072927	20072950
chr12_3788	p2	(GT)8	16	20075002	20075017
chr12_3789	p2	(CT)9	18	20076569	20076586
chr12_3790	p2	(AC)9	18	20078754	20078771
chr12_3791	p2	(GT)18	36	20079293	20079328
chr12_3792	p2	(GA)6	12	20080635	20080646
chr12_3793	p3	(ATA)6	18	20083413	20083430
chr12_3794	c	(CA)6cg(CA)8	30	20085878	20085907
chr12_3795	p2	(GT)14	28	20086934	20086961
chr12_3796	p4	(TATC)6	24	20099382	20099405
chr12_3797	c	tttcacccgacagtaatagcctgcatcagcgtcattatgggcaatatttcatg	104	20103988	20104091
chr12_3798	p2	(TG)8	16	20109101	20109116
chr12_3799	p2	(TG)7	14	20111369	20111382
chr12_3800	p3	(AGT)11	33	20117668	20117700
chr12_3801	p2	(TG)6	12	20120035	20120046
chr12_3802	p2	(GT)9	18	20127086	20127103
chr12_3803	p2	(AC)13	26	20133309	20133334
chr12_3804	c	(TCT)5tcc(TCT)5	33	20139544	20139576
chr12_3805	p3	(CTT)5	15	20143697	20143711
chr12_3806	p2	(AC)11	22	20145830	20145851
chr12_3807	p2	(AC)6	12	20146277	20146288
chr12_3808	p2	(CA)10	20	20146422	20146441
chr12_3809	p2	(CA)14	28	20149586	20149613

chr12_3810	p2	(TC)6	12	20154060	20154071
chr12_3811	p2	(GA)8	16	20157508	20157523
chr12_3812	p2	(GA)8	16	20164887	20164902
chr12_3813	p2	(TG)6	12	20166341	20166352
chr12_3814	p2	(TG)7	14	20170366	20170379
chr12_3815	p2	(AC)13	26	20170949	20170974
chr12_3816	p2	(AC)6	12	20177038	20177049
chr12_3817	p3	(TCC)6	18	20180935	20180952
chr12_3818	p2	(CA)6	12	20181363	20181374
chr12_3819	p3	(CTG)5	15	20181572	20181586
chr12_3820	p4	(TAGC)6	24	20184436	20184459
chr12_3821	p3	(TAT)6	18	20218715	20218732
chr12_3822	p2	(CA)6	12	20239031	20239042
chr12_3823	p2	(GT)6	12	20246940	20246951
chr12_3824	p2	(TG)7	14	20251984	20251997
chr12_3825	p2	(CA)6	12	20266369	20266380
chr12_3826	p2	(GT)20	40	20270016	20270055
chr12_3827	p3	(ATT)5	15	20280648	20280662
chr12_3828	p2	(TG)6	12	20282022	20282033
chr12_3829	p2	(TG)13	26	20283483	20283508
chr12_3830	p2	(AC)8	16	20294654	20294669
chr12_3831	p2	(CA)14	28	20297594	20297621
chr12_3832	c	10tgtggttggaattcactgagttctcaatattcttctcaaattaaatggacc(	89	20313689	20313777
chr12_3833	p2	(TG)10	20	20318409	20318428
chr12_3834	p3	(CAG)6	18	20322740	20322757
chr12_3835	p2	(TG)6	12	20322998	20323009
chr12_3836	p2	(TG)7	14	20329081	20329094
chr12_3837	p2	(TC)7	14	20332323	20332336
chr12_3838	p2	(GT)7	14	20337925	20337938
chr12_3839	p2	(GT)7	14	20355108	20355121
chr12_3840	p2	(TG)6	12	20358574	20358585
chr12_3841	c	ıtcgttgtacttagtcagcaaaggtgtatttggatcgatggagctttgcaaagt	105	20362898	20363002
chr12_3842	p2	(CA)14	28	20378337	20378364
chr12_3843	p2	(TG)18	36	20383509	20383544
chr12_3844	p2	(GT)7	14	20387407	20387420
chr12_3845	p2	(GA)11	22	20388590	20388611
chr12_3846	p2	(TG)8	16	20390936	20390951
chr12_3847	p2	(TG)7	14	20393652	20393665
chr12_3848	p2	(TG)16	32	20395740	20395771
chr12_3849	p2	(TG)6	12	20403503	20403514
chr12_3850	p2	(TG)21	42	20406600	20406641
chr12_3851	p2	(CA)9	18	20416676	20416693
chr12_3852	p3	(TTG)5	15	20417954	20417968
chr12_3853	p2	(GT)11	22	20418388	20418409
chr12_3854	p4	(ATGG)6	24	20419046	20419069
chr12_3855	p2	(TG)15	30	20420752	20420781
chr12_3856	p2	(GA)7	14	20423018	20423031
chr12_3857	p3	(AAT)5	15	20429790	20429804
chr12_3858	p2	(CA)10	20	20431518	20431537
chr12_3859	p2	(CA)8	16	20432884	20432899
chr12_3860	p2	(TG)7	14	20434840	20434853
chr12_3861	p3	(CTG)5	15	20443162	20443176

chr12_3862	p2	(GT)7	14	20447599	20447612
chr12_3863	p2	(CA)11	22	20450250	20450271
chr12_3864	p3	(ACC)7	21	20454230	20454250
chr12_3865	p2	(AC)6	12	20456405	20456416
chr12_3866	p2	(AC)6	12	20463229	20463240
chr12_3867	p2	(TC)7	14	20473954	20473967
chr12_3868	p2	(CA)14	28	20486349	20486376
chr12_3869	p2	(TA)6	12	20487347	20487358
chr12_3870	p3	(GAG)5	15	20488542	20488556
chr12_3871	p2	(CA)7	14	20489517	20489530
chr12_3872	p3	(CTG)5	15	20492898	20492912
chr12_3873	p3	(CCT)5	15	20493432	20493446
chr12_3874	c	(TG)6tt(AG)9	32	20511616	20511647
chr12_3875	p2	(CA)6	12	20547896	20547907
chr12_3876	p2	(AC)10	20	20549198	20549217
chr12_3877	p3	(GAG)7	21	20551709	20551729
chr12_3878	p2	(AC)6	12	20554297	20554308
chr12_3879	p2	(GT)18	36	20554462	20554497
chr12_3880	p2	(GT)12	24	20555797	20555820
chr12_3881	p2	(TG)11	22	20556719	20556740
chr12_3882	p3	(GGT)7	21	20557696	20557716
chr12_3883	p2	(TG)8	16	20570041	20570056
chr12_3884	p2	(CA)16	32	20571061	20571092
chr12_3885	p2	(AC)6	12	20571218	20571229
chr12_3886	p2	(TG)7	14	20575435	20575448
chr12_3887	p2	(TG)11	22	20578236	20578257
chr12_3888	p2	(AC)15	30	20581142	20581171
chr12_3889	p2	(TA)11	22	20583176	20583197
chr12_3890	p2	(TC)6	12	20595406	20595417
chr12_3891	p2	(AC)7	14	20595647	20595660
chr12_3892	p2	(GT)6	12	20595934	20595945
chr12_3893	p2	(CA)7	14	20622000	20622013
chr12_3894	p2	(TG)9	18	20623059	20623076
chr12_3895	p3	(GCT)5	15	20635936	20635950
chr12_3896	p2	(GT)6	12	20641034	20641045
chr12_3897	p2	(TG)15	30	20642250	20642279
chr12_3898	p2	(TG)16	32	20643804	20643835
chr12_3899	p2	(GT)13	26	20644669	20644694
chr12_3900	p2	(TG)8	16	20646501	20646516
chr12_3901	p2	(AC)10	20	20648823	20648842
chr12_3902	p2	(GT)14	28	20653401	20653428
chr12_3903	p2	(TA)6	12	20659067	20659078
chr12_3904	p3	(TTG)7	21	20659382	20659402
chr12_3905	p2	(TG)6	12	20669199	20669210
chr12_3906	c	(AAT)6tattattattataac(AAT)7	54	20676069	20676122
chr12_3907	c	(TA)8(CA)7	30	20678077	20678106
chr12_3908	p2	(GC)6	12	20688432	20688443
chr12_3909	p2	(AC)8	16	20701619	20701634
chr12_3910	p2	(CA)10	20	20716194	20716213
chr12_3911	p2	(GT)12	24	20720777	20720800
chr12_3912	p2	(TG)10	20	20721884	20721903
chr12_3913	p3	(ATT)5	15	20764960	20764974

chr12_3914	c	(ATCT)31(GTCT)6atctatctatctat(AC)14	190	20766121	20766310
chr12_3915	p2	(TG)6	12	20778467	20778478
chr12_3916	p2	(CA)6	12	20784294	20784305
chr12_3917	p2	(AC)6	12	20804136	20804147
chr12_3918	p2	(CA)9	18	20812058	20812075
chr12_3919	p2	(TC)7	14	20831790	20831803
chr12_3920	p2	(AC)8	16	20837613	20837628
chr12_3921	p2	(TG)8	16	20845653	20845668
chr12_3922	p2	(CA)10	20	20847132	20847151
chr12_3923	p2	(AC)12	24	20847324	20847347
chr12_3924	p3	(CTC)5	15	20847614	20847628
chr12_3925	c	(CT)6cacacacataagcaacgactt(TC)7	47	20852263	20852309
chr12_3926	p2	(AC)13	26	20855629	20855654
chr12_3927	p3	(AAC)8	24	20858007	20858030
chr12_3928	p2	(CT)8	16	20858713	20858728
chr12_3929	p2	(CA)6	12	20859513	20859524
chr12_3930	p3	(GTA)10	30	20861260	20861289
chr12_3931	p2	(CA)8	16	20870236	20870251
chr12_3932	p2	(TG)16	32	20883027	20883058
chr12_3933	p2	(CA)10	20	20885370	20885389
chr12_3934	p2	(AC)20	40	20912372	20912411
chr12_3935	p3	(TCT)5	15	20923272	20923286
chr12_3936	p2	(TG)16	32	20926379	20926410
chr12_3937	p2	(AG)11	22	20927983	20928004
chr12_3938	p2	(AT)9	18	20932541	20932558
chr12_3939	p3	(GAT)5	15	20935700	20935714
chr12_3940	p4	(AAAG)15	60	20942029	20942088
chr12_3941	p2	(AG)10	20	20946361	20946380
chr12_3942	p3	(ATA)5	15	20952883	20952897
chr12_3943	p2	(TG)6	12	20953139	20953150
chr12_3944	p2	(CA)7	14	20960388	20960401
chr12_3945	p2	(GT)7	14	20971449	20971462
chr12_3946	p3	(TTG)5	15	20978929	20978943
chr12_3947	p3	(TTG)5	15	20989237	20989251
chr12_3948	p2	(AG)7	14	20990719	20990732
chr12_3949	p2	(CA)6	12	20991344	20991355
chr12_3950	p2	(GT)6	12	20992850	20992861
chr12_3951	p2	(CA)6	12	21000336	21000347
chr12_3952	p2	(TG)20	40	21002470	21002509
chr12_3953	p3	(ACA)5	15	21022676	21022690
chr12_3954	c	(GT)7gcacaagtcatgcagtcagcttttgctagcctggccttcg(GT)1[illegible]	74	21049199	21049272
chr12_3955	p2	(GT)21	42	21051732	21051773
chr12_3956	p2	(AC)8	16	21053917	21053932
chr12_3957	p2	(GT)7	14	21054495	21054508
chr12_3958	p2	(AG)6	12	21064998	21065009
chr12_3959	p2	(GT)9	18	21078258	21078275
chr12_3960	p2	(TG)6	12	21079287	21079298
chr12_3961	p2	(AC)6	12	21084074	21084085
chr12_3962	p2	(AG)10	20	21092041	21092060
chr12_3963	p2	(GT)6	12	21092231	21092242
chr12_3964	p2	(AC)12	24	21098376	21098399
chr12_3965	p2	(CT)7	14	21106856	21106869

chr12_3966	p2	(TG)8	16	21107892	21107907
chr12_3967	c	(GA)9(GT)10	38	21108814	21108851
chr12_3968	p2	(CT)6	12	21111216	21111227
chr12_3969	p4	(TTGA)7	28	21114006	21114033
chr12_3970	p3	(GCT)6	18	21120986	21121003
chr12_3971	p3	(GCT)6	18	21121434	21121451
chr12_3972	p2	(TG)17	34	21123787	21123820
chr12_3973	p3	(AAG)5	15	21126694	21126708
chr12_3974	p3	(CAG)7	21	21130723	21130743
chr12_3975	p2	(AC)24	48	21134181	21134228
chr12_3976	c	(AC)8at(AC)6	30	21134838	21134867
chr12_3977	p2	(TG)7	14	21137981	21137994
chr12_3978	p2	(AC)18	36	21159545	21159580
chr12_3979	p4	(CATT)5	20	21161454	21161473
chr12_3980	p2	(CA)11	22	21164589	21164610
chr12_3981	p2	(AC)6	12	21186937	21186948
chr12_3982	p2	(TA)6	12	21193839	21193850
chr12_3983	p2	(GT)17	34	21197810	21197843
chr12_3984	p2	(GT)7	14	21198950	21198963
chr12_3985	p2	(CA)8	16	21224992	21225007
chr12_3986	p2	(AC)8	16	21231376	21231391
chr12_3987	p3	(GTT)7	21	21233483	21233503
chr12_3988	p2	(TG)12	24	21235845	21235868
chr12_3989	c	(AAT)5agtaatcgtgctacgagaaacaactggcagacttcg(AC)6	63	21241251	21241313
chr12_3990	p2	(AC)6	12	21261473	21261484
chr12_3991	p2	(TA)6	12	21261654	21261665
chr12_3992	p3	(CAC)7	21	21264956	21264976
chr12_3993	p2	(CA)7	14	21268412	21268425
chr12_3994	p2	(CA)7	14	21279615	21279628
chr12_3995	p2	(AC)6	12	21282624	21282635
chr12_3996	p2	(TG)21	42	21285120	21285161
chr12_3997	p2	(CT)10	20	21285521	21285540
chr12_3998	p2	(AC)10	20	21286460	21286479
chr12_3999	p2	(CA)6	12	21289763	21289774
chr12_4000	p2	(AG)6	12	21295770	21295781
chr12_4001	p2	(AG)6	12	21297829	21297840
chr12_4002	c	(CA)6taccttactgtacgtg(CA)7	42	21298402	21298443
chr12_4003	p2	(GT)6	12	21299711	21299722
chr12_4004	p3	(AGG)5	15	21302418	21302432
chr12_4005	p2	(TG)8	16	21304558	21304573
chr12_4006	p2	(TG)12	24	21306279	21306302
chr12_4007	p2	(TA)8	16	21310692	21310707
chr12_4008	c	(TC)7(AC)17	48	21317376	21317423
chr12_4009	c	[illegible]accagcttccccctgctgttctgattgcagcaaagatcagaccgaccaga[illegible]	101	21325538	21325638
chr12_4010	p2	(AC)11	22	21327331	21327352
chr12_4011	p2	(TG)20	40	21330320	21330359
chr12_4012	p2	(AC)8	16	21333123	21333138
chr12_4013	p2	(AC)6	12	21335696	21335707
chr12_4014	p3	(TAT)6	18	21346825	21346842
chr12_4015	p2	(AC)14	28	21352340	21352367
chr12_4016	p2	(AC)6	12	21352635	21352646
chr12_4017	p5	(CTAAG)6	30	21353712	21353741

chr12_4018	p2	(TG)6	12	21355403	21355414
chr12_4019	p2	(AC)6	12	21379079	21379090
chr12_4020	p2	(AG)6	12	21387516	21387527
chr12_4021	p2	(GA)8	16	21388064	21388079
chr12_4022	p2	(CA)7	14	21389289	21389302
chr12_4023	p3	(ATC)5	15	21390898	21390912
chr12_4024	p2	(CA)18	36	21391378	21391413
chr12_4025	p2	(CA)10	20	21393272	21393291
chr12_4026	p2	(CA)8	16	21396468	21396483
chr12_4027	p2	(TG)10	20	21409079	21409098
chr12_4028	p2	(GT)6	12	21455412	21455423
chr12_4029	p2	(CA)8	16	21458746	21458761
chr12_4030	p2	(CA)11	22	21459295	21459316
chr12_4031	p3	(TTA)5	15	21461724	21461738
chr12_4032	p3	(ATT)6	18	21464682	21464699
chr12_4033	p2	(TA)6	12	21470575	21470586
chr12_4034	p2	(TA)6	12	21472512	21472523
chr12_4035	p2	(CT)7	14	21475253	21475266
chr12_4036	p2	(GA)6	12	21479355	21479366
chr12_4037	c	(TC)10cctctctctct(CA)6	43	21483983	21484025
chr12_4038	p2	(CT)8	16	21503824	21503839
chr12_4039	p2	(AC)6	12	21505988	21505999
chr12_4040	p3	(ACA)8	24	21510423	21510446
chr12_4041	p3	(GCT)5	15	21518850	21518864
chr12_4042	p2	(CA)13	26	21525164	21525189
chr12_4043	p2	(GT)6	12	21530048	21530059
chr12_4044	c	(AC)9(AG)6	30	21532674	21532703
chr12_4045	c	(TG)10agtgtgt(GA)6	39	21538436	21538474
chr12_4046	p2	(AC)19	38	21549357	21549394
chr12_4047	p2	(AC)6	12	21554955	21554966
chr12_4048	p3	(GAT)5	15	21571032	21571046
chr12_4049	p4	(GATA)20	80	21571879	21571958
chr12_4050	p2	(TG)9	18	21579851	21579868
chr12_4051	p2	(GT)7	14	21580242	21580255
chr12_4052	p2	(TG)6	12	21585722	21585733
chr12_4053	p3	(CAG)5	15	21587335	21587349
chr12_4054	p2	(GA)9	18	21588886	21588903
chr12_4055	p3	(CCT)7	21	21592930	21592950
chr12_4056	p2	(GT)8	16	21599699	21599714
chr12_4057	p2	(CA)8	16	21601773	21601788
chr12_4058	p2	(TC)6	12	21603010	21603021
chr12_4059	p5	(TGATT)6	30	21624878	21624907
chr12_4060	p3	(ATA)8	24	21637167	21637190
chr12_4061	p2	(TG)10	20	21680420	21680439
chr12_4062	p3	(AAC)6	18	21684303	21684320
chr12_4063	p2	(CA)9	18	21685417	21685434
chr12_4064	p2	(AT)9	18	21690925	21690942
chr12_4065	p2	(GT)6	12	21695681	21695692
chr12_4066	p3	(ACT)8	24	21722026	21722049
chr12_4067	p2	(GT)16	32	21731846	21731877
chr12_4068	p2	(CT)9	18	21732520	21732537
chr12_4069	p2	(CT)7	14	21740852	21740865

chr12_4070	p2	(TC)27	54	21743330	21743383
chr12_4071	p2	(AG)9	18	21752845	21752862
chr12_4072	p2	(GT)15	30	21753288	21753317
chr12_4073	p2	(TG)7	14	21753493	21753506
chr12_4074	p3	(GTT)9	27	21764973	21764999
chr12_4075	p3	(ATT)9	27	21767382	21767408
chr12_4076	p2	(AC)6	12	21768339	21768350
chr12_4077	p2	(GT)6	12	21773613	21773624
chr12_4078	c	atagtttatttttttgagcgatgcaaacatcatcccaggctattttggtctcgaa	103	21778339	21778441
chr12_4079	p3	(CTT)5	15	21779072	21779086
chr12_4080	p2	(TG)7	14	21781053	21781066
chr12_4081	p4	(ATAA)5	20	21786918	21786937
chr12_4082	p2	(GT)6	12	21793065	21793076
chr12_4083	p2	(TG)6	12	21795178	21795189
chr12_4084	p2	(AC)6	12	21795882	21795893
chr12_4085	p2	(GT)12	24	21796616	21796639
chr12_4086	p4	(TGGA)5	20	21801766	21801785
chr12_4087	c	(GT)12ctttccatgggc(CT)9	54	21807907	21807960
chr12_4088	p2	(AC)16	32	21808080	21808111
chr12_4089	c	(CT)19(GT)6	50	21808224	21808273
chr12_4090	p2	(CA)20	40	21811021	21811060
chr12_4091	c	(TG)26gtgtctctttgaagcgggtgactg(CA)14	104	21811202	21811305
chr12_4092	p2	(TC)6	12	21816544	21816555
chr12_4093	p2	(GT)6	12	21820647	21820658
chr12_4094	p2	(TG)9	18	21823884	21823901
chr12_4095	p4	(CCTA)5	20	21826732	21826751
chr12_4096	p3	(ATT)5	15	21833608	21833622
chr12_4097	p3	(TCA)6	18	21841018	21841035
chr12_4098	p2	(TG)9	18	21843861	21843878
chr12_4099	p2	(CA)17	34	21851607	21851640
chr12_4100	p3	(CTA)5	15	21859653	21859667
chr12_4101	p2	(CA)6	12	21864663	21864674
chr12_4102	p2	(CA)8	16	21869980	21869995
chr12_4103	p2	(TG)6	12	21871424	21871435
chr12_4104	p3	(TTA)6	18	21873244	21873261
chr12_4105	p2	(CT)13	26	21892102	21892127
chr12_4106	p2	(GT)10	20	21910308	21910327
chr12_4107	p2	(TG)13	26	21911854	21911879
chr12_4108	p2	(TG)10	20	21919666	21919685
chr12_4109	p3	(GCA)9	27	21922814	21922840
chr12_4110	p2	(AC)6	12	21923027	21923038
chr12_4111	p3	(AAG)5	15	21934873	21934887
chr12_4112	p2	(AC)10	20	21937544	21937563
chr12_4113	p4	(TGTT)5	20	21941303	21941322
chr12_4114	p2	(TC)6	12	21944056	21944067
chr12_4115	p2	(CA)6	12	21949032	21949043
chr12_4116	p3	(TTA)5	15	21954981	21954995
chr12_4117	p2	(TG)6	12	21955850	21955861
chr12_4118	p2	(TG)10	20	21956643	21956662
chr12_4119	p2	(TG)7	14	21956891	21956904
chr12_4120	p2	(TG)6	12	21959229	21959240
chr12_4121	p2	(CA)8	16	21963677	21963692

chr12_4122	p2	(AT)6	12	21975528	21975539
chr12_4123	c	(GT)7a(TG)6	27	21980385	21980411
chr12_4124	p2	(AC)9	18	21981626	21981643
chr12_4125	p2	(TG)6	12	21982981	21982992
chr12_4126	p2	(AC)6	12	21994818	21994829
chr12_4127	p2	(TG)9	18	21998398	21998415
chr12_4128	p2	(TA)6	12	22001467	22001478
chr12_4129	p2	(AC)8	16	22003110	22003125
chr12_4130	p2	(TA)6	12	22006012	22006023
chr12_4131	c	ccatcacacagtctttctctttctgctgaccagcagaaaccctgcacacctc	122	22008533	22008654
chr12_4132	p2	(TG)11	22	22010105	22010126
chr12_4133	p2	(GA)8	16	22012459	22012474
chr12_4134	p3	(TTG)13	39	22014850	22014888
chr12_4135	p2	(TG)6	12	22016285	22016296
chr12_4136	p3	(AAG)5	15	22019707	22019721
chr12_4137	p2	(CT)8	16	22020352	22020367
chr12_4138	p2	(TG)8	16	22021382	22021397
chr12_4139	p2	(TA)6	12	22023259	22023270
chr12_4140	c	atgacaggctgagagcagatgtccgtcaactctcccagtccagctaatta	123	22027515	22027637
chr12_4141	p2	(AT)6	12	22031735	22031746
chr12_4142	p3	(ATT)8	24	22035335	22035358
chr12_4143	p2	(AT)11	22	22037241	22037262
chr12_4144	p2	(AT)6	12	22045652	22045663
chr12_4145	p2	(GT)9	18	22047992	22048009
chr12_4146	p4	(TCCC)5	20	22059960	22059979
chr12_4147	p2	(CA)7	14	22093347	22093360
chr12_4148	p2	(AC)9	18	22107250	22107267
chr12_4149	p2	(AT)6	12	22118228	22118239
chr12_4150	p3	(TCA)8	24	22127501	22127524
chr12_4151	p2	(AC)13	26	22137165	22137190
chr12_4152	p2	(AT)7	14	22141524	22141537
chr12_4153	c	ccggtgcacgctatcgtccatctgttgtctcacctgtttagtagattgttcata	124	22142288	22142411
chr12_4154	p2	(CA)10	20	22153184	22153203
chr12_4155	p2	(AC)6	12	22155234	22155245
chr12_4156	p2	(GT)6	12	22156071	22156082
chr12_4157	p2	(AC)15	30	22156984	22157013
chr12_4158	p2	(AC)9	18	22157176	22157193
chr12_4159	p2	(CA)7	14	22165431	22165444
chr12_4160	p2	(CA)8	16	22172112	22172127
chr12_4161	p2	(AG)8	16	22172461	22172476
chr12_4162	c	(TG)7g(GT)13	41	22173838	22173878
chr12_4163	p5	(AGTAG)5	25	22174158	22174182
chr12_4164	p2	(TG)8	16	22176992	22177007
chr12_4165	p2	(CA)10	20	22183863	22183882
chr12_4166	p2	(CA)13	26	22187494	22187519
chr12_4167	p2	(TG)11	22	22193172	22193193
chr12_4168	p2	(AT)6	12	22211876	22211887
chr12_4169	p2	(AC)6	12	22212019	22212030
chr12_4170	p2	(TC)6	12	22214499	22214510
chr12_4171	p2	(CA)15	30	22214919	22214948
chr12_4172	p2	(CA)10	20	22215132	22215151
chr12_4173	p2	(GT)6	12	22220340	22220351

chr12_4174	p2	(AC)8	16	22220769	22220784
chr12_4175	p2	(GT)9	18	22225161	22225178
chr12_4176	p3	(AAT)7	21	22226706	22226726
chr12_4177	p2	(CA)16	32	22243823	22243854
chr12_4178	p3	(TAG)5	15	22244491	22244505
chr12_4179	p5	(GGTAA)8	40	22245116	22245155
chr12_4180	c	(TC)8ttt(CTCA)5cacact(CA)25	95	22249398	22249492
chr12_4181	p2	(CA)8	16	22251712	22251727
chr12_4182	p2	(TG)15	30	22252080	22252109
chr12_4183	p2	(TC)14	28	22254107	22254134
chr12_4184	p2	(AC)9	18	22255629	22255646
chr12_4185	p2	(AC)16	32	22257120	22257151
chr12_4186	p2	(AC)7	14	22257497	22257510
chr12_4187	c	taatgatatcattcatagtgtgcagaggatgttgacagtggcagaggaattg	94	22258216	22258309
chr12_4188	p2	(GT)19	38	22260749	22260786
chr12_4189	c	actgcaggacacagcaaagtggagaaatagagggtgtttctcttttacccc	162	22262217	22262378
chr12_4190	p2	(CA)18	36	22262561	22262596
chr12_4191	p3	(TTC)7	21	22263142	22263162
chr12_4192	p2	(CA)6	12	22267540	22267551
chr12_4193	p2	(GT)12	24	22272081	22272104
chr12_4194	p3	(TGT)8	24	22274231	22274254
chr12_4195	p2	(CA)7	14	22275197	22275210
chr12_4196	p2	(CA)29	58	22276861	22276918
chr12_4197	p2	(GT)14	28	22277068	22277095
chr12_4198	p2	(CT)6	12	22277794	22277805
chr12_4199	p2	(GA)6	12	22278069	22278080
chr12_4200	p2	(AC)6	12	22284423	22284434
chr12_4201	p3	(TCT)6	18	22296908	22296925
chr12_4202	p2	(TC)7	14	22299884	22299897
chr12_4203	p2	(GT)8	16	22300034	22300049
chr12_4204	p2	(AC)8	16	22304398	22304413
chr12_4205	p2	(GT)13	26	22304558	22304583
chr12_4206	p2	(TC)7	14	22304893	22304906
chr12_4207	p4	(ATTT)5	20	22307990	22308009
chr12_4208	p2	(TG)7	14	22308163	22308176
chr12_4209	c	(GT)6gg(GT)11	36	22308350	22308385
chr12_4210	p2	(CA)8	16	22308560	22308575
chr12_4211	c	(AC)9ag(AC)13	46	22309557	22309602
chr12_4212	p2	(AC)21	42	22309873	22309914
chr12_4213	p2	(AC)7	14	22310121	22310134
chr12_4214	p2	(GT)8	16	22310873	22310888
chr12_4215	p2	(AC)15	30	22311165	22311194
chr12_4216	c	(AC)9ag(AC)8	36	22314255	22314290
chr12_4217	p2	(TA)9	18	22336856	22336873
chr12_4218	p2	(TG)7	14	22342104	22342117
chr12_4219	p2	(AC)8	16	22342607	22342622
chr12_4220	p4	(GGAT)5	20	22353321	22353340
chr12_4221	p2	(GT)9	18	22355835	22355852
chr12_4222	p2	(TG)7	14	22360684	22360697
chr12_4223	p2	(CA)10	20	22360912	22360931
chr12_4224	p3	(TAA)5	15	22362260	22362274
chr12_4225	p2	(TG)14	28	22362532	22362559

chr12_4226	p2	(AG)9	18	22369530	22369547
chr12_4227	c	tgtgtttatgataataggccaaatgcccctattgattctttctctgtctgtgtctg	94	22369887	22369980
chr12_4228	p2	(AC)15	30	22381342	22381371
chr12_4229	p2	(GT)7	14	22381579	22381592
chr12_4230	p4	(TGTT)5	20	22382261	22382280
chr12_4231	p2	(TA)10	20	22391761	22391780
chr12_4232	p2	(CA)7	14	22398143	22398156
chr12_4233	p2	(TG)23	46	22401875	22401920
chr12_4234	p2	(CA)10	20	22402246	22402265
chr12_4235	p2	(TG)10	20	22402387	22402406
chr12_4236	p3	(CAG)5	15	22412253	22412267
chr12_4237	p2	(GT)9	18	22415860	22415877
chr12_4238	p2	(AC)14	28	22416022	22416049
chr12_4239	p3	(ATT)5	15	22422802	22422816
chr12_4240	p3	(TTA)7	21	22424577	22424597
chr12_4241	p2	(CA)11	22	22430779	22430800
chr12_4242	p2	(AT)7	14	22438248	22438261
chr12_4243	c	(TC)8gctct(CA)20	61	22441504	22441564
chr12_4244	p2	(CA)12	24	22442327	22442350
chr12_4245	p3	(AAT)5	15	22444980	22444994
chr12_4246	p3	(CAA)5	15	22450136	22450150
chr12_4247	p2	(GT)6	12	22450525	22450536
chr12_4248	p2	(AC)8	16	22452906	22452921
chr12_4249	c	catcagggcgaaaaggggctcaggctttcatctgcccgccgctggttcaa	157	22461030	22461186
chr12_4250	p2	(GT)18	36	22461313	22461348
chr12_4251	p4	(TCAT)5	20	22463504	22463523
chr12_4252	p2	(GT)13	26	22465846	22465871
chr12_4253	p2	(TG)9	18	22471334	22471351
chr12_4254	p2	(TC)10	20	22474215	22474234
chr12_4255	p2	(CA)8	16	22479684	22479699
chr12_4256	p3	(TAT)8	24	22488504	22488527
chr12_4257	p2	(TG)8	16	22494141	22494156
chr12_4258	p2	(AC)6	12	22494267	22494278
chr12_4259	p4	(ATTC)6	24	22499856	22499879
chr12_4260	p2	(TC)18	36	22510922	22510957
chr12_4261	p2	(AC)17	34	22521546	22521579
chr12_4262	p2	(GT)10	20	22544436	22544455
chr12_4263	p4	(GATG)5	20	22544605	22544624
chr12_4264	p3	(GAA)9	27	22545854	22545880
chr12_4265	p4	(GATA)8	32	22548746	22548777
chr12_4266	p2	(AC)11	22	22564956	22564977
chr12_4267	p2	(TC)7	14	22572305	22572318
chr12_4268	c	(CTG)6gtccacccactccgaccagtactactgcttctc(CTG)6	69	22573010	22573078
chr12_4269	p3	(AAC)6	18	22583854	22583871
chr12_4270	p3	(CTT)6	18	22584562	22584579
chr12_4271	p2	(TG)8	16	22597563	22597578
chr12_4272	p3	(TGG)5	15	22597816	22597830
chr12_4273	p2	(AG)9	18	22617460	22617477
chr12_4274	p3	(GGA)11	33	22617808	22617840
chr12_4275	p5	(AAATC)5	25	22629236	22629260
chr12_4276	p3	(ATC)5	15	22647302	22647316
chr12_4277	p2	(TG)18	36	22657852	22657887

chr12_4278	p2	(GT)6	12	22660611	22660622
chr12_4279	p2	(TC)7	14	22667871	22667884
chr12_4280	p2	(TG)16	32	22679459	22679490
chr12_4281	p3	(TTC)5	15	22680610	22680624
chr12_4282	p2	(AG)7	14	22693805	22693818
chr12_4283	p2	(CA)7	14	22697873	22697886
chr12_4284	p2	(CA)6	12	22710402	22710413
chr12_4285	p3	(TTG)6	18	22714964	22714981
chr12_4286	p3	(AAC)7	21	22718187	22718207
chr12_4287	p2	(AC)17	34	22727994	22728027
chr12_4288	p2	(TG)7	14	22734829	22734842
chr12_4289	p2	(GT)6	12	22737483	22737494
chr12_4290	p2	(TG)16	32	22757652	22757683
chr12_4291	p4	(AATG)5	20	22758553	22758572
chr2_1	p4	(TTGA)5	20	1421	1440
chr2_2	p2	(TA)7	14	1764	1777
chr2_3	p2	(TG)9	18	8695	8712
chr2_4	p3	(CTC)6	18	22810	22827
chr2_5	p2	(TG)21	42	26500	26541
chr2_6	p2	(TG)21	42	29438	29479
chr2_7	p2	(AG)8	16	38356	38371
chr2_8	p2	(GT)11	22	41002	41023
chr2_9	p2	(GT)25	50	49905	49954
chr2_10	c	(AC)6ag(AC)10	34	54356	54389
chr2_11	p2	(CA)7	14	54896	54909
chr2_12	p2	(AC)6	12	61728	61739
chr2_13	p3	(ATA)9	27	63217	63243
chr2_14	p2	(AC)10	20	72028	72047
chr2_15	p2	(AG)7	14	77617	77630
chr2_16	p5	(AGTAG)5	25	85480	85504
chr2_17	p2	(TG)6	12	89223	89234
chr2_18	p2	(CA)12	24	89428	89451
chr2_19	p2	(CT)6	12	100621	100632
chr2_20	p3	(GGA)8	24	118763	118786
chr2_21	p2	(TG)18	36	132650	132685
chr2_22	c	(GA)8(GT)6	28	134988	135015
chr2_23	p2	(AC)10	20	135195	135214
chr2_24	c	cagacgtactgtgtgaagatgcaaaaatgctgcagagctaaatattgtgag	78	148996	149073
chr2_25	p2	(CA)23	46	151935	151980
chr2_26	p2	(AC)12	24	158721	158744
chr2_27	p2	(TA)6	12	161133	161144
chr2_28	p2	(AG)10	20	161724	161743
chr2_29	p2	(CA)11	22	161949	161970
chr2_30	p2	(GA)11	22	166175	166196
chr2_31	p3	(TAA)5	15	185779	185793
chr2_32	c	(AC)8(AT)6	28	190985	191012
chr2_33	p4	(TTAC)6	24	214476	214499
chr2_34	p2	(TG)11	22	218833	218854
chr2_35	p2	(TG)6	12	227015	227026
chr2_36	p2	(TG)6	12	252067	252078
chr2_37	p2	(AC)8	16	258786	258801
chr2_38	p3	(TTA)9	27	259630	259656

chr2_39	p3	(CTT)5	15	263155	263169
chr2_40	p2	(TG)22	44	269490	269533
chr2_41	p3	(ATT)7	21	271750	271770
chr2_42	p2	(GT)8	16	276445	276460
chr2_43	p3	(CAA)5	15	293833	293847
chr2_44	p2	(GT)17	34	300758	300791
chr2_45	p2	(GT)9	18	302718	302735
chr2_46	p2	(CA)8	16	305970	305985
chr2_47	p2	(TG)17	34	313572	313605
chr2_48	p2	(TC)9	18	315538	315555
chr2_49	p2	(GT)15	30	317954	317983
chr2_50	p2	(CT)6	12	320471	320482
chr2_51	p2	(TA)8	16	326929	326944
chr2_52	p2	(AC)6	12	366981	366992
chr2_53	p2	(AC)10	20	374069	374088
chr2_54	p2	(TG)17	34	388187	388220
chr2_55	p2	(TG)10	20	389899	389918
chr2_56	c	6tcatgaggatcacaaatgtgtgctgcgcaatctgcacgtataaagccatt(	74	391799	391872
chr2_57	p2	(AC)9	18	393250	393267
chr2_58	p2	(AC)9	18	398248	398265
chr2_59	p2	(AC)6	12	409246	409257
chr2_60	p2	(CA)6	12	411915	411926
chr2_61	p2	(TC)10	20	423123	423142
chr2_62	p2	(GT)13	26	426315	426340
chr2_63	p2	(TC)8	16	429651	429666
chr2_64	p2	(TA)9	18	437679	437696
chr2_65	p2	(TG)11	22	460770	460791
chr2_66	p2	(CA)6	12	462750	462761
chr2_67	p2	(CA)7	14	470865	470878
chr2_68	p2	(GT)10	20	472248	472267
chr2_69	p4	(ATGG)5	20	472404	472423
chr2_70	c	(TG)6ctcaacaagtccttctgcacaacaaa(AAC)5	53	478171	478223
chr2_71	p2	(TG)9	18	482607	482624
chr2_72	p2	(GT)17	34	499315	499348
chr2_73	p3	(CAG)5	15	520541	520555
chr2_74	p3	(ATT)6	18	521439	521456
chr2_75	p2	(GT)21	42	521721	521762
chr2_76	p4	(AGGT)5	20	524485	524504
chr2_77	p4	(GAAA)6	24	526781	526804
chr2_78	p2	(CA)30	60	527205	527264
chr2_79	p3	(TTA)5	15	529842	529856
chr2_80	p5	(CTACT)8	40	538567	538606
chr2_81	p3	(CAA)6	18	539356	539373
chr2_82	p2	(GT)25	50	544252	544301
chr2_83	p3	(TAT)5	15	547789	547803
chr2_84	c	(CA)7cg(CA)14	44	558826	558869
chr2_85	p2	(TA)6	12	576597	576608
chr2_86	p2	(AG)6	12	580475	580486
chr2_87	p5	(AATCA)6	30	587697	587726
chr2_88	c	(TG)21agagagc(GA)7gggggggg(GA)8	87	587958	588044
chr2_89	p2	(TA)8	16	593785	593800
chr2_90	p2	(AC)6	12	595394	595405

chr2_91	c	(TC)8at(AC)20	58	615991	616048
chr2_92	c*	(GT)11(GTGA)6*	44	641989	642032
chr2_93	p3	(TCA)8	24	655436	655459
chr2_94	p2	(AC)7	14	681976	681989
chr2_95	p2	(CA)6	12	683616	683627
chr2_96	p4	(TCCA)5	20	703637	703656
chr2_97	p3	(ATA)5	15	706563	706577
chr2_98	c	(TAT)5tgttgttcttgttg(TTA)5	44	708694	708737
chr2_99	p2	(CT)7	14	710350	710363
chr2_100	p2	(TA)7	14	719727	719740
chr2_101	p4	(TTTA)5	20	727943	727962
chr2_102	p3	(ATT)6	18	728798	728815
chr2_103	p2	(AC)11	22	730377	730398
chr2_104	p2	(TG)10	20	735207	735226
chr2_105	p3	(ATA)5	15	750526	750540
chr2_106	p2	(TC)9	18	763494	763511
chr2_107	p2	(TG)18	36	765978	766013
chr2_108	p2	(GT)15	30	774505	774534
chr2_109	p2	(TC)6	12	800774	800785
chr2_110	p2	(TG)16	32	807858	807889
chr2_111	p2	(TC)19	38	808346	808383
chr2_112	p3	(TTA)5	15	845337	845351
chr2_113	p4	(TATT)5	20	854564	854583
chr2_114	p2	(GT)16	32	859750	859781
chr2_115	p2	(TG)7	14	863989	864002
chr2_116	p2	(TG)6	12	867299	867310
chr2_117	c	(AG)6g(GA)9	31	868470	868500
chr2_118	c	)15agacttcacatggaaactgtacagtagttgtcagtatcatcttgaaa(A[illegible]	95	869847	869941
chr2_119	p2	(GA)8	16	870445	870460
chr2_120	p2	(TG)11	22	874436	874457
chr2_121	c	(CA)6t(AC)6	25	874676	874700
chr2_122	p2	(TG)19	38	875118	875155
chr2_123	p3	(TTG)5	15	881087	881101
chr2_124	p3	(CCT)7	21	882538	882558
chr2_125	p2	(TG)6	12	885258	885269
chr2_126	p2	(TG)7	14	885804	885817
chr2_127	p2	(TG)10	20	888203	888222
chr2_128	p2	(TG)6	12	889856	889867
chr2_129	p2	(TG)7	14	891438	891451
chr2_130	p2	(TG)6	12	891798	891809
chr2_131	p2	(TG)14	28	893053	893080
chr2_132	p3	(CTG)5	15	893478	893492
chr2_133	c	(AAAT)6tgctgtt(CA)14	59	897751	897809
chr2_134	p2	(AC)6	12	905048	905059
chr2_135	p2	(TG)11	22	908598	908619
chr2_136	p2	(AC)6	12	908888	908899
chr2_137	p2	(TG)14	28	910718	910745
chr2_138	p2	(GA)6	12	916230	916241
chr2_139	p2	(GT)14	28	918157	918184
chr2_140	p2	(GT)6	12	919685	919696
chr2_141	p2	(CA)15	30	920587	920616
chr2_142	p2	(TC)9	18	921490	921507

chr2_143	p2	(TA)8	16	922080	922095
chr2_144	p2	(GT)28	56	922689	922744
chr2_145	c	(TG)17(AG)16	66	923517	923582
chr2_146	p2	(CA)6	12	924371	924382
chr2_147	p2	(TG)10	20	925703	925722
chr2_148	c	gagggtgaggatgaggaagagggaggaagggtgaagagtagcatgaga	113	930537	930649
chr2_149	p3	(GGA)6	18	932952	932969
chr2_150	p2	(CA)12	24	933128	933151
chr2_151	p2	(GT)19	38	937155	937192
chr2_152	p2	(AC)10	20	942330	942349
chr2_153	p2	(GA)6	12	942550	942561
chr2_154	p2	(AC)6	12	942984	942995
chr2_155	p2	(TG)10	20	943309	943328
chr2_156	p2	(TG)6	12	943594	943605
chr2_157	p2	(AC)6	12	943796	943807
chr2_158	p2	(GT)8	16	949450	949465
chr2_159	p2	(TG)8	16	953852	953867
chr2_160	p2	(CA)11	22	955965	955986
chr2_161	c	catgcgttgatttataaaatttgtgtcgtgttttgatcactgtgcttgtcaggata	120	963218	963337
chr2_162	c	(TG)22(AG)6	56	963953	964008
chr2_163	p2	(TG)10	20	964912	964931
chr2_164	p2	(TG)6	12	965496	965507
chr2_165	c	gtggctagcagcagagctctggg(AC)7aaatacacacaatgtgacagc	238	965610	965847
chr2_166	p2	(TG)6	12	967070	967081
chr2_167	c	gtggctagcagcagagctctggg(AC)7aaatacacacaatgtgacagc	238	967184	967421
chr2_168	p2	(TG)7	14	972586	972599
chr2_169	p2	(CT)10	20	973093	973112
chr2_170	p2	(AG)6	12	975589	975600
chr2_171	c	(TC)12(AC)14	52	976424	976475
chr2_172	p2	(CA)12	24	977085	977108
chr2_173	p2	(CA)16	32	978040	978071
chr2_174	p2	(TG)11	22	978431	978452
chr2_175	p2	(AG)7	14	981114	981127
chr2_176	p4	(ATGG)5	20	983292	983311
chr2_177	p2	(TC)7	14	985246	985259
chr2_178	p2	(CA)16	32	986609	986640
chr2_179	p2	(CA)16	32	989189	989220
chr2_180	p2	(CT)14	28	991937	991964
chr2_181	p2	(AC)8	16	992756	992771
chr2_182	p2	(AC)7	14	993125	993138
chr2_183	p2	(CA)6	12	996199	996210
chr2_184	p2	(GA)7	14	999773	999786
chr2_185	c	(CA)11cgtacatg(CA)6	42	999919	999960
chr2_186	p5	(CAAAT)5	25	1001634	1001658
chr2_187	p5	(GAGAG)5	25	1002072	1002096
chr2_188	p2	(AC)6	12	1003277	1003288
chr2_189	p3	(CAG)5	15	1003846	1003860
chr2_190	p2	(GT)34	68	1006106	1006173
chr2_191	p3	(CTC)5	15	1006294	1006308
chr2_192	p4	(TCCA)5	20	1006850	1006869
chr2_193	p2	(CA)6	12	1014741	1014752
chr2_194	p4	(TTTG)8	32	1021223	1021254

chr2_195	p2	(AG)7	14	1023056	1023069
chr2_196	c	(CA)6t(AC)7	27	1023731	1023757
chr2_197	p2	(GT)21	42	1029475	1029516
chr2_198	p2	(TG)11	22	1036938	1036959
chr2_199	p2	(GT)7	14	1038162	1038175
chr2_200	p3	(TGT)6	18	1038624	1038641
chr2_201	p2	(GA)8	16	1039713	1039728
chr2_202	p2	(AG)7	14	1042139	1042152
chr2_203	p2	(TC)6	12	1042818	1042829
chr2_204	p2	(CT)6	12	1043706	1043717
chr2_205	p2	(AC)9	18	1044419	1044436
chr2_206	p2	(TG)6	12	1045432	1045443
chr2_207	c	(AC)11(AT)7	36	1048243	1048278
chr2_208	p2	(GT)6	12	1053887	1053898
chr2_209	p2	(GT)14	28	1061154	1061181
chr2_210	p2	(GT)6	12	1071192	1071203
chr2_211	p2	(TG)7	14	1074584	1074597
chr2_212	p2	(GT)19	38	1091557	1091594
chr2_213	p4	(ATGG)6	24	1095754	1095777
chr2_214	c	(CT)10ccccctccctctcacacacacaccta(CT)20(CA)9	104	1096316	1096419
chr2_215	p3	(ATA)6	18	1104808	1104825
chr2_216	p2	(TG)17	34	1108377	1108410
chr2_217	c	(GT)14gcgc(GCGT)5	52	1109342	1109393
chr2_218	c	6tatatgtatgtatgtgtgtgtgtatatatgtatatatgtatatgtg(TA)7tg('	86	1109502	1109587
chr2_219	p2	(GT)7	14	1131976	1131989
chr2_220	p2	(GT)9	18	1133606	1133623
chr2_221	p2	(GT)19	38	1142359	1142396
chr2_222	p3	(AAT)6	18	1156712	1156729
chr2_223	p2	(TA)6	12	1171803	1171814
chr2_224	p3	(TAT)5	15	1180873	1180887
chr2_225	p2	(CA)9	18	1182986	1183003
chr2_226	p2	(GT)14	28	1184111	1184138
chr2_227	p2	(GT)8	16	1184600	1184615
chr2_228	p2	(AC)6	12	1196901	1196912
chr2_229	p3	(AAT)5	15	1213520	1213534
chr2_230	p2	(GT)10	20	1227377	1227396
chr2_231	p2	(AC)12	24	1230861	1230884
chr2_232	p2	(TA)7	14	1231471	1231484
chr2_233	p2	(CA)15	30	1246512	1246541
chr2_234	p3	(TAA)5	15	1247345	1247359
chr2_235	p2	(AC)19	38	1256269	1256306
chr2_236	p2	(AC)8	16	1258536	1258551
chr2_237	p2	(GT)11	22	1258893	1258914
chr2_238	p4	(TGTT)5	20	1261415	1261434
chr2_239	p2	(CA)46	92	1278227	1278318
chr2_240	c	(TG)7tatacatc(TA)7(GA)10	56	1295494	1295549
chr2_241	p4	(CAGT)6	24	1312958	1312981
chr2_242	p2	(GT)6	12	1313725	1313736
chr2_243	p2	(TG)13	26	1325247	1325272
chr2_244	p2	(TA)6	12	1332160	1332171
chr2_245	p5	(TTTGT)5	25	1361096	1361120
chr2_246	p2	(AC)11	22	1369786	1369807

chr2_247	p2	(TG)7	14	1380457	1380470
chr2_248	c	(CTAT)5(CCAT)7(CTAT)6(CCAT)5	92	1394072	1394163
chr2_249	p3	(ATT)9	27	1402515	1402541
chr2_250	p2	(AT)6	12	1404740	1404751
chr2_251	p2	(TG)8	16	1406271	1406286
chr2_252	p3	(TTA)6	18	1409752	1409769
chr2_253	p2	(CA)8	16	1412952	1412967
chr2_254	p2	(TC)6	12	1416635	1416646
chr2_255	p5	(ATAAG)12	60	1423252	1423311
chr2_256	p2	(AC)14	28	1432144	1432171
chr2_257	p2	(CA)15	30	1439107	1439136
chr2_258	p2	(AC)6	12	1450568	1450579
chr2_259	p2	(GA)10	20	1453816	1453835
chr2_260	p5	(AAATC)6	30	1469931	1469960
chr2_261	c	(TG)12tc(TG)12	50	1475992	1476041
chr2_262	c	(AC)7c(CA)12	39	1476642	1476680
chr2_263	p2	(TG)6	12	1496145	1496156
chr2_264	p2	(CA)14	28	1497484	1497511
chr2_265	p2	(CT)7	14	1497704	1497717
chr2_266	p2	(TC)30	60	1505273	1505332
chr2_267	p2	(GA)6	12	1524090	1524101
chr2_268	p2	(GT)19	38	1539243	1539280
chr2_269	p3	(ATA)7	21	1548747	1548767
chr2_270	p2	(AC)6	12	1549865	1549876
chr2_271	p2	(TG)18	36	1556237	1556272
chr2_272	p3	(AAC)5	15	1574296	1574310
chr2_273	p2	(AC)7	14	1575484	1575497
chr2_274	p2	(GT)6	12	1590812	1590823
chr2_275	c	gtaaaaagacatgggcagactaacagaatcagatttggacataacagtga	182	1610984	1611165
chr2_276	p2	(CA)8	16	1611910	1611925
chr2_277	c	(TC)13(AC)7	40	1612927	1612966
chr2_278	p3	(AAC)5	15	1617725	1617739
chr2_279	p2	(AC)22	44	1623239	1623282
chr2_280	p2	(AC)14	28	1633168	1633195
chr2_281	p2	(GT)21	42	1636858	1636899
chr2_282	c	(TC)20(AC)17	74	1637392	1637465
chr2_283	p5	(ACAAA)5	25	1638873	1638897
chr2_284	p2	(GT)13	26	1639224	1639249
chr2_285	p2	(AC)6	12	1650402	1650413
chr2_286	p3	(ATT)6	18	1654992	1655009
chr2_287	p2	(GT)18	36	1658467	1658502
chr2_288	p2	(GT)6	12	1661203	1661214
chr2_289	p4	(ATGG)7	28	1663744	1663771
chr2_290	p2	(CA)33	66	1665682	1665747
chr2_291	p2	(GT)20	40	1703564	1703603
chr2_292	p2	(AC)14	28	1722236	1722263
chr2_293	p3	(TCT)5	15	1723250	1723264
chr2_294	p2	(AC)14	28	1724526	1724553
chr2_295	p3	(TCT)5	15	1725540	1725554
chr2_296	p3	(ATA)6	18	1733042	1733059
chr2_297	c	(AG)6(TG)12	36	1737238	1737273
chr2_298	p2	(TG)7	14	1742150	1742163

chr2_299	p3	(AAT)5	15	1747871	1747885
chr2_300	p2	(GT)8	16	1749783	1749798
chr2_301	p2	(CA)31	62	1756476	1756537
chr2_302	p4	(CAAA)5	20	1770638	1770657
chr2_303	p5	(AAATC)5	25	1780241	1780265
chr2_304	p3	(AGG)5	15	1781543	1781557
chr2_305	p2	(AC)6	12	1811125	1811136
chr2_306	p2	(AC)12	24	1811657	1811680
chr2_307	p2	(TA)6	12	1812284	1812295
chr2_308	p2	(TG)11	22	1819714	1819735
chr2_309	p2	(TA)7	14	1839301	1839314
chr2_310	p2	(AC)9	18	1843890	1843907
chr2_311	p2	(TG)7	14	1867484	1867497
chr2_312	p3	(ATA)5	15	1881105	1881119
chr2_313	p3	(ATA)8	24	1882693	1882716
chr2_314	c	(AC)7atg(CA)12	41	1932142	1932182
chr2_315	p3	(TAT)6	18	1955382	1955399
chr2_316	p2	(AC)9	18	1955507	1955524
chr2_317	p2	(TG)21	42	1956350	1956391
chr2_318	p2	(TG)16	32	1956627	1956658
chr2_319	p4	(AATA)7	28	1956849	1956876
chr2_320	p4	(AAAT)5	20	1957929	1957948
chr2_321	p2	(AC)20	40	1964157	1964196
chr2_322	p2	(AC)14	28	1970964	1970991
chr2_323	p4	(TTCA)5	20	1986749	1986768
chr2_324	p2	(TC)6	12	2021529	2021540
chr2_325	p2	(AC)14	28	2039563	2039590
chr2_326	p4	(AATA)5	20	2041144	2041163
chr2_327	p2	(AC)14	28	2046227	2046254
chr2_328	p4	(GGAG)6	24	2055799	2055822
chr2_329	p2	(TG)6	12	2056524	2056535
chr2_330	c	(AC)7gcacacacg(CA)8	39	2057252	2057290
chr2_331	p4	(AATA)5	20	2060636	2060655
chr2_332	p3	(TTA)5	15	2065119	2065133
chr2_333	p3	(GAT)16	48	2084553	2084600
chr2_334	p4	(ATCT)6	24	2099927	2099950
chr2_335	p2	(GA)6	12	2113693	2113704
chr2_336	p2	(CA)23	46	2117247	2117292
chr2_337	p4	(AAAC)8	32	2125030	2125061
chr2_338	p2	(CA)10	20	2139084	2139103
chr2_339	p2	(CT)9	18	2152916	2152933
chr2_340	p2	(TG)15	30	2157083	2157112
chr2_341	p2	(AT)6	12	2181566	2181577
chr2_342	p2	(GT)6	12	2199388	2199399
chr2_343	p3	(ATT)5	15	2204488	2204502
chr2_344	p2	(TA)14	28	2209773	2209800
chr2_345	p2	(TC)7	14	2210354	2210367
chr2_346	p2	(AT)8	16	2210879	2210894
chr2_347	p3	(ATT)5	15	2218344	2218358
chr2_348	p3	(ATT)5	15	2222147	2222161
chr2_349	p3	(ATT)6	18	2225749	2225766
chr2_350	p2	(AC)19	38	2226424	2226461

chr2_351	p2	(AC)11	22	2230197	2230218
chr2_352	p3	(TAA)5	15	2251526	2251540
chr2_353	p6	(ATATAG)6	36	2263176	2263211
chr2_354	p5	(TTAGC)7	35	2265300	2265334
chr2_355	c	(TA)8ttacatagtactaaataacgttttat(AG)7	56	2277904	2277959
chr2_356	p3	(TCC)5	15	2282409	2282423
chr2_357	p2	(AC)6	12	2287968	2287979
chr2_358	p2	(TG)6	12	2304860	2304871
chr2_359	p4	(AGAT)10	40	2311755	2311794
chr2_360	p2	(CA)10	20	2312423	2312442
chr2_361	p2	(AC)8	16	2331023	2331038
chr2_362	p2	(AT)6	12	2339382	2339393
chr2_363	c	(TTA)7tttctgcatttctcagtacctacatactgtatatatgc(GT)11	81	2340329	2340409
chr2_364	p2	(TG)8	16	2345029	2345044
chr2_365	p2	(GT)13	26	2345621	2345646
chr2_366	p2	(GA)6	12	2354421	2354432
chr2_367	p2	(CA)22	44	2355693	2355736
chr2_368	c	(AC)7ag(AC)15	46	2356064	2356109
chr2_369	p2	(TG)7	14	2358600	2358613
chr2_370	p3	(CTG)6	18	2359430	2359447
chr2_371	p4	(TTTG)5	20	2363508	2363527
chr2_372	p2	(GT)6	12	2364708	2364719
chr2_373	p2	(AC)9	18	2373720	2373737
chr2_374	p2	(AT)6	12	2382426	2382437
chr2_375	p2	(AC)14	28	2407599	2407626
chr2_376	p2	(TG)25	50	2412470	2412519
chr2_377	p4	(GGAA)5	20	2431038	2431057
chr2_378	p3	(AAC)5	15	2438399	2438413
chr2_379	p3	(CTG)5	15	2441596	2441610
chr2_380	p3	(TAT)6	18	2450348	2450365
chr2_381	p2	(CA)12	24	2458375	2458398
chr2_382	p3	(TAA)7	21	2466043	2466063
chr2_383	p3	(TTA)5	15	2472630	2472644
chr2_384	p2	(AC)8	16	2479123	2479138
chr2_385	p3	(TTC)6	18	2483389	2483406
chr2_386	p2	(AG)6	12	2495030	2495041
chr2_387	p2	(TG)10	20	2495265	2495284
chr2_388	p2	(TG)11	22	2502742	2502763
chr2_389	p2	(CA)7	14	2512944	2512957
chr2_390	p2	(AC)6	12	2517872	2517883
chr2_391	p2	(CA)11	22	2518413	2518434
chr2_392	p2	(AC)6	12	2518735	2518746
chr2_393	p2	(CA)8	16	2520758	2520773
chr2_394	p2	(TA)7	14	2523940	2523953
chr2_395	p2	(GT)6	12	2529403	2529414
chr2_396	p2	(TC)7	14	2530614	2530627
chr2_397	p3	(AAG)5	15	2530940	2530954
chr2_398	p3	(CTC)5	15	2535348	2535362
chr2_399	p2	(CA)6	12	2536822	2536833
chr2_400	c	(CA)6aa(AC)29	72	2539353	2539424
chr2_401	p3	(ACC)5	15	2540050	2540064
chr2_402	p2	(GT)7	14	2543077	2543090

chr2_403	p2	(GT)8	16	2549078	2549093
chr2_404	p2	(TG)12	24	2555643	2555666
chr2_405	p2	(AT)6	12	2555902	2555913
chr2_406	c	cacatacatac(AT)7ctatatctatatatagatatagatatatatatc(TA)	124	2557288	2557411
chr2_407	p2	(AC)13	26	2562170	2562195
chr2_408	p2	(TG)22	44	2562313	2562356
chr2_409	p2	(AC)6	12	2562956	2562967
chr2_410	p2	(CA)22	44	2568075	2568118
chr2_411	p2	(AC)6	12	2571347	2571358
chr2_412	p2	(AT)8	16	2576159	2576174
chr2_413	p2	(AG)10	20	2580524	2580543
chr2_414	c	(CT)9cacacacacgcg(CA)6	42	2590303	2590344
chr2_415	c	(CA)6cg(CA)9	32	2592096	2592127
chr2_416	p2	(TG)7	14	2592757	2592770
chr2_417	p2	(AT)7	14	2593672	2593685
chr2_418	p2	(TA)9	18	2597088	2597105
chr2_419	c	(AC)6agag(AC)15	46	2597429	2597474
chr2_420	p2	(AC)6	12	2598611	2598622
chr2_421	p2	(AC)13	26	2598849	2598874
chr2_422	c	(CA)9cgcacacacg(CA)7	42	2599047	2599088
chr2_423	p2	(TG)12	24	2602443	2602466
chr2_424	p2	(TG)18	36	2602901	2602936
chr2_425	p2	(TG)13	26	2603406	2603431
chr2_426	p2	(CA)9	18	2603623	2603640
chr2_427	c	(TG)17agagagg(GA)7	55	2603782	2603836
chr2_428	p2	(TC)6	12	2604155	2604166
chr2_429	p2	(AC)6	12	2605334	2605345
chr2_430	c	(CA)6aagacaattgcaccataggcaaattgaaaat(AC)17	77	2606850	2606926
chr2_431	p2	(AC)8	16	2607338	2607353
chr2_432	p2	(TC)7	14	2608875	2608888
chr2_433	c	(GA)6a(AG)10	33	2613166	2613198
chr2_434	c	(GA)6a(AG)10	33	2614196	2614228
chr2_435	p2	(CA)6	12	2635615	2635626
chr2_436	p2	(TG)10	20	2643050	2643069
chr2_437	p2	(AC)6	12	2646495	2646506
chr2_438	p2	(GT)6	12	2648071	2648082
chr2_439	p2	(CA)8	16	2648619	2648634
chr2_440	p3	(AAT)8	24	2660107	2660130
chr2_441	p2	(AC)23	46	2660367	2660412
chr2_442	p2	(AC)8	16	2702470	2702485
chr2_443	c	(AT)10gta(TG)13	49	2704431	2704479
chr2_444	p4	(TTAA)5	20	2730505	2730524
chr2_445	p2	(GA)8	16	2753215	2753230
chr2_446	p2	(CA)23	46	2756330	2756375
chr2_447	p3	(TGC)7	21	2757630	2757650
chr2_448	p4	(ATGA)5	20	2762981	2763000
chr2_449	p2	(TG)8	16	2768407	2768422
chr2_450	p2	(TG)15	30	2775221	2775250
chr2_451	p2	(CA)31	62	2782057	2782118
chr2_452	p3	(AAG)6	18	2782794	2782811
chr2_453	p2	(AT)10	20	2783351	2783370
chr2_454	p2	(AC)11	22	2783516	2783537

chr2_455	p2	(GA)8	16	2784607	2784622
chr2_456	p2	(CA)16	32	2786138	2786169
chr2_457	p2	(AC)11	22	2789313	2789334
chr2_458	p2	(GA)9	18	2790404	2790421
chr2_459	c	(TG)7tt(TG)11	38	2791118	2791155
chr2_460	p2	(CA)16	32	2791603	2791634
chr2_461	p2	(AG)6	12	2794805	2794816
chr2_462	c	(CA)16a(AC)9	51	2797509	2797559
chr2_463	p2	(CA)21	42	2798302	2798343
chr2_464	p2	(GT)8	16	2800513	2800528
chr2_465	p2	(AC)10	20	2803336	2803355
chr2_466	p2	(CA)11	22	2803792	2803813
chr2_467	p2	(CA)10	20	2805085	2805104
chr2_468	p2	(TG)13	26	2806748	2806773
chr2_469	p2	(TG)9	18	2813407	2813424
chr2_470	p2	(TG)6	12	2813843	2813854
chr2_471	p2	(CA)7	14	2815365	2815378
chr2_472	p3	(ATG)5	15	2820552	2820566
chr2_473	p3	(CCT)6	18	2826115	2826132
chr2_474	p2	(GA)11	22	2827515	2827536
chr2_475	p2	(CA)25	50	2829397	2829446
chr2_476	p2	(AC)6	12	2834148	2834159
chr2_477	p2	(TG)6	12	2837833	2837844
chr2_478	p2	(AC)17	34	2838983	2839016
chr2_479	p2	(CA)6	12	2839501	2839512
chr2_480	p2	(AC)6	12	2850640	2850651
chr2_481	p2	(TA)6	12	2855319	2855330
chr2_482	p3	(TAT)5	15	2861905	2861919
chr2_483	p2	(AC)11	22	2869674	2869695
chr2_484	p2	(CA)42	84	2871735	2871818
chr2_485	p2	(CA)11	22	2876022	2876043
chr2_486	p3	(GAG)5	15	2887164	2887178
chr2_487	p4	(ATTT)6	24	2887532	2887555
chr2_488	p2	(CA)12	24	2890442	2890465
chr2_489	c	(CT)10(CA)13	46	2896835	2896880
chr2_490	c	(TG)9tac(GT)6	33	2899814	2899846
chr2_491	p2	(CA)10	20	2901209	2901228
chr2_492	p2	(CT)6	12	2902336	2902347
chr2_493	p2	(TG)10	20	2907591	2907610
chr2_494	p2	(TG)9	18	2913200	2913217
chr2_495	c	(TAAG)6taaa(AAAT)6	52	2913775	2913826
chr2_496	p2	(AC)31	62	2915007	2915068
chr2_497	p2	(AC)24	48	2923870	2923917
chr2_498	p2	(TG)13	26	2926628	2926653
chr2_499	p2	(TG)7	14	2927564	2927577
chr2_500	p2	(TG)12	24	2927697	2927720
chr2_501	p2	(AC)11	22	2930961	2930982
chr2_502	p2	(GA)6	12	2931194	2931205
chr2_503	p2	(AG)7	14	2931702	2931715
chr2_504	p2	(AC)14	28	2934120	2934147
chr2_505	p2	(TG)13	26	2934272	2934297
chr2_506	p2	(AC)12	24	2938087	2938110

chr2_507	p3	(CAC)6	18	2941917	2941934
chr2_508	p2	(GA)7	14	2942945	2942958
chr2_509	p2	(AG)8	16	2944046	2944061
chr2_510	p2	(GT)9	18	2966949	2966966
chr2_511	p2	(TG)11	22	2975291	2975312
chr2_512	p2	(TG)26	52	2980759	2980810
chr2_513	p2	(TC)10	20	2983203	2983222
chr2_514	p2	(CT)15	30	2983827	2983856
chr2_515	p3	(TTA)12	36	2990553	2990588
chr2_516	p2	(CT)6	12	2998915	2998926
chr2_517	p3	(GAT)6	18	3003496	3003513
chr2_518	p4	(TGAT)6	24	3009535	3009558
chr2_519	p2	(TG)8	16	3012870	3012885
chr2_520	p2	(CA)9	18	3014730	3014747
chr2_521	p2	(TG)6	12	3014949	3014960
chr2_522	p3	(CAT)10	30	3016661	3016690
chr2_523	p2	(GT)12	24	3016950	3016973
chr2_524	p3	(ATA)5	15	3017470	3017484
chr2_525	p2	(AT)9	18	3018553	3018570
chr2_526	p2	(GT)12	24	3034761	3034784
chr2_527	p2	(CA)16	32	3040428	3040459
chr2_528	p3	(AAT)5	15	3042066	3042080
chr2_529	p2	(AC)12	24	3042753	3042776
chr2_530	p3	(ATT)5	15	3042886	3042900
chr2_531	c	(TTA)12ta(ATT)8	62	3044038	3044099
chr2_532	p3	(AAT)5	15	3063589	3063603
chr2_533	p2	(CA)6	12	3068765	3068776
chr2_534	c	(AC)18ag(AC)6	50	3083672	3083721
chr2_535	p2	(TG)7	14	3116679	3116692
chr2_536	c	(AC)7atatatagatagatagac(AGAT)7	60	3118502	3118561
chr2_537	p5	(CTTCC)8	40	3125088	3125127
chr2_538	p2	(AG)8	16	3136020	3136035
chr2_539	p2	(TC)13	26	3157712	3157737
chr2_540	p2	(GT)12	24	3160768	3160791
chr2_541	p2	(TG)12	24	3163651	3163674
chr2_542	p4	(AATG)7	28	3165977	3166004
chr2_543	p2	(CT)13	26	3168157	3168182
chr2_544	p2	(CT)6	12	3168309	3168320
chr2_545	p3	(ATT)6	18	3171021	3171038
chr2_546	p2	(GT)16	32	3172614	3172645
chr2_547	p3	(ATT)6	18	3173715	3173732
chr2_548	p2	(CA)12	24	3181598	3181621
chr2_549	p2	(CA)8	16	3181805	3181820
chr2_550	p3	(ATT)6	18	3184783	3184800
chr2_551	p4	(ATTT)9	36	3191697	3191732
chr2_552	p2	(AC)7	14	3196130	3196143
chr2_553	p2	(AC)7	14	3198671	3198684
chr2_554	p2	(TG)15	30	3205707	3205736
chr2_555	p2	(AC)6	12	3206190	3206201
chr2_556	p2	(AC)10	20	3212686	3212705
chr2_557	c	tttaggggtacagaactttgtcgtatattctatacgtcagatgatgtggactata	115	3245045	3245159
chr2_558	p4	(TCCA)6	24	3245561	3245584

chr2_559	p4	(TTTC)9	36	3256299	3256334
chr2_560	p2	(TG)13	26	3256579	3256604
chr2_561	p2	(CA)6	12	3257165	3257176
chr2_562	c	(GT)16gagagagagc(GA)6	54	3258579	3258632
chr2_563	p2	(AC)15	30	3259020	3259049
chr2_564	p2	(AC)15	30	3261374	3261403
chr2_565	p2	(CA)9	18	3262012	3262029
chr2_566	p2	(AC)6	12	3262152	3262163
chr2_567	p2	(TG)8	16	3262964	3262979
chr2_568	p2	(AC)7	14	3272503	3272516
chr2_569	p2	(CA)6	12	3281114	3281125
chr2_570	p2	(AC)8	16	3286779	3286794
chr2_571	p2	(TG)10	20	3287877	3287896
chr2_572	p3	(GAG)5	15	3288772	3288786
chr2_573	p2	(TA)8	16	3291244	3291259
chr2_574	p4	(TTTA)5	20	3301600	3301619
chr2_575	p2	(GA)6	12	3301976	3301987
chr2_576	p2	(CA)13	26	3312895	3312920
chr2_577	p3	(GGA)5	15	3313764	3313778
chr2_578	p3	(AGC)5	15	3318433	3318447
chr2_579	p2	(GT)12	24	3328813	3328836
chr2_580	p2	(TG)12	24	3347684	3347707
chr2_581	p2	(CT)9	18	3348987	3349004
chr2_582	p2	(GT)17	34	3353234	3353267
chr2_583	p2	(GT)18	36	3354588	3354623
chr2_584	p2	(GT)21	42	3359944	3359985
chr2_585	p2	(CA)6	12	3363386	3363397
chr2_586	p2	(GT)6	12	3365135	3365146
chr2_587	p2	(TC)11	22	3368629	3368650
chr2_588	p2	(TG)19	38	3378170	3378207
chr2_589	p2	(GT)7	14	3381177	3381190
chr2_590	p2	(TG)10	20	3382917	3382936
chr2_591	p2	(CA)23	46	3395592	3395637
chr2_592	p2	(GT)24	48	3403599	3403646
chr2_593	p2	(AC)6	12	3415285	3415296
chr2_594	p2	(GT)7	14	3419571	3419584
chr2_595	p3	(TAA)6	18	3423647	3423664
chr2_596	p2	(CA)10	20	3429379	3429398
chr2_597	p3	(CAG)6	18	3431083	3431100
chr2_598	p3	(CAG)6	18	3431288	3431305
chr2_599	p2	(GA)7	14	3440468	3440481
chr2_600	p2	(TA)7	14	3450774	3450787
chr2_601	c	(AG)6g(GA)11	35	3451624	3451658
chr2_602	p4	(CATC)5	20	3452722	3452741
chr2_603	p5	(ATATC)12	60	3461862	3461921
chr2_604	p2	(AC)33	66	3463349	3463414
chr2_605	p2	(TG)7	14	3512753	3512766
chr2_606	p2	(TA)8	16	3513655	3513670
chr2_607	p2	(GT)20	40	3523124	3523163
chr2_608	p4	(CCTA)9	36	3533961	3533996
chr2_609	p3	(AGA)17	51	3536185	3536235
chr2_610	p2	(GT)11	22	3545513	3545534

chr2_611	p2	(AG)6	12	3570809	3570820
chr2_612	p2	(AC)10	20	3590760	3590779
chr2_613	p2	(AC)6	12	3598489	3598500
chr2_614	p2	(CA)8	16	3600892	3600907
chr2_615	p2	(AC)9	18	3610172	3610189
chr2_616	p2	(AC)6	12	3614949	3614960
chr2_617	p4	(ATAC)6	24	3617304	3617327
chr2_618	c	(AT)8(GT)15	46	3621593	3621638
chr2_619	p3	(ATC)7	21	3629606	3629626
chr2_620	c	:acacacatacagtacatatcgtattccccttttttctcaccagccccactccc	98	3632121	3632218
chr2_621	p3	(AAG)5	15	3633398	3633412
chr2_622	p2	(CA)18	36	3633531	3633566
chr2_623	p2	(CA)8	16	3636871	3636886
chr2_624	p2	(AC)15	30	3637040	3637069
chr2_625	p2	(GT)16	32	3637192	3637223
chr2_626	p2	(AC)6	12	3637359	3637370
chr2_627	p2	(TG)6	12	3640660	3640671
chr2_628	p2	(AC)28	56	3651965	3652020
chr2_629	p3	(TAA)5	15	3676888	3676902
chr2_630	p4	(AAAT)5	20	3700913	3700932
chr2_631	p3	(TAG)5	15	3707172	3707186
chr2_632	p2	(GT)6	12	3710589	3710600
chr2_633	p2	(TA)13	26	3711778	3711803
chr2_634	p3	(ATT)5	15	3719499	3719513
chr2_635	p3	(GCT)5	15	3727530	3727544
chr2_636	p3	(CTT)5	15	3728332	3728346
chr2_637	p2	(CA)6	12	3732742	3732753
chr2_638	p3	(CAT)11	33	3757113	3757145
chr2_639	c	(TC)8(AC)21	58	3766725	3766782
chr2_640	p2	(AC)12	24	3767512	3767535
chr2_641	p4	(TTGA)6	24	3771273	3771296
chr2_642	p3	(AAT)6	18	3773185	3773202
chr2_643	p2	(GA)12	24	3775920	3775943
chr2_644	p2	(CA)17	34	3779619	3779652
chr2_645	p2	(AT)7	14	3780709	3780722
chr2_646	p2	(GT)6	12	3780973	3780984
chr2_647	p2	(CA)6	12	3784202	3784213
chr2_648	p2	(CA)6	12	3786616	3786627
chr2_649	p3	(TCT)6	18	3800803	3800820
chr2_650	p2	(AC)7	14	3801930	3801943
chr2_651	p2	(AT)7	14	3810407	3810420
chr2_652	p2	(AC)7	14	3812641	3812654
chr2_653	p2	(TC)6	12	3824048	3824059
chr2_654	p2	(CA)14	28	3829127	3829154
chr2_655	p3	(AAG)8	24	3850299	3850322
chr2_656	p2	(CA)7	14	3852437	3852450
chr2_657	p2	(AC)16	32	3853659	3853690
chr2_658	p2	(CA)12	24	3853959	3853982
chr2_659	p2	(AC)11	22	3858468	3858489
chr2_660	p2	(CA)6	12	3873285	3873296
chr2_661	p2	(TC)6	12	3875278	3875289
chr2_662	c	21agacagagactcacacaacacgtgcagggtttatactgtatatgtac(G	131	3878207	3878337

chr2_663	p2	(CA)14	28	3881698	3881725
chr2_664	c	(TG)22(AG)6	56	3883718	3883773
chr2_665	p2	(AC)19	38	3883926	3883963
chr2_666	p3	(CAT)7	21	3899180	3899200
chr2_667	p2	(GT)18	36	3903059	3903094
chr2_668	p2	(CA)8	16	3910047	3910062
chr2_669	p2	(TG)11	22	3914595	3914616
chr2_670	c	(AC)10g(CA)12	45	3916050	3916094
chr2_671	p2	(CA)14	28	3916687	3916714
chr2_672	p2	(TG)7	14	3920525	3920538
chr2_673	p2	(GT)6	12	3920827	3920838
chr2_674	p3	(ATA)6	18	3924463	3924480
chr2_675	c	(CA)6gacagaagtggatca(AC)6	39	3925278	3925316
chr2_676	p2	(TG)6	12	3931835	3931846
chr2_677	p2	(TC)6	12	3932435	3932446
chr2_678	p2	(GT)7	14	3935974	3935987
chr2_679	p2	(TG)11	22	3941546	3941567
chr2_680	p2	(TC)6	12	3942003	3942014
chr2_681	p2	(TA)6	12	3953288	3953299
chr2_682	p4	(ATGG)5	20	3958816	3958835
chr2_683	p3	(AAG)5	15	3974127	3974141
chr2_684	p2	(TG)22	44	3994212	3994255
chr2_685	p3	(AAT)5	15	4001389	4001403
chr2_686	p2	(TG)8	16	4009727	4009742
chr2_687	p4	(TTTG)5	20	4012957	4012976
chr2_688	p2	(AC)8	16	4016992	4017007
chr2_689	p2	(AC)19	38	4024634	4024671
chr2_690	p2	(TG)6	12	4041292	4041303
chr2_691	p2	(TG)16	32	4041885	4041916
chr2_692	p2	(CA)8	16	4045121	4045136
chr2_693	p2	(AC)6	12	4053685	4053696
chr2_694	p2	(CA)6	12	4057801	4057812
chr2_695	p2	(CA)14	28	4058589	4058616
chr2_696	p4	(AAAT)5	20	4059097	4059116
chr2_697	p2	(CA)16	32	4078633	4078664
chr2_698	p3	(TTA)5	15	4085186	4085200
chr2_699	p2	(AC)13	26	4089159	4089184
chr2_700	p2	(AG)6	12	4111340	4111351
chr2_701	p4	(TATC)5	20	4112043	4112062
chr2_702	c	(CA)7cgcacccacat(AC)16	57	4119840	4119896
chr2_703	p2	(CA)6	12	4120131	4120142
chr2_704	p4	(GATA)7	28	4125520	4125547
chr2_705	p2	(GA)9	18	4127435	4127452
chr2_706	p2	(GT)14	28	4128279	4128306
chr2_707	p2	(AC)9	18	4130442	4130459
chr2_708	p2	(AG)13	26	4133398	4133423
chr2_709	p2	(TG)14	28	4135845	4135872
chr2_710	p2	(TG)13	26	4140948	4140973
chr2_711	p2	(CA)23	46	4143189	4143234
chr2_712	p2	(AC)15	30	4147745	4147774
chr2_713	p3	(TCC)6	18	4152571	4152588
chr2_714	p2	(CT)7	14	4155726	4155739

chr2_715	c	(GT)22(GA)6	56	4156285	4156340
chr2_716	c	(CA)6cg(CA)16	46	4156500	4156545
chr2_717	c	atgtctgcatttgtgtcagcgtgcattaggagagcctacgtactgtaggtatg	107	4162848	4162954
chr2_718	p4	(TTAT)5	20	4169168	4169187
chr2_719	p2	(GA)16	32	4174629	4174660
chr2_720	p2	(TG)9	18	4177561	4177578
chr2_721	p2	(AC)25	50	4178472	4178521
chr2_722	p2	(GT)14	28	4180199	4180226
chr2_723	p2	(AG)10	20	4180588	4180607
chr2_724	p2	(CA)7	14	4182111	4182124
chr2_725	p2	(TG)8	16	4182955	4182970
chr2_726	c	;ttgctcaccctctttctattccctatcctgtgtttcggtgtctgttcattgcctctc	97	4183988	4184084
chr2_727	p3	(ATA)7	21	4188734	4188754
chr2_728	p2	(GT)6	12	4189386	4189397
chr2_729	p4	(AATG)5	20	4189538	4189557
chr2_730	p2	(AG)13	26	4191345	4191370
chr2_731	p2	(GT)7	14	4193255	4193268
chr2_732	p2	(GT)14	28	4193379	4193406
chr2_733	p2	(AC)26	52	4193516	4193567
chr2_734	p2	(CA)11	22	4193716	4193737
chr2_735	p2	(TC)8	16	4193956	4193971
chr2_736	p2	(AC)11	22	4195906	4195927
chr2_737	p2	(CT)7	14	4199210	4199223
chr2_738	p2	(TC)6	12	4199421	4199432
chr2_739	p2	(CA)22	44	4200154	4200197
chr2_740	p2	(AC)7	14	4201111	4201124
chr2_741	p2	(AC)6	12	4202536	4202547
chr2_742	p2	(TG)24	48	4203262	4203309
chr2_743	p2	(TG)7	14	4204211	4204224
chr2_744	c	(TG)16(AG)8	48	4212104	4212151
chr2_745	p2	(CA)7	14	4212311	4212324
chr2_746	p2	(GA)10	20	4214248	4214267
chr2_747	p2	(AG)8	16	4214403	4214418
chr2_748	p2	(TG)17	34	4214788	4214821
chr2_749	p2	(TG)6	12	4227750	4227761
chr2_750	p2	(GT)9	18	4228328	4228345
chr2_751	p2	(TC)8	16	4229913	4229928
chr2_752	p2	(AC)6	12	4237877	4237888
chr2_753	p2	(AC)6	12	4238040	4238051
chr2_754	p2	(TG)7	14	4239058	4239071
chr2_755	p2	(CA)16	32	4239423	4239454
chr2_756	p2	(CT)12	24	4241918	4241941
chr2_757	p2	(GT)6	12	4246609	4246620
chr2_758	p2	(CA)17	34	4247365	4247398
chr2_759	p2	(GT)7	14	4250895	4250908
chr2_760	p2	(AC)6	12	4254822	4254833
chr2_761	p2	(AC)15	30	4269136	4269165
chr2_762	c	(AC)11g(CA)8	39	4271685	4271723
chr2_763	p2	(TC)9	18	4273160	4273177
chr2_764	p2	(TG)6	12	4275277	4275288
chr2_765	c	(ATAG)6(ACAG)5(ATAG)18	116	4276497	4276612
chr2_766	p2	(TG)8	16	4280290	4280305

chr2_767	p2	(GT)6	12	4280551	4280562
chr2_768	c	(AC)13ag(AC)13g(CA)6	67	4283773	4283839
chr2_769	p2	(CA)7	14	4287686	4287699
chr2_770	p2	(GT)6	12	4304152	4304163
chr2_771	p2	(CA)6	12	4311312	4311323
chr2_772	p2	(GT)10	20	4312309	4312328
chr2_773	p2	(CA)7	14	4313640	4313653
chr2_774	p4	(ATGG)5	20	4314724	4314743
chr2_775	p4	(AGAT)14	56	4317608	4317663
chr2_776	p3	(GAG)6	18	4318976	4318993
chr2_777	p2	(CA)9	18	4319319	4319336
chr2_778	p2	(GA)7	14	4319518	4319531
chr2_779	p2	(AC)7	14	4319641	4319654
chr2_780	p3	(ATT)6	18	4328178	4328195
chr2_781	p2	(CA)6	12	4342405	4342416
chr2_782	p2	(TC)6	12	4343484	4343495
chr2_783	p2	(CA)6	12	4344335	4344346
chr2_784	p2	(CA)8	16	4344584	4344599
chr2_785	p2	(AC)6	12	4349293	4349304
chr2_786	p2	(CA)17	34	4349822	4349855
chr2_787	p2	(CA)9	18	4352040	4352057
chr2_788	p2	(CA)19	38	4352293	4352330
chr2_789	p2	(AC)6	12	4353961	4353972
chr2_790	c	(TC)6gctctctctctcctttacccccacaccaccac(CA)13	70	4355775	4355844
chr2_791	p3	(ATG)8	24	4356672	4356695
chr2_792	p2	(AC)7	14	4362590	4362603
chr2_793	p2	(TG)25	50	4364624	4364673
chr2_794	p2	(GA)6	12	4364824	4364835
chr2_795	p2	(TG)6	12	4371094	4371105
chr2_796	p2	(AC)10	20	4375450	4375469
chr2_797	p2	(TG)18	36	4377748	4377783
chr2_798	p2	(AC)21	42	4378556	4378597
chr2_799	p2	(AC)13	26	4381574	4381599
chr2_800	c	(CT)12ttctctcttct(TC)22	79	4386287	4386365
chr2_801	p2	(GT)6	12	4403194	4403205
chr2_802	c	(TG)12(AG)7	38	4407301	4407338
chr2_803	p2	(TG)7	14	4409056	4409069
chr2_804	p2	(TG)6	12	4410727	4410738
chr2_805	p2	(TC)9	18	4411137	4411154
chr2_806	p2	(CA)18	36	4411450	4411485
chr2_807	p2	(AC)14	28	4415431	4415458
chr2_808	p2	(TG)17	34	4418018	4418051
chr2_809	p2	(TC)21	42	4418295	4418336
chr2_810	p2	(TG)9	18	4420206	4420223
chr2_811	p2	(GA)7	14	4420335	4420348
chr2_812	p2	(CA)8	16	4425716	4425731
chr2_813	p2	(AC)6	12	4446334	4446345
chr2_814	p2	(CT)28	56	4452864	4452919
chr2_815	p2	(CT)14	28	4475453	4475480
chr2_816	p3	(CCT)5	15	4475599	4475613
chr2_817	c	(ATCT)7g(TCTA)6	53	4476003	4476055
chr2_818	p2	(TG)20	40	4476466	4476505

chr2_819	p6	(AATACC)8	48	4482142	4482189
chr2_820	p2	(GT)6	12	4493652	4493663
chr2_821	p4	(TCCA)6	24	4499296	4499319
chr2_822	c	AC)7atatacacatatatataaatatatacatatatataaatatatat(AC)2	100	4505549	4505648
chr2_823	p2	(TG)11	22	4514083	4514104
chr2_824	c	(CA)13c(CAT)14	69	4514280	4514348
chr2_825	p2	(CT)9	18	4517508	4517525
chr2_826	p2	(CA)8	16	4522310	4522325
chr2_827	p2	(CA)6	12	4537716	4537727
chr2_828	p2	(TA)7	14	4543387	4543400
chr2_829	p2	(AT)9	18	4544417	4544434
chr2_830	p2	(GA)9	18	4550949	4550966
chr2_831	p2	(AC)6	12	4551489	4551500
chr2_832	p2	(CA)16	32	4551743	4551774
chr2_833	p3	(TCT)5	15	4566792	4566806
chr2_834	p2	(TG)8	16	4579584	4579599
chr2_835	p2	(TA)6	12	4591957	4591968
chr2_836	p3	(ACC)6	18	4600005	4600022
chr2_837	p2	(TG)6	12	4601588	4601599
chr2_838	c	(TG)13cgcgcgtgtgcgtgcatgtgc(GT)6	59	4606765	4606823
chr2_839	p2	(TG)19	38	4609847	4609884
chr2_840	p3	(TGC)5	15	4623698	4623712
chr2_841	p2	(GT)11	22	4625541	4625562
chr2_842	c	(GT)24(AC)6	60	4639572	4639631
chr2_843	c	[illegible])12agggatgacaacctcccatttgaatggtcatgttgctggagag(GG[illegible]	82	4643478	4643559
chr2_844	p2	(CA)8	16	4643688	4643703
chr2_845	p2	(TG)6	12	4646009	4646020
chr2_846	p2	(TG)6	12	4649449	4649460
chr2_847	p3	(AAG)7	21	4652961	4652981
chr2_848	p2	(AC)7	14	4658760	4658773
chr2_849	p2	(TG)10	20	4660542	4660561
chr2_850	p2	(TG)6	12	4662190	4662201
chr2_851	p2	(GT)10	20	4662327	4662346
chr2_852	p2	(AC)9	18	4663196	4663213
chr2_853	p2	(TG)6	12	4676616	4676627
chr2_854	p2	(TG)11	22	4679529	4679550
chr2_855	p3	(AGC)5	15	4684534	4684548
chr2_856	c	atatatc(TA)6gatatatatagatatcgatatctatctgtgtgtatgtgtatgt	294	4684855	4685148
chr2_857	p3	(GTT)10	30	4685728	4685757
chr2_858	p2	(AC)9	18	4686563	4686580
chr2_859	p2	(GT)10	20	4686713	4686732
chr2_860	c	(AC)8tctcacacaca(CT)7	41	4689174	4689214
chr2_861	p2	(AC)6	12	4692233	4692244
chr2_862	p2	(TG)9	18	4693106	4693123
chr2_863	p2	(TG)27	54	4695952	4696005
chr2_864	p2	(GT)16	32	4696464	4696495
chr2_865	p2	(AC)7	14	4697668	4697681
chr2_866	p2	(TG)28	56	4700975	4701030
chr2_867	p2	(GT)31	62	4714440	4714501
chr2_868	p2	(GT)11	22	4714680	4714701
chr2_869	p4	(ATTT)6	24	4715578	4715601
chr2_870	p2	(AG)7	14	4723012	4723025

chr2_871	c	accgacagagaagcattgtccttgagggttggttgtggcctctctaaccatt[illegible]	134	4724453	4724586
chr2_872	p2	(CA)13	26	4725174	4725199
chr2_873	p2	(GT)11	22	4726629	4726650
chr2_874	p2	(AT)7	14	4730856	4730869
chr2_875	p2	(TG)6	12	4731415	4731426
chr2_876	p2	(GT)11	22	4739600	4739621
chr2_877	p3	(TGA)5	15	4741551	4741565
chr2_878	p2	(TG)17	34	4748516	4748549
chr2_879	p2	(GT)6	12	4750966	4750977
chr2_880	p2	(TG)14	28	4763370	4763397
chr2_881	p2	(GT)6	12	4774127	4774138
chr2_882	p2	(AC)6	12	4777938	4777949
chr2_883	p2	(AC)6	12	4780973	4780984
chr2_884	c*	(ATAC)6(AT)6ac(ACAT)5(AT)9*acat(AC)9	96	4789593	4789688
chr2_885	p2	(TG)7	14	4791103	4791116
chr2_886	c	(TGA)5ta(ATG)9	44	4821272	4821315
chr2_887	p5	(AAATC)5	25	4823290	4823314
chr2_888	p2	(AC)11	22	4837737	4837758
chr2_889	p2	(TA)6	12	4844596	4844607
chr2_890	p2	(GT)8	16	4844922	4844937
chr2_891	p2	(GT)19	38	4845900	4845937
chr2_892	p2	(AC)8	16	4879617	4879632
chr2_893	p2	(GT)16	32	4899295	4899326
chr2_894	p3	(CTC)10	30	4911097	4911126
chr2_895	p2	(TG)12	24	4922163	4922186
chr2_896	p2	(AC)6	12	4924998	4925009
chr2_897	p2	(GT)6	12	4927376	4927387
chr2_898	p2	(GT)8	16	4928415	4928430
chr2_899	p2	(GT)6	12	4928621	4928632
chr2_900	p2	(GT)8	16	4928930	4928945
chr2_901	p2	(GT)8	16	4929150	4929165
chr2_902	p2	(AC)19	38	4930356	4930393
chr2_903	p4	(CAAA)6	24	4937250	4937273
chr2_904	p2	(AC)22	44	4940486	4940529
chr2_905	p3	(TAT)8	24	4941299	4941322
chr2_906	p2	(GT)11	22	4955353	4955374
chr2_907	p2	(TC)7	14	4955949	4955962
chr2_908	p2	(AG)6	12	4960891	4960902
chr2_909	p2	(CT)7	14	4963146	4963159
chr2_910	p3	(CTC)5	15	4963303	4963317
chr2_911	p2	(TG)9	18	4964809	4964826
chr2_912	p2	(TG)17	34	4968477	4968510
chr2_913	p2	(TG)14	28	4969736	4969763
chr2_914	p3	(GAG)5	15	4973402	4973416
chr2_915	p2	(GA)11	22	4973689	4973710
chr2_916	p2	(GT)6	12	4974703	4974714
chr2_917	p2	(GT)15	30	4974852	4974881
chr2_918	p2	(GT)7	14	4975488	4975501
chr2_919	p2	(CA)7	14	4975761	4975774
chr2_920	p2	(AC)8	16	4980372	4980387
chr2_921	p4	(TCCA)5	20	4981546	4981565
chr2_922	p2	(CA)8	16	4989953	4989968

chr2_923	p2	(GT)10	20	4991193	4991212
chr2_924	p2	(TG)15	30	4991392	4991421
chr2_925	c	ggctgctgaactgactgagtgagcgtactgttcaaagcaagtgtagtatc(	83	4995166	4995248
chr2_926	c	(CT)6(CA)12	36	4996097	4996132
chr2_927	p2	(AC)10	20	4999641	4999660
chr2_928	p2	(GT)11	22	5000931	5000952
chr2_929	p2	(CA)9	18	5001642	5001659
chr2_930	p2	(TC)6	12	5005492	5005503
chr2_931	p2	(CA)12	24	5006632	5006655
chr2_932	p2	(GT)6	12	5009646	5009657
chr2_933	p2	(TG)7	14	5012684	5012697
chr2_934	p2	(TG)7	14	5014821	5014834
chr2_935	p2	(AC)20	40	5019348	5019387
chr2_936	p2	(AC)18	36	5023836	5023871
chr2_937	p2	(AT)7	14	5024267	5024280
chr2_938	p2	(GT)12	24	5027171	5027194
chr2_939	p2	(GT)6	12	5035471	5035482
chr2_940	p2	(TG)8	16	5039235	5039250
chr2_941	p2	(TG)12	24	5043982	5044005
chr2_942	p2	(TG)7	14	5045796	5045809
chr2_943	p2	(AC)20	40	5073232	5073271
chr2_944	p2	(GT)9	18	5079500	5079517
chr2_945	p2	(TG)6	12	5083996	5084007
chr2_946	p2	(TG)18	36	5084511	5084546
chr2_947	p2	(AC)10	20	5085412	5085431
chr2_948	p2	(TG)15	30	5088285	5088314
chr2_949	c	cacggacagtagaagccacagtgtttgtttcatgccactcgtgccgagcttg	174	5089313	5089486
chr2_950	p3	(TCT)5	15	5092069	5092083
chr2_951	p2	(AC)23	46	5097862	5097907
chr2_952	p2	(AG)6	12	5108971	5108982
chr2_953	p4	(AATA)5	20	5110888	5110907
chr2_954	p2	(TC)8	16	5121685	5121700
chr2_955	p2	(AC)19	38	5193464	5193501
chr2_956	p2	(TG)26	52	5194725	5194776
chr2_957	p4	(AATG)5	20	5196721	5196740
chr2_958	p2	(AC)6	12	5215655	5215666
chr2_959	p2	(AC)6	12	5215793	5215804
chr2_960	p2	(TG)8	16	5221596	5221611
chr2_961	p2	(AC)6	12	5227439	5227450
chr2_962	p2	(GT)28	56	5227626	5227681
chr2_963	p2	(TG)6	12	5237004	5237015
chr2_964	p2	(AC)7	14	5241527	5241540
chr2_965	p3	(GAG)6	18	5241696	5241713
chr2_966	p2	(CA)8	16	5241886	5241901
chr2_967	p2	(AC)27	54	5242050	5242103
chr2_968	p2	(AC)12	24	5244086	5244109
chr2_969	p3	(AGA)5	15	5261152	5261166
chr2_970	c	(AT)7gtatgtatatgta(TG)23	73	5271606	5271678
chr2_971	p2	(CA)10	20	5271827	5271846
chr2_972	p2	(AC)11	22	5276892	5276913
chr2_973	p2	(TG)6	12	5280583	5280594
chr2_974	p2	(CA)8	16	5283920	5283935

chr2_975	p2	(GT)6	12	5287730	5287741
chr2_976	p2	(TG)9	18	5291829	5291846
chr2_977	p2	(AC)6	12	5297012	5297023
chr2_978	p4	(TATC)5	20	5299894	5299913
chr2_979	p2	(CA)26	52	5300511	5300562
chr2_980	p2	(CA)18	36	5300754	5300789
chr2_981	p2	(TG)15	30	5303306	5303335
chr2_982	p2	(AC)8	16	5304268	5304283
chr2_983	p5	(AATAA)5	25	5305805	5305829
chr2_984	p2	(TG)6	12	5306169	5306180
chr2_985	p2	(AG)6	12	5306333	5306344
chr2_986	p2	(GT)22	44	5307567	5307610
chr2_987	p2	(AC)6	12	5314001	5314012
chr2_988	p2	(GT)26	52	5325720	5325771
chr2_989	p3	(TAT)6	18	5328505	5328522
chr2_990	p2	(CA)6	12	5337009	5337020
chr2_991	c	(TG)6(TA)6(CA)8	40	5338565	5338604
chr2_992	p2	(CA)15	30	5340446	5340475
chr2_993	p2	(GT)12	24	5341838	5341861
chr2_994	c	[illegible]tgtgat(TTA)5atatcattaatgataattaatactaatattggtcatacaaa	155	5342445	5342599
chr2_995	p2	(AC)12	24	5371529	5371552
chr2_996	p2	(TG)7	14	5372244	5372257
chr2_997	p2	(TG)7	14	5380416	5380429
chr2_998	p2	(CA)13	26	5382987	5383012
chr2_999	p2	(CA)7	14	5385112	5385125
chr2_1000	p2	(GT)8	16	5385555	5385570
chr2_1001	p2	(TG)6	12	5386484	5386495
chr2_1002	p2	(TA)8	16	5387904	5387919
chr2_1003	p2	(AC)16	32	5389269	5389300
chr2_1004	p2	(TG)17	34	5390725	5390758
chr2_1005	p2	(CT)7	14	5392584	5392597
chr2_1006	p2	(AT)6	12	5403009	5403020
chr2_1007	p3	(CCA)5	15	5407699	5407713
chr2_1008	p2	(TG)11	22	5410140	5410161
chr2_1009	p3	(AAG)6	18	5413709	5413726
chr2_1010	c	(CA)20(TACA)6	64	5417940	5418003
chr2_1011	p2	(AC)6	12	5418321	5418332
chr2_1012	p2	(GT)13	26	5421822	5421847
chr2_1013	p2	(TC)6	12	5426106	5426117
chr2_1014	p3	(ATT)5	15	5427365	5427379
chr2_1015	p2	(CA)9	18	5431600	5431617
chr2_1016	p4	(TCAT)7	28	5437353	5437380
chr2_1017	p2	(AG)9	18	5452335	5452352
chr2_1018	p2	(TA)6	12	5458649	5458660
chr2_1019	p2	(TC)14	28	5461560	5461587
chr2_1020	p2	(GT)17	34	5462641	5462674
chr2_1021	p2	(AC)20	40	5463362	5463401
chr2_1022	p2	(TG)19	38	5473091	5473128
chr2_1023	p2	(AC)8	16	5473310	5473325
chr2_1024	p2	(AC)17	34	5477632	5477665
chr2_1025	p2	(CA)7	14	5478264	5478277
chr2_1026	c	(TA)7tgta(TG)14	46	5484479	5484524

chr2_1027	p2	(CA)6	12	5486893	5486904
chr2_1028	p3	(TGG)5	15	5494731	5494745
chr2_1029	p2	(GA)7	14	5498059	5498072
chr2_1030	p3	(GAG)5	15	5502773	5502787
chr2_1031	p2	(GT)14	28	5502924	5502951
chr2_1032	p2	(GT)6	12	5505430	5505441
chr2_1033	p2	(AG)7	14	5507797	5507810
chr2_1034	p3	(TGA)5	15	5512365	5512379
chr2_1035	p2	(GT)11	22	5512981	5513002
chr2_1036	c	(CT)6g(TC)6	25	5515958	5515982
chr2_1037	p2	(TG)17	34	5519966	5519999
chr2_1038	p2	(TG)10	20	5520202	5520221
chr2_1039	p2	(AC)14	28	5524892	5524919
chr2_1040	p2	(AT)7	14	5529541	5529554
chr2_1041	c	ccttccagactggaggacgaccagaaccaagtaagaagacacctttaacc	142	5531963	5532104
chr2_1042	p3	(GGA)5	15	5535499	5535513
chr2_1043	p2	(AC)13	26	5537613	5537638
chr2_1044	p2	(AC)18	36	5539976	5540011
chr2_1045	p2	(TG)9	18	5541197	5541214
chr2_1046	p2	(GT)10	20	5541398	5541417
chr2_1047	p2	(CA)6	12	5542208	5542219
chr2_1048	p2	(CA)17	34	5542407	5542440
chr2_1049	p2	(GT)12	24	5546925	5546948
chr2_1050	p4	(ATGG)5	20	5552396	5552415
chr2_1051	p2	(GA)10	20	5557024	5557043
chr2_1052	p3	(AGA)7	21	5557275	5557295
chr2_1053	p2	(AG)9	18	5563063	5563080
chr2_1054	p2	(AC)6	12	5569985	5569996
chr2_1055	c	(TA)7(TG)14	42	5573229	5573270
chr2_1056	p2	(GT)9	18	5585576	5585593
chr2_1057	p2	(GA)10	20	5591489	5591508
chr2_1058	p2	(GT)6	12	5603102	5603113
chr2_1059	p2	(TG)23	46	5603253	5603298
chr2_1060	p2	(TG)6	12	5603762	5603773
chr2_1061	p5	(GGAGA)5	25	5605031	5605055
chr2_1062	p2	(GT)11	22	5607247	5607268
chr2_1063	p2	(TG)12	24	5607460	5607483
chr2_1064	c	caaggaaaaacatgttgcctgccttcatgtcattgcaaacatgcacacagt	119	5607727	5607845
chr2_1065	p2	(CA)7	14	5608978	5608991
chr2_1066	p3	(TAA)7	21	5609718	5609738
chr2_1067	p3	(TAA)5	15	5618309	5618323
chr2_1068	p2	(AC)8	16	5619915	5619930
chr2_1069	p2	(CA)9	18	5620196	5620213
chr2_1070	p3	(AAG)5	15	5622387	5622401
chr2_1071	p2	(TC)7	14	5628111	5628124
chr2_1072	p2	(CA)7	14	5630719	5630732
chr2_1073	p2	(CA)7	14	5631576	5631589
chr2_1074	p2	(GT)14	28	5641058	5641085
chr2_1075	p2	(CA)15	30	5648309	5648338
chr2_1076	p2	(TG)6	12	5648634	5648645
chr2_1077	p2	(CA)10	20	5655570	5655589
chr2_1078	p2	(CA)8	16	5655882	5655897

chr2_1079	p3	(TTG)7	21	5656491	5656511
chr2_1080	p2	(GT)13	26	5659045	5659070
chr2_1081	p2	(GT)14	28	5660027	5660054
chr2_1082	p2	(AC)18	36	5660433	5660468
chr2_1083	p2	(GT)12	24	5661517	5661540
chr2_1084	p3	(GTT)6	18	5663422	5663439
chr2_1085	p4	(TATT)5	20	5669852	5669871
chr2_1086	p2	(AC)11	22	5673755	5673776
chr2_1087	c	(CTA)7c(GTA)7gtggtagtagtggtg(GTA)5	73	5683208	5683280
chr2_1088	c	(CA)9tacaca(CT)13	50	5693706	5693755
chr2_1089	p4	(CTAC)6	24	5696614	5696637
chr2_1090	p2	(CA)23	46	5701740	5701785
chr2_1091	p2	(AC)14	28	5706496	5706523
chr2_1092	p3	(CCT)5	15	5711151	5711165
chr2_1093	c	(TTA)5ctattattattg(TTA)6	45	5711567	5711611
chr2_1094	c	(AC)12atacacact(CA)11	55	5712957	5713011
chr2_1095	p2	(TG)9	18	5719886	5719903
chr2_1096	p2	(CA)15	30	5720111	5720140
chr2_1097	p2	(GA)6	12	5721798	5721809
chr2_1098	p3	(TCT)5	15	5723737	5723751
chr2_1099	p2	(TG)18	36	5736290	5736325
chr2_1100	p2	(AC)25	50	5761176	5761225
chr2_1101	p2	(CA)17	34	5762449	5762482
chr2_1102	p3	(TAA)5	15	5764106	5764120
chr2_1103	c	(TG)15aat(GA)12	57	5768219	5768275
chr2_1104	c	(GA)6gcaagtgttgaaagccagagcagagactgaag(AC)12	68	5776512	5776579
chr2_1105	p2	(GT)9	18	5789614	5789631
chr2_1106	p2	(GT)23	46	5793263	5793308
chr2_1107	p5	(TTATG)7	35	5796772	5796806
chr2_1108	p2	(AG)7	14	5816150	5816163
chr2_1109	p2	(AG)6	12	5816697	5816708
chr2_1110	p2	(AG)7	14	5817948	5817961
chr2_1111	p2	(AC)6	12	5824020	5824031
chr2_1112	p2	(AC)6	12	5824293	5824304
chr2_1113	p2	(AC)6	12	5825613	5825624
chr2_1114	p3	(ATA)5	15	5827279	5827293
chr2_1115	p2	(CA)6	12	5830698	5830709
chr2_1116	p2	(AG)6	12	5837865	5837876
chr2_1117	p2	(GT)7	14	5843574	5843587
chr2_1118	c	(TG)10(GT)8	36	5844221	5844256
chr2_1119	p2	(GT)16	32	5844617	5844648
chr2_1120	p2	(TG)28	56	5844923	5844978
chr2_1121	p5	(TTGAT)5	25	5848559	5848583
chr2_1122	p2	(AC)7	14	5852393	5852406
chr2_1123	p2	(TG)19	38	5852535	5852572
chr2_1124	p2	(TG)6	12	5856310	5856321
chr2_1125	p3	(GTA)13	39	5869902	5869940
chr2_1126	p2	(CA)33	66	5870325	5870390
chr2_1127	p2	(AT)7	14	5874613	5874626
chr2_1128	p3	(CTA)10	30	5876186	5876215
chr2_1129	p3	(AGA)5	15	5879843	5879857
chr2_1130	p3	(AAT)7	21	5887772	5887792

chr2_1131	p2	(TG)13	26	5901772	5901797
chr2_1132	p2	(AC)7	14	5912681	5912694
chr2_1133	p2	(TA)7	14	5917165	5917178
chr2_1134	p2	(AC)17	34	5922917	5922950
chr2_1135	p2	(GT)21	42	5923186	5923227
chr2_1136	c	[illegible]gagaggcaagagacagaacagctgttcacctccacagtcctgtaaagcc	123	5923713	5923835
chr2_1137	p2	(GT)15	30	5924289	5924318
chr2_1138	p2	(TG)8	16	5924538	5924553
chr2_1139	p2	(CA)20	40	5925997	5926036
chr2_1140	p2	(AC)15	30	5926142	5926171
chr2_1141	p2	(AG)6	12	5926743	5926754
chr2_1142	p2	(TG)14	28	5928025	5928052
chr2_1143	p2	(GT)9	18	5930182	5930199
chr2_1144	p2	(GT)19	38	5930570	5930607
chr2_1145	p2	(TG)6	12	5930772	5930783
chr2_1146	p2	(AC)9	18	5932890	5932907
chr2_1147	c	(TC)10tgctgttcctgctc(CT)6	46	5933038	5933083
chr2_1148	p2	(CA)12	24	5935040	5935063
chr2_1149	p2	(AG)6	12	5936396	5936407
chr2_1150	p2	(TG)10	20	5936568	5936587
chr2_1151	p2	(AG)6	12	5936918	5936929
chr2_1152	p2	(AG)8	16	5937553	5937568
chr2_1153	p2	(AC)7	14	5942030	5942043
chr2_1154	p2	(GT)16	32	5942246	5942277
chr2_1155	p2	(AC)7	14	5943134	5943147
chr2_1156	p2	(GT)9	18	5943467	5943484
chr2_1157	p2	(CT)9	18	5943814	5943831
chr2_1158	c	(TG)17agacagagagagac(AG)7	62	5945291	5945352
chr2_1159	p2	(CA)6	12	5945502	5945513
chr2_1160	p2	(TG)7	14	5945742	5945755
chr2_1161	c	(TCA)6g(CT)9(CA)15	67	5952810	5952876
chr2_1162	p2	(CA)11	22	5954492	5954513
chr2_1163	c	[illegible])11tctggaacagaatgaaagatgacatcaaaactataaaaactgttcccc[illegible]	105	5955741	5955845
chr2_1164	p2	(CA)7	14	5955961	5955974
chr2_1165	p2	(AC)10	20	5957620	5957639
chr2_1166	p2	(GT)8	16	5957918	5957933
chr2_1167	p2	(TC)9	18	5958821	5958838
chr2_1168	p2	(TG)12	24	5958960	5958983
chr2_1169	p2	(CA)9	18	5960151	5960168
chr2_1170	p2	(CA)13	26	5963276	5963301
chr2_1171	p2	(CA)6	12	5963874	5963885
chr2_1172	p2	(TG)9	18	5964562	5964579
chr2_1173	p2	(AT)6	12	5965924	5965935
chr2_1174	p2	(GT)6	12	5967137	5967148
chr2_1175	p2	(AC)21	42	5969068	5969109
chr2_1176	p2	(AC)21	42	5969238	5969279
chr2_1177	p2	(TC)8	16	5975181	5975196
chr2_1178	p2	(CA)9	18	5976845	5976862
chr2_1179	p2	(GT)15	30	5976990	5977019
chr2_1180	p2	(AC)6	12	5979396	5979407
chr2_1181	p2	(AC)6	12	5983570	5983581
chr2_1182	p2	(CA)14	28	5995828	5995855

chr2_1183	p2	(GT)9	18	6006752	6006769
chr2_1184	p2	(AC)14	28	6014198	6014225
chr2_1185	p2	(CT)6	12	6017048	6017059
chr2_1186	p2	(AG)7	14	6018156	6018169
chr2_1187	p2	(GT)10	20	6018330	6018349
chr2_1188	p2	(TG)8	16	6039280	6039295
chr2_1189	p5	(TATGT)25	125	6066415	6066539
chr2_1190	p3	(AGG)5	15	6067265	6067279
chr2_1191	p2	(TG)6	12	6094650	6094661
chr2_1192	p2	(TG)15	30	6108045	6108074
chr2_1193	c	(TC)6g(CT)12cacacacat(AC)6	58	6108489	6108546
chr2_1194	p2	(CA)15	30	6111456	6111485
chr2_1195	c	(AG)6aaagagagacagc(GA)20cagagagagaa(AG)8	92	6122622	6122713
chr2_1196	p3	(ATA)5	15	6122981	6122995
chr2_1197	p2	(TG)14	28	6126824	6126851
chr2_1198	p4	(ATGG)5	20	6130197	6130216
chr2_1199	p2	(GT)29	58	6141368	6141425
chr2_1200	p2	(GT)27	54	6141527	6141580
chr2_1201	p2	(CT)6	12	6150473	6150484
chr2_1202	p2	(TG)7	14	6150959	6150972
chr2_1203	p2	(CA)6	12	6153690	6153701
chr2_1204	p3	(ATA)5	15	6162151	6162165
chr2_1205	p2	(TG)6	12	6167702	6167713
chr2_1206	p3	(ATA)5	15	6167824	6167838
chr2_1207	p2	(CA)6	12	6176867	6176878
chr2_1208	p3	(TAA)6	18	6183760	6183777
chr2_1209	p2	(TG)6	12	6190227	6190238
chr2_1210	c	(TG)14(AG)35	98	6192965	6193062
chr2_1211	p5	(CTCTC)5	25	6194717	6194741
chr2_1212	p2	(TG)13	26	6204929	6204954
chr2_1213	p3	(TGA)5	15	6205980	6205994
chr2_1214	p5	(CTTGA)6	30	6209760	6209789
chr2_1215	p4	(TTAT)5	20	6236706	6236725
chr2_1216	p2	(TC)9	18	6237271	6237288
chr2_1217	p2	(TC)6	12	6272656	6272667
chr2_1218	p2	(AC)13	26	6290139	6290164
chr2_1219	p3	(ATC)5	15	6292398	6292412
chr2_1220	p3	(GAA)14	42	6296310	6296351
chr2_1221	p2	(TG)7	14	6296877	6296890
chr2_1222	p3	(TTA)5	15	6323902	6323916
chr2_1223	p2	(TG)16	32	6324195	6324226
chr2_1224	p2	(TG)15	30	6326520	6326549
chr2_1225	p2	(TG)7	14	6330819	6330832
chr2_1226	p2	(TC)7	14	6331166	6331179
chr2_1227	p3	(TTG)6	18	6333004	6333021
chr2_1228	p4	(CATC)6	24	6334286	6334309
chr2_1229	p2	(AC)21	42	6335824	6335865
chr2_1230	p2	(TG)14	28	6345992	6346019
chr2_1231	p2	(TG)6	12	6350968	6350979
chr2_1232	c	ggattgtgtttgctatgtgaaaaacctttgtctctaaacccatcgtgcatgctt[illegible]	126	6352097	6352222
chr2_1233	p2	(TC)6	12	6354410	6354421
chr2_1234	p2	(CT)9	18	6359156	6359173

chr2_1235	p2	(AT)7	14	6371127	6371140
chr2_1236	p2	(CT)7	14	6377205	6377218
chr2_1237	c	(ACAG)5(AC)14	48	6382098	6382145
chr2_1238	c	(TG)8(AG)7	30	6383579	6383608
chr2_1239	p2	(AC)7	14	6384844	6384857
chr2_1240	p2	(GT)15	30	6386003	6386032
chr2_1241	p2	(AC)17	34	6390017	6390050
chr2_1242	p2	(TG)7	14	6396360	6396373
chr2_1243	p2	(GT)22	44	6400437	6400480
chr2_1244	p2	(AG)7	14	6404003	6404016
chr2_1245	p2	(GT)6	12	6413023	6413034
chr2_1246	p2	(AC)12	24	6414406	6414429
chr2_1247	p2	(CA)13	26	6414837	6414862
chr2_1248	p3	(AAG)18	54	6420298	6420351
chr2_1249	p2	(GT)6	12	6425667	6425678
chr2_1250	p2	(GT)13	26	6440692	6440717
chr2_1251	c	(TG)28tatattgtgattgcgtagcttgttgcaatgtatttgctgta(TG)27	151	6447486	6447636
chr2_1252	p4	(AATA)5	20	6448425	6448444
chr2_1253	p2	(CA)18	36	6452714	6452749
chr2_1254	c	(TG)6(AG)27	66	6460619	6460684
chr2_1255	p2	(GT)7	14	6460860	6460873
chr2_1256	p2	(AC)13	26	6461352	6461377
chr2_1257	p2	(GC)6	12	6465400	6465411
chr2_1258	p2	(GT)11	22	6466795	6466816
chr2_1259	p2	(AT)6	12	6474058	6474069
chr2_1260	p2	(TG)7	14	6487674	6487687
chr2_1261	p2	(AC)23	46	6489977	6490022
chr2_1262	p2	(GT)6	12	6498403	6498414
chr2_1263	p2	(TG)10	20	6499216	6499235
chr2_1264	p3	(AAT)7	21	6506127	6506147
chr2_1265	p2	(TG)16	32	6509696	6509727
chr2_1266	p2	(CA)9	18	6511565	6511582
chr2_1267	c	(AT)8(GT)11atagg(GA)7	57	6513149	6513205
chr2_1268	p4	(CCTA)5	20	6523871	6523890
chr2_1269	p2	(TG)8	16	6527372	6527387
chr2_1270	p2	(CA)6	12	6527549	6527560
chr2_1271	p2	(AC)6	12	6528814	6528825
chr2_1272	p2	(CA)13	26	6536376	6536401
chr2_1273	p2	(TA)9	18	6556393	6556410
chr2_1274	p2	(AC)18	36	6558371	6558406
chr2_1275	p3	(TTG)9	27	6558602	6558628
chr2_1276	p2	(TG)25	50	6560944	6560993
chr2_1277	p2	(AC)7	14	6561161	6561174
chr2_1278	p3	(ATA)5	15	6561536	6561550
chr2_1279	p3	(TTA)6	18	6564017	6564034
chr2_1280	p2	(AC)8	16	6572790	6572805
chr2_1281	p3	(AAG)5	15	6580863	6580877
chr2_1282	p3	(AAC)6	18	6585895	6585912
chr2_1283	p2	(AC)8	16	6590741	6590756
chr2_1284	p2	(CA)21	42	6596480	6596521
chr2_1285	p3	(TTA)11	33	6685078	6685110
chr2_1286	p2	(AC)14	28	6717651	6717678

chr2_1287	p2	(TG)7	14	6722680	6722693
chr2_1288	p2	(AC)15	30	6727221	6727250
chr2_1289	p2	(GT)6	12	6728944	6728955
chr2_1290	p2	(AC)6	12	6729578	6729589
chr2_1291	p4	(TGTT)5	20	6737305	6737324
chr2_1292	p2	(GT)7	14	6750134	6750147
chr2_1293	p2	(CA)6	12	6755325	6755336
chr2_1294	p2	(TA)7	14	6757788	6757801
chr2_1295	p2	(AC)6	12	6760446	6760457
chr2_1296	p2	(AT)13	26	6778143	6778168
chr2_1297	p3	(TTA)6	18	6779693	6779710
chr2_1298	p2	(TC)6	12	6794046	6794057
chr2_1299	p2	(CA)6	12	6795427	6795438
chr2_1300	p2	(TG)10	20	6797715	6797734
chr2_1301	p3	(ATG)5	15	6804128	6804142
chr2_1302	p3	(ATT)8	24	6804564	6804587
chr2_1303	p2	(AC)10	20	6817785	6817804
chr2_1304	p4	(AATT)5	20	6818343	6818362
chr2_1305	c	(GA)9a(AG)6	31	6826877	6826907
chr2_1306	p2	(AC)6	12	6827738	6827749
chr2_1307	p3	(TAA)5	15	6835026	6835040
chr2_1308	p2	(TG)6	12	6836005	6836016
chr2_1309	p4	(ATAG)5	20	6837847	6837866
chr2_1310	c	ttttcttttttgttattttttgtgacattgaccttgtccgtaatcctgctcagcaaa[illegible]	97	6849817	6849913
chr2_1311	p2	(AC)8	16	6851464	6851479
chr2_1312	p5	(CTCCT)8	40	6854288	6854327
chr2_1313	p5	(AGTTG)5	25	6857517	6857541
chr2_1314	p2	(AC)6	12	6867592	6867603
chr2_1315	p2	(CT)20	40	6869253	6869292
chr2_1316	p2	(TG)15	30	6885544	6885573
chr2_1317	p2	(TG)18	36	6885720	6885755
chr2_1318	p2	(AC)6	12	6906659	6906670
chr2_1319	p2	(TG)7	14	6927471	6927484
chr2_1320	p2	(TG)6	12	6940382	6940393
chr2_1321	p4	(ATGG)5	20	6941673	6941692
chr2_1322	p3	(AAT)5	15	6967481	6967495
chr2_1323	p4	(AAAT)5	20	6970979	6970998
chr2_1324	p2	(TG)7	14	6979626	6979639
chr2_1325	p2	(GA)6	12	6983751	6983762
chr2_1326	p2	(TG)13	26	6986801	6986826
chr2_1327	p2	(AT)7	14	7022350	7022363
chr2_1328	p2	(TG)20	40	7036853	7036892
chr2_1329	p2	(CA)17	34	7042301	7042334
chr2_1330	p3	(TTA)7	21	7046099	7046119
chr2_1331	p2	(AC)7	14	7048148	7048161
chr2_1332	p2	(CA)8	16	7061145	7061160
chr2_1333	p2	(AT)6	12	7063290	7063301
chr2_1334	p2	(AC)9	18	7119562	7119579
chr2_1335	p3	(TCT)5	15	7126793	7126807
chr2_1336	p3	(AAG)5	15	7140447	7140461
chr2_1337	p2	(TG)17	34	7141288	7141321
chr2_1338	p2	(CA)10	20	7144621	7144640

chr2_1339	p3	(AAT)5	15	7151767	7151781
chr2_1340	p2	(TG)6	12	7157474	7157485
chr2_1341	p4	(TTTA)5	20	7159960	7159979
chr2_1342	p2	(TG)6	12	7178341	7178352
chr2_1343	c	(TA)6gtatatacag(CA)19	60	7180648	7180707
chr2_1344	p2	(AG)11	22	7183586	7183607
chr2_1345	p3	(AAG)5	15	7203710	7203724
chr2_1346	p2	(TA)6	12	7205127	7205138
chr2_1347	p2	(TG)6	12	7226350	7226361
chr2_1348	c	(GT)7ttttgtgtgagaccttgtttatatacttgtggggatggag(CA)15	84	7248497	7248580
chr2_1349	p2	(AT)6	12	7266106	7266117
chr2_1350	p2	(AG)11	22	7279366	7279387
chr2_1351	p2	(GT)10	20	7295805	7295824
chr2_1352	p2	(TG)6	12	7297339	7297350
chr2_1353	p2	(GT)10	20	7310091	7310110
chr2_1354	p3	(TAT)6	18	7310682	7310699
chr2_1355	p6	(GAATCA)5	30	7319079	7319108
chr2_1356	p2	(TA)6	12	7319404	7319415
chr2_1357	p2	(TG)10	20	7323078	7323097
chr2_1358	p2	(CA)7	14	7329729	7329742
chr2_1359	p2	(AC)21	42	7348093	7348134
chr2_1360	p2	(GT)6	12	7354326	7354337
chr2_1361	p2	(AC)7	14	7371508	7371521
chr2_1362	p2	(CA)15	30	7374158	7374187
chr2_1363	p2	(CA)6	12	7374960	7374971
chr2_1364	p2	(CA)26	52	7399260	7399311
chr2_1365	p2	(AC)13	26	7421993	7422018
chr2_1366	p2	(CA)16	32	7432042	7432073
chr2_1367	p2	(TG)12	24	7447355	7447378
chr2_1368	p2	(AT)6	12	7448971	7448982
chr2_1369	p2	(AT)8	16	7449112	7449127
chr2_1370	p2	(TG)9	18	7456485	7456502
chr2_1371	p2	(GT)13	26	7476852	7476877
chr2_1372	p2	(CA)6	12	7478673	7478684
chr2_1373	p3	(ATT)5	15	7484826	7484840
chr2_1374	p2	(AC)9	18	7486052	7486069
chr2_1375	p2	(AC)8	16	7496757	7496772
chr2_1376	p2	(TG)6	12	7497593	7497604
chr2_1377	c	(GA)12tggagtgcgacg(GA)7	50	7498898	7498947
chr2_1378	p2	(GT)17	34	7502462	7502495
chr2_1379	p2	(TG)9	18	7512844	7512861
chr2_1380	p2	(TG)9	18	7519636	7519653
chr2_1381	p4	(ATGG)5	20	7522178	7522197
chr2_1382	p3	(ATT)5	15	7522378	7522392
chr2_1383	p2	(GT)19	38	7524327	7524364
chr2_1384	p2	(TA)6	12	7535576	7535587
chr2_1385	c	(TAT)26tannnnnnnnnnnnnnn(TTA)6	114	7565339	7565452
chr2_1386	p4	(GGAT)5	20	7577501	7577520
chr2_1387	p2	(AC)11	22	7580156	7580177
chr2_1388	p2	(TG)13	26	7584320	7584345
chr2_1389	p2	(TG)9	18	7584611	7584628
chr2_1390	p2	(GT)8	16	7599715	7599730

chr2_1391	p2	(GT)18	36	7605104	7605139
chr2_1392	p2	(TG)9	18	7620845	7620862
chr2_1393	p3	(AAG)6	18	7626904	7626921
chr2_1394	p2	(GT)27	54	7646806	7646859
chr2_1395	p2	(AG)8	16	7656828	7656843
chr2_1396	c	(TG)8(AG)11	38	7664683	7664720
chr2_1397	p3	(ATA)5	15	7665014	7665028
chr2_1398	p3	(TTG)5	15	7673764	7673778
chr2_1399	p3	(AAT)5	15	7678527	7678541
chr2_1400	p2	(AC)6	12	7688199	7688210
chr2_1401	p2	(TG)7	14	7703172	7703185
chr2_1402	p2	(TG)6	12	7717821	7717832
chr2_1403	p2	(TG)6	12	7721440	7721451
chr2_1404	p3	(GAT)5	15	7724422	7724436
chr2_1405	p3	(CAG)5	15	7726396	7726410
chr2_1406	c	(TG)7(TA)10	34	7736425	7736458
chr2_1407	p3	(AGG)5	15	7739542	7739556
chr2_1408	p3	(AGG)6	18	7799375	7799392
chr2_1409	p2	(TC)16	32	7805257	7805288
chr2_1410	p2	(CA)16	32	7808415	7808446
chr2_1411	p2	(AC)9	18	7810258	7810275
chr2_1412	p2	(AC)9	18	7815541	7815558
chr2_1413	p3	(ATA)6	18	7834652	7834669
chr2_1414	p2	(TG)6	12	7843592	7843603
chr2_1415	p3	(AGC)8	24	7846806	7846829
chr2_1416	p2	(CA)7	14	7854810	7854823
chr2_1417	p2	(AC)6	12	7856192	7856203
chr2_1418	p5	(AAATC)16	80	7863551	7863630
chr2_1419	p4	(TTTG)5	20	7867419	7867438
chr2_1420	p4	(TTTG)5	20	7869696	7869715
chr2_1421	p2	(AC)8	16	7876215	7876230
chr2_1422	p5	(ACGGC)6	30	7886957	7886986
chr2_1423	p2	(CA)10	20	7888504	7888523
chr2_1424	p2	(TG)42	84	7888740	7888823
chr2_1425	p2	(AT)7	14	7909315	7909328
chr2_1426	p2	(CA)10	20	7918817	7918836
chr2_1427	p3	(TAT)7	21	7924435	7924455
chr2_1428	p2	(TG)24	48	7941509	7941556
chr2_1429	p2	(CA)22	44	7973785	7973828
chr2_1430	p2	(TG)6	12	7985218	7985229
chr2_1431	p2	(GT)11	22	7996023	7996044
chr2_1432	p2	(CA)8	16	7998081	7998096
chr2_1433	p2	(GT)7	14	7998557	7998570
chr2_1434	p2	(TG)24	48	8003036	8003083
chr2_1435	p2	(GT)8	16	8009470	8009485
chr2_1436	p2	(CT)7	14	8017131	8017144
chr2_1437	p2	(AC)14	28	8017251	8017278
chr2_1438	p2	(AC)14	28	8022411	8022438
chr2_1439	p2	(CT)7	14	8022690	8022703
chr2_1440	p2	(CA)6	12	8022807	8022818
chr2_1441	p3	(CAG)6	18	8025425	8025442
chr2_1442	p2	(CA)15	30	8026186	8026215

chr2_1443	p2	(GT)6	12	8027875	8027886
chr2_1444	p4	(TTCA)5	20	8028160	8028179
chr2_1445	c	(TG)8cgccaggcctatggcatgtctgag(GT)6	52	8029222	8029273
chr2_1446	p2	(TG)16	32	8033407	8033438
chr2_1447	p2	(CT)6	12	8044259	8044270
chr2_1448	p2	(TG)6	12	8054340	8054351
chr2_1449	p2	(AG)6	12	8062804	8062815
chr2_1450	p2	(TG)9	18	8063627	8063644
chr2_1451	p2	(TG)13	26	8068096	8068121
chr2_1452	c	cgtgcgtgtgcgtgcgtgcgtgtgcgtgtgtgcgtgtgcgtgtgcgtgtgc(	85	8075800	8075884
chr2_1453	p2	(AC)6	12	8077753	8077764
chr2_1454	p3	(GGT)6	18	8079495	8079512
chr2_1455	p2	(AG)8	16	8080636	8080651
chr2_1456	p2	(TG)7	14	8086961	8086974
chr2_1457	p2	(TG)8	16	8090906	8090921
chr2_1458	p2	(TG)24	48	8091884	8091931
chr2_1459	p2	(GT)7	14	8099670	8099683
chr2_1460	p2	(AC)27	54	8118957	8119010
chr2_1461	p2	(CA)12	24	8122285	8122308
chr2_1462	p2	(AC)11	22	8130310	8130331
chr2_1463	p2	(TG)13	26	8134989	8135014
chr2_1464	p3	(CAT)7	21	8137430	8137450
chr2_1465	p2	(TG)13	26	8137986	8138011
chr2_1466	p2	(TG)16	32	8138159	8138190
chr2_1467	p2	(GT)6	12	8146411	8146422
chr2_1468	p2	(TG)8	16	8154689	8154704
chr2_1469	p2	(CA)11	22	8160225	8160246
chr2_1470	p3	(CAG)5	15	8161238	8161252
chr2_1471	p3	(TGC)5	15	8163231	8163245
chr2_1472	p2	(GT)7	14	8177708	8177721
chr2_1473	p2	(TC)11	22	8178602	8178623
chr2_1474	p2	(CA)11	22	8178939	8178960
chr2_1475	c	(GT)25(GA)14	78	8179184	8179261
chr2_1476	c	(AG)40g(GA)6	93	8187402	8187494
chr2_1477	p2	(GA)8	16	8210511	8210526
chr2_1478	p3	(TGA)5	15	8237892	8237906
chr2_1479	p2	(TA)6	12	8238475	8238486
chr2_1480	p2	(AT)7	14	8244060	8244073
chr2_1481	p3	(GTA)7	21	8244184	8244204
chr2_1482	p2	(GT)7	14	8245031	8245044
chr2_1483	p2	(CA)16	32	8269407	8269438
chr2_1484	p2	(TG)13	26	8269684	8269709
chr2_1485	p3	(TAC)5	15	8277023	8277037
chr2_1486	p3	(TAA)5	15	8285872	8285886
chr2_1487	p2	(TG)7	14	8295648	8295661
chr2_1488	p3	(GCA)5	15	8298123	8298137
chr2_1489	p2	(CA)7	14	8301533	8301546
chr2_1490	p2	(AC)6	12	8311918	8311929
chr2_1491	p3	(TGA)9	27	8313089	8313115
chr2_1492	p2	(CA)10	20	8315966	8315985
chr2_1493	p2	(AC)15	30	8327504	8327533
chr2_1494	c	6aaatcgctttatacgtgcagattgcgcagcacgcatttgtgaacctcatg(.	76	8348530	8348605

chr2_1495	p4	(AAAT)6	24	8358091	8358114
chr2_1496	c	(TG)18tatac(AT)7	55	8361835	8361889
chr2_1497	p2	(TC)6	12	8362366	8362377
chr2_1498	p4	(TTTA)5	20	8366270	8366289
chr2_1499	p2	(TG)6	12	8388033	8388044
chr2_1500	p2	(TG)6	12	8403102	8403113
chr2_1501	p2	(CA)11	22	8404094	8404115
chr2_1502	p3	(ATA)5	15	8408875	8408889
chr2_1503	p2	(AC)25	50	8412701	8412750
chr2_1504	p2	(AC)8	16	8415632	8415647
chr2_1505	p5	(TATTC)7	35	8423783	8423817
chr2_1506	p2	(CA)9	18	8425552	8425569
chr2_1507	p2	(CT)8	16	8453533	8453548
chr2_1508	p2	(GT)13	26	8458574	8458599
chr2_1509	p3	(GTG)5	15	8461297	8461311
chr2_1510	p2	(CA)12	24	8461861	8461884
chr2_1511	p2	(CA)16	32	8463700	8463731
chr2_1512	p2	(AT)7	14	8466940	8466953
chr2_1513	p3	(TTA)8	24	8477745	8477768
chr2_1514	p4	(ATTG)5	20	8479932	8479951
chr2_1515	p2	(CT)6	12	8489095	8489106
chr2_1516	p2	(TG)7	14	8504897	8504910
chr2_1517	p2	(CA)20	40	8505953	8505992
chr2_1518	p2	(AC)24	48	8506645	8506692
chr2_1519	p2	(TG)14	28	8507516	8507543
chr2_1520	p2	(TG)6	12	8516781	8516792
chr2_1521	p2	(GT)18	36	8540693	8540728
chr2_1522	p5	(TATTA)5	25	8545742	8545766
chr2_1523	p2	(TC)11	22	8549414	8549435
chr2_1524	p2	(GT)8	16	8564754	8564769
chr2_1525	p2	(TG)8	16	8589265	8589280
chr2_1526	p2	(TG)8	16	8593488	8593503
chr2_1527	c	(AC)12t(CA)31	87	8595815	8595901
chr2_1528	p2	(CA)13	26	8598818	8598843
chr2_1529	p2	(TA)6	12	8604362	8604373
chr2_1530	p3	(ATT)6	18	8608612	8608629
chr2_1531	p2	(AG)7	14	8608796	8608809
chr2_1532	c	(AC)6caagacaggtttg(AT)8	41	8609751	8609791
chr2_1533	p2	(AC)8	16	8610855	8610870
chr2_1534	p2	(CT)6	12	8617657	8617668
chr2_1535	p2	(AT)7	14	8618175	8618188
chr2_1536	p2	(CA)7	14	8633950	8633963
chr2_1537	p3	(TTA)5	15	8648181	8648195
chr2_1538	p2	(CA)8	16	8649930	8649945
chr2_1539	p2	(CA)29	58	8655764	8655821
chr2_1540	p2	(GA)25	50	8656085	8656134
chr2_1541	p3	(AGA)5	15	8665056	8665070
chr2_1542	p4	(TATC)5	20	8667394	8667413
chr2_1543	p2	(TA)10	20	8693803	8693822
chr2_1544	p3	(ATA)5	15	8695627	8695641
chr2_1545	p2	(AC)6	12	8706583	8706594
chr2_1546	p2	(AC)8	16	8711983	8711998

chr2_1547	p3	(TTG)5	15	8721073	8721087
chr2_1548	p3	(TTA)5	15	8737170	8737184
chr2_1549	p3	(ACA)5	15	8740538	8740552
chr2_1550	p3	(TAA)6	18	8765344	8765361
chr2_1551	c*	(CA)15(ACA)9*	54	8766668	8766721
chr2_1552	p2	(CA)7	14	8767105	8767118
chr2_1553	p2	(TG)23	46	8774057	8774102
chr2_1554	p2	(TG)10	20	8776059	8776078
chr2_1555	p2	(CA)10	20	8802933	8802952
chr2_1556	p3	(ATT)5	15	8818028	8818042
chr2_1557	p2	(CA)13	26	8821956	8821981
chr2_1558	p2	(CA)12	24	8822206	8822229
chr2_1559	c	catgtcattgatgcatcaagagacgtggtgggatgttcttcacgtaaatgaga	129	8836952	8837080
chr2_1560	p2	(AG)7	14	8853533	8853546
chr2_1561	p2	(GT)8	16	8856997	8857012
chr2_1562	p2	(GT)11	22	8869467	8869488
chr2_1563	p2	(CA)8	16	8873796	8873811
chr2_1564	p2	(CA)7	14	8874081	8874094
chr2_1565	c	(GT)15gc(GT)9	50	8875010	8875059
chr2_1566	p2	(AC)9	18	8884769	8884786
chr2_1567	p2	(CA)16	32	8885431	8885462
chr2_1568	p2	(TG)7	14	8892537	8892550
chr2_1569	p2	(AC)22	44	8900855	8900898
chr2_1570	p2	(GT)6	12	8901981	8901992
chr2_1571	p2	(GT)12	24	8905187	8905210
chr2_1572	p4	(TTTA)5	20	8906506	8906525
chr2_1573	p2	(GT)7	14	8925759	8925772
chr2_1574	p2	(AG)6	12	8935051	8935062
chr2_1575	p3	(TGC)5	15	8936359	8936373
chr2_1576	p2	(GT)12	24	8939112	8939135
chr2_1577	p3	(CAA)7	21	8945240	8945260
chr2_1578	p2	(GT)18	36	8950094	8950129
chr2_1579	p2	(GT)17	34	8951887	8951920
chr2_1580	p2	(AC)8	16	8952194	8952209
chr2_1581	p2	(GT)10	20	8954046	8954065
chr2_1582	p2	(CA)12	24	8954866	8954889
chr2_1583	p2	(TG)14	28	8956076	8956103
chr2_1584	p2	(TG)8	16	8956530	8956545
chr2_1585	p3	(ATA)7	21	8964077	8964097
chr2_1586	p2	(AC)6	12	8965247	8965258
chr2_1587	p2	(AT)6	12	8969045	8969056
chr2_1588	p2	(TC)6	12	8973938	8973949
chr2_1589	p3	(ATT)8	24	8980172	8980195
chr2_1590	p2	(TG)6	12	8986682	8986693
chr2_1591	p2	(TG)16	32	8991785	8991816
chr2_1592	p2	(TG)9	18	8993519	8993536
chr2_1593	p4	(TTTA)5	20	8998365	8998384
chr2_1594	p3	(ACA)6	18	9000296	9000313
chr2_1595	p2	(AC)10	20	9001169	9001188
chr2_1596	c	(AT)7t(TA)7	29	9002386	9002414
chr2_1597	p3	(AGG)5	15	9005096	9005110
chr2_1598	p2	(GT)7	14	9005492	9005505

chr2_1599	p3	(CTC)5	15	9007521	9007535
chr2_1600	p2	(AC)11	22	9009333	9009354
chr2_1601	c	(AC)11ctaacagcac(CA)7	46	9009629	9009674
chr2_1602	c	(TG)14tag(GT)7	45	9013009	9013053
chr2_1603	p2	(TG)13	26	9013234	9013259
chr2_1604	p2	(GT)18	36	9013852	9013887
chr2_1605	p2	(CA)12	24	9014083	9014106
chr2_1606	p3	(CTT)5	15	9015780	9015794
chr2_1607	p3	(ATA)5	15	9019656	9019670
chr2_1608	p2	(AG)6	12	9023989	9024000
chr2_1609	p2	(GT)18	36	9028726	9028761
chr2_1610	p3	(AAT)6	18	9031865	9031882
chr2_1611	p2	(TG)20	40	9034134	9034173
chr2_1612	p3	(AAT)5	15	9039030	9039044
chr2_1613	p2	(AC)7	14	9050809	9050822
chr2_1614	p2	(AC)6	12	9066063	9066074
chr2_1615	p3	(TTA)5	15	9067772	9067786
chr2_1616	c	(TG)15(CG)7	44	9070436	9070479
chr2_1617	p2	(GT)7	14	9070597	9070610
chr2_1618	p3	(GGT)5	15	9082887	9082901
chr2_1619	p3	(ATA)6	18	9085223	9085240
chr2_1620	p3	(ATA)6	18	9090982	9090999
chr2_1621	p2	(GT)8	16	9110559	9110574
chr2_1622	p2	(CA)13	26	9129012	9129037
chr2_1623	p5	(CAAAT)6	30	9135494	9135523
chr2_1624	p3	(TAC)5	15	9141005	9141019
chr2_1625	c	(TC)7acacacacacag(AC)14	54	9145629	9145682
chr2_1626	p3	(ATA)12	36	9150219	9150254
chr2_1627	p2	(AC)13	26	9152074	9152099
chr2_1628	p3	(ATT)5	15	9153290	9153304
chr2_1629	p4	(TCTA)12	48	9154116	9154163
chr2_1630	p2	(AT)7	14	9160637	9160650
chr2_1631	p2	(AC)14	28	9171159	9171186
chr2_1632	p3	(AAT)5	15	9182281	9182295
chr2_1633	p2	(AG)8	16	9191666	9191681
chr2_1634	p3	(AAT)5	15	9196425	9196439
chr2_1635	p2	(AC)12	24	9203232	9203255
chr2_1636	p4	(AAAT)6	24	9206990	9207013
chr2_1637	c	(AGAGC)5(AGAGA)5agagc(AGAGA)9	100	9231287	9231386
chr2_1638	p2	(GT)20	40	9231974	9232013
chr2_1639	p3	(TGG)5	15	9237285	9237299
chr2_1640	p4	(TTTG)5	20	9239288	9239307
chr2_1641	c	gtttgacaggctgagcagcagacacaggggtccacagcagggtgtatgtg	88	9252706	9252793
chr2_1642	p2	(AC)7	14	9254641	9254654
chr2_1643	c	(TG)8tc(TG)11	40	9280685	9280724
chr2_1644	c	(TG)8cgcgtgcgc(GT)8	41	9300876	9300916
chr2_1645	c	(CT)19(GT)12	62	9314235	9314296
chr2_1646	p2	(AC)8	16	9328538	9328553
chr2_1647	p2	(TG)11	22	9328834	9328855
chr2_1648	p4	(ATGG)7	28	9346430	9346457
chr2_1649	p2	(TG)12	24	9357960	9357983
chr2_1650	p2	(TG)18	36	9361825	9361860

chr2_1651	p2	(TG)16	32	9363828	9363859
chr2_1652	p2	(TG)6	12	9366306	9366317
chr2_1653	p2	(CA)12	24	9369990	9370013
chr2_1654	p2	(GT)6	12	9370554	9370565
chr2_1655	p2	(TG)20	40	9370837	9370876
chr2_1656	p2	(TG)6	12	9380519	9380530
chr2_1657	p2	(AC)6	12	9386609	9386620
chr2_1658	c	aaggggagtttttcctccccactgttgccaagtgcttgctcaagagggattt	157	9390377	9390533
chr2_1659	p4	(TCCA)8	32	9399419	9399450
chr2_1660	p2	(TG)10	20	9402258	9402277
chr2_1661	p2	(TC)7	14	9404770	9404783
chr2_1662	p2	(AC)6	12	9410919	9410930
chr2_1663	p2	(AC)10	20	9429520	9429539
chr2_1664	p2	(CT)7	14	9429785	9429798
chr2_1665	p2	(TG)6	12	9430381	9430392
chr2_1666	p4	(TCAT)5	20	9442646	9442665
chr2_1667	p2	(AG)6	12	9443857	9443868
chr2_1668	c	(CA)18t(AC)7gcatgcacacaa(AC)7	77	9445248	9445324
chr2_1669	p2	(CA)9	18	9450972	9450989
chr2_1670	p3	(CCA)5	15	9456247	9456261
chr2_1671	p3	(CAT)5	15	9456488	9456502
chr2_1672	p3	(ATA)7	21	9461239	9461259
chr2_1673	p2	(GT)9	18	9462055	9462072
chr2_1674	p2	(TG)6	12	9471197	9471208
chr2_1675	p5	(TATAC)10	50	9473660	9473709
chr2_1676	p2	(TG)9	18	9476909	9476926
chr2_1677	p4	(ATCC)5	20	9484171	9484190
chr2_1678	p2	(AT)9	18	9490609	9490626
chr2_1679	p2	(AT)9	18	9498905	9498922
chr2_1680	p2	(GT)7	14	9502362	9502375
chr2_1681	p2	(TG)16	32	9503586	9503617
chr2_1682	p3	(GCA)5	15	9512389	9512403
chr2_1683	p2	(AC)7	14	9514803	9514816
chr2_1684	p3	(ATC)5	15	9516818	9516832
chr2_1685	p3	(CAC)8	24	9518329	9518352
chr2_1686	p2	(TG)11	22	9574392	9574413
chr2_1687	p2	(GT)24	48	9581258	9581305
chr2_1688	p2	(AC)12	24	9609372	9609395
chr2_1689	p3	(ACT)6	18	9618251	9618268
chr2_1690	p2	(GA)6	12	9625350	9625361
chr2_1691	p2	(GT)6	12	9655260	9655271
chr2_1692	p3	(TAA)7	21	9660523	9660543
chr2_1693	p3	(TAA)7	21	9663516	9663536
chr2_1694	p2	(TC)6	12	9667616	9667627
chr2_1695	p2	(AC)26	52	9674537	9674588
chr2_1696	p2	(AC)9	18	9676509	9676526
chr2_1697	p2	(TC)7	14	9677305	9677318
chr2_1698	p3	(TCA)5	15	9700932	9700946
chr2_1699	p3	(AGA)5	15	9708915	9708929
chr2_1700	p5	(ATCAA)5	25	9710602	9710626
chr2_1701	p2	(TG)16	32	9712290	9712321
chr2_1702	p2	(AT)8	16	9714734	9714749

chr2_1703	p2	(GT)7	14	9727827	9727840
chr2_1704	p2	(GT)16	32	9743768	9743799
chr2_1705	p2	(CA)7	14	9750464	9750477
chr2_1706	p2	(GT)25	50	9792104	9792153
chr2_1707	p2	(TA)6	12	9792607	9792618
chr2_1708	p4	(ATGT)5	20	9811727	9811746
chr2_1709	c	(TTTA)5(ATTA)6	44	9817859	9817902
chr2_1710	p2	(AC)8	16	9818257	9818272
chr2_1711	p2	(AC)8	16	9824672	9824687
chr2_1712	p3	(CCA)5	15	9826005	9826019
chr2_1713	p2	(CA)7	14	9826431	9826444
chr2_1714	p2	(AC)13	26	9826867	9826892
chr2_1715	p2	(TA)7	14	9844394	9844407
chr2_1716	p3	(ATT)7	21	9851643	9851663
chr2_1717	p4	(AATA)6	24	9866800	9866823
chr2_1718	c	(CTC)5(ATC)13	54	9870405	9870458
chr2_1719	p2	(AC)6	12	9874651	9874662
chr2_1720	p2	(AC)6	12	9877698	9877709
chr2_1721	p2	(TG)15	30	9905756	9905785
chr2_1722	p4	(CATC)5	20	9911990	9912009
chr2_1723	p3	(AAT)6	18	9912383	9912400
chr2_1724	p3	(ATG)5	15	9916364	9916378
chr2_1725	p2	(TG)32	64	9947328	9947391
chr2_1726	p2	(CA)14	28	9951636	9951663
chr2_1727	p3	(TTG)5	15	9953488	9953502
chr2_1728	p2	(AC)13	26	9960300	9960325
chr2_1729	p2	(TG)22	44	9975575	9975618
chr2_1730	p3	(TTA)5	15	9997120	9997134
chr2_1731	p2	(AC)7	14	10009267	10009280
chr2_1732	p2	(AC)9	18	10010551	10010568
chr2_1733	p2	(TG)6	12	10016568	10016579
chr2_1734	p2	(CA)17	34	10018548	10018581
chr2_1735	p2	(AC)6	12	10020449	10020460
chr2_1736	p2	(GT)10	20	10020718	10020737
chr2_1737	p2	(TG)12	24	10053258	10053281
chr2_1738	p3	(TTA)5	15	10053433	10053447
chr2_1739	p2	(TG)6	12	10061896	10061907
chr2_1740	p3	(TCA)5	15	10098162	10098176
chr2_1741	p2	(AC)9	18	10123425	10123442
chr2_1742	p2	(GT)6	12	10124862	10124873
chr2_1743	c	gagagaggagacacaaagttagtaatgtgtaatggtggcatataaaagcat	111	10125370	10125480
chr2_1744	p3	(ATC)5	15	10128009	10128023
chr2_1745	p2	(GT)11	22	10129571	10129592
chr2_1746	p2	(GA)7	14	10136581	10136594
chr2_1747	p2	(AC)16	32	10150189	10150220
chr2_1748	p3	(ATC)7	21	10162052	10162072
chr2_1749	p2	(CA)8	16	10163052	10163067
chr2_1750	p2	(GA)6	12	10163807	10163818
chr2_1751	p2	(GA)7	14	10191228	10191241
chr2_1752	p3	(AAT)6	18	10195449	10195466
chr2_1753	c	5tcatgaggatcacaaatgcgtgctgcgcaatctgcacgtataaagccatt(	74	10196143	10196216
chr2_1754	p5	(AATCA)5	25	10203089	10203113

chr2_1755	p3	(TAT)8	24	10204850	10204873
chr2_1756	p3	(ATT)6	18	10211497	10211514
chr2_1757	p2	(AT)6	12	10237079	10237090
chr2_1758	c	(GC)8(AC)12	40	10238076	10238115
chr2_1759	p3	(ACA)5	15	10260378	10260392
chr2_1760	p2	(TA)7	14	10260617	10260630
chr2_1761	c	(AT)9ac(AT)8	36	10282724	10282759
chr2_1762	p2	(TG)9	18	10286359	10286376
chr2_1763	p4	(CCAT)7	28	10331460	10331487
chr2_1764	p3	(AAT)5	15	10339113	10339127
chr2_1765	p2	(CA)29	58	10342829	10342886
chr2_1766	p2	(CT)6	12	10352433	10352444
chr2_1767	p2	(TG)8	16	10355054	10355069
chr2_1768	p2	(AG)6	12	10380208	10380219
chr2_1769	p2	(AC)7	14	10380642	10380655
chr2_1770	p2	(TC)8	16	10390212	10390227
chr2_1771	p2	(TG)14	28	10391097	10391124
chr2_1772	p2	(TG)6	12	10393607	10393618
chr2_1773	p4	(CCAT)5	20	10398173	10398192
chr2_1774	p3	(TTG)5	15	10401275	10401289
chr2_1775	p3	(TTA)5	15	10426607	10426621
chr2_1776	p2	(AC)6	12	10429671	10429682
chr2_1777	p2	(TG)6	12	10435026	10435037
chr2_1778	p4	(TCCA)5	20	10444147	10444166
chr2_1779	p2	(AG)7	14	10466963	10466976
chr2_1780	p2	(GC)6	12	10473793	10473804
chr2_1781	c*	(TATTA)7(TAT)5*	45	10475111	10475155
chr2_1782	p2	(AC)6	12	10477331	10477342
chr2_1783	p2	(TC)13	26	10489044	10489069
chr2_1784	p2	(TG)16	32	10490021	10490052
chr2_1785	p2	(CA)6	12	10491006	10491017
chr2_1786	p2	(AT)7	14	10503795	10503808
chr2_1787	p3	(ATT)10	30	10506827	10506856
chr2_1788	c	(TA)6tgattctgtttaattttgttttgttc(TA)6	50	10511076	10511125
chr2_1789	p2	(AC)7	14	10515906	10515919
chr2_1790	c	(TAA)5(GAA)5	30	10533753	10533782
chr2_1791	p2	(AC)10	20	10535055	10535074
chr2_1792	p2	(AG)16	32	10535870	10535901
chr2_1793	p5	(ATTTG)5	25	10542220	10542244
chr2_1794	p3	(TAT)6	18	10556245	10556262
chr2_1795	p2	(CA)12	24	10563940	10563963
chr2_1796	p2	(TA)6	12	10566909	10566920
chr2_1797	p4	(AGGT)6	24	10573035	10573058
chr2_1798	p2	(GT)7	14	10588769	10588782
chr2_1799	p3	(TAA)8	24	10593568	10593591
chr2_1800	p2	(TG)16	32	10595669	10595700
chr2_1801	c	(GATG)6gacgg(AT)11	51	10601937	10601987
chr2_1802	p2	(TG)22	44	10617199	10617242
chr2_1803	p2	(GT)6	12	10632974	10632985
chr2_1804	p3	(TTG)5	15	10658095	10658109
chr2_1805	p2	(AC)13	26	10659729	10659754
chr2_1806	p2	(GT)8	16	10666429	10666444

chr2_1807	p2	(TG)18	36	10680194	10680229
chr2_1808	p3	(TTA)5	15	10689485	10689499
chr2_1809	p2	(CT)6	12	10722286	10722297
chr2_1810	p2	(TC)6	12	10725275	10725286
chr2_1811	p3	(ATG)9	27	10728497	10728523
chr2_1812	p2	(CA)7	14	10734621	10734634
chr2_1813	p2	(CA)7	14	10734858	10734871
chr2_1814	p2	(TG)17	34	10744020	10744053
chr2_1815	p2	(CT)10	20	10748081	10748100
chr2_1816	p5	(TGATT)6	30	10751139	10751168
chr2_1817	p4	(CATC)5	20	10757983	10758002
chr2_1818	p3	(GAT)6	18	10759669	10759686
chr2_1819	c	gttagtagttgaacagctgcttgtggaacaagactccacacacacataggc	128	10762130	10762257
chr2_1820	p3	(TAT)5	15	10765802	10765816
chr2_1821	p3	(TTA)5	15	10766181	10766195
chr2_1822	p2	(TG)6	12	10767491	10767502
chr2_1823	p4	(TATC)6	24	10769437	10769460
chr2_1824	p2	(TG)7	14	10783814	10783827
chr2_1825	p2	(GT)7	14	10796478	10796491
chr2_1826	p2	(AC)7	14	10805490	10805503
chr2_1827	p3	(ACA)7	21	10808046	10808066
chr2_1828	p3	(AAG)5	15	10824975	10824989
chr2_1829	p3	(ATC)5	15	10825137	10825151
chr2_1830	p2	(AC)20	40	10835411	10835450
chr2_1831	p3	(AAC)5	15	10845286	10845300
chr2_1832	p2	(CA)9	18	10849761	10849778
chr2_1833	p2	(GT)26	52	10852060	10852111
chr2_1834	p2	(GT)7	14	10882121	10882134
chr2_1835	p2	(AT)6	12	10884490	10884501
chr2_1836	p2	(TG)6	12	10884661	10884672
chr2_1837	p2	(CA)10	20	10887570	10887589
chr2_1838	p2	(TA)6	12	10889117	10889128
chr2_1839	p2	(GA)7	14	10901949	10901962
chr2_1840	p3	(CTT)5	15	10916694	10916708
chr2_1841	p2	(AT)7	14	10936005	10936018
chr2_1842	c	tggagcgctgatggtgcaataattgtttttaaaacctttcatc(TG)13(TA	116	10938398	10938513
chr2_1843	p2	(AC)6	12	10942044	10942055
chr2_1844	p2	(TG)6	12	10943889	10943900
chr2_1845	p2	(TG)13	26	10944121	10944146
chr2_1846	p2	(TG)17	34	10945504	10945537
chr2_1847	p3	(ATT)5	15	10948655	10948669
chr2_1848	p2	(CA)6	12	10965396	10965407
chr2_1849	p2	(TA)6	12	10983605	10983616
chr2_1850	p2	(AC)7	14	10988363	10988376
chr2_1851	p2	(TG)14	28	10989709	10989736
chr2_1852	p2	(AG)6	12	11011434	11011445
chr2_1853	p2	(TG)7	14	11014355	11014368
chr2_1854	c	(CT)6(CA)6	24	11018176	11018199
chr2_1855	p2	(CA)6	12	11020238	11020249
chr2_1856	p2	(CA)6	12	11020480	11020491
chr2_1857	p2	(GT)6	12	11037891	11037902
chr2_1858	p2	(AT)8	16	11041035	11041050

chr2_1859	p2	(GT)6	12	11042783	11042794
chr2_1860	p4	(ATAG)6	24	11061051	11061074
chr2_1861	p2	(GT)7	14	11106647	11106660
chr2_1862	c	(AT)10(AC)13	46	11117590	11117635
chr2_1863	p2	(CA)10	20	11130405	11130424
chr2_1864	p2	(AG)7	14	11148183	11148196
chr2_1865	c	(AC)6at(AC)12	38	11151429	11151466
chr2_1866	p2	(CA)6	12	11158343	11158354
chr2_1867	p2	(AT)6	12	11161776	11161787
chr2_1868	p2	(TG)6	12	11170693	11170704
chr2_1869	p3	(AGA)11	33	11185636	11185668
chr2_1870	c	(GT)9(AT)7(AC)9	50	11186700	11186749
chr2_1871	p2	(TA)10	20	11188198	11188217
chr2_1872	p2	(TG)8	16	11189255	11189270
chr2_1873	p3	(TAA)7	21	11194302	11194322
chr2_1874	p3	(CAA)5	15	11200143	11200157
chr2_1875	p2	(CA)6	12	11201322	11201333
chr2_1876	p2	(GT)10	20	11214884	11214903
chr2_1877	p3	(ATT)5	15	11246390	11246404
chr2_1878	c	(TG)7tt(TG)19	54	11271584	11271637
chr2_1879	c	(TG)6ggtgtgtgg(GT)6	33	11278379	11278411
chr2_1880	p3	(GTA)7	21	11281695	11281715
chr2_1881	p4	(ATGG)5	20	11303037	11303056
chr2_1882	p4	(AGAT)6	24	11309533	11309556
chr2_1883	p2	(AC)11	22	11324174	11324195
chr2_1884	p4	(ATCC)5	20	11329113	11329132
chr2_1885	p2	(GT)7	14	11333926	11333939
chr2_1886	p2	(TG)6	12	11335093	11335104
chr2_1887	p2	(AT)9	18	11360763	11360780
chr2_1888	p2	(TC)6	12	11361327	11361338
chr2_1889	c	(TG)10(CA)9	38	11367755	11367792
chr2_1890	p3	(TTC)5	15	11373120	11373134
chr2_1891	p4	(AAAT)6	24	11378100	11378123
chr2_1892	p2	(CA)6	12	11382916	11382927
chr2_1893	p2	(AC)6	12	11385923	11385934
chr2_1894	c	(AT)7acacatacacat(AC)7	40	11390568	11390607
chr2_1895	p3	(TTC)7	21	11395295	11395315
chr2_1896	p3	(CAA)5	15	11403055	11403069
chr2_1897	p2	(CA)14	28	11407543	11407570
chr2_1898	p4	(CTTT)7	28	11407916	11407943
chr2_1899	p3	(ATG)5	15	11410240	11410254
chr2_1900	p2	(AT)6	12	11423174	11423185
chr2_1901	p2	(GT)22	44	11426850	11426893
chr2_1902	p2	(TA)7	14	11434918	11434931
chr2_1903	p3	(ACT)6	18	11437281	11437298
chr2_1904	c	(TTG)5tatgtgcagaaggactttttgtg(CA)6	50	11439504	11439553
chr2_1905	p2	(CA)6	12	11452609	11452620
chr2_1906	p2	(CA)12	24	11456853	11456876
chr2_1907	p3	(CAG)5	15	11456995	11457009
chr2_1908	p2	(CT)8	16	11458471	11458486
chr2_1909	p2	(CA)6	12	11471835	11471846
chr2_1910	p4	(TTTA)5	20	11492419	11492438

chr2_1911	p2	(TG)6	12	11522726	11522737
chr2_1912	p2	(TG)6	12	11546140	11546151
chr2_1913	p3	(ACT)5	15	11548056	11548070
chr2_1914	p2	(AC)11	22	11554892	11554913
chr2_1915	p2	(TG)8	16	11585248	11585263
chr2_1916	p4	(ATCC)5	20	11593618	11593637
chr2_1917	p3	(AAG)7	21	11597705	11597725
chr2_1918	p2	(AC)6	12	11598298	11598309
chr2_1919	p4	(TCCA)5	20	11614460	11614479
chr2_1920	p4	(AATG)6	24	11644256	11644279
chr2_1921	p3	(ATA)5	15	11645324	11645338
chr2_1922	p2	(AC)8	16	11657509	11657524
chr2_1923	p3	(TAT)5	15	11657845	11657859
chr2_1924	p2	(TG)6	12	11687886	11687897
chr2_1925	p3	(ATA)6	18	11695060	11695077
chr2_1926	p2	(TA)7	14	11699023	11699036
chr2_1927	p4	(AGAT)6	24	11717650	11717673
chr2_1928	p2	(TG)15	30	11741182	11741211
chr2_1929	p2	(GT)14	28	11741635	11741662
chr2_1930	p2	(TG)6	12	11744690	11744701
chr2_1931	p2	(CT)6	12	11745806	11745817
chr2_1932	p3	(TAA)7	21	11746160	11746180
chr2_1933	p2	(GA)7	14	11751460	11751473
chr2_1934	p2	(GA)6	12	11753715	11753726
chr2_1935	c	(CA)6cg(CA)6	26	11760909	11760934
chr2_1936	p2	(CA)8	16	11765972	11765987
chr2_1937	p4	(ATGG)5	20	11779263	11779282
chr2_1938	p2	(TG)6	12	11780491	11780502
chr2_1939	p2	(TG)6	12	11781939	11781950
chr2_1940	p2	(TA)6	12	11809471	11809482
chr2_1941	c	(TA)12(CA)8	40	11815176	11815215
chr2_1942	c	T)6ttgtctgtctatctatctctctgcctctctgtctgtctgtctctctgcctttct	211	11826715	11826925
chr2_1943	c	(CA)10(TA)10	40	11828075	11828114
chr2_1944	p5	(ATAGA)9	45	11836857	11836901
chr2_1945	c	(AAT)5cacctttatttggccaagcatgtt(AC)8	55	11840851	11840905
chr2_1946	p2	(AC)9	18	11841137	11841154
chr2_1947	p4	(AAAT)5	20	11846935	11846954
chr2_1948	p2	(CA)9	18	11858083	11858100
chr2_1949	p3	(AGG)7	21	11867408	11867428
chr2_1950	p2	(AG)6	12	11889282	11889293
chr2_1951	p6	(TAGATA)5	30	11894394	11894423
chr2_1952	p2	(TA)9	18	11900204	11900221
chr2_1953	p2	(CA)11	22	11900437	11900458
chr2_1954	p5	(AATAG)7	35	11912868	11912902
chr2_1955	p2	(CT)6	12	11913924	11913935
chr2_1956	p2	(GA)13	26	11914850	11914875
chr2_1957	p2	(AC)16	32	11919110	11919141
chr2_1958	p2	(AC)50	100	11921124	11921223
chr2_1959	c	(TA)8tctagatatatc(TAGA)5	48	11921992	11922039
chr2_1960	p2	(TG)8	16	11925311	11925326
chr2_1961	c	(CTT)7ctacctactac(TCT)13	71	11936229	11936299
chr2_1962	p2	(CA)6	12	11948180	11948191

chr2_1963	p2	(GT)20	40	11962217	11962256
chr2_1964	p2	(AT)6	12	11991397	11991408
chr2_1965	p4	(ATGG)5	20	12000181	12000200
chr2_1966	p3	(TTC)11	33	12000865	12000897
chr2_1967	p2	(TC)10	20	12015236	12015255
chr2_1968	p2	(CA)21	42	12073537	12073578
chr2_1969	p2	(TG)9	18	12073971	12073988
chr2_1970	p2	(CA)11	22	12095609	12095630
chr2_1971	c	(ATATC)8gtatcgtatcgtatcg(TATCA)15	131	12095827	12095957
chr2_1972	c	(TCTA)7(TCTG)5	48	12100787	12100834
chr2_1973	p2	(TC)7	14	12107201	12107214
chr2_1974	p2	(TG)8	16	12112461	12112476
chr2_1975	p2	(TG)13	26	12116901	12116926
chr2_1976	p2	(TG)10	20	12125215	12125234
chr2_1977	p2	(CA)6	12	12127538	12127549
chr2_1978	p2	(AC)9	18	12132302	12132319
chr2_1979	p2	(GT)6	12	12134580	12134591
chr2_1980	p3	(AGA)8	24	12151008	12151031
chr2_1981	c	(TG)8ta(TG)6	30	12153770	12153799
chr2_1982	p3	(TAT)5	15	12170553	12170567
chr2_1983	p2	(GA)11	22	12170867	12170888
chr2_1984	p2	(CT)6	12	12196724	12196735
chr2_1985	p2	(TG)17	34	12207458	12207491
chr2_1986	p2	(AC)6	12	12219306	12219317
chr2_1987	p2	(TG)6	12	12239623	12239634
chr2_1988	p3	(CTT)11	33	12240217	12240249
chr2_1989	p3	(TTA)5	15	12263829	12263843
chr2_1990	c	(CA)6gtttattctctgtctgactcttga(GT)18	72	12266228	12266299
chr2_1991	p3	(AAT)5	15	12269407	12269421
chr2_1992	p4	(TGTA)9	36	12270153	12270188
chr2_1993	p3	(GTA)6	18	12271949	12271966
chr2_1994	p2	(AC)8	16	12279459	12279474
chr2_1995	p2	(GT)21	42	12285214	12285255
chr2_1996	p4	(ATTG)6	24	12303826	12303849
chr2_1997	p2	(CT)7	14	12315305	12315318
chr2_1998	p2	(AT)9	18	12318765	12318782
chr2_1999	p3	(TGA)11	33	12323327	12323359
chr2_2000	p2	(AC)6	12	12336430	12336441
chr2_2001	p2	(CT)6	12	12338959	12338970
chr2_2002	p2	(GT)6	12	12360190	12360201
chr2_2003	p4	(TACC)6	24	12363352	12363375
chr2_2004	p3	(AGA)7	21	12380604	12380624
chr2_2005	p5	(AGTTG)5	25	12383109	12383133
chr2_2006	p2	(TG)7	14	12383691	12383704
chr2_2007	p2	(TG)6	12	12388545	12388556
chr2_2008	p2	(GT)11	22	12393672	12393693
chr2_2009	p2	(GT)7	14	12402339	12402352
chr2_2010	p2	(AG)6	12	12406508	12406519
chr2_2011	p2	(TG)6	12	12413958	12413969
chr2_2012	p2	(TG)6	12	12421921	12421932
chr2_2013	p2	(CT)6	12	12423275	12423286
chr2_2014	p4	(TTAC)5	20	12434605	12434624

chr2_2015	p2	(TG)8	16	12440843	12440858
chr2_2016	p2	(TC)7	14	12442320	12442333
chr2_2017	p2	(TG)9	18	12447100	12447117
chr2_2018	p2	(TG)20	40	12477957	12477996
chr2_2019	p2	(AT)19	38	12489396	12489433
chr2_2020	p2	(GT)8	16	12532774	12532789
chr2_2021	p4	(CCAT)5	20	12546757	12546776
chr2_2022	p2	(TC)9	18	12562347	12562364
chr2_2023	c	(AT)8(GT)9	34	12563545	12563578
chr2_2024	p3	(ATT)5	15	12567022	12567036
chr2_2025	p2	(GT)11	22	12575715	12575736
chr2_2026	p2	(CT)6	12	12592465	12592476
chr2_2027	p2	(AC)26	52	12593292	12593343
chr2_2028	p3	(TAG)7	21	12597899	12597919
chr2_2029	p2	(AT)7	14	12599542	12599555
chr2_2030	p2	(CA)8	16	12603588	12603603
chr2_2031	p2	(AC)6	12	12604395	12604406
chr2_2032	p2	(CA)6	12	12618254	12618265
chr2_2033	p3	(TAA)5	15	12623238	12623252
chr2_2034	c	(GT)6gcatgt(GA)7	32	12628672	12628703
chr2_2035	p4	(GATA)5	20	12633116	12633135
chr2_2036	c	(AAC)6(AAT)10	48	12641350	12641397
chr2_2037	p3	(ATT)6	18	12643653	12643670
chr2_2038	c	atttatacatttgtgacaatttcgccaaaaccggaaacatttgagcttcataaa	114	12654721	12654834
chr2_2039	p2	(GT)6	12	12661517	12661528
chr2_2040	p2	(TG)8	16	12663388	12663403
chr2_2041	p2	(GA)9	18	12666005	12666022
chr2_2042	p2	(GT)13	26	12666364	12666389
chr2_2043	p2	(GT)23	46	12669196	12669241
chr2_2044	p5	(ATTTG)7	35	12673376	12673410
chr2_2045	p2	(CA)15	30	12674195	12674224
chr2_2046	p4	(GATT)6	24	12676471	12676494
chr2_2047	p4	(AATG)6	24	12685744	12685767
chr2_2048	p4	(TACA)5	20	12694652	12694671
chr2_2049	p2	(AT)12	24	12696047	12696070
chr2_2050	p5	(CATCA)6	30	12696557	12696586
chr2_2051	p2	(AT)7	14	12699810	12699823
chr2_2052	p4	(ATAG)6	24	12702495	12702518
chr2_2053	p6	(GATTCT)6	36	12705885	12705920
chr2_2054	p2	(GT)7	14	12709662	12709675
chr2_2055	p2	(AG)16	32	12714217	12714248
chr2_2056	p2	(CA)6	12	12716296	12716307
chr2_2057	p2	(CA)7	14	12737266	12737279
chr2_2058	p3	(TAT)5	15	12754814	12754828
chr2_2059	p4	(TCTA)5	20	12756754	12756773
chr2_2060	p2	(AC)9	18	12757635	12757652
chr2_2061	p2	(AC)17	34	12766185	12766218
chr2_2062	p2	(AC)8	16	12774131	12774146
chr2_2063	p3	(CTT)6	18	12784381	12784398
chr2_2064	p2	(TG)7	14	12813108	12813121
chr2_2065	p2	(TG)8	16	12836145	12836160
chr2_2066	p2	(AC)16	32	12856302	12856333

chr2_2067	p2	(GT)7	14	12866821	12866834
chr2_2068	p2	(TA)13	26	12867532	12867557
chr2_2069	p2	(AC)9	18	12868142	12868159
chr2_2070	p2	(AC)9	18	12869158	12869175
chr2_2071	p2	(AC)11	22	12892574	12892595
chr2_2072	p2	(TG)6	12	12899660	12899671
chr2_2073	p2	(AC)6	12	12907299	12907310
chr2_2074	p2	(TA)6	12	12909693	12909704
chr2_2075	p3	(TAT)5	15	12913481	12913495
chr2_2076	p2	(AC)6	12	12914542	12914553
chr2_2077	p2	(TC)11	22	12921269	12921290
chr2_2078	p2	(CA)7	14	12927132	12927145
chr2_2079	p2	(GT)15	30	12927413	12927442
chr2_2080	p2	(GT)11	22	12939800	12939821
chr2_2081	p2	(GT)16	32	12942571	12942602
chr2_2082	p3	(TTA)5	15	12950136	12950150
chr2_2083	p2	(CA)6	12	12968134	12968145
chr2_2084	p2	(TG)10	20	12969522	12969541
chr2_2085	p2	(AT)8	16	12976835	12976850
chr2_2086	p4	(TTAT)5	20	12985905	12985924
chr2_2087	p3	(TAT)9	27	13006360	13006386
chr2_2088	p3	(CAG)5	15	13008612	13008626
chr2_2089	p2	(CA)13	26	13010900	13010925
chr2_2090	p2	(AG)7	14	13020193	13020206
chr2_2091	p2	(AG)7	14	13028178	13028191
chr2_2092	p3	(ATC)6	18	13032655	13032672
chr2_2093	p2	(TG)10	20	13038964	13038983
chr2_2094	p2	(TG)10	20	13041871	13041890
chr2_2095	p2	(AT)8	16	13046933	13046948
chr2_2096	p2	(TG)7	14	13053647	13053660
chr2_2097	p4	(AATG)5	20	13073605	13073624
chr2_2098	p2	(GT)7	14	13076965	13076978
chr2_2099	p4	(GATT)6	24	13086062	13086085
chr2_2100	c	(CA)19cg(CA)7	54	13105486	13105539
chr2_2101	p2	(CA)6	12	13114064	13114075
chr2_2102	p2	(AC)15	30	13118523	13118552
chr2_2103	p3	(TAA)6	18	13119493	13119510
chr2_2104	p2	(GT)7	14	13120538	13120551
chr2_2105	p2	(TG)8	16	13136494	13136509
chr2_2106	p2	(CT)6	12	13156711	13156722
chr2_2107	c	(TG)8tttgtgtgtt(TG)8	42	13156852	13156893
chr2_2108	p2	(TG)12	24	13157224	13157247
chr2_2109	p2	(TG)9	18	13157876	13157893
chr2_2110	p2	(AT)6	12	13158729	13158740
chr2_2111	c	(GA)8tagac(AG)8acagagagagacagac(AG)13	79	13159471	13159549
chr2_2112	p2	(CT)6	12	13175007	13175018
chr2_2113	p2	(GT)7	14	13176821	13176834
chr2_2114	p2	(TA)6	12	13205663	13205674
chr2_2115	p3	(GGT)6	18	13218383	13218400
chr2_2116	p2	(TC)6	12	13220770	13220781
chr2_2117	p2	(TA)7	14	13226726	13226739
chr2_2118	p2	(TG)17	34	13231727	13231760

chr2_2119	p2	(AC)6	12	13246053	13246064
chr2_2120	p2	(AC)7	14	13246336	13246349
chr2_2121	p2	(CA)7	14	13257888	13257901
chr2_2122	p2	(GT)7	14	13262003	13262016
chr2_2123	p2	(GT)28	56	13264064	13264119
chr2_2124	p2	(CT)6	12	13269739	13269750
chr2_2125	p2	(CA)7	14	13270459	13270472
chr2_2126	p2	(GT)15	30	13276015	13276044
chr2_2127	p2	(GT)6	12	13286571	13286582
chr2_2128	p3	(TGA)8	24	13286839	13286862
chr2_2129	p2	(TG)12	24	13290214	13290237
chr2_2130	p2	(TC)7	14	13292311	13292324
chr2_2131	p3	(TAA)7	21	13316941	13316961
chr2_2132	p4	(AAAG)5	20	13319444	13319463
chr2_2133	p5	(GATTT)5	25	13338135	13338159
chr2_2134	p2	(TA)8	16	13341648	13341663
chr2_2135	c	tgttttactggaaagcaccttgtaacgcaggtttagaaaggtgctatataaat	104	13354097	13354200
chr2_2136	p3	(TAT)11	33	13360062	13360094
chr2_2137	c	ggataaaaagttgagtttaatgtaaatgaagagcaatgttactggacaggag	121	13383009	13383129
chr2_2138	c*	(TACA)7(AC)15*(AT)6	67	13388455	13388521
chr2_2139	p4	(AGGT)7	28	13395111	13395138
chr2_2140	p2	(AC)6	12	13395434	13395445
chr2_2141	p2	(AC)7	14	13397879	13397892
chr2_2142	p5	(AATCA)6	30	13405763	13405792
chr2_2143	p2	(CA)6	12	13410814	13410825
chr2_2144	p2	(AT)6	12	13418302	13418313
chr2_2145	p2	(AC)6	12	13425707	13425718
chr2_2146	c	(TA)6t(TA)7	27	13428153	13428179
chr2_2147	c	(AT)7(AC)24	62	13449252	13449313
chr2_2148	p2	(TA)13	26	13450575	13450600
chr2_2149	p2	(AC)14	28	13462115	13462142
chr2_2150	p2	(TG)13	26	13470895	13470920
chr2_2151	p2	(AC)8	16	13479652	13479667
chr2_2152	p2	(TG)15	30	13491201	13491230
chr2_2153	p2	(TA)20	40	13499730	13499769
chr2_2154	p4	(TCTA)14	56	13526215	13526270
chr2_2155	p2	(TG)10	20	13532478	13532497
chr2_2156	p2	(TG)6	12	13532658	13532669
chr2_2157	p2	(AC)12	24	13536182	13536205
chr2_2158	p2	(AC)15	30	13536958	13536987
chr2_2159	p3	(TAT)5	15	13556492	13556506
chr2_2160	p2	(GA)9	18	13582771	13582788
chr2_2161	p3	(TCC)8	24	13585421	13585444
chr2_2162	p5	(AATAA)6	30	13588676	13588705
chr2_2163	p2	(GT)8	16	13607783	13607798
chr2_2164	p2	(AG)8	16	13607999	13608014
chr2_2165	p2	(TA)6	12	13621458	13621469
chr2_2166	p3	(ACA)5	15	13623648	13623662
chr2_2167	p2	(CA)28	56	13624211	13624266
chr2_2168	p2	(AG)6	12	13626478	13626489
chr2_2169	p2	(CA)26	52	13628369	13628420
chr2_2170	p2	(TG)14	28	13637972	13637999

chr2_2171	p2	(AT)14	28	13647458	13647485
chr2_2172	p3	(TAA)6	18	13661167	13661184
chr2_2173	p2	(AG)7	14	13687991	13688004
chr2_2174	p2	(AC)7	14	13706423	13706436
chr2_2175	p4	(AACA)6	24	13709908	13709931
chr2_2176	p3	(TTA)6	18	13725035	13725052
chr2_2177	p2	(GT)9	18	13730802	13730819
chr2_2178	p4	(CCAT)10	40	13740043	13740082
chr2_2179	p2	(CA)6	12	13757984	13757995
chr2_2180	p2	(TG)8	16	13769138	13769153
chr2_2181	p4	(AAAC)7	28	13769283	13769310
chr2_2182	p5	(ATCTG)5	25	13773930	13773954
chr2_2183	p2	(CA)13	26	13779320	13779345
chr2_2184	p4	(ATGG)5	20	13789260	13789279
chr2_2185	p2	(AC)8	16	13790325	13790340
chr2_2186	p3	(AAT)7	21	13793510	13793530
chr2_2187	p2	(AG)13	26	13797123	13797148
chr2_2188	p4	(ATGG)5	20	13810513	13810532
chr2_2189	p3	(CTT)5	15	13823743	13823757
chr2_2190	p3	(TAT)5	15	13840656	13840670
chr2_2191	p4	(ACAT)5	20	13847669	13847688
chr2_2192	p2	(AC)9	18	13852868	13852885
chr2_2193	p3	(CTC)5	15	13855418	13855432
chr2_2194	p2	(AC)6	12	13860382	13860393
chr2_2195	p2	(AC)9	18	13870040	13870057
chr2_2196	p2	(AC)12	24	13873399	13873422
chr2_2197	p2	(TG)8	16	13879053	13879068
chr2_2198	p3	(ATT)6	18	13890797	13890814
chr2_2199	p2	(TG)6	12	13895700	13895711
chr2_2200	p3	(GGA)6	18	13900613	13900630
chr2_2201	c	(TC)8g(CT)7	31	13906246	13906276
chr2_2202	p3	(ATT)5	15	13918974	13918988
chr2_2203	p2	(TG)15	30	13931824	13931853
chr2_2204	p2	(TC)22	44	13933879	13933922
chr2_2205	p3	(CAA)5	15	13942308	13942322
chr2_2206	p3	(ATT)5	15	13943462	13943476
chr2_2207	c	(TG)8g(GT)11	39	13946680	13946718
chr2_2208	p2	(GT)6	12	13966770	13966781
chr2_2209	p2	(GT)10	20	13976775	13976794
chr2_2210	p2	(CA)15	30	14020017	14020046
chr2_2211	p3	(AAG)5	15	14028561	14028575
chr2_2212	p2	(TG)6	12	14034038	14034049
chr2_2213	p3	(TTC)7	21	14039543	14039563
chr2_2214	p2	(CA)14	28	14042908	14042935
chr2_2215	p4	(TAGA)17	68	14057879	14057946
chr2_2216	c	(TG)9tt(TG)6	32	14064351	14064382
chr2_2217	p2	(CA)6	12	14064665	14064676
chr2_2218	p6	(TTATCC)5	30	14076190	14076219
chr2_2219	p2	(GA)9	18	14077300	14077317
chr2_2220	p2	(AC)6	12	14082435	14082446
chr2_2221	c	(TG)14(AG)13	54	14091682	14091735
chr2_2222	p3	(CTC)5	15	14100989	14101003

chr2_2223	p2	(AC)7	14	14101474	14101487
chr2_2224	p2	(AG)6	12	14106454	14106465
chr2_2225	p2	(AC)6	12	14127597	14127608
chr2_2226	p2	(TG)9	18	14138152	14138169
chr2_2227	p2	(TG)7	14	14139908	14139921
chr2_2228	p2	(GT)8	16	14146084	14146099
chr2_2229	p2	(AC)6	12	14149708	14149719
chr2_2230	p3	(TAT)5	15	14159053	14159067
chr2_2231	p2	(TG)9	18	14161712	14161729
chr2_2232	p2	(AT)7	14	14184755	14184768
chr2_2233	p2	(TG)13	26	14188161	14188186
chr2_2234	p3	(GGA)5	15	14191161	14191175
chr2_2235	p3	(TGA)6	18	14194914	14194931
chr2_2236	p2	(GT)11	22	14198623	14198644
chr2_2237	p2	(AC)16	32	14204619	14204650
chr2_2238	p3	(TAT)5	15	14215154	14215168
chr2_2239	p2	(CA)10	20	14219926	14219945
chr2_2240	p4	(TGTC)5	20	14232191	14232210
chr2_2241	p2	(TG)7	14	14238061	14238074
chr2_2242	p3	(ATT)8	24	14241172	14241195
chr2_2243	p2	(TG)29	58	14243790	14243847
chr2_2244	p2	(GT)17	34	14249058	14249091
chr2_2245	p3	(TAT)10	30	14249281	14249310
chr2_2246	c	[illegible])7tgtatgtatgtg(TA)6tgtatgtatgtatgtg(TA)7cacatacataca[illegible]	258	14251063	14251320
chr2_2247	p2	(AC)11	22	14256540	14256561
chr2_2248	p2	(TG)24	48	14265998	14266045
chr2_2249	p2	(CA)10	20	14268840	14268859
chr2_2250	p5	(AAATC)5	25	14285583	14285607
chr2_2251	p3	(TTG)5	15	14293418	14293432
chr2_2252	p2	(GA)9	18	14297717	14297734
chr2_2253	p4	(GACA)5	20	14298269	14298288
chr2_2254	c	(TGC)7(TTC)7tgcttcttcttcttctgttg(CTT)7	83	14327240	14327322
chr2_2255	p2	(AG)6	12	14359276	14359287
chr2_2256	p2	(AC)16	32	14369779	14369810
chr2_2257	c	(TA)12(CA)8	40	14373247	14373286
chr2_2258	p3	(TGT)5	15	14378226	14378240
chr2_2259	p2	(AC)12	24	14389253	14389276
chr2_2260	p2	(GT)29	58	14393422	14393479
chr2_2261	p2	(GT)8	16	14395468	14395483
chr2_2262	p3	(ATT)7	21	14400518	14400538
chr2_2263	p2	(GT)9	18	14400701	14400718
chr2_2264	p3	(CTG)5	15	14410898	14410912
chr2_2265	p3	(ATA)5	15	14414832	14414846
chr2_2266	p2	(TG)21	42	14419174	14419215
chr2_2267	p2	(GT)23	46	14419455	14419500
chr2_2268	p4	(GGTA)10	40	14427049	14427088
chr2_2269	c	(TG)6(AG)9	30	14432178	14432207
chr2_2270	p2	(TG)6	12	14433058	14433069
chr2_2271	p2	(TC)10	20	14436480	14436499
chr2_2272	p2	(AT)6	12	14447581	14447592
chr2_2273	p2	(AC)7	14	14450547	14450560
chr2_2274	p6	(GAGACA)5	30	14453960	14453989

chr2_2275	p2	(AG)6	12	14454107	14454118
chr2_2276	p2	(TG)16	32	14457102	14457133
chr2_2277	c	tgattaatgaatcatgtgacataattccacagatagttgaagccttggctttg(	107	14457664	14457770
chr2_2278	p2	(GT)6	12	14475558	14475569
chr2_2279	p2	(TG)15	30	14480630	14480659
chr2_2280	p2	(GT)13	26	14490552	14490577
chr2_2281	p2	(CA)10	20	14494513	14494532
chr2_2282	p2	(TC)13	26	14497817	14497842
chr2_2283	p2	(TG)6	12	14509453	14509464
chr2_2284	p2	(TG)7	14	14515796	14515809
chr2_2285	p2	(TG)7	14	14516586	14516599
chr2_2286	p3	(AAT)5	15	14525563	14525577
chr2_2287	p3	(TTA)5	15	14527614	14527628
chr2_2288	p2	(TG)7	14	14534742	14534755
chr2_2289	p2	(TG)7	14	14536871	14536884
chr2_2290	p2	(AC)11	22	14543092	14543113
chr2_2291	p3	(TCC)5	15	14544639	14544653
chr2_2292	p2	(TG)8	16	14560521	14560536
chr2_2293	p2	(TG)11	22	14565332	14565353
chr2_2294	p2	(CA)7	14	14573694	14573707
chr2_2295	p3	(TTG)6	18	14574682	14574699
chr2_2296	p2	(GA)6	12	14582402	14582413
chr2_2297	p2	(GT)8	16	14603430	14603445
chr2_2298	p2	(AC)17	34	14607084	14607117
chr2_2299	p2	(TG)7	14	14607500	14607513
chr2_2300	p2	(TG)8	16	14608430	14608445
chr2_2301	p3	(GAG)6	18	14611980	14611997
chr2_2302	p2	(AC)11	22	14617765	14617786
chr2_2303	p2	(GA)20	40	14625694	14625733
chr2_2304	p2	(CA)17	34	14634004	14634037
chr2_2305	p2	(AC)11	22	14634647	14634668
chr2_2306	p2	(TG)6	12	14645399	14645410
chr2_2307	c	(GT)8(AT)8	32	14652064	14652095
chr2_2308	p2	(AC)6	12	14660296	14660307
chr2_2309	p2	(AG)8	16	14662985	14663000
chr2_2310	p3	(TTA)6	18	14663675	14663692
chr2_2311	p2	(AC)19	38	14674326	14674363
chr2_2312	p2	(CA)7	14	14681219	14681232
chr2_2313	p2	(TG)6	12	14687279	14687290
chr2_2314	p2	(AC)6	12	14690038	14690049
chr2_2315	p2	(AC)15	30	14692213	14692242
chr2_2316	p2	(TG)11	22	14700184	14700205
chr2_2317	p2	(AC)6	12	14708031	14708042
chr2_2318	p2	(CA)16	32	14711247	14711278
chr2_2319	p2	(GT)8	16	14713057	14713072
chr2_2320	p2	(TG)6	12	14720975	14720986
chr2_2321	p2	(GT)18	36	14728904	14728939
chr2_2322	p2	(TG)6	12	14731837	14731848
chr2_2323	p4	(TCTA)15	60	14755198	14755257
chr2_2324	p2	(TG)26	52	14763679	14763730
chr2_2325	p2	(GT)6	12	14776761	14776772
chr2_2326	p2	(GA)6	12	14780907	14780918

chr2_2327	p3	(GTG)5	15	14781049	14781063
chr2_2328	p3	(AAT)5	15	14789070	14789084
chr2_2329	p2	(TG)6	12	14791208	14791219
chr2_2330	p2	(TG)12	24	14791541	14791564
chr2_2331	p2	(AC)13	26	14801342	14801367
chr2_2332	p5	(CATCT)11	55	14802638	14802692
chr2_2333	p2	(AG)7	14	14804241	14804254
chr2_2334	p2	(TG)6	12	14808413	14808424
chr2_2335	p2	(CA)6	12	14808704	14808715
chr2_2336	p2	(GA)21	42	14818568	14818609
chr2_2337	p2	(TG)6	12	14825340	14825351
chr2_2338	p2	(AC)6	12	14832189	14832200
chr2_2339	p2	(GA)8	16	14835335	14835350
chr2_2340	p2	(CA)14	28	14839300	14839327
chr2_2341	p2	(TG)28	56	14846673	14846728
chr2_2342	p2	(TG)7	14	14848486	14848499
chr2_2343	c	(CA)7cccacccacc(CA)17	58	14848707	14848764
chr2_2344	p2	(CT)8	16	14857267	14857282
chr2_2345	p3	(CTC)5	15	14864545	14864559
chr2_2346	p2	(TG)15	30	14867377	14867406
chr2_2347	p2	(CA)6	12	14868298	14868309
chr2_2348	p2	(AC)16	32	14872441	14872472
chr2_2349	p2	(AC)7	14	14876221	14876234
chr2_2350	p2	(TG)6	12	14878509	14878520
chr2_2351	p2	(TA)9	18	14879036	14879053
chr2_2352	c	gatggcaggcattgcaaaatgaacaagcattctgacatattcatacacactct	146	14879280	14879425
chr2_2353	p2	(GT)14	28	14879580	14879607
chr2_2354	p3	(GCA)6	18	14882633	14882650
chr2_2355	p2	(CT)6	12	14883815	14883826
chr2_2356	c	cacactgtaaccctctgacagatcacagacatcatgtctctaggccaaccg	111	14884013	14884123
chr2_2357	p2	(TG)28	56	14884553	14884608
chr2_2358	p2	(CA)10	20	14886080	14886099
chr2_2359	p2	(AC)7	14	14888208	14888221
chr2_2360	p2	(CA)11	22	14890035	14890056
chr2_2361	p2	(TG)10	20	14890714	14890733
chr2_2362	p2	(AC)8	16	14891794	14891809
chr2_2363	p2	(GT)12	24	14892609	14892632
chr2_2364	p2	(CA)7	14	14892885	14892898
chr2_2365	p2	(CA)17	34	14896105	14896138
chr2_2366	p3	(GGT)5	15	14897787	14897801
chr2_2367	c	(TC)9acttgctcc(GT)7	41	14898875	14898915
chr2_2368	p2	(GT)19	38	14906257	14906294
chr2_2369	p2	(AG)10	20	14909475	14909494
chr2_2370	c	ctgtttcatttaacatggaagtatctcccttcacaaatacagacacagcatcc	142	14912257	14912398
chr2_2371	p2	(GT)8	16	14912532	14912547
chr2_2372	p2	(CT)7	14	14921978	14921991
chr2_2373	c	tcaactctagagtttttgcccggctgtgtggaacatagcagatgtgctcagt	130	14922324	14922453
chr2_2374	p2	(GT)6	12	14924877	14924888
chr2_2375	p2	(CA)6	12	14927041	14927052
chr2_2376	p2	(AC)12	24	14927546	14927569
chr2_2377	p4	(AGAT)13	52	14931542	14931593
chr2_2378	p2	(GT)7	14	14935527	14935540

chr2_2379	p2	(GT)6	12	14939062	14939073
chr2_2380	p2	(TC)7	14	14944394	14944407
chr2_2381	p2	(CT)9	18	14948615	14948632
chr2_2382	p2	(AG)9	18	14951446	14951463
chr2_2383	c	(TG)7(TA)9	32	14961093	14961124
chr2_2384	p3	(TCA)6	18	14964123	14964140
chr2_2385	p2	(AT)8	16	14968939	14968954
chr2_2386	p2	(GT)18	36	14978512	14978547
chr2_2387	p2	(TG)6	12	14994805	14994816
chr2_2388	p2	(CA)10	20	14995294	14995313
chr2_2389	p2	(GT)20	40	14997052	14997091
chr2_2390	p3	(TGT)9	27	15000427	15000453
chr2_2391	p4	(TTTA)5	20	15002127	15002146
chr2_2392	p2	(GT)9	18	15003088	15003105
chr2_2393	c	(GT)7agat(AC)11	40	15006188	15006227
chr2_2394	p2	(AC)6	12	15007215	15007226
chr2_2395	p3	(GAG)8	24	15011691	15011714
chr2_2396	p3	(GAG)7	21	15013416	15013436
chr2_2397	p3	(GAG)5	15	15013935	15013949
chr2_2398	p3	(CAG)5	15	15015219	15015233
chr2_2399	p2	(CA)9	18	15017182	15017199
chr2_2400	p3	(TGT)6	18	15017458	15017475
chr2_2401	p2	(AC)10	20	15025408	15025427
chr2_2402	p2	(GA)10	20	15026144	15026163
chr2_2403	p2	(CT)6	12	15032926	15032937
chr2_2404	p2	(GT)6	12	15040143	15040154
chr2_2405	c	[illegible]gctcatgtaaaaccagatcaaaaacacaagatggcttcctgaatgggctg[illegible]	117	15045052	15045168
chr2_2406	p2	(TG)16	32	15047872	15047903
chr2_2407	p3	(ATA)5	15	15048809	15048823
chr2_2408	p2	(CA)12	24	15071395	15071418
chr2_2409	p5	(CTTCT)13	65	15072397	15072461
chr2_2410	p2	(AT)9	18	15075193	15075210
chr2_2411	p2	(GT)26	52	15075353	15075404
chr2_2412	p2	(GT)7	14	15083812	15083825
chr2_2413	p3	(ATG)5	15	15084239	15084253
chr2_2414	p2	(TC)7	14	15087564	15087577
chr2_2415	p2	(TA)14	28	15104607	15104634
chr2_2416	p2	(AC)12	24	15105858	15105881
chr2_2417	p2	(TA)14	28	15115565	15115592
chr2_2418	p3	(TTA)5	15	15121201	15121215
chr2_2419	p5	(TTGAT)5	25	15140663	15140687
chr2_2420	p2	(GT)9	18	15151538	15151555
chr2_2421	c	(TC)7(AC)7	28	15176498	15176525
chr2_2422	p4	(AAAT)5	20	15179686	15179705
chr2_2423	c	(TC)27ccag(CT)9	76	15181741	15181816
chr2_2424	p2	(TG)7	14	15202030	15202043
chr2_2425	p2	(CA)17	34	15207258	15207291
chr2_2426	p2	(TG)14	28	15208472	15208499
chr2_2427	p3	(TAA)11	33	15209278	15209310
chr2_2428	p2	(CA)8	16	15213183	15213198
chr2_2429	p2	(AG)11	22	15215389	15215410
chr2_2430	p2	(TG)16	32	15217394	15217425

chr2_2431	p2	(CA)19	38	15220929	15220966
chr2_2432	p2	(TG)15	30	15230064	15230093
chr2_2433	p3	(TTA)8	24	15230859	15230882
chr2_2434	p2	(TC)7	14	15237160	15237173
chr2_2435	c	gctgaaggcagtatttctaagcttagtcttgaaagtactgtaagttgtcaaac	172	15238281	15238452
chr2_2436	p2	(TG)6	12	15240083	15240094
chr2_2437	p2	(AC)9	18	15249091	15249108
chr2_2438	p2	(GT)12	24	15251228	15251251
chr2_2439	p2	(GT)8	16	15251365	15251380
chr2_2440	p4	(TCTG)5	20	15253066	15253085
chr2_2441	c	(TATC)5tatgtatctatctatg(TATC)5(TACC)5	76	15257173	15257248
chr2_2442	p2	(TG)7	14	15281318	15281331
chr2_2443	p2	(CT)11	22	15291011	15291032
chr2_2444	p2	(TG)17	34	15291888	15291921
chr2_2445	p2	(CA)9	18	15307438	15307455
chr2_2446	c	(AT)9(AG)7	32	15315203	15315234
chr2_2447	p2	(AC)24	48	15317988	15318035
chr2_2448	p2	(CT)6	12	15334428	15334439
chr2_2449	p2	(CA)16	32	15335131	15335162
chr2_2450	p2	(AT)7	14	15340473	15340486
chr2_2451	p2	(CA)6	12	15345401	15345412
chr2_2452	p2	(TG)16	32	15360866	15360897
chr2_2453	p4	(TCTT)5	20	15361599	15361618
chr2_2454	p3	(GAT)6	18	15361831	15361848
chr2_2455	p3	(AGA)5	15	15376750	15376764
chr2_2456	p2	(TC)8	16	15377420	15377435
chr2_2457	p2	(TG)10	20	15383461	15383480
chr2_2458	p2	(CA)6	12	15385580	15385591
chr2_2459	p2	(GT)7	14	15390296	15390309
chr2_2460	p3	(GAG)5	15	15392501	15392515
chr2_2461	p2	(AC)7	14	15397265	15397278
chr2_2462	p2	(AT)6	12	15401473	15401484
chr2_2463	p2	(CA)6	12	15402541	15402552
chr2_2464	p2	(TG)30	60	15407802	15407861
chr2_2465	p2	(CA)7	14	15411099	15411112
chr2_2466	c	aacaacatgcgcagcctgaggaggccaaggtaagaaggacagtgggag	128	15429985	15430112
chr2_2467	c	(GT)7gggtgtgtgtgg(GT)17	60	15434166	15434225
chr2_2468	p2	(TC)11	22	15435155	15435176
chr2_2469	p2	(TG)7	14	15442057	15442070
chr2_2470	c	(TG)10at(AG)7	36	15442400	15442435
chr2_2471	p2	(TG)13	26	15442572	15442597
chr2_2472	p2	(AC)8	16	15445852	15445867
chr2_2473	p2	(CA)7	14	15447777	15447790
chr2_2474	p2	(TG)15	30	15457712	15457741
chr2_2475	p2	(CT)18	36	15458161	15458196
chr2_2476	p2	(TG)6	12	15458557	15458568
chr2_2477	p3	(ATA)5	15	15461065	15461079
chr2_2478	p2	(GT)8	16	15462178	15462193
chr2_2479	p2	(TG)8	16	15462364	15462379
chr2_2480	p2	(GT)9	18	15467130	15467147
chr2_2481	p2	(AC)6	12	15467271	15467282
chr2_2482	p2	(TG)9	18	15467406	15467423

chr2_2483	p2	(TG)6	12	15469729	15469740
chr2_2484	p2	(TG)7	14	15471060	15471073
chr2_2485	p2	(CT)12	24	15471358	15471381
chr2_2486	c	(GT)7ga(GT)6(GA)8	44	15475383	15475426
chr2_2487	c	ctccacacac(AG)9gagagagaattcaattactcattcacagcagctga	185	15475555	15475739
chr2_2488	p2	(TG)11	22	15476294	15476315
chr2_2489	p2	(TC)7	14	15476486	15476499
chr2_2490	p2	(GT)6	12	15476912	15476923
chr2_2491	p3	(TAA)7	21	15479142	15479162
chr2_2492	p2	(TG)25	50	15483980	15484029
chr2_2493	p2	(CA)18	36	15484703	15484738
chr2_2494	p2	(AG)7	14	15485060	15485073
chr2_2495	p2	(AC)9	18	15485228	15485245
chr2_2496	p3	(AAC)8	24	15486833	15486856
chr2_2497	p2	(TC)7	14	15491511	15491524
chr2_2498	p2	(AC)8	16	15492926	15492941
chr2_2499	p2	(CA)7	14	15496957	15496970
chr2_2500	p4	(AAAC)5	20	15505285	15505304
chr2_2501	p2	(GA)6	12	15527475	15527486
chr2_2502	p2	(AC)18	36	15532745	15532780
chr2_2503	p2	(CA)12	24	15532895	15532918
chr2_2504	p2	(AC)6	12	15534746	15534757
chr2_2505	p2	(AC)8	16	15558321	15558336
chr2_2506	p5	(TTAGA)6	30	15559529	15559558
chr2_2507	p2	(AC)15	30	15559965	15559994
chr2_2508	p2	(CA)7	14	15560271	15560284
chr2_2509	p2	(AC)14	28	15564197	15564224
chr2_2510	p2	(GT)13	26	15572966	15572991
chr2_2511	p2	(AC)7	14	15576569	15576582
chr2_2512	p2	(AC)9	18	15581958	15581975
chr2_2513	p2	(CA)14	28	15587271	15587298
chr2_2514	p2	(TG)6	12	15592652	15592663
chr2_2515	p2	(GT)16	32	15609325	15609356
chr2_2516	p3	(CAT)5	15	15618293	15618307
chr2_2517	p2	(GT)7	14	15643999	15644012
chr2_2518	p4	(TTTA)6	24	15651257	15651280
chr2_2519	p2	(CT)6	12	15653185	15653196
chr2_2520	p2	(CT)8	16	15663954	15663969
chr2_2521	p2	(CA)7	14	15678687	15678700
chr2_2522	p2	(CT)7	14	15683661	15683674
chr2_2523	p2	(GA)7	14	15698570	15698583
chr2_2524	c	aacttgtgcacaagtaacagattagcccacaaatctgcaggaatgaatgca	148	15699415	15699562
chr2_2525	p2	(TG)21	42	15699958	15699999
chr2_2526	p5	(TGTGC)7	35	15703514	15703548
chr2_2527	p3	(TCT)6	18	15705068	15705085
chr2_2528	p2	(CA)6	12	15706153	15706164
chr2_2529	p5	(AAATA)5	25	15708942	15708966
chr2_2530	p2	(AC)6	12	15712201	15712212
chr2_2531	p3	(AAG)7	21	15712515	15712535
chr2_2532	p2	(TG)14	28	15714520	15714547
chr2_2533	p2	(GT)6	12	15742139	15742150
chr2_2534	p2	(AG)6	12	15759758	15759769

chr2_2535	p2	(TG)11	22	15765692	15765713
chr2_2536	p3	(GAA)8	24	15771374	15771397
chr2_2537	p3	(GGT)5	15	15773919	15773933
chr2_2538	p2	(AG)6	12	15784412	15784423
chr2_2539	p2	(CA)8	16	15785204	15785219
chr2_2540	p2	(GT)12	24	15788165	15788188
chr2_2541	p2	(AC)6	12	15791418	15791429
chr2_2542	p2	(GA)17	34	15792688	15792721
chr2_2543	p2	(GA)6	12	15799532	15799543
chr2_2544	p2	(TG)6	12	15802902	15802913
chr2_2545	p2	(GT)10	20	15808872	15808891
chr2_2546	c	(TA)8tc(TA)13gatatatatatc(TA)6gatatatatatc(TA)7	94	15814893	15814986
chr2_2547	p2	(TG)6	12	15818554	15818565
chr2_2548	p2	(AC)9	18	15818699	15818716
chr2_2549	p2	(TG)10	20	15820105	15820124
chr2_2550	p2	(AC)10	20	15825757	15825776
chr2_2551	p2	(GA)10	20	15826173	15826192
chr2_2552	p2	(TG)6	12	15831879	15831890
chr2_2553	p2	(TG)6	12	15832436	15832447
chr2_2554	p2	(TG)6	12	15848049	15848060
chr2_2555	p2	(TG)9	18	15849362	15849379
chr2_2556	p2	(TC)6	12	15849746	15849757
chr2_2557	p2	(AC)7	14	15850615	15850628
chr2_2558	p2	(CT)8	16	15858845	15858860
chr2_2559	c	(TG)13(TA)7	40	15872646	15872685
chr2_2560	p2	(CA)9	18	15904132	15904149
chr2_2561	p2	(TG)11	22	15910029	15910050
chr2_2562	p2	(GT)8	16	15926672	15926687
chr2_2563	p2	(AC)8	16	15932764	15932779
chr2_2564	p2	(CA)17	34	15936162	15936195
chr2_2565	p2	(TG)6	12	15938287	15938298
chr2_2566	p2	(TG)22	44	15942409	15942452
chr2_2567	p4	(AATA)5	20	15943350	15943369
chr2_2568	p2	(TA)8	16	15945300	15945315
chr2_2569	c	(CA)11tacccagagatctagtg(GT)10	59	15946729	15946787
chr2_2570	p2	(TG)11	22	15948894	15948915
chr2_2571	p2	(CA)11	22	15958638	15958659
chr2_2572	p2	(CA)12	24	15960145	15960168
chr2_2573	p2	(CA)7	14	15968772	15968785
chr2_2574	p2	(AG)6	12	15977685	15977696
chr2_2575	c	(ATCT)5(GTCT)25	120	15980390	15980509
chr2_2576	p2	(TC)6	12	15990046	15990057
chr2_2577	p2	(GT)6	12	15998092	15998103
chr2_2578	p2	(AC)8	16	16007738	16007753
chr2_2579	p2	(AC)7	14	16008615	16008628
chr2_2580	c	(TC)12(TG)11agt(GA)14	77	16017515	16017591
chr2_2581	p2	(AC)13	26	16018168	16018193
chr2_2582	p2	(TC)6	12	16029080	16029091
chr2_2583	p2	(TG)6	12	16038241	16038252
chr2_2584	p2	(CA)6	12	16039474	16039485
chr2_2585	p2	(AC)8	16	16041901	16041916
chr2_2586	p2	(TG)9	18	16049471	16049488

chr2_2587	p4	(ATGG)6	24	16055387	16055410
chr2_2588	p2	(AG)14	28	16073739	16073766
chr2_2589	p2	(TA)10	20	16096379	16096398
chr2_2590	p2	(TG)12	24	16096755	16096778
chr2_2591	p2	(CA)22	44	16096934	16096977
chr2_2592	p2	(TC)6	12	16097121	16097132
chr2_2593	p2	(TG)10	20	16100847	16100866
chr2_2594	c	(GA)7ggggggac(AG)7	36	16105625	16105660
chr2_2595	p3	(CTG)6	18	16113562	16113579
chr2_2596	p3	(AAC)8	24	16129378	16129401
chr2_2597	p2	(AC)35	70	16145008	16145077
chr2_2598	p2	(CA)8	16	16145345	16145360
chr2_2599	p2	(TG)13	26	16163825	16163850
chr2_2600	p2	(CA)8	16	16164463	16164478
chr2_2601	p3	(ATT)6	18	16175946	16175963
chr2_2602	p2	(TG)7	14	16185975	16185988
chr2_2603	p2	(TG)16	32	16186490	16186521
chr2_2604	p4	(TTTA)6	24	16191154	16191177
chr2_2605	p2	(CA)16	32	16192759	16192790
chr2_2606	p2	(CT)12	24	16200608	16200631
chr2_2607	p2	(CA)15	30	16202113	16202142
chr2_2608	p2	(AC)6	12	16207706	16207717
chr2_2609	p2	(TA)9	18	16211985	16212002
chr2_2610	p2	(TA)7	14	16219022	16219035
chr2_2611	p4	(TTTG)7	28	16228234	16228261
chr2_2612	p2	(AT)7	14	16229344	16229357
chr2_2613	p4	(GATA)23	92	16232394	16232485
chr2_2614	p2	(AC)6	12	16235971	16235982
chr2_2615	p2	(CT)6	12	16241660	16241671
chr2_2616	p5	(AGGAC)5	25	16249101	16249125
chr2_2617	p2	(TC)12	24	16262475	16262498
chr2_2618	p2	(TC)24	48	16272363	16272410
chr2_2619	p3	(ATC)5	15	16273064	16273078
chr2_2620	p2	(AC)13	26	16274280	16274305
chr2_2621	p2	(AC)6	12	16283919	16283930
chr2_2622	c	(TG)9g(GT)9	37	16285291	16285327
chr2_2623	p3	(AAT)5	15	16289012	16289026
chr2_2624	p2	(CA)8	16	16292505	16292520
chr2_2625	p3	(ATC)10	30	16308581	16308610
chr2_2626	p2	(CT)6	12	16311451	16311462
chr2_2627	c	(TA)6tttttataaatctatatatc(AT)6	45	16321122	16321166
chr2_2628	p2	(TA)7	14	16322426	16322439
chr2_2629	p3	(TAT)14	42	16328972	16329013
chr2_2630	p3	(GAT)6	18	16340550	16340567
chr2_2631	p2	(GT)19	38	16356521	16356558
chr2_2632	p3	(TGA)6	18	16356765	16356782
chr2_2633	p2	(AC)6	12	16366821	16366832
chr2_2634	p3	(CAG)5	15	16376398	16376412
chr2_2635	p2	(TG)6	12	16382209	16382220
chr2_2636	p2	(TA)6	12	16399853	16399864
chr2_2637	p4	(ATGG)5	20	16423138	16423157
chr2_2638	p3	(TCT)5	15	16433714	16433728

chr2_2639	p2	(AC)7	14	16446430	16446443
chr2_2640	p2	(TA)10	20	16447444	16447463
chr2_2641	p2	(GT)6	12	16452962	16452973
chr2_2642	p2	(TG)6	12	16457305	16457316
chr2_2643	p2	(GA)9	18	16458774	16458791
chr2_2644	p2	(CA)12	24	16496279	16496302
chr2_2645	p3	(TGA)7	21	16498585	16498605
chr2_2646	p2	(TA)6	12	16504141	16504152
chr2_2647	p2	(TC)6	12	16508081	16508092
chr2_2648	p3	(ACT)7	21	16509615	16509635
chr2_2649	p3	(TGT)5	15	16510002	16510016
chr2_2650	p3	(AAC)5	15	16510581	16510595
chr2_2651	p2	(GT)9	18	16512860	16512877
chr2_2652	p2	(TG)6	12	16515219	16515230
chr2_2653	p2	(CA)20	40	16516245	16516284
chr2_2654	p2	(AC)9	18	16517255	16517272
chr2_2655	p2	(TG)10	20	16520344	16520363
chr2_2656	p4	(AAAC)5	20	16520857	16520876
chr2_2657	p2	(TG)6	12	16524960	16524971
chr2_2658	p3	(GCA)7	21	16525418	16525438
chr2_2659	p3	(ATA)17	51	16548051	16548101
chr2_2660	c	(ACT)9(GCT)6	45	16583133	16583177
chr2_2661	p2	(AT)6	12	16594315	16594326
chr2_2662	p2	(AC)16	32	16596265	16596296
chr2_2663	p2	(TG)18	36	16612428	16612463
chr2_2664	p2	(GT)7	14	16614406	16614419
chr2_2665	p2	(AC)10	20	16619992	16620011
chr2_2666	p2	(TG)6	12	16625829	16625840
chr2_2667	p2	(TG)9	18	16645534	16645551
chr2_2668	p2	(GT)6	12	16658007	16658018
chr2_2669	p3	(TAA)6	18	16658480	16658497
chr2_2670	c	(GTC)6(TTC)10	48	16665887	16665934
chr2_2671	p4	(TCTA)17	68	16669498	16669565
chr2_2672	p2	(TC)6	12	16672052	16672063
chr2_2673	p2	(AC)10	20	16676937	16676956
chr2_2674	p2	(CA)7	14	16685075	16685088
chr2_2675	p2	(TG)19	38	16689603	16689640
chr2_2676	p2	(CA)20	40	16692182	16692221
chr2_2677	p2	(CT)6	12	16701432	16701443
chr2_2678	p4	(TCTA)9	36	16706035	16706070
chr2_2679	p2	(AC)10	20	16707464	16707483
chr2_2680	p3	(CAA)7	21	16711684	16711704
chr2_2681	p2	(CA)6	12	16725884	16725895
chr2_2682	p2	(TG)10	20	16729689	16729708
chr2_2683	p4	(TAGA)15	60	16750452	16750511
chr2_2684	c	(CA)22cta(CT)8	63	16751776	16751838
chr2_2685	p4	(TTGT)6	24	16755431	16755454
chr2_2686	p2	(TG)7	14	16773693	16773706
chr2_2687	p2	(GT)8	16	16786558	16786573
chr2_2688	p2	(TG)10	20	16820349	16820368
chr2_2689	p2	(TA)6	12	16821527	16821538
chr2_2690	p2	(AT)7	14	16835367	16835380

chr2_2691	p4	(TTTA)6	24	16840486	16840509
chr2_2692	p2	(CA)25	50	16842071	16842120
chr2_2693	c	(GT)12gagt(GA)13	54	16851958	16852011
chr2_2694	p2	(AT)14	28	16855447	16855474
chr2_2695	p4	(ATTT)6	24	16855628	16855651
chr2_2696	p2	(CA)14	28	16860151	16860178
chr2_2697	p3	(GGA)6	18	16860768	16860785
chr2_2698	p2	(TG)13	26	16866545	16866570
chr2_2699	p4	(GGTA)9	36	16885798	16885833
chr2_2700	p3	(CAA)6	18	16896671	16896688
chr2_2701	p4	(GATA)17	68	16910039	16910106
chr2_2702	p3	(TGG)5	15	16927445	16927459
chr2_2703	c	(CT)8cacact(CA)20	62	16938363	16938424
chr2_2704	p2	(GT)12	24	16956745	16956768
chr2_2705	p2	(CA)25	50	16972055	16972104
chr2_2706	c	(TC)7tt(TC)13(TG)8	58	16995054	16995111
chr2_2707	c	:atccttgtacagtttccacttttacagagagttgaagcttaacactgttacttca	141	17013699	17013839
chr2_2708	p2	(AC)12	24	17013992	17014015
chr2_2709	p2	(CA)6	12	17014596	17014607
chr2_2710	p2	(AG)6	12	17017363	17017374
chr2_2711	p2	(TC)6	12	17022541	17022552
chr2_2712	p2	(GA)11	22	17026487	17026508
chr2_2713	p2	(GT)6	12	17032157	17032168
chr2_2714	p2	(TG)6	12	17032820	17032831
chr2_2715	p2	(AC)10	20	17037012	17037031
chr2_2716	p2	(GT)11	22	17038865	17038886
chr2_2717	p2	(AC)9	18	17039439	17039456
chr2_2718	p2	(AC)10	20	17042025	17042044
chr2_2719	p4	(CAGT)7	28	17042841	17042868
chr2_2720	p2	(GT)6	12	17062958	17062969
chr2_2721	p2	(CA)6	12	17071587	17071598
chr2_2722	p3	(TCA)5	15	17073303	17073317
chr2_2723	p2	(AC)23	46	17073802	17073847
chr2_2724	p2	(AG)6	12	17075151	17075162
chr2_2725	p2	(TG)17	34	17081364	17081397
chr2_2726	p2	(TA)16	32	17081823	17081854
chr2_2727	c	Γ)13gtctgtcgctccccctacttcgctctgccagcaggcactgtgat(AC	122	17086061	17086182
chr2_2728	p2	(AC)6	12	17086814	17086825
chr2_2729	c	(AC)9(AT)11	40	17098212	17098251
chr2_2730	p2	(CA)10	20	17103629	17103648
chr2_2731	p3	(AAT)7	21	17105194	17105214
chr2_2732	p5	(GAGAG)7	35	17116439	17116473
chr2_2733	p3	(AAT)5	15	17121159	17121173
chr2_2734	p3	(TAG)5	15	17130133	17130147
chr2_2735	p2	(AC)10	20	17146410	17146429
chr2_2736	p2	(TG)9	18	17159430	17159447
chr2_2737	p2	(AC)7	14	17159875	17159888
chr2_2738	c	(AC)7a(AC)22	59	17177889	17177947
chr2_2739	p4	(ATTA)5	20	17183077	17183096
chr2_2740	p2	(CT)6	12	17192338	17192349
chr2_2741	p3	(ATA)5	15	17199802	17199816
chr2_2742	p3	(TTC)5	15	17206715	17206729

chr2_2743	p2	(GT)6	12	17208425	17208436
chr2_2744	p2	(TA)6	12	17210067	17210078
chr2_2745	p3	(CAA)5	15	17213595	17213609
chr2_2746	p3	(ATC)5	15	17218230	17218244
chr2_2747	p2	(TA)7	14	17234081	17234094
chr2_2748	p2	(AC)21	42	17240125	17240166
chr2_2749	p2	(AC)6	12	17240285	17240296
chr2_2750	p2	(GT)6	12	17242849	17242860
chr2_2751	p2	(TC)6	12	17247603	17247614
chr2_2752	p2	(GT)14	28	17252794	17252821
chr2_2753	p2	(GT)14	28	17253304	17253331
chr2_2754	p2	(TG)7	14	17261831	17261844
chr2_2755	p2	(AC)30	60	17264183	17264242
chr2_2756	p5	(AAATC)5	25	17272421	17272445
chr2_2757	p2	(CA)16	32	17274432	17274463
chr2_2758	p2	(GA)8	16	17275720	17275735
chr2_2759	p2	(TG)8	16	17276425	17276440
chr2_2760	p2	(CA)18	36	17282551	17282586
chr2_2761	c*	(TG)14(GTG)5*	40	17283187	17283226
chr2_2762	p2	(AT)7	14	17284261	17284274
chr2_2763	p2	(AC)7	14	17287126	17287139
chr2_2764	p2	(CA)8	16	17296788	17296803
chr2_2765	p2	(TG)11	22	17301054	17301075
chr2_2766	p2	(CA)21	42	17302376	17302417
chr2_2767	p2	(TG)9	18	17305184	17305201
chr2_2768	p2	(CA)8	16	17308022	17308037
chr2_2769	p2	(TG)12	24	17308947	17308970
chr2_2770	p2	(AC)11	22	17310253	17310274
chr2_2771	p3	(TTG)5	15	17338223	17338237
chr2_2772	p2	(TG)7	14	17353590	17353603
chr2_2773	p3	(CAA)5	15	17356340	17356354
chr2_2774	p2	(AC)9	18	17359303	17359320
chr2_2775	p2	(CT)7	14	17363350	17363363
chr2_2776	p2	(AC)9	18	17376219	17376236
chr2_2777	p2	(TG)9	18	17386440	17386457
chr2_2778	p2	(TG)6	12	17396831	17396842
chr2_2779	p2	(TA)7	14	17404094	17404107
chr2_2780	p2	(AC)11	22	17406954	17406975
chr2_2781	p3	(AAC)6	18	17407947	17407964
chr2_2782	p2	(AC)7	14	17410636	17410649
chr2_2783	p2	(CA)7	14	17413583	17413596
chr2_2784	p3	(TTC)5	15	17417500	17417514
chr2_2785	p2	(GT)11	22	17422614	17422635
chr2_2786	p2	(AC)13	26	17428169	17428194
chr2_2787	p2	(GT)17	34	17430255	17430288
chr2_2788	p4	(ATTC)6	24	17436528	17436551
chr2_2789	p4	(ATCC)5	20	17446034	17446053
chr2_2790	p2	(TG)6	12	17453943	17453954
chr2_2791	p2	(AC)9	18	17459047	17459064
chr2_2792	p3	(TAT)7	21	17468078	17468098
chr2_2793	p2	(TC)6	12	17473765	17473776
chr2_2794	p2	(TG)7	14	17479127	17479140

chr2_2795	p2	(GT)9	18	17492500	17492517
chr2_2796	p2	(GT)17	34	17508256	17508289
chr2_2797	p3	(ATT)5	15	17511054	17511068
chr2_2798	p2	(TG)13	26	17511645	17511670
chr2_2799	p2	(AC)26	52	17518711	17518762
chr2_2800	p3	(AAT)8	24	17523265	17523288
chr2_2801	p2	(CT)6	12	17529179	17529190
chr2_2802	p2	(GT)9	18	17534233	17534250
chr2_2803	p2	(TG)8	16	17549661	17549676
chr2_2804	p2	(AC)8	16	17557986	17558001
chr2_2805	p2	(CA)20	40	17561368	17561407
chr2_2806	p2	(TG)6	12	17564852	17564863
chr2_2807	p2	(GT)9	18	17571255	17571272
chr2_2808	p2	(GT)19	38	17575694	17575731
chr2_2809	p2	(CT)7	14	17579654	17579667
chr2_2810	p2	(GT)10	20	17590676	17590695
chr2_2811	p2	(TG)15	30	17595545	17595574
chr2_2812	c	(TG)6ta(TG)8	30	17596473	17596502
chr2_2813	p2	(CA)6	12	17599711	17599722
chr2_2814	p2	(TA)8	16	17604223	17604238
chr2_2815	p2	(CT)6	12	17630426	17630437
chr2_2816	p2	(CA)15	30	17651382	17651411
chr2_2817	p2	(CT)11	22	17651558	17651579
chr2_2818	c	(GA)7a(AG)12	39	17652163	17652201
chr2_2819	p5	(TATTC)6	30	17660416	17660445
chr2_2820	p3	(TTA)5	15	17665205	17665219
chr2_2821	p3	(TTG)7	21	17668723	17668743
chr2_2822	p3	(TTA)7	21	17686071	17686091
chr2_2823	p3	(TGC)7	21	17698380	17698400
chr2_2824	p2	(GT)27	54	17708571	17708624
chr2_2825	p2	(AC)7	14	17715436	17715449
chr2_2826	c	(ATT)10g(TTA)7	52	17727856	17727907
chr2_2827	p2	(TG)16	32	17730698	17730729
chr2_2828	p2	(GA)8	16	17772450	17772465
chr2_2829	p2	(CT)21	42	17772747	17772788
chr2_2830	p3	(AAC)5	15	17784240	17784254
chr2_2831	c	(TG)6ta(TG)8	30	17786176	17786205
chr2_2832	p2	(TG)13	26	17817449	17817474
chr2_2833	p4	(TACC)9	36	17818062	17818097
chr2_2834	c*	(TCTA)6(TA)6*	34	17833850	17833883
chr2_2835	p2	(AT)6	12	17844456	17844467
chr2_2836	p2	(AC)24	48	17845163	17845210
chr2_2837	p2	(GT)9	18	17854864	17854881
chr2_2838	p2	(CA)15	30	17855375	17855404
chr2_2839	p2	(AC)6	12	17867330	17867341
chr2_2840	p4	(GAGG)6	24	17872206	17872229
chr2_2841	p2	(AC)11	22	17875894	17875915
chr2_2842	p2	(CA)8	16	17877003	17877018
chr2_2843	p2	(GT)14	28	17880617	17880644
chr2_2844	c	(AC)6cct(CA)7	29	17882246	17882274
chr2_2845	p3	(AAT)5	15	17883882	17883896
chr2_2846	p2	(GT)14	28	17886421	17886448

chr2_2847	p4	(AAAC)5	20	17887045	17887064
chr2_2848	c	(TA)8cacacatacatatacacacat(AC)10	57	17890791	17890847
chr2_2849	p2	(TA)8	16	17897661	17897676
chr2_2850	p2	(AC)14	28	17898004	17898031
chr2_2851	p2	(GT)6	12	17898189	17898200
chr2_2852	c	:ctatgaacactgaaaacccatcatacagtacacaggtgctaatgaaagtct	119	17898474	17898592
chr2_2853	p2	(CA)7	14	17900032	17900045
chr2_2854	p2	(CT)6	12	17900685	17900696
chr2_2855	p2	(AC)13	26	17902737	17902762
chr2_2856	p2	(TC)9	18	17908707	17908724
chr2_2857	p2	(CA)8	16	17910778	17910793
chr2_2858	p2	(GA)15	30	17924034	17924063
chr2_2859	p2	(TG)25	50	17927549	17927598
chr2_2860	p2	(AC)7	14	17931647	17931660
chr2_2861	p2	(CA)7	14	17933274	17933287
chr2_2862	p2	(TG)10	20	17950481	17950500
chr2_2863	p2	(TG)6	12	17951797	17951808
chr2_2864	p2	(CA)29	58	17967020	17967077
chr2_2865	p4	(AATA)5	20	17972219	17972238
chr2_2866	p2	(GT)7	14	17977257	17977270
chr2_2867	p2	(CT)8	16	17992983	17992998
chr2_2868	p2	(TG)6	12	17996349	17996360
chr2_2869	p2	(TG)9	18	17999922	17999939
chr2_2870	p2	(CA)6	12	18006082	18006093
chr2_2871	c	GA)8tacaataagatatagatgtgtgtagatgtaggtgtgtgcacac(GT)	71	18011282	18011352
chr2_2872	p2	(CT)6	12	18011617	18011628
chr2_2873	p2	(AT)7	14	18013504	18013517
chr2_2874	c*	(AG)8(GAGG)5*	33	18021232	18021264
chr2_2875	p2	(AC)6	12	18023638	18023649
chr2_2876	p2	(TG)6	12	18032600	18032611
chr2_2877	p2	(AC)6	12	18042029	18042040
chr2_2878	p3	(GAG)7	21	18048069	18048089
chr2_2879	p3	(TGA)5	15	18049124	18049138
chr2_2880	p2	(TG)6	12	18049484	18049495
chr2_2881	p2	(TG)6	12	18056549	18056560
chr2_2882	p2	(AC)7	14	18076090	18076103
chr2_2883	p3	(CTG)5	15	18082841	18082855
chr2_2884	p2	(AC)19	38	18083825	18083862
chr2_2885	p2	(CA)14	28	18084311	18084338
chr2_2886	p2	(AC)6	12	18108411	18108422
chr2_2887	p2	(TG)8	16	18119341	18119356
chr2_2888	c	(GGAC)5ggat(GGAC)6agacggc(CAGA)9	91	18122719	18122809
chr2_2889	p2	(TG)12	24	18135001	18135024
chr2_2890	p2	(TG)6	12	18139620	18139631
chr2_2891	p2	(CA)6	12	18140374	18140385
chr2_2892	p2	(TG)6	12	18143942	18143953
chr2_2893	p2	(TG)16	32	18150630	18150661
chr2_2894	p4	(TAGC)5	20	18155171	18155190
chr2_2895	p2	(TG)13	26	18161916	18161941
chr2_2896	p2	(TG)6	12	18163181	18163192
chr2_2897	p3	(AAT)6	18	18167366	18167383
chr2_2898	p2	(CA)10	20	18169895	18169914

chr2_2899	c	gcatg(GT)13gagt(GA)12gctgtcccatctctcacacaatggctcca	133	18174919	18175051
chr2_2900	p2	(TA)21	42	18177735	18177776
chr2_2901	p2	(GA)20	40	18183760	18183799
chr2_2902	c	(GT)24gg(GT)6	62	18184328	18184389
chr2_2903	c	(GT)10(GA)9	38	18201502	18201539
chr2_2904	p3	(AAG)5	15	18202022	18202036
chr2_2905	p2	(AG)7	14	18208820	18208833
chr2_2906	p2	(TA)6	12	18213668	18213679
chr2_2907	p4	(ACGG)5	20	18215202	18215221
chr2_2908	p2	(GT)40	80	18216902	18216981
chr2_2909	p3	(TGG)8	24	18221469	18221492
chr2_2910	p5	(ATTTG)5	25	18226957	18226981
chr2_2911	p4	(ATAG)7	28	18235200	18235227
chr2_2912	p4	(TCTA)17	68	18261027	18261094
chr2_2913	p2	(TC)9	18	18261685	18261702
chr2_2914	p2	(TC)6	12	18272458	18272469
chr2_2915	c	(TG)10ttgaggaaac(TG)6	42	18275243	18275284
chr2_2916	p2	(TG)24	48	18284333	18284380
chr2_2917	p2	(GT)15	30	18304957	18304986
chr2_2918	c	(CA)9ctgcattgtttaatcacagttcttatataa(AT)8	64	18315687	18315750
chr2_2919	p4	(TTTG)6	24	18354772	18354795
chr2_2920	p2	(TG)9	18	18378471	18378488
chr2_2921	p2	(GT)6	12	18379420	18379431
chr2_2922	c	(CA)10cc(CA)7	36	18381539	18381574
chr2_2923	p2	(AC)9	18	18382818	18382835
chr2_2924	p3	(TAA)6	18	18384307	18384324
chr2_2925	p2	(TG)9	18	18392592	18392609
chr2_2926	c	(GT)14a(TG)10	49	18393127	18393175
chr2_2927	p2	(AT)6	12	18397439	18397450
chr2_2928	p5	(TTGAT)5	25	18397972	18397996
chr2_2929	p3	(TAT)5	15	18415011	18415025
chr2_2930	p3	(TTA)7	21	18450726	18450746
chr2_2931	p3	(TGG)7	21	18455858	18455878
chr2_2932	c	(GT)18(GC)7	50	18463420	18463469
chr2_2933	p2	(AC)18	36	18466467	18466502
chr2_2934	p2	(CA)18	36	18469134	18469169
chr2_2935	p2	(AT)7	14	18479815	18479828
chr2_2936	p3	(ATT)5	15	18506083	18506097
chr2_2937	c	(TA)7tgtatgtata(TG)7	38	18513902	18513939
chr2_2938	p2	(CT)13	26	18514320	18514345
chr2_2939	p2	(CT)7	14	18515389	18515402
chr2_2940	p2	(AG)6	12	18520578	18520589
chr2_2941	p2	(AC)9	18	18521715	18521732
chr2_2942	p2	(AC)7	14	18530726	18530739
chr2_2943	p2	(CA)7	14	18533065	18533078
chr2_2944	p2	(AC)9	18	18543305	18543322
chr2_2945	c	(TG)6tatata(TG)8	34	18548262	18548295
chr2_2946	p3	(CTC)5	15	18549368	18549382
chr2_2947	p2	(AC)6	12	18554867	18554878
chr2_2948	p2	(GT)6	12	18555870	18555881
chr2_2949	p2	(CA)10	20	18567713	18567732
chr2_2950	p2	(AC)6	12	18567834	18567845

chr2_2951	p2	(AC)6	12	18607076	18607087
chr2_2952	c	(CT)9(GT)17	52	18616326	18616377
chr2_2953	p2	(TG)17	34	18626129	18626162
chr2_2954	p2	(TG)6	12	18626470	18626481
chr2_2955	p3	(AAG)5	15	18627610	18627624
chr2_2956	p2	(GT)6	12	18636586	18636597
chr2_2957	p2	(CA)9	18	18636717	18636734
chr2_2958	p2	(AC)6	12	18636901	18636912
chr2_2959	p3	(CTG)6	18	18638750	18638767
chr2_2960	p2	(AC)9	18	18642374	18642391
chr2_2961	p2	(AC)8	16	18642551	18642566
chr2_2962	p2	(AG)6	12	18642951	18642962
chr2_2963	p2	(GA)7	14	18644326	18644339
chr2_2964	p2	(TC)6	12	18645164	18645175
chr2_2965	p2	(CA)6	12	18648386	18648397
chr2_2966	p4	(GATA)14	56	18648726	18648781
chr2_2967	p2	(GT)12	24	18650097	18650120
chr2_2968	p2	(AC)6	12	18655066	18655077
chr2_2969	p2	(AC)7	14	18656381	18656394
chr2_2970	p2	(TA)6	12	18657487	18657498
chr2_2971	p2	(GT)7	14	18660023	18660036
chr2_2972	p4	(TCTA)5	20	18660273	18660292
chr2_2973	p2	(AC)6	12	18661222	18661233
chr2_2974	p2	(AC)7	14	18662998	18663011
chr2_2975	p2	(TG)8	16	18665795	18665810
chr2_2976	p2	(TA)6	12	18666187	18666198
chr2_2977	p4	(TGTT)6	24	18670420	18670443
chr2_2978	p2	(CT)6	12	18671167	18671178
chr2_2979	p2	(TG)10	20	18671491	18671510
chr2_2980	p2	(AC)7	14	18671694	18671707
chr2_2981	p2	(TG)11	22	18675962	18675983
chr2_2982	p2	(TG)11	22	18676381	18676402
chr2_2983	p3	(ACA)5	15	18677175	18677189
chr2_2984	p2	(GT)36	72	18684972	18685043
chr2_2985	p2	(TG)8	16	18685272	18685287
chr2_2986	p2	(CA)12	24	18685678	18685701
chr2_2987	p2	(GT)6	12	18686471	18686482
chr2_2988	p2	(CA)18	36	18694656	18694691
chr2_2989	p2	(AC)7	14	18695207	18695220
chr2_2990	p2	(TG)6	12	18698834	18698845
chr2_2991	p2	(TG)6	12	18699501	18699512
chr2_2992	p4	(GATA)16	64	18703744	18703807
chr2_2993	p2	(GT)11	22	18704067	18704088
chr2_2994	p2	(GT)6	12	18705875	18705886
chr2_2995	p3	(TCC)7	21	18706407	18706427
chr2_2996	p2	(CA)16	32	18709793	18709824
chr2_2997	p2	(GT)15	30	18710419	18710448
chr2_2998	p2	(TG)6	12	18716337	18716348
chr2_2999	c	(GT)8(GA)8	32	18717329	18717360
chr2_3000	c	(AAT)9(TAT)7	48	18718360	18718407
chr2_3001	p2	(GT)6	12	18718745	18718756
chr2_3002	p2	(GT)21	42	18730638	18730679

chr2_3003	p2	(GA)6	12	18733450	18733461
chr2_3004	p2	(CA)10	20	18771098	18771117
chr2_3005	p3	(AAC)7	21	18773109	18773129
chr2_3006	p5	(CCTCT)8	40	18773499	18773538
chr2_3007	p2	(GT)15	30	18777709	18777738
chr2_3008	c	(TG)8tttgtgaa(GT)7gg(GA)7	54	18781213	18781266
chr2_3009	p2	(CA)16	32	18785410	18785441
chr2_3010	p2	(CA)6	12	18785604	18785615
chr2_3011	p2	(TG)9	18	18787772	18787789
chr2_3012	p3	(ACC)5	15	18788720	18788734
chr2_3013	p2	(TG)6	12	18790326	18790337
chr2_3014	p2	(TG)23	46	18820465	18820510
chr2_3015	p2	(TC)6	12	18820788	18820799
chr2_3016	p2	(AG)24	48	18842423	18842470
chr2_3017	p2	(CA)9	18	18845317	18845334
chr2_3018	p3	(ATA)5	15	18864364	18864378
chr2_3019	p3	(AAT)6	18	18864882	18864899
chr2_3020	p2	(GT)13	26	18866676	18866701
chr2_3021	p3	(TTC)18	54	18868216	18868269
chr2_3022	p3	(ATA)5	15	18873559	18873573
chr2_3023	p3	(TAA)5	15	18874082	18874096
chr2_3024	p2	(GT)13	26	18875872	18875897
chr2_3025	p3	(TTC)14	42	18877412	18877453
chr2_3026	p3	(ATG)6	18	18882405	18882422
chr2_3027	c	TAG)5atggatgatggatagatggatagatagatagatagatg(GAT	90	18882759	18882848
chr2_3028	p3	(TAT)6	18	18888803	18888820
chr2_3029	p3	(TGC)8	24	18895923	18895946
chr2_3030	p2	(TG)6	12	18905378	18905389
chr2_3031	p2	(TA)8	16	18910095	18910110
chr2_3032	p3	(AAC)10	30	18916192	18916221
chr2_3033	c	(CT)9(CA)6	30	18917786	18917815
chr2_3034	p2	(AC)15	30	18921082	18921111
chr2_3035	p2	(AG)6	12	18925614	18925625
chr2_3036	p2	(AT)8	16	18956779	18956794
chr2_3037	p2	(CA)7	14	18962889	18962902
chr2_3038	p2	(AC)16	32	18970043	18970074
chr2_3039	p2	(TG)6	12	18975515	18975526
chr2_3040	p2	(TG)8	16	18988335	18988350
chr2_3041	p4	(TATC)17	68	19027607	19027674
chr2_3042	p2	(AC)27	54	19027787	19027840
chr2_3043	p2	(TG)11	22	19044105	19044126
chr2_3044	p4	(GGGA)5	20	19048560	19048579
chr2_3045	p2	(CA)7	14	19052777	19052790
chr2_3046	p2	(CA)10	20	19060677	19060696
chr2_3047	p3	(AAT)5	15	19062043	19062057
chr2_3048	p2	(CA)17	34	19063411	19063444
chr2_3049	p2	(GT)6	12	19068830	19068841
chr2_3050	p2	(AC)12	24	19068971	19068994
chr2_3051	p2	(GT)9	18	19086949	19086966
chr2_3052	p2	(AT)8	16	19092635	19092650
chr2_3053	p2	(AC)7	14	19104069	19104082
chr2_3054	p5	(GGTGT)6	30	19105304	19105333

chr2_3055	p2	(CT)6	12	19108695	19108706
chr2_3056	p2	(CA)21	42	19110865	19110906
chr2_3057	p2	(TC)10	20	19117261	19117280
chr2_3058	p2	(GT)8	16	19120790	19120805
chr2_3059	p2	(AC)20	40	19123195	19123234
chr2_3060	p2	(CA)7	14	19128262	19128275
chr2_3061	p2	(TC)6	12	19134954	19134965
chr2_3062	p2	(AG)11	22	19136699	19136720
chr2_3063	p2	(TG)7	14	19138235	19138248
chr2_3064	p2	(AC)9	18	19146222	19146239
chr2_3065	p2	(CA)6	12	19147692	19147703
chr2_3066	p2	(AC)18	36	19150693	19150728
chr2_3067	p2	(TG)12	24	19152099	19152122
chr2_3068	p2	(AG)6	12	19154163	19154174
chr2_3069	p3	(TTG)5	15	19158405	19158419
chr2_3070	p2	(AC)6	12	19166213	19166224
chr2_3071	p2	(AC)10	20	19166425	19166444
chr2_3072	p2	(TG)6	12	19174971	19174982
chr2_3073	p5	(TCTCC)5	25	19176547	19176571
chr2_3074	p2	(AC)6	12	19180527	19180538
chr2_3075	p2	(TC)12	24	19184188	19184211
chr2_3076	p2	(GT)6	12	19184760	19184771
chr2_3077	c	(AC)15g(CA)21	73	19190011	19190083
chr2_3078	p2	(AC)8	16	19202837	19202852
chr2_3079	p2	(GT)9	18	19202976	19202993
chr2_3080	c	(TG)7tatgtgtgtgtgtatatatgtatatatgtatatgtg(TA)7tg(TA)6	78	19212294	19212371
chr2_3081	p2	(GT)12	24	19226387	19226410
chr2_3082	p2	(TG)6	12	19227997	19228008
chr2_3083	p2	(TG)18	36	19229297	19229332
chr2_3084	p2	(AC)8	16	19234507	19234522
chr2_3085	c	tactgttctgtatgctctcctttctctcactgtcttttcttaatgcataatgaatttt	136	19238802	19238937
chr2_3086	p2	(CT)7	14	19239137	19239150
chr2_3087	p2	(GT)7	14	19245818	19245831
chr2_3088	p2	(AC)6	12	19248574	19248585
chr2_3089	p2	(TG)6	12	19253551	19253562
chr2_3090	p2	(CT)6	12	19254094	19254105
chr2_3091	p2	(GT)18	36	19268488	19268523
chr2_3092	p3	(ATC)9	27	19274314	19274340
chr2_3093	p2	(GT)8	16	19274897	19274912
chr2_3094	p2	(CA)7	14	19275257	19275270
chr2_3095	c	(AC)6cacagtgcacccacacact(GA)9	49	19275671	19275719
chr2_3096	p2	(GT)20	40	19290068	19290107
chr2_3097	p2	(TG)8	16	19297551	19297566
chr2_3098	c	tgggttgtatctggatttcatcaccactgaacaatcctgctcccaattaactca	107	19304817	19304923
chr2_3099	p2	(TG)14	28	19311058	19311085
chr2_3100	p2	(TC)6	12	19318483	19318494
chr2_3101	p2	(TG)8	16	19321766	19321781
chr2_3102	p2	(AC)6	12	19333388	19333399
chr2_3103	p2	(AC)17	34	19335796	19335829
chr2_3104	p2	(GT)16	32	19347512	19347543
chr2_3105	p2	(TC)11	22	19348556	19348577
chr2_3106	p3	(ATT)10	30	19348987	19349016

chr2_3107	p2	(TA)6	12	19357146	19357157
chr2_3108	p2	(AC)6	12	19368008	19368019
chr2_3109	p2	(TC)7	14	19370357	19370370
chr2_3110	p2	(GT)16	32	19372798	19372829
chr2_3111	p5	(AAATC)6	30	19378264	19378293
chr2_3112	c	(CT)12(CA)10	44	19382235	19382278
chr2_3113	p2	(CA)7	14	19389275	19389288
chr2_3114	p2	(TG)9	18	19389878	19389895
chr2_3115	p2	(CA)8	16	19393108	19393123
chr2_3116	p2	(TC)8	16	19403161	19403176
chr2_3117	p2	(CA)7	14	19403746	19403759
chr2_3118	p3	(TCA)10	30	19410655	19410684
chr2_3119	p2	(TG)13	26	19412326	19412351
chr2_3120	p2	(GA)11	22	19412814	19412835
chr2_3121	p2	(TG)7	14	19418091	19418104
chr2_3122	p2	(CA)8	16	19425110	19425125
chr2_3123	p2	(AC)23	46	19427341	19427386
chr2_3124	p2	(TG)10	20	19429792	19429811
chr2_3125	p3	(TTA)6	18	19430102	19430119
chr2_3126	p2	(GT)9	18	19431314	19431331
chr2_3127	p2	(GT)6	12	19434439	19434450
chr2_3128	p2	(GT)8	16	19437088	19437103
chr2_3129	p2	(AC)6	12	19442919	19442930
chr2_3130	p2	(GT)9	18	19445270	19445287
chr2_3131	p2	(AG)7	14	19445812	19445825
chr2_3132	p2	(GT)12	24	19446360	19446383
chr2_3133	p2	(TG)18	36	19449404	19449439
chr2_3134	p2	(AC)9	18	19458052	19458069
chr2_3135	p2	(TC)7	14	19460078	19460091
chr2_3136	p2	(AC)7	14	19465252	19465265
chr2_3137	p6	(CCATCA)5	30	19465593	19465622
chr2_3138	p2	(CT)7	14	19473737	19473750
chr2_3139	p2	(TG)14	28	19473947	19473974
chr2_3140	p2	(TG)6	12	19474163	19474174
chr2_3141	p2	(GT)9	18	19476892	19476909
chr2_3142	p3	(CTT)6	18	19482815	19482832
chr2_3143	p2	(AC)15	30	19496613	19496642
chr2_3144	p3	(ATT)5	15	19514068	19514082
chr2_3145	p4	(TTCA)5	20	19556076	19556095
chr2_3146	p3	(GGA)6	18	19558587	19558604
chr2_3147	p2	(TG)9	18	19561589	19561606
chr2_3148	p2	(AC)6	12	19569330	19569341
chr2_3149	p3	(TTG)6	18	19570697	19570714
chr2_3150	p2	(GT)6	12	19576176	19576187
chr2_3151	p2	(CA)14	28	19576672	19576699
chr2_3152	p2	(AG)14	28	19578196	19578223
chr2_3153	p3	(GGT)6	18	19578407	19578424
chr2_3154	p3	(TTA)5	15	19580177	19580191
chr2_3155	p3	(CTT)8	24	19587442	19587465
chr2_3156	p3	(CTC)5	15	19589006	19589020
chr2_3157	p3	(TTA)8	24	19589591	19589614
chr2_3158	p2	(CT)7	14	19589847	19589860

chr2_3159	c	(TG)6tatgtga(GT)6	31	19595052	19595082
chr2_3160	p2	(TG)8	16	19595251	19595266
chr2_3161	p2	(TG)7	14	19606658	19606671
chr2_3162	p2	(TG)8	16	19611939	19611954
chr2_3163	p2	(CA)19	38	19616519	19616556
chr2_3164	p3	(CCT)5	15	19617940	19617954
chr2_3165	p2	(AC)11	22	19640183	19640204
chr2_3166	p3	(CAG)5	15	19646825	19646839
chr2_3167	p2	(CT)6	12	19657160	19657171
chr2_3168	p2	(TG)7	14	19672621	19672634
chr2_3169	p2	(AT)7	14	19680586	19680599
chr2_3170	p2	(GT)8	16	19682645	19682660
chr2_3171	p2	(TG)6	12	19687302	19687313
chr2_3172	p2	(TG)10	20	19694239	19694258
chr2_3173	p2	(GT)8	16	19695089	19695104
chr2_3174	p2	(GA)6	12	19710533	19710544
chr2_3175	p2	(GT)18	36	19712457	19712492
chr2_3176	p2	(GA)7	14	19712964	19712977
chr2_3177	p2	(TG)9	18	19722624	19722641
chr2_3178	p2	(GT)19	38	19741127	19741164
chr2_3179	p2	(CA)15	30	19741715	19741744
chr2_3180	p3	(ATC)5	15	19745576	19745590
chr2_3181	p3	(CAC)5	15	19752548	19752562
chr2_3182	p2	(GT)9	18	19754037	19754054
chr2_3183	c*	(TTA)5(TA)7*	27	19758896	19758922
chr2_3184	p3	(AGG)11	33	19766470	19766502
chr2_3185	p2	(CA)19	38	19780056	19780093
chr2_3186	p2	(TA)8	16	19780384	19780399
chr2_3187	p5	(ATTTG)5	25	19798855	19798879
chr2_3188	p3	(TAT)6	18	19837903	19837920
chr2_3189	p3	(TAT)5	15	19844584	19844598
chr2_3190	p2	(CT)10	20	19857684	19857703
chr2_3191	p2	(AT)10	20	19858584	19858603
chr2_3192	p2	(TG)17	34	19858758	19858791
chr2_3193	p2	(TG)10	20	19878432	19878451
chr2_3194	p2	(CT)8	16	19882640	19882655
chr2_3195	p2	(TC)10	20	19903550	19903569
chr2_3196	p2	(TG)7	14	19907767	19907780
chr2_3197	p3	(CCA)5	15	19908476	19908490
chr2_3198	p3	(TCC)6	18	19913485	19913502
chr2_3199	p2	(TC)6	12	19914532	19914543
chr2_3200	p2	(GT)12	24	19917317	19917340
chr2_3201	p2	(GT)9	18	19922179	19922196
chr2_3202	p2	(TG)8	16	19926318	19926333
chr2_3203	c	(TTA)5(GTA)6	33	19928354	19928386
chr2_3204	p3	(TTA)11	33	19935109	19935141
chr2_3205	p2	(GT)9	18	19935412	19935429
chr2_3206	p2	(TA)6	12	19945554	19945565
chr2_3207	p2	(GA)10	20	19964261	19964280
chr2_3208	p2	(GT)38	76	19986456	19986531
chr2_3209	p2	(GA)6	12	19993013	19993024
chr2_3210	p2	(GC)6	12	20000129	20000140

chr2_3211	p2	(TC)6	12	20000803	20000814
chr2_3212	p3	(TTC)5	15	20003500	20003514
chr2_3213	p2	(TG)24	48	20014181	20014228
chr2_3214	p3	(ATC)6	18	20021097	20021114
chr2_3215	p3	(TCC)8	24	20042608	20042631
chr2_3216	p2	(GT)6	12	20072457	20072468
chr2_3217	p2	(AC)8	16	20094524	20094539
chr2_3218	c	(AC)6aa(AC)8	30	20097008	20097037
chr2_3219	p3	(AAC)10	30	20100277	20100306
chr2_3220	p2	(AC)7	14	20100533	20100546
chr2_3221	p3	(ATT)5	15	20104287	20104301
chr2_3222	p2	(CA)7	14	20109717	20109730
chr2_3223	p3	(TGT)5	15	20110620	20110634
chr2_3224	p4	(CAAT)6	24	20119822	20119845
chr2_3225	p2	(TC)6	12	20128136	20128147
chr2_3226	c	(CA)11gagagagaa(AG)11	53	20129215	20129267
chr2_3227	p3	(AAT)5	15	20141394	20141408
chr2_3228	p2	(CA)6	12	20145884	20145895
chr2_3229	p2	(TG)6	12	20155598	20155609
chr2_3230	p2	(GT)25	50	20176933	20176982
chr2_3231	p5	(TTTGA)5	25	20196312	20196336
chr2_3232	p3	(TAT)5	15	20201791	20201805
chr2_3233	p2	(TG)7	14	20203969	20203982
chr2_3234	c	(CG)8(CA)14	44	20210058	20210101
chr2_3235	c	ggcatgaatatccttacaagtagtagtagcttcatttttaaaaatcacaaaa	125	20225069	20225193
chr2_3236	p3	(AGT)5	15	20225314	20225328
chr2_3237	p3	(CTC)5	15	20226272	20226286
chr2_3238	p2	(CA)6	12	20271937	20271948
chr2_3239	p2	(TG)11	22	20288298	20288319
chr2_3240	p2	(GA)9	18	20297533	20297550
chr2_3241	p2	(CT)8	16	20323941	20323956
chr2_3242	c	(TC)6tttatatatacatacagtaggtatgtatatat(ATTC)5	64	20342610	20342673
chr2_3243	p2	(CA)6	12	20349053	20349064
chr2_3244	p3	(TCA)9	27	20349811	20349837
chr2_3245	p3	(TAA)5	15	20352435	20352449
chr2_3246	p2	(GT)6	12	20358034	20358045
chr2_3247	p2	(GT)7	14	20360830	20360843
chr2_3248	p3	(ATG)5	15	20362048	20362062
chr2_3249	p2	(CA)9	18	20364131	20364148
chr2_3250	p2	(GT)11	22	20364975	20364996
chr2_3251	p2	(GT)11	22	20376128	20376149
chr2_3252	p2	(TG)7	14	20377510	20377523
chr2_3253	p3	(ATT)5	15	20379817	20379831
chr2_3254	p2	(TG)6	12	20383073	20383084
chr2_3255	p2	(GT)8	16	20383340	20383355
chr2_3256	p2	(TC)6	12	20401195	20401206
chr2_3257	p3	(TAA)5	15	20401829	20401843
chr2_3258	p2	(AC)9	18	20404137	20404154
chr2_3259	c	(TC)7tttca(CT)8	35	20405115	20405149
chr2_3260	p2	(TC)6	12	20410672	20410683
chr2_3261	p2	(CA)16	32	20413717	20413748
chr2_3262	p2	(AC)10	20	20414864	20414883

chr2_3263	p3	(AAC)5	15	20415119	20415133
chr2_3264	p2	(AC)6	12	20421660	20421671
chr2_3265	p2	(CA)6	12	20423694	20423705
chr2_3266	p2	(CT)40	80	20426554	20426633
chr2_3267	p5	(ACTGC)6	30	20430351	20430380
chr2_3268	p3	(GGT)5	15	20430704	20430718
chr2_3269	p2	(GT)9	18	20433626	20433643
chr2_3270	p2	(TA)7	14	20435399	20435412
chr2_3271	p2	(TG)13	26	20436669	20436694
chr2_3272	p4	(TTAT)5	20	20437000	20437019
chr2_3273	p2	(AC)6	12	20439980	20439991
chr2_3274	p2	(CA)6	12	20444596	20444607
chr2_3275	p3	(ATT)6	18	20451092	20451109
chr2_3276	c	ctgatggcatgccagaggaggaaacttgtcagctgttcccgctgtgctgtaa	112	20456450	20456561
chr2_3277	p2	(TC)9	18	20459033	20459050
chr2_3278	p2	(TG)8	16	20463181	20463196
chr2_3279	c	(CA)6g(AC)8	29	20463852	20463880
chr2_3280	p2	(CA)15	30	20464178	20464207
chr2_3281	p2	(AG)6	12	20465046	20465057
chr2_3282	c	C)8aagacataatccaacgttttgtaagactgttggtgtgtctgtag(GT)	92	20465261	20465352
chr2_3283	p2	(AT)13	26	20467324	20467349
chr2_3284	c	(GA)6a(AG)9	31	20467541	20467571
chr2_3285	p2	(CA)8	16	20468011	20468026
chr2_3286	p2	(TG)6	12	20477306	20477317
chr2_3287	p2	(TC)7	14	20477442	20477455
chr2_3288	p2	(TC)6	12	20479413	20479424
chr2_3289	c	(TC)10acacacact(CA)9	47	20486633	20486679
chr2_3290	p2	(CA)13	26	20488621	20488646
chr2_3291	p2	(GT)16	32	20498991	20499022
chr2_3292	p2	(TC)7	14	20499179	20499192
chr2_3293	p2	(AC)13	26	20499440	20499465
chr2_3294	p2	(CA)8	16	20499632	20499647
chr2_3295	p2	(CA)10	20	20511137	20511156
chr2_3296	p2	(CA)12	24	20513764	20513787
chr2_3297	p2	(GA)7	14	20516328	20516341
chr2_3298	c	(ATA)5ttgataac(AAT)6	41	20521223	20521263
chr2_3299	c	(TG)8agtttgtatgta(TG)9	46	20545882	20545927
chr2_3300	p5	(GATTT)5	25	20551230	20551254
chr2_3301	c	gtgcgattgtaaacaagagcagacatgtggtaaggtggaacacgtggtgg	93	20557660	20557752
chr2_3302	p3	(CTC)6	18	20557947	20557964
chr2_3303	c	(GT)13(GC)7	40	20561206	20561245
chr2_3304	p2	(AG)7	14	20564512	20564525
chr2_3305	p2	(TA)6	12	20575350	20575361
chr2_3306	c	(GT)13(GA)9	44	20584825	20584868
chr2_3307	p2	(TG)12	24	20585437	20585460
chr2_3308	p2	(TG)9	18	20596027	20596044
chr2_3309	p3	(CAG)5	15	20605044	20605058
chr2_3310	p2	(TA)6	12	20607033	20607044
chr2_3311	p2	(CA)19	38	20608387	20608424
chr2_3312	c	(TCC)7tcatcctcc(TCA)6	48	20615349	20615396
chr2_3313	p2	(GT)18	36	20615634	20615669
chr2_3314	p3	(ATA)7	21	20620093	20620113

chr2_3315	p2	(AC)12	24	20625541	20625564
chr2_3316	p2	(CA)16	32	20626324	20626355
chr2_3317	p4	(GATA)7	28	20639624	20639651
chr2_3318	p2	(TC)6	12	20644613	20644624
chr2_3319	p2	(CA)11	22	20644830	20644851
chr2_3320	p2	(TG)8	16	20651353	20651368
chr2_3321	p2	(CA)7	14	20653303	20653316
chr2_3322	p2	(CA)8	16	20661014	20661029
chr2_3323	c	(TTC)10actttagctgtgaaactgtcctct(CTC)5	69	20663314	20663382
chr2_3324	p2	(CT)8	16	20664326	20664341
chr2_3325	p2	(TG)21	42	20664937	20664978
chr2_3326	p2	(AT)11	22	20665595	20665616
chr2_3327	p2	(GT)7	14	20669110	20669123
chr2_3328	p2	(GA)6	12	20671629	20671640
chr2_3329	p2	(GT)6	12	20681770	20681781
chr2_3330	p2	(TG)34	68	20682002	20682069
chr2_3331	p2	(TG)13	26	20682193	20682218
chr2_3332	p2	(CA)8	16	20682791	20682806
chr2_3333	p2	(CA)8	16	20686764	20686779
chr2_3334	p4	(AAAT)5	20	20687559	20687578
chr2_3335	p2	(GT)10	20	20688311	20688330
chr2_3336	p2	(CA)22	44	20689899	20689942
chr2_3337	p2	(AT)19	38	20693683	20693720
chr2_3338	p2	(AG)7	14	20694852	20694865
chr2_3339	p2	(CA)7	14	20696256	20696269
chr2_3340	p2	(TG)23	46	20701502	20701547
chr2_3341	p2	(GT)14	28	20708135	20708162
chr2_3342	c	(TG)8ta(TG)8	34	20708979	20709012
chr2_3343	p2	(GA)11	22	20712566	20712587
chr2_3344	p3	(TTA)5	15	20713977	20713991
chr2_3345	p2	(GT)14	28	20714708	20714735
chr2_3346	p2	(AC)10	20	20716257	20716276
chr2_3347	p2	(TG)7	14	20722986	20722999
chr2_3348	p2	(TG)15	30	20725128	20725157
chr2_3349	p2	(TG)6	12	20734002	20734013
chr2_3350	p2	(AC)39	78	20739968	20740045
chr2_3351	p2	(GT)17	34	20742341	20742374
chr2_3352	p2	(TG)14	28	20743327	20743354
chr2_3353	p2	(GT)18	36	20743615	20743650
chr2_3354	c	(AC)14(TC)6	40	20743754	20743793
chr2_3355	p2	(AC)6	12	20744962	20744973
chr2_3356	p2	(AC)11	22	20745106	20745127
chr2_3357	p2	(GT)11	22	20753820	20753841
chr2_3358	p3	(CCT)6	18	20759718	20759735
chr2_3359	p2	(GT)21	42	20783564	20783605
chr2_3360	p2	(AC)16	32	20783763	20783794
chr2_3361	p2	(TG)8	16	20787581	20787596
chr2_3362	p2	(TG)7	14	20800709	20800722
chr2_3363	p2	(TG)10	20	20802201	20802220
chr2_3364	p2	(TG)6	12	20806313	20806324
chr2_3365	p3	(TAT)7	21	20807093	20807113
chr2_3366	p2	(GT)14	28	20808403	20808430

chr2_3367	p2	(CA)6	12	20809279	20809290
chr2_3368	p3	(ACA)5	15	20840103	20840117
chr2_3369	p3	(ATC)5	15	20840222	20840236
chr2_3370	p2	(TG)18	36	20841899	20841934
chr2_3371	p2	(CA)16	32	20847729	20847760
chr2_3372	p2	(TG)10	20	20851009	20851028
chr2_3373	c	(CT)9t(TC)7act(CA)14	64	20851669	20851732
chr2_3374	p2	(TG)6	12	20853712	20853723
chr2_3375	p4	(TTTA)6	24	20854614	20854637
chr2_3376	p2	(TG)12	24	20859919	20859942
chr2_3377	p2	(AC)26	52	20870068	20870119
chr2_3378	c	(GT)6ga(GT)13	40	20871013	20871052
chr2_3379	p2	(AT)6	12	20874376	20874387
chr2_3380	p2	(AT)9	18	20878919	20878936
chr2_3381	p2	(GT)19	38	20880302	20880339
chr2_3382	p2	(GT)27	54	20887455	20887508
chr2_3383	p3	(TGC)5	15	20888325	20888339
chr2_3384	p2	(GT)10	20	20888733	20888752
chr2_3385	p2	(CA)24	48	20890503	20890550
chr2_3386	c	(CTCA)5cacat(AC)12	49	20891345	20891393
chr2_3387	p4	(TTTG)7	28	20898681	20898708
chr2_3388	p2	(TC)6	12	20899082	20899093
chr2_3389	c	(ATG)5(ACG)7	36	20931225	20931260
chr2_3390	p2	(TC)15	30	20938008	20938037
chr2_3391	p2	(TA)8	16	20958990	20959005
chr2_3392	p5	(GATTT)5	25	20962183	20962207
chr2_3393	c	(GT)6ctctctctcgtataggagagtaagc(CAG)8	61	20972098	20972158
chr2_3394	p2	(CA)9	18	20972260	20972277
chr2_3395	p2	(GT)9	18	20983550	20983567
chr2_3396	p3	(CTT)5	15	20990284	20990298
chr2_3397	p2	(TG)12	24	21008333	21008356
chr2_3398	p2	(TG)15	30	21009572	21009601
chr2_3399	p2	(CA)10	20	21010763	21010782
chr2_3400	p2	(AC)6	12	21012217	21012228
chr2_3401	p2	(TG)23	46	21014730	21014775
chr2_3402	p2	(TG)6	12	21026700	21026711
chr2_3403	p2	(TG)6	12	21031279	21031290
chr2_3404	p2	(GA)6	12	21033898	21033909
chr2_3405	p2	(AC)15	30	21043336	21043365
chr2_3406	p2	(TG)11	22	21051246	21051267
chr2_3407	p3	(TTA)5	15	21052648	21052662
chr2_3408	p2	(GT)14	28	21053468	21053495
chr2_3409	p2	(GT)6	12	21055672	21055683
chr2_3410	p2	(GT)7	14	21059441	21059454
chr2_3411	p2	(AC)6	12	21059612	21059623
chr2_3412	c	gatttgggtgggatgcaagggtggcagggagttatgtgtgatgagggagg	109	21063790	21063898
chr2_3413	p2	(GT)6	12	21069857	21069868
chr2_3414	p2	(CA)13	26	21073565	21073590
chr2_3415	p2	(TG)15	30	21074531	21074560
chr2_3416	p2	(TA)9	18	21076428	21076445
chr2_3417	p2	(TG)28	56	21078346	21078401
chr2_3418	p2	(CT)6	12	21079915	21079926

chr2_3419	p2	(CA)15	30	21083815	21083844
chr2_3420	p2	(TG)10	20	21098274	21098293
chr2_3421	p2	(CA)16	32	21098626	21098657
chr2_3422	p3	(AGA)7	21	21100347	21100367
chr2_3423	p2	(TG)8	16	21100513	21100528
chr2_3424	p2	(GA)12	24	21100715	21100738
chr2_3425	p2	(AC)8	16	21102711	21102726
chr2_3426	p3	(TAA)5	15	21104432	21104446
chr2_3427	p2	(AC)6	12	21111029	21111040
chr2_3428	p2	(TC)7	14	21114768	21114781
chr2_3429	p2	(AC)13	26	21118959	21118984
chr2_3430	p2	(TG)11	22	21119129	21119150
chr2_3431	p3	(TTA)5	15	21119946	21119960
chr2_3432	p2	(GT)16	32	21123683	21123714
chr2_3433	p2	(TG)8	16	21124499	21124514
chr2_3434	p2	(TG)18	36	21125466	21125501
chr2_3435	p4	(TTTA)5	20	21125797	21125816
chr2_3436	c	(TG)11(AG)10	42	21128117	21128158
chr2_3437	p2	(CA)11	22	21128361	21128382
chr2_3438	p2	(CA)24	48	21143598	21143645
chr2_3439	p2	(TG)6	12	21155124	21155135
chr2_3440	p2	(AC)9	18	21156369	21156386
chr2_3441	p2	(CA)11	22	21156503	21156524
chr2_3442	c	AG)6cgagagagactggacttgcaatttgaaattgtgagtgacat(GA)	67	21156669	21156735
chr2_3443	c	gctaccttgacggtgtaacattgggaccagacaggagggagagtggattt	116	21157573	21157688
chr2_3444	p2	(TG)8	16	21159976	21159991
chr2_3445	p2	(CA)21	42	21163718	21163759
chr2_3446	p2	(CA)6	12	21164633	21164644
chr2_3447	p2	(TG)6	12	21165857	21165868
chr2_3448	p2	(TG)6	12	21166886	21166897
chr2_3449	p2	(CT)7	14	21170604	21170617
chr2_3450	p2	(CT)8	16	21171054	21171069
chr2_3451	p2	(GT)7	14	21171293	21171306
chr2_3452	p2	(GT)9	18	21171718	21171735
chr2_3453	p2	(AC)14	28	21172212	21172239
chr2_3454	p2	(GT)6	12	21174999	21175010
chr2_3455	p2	(TG)6	12	21175475	21175486
chr2_3456	p2	(AC)6	12	21176966	21176977
chr2_3457	p3	(CAA)8	24	21181409	21181432
chr2_3458	c	(AG)9g(GA)7	33	21181936	21181968
chr2_3459	p2	(TG)18	36	21183073	21183108
chr2_3460	p2	(GA)6	12	21183689	21183700
chr2_3461	p2	(TG)6	12	21187025	21187036
chr2_3462	p2	(GA)6	12	21189062	21189073
chr2_3463	p2	(CA)7	14	21189796	21189809
chr2_3464	p2	(GA)6	12	21193740	21193751
chr2_3465	p2	(TC)7	14	21196664	21196677
chr2_3466	p3	(CTT)10	30	21196983	21197012
chr2_3467	p2	(AG)8	16	21198555	21198570
chr2_3468	p2	(CA)7	14	21200503	21200516
chr2_3469	c	G)10tcttagtgtgtatgcccgcatgtgcttgtgccttttgtatatatacc(T	82	21202319	21202400
chr2_3470	p4	(CTTT)5	20	21204159	21204178

chr2_3471	p2	(CA)13	26	21205787	21205812
chr2_3472	p2	(TG)19	38	21207021	21207058
chr2_3473	p2	(CA)13	26	21208391	21208416
chr2_3474	p2	(TG)10	20	21210028	21210047
chr2_3475	p2	(TG)14	28	21210193	21210220
chr2_3476	p2	(CA)7	14	21210429	21210442
chr2_3477	c	;cacatttgtgggtctgagttggtttgtgcatgtactatatgcactgagtttgct	107	21215667	21215773
chr2_3478	p2	(TG)15	30	21218171	21218200
chr2_3479	p2	(GT)20	40	21220725	21220764
chr2_3480	p2	(CA)13	26	21221827	21221852
chr2_3481	p2	(CA)6	12	21222863	21222874
chr2_3482	p2	(GA)12	24	21227574	21227597
chr2_3483	p2	(GT)8	16	21230004	21230019
chr2_3484	c	(CA)6gtacaatccaaaccctttcaagtaactcaagt(AC)11	66	21233631	21233696
chr2_3485	p2	(CA)19	38	21235029	21235066
chr2_3486	p2	(GA)6	12	21239747	21239758
chr2_3487	p2	(CT)6	12	21240451	21240462
chr2_3488	p2	(GT)7	14	21241585	21241598
chr2_3489	p2	(TG)15	30	21242905	21242934
chr2_3490	p2	(GT)6	12	21243213	21243224
chr2_3491	p2	(GA)10	20	21244492	21244511
chr2_3492	p2	(TG)6	12	21248242	21248253
chr2_3493	p2	(AG)6	12	21248914	21248925
chr2_3494	c	(TG)6g(GT)12	37	21249499	21249535
chr2_3495	p2	(AC)6	12	21249900	21249911
chr2_3496	p2	(TC)6	12	21250366	21250377
chr2_3497	c	(AC)24(TC)8	64	21251600	21251663
chr2_3498	p2	(TC)6	12	21255346	21255357
chr2_3499	p2	(AG)9	18	21256687	21256704
chr2_3500	p4	(TATC)5	20	21257094	21257113
chr2_3501	p2	(AC)9	18	21257589	21257606
chr2_3502	p2	(AC)21	42	21257801	21257842
chr2_3503	p2	(CT)9	18	21261152	21261169
chr2_3504	p2	(CT)17	34	21261324	21261357
chr2_3505	p2	(AC)9	18	21265091	21265108
chr2_3506	p2	(GT)7	14	21265308	21265321
chr2_3507	p3	(ATT)5	15	21266153	21266167
chr2_3508	p2	(AG)7	14	21268580	21268593
chr2_3509	p2	(GT)8	16	21270429	21270444
chr2_3510	p2	(GT)7	14	21275239	21275252
chr2_3511	c	(TG)6c(GT)7	27	21275661	21275687
chr2_3512	p2	(CA)6	12	21276605	21276616
chr2_3513	p2	(AC)7	14	21277066	21277079
chr2_3514	c	tctgttgtggatacaggctgacctgaataaagacatacactgacacacatac	132	21279075	21279206
chr2_3515	c	CA)7cc(CA)7cc(CA)7cc(CA)7cc(CA)7cc(CA)7cc(C.	586	21279657	21280242
chr2_3516	p2	(TA)6	12	21281869	21281880
chr2_3517	p2	(TG)6	12	21284139	21284150
chr2_3518	p2	(AT)6	12	21288164	21288175
chr2_3519	p2	(TG)6	12	21290791	21290802
chr2_3520	p2	(TG)6	12	21291078	21291089
chr2_3521	p2	(CA)7	14	21296183	21296196
chr2_3522	c	atatatactgtatatatatttatgtgtacgtatgtatacacatgtgtacacgcat	136	21298568	21298703

chr2_3523	p2	(GT)7	14	21299314	21299327
chr2_3524	c	(CA)10cgctctctct(CA)6	42	21300494	21300535
chr2_3525	p2	(CT)7	14	21304532	21304545
chr2_3526	p2	(TG)7	14	21305324	21305337
chr2_3527	p2	(CA)14	28	21312035	21312062
chr2_3528	p2	(TG)6	12	21313592	21313603
chr2_3529	p2	(TG)9	18	21314592	21314609
chr2_3530	p2	(TC)7	14	21315522	21315535
chr2_3531	p3	(CCA)5	15	21315907	21315921
chr2_3532	p2	(CT)6	12	21318360	21318371
chr2_3533	p2	(TG)21	42	21318931	21318972
chr2_3534	p2	(GT)8	16	21320705	21320720
chr2_3535	p2	(CA)6	12	21327843	21327854
chr2_3536	p2	(TG)16	32	21329976	21330007
chr2_3537	p2	(AG)6	12	21330364	21330375
chr2_3538	p2	(AC)6	12	21334223	21334234
chr2_3539	p2	(CA)6	12	21335617	21335628
chr2_3540	p2	(AC)16	32	21339623	21339654
chr2_3541	c	(GT)10(GA)8	36	21340328	21340363
chr2_3542	p2	(AG)6	12	21342659	21342670
chr2_3543	p2	(TG)11	22	21345476	21345497
chr2_3544	c	ctactcttcccttgtcctgacagatcggagtctcatccctatccatttcacctca	135	21347870	21348004
chr2_3545	p2	(TG)11	22	21348927	21348948
chr2_3546	p2	(CT)6	12	21356029	21356040
chr2_3547	p2	(TG)6	12	21358285	21358296
chr2_3548	p2	(TC)11	22	21362598	21362619
chr2_3549	p2	(TG)13	26	21366161	21366186
chr2_3550	p2	(GA)11	22	21367459	21367480
chr2_3551	c	(AG)13(TG)12(AG)6	62	21368359	21368420
chr2_3552	p2	(GT)14	28	21369415	21369442
chr2_3553	p2	(AT)6	12	21371601	21371612
chr2_3554	p2	(AC)6	12	21373491	21373502
chr2_3555	p2	(GA)8	16	21374544	21374559
chr2_3556	p4	(TAGA)13	52	21374762	21374813
chr2_3557	p3	(GTT)5	15	21375792	21375806
chr2_3558	p2	(CA)8	16	21376408	21376423
chr2_3559	p3	(CCT)7	21	21377731	21377751
chr2_3560	c	CA)7cttgccttcctgatcacacgtttcatttgcctctgcactgcccccatag	102	21380704	21380805
chr2_3561	p2	(AC)9	18	21387749	21387766
chr2_3562	p2	(TG)13	26	21393796	21393821
chr2_3563	p2	(AG)7	14	21395336	21395349
chr2_3564	p4	(CTCC)5	20	21398096	21398115
chr2_3565	p2	(TC)6	12	21401110	21401121
chr2_3566	p2	(AC)6	12	21407662	21407673
chr2_3567	p2	(TG)13	26	21409042	21409067
chr2_3568	p2	(CA)7	14	21409530	21409543
chr2_3569	p2	(CA)6	12	21410904	21410915
chr2_3570	p2	(AC)24	48	21416211	21416258
chr2_3571	p2	(CA)8	16	21416759	21416774
chr2_3572	c	(CA)11gagtgacagacat(AC)7	49	21416956	21417004
chr2_3573	p2	(GT)6	12	21419145	21419156
chr2_3574	p2	(TG)17	34	21419308	21419341

chr2_3575	p2	(GA)9	18	21423336	21423353
chr2_3576	p2	(AC)9	18	21427396	21427413
chr2_3577	p2	(CA)8	16	21427974	21427989
chr2_3578	p2	(AC)7	14	21428150	21428163
chr2_3579	p2	(TC)8	16	21435939	21435954
chr2_3580	p2	(GT)15	30	21439179	21439208
chr2_3581	p2	(CA)17	34	21439676	21439709
chr2_3582	p2	(AC)10	20	21442888	21442907
chr2_3583	p2	(TG)7	14	21449823	21449836
chr2_3584	p2	(TG)15	30	21450210	21450239
chr2_3585	p2	(AG)6	12	21451318	21451329
chr2_3586	p2	(CA)17	34	21455772	21455805
chr2_3587	p2	(TG)6	12	21460673	21460684
chr2_3588	p2	(CA)6	12	21462418	21462429
chr2_3589	p2	(AC)13	26	21462822	21462847
chr2_3590	p2	(GT)9	18	21464293	21464310
chr2_3591	p2	(AG)7	14	21465569	21465582
chr2_3592	p2	(TC)7	14	21466543	21466556
chr2_3593	p2	(GT)18	36	21469220	21469255
chr2_3594	p2	(AG)6	12	21471763	21471774
chr2_3595	p2	(CA)22	44	21472713	21472756
chr2_3596	p2	(AG)9	18	21489336	21489353
chr2_3597	p2	(TG)10	20	21491744	21491763
chr2_3598	p2	(AC)8	16	21495712	21495727
chr2_3599	p4	(AAAC)5	20	21496646	21496665
chr2_3600	p2	(GT)7	14	21496815	21496828
chr2_3601	p2	(AG)7	14	21497323	21497336
chr2_3602	p2	(CT)6	12	21500391	21500402
chr2_3603	p2	(GT)14	28	21507620	21507647
chr2_3604	p2	(AC)7	14	21507996	21508009
chr2_3605	p2	(CA)6	12	21518240	21518251
chr2_3606	p3	(GAA)5	15	21519843	21519857
chr2_3607	p2	(TC)8	16	21521936	21521951
chr2_3608	c	(AC)7atg(CA)7	31	21529591	21529621
chr2_3609	p2	(TC)7	14	21532969	21532982
chr2_3610	c	(CT)6ccttctctacct(CA)8	40	21536622	21536661
chr2_3611	p2	(AT)8	16	21537080	21537095
chr2_3612	p2	(CT)16	32	21537372	21537403
chr2_3613	p3	(TTA)5	15	21543371	21543385
chr2_3614	p2	(CA)19	38	21544167	21544204
chr2_3615	p2	(CA)13	26	21548323	21548348
chr2_3616	p2	(AG)6	12	21552423	21552434
chr2_3617	p2	(CT)7	14	21552928	21552941
chr2_3618	p2	(TG)13	26	21553307	21553332
chr2_3619	p2	(CA)10	20	21555015	21555034
chr2_3620	p2	(CA)17	34	21562146	21562179
chr2_3621	p2	(AG)8	16	21562386	21562401
chr2_3622	p2	(AC)8	16	21564456	21564471
chr2_3623	p3	(CAA)7	21	21567641	21567661
chr2_3624	p2	(GA)10	20	21570596	21570615
chr2_3625	p4	(AAGA)7	28	21573341	21573368
chr2_3626	p2	(GT)10	20	21576824	21576843

chr2_3627	p2	(CA)6	12	21577057	21577068
chr2_3628	p2	(AT)7	14	21579233	21579246
chr2_3629	p2	(CT)13	26	21580525	21580550
chr2_3630	p2	(CT)8	16	21582858	21582873
chr2_3631	p2	(CT)8	16	21583918	21583933
chr2_3632	p2	(GA)11	22	21591130	21591151
chr2_3633	p2	(TC)7	14	21593124	21593137
chr2_3634	p2	(GT)6	12	21598461	21598472
chr2_3635	p2	(CA)6	12	21605779	21605790
chr2_3636	p2	(TG)8	16	21606656	21606671
chr2_3637	p2	(AC)10	20	21613033	21613052
chr2_3638	p2	(TG)25	50	21613197	21613246
chr2_3639	p2	(TG)20	40	21618433	21618472
chr2_3640	p2	(GT)13	26	21627624	21627649
chr2_3641	p2	(AC)11	22	21635384	21635405
chr2_3642	p2	(TG)11	22	21641834	21641855
chr2_3643	c	aaatgtgagtagcaatgcttcctaataacatgctgaaagaaaaggcggag	141	21642282	21642422
chr2_3644	p2	(AG)8	16	21643486	21643501
chr2_3645	p2	(TG)11	22	21650847	21650868
chr2_3646	c	aaatgtgagtagcaatgcttcctaataacatgctgaaagaaaaggcggag	141	21651295	21651435
chr2_3647	p2	(AG)8	16	21652499	21652514
chr2_3648	p2	(GA)6	12	21655576	21655587
chr2_3649	p2	(CT)7	14	21668596	21668609
chr2_3650	p2	(AC)6	12	21671930	21671941
chr2_3651	p2	(AC)18	36	21673626	21673661
chr2_3652	c	(GT)7gagagtgtgtgaa(TG)7	41	21674243	21674283
chr2_3653	p2	(TG)26	52	21676155	21676206
chr2_3654	p2	(CA)16	32	21676576	21676607
chr2_3655	p2	(GT)16	32	21688073	21688104
chr2_3656	p2	(AC)6	12	21689922	21689933
chr2_3657	p2	(GA)13	26	21690730	21690755
chr2_3658	p2	(CA)7	14	21697908	21697921
chr2_3659	p3	(GAA)5	15	21699636	21699650
chr2_3660	p2	(TG)6	12	21742106	21742117
chr2_3661	p2	(AC)10	20	21744077	21744096
chr2_3662	p2	(CT)10	20	21745863	21745882
chr2_3663	p3	(AAT)6	18	21766575	21766592
chr2_3664	c	(AC)18atacacat(AC)16	76	21777788	21777863
chr2_3665	p2	(GT)9	18	21781403	21781420
chr2_3666	p2	(GT)13	26	21782754	21782779
chr2_3667	p2	(AC)9	18	21784068	21784085
chr2_3668	p2	(GT)6	12	21787389	21787400
chr2_3669	p2	(AC)8	16	21792901	21792916
chr2_3670	p2	(GA)6	12	21793240	21793251
chr2_3671	p2	(CA)6	12	21796621	21796632
chr2_3672	p2	(GT)6	12	21800378	21800389
chr2_3673	p3	(TGA)5	15	21801501	21801515
chr2_3674	c	(TG)16(AG)14	60	21802260	21802319
chr2_3675	p2	(TG)6	12	21802769	21802780
chr2_3676	p2	(TG)7	14	21802933	21802946
chr2_3677	p3	(GGA)7	21	21807632	21807652
chr2_3678	p2	(AC)17	34	21809492	21809525

chr2_3679	p2	(TG)21	42	21810660	21810701
chr2_3680	p2	(CA)16	32	21813525	21813556
chr2_3681	p2	(CA)6	12	21815623	21815634
chr2_3682	p2	(AC)7	14	21828744	21828757
chr2_3683	p2	(TC)6	12	21838435	21838446
chr2_3684	c	(TG)15gc(TG)13	58	21841309	21841366
chr2_3685	p2	(TG)20	40	21851164	21851203
chr2_3686	p2	(AC)6	12	21851996	21852007
chr2_3687	p5	(TCTTC)5	25	21856119	21856143
chr2_3688	p2	(AG)7	14	21859536	21859549
chr2_3689	c	gaaatggtagtcctcactcccaatgtacatttacctggacctcacctcattgc	126	21860680	21860805
chr2_3690	p2	(GT)10	20	21864254	21864273
chr2_3691	p3	(TCA)5	15	21866904	21866918
chr2_3692	c	gaga(AGG)5aagac(GAG)6aaagaggaagaggagcaggacaa	139	21868380	21868518
chr2_3693	p2	(TG)12	24	21877775	21877798
chr2_3694	p2	(TC)6	12	21879822	21879833
chr2_3695	p2	(GA)8	16	21883856	21883871
chr2_3696	p2	(GT)11	22	21885768	21885789
chr2_3697	p2	(TG)11	22	21891714	21891735
chr2_3698	p2	(GT)6	12	21900606	21900617
chr2_3699	p2	(CA)9	18	21903534	21903551
chr2_3700	p2	(TG)10	20	21912910	21912929
chr2_3701	p2	(CA)6	12	21920878	21920889
chr2_3702	c	gaatgctgaatccaaagacaagcaggatcacattaccttgtgacataggac	128	21921239	21921366
chr2_3703	p3	(AAT)6	18	21924136	21924153
chr2_3704	p2	(AT)6	12	21925306	21925317
chr2_3705	p2	(CA)7	14	21928322	21928335
chr2_3706	p2	(GA)9	18	21932509	21932526
chr2_3707	p2	(TG)6	12	21932789	21932800
chr2_3708	p2	(TG)7	14	21935097	21935110
chr2_3709	p2	(GT)6	12	21937283	21937294
chr2_3710	p3	(AAC)6	18	21943446	21943463
chr2_3711	p2	(AC)15	30	21945775	21945804
chr2_3712	p2	(CA)13	26	21946087	21946112
chr2_3713	p2	(AT)8	16	21946975	21946990
chr2_3714	p2	(TG)25	50	21958067	21958116
chr2_3715	p2	(TG)6	12	21958472	21958483
chr2_3716	p4	(TCTA)14	56	21960924	21960979
chr2_3717	p2	(GT)7	14	21963529	21963542
chr2_3718	c	(TG)8tatgtatgc(GT)6	37	21967733	21967769
chr2_3719	p2	(TG)10	20	21972251	21972270
chr2_3720	p2	(CA)11	22	21972680	21972701
chr2_3721	c	(CA)13cttacacc(CA)6	46	21973163	21973208
chr2_3722	c	catgttcagcaactgtactgtacacctctccccactctttttacagcacaca	126	21974498	21974623
chr2_3723	p4	(ATCT)5	20	21975438	21975457
chr2_3724	c	(GT)21cactgctctttatgtgtggtatatggggtgta(GT)20	114	21985529	21985642
chr2_3725	p2	(CA)8	16	21985769	21985784
chr2_3726	p2	(TC)15	30	21991219	21991248
chr2_3727	p2	(CA)6	12	21996884	21996895
chr2_3728	p2	(CA)10	20	21997574	21997593
chr2_3729	p2	(TG)9	18	21999245	21999262
chr2_3730	p2	(GT)7	14	21999376	21999389

chr2_3731	p2	(TG)7	14	22005229	22005242
chr2_3732	p4	(TGAC)5	20	22007248	22007267
chr2_3733	c	(GA)6a(AG)7ggg(GA)6	42	22008912	22008953
chr2_3734	p2	(CA)8	16	22011177	22011192
chr2_3735	p2	(TG)12	24	22012994	22013017
chr2_3736	p3	(CTG)7	21	22014405	22014425
chr2_3737	p2	(TC)13	26	22015048	22015073
chr2_3738	p2	(GA)8	16	22015541	22015556
chr2_3739	p2	(TG)6	12	22015756	22015767
chr2_3740	p2	(AC)6	12	22018959	22018970
chr2_3741	p2	(TG)6	12	22023735	22023746
chr2_3742	p2	(AC)12	24	22024200	22024223
chr2_3743	c	cattagatctatgtgctgaatcgctgctccctctacatctctcccatcctcccc	107	22025803	22025909
chr2_3744	p2	(CA)7	14	22028426	22028439
chr2_3745	p2	(TG)8	16	22033938	22033953
chr2_3746	p2	(TC)8	16	22044472	22044487
chr2_3747	p2	(AC)6	12	22046795	22046806
chr2_3748	p2	(CA)15	30	22050858	22050887
chr2_3749	p2	(TG)16	32	22058648	22058679
chr2_3750	p2	(GT)6	12	22060125	22060136
chr2_3751	p2	(TG)8	16	22060239	22060254
chr2_3752	c*	(CA)9(CAC)6*ag(AC)7	50	22060389	22060438
chr2_3753	p2	(GT)6	12	22061029	22061040
chr2_3754	p2	(CT)9	18	22069508	22069525
chr2_3755	c	(CCT)7(CAT)5	36	22074472	22074507
chr2_3756	p3	(TCC)6	18	22082100	22082117
chr2_3757	c	(CA)6g(AC)12	37	22085529	22085565
chr2_3758	c	(AC)11(AT)15	52	22085834	22085885
chr2_3759	p2	(TG)6	12	22086423	22086434
chr2_3760	c	(TC)6tttctcaactctctttcttatg(CA)6	46	22089840	22089885
chr2_3761	c	(TA)6c(AT)13	39	22090637	22090675
chr2_3762	c	(GT)6atata(TG)25	67	22091708	22091774
chr2_3763	c	(TG)9g(GT)15	49	22093191	22093239
chr2_3764	p2	(TG)25	50	22099115	22099164
chr2_3765	c	tgtcagtgcggtaagtgtgtgtgcatgcatgtgcatgtgaaatgcaaaaatg	94	22100815	22100908
chr2_3766	c	(AG)6(TG)7	26	22101179	22101204
chr2_3767	p2	(GT)14	28	22103567	22103594
chr2_3768	p2	(AC)16	32	22103766	22103797
chr2_3769	p2	(GT)13	26	22104250	22104275
chr2_3770	c	(TG)16(AG)25tgtgt(GA)10	107	22104772	22104878
chr2_3771	p2	(TC)16	32	22108514	22108545
chr2_3772	p2	(AC)8	16	22109902	22109917
chr2_3773	p4	(AAAT)8	32	22111067	22111098
chr2_3774	p2	(TG)19	38	22127643	22127680
chr2_3775	p2	(AT)6	12	22135298	22135309
chr2_3776	p2	(TG)8	16	22151968	22151983
chr2_3777	p2	(AC)9	18	22153017	22153034
chr2_3778	p2	(CA)6	12	22156357	22156368
chr2_3779	p2	(CA)6	12	22159947	22159958
chr2_3780	p4	(ATTC)5	20	22164832	22164851
chr2_3781	p2	(GT)7	14	22166140	22166153
chr2_3782	p4	(ATTG)6	24	22166642	22166665

chr2_3783	p2	(CA)19	38	22167832	22167869
chr2_3784	p2	(TC)6	12	22171145	22171156
chr2_3785	p2	(CT)12	24	22171582	22171605
chr2_3786	p3	(AAG)6	18	22172410	22172427
chr2_3787	p2	(GT)11	22	22175189	22175210
chr2_3788	p2	(TG)7	14	22176562	22176575
chr2_3789	p2	(GT)20	40	22179334	22179373
chr2_3790	p3	(TTA)5	15	22179709	22179723
chr2_3791	p2	(TC)7	14	22180929	22180942
chr2_3792	p2	(AG)6	12	22182519	22182530
chr2_3793	c	(TG)8(AG)9	34	22184866	22184899
chr2_3794	p4	(TTTA)5	20	22191924	22191943
chr2_3795	p3	(CTT)5	15	22192519	22192533
chr2_3796	p2	(GA)7	14	22194349	22194362
chr2_3797	p3	(TCT)10	30	22217324	22217353
chr2_3798	p2	(GA)6	12	22221878	22221889
chr2_3799	p4	(GATA)6	24	22226952	22226975
chr2_3800	p2	(TA)7	14	22232986	22232999
chr2_3801	p2	(AG)9	18	22233223	22233240
chr2_3802	p4	(ATAC)6	24	22241030	22241053
chr2_3803	p4	(AAAC)5	20	22245870	22245889
chr2_3804	c	(AC)8(AG)14	44	22250149	22250192
chr2_3805	p4	(AAAT)5	20	22262773	22262792
chr2_3806	c	(TA)6gaa(AG)6	27	22267083	22267109
chr2_3807	p2	(CA)8	16	22276703	22276718
chr2_3808	p2	(TG)9	18	22281263	22281280
chr2_3809	p3	(TAG)5	15	22282790	22282804
chr2_3810	p4	(GAAG)6	24	22284205	22284228
chr2_3811	p2	(GT)6	12	22291762	22291773
chr2_3812	p2	(TG)11	22	22300422	22300443
chr2_3813	p2	(TG)6	12	22317087	22317098
chr2_3814	p2	(AG)8	16	22324832	22324847
chr2_3815	p4	(ATCT)23	92	22330516	22330607
chr2_3816	p2	(TG)6	12	22336024	22336035
chr2_3817	p3	(AAT)5	15	22338778	22338792
chr2_3818	p4	(TTGA)5	20	22348225	22348244
chr2_3819	p2	(AC)9	18	22349098	22349115
chr2_3820	p2	(TA)10	20	22351610	22351629
chr2_3821	p2	(CA)6	12	22357776	22357787
chr2_3822	p2	(AC)9	18	22362035	22362052
chr2_3823	p2	(TG)12	24	22380794	22380817
chr2_3824	p4	(ATGA)9	36	22386968	22387003
chr2_3825	p2	(AT)6	12	22393366	22393377
chr2_3826	p2	(AT)9	18	22394397	22394414
chr2_3827	p2	(GT)7	14	22397187	22397200
chr2_3828	p2	(TG)13	26	22409625	22409650
chr2_3829	p2	(GT)6	12	22414638	22414649
chr2_3830	p3	(TTA)6	18	22425344	22425361
chr2_3831	p2	(GT)6	12	22430859	22430870
chr2_3832	p3	(GAA)5	15	22433420	22433434
chr2_3833	p2	(AC)9	18	22433971	22433988
chr2_3834	p2	(GT)8	16	22445099	22445114

chr2_3835	p5	(CAAAT)6	30	22451601	22451630
chr2_3836	c	gatagatagataaatagac(AGAT)5aatttatgtatgtgcaatgctaa(	102	22489503	22489604
chr2_3837	p2	(TG)6	12	22494504	22494515
chr2_3838	p2	(GT)6	12	22519562	22519573
chr2_3839	p4	(TATC)6	24	22525384	22525407
chr2_3840	p2	(TG)10	20	22527772	22527791
chr2_3841	p5	(ATCAA)6	30	22530549	22530578
chr2_3842	p2	(TG)15	30	22530965	22530994
chr2_3843	p4	(TCCA)5	20	22531520	22531539
chr2_3844	c	(TC)7(AC)12	38	22542572	22542609
chr2_3845	p4	(TAGA)24	96	22576261	22576356
chr2_3846	p3	(AAT)5	15	22576791	22576805
chr2_3847	p2	(GT)9	18	22588095	22588112
chr2_3848	p4	(ATTT)5	20	22588390	22588409
chr2_3849	p2	(CA)17	34	22596817	22596850
chr2_3850	p2	(TG)14	28	22601626	22601653
chr2_3851	p2	(TG)20	40	22602103	22602142
chr2_3852	p2	(AC)6	12	22608079	22608090
chr2_3853	p5	(ATTTG)5	25	22610036	22610060
chr2_3854	p2	(TC)8	16	22629203	22629218
chr2_3855	p2	(TG)9	18	22636572	22636589
chr2_3856	c	(CT)6t(TC)6	25	22637324	22637348
chr2_3857	p2	(GA)11	22	22677068	22677089
chr2_3858	p3	(TCA)5	15	22677927	22677941
chr2_3859	p2	(AC)23	46	22687078	22687123
chr2_3860	c	(AT)9g(CA)11	41	22689928	22689968
chr2_3861	c	TC)10gctctctctttctttctctttcttctccgtccttgtcctttctca(CCT)	80	22694384	22694463
chr2_3862	p2	(GT)7	14	22699258	22699271
chr2_3863	p2	(TG)7	14	22700316	22700329
chr2_3864	p2	(TG)10	20	22701172	22701191
chr2_3865	p2	(AC)9	18	22701402	22701419
chr2_3866	p2	(GT)24	48	22703475	22703522
chr2_3867	p2	(AC)7	14	22703805	22703818
chr2_3868	p2	(TG)8	16	22710259	22710274
chr2_3869	p2	(AC)6	12	22713920	22713931
chr2_3870	p2	(AC)9	18	22720056	22720073
chr2_3871	p2	(TC)6	12	22726409	22726420
chr2_3872	c	(TC)22g(CT)7	59	22729373	22729431
chr2_3873	p2	(CA)25	50	22736101	22736150
chr2_3874	p3	(ATA)6	18	22751593	22751610
chr2_3875	p2	(TA)7	14	22762538	22762551
chr2_3876	p2	(TG)18	36	22796045	22796080
chr2_3877	p2	(AT)6	12	22799494	22799505
chr2_3878	p2	(TG)12	24	22804661	22804684
chr2_3879	p2	(AG)17	34	22805265	22805298
chr2_3880	p3	(CTG)5	15	22816600	22816614
chr2_3881	p2	(AC)6	12	22827125	22827136
chr2_3882	p2	(AC)16	32	22827361	22827392
chr2_3883	p2	(CA)10	20	22830240	22830259
chr2_3884	p2	(GA)6	12	22832332	22832343
chr2_3885	p2	(CA)15	30	22834051	22834080
chr2_3886	p4	(GATA)6	24	22836292	22836315

chr2_3887	p2	(TG)17	34	22836695	22836728
chr2_3888	p2	(CA)12	24	22844647	22844670
chr2_3889	p2	(GT)7	14	22850417	22850430
chr2_3890	c	(GT)13(GA)16	58	22853829	22853886
chr2_3891	p2	(CT)6	12	22855888	22855899
chr2_3892	p2	(AC)26	52	22857771	22857822
chr2_3893	p2	(GT)7	14	22865904	22865917
chr2_3894	p2	(CA)7	14	22870385	22870398
chr2_3895	p3	(CAG)5	15	22872832	22872846
chr2_3896	p2	(TA)7	14	22880659	22880672
chr2_3897	p2	(AC)6	12	22887537	22887548
chr2_3898	p2	(CT)6	12	22891560	22891571
chr2_3899	p2	(CT)6	12	22891685	22891696
chr2_3900	p3	(TAA)5	15	22895285	22895299
chr2_3901	p2	(AG)6	12	22913323	22913334
chr2_3902	p2	(CA)17	34	22917202	22917235
chr2_3903	p2	(CT)10	20	22929020	22929039
chr2_3904	p2	(TG)6	12	22931414	22931425
chr2_3905	p3	(AAC)5	15	22932531	22932545
chr2_3906	p2	(AC)6	12	22945520	22945531
chr2_3907	c	(AG)9taaa(TG)10	42	22952840	22952881
chr2_3908	p2	(AG)6	12	22970474	22970485
chr2_3909	p2	(TG)17	34	22971249	22971282
chr2_3910	p2	(AC)17	34	22995513	22995546
chr2_3911	p2	(CA)6	12	23003015	23003026
chr2_3912	p2	(GT)6	12	23006818	23006829
chr2_3913	p2	(GA)6	12	23008661	23008672
chr2_3914	p2	(AC)12	24	23009765	23009788
chr2_3915	p2	(GT)6	12	23016080	23016091
chr2_3916	p2	(CA)6	12	23018278	23018289
chr2_3917	p3	(TAA)5	15	23019062	23019076
chr2_3918	p2	(TG)15	30	23019819	23019848
chr2_3919	p2	(AC)6	12	23019953	23019964
chr2_3920	p2	(AC)6	12	23020183	23020194
chr2_3921	p2	(AT)6	12	23020537	23020548
chr2_3922	p2	(AG)11	22	23023198	23023219
chr2_3923	p2	(TC)19	38	23027862	23027899
chr2_3924	c	(CTT)5(TC)14	43	23032510	23032552
chr2_3925	c	(GA)7acgaagtgttcatctctgggggaacagagagtggggaa(AG)6	64	23047116	23047179
chr2_3926	p2	(AT)8	16	23052358	23052373
chr2_3927	p2	(AC)6	12	23076231	23076242
chr2_3928	p2	(CA)9	18	23082025	23082042
chr2_3929	p2	(CA)13	26	23082601	23082626
chr2_3930	p2	(CA)14	28	23082897	23082924
chr2_3931	p2	(GT)15	30	23092671	23092700
chr2_3932	p3	(CAT)5	15	23093331	23093345
chr2_3933	p5	(ATAAT)5	25	23096820	23096844
chr2_3934	p2	(CA)29	58	23097218	23097275
chr2_3935	p2	(CA)24	48	23097768	23097815
chr2_3936	p2	(CA)12	24	23099073	23099096
chr2_3937	c	(GT)6tc(GT)9	32	23101403	23101434
chr2_3938	p2	(AC)6	12	23102177	23102188

chr2_3939	p2	(GT)8	16	23107045	23107060
chr2_3940	p2	(TG)6	12	23107356	23107367
chr2_3941	c	(AC)8t(CA)8	33	23110437	23110469
chr2_3942	c	cagaagccagagtgtgagcaagagatgggaagagaagaagggggttgtg	91	23111890	23111980
chr2_3943	c	(TC)9(AC)12	42	23113131	23113172
chr2_3944	p2	(TG)19	38	23113459	23113496
chr2_3945	p2	(TG)15	30	23113722	23113751
chr2_3946	c	gagtgggtcaggattgagacaaccggagtctggcccttgctttcttaccctc	118	23114557	23114674
chr2_3947	p2	(GT)13	26	23115369	23115394
chr2_3948	p2	(GT)14	28	23115622	23115649
chr2_3949	p2	(TG)15	30	23117793	23117822
chr2_3950	p2	(TC)21	42	23119417	23119458
chr2_3951	p2	(TG)6	12	23119620	23119631
chr2_3952	p2	(TG)19	38	23119766	23119803
chr2_3953	p2	(GT)7	14	23121045	23121058
chr2_3954	p2	(AT)6	12	23129704	23129715
chr2_3955	p2	(TG)24	48	23147363	23147410
chr2_3956	p2	(TG)6	12	23148098	23148109
chr2_3957	p2	(AC)9	18	23148308	23148325
chr2_3958	p2	(CA)16	32	23148870	23148901
chr2_3959	p2	(AG)6	12	23149749	23149760
chr2_3960	p2	(TG)7	14	23151428	23151441
chr2_3961	p2	(TG)6	12	23151777	23151788
chr2_3962	c	(CT)10(CA)8	36	23152131	23152166
chr2_3963	p2	(CA)7	14	23154907	23154920
chr2_3964	c	(CG)8(CA)9	34	23155075	23155108
chr2_3965	p2	(GT)18	36	23156659	23156694
chr2_3966	p3	(ATA)6	18	23160948	23160965
chr2_3967	p2	(CA)27	54	23172023	23172076
chr2_3968	p2	(AC)8	16	23176204	23176219
chr2_3969	p2	(AC)14	28	23178314	23178341
chr2_3970	p2	(AC)14	28	23182676	23182703
chr2_3971	p2	(AC)7	14	23193118	23193131
chr2_3972	p2	(GA)7	14	23197859	23197872
chr2_3973	p2	(TG)6	12	23202280	23202291
chr2_3974	p2	(TG)6	12	23204548	23204559
chr2_3975	p2	(TG)6	12	23208171	23208182
chr2_3976	p2	(AC)12	24	23208788	23208811
chr2_3977	c	(AC)10agagagagc(GA)18	65	23213701	23213765
chr2_3978	p2	(AC)6	12	23220193	23220204
chr2_3979	p2	(TG)13	26	23220530	23220555
chr2_3980	p2	(TG)6	12	23220773	23220784
chr2_3981	p2	(TC)6	12	23221175	23221186
chr2_3982	p2	(GT)15	30	23222714	23222743
chr2_3983	p2	(CT)6	12	23222897	23222908
chr2_3984	p2	(GT)6	12	23224224	23224235
chr2_3985	p2	(AC)12	24	23225167	23225190
chr2_3986	p2	(TG)10	20	23225481	23225500
chr2_3987	c	(GA)6ag(GA)6	26	23226477	23226502
chr2_3988	p2	(AC)13	26	23226711	23226736
chr2_3989	p2	(AC)6	12	23228147	23228158
chr2_3990	p2	(CA)14	28	23228520	23228547

chr2_3991	p2	(TG)15	30	23228657	23228686
chr2_3992	p2	(GT)14	28	23229708	23229735
chr2_3993	p2	(AC)7	14	23230716	23230729
chr2_3994	c	(AC)6at(AC)13	40	23231090	23231129
chr2_3995	p2	(AC)12	24	23231369	23231392
chr2_3996	p2	(TG)12	24	23231537	23231560
chr2_3997	p2	(TG)7	14	23232145	23232158
chr2_3998	c	(TC)7(AC)6	26	23232588	23232613
chr2_3999	p2	(TA)8	16	23233158	23233173
chr2_4000	p2	(TG)10	20	23241378	23241397
chr2_4001	p2	(AC)18	36	23241542	23241577
chr2_4002	c	(TG)8tc(TG)6tc(TG)6	44	23242237	23242280
chr2_4003	p2	(GT)7	14	23243029	23243042
chr2_4004	p4	(ATTG)7	28	23243591	23243618
chr2_4005	p2	(AC)7	14	23245160	23245173
chr2_4006	p2	(GT)6	12	23245424	23245435
chr2_4007	c	(CA)9(TA)6	30	23246348	23246377
chr2_4008	p2	(CA)8	16	23247312	23247327
chr2_4009	c	(CA)30c(CA)6	73	23251183	23251255
chr2_4010	p2	(AC)11	22	23253739	23253760
chr2_4011	p2	(GA)15	30	23257453	23257482
chr2_4012	p3	(TAA)6	18	23273843	23273860
chr2_4013	p2	(TA)6	12	23291720	23291731
chr2_4014	p2	(AC)6	12	23297527	23297538
chr2_4015	p2	(GT)15	30	23301416	23301445
chr2_4016	p2	(CA)9	18	23317223	23317240
chr2_4017	p4	(TAGG)5	20	23320709	23320728
chr2_4018	p3	(AAT)5	15	23326094	23326108
chr2_4019	p3	(ATA)14	42	23341569	23341610
chr2_4020	p3	(TAC)5	15	23377127	23377141
chr2_4021	p3	(TCC)9	27	23385172	23385198
chr2_4022	p2	(GT)18	36	23386148	23386183
chr2_4023	p2	(GT)29	58	23401784	23401841
chr2_4024	p2	(TG)6	12	23409309	23409320
chr2_4025	p2	(GT)6	12	23409948	23409959
chr2_4026	p2	(AC)6	12	23410243	23410254
chr2_4027	p2	(TG)7	14	23416553	23416566
chr2_4028	p3	(GAT)8	24	23417309	23417332
chr2_4029	p2	(TG)10	20	23418001	23418020
chr2_4030	p4	(ATGG)5	20	23424754	23424773
chr2_4031	p2	(AG)6	12	23426571	23426582
chr2_4032	p2	(TG)6	12	23437166	23437177
chr2_4033	p2	(AC)8	16	23443792	23443807
chr2_4034	p2	(GT)10	20	23449959	23449978
chr2_4035	p2	(TA)6	12	23451599	23451610
chr2_4036	p2	(TC)7	14	23454863	23454876
chr2_4037	p2	(TC)6	12	23455447	23455458
chr2_4038	p2	(TG)35	70	23455612	23455681
chr2_4039	p2	(TG)13	26	23457609	23457634
chr2_4040	p2	(AC)6	12	23459644	23459655
chr2_4041	p2	(GA)10	20	23461142	23461161
chr2_4042	p2	(TC)6	12	23462193	23462204

chr2_4043	p2	(AC)10	20	23462672	23462691
chr2_4044	p2	(TG)6	12	23463651	23463662
chr2_4045	p2	(CA)6	12	23480427	23480438
chr2_4046	p2	(TC)9	18	23483456	23483473
chr2_4047	p2	(TG)15	30	23485440	23485469
chr2_4048	p2	(TC)9	18	23506500	23506517
chr2_4049	p2	(AC)14	28	23521589	23521616
chr2_4050	p3	(TCT)5	15	23532886	23532900
chr2_4051	p2	(AC)14	28	23556362	23556389
chr2_4052	p2	(TG)13	26	23560599	23560624
chr2_4053	p2	(GT)8	16	23567087	23567102
chr2_4054	p2	(TG)6	12	23567627	23567638
chr2_4055	p2	(TG)11	22	23574679	23574700
chr2_4056	p2	(AC)25	50	23579945	23579994
chr2_4057	p2	(CA)6	12	23583323	23583334
chr2_4058	p2	(AC)6	12	23583657	23583668
chr2_4059	p2	(CA)21	42	23584225	23584266
chr2_4060	p4	(ATTT)6	24	23601697	23601720
chr2_4061	p3	(ATT)6	18	23613502	23613519
chr2_4062	p2	(AC)18	36	23622820	23622855
chr2_4063	p3	(AAC)13	39	23627925	23627963
chr2_4064	p2	(GT)48	96	23633925	23634020
chr2_4065	p2	(AC)6	12	23641849	23641860
chr2_4066	p3	(TTG)5	15	23646551	23646565
chr2_4067	p2	(GT)12	24	23647332	23647355
chr2_4068	p2	(AC)6	12	23648591	23648602
chr2_4069	p2	(TA)6	12	23649228	23649239
chr2_4070	p2	(AG)6	12	23656644	23656655
chr2_4071	p2	(TG)6	12	23673127	23673138
chr2_4072	p2	(AC)7	14	23675624	23675637
chr2_4073	p3	(AGT)8	24	23683907	23683930
chr2_4074	p3	(TTG)5	15	23731083	23731097
chr2_4075	p2	(TG)6	12	23734981	23734992
chr2_4076	p2	(AT)9	18	23738851	23738868
chr2_4077	p2	(TG)8	16	23748341	23748356
chr2_4078	c	'G)21ctttcacatctcagtgttagtactttttagtgcttgttgtgtgtc(TG)1	111	23765884	23765994
chr2_4079	p5	(GCTAA)5	25	23770296	23770320
chr2_4080	p2	(TG)8	16	23778631	23778646
chr2_4081	p2	(AC)17	34	23782131	23782164
chr2_4082	p2	(TG)10	20	23784136	23784155
chr2_4083	c	(GT)19ttgtgtgtaagcacagctatatgatgtcttctttcgcgc(GT)18	113	23789444	23789556
chr2_4084	p2	(AG)9	18	23790924	23790941
chr2_4085	c	aacataaccctctcattgtgtgccatgtccaaatagaccagttttatattagac	138	23795851	23795988
chr2_4086	p2	(CA)7	14	23800083	23800096
chr2_4087	p3	(AAT)5	15	23806511	23806525
chr2_4088	p2	(TG)13	26	23807283	23807308
chr2_4089	p3	(CAG)5	15	23817494	23817508
chr2_4090	p2	(AC)7	14	23830801	23830814
chr2_4091	p2	(TG)14	28	23836537	23836564
chr2_4092	p3	(CTT)5	15	23836701	23836715
chr2_4093	p2	(GT)6	12	23840932	23840943
chr2_4094	p2	(GT)6	12	23842667	23842678

chr2_4095	p2	(AT)7	14	23847986	23847999
chr2_4096	c	(AC)14(CT)8ttcttgctct(CA)6	66	23848670	23848735
chr2_4097	p2	(AC)6	12	23863482	23863493
chr2_4098	p2	(TG)6	12	23872607	23872618
chr2_4099	p2	(GT)7	14	23897318	23897331
chr2_4100	p5	(AATCA)5	25	23908895	23908919
chr2_4101	c	(TG)22(GT)10	64	23916874	23916937
chr2_4102	p2	(GT)10	20	23919465	23919484
chr2_4103	c	(TA)6c(GT)15	43	23921378	23921420
chr2_4104	p3	(AGG)5	15	23931659	23931673
chr2_4105	c	(AC)6(AT)6	24	23959101	23959124
chr2_4106	p4	(AATA)5	20	23971302	23971321
chr2_4107	c	(GT)10t(TG)9	39	23989687	23989725
chr2_4108	p2	(AG)6	12	23990208	23990219
chr2_4109	p2	(TC)25	50	23994996	23995045
chr2_4110	p2	(CA)16	32	23999872	23999903
chr2_4111	p2	(GT)6	12	24000017	24000028
chr2_4112	p3	(AGG)6	18	24011965	24011982
chr2_4113	p2	(TG)12	24	24021003	24021026
chr2_4114	c	(CT)9cctttgtgcatg(CA)6	42	24024952	24024993
chr2_4115	p2	(GT)18	36	24031366	24031401
chr2_4116	p3	(TTG)6	18	24052708	24052725
chr2_4117	p2	(CA)6	12	24053780	24053791
chr2_4118	p2	(GT)9	18	24068956	24068973
chr2_4119	p2	(CA)7	14	24072715	24072728
chr2_4120	p3	(TCA)5	15	24097215	24097229
chr2_4121	p2	(GT)24	48	24126270	24126317
chr2_4122	p2	(GT)19	38	24128927	24128964
chr2_4123	p2	(AC)15	30	24130753	24130782
chr2_4124	p3	(GCT)5	15	24133816	24133830
chr2_4125	p2	(TG)7	14	24138951	24138964
chr2_4126	p2	(AC)6	12	24139488	24139499
chr2_4127	p2	(AC)6	12	24140888	24140899
chr2_4128	p2	(CA)7	14	24144394	24144407
chr2_4129	p4	(TGAA)7	28	24152786	24152813
chr2_4130	p2	(TG)7	14	24156442	24156455
chr2_4131	p2	(TG)8	16	24157150	24157165
chr2_4132	p2	(TC)10	20	24159401	24159420
chr2_4133	p2	(AC)7	14	24170101	24170114
chr2_4134	p2	(AT)8	16	24182245	24182260
chr2_4135	p2	(AC)6	12	24191257	24191268
chr2_4136	p2	(GA)8	16	24205104	24205119
chr2_4137	p2	(AC)7	14	24215434	24215447
chr2_4138	p2	(CA)8	16	24228094	24228109
chr2_4139	p2	(AC)6	12	24228354	24228365
chr2_4140	p2	(GA)8	16	24229046	24229061
chr2_4141	p2	(TG)11	22	24231382	24231403
chr2_4142	p2	(TG)10	20	24236156	24236175
chr2_4143	p2	(TC)10	20	24237924	24237943
chr2_4144	c	(GA)7(GT)6	26	24238087	24238112
chr2_4145	c	(GT)7gcacc(AG)6	31	24241132	24241162
chr2_4146	c	(AC)8atatgcacaa(AC)9	44	24242324	24242367

chr2_4147	p3	(ATA)7	21	24243565	24243585
chr2_4148	p2	(TG)7	14	24244691	24244704
chr2_4149	p2	(TA)6	12	24246470	24246481
chr2_4150	p2	(AG)7	14	24251490	24251503
chr2_4151	p2	(TG)9	18	24254031	24254048
chr2_4152	p2	(TA)7	14	24260983	24260996
chr2_4153	p2	(CT)8	16	24261879	24261894
chr2_4154	p2	(TG)6	12	24274025	24274036
chr2_4155	p2	(TG)18	36	24277426	24277461
chr2_4156	p3	(AAT)5	15	24283565	24283579
chr2_4157	p2	(AG)7	14	24284230	24284243
chr2_4158	p5	(CAAAT)6	30	24297150	24297179
chr2_4159	p2	(TG)17	34	24304126	24304159
chr2_4160	p2	(AC)8	16	24310171	24310186
chr2_4161	p4	(CCAT)6	24	24311064	24311087
chr2_4162	p2	(TG)14	28	24314066	24314093
chr2_4163	p3	(AGA)8	24	24315248	24315271
chr2_4164	p2	(AT)13	26	24316370	24316395
chr2_4165	p2	(TG)15	30	24325368	24325397
chr2_4166	p2	(CA)6	12	24325776	24325787
chr2_4167	p2	(GA)7	14	24328535	24328548
chr2_4168	p4	(AAAG)8	32	24330323	24330354
chr2_4169	p2	(CA)12	24	24331519	24331542
chr2_4170	p2	(GA)17	34	24333338	24333371
chr2_4171	p3	(TCC)5	15	24336851	24336865
chr2_4172	p2	(TG)18	36	24337463	24337498
chr2_4173	p2	(GT)7	14	24354921	24354934
chr2_4174	p2	(AC)9	18	24364211	24364228
chr2_4175	p2	(AG)7	14	24372685	24372698
chr2_4176	p2	(TA)7	14	24376147	24376160
chr2_4177	p2	(CA)9	18	24378582	24378599
chr2_4178	p2	(TG)6	12	24389191	24389202
chr2_4179	p3	(ACA)5	15	24391185	24391199
chr2_4180	p2	(AT)8	16	24394506	24394521
chr2_4181	p2	(CA)8	16	24395567	24395582
chr2_4182	p2	(TG)24	48	24396973	24397020
chr2_4183	p2	(TA)7	14	24401550	24401563
chr2_4184	p2	(AC)24	48	24403928	24403975
chr2_4185	p2	(TG)6	12	24413177	24413188
chr2_4186	p2	(TG)22	44	24422624	24422667
chr2_4187	c	(AC)21gtcctgtgttataggtgttgctctctaccttt(AC)18	109	24422938	24423046
chr2_4188	p2	(GT)15	30	24430698	24430727
chr2_4189	p2	(CA)16	32	24433926	24433957
chr2_4190	p2	(TG)13	26	24441714	24441739
chr2_4191	p5	(AAATC)6	30	24446555	24446584
chr2_4192	p2	(AC)6	12	24448662	24448673
chr2_4193	p2	(GT)7	14	24449146	24449159
chr2_4194	p3	(TCT)5	15	24449687	24449701
chr2_4195	c	(CA)9atct(CA)13	48	24452617	24452664
chr2_4196	p2	(AC)6	12	24457309	24457320
chr2_4197	p3	(CTT)5	15	24470637	24470651
chr2_4198	p2	(CA)27	54	24470970	24471023

chr2_4199	p2	(TC)9	18	24472575	24472592
chr2_4200	p2	(CA)17	34	24472816	24472849
chr2_4201	p2	(TG)7	14	24479647	24479660
chr2_4202	p3	(TAT)5	15	24480614	24480628
chr2_4203	p2	(TA)12	24	24481414	24481437
chr2_4204	p3	(AAC)7	21	24485392	24485412
chr2_4205	p3	(TAG)5	15	24489215	24489229
chr2_4206	p2	(TG)7	14	24494140	24494153
chr2_4207	p2	(AG)15	30	24498142	24498171
chr2_4208	p2	(GT)16	32	24501585	24501616
chr2_4209	p2	(GT)19	38	24510348	24510385
chr2_4210	p2	(TG)14	28	24511579	24511606
chr2_4211	p2	(CA)6	12	24512995	24513006
chr2_4212	p2	(CT)17	34	24515028	24515061
chr2_4213	p2	(GT)11	22	24515977	24515998
chr2_4214	p2	(CA)16	32	24519495	24519526
chr2_4215	p2	(AC)6	12	24520637	24520648
chr2_4216	c	(TA)8(GA)10	36	24523394	24523429
chr2_4217	p2	(CA)6	12	24525781	24525792
chr2_4218	p2	(AC)7	14	24529899	24529912
chr2_4219	p2	(AC)32	64	24530402	24530465
chr2_4220	p2	(CT)21	42	24531632	24531673
chr2_4221	p2	(TG)6	12	24536729	24536740
chr2_4222	p2	(CA)23	46	24541056	24541101
chr2_4223	p2	(CA)9	18	24543169	24543186
chr2_4224	p2	(GT)6	12	24550239	24550250
chr2_4225	p2	(TG)7	14	24550888	24550901
chr2_4226	p2	(CA)13	26	24555912	24555937
chr2_4227	p2	(TG)9	18	24557415	24557432
chr2_4228	p2	(AT)7	14	24558741	24558754
chr2_4229	p2	(GT)17	34	24559246	24559279
chr2_4230	p2	(AT)7	14	24560007	24560020
chr2_4231	p2	(GT)17	34	24560512	24560545
chr2_4232	p2	(GT)9	18	24560754	24560771
chr2_4233	p2	(GT)17	34	24568691	24568724
chr2_4234	p2	(AG)7	14	24569869	24569882
chr2_4235	p3	(AAT)5	15	24576365	24576379
chr2_4236	p2	(AC)6	12	24580370	24580381
chr2_4237	p2	(AC)17	34	24597184	24597217
chr2_4238	p2	(TG)8	16	24597516	24597531
chr2_4239	p2	(AT)6	12	24600271	24600282
chr2_4240	p2	(AC)18	36	24606289	24606324
chr2_4241	p2	(AC)6	12	24611647	24611658
chr2_4242	p2	(AC)7	14	24617571	24617584
chr2_4243	p2	(TA)7	14	24625292	24625305
chr2_4244	p2	(GT)12	24	24627399	24627422
chr2_4245	p2	(TA)6	12	24631371	24631382
chr2_4246	p2	(CA)8	16	24638419	24638434
chr2_4247	p2	(AT)13	26	24639746	24639771
chr2_4248	p2	(TA)7	14	24641142	24641155
chr2_4249	p2	(CT)7	14	24643364	24643377
chr2_4250	p2	(TA)12	24	24651450	24651473

chr2_4251	p2	(AC)20	40	24660513	24660552
chr2_4252	p2	(TG)9	18	24680760	24680777
chr2_4253	c	(TG)17agt(GA)16	69	24680937	24681005
chr2_4254	p2	(GT)7	14	24681425	24681438
chr2_4255	p2	(CA)6	12	24682937	24682948
chr2_4256	c	(AC)9(CA)12	42	24684760	24684801
chr2_4257	p2	(TG)8	16	24685623	24685638
chr2_4258	p2	(AC)6	12	24686004	24686015
chr2_4259	p2	(AG)16	32	24688327	24688358
chr2_4260	p2	(TC)13	26	24691258	24691283
chr2_4261	p2	(TC)14	28	24691393	24691420
chr2_4262	p3	(CAG)5	15	24692819	24692833
chr2_4263	c	GT)20gagtgagtgagtgagtgaggggggggagagagggggc(GA)1	104	24704897	24705000
chr2_4264	c	(AC)9aa(AC)6	32	24723960	24723991
chr2_4265	c	(TC)6(AC)10	32	24724879	24724910
chr2_4266	p2	(AG)7	14	24727465	24727478
chr2_4267	p2	(GT)8	16	24736442	24736457
chr2_4268	p2	(TG)6	12	24740767	24740778
chr2_4269	p2	(GT)11	22	24740911	24740932
chr2_4270	c	(GA)8a(AG)6	29	24742171	24742199
chr2_4271	p3	(AAC)5	15	24744364	24744378
chr2_4272	p2	(TG)14	28	24753913	24753940
chr2_4273	p2	(AC)15	30	24754720	24754749
chr2_4274	p2	(CA)15	30	24758574	24758603
chr2_4275	p2	(TG)8	16	24758734	24758749
chr2_4276	p2	(TG)13	26	24762700	24762725
chr2_4277	p2	(GT)6	12	24766397	24766408
chr2_4278	p2	(CA)16	32	24773057	24773088
chr2_4279	p5	(GAAGA)10	50	24784599	24784648
chr2_4280	p2	(CA)6	12	24784979	24784990
chr2_4281	p2	(CT)8	16	24787427	24787442
chr2_4282	p2	(GT)8	16	24787674	24787689
chr2_4283	p2	(GT)8	16	24798947	24798962
chr2_4284	p2	(AG)6	12	24799601	24799612
chr2_4285	p3	(ATA)7	21	24805230	24805250
chr2_4286	c	nnnnnnnnnnnnnnnnnnnnnnnnnnnnnnnnnnnnnnnnn	139	24808542	24808680
chr2_4287	p2	(CT)6	12	24810175	24810186
chr2_4288	p2	(AC)15	30	24817298	24817327
chr2_4289	p2	(GT)12	24	24823102	24823125
chr2_4290	p2	(GT)6	12	24827036	24827047
chr2_4291	p2	(TG)13	26	24831112	24831137
chr2_4292	c	(TG)6(AG)24	60	24834954	24835013
chr2_4293	p2	(AC)6	12	24840121	24840132
chr2_4294	p4	(ATTT)5	20	24846132	24846151
chr2_4295	p2	(TA)8	16	24850428	24850443
chr2_4296	p3	(TCA)6	18	24852917	24852934
chr2_4297	p2	(AC)16	32	24855716	24855747
chr2_4298	p2	(CA)6	12	24855935	24855946
chr2_4299	p2	(AC)10	20	24867990	24868009
chr2_4300	p4	(AGGT)9	36	24872014	24872049
chr2_4301	p5	(AGAAT)5	25	24874620	24874644
chr2_4302	p2	(TG)16	32	24884631	24884662

chr2_4303	p2	(AC)8	16	24893246	24893261
chr2_4304	p2	(TG)6	12	24901534	24901545
chr2_4305	p2	(GT)8	16	24902085	24902100
chr2_4306	p3	(AAG)7	21	24905081	24905101
chr2_4307	p2	(TG)6	12	24906327	24906338
chr2_4308	p2	(CT)9	18	24908640	24908657
chr2_4309	p2	(TG)8	16	24909660	24909675
chr2_4310	p2	(CA)7	14	24917799	24917812
chr2_4311	p2	(CT)9	18	24923016	24923033
chr2_4312	p3	(AAC)8	24	24936487	24936510
chr2_4313	p2	(CA)20	40	24959506	24959545
chr2_4314	p2	(AC)21	42	24964320	24964361
chr2_4315	p2	(AC)10	20	24965267	24965286
chr2_4316	p2	(AC)12	24	24967090	24967113
chr2_4317	p2	(CA)10	20	24968817	24968836
chr2_4318	p2	(CA)6	12	24971252	24971263
chr2_4319	p2	(AC)6	12	24972629	24972640
chr2_4320	p2	(CA)8	16	24973010	24973025
chr2_4321	p2	(CA)6	12	24975531	24975542
chr2_4322	c	(TG)11tcaggagacagtgttaaagagtggcg(GT)6	60	24977541	24977600
chr2_4323	p2	(TG)6	12	24983030	24983041
chr2_4324	c	(CA)9cgcccacacccacacacgcacacacacacc(CA)18	84	24984796	24984879
chr2_4325	p2	(TG)10	20	24987440	24987459
chr2_4326	p2	(AC)10	20	24991942	24991961
chr2_4327	p2	(GT)23	46	24992906	24992951
chr2_4328	p2	(GT)6	12	24997468	24997479
chr2_4329	p2	(GT)19	38	24998643	24998680
chr2_4330	p2	(CA)6	12	24998786	24998797
chr2_4331	p2	(TG)7	14	25002258	25002271
chr2_4332	p2	(AG)9	18	25010962	25010979
chr2_4333	p2	(CA)8	16	25012763	25012778
chr2_4334	c	(TG)11tc(AG)6	36	25013394	25013429
chr2_4335	p2	(TG)13	26	25017913	25017938
chr2_4336	p2	(CA)6	12	25018143	25018154
chr2_4337	c	(AC)20ag(AC)6	54	25020444	25020497
chr2_4338	p2	(TG)13	26	25020723	25020748
chr2_4339	p2	(AC)21	42	25020919	25020960
chr2_4340	p2	(AC)13	26	25021249	25021274
chr2_4341	c	(TG)6agacccacttagt(TG)6	37	25022332	25022368
chr2_4342	c	(TC)8tgcac(CA)9	39	25022537	25022575
chr2_4343	p2	(GA)16	32	25023670	25023701
chr2_4344	p2	(AC)9	18	25026271	25026288
chr2_4345	p2	(AC)14	28	25026423	25026450
chr2_4346	p2	(TG)6	12	25030738	25030749
chr2_4347	p2	(AT)6	12	25035667	25035678
chr2_4348	p3	(AAC)5	15	25036100	25036114
chr2_4349	p2	(AC)17	34	25039104	25039137
chr2_4350	p2	(TG)11	22	25040293	25040314
chr2_4351	c	(ATA)5a(AAT)7	37	25040849	25040885
chr2_4352	c	(TAT)6(TAC)25	93	25042721	25042813
chr2_4353	p2	(TG)33	66	25046180	25046245
chr2_4354	p4	(TTTG)5	20	25056286	25056305

chr2_4355	p2	(TG)8	16	25062084	25062099
chr2_4356	p2	(CA)7	14	25062244	25062257
chr2_4357	p2	(TC)6	12	25065190	25065201
chr2_4358	p2	(CA)8	16	25079574	25079589
chr2_4359	p2	(TG)8	16	25082192	25082207
chr2_4360	p2	(TC)6	12	25083431	25083442
chr2_4361	p2	(AG)21	42	25085433	25085474
chr2_4362	p2	(GT)7	14	25087917	25087930
chr2_4363	p2	(AG)6	12	25090159	25090170
chr2_4364	p2	(GT)9	18	25093592	25093609
chr2_4365	c	gagaaacacacccagctctccctcatgtgcatatataaatatacagtatgtat	77	25095985	25096061
chr2_4366	p2	(CA)6	12	25103162	25103173
chr2_4367	p4	(TTTA)5	20	25122057	25122076
chr2_4368	p2	(TG)8	16	25123203	25123218
chr2_4369	p2	(TG)13	26	25123682	25123707
chr2_4370	p3	(CTA)8	24	25125204	25125227
chr2_4371	p5	(TAACC)6	30	25133262	25133291
chr2_4372	p2	(AT)8	16	25139225	25139240
chr2_4373	p2	(CA)8	16	25151944	25151959
chr2_4374	p2	(TG)8	16	25175923	25175938
chr2_4375	p2	(TA)8	16	25180626	25180641
chr2_4376	p2	(CA)6	12	25181895	25181906
chr2_4377	p2	(AG)6	12	25184855	25184866
chr2_4378	p3	(ATT)5	15	25192077	25192091
chr2_4379	p2	(AC)6	12	25194956	25194967
chr2_4380	p2	(TA)7	14	25198307	25198320
chr2_4381	p2	(TC)6	12	25204359	25204370
chr2_4382	p2	(CT)12	24	25204801	25204824
chr2_4383	c	atccacacacagagatcattgtgtagaggaggccgtgcgctgcgcgtctga	130	25205214	25205343
chr2_4384	p2	(TC)7	14	25207004	25207017
chr2_4385	p4	(ATCC)7	28	25213414	25213441
chr2_4386	p2	(TC)11	22	25218974	25218995
chr2_4387	p2	(AC)6	12	25220918	25220929
chr2_4388	p2	(AC)9	18	25221313	25221330
chr2_4389	p2	(AG)8	16	25221471	25221486
chr2_4390	c	(CT)6(CA)9	30	25221843	25221872
chr2_4391	p2	(GT)6	12	25223857	25223868
chr2_4392	p5	(TGTAC)7	35	25233270	25233304
chr2_4393	p2	(AC)6	12	25234080	25234091
chr2_4394	p2	(AC)12	24	25245318	25245341
chr2_4395	p2	(AC)8	16	25251683	25251698
chr2_4396	c	(TTA)8ttttattacaattt(TTA)5	53	25255956	25256008
chr2_4397	p2	(TG)9	18	25256153	25256170
chr2_4398	c	agagtttgtacattaatggtgggtcccttggcgctgatatagaccagctcgc	156	25256559	25256714
chr2_4399	p3	(AGT)6	18	25261673	25261690
chr2_4400	c	(CA)6(CG)6	24	25264186	25264209
chr2_4401	p2	(CA)12	24	25265815	25265838
chr2_4402	p2	(CA)18	36	25265993	25266028
chr2_4403	p2	(TG)14	28	25267224	25267251
chr2_4404	p2	(GT)6	12	25269359	25269370
chr2_4405	p2	(CA)19	38	25270847	25270884
chr2_4406	p3	(TGC)6	18	25272110	25272127

chr2_4407	p2	(TC)6	12	25281407	25281418
chr2_4408	p2	(TG)15	30	25284868	25284897
chr2_4409	p3	(TCC)6	18	25285809	25285826
chr2_4410	p2	(AG)9	18	25288273	25288290
chr2_4411	p2	(GT)7	14	25298423	25298436
chr2_4412	p2	(CA)15	30	25302048	25302077
chr2_4413	p2	(GT)11	22	25318395	25318416
chr2_4414	p2	(TG)16	32	25319410	25319441
chr2_4415	p2	(CA)6	12	25319616	25319627
chr2_4416	p2	(CA)10	20	25320758	25320777
chr2_4417	p2	(CA)12	24	25321132	25321155
chr2_4418	p2	(AC)10	20	25323070	25323089
chr2_4419	p2	(AC)26	52	25323248	25323299
chr2_4420	p2	(AG)19	38	25328824	25328861
chr2_4421	p2	(CA)12	24	25333069	25333092
chr2_4422	p2	(CA)12	24	25344777	25344800
chr2_4423	p5	(CTCAA)5	25	25348098	25348122
chr2_4424	p2	(GT)6	12	25352719	25352730
chr2_4425	p2	(CA)8	16	25353860	25353875
chr2_4426	p2	(GT)9	18	25358192	25358209
chr2_4427	p3	(TTA)7	21	25366337	25366357
chr2_4428	p4	(TTTG)5	20	25374987	25375006
chr2_4429	p3	(AAC)5	15	25375144	25375158
chr2_4430	p2	(TG)6	12	25385178	25385189
chr2_4431	p2	(AG)15	30	25387071	25387100
chr2_4432	c	(TG)7c(GT)7	29	25390016	25390044
chr2_4433	p3	(TTG)6	18	25415909	25415926
chr2_4434	p2	(AC)19	38	25430512	25430549
chr2_4435	p3	(ATG)5	15	25431306	25431320
chr2_4436	p3	(GAA)5	15	25450030	25450044
chr2_4437	c	(CA)6cgctcacgggcgcgcgctcgcacg(CA)10	56	25478618	25478673
chr2_4438	p2	(AT)6	12	25504933	25504944
chr2_4439	p2	(TG)6	12	25517028	25517039
chr2_4440	p2	(AC)6	12	25521858	25521869
chr2_4441	p2	(AC)17	34	25523038	25523071
chr2_4442	p3	(TCC)6	18	25525781	25525798
chr2_4443	p2	(TG)7	14	25529448	25529461
chr2_4444	p2	(TG)9	18	25532387	25532404
chr2_4445	p2	(TG)10	20	25532527	25532546
chr2_4446	p2	(TA)8	16	25533084	25533099
chr2_4447	p3	(ATA)6	18	25539299	25539316
chr2_4448	p2	(AG)6	12	25547300	25547311
chr2_4449	p2	(AG)11	22	25548407	25548428
chr2_4450	p2	(AC)6	12	25549618	25549629
chr2_4451	p2	(CT)7	14	25554200	25554213
chr2_4452	p2	(TG)13	26	25558859	25558884
chr2_4453	p2	(TG)6	12	25559115	25559126
chr2_4454	p4	(TGAC)5	20	25562633	25562652
chr2_4455	c	(TA)11cat(AC)18	61	25565407	25565467
chr2_4456	p2	(CA)7	14	25567719	25567732
chr2_4457	p2	(AC)6	12	25569480	25569491
chr2_4458	p2	(TG)15	30	25570903	25570932

chr2_4459	p2	(TG)16	32	25571040	25571071
chr2_4460	p2	(CA)13	26	25571253	25571278
chr2_4461	p2	(GA)10	20	25574511	25574530
chr2_4462	c	tgttggggttacaggcagtttgtaggtcagctccctgctgacctacaaacac	120	25577240	25577359
chr2_4463	c	(GT)6ttt(TG)7	29	25578566	25578594
chr2_4464	p2	(TG)13	26	25579921	25579946
chr2_4465	p2	(GA)8	16	25581676	25581691
chr2_4466	p2	(TG)15	30	25584374	25584403
chr2_4467	p2	(AC)7	14	25584576	25584589
chr2_4468	p2	(TC)7	14	25586614	25586627
chr2_4469	p2	(CT)6	12	25586833	25586844
chr2_4470	c	(TC)12act(CA)6	39	25588825	25588863
chr2_4471	p2	(AC)9	18	25592973	25592990
chr2_4472	p2	(AC)16	32	25593648	25593679
chr2_4473	p2	(AT)6	12	25618519	25618530
chr2_4474	p2	(CA)26	52	25621878	25621929
chr2_4475	p2	(CA)13	26	25623829	25623854
chr2_4476	p2	(AC)16	32	25631668	25631699
chr2_4477	p2	(AG)7	14	25631877	25631890
chr2_4478	p2	(AC)8	16	25632051	25632066
chr2_4479	p2	(AC)10	20	25637888	25637907
chr2_4480	p2	(TG)14	28	25638047	25638074
chr2_4481	p2	(GT)34	68	25638995	25639062
chr2_4482	p2	(CA)6	12	25653578	25653589
chr2_4483	p2	(TG)9	18	25656945	25656962
chr2_4484	p2	(GT)6	12	25657662	25657673
chr2_4485	p2	(CA)22	44	25659192	25659235
chr2_4486	p2	(CA)15	30	25660601	25660630
chr2_4487	p2	(GT)12	24	25661353	25661376
chr2_4488	p3	(AAT)5	15	25664405	25664419
chr2_4489	p3	(CTG)5	15	25667764	25667778
chr2_4490	p2	(GT)11	22	25677370	25677391
chr2_4491	p2	(TG)7	14	25678580	25678593
chr2_4492	p2	(GT)7	14	25680157	25680170
chr2_4493	p2	(AC)8	16	25680321	25680336
chr2_4494	p2	(AC)14	28	25681865	25681892
chr2_4495	p2	(CA)20	40	25685643	25685682
chr2_4496	c	(CA)14ctatggattctcacttaaaaaaaaaaaaaatcagtgt(AC)7	79	25690590	25690668
chr2_4497	p2	(AG)6	12	25691357	25691368
chr2_4498	p2	(TG)8	16	25695446	25695461
chr2_4499	p3	(AAC)6	18	25697225	25697242
chr2_4500	p2	(GT)9	18	25704098	25704115
chr2_4501	p2	(GT)10	20	25706696	25706715
chr2_4502	p2	(TG)10	20	25708761	25708780
chr2_4503	c	acatacaagcacgcttttaaattttggtgggtctttacccctcatccttcatcac	132	25712840	25712971
chr2_4504	p2	(AT)10	20	25713229	25713248
chr2_4505	p2	(AC)6	12	25713719	25713730
chr2_4506	p3	(ATC)6	18	25715269	25715286
chr2_4507	p2	(TC)9	18	25719034	25719051
chr2_4508	p2	(TG)7	14	25722801	25722814
chr2_4509	p2	(AC)6	12	25724946	25724957
chr2_4510	p2	(CA)7	14	25725836	25725849

chr2_4511	p2	(GT)12	24	25727835	25727858
chr2_4512	p2	(CA)10	20	25730145	25730164
chr2_4513	p3	(GAG)6	18	25731597	25731614
chr2_4514	p2	(AC)6	12	25732502	25732513
chr2_4515	p2	(GT)9	18	25735307	25735324
chr2_4516	p2	(TG)9	18	25738043	25738060
chr2_4517	p2	(GT)8	16	25738217	25738232
chr2_4518	p2	(AC)9	18	25738472	25738489
chr2_4519	p2	(TA)10	20	25741076	25741095
chr2_4520	c	(AC)8(AT)7acat(AC)7	48	25742474	25742521
chr2_4521	p2	(AC)9	18	25743576	25743593
chr2_4522	p2	(GT)19	38	25744740	25744777
chr2_4523	c	cccccaccgctaatgagctatttacacagaggactgtttgtgcacatccatag	139	25745776	25745914
chr2_4524	p2	(CA)12	24	25747554	25747577
chr2_4525	p2	(GA)7	14	25749697	25749710
chr2_4526	p2	(AC)22	44	25750967	25751010
chr2_4527	p2	(AC)7	14	25754917	25754930
chr2_4528	p3	(TAT)6	18	25758881	25758898
chr2_4529	p3	(ATT)5	15	25762453	25762467
chr2_4530	p2	(TG)6	12	25763035	25763046
chr2_4531	p2	(TG)6	12	25766777	25766788
chr2_4532	c	gagcaaggatgagggtacagtatgtgaaaggacagcacacgcacacgca	114	25767788	25767901
chr2_4533	c	gactcggcctaacccaactgcaaaaccagacactgctgcgacaatgtcacg	113	25770972	25771084
chr2_4534	p2	(TC)12	24	25771683	25771706
chr2_4535	p3	(ATT)8	24	25772199	25772222
chr2_4536	p2	(TG)16	32	25774640	25774671
chr2_4537	p3	(TAT)5	15	25776064	25776078
chr2_4538	p4	(ATAA)5	20	25776556	25776575
chr2_4539	p2	(TG)6	12	25778080	25778091
chr2_4540	p3	(TTA)5	15	25778324	25778338
chr2_4541	p2	(AG)6	12	25778632	25778643
chr2_4542	p2	(TG)10	20	25781039	25781058
chr2_4543	p2	(TG)6	12	25781195	25781206
chr2_4544	c	(TC)16(AC)8	48	25781464	25781511
chr2_4545	p2	(GT)6	12	25781851	25781862
chr2_4546	c	(AC)6g(CA)6	25	25788741	25788765
chr2_4547	p2	(TG)18	36	25790916	25790951
chr2_4548	p2	(AC)15	30	25792622	25792651
chr2_4549	p2	(GA)9	18	25792881	25792898
chr2_4550	p3	(CAC)5	15	25793316	25793330
chr2_4551	p2	(CT)6	12	25794327	25794338
chr2_4552	p2	(CA)7	14	25795689	25795702
chr2_4553	p2	(TG)7	14	25800998	25801011
chr2_4554	c	(AG)8actagcagggaagctgattaaaaaaatgagaa(AG)7	62	25803596	25803657
chr2_4555	p4	(AGAT)6	24	25805494	25805517
chr2_4556	p2	(GT)6	12	25807138	25807149
chr2_4557	p2	(TG)11	22	25809025	25809046
chr2_4558	p2	(GT)12	24	25831944	25831967
chr2_4559	p2	(GA)19	38	25832083	25832120
chr2_4560	p2	(AC)6	12	25832232	25832243
chr2_4561	p2	(CA)6	12	25835688	25835699
chr2_4562	p4	(TTTG)5	20	25838244	25838263

chr2_4563	p2	(TC)10	20	25843451	25843470
chr2_4564	p2	(TG)6	12	25845802	25845813
chr2_4565	p2	(TG)9	18	25846903	25846920
chr2_4566	p2	(CA)11	22	25855297	25855318
chr2_4567	p2	(TG)14	28	25856158	25856185
chr2_4568	p2	(AG)6	12	25858242	25858253
chr2_4569	p2	(TC)11	22	25860021	25860042
chr2_4570	p2	(CA)7	14	25865869	25865882
chr2_4571	p2	(GT)7	14	25867545	25867558
chr2_4572	p2	(GT)19	38	25868032	25868069
chr2_4573	p4	(ATAC)6	24	25868885	25868908
chr2_4574	p2	(TA)7	14	25870292	25870305
chr2_4575	p2	(CA)6	12	25872178	25872189
chr2_4576	p2	(CA)10	20	25872515	25872534
chr2_4577	p2	(CA)19	38	25878379	25878416
chr2_4578	p2	(AC)8	16	25888341	25888356
chr2_4579	p2	(GA)6	12	25890119	25890130
chr2_4580	p2	(TA)6	12	25896724	25896735
chr2_4581	p2	(AC)15	30	25905006	25905035
chr2_4582	p2	(AC)14	28	25905625	25905652
chr2_4583	p2	(AG)6	12	25906588	25906599
chr2_4584	p2	(CT)13	26	25910269	25910294
chr2_4585	p2	(GT)18	36	25916585	25916620
chr2_4586	c	(TC)8(AC)11	38	25921402	25921439
chr2_4587	p3	(TTG)5	15	25923612	25923626
chr2_4588	p3	(CCT)5	15	25925363	25925377
chr2_4589	p2	(CA)20	40	25926936	25926975
chr2_4590	p2	(CA)18	36	25931482	25931517
chr2_4591	p2	(TG)6	12	25934993	25935004
chr2_4592	p2	(CA)6	12	25936197	25936208
chr2_4593	p4	(CCAT)7	28	25937313	25937340
chr2_4594	p4	(CCTA)6	24	25945001	25945024
chr2_4595	p2	(AC)18	36	25946327	25946362
chr2_4596	p3	(GAG)6	18	25951054	25951071
chr2_4597	p3	(TGC)5	15	25952735	25952749
chr2_4598	p3	(TAT)12	36	25953082	25953117
chr2_4599	p5	(TTGAT)5	25	25955141	25955165
chr2_4600	p2	(TG)8	16	25959254	25959269
chr2_4601	p2	(TG)6	12	25960855	25960866
chr2_4602	p2	(AC)8	16	25961805	25961820
chr2_4603	p2	(CT)7	14	25963187	25963200
chr2_4604	p2	(AC)8	16	25963599	25963614
chr2_4605	p2	(CA)18	36	25968362	25968397
chr2_4606	p2	(CA)12	24	25971146	25971169
chr2_4607	p2	(AG)7	14	25975649	25975662
chr2_4608	p2	(AC)6	12	25988384	25988395
chr2_4609	p2	(AC)12	24	25989393	25989416
chr2_4610	p2	(AC)10	20	25991559	25991578
chr2_4611	p2	(AG)10	20	25993583	25993602
chr2_4612	p2	(CT)9	18	25996102	25996119
chr2_4613	c	(GT)13(GA)24	74	25996270	25996343
chr2_4614	p2	(TG)7	14	25997397	25997410

chr2_4615	p5	(TGTTT)5	25	26010870	26010894
chr2_4616	p5	(TCATG)5	25	26020108	26020132
chr2_4617	p3	(ATA)5	15	26027341	26027355
chr2_4618	p2	(TG)18	36	26034283	26034318
chr2_4619	p2	(TG)8	16	26035357	26035372
chr2_4620	p2	(CA)6	12	26038154	26038165
chr2_4621	c	ttctctctatctctgtatgcccctc(CA)6ctttgcctcccctgctctcatctc	108	26040944	26041051
chr2_4622	p2	(TC)21	42	26043854	26043895
chr2_4623	p2	(CA)7	14	26051565	26051578
chr2_4624	p5	(AAATC)5	25	26069990	26070014
chr2_4625	p2	(AC)19	38	26078035	26078072
chr2_4626	p2	(TA)7	14	26085191	26085204
chr2_4627	p3	(AAT)5	15	26093743	26093757
chr2_4628	p3	(GAT)6	18	26096546	26096563
chr2_4629	p4	(AAAC)5	20	26109945	26109964
chr2_4630	c	(TG)6taggagaga(GT)6	33	26116074	26116106
chr2_4631	p2	(CA)21	42	26116719	26116760
chr2_4632	p2	(AC)10	20	26120210	26120229
chr2_4633	c	gtccttcaggtgttttcgctggctgtcacacggccatctttcagattaattaaa	135	26122914	26123048
chr2_4634	p2	(AC)8	16	26126548	26126563
chr2_4635	p2	(TG)12	24	26128134	26128157
chr2_4636	p2	(AC)15	30	26129541	26129570
chr2_4637	p2	(CA)27	54	26133921	26133974
chr2_4638	p2	(AG)7	14	26134328	26134341
chr2_4639	p2	(AG)6	12	26138385	26138396
chr2_4640	p2	(CA)6	12	26140400	26140411
chr2_4641	p2	(GT)6	12	26146170	26146181
chr2_4642	p2	(GT)13	26	26146317	26146342
chr2_4643	p2	(TG)20	40	26146474	26146513
chr2_4644	p2	(TA)9	18	26146678	26146695
chr2_4645	p4	(CTGT)7	28	26150818	26150845
chr2_4646	p3	(CTT)5	15	26160057	26160071
chr2_4647	p2	(CT)19	38	26160995	26161032
chr2_4648	p2	(TG)6	12	26162750	26162761
chr2_4649	p2	(TA)7	14	26165063	26165076
chr2_4650	p3	(ATC)5	15	26174169	26174183
chr2_4651	p2	(GT)11	22	26187055	26187076
chr2_4652	p2	(GT)11	22	26189345	26189366
chr2_4653	p2	(CA)26	52	26192225	26192276
chr2_4654	p2	(AG)9	18	26192595	26192612
chr2_4655	p2	(GT)6	12	26193889	26193900
chr2_4656	p2	(AC)6	12	26196019	26196030
chr2_4657	p2	(AG)13	26	26196232	26196257
chr2_4658	p4	(AAAT)5	20	26200509	26200528
chr2_4659	p4	(CTAC)6	24	26203083	26203106
chr2_4660	p2	(GT)7	14	26204044	26204057
chr2_4661	c	(CA)9a(AC)18c(CA)6	68	26204574	26204641
chr2_4662	p2	(CA)13	26	26205000	26205025
chr2_4663	p2	(CA)17	34	26207344	26207377
chr2_4664	p3	(ATT)5	15	26214146	26214160
chr2_4665	p2	(GT)16	32	26216415	26216446
chr2_4666	p4	(ATCC)6	24	26217084	26217107

chr2_4667	p3	(AGT)6	18	26217687	26217704
chr2_4668	p2	(TG)13	26	26226234	26226259
chr2_4669	p2	(AC)19	38	26228681	26228718
chr2_4670	p2	(CA)10	20	26230722	26230741
chr2_4671	p2	(CA)17	34	26232898	26232931
chr2_4672	p2	(AC)17	34	26234109	26234142
chr2_4673	p2	(CT)10	20	26237482	26237501
chr2_4674	c	.cacataaggttttgttgagcttgagacattaaaaaaaaacacagagccaaa	109	26242064	26242172
chr2_4675	p2	(AC)7	14	26251048	26251061
chr2_4676	p3	(TGA)5	15	26255794	26255808
chr2_4677	p3	(ACA)8	24	26256590	26256613
chr2_4678	p3	(CAG)5	15	26264427	26264441
chr2_4679	p2	(TA)6	12	26269538	26269549
chr2_4680	p2	(AC)6	12	26269876	26269887
chr2_4681	p2	(GT)13	26	26344931	26344956
chr2_4682	p2	(AG)6	12	26355396	26355407
chr2_4683	p3	(CAT)5	15	26369677	26369691
chr2_4684	p2	(AT)6	12	26371038	26371049
chr2_4685	p3	(CTG)5	15	26374585	26374599
chr2_4686	p3	(TTG)6	18	26402329	26402346
chr2_4687	p2	(AT)6	12	26404073	26404084
chr2_4688	p2	(CT)7	14	26406285	26406298
chr2_4689	p2	(TG)6	12	26413641	26413652
chr2_4690	p2	(GT)13	26	26416913	26416938
chr2_4691	p2	(GT)15	30	26417593	26417622
chr2_4692	p3	(GAT)5	15	26428498	26428512
chr2_4693	p4	(CATT)6	24	26441415	26441438
chr2_4694	p2	(CA)17	34	26473226	26473259
chr2_4695	p2	(GT)6	12	26518751	26518762
chr2_4696	p2	(TC)6	12	26521969	26521980
chr2_4697	p3	(TGT)9	27	26522230	26522256
chr2_4698	p2	(TG)6	12	26530794	26530805
chr2_4699	p3	(AAC)5	15	26538170	26538184
chr2_4700	c	(GT)16(GA)10	52	26543914	26543965
chr2_4701	p3	(TGT)5	15	26546959	26546973
chr2_4702	p2	(CA)24	48	26548666	26548713
chr2_4703	p2	(TG)12	24	26549436	26549459
chr2_4704	p2	(TG)7	14	26550611	26550624
chr2_4705	p2	(TC)8	16	26553561	26553576
chr2_4706	c	(ATGT)5atgcaagttcttgctcaca(GT)22	83	26569003	26569085
chr2_4707	p2	(GT)16	32	26573885	26573916
chr2_4708	p2	(TG)6	12	26581534	26581545
chr2_4709	p2	(CA)11	22	26581822	26581843
chr2_4710	p2	(AG)9	18	26585431	26585448
chr2_4711	p2	(GT)15	30	26587570	26587599
chr2_4712	p2	(TC)8	16	26587896	26587911
chr2_4713	p2	(AC)8	16	26591757	26591772
chr2_4714	p3	(TAT)11	33	26594711	26594743
chr2_4715	p2	(GT)24	48	26597444	26597491
chr2_4716	p3	(TAT)6	18	26599561	26599578
chr2_4717	p3	(ATT)5	15	26600142	26600156
chr2_4718	p2	(AC)6	12	26600431	26600442

chr2_4719	p2	(GA)6	12	26629725	26629736
chr2_4720	p3	(GAT)7	21	26636489	26636509
chr2_4721	c	(AC)6ta(AC)7	28	26638889	26638916
chr2_4722	p2	(AC)6	12	26645708	26645719
chr2_4723	p2	(GT)13	26	26647838	26647863
chr2_4724	p2	(TG)21	42	26649992	26650033
chr2_4725	p2	(TG)8	16	26655069	26655084
chr2_4726	p2	(CT)6	12	26656008	26656019
chr2_4727	p2	(TC)9	18	26656329	26656346
chr2_4728	c	(TC)25(AC)14	78	26669121	26669198
chr2_4729	p2	(AC)6	12	26670955	26670966
chr2_4730	c	(TG)6(AG)6	24	26671126	26671149
chr2_4731	p2	(GT)6	12	26673238	26673249
chr2_4732	p2	(TG)8	16	26673980	26673995
chr2_4733	p3	(CAG)5	15	26678210	26678224
chr2_4734	p2	(GA)6	12	26682588	26682599
chr2_4735	p2	(GT)13	26	26683706	26683731
chr2_4736	p2	(AG)6	12	26684160	26684171
chr2_4737	p2	(TG)21	42	26685728	26685769
chr2_4738	p2	(CT)7	14	26686069	26686082
chr2_4739	p2	(TG)13	26	26686493	26686518
chr2_4740	p2	(TG)14	28	26689126	26689153
chr2_4741	p2	(AG)6	12	26693645	26693656
chr2_4742	c	(GT)17(GA)6	46	26694635	26694680
chr2_4743	p2	(GT)19	38	26703352	26703389
chr2_4744	p2	(GT)6	12	26706973	26706984
chr2_4745	p2	(GT)6	12	26707302	26707313
chr2_4746	p4	(TATG)5	20	26715582	26715601
chr2_4747	p2	(GT)6	12	26716374	26716385
chr2_4748	p2	(TG)13	26	26716684	26716709
chr2_4749	p2	(TG)12	24	26723429	26723452
chr2_4750	c	(TG)6(AG)6	24	26728772	26728795
chr2_4751	p2	(AC)8	16	26731866	26731881
chr2_4752	p2	(TG)19	38	26732586	26732623
chr2_4753	p2	(TC)11	22	26733673	26733694
chr2_4754	p2	(GA)7	14	26735169	26735182
chr2_4755	p3	(GCT)5	15	26737372	26737386
chr2_4756	p2	(TG)20	40	26737882	26737921
chr2_4757	p2	(AC)6	12	26740586	26740597
chr2_4758	p2	(GT)10	20	26754005	26754024
chr2_4759	p2	(TA)10	20	26758328	26758347
chr2_4760	p3	(GGA)8	24	26762796	26762819
chr2_4761	p2	(AT)7	14	26772351	26772364
chr2_4762	p2	(GT)9	18	26796655	26796672
chr2_4763	p2	(GT)10	20	26812141	26812160
chr2_4764	p4	(GACA)10	40	26812967	26813006
chr2_4765	p2	(TG)15	30	26817216	26817245
chr2_4766	p2	(CA)8	16	26817531	26817546
chr2_4767	p4	(GTCT)6	24	26823283	26823306
chr2_4768	p2	(TA)7	14	26829287	26829300
chr2_4769	p4	(TCCA)6	24	26830807	26830830
chr2_4770	p2	(AC)7	14	26836839	26836852

chr2_4771	p3	(TTA)6	18	26849345	26849362
chr2_4772	c	(TC)10acacacacacg(CA)14	59	26853481	26853539
chr2_4773	p2	(AC)6	12	26856711	26856722
chr2_4774	p2	(GA)7	14	26864650	26864663
chr2_4775	p2	(TC)6	12	26865920	26865931
chr2_4776	p2	(AC)14	28	26870343	26870370
chr2_4777	p2	(TG)6	12	26870940	26870951
chr2_4778	p4	(CAAA)5	20	26872179	26872198
chr2_4779	p2	(CA)9	18	26879521	26879538
chr2_4780	p3	(AAC)5	15	26883648	26883662
chr2_4781	p2	(TC)6	12	26885961	26885972
chr2_4782	p2	(AC)8	16	26907799	26907814
chr2_4783	p3	(TTG)6	18	26915696	26915713
chr2_4784	p3	(AGC)7	21	26924934	26924954
chr2_4785	p2	(TG)13	26	26932685	26932710
chr2_4786	p2	(AG)7	14	26935710	26935723
chr2_4787	p2	(CA)12	24	26943346	26943369
chr2_4788	c	(CTG)5ctaacttcacacgcatgaaccaaaaaggcacaaat(AC)6	62	26952180	26952241
chr2_4789	p5	(AGGAC)8	40	26979289	26979328
chr2_4790	p4	(TGGA)8	32	26979725	26979756
chr2_4791	p2	(CT)7	14	26981114	26981127
chr2_4792	p2	(TG)6	12	27003483	27003494
chr2_4793	p2	(GT)7	14	27011843	27011856
chr2_4794	p2	(GT)8	16	27014213	27014228
chr2_4795	p3	(TCT)8	24	27023878	27023901
chr2_4796	p2	(TA)6	12	27031367	27031378
chr2_4797	p2	(AT)7	14	27034623	27034636
chr2_4798	p2	(TG)8	16	27038665	27038680
chr2_4799	p2	(CA)6	12	27046408	27046419
chr2_4800	p2	(TA)6	12	27066306	27066317
chr2_4801	p2	(AG)6	12	27082604	27082615
chr2_4802	p2	(TG)6	12	27083498	27083509
chr2_4803	p2	(GT)6	12	27102323	27102334
chr2_4804	p2	(TG)9	18	27109245	27109262
chr2_4805	p2	(TC)6	12	27115108	27115119
chr2_4806	p2	(GT)17	34	27118875	27118908
chr2_4807	p2	(AC)8	16	27121207	27121222
chr2_4808	c	(GT)6ttgtgat(AG)9	37	27121548	27121584
chr2_4809	p3	(TTC)7	21	27123560	27123580
chr2_4810	p3	(ATG)5	15	27128568	27128582
chr2_4811	p2	(GT)19	38	27135566	27135603
chr2_4812	p3	(CTC)6	18	27137864	27137881
chr2_4813	p3	(CCT)9	27	27138065	27138091
chr2_4814	p3	(GCT)5	15	27161183	27161197
chr2_4815	p4	(CATC)8	32	27175519	27175550
chr2_4816	p2	(AT)6	12	27181052	27181063
chr2_4817	p2	(AG)23	46	27181521	27181566
chr2_4818	c	(GC)6(AC)22	56	27183816	27183871
chr2_4819	p3	(CAG)5	15	27187902	27187916
chr2_4820	p2	(CA)6	12	27189940	27189951
chr2_4821	p2	(GT)17	34	27199366	27199399
chr2_4822	p3	(GCT)6	18	27200595	27200612

chr2_4823	p3	(ACA)5	15	27208141	27208155
chr2_4824	p2	(GT)6	12	27210122	27210133
chr2_4825	p2	(AC)7	14	27215274	27215287
chr2_4826	p2	(CA)6	12	27225740	27225751
chr2_4827	c	ctcaccctcctcctcctctacaaagcggatctactttgatctccaaacacgtg	139	27226374	27226512
chr2_4828	p3	(TGT)6	18	27235817	27235834
chr2_4829	p2	(TG)13	26	27238131	27238156
chr2_4830	p3	(ATA)5	15	27258759	27258773
chr2_4831	p2	(CA)6	12	27274288	27274299
chr2_4832	c	(CA)9ctcact(CA)8	40	27275379	27275418
chr2_4833	p2	(AC)20	40	27278585	27278624
chr2_4834	p2	(CA)8	16	27287423	27287438
chr2_4835	p4	(ATTT)5	20	27288068	27288087
chr2_4836	p2	(CT)16	32	27289749	27289780
chr2_4837	p2	(GT)6	12	27312220	27312231
chr2_4838	p3	(TCT)5	15	27312746	27312760
chr2_4839	c	(TC)12tgt(CA)6	39	27323588	27323626
chr2_4840	p5	(AAATA)5	25	27325550	27325574
chr2_4841	p4	(AGAT)49	196	27332108	27332303
chr2_4842	p2	(GA)6	12	27332495	27332506
chr2_4843	p2	(AC)21	42	27343638	27343679
chr2_4844	p2	(CA)13	26	27349750	27349775
chr2_4845	p3	(TTA)5	15	27350823	27350837
chr2_4846	p2	(CA)20	40	27356013	27356052
chr2_4847	p3	(TCC)12	36	27358058	27358093
chr2_4848	p2	(AC)7	14	27359298	27359311
chr2_4849	p2	(CA)12	24	27360901	27360924
chr2_4850	p4	(CATC)5	20	27363309	27363328
chr2_4851	p2	(TG)6	12	27373193	27373204
chr2_4852	p3	(TAA)6	18	27381107	27381124
chr2_4853	p2	(AC)14	28	27382192	27382219
chr2_4854	p2	(CA)16	32	27390985	27391016
chr2_4855	p2	(TG)14	28	27391132	27391159
chr2_4856	c	(TC)6atacacag(AC)8	36	27395308	27395343
chr2_4857	p2	(GT)7	14	27399707	27399720
chr2_4858	c	ıtatat(AC)7atatacatatacacatatat(AC)9atatacacatatat(A(	180	27422783	27422962
chr2_4859	p2	(AC)18	36	27447720	27447755
chr2_4860	p2	(CT)7	14	27457722	27457735
chr2_4861	p2	(TA)7	14	27473386	27473399
chr2_4862	p3	(AAG)6	18	27481352	27481369
chr2_4863	p3	(AGC)5	15	27486967	27486981
chr2_4864	p4	(TGTT)7	28	27495816	27495843
chr2_4865	p2	(AT)8	16	27496248	27496263
chr2_4866	p3	(AAG)5	15	27534096	27534110
chr2_4867	p2	(TA)9	18	27574085	27574102
chr2_4868	c	(AC)10aatagtactaac(CA)6	44	27617907	27617950
chr2_4869	p2	(CA)9	18	27629090	27629107
chr2_4870	c	(AC)8atacagctggtgtcactttgtatacagtgtgcatc(TG)9	69	27642933	27643001
chr2_4871	p2	(AG)12	24	27644998	27645021
chr2_4872	p2	(AG)15	30	27645128	27645157
chr2_4873	p2	(TG)8	16	27651127	27651142
chr2_4874	p2	(CA)30	60	27655323	27655382

chr2_4875	p2	(GT)22	44	27657221	27657264
chr2_4876	p2	(AC)25	50	27660316	27660365
chr2_4877	p2	(TG)6	12	27663563	27663574
chr2_4878	p3	(CTG)5	15	27672114	27672128
chr2_4879	p2	(CG)7	14	27691797	27691810
chr2_4880	p4	(ATGG)5	20	27708361	27708380
chr2_4881	p2	(TG)8	16	27717903	27717918
chr2_4882	p2	(AC)8	16	27730433	27730448
chr2_4883	c	tcagctccctacctgctcccctctttctattaccttcctgcatatctaaacacaa	92	27734082	27734173
chr2_4884	p2	(CA)20	40	27744146	27744185
chr2_4885	p2	(GT)8	16	27744861	27744876
chr2_4886	c	cagggtgttactgccttgtggtttgggca(TG)8accacacgaatcaatag	204	27754254	27754457
chr2_4887	p2	(CA)7	14	27755359	27755372
chr2_4888	p4	(ATTT)5	20	27756064	27756083
chr2_4889	p2	(GT)16	32	27756930	27756961
chr2_4890	p2	(TG)9	18	27757746	27757763
chr2_4891	p2	(TG)6	12	27758396	27758407
chr2_4892	p2	(TG)23	46	27759480	27759525
chr2_4893	c	(TG)6c(GT)13	39	27769258	27769296
chr2_4894	p2	(AC)8	16	27771867	27771882
chr2_4895	p2	(GT)12	24	27781364	27781387
chr2_4896	p2	(CT)7	14	27781609	27781622
chr2_4897	c	cacgcacacaa(TG)7ttctgaattaaggattttgtcatactggatcatttcc	148	27783374	27783521
chr2_4898	p2	(TG)9	18	27783628	27783645
chr2_4899	p2	(TG)14	28	27783774	27783801
chr2_4900	c	(TG)17aatgaaacaggaaaaagtgaaatgtgct(TG)6	74	27787903	27787976
chr2_4901	p2	(CA)14	28	27789006	27789033
chr2_4902	p2	(AG)6	12	27789469	27789480
chr2_4903	p2	(TC)19	38	27792156	27792193
chr2_4904	p2	(CA)15	30	27794221	27794250
chr2_4905	c	(AC)6gcacg(CA)7	31	27794512	27794542
chr2_4906	p3	(TCC)5	15	27794989	27795003
chr2_4907	p2	(AC)8	16	27797925	27797940
chr2_4908	p2	(TG)10	20	27800128	27800147
chr2_4909	p2	(CA)6	12	27800310	27800321
chr2_4910	p2	(GA)9	18	27801232	27801249
chr2_4911	p2	(CT)6	12	27809460	27809471
chr2_4912	c	(TG)7tc(TG)6	28	27810809	27810836
chr2_4913	p2	(TG)6	12	27811034	27811045
chr2_4914	p2	(TC)15	30	27811346	27811375
chr2_4915	p2	(TG)7	14	27814134	27814147
chr2_4916	p2	(TA)8	16	27815236	27815251
chr2_4917	p3	(TTA)7	21	27815540	27815560
chr2_4918	p2	(GT)8	16	27819438	27819453
chr2_4919	p2	(TG)7	14	27819615	27819628
chr2_4920	p2	(AC)20	40	27819822	27819861
chr2_4921	p2	(CA)22	44	27820945	27820988
chr2_4922	p2	(TG)6	12	27822799	27822810
chr2_4923	p2	(TG)6	12	27825171	27825182
chr2_4924	p2	(CA)13	26	27829366	27829391
chr2_4925	c	aatccctagatacaagatgtggctgctgagctcaccaaactgcaacagaat	109	27829607	27829715
chr2_4926	p2	(GA)6	12	27831798	27831809

chr2_4927	p2	(TG)11	22	27831976	27831997
chr2_4928	p2	(CA)19	38	27832319	27832356
chr2_4929	p5	(CAAAT)6	30	27833174	27833203
chr2_4930	p2	(GT)7	14	27837355	27837368
chr2_4931	p2	(GT)12	24	27839058	27839081
chr2_4932	p2	(CA)6	12	27839631	27839642
chr2_4933	p4	(TCTA)271	1084	27840568	27841651
chr2_4934	c	(CT)7aattctgttttacacaccgtgc(CT)6	48	27845077	27845124
chr2_4935	c	aaattcagacacacattaagcatgttagccagctttaatcacgcagcatggc	84	27845759	27845842
chr2_4936	p2	(CA)6	12	27849437	27849448
chr2_4937	p2	(GT)8	16	27849849	27849864
chr2_4938	c	tgactctgcatttgttgtattcatttatttatttttatttttgcacttgtggccaagaa	116	27851158	27851273
chr2_4939	p2	(CA)10	20	27853688	27853707
chr2_4940	p2	(CA)6	12	27858062	27858073
chr2_4941	p2	(TG)16	32	27859241	27859272
chr2_4942	p3	(TGA)5	15	27864373	27864387
chr2_4943	p2	(TG)8	16	27864593	27864608
chr2_4944	p2	(AG)10	20	27865069	27865088
chr2_4945	p4	(CCAT)5	20	27866196	27866215
chr2_4946	p2	(TG)12	24	27871455	27871478
chr2_4947	p3	(AAC)6	18	27874163	27874180
chr2_4948	p2	(GT)6	12	27875679	27875690
chr2_4949	p2	(GT)8	16	27875837	27875852
chr2_4950	p2	(TG)8	16	27876563	27876578
chr2_4951	p2	(AC)12	24	27877483	27877506
chr2_4952	p2	(TG)9	18	27883178	27883195
chr2_4953	p2	(TG)6	12	27883639	27883650
chr2_4954	p4	(TGGA)5	20	27892287	27892306
chr2_4955	p2	(AC)8	16	27892785	27892800
chr2_4956	p2	(GT)10	20	27903109	27903128
chr2_4957	p2	(AG)13	26	27908720	27908745
chr2_4958	p2	(TG)9	18	27909980	27909997
chr2_4959	p2	(CA)6	12	27911476	27911487
chr2_4960	p2	(AG)8	16	27916874	27916889
chr2_4961	p4	(TTAC)5	20	27929049	27929068
chr2_4962	p2	(CA)20	40	27932787	27932826
chr2_4963	p2	(AG)8	16	27933352	27933367
chr2_4964	p2	(GT)8	16	27933867	27933882
chr2_4965	p2	(AC)6	12	27934473	27934484
chr2_4966	p2	(GT)6	12	27939823	27939834
chr2_4967	c	(TC)11(AC)18	58	27939973	27940030
chr2_4968	p2	(TG)7	14	27941778	27941791
chr2_4969	p2	(AC)7	14	27942536	27942549
chr2_4970	c	aactcagacttggccctatacagggcaaacaacactgaaaggagctgcca	113	27944724	27944836
chr2_4971	p2	(AC)11	22	27947078	27947099
chr2_4972	p2	(TG)6	12	27948734	27948745
chr2_4973	p2	(AC)8	16	27948888	27948903
chr2_4974	p2	(TG)6	12	27949266	27949277
chr2_4975	p2	(CA)7	14	27950674	27950687
chr2_4976	c	(TG)7(AG)6	26	27951367	27951392
chr2_4977	p2	(TG)6	12	27954171	27954182
chr2_4978	c	tgttctgccctctgggtcagtgtgcccctatctgttctgcccatgtaagaaat	105	27955360	27955464

chr2_4979	p2	(CA)7	14	27955860	27955873
chr2_4980	p2	(CA)7	14	27957617	27957630
chr2_4981	p2	(TG)6	12	27957766	27957777
chr2_4982	p2	(AC)13	26	27957978	27958003
chr2_4983	p2	(TG)14	28	27958192	27958219
chr2_4984	p2	(GA)7	14	27959918	27959931
chr2_4985	c	(AC)10gcg(CA)14	51	27962409	27962459
chr2_4986	p2	(TG)18	36	27962635	27962670
chr2_4987	p2	(AG)7	14	27967604	27967617
chr2_4988	p3	(TCT)6	18	27969694	27969711
chr2_4989	p2	(TG)8	16	27970692	27970707
chr2_4990	p2	(GA)6	12	27974238	27974249
chr2_4991	p2	(CA)26	52	27975771	27975822
chr2_4992	c	cttgatgtgtttaatgggtctgtagtaaattgcggctaagtgccttcaaccaat	137	27978109	27978245
chr2_4993	c	(TG)6c(GT)11(GA)7	49	27980796	27980844
chr2_4994	c	(GT)6(GA)7	26	27981019	27981044
chr2_4995	c	gtgtatgcaggaacctacatgaacgcacatccaaacatttgacacacgaga	121	27981319	27981439
chr2_4996	c	(TGTT)5(TG)6	32	27982594	27982625
chr2_4997	c	ccccttggaatctcaatgtcgaataatcaagattgacactggcacacaatg	110	27984287	27984396
chr2_4998	p2	(GT)8	16	27990516	27990531
chr2_4999	p2	(CA)9	18	27990884	27990901
chr2_5000	p2	(TG)6	12	27996239	27996250
chr2_5001	p3	(TAA)5	15	28001406	28001420
chr2_5002	p2	(TC)6	12	28004033	28004044
chr2_5003	p2	(TG)8	16	28005526	28005541
chr2_5004	c	(CT)6t(TC)7	27	28007088	28007114
chr2_5005	p2	(TG)18	36	28013079	28013114
chr2_5006	p2	(GA)9	18	28014778	28014795
chr2_5007	p2	(GT)6	12	28017130	28017141
chr2_5008	p2	(CA)7	14	28017829	28017842
chr2_5009	p2	(CA)15	30	28020153	28020182
chr2_5010	p2	(TA)10	20	28025576	28025595
chr2_5011	p2	(CA)10	20	28035036	28035055
chr2_5012	p2	(CA)9	18	28036172	28036189
chr2_5013	p2	(TG)6	12	28038397	28038408
chr2_5014	p4	(ATGG)5	20	28039327	28039346
chr2_5015	c	(TG)12tccctctgtttcatttccctttgcgta(TG)16	83	28040437	28040519
chr2_5016	p2	(GT)10	20	28043137	28043156
chr2_5017	p2	(TG)13	26	28044013	28044038
chr2_5018	p2	(TG)8	16	28047149	28047164
chr2_5019	c	(AC)6(GT)9	30	28052086	28052115
chr2_5020	p2	(AT)9	18	28052902	28052919
chr2_5021	p2	(TA)8	16	28054343	28054358
chr2_5022	p2	(AC)8	16	28062081	28062096
chr2_5023	c	(TG)6c(GT)9(GA)17	65	28067835	28067899
chr2_5024	p2	(AC)6	12	28074649	28074660
chr2_5025	p4	(AATG)6	24	28075092	28075115
chr2_5026	p2	(AG)7	14	28075668	28075681
chr2_5027	p2	(AC)6	12	28076031	28076042
chr2_5028	p2	(TC)7	14	28077619	28077632
chr2_5029	p2	(AC)17	34	28078739	28078772
chr2_5030	c	(TG)12cgcgcacgcccatgcaagtggtgt(GA)17	82	28079614	28079695

chr2_5031	p4	(CATC)5	20	28085998	28086017
chr2_5032	p2	(TG)8	16	28087992	28088007
chr2_5033	p2	(CA)13	26	28088585	28088610
chr2_5034	p2	(GA)12	24	28089725	28089748
chr2_5035	p2	(GT)7	14	28090464	28090477
chr2_5036	p2	(AC)6	12	28093715	28093726
chr2_5037	p2	(GT)7	14	28096912	28096925
chr2_5038	p2	(GA)9	18	28105018	28105035
chr2_5039	p2	(GA)6	12	28106449	28106460
chr2_5040	p2	(GT)6	12	28107784	28107795
chr2_5041	p2	(TG)8	16	28111047	28111062
chr2_5042	p2	(TC)7	14	28111286	28111299
chr2_5043	p2	(AC)11	22	28113742	28113763
chr2_5044	p2	(AG)6	12	28114560	28114571
chr2_5045	p2	(GA)6	12	28115947	28115958
chr2_5046	p3	(TTG)5	15	28117285	28117299
chr2_5047	p2	(TG)6	12	28124824	28124835
chr2_5048	p2	(TA)12	24	28134734	28134757
chr2_5049	p2	(TG)8	16	28145169	28145184
chr2_5050	c	(TG)12(AG)12	48	28145607	28145654
chr2_5051	p2	(TG)14	28	28147878	28147905
chr2_5052	p2	(CA)6	12	28149461	28149472
chr2_5053	p2	(AC)6	12	28154649	28154660
chr2_5054	p2	(GT)8	16	28156181	28156196
chr2_5055	p2	(AC)24	48	28156403	28156450
chr2_5056	p2	(TG)9	18	28157052	28157069
chr2_5057	p2	(GT)15	30	28159451	28159480
chr2_5058	p2	(TG)16	32	28167405	28167436
chr2_5059	p2	(CT)6	12	28179507	28179518
chr2_5060	c	tgaatgttagaaagatagggagaaaaggaggaagagtttgtgtgtacttata	134	28179699	28179832
chr2_5061	p2	(GA)6	12	28182283	28182294
chr2_5062	p2	(CA)8	16	28186376	28186391
chr2_5063	p2	(TG)6	12	28188200	28188211
chr2_5064	c	(TC)8ttg(CT)6	31	28188388	28188418
chr2_5065	p2	(GT)10	20	28196420	28196439
chr2_5066	c	:acatataagatggttcctactgtaaagcacacatactgtacacacaaaatga	117	28199000	28199116
chr2_5067	c	(AC)15attacactcacatgcaagtggacactacactacacaa(AC)18	103	28203822	28203924
chr2_5068	p2	(TG)8	16	28204050	28204065
chr2_5069	p2	(CA)18	36	28205730	28205765
chr2_5070	p2	(CA)6	12	28207150	28207161
chr2_5071	p2	(TG)31	62	28207362	28207423
chr2_5072	p2	(GA)7	14	28208423	28208436
chr2_5073	p2	(TG)31	62	28220866	28220927
chr2_5074	p2	(AC)8	16	28225252	28225267
chr2_5075	p2	(AC)7	14	28228033	28228046
chr2_5076	p2	(TG)6	12	28229167	28229178
chr2_5077	p2	(CA)6	12	28233083	28233094
chr2_5078	p2	(AC)6	12	28233382	28233393
chr2_5079	p2	(TG)22	44	28235484	28235527
chr2_5080	p2	(GT)6	12	28235796	28235807
chr2_5081	p2	(CA)6	12	28238124	28238135
chr2_5082	p2	(GT)38	76	28238277	28238352

chr2_5083	p2	(GT)28	56	28244304	28244359
chr2_5084	p2	(TG)10	20	28248928	28248947
chr2_5085	p2	(AC)15	30	28253570	28253599
chr2_5086	p2	(AG)10	20	28255567	28255586
chr2_5087	c	(TG)14(AG)7	42	28255913	28255954
chr2_5088	p2	(GT)15	30	28257711	28257740
chr2_5089	p5	(TTTTC)5	25	28257916	28257940
chr2_5090	p2	(AG)15	30	28262708	28262737
chr2_5091	p3	(GAT)6	18	28265633	28265650
chr2_5092	p2	(CT)6	12	28266278	28266289
chr2_5093	p2	(TG)12	24	28270804	28270827
chr2_5094	p2	(AG)8	16	28316169	28316184
chr2_5095	p2	(CA)6	12	28348805	28348816
chr2_5096	p2	(GT)10	20	28355991	28356010
chr2_5097	p2	(CA)9	18	28372141	28372158
chr2_5098	p2	(AT)8	16	28374871	28374886
chr2_5099	p3	(ATT)5	15	28396140	28396154
chr2_5100	p2	(AT)6	12	28411637	28411648
chr2_5101	p3	(TTA)9	27	28416948	28416974
chr2_5102	c	(TG)19(CG)7	52	28435198	28435249
chr2_5103	c	gagttgaaacaagatcggccagtgtgtacttccatgagtggaactccctct	87	28443531	28443617
chr2_5104	p3	(AGC)5	15	28444357	28444371
chr2_5105	c	(TG)9tatgtat(GA)8	41	28447530	28447570
chr2_5106	c	(GT)15t(TG)11	53	28448534	28448586
chr2_5107	c	ctggattcacaggattgctctaaaaagccttcacactgaatgagggatgat	143	28448765	28448907
chr2_5108	p2	(GT)7	14	28455312	28455325
chr2_5109	p2	(GT)10	20	28459212	28459231
chr2_5110	p2	(TG)14	28	28459614	28459641
chr2_5111	p2	(TG)10	20	28460439	28460458
chr2_5112	c	(GT)6g(GT)30	73	28468321	28468393
chr2_5113	p2	(AC)6	12	28475742	28475753
chr2_5114	p2	(TG)11	22	28477114	28477135
chr2_5115	p2	(AC)9	18	28477361	28477378
chr2_5116	p2	(GT)17	34	28481851	28481884
chr2_5117	p2	(GA)6	12	28482237	28482248
chr2_5118	p2	(GT)17	34	28483438	28483471
chr2_5119	p2	(CA)11	22	28486593	28486614
chr2_5120	p3	(ATT)5	15	28523151	28523165
chr2_5121	p2	(AC)6	12	28527819	28527830
chr2_5122	p2	(GT)6	12	28534713	28534724
chr2_5123	c	nnnnnnnnnnnnnnnnnnnnnnnnnnnnngcgcacacacacac	80	28542114	28542193
chr2_5124	c	(GT)8gcaagatagagtaaaactgtgtgttgtcctgtgtgc(GT)7	66	28553250	28553315
chr2_5125	p3	(ATT)6	18	28571382	28571399
chr2_5126	c	cccacacacaaacagaaacatatattctgtcatttttcagtgtagttacatttc	114	28580691	28580804
chr2_5127	p2	(CA)11	22	28580984	28581005
chr2_5128	p2	(CA)6	12	28581348	28581359
chr2_5129	p2	(TG)6	12	28600392	28600403
chr2_5130	p5	(AAATC)5	25	28629989	28630013
chr2_5131	c	(TAT)5(TAC)5	30	28634063	28634092
chr2_5132	c	(TAT)5(TAC)6	33	28653352	28653384
chr2_5133	p5	(TGATG)5	25	28656254	28656278
chr2_5134	p5	(CCTCT)10	50	28665347	28665396

chr2_5135	p2	(AC)8	16	28682272	28682287
chr2_5136	p2	(GT)6	12	28682454	28682465
chr2_5137	p3	(TTG)5	15	28687637	28687651
chr2_5138	p2	(CA)10	20	28688092	28688111
chr2_5139	p2	(CA)6	12	28696182	28696193
chr2_5140	p2	(GT)13	26	28697181	28697206
chr2_5141	p2	(CT)15	30	28708955	28708984
chr2_5142	p2	(TA)10	20	28711632	28711651
chr2_5143	p2	(CA)6	12	28717128	28717139
chr2_5144	p2	(TG)11	22	28727729	28727750
chr2_5145	p2	(GT)7	14	28735525	28735538
chr2_5146	p2	(TG)6	12	28736064	28736075
chr2_5147	p2	(GT)7	14	28742828	28742841
chr2_5148	p2	(TG)6	12	28761083	28761094
chr2_5149	p2	(CA)8	16	28770654	28770669
chr2_5150	p2	(TG)18	36	28788882	28788917
chr2_5151	p2	(TC)6	12	28792810	28792821
chr2_5152	p4	(AAAT)6	24	28805750	28805773
chr2_5153	p2	(GT)8	16	28814091	28814106
chr2_5154	p2	(TC)6	12	28824798	28824809
chr2_5155	p2	(AC)14	28	28830882	28830909
chr2_5156	c	agatacatagagatgctgccatcagtgattaattactcccttgtaagcttttga	151	28831232	28831382
chr2_5157	p2	(TC)24	48	28841213	28841260
chr2_5158	p3	(TTG)6	18	28841736	28841753
chr2_5159	p2	(CA)6	12	28842321	28842332
chr2_5160	p3	(TTA)8	24	28845596	28845619
chr2_5161	p2	(CA)13	26	28846648	28846673
chr2_5162	p2	(CA)17	34	28858618	28858651
chr2_5163	p2	(GT)8	16	28863219	28863234
chr2_5164	p2	(TG)10	20	28874668	28874687
chr2_5165	p2	(GT)11	22	28875691	28875712
chr2_5166	p2	(TG)6	12	28892453	28892464
chr2_5167	p3	(ATC)5	15	28898989	28899003
chr2_5168	p2	(GA)7	14	28900059	28900072
chr2_5169	p2	(CA)14	28	28903082	28903109
chr2_5170	p2	(CA)18	36	28904664	28904699
chr2_5171	p2	(CA)15	30	28913033	28913062
chr2_5172	c	(TG)7a(GT)9	33	28917815	28917847
chr2_5173	p2	(TG)16	32	28927600	28927631
chr2_5174	p2	(TC)6	12	28929148	28929159
chr2_5175	p4	(CAAA)5	20	28930570	28930589
chr2_5176	p2	(CT)10	20	28934790	28934809
chr2_5177	p2	(GT)9	18	28941380	28941397
chr2_5178	p2	(CA)23	46	28958571	28958616
chr2_5179	p2	(CT)13	26	28964355	28964380
chr2_5180	p2	(TG)13	26	28967917	28967942
chr2_5181	p2	(GT)7	14	28975032	28975045
chr2_5182	p2	(CA)22	44	28992577	28992620
chr2_5183	p4	(TCTG)6	24	29001664	29001687
chr2_5184	p2	(AG)6	12	29019738	29019749
chr2_5185	c	:ttatgccacgcgcnnnnnnnnncccttcacacactgtctctgtgactcggc	142	29022642	29022783
chr2_5186	p4	(AGAT)7	28	29026627	29026654

chr2_5187	p4	(GACA)5	20	29027409	29027428
chr2_5188	p2	(CA)6	12	29030384	29030395
chr2_5189	p2	(TC)7	14	29033589	29033602
chr2_5190	p2	(CT)6	12	29048302	29048313
chr2_5191	p2	(TG)6	12	29059576	29059587
chr2_5192	p2	(AT)6	12	29073539	29073550
chr2_5193	p2	(TC)8	16	29086566	29086581
chr2_5194	p2	(GT)22	44	29096971	29097014
chr2_5195	p2	(GT)6	12	29136165	29136176
chr2_5196	p2	(GT)12	24	29150819	29150842
chr2_5197	p2	(GT)9	18	29152922	29152939
chr2_5198	c	(GT)6ga(GT)9	32	29157756	29157787
chr2_5199	p2	(GT)22	44	29166440	29166483
chr2_5200	p2	(AC)6	12	29175061	29175072
chr2_5201	p2	(CA)23	46	29185127	29185172
chr2_5202	p3	(TAA)5	15	29188010	29188024
chr2_5203	p4	(TCCA)6	24	29193147	29193170
chr2_5204	c	atgtaaaggggttgagaatgacaccgactccctagagagccggtcataat[illegible]	158	29224743	29224900
chr2_5205	p2	(AC)12	24	29228012	29228035
chr2_5206	p2	(TC)6	12	29249986	29249997
chr2_5207	c	(TG)8tttgtt(TG)6	34	29318089	29318122
chr2_5208	p2	(TG)6	12	29322914	29322925
chr2_5209	p2	(AC)7	14	29327321	29327334
chr2_5210	p3	(GAG)9	27	29342846	29342872
chr2_5211	p2	(TG)21	42	29344694	29344735
chr2_5212	p2	(AC)7	14	29368456	29368469
chr2_5213	c	(TG)10ta(TG)8tatatgtatatata(TG)10ta(TG)13	100	29382199	29382298
chr2_5214	p4	(TCAT)6	24	29382416	29382439
chr2_5215	p2	(TG)14	28	29385081	29385108
chr2_5216	c	[illegible]gtgcactctgagtgtggacacatctgtgtgtacctgtgagtgtctacatcct(	221	29389831	29390051
chr2_5217	p2	(AC)7	14	29391983	29391996
chr2_5218	p3	(GAT)14	42	29401814	29401855
chr2_5219	p3	(GAT)5	15	29403015	29403029
chr2_5220	p4	(TTGT)5	20	29404627	29404646
chr2_5221	p3	(ATG)7	21	29428287	29428307
chr2_5222	p3	(ATT)5	15	29430180	29430194
chr2_5223	p2	(CA)18	36	29433860	29433895
chr2_5224	p3	(CAT)5	15	29435602	29435616
chr2_5225	p2	(GA)9	18	29437500	29437517
chr2_5226	p2	(CA)7	14	29438450	29438463
chr2_5227	p2	(CA)9	18	29467389	29467406
chr2_5228	p2	(GT)27	54	29467601	29467654
chr2_5229	p2	(TG)13	26	29468073	29468098
chr2_5230	p2	(AC)12	24	29468846	29468869
chr2_5231	p3	(TTA)5	15	29475532	29475546
chr2_5232	p2	(TG)19	38	29498573	29498610
chr2_5233	p3	(CAC)5	15	29511659	29511673
chr2_5234	p2	(AG)6	12	29511790	29511801
chr2_5235	p2	(CA)6	12	29514017	29514028
chr2_5236	p3	(ATC)5	15	29514849	29514863
chr2_5237	p2	(AC)16	32	29515680	29515711
chr2_5238	p2	(CA)10	20	29518381	29518400

chr2_5239	p2	(CT)7	14	29524369	29524382
chr2_5240	p2	(GA)6	12	29528116	29528127
chr2_5241	p2	(AC)6	12	29528363	29528374
chr2_5242	p2	(TC)14	28	29544291	29544318
chr2_5243	c	(TC)10(TG)13	46	29600681	29600726
chr2_5244	p2	(CA)6	12	29602364	29602375
chr2_5245	p2	(AC)7	14	29613094	29613107
chr2_5246	p3	(CCT)5	15	29630787	29630801
chr2_5247	p2	(TG)6	12	29678524	29678535
chr2_5248	p2	(GT)6	12	29697641	29697652
chr2_5249	p2	(TG)8	16	29708764	29708779
chr2_5250	p3	(CAG)7	21	29717896	29717916
chr2_5251	p2	(TG)8	16	29721734	29721749
chr2_5252	p3	(ATT)5	15	29726535	29726549
chr2_5253	p2	(CA)9	18	29755591	29755608
chr2_5254	p2	(TA)12	24	29757645	29757668
chr2_5255	p3	(ATA)10	30	29763960	29763989
chr2_5256	p2	(CA)7	14	29783592	29783605
chr2_5257	p2	(TG)7	14	29803423	29803436
chr2_5258	p2	(CA)23	46	29803724	29803769
chr2_5259	p2	(CA)15	30	29804034	29804063
chr2_5260	p2	(AC)8	16	29807955	29807970
chr2_5261	p2	(GT)10	20	29811226	29811245
chr2_5262	p2	(TG)7	14	29812820	29812833
chr2_5263	p2	(AG)6	12	29813952	29813963
chr2_5264	p4	(TTAT)5	20	29818451	29818470
chr2_5265	p4	(ACAT)12	48	29820582	29820629
chr2_5266	p2	(TG)14	28	29830810	29830837
chr2_5267	p2	(CA)9	18	29845734	29845751
chr2_5268	p4	(ACAT)5	20	29847485	29847504
chr2_5269	p2	(TG)15	30	29850762	29850791
chr2_5270	p2	(CT)6	12	29853036	29853047
chr2_5271	p2	(GT)8	16	29860765	29860780
chr2_5272	p2	(AC)13	26	29866846	29866871
chr2_5273	p2	(TG)23	46	29867360	29867405
chr2_5274	p2	(AC)7	14	29870150	29870163
chr2_5275	p2	(AC)9	18	29870425	29870442
chr2_5276	p2	(GA)7	14	29871321	29871334
chr2_5277	p2	(TG)19	38	29878664	29878701
chr2_5278	p2	(GT)16	32	29878925	29878956
chr2_5279	p2	(TG)16	32	29883281	29883312
chr2_5280	p2	(CA)11	22	29897017	29897038
chr2_5281	p2	(AC)15	30	29900778	29900807
chr2_5282	p4	(AACA)6	24	29903684	29903707
chr2_5283	p2	(AC)6	12	29913957	29913968
chr2_5284	p2	(CT)7	14	29920248	29920261
chr2_5285	p2	(GT)17	34	29922469	29922502
chr2_5286	p2	(AC)9	18	29935008	29935025
chr2_5287	p2	(CA)20	40	29942609	29942648
chr2_5288	p2	(AC)7	14	29947243	29947256
chr2_5289	p2	(CT)8	16	29947924	29947939
chr2_5290	p3	(CTG)6	18	29958166	29958183

chr2_5291	p2	(TG)14	28	29971565	29971592
chr2_5292	p2	(CT)8	16	29980448	29980463
chr2_5293	p2	(CA)18	36	29982710	29982745
chr2_5294	p2	(CT)9	18	29987477	29987494
chr2_5295	p2	(CA)10	20	29993399	29993418
chr2_5296	p3	(CCA)5	15	29996727	29996741
chr2_5297	p2	(AC)9	18	30000938	30000955
chr2_5298	p2	(AT)8	16	30009933	30009948
chr2_5299	c*	(TG)9tatatat(ATAC)10(AC)11*	85	30010235	30010319
chr2_5300	p2	(AC)8	16	30010583	30010598
chr2_5301	p2	(TG)8	16	30013392	30013407
chr2_5302	p2	(GA)7	14	30017488	30017501
chr2_5303	c	ccagggagtaagccacaggttaggaatgactggtcaaagagcatggcctt	113	30022462	30022574
chr2_5304	p2	(GT)14	28	30022976	30023003
chr2_5305	c	(TG)6tatgtatgtatgtata(TG)6	40	30023244	30023283
chr2_5306	p2	(AG)8	16	30026973	30026988
chr2_5307	c	cgtgtgtgcgtgtgtgcgtgtgtgcgtgtgtgcgtgtgtgcgtgtgtgcgtgt	111	30027655	30027765
chr2_5308	p2	(CA)13	26	30027923	30027948
chr2_5309	p2	(CT)9	18	30030350	30030367
chr2_5310	p3	(TTC)7	21	30031437	30031457
chr2_5311	p2	(TG)9	18	30042736	30042753
chr2_5312	p4	(TTAT)5	20	30042924	30042943
chr2_5313	p2	(TG)9	18	30043202	30043219
chr2_5314	p3	(CCA)9	27	30044518	30044544
chr2_5315	p2	(TG)7	14	30044964	30044977
chr2_5316	p2	(TA)7	14	30045780	30045793
chr2_5317	p2	(TG)10	20	30047776	30047795
chr2_5318	p2	(TG)8	16	30051747	30051762
chr2_5319	p3	(ACC)5	15	30052057	30052071
chr2_5320	p2	(CA)6	12	30054239	30054250
chr2_5321	c	(TG)8c(GT)6	29	30056670	30056698
chr2_5322	p2	(GT)20	40	30056806	30056845
chr2_5323	p2	(AC)13	26	30057450	30057475
chr2_5324	p2	(AC)6	12	30060152	30060163
chr2_5325	p2	(CA)6	12	30061439	30061450
chr2_5326	p2	(GT)10	20	30062332	30062351
chr2_5327	p2	(CA)14	28	30062904	30062931
chr2_5328	p2	(CT)19	38	30063079	30063116
chr2_5329	p2	(GT)18	36	30065117	30065152
chr2_5330	p2	(AC)11	22	30068218	30068239
chr2_5331	p2	(CA)6	12	30069263	30069274
chr2_5332	c	(TG)6tatgtctgagtgtgtgtgtgcatacatgccta(TG)16	76	30076516	30076591
chr2_5333	p3	(GCA)10	30	30081001	30081030
chr2_5334	p2	(GT)26	52	30083699	30083750
chr2_5335	p3	(GCA)5	15	30086940	30086954
chr2_5336	p2	(AT)8	16	30089056	30089071
chr2_5337	p2	(CA)19	38	30089813	30089850
chr2_5338	p2	(TC)6	12	30091175	30091186
chr2_5339	p4	(TTTA)6	24	30095369	30095392
chr2_5340	p2	(CA)11	22	30098567	30098588
chr2_5341	p5	(AATAG)5	25	30108425	30108449
chr2_5342	p2	(CA)21	42	30110010	30110051

chr2_5343	p2	(AC)6	12	30110935	30110946
chr2_5344	p2	(TG)6	12	30122577	30122588
chr2_5345	p2	(AC)7	14	30126300	30126313
chr2_5346	p2	(GT)14	28	30140442	30140469
chr2_5347	p2	(TG)6	12	30143543	30143554
chr2_5348	p2	(GA)6	12	30146086	30146097
chr2_5349	p3	(CCT)7	21	30151704	30151724
chr2_5350	p2	(GT)8	16	30154316	30154331
chr2_5351	p2	(CA)7	14	30157751	30157764
chr2_5352	p4	(ATTT)5	20	30171423	30171442
chr2_5353	c	tgagctgtgctgaggtaattgattagctgtcagagccacacacacacttcat	133	30174492	30174624
chr2_5354	p4	(GTTA)5	20	30175317	30175336
chr2_5355	p2	(TG)8	16	30178019	30178034
chr2_5356	p2	(TG)18	36	30178217	30178252
chr2_5357	p2	(TG)28	56	30178855	30178910
chr2_5358	p2	(AC)17	34	30179152	30179185
chr2_5359	p2	(AC)16	32	30179621	30179652
chr2_5360	p3	(AAT)5	15	30180494	30180508
chr2_5361	c	(TG)6(AG)8	28	30181442	30181469
chr2_5362	p2	(TC)6	12	30190174	30190185
chr2_5363	p2	(CA)6	12	30209043	30209054
chr2_5364	p2	(CA)6	12	30212266	30212277
chr2_5365	p2	(GT)17	34	30213845	30213878
chr2_5366	p2	(TA)6	12	30215751	30215762
chr2_5367	p4	(ATTC)5	20	30216027	30216046
chr2_5368	p2	(AC)7	14	30221859	30221872
chr2_5369	p2	(TG)6	12	30229260	30229271
chr2_5370	p2	(TG)12	24	30229967	30229990
chr2_5371	p3	(CCT)5	15	30230104	30230118
chr2_5372	p2	(CT)15	30	30230269	30230298
chr2_5373	p2	(TC)6	12	30231514	30231525
chr2_5374	p2	(CA)16	32	30236492	30236523
chr2_5375	p3	(GGA)5	15	30242789	30242803
chr2_5376	p2	(AC)6	12	30243388	30243399
chr2_5377	p2	(CA)9	18	30244966	30244983
chr2_5378	p2	(GT)14	28	30246687	30246714
chr2_5379	p2	(GT)16	32	30246822	30246853
chr2_5380	c	tgtaccattcttcaggctctactcattaataagggagtactcagtcatggagc	124	30246963	30247086
chr2_5381	c	(TG)13(AG)62	150	30248431	30248580
chr2_5382	p2	(GT)7	14	30248791	30248804
chr2_5383	c	(GT)32gctctgcaga(TG)6	86	30252404	30252489
chr2_5384	p2	(TG)7	14	30255533	30255546
chr2_5385	p2	(CA)11	22	30256687	30256708
chr2_5386	p2	(GT)13	26	30258795	30258820
chr2_5387	p2	(AC)6	12	30259982	30259993
chr2_5388	p2	(TG)6	12	30262617	30262628
chr2_5389	p2	(TG)7	14	30270109	30270122
chr2_5390	p2	(AT)11	22	30291923	30291944
chr2_5391	c	(TC)15(AC)20	70	30292215	30292284
chr2_5392	p2	(TG)12	24	30294572	30294595
chr2_5393	p2	(AT)6	12	30301590	30301601
chr2_5394	p2	(GT)9	18	30301824	30301841

chr2_5395	c	(AC)8(AT)8	32	30313942	30313973
chr2_5396	c	(TC)14(AC)18	64	30314304	30314367
chr2_5397	p2	(CT)8	16	30319235	30319250
chr2_5398	p2	(TG)26	52	30322541	30322592
chr2_5399	p2	(TG)26	52	30322718	30322769
chr2_5400	p2	(AC)14	28	30323714	30323741
chr2_5401	p2	(TC)7	14	30329913	30329926
chr2_5402	p2	(GT)16	32	30332503	30332534
chr2_5403	p2	(AC)9	18	30332715	30332732
chr2_5404	p2	(GT)13	26	30337625	30337650
chr2_5405	c	(TC)16(AC)7	46	30339439	30339484
chr2_5406	p2	(AG)10	20	30339876	30339895
chr2_5407	p2	(AC)8	16	30341700	30341715
chr2_5408	c	(GC)6(AC)14	40	30343704	30343743
chr2_5409	p2	(CA)6	12	30345254	30345265
chr2_5410	p2	(TG)7	14	30347794	30347807
chr2_5411	p3	(CTC)5	15	30353744	30353758
chr2_5412	p2	(CA)8	16	30390238	30390253
chr2_5413	p2	(AC)6	12	30396924	30396935
chr2_5414	p2	(CA)9	18	30398364	30398381
chr2_5415	p5	(TTGAT)6	30	30403417	30403446
chr2_5416	p2	(AC)17	34	30413040	30413073
chr2_5417	p2	(TA)10	20	30414380	30414399
chr2_5418	c	(AT)6aca(AT)6	27	30416607	30416633
chr2_5419	c	(AT)6(GT)11a(TATATC)5	65	30417831	30417895
chr2_5420	p2	(AT)7	14	30419284	30419297
chr2_5421	p2	(AC)7	14	30426102	30426115
chr2_5422	p4	(CATC)5	20	30429526	30429545
chr2_5423	p2	(GT)6	12	30444881	30444892
chr2_5424	p2	(CT)6	12	30451961	30451972
chr2_5425	c	(ATTT)5(TA)6	32	30469319	30469350
chr2_5426	p4	(CACG)6	24	30474187	30474210
chr2_5427	p2	(CA)8	16	30480620	30480635
chr2_5428	p2	(AC)9	18	30481649	30481666
chr2_5429	p2	(GA)6	12	30509394	30509405
chr2_5430	p2	(GA)6	12	30519716	30519727
chr2_5431	c	(TC)8(AC)6at(AC)13	56	30519996	30520051
chr2_5432	p2	(CA)11	22	30534112	30534133
chr2_5433	p2	(TG)12	24	30543333	30543356
chr2_5434	p2	(GT)14	28	30543705	30543732
chr2_5435	p2	(AC)6	12	30547712	30547723
chr2_5436	p4	(CATC)8	32	30550496	30550527
chr2_5437	p2	(AC)11	22	30551342	30551363
chr2_5438	p2	(CA)8	16	30551607	30551622
chr2_5439	p2	(CT)11	22	30568768	30568789
chr2_5440	p2	(TG)9	18	30569515	30569532
chr2_5441	c	(TG)7g(GT)18	51	30576396	30576446
chr2_5442	p2	(CA)11	22	30578625	30578646
chr2_5443	p3	(AGG)6	18	30579857	30579874
chr2_5444	p2	(AC)7	14	30605498	30605511
chr2_5445	p2	(CT)8	16	30633071	30633086
chr2_5446	p2	(AC)6	12	30636365	30636376

chr2_5447	p2	(AC)11	22	30636646	30636667
chr2_5448	p2	(CA)6	12	30637545	30637556
chr2_5449	p2	(AC)15	30	30642715	30642744
chr2_5450	p2	(AC)11	22	30653896	30653917
chr2_5451	p3	(GAG)9	27	30657514	30657540
chr2_5452	c	aacagacacacacgcaaatgagcaggtgtgtgtagcaagcaaacaggca	90	30658579	30658668
chr2_5453	c	(AC)21agtacatataaaaagggagaaaaag(AC)11	89	30663456	30663544
chr2_5454	c	(TG)7t(AC)7	29	30675552	30675580
chr2_5455	p2	(GT)7	14	30675807	30675820
chr2_5456	p2	(TG)10	20	30682378	30682397
chr2_5457	p2	(GA)6	12	30683883	30683894
chr2_5458	p2	(AC)6	12	30695622	30695633
chr2_5459	p2	(CA)16	32	30696480	30696511
chr2_5460	p2	(AC)23	46	30698131	30698176
chr2_5461	p4	(TCCA)5	20	30700794	30700813
chr2_5462	p2	(GT)7	14	30703474	30703487
chr2_5463	p2	(AC)11	22	30706777	30706798
chr2_5464	p4	(TCTT)8	32	30710848	30710879
chr2_5465	p2	(CA)13	26	30711442	30711467
chr2_5466	p2	(CA)6	12	30712614	30712625
chr2_5467	p2	(TG)8	16	30738770	30738785
chr2_5468	p2	(CA)15	30	30742010	30742039
chr2_5469	p2	(TG)8	16	30744639	30744654
chr2_5470	p2	(GT)11	22	30744837	30744858
chr2_5471	p3	(TGA)5	15	30782897	30782911
chr2_5472	c	ctgcacacaagcatggtgaccagatatttcagatatatggactcgtactgtat	123	30784367	30784489
chr2_5473	p3	(GCT)6	18	30787161	30787178
chr2_5474	p2	(AC)22	44	30787434	30787477
chr2_5475	p2	(AC)25	50	30787641	30787690
chr2_5476	p2	(CA)8	16	30791487	30791502
chr2_5477	p2	(AC)8	16	30791992	30792007
chr2_5478	c	(TG)7tttgca(TG)6	32	30792279	30792310
chr2_5479	p2	(GT)6	12	30794063	30794074
chr2_5480	p2	(CA)10	20	30794919	30794938
chr2_5481	p2	(GT)15	30	30796856	30796885
chr2_5482	p2	(GT)17	34	30805951	30805984
chr2_5483	p4	(AGAT)20	80	30810034	30810113
chr2_5484	p2	(TG)6	12	30818934	30818945
chr2_5485	p2	(CA)42	84	30819126	30819209
chr2_5486	p5	(ATCAA)5	25	30828809	30828833
chr2_5487	p4	(ATTC)5	20	30845330	30845349
chr2_5488	p3	(TAA)11	33	30845707	30845739
chr2_5489	p2	(CA)16	32	30854820	30854851
chr2_5490	p2	(GT)27	54	30859685	30859738
chr2_5491	p3	(TCT)10	30	30864700	30864729
chr2_5492	p2	(TG)6	12	30865372	30865383
chr2_5493	p2	(CA)8	16	30868929	30868944
chr2_5494	p3	(ATA)7	21	30875264	30875284
chr2_5495	p2	(CA)6	12	30875593	30875604
chr2_5496	c	(TC)17tttctctctc(GT)10	64	30884558	30884621
chr2_5497	c	catgaagcctttcactgcaatttatttctagagttttaaagattactgtttattcat	166	30894016	30894181
chr2_5498	p2	(CT)9	18	30898906	30898923

chr2_5499	p2	(TA)10	20	30910574	30910593
chr2_5500	p2	(AC)14	28	30911864	30911891
chr2_5501	p2	(GT)6	12	30929936	30929947
chr2_5502	p2	(TG)6	12	30952414	30952425
chr2_5503	p3	(TTC)5	15	30957806	30957820
chr2_5504	p2	(AC)26	52	30958699	30958750
chr2_5505	p4	(TTTG)5	20	30966561	30966580
chr2_5506	p2	(TG)9	18	30967506	30967523
chr2_5507	p2	(GT)9	18	30970474	30970491
chr2_5508	p2	(CA)6	12	30970649	30970660
chr2_5509	p2	(GA)10	20	30970843	30970862
chr2_5510	p2	(GT)14	28	30972351	30972378
chr2_5511	p2	(TG)14	28	30973806	30973833
chr2_5512	p2	(AC)11	22	30991331	30991352
chr2_5513	p2	(GT)6	12	30993511	30993522
chr2_5514	p2	(TG)6	12	30995001	30995012
chr2_5515	p2	(GT)6	12	30997086	30997097
chr2_5516	p2	(GT)7	14	31008734	31008747
chr2_5517	p2	(CA)12	24	31010409	31010432
chr2_5518	p2	(GT)7	14	31011337	31011350
chr2_5519	p2	(AC)6	12	31013706	31013717
chr2_5520	p2	(AC)6	12	31021767	31021778
chr2_5521	p2	(AC)6	12	31022636	31022647
chr2_5522	p2	(AC)8	16	31022897	31022912
chr2_5523	p2	(CA)16	32	31023042	31023073
chr2_5524	p2	(TG)6	12	31025721	31025732
chr2_5525	c	)13aacatgcccatatacccgcattcagactcgcattactagccatgct(G	87	31025971	31026057
chr2_5526	p2	(TG)6	12	31026985	31026996
chr2_5527	p2	(TA)6	12	31029514	31029525
chr2_5528	p2	(AG)12	24	31033392	31033415
chr2_5529	p2	(CA)9	18	31034834	31034851
chr2_5530	p2	(TG)26	52	31047564	31047615
chr2_5531	p3	(GAG)5	15	31047873	31047887
chr2_5532	p2	(CA)6	12	31048015	31048026
chr2_5533	p2	(TG)6	12	31048981	31048992
chr2_5534	c	(AC)9aattctgtgtgcttgtgtgcactc(TG)6	54	31051900	31051953
chr2_5535	p2	(CA)8	16	31059758	31059773
chr2_5536	p2	(AC)6	12	31060072	31060083
chr2_5537	p4	(AATA)5	20	31075884	31075903
chr2_5538	p2	(AC)9	18	31079238	31079255
chr2_5539	p2	(AC)6	12	31084060	31084071
chr2_5540	p2	(AC)21	42	31094950	31094991
chr2_5541	p2	(GT)8	16	31096614	31096629
chr2_5542	p2	(AT)11	22	31100983	31101004
chr2_5543	p2	(AC)6	12	31106649	31106660
chr2_5544	p2	(AC)6	12	31111943	31111954
chr2_5545	p3	(ACA)5	15	31113106	31113120
chr2_5546	p2	(TG)6	12	31116432	31116443
chr2_5547	p4	(GAGG)5	20	31122509	31122528
chr2_5548	p2	(AC)8	16	31123789	31123804
chr2_5549	p2	(GT)11	22	31125719	31125740
chr2_5550	p2	(AG)14	28	31128597	31128624

chr2_5551	p2	(AC)7	14	31129933	31129946
chr2_5552	p2	(GA)6	12	31134873	31134884
chr2_5553	p2	(AC)7	14	31138947	31138960
chr2_5554	p2	(AC)17	34	31146757	31146790
chr2_5555	p3	(TTC)5	15	31149618	31149632
chr2_5556	c	gatgcttacgcatccacacagtaataataaataaataggataacatagtatg	148	31156212	31156359
chr2_5557	c	tcttgaagagaaggcagctgctcttataatcactgattcttccaaaaatactc	154	31158797	31158950
chr2_5558	p2	(CA)10	20	31164464	31164483
chr2_5559	p2	(GA)6	12	31164662	31164673
chr2_5560	p2	(CA)14	28	31167483	31167510
chr2_5561	p2	(TG)6	12	31170417	31170428
chr2_5562	p2	(AC)14	28	31172617	31172644
chr2_5563	p2	(GT)10	20	31173692	31173711
chr2_5564	p2	(TC)20	40	31174721	31174760
chr2_5565	p3	(GAG)5	15	31179692	31179706
chr2_5566	p2	(TG)6	12	31180612	31180623
chr2_5567	p2	(TG)23	46	31180808	31180853
chr2_5568	p2	(CA)17	34	31188016	31188049
chr2_5569	p2	(GT)12	24	31188171	31188194
chr2_5570	p4	(AAAT)5	20	31189540	31189559
chr2_5571	p2	(AC)6	12	31191365	31191376
chr2_5572	p2	(TG)6	12	31194810	31194821
chr2_5573	c	ttcactgattgagattctcaagggagcgaatcctgaacctcacctgctgcac	118	31196817	31196934
chr2_5574	p2	(TG)9	18	31199494	31199511
chr2_5575	p2	(CA)8	16	31199800	31199815
chr2_5576	p2	(AC)6	12	31205543	31205554
chr2_5577	p2	(CA)22	44	31206819	31206862
chr2_5578	p2	(CA)7	14	31214476	31214489
chr2_5579	p2	(TC)8	16	31217509	31217524
chr2_5580	p2	(TG)6	12	31230644	31230655
chr2_5581	p2	(GT)6	12	31232678	31232689
chr2_5582	p2	(CA)11	22	31232873	31232894
chr2_5583	p2	(AC)10	20	31234567	31234586
chr2_5584	p2	(AC)6	12	31235609	31235620
chr2_5585	c	ataaatactggagaggacgtgggtctggtgtcatggaccagtgaactgacc	81	31239117	31239197
chr2_5586	p2	(CA)6	12	31239432	31239443
chr2_5587	c	(GT)19gagaa(AG)8	59	31240769	31240827
chr2_5588	p2	(AT)11	22	31241294	31241315
chr2_5589	p2	(AG)6	12	31241452	31241463
chr2_5590	p2	(AC)9	18	31242953	31242970
chr2_5591	p2	(CT)7	14	31246213	31246226
chr2_5592	p2	(AC)7	14	31250692	31250705
chr2_5593	p2	(GA)6	12	31251210	31251221
chr2_5594	p2	(GT)9	18	31251473	31251490
chr2_5595	c	(TG)8taactgtaccta(TG)8	44	31254274	31254317
chr2_5596	p2	(AC)28	56	31260690	31260745
chr2_5597	p2	(AC)16	32	31262176	31262207
chr2_5598	c	(TC)7(AC)17	48	31264028	31264075
chr2_5599	p3	(TGT)7	21	31293469	31293489
chr2_5600	p2	(GA)7	14	31294605	31294618
chr2_5601	p2	(TC)7	14	31296056	31296069
chr2_5602	p2	(CA)11	22	31296956	31296977

chr2_5603	p2	(AC)16	32	31303912	31303943
chr2_5604	p3	(AGA)5	15	31311302	31311316
chr2_5605	p4	(TTTC)8	32	31326565	31326596
chr2_5606	p2	(AC)6	12	31329059	31329070
chr2_5607	c	(CA)16cctgttcacaactcacc(CT)11	71	31360320	31360390
chr2_5608	c	(GGT)7agtggtaa(TGG)6	47	31376476	31376522
chr2_5609	p2	(AG)13	26	31384926	31384951
chr2_5610	p2	(AG)6	12	31389542	31389553
chr2_5611	p2	(AC)6	12	31395198	31395209
chr2_5612	p2	(AG)6	12	31401105	31401116
chr2_5613	p3	(CAG)5	15	31406243	31406257
chr2_5614	p2	(TG)6	12	31407683	31407694
chr2_5615	p2	(AC)6	12	31408521	31408532
chr2_5616	c	(TGT)8(TCT)8	48	31420312	31420359
chr2_5617	p2	(TG)6	12	31427496	31427507
chr2_5618	p2	(AC)7	14	31429649	31429662
chr2_5619	p3	(TAA)8	24	31436961	31436984
chr2_5620	p3	(ATA)6	18	31448777	31448794
chr2_5621	p2	(AG)7	14	31456975	31456988
chr2_5622	p2	(CA)6	12	31459251	31459262
chr2_5623	p3	(AAG)5	15	31467838	31467852
chr2_5624	p2	(AT)8	16	31473048	31473063
chr2_5625	p2	(AC)6	12	31483693	31483704
chr2_5626	p3	(TCT)5	15	31510824	31510838
chr2_5627	p4	(ATGG)6	24	31532215	31532238
chr2_5628	p2	(GT)6	12	31533187	31533198
chr2_5629	p2	(AC)6	12	31543284	31543295
chr2_5630	p4	(GACA)5	20	31548989	31549008
chr2_5631	p2	(TG)19	38	31553531	31553568
chr2_5632	p2	(CA)11	22	31579092	31579113
chr2_5633	p3	(TAT)5	15	31591560	31591574
chr2_5634	p3	(TCT)5	15	31604191	31604205
chr2_5635	p2	(TG)8	16	31617045	31617060
chr2_5636	p3	(TTG)14	42	31623057	31623098
chr2_5637	p2	(GT)6	12	31631208	31631219
chr2_5638	p2	(CA)6	12	31632085	31632096
chr2_5639	c	(CT)12(CA)9	42	31636695	31636736
chr2_5640	p3	(ATT)5	15	31637313	31637327
chr2_5641	c	gtgtggatacatttttattcattgccatcgttgtgaggacattttagccagtata	124	31637629	31637752
chr2_5642	p2	(AC)6	12	31642055	31642066
chr2_5643	p2	(CA)6	12	31647529	31647540
chr2_5644	p2	(AC)7	14	31651711	31651724
chr2_5645	p2	(TG)16	32	31651978	31652009
chr2_5646	p2	(AC)8	16	31653828	31653843
chr2_5647	p2	(CA)13	26	31657341	31657366
chr2_5648	p2	(AC)14	28	31662908	31662935
chr2_5649	p2	(AC)19	38	31664489	31664526
chr2_5650	p2	(CA)12	24	31666061	31666084
chr2_5651	p2	(CA)8	16	31673202	31673217
chr2_5652	p2	(GT)7	14	31673728	31673741
chr2_5653	p3	(AAT)5	15	31675977	31675991
chr2_5654	p2	(CA)6	12	31683832	31683843

chr2_5655	p2	(GT)9	18	31686026	31686043
chr2_5656	p2	(GA)6	12	31695105	31695116
chr2_5657	p2	(AC)13	26	31696600	31696625
chr2_5658	p2	(CA)17	34	31702512	31702545
chr2_5659	p2	(TG)13	26	31703568	31703593
chr2_5660	p2	(AT)8	16	31714203	31714218
chr2_5661	p2	(TG)7	14	31718113	31718126
chr2_5662	p3	(AGA)6	18	31736492	31736509
chr2_5663	c	(AT)9gtgtatacatatata(TATG)14	89	31740241	31740329
chr2_5664	p2	(GA)16	32	31743599	31743630
chr2_5665	p2	(AC)21	42	31745016	31745057
chr2_5666	p2	(TG)6	12	31782567	31782578
chr2_5667	p2	(CA)8	16	31786272	31786287
chr2_5668	c	ıtgtataccacatatatgcttttttaaaagcctctggctagcacacagggaca	152	31786541	31786692
chr2_5669	p3	(AAC)7	21	31786833	31786853
chr2_5670	p2	(CA)6	12	31786976	31786987
chr2_5671	p2	(TA)10	20	31789428	31789447
chr2_5672	p2	(GT)15	30	31792721	31792750
chr2_5673	c	(CT)6caac(CA)6	28	31794591	31794618
chr2_5674	p2	(GA)6	12	31795697	31795708
chr2_5675	c	T)16atctgttggttggaggtggaagggagggtgggatgtatgtgtc(TC	93	31797810	31797902
chr2_5676	c	(TG)8tc(TG)16	50	31799574	31799623
chr2_5677	p2	(TG)6	12	31800394	31800405
chr2_5678	p2	(CA)7	14	31800679	31800692
chr2_5679	p2	(TG)14	28	31803738	31803765
chr2_5680	p4	(TTTG)5	20	31805681	31805700
chr2_5681	p3	(GTT)9	27	31807201	31807227
chr2_5682	p2	(GA)7	14	31814418	31814431
chr2_5683	p2	(GT)14	28	31818421	31818448
chr2_5684	p2	(AC)6	12	31818818	31818829
chr2_5685	p2	(GA)8	16	31819022	31819037
chr2_5686	p2	(CT)6	12	31820430	31820441
chr2_5687	p3	(AAG)10	30	31826677	31826706
chr2_5688	p2	(AG)9	18	31827770	31827787
chr2_5689	p2	(TG)6	12	31830047	31830058
chr2_5690	p2	(TG)6	12	31839811	31839822
chr2_5691	p2	(AC)6	12	31840146	31840157
chr2_5692	p2	(AG)7	14	31841431	31841444
chr2_5693	c	aactgaggagggaaagtaattgaggaggcagtcaagtgaaaacagatttg	118	31842618	31842735
chr2_5694	p3	(CAG)5	15	31842851	31842865
chr2_5695	p2	(GT)8	16	31847045	31847060
chr2_5696	p2	(CA)22	44	31848242	31848285
chr2_5697	c	(CA)15(TA)6	42	31855796	31855837
chr2_5698	p2	(CA)6	12	31856190	31856201
chr2_5699	p2	(AC)13	26	31856462	31856487
chr2_5700	p4	(AACA)6	24	31856722	31856745
chr2_5701	p2	(AC)18	36	31857992	31858027
chr2_5702	p2	(GT)7	14	31863127	31863140
chr2_5703	p2	(TG)9	18	31865408	31865425
chr2_5704	p2	(TG)6	12	31869033	31869044
chr2_5705	p2	(TG)6	12	31869713	31869724
chr2_5706	p3	(TTG)7	21	31876608	31876628

chr2_5707	p3	(GAG)5	15	31882102	31882116
chr2_5708	p2	(TG)14	28	31882639	31882666
chr2_5709	p2	(GA)7	14	31884928	31884941
chr2_5710	c	)17ttatctgataatgagttgtgcagggtcaccgagggagaaacactc(T(	111	31887620	31887730
chr2_5711	p2	(AG)12	24	31902345	31902368
chr2_5712	p2	(AC)9	18	31916076	31916093
chr2_5713	c*	(CAGA)7(AC)7*	41	31917260	31917300
chr2_5714	p2	(AC)14	28	31919155	31919182
chr2_5715	p2	(CA)8	16	31925129	31925144
chr2_5716	p3	(TCC)5	15	31927107	31927121
chr2_5717	p2	(CA)14	28	31939708	31939735
chr2_5718	p3	(AGG)5	15	31947381	31947395
chr2_5719	c	(AC)12agtacaagcgcacttctt(TC)6	54	31949185	31949238
chr2_5720	p2	(GT)11	22	31959305	31959326
chr2_5721	p2	(CA)8	16	31959709	31959724
chr2_5722	p2	(AC)7	14	31965009	31965022
chr2_5723	p2	(CA)15	30	31965946	31965975
chr2_5724	p2	(TG)12	24	31967372	31967395
chr2_5725	p2	(AC)6	12	31967598	31967609
chr2_5726	c	(GA)6c(AG)7	27	31973554	31973580
chr2_5727	p2	(GA)6	12	31975323	31975334
chr2_5728	p2	(AC)6	12	31988543	31988554
chr2_5729	p4	(ATGG)5	20	32021182	32021201
chr2_5730	p4	(AATC)5	20	32026392	32026411
chr2_5731	p2	(TG)6	12	32036201	32036212
chr2_5732	p2	(TG)11	22	32043128	32043149
chr2_5733	p3	(CAG)5	15	32048911	32048925
chr2_5734	p2	(AG)6	12	32050412	32050423
chr2_5735	p2	(TG)6	12	32052647	32052658
chr2_5736	c	(AAC)6cgcaactactaccactac(TAA)5	51	32082870	32082920
chr2_5737	p4	(GATG)5	20	32086913	32086932
chr2_5738	p4	(TCTA)8	32	32087470	32087501
chr2_5739	c	(AG)8gggcacatagacagagt(GA)6	45	32105168	32105212
chr2_5740	p2	(AC)7	14	32106228	32106241
chr2_5741	p2	(GT)6	12	32107319	32107330
chr2_5742	p2	(CA)11	22	32107977	32107998
chr2_5743	p2	(TA)23	46	32108370	32108415
chr2_5744	p2	(GT)23	46	32112774	32112819
chr2_5745	p2	(GT)14	28	32115424	32115451
chr2_5746	p2	(GT)17	34	32118448	32118481
chr2_5747	p2	(AC)12	24	32119235	32119258
chr2_5748	p2	(CT)10	20	32120114	32120133
chr2_5749	p2	(GT)11	22	32122736	32122757
chr2_5750	p2	(TG)7	14	32122905	32122918
chr2_5751	p2	(TG)23	46	32123428	32123473
chr2_5752	c	(AC)12agatgtaccctggtgtgttgac(TG)7	60	32126861	32126920
chr2_5753	p2	(CA)11	22	32127770	32127791
chr2_5754	p2	(CA)14	28	32128557	32128584
chr2_5755	p2	(TG)16	32	32130424	32130455
chr2_5756	p2	(GT)18	36	32136169	32136204
chr2_5757	p2	(GT)6	12	32137265	32137276
chr2_5758	p3	(TGT)5	15	32137611	32137625

chr2_5759	p2	(AC)8	16	32138470	32138485
chr2_5760	p2	(CT)17	34	32141218	32141251
chr2_5761	p2	(TG)25	50	32145607	32145656
chr2_5762	c	(CA)6g(AC)6	25	32147523	32147547
chr2_5763	p3	(TAA)8	24	32148786	32148809
chr2_5764	p3	(AGC)6	18	32151346	32151363
chr2_5765	p2	(TG)6	12	32156008	32156019
chr2_5766	p2	(GT)6	12	32159482	32159493
chr2_5767	p2	(GA)10	20	32160265	32160284
chr2_5768	p2	(CA)8	16	32175558	32175573
chr2_5769	p2	(GA)11	22	32182046	32182067
chr2_5770	p2	(CT)6	12	32183689	32183700
chr2_5771	p2	(CA)7	14	32203242	32203255
chr2_5772	p2	(AC)18	36	32204408	32204443
chr2_5773	p3	(CTA)5	15	32212955	32212969
chr2_5774	p2	(TG)6	12	32219630	32219641
chr2_5775	p2	(CA)8	16	32221085	32221100
chr2_5776	p3	(AAC)5	15	32227258	32227272
chr2_5777	p2	(CA)8	16	32227551	32227566
chr2_5778	p2	(GT)14	28	32232625	32232652
chr2_5779	p2	(TA)6	12	32234465	32234476
chr2_5780	p5	(TAACA)7	35	32237698	32237732
chr2_5781	p2	(CA)6	12	32239551	32239562
chr2_5782	p2	(CA)9	18	32249402	32249419
chr2_5783	p2	(GT)9	18	32258269	32258286
chr2_5784	p2	(AC)24	48	32258414	32258461
chr2_5785	p2	(CA)6	12	32258710	32258721
chr2_5786	p2	(AC)6	12	32263605	32263616
chr2_5787	p4	(TTTA)5	20	32264464	32264483
chr2_5788	p2	(AC)8	16	32267080	32267095
chr2_5789	p2	(AC)10	20	32269052	32269071
chr2_5790	p2	(AC)6	12	32269914	32269925
chr2_5791	c	(AC)7cacagatgacatagcttgtgattttca(TG)6	53	32270867	32270919
chr2_5792	p3	(TCC)5	15	32274662	32274676
chr2_5793	p2	(AT)7	14	32279219	32279232
chr2_5794	p4	(AATC)5	20	32281239	32281258
chr2_5795	p2	(AC)9	18	32286598	32286615
chr2_5796	p2	(AT)7	14	32296639	32296652
chr2_5797	p2	(TG)18	36	32304366	32304401
chr2_5798	p2	(CA)7	14	32313556	32313569
chr2_5799	p3	(GAA)5	15	32315765	32315779
chr2_5800	p2	(GT)11	22	32320421	32320442
chr2_5801	p2	(TG)10	20	32320944	32320963
chr2_5802	p2	(AC)11	22	32324000	32324021
chr2_5803	p2	(CA)8	16	32327054	32327069
chr2_5804	p2	(TG)7	14	32327269	32327282
chr2_5805	p2	(GT)13	26	32347089	32347114
chr2_5806	p2	(GT)10	20	32348833	32348852
chr2_5807	p4	(GATG)5	20	32352849	32352868
chr2_5808	p2	(GT)7	14	32353648	32353661
chr2_5809	p2	(GT)17	34	32353941	32353974
chr2_5810	p2	(TA)7	14	32363407	32363420

chr2_5811	p2	(GT)8	16	32364716	32364731
chr2_5812	p2	(TG)10	20	32390744	32390763
chr2_5813	p3	(TGT)5	15	32398844	32398858
chr2_5814	p2	(CA)25	50	32412090	32412139
chr2_5815	p2	(CT)17	34	32412577	32412610
chr2_5816	p2	(GT)10	20	32422841	32422860
chr2_5817	p3	(ATT)7	21	32427976	32427996
chr2_5818	p2	(CT)6	12	32443919	32443930
chr2_5819	p2	(AC)6	12	32446759	32446770
chr2_5820	p2	(TC)6	12	32450605	32450616
chr2_5821	p2	(GT)7	14	32455949	32455962
chr2_5822	p2	(GA)6	12	32456463	32456474
chr2_5823	p2	(CA)6	12	32477867	32477878
chr2_5824	c	attccccatcttatcttgatgcattagcaagtgcagagtcatcggttatcctct	109	32478304	32478412
chr2_5825	p3	(GGA)6	18	32479218	32479235
chr2_5826	p2	(AG)6	12	32483816	32483827
chr2_5827	p4	(TCTG)6	24	32484524	32484547
chr2_5828	p2	(TC)7	14	32490479	32490492
chr2_5829	p2	(TG)11	22	32499205	32499226
chr2_5830	p2	(TG)25	50	32503874	32503923
chr2_5831	p2	(CA)10	20	32506344	32506363
chr2_5832	p2	(TG)6	12	32510337	32510348
chr2_5833	p2	(TG)24	48	32523928	32523975
chr2_5834	p2	(TC)7	14	32533440	32533453
chr2_5835	p4	(ACAT)5	20	32535433	32535452
chr2_5836	p2	(CA)12	24	32535767	32535790
chr2_5837	p2	(CA)7	14	32546889	32546902
chr2_5838	p3	(TAT)6	18	32552294	32552311
chr2_5839	p2	(TC)6	12	32562208	32562219
chr2_5840	p2	(GT)13	26	32567246	32567271
chr2_5841	p3	(ATT)6	18	32573659	32573676
chr2_5842	p2	(CA)18	36	32588724	32588759
chr2_5843	p3	(GGA)6	18	32593974	32593991
chr2_5844	p3	(ATA)5	15	32620061	32620075
chr2_5845	p2	(AC)13	26	32620577	32620602
chr2_5846	p2	(GT)7	14	32620762	32620775
chr2_5847	p2	(TG)7	14	32621960	32621973
chr2_5848	p2	(GT)16	32	32630382	32630413
chr2_5849	p2	(AT)6	12	32632594	32632605
chr2_5850	p2	(TG)14	28	32640106	32640133
chr2_5851	p2	(TG)25	50	32641737	32641786
chr2_5852	p2	(TG)7	14	32644320	32644333
chr2_5853	p2	(CA)22	44	32648715	32648758
chr2_5854	p2	(AC)28	56	32650080	32650135
chr2_5855	p3	(GGA)7	21	32650581	32650601
chr2_5856	p2	(TG)8	16	32654095	32654110
chr2_5857	p2	(AC)9	18	32655376	32655393
chr2_5858	p2	(TG)10	20	32655572	32655591
chr2_5859	c	(TG)20(AG)6	52	32660123	32660174
chr2_5860	p2	(GT)6	12	32662719	32662730
chr2_5861	p2	(AC)6	12	32662893	32662904
chr2_5862	p2	(TG)10	20	32665411	32665430

chr2_5863	p2	(TG)12	24	32665995	32666018
chr2_5864	p2	(AC)6	12	32666709	32666720
chr2_5865	c	(AC)20(AT)8	56	32669689	32669744
chr2_5866	p2	(TG)13	26	32675754	32675779
chr2_5867	p2	(AC)6	12	32688131	32688142
chr2_5868	p2	(AT)6	12	32692307	32692318
chr2_5869	p3	(TCT)5	15	32712211	32712225
chr2_5870	p5	(AAATC)5	25	32717844	32717868
chr2_5871	p2	(TG)9	18	32723571	32723588
chr2_5872	p3	(ATT)6	18	32757104	32757121
chr2_5873	c	(GT)14gg(GT)9	48	32762855	32762902
chr2_5874	p3	(ATC)6	18	32763313	32763330
chr2_5875	p2	(TG)7	14	32764925	32764938
chr2_5876	p3	(GAT)8	24	32769046	32769069
chr2_5877	c	(TG)9agacttgtctcgggtgtgtgtttgca(TG)7	58	32775549	32775606
chr2_5878	p2	(TC)6	12	32776622	32776633
chr2_5879	p2	(TC)6	12	32784686	32784697
chr2_5880	p2	(CA)22	44	32791907	32791950
chr2_5881	p3	(TAT)5	15	32812287	32812301
chr2_5882	p2	(AC)12	24	32814259	32814282
chr2_5883	p2	(TG)13	26	32814725	32814750
chr2_5884	p2	(TG)15	30	32815443	32815472
chr2_5885	p2	(TG)9	18	32815639	32815656
chr2_5886	p5	(TGTGA)8	40	32822857	32822896
chr2_5887	c	(AG)12acagc(GA)7	43	32826500	32826542
chr2_5888	p3	(TAA)5	15	32827826	32827840
chr2_5889	p2	(TG)18	36	32832526	32832561
chr2_5890	p2	(CT)6	12	32832979	32832990
chr2_5891	p2	(GT)6	12	32833492	32833503
chr2_5892	p2	(GT)8	16	32840721	32840736
chr2_5893	p3	(GTT)5	15	32847277	32847291
chr2_5894	p2	(TG)6	12	32855940	32855951
chr2_5895	c	(GT)17(GA)12	58	32871194	32871251
chr2_5896	p2	(GT)6	12	32883931	32883942
chr2_5897	p2	(CA)15	30	32884071	32884100
chr2_5898	p2	(GT)11	22	32885166	32885187
chr2_5899	p2	(AC)17	34	32885303	32885336
chr2_5900	p2	(TG)9	18	32887634	32887651
chr2_5901	p2	(TG)14	28	32888878	32888905
chr2_5902	p2	(GT)20	40	32894342	32894381
chr2_5903	p2	(AC)24	48	32894925	32894972
chr2_5904	p2	(CA)6	12	32899694	32899705
chr2_5905	p2	(AG)8	16	32914077	32914092
chr2_5906	p2	(AC)10	20	32914459	32914478
chr2_5907	p2	(TC)8	16	32918013	32918028
chr2_5908	p2	(AC)7	14	32919171	32919184
chr2_5909	p2	(TG)6	12	32920228	32920239
chr2_5910	p2	(AT)9	18	32941964	32941981
chr2_5911	p2	(CA)6	12	32945632	32945643
chr2_5912	p4	(CATC)5	20	32947162	32947181
chr2_5913	p2	(AC)13	26	32950597	32950622
chr2_5914	p2	(AC)10	20	32956013	32956032

chr2_5915	p2	(AT)6	12	32966328	32966339
chr2_5916	p3	(ATT)9	27	32972604	32972630
chr2_5917	p3	(CTC)5	15	32976033	32976047
chr2_5918	p2	(GT)17	34	32978830	32978863
chr2_5919	p2	(AC)14	28	32979100	32979127
chr2_5920	p2	(TC)6	12	32982073	32982084
chr2_5921	p2	(AC)15	30	32985087	32985116
chr2_5922	p2	(TG)12	24	32986856	32986879
chr2_5923	p2	(GT)6	12	32989163	32989174
chr2_5924	p2	(GT)16	32	33000188	33000219
chr2_5925	p2	(TA)8	16	33006995	33007010
chr2_5926	p2	(AC)9	18	33009508	33009525
chr2_5927	p2	(GT)7	14	33011938	33011951
chr2_5928	p2	(AC)13	26	33014234	33014259
chr2_5929	p3	(TAT)5	15	33019800	33019814
chr2_5930	p3	(AAT)5	15	33029015	33029029
chr2_5931	c	(TAGA)7aagac(AGAT)5	53	33033819	33033871
chr2_5932	p2	(GA)6	12	33035362	33035373
chr2_5933	p2	(TG)10	20	33046589	33046608
chr2_5934	p2	(CA)8	16	33062625	33062640
chr2_5935	p2	(TG)7	14	33076136	33076149
chr2_5936	p2	(TG)10	20	33082908	33082927
chr2_5937	p2	(CA)6	12	33089144	33089155
chr2_5938	p2	(AC)11	22	33089740	33089761
chr2_5939	p2	(TG)20	40	33093704	33093743
chr2_5940	p3	(TAA)7	21	33100985	33101005
chr2_5941	p2	(TA)7	14	33103534	33103547
chr2_5942	p2	(GT)14	28	33110428	33110455
chr2_5943	p2	(AC)7	14	33113098	33113111
chr2_5944	p2	(AT)7	14	33121900	33121913
chr2_5945	p2	(CA)9	18	33132487	33132504
chr2_5946	c	(TC)8tttctctctgta(TG)14	56	33135771	33135826
chr2_5947	c	(CA)18ctgaaaaaaat(AC)7	61	33141299	33141359
chr2_5948	p3	(TTA)5	15	33153614	33153628
chr2_5949	c	(TG)15tagactgaagctggttgtgaggggcaca(GAT)7	79	33167892	33167970
chr2_5950	p2	(CA)6	12	33170197	33170208
chr2_5951	p2	(GT)7	14	33177220	33177233
chr2_5952	p3	(AAC)5	15	33178011	33178025
chr2_5953	p2	(GT)6	12	33181033	33181044
chr2_5954	p2	(CA)14	28	33199311	33199338
chr2_5955	p2	(AG)6	12	33202798	33202809
chr2_5956	p2	(AC)15	30	33244523	33244552
chr2_5957	p2	(TG)12	24	33247029	33247052
chr2_5958	p2	(TG)8	16	33250312	33250327
chr2_5959	p2	(AC)9	18	33250547	33250564
chr2_5960	p2	(CT)6	12	33259467	33259478
chr2_5961	p3	(TCT)6	18	33260500	33260517
chr2_5962	p2	(CA)17	34	33268300	33268333
chr2_5963	c*	(TA)7(ATAC)7*ata(TG)11	64	33271886	33271949
chr2_5964	c	(AC)9agac(AG)6	34	33280631	33280664
chr2_5965	c	[illegible]tgcatcattgtagaagtgcaccactataagctgactgtgtgccctttgtagtt[illegible]	101	33291647	33291747
chr2_5966	p3	(ATA)5	15	33298990	33299004

chr2_5967	p3	(AGA)5	15	33304627	33304641
chr2_5968	p2	(AC)8	16	33309377	33309392
chr2_5969	c	(AC)17(AT)7	48	33321054	33321101
chr2_5970	p2	(AC)7	14	33334228	33334241
chr2_5971	p3	(GAT)16	48	33339743	33339790
chr2_5972	p2	(CA)6	12	33365680	33365691
chr2_5973	p2	(GA)9	18	33401293	33401310
chr2_5974	p2	(AC)12	24	33404033	33404056
chr2_5975	c	(CA)7tgcacgcacacacacactctctctg(TC)7	53	33404172	33404224
chr2_5976	p2	(AC)9	18	33408110	33408127
chr2_5977	p2	(GA)7	14	33423087	33423100
chr2_5978	p2	(CA)13	26	33424401	33424426
chr2_5979	p2	(CA)10	20	33450260	33450279
chr2_5980	p2	(CA)14	28	33453944	33453971
chr2_5981	p2	(TG)6	12	33458040	33458051
chr2_5982	c	(TC)14act(CA)10	51	33465496	33465546
chr2_5983	p2	(CA)21	42	33484883	33484924
chr2_5984	p2	(TA)10	20	33488842	33488861
chr2_5985	p4	(ATAG)5	20	33492462	33492481
chr2_5986	p2	(TG)6	12	33493254	33493265
chr2_5987	p2	(AT)10	20	33499619	33499638
chr2_5988	c	)7ccc(AT)8gtatgtatg(TA)7tgtatgtatg(TA)7tgtatgtatg(T	106	33503592	33503697
chr2_5989	p2	(CA)6	12	33519587	33519598
chr2_5990	p2	(TG)6	12	33519979	33519990
chr2_5991	p2	(TG)13	26	33525337	33525362
chr2_5992	p4	(CATC)7	28	33545473	33545500
chr2_5993	p2	(AT)10	20	33550641	33550660
chr2_5994	p4	(CCAT)5	20	33557916	33557935
chr2_5995	p4	(TACC)7	28	33558673	33558700
chr2_5996	p2	(AC)7	14	33578628	33578641
chr2_5997	p2	(CT)6	12	33581231	33581242
chr2_5998	p2	(TG)9	18	33587887	33587904
chr2_5999	p2	(TG)13	26	33594612	33594637
chr2_6000	p2	(TG)7	14	33595527	33595540
chr2_6001	p3	(ATC)10	30	33596059	33596088
chr2_6002	c	(TG)17tcgtgacactgagctttggtcgctcag(CT)7	75	33599426	33599500
chr2_6003	p2	(TG)6	12	33601335	33601346
chr2_6004	c	ıgagactttattggggtagagggagaaataataaaaagagagaaaatggg	114	33603199	33603312
chr2_6005	p2	(GA)6	12	33609864	33609875
chr2_6006	p2	(CA)19	38	33612013	33612050
chr2_6007	c*	(GT)6(GTGC)6*	34	33612297	33612330
chr2_6008	p2	(GT)7	14	33612994	33613007
chr2_6009	c	ttgcatgtgtgcca(GT)10gaggttgtcagaaatcactggtaaagatgg	190	33619636	33619825
chr2_6010	c	(TG)12(AG)6	36	33620195	33620230
chr2_6011	p2	(CA)6	12	33622317	33622328
chr2_6012	p2	(AC)6	12	33634466	33634477
chr2_6013	p2	(TG)7	14	33639909	33639922
chr2_6014	p2	(GA)10	20	33640217	33640236
chr2_6015	p2	(AC)6	12	33642957	33642968
chr2_6016	p2	(CA)6	12	33644676	33644687
chr2_6017	p2	(AC)13	26	33656755	33656780
chr2_6018	p2	(TG)8	16	33662485	33662500

chr2_6019	p2	(CA)6	12	33666622	33666633
chr2_6020	p2	(CA)7	14	33670566	33670579
chr2_6021	p3	(TTG)5	15	33676488	33676502
chr2_6022	p2	(TG)17	34	33690159	33690192
chr2_6023	p5	(CACAC)5	25	33691100	33691124
chr2_6024	p2	(TC)8	16	33722492	33722507
chr2_6025	p2	(AC)7	14	33733234	33733247
chr2_6026	c	(GT)8a(TG)7ta(TG)7ta(TG)7ta(TG)7ta(TG)7	95	33737653	33737747
chr2_6027	p2	(GT)11	22	33774562	33774583
chr2_6028	p2	(CT)9	18	33782986	33783003
chr2_6029	c	(AC)16aaacacacacaa(AC)11	66	33789371	33789436
chr2_6030	p2	(GT)14	28	33803511	33803538
chr2_6031	p2	(AC)17	34	33806685	33806718
chr2_6032	p2	(AC)6	12	33818330	33818341
chr2_6033	p2	(AC)11	22	33829677	33829698
chr2_6034	p3	(AGA)5	15	33839508	33839522
chr2_6035	p2	(AC)9	18	33871314	33871331
chr2_6036	p2	(CA)7	14	33873337	33873350
chr2_6037	p2	(TG)6	12	33875614	33875625
chr2_6038	p2	(CA)6	12	33919485	33919496
chr2_6039	p2	(CT)6	12	33935126	33935137
chr2_6040	p2	(TG)6	12	33935598	33935609
chr2_6041	p3	(AAC)11	33	33940817	33940849
chr2_6042	p2	(AC)15	30	33942904	33942933
chr2_6043	c	ggtggggagatcaagtgttcgcaaggctgtatgtttttca(TC)8ta(TC)[illegible]	140	33951101	33951240
chr2_6044	p2	(CA)8	16	33952020	33952035
chr2_6045	p2	(AC)7	14	33966195	33966208
chr2_6046	p2	(CA)13	26	33966506	33966531
chr2_6047	c	(GA)8aagtgt(GTGA)7	50	33971264	33971313
chr2_6048	p2	(GA)6	12	33974588	33974599
chr2_6049	p2	(GT)13	26	33974826	33974851
chr2_6050	p2	(TC)10	20	33985763	33985782
chr2_6051	p2	(GT)15	30	33993639	33993668
chr2_6052	p2	(GT)7	14	33997434	33997447
chr2_6053	p2	(TG)6	12	34001322	34001333
chr2_6054	p2	(GA)6	12	34005236	34005247
chr2_6055	p2	(CA)12	24	34010307	34010330
chr2_6056	p3	(ATA)7	21	34011016	34011036
chr2_6057	c	(GT)6gagtgagcgagtgagc(GA)56	140	34011985	34012124
chr2_6058	p2	(AC)6	12	34022120	34022131
chr2_6059	p3	(GTT)6	18	34022477	34022494
chr2_6060	p2	(TA)9	18	34049976	34049993
chr2_6061	p3	(AAT)5	15	34058344	34058358
chr2_6062	p3	(AAT)5	15	34059149	34059163
chr2_6063	p2	(TA)7	14	34066599	34066612
chr2_6064	p5	(AAATC)5	25	34072845	34072869
chr2_6065	p3	(TAT)6	18	34075094	34075111
chr2_6066	p2	(AC)8	16	34085710	34085725
chr2_6067	p2	(GT)8	16	34093321	34093336
chr2_6068	p2	(AT)6	12	34095119	34095130
chr2_6069	p2	(GT)6	12	34116256	34116267
chr2_6070	p2	(AC)8	16	34122889	34122904

chr2_6071	p2	(AC)6	12	34123464	34123475
chr2_6072	c	gtagt(GA)6ggtgtttgtgtagtgagaaagagagaaaggtgtggttgtctg	153	34135197	34135349
chr2_6073	p4	(AACA)5	20	34139582	34139601
chr2_6074	p3	(TTG)5	15	34143377	34143391
chr2_6075	p2	(TG)9	18	34143612	34143629
chr2_6076	p2	(AC)8	16	34144454	34144469
chr2_6077	p2	(AC)7	14	34156452	34156465
chr2_6078	p2	(GT)8	16	34161574	34161589
chr2_6079	p2	(TC)6	12	34162748	34162759
chr2_6080	p4	(CACT)5	20	34170943	34170962
chr2_6081	p3	(ATA)7	21	34173693	34173713
chr2_6082	p3	(ATA)7	21	34174225	34174245
chr2_6083	p2	(GA)6	12	34177378	34177389
chr2_6084	p3	(AAG)6	18	34182884	34182901
chr2_6085	c	gtcggagcttgagaaaatgacccctggactgagtgatggatgaagtggagt	145	34202444	34202588
chr2_6086	p2	(CA)28	56	34210463	34210518
chr2_6087	p2	(AT)6	12	34242324	34242335
chr2_6088	p2	(TG)10	20	34266102	34266121
chr2_6089	p2	(AG)6	12	34270180	34270191
chr2_6090	p2	(AC)6	12	34278691	34278702
chr2_6091	p3	(TAT)5	15	34280916	34280930
chr2_6092	c	(TA)6catacacatacacacac(AT)8	45	34306918	34306962
chr2_6093	p3	(CAG)5	15	34318892	34318906
chr2_6094	p2	(GA)6	12	34337152	34337163
chr2_6095	p2	(GT)8	16	34342735	34342750
chr2_6096	p2	(AG)6	12	34351827	34351838
chr2_6097	p2	(TG)6	12	34361010	34361021
chr2_6098	p4	(ATTT)5	20	34370256	34370275
chr2_6099	p4	(GGTA)5	20	34397358	34397377
chr2_6100	p4	(TACA)5	20	34400480	34400499
chr2_6101	p3	(ATT)6	18	34405160	34405177
chr2_6102	c	(AC)8ctacacacagtt(AC)15	58	34412616	34412673
chr2_6103	p2	(TC)7	14	34416919	34416932
chr2_6104	p2	(TG)6	12	34444699	34444710
chr2_6105	p2	(CT)8	16	34486615	34486630
chr2_6106	p3	(TAA)10	30	34501408	34501437
chr2_6107	p2	(GA)16	32	34511666	34511697
chr2_6108	p3	(TCT)5	15	34514327	34514341
chr2_6109	p2	(TC)9	18	34519500	34519517
chr2_6110	p2	(TG)15	30	34528876	34528905
chr2_6111	p3	(CAA)6	18	34534718	34534735
chr2_6112	p2	(TA)9	18	34546602	34546619
chr2_6113	p2	(CA)11	22	34564818	34564839
chr2_6114	p3	(GAG)5	15	34572504	34572518
chr2_6115	p3	(TAA)6	18	34576446	34576463
chr2_6116	p2	(CA)6	12	34580780	34580791
chr2_6117	p5	(TCCTC)5	25	34593983	34594007
chr2_6118	p4	(GACA)5	20	34607005	34607024
chr2_6119	p2	(CA)11	22	34608722	34608743
chr2_6120	p3	(TGA)5	15	34612164	34612178
chr2_6121	p2	(AC)14	28	34614936	34614963
chr2_6122	p2	(AC)18	36	34624282	34624317

chr2_6123	p2	(TG)6	12	34632814	34632825
chr2_6124	p2	(AC)12	24	34633037	34633060
chr2_6125	p3	(TCT)5	15	34635296	34635310
chr2_6126	p3	(TGC)7	21	34663596	34663616
chr2_6127	c	(GT)8gcgc(GT)11	42	34673909	34673950
chr2_6128	p3	(TCC)6	18	34694497	34694514
chr2_6129	c	(TC)6gctgtgggtt(CA)14gaagaacacgtc(CA)10	82	34696776	34696857
chr2_6130	p2	(AC)6	12	34701056	34701067
chr2_6131	p4	(AAAC)5	20	34702025	34702044
chr2_6132	p2	(CA)12	24	34709175	34709198
chr2_6133	p3	(ACA)8	24	34711512	34711535
chr2_6134	p4	(GACA)5	20	34715678	34715697
chr2_6135	p2	(GT)14	28	34722340	34722367
chr2_6136	p2	(CA)11	22	34723921	34723942
chr2_6137	p2	(AC)12	24	34724925	34724948
chr2_6138	p2	(CA)13	26	34728236	34728261
chr2_6139	p2	(CA)6	12	34731092	34731103
chr2_6140	p2	(AC)20	40	34731288	34731327
chr2_6141	p2	(CT)7	14	34733527	34733540
chr2_6142	p2	(AC)6	12	34736986	34736997
chr2_6143	p2	(AC)23	46	34740520	34740565
chr2_6144	c	:aagtcgtcctcctgtaacacacacacagacgcacattatgaacacatcaaa	120	34746712	34746831
chr2_6145	p2	(CA)6	12	34749130	34749141
chr2_6146	p2	(GT)6	12	34750038	34750049
chr2_6147	p2	(CT)7	14	34750408	34750421
chr2_6148	c	(AC)6agggg(GA)6	29	34754798	34754826
chr2_6149	c	gtcatgcaatacaagccctgctgagtcacatgaccaacatcaaccaatgag	129	34767626	34767754
chr2_6150	p3	(CTC)5	15	34770784	34770798
chr2_6151	p3	(TTG)5	15	34776226	34776240
chr2_6152	p2	(CA)14	28	34792149	34792176
chr2_6153	p2	(AT)6	12	34796294	34796305
chr2_6154	p2	(TG)6	12	34797176	34797187
chr2_6155	p4	(CAGT)5	20	34816455	34816474
chr2_6156	p4	(ACAG)6	24	34817338	34817361
chr2_6157	p2	(CA)16	32	34821352	34821383
chr2_6158	p2	(TC)10	20	34821916	34821935
chr2_6159	p2	(TG)11	22	34832422	34832443
chr2_6160	p2	(TA)7	14	34833103	34833116
chr2_6161	p2	(AC)17	34	34834803	34834836
chr2_6162	p2	(CA)16	32	34846793	34846824
chr2_6163	p2	(TA)8	16	34862647	34862662
chr2_6164	p4	(CTAC)5	20	34867486	34867505
chr2_6165	p2	(TG)6	12	34867653	34867664
chr2_6166	p2	(AC)7	14	34868695	34868708
chr2_6167	p2	(CA)8	16	34871917	34871932
chr2_6168	p3	(AGA)5	15	34877631	34877645
chr2_6169	p2	(TG)8	16	34898106	34898121
chr2_6170	p2	(AC)11	22	34900712	34900733
chr2_6171	p2	(AC)16	32	34902326	34902357
chr2_6172	p3	(TTG)9	27	34903018	34903044
chr2_6173	c	ggcagatgaaacatatttacagttctgagtgtaaaatgatgtctcatttatctat	110	34910386	34910495
chr2_6174	p3	(TCT)6	18	34910629	34910646

chr2_6175	p2	(AC)12	24	34928616	34928639
chr2_6176	p2	(CT)6	12	34932026	34932037
chr2_6177	p3	(TCC)6	18	34938047	34938064
chr2_6178	p2	(TA)10	20	34943417	34943436
chr2_6179	p2	(CA)7	14	34947612	34947625
chr2_6180	c	(GT)6gagtgt(GA)11	40	34948118	34948157
chr2_6181	p3	(CAT)6	18	34949813	34949830
chr2_6182	p2	(AG)6	12	34950563	34950574
chr2_6183	p3	(CAA)10	30	34951657	34951686
chr2_6184	p3	(TAT)6	18	34953980	34953997
chr2_6185	p2	(AT)6	12	34955139	34955150
chr2_6186	p2	(CA)6	12	34956268	34956279
chr2_6187	p2	(TG)6	12	34958956	34958967
chr2_6188	c	(TG)6taaatatac(AT)13	47	34967772	34967818
chr2_6189	p2	(TG)7	14	34970289	34970302
chr2_6190	p3	(CCT)5	15	34972994	34973008
chr2_6191	p3	(ACA)9	27	34976582	34976608
chr2_6192	p2	(TG)6	12	34978860	34978871
chr2_6193	p2	(CA)17	34	34979938	34979971
chr2_6194	p2	(TC)6	12	34981991	34982002
chr2_6195	p3	(CTT)6	18	34990711	34990728
chr2_6196	p2	(GT)8	16	34995911	34995926
chr2_6197	p2	(AG)7	14	34997061	34997074
chr2_6198	p2	(GA)6	12	34997226	34997237
chr2_6199	p2	(TG)6	12	34997601	34997612
chr2_6200	p3	(CTC)6	18	35001286	35001303
chr2_6201	p4	(AGGT)5	20	35004753	35004772
chr2_6202	p2	(GT)13	26	35012246	35012271
chr2_6203	c	)7tgtatgtgtgtg(TA)7(TG)6(TA)9(TG)6tatgtatatatata(T	114	35023602	35023715
chr2_6204	p2	(AC)8	16	35035736	35035751
chr2_6205	p2	(TG)9	18	35036516	35036533
chr2_6206	p3	(AAG)6	18	35040489	35040506
chr2_6207	p2	(AC)15	30	35053807	35053836
chr2_6208	p3	(GCA)5	15	35056972	35056986
chr2_6209	p2	(AC)14	28	35057276	35057303
chr2_6210	p2	(TC)12	24	35063217	35063240
chr2_6211	p3	(CCT)6	18	35063708	35063725
chr2_6212	c	catgatgcaggtttacatggatgtgtgttagtctgcatgtgcatgtgtaaact	86	35063935	35064020
chr2_6213	p3	(CTC)7	21	35068516	35068536
chr2_6214	p2	(GT)14	28	35070610	35070637
chr2_6215	c	aaacctacagaggacacccacatactgactgatacatgacttcacaggca	125	35073677	35073801
chr2_6216	p3	(ATC)6	18	35077446	35077463
chr2_6217	p3	(CCT)7	21	35078100	35078120
chr2_6218	p2	(TG)28	56	35078982	35079037
chr2_6219	p2	(CT)6	12	35079207	35079218
chr2_6220	p3	(GAG)5	15	35084182	35084196
chr2_6221	p3	(GAG)7	21	35089861	35089881
chr2_6222	p2	(AC)8	16	35090246	35090261
chr2_6223	p3	(GGA)5	15	35095348	35095362
chr2_6224	p2	(GT)6	12	35099375	35099386
chr2_6225	p2	(AC)7	14	35110183	35110196
chr2_6226	c	(GAG)5agtttcatcaatc(CT)9	46	35111580	35111625

chr2_6227	c	(AGG)6cgctgcctgaacggaggtccacataatcat(CA)7	62	35114171	35114232
chr2_6228	p3	(TTC)8	24	35118585	35118608
chr2_6229	p2	(TG)28	56	35119216	35119271
chr2_6230	p2	(AC)19	38	35119410	35119447
chr2_6231	p2	(CA)10	20	35143995	35144014
chr2_6232	p2	(AC)6	12	35148667	35148678
chr2_6233	p3	(GGA)5	15	35148829	35148843
chr2_6234	c	(CTC)6ttcctgttt(CTC)5	42	35156373	35156414
chr2_6235	p2	(AC)20	40	35156860	35156899
chr2_6236	p3	(AAT)5	15	35158640	35158654
chr2_6237	p3	(AGG)5	15	35180230	35180244
chr2_6238	p2	(GT)13	26	35180418	35180443
chr2_6239	p2	(CA)7	14	35181127	35181140
chr2_6240	p2	(AC)6	12	35183726	35183737
chr2_6241	p2	(AC)19	38	35185372	35185409
chr2_6242	p3	(TAA)5	15	35186164	35186178
chr2_6243	p3	(ACA)7	21	35186451	35186471
chr2_6244	p2	(GT)22	44	35188785	35188828
chr2_6245	c	aagctgcagctgtgtacaaagccgcccggcagctcaacatgactggctctg	110	35209757	35209866
chr2_6246	p2	(GA)7	14	35218959	35218972
chr2_6247	p3	(CTA)5	15	35221532	35221546
chr2_6248	p2	(GT)12	24	35222191	35222214
chr2_6249	p3	(GAA)5	15	35227009	35227023
chr2_6250	p2	(CA)11	22	35242566	35242587
chr2_6251	c	(GGA)5gaggatgaa(GAG)5(GT)6	51	35255264	35255314
chr2_6252	p2	(CA)7	14	35266007	35266020
chr2_6253	c	(AC)6at(AC)17	48	35268827	35268874
chr2_6254	p2	(AC)10	20	35278170	35278189
chr2_6255	p2	(TG)11	22	35281013	35281034
chr2_6256	p2	(CA)27	54	35287113	35287166
chr2_6257	p2	(CA)51	102	35289402	35289503
chr2_6258	c	cactgcagtaggccttaatgacagctgccttttaatgctgcatggagagggg	137	35292111	35292247
chr2_6259	p2	(CA)7	14	35292411	35292424
chr2_6260	c	(TG)9c(GT)7	33	35295055	35295087
chr2_6261	p2	(TG)24	48	35295216	35295263
chr2_6262	c	ttgtggggggggggggggctgtctctatgaaatggctcttttgcaacaaat	123	35298422	35298544
chr2_6263	p2	(TG)6	12	35303106	35303117
chr2_6264	p2	(TG)13	26	35321741	35321766
chr2_6265	p2	(CT)6	12	35323989	35324000
chr2_6266	c	cttcctgactggagtttggggaaacacctaccagaccagctggcagagccc	120	35325651	35325770
chr2_6267	p2	(CA)7	14	35327391	35327404
chr2_6268	p3	(TTA)6	18	35332870	35332887
chr2_6269	p2	(TG)8	16	35335183	35335198
chr2_6270	c	T)19ggtcataaagtacaggaaagggctcggtaagtacagatgaacc(GT	95	35339061	35339155
chr2_6271	p3	(AAG)5	15	35341594	35341608
chr2_6272	p2	(GT)23	46	35342922	35342967
chr2_6273	p3	(TCC)8	24	35350962	35350985
chr2_6274	p2	(CA)6	12	35351676	35351687
chr2_6275	p2	(AC)6	12	35353488	35353499
chr2_6276	p2	(AT)8	16	35355277	35355292
chr2_6277	p2	(TG)7	14	35356470	35356483
chr2_6278	p3	(ATA)5	15	35370749	35370763

chr2_6279	c	(TC)9act(CA)13	47	35381578	35381624
chr2_6280	c	gacgttcttgtaatataaacctttaaacctacctgttgtctgtctcgggaacag	92	35391117	35391208
chr2_6281	c	(GT)7gc(GT)7gc(GT)7gc(GT)7gc(GT)7	78	35396078	35396155
chr2_6282	p3	(CCT)6	18	35409408	35409425
chr2_6283	p2	(TG)14	28	35409629	35409656
chr2_6284	p2	(AC)6	12	35412614	35412625
chr2_6285	p2	(CA)8	16	35413423	35413438
chr2_6286	p2	(AC)7	14	35414270	35414283
chr2_6287	p2	(AC)11	22	35416430	35416451
chr2_6288	p2	(GT)13	26	35418726	35418751
chr2_6289	p2	(TG)9	18	35423921	35423938
chr2_6290	p2	(AC)11	22	35425257	35425278
chr2_6291	p2	(CA)10	20	35440186	35440205
chr2_6292	p2	(TG)15	30	35443043	35443072
chr2_6293	p2	(TG)16	32	35444566	35444597
chr2_6294	p2	(CA)19	38	35446852	35446889
chr2_6295	p3	(TCT)5	15	35447836	35447850
chr2_6296	p3	(GCT)5	15	35461426	35461440
chr2_6297	p3	(TTA)6	18	35465927	35465944
chr2_6298	p2	(CA)6	12	35468780	35468791
chr2_6299	p2	(AC)23	46	35470233	35470278
chr2_6300	c	(GA)10a(AG)6	33	35471960	35471992
chr2_6301	p2	(CA)6	12	35473166	35473177
chr2_6302	p2	(GA)6	12	35481437	35481448
chr2_6303	p2	(GA)11	22	35484562	35484583
chr2_6304	c	cagaggagaaacaatatgaggagatatggtttctgagactcctgctgacctg	103	35484918	35485020
chr2_6305	p2	(GT)12	24	35486348	35486371
chr2_6306	p2	(AC)6	12	35486562	35486573
chr2_6307	p2	(CA)9	18	35487927	35487944
chr2_6308	p3	(GAG)5	15	35488135	35488149
chr2_6309	p2	(AC)7	14	35495100	35495113
chr2_6310	p2	(CA)8	16	35495388	35495403
chr2_6311	p2	(CA)15	30	35509286	35509315
chr2_6312	p2	(GT)17	34	35514785	35514818
chr2_6313	p2	(AG)6	12	35516821	35516832
chr2_6314	c	ctgtggcggcaggagctgagcgtggtgcttcacatg(GA)6ggatgtta	170	35519155	35519324
chr2_6315	p2	(GT)7	14	35522279	35522292
chr2_6316	p2	(AC)6	12	35524107	35524118
chr2_6317	p2	(CA)20	40	35524564	35524603
chr2_6318	p2	(GT)6	12	35525278	35525289
chr2_6319	p2	(GA)6	12	35525437	35525448
chr2_6320	p2	(TG)9	18	35525678	35525695
chr2_6321	p2	(GT)12	24	35527801	35527824
chr2_6322	p2	(AC)15	30	35535364	35535393
chr2_6323	p2	(CA)9	18	35536320	35536337
chr2_6324	p2	(AC)21	42	35537412	35537453
chr2_6325	c	(CAG)5acagcagacctccagcagctccactctccctccca(TCT)6	68	35542182	35542249
chr2_6326	p2	(AC)6	12	35542952	35542963
[truncated: 437,938 more chars]
